# Comparative transcriptome analysis reveals resistant and susceptible genes in tobacco cultivars in response to infection by *Phytophthora nicotianae*

**DOI:** 10.1038/s41598-020-80280-7

**Published:** 2021-01-12

**Authors:** He Meng, Mingming Sun, Zipeng Jiang, Yutong Liu, Ying Sun, Dan Liu, Caihong Jiang, Min Ren, Guangdi Yuan, Wenlong Yu, Quanfu Feng, Aiguo Yang, Lirui Cheng, Yuanying Wang

**Affiliations:** 1 Key Laboratory of Tobacco Genetic Improvement and Biotechnology, Tobacco Research Institute, Chinese Academy of Agricultural Sciences, Qingdao, 266100 China; 2 College of Agronomy, Qingdao Agricultural University, Qingdao, 266109 China

**Keywords:** Plant genetics, Plant immunity

## Abstract

*Phytophthora nicotianae* is highly pathogenic to *Solanaceous* crops and is a major problem in tobacco production. The tobacco cultivar Beihart1000-1 (BH) is resistant, whereas the Xiaohuangjin 1025 (XHJ) cultivar is susceptible to infection. Here, BH and XHJ were used as models to identify resistant and susceptible genes using RNA sequencing (RNA-seq). Roots were sampled at 0, 6, 12, 24, and 60 h post infection. In total, 23,753 and 25,187 differentially expressed genes (DEGs) were identified in BH and XHJ, respectively. By mapping upregulated DEGs to the KEGG database, changes of the rich factor of “plant pathogen interaction pathway” were corresponded to the infection process. Of all the DEGs in this pathway, 38 were specifically regulated in BH. These genes included 11 disease-resistance proteins, 3 pathogenesis-related proteins, 4 *RLP/RLKs*, 2 *CNGCs*, 7 calcium-dependent protein kinases, 4 calcium-binding proteins, 1 mitogen-activated protein kinase kinase, 1 protein *EDS1L*, 2 *WRKY* transcription factors, 1 mannosyltransferase, and 1 calmodulin-like protein. By combining the analysis of reported susceptible (*S*) gene homologs and DEGs in XHJ, 9 *S* gene homologs were identified, which included 1 calmodulin-binding transcription activator, 1 cyclic nucleotide-gated ion channel, 1 protein trichome birefringence-like protein, 1 plant UBX domain-containing protein, 1 ADP-ribosylation factor GTPase-activating protein, 2 callose synthases, and 2 cellulose synthase A catalytic subunits. qRT-PCR was used to validate the RNA-seq data. The comprehensive transcriptome dataset described here, including candidate resistant and susceptible genes, will provide a valuable resource for breeding tobacco plants resistant to *P. nicotianae* infections.

## Introduction

As a typical oomycete, *Phytophthora nicotianae* has a broad host range; this pathogen causes root rot, crown rot, fruit rot, and leaf and stem infections ^1–4^. *P. nicotianae* can attack all parts of *Nicotiana tabacum*, including the roots, stems, and leaves at any stage of its growth, and the most common symptom of infection is a black base or shank of the stalk ^5^*.* The disease can be devastating to tobacco in the greenhouse, as well as in the field, leading to severe yield losses every year worldwide ^6^. In a recent ranking of oomycete species based on scientific and economic importance, *P. nicotianae* was listed eighth ^7^. Additionally, *P. nicotianae* is well adapted to high temperatures*;* therefore, it is gaining importance in agriculture and plant health worldwide as the trend of global warming increases^8^.

Multiple sources have been used to improve resistance in cultivated tobacco for *P. nicotianae*. For example, the *Php* and *Phl* genes were introgressed from the closely related species *N. plumbaginifolia* and *N. longiflora*, providing immunity to race 0 of *P. nicotianae* ^4^. In addition to dominant resistance, polygenic resistance, which occurs in commercial flue-cured and burley tobacco cultivars, was likely derived from the cigar tobacco cultivar Florida 301 ^9^. This cultivar was produced from crossing the cultivars Big Cuba and Little Cuba by Tisdale in the 1930s, and it is resistant to all known strains of *P. nicotianae* ^9^. The cultivar Beinhart 1000 (BH), which originated from selection of the cultivar Quin Diaz, has the highest reported level of quantitative resistance to *P. nicotianae*, and resistance in this line may be effective against all races ^10^. Another alien gene, *Wz*, introgression from *N. rustica,* has been found to confer a high level of resistance to race 0 and 1 ^11^. Although multiple resistance sources are used in breeding against *P. nicotianae*, none of these resistant genes have been cloned, and the mechanism of resistance has not been elucidated.

The coevolution of plants and pathogens has resulted in the development of a multifaceted and sophisticated plant immune system. In addition to barriers at the surface of plant cells, plants have developed two layers of induced defense responses that rely on the recognition of pathogen-, microbe-, or damage-associated molecular patterns (PAMPs, MAMPs, or DAMPs, respectively) and pathogen effectors, known as PAMP-triggered immunity (PTI) and effector-triggered immunity (ETI) ^12^. This type of immune system has been termed a “zigzag” model ^12^. Conversely, pathogens require host cooperation to establish a compatible interaction. Plant genes that facilitate infections can be considered as susceptibility (*S*) genes ^13^, and disrupting these *S* genes may interfere with the compatibility between the host and the pathogen ^13^. A breeding strategy that involved disabling plant *S* genes was proposed in 2010 ^14^. Recently, more attention has been paid to the study and exploitation of plant *S* genes as a source of broad-spectrum and durable resistance ^15^. Functional screens in *Arabidopsis* have yielded many *S* gene candidates ^13^. Based on available genome and transcriptome sequencing data, homologous gene sequences from other crops can be identified.

Thus, systematic determination of key resistance and susceptibility genes in response to infection by *P. nicotianae* would help to accelerate the breeding of new strains. RNA sequencing (RNA-seq) is a powerful tool for studying disease resistance in plants. In this study, we used the resistant cultivar Beinhart 1000–1 (hereafter, BH), a selection of Beinhart 1000, and the susceptible cultivar Xiaohuangjin 1025 (hereafter, XHJ) ^16^ as models. RNA-seq was used to analyze the gene expression profiles of roots from both cultivars at 6, 12, 24, and 60 h post-inoculation (hpi) with *P. nicotianae*. We identified DEGs in both cultivars during the infection process and screened tobacco resistant and susceptible genes to *P. nicotianae*. Our results provide a basis for understanding the mechanisms of the responses of tobacco to *P. nicotianae* and provide a potentially valuable resource for the future development of resistant plants.

## Results

### Transcriptome sequencing of resistant and susceptible tobacco cultivars infected by *P. nicotianae*

**Figure 1:**
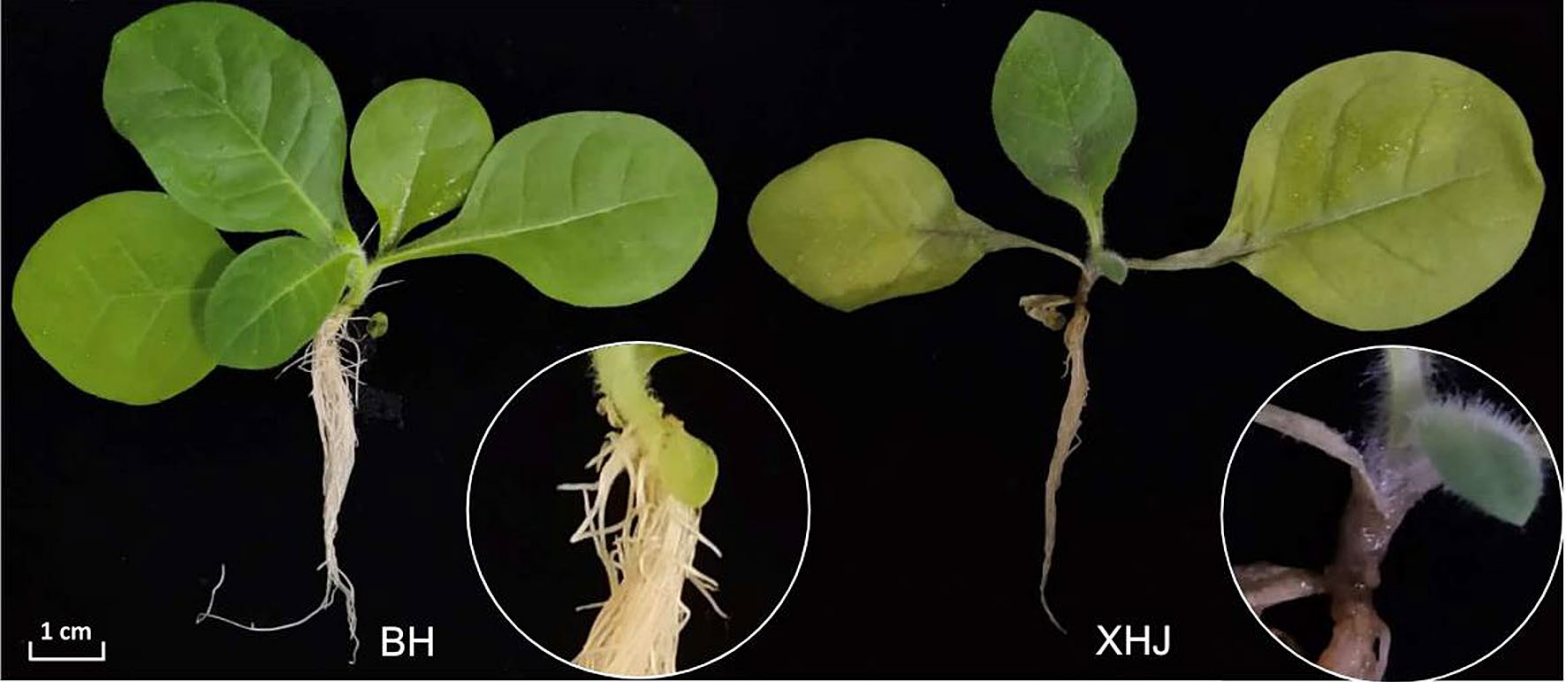
Disease symptoms in the cultivars BH (resistant) and XHJ (susceptible) at 5 dpi by *Phytophthora nicotianae*. The basal parts of stems are magnified and shown in the circles. In BH (Left), no symptoms were apparent. In XHJ (right), leaves were withered and the basal part of stem was severely necrosed and became black.

The primary symptoms induced by *P. nicotianae* infection, such as slight leaf wilting, appeared in XHJ at 60 hpi. At 5 days post-inoculation (dpi), differences in symptoms were observed between the two cultivars. Leaf wilting and severe stem necrosis occurred in XHJ, whereas these symptoms were not apparent in BH (Fig. 1).

To investigate the transcriptional differences between BH and XHJ in response to *P. nicotianae*, we exposed 6-week-old seedlings of BH and XHJ to this pathogen. Five sequencing libraries were generated for each cultivar from the total RNA of healthy root tissues and infected root tissues at 6, 12, 24, and 60 hpi. A total of 1,475,991,460 clean reads (221.4 GB) were generated using Illumina RNA-Seq deep sequencing. Clean data were submitted to the NCBI Sequence Reads Archive (SRA) database (Accession number: PRJNA679433). The reads of all samples (inoculated BH, non-inoculated BH, inoculated XHJ, and non-inoculated XHJ) were used for transcriptome assembly (Table 1). On average, 91.09% (BH at 0 hpi), 92.19% (BH at 6 hpi), 92.08% (BH at 12 hpi), 89.33% (BH at 24 hpi), 77.94% (BH at 60 hpi), 90.60% (XHJ at 0 hpi), 92.65% (XHJ at 6 hpi), 92.26% (XHJ at 12 hpi), 71.26% (XHJ at 24 hpi), and 7.26% (XHJ at 60 hpi ) were mapped to the reference transcriptome (Supplementary Table S1).

**Table 1:** Statistics from Illumina sequencing.

Sample name	Raw reads	Clean reads	Clean bases	Q20 (%)	Q30 (%)	Notes
BH0_1	47,707,322	45,948,242	6.89	96.56	91.35	Replicate1
BH0_2	49,859,176	48,045,940	7.21	96.38	91.02	Replicate2
BH0_3	53,036,808	50,920,626	7.64	96.7	91.7	Replicate3
BH6_1	50,403,072	48,681,604	7.3	96.31	90.86	Replicate1
BH6_2	52,684,610	49,930,296	7.49	96.37	90.76	Replicate2
BH6_3	43,860,286	41,261,766	6.19	96.26	90.62	Replicate3
BH12_1	55,889,362	52,833,466	7.93	96.2	90.35	Replicate1
BH12_2	55,168,106	52,159,774	7.82	96.32	90.66	Replicate2
BH12_3	48,204,196	47,223,844	7.08	96.8	91.87	Replicate3
BH24_1	49,009,578	47,107,542	7.07	96.52	91.47	Replicate1
BH24_2	67,343,622	65,207,594	9.78	96.36	91.15	Replicate2
BH24_3	55,952,008	54,131,198	8.12	96.45	91.44	Replicate3
BH60_1	47,407,132	46,216,846	6.93	96.42	91.26	Replicate1
BH60_2	54,957,870	52,826,334	7.92	96.16	90.83	Replicate2
BH60_3	48,375,044	47,254,888	7.09	96.17	90.1	Replicate3
XHJ0_1	61,242,928	59,415,020	8.91	96.54	91.58	Replicate1
XHJ0_2	49,787,856	47,909,420	7.19	96.02	90.55	Replicate2
XHJ0_3	61,476,162	59,300,820	8.9	96.17	90.76	Replicate3
XHJ6_1	44,092,690	43,264,280	6.49	95.98	89.42	Replicate1
XHJ6_2	54,081,282	52,201,798	7.83	96.86	92.42	Replicate2
XHJ6_3	49,882,966	48,188,662	7.23	96.62	91.91	Replicate3
XHJ12_1	57,083,078	52,479,764	7.87	96.76	92.08	Replicate1
XHJ12_2	52,227,998	50,270,384	7.54	96.51	91.62	Replicate2
XHJ12_3	44,692,876	42,026,640	6.3	95.83	89.38	Replicate3
XHJ24_1	51,180,362	49,309,828	7.4	96.61	91.85	Replicate1
XHJ24_2	44,955,892	44,273,812	6.64	96.73	91.92	Replicate2
XHJ24_3	43,910,138	42,618,924	6.39	95.87	89.29	Replicate3
XHJ60_1	47,641,186	45,981,950	6.9	94.52	86.82	Replicate1
XHJ60_2	46,894,120	45,557,104	6.83	95.06	87.7	Replicate2
XHJ60_3	43,727,590	43,443,094	6.52	96.08	90.75	Replicate3
Total	1,532,735,316	1,475,991,460	221.4	96.27133	90.783	

### Identification of DEGs in resistant and susceptible tobacco cultivars during the infection process and GO enrichment analysis

**Figure 2:**
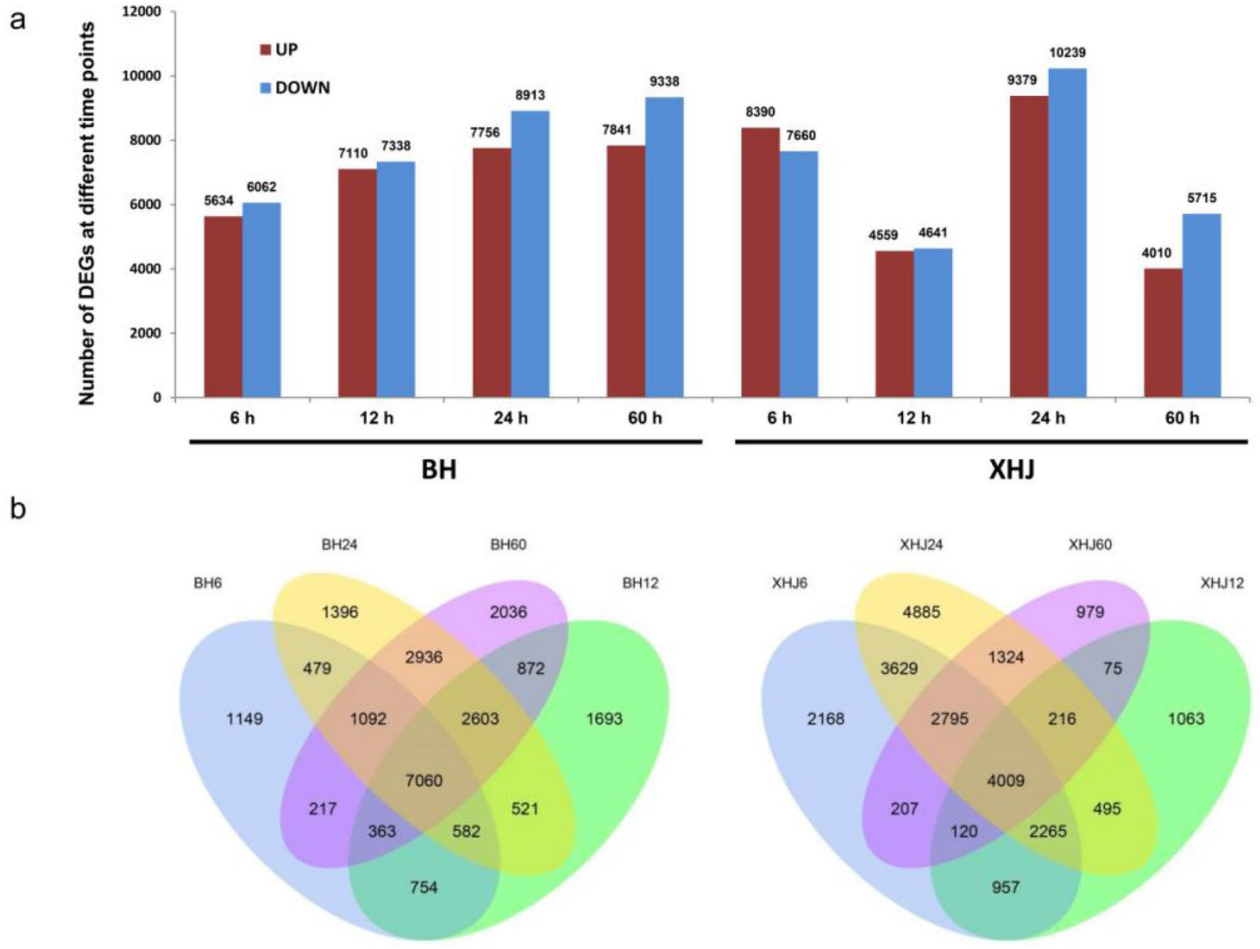
Identification of DEGs in cultivars BH and XHJ following infection with *P. nicotianae.* (**a**) Number of DEGs at different time points (log2 Ratio ≥ 1; p ≤ 0.001). (**b**) Venn diagrams showing the numbers of specific and common DEGs at each time point.

The fragments per kilobase per million (FPKM) values for each unigene in all 30 libraries were computed and are displayed in Supplementary Table S2. Gene expression profiles in healthy BH and XHJ roots were used as the baselines. If there was a two-fold (or more) difference in gene expression in infected roots relative to the baseline (*p* < 0.001), the gene was regarded as a DEG. As shown in Fig. 2, for BH inoculated with *P. nicotianae*, there were 11,696 DEGs at 6 hpi, and the number of DEGs increased gradually between 12 hpi (14,448) and 24 hpi (16,669) and finally increased to 17,179 at 60 hpi. In comparison, in *P. nicotianae-*infected XHJ roots, there were 16,050 DEGs at 6 hpi and only 9,200 DEGs at 12 hpi, which then increased to 19,618 at 24 hpi and again decreased to 9,725 at 60 hpi. Venn diagrams were generated from the DEGs identified at 6, 12, 24, and 60 hpi, corresponding to each of the cultivar-pathogen combinations. At all-time points, 23,753 DEGs were identified in the BH group, whereas there were 7,060 DEGs shared at all-time points. A total of 25,187 DEGs were identified in the XHJ group, with a total of 4,009 shared DEGs at all-time points.

To identify differences in Gene Ontology (GO) term enrichment analysis between the two cultivars after inoculation, all DEGs at different time points in BH (23,753) and XHJ (25,187) were analyzed using GOseq, with a cut-off corrected *p* value of 10^−10^. 29 and 15 GO terms were identified in BH and XHJ respectively (Table 2). The most significantly enriched GO term in BH and XHJ was the structural constituent of the ribosome. In biological process (BP) terms, expression of genes related to metabolic processes, cellular protein metabolic processes, protein phosphorylation, and phosphorylation were remarkably more significant in BH than in XHJ. In cellular component (CC) terms, intracellular non-membrane-bound organelles and non-membrane-bounded organelles were more significant in BH than in XHJ. In molecular function (MF) terms, structural molecule activity, antioxidant activity, oxidoreductase activity, acting on peroxide as an acceptor, protein kinase activity, peroxidase activity, heme binding, and tetrapyrrole binding were remarkably more significant in BH than in XHJ. In contrast, transferase activity was more significant in XHJ than in BH.

**Table 2:**
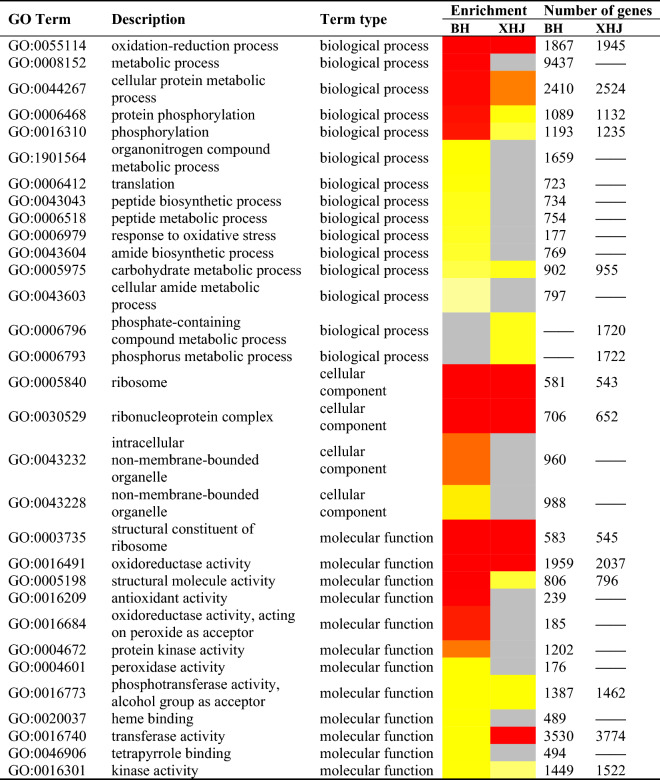
Comparison of Gene Ontology (GO) term enrichment analysis of DEGs in BH group and XHJ group. Significance levels are based on enrichment and lowest P values with a cutoff of < 10^−10^.

Different color means different degree of enrichment, higher significant terms indicated by red, lower significant terms indicated by yellow, gray means no term assigned in that particular category.

### Screening and analysis of resistant genes against *P. nicotianae* in tobacco

**Figure 3:**
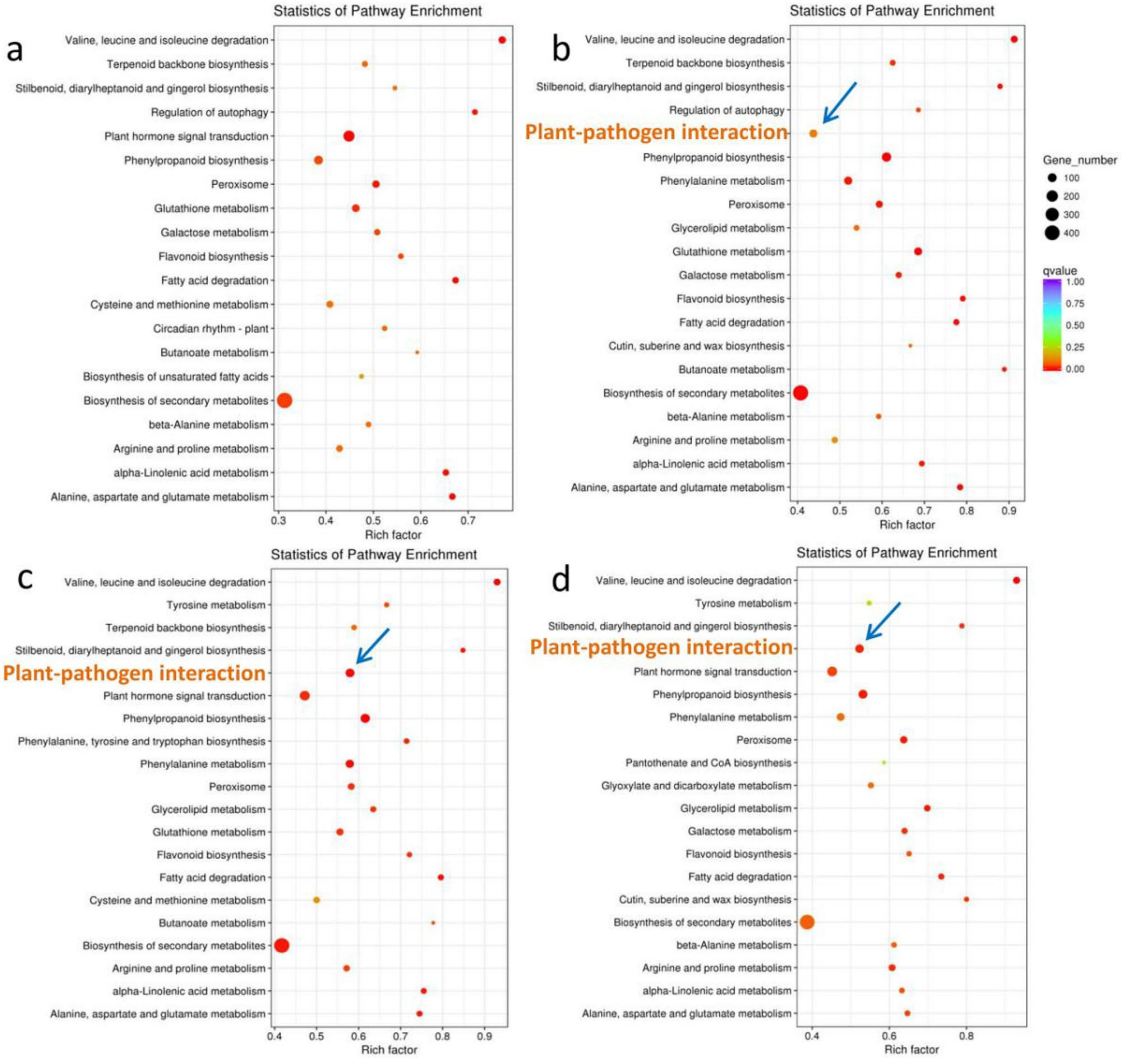
Scatterplot of KEGG pathways enrichment analysis for upregulated DEGs at (**a**) 6, (**b**) 12, (**c**) 24, and (**d**) 60 hpi in BH. The rich factor is the ratio of the number of DEGs annotated in a given pathway term to the number of all genes annotated in the pathway term. A higher rich factor indicates greater intensity. The Q value is the corrected P value and ranges from 0 to 1, with a lower Q value indicating greater intensity. The sizes of the circles indicate the number of genes. The top 20 enriched pathway terms in the KEGG database are listed. The blue arrows indicates the plant-pathogen interaction pathway.

To further elucidate the functions of DEGs and to analyze the resistance mechanism of BH, KEGG pathway enrichment analysis involving upregulated DEGs at different infection time points was conducted by mapping to the KEGG database. The top 20 metabolic pathways associated with these DEGs in BH are shown in Fig. 3. Among these, changes in the rich factor of the “plant-pathogen interaction” pathway corresponded to the infection process and were much higher in BH than in XHJ (Supplementary Fig. 1). As shown in Fig. 3, the rich factor of the “plant pathogen interaction pathway” in BH was nearly 0.45 at 12 hpi, which increased to almost 0.58 at 24 hpi and declined to 0.52 thereafter.

**Figure 4:**
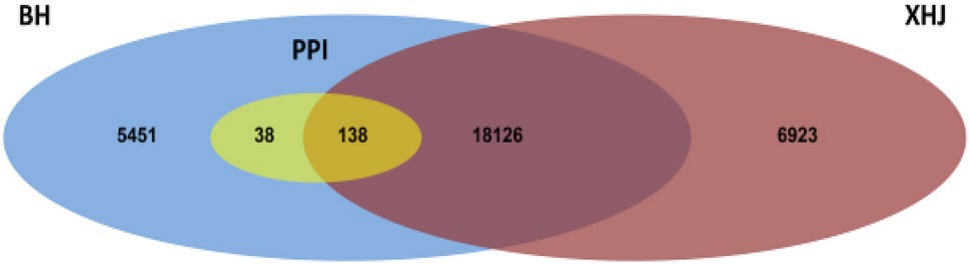
Venn diagram of DEG identified in the BH group, XHJ group, and all DEGs in “plant pathogen interaction pathway”. PPI means DEGs in “plant pathogen interaction pathway”.

To investigate the differences in resistance mechanisms between BH and XHJ, a Venn diagram was generated from the DEGs identified in the BH and XHJ groups and all DEGs in the “plant pathogen interaction pathway” (Fig. 4). In total, 5,489 DEGs were specifically identified in BH, 6,923 DEGs were specifically identified in XHJ, and 18,264 DEGs were common to both BH and XHJ. Of all the DEGs in the “plant pathogen interaction pathway”, 38 were specifically regulated in BH.

**Figure 5:**
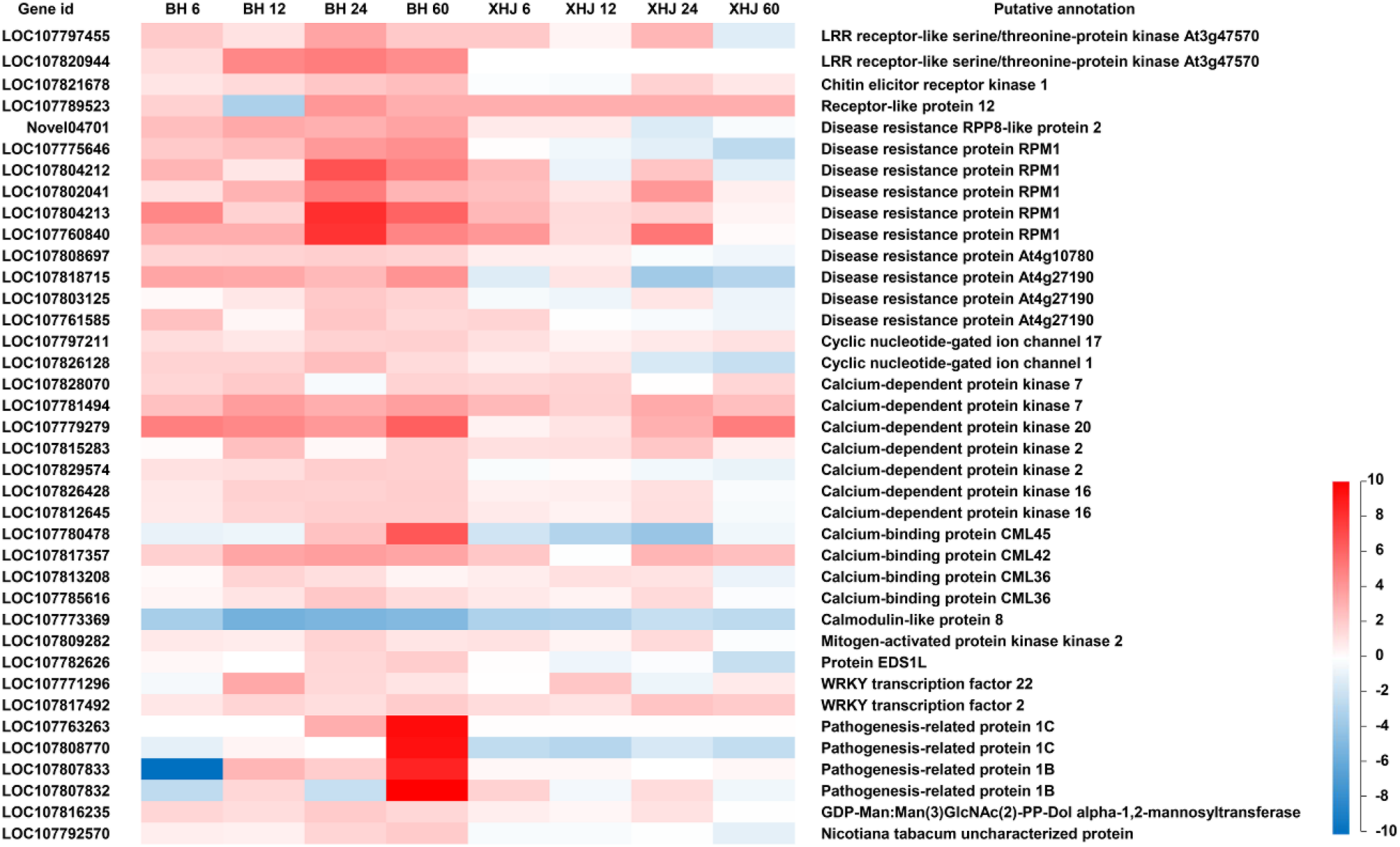
Expression profiles in BH and XHJ of 38 specifically regulated DEGs in BH within the “plant pathogen interaction pathway”. Genes in red are upregulated, whereas those in blue are downregulated. The regulation of genes is based on log2 fold change compared to the mock-infected control samples.

The expression data of the 38 DEGs are shown in Fig. 5. Remarkably, there were 11 disease resistance proteins upregulated in BH, especially at 24 hpi and 4 pathogenesis-related proteins upregulated in BH, especially at 60 hpi. In addition, 4 *RLP/RLKs*, 2 *CNGCs*, 7 calcium-dependent protein kinases, 4 calcium-binding proteins, 1 mitogen-activated protein kinase kinase, 1 protein *EDS1L*, 2 *WRKY* transcription factors, and 1 mannosyltransferase were upregulated in BH, whereas 1 calmodulin-like protein was downregulated in BH.

By further focusing on the disease resistance proteins and pathogenesis-related protein, we found that, 1 disease resistance protein was a homolog of At4g10780, 3 were homologs of At4g27190, 4 were homologs of *RPM1* and 1 was a homolog of *RPP8*. Pathogenesis-related proteins were homologs of pathogenesis-related protein 1B and 1C. The fold change versus the mock-infected control is shown in Table 3.

**Table 3:** Key disease resistance proteins and pathogenesis-related proteins significantly induced in BH. The gene expression data at 6, 12, 24, and 60 hpi were compared to data in mock-infected control and the fold change is demonstrated.

Gene ID	Putative annotation	Fold change versus mock control in BH				Fold change versus mock control in XHJ			
		6 hpi	12 hpi	24 hpi	60 hpi	6 hpi	12 hpi	24 hpi	60 hpi
LOC107808697	Disease resistance protein At4g10780	2.10	2.14	**2.27**	2.12	1.37	1.36	0.88	0.69
LOC107818715	Disease resistance protein At4g27190	**4.67**	4.39	3.29	6.30	0.45	1.60	0.10	0.16
LOC107803125	Disease resistance protein At4g27190	1.12	1.52	**2.50**	2.15	0.82	0.66	1.56	0.64
LOC107761585	Disease resistance protein At4g27190	**2.88**	1.18	2.67	1.96	2.09	1.00	0.84	0.66
LOC107804213	Disease resistance protein RPM1	7.79	2.18	**35.06**	14.21	3.37	1.86	2.15	1.22
LOC107804212	Disease resistance protein RPM1	3.54	1.55	**18.99**	8.62	3.28	0.62	2.72	0.50
LOC107802041	Disease resistance protein RPM1	1.69	3.69	**9.13**	3.55	2.92	1.56	5.89	1.33
LOC107775646	Disease resistance protein RPM1	2.50	3.01	**5.75**	**6.76**	1.04	0.71	0.51	0.20
Novel04701	Disease resistance RPP8-like protein 2	3.05	**4.38**	3.86	**4.79**	1.43	1.43	0.42	0.86
LOC107807833	Pathogenesis-related protein 1B	1.00	3.46	2.36	**42.20**	1.15	1.15	1.00	1.15
LOC107808770	Pathogenesis-related protein 1C	0.54	1.22	1.00	**57.99**	0.22	0.17	0.38	0.23

### Screening and analysis of *P. nicotianae* susceptible genes in tobacco

**Figure 6:**
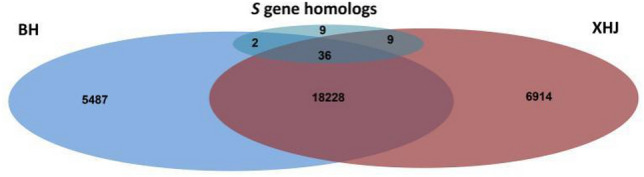
Venn diagram of DEG identified in the BH group, XHJ group and *S* gene homologs.

To screen for *S* genes in tobacco, 28 *S* genes reported to interact with oomycetes or fungi in other crops were chosen, and their 56 homologs in tobacco were identified (Supplementary Table S3). Venn diagrams were generated from the DEGs identified in the BH group, XHJ group, and 56 *S* gene homologs (Fig. 6); 9 *S* gene homologs were specifically identified in XHJ.

**Figure 7:**
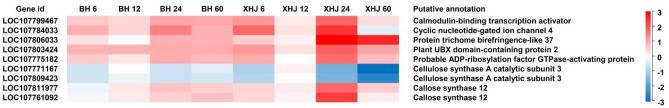
Expression profiles in BH and XHJ of 9 *S* gene homologs specifically identified in XHJ. Genes in red are upregulated whereas those in blue are downregulated. The regulation of genes is based on log2 fold change compared to the mock-infected control samples.

Expression data of the 9 *S* gene homologs specifically identified in XHJ are shown in Fig. 7. These included 1 calmodulin-binding transcription activator, 1 cyclic nucleotide-gated ion channel, 1 protein trichome birefringence-like protein, 1 plant UBX domain-containing protein, 1 probable ADP-ribosylation factor GTPase-activating protein, and 2 callose synthases, all of which were specifically upregulated in XHJ, especially at 24 hpi. Two cellulose synthase A catalytic subunits were specifically downregulated in XHJ, especially at 60 hpi. Pathogens require the cooperation of host *S* genes to establish a compatible interaction. During the infection process, pathogens deploy effectors to inhibit defense networks, thereby activating *S* genes. Therefore, gene upregulation may be a criterion for screening candidate *S* genes.

In XHJ, at 24 hpi, expression of homologs of calmodulin-binding transcription activator 3, cyclic nucleotide-gated ion channel 4, protein trichome birefringence-like 37, plant UBX domain-containing protein 2, ADP-ribosylation factor GTPase-activating protein, and 2 callose synthases increased more than threefold compared to the mock control (Table 4).

**Table 4:** Analysis of candidate susceptible genes significantly induced in XHJ. The gene expression data at 6, 12, 24 and 60 hpi were compared to data in mock-infected control and the fold change is demonstrated.

Gene ID	Putative annotation	Fold change versus mock control in BH				Fold change versus mock control in XHJ			
		6 hpi	12 hpi	24 hpi	60 hpi	6 hpi	12 hpi	24 hpi	60 hpi
LOC107799467	Calmodulin-binding transcription activator 3	1.54	1.38	2.03	2.00	2.23	1.19	**3.38**	1.32
LOC107784033	Cyclic nucleotide-gated ion channel 4	1.87	1.39	2.73	1.88	3.30	1.26	**5.00**	0.77
LOC107806033	Protein trichome birefringence-like 37	1.26	0.99	1.86	1.85	1.52	1.13	**7.50**	**5.61**
LOC107803424	Plant UBX domain-containing protein 2	1.83	1.85	1.94	1.93	2.24	1.27	**3.56**	2.04
LOC107775182	ADP-ribosylation factor GTPase-activating protein	1.53	1.23	1.94	1.72	1.93	1.00	**3.15**	1.67
LOC107811977	Callose synthase 12	0.85	1.24	1.64	1.48	1.41	1.17	**3.29**	1.50
LOC107761092	Callose synthase 12	0.91	1.19	1.45	1.63	1.38	1.09	**4.58**	1.15

### Identification of *P. nicotianae* genes during the early infection stage

To identify DEGs in *P. nicotianae* during the early infection stage in the susceptible variety, reads from 9 sequencing libraries (XHJ at 6 hpi, XHJ at 12 hpi, and XHJ at 24 hpi) were aligned to the reference genome of *P. nicotianae* race 0 (NCBI: PRJNA294216). On average, 0.08% (XHJ at 6 hpi), 0.09% (XHJ at 12 hpi), and 6.17% (XHJ at 24 hpi) were mapped to the reference genome. The fragments per kilobase per million (FPKM) values for each unigene in 9 libraries were computed and are shown in Supplementary Table S4. Within these genes, some RxLR effectors ^17^, AM587_10007145, AM587_10001643, and AM587_10002874 were sharply expressed at 6 hpi and 24 hpi in XHJ.

### Verification of DEGs using quantitative reverse-transcription PCR (qRT-PCR)

**Figure 8:**
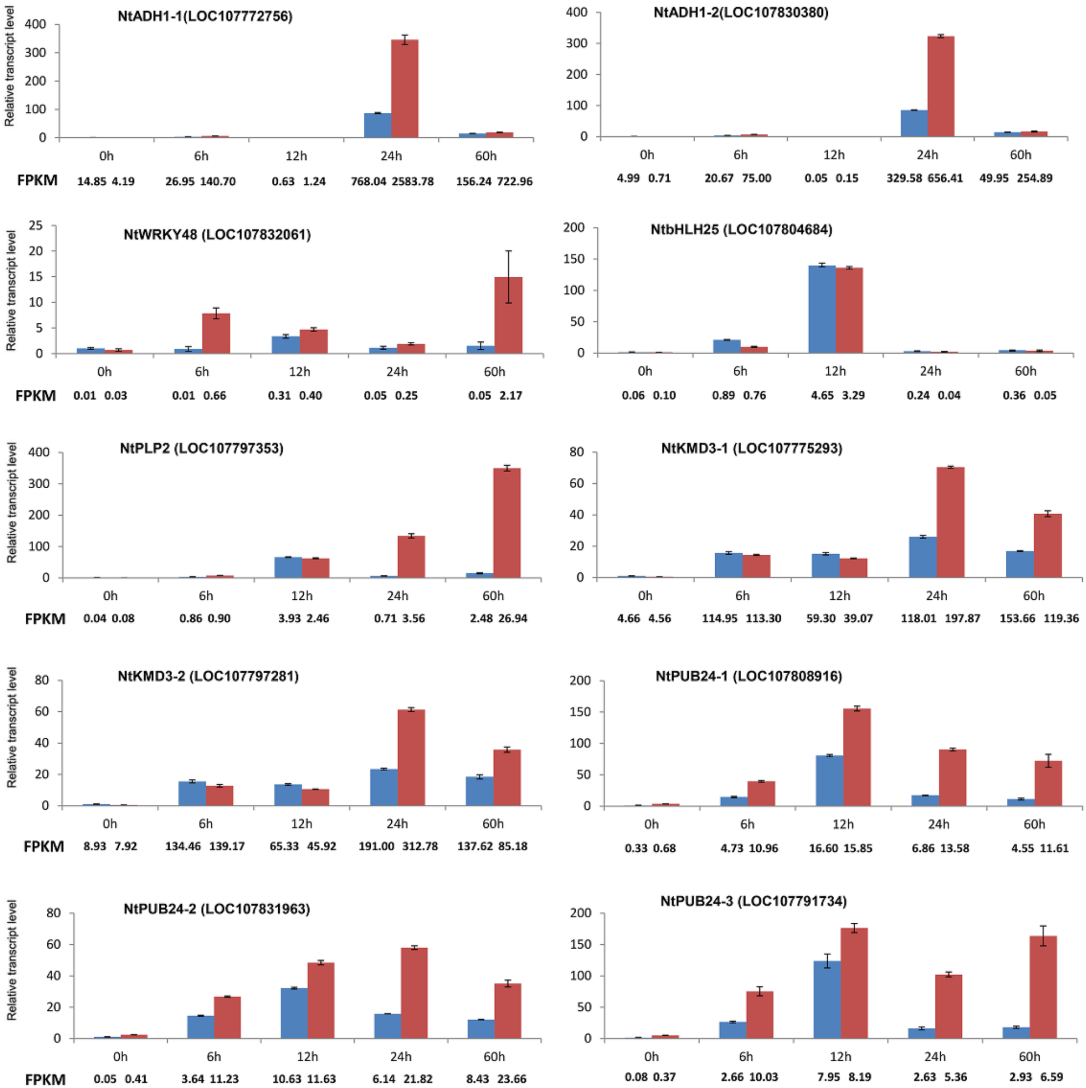
The relative expression levels of identified susceptible genes at each time point after *P. nicotianae* inoculation in BH and XHJ. Blue bars indicate BH, whereas red bars indicate XHJ. Actin was used as an internal control, and the transcript level in non-infected plants was set as 1.0. Error bars represent standard error (*n* = 3).

To confirm the results obtained using RNA-seq, we chose 10 sharply upregulated homologs of 6 reported *S* genes (*ADH1, WRKY48*, *bHLH25*, *PLP2*, *KMD3*, *PUB24*) ^13^ and performed qRT-PCR. The results of the qRT-PCR correlated with the RNA-seq data (evaluated by FPKM), and the gene expression increased more significantly in XHJ than in BH (Fig. 8). Expression of homologs of *PUB24* and *bHLH25* rose sharply at 12 hpi, homologs of *ADH1* and *KMD3* rose sharply at 24 hpi, and the homologs of *WRKY48* and *PLP2* rose sharply at 60 hpi.

## Discussion

The transcriptome has been widely used to study the defense response in plants and to identify specific genes that interact with pathogens. In *Lomandra longifolia* roots, callose synthase genes, *MAPK* 15, 2 *PR* genes, and 5 receptor-like protein genes were found to be significantly expressed when infected by *Phytophthora cinnamomi* ^18^. In *N. benthamiana* leaves, expression of 13 b-1,3-glucanases from the *PR-2* family, and 16 chitinases from the *PR-3*, *PR-4*, *PR-8*, and *PR-11* families were induced following infection with *P. parasitica* ^19^*.* In sugarcane stalks, cytochrome P450, chitinase, NBS-LRR domain-containing proteins, and leucine zipper domain proteins were identified when infected by *Sporisorium scitamineum* ^20^. Furthermore, pathogens deploy effector proteins to inhibit defense networks; therefore, susceptibility factors encoded by *S* genes can be activated during infection ^13^. In pepper, the expression of *CaMlo2* is upregulated at an earlier time point following *Leveillula taurica* infection, and complementation experiments confirmed the role of *CaMlo2* as a susceptibility factor to different powdery mildews ^21^. *SWEET* sugar transporters, susceptible factors, can be upregulated during pathogen attack to export sugars from cells into the extracellular spaces ^22^. The transcription factors *bHLH25* and *bHLH27* positively influence cyst nematode parasitism and were upregulated at 1 dpi ^23^. Thus, it is feasible to identify resistant and susceptible genes using RNA-seq.

By profiling genes specifically regulated in resistant tobacco before and after infection with *P. nicotianae*, 11 disease resistance proteins, 3 pathogenesis-related proteins, 4 *RLP/RLKs*, 2 *CNGCs*, 7 calcium-dependent protein kinases, 4 calcium-binding protein, 1 mitogen-activated protein kinase kinase, 1 protein *EDS1L*, 2 *WRKY* transcription factor, 1 mannosyltransferase and 1 calmodulin-like protein were identified. In *Arabidopsis*, *RPM1* confers resistance against *Pseudomonas syringae* expressing AvrRpm1 or AvrB ^24^, whereas *RPP8* confers resistance to *P. parasitica* ^25^*. PR-1* proteins are produced abundantly during defense responses, and have been shown to possess sterol-binding activity ^26^. Cyclic nucleotide-gated channels (*CNGCs*) are nonselective cation channels that permit the diffusion of divalent and monovalent cations. *CNGCs* are involved in both basal and *R* gene-mediated plant immunity ^27^. In plants, Ca^2+^-stimulated protein kinase activities occur via activation of calcium-dependent protein kinases (*CDPKs*) ^28^. *NtCDPK2* was initially identified in the Cf-9/Avr9 pathosystem and is activated in response to race-specific elicitation ^29^. The *Arabidopsis* calmodulin-like protein CML36 is a Ca^2+^ sensor that interacts with ACA8 and stimulates its activity ^30^. The wheat calmodulin-like protein TaCML36 positively participates in the immune response to *Rhizoctonia cerealis* ^31^. *EDS1* family members control plant basal immunity and ETI ^32^. Remarkably, the homologs of *RPM1* (LOC107804213, LOC107804212, LOC107802041 and LOC107775646) increased sharply in BH at 24 hpi, whereas the homologs of pathogenesis-related protein 1 (LOC107807833 and LOC107808770) increased sharply in BH at 60 hpi. By profiling *P. nicotianae* genes during the early infection stage, some RxLR effectors were upregulated at 24 hpi. *Phtophthora* RxLR effectors affect various aspects of plant immune systems. Some of them inhibit the positive regulation of plant immunity ^33–35^, whereas some of them promote negative regulators of plant immunity ^36–38^. The specific recognition of RxLR effectors by one of the NB-LRR proteins will activate plant immunity ^11^. In *N. benthamiana* leaves infected with *P. nicotianae,* biotrophic growth was dominant before 24 hpi, followed by a rapid switch to necrotrophic growth ^39^. It is speculated that 24 hpi is a key time point of interaction between tobacco roots and *P. nicotianae*. With the spread of abundant hyphae invasion at 24 hpi, some effectors were recognized by LOC107804213, LOC107804212, LOC107802041, and LOC107775646 to trigger plant immune response. The pathogenesis-related *PR-1* proteins were activated during defense responses around 60 hpi and inhibited the growth of *P. nicotianae* by sequestrating sterol from this pathogen.

By profiling genes specifically regulated in susceptible tobacco before and after infection with *P. nicotianae*, 9 *S* gene homologs were identified. These genes included 1 calmodulin-binding transcription activator (*CAMTA3*), 1 cyclic nucleotide-gated ion channel (*CNGC4*), 1 protein trichome birefringence-like protein (*PMR5*), 1 plant UBX domain-containing protein (*PUX2*), 1 probable ADP-ribosylation factor GTPase-activating protein (*AGD5*), 2 callose synthases (*PMR4*) and 2 cellulose synthase A catalytic subunits (*CESA3*). By focusing on the upregulated genes, *CAMTA3* negatively regulates SA accumulation and plant defenses through calmodulin binding ^40^. Mutations in the *pmr5* gene can render *Arabidopsis* resistant to the powdery mildew species *Erysiphe cichoracearum* and *E. orontii* ^41^. Mutants of *PUX2*, a plant ubiquitin regulatory X domain-containing protein 2, results in significantly enhanced resistance to powdery mildew *Golovinomyces orontii* in *Arabidopsis* ^42^. *Arabidopsis* ARF-GAP protein, *AGD5*, is a susceptibility factor for *Hyaloperonospora arabidopsidis* ^43^. *CNGC4* is a cyclic nucleotide-gated ion channel gene, the mutation of which can enhance resistance to *Pseudomonas syringae* ^44^. In *Arabidopsis*, mutation in *CNGC4* lead to high expression of *PR-1*, elevated levels of SA, and elevation “SAR-like” resistance in response to virulent pathogens ^44^. *PMR4* (also known as *GSL5*) is a callose synthase gene. Silencing of the ortholog in tomato resulted in increased resistance to the adapted powdery mildew pathogen ^45^, and silencing of potato orthologs increased the resistance to late blight ^46^. In addition, we identified several *S* gene homologs that were sharply upregulated in both BH and XHJ cultivars. They were homologs of *ADH1* (LOC107772756, LOC107830380), *WRKY48* (LOC107832061), *bHLH25* (LOC107804684), *PLP2* (LOC107797353), *KMD3* (LOC107775293, LOC107797281), *PUB24* (LOC107808916, LOC107831963, LOC107791734). Alcohol dehydrogenase 1 (*ADH1*) of barley modulates susceptibility to the fungus *Blumeria graminis* f*.*sp*. hordei* ^47^. *WRKY48* negatively regulates PR gene expression and basal resistance to the bacterial pathogen *P. syringae* ^48^. *Arabidopsis bHLH25* and *bHLH27* transcription factors positively influence the susceptibility to the cyst nematode *Heterodera schachtii* ^23^. Patatin-like protein 2 (*PLP2*) promotes cell death and negatively regulates *Arabidopsis* resistance to the fungus *Botrytis cinerea* ^49^. Expression of the F-box/Kelch-repeat protein At2g44130 (*KMD3*) promotes susceptibility to the root-knot nematode *Meloidogyne incognita* ^50^. A homologous triplet of U-box type E3 ubiquitin ligases (*PUBs*), *PUB22*, *PUB23*, and *PUB24* in *Arabidopsis*, negatively regulates PTI in response to several distinct PAMPs^51^. These genes were not specifically regulated in XHJ, suggesting that they may not be key *S* genes determining different resistance in BH and XHJ. As pathogens can regulate *S* gene expression, further study of these sharply upregulated *S* genes in both BH and XHJ will contribute to revealing the interaction between tobacco and *P. nicotianae*. Further research on the *S* genes in XHJ will contribute to uncovering the differences in resistance mechanisms between BH and XHJ. Remarkably, all upregulated genes increased sharply in XHJ at 24 hpi, which confirmed the hypothesis that 24 hpi was a key time point of interaction between tobacco roots and *P. nicotianae*.

In this study, we identified candidate genes related to resistance and susceptibility to *P. nicotianae* in tobacco-resistant and -susceptible materials. Our results provide further insights into the molecular mechanisms underlying the interaction between *P. nicotianae* and tobacco, which will be useful in organizing resistant breeding practices. However, further reliable evidence is required to validate our results. For example, the use of VIGS to transiently silence genes to elucidate their function, and the application of CRISPR/Cas9 to fully knockout candidate genes would help to verify our results.

In this study, we provided insight into the *P. nicotianae* infection process in tobacco cultivars and investigated resistant and susceptible genes using the Illumina HiSeq platform. The resistant tobacco cultivar BH and susceptible tobacco cultivar XHJ were used as research objects, and the samples were collected at 0, 6, 12, 24, and 60 hpi. Thirty-eight defense-related genes were identified in BH, whereas nine susceptible genes were identified in XHJ. Our results provide a valuable resource for resistant breeding to *P. nicotianae* although further research is needed to explore the function of the identified resistant and susceptible genes.

## Materials and methods

### Plant growth condition and inoculation treatments

The resistant tobacco cultivar BH and the susceptible tobacco cultivar XHJ were cultivated in Hogland nutrient solution in a growth chamber under a 16 h light/8 h dark photoperiod, at 22 °C in the Tobacco Research Institute of Chinese Academy of Agricultural Sciences. A field-isolate of *P. nicotianae* race 0 was used for all inoculations throughout the study. Mycelial cultures of *P. nicotianae* were grown on oatmeal agar medium at 25 °C for 14 days. The roots of 8-week-old tobacco plantlets were laid on oatmeal agar medium and inoculated at 25 °C in the dark. The infected roots were harvested at five time points: 0, 6, 12, 24, and 60 hpi. Three independent experiments were performed for each treatment condition. Roots from all groups were sampled and immediately stored at − 80 °C.

### RNA-seq

Total RNA was isolated from samples using TRIzol reagent according to the method of kit instructions. After quality confirmation, RNA samples were sent to Novogene (Beijing, China) for RNA sequencing (Illumina Novaseq platform with 150-bp paired-end reads). According to the manufacturer’s instructions, cDNA library construction and Illumina sequencing were performed with three technical replicates performed per sample.

### Transcriptome data processing

The sequencing data were filtered with SOAPnuke (v1.5.2) by: (1) Removing reads containing a sequencing adapter; (2) Removing reads with a low-quality base ratio more than 20% (base quality less than or equal to 5); (3) Removing reads whose unknown base (‘N’ base) ratio is more than 5%. Thus, clean reads were obtained and stored in FASTQ format. De novo assembly of the transcriptome was performed with the short reads assembling program, Trinity v2.0.6 ^52^. The candidates that had the probable longest open reading frame were generated from the Trinity assembly result. A set of candidates was used as the reference transcriptome. If multiple transcripts belonged to one unigene, the coding sequences of a transcript were extracted and used for functional annotations of the unigene. Tgicl (v2.0.6) was used to perform clustering and to eliminate redundant data in the assembled transcripts to obtain unique genes. A transdecoder was used to identify coding region sequences of the unigene. All assembled unigenes were compared using the public protein databases, including the NCBI non-redundant database, Swiss-Prot, and KEGG databases, using the software BLAST (v2.2.23) with a cut-off E-value of 10^−5^. GO annotation was performed using Blast2GO (v2.5.0) with NR annotations.

### Screening of DEGs

The expression levels of the unigenes were calculated using the FPKM methods ^53,54^. The FPKM values of each unigene were calculated based on the length of the gene and the mapped reads count. DEGs were detected using the edgeR program package (3. 22. 5). The *p* value (< 0.05) threshold in multiple tests and analyses was determined using the false discovery rate (FDR). The DEGs were deemed significant according to the following criteria: *p* < 0.001, and the absolute value of log2 (ratio) ≥ 1. All DEGs were mapped to each term of the KEGG (KOBAS v2.0 procedure) or GO (GOSeq release2.12 procedure) databases, and significant pathways were defined based on a corrected *p*-value ≤ 0.05.

### qRT-PCR validation

With the isolated total RNA, first-strand cDNA was synthesized using a cDNA Synthesis Kit (Takara, Japan). The actin gene (GenBank no. X63603) served as an internal control. Primer sets were designed using Primer Premier 6.0 software, and the primers are listed in Supplementary Table S4. qRT-PCR was performed using the SYBR Green Quantitative RT-qPCR kit (New England Biolab). All independent samples were analyzed in triplicates.

## Supplementary Information

Supplementary Figure S1.

Supplementary Table S1.

Supplementary Table S2.

Supplementary Table S3.

Supplementary Table S4.

Supplementary Table S5.
