# Supplementary material for: Comparative transcriptome analysis reveals resistant and susceptible genes in tobacco cultivars in response to infection by Phytophthora nicotianae
Source: Sci Rep. 2021 Jan 12;11:809. doi: 10.1038/s41598-020-80280-7 (PMC7804271; doi:10.1038/s41598-020-80280-7)
Supplement: Supplementary file 1 — Supplementary Figure S1. [file 41598_2020_80280_MOESM1_ESM.docx]

Comparative transcriptome analysis reveals resistant and susceptible genes in tobacco cultivars in response to infection by *Phytophthora nicotianae*

He Meng, Mingming Sun, Zipeng Jiang, Yutong Liu, Ying Sun, Dan Liu, Caihong Jiang, Min Ren, Guangdi Yuan, Wenlong Yu, Quanfu Feng, Aiguo Yang, Lirui Cheng, Yuanying Wang

**Supplementary Figure S1.** Scatterplot of KEGG pathways enriched for upregulated DEGs in XHJ.

**Supplementary Table S1.** Total reads mapped to the reference genome.

**Supplementary Table S2.** FPKM values of unigenes.

**Supplementary Table S3.** Homologs of the 28 *S* genes in tobacco.

**Supplementary Table S4.** FPKM values of unigenes in the genome of *Phytophthora nicotinae.*

**Supplementary Table S5.** List of primers used in this study.


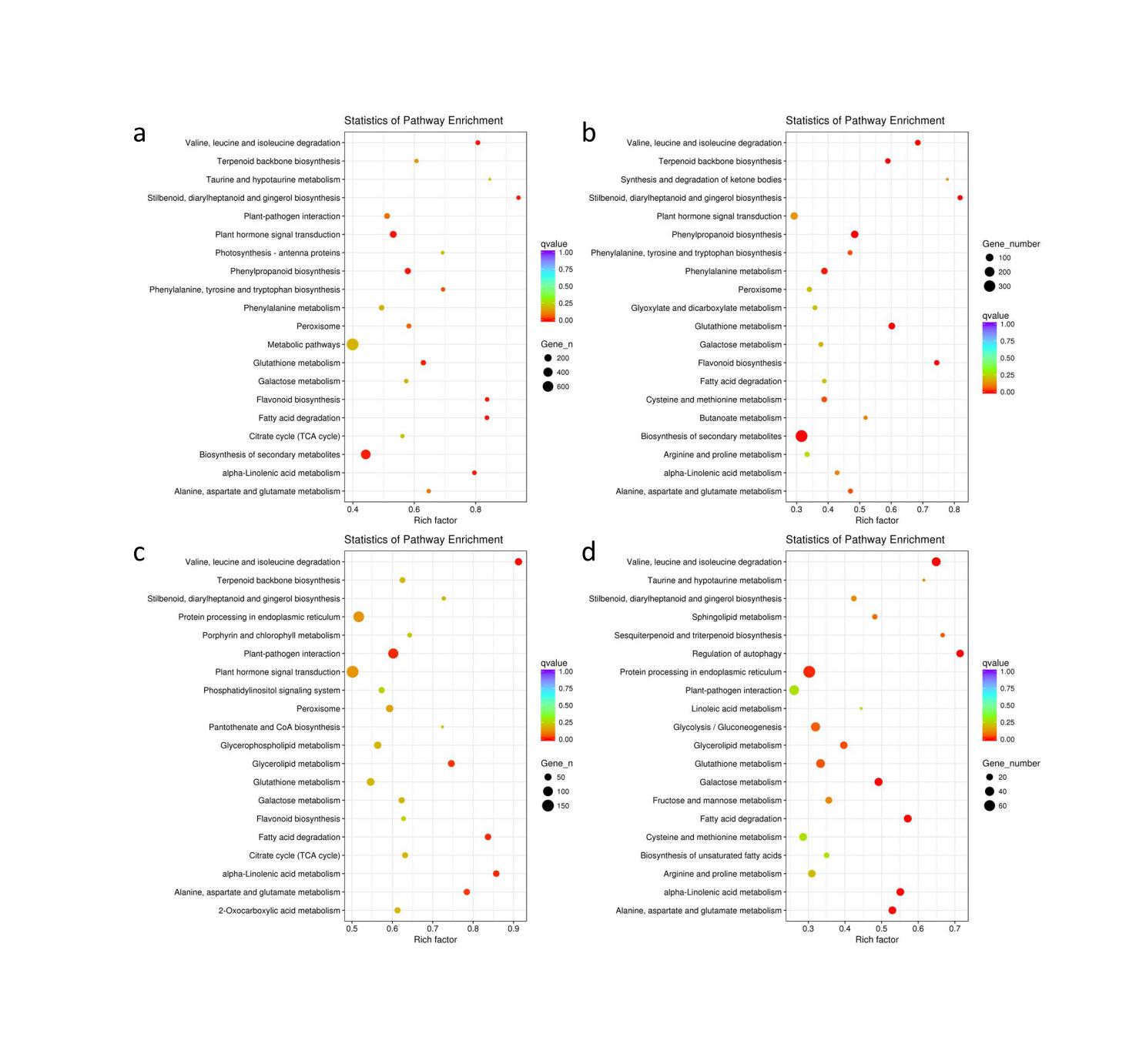


**Supplementary Figure S1.** Scatterplot of KEGG pathways enriched for upregulated DEGs in XHJ at **(a)** 6, **(b)** 12, **(c)** 24, and **(d)** 60 hpi. The rich factor is the ratio of the number of DEGs annotated in a given pathway term to the number of all genes annotated in the pathway term. A greater rich factor indicates greater intensity. The *Q* value is the corrected *p* value and ranges from 0 to 1, with a lower *Q* value indicating greater intensity. The size of the circles indicates the number of genes. The top 20 enriched pathway terms in the KEGG database are listed. The blue arrow indicates the significantly enriched pathway associated with plant-pathogen interaction. The top 20 enriched pathway terms in the KEGG database are listed.
