# Supplementary material for: Comparative transcriptome analysis reveals resistant and susceptible genes in tobacco cultivars in response to infection by Phytophthora nicotianae
Source: Sci Rep. 2021 Jan 12;11:809. doi: 10.1038/s41598-020-80280-7 (PMC7804271; doi:10.1038/s41598-020-80280-7)
Supplement: Supplementary file 3 — Supplementary Table S2. [file 41598_2020_80280_MOESM3_ESM.pdf]

Table S2. FPKM values of unigenes

geneID	BH0_1	BH0_2	BH0_3	BH6_1	BH6_2	BH6_3	BH12_1	BH12_2	BH12_3	BH24_1	BH24_2	BH24_3	BH60_1	BH60_2	BH60_3	XHJ0_1	XHJ0_2	XHJ0_3	XHJ6_1	XHJ6_2	XHJ6_3	XHJ12_1	XHJ12_2	XHJ12_3	XHJ24_1	XHJ24_2	XHJ24_3	XHJ60_1	XHJ60_2	XHJ60_3
LOC107766091	0	0	0	0	0	0	0	0	0	0	0	0	0	0	0	0	0	0	0	0	0	0	0	0	0	0	0	0	0	0
LOC107787595	6.49071	5.842451	6.524561	4.331545	4.136199	6.030636	4.48069	5.135036	7.498089	5.521825	5.281744	5.190174	7.552631	7.692988	3.947785	6.727848	5.986718	6.850413	2.412275	2.417713	2.314261	4.751356	5.772357	7.872892	1.283279	1.375144	4.293513	0	3.08628	1.651565
LOC107814016	0	0	0	0	0	0	0	0	0	0	0	0	0	0	0	0	0	0	0	0	0	0	0	0	0	0	0	0	0	0
107829445	0	0	0	0	0	0	0	0	0	0	0	0	0	0	0	0	0	0	0	0	0	0	0	0	0	0	0	0	0	0
LOC107776387	0.242561	0.503536	0.398279	0.341831	0.143089	0.144879	0.203447	0.195737	0.126515	0.131404	0.179533	0.034154	0.105009	0.058805	0.152141	0.431808	0.390558	0.371726	0.205112	0.21659	0.13519	0.461408	0.392251	0.253525	0.034075	0.150003	0	0	0.324382	0
LOC107812530	0	0	0	0	0	0	0	0	0	0	0	0	0	0	0	0	0	0	0	0	0	0	0	0	0	0	0	0	0	0
LOC107795607	0	0	0	0	0	0	0	0	0	0	0	0	0	0	0	0	0	0	0	0	0	0	0	0	0	0	0	0	0	0
LOC107811298	0	0	0	0	0	0	0	0	0	0	0	0	0	0	0	0	0	0	0	0	0	0	0	0	0	0	0	0	0	0
LOC107775718	9.570422	10.13067	9.262069	12.25634	9.644059	13.29166	6.730961	6.509352	8.730598	7.508196	9.410309	8.428929	8.601613	8.19297	6.149697	10.00081	10.45067	9.94475	6.547632	7.063861	8.184407	7.72081	8.585231	12.41166	1.611038	1.05627	3.460733	0	1.305253	1.676356
LOC107802209	0.264433	0.160665	0.153244	0.399271	0.428977	0.142149	1.127439	0.979068	0.475834	0.644639	1.205234	0.539884	0.466087	0.336564	1.515354	0.525482	0.127732	0.377863	0.872067	0.838845	1.085253	1.214592	0.874678	0.529738	0.752228	1.962342	0.208359	4.727674	0.848716	1.362524
LOC107762730	0	0	0	0	0	0	0	0	0	0	0	0	0	0	0	0	0	0	0	0	0	0	0	0	0	0	0	0	0	0
LOC107781485	0.256973	0.351299	0.670145	0.038801	0	0.644648	0	0.036594	0	1.127617	2.132248	0.289464	0.238389	0.046724	0.043958	0	0.041376	0	0	0.833297	0	0	0	0	0.487339	0.238373	1.417369	0	0	0
LOC107775940	0	0	0	0	0	0	0	0	0	0	0	0	0	0	0	0	0	0	0	0	0	0	0	0	0	0	0	0	0	0
LOC107778165	0.745301	1.05963	1.269834	0.810246	0.949668	1.346167	1.250232	1.248139	1.539387	1.075609	1.588721	1.737837	0.929241	1.365986	1.223931	1.177446	0.691223	1.200212	0.363014	0.630473	0.462214	1.145254	0.657405	1.838413	0.339223	0.290369	0.528532	0	0.430577	1.843324
LOC107793677	9.592605	8.931163	9.898395	6.627222	6.839387	6.677626	8.682443	8.255147	10.10741	3.701223	5.323003	4.431018	4.532652	5.345462	4.53322	10.38835	8.467211	8.798127	4.551829	4.261668	4.216959	9.048435	12.66153	13.41634	1.65778	1.728441	3.588908	0	2.325723	0
LOC107811248	0	0	0	0	0	0	0	0	0	0	0	0	0	0	0	0	0	0	0	0	0	0	0	0	0	0	0	0	0	0
LOC107764103	11.91554	12.18929	10.82298	21.45758	19.83929	25.98735	13.66538	13.04826	14.83968	15.2709	14.5278	16.9283	13.80596	13.63597	13.17822	8.625778	12.31017	9.173619	17.37166	16.58013	22.0892	10.76331	13.25585	12.50496	6.456373	5.549094	7.990069	17.39041	9.59997	15.03584
LOC107815888	6.153906	6.745139	6.593521	6.945757	6.621294	6.85799	6.276166	7.127211	5.931115	6.339754	6.881259	7.288951	6.12231	5.84374	8.708383	4.06789	3.792281	5.121487	10.16556	9.858124	8.91282	8.332135	7.664335	5.983313	12.53654	23.66836	9.303215	16.08307	8.366456	3.792412
LOC107782518	4.42721	4.909885	3.84848	5.315555	3.728692	6.422852	9.752983	6.790681	7.862219	5.565404	9.126077	5.407561	15.6166	8.670487	9.087934	2.981171	5.050019	3.181372	1.407266	1.94024	1.508053	4.944553	2.917057	2.062781	0.674378	0.296874	1.449999	0	2.311175	0
LOC107803020	0	0	0	0	0	0	0	0	0	0	0	0	0	0	0	0	0	0	0	0	0	0	0	0	0	0	0	0	0	0
LOC107824932	1.134771	1.933091	1.447382	1.566087	1.576562	2.200593	1.672289	1.549514	1.463643	1.416791	1.821935	0.958686	1.522975	1.064431	1.425939	1.232885	1.27046	1.589253	1.076156	1.507193	0.657713	2.081347	1.674475	1.596196	0.630193	0.782097	0.413644	0	0.561637	1.311488
LOC107818968	0	0	0	0	0	0	0	0	0	0	0	0	0	0	0	0	0	0	0	0	0	0	0	0	0	0	0	0	0	0
LOC107822802	0.226752	0.206657	0.065704	0.205426	0.133764	0	0.824143	0.710392	0.354808	0.073704	0.264998	0.127711	0	0.082459	0.387887	0.563253	0.146041	0.169053	0.230092	0.255756	0	0.757467	0.20001	0.237001	0.477806	0.946527	0.089334	0	0.727777	0
LOC107798537	0	0	0	0	0	0	0	0	0	0	0	0	0	0	0	0	0	0	0	0	0	0	0	0	0	0	0	0	0	0
LOC107765597	0	0.045335	0.172963	0	0.044016	0.05348	0	0.085004	0.046701	0.048506	0	0	0	0	0	0.074138	0.048056	0.074172	0.050476	0	0.090734	0	0	0.103983	0	0	0.117585	0	0	0
LOC107801493	0	0	0	0	0.030058	0.073041	0.028491	0.029024	0.095674	0	0	0	0	0	0	0	0	0	0	0	0	0.141841	0	0	0	0	0	0	0	0
LOC107808418	1.695327	1.438524	2.134341	2.224379	1.913968	1.696982	2.1084	1.773205	1.86607	1.824174	2.111082	2.420025	1.919801	2.040848	1.950042	1.786129	1.694305	2.24458	1.63132	1.582488	1.572825	1.611096	2.217303	2.413525	0.886928	0.610067	1.174606	0	0	0
LOC107779071	3.295039	2.951246	3.061846	2.882203	2.815127	3.237176	2.763691	2.863912	3.893578	1.274152	1.872288	1.871826	1.328099	2.231209	1.865897	3.471522	2.250253	3.091917	1.38355	1.393688	2.020692	2.988995	4.159219	7.184842	0.430958	0	0.33573	0	0.547017	0
LOC107781597	4.318379	3.057931	2.619611	2.730119	1.264565	1.00204	0.286634	0.132724	0.46668	0.636189	1.938823	1.916007	2.213424	0.88122	0.446415	4.12097	6.002758	4.400803	0.06305	0.078843	0.311675	0.207562	0.219229	0.389661	0	0	0.073439	0	0	0
LOC107800758	0	0	0	0	0	0	0	0	0	0	0	0	0	0	0	0	0	0	0	0	0	0	0	0	0	0	0	0	0	0
LOC107779684	0	0	0	0.484837	0.059194	0.071922	0.280544	0.743054	0.314026	0.456626	0.750523	0.395611	0.372349	0.291922	0.205981	0	0	0.099748	0.746699	1.075203	0.366063	1.117336	0.649076	0.629279	0.169154	0.372325	0.316264	0	0	0
LOC107795295	2.255804	2.590418	2.490371	3.351576	3.852441	2.861812	3.443512	3.353711	3.430914	4.531369	3.558985	3.373205	4.97216	4.552796	3.496096	3.227573	2.985636	2.606786	5.035874	3.740179	4.258675	3.372146	3.163731	4.338288	6.417049	5.147609	5.279033	0	7.16776	5.57919
Novel01700	0	0.278192	0.159205	0.221228	0.378139	0.328174	0.051204	0.156485	0.11463	0	0.599303	0.154727	0.271841	0	0.375952	0.136481	0	0.045514	1.858439	1.600932	0.668129	0.9177	0.053849	0.127616	0.617473	1.019337	0	0	0.29391	0
LOC107828529	1.59362	2.369685	2.223771	2.089622	2.245091	2.367109	2.268766	2.114113	1.732396	4.232552	5.131435	1.824637	1.4239	1.669933	2.474993	2.781407	1.985236	2.391855	2.829916	4.363563	0.994437	3.187092	1.886568	1.534152	4.957517	5.631043	2.230496	0	1.615211	5.1861
LOC107780885	2.203679	3.862273	1.719139	1.996422	2.249954	2.308475	3.033142	2.607069	1.856715	1.157081	1.584842	1.28889	2.453167	1.47945	0.985911	2.652759	3.275292	1.390177	1.662772	1.959759	2.370525	7.786097	2.940607	1.299289	0.571512	1.729685	0.400703	0	2.448299	3.493764
LOC107827812	5.494835	5.481982	7.094157	5.213689	4.660799	6.327096	4.008799	4.667156	5.891403	4.280186	3.556599	6.977019	3.655678	5.852711	2.502847	4.967014	5.685406	4.896567	2.540444	2.915472	3.232193	3.013998	4.215863	4.519797	1.315435	0.497647	4.457731	0	1.095732	0
LOC107766627	1.634435	1.276788	1.217811	4.336381	3.236859	5.187977	3.688297	4.256018	5.041863	2.466566	2.510433	3.813683	4.288765	5.603993	3.554786	1.507979	1.654193	1.827811	2.44828	2.139769	3.12323	2.632422	3.226617	5.653697	0.93481	0.487328	1.195858	5.565964	1.686161	0
LOC107798260	6.612154	3.196487	7.09729	0.208357	0.457894	0.247265	0	0.147381	0.377865	14.29704	0.403169	0.1943	5.504555	5.896281	0.53112	3.127818	2.999534	1.800391	0.350064	0.291831	0.209753	0.336121	0.862175	1.382204	64.26121	1.200043	83.65485	28.78294	93.83893	3.555126
LOC107771161	0	0	0	0	0	0	0	0	0	0	0	0	0	0	0	0	0	0	0	0	0	0	0	0	0	0	0	0	0	0
107791553	0	0	0	0	0	0	0	0	0	0	0	0	0	0	0	0	0	0	0	0	0	0	0	0	0	0	0	0	0	0
LOC107767180	0	0	0	0	0	0	0	0	0	0	0	0	0	0	0	0	0	0	0	0	0	0	0	0	0	0	0	0	0	0
LOC107775703	0.415712	0.315725	0.421598	0.156923	0	0.148981	0.261507	0.177598	0.260193	0.033781	0.024291	0	0.077129	0.188967	0	0.567947	0.267742	0.309931	0.035153	0.205137	0	0.144655	0.4278	0	0	0	0	0	0	0
LOC107794746	0	0	0.104914	0.10934	0.035598	0.129757	0	0	0	0.03923	0	0	0	0.043889	0	0	0	0.029993	0	0.102096	0	0.033597	0	0.042049	0	0.055977	0	0	0	0
LOC107807054	85.93226	102.5689	90.19664	36.8288	26.99042	90.86109	5.028494	6.021213	4.295531	27.99998	38.16689	9.241356	2.517394	6.999535	9.985756	77.16554	76.71778	82.61161	20.59882	34.92279	11.51114	21.7401	37.89897	9.784158	2.859066	2.048909	12.92872	0	2.278688	6.503453
Novel01355	10.03486	13.76856	11.79055	16.58372	10.53831	15.05676	11.83883	16.58279	11.38823	7.74218	9.8201	8.943537	3.191699	10.34603	7.017245	10.10761	10.22718	9.8656	17.12033	16.13805	17.29821	12.34033	15.56292	15.55976	2.788377	6.597795	8.471712	15.77218	2.654472	20.45507
LOC107812162	0.421568	0.57631	0.389363	0.596748	0.489603	0.6232	21.4141	13.66504	4.873117	0.231233	3.584191	0.089038	7.450055	5.173981	7.92355	0.510498	0.40727	0.353584	0.13368	0.31204	0.144179	21.67382	2.277584	1.156633	0.099936	0	0.062282	0	0.126849	0
LOC107816779	3.149864	3.677093	4.830149	0.705389	0.563705	1.445917	0.32653	0.272157	0.697774	0.310603	0.397067	0.657798	1.063761	1.274151	0.508551	2.79564	4.410689	3.562115	0.502783	0.419146	0.710113	0.620688	0.998971	0.850803	0	0.049245	0.627453	0	0.340777	0
LOC107799388	0.022337	0.081429	0	0	0.03953	0.024015	0	0.057255	0	0.043562	0.015663	0	0	0	0	0	0	0.033306	0	0.094477	0	0.018654	0.059108	0.070039	0.056481	0	0	0	0	0
LOC107769681	4.052381	4.891055	3.498847	6.548719	5.354491	6.829586	6.108845	6.152893	6.323785	5.526876	6.124653	5.204743	7.041078	7.139234	3.878222	4.223694	3.862036	5.103415	5.251232	5.049404	5.244097	3.750114	4.951121	4.321403	6.300448	3.428874	4.595416	5.874376	11.46848	5.079017
LOC107788394	3.831012	2.317364	3.595456	3.132847	3.059938	3.535612	2.616085	2.404297	2.100741	2.975352	2.187082	2.176791	2.528649	3.032872	1.809437	2.172736	2.390963	2.67925	2.029728	1.92152	2.62819	1.557219	2.631594	2.870247	1.714536	1.084984	2.40422	2.424527	3.101179	0
LOC107784968	19.35154	21.87797	20.55779	12.34735	9.769861	14.90832	12.38732	10.38731	13.95487	7.455465	12.37055	7.462239	9.542097	8.185595	8.480907	17.94194	18.02999	20.41078	8.023302	10.64557	6.193368	11.26311	13.32037	13.77369	2.701793	3.171682	4.742776	1.698048	2.057637	4.404424
LOC107807681	1.185398	0.337607	0.611824	0.201358	0.098336	0.119479	0.124281	0.126604	0.069556	0	0.15585	0.187773	0.123713	0.040413	0.038021	0.77294	1.46728	1.325646	0.300715	0.094009	0.236491	0.340298	0.424775	0.348462	0	0	0	0	0	0
LOC107776284	2.963059	4.530285	3.434299	3.535169	3.195005	4.78718	5.092716	3.541639	4.788431	3.3946	7.686351	3.542886	3.821235	3.214888	4.686444	4.235161	3.80111	3.452452	1.823741	2.972241	1.786806	4.516352	4.570254	4.738562	0.614132	1.013823	0.880309	1.157927	0.77952	0
Novel01018	0.026133	0.031756	0.030289	0.039459	0.038541	0.074924	0.058451	0.044658	0.057248	0.025483	0.036649	0.036797	0.048487	0.05702	0	0	0.008416	0.032472	0.044197	0.022107	0.039723	0.01455	0.069154	0.072839	0.011013	0	0.010296	0	0	0
LOC107812683	0	0	0	0	0	0	0	0	0	0	0	0	0	0	0	0	0	0	0	0	0	0	0	0	0	0	0	0	0	0
LOC107801796	5.362865	6.628375	5.044988	4.725375	3.77033	4.502013	3.820275	3.452325	5.241807	3.15201	2.575647	4.468633	5.152205	4.327857	1.960435	5.091321	5.464868	4.381636	3.05639	3.542288	3.283015	3.987855	6.091202	4.683851	1.486091	2.248832	2.691681	0	2.122088	9.084767
LOC107830854	22.97196	23.2374	23.28829	15.95729	15.2317	16.0899	20.01658	19.47	17.86657	10.29945	12.8475	9.573599	16.38573	14.41191	15.29712	25.03557	25.13801	26.63113	12.12389	10.26338	17.29987	22.32189	19.36644	21.82168	2.219144	3.89907	5.896063	2.202171	5.930037	3.808018
LOC107825886	31.71673	39.49038	34.89894	32.92631	29.89084	29.59926	29.79148	28.66114	27.31409	23.82821	27.13833	20.61933	21.49295	22.57451	24.11235	36.50738	35.76685	37.09547	28.35537	32.0416	29.43697	35.2756	30.10827	31.67149	14.49697	27.0285	16.75749	10.53994	24.83432	25.51608
LOC107826539	2.252552	5.522653	3.888612	9.168769	6.288425	5.83271	6.599288	7.454597	2.353082	12.74602	16.30657	5.655449	8.264393	6.956931	32.07434	9.953414	2.697206	5.440222	6.3743	8.158777	4.224469	4.053778	4.085737	3.150209	2.607242	1.103613	3.862261	0	1.832888	1.961671
LOC107811031	0	0.09644	0	0	0	0	0	0	0	0	0	0.089398	0	0	0	0.039428	0	0.039446	0	0	0	0	0	0	0.133786	0	0	0	0	0
LOC107807976	0.107904	0.140488	0.200997	0.111721	0.02728	0.182302	0	0.14488	0.043417	0.165346	0.010809	0.065115	0.171601	0.554957	0.759428	0.091897	0.07446	0.126417	0	0.03912	0.014059	0	0.013597	0.048335	0	0	0.018219	0	0	0
LOC107801829	5.939281	7.507754	8.31313	0.805583	0.608684	1.515192	0.576957	0.802775	0.535554	0.638052	0.694105	0.326007	1.531522	1.555803	0.740464	6.263858	4.035942	7.042249	0.74909	0.539323	0.153015	0.574469	1.30231	0.718975	0.042424	0.116724	0.674214	0	0	0
LOC107832292	0	0	0	0	0	0	0	0	0	0	0	0	0	0	0	0	0	0	0	0	0	0	0	0	0	0	0	0	0	0
LOC107791522	1.998701	1.758757	1.917163	2.122916	1.219712	2.222947	1.44517	1.354418	1.294117	1.612954	1.691454	2.096142	1.764641	2.030107	1.27329	1.489435	1.930914	1.2332	0.979104	1.282648	2.137134	0.748248	1.459024	1.296647	0.522821	0.287695	0.407294	0	1.659045	0
LOC107807745	2.032957	2.004038	2.362286	2.123658	2.23944	2.007255	2.018316	1.737014	2.356573	1.173251	1.556425	1.59482	0.923721	1.425765	1.10716	2.071479	2.164416	2.536404	1.157752	1.509161	1.0784	2.546663	2.323844	2.818673	0.131137	0.432966	0.367774	0	0.499356	1.282661
LOC107774468	0.310859	0.519401	0.225186	0.985679	1.879635	0.946933	2.390034	3.718491	1.945655	1.566156	2.325062	2.18852	2.249343	1.752182	2.39293	0	0	0	0.210292	0.087655	0.047251	0	0	0.108303	0	0.144179	0.061235	0	0	0
LOC107773202	0.905702	1.788444	0.131218	0.136753	0.400712	0.32458	1.392694	1.289761	0.141719	0	0.740925	0	1.00824	0	0.929588	5.399441	1.604138	2.250797	0.612696	0.383081	0.413008	2.143063	0.399444	0.157773	0.572541	0.630109	0.713645	0	1.453457	9.333486
LOC107765569	1.001332	1.011632	0.843446	0.794641	1.030284	0.959719	0.859392	0.829036	0.787056	0.961287	1.567557	0.373793	0.967799	1.236364	0.796698	0.613152	0.457439	0.596071	0.401707	0.512175	0.226538	0.434325	0.581981	0.332638	0.215905	0.259215	0.348626	2.775558	0.186852	0
LOC107790939	0	0	0	0	0	0	0	0	0	0	0	0	0	0	0	0	0	0	0	0	0	0	0	0	0	0	0	0	0	0
LOC107784601	0	0	0	0	0	0	0	0	0	0	0	0	0	0	0	0	0	0	0	0	0	0	0	0	0.24347	0.178634	0	0	0	0
LOC107824131	0	0	0	0	0	0	0	0	0	0	0	0	0	0	0	0	0	0	0	0	0	0	0	0	0	0	0	0	0	0
LOC107778431	0.372504	3.577716	5.106229	6.411921	13.61567	3.820031	13.651	21.17768	24.4537	12.07069	14.58701	23.04584	13.71622	22.101	38.90919	11.16767	1.024245	5.981605	24.74388	21.98516	23.70268	23.04441	17.66712	19.82643	54.16017	115.5835	27.12737	32.78681	46.07571	79.72778
LOC107820557	0	0.103665	0.148315	0.103048	0	0.061145	0.047702	0.097187	0.053395	0.221832	0.159517	0	0.126623	0.062045	0.058373	0.25429	0.274719	0.169604	0.115421	0	0.311213	0.094992	0	0.118887	0	0	0	0	0	0
LOC107784780	0	0	0	0	0	0	0	0	0	0	0	0	0	0	0	0	0	0	0	0	0	0	0	0	0	0	0	0	0	0
LOC107778702	0	0.117871	0	0.058584	0.057221	0.139049	0.108477	0.165758	0	0.126116	0	0	0.143975	0.211644	0.066372	0.192758	0.062473	0.144635	0.328095	0.273517	0.058977	0.108009	0	0	0.40879	0.179957	0	0	0.311327	0
LOC107763966	6.107887	5.397902	4.987672	5.281917	4.09444	7.760676	3.104817	3.953595	4.517976	4.692576	4.86688	5.472847	4.326882	3.02882	2.18464	2.689585	4.738467	2.552826	3.568466	2.505141	5.739299	2.782285	2.367261	6.964322	0.936027	2.060286	3.281378	0	0.891077	0
LOC107832747	13.06196	11.46346	13.03663	6.414777	6.888147	8.700164	9.959659	10.37136	11.39605	4.031257	5.458425	4.83018	3.525031	4.318167	3.385463	11.43802	13.1288	13.11548	6.337106	5.84099	6.056637	10.79819	12.56892	17.05395	3.725444	1.591056	4.678237	0	2.117337	5.438656
LOC107763097	0	0	0.025268	0.052667	0	0	0.02438	0	0	0	0	0	0	0	0	0.064984	0.028082	0	0.029496	0.024589	0	0	0.02564	0	0.03675	0.080891	0.034356	0	0	0
Novel04255	14.30355	15.43919	13.05893	9.085283	8.025352	11.03956	8.444818	8.807559	10.2404	5.181713	4.818719	4.388058	5.648631	4.751082	3.649689	12.59432	11.73409	14.62563	6.811159	6.523087	7.652141	7.640968	11.48893	10.64879	1.818506	1.612201	3.447241	11.42912	1.92353	2.470419
LOC107815585	11.80091	11.88285	10.86171	21.96993	19.49571	20.86449	15.0241	12.04289	14.91869	19.29179	19.50188	17.22394	19.38623	16.87477	12.23604	13.23607	11.34236	11.52076	16.44614	19.26341	13.23996	12.82947	14.50897	17.27119	10.91331	8.567015	17.21171	3.237592	7.483148	4.665398
LOC107783006	0.018621	0.067882	0.145678	0.084347	0.164767	0.020019	0.702806	0.811407	0.419564	0.290519	0.600612	0.298893	0.538947	1.239164	1.853833	0.138761	0.017989	0	0.113369	0.236276	0.169823	1.026337	0.21352	0.253009	0.4473	0.958641	0.066024	0	4.571957	26.48082
LOC107813064	16.60435	18.45395	17.13326	15.50202	14.35615	14.10421	17.32875	14.62041	17.99863	14.61547	15.06477	14.37584	16.68516	15.90207	12.26132	16.31707	17.29633	17.55302	10.83606	10.10572	9.681404	14.23721	17.30145	19.77262	7.37157	7.627763	11.49786	2.266118	11.89936	0
LOC107823184	0	0	0	0	0.140452	0.056883	0	0	0	0	0	0.357591	0	0	0	0	0	0	0	0	0	0	0	0	0	0	0	0	0	0
LOC107804641	0	0	0	0	0	0	0	0	0	0	0	0	0	0	0	0	0	0	0	0	0	0	0	0	0	0	0	0	0	0
LOC107766987	0	0	0	0	0	0	0	0	0	0	0	0	0	0	0	0	0	0	0	0	0	0	0	0	0	0	0	0	0	0
LOC107786820	0	0	0	0	0.128747	0	0	0	0	0	0	0	0	0	0	0	0	0	0	0	0	0	0.12834	0	0	0	0.171969	0	0	0
LOC107810187	23.95124	20.71935	23.57872	18.82384	16.30871	21.91832	13.0888	14.41064	18.16059	12.20759	9.1136	13.18375	11.32325	11.57102	9.458393	17.3626	22.25703	20.38447	14.24721	11.65657	14.03404	13.03236	17.3307	19.17292	6.649873	5.745932	9.658779	12.4345	6.906032	32.25281
LOC107788014	14.80018	13.66448	11.29925	13.56367	14.72845	13.81346	9.642987	7.898965	11.67991	15.45693	12.42339	16.99765	10.84058	12.2384	3.874678	8.759351	15.23071	13.72074	7.879113	7.856711	10.8571	5.284354	6.489528	7.667314	7.620364	2.298101	14.14621	1.533945	8.467787	1.326257
LOC107817506	1.697827	1.81074	1.695697	1.489055	1.134753	1.84479	1.166501	1.466102	1.509208	0.757347	1.190517	1.03763	1.508014	1.261103	1.019608	1.130624	1.15166	1.198472	0.788106	0.779239	1.037789	1.025715	1.497595	1.906717	0.730747	0.251319	0.789869	0	0.521741	0
LOC107788296	0.460819	0.503976	0.74775	0.612302	0.598052	1.354194	0.773018	0.971216	0.548007	0.389443	0.60317	0.337404	0.957582	0.335154	0.630629	0.618125	0.296793	0.34356	0.65465	0.701678	0.476311	0.538779	0.406473	0.577977	0.194205	0.128239	0.326791	0	0.443709	0
LOC107775793	1.508688	1.503888	3.770478	1.836631	2.628254	1.419271	1.739927	0.886231	0.929527	4.919196	4.826637	3.305946	10.23441	10.33834	7.403651	2.002617	1.320877	2.249582	0.861138	1.515542	1.289952	0.354359	0.873313	1.18266	5.185853	1.377619	8.525676	3.371641	22.92498	0
LOC107770771	12.15756	42.7154	16.54385	109.2149	82.19166	19.54521	283.8025	284.2554	58.99038	162.6483	325.1408	44.44386	62.61039	40.96748	501.5181	48.72832	13.00756	17.06908	78.40114	202.5654	53.67786	82.83787	151.4858	30.40198	147.6751	178.9656	29.67856	186.8884	3.008593	38.63982
LOC107787945	21.02681	20.33362	23.30116	13.66891	13.16143	13.40277	12.38565	12.80012	15.37077	6.104183	8.028546	6.463757	7.564203	8.346839	5.656178	16.54636	21.089	16.91294	6.623561	6.200639	10.24715	9.830047	15.33788	18.78968	1.014667	0.893352	2.339755	0	3.091009	6.616383
LOC107791197	0	0	0	0	0	0	0	0	0	0	0	0	0	0	0	0	0	0	0	0	0	0	0	0	0	0	0	0	0	0
LOC107761002	13.59577	13.65673	13.55994	14.18037	15.38666	14.87557	16.17529	15.65615	13.26585	16.7478	16.8379	15.29973	13.70773	15.6191	18.69715	12.30121	13.85719	12.12762	23.41804	23.43148	24.0196	15.92708	16.75161	14.18237	25.76689	38.61552	17.93173	32.47333	17.10314	52.85009
LOC107761625	0.352133	0.240694	0.612203	0.478522	0.389488	0	0.443023	0.150436	0.661196	1.287652	0.679018	0.743729	1.567997	1.056439	0	0.459217	0.085047	0.459427	0.17866	0.14894	0.321151	0.147038	0.310604	0	0.445203	0.367475	0.104048	0	1.27147	5.443226
LOC107805376	1.001619	0.703345	0.542395	51.23183	90.08281	40.72661	44.74572	54.19667	31.15535	74.98214	47.30982	73.61903	86.75194	92.05696	80.43949	2.679727	2.395326	3.293044	112.1011	78.11837	114.9654	47.37759	26.99727	32.05893	233.6314	207.5441	153.8013	140.9114	187.6687	237.5738
LOC107769017	0	0	0	0	0	0	0	0	0	0	0	0	0	0	0	0	0	0	0	0	0	0	0	0	0	0	0	0	0	0
LOC107769593	0	0.055899	0	0.166698	0	0	0	0.26203	0.057584	0.059809	0.043008	0	0	0.066913	0.125904	0.228533	0	0.137183	0.062238	0.051885	0	0.051222	0.486911	0	0.077546	0.170685	0.072493	0	0	0
LOC107818411	0	0	0	0	0	0	0	0	0	0	0	0	0	0	0	0	0	0	0	0	0	0	0	0	0	0	0	0	0	0
LOC107799945	1.959787	2.861826	2.129502	2.037753	1.931211	1.819691	1.979981	1.959921	2.007201	1.67216	2.217465	0.9031	1.264372	1.141903	1.920041	3.684281	2.323619	2.889002	1.10732	1.827397	0.771814	3.217543	1.709018	2.444076	1.238883	1.7353	1.105512	0	0.107217	0
LOC107792628	0	0.337369	0.241339	0	0	0	0	0	0	0	0	0	0	0	0	0.448264	0.044703	0	0	0	0	0	0	0	0	0	0	0	0	0
LOC107826267	0	0.076258	0.072736	0	0.07404	0.08996	0.070181	0.142986	0	0	0	0	0.093147	0	0.08588	0.124708	0	0	0	0	0	0	0.073806	0.174912	0	0	0	0	0	0
LOC107766044	0.478469	0.688524	0.415923	0.319398	0.378814	0.135372	0.591408	0.6455	0.401923	0.19645	0.741638	0.212749	0.140168	0.082419	0.336007	0.562983	0.340599	0.413043	0.204428	0.127816	0.068901	0.420613	0.599743	0.184246	0	0.03504	0.029764	0	0	0
LOC107780177	10.48126	8.028832	6.827586	11.34651	11.01201	18.17246	14.4886	10.72389	16.49179	17.6725	22.58798	17.26137	31.57713	33.23253	24.51116	11.90382	15.20148	14.67887	5.762103	7.205373	5.348247	12.67548	14.32415	15.75303	1.375472	2.732175	3.669359	1.897616	4.08794	0
LOC107828333	17.45992	14.80753	15.17754	13.00575	13.51848	16.99868	11.22736	10.35988	15.48147	9.884323	9.658293	11.34979	12.14789	11.64039	6.570807	12.50516	13.86904	15.94596	7.825062	9.477347	8.138801	10.28787	14.63076	18.09703	3.311197	2.969292	6.821418	3.46842	7.004856	5.997629
LOC107822610	3.626291	3.819016	4.588287	2.664692	2.317453	3.032327	1.520763	1.480347	2.269681	1.964495	2.344988	2.042394	2.915494	2.505533	1.157932	4.113526	3.114046	4.746239	1.144799	0.852109	1.065666	1.783397	2.594443	2.821592	0.509415	0.224254	1.190551	0	1.939806	0
LOC107832274	0.173827	0.528072	0.705151	1.94223	2.563554	1.557374	1.555156	1.930789	1.876755	0.904016	1.625165	1.639865	0.677272	0.410879	1.338082	1.057876	0.503793	0.367186	4.733058	6.200413	3.249923	2.467842	3.398739	1.695713	0.586053	1.854314	0.547864	0	0.139477	1.791327
LOC107829009	3.702939	3.661117	3.257903	4.432238	3.356037	3.812217	1.938781	1.246382	1.474871	1.903894	2.690922	0.929024	2.173505	3.109364	2.372493	2.558749	2.666752	2.258753	1.662403	1.196019	1.24852	1.274452	1.32629	0.98517	0.198615	0.156132	0.159148	0	0.756308	0
LOC107766434	294.161	221.0297	312.2812	105.2374	89.2527	168.5453	83.58964	102.3417	134.8217	37.33584	48.10487	53.88547	30.23858	52.39019	33.77913	152.6105	242.2687	209.8404	46.94253	39.76107	82.77527	85.47443	140.6516	167.5365	7.853105	5.933806	33.091	39.7794	6.694907	34.39348
LOC107764940	0	0	0	0	0	0.167603	0	0	0	0	0	0	0	0	0	0	0	0	0	0	0	0	0	0	0	0	0	0	0	0
Novel00687	0.234887	1.605528	1.225093	4.149513	1.662747	2.777853	0.591029	0.200694	0.110261	0.801655	0.494108	0.297658	0.522959	0.384375	0.120541	3.150683	1.474983	1.83874	1.072561	0.695443	0.963993	0.4904	0.932336	0.613759	0	0	0	0	0	0
LOC107832165	1.063314	0.463473	0.843941	0.293181	0.204541	0.198816	0.542863	0.711019	0.824671	0.270487	0.291755	0.742088	1.389556	1.412202	0.949005	0.757931	0.357305	0.585942	0.23456	0.039108	0.210817	1.621567	0.407788	0.096641	0.1169	0.128654	0.218565	0	0	0
LOC107771033	0	0	0	0	0	0	0	0	0	0	0	0	0.134977	0	0	0	0	0	0	0.102569	0	0	0	0	0	0	0	0	0	0
LOC107781984	264.5922	259.7473	340.0467	260.6406	208.0377	299.5574	249.6878	268.7704	301.9127	185.1623	163.8131	181.6183	159.4645	207.0011	226.9754	303.975	271.2307	309.0287	202.5362	204.4001	239.1793	203.4467	241.3279	269.9583	120.619	138.3685	187.4852	207.8236	164.8179	122.712
LOC107820584	0	0	0.028709	0.119681	0.146119	0.284059	0.332407	0.19753	0.062013	0	0	0.139508	0.036765	0.07206	0.067795	0.024611	0	0.073868	0.201077	0.055876	0.150603	0.137906	0.116526	0.034519	0	0.091907	0	0	0.318001	2.042068
Novel01027	0	0	0	0	0	0	0	0	0	0	0	0	0	0	0	0	0	0	0	0	0	0	0	0	0	0	0	0	0	0
LOC107772139	0	0	0	0	0	0	0	0	0	0	0	0	0	0	0	0	0	0	0	0	0	0	0	0	0	0	0	0	0	0
LOC107788124	0	0	0	0	0.02342	0	0.0222	0	0.024849	0	0	0.067082	0.058929	0.086625	0	0	0	0.019733	0	0.02239	0	0	0.023346	0	0.133853	0.036828	0.093848	0	0	0
LOC107778989	114.958	98.13099	116.9977	61.46945	61.08253	91.89357	49.28661	55.54324	74.05775	29.09014	32.4964	30.53673	25.56367	31.71289	16.37757	70.36363	115.9518	95.6887	38.57874	36.38675	48.27741	48.26292	86.16291	111.8876	8.683669	3.089065	23.2875	0	2.672051	17.15878
LOC107773118	0	0.226245	0.071931	0	0	0	0.069405	0	0	0	0	0	0.092117	0	0.084931	0	0	0.123385	0	0	0.075468	0	0	0.172977	0	0	0	0	0	0
LOC107779128	3.840032	4.286169	3.863146	4.964237	6.414038	5.752073	3.944576	4.718781	3.362145	4.333541	5.264248	4.410607	5.283432	5.554334	3.055617	3.504652	3.876526	3.892267	5.604098	6.204814	3.816976	3.711369	3.958032	4.28418	6.109622	4.442598	6.680424	0	4.56989	10.67123
LOC107801566	3.140113	5.236533	4.007335	11.16729	6.266582	4.166135	27.93454	27.08686	11.25914	17.13421	33.3791	7.873899	13.49903	6.450538	39.2237	6.049191	3.549973	3.412011	5.965969	14.9206	4.509019	15.95753	19.00546	4.15493	2.829722	6.042564	1.776715	4.777925	0.321652	2.06551
LOC107817092	0	0	0	0	0	0	0	0	0	0	0	0	0	0	0	0	0	0	0	0	0	0.072906	0	0	0.110373	0	0	0	0	0
LOC107816398	0.330889	0.693599	0.258871	0.269792	0.146396	0.46247	0.055506	0.084816	0.155327	0.096798	0.139212	0	0.110505	0	0	0.197263	0.6713	0.370038	0.302187	0.251919	0.090533	0.0829	0.233493	0.138338	0	0	0	0	0	0
LOC107817051	1.487549	2.0952	1.449834	2.123561	1.555605	2.229323	4.007672	2.696066	2.962435	1.75824	2.117749	1.142475	1.806502	1.426135	2.267037	1.679581	1.65484	1.915599	1.41798	0.953306	0.822224	3.463357	2.624233	2.59131	0.626905	0.313609	0.69261	0	0.434036	0
LOC107824052	2.106142	2.399361	1.501276	2.785765	2.870026	3.351254	1.660522	1.511618	2.27394	5.298687	5.892545	7.259657	3.821677	2.987007	2.788576	1.22421	2.563735	1.821454	1.410531	1.425321	1.517461	0.914625	1.876334	3.324023	0.878725	0.586109	1.443789	0	1.520958	0
LOC107791212	4.929417	5.79574	6.411202	9.613406	9.090009	9.588054	8.900253	9.516802	5.723119	8.219268	10.65977	7.311733	8.042742	7.799793	8.187762	7.150526	5.162114	6.648826	10.50045	11.61856	7.378458	7.070642	6.671469	7.197384	11.37035	9.162685	7.51621	8.073046	5.072482	13.96
LOC107787265	0	0	0	0	0	0	0	0	0	0	0	0	0	0	0	0	0	0	0	0	0	0	0	0	0	0	0	0	0	0
LOC107804409	4.294043	5.861616	4.998886	5.912397	4.352024	5.389448	6.298832	5.155921	5.239092	4.888181	6.128086	5.082014	6.591186	5.57198	6.562394	5.948761	5.080428	5.697631	3.992591	4.096525	3.398675	6.240074	5.906701	5.951225	3.420016	1.368688	3.376023	0	6.466021	7.01781
LOC107770728	0	0.021095	0	0	0	0.024885	0	0	0	0.180563	0	0.019555	0.051533	0	0	0	0	0	0	0.023487	0	0	0	0	0.087791	0.096619	0.054714	0	0.111434	0
LOC107770308	0	0	0	0	0	0	0	0	0	0	0	0	0	0	0	0	0	0	0	0	0	0	0	0	0	0	0	0	0	0
LOC107760019	0.018232	0.033232	0.110939	0.016517	0.032265	0.078405	0.030583	0.062311	0.102701	0.017778	0.063921	0.015403	0.040592	0.05967	0.07485	0	0	0	0	0	0	0	0	0	0	0	0	0	0	0
LOC107819581	0.333466	0.44418	0.535153	0.929548	0.907915	0.827345	0.666958	0.460259	0.650227	0.400211	0.791412	0.628455	0.571106	0.86751	0.579209	0.573459	0.247812	0.286861	0.442494	0.542479	0.210549	0.556972	0.565651	0.375349	0.324309	0.107075	0.39413	0	0.246988	0
LOC107783518	17.83908	9.582938	13.14368	5.554663	5.057154	5.100532	5.16587	4.693553	12.16387	4.220336	6.867181	7.47688	18.62334	15.95061	5.552661	16.31218	13.48169	17.66411	1.435762	1.31427	1.669969	5.93135	4.771965	7.742337	0.596296	0.424634	2.983945	0	0.534269	1.715423
LOC107763444	1.514742	1.71182	2.291115	0.960595	0.991854	1.367967	0.762287	0.93185	0.910148	0.738532	0.552312	0.793411	0.573319	0.594903	0.497501	1.693181	1.55117	2.055333	1.045197	1.25574	0.856511	0.8602	1.630045	1.424887	0.383019	0.295071	0.859345	0	0.14585	0
LOC107832273	1.389247	0.51583	0.805095	0.046614	0.182118	0.165957	0.086313	0.043963	0.096614	0.100348	0.144317	0.173878	0.171837	0.1684	0	0.805209	0.646211	0.537052	0.104423	0.043526	0.093853	0.128911	0.272313	0.215117	0	0	0	0	0.247716	0
LOC107780721	0	0	0	0	0	0	0	0	0	0	0	0	0	0	0	0	0	0	0	0	0	0	0	0	0	0	0	0	0	0

LOC107784314	0	0	0	0	0	0	0	0	0	0	0	0	0	0	0	0	0	0	0	0	0	0	0	0	0	0	0	0	0	0
LOC107779085	1.615971	0.457063	0.581267	0.252412	0.049308	0.599092	0	0.047611	0.261577	0.163011	0.078146	0	0	0.060791	0	0.3322	0.215333	0.373896	0.056544	0.047138	0.101641	0.186143	0.049152	0	0	0	0.06586	0	0.268271	0
LOC107813464	1.685785	1.463224	1.395636	0.618167	0.71033	1.812425	0.302987	0.342948	0.376831	0.508812	0.084434	0.203457	0	0.218942	0.164785	0.568303	1.163293	1.196976	1.303328	1.493966	0.622307	0.268161	1.062124	0.167808	0	0.390941	0.142319	0	0	0
LOC107810544	0	0	0	0	0	0	0	0	0	0	0	0	0	0	0	0	0	0	0	0	0	0	0	0	0	0	0	0	0	0
LOC107807023	1.425721	2.626986	1.778197	3.341394	3.099079	2.63246	4.315328	3.681007	2.822552	2.871186	4.259662	1.859103	3.174266	3.279848	5.694228	1.986318	1.377367	1.548189	2.075734	2.097499	1.922156	3.390784	2.788548	2.170456	3.252418	3.881323	0.989073	0	1.492159	1.916402
LOC107766001	0	0	0	0	0	0	0	0.358038	0	0	0.097943	0	0	0	0	0	0	0	0.104137	0	0	0	0	0.123206	0	0	0	0	0	0
LOC107800002	63.74314	44.48285	56.06909	41.17858	39.83721	61.20146	24.05431	26.26102	29.0588	35.56386	29.3678	52.02339	23.13119	41.32296	25.21442	31.77517	57.19004	42.76283	25.91678	22.52103	40.66476	18.25667	24.81948	17.64583	19.01886	10.69146	34.15209	15.4789	20.31987	33.45785
LOC107787897	2.2859	1.011896	1.220637	12.01127	13.03206	11.34017	5.313612	8.063657	10.27064	16.65412	10.51028	22.2087	12.46901	14.9272	5.932496	2.190157	1.451216	3.238047	15.67369	17.81779	21.1754	6.272477	7.143515	10.34192	16.76242	8.905856	45.62097	7.006035	5.817	0
LOC107795958	0.391283	0.309059	0.430836	0.307218	0.184658	0.252406	0.284428	0.111441	0.269393	0.22893	0.256077	0.176302	0.058078	0.14229	0.294508	0.544291	0.478816	0.447301	0.185289	0.0662	0.166533	0.37034	0.368147	0.436234	0.09894	0.108888	0.092493	0	0.125585	0
LOC107829470	0	0	0	0.022123	0	0	0.030722	0	0.011463	0.023812	0.068492	0.02063	0.027184	0	0.050127	0.07279	0.023591	0.036412	0	0	0	0.040787	0.01077	0.012762	0	0.016989	0	0	0	0
LOC107822413	31.52325	37.58158	35.84563	29.06914	23.48148	22.05036	27.74917	25.85991	23.73333	17.16657	21.52644	14.03019	16.79788	14.61707	23.94329	36.2869	31.12435	36.59446	19.84376	22.11816	17.5583	34.00277	27.30838	27.01109	6.24961	12.12699	6.764854	3.100905	3.13131	8.043178
LOC107767744	0	0	0	0	0	0	0	0	0	0	0	0	0	0	0	0	0	0	0	0	0	0	0	0	0	0	0	0	0	0
107760433	0	0	0	0	0	0	0	0	0	0	0	0	0	0	0	0	0	0	0	0	0	0	0	0	0	0	0	0	0	0
LOC107781308	11.94211	25.14401	26.03821	31.67131	60.99662	26.18695	52.85864	62.67004	66.82666	52.30672	70.72144	85.97317	46.94625	43.64251	46.52018	69.54946	22.84571	55.24192	87.9857	88.98609	133.7522	54.67141	53.64208	71.26625	64.97819	96.35422	75.51087	188.8449	96.20213	104.7893
LOC107832174	0	0	0	0	0	0	0	0	0	0	0	0	0	0	0	0	0	0	0	0	0	0	0	0	0	0	0	0	0	0
LOC107816557	1.668145	1.418955	1.611204	0.638085	0.393623	1.215567	0.342013	0.459266	0.452437	0.054222	0.207947	0.093953	0.288867	0.262869	0.095119	1.187855	1.128098	1.423315	0.206888	0.376303	0.321179	0.185748	0.457773	0.232473	0.023434	0	0.043814	0	0	0
LOC107815777	2.030223	2.590418	2.099219	3.020291	2.108495	2.423988	2.957557	2.273341	2.397627	1.792171	3.154384	1.236742	1.92701	1.562069	2.961141	2.890483	2.209118	2.716545	2.244458	3.51621	1.335099	3.239283	2.290328	2.300683	0.716008	1.72468	0.618834	3.056625	0.823092	0
LOC107777773	0	0	0	0	0	0	0	0	0	0	0.038148	0	0.098524	0	0	0	0	0	0	0	0	0	0	0	0	0	0	0	0	0
LOC107818306	0	0	0	0	0	0	0	0	0	0	0	0	0	0	0	0	0	0	0	0	0	0	0	0	0	0	0	0	0	0
LOC107805258	16.53693	12.40759	12.3025	19.99872	19.12494	28.77753	14.57987	14.91823	13.21481	26.47598	25.18695	24.9526	25.68717	19.6353	14.76261	6.362282	6.464733	7.626766	8.585389	10.4105	8.838729	5.459934	7.80217	9.486318	7.098895	2.675573	11.27265	5.500578	7.406015	0
LOC107791152	0	0.037383	0	0	0.018148	0	0.034403	0	0	0.019999	0	0	0.022831	0	0	0	0.05944	0	0.041622	0.017349	0.018704	0.051383	0	0	0	0	0	0	0	0
Novel04519	1.816467	1.709764	1.863757	1.025145	0.76414	1.248591	0.19981	0.228989	0.251613	0.203262	0.187924	0.125787	0.066299	0.032486	0.061127	1.486778	1.524714	2.242288	0.815854	0.579376	0.461689	0.17408	0.866788	0.902599	0	0.041434	0.035195	0	0	0
LOC107780952	17.50551	15.87102	17.27782	15.36351	12.0209	16.56602	15.8297	14.84033	16.26374	7.112534	8.566838	8.742538	11.11389	12.08523	8.422113	12.09392	18.32111	13.42496	8.326584	7.404228	9.396255	13.4391	15.23996	16.10495	3.227625	2.727548	4.57984	9.780401	4.608939	5.637458
LOC107790317	1.721401	1.718258	1.710145	5.495387	4.75096	5.508084	2.096966	1.750966	1.92396	3.517031	3.994751	3.739595	2.053165	2.638092	2.692264	2.443409	3.246844	1.71117	2.869675	2.010924	1.644706	1.540271	0.759195	1.713531	0.310911	0.114057	0.53286	2.931059	0.78928	0
LOC107813563	0	0.085551	0	0.085042	0.083063	0.403689	0	0.160411	0.08813	0.091536	0	0	0	0	0	0	0.181374	0	0.095253	0	0.171223	0	0.1656	0	0	0	0	0	0	0
LOC107831569	4.740773	18.75786	4.724134	7.018496	2.353263	0.994519	5.237066	4.44583	0.651343	9.245705	38.3503	2.735215	2.18823	1.009161	27.29595	102.5382	4.021451	8.189574	5.279937	16.43273	10.54552	5.504175	5.303588	1.571115	12.71851	24.93776	4.236575	0	0	0
LOC107774653	2.332432	4.585491	3.997138	1.177279	0.943492	0.859766	3.52136	4.469775	5.318062	0.162458	0.607472	0.2815	0.890227	1.599443	1.094375	3.799058	4.023796	3.154911	0.845282	0.479176	0.972442	4.841861	3.938374	4.214011	0.463399	2.132697	0.039382	2.382884	0.160417	2.060254
LOC107775831	0	0	0	0	0	0	0	0	0	0	0	0	0	0	0	0	0	0	0	0	0	0	0	0	0	0	0	0	0	0
LOC107779760	90.26993	86.31929	90.8292	99.67005	96.38944	122.8997	78.73601	76.32763	86.09351	108.6286	110.0304	93.9049	90.2127	84.18065	79.19762	82.84953	106.5959	99.7815	84.58631	101.1898	65.96121	78.582	88.48568	97.75569	48.27919	52.13752	69.75665	45.89691	40.40501	36.63019
Novel02671	1.665787	2.024213	0.965356	5.868796	3.766885	7.163701	6.985838	5.693176	5.039281	5.955962	6.35939	5.472847	5.563134	3.634585	1.899687	1.379274	1.430481	1.793878	4.319722	4.227425	3.882467	6.801142	4.734523	4.062521	1.638048	2.317822	6.343997	0	3.564308	0
LOC107832757	1.234078	3.717792	2.443504	3.540372	5.09597	3.206397	6.267951	4.481327	3.540188	3.275873	5.937169	2.374774	3.663448	2.056876	6.544224	2.605637	3.411083	2.530159	1.704461	2.551866	3.032597	5.009914	5.200795	2.078105	0.260042	0.333886	0.567226	0	1.320287	0
LOC107773445	48.07458	54.67673	56.86379	52.97881	37.31911	44.61457	34.83893	29.08714	34.65553	25.65505	24.03858	26.43662	15.6203	18.04768	19.84335	39.41964	36.31573	36.37659	31.83419	35.98053	36.53735	24.83471	24.82413	31.74844	9.323767	9.09646	14.4642	19.95529	14.96928	12.32389
LOC107781988	3.261256	2.911575	2.545661	2.050084	0.70672	3.363137	0	0.056867	0.062486	0.713908	0.233346	0.506057	0	0.07261	0	0.89276	2.507658	0.893169	0.877977	2.083174	0.364202	0	0.176121	0	0	0.092608	0.235992	0	0	0
Novel01122	0.912085	0.534377	0.962754	0.590217	0.172944	1.050646	0.16393	0.22266	0.061165	0.063528	0.137047	0.330238	0	0.071074	0	0.485491	0.881151	0.874284	0.528869	0.220446	0.713003	0.054408	0.172397	0.272375	0	0	0	0	0	0
LOC107830339	0.469774	0.599397	0.530874	1.319332	2.286276	0.656583	1.41847	1.485133	1.234924	1.420075	1.976433	1.706575	3.242343	2.61375	1.446488	1.01522	0.408457	0.350236	2.049784	1.390887	1.75661	0.941569	0.745869	0.736511	2.791502	1.568774	0.721805	3.359546	5.88031	5.809362
LOC107824216	0	0.037857	0	0	0	0	0	0	0	0	0	0	0	0	0	0	0	0	0	0	0	0	0	0	0	0	0	0	0	0
LOC107815844	5.295222	4.065095	4.934773	6.720406	6.036353	5.539146	6.581972	5.135784	5.91192	5.41936	6.104728	4.937026	7.461328	6.297262	5.410389	4.781421	3.110856	5.014789	4.62291	4.459216	4.328995	5.856398	4.460342	6.581644	4.73459	4.314545	4.820757	0	9.875699	8.84896
LOC107816858	12.82213	15.91256	13.5175	15.8178	15.57039	12.46548	20.82249	24.70832	17.67278	16.09454	16.73835	12.10012	15.64041	15.32762	12.04026	20.53323	17.79021	17.69514	12.04211	17.65464	13.43566	22.66926	22.74025	18.81939	10.86487	12.1471	13.3813	19.50986	9.850579	16.86833
LOC107793338	1.799241	1.655111	1.315549	3.21434	3.943018	3.561472	2.510466	2.112019	2.099668	4.000886	3.973541	2.841212	3.725094	3.173633	3.192877	2.305668	1.543277	1.980774	2.986032	3.086741	1.564258	2.864756	2.135842	1.933292	3.42281	2.058963	2.742663	0	3.966795	2.079435
LOC107821851	0	0	0	0	0	0	0	0	0	0	0	0	0	0	0	0	0	0	0	0	0	0	0	0	0	0	0	0	0	0
LOC107815906	4.142635	1.279831	2.197284	0.572494	0.683431	0.754888	1.413399	1.319843	4.944011	0.479274	3.249458	1.482972	3.282862	16.31584	3.459163	0.313942	1.492321	0.209391	0.356242	0.890945	0.512292	2.580058	1.672205	3.008901	0	0.097698	0.248962	5.021305	0	0
LOC107771915	0	0	0	0	0	0	0	0	0	0	0	0	0	0	0	0	0	0	0	0	0	0	0	0	0	0	0	0	0	0
LOC107809556	0.528807	0.228289	0.290325	0.302572	0.369413	0.299228	0.793692	0.59451	0.783896	0.325676	0.526926	0.634857	0.650642	0.303633	0.28566	0.684434	0.752865	0.643247	0.225936	0.141264	0.279215	0.51135	0.785588	1.105419	0.211128	0.116178	0.230266	0	0.133993	0
LOC107796842	6.751169	5.092021	5.329002	4.991396	6.24854	6.924616	5.59741	4.177104	5.391194	5.977887	5.985421	6.162484	7.256341	6.687935	3.504431	2.081785	1.274452	2.429861	1.653598	1.181591	1.061583	1.29611	1.02672	1.541035	1.471643	0.539871	1.650898	5.549472	0.373592	0
LOC107791720	0	0.071786	0.616231	0.356793	0	0.931521	0	0	0	51.07688	24.90925	21.22759	10.08366	1.9764	0.242532	0	0.076095	0.058724	5.3551	17.7905	1.293056	0	0	0	17.92528	14.13813	127.0757	11.26585	4.171313	24.35127
LOC107759529	5.582341	4.096522	5.734904	4.170646	3.079245	4.910481	5.381432	4.367067	6.432073	1.413893	1.931753	1.653703	2.501877	2.57048	1.302173	4.105942	4.762659	3.72947	1.949496	1.809184	2.0497	4.540869	7.098225	7.198608	0.091659	0	0.471277	0	0	2.241328
LOC107806690	0	0	0	0	0	0	0	0	0	0	0	0	0	0.063073	0	0	0	0	0	0	0	0.048282	0	0	0	0	0	0	0	0
LOC107800158	0	0	0	0	0	0	0	0	0	0	0	0	0	0	0	0	0	0	0	0	0	0	0	0	0	0	0	0	0	0
LOC107794973	9.26051	10.19811	8.217673	17.08498	13.18772	17.2159	16.01983	14.67097	16.34686	12.46351	12.44583	13.97642	14.49693	15.312	12.07893	7.116598	10.57729	9.708896	9.886349	12.48505	11.78885	13.45341	13.69906	16.18222	9.085974	6.039996	9.520025	6.898515	6.966155	11.92899
107786784	0	0	0	0	0	0	0	0	0	0	0	0	0	0	0	0	0	0	0	0	0	0	0	0	0	0	0	0	0	0
LOC107810970	0.534487	0.378871	0.206497	0.053802	0	0	0.099622	0.101485	0.055756	0	0	0	0.066111	0	0	0.973624	1.549079	0.929794	0	0	0	0.099192	0.052384	0	0	0	0	0	0	0
LOC107784148	3.750924	3.681466	4.591848	0.965154	0.628462	1.503315	0.61432	0.758554	0.916849	0.692568	1.1361	0.731281	0.271783	0.556909	0.592284	2.795193	2.530163	2.978492	0.630609	0.657135	0.688229	0.77849	1.409565	0.858327	0.224488	0.12353	0.131162	0	0.106854	0
LOC107811468	1.368935	1.247615	0.75157	3.002554	3.187693	3.67943	3.142399	2.677894	4.092399	3.653367	4.622666	5.508659	9.263812	8.88204	6.803337	1.288587	1.49652	1.101172	1.937429	1.493242	2.694113	3.158933	2.097221	2.183868	4.008052	2.305777	3.95977	2.576268	6.243668	17.81964
LOC107800741	0	0	0	0	0	0	0	0	0	0	0	0	0	0	0	0	0	0	0	0	0	0	0	0	0	0	0	0	0	0
LOC107766433	1.075836	1.041773	1.198227	1.492437	1.487454	1.51811	1.663708	1.522464	2.114776	1.671965	1.485179	2.698285	2.806958	2.053947	1.897858	1.102357	1.071828	1.052732	1.159914	1.251364	0.98118	1.263458	1.334473	1.3353	1.147655	0.888812	1.072872	2.404296	1.780438	0
LOC107802342	14.9815	18.9636	18.32881	0.9551	1.276563	1.014141	1.419451	2.062319	1.302342	0.973924	2.431721	0.703155	0.617689	0.817204	1.110532	60.67966	40.23066	49.64153	3.659787	3.543835	3.896593	6.765446	10.44938	5.335543	1.192592	1.891551	1.442787	3.968108	1.335672	1.715423
LOC107766810	0	0	0	0	0	0	0	0	0	0	0	0	0	0	0	0	0	0	0	0	0	0	0	0	0	0	0	0	0	0
LOC107780897	0.044655	0.488365	0.077634	0.202274	0.039513	0.048009	0.41199	0.305232	0	0.087088	0.093935	0.037725	0.646231	0	0.549986	0.998298	0.60396	0.565961	0.317186	0.151099	0.203628	0.559382	0.157553	0	0.282284	0	0	0	1.289897	0
LOC107814293	0	0	0	0	0	0	0	0	0	0	0	0.075258	0	0	0	0	0.25818	0	0	0.075357	0	0.223182	0	0	0.112626	0.12395	0	0	0	0
LOC107799083	0.05307	0	0	0.096157	0.469595	0.057056	0.044512	0	0.049824	0	0	0.044835	0.059078	0.057896	0.163408	0	0.05127	0	0.215406	0.089787	0	0.221599	0	0.166405	0	0.147685	0	0	0.255496	0
107792564	0	0	0	0	0	0	0	0	0	0	0	0	0	0	0	0	0	0	0	0	0	0	0	0	0	0	0	0	0	0
LOC107763138	1.697104	1.680367	2.076287	1.784248	1.538789	1.148819	1.546447	1.503758	1.750681	0.919384	1.219394	0.47792	0.862986	0.64001	1.032214	2.014127	1.497856	2.04629	0.531514	0.974813	0.726123	1.872231	1.958982	1.751914	0.291385	0.204071	0.222871	0	0.30261	0
LOC107811752	0	0	0	0	0	0	0	0	0	0	0	0	0	0	0	0	0	0	0	0	0	0	0	0	0	0	0	0	0	0
LOC107761472	0	0	0	0	0	0	0	0	0	0	0	0	0	0	0	0	0	0	0	0	0	0	0	0	0	0	0	0	0	0
LOC107762306	9.473855	11.87537	10.92633	5.413185	4.713896	6.300182	5.422207	4.441023	5.663805	2.779885	3.897	2.773345	4.327561	4.14677	4.24119	10.82594	8.735251	11.77551	3.462582	3.836599	2.179775	6.132295	5.841976	6.124837	1.12861	0.841416	1.259275	0	0.901119	1.780495
LOC107759985	2.957818	4.037202	3.271299	1.597716	1.290204	2.732126	0.489182	0.569519	0.677936	0.866628	1.139252	0.586584	1.406734	1.136209	0.456086	3.818491	2.240388	3.571768	0.69046	0.975001	0.291288	0.429087	0.734927	0.653136	0.140454	0.135254	0.262603	0	0.467981	0
LOC107809034	7.519486	5.707005	6.268822	0.581252	0.295217	0.855343	1.033215	1.184103	2.168485	0.300305	0.359909	0.433629	0.571385	0.81194	0.131703	7.802867	8.504124	10.44685	0.338544	0.325647	0.351087	1.350247	2.082617	1.63625	0.032447	0	0.060665	0	0	0
LOC107801709	1.81514	0.945301	0.676227	0.293647	0.516264	0.418178	0.652473	0.886231	0.365171	0.505712	0.590932	1.697782	0.938154	0.990114	0.399216	0.531396	1.377811	0.676632	0.723595	0.767742	0.41386	0.487244	0.285906	0.813079	1.065484	1.713824	0.996055	0	1.872585	0
LOC107812588	1.587266	0.927306	0.778335	0.331842	0.360133	0.13127	0.136544	0.069549	0.382101	0	0.028538	0.137535	0.362455	0.222004	0.125317	0.727897	0.6291	1.001316	0.371688	0.344287	0.890839	0.135956	0.107698	0.127616	0.977665	2.491713	0.048103	2.910562	1.76346	0
LOC107762029	0.601863	0.613056	0.646289	0.416961	0.281948	0.418696	0.326641	0.438623	0.365624	0.155354	0.248251	0.08973	0.137942	0.231742	0.218024	0.712338	0.478841	0.739059	0.269439	0.269542	0.113011	0.428715	0.59334	0.296031	0.134283	0.172416	0.146455	0	0.085223	1.094532
LOC107814274	0	0	0	0	0	0	0	0	0	0	0	0	0	0	0	0	0	0	0	0	0	0	0	0	0	0	0	0	0	0
LOC107808523	2.037271	2.409135	1.697777	1.098247	1.087586	0.959395	0.988531	0.704912	1.059087	2.561232	2.29038	1.280189	3.11136	2.351141	0.864049	3.023807	2.000707	2.585849	0.768824	0.997003	0.399245	0.885842	0.816822	0.809507	3.789091	0.421693	3.203888	1.20408	12.23992	2.082108
LOC107777595	0.542444	0.463473	0.471535	0.307142	0.059999	0.109349	0.113743	0.173805	0.190976	0	0.071318	0	0	0.110959	0.139187	0.555816	0.622305	0.404415	0.034402	0.114718	0.12368	0.141565	0.358854	0.07087	0	0.094346	0.04007	0	0	0
LOC107812725	0.015056	0.054887	0.013088	0	0.026645	0.016187	0.164165	0.09005	0.014135	0.014681	0.105573	0.025439	0.033521	0.082127	0.401779	0.033659	0	0.011225	0	0.012736	0.013731	0.012574	0.026561	0.031473	0.019035	0	0	0	0	0.930934
LOC107806008	5.513659	5.570371	5.275913	7.357118	6.788785	6.800953	6.883041	6.591776	6.360232	6.418474	8.077022	5.380277	6.256833	6.248276	9.322109	7.373518	4.542112	5.847344	8.045351	8.496755	4.63874	8.459615	6.880408	6.634007	4.45816	6.155325	3.990848	0	5.555871	5.285548
LOC107781893	3.388603	5.234396	4.068978	2.055274	1.931211	2.037701	4.190992	3.974923	1.914214	2.352225	4.570954	1.116001	1.982024	2.913579	6.160132	6.655376	4.300167	4.924236	1.10732	1.238922	0.70714	4.820441	4.204825	1.950973	0.798754	0.799151	0.746705	0	0.691269	1.775614
LOC107788291	29.08359	21.5988	27.22405	5.309472	5.056261	7.009795	6.113776	5.60216	6.9466	3.464656	4.905724	4.053843	5.219346	4.715358	5.000163	16.70523	20.5244	20.45416	4.237242	4.492939	5.244813	8.351071	8.88508	7.963245	2.408143	3.618647	2.943908	0	2.468844	4.529678
Novel02690	0.027951	0.127368	0.048594	0.101288	0.049465	0.180303	0.046887	0.023882	0	0	0.039198	0	0	0.030493	0	0.499895	0.297031	0.541801	0.34035	0.4256	0.382374	0.303452	0.098618	0.35057	0.176691	0.077783	0.198213	0	0.941955	0
LOC107791605	40.36355	56.89696	36.65399	5.047182	3.286482	3.699502	15.53008	15.44713	16.35796	3.888021	4.940568	3.876077	3.404957	4.826531	4.036291	29.46843	30.28808	24.10677	2.494068	2.63363	2.440865	16.28415	14.54967	7.478517	0.897718	0.303994	1.226553	7.812083	1.051824	3.377184
LOC107799713	0.491829	0.709715	0.53442	1.336716	1.088006	0.749099	8.903512	6.723709	5.194693	0.839291	1.695614	0.727144	3.650071	2.593378	2.397798	0.549767	0.712722	0.580575	0.914969	0.693422	1.495187	6.674509	2.783713	2.441782	1.761826	3.136576	0.339093	2.931059	2.170519	22.80788
NitaMp012	0	0	0.120937	0.063019	0.061553	0.149574	0.058344	0	0.195922	0.135662	0.195106	0	0.077437	0.303552	0	0	0.336011	0.155583	0	0.117688	0.190324	0.116185	0	0.145411	0.087947	0.483948	0	0	0	0
LOC107821863	0.369108	0.152907	0.116676	0.243195	0.207843	0.18038	0.900616	1.146821	1.575158	0.098162	0.376464	0.510271	3.137751	2.013393	0.861005	0.250054	0.194503	0.350236	0.102149	0.113542	0.030603	1.989624	0.443969	0.806655	0	0	0.158638	4.799351	0.161547	0
LOC107759878	0.614703	0.829565	0.637107	0.642565	0.460248	0.800682	0.634557	0.434315	0.6437	0.311234	0.663123	0.309593	0.394786	0.232134	0.412521	0.836872	0.559594	0.669804	0.467818	0.459997	0.301872	0.750287	0.802886	0.766042	0.23913	0.279622	0.237519	0	0.227646	0
LOC107771602	2.412226	2.51251	2.456365	2.372671	2.012525	2.000652	2.427885	2.296622	2.394116	1.814573	1.933091	1.863237	3.222388	2.481242	1.839197	2.87615	2.530163	2.1581	1.39872	1.749066	1.131424	1.554053	1.641402	2.737366	1.045641	0.287695	0.733129	0	1.990854	4.261473
107797837	0.717531	0	0	0	0	0	0	0	0	0	0	0	0	0	0	0	0	0	0	0	0	0	0	0	0	0	0	0	0	0
LOC107787360	0.717084	0.653534	0.566678	0.885876	0.663366	1.016257	0.710804	0.640546	0.734433	0.762816	0.685664	0.605813	1.596537	1.493489	0.602178	0.485793	0.535318	0.63182	0.429974	0.827187	0.624266	0.435532	0.345009	0.647292	0.618145	0.272119	0.423768	0	0.313845	0
LOC107800220	4.404128	5.137689	4.594098	7.235049	7.222555	4.166779	8.766931	8.629828	5.678444	4.809931	6.835166	3.770339	3.464601	3.075	8.859741	6.520163	5.61628	4.815748	5.779913	7.053782	4.391525	11.18113	6.267368	4.419066	3.415136	4.167055	1.526895	0	2.544365	7.261703
LOC107811593	1.435999	2.181226	2.674893	1.548741	1.331174	0.882214	1.491204	1.928074	1.28398	1.26692	2.63718	1.039861	1.522451	2.238006	2.316081	2.853621	1.585481	1.78433	1.248988	2.429512	1.870936	1.998733	1.688861	1.786792	1.469722	1.141764	0.404104	0	1.97526	12.68428
LOC107772387	0.363244	0.264842	0.568368	0.460713	0.192853	0.156213	0.426536	0.186219	0.204618	0.42505	0.509414	0.306878	0.646989	0.475537	0.447388	0.595517	0.350924	0.758278	0.147438	0	0	0.060671	0.192243	0.075932	0	0	0.085865	0	0	0
LOC107781575	13.82514	17.06608	13.06171	22.22725	18.867	20.62079	24.08974	19.35488	21.81557	13.89266	15.72463	12.25563	24.42223	25.2792	19.81901	14.41384	16.02123	15.07372	9.451327	12.71089	10.18172	16.93917	20.66799	17.50286	4.021032	2.98033	7.6331	4.641752	7.343381	10.03321
LOC107830072	43.57322	36.78036	43.469	6.451999	6.234084	8.233136	4.624543	6.608514	8.555548	3.883038	3.436605	8.410445	9.121575	8.270805	2.279347	31.72895	30.85023	30.20198	12.04453	6.607583	10.61583	5.819704	11.5506	8.003993	3.098196	3.516267	8.145877	5.476453	4.055443	33.14479
LOC107768211	0.429342	0.293469	0.093305	0	0	0.461595	0	0.09171	0	0	0.150528	0.18136	0	0.234196	0	0	0	0	0	0.090799	0	0	0	0	0	0	0.126862	0	0	0
LOC107816864	0	0	0	0	0	0	0	0	0	0	0	0	0	0	0	0	0	0	0	0	0	0	0	0	0	0	0	0	0	0
LOC107829631	0	0	0	0	0	0.086372	0	0	0	0	0	0	0	0	0	0.059867	0	0	0	0	0	0	0	0.083968	0	0	0.094952	0	0	0
LOC107774146	1.173254	0.959351	1.153328	1.003306	0.98966	0.660168	1.103617	0.899403	1.317685	1.753529	1.314761	1.232049	1.879783	1.42351	1.057892	1.201155	1.016938	1.128132	0.923501	1.1038	0.580015	1.291158	1.015544	1.203364	2.453765	1.190045	1.931001	1.568304	4.856622	4.067891
LOC107803254	9.691656	10.4155	8.960429	8.069617	7.336536	8.070763	5.82643	6.318309	7.31072	5.89979	4.988821	5.726714	4.178355	6.356096	4.254893	8.850367	9.795924	11.44392	8.015339	7.582404	8.379124	4.818825	4.595534	7.611926	3.187249	3.351821	3.310624	0	4.854702	0
LOC107816524	1.64097	1.316077	1.654694	0.356793	0.290408	1.129116	0.055054	0.112167	0.554621	0.448043	0.690389	0.720895	0.21921	0.57287	0.269479	0.782626	1.838965	1.468095	0.199817	0.499733	0.47891	0.054817	0.289489	0.205817	0.082987	0	0.387899	0	0.316009	0
107769590	0	0	0	0	0	0	0	0	0	0	0	0	0	0	0	0	0	0	0	0	0	0	0	0	0	0	0	0	0	0
LOC107825010	7.114574	7.298479	9.172253	4.359237	3.467056	3.879946	3.171029	2.936659	3.968964	1.541691	2.24132	1.887383	2.410448	1.949787	1.481607	6.044552	5.943558	7.636022	2.301831	2.703937	1.786719	3.47309	3.910834	4.957434	0.608356	0.239116	1.096807	0	0.661876	0
LOC107804236	0	0	0	0	0	0	0	0	0	0	0	0	0	0	0	0	0	0	0	0	0	0	0	0	0	0	0	0	0	0
LOC107806632	0	0	0.043866	0	0	0	0	0	0	0	0	0	0	0	0	0	0	0	0	0	0	0	0	0	0	0	0	0	0	0
LOC107806216	16.03314	15.9346	13.05436	15.05101	14.95752	13.18168	14.54297	16.30261	13.1455	17.82739	15.87169	13.54524	21.07878	20.73637	15.6369	18.32734	17.59199	16.11814	17.07911	15.0972	15.15181	15.87376	16.19004	13.0423	21.83005	15.24276	16.20601	10.37644	35.27653	4.485761
LOC107811315	6.61921	7.845859	3.392496	6.86324	6.06025	5.183087	34.36992	26.80705	14.44041	3.619034	9.524248	6.270902	6.303771	7.429939	9.346351	3.792148	6.135968	2.995172	4.193095	4.952067	5.862356	22.30322	18.19072	10.23763	0.24187	0.851806	2.577643	2.736226	0.736815	0
Novel02672	2.839011	4.010484	3.640141	3.215001	2.637751	1.526142	3.750392	2.668301	3.66491	0.415259	0.94559	0.539657	0.079011	0.542014	0.874165	3.226343	3.291269	3.439481	1.656479	0.840563	1.682997	4.860412	4.695367	4.302634	0.448672	0	0.335548	0	0.683401	0
LOC107821730	15.72357	14.06636	19.11867	4.893906	4.865371	8.400528	6.634474	6.42885	7.879096	1.693156	2.367413	4.971205	2.040309	3.893773	1.881145	8.19487	17.33371	12.51369	3.719617	3.264064	3.870965	6.364186	9.18943	8.469197	0.609797	0.268444	2.052221	0	0	0
LOC107831675	3.213192	3.186819	2.423477	2.333072	2.675994	2.540129	2.635593	2.906935	2.550875	2.234758	2.236526	2.155707	1.656979	1.984695	1.988459	2.975487	3.195514	3.258682	2.613215	3.157839	2.391793	3.4332	2.750888	3.08679	1.822129	2.071089	1.005283	0	1.70619	5.843427
LOC107815698	0	0	0	0	0	0	0	0	0	0	0	0	0	0	0	0	0	0	0	0	0	0	0	0	0	0	0	0	0	0
LOC107782121	3.081162	1.68486	3.080148	2.000487	1.72673	1.932365	0.99065	0.92142	1.54279	0.050075	0.792188	0.347074	0	0.056023	0.158121	1.989941	2.827829	2.603422	1.511166	0.564731	1.03036	1.50101	3.080161	4.884319	0	0	0	0.789032	0	0
107816637	0	0	0	0	0	0	0	0	0	0	0	0	0	0	0	0	0	0	0	0	0	0	0	0	0	0	0	0	0	0
LOC107766382	0.424988	0.580986	0.785045	0.33689	0.376057	0.285571	0.311899	0.317731	0.299248	0.207208	0.149001	0.269281	0	0.11591	0.327147	0.672991	0.461896	0.554481	0.215624	0.404449	0.145349	0.266189	0.281151	0.666296	0.268657	0.073917	0.125575	0	0.255755	0
Novel03473	0.524991	0.500213	0.622314	0.713423	0.886861	0.846644	0.760574	0.285451	0.672115	0.721359	0.719515	1.108817	0.557862	0.807042	0.514344	0	0	0	0	0	0	0	0	0	0	0	0	0	0.255755	0
LOC107781814	4.266997	5.530797	5.143438	2.285048	1.812346	3.160308	1.192971	1.150465	1.281934	1.349968	1.43617	0.38452	1.414469	1.634452	1.36252	4.691929	3.975708	5.344462	2.732619	2.197837	1.210712	1.678795	2.492455	1.149654	0.52749	0.765241	0.694847	1.356225	0.913015	0
LOC107817477	0.293106	0.675682	0.839311	0.562318	0.564488	0.333662	1.243664	1.620479	1.34353	1.00876	2.889455	1.354649	0.537418	0.489051	4.247096	5.935964	0.283165	1.915289	1.399641	2.202357	1.226516	2.850972	1.216653	1.08125	0.719352	2.039178	1.100417	0	1.162096	1.06607
LOC107784241	1.67807	2.387665	1.354517	3.412798	3.666712	2.650967	2.326634	3.496689	2.491777	3.973945	4.214378	2.806428	5.222918	5.043743	5.430597	2.105433	2.067802	2.565975	6.463651	6.605169	3.87292	3.718001	3.564488	2.953026	10.30487	10.53093	5.686935	4.898194	9.562693	23.29252
LOC107761809	0	0	0	0	0	0	0	0	0	0	0	0	0	0	0	0	0	0	0.065863	0	0	0	0	0	0	0.090313	0	0	0	0
LOC107794059	0	0	0	0	0.104862	0	0	0.101255	0	0	0.100117	0.131923	0.193927	0.121632	0	0	0	0	0	0	0.05404	0	0	0	0.299656	0.082446	0.140065	0	0	0
LOC107784463	0.054905	0	0	0.049742	0.048584	0	0.046052	0.046913	0.103095	0	0	0.153999	0.046386	0	0.059899	0.16906	0	0	0	0	0	0	0.045853	0	0	0	0	0	0	0
Novel01163	4.55727	4.468837	3.359785	3.031165	3.368984	5.023664	2.999841	3.450251	3.032907	4.106401	3.438261	4.093786	3.917294	2.643213	1.539416	3.224129	3.733941	3.569671	2.34147	2.391161	4.051084	2.746026	4.325116	4.763257	1.677479	2.648818	2.590891	0	1.110902	0
LOC107766069	0	0	0	0	0	0	0	0	0	0	0	0	0	0	0	0	0	0	0	0	0	0	0	0	0	0	0	0	0	0
LOC107772406	3.37108	3.927908	2.856221	2.551466	2.378811	2.546202	3.06011	2.643352	3.225012	3.536892	6.163842	4.253948	3.657728	3.375089	2.781127	4.944768	4.761447	4.931126	1.818621	3.393164	1.770746	3.421096	3.613386	3.746456	1.456657	2.167182	2.244437	0	1.335345	0
LOC107766521	0	0	0	0	0	0	0	0	0	0	0	0	0	0	0	0	0	0	0	0	0	0	0	0	0	0	0	0	0	0
LOC107762151	13.98392	16.45876	13.89248	14.98529	14.42442	11.46912	14.47683	13.48444	13.12872	14.88281	15.04449	15.62824	18.1023	14.763	14.39372	15.60147	15.32379	14.50648	14.18991	14.6685	14.46619	12.57924	13.60346	12.61151	16.61913	13.34236	19.31399	8.571839	12.88765	4.940838
LOC107825478	0	0	0.019883	0.082888	0.263117	0.049183	0.613913	0.508132	0.150321	0	0.224542	0.09662	0.050926	0.049907	0.140859	0	0.508242	0.204636	0.487414	0.193492	0.417216	1.107919	0.786853	0.3347	0.578375	2.036894	0.486618	6.543046	0.22024	1.414288
LOC107811968	19.91653	18.93781	19.9381	12.53916	10.33867	17.39299	11.03606	9.890879	13.8045	11.11282	11.9236	9.572418	10.52539	10.43256	7.74869	18.43172	16.98352	19.94111	9.922561	11.10023	7.016501	11.10856	15.03085	16.08226	6.681482	2.801254	11.51493	2.570963	3.807719	13.33721
Novel01046	0.115884	0.211229	0	0.524927	0.820337	0.87213	0.583184	0.594089	0.652784	1.017018	0.731324	2.05595	0.387012	0.379273	0	0	0	0.823141	0.196061	0.73982	0.193556	0.511089	0	0.732566	1.934937	0	0	0	0.557909	0
LOC107808713	3.294097	2.078421	3.335492	12.62582	25.78507	22.45581	6.436648	7.175596	7.612661	32.12161	16.75256	33.88464	24.50063	29.49992	11.0346	2.643593	2.657807	3.292511	39.30336	29.58061	28.62312	7.527424	5.811223	12.14505	69.56489	35.6606	58.61428	41.41969	77.02921	22.38231
LOC107761206	0.66284	0.56195	0.428794	0.251372	0.354643	0.497186	0.840393	0.408298	0.694664	0.631321	0.853905	0.251	0.497642	0.655858	0.490464	1.068308	1.102013	1.206707	0.281555	0.234719	0.548287	1.351704	0.84301	1.337265	0.350804	0.193038	0.127534	0	0.593702	0
LOC107767804	60.65174	62.9962	59.42142	46.70914	35.14407	46.6903	56.26486	55.28378	64.49586	32.1544	33.39155	38.24473	25.48101	35.20104	35.82138	64.47171	69.04922	60.00262	40.21566	46.27473	51.9567	71.56556	83.18433	69.01892	4.727723	17.9743	17.20987	6.077707	6.000915	12.26124
LOC107762392	4.640424	4.654455	4.38312	4.110045	3.45238	4.208618	2.337449	2.425458	3.918537	1.403003	1.826893	1.314087	1.616112	1.258548	1.197353	8.867289	5.096641	9.528487	2.69637	2.576781	2.801727	2.305719	3.692992	4.674047	0.180268	0.360716	0.490244	0	0.31202	0
LOC107788450	13.02126	9.425448	10.69027	14.85498	12.99196	17.36968	13.14624	11.03415	16.17414	11.96584	13.24888	17.22541	20.16239	22.71135	12.34719	10.86148	14.00325	13.10367	8.536843	7.910013	9.334326	9.129122	10.28033	12.04161	5.114995	3.35521	8.043376	3.832116	12.38301	9.939804
LOC107787022	0	0	0	0	0	0	0	0	0	0	0	0	0	0	0	0	0	0	0	0	0	0	0	0	0	0	0	0	0	0
LOC107807083	4.270452	5.199456	4.611265	2.387783	1.445363	2.25976	1.213288	0.940704	1.002322	0.585595	0.818793	0.197301	0.29712	0.80074	0.513642	5.817726	4.70578	5.720896	0.744796	1.213577	1.064964	0.780143	1.88341	0.871772	0.168723	0.092844	0.197161	0	0.321242	0
LOC107795369	9.02645	10.95865	20.76173	3.40232	3.498042	3.258458	6.134064	6.079882	11.81471	11.66095	9.055131	10.85423	7.00452	4.600251	6.255228	29.28737	6.90623	18.69061	4.31228	5.796486	6.039004	6.052588	8.97896	21.27914	1.999213	4.033755	6.346611	2.355908	9.040229	2.036931
LOC107807897	0	0	0	0	0	0	0	0	0	0	0	0	0	0	0	0	0	0	0	0	0	0	0	0	0	0	0	0	0	0
LOC107790931	1.498426	2.078133	1.359183	2.774018	2.70946	1.540948	1.475367	1.669951	1.284459	1.65174	2.512537	1.816308	2.828464	2.061155	2.340349	1.116629	1.762302	1.602853	1.057739	1.267565	0.831837	1.36019	1.206779	1.089499	0.658945	0.99715	1.232013	4.659072	4.077454	4.028258
LOC107817940	60.76756	68.26544	58.02477	86.67023	75.41454	77.88196	77.68249	93.41852	89.59692	91.40074	90.73187	100.9419	58.63636	64.3747	72.06282	46.98877	44.36993	45.87329	102.1363	101.8676	107.1509	68.62815	90.38703	88.15973	70.15437	109.2831	112.0318	96.47878	51.1127	36.26786
LOC107800805	3.054092	1.982716	2.218442	1.62979	1.517821	1.84417	1.473796	1.644342	1.963915	1.387072	1.114769	1.661213	1.071188	1.825679	0.815864	1.839436	2.101733	1.996234	1.188689	0.743213	1.182834	0.873477	1.623727	1.355564	1.904203	0.756768	1.285643	0	1.409923	2.586837
LOC107813786	0.071773	0.039247	0.037434	0	0	0.01204	0	0	0	0.027995	0.020131	0	0	0.01566	0.014733	0.021394	0.013868	0	0.029132	0	0.011988	0.012662	0	0.018149	0	0	0	0	0.069108	0
LOC107795658	0.723228	0.939264	0.660121	0.262083	0.383976	0.272145	0.288136	0.208832	0.322524	4.830882	2.954019	1.726083	1.65047	1.558286	0.482496	0.660214	0.803504	0.552677	1.027434	1.147127	0.676086	0.271794	0.478451	0.226775	3.108891	1.761052	4.252614	1.293025	3.394828	1.117956
LOC107773148	0.191867	0.961748	0.250179	0	0	0	0	0.081968	0	0	0	0	0	0	0	0.500429	0.37072	0	0.097347	0	0	0.160233	0.16924	0	0.133485	0	0	0	0	0
LOC107764679	0	0.151789	0.289556	0.678984	0.368434	0.358122	1.117537	1.138434	0.547273	0.162407	0	0.492469	0.927025	0.363394	1.453004	1.054958	0.160901	0.993357	3.71806	2.183784	1.291114	1.390897	0.807991	0.609271	1.473983	4.171342	0.49212	11.91063	0.801828	5.148998

LOC107800775	3.60618	3.286588	3.330699	3.961257	4.028619	4.797892	3.024658	3.004187	3.851166	2.285712	3.31886	3.770169	3.562824	4.229227	2.313303	3.560711	4.006454	3.528735	3.064666	3.851357	3.576675	2.258711	2.743517	2.732654	3.476472	3.010642	3.729438	3.223661	2.387197	0
LOC107819668	0.167466	0.220458	0.16175	0.134859	0.13172	0.140036	0.218495	0.143088	0.209633	0.163301	0.182665	0.2358	0.269282	0.162398	0.038196	0.138662	0.14381	0.180344	0.13217	0.125924	0.084851	0.202014	0.213368	0.175036	0.094102	0.103563	0.197932	0	0.089583	0
LOC107825660	0.212064	0.257693	0.24579	0.320198	0.125099	0.151996	0.177867	0.060398	0.199095	0.344648	0.148699	0.059719	0	0.385585	0.072552	0.105353	0.136581	0.158103	0	0.059797	0	0.295167	0	0.073883	0	0	0.167095	0	0	0
LOC107824291	1.555024	1.566393	1.920907	1.631211	0.651784	2.375774	1.304266	0.769221	1.844116	0.957692	0.746052	1.037153	1.002201	1.339307	1.008022	1.707714	2.134825	1.281372	0.913539	1.176976	1.343566	2.392234	1.804782	1.28314	0.20695	0	0.096732	0	0.394023	0
LOC107766099	0	0	0	0	0	0	0	0	0	0	0	0	0	0	0	0	0	0	0	0	0	0	0	0	0	0	0	0	0	0
LOC107827359	164.3969	135.2219	148.6733	130.6382	120.1588	145.3589	114.3915	118.635	143.5212	99.81608	112.1205	116.5253	85.3993	89.28038	85.27631	131.5636	154.6968	145.8977	89.30762	93.36979	97.90855	121.9887	139.5921	172.8917	44.55065	32.21457	75.06668	35.22784	32.88549	34.51927
LOC107828895	0	0	0	0	0	0	0	0	0	0	0.020236	0	0	0	0	0	0	0.021516	0	0.024413	0.02632	0	0	0	0	0	0	0	0	0
LOC107777402	0.367255	0.376546	0.119718	1.871519	1.259265	0.345489	1.69418	1.490516	0.258596	1.701071	4.249072	0.349051	0.61325	0.250411	3.10976	0.615778	0.1774	0.034226	0.652164	1.553363	0.753621	1.265155	1.093309	0.431837	2.031409	4.471326	0.542583	3.282992	0	0
LOC107765446	0	0	0	0	0.058819	0.071465	0.167258	0.511158	0.124813	0.194455	4.567785	0.954672	0.739973	0.652657	10.02905	0	0.128435	0	0	0.281153	0	3.441764	0.23453	0.347381	0.084041	0.092491	0	0	0	0
LOC107790857	1.042383	1.187505	0.792856	1.298476	0.922369	2.171336	0.928935	0.612315	1.223294	0.444699	0.319777	0.495357	0.652723	0.710743	0.334336	0.825334	1.699363	1.554282	1.388282	1.653346	0.475336	0.707299	1.436642	0.476656	0.41184	0.5439	0.693007	4.659072	0.627301	0
LOC107796137	0	0	0	0	0.126984	0	0	0	0	0	0.201253	0	0	0	0	0	0.138639	0	0	0	0	0	0.253164	0	0	0	0	0	0	0
LOC107794561	1.810824	1.13079	1.544961	0.12152	0.148364	0.25237	0.112505	0.143261	0.409279	0.0327	0	0.02833	0.03733	0	0	0.449809	0.874704	0.525018	0.30625	0.028367	0.122333	0.084015	0.354948	0.245346	0	0	0	0	0	0
LOC107771906	0.022447	0	0.009756	0.010168	0.009931	0	0.018827	0.028769	0	0	0	0.028446	0.049976	0.061221	0.011519	0.008364	0.010843	0	0.022778	0	0	0.028119	0.0099	0	0	0.04685	0	0	0	0
Novel00206	0	0	0	0	0	0	0	0	0	0.325953	0.164072	0.112959	0	0.036467	0	0	0	0	0.135677	0.056554	0.030486	0	0	0	0.972017	3.116232	1.303756	0	1.126499	0
LOC107770029	0.694479	0.34081	0.51082	0.50817	0.732698	1.694321	0.448069	0.228224	0.727239	0.807437	0.692995	0.902639	0.535226	0.670222	0.548303	0.457812	0.722535	0.557592	0.731814	0.74565	0.36541	0.356909	0.801062	0.697951	0.168852	0.111498	0.473549	0	0.385785	0
LOC107765235	18.53288	18.29056	19.79863	13.43188	13.96082	12.47941	13.60128	12.14711	15.45349	6.158712	8.139139	5.541785	7.248016	6.916841	7.158142	17.08168	16.7264	18.50757	9.106006	8.498876	6.49404	15.04963	18.08949	21.07822	2.466441	3.393045	3.37212	3.487797	3.521995	9.046703
LOC107831989	0	0	0	0	0	0	0	0	0	0	0	0	0	0	0	0	0	0	0	0	0	0	0	0	0	0	0	0	0	0
LOC107826714	0.151825	0.41511	0	0.412638	0	0	0	0	0	0	0.10646	0	0	0.165634	0	0.452562	0	0.113192	0.154062	0.256868	0	0	0	0	0	0	0	0	0	0
LOC107815050	6.204689	6.285926	5.321369	8.431701	5.563848	5.956071	5.273838	5.419791	7.30755	3.754461	5.962833	1.61469	2.312661	2.629035	3.354741	4.768233	5.43229	5.245394	1.995636	1.593367	3.107277	3.654948	6.108198	6.485139	1.540906	0.963546	2.193485	0	0	0
LOC107803341	4.280714	4.623245	4.35109	5.496534	5.27914	4.892199	5.611797	4.708746	4.857517	4.124935	5.081515	4.000894	6.097397	6.343176	5.690933	4.872903	3.500547	4.196636	3.471598	3.720986	2.044264	4.517931	3.998858	4.280437	2.855215	1.47735	1.892318	1.205242	3.732317	2.084118
LOC107780203	4.157124	2.210078	3.011416	2.803685	1.655324	4.047305	2.905632	3.157297	6.873425	1.396282	2.817812	2.595018	4.293541	3.779348	1.422253	2.357841	2.945163	2.376139	0.632754	0.937771	0.8004	3.818895	3.40203	4.972639	0.175196	0.06427	0.491339	3.303257	1.22307	1.428007
LOC107787755	0	0	0	0	0	0	0	0	0	0	0	0	0	0	0	0	0	0	0	0	0	0	0	0	0	0	0	0	0	0
107826791	0	0	0	0	0	0	0	0	0	0	0	0	0	0	0	0	0	0	0	0	0	0	0	0	0	0	0	0	0	0
LOC107821475	0.038122	0.034744	0	0.034537	0.303596	0.040986	1.37491	1.53091	0.322116	0.223043	0.534625	0.032207	0.806323	0.665429	1.252079	0.056817	0.110487	0.056843	0.348153	0.322487	0.69536	1.241634	0.336262	0.159381	0.289188	0.795662	0	0	0.183533	7.07144
LOC107783039	0	0	0	0	0	0	0.042489	0.043283	0.047559	0	0.142084	0.042797	0.112785	0.110529	1.403821	0	0.048939	0.037767	0	0.042853	0	0.465359	0.044683	0	0	0	0.059873	0	0	0
LOC107807415	0.062431	0	0	0	0	0	0	0	0	0	0	0	0	0	0	0	0	0.046545	0	0	0	0	0	0	0	0	0	0	0	0
LOC107759617	6.347852	6.986004	5.101596	10.3081	9.820913	13.3919	7.735183	7.470486	8.545904	8.564721	7.894435	8.499599	9.199806	9.320702	5.818661	6.039476	7.366785	6.964949	9.763322	9.625196	9.466858	8.268628	8.592522	8.554267	4.997081	4.99957	6.322989	2.855102	5.189573	7.405609
LOC107809497	25.64931	22.69664	22.32881	17.8668	14.41604	24.12912	17.26057	13.66531	19.18056	11.88764	16.54753	13.25982	14.40109	16.02465	14.46385	16.69687	22.29425	22.48579	8.473954	9.177306	9.622247	15.31808	18.80981	20.76891	2.780934	2.07495	5.640078	5.332233	3.769161	2.305138
107767935	0	0	0	0	0	0	0	0	0	0	0	0	0	0	0	0	0	0	0	0	0	0	0	0	0	0	0	0	0	0
LOC107769029	0	0	0	0	0	0	0	0	0.033915	0	0	0	0	0	0	0	0	0	0	0	0	0	0	0	0	0	0	0	0	0
107804750	0	0	0	0	0	0	0	0	0	0	0	0	0	0	0	0	0	0	0	0	0	0	0	0	0	0	0	0	0	0
LOC107809293	0	0	0	0	0	0	0	0	0	0	0	0	0	0	0	0.075646	0	0	0	0	0	0	0	0	0	0	0	0	0	0
LOC107762677	0.275087	0.23596	0.154729	0.293194	0.243415	0.139177	0.230727	0.235042	0.364607	0.426035	0.272317	0.287084	0.30623	0.353066	0.166083	0.627044	0.500248	0.627331	0.295559	0.27377	0.088547	0.472978	0.371104	0.507389	0.122751	0.090062	0.191253	0	0.077904	0
LOC107820397	0	0	0	0	0	0	0	0	0	0	0	0	0	0	0	0.032206	0.041752	0	0	0	0	0	0	0	0	0	0	0	0	0
LOC107815366	13.58935	16.73382	15.91355	6.31178	4.992919	6.632137	6.147904	5.069201	4.462842	1.663051	3.460413	1.164926	2.201516	2.830466	2.513944	17.33318	12.67272	17.27349	5.283838	5.847588	4.517334	7.863867	8.817987	6.65615	1.009302	1.413726	1.436744	0	0.611439	3.365486
LOC107791482	0	0	0	0	0	0	0	0	0	0	0	0	0	0	0	0	0	0	0	0	0	0	0	0	0	0	0	0	0	0
LOC107831471	0	0	0.021109	0	0.064463	0	0.020368	0.041497	0.045597	0	0	0.041031	0.054066	0	0	0.108577	0.09384	0.054313	0.123207	0.102711	0	0.141959	0.085679	0.025381	0	0.033789	0.028701	0	0	0
LOC107766229	32.38534	28.48862	38.18865	19.38957	16.69563	29.67116	17.71498	19.24933	18.77165	14.55417	17.96943	11.89567	9.091326	11.06008	13.00673	36.72503	37.27229	32.12287	11.4304	19.53437	14.63957	27.98657	33.53401	37.67557	6.052726	3.526578	10.31811	20.13921	8.134672	0
LOC107777054	1.922846	1.995832	1.625059	1.580705	1.748724	1.875879	1.567975	2.053665	1.504377	1.128483	1.073647	0.917527	0.852252	0.874055	1.09642	2.069755	1.685628	2.588379	1.553711	1.641657	0.746933	1.977537	2.072984	1.972541	0.540236	0.966153	0.778593	1.273247	1.114301	1.100856
LOC107821393	14.21812	12.86172	12.31356	16.52011	23.43176	18.35483	17.77719	19.28381	15.18465	35.35695	35.09798	41.70661	29.83159	28.65837	16.98006	10.1227	14.24653	14.73784	36.57844	28.30275	41.64918	14.30168	16.64406	24.1971	32.27665	26.91729	28.29932	22.67948	54.70998	16.34066
LOC107829036	0	0	0	0	0	0	0	0	0	0	0	0	0	0	0	0	0	0	0	0	0	0	0	0	0	0	0	0	0	0
LOC107760815	2.438808	2.421718	2.373138	1.38502	1.320578	2.622016	2.045533	1.897176	1.982094	0.816378	1.505903	1.568343	1.45876	1.350165	1.270243	2.061531	1.934106	2.333853	1.071153	1.354848	1.128716	1.975915	3.499696	3.005586	0.690312	0.253241	1.07555	0	0.350486	0
LOC107813196	11.82434	10.87096	7.183033	2.067277	1.223738	1.635541	1.043956	0.708985	0.746573	1.213708	0.848518	1.05153	0.654302	0.678936	0.496803	6.286577	9.65303	6.676103	0.631501	0.526452	0.72524	1.097202	1.372349	1.047968	2.141896	0.048107	1.103323	0	3.329041	0
LOC107825781	0	0	0	0	0	0	0	0	0	0	0	0	0	0	0	0	0	0	0	0	0	0	0	0	0	0	0	0	0	0
LOC107790566	0	0	0	0	0.058596	0	0	0	0	0	0	0	0	0	0	0.049347	0	0	0	0	0	0	0	0	0	0	0	0	0	0
LOC107822017	0	0.3151	0	0	0	0	0.289988	0	0	0	0.121217	0.146046	0	0	0	0.128823	0	0	0	0.146237	0	0	0	0	0	0	0	0	0	0
LOC107809415	0	0	0	0.059465	0.232326	0.07057	0	0	0	0.384037	0.276156	5.656255	0.07307	0	0	0	0	0	1.598537	0.666311	0.538773	0.383717	0.057898	0.137211	0.663899	0.091332	0.543058	0	0.948026	0
LOC107774192	0	0.021465	0.040947	0.042674	0.02084	0	0.019754	0.140865	0.044223	0.091865	0.049544	0.07959	0	0	0	0.052653	0.022753	0.087795	0.143394	0.11954	0.107399	0.078676	0.062323	0.049233	0.059554	0	0	0	0	1.456259
LOC107817078	88.84172	92.93843	90.45177	143.3192	125.6559	192.3381	95.85716	98.05163	89.92366	147.1519	134.3512	169.6212	123.6903	130.9259	103.4983	21.50346	24.12745	27.5347	42.25056	45.50365	33.89755	20.75862	26.66322	23.15287	29.44388	29.34944	32.29816	61.68388	37.93947	29.0903
LOC107825307	7.094226	7.13983	7.434282	8.102763	9.204342	5.006786	9.199133	10.41239	5.597981	8.466837	9.140218	11.83972	8.381894	9.567482	11.9494	6.227191	5.949614	5.905561	11.50472	11.2477	14.09122	7.34211	6.871817	5.276849	5.530126	8.902156	5.066891	4.66875	7.228942	8.073252
Novel03627	0.067521	0.184612	0.014674	0.137635	0.104558	0.018148	0.127425	0.201923	0.142632	0.098763	0.260403	0.085566	0.319455	0.313066	0.450464	0.213848	0.114155	0.02517	0.051387	0.042839	0	0.112778	0.119117	0.017643	0.021342	0	0.079805	0	0.081268	0
LOC107802683	0	0	0	0	0.114584	0	0.108611	0.055321	0	0.315679	0	0.273497	0	0.353175	0.332269	0.144747	0.06255	0.048271	0.0657	0	0.05905	0.054071	0	0	0.081859	0.09009	0.076525	0	0.623424	0
LOC107777682	23.79811	13.95074	19.12786	8.233925	5.925907	6.557185	7.522802	7.663474	9.543371	3.848258	5.031332	5.758806	11.04959	7.175584	6.505345	13.18939	16.29009	17.83166	2.305653	2.731418	2.944801	8.489076	8.333312	7.874613	0.151196	1.164791	1.272096	0	1.151483	0
107816111	0	0	0	0	0	0	0	0	0	0	0	0	0	0	0	0	0	0	0	0	0	0	0	0	0	0	0	0	0	0
LOC107793287	9.491848	12.29607	10.62428	25.73988	28.51169	23.4815	26.54628	29.78221	24.14116	23.24772	24.15192	23.86919	22.54649	23.20382	25.60986	13.41332	12.81949	13.27745	33.95245	35.76999	35.43756	29.53363	28.7855	25.34966	39.73437	46.33568	31.06689	38.59405	38.20831	47.10865
107818670	0	0	0	0	0	0	0	0	0	0.699683	0	0	0	0	0	0	0	0	0	0	0	0	0	0	0	0	0	0	0	0
LOC107779444	0.508541	0.193114	0.331548	0.038393	0.149997	0.136686	0.142179	0.036209	0.198934	0.082649	0.267442	0.035802	0.094353	0.416095	0.260976	0.221063	0.286588	0.12638	0.129008	0	0	0.212348	0.112142	0.044294	0	0	0.100176	3.030658	0.204025	0
LOC107788666	2.266432	2.983606	2.495538	7.209268	13.72645	4.927532	9.082336	11.66204	9.22058	11.83609	7.769537	10.76512	6.223458	6.04405	5.479502	6.380469	1.557023	2.816202	32.75964	24.79638	44.00465	12.82868	14.48268	15.31872	42.28144	25.78923	25.0611	36.01821	25.70243	49.82645
LOC107782919	0	0	0	0	0	0	0	0	0	0	0	0	0	0	0	0	0	0	0	0	0	0	0	0	0	0	0	0	0	0
LOC107817208	160.9601	132.7032	165.5841	75.5856	70.44565	85.81635	66.30761	73.18638	100.5051	30.61903	36.29031	40.55444	29.46428	39.93999	35.00892	102.0875	150.5346	120.0026	33.34296	24.85808	56.13436	68.7484	103.9248	127.2839	2.547075	3.576468	15.02558	9.936057	8.695683	17.18154
LOC107792078	284.2236	285.9637	288.477	136.6009	93.78092	133.3582	187.7736	192.1774	268.8031	114.9594	146.2661	124.8634	78.84293	105.7241	91.16421	215.8962	279.5145	229.613	65.38678	83.1406	94.80751	248.4543	308.9638	283.8438	45.84734	47.67478	107.7317	32.01618	49.71652	20.29967
LOC107826192	114.7444	62.50654	93.19957	46.41537	45.19033	73.91298	41.32463	45.31412	71.92053	38.14805	37.53216	59.32517	49.38118	50.35793	19.48842	51.71933	79.22572	71.75683	28.56886	25.47806	44.63624	35.26836	72.91318	93.92586	7.657143	5.466196	32.50205	11.70592	3.940232	20.242
LOC107763579	0.379014	0.069085	0.197681	0.412041	0.067075	0.081497	0.063579	0.194304	0	0.295669	0.106306	0.128081	0.16877	0.248092	0.544614	0.169465	0.292928	0.056514	0.307678	0.448868	0.276534	0.189915	0.066863	0.079229	0.095838	0	0	0	0	0
LOC107824940	0	0	0	0	0	0	0	0	0	0	0	0	0	0	0	0	0	0	0	0	0	0	0	0	0	0	0	0	0	4.217083
LOC107830985	0	0	0	0	0	0	0	0	0	0	0	0	0	0	0	0	0	0	0	0	0	0	0	0	0	0	0	0	0	0
LOC107762321	5.255094	5.943811	7.187011	1.860918	1.953931	2.926153	1.651083	1.784337	2.667741	1.251884	0.984234	1.315987	2.286662	1.792745	0.755467	5.701947	5.887176	6.623413	1.823821	2.53405	0.702518	1.572486	2.778184	1.985932	0.259702	0.071454	0.809264	0	0.32964	1.058405
LOC107814565	3.925899	4.00168	3.727029	1.544342	1.416983	2.665782	0.563246	0.926872	1.551919	0.402973	1.159092	0.610973	0.632553	1.239805	0.689246	4.15741	3.792749	4.236338	1.04835	1.485729	1.13068	0.560817	0.956855	1.403777	0.587786	0.359382	0.671593	0	0.994772	0
LOC107781359	1.009139	1.5287	1.11431	3.669269	3.390806	2.023281	2.447721	1.491435	1.651591	1.063825	1.902894	1.520762	1.472548	2.365492	3.359197	1.22964	0.988087	0.894693	4.815588	4.602853	2.213819	2.254948	3.440221	2.09525	0.103448	0.341548	0.580243	0.975239	0.722188	0
LOC107806485	0.033068	0.090412	0	0.629117	0.468172	0.746596	0.083207	0.084763	0.372549	0.193473	0.069562	0.055874	0	0.036076	0.03394	0	0.031946	0.098614	0.301996	0.307706	0.120634	0.055232	0.116673	0.034563	0	0	0.039084	0	0	0
LOC107832103	1.898247	1.081261	1.804804	0.107482	0.419924	0	0.049754	0.10137	0.111385	2.776546	0	0	0.792433	0.647155	0	1.370371	1.490017	1.415224	0.180582	0.050181	0.486909	0.297239	0.523244	0.682016	40.19936	1.320636	35.54665	4.242235	23.70386	22.00715
LOC107802576	0	0	0	0	0	0	0	0	0	0	0	0	0	0	0	0	0	0	0	0	0	0	0	0	0	0	0	0	0	0
LOC107765051	10.20478	12.68236	9.553165	12.28358	10.38749	16.11182	9.127803	10.09115	7.336265	8.733173	11.13371	6.661897	7.149713	7.201358	8.862533	11.35374	11.34092	11.09292	12.02061	15.87663	8.883886	10.93593	11.26735	10.29309	14.6613	14.34814	15.68803	12.75849	8.245498	11.03106
LOC107803464	0	0	0	0	0	0	0	0	0	0	0	0	0	0	0	0	0	0	0	0	0	0	0	0	0	0	0	0	0	0
LOC107809358	1.861741	1.525166	2.272994	1.231818	0.88848	1.012044	0.91235	0.840044	1.472936	0.734332	0.777405	1.060349	1.094475	1.300796	0.322052	1.480902	2.000688	1.793492	0.573118	0.389302	0.515105	0.786129	1.439213	2.077075	0.264473	0.087319	0.420306	0	0.302126	0
LOC107798002	0	0	0	0.125042	1.221319	0	0.057883	0.353792	0.259164	3.095569	0.338737	0.058303	1.075545	0.978747	0.566652	0.051427	0	0	5.812336	1.868129	9.440953	0.633967	0.6696	1.370486	9.510421	9.698477	5.138664	0	13.95434	34.1367
Novel02239	0	0	0	0	0	0	0	0	0	0	0	0.046728	0	0	0	2.143337	1.763366	1.690713	1.571529	2.105528	2.42135	2.95628	3.805479	3.815547	0.629372	0.615693	0.849855	3.955537	2.396593	0
LOC107787331	2.667145	2.565817	3.50994	3.557328	4.064557	3.783516	3.231293	3.639879	3.16482	5.165484	4.078083	3.661577	6.103156	4.647476	4.220288	3.754279	3.364007	3.755998	4.059651	3.258986	2.939248	3.52672	3.463547	3.484624	5.33913	5.515163	3.63396	10.59659	3.566829	16.03327
LOC107770124	0	0.255624	0.121908	1.461082	0.992751	1.206204	0.117626	0	0.131664	0.068376	0	0	0.234176	0.305991	0.431816	0.052254	0	0	0.853837	0.474535	1.279015	0	0	0.219869	0	0	0	0	0	0
LOC107817966	0	0	0	0	0	0	0	0	0	0	0	0	0	0	0	0	0	0	0	0	0	0	0	0	0	0	0	0	0	0
LOC107805707	16.9921	19.41485	17.99519	8.037563	5.455439	11.15515	8.660508	8.993765	11.95293	8.749054	10.01692	9.909033	10.4902	8.639916	5.401838	12.10224	19.75706	12.81781	2.18709	1.738466	4.434267	6.279001	10.16895	9.430159	0.57035	0.767185	1.777282	0	2.654472	0
LOC107817507	0	0	0	0	0	0	0	0	0	0	0	0	0	0	0	0	0	0	0	0	0	0	0	0	0	0	0	0	0	0
LOC107760236	9.048477	10.94803	9.639941	13.34443	10.51453	12.16027	11.80929	11.25254	10.71653	10.06578	10.93931	8.588972	10.10184	9.431767	10.98174	11.79045	9.821729	11.61169	9.933725	11.09666	7.754376	12.14922	11.16931	10.68041	6.558291	6.548401	6.438255	2.789198	5.820863	7.234668
Novel04500	0	0	0.049871	0.051975	0	0	0	0	0.053862	0	0.040229	0	0	0	0	1.881131	2.10616	2.01031	0.75681	0.533851	1.203437	1.38945	1.821782	1.918854	0.072534	0.159655	0.135616	0	0	0
LOC107784350	10.16646	12.97166	26.01193	2.934254	2.467916	3.172656	8.511956	6.810831	7.65267	5.263849	4.403409	2.55387	4.847472	4.534595	5.078776	26.362	8.969866	17.29205	2.264091	2.907299	2.018502	8.324979	6.015361	6.375589	1.569719	1.251848	1.552502	1.286806	5.284323	10.01322
LOC107773965	4.092169	3.480874	4.268683	3.460145	3.62102	3.373013	2.974641	3.729557	3.457718	2.92628	3.156374	3.11146	2.885124	2.827425	3.080066	3.354438	4.744055	3.355974	2.21464	3.346304	3.607723	4.556636	5.053389	3.564278	4.828829	1.708186	3.063189	0	7.223758	8.434165
LOC107764403	1.948238	2.942387	2.612924	1.613719	1.280633	1.316602	2.007573	1.712188	1.724572	0.542794	1.288042	0.329185	0.805556	0.971626	1.028375	2.364407	1.451953	1.618493	0.677807	1.224284	0.304599	3.254048	2.258566	1.629038	0.844514	0.774523	0.263161	0	0.535972	0
LOC107829657	1.11341	0.947087	1.053896	0.672462	0.503556	0.744832	0.498061	0.549656	0.603962	0.989207	0.850122	0.585287	0.90894	0.836777	0.380925	1.475048	1.386391	1.254365	0.677887	0.648843	0.293352	0.80586	0.807506	0.801685	0.594358	0.137709	0.672602	0	0.238238	0
LOC107814661	7.542082	8.574913	7.203594	13.6057	14.03986	9.770936	15.91747	13.99188	10.36458	14.01948	17.51159	7.542572	13.94777	12.09522	23.11652	12.71608	7.39558	9.78392	16.12909	16.93912	9.053833	22.06081	11.70029	8.395376	16.91357	26.23564	6.30675	8.76469	12.61782	34.97537
LOC107790025	2.594191	1.981827	2.785681	1.555283	0.708908	1.148441	1.215921	1.173466	2.399724	0.595215	0.615266	0.451221	0.297283	0.832393	0.352404	2.359634	2.28507	2.019406	0.696813	0.645443	0.661073	1.178818	2.3892	3.788051	0.192932	0.159248	0.450901	0	0	0
LOC107799242	0	0	0	0	0	0	0	0	0	0	0	0	0	0	0	0	0	0	0	0	0	0	0	0	0	0	0	0	0	0
LOC107812722	0	0	0	0	0	0	0	0	0	0	0	0	0	0	0	0	0	0	0	0	0	0	0	0	0	0	0	0	0	0
LOC107790736	4.387041	2.688477	4.240944	2.398367	3.212642	2.155001	1.998404	1.42181	2.911529	2.286474	1.856325	2.140705	3.578585	3.424499	1.940836	3.94562	5.517033	4.511346	1.458305	1.343687	1.690101	1.137021	1.801394	2.885598	1.291008	0.420983	2.19027	0	5.280195	0
LOC107784817	0.155383	0.410677	0.175593	0.999464	0.687468	0.233879	3.427595	2.907528	0.846109	1.333363	2.843725	0.249417	1.089743	0.796724	5.310728	0.532643	0.210159	0.150598	1.087939	1.590469	0.410966	2.867802	1.836584	0.584664	0.569711	1.36209	0.257111	0	0.52365	0.960759
LOC107812964	0.621104	0.723298	0.809869	0	0.030533	0	0.028941	0	0	0	0.024195	0	0	0.037644	0	0.951408	0.633374	0.437334	0.070028	0	0.06294	0.028817	0.091309	0	0	0	0	0	0	4.267088
LOC107770590	0	0	0	0	0	0	0	0	0	0	0	0	0	0	0	0	0	0	0	0	0	0	0	0	0	0	0	0	0	0
107763427	0	0	0	0	0	0	0	0	0	0	0	0	0	0	0	0	0	0	0	0	0	0	0	0	0	0	0	0	0	0
LOC107829794	18.52535	13.49496	16.10369	17.25579	14.71857	21.81053	12.5836	12.8189	13.65672	11.00411	11.16041	14.05259	11.69102	19.56978	12.51972	13.7565	15.75444	14.88133	8.273873	8.718466	12.55259	8.825007	13.7514	13.97659	2.721532	2.17831	5.39677	4.664874	3.76849	0
LOC107767447	26.99912	23.60066	31.2717	17.29517	15.2327	16.056	18.94309	17.0973	24.76088	11.83664	13.05108	12.21277	12.20249	12.84129	8.909854	23.08018	29.85023	27.03976	8.99542	9.552718	9.695143	18.03199	26.3782	31.02604	7.301376	3.889803	10.60795	5.261086	7.969008	0
LOC107830736	0.829778	0.756241	0.450818	1.409507	1.743826	1.895741	1.826924	1.772463	1.265928	0.809139	1.672793	0.525765	1.616512	0.905247	1.277493	0.927528	1.603271	1.082611	1.263003	1.491613	0.662175	1.645803	1.189369	1.409337	1.442502	0.144322	0		0.499356	0
LOC107761045	4.507198	3.828587	4.412517	4.559016	3.755939	5.033974	4.330922	4.748409	4.806718	5.248502	4.725333	4.510216	5.796889	6.444783	2.290575	3.880519	4.269782	3.066688	5.772496	4.47909	5.746914	2.813923	4.323033	4.482236	1.770398	1.765744	6.93046	0	6.530843	10.82279
LOC107821731	19.89619	20.02948	18.08951	20.87582	20.61455	25.70174	17.28378	15.26517	20.77609	19.50028	18.43554	24.48432	18.81511	18.77106	12.76306	0	0.049034	0.075681	0	0	0	0	0	0	0	0	0	0	0	0
LOC107831657	3.420075	5.730441	3.81916	5.243471	3.794524	2.913314	7.612728	5.529711	3.976597	3.719813	6.917765	2.80047	3.338687	3.214515	6.966538	7.391067	3.736152	5.334989	3.523847	4.295212	2.111435	7.557928	4.9428	2.942221	1.463513	4.42933	1.212676	0	0.759944	1.62668
LOC107829039	1.711643	3.103985	2.171112	5.18997	4.590087	3.999673	10.65002	8.953923	5.739163	4.615484	10.65488	4.220086	5.774603	4.858838	14.5562	3.462121	2.682867	2.343861	4.413034	4.698392	2.883158	11.8585	8.19596	5.732129	4.858042	12.00534	2.539102	8.743333	3.699797	1.079933
LOC107769438	53.68563	44.29952	55.49682	32.07377	26.70528	38.21908	25.06989	27.02638	32.55725	25.89216	24.31624	31.25825	20.51345	28.17614	19.50889	27.89649	56.91114	38.72681	24.58803	23.0754	29.24499	23.99239	37.49947	29.42099	15.59289	18.37207	28.98217	20.75287	13.27236	35.88609
LOC107817340	0	0	0.074157	0	0	0	0.107329	0.036445	0.040046	0.041594	0	0	0.047484	0	0.043779	0.095359	0	0.031801	0.216414	0.144331	0	0.035622	0	0.089165	0.107857	0	0.100828	0	0	0
LOC107813798	5.070431	5.625654	4.867544	3.155572	2.106775	4.40847	4.659578	4.445331	4.511979	1.07485	1.762237	1.340966	1.963296	1.154419	1.900647	4.402764	5.792965	4.930724	2.10279	3.207603	2.211631	3.240275	4.394674	3.548437	1.114881	1.22698	1.771797	0	0.424537	2.726195
LOC107806196	0	0	0.032179	0	0	0.079597	0	0.063258	0	0.21658	0.103827	0.031273	0.041208	0.040384	0	0	0.107286	0.055196	0.075126	0.125257	0.033761	0	0	0	0.046801	0	0.26251	0	0	0
LOC107820229	0	0	0	0	0	0	0	0	0	0	0	0	0	0	0	0	0	0	0	0	0	0	0	0	0	0	0	0	0	0
LOC107774277	0	0	0	0	0	0	0	0	0	0	0	0	0	0	0	0	0	0	0	0	0	0	0	0	0	0	0	0	0	0
LOC107778374	0.303043	0.212451	0.445802	0.14783	0.185644	0.451119	0.058656	0.119505	0.131312	0.045462	0.081728	0.118163	0.1038	0.076293	0	0.364799	0.540489	0.486621	0.307507	0.236634	0.14882	0.136273	0.164495	0.243646	0	0.097307	0.220414	0	0	0
LOC107803631	8.039845	10.18907	9.118521	13.03562	16.39621	13.24396	19.44867	16.45133	16.35975	12.38227	9.508819	12.82663	19.78234	14.6812	14.73296	7.35413	8.433876	7.794832	12.57005	9.282264	15.137	15.0423	13.30069	14.6065	10.12118	15.55597	4.69005	4.935301	21.596	2.133544
LOC107812270	7.67545	9.862639	9.797777	5.91993	5.323258	7.24842	6.553726	6.114997	6.102421	3.809695	4.994136	3.680354	4.811046	4.978861	4.222823	7.549045	7.715744	7.42362	3.29784	3.743656	3.405473	4.937409	6.831248	7.155094	1.267653	0.625395	1.307642	0	2.49678	0
LOC107796542	129.6433	164.3778	122.3172	74.7846	66.93825	55.90881	133.0533	110.7192	119.3616	52.49037	107.5922	64.26664	107.1396	101.0929	150.7296	202.1548	127.2886	140.6366	68.59575	74.10918	71.44339	172.1376	135.6239	117.8432	16.99029	27.14452	27.82231	20.51657	17.01035	47.6145
LOC107783513	0.405908	0.257346	0.2608	0.559592	0.421639	0.53127	0.532882	0.769033	0.795306	1.961848	1.435489	1.580424	0.530447	1.386236	2.535903	0.236726	0.221645	0.328936	1.432651	2.672315	1.046203	0.928522	0.513706	0.830064	2.744456	9.945236	1.898143	1.262094	1.86922	2.182426
LOC107775289	2.37501	2.125174	2.439918	2.855811	2.369037	3.249815	1.81093	2.361336	2.756799	2.40015	2.604015	3.429247	2.451611	1.790159	1.152341	1.737681	2.294459	1.867253	1.314543	0.986283	1.575308	3.13743	2.970971	3.520437	0.272976	0.240338	1.173866	0	0.623681	0
LOC107800840	0	0	0	0	0	0	0	0	0	0	0.064099	0.077228	0	0.099727	0.187648	0	0	0	0	0.154659	0.08337	0	0.080632	0	0.115574	0.127195	0	0	0	0
LOC107801161	6.957232	7.913905	7.033	6.903176	7.513979	6.917479	9.608551	9.058206	7.628567	8.756523	10.36816	7.247644	8.07487	8.541126	12.68863	17.52867	12.71819	14.75475	11.51849	14.66172	9.636938	16.20731	12.99635	11.37228	8.377196	14.07188	8.058019	6.234843	11.08092	12.93763
LOC107810907	15.05473	13.63934	13.12547	13.03355	12.9667	13.2167	11.28216	12.59677	12.54501	13.42071	10.77501	11.74029	12.94122	13.65425	8.320178	10.05709	15.3617	12.65184	11.25399	11.60491	13.6083	10.89873	13.00425	14.10581	13.17721	14.6261	15.16131	6.370567	15.65371	22.03211
LOC107795492	3.242818	2.053773	1.863349	1.145255	1.459047	1.477299	1.613495	1.127086	2.734891	0.696747	0.924963	1.346594	2.447424	3.35787	0.507713	0.81917	3.292132	1.475182	0.278863	0.139485	0.451145	0.826219	2.521013	1.953209	0.06949	0	0.064962	0	0	0
LOC107812917	4.429171	6.786241	5.858976	5.059371	4.061223	6.245681	6.460759	5.923415	6.267584	5.289211	6.661582	6.697435	4.108846	3.676528	5.105992	5.620627	6.418427	5.9582	6.83933	8.661021	7.58134	7.183403	8.011816	5.602207	2.881066	3.170751	4.362436	4.590556	1.854227	0
LOC107790658	0.202043	0.368275	0.263448	0	0.044695	0.162916	0.169463	0.215789	0.094844	0.049254	0.283346	0.341384	0	0.11021	0.103686	0.037641	0	0	0.051255	0	0.092134	0.042183	0	0	0	0	0	0	0	0
LOC107768953	3.804341	3.540181	4.142493	3.482819	2.94111	5.553968	2.821396	2.1214	3.158119	1.874381	1.937524	1.894576	1.337383	2.05333	1.109749	3.043894	3.327125	3.642403	2.519407	1.998678	1.935684	3.009888	3.426326	4.101863	0.911339	0.167162	3.691801	0	0.385588	0
LOC107804660	19.69099	16.60784	22.37217	9.897537	9.132255	10.72439	10.39474	9.55603	12.97317	9.895356	9.689358	10.61607	10.62365	10.92939	9.130088	15.8322	17.47961	17.61012	7.536714	7.013568	9.530615	9.123791	11.8458	12.63747	3.657876	3.304653	4.899614	0	7.69206	0
LOC107779220	277.4616	254.6281	281.1066	216.5884	193.5277	228.1907	193.9325	179.3987	237.8391	132.7941	146.6862	147.4562	142.6233	145.5597	125.4123	236.5865	260.9776	251.5905	122.2752	127.6129	140.3398	185.9169	227.6914	271.4438	35.56679	45.1237	74.87199	44.51826	43.38492	29.32627
LOC107778008	0.117443	0.160553	0.255228	0.265994	0.155882	0.126266	0.246262	0.200694	0.330783	0.343566	0.86469	0.595317	0.392219	0.448437	0.180811	0.087519	0.11346	0	0.35752	0.198698	0.053555	0.09808	0	0.061376	0.37121	0.081707	0.416426	0	0.565414	0
LOC107819700	0	0	0	0	0	0	0	0	0	0	0	0	0	0	0	0	0	0	0	0	0	0	0	0	0	0	0	0	0	0
LOC107762284	12.34236	15.30898	12.71773	17.18141	17.9536	13.52846	18.22974	16.56438	17.97483	16.73208	16.04243	17.59902	21.91647	20.03315	17.42603	16.6453	13.26154	15.03701	17.83931	15.73757	16.36302	17.04496	15.82565	17.1164	13.01643	18.51386	9.535357	8.61125	20.57978	37.22666
LOC107818914	2.54978	2.722177	3.31415	2.529969	2.320676	2.19306	2.871848	2.593573	4.058138	1.72861	2.622264	1.866908	2.243727	2.25184	1.694835	2.3706	2.909042	3.059655	1.059577	0.801148	1.262383	3.183942	3.170124	4.086378	0.307019	0.3041	0.774935	1.736622	0.58455	1.501493
LOC107800953	3.192923	6.44519	3.968334	3.896678	2.965406	2.83701	4.29372	3.810349	2.03147	3.6024	4.921834	1.538226	1.791891	1.986357	5.362573	7.806692	2.090412	2.773924	2.945416	4.397484	2.093751	1.696859	2.25775	1.130801	0.934141	1.248367	0.966836	0	0.762241	0
LOC107788628	0	0	0.084791	0.176735	0	0	0	0	0	0.095115	0	0	0	0.106413	0	0	0	0	0	0	0	0	0	0	0	0	0	0	0	0
LOC107798080	2.600833	3.263063	2.943508	4.222841	4.244118	3.449874	4.384109	4.134199	3.773655	3.639526	4.316498	2.775106	4.258323	3.445391	4.73216	4.253848	2.651307	3.393305	3.804483	3.943082	2.52603	4.837689	4.193448	3.795155	3.811373	3.959607	2.794523	8.454379	5.041057	3.132729
LOC107826470	2.494274	6.819671	2.759552	0.718991	0.702258	0.731359	5.420325	7.071628	3.193275	2.653345	2.941478	0.095783	2.776661	1.11319	3.840082	2.1122	1.642963	1.436954	0.230092	0	0	4.639484	3.500183	0.237001	0	0	0	0	0	0
LOC107771202	9.103787	13.37738	10.15022	14.76196	10.8284	9.291208	20.74929	18.629	15.97924	13.09086	14.77938	13.01362	16.92749	14.93366	24.1045	15.73137	13.89359	12.12362	6.727597	6.250231	9.235369	19.28249	16.03119	14.9969	1.918326	4.956752	3.430707	4.71775	3.493606	4.078992
LOC107790631	0	0	0	0	0	0	0	0	0	0	0	0	0	0	0	0	0	0	0	0	0	0	0	0	0	0	0	0	0	0
LOC107791407	0	0	0	0	0	0	0	0	0	0	0	0	0	0	0	0	0	0	0	0	0	0	0	0	0	0	0.034166	0	0	0
LOC107764795	5.614705	5.315193	5.132653	6.530587	8.365909	7.789025	4.132026	5.106863	4.3531	5.192463	4.953043	6.855063	8.992464	8.338403	5.390987	4.076108	5.459272	6.049446	8.748288	8.549377	8.655644	4.56798	3.418851	3.710399	5.404167	5.040292	5.180462	7.771563	13.07961	2.239778
LOC107806584	0	0	0	0	0	0	0	0	0	0	0	0	0.08716	0	0	0	0	0	0	0	0	0	0	0	0	0	0	0	0	0
LOC107794896	0	0	0	0	0	0	0	0	0	0	0	0	0	0	0	0	0	0	0	0	0	0	0	0	0	0	0	0	0	0
LOC107789997	0.236235	0.234872	0.186686	1.031173	0.969169	1.292997	0.270192	0.293594	0.201625	0.649194	1.129419	0.508017	0.502053	0.328008	0.991903	0.880213	0.435698	0.944661	0.849899	0.599516	0.724697	0.215221	0.397807	0.404039	0.162913	0.388468	0.152297	1.535832	0.723749	2.655777
LOC107805673	0	0	0	0	0	0	0	0	0	0	0	0	0	0	0	0	0	0	0	0	0	0	0	0	0	0	0	0	0	0
LOC107759801	9.043766	11.13973	9.444597	7.857636	6.664214	7.234292	8.154948	8.993135	8.983319	5.116718	6.601214	5.632531	5.188446	5.65713	5.322259	9.20058	9.124688	10.76961	6.984534	5.117676	8.670322	9.640628	10.48199	10.09777	5.619474	4.810167	6.055886	2.207362	2.823407	11.45098

LOC107767914	0	0	0	0	0	0	0	0	0	0	0	0	0	0	0	0	0	0	0	0	0	0	0	0	0	0	0	0	0	0
LOC107777868	1.003729	1.118058	0.937151	1.39767	1.611855	1.818536	1.714921	1.334065	1.239009	0.779385	1.107857	0.800868	1.158749	0.912515	1.144666	1.163524	0.826029	1.219488	0.905346	1.273628	0.593326	1.381544	1.180473	1.651356	0.376005	0.232769	0.351504	0	0.447436	0
LOC107820343	0.050953	0	0	0	0	0	0	0	0	0	0	0	0	0	0.209187	0	0.049225	0	0.051704	0.086206	0.04647	0	0	0.053256	0	0	0	0	0	0
LOC107758910	0	0	0	0	0	0	0	0	0	0	0	0	0	0	0	0	0	0	0	0	0	0	0	0	0	0	0	0	0	0
LOC107782685	0.05756	0.02623	0.025018	0.052147	0	0	0.048278	0	0	0	0.242167	0.048628	0.064077	0.062795	0.265852	0.021447	0	0.021457	0	0	0	0.120175	0	0	0.072774	0	0	0	0	1.779516
LOC107813766	1.814185	2.638413	1.912571	1.713498	1.502843	1.203482	2.266265	2.17672	2.246823	1.881978	4.195248	2.413146	3.136788	2.610842	2.852471	1.869694	2.12556	1.49644	0.783366	1.502023	0.422443	3.642623	3.370699	2.622384	0.780828	0.268543	1.049299	0	2.415824	0
LOC107770346	7.452212	7.204052	6.912679	7.398418	6.889156	7.192917	7.748212	8.055844	7.376464	6.756078	7.713031	6.617667	6.758645	6.805301	7.06226	8.019602	7.93551	8.023274	6.184886	6.445044	5.037693	8.152236	7.035367	8.560489	5.689239	6.062509	5.740622	8.513394	4.470372	2.94429
LOC107791327	0	0	0	0	0	0	0	0	0	0.049637	0	0	0	0	0	0	0	0	0	0	0	0	0	0	0	0	0.060164	0	0	0
Novel03296	0	0	0	0	0	0	0	0	0	0	0	0	0	0	0	0	0	0	0	0	0	0	0	0	0	0	0	0	0	0
LOC107779706	21.22837	36.39943	21.80072	36.80882	26.03585	11.88924	164.5113	150.1665	98.35286	28.35465	77.40379	24.3573	39.78921	22.45895	33.84774	34.17727	16.02516	21.78925	31.10933	28.31476	52.36375	140.4664	180.5857	69.35004	18.03838	7.418777	16.39617	21.18319	9.982415	39.68271
LOC107798736	0.527384	0.269161	0.348417	0.267558	0.373331	0.612362	0.424646	0.558758	0.455521	0.164565	0.266257	0.178219	0.117418	0.276168	0.324775	0.503049	0.183419	0.456097	0.256873	0.463976	0.192393	0.651839	0.502402	0.749661	0.186697	0.146763	0.099732	0	0	0
LOC107802191	2.141446	2.693296	2.475813	22.21347	14.9128	13.99808	17.99715	18.64474	14.21412	3.278443	3.678878	2.568997	2.169337	2.686644	2.923235	2.090508	1.779182	1.468816	32.09508	38.27733	27.69403	25.37707	22.64787	14.95143	4.62972	8.253673	5.871958	10.72006	2.680517	1.324088
LOC107787327	0	0	0	0	0	0	0	0	0	0	0	0	0	0	0	0	0	0	0	0	0	0	0	0	0	0	0	0	0	0
Novel00572	0.197128	0.230989	0.244799	0.127563	0.274107	0.151383	0.18896	0.312802	0.185073	0.247146	0.17772	0.166539	0.407542	0.24578	0.028904	1.594916	0.952212	1.427683	0.257184	0.500271	0.616403	1.12887	1.1178	1.560004	0.284834	0.078368	0.432695	0	0.271156	0
LOC107819417	8.257451	7.425306	7.489074	6.957174	7.038822	6.00729	7.202915	6.961318	7.674934	4.562832	4.786507	4.278689	3.952693	4.44418	5.650147	8.737916	7.259426	8.988169	5.55813	4.423982	3.966292	7.148866	7.696356	7.537465	2.540382	1.761745	2.342314	1.968415	2.915316	5.105706
LOC107769923	0	0	0	0	0	0.086108	0	0	0	0	0	0	0	0	0	0	0	0	0	0	0	0	0	0	0	0	0	0	0	0
LOC107770077	75.77468	11.88725	12.47798	5.939491	5.801266	6.900214	4.804285	3.596885	5.44245	38.96379	4.500357	3.148355	12.75289	13.02486	3.541578	8.845527	9.600617	6.071222	9.663884	8.93199	6.986305	4.841201	6.513384	6.058991	281.4736	8.354134	305.8728	103.6413	298.357	34.1367
107776696	0	0	0.641304	0	0	0	0	0	0	0	0	0	0	0	0	0	0	0	0	0	0	0	0	0	0	0	0	0	0	0
LOC107796680	3.092299	3.019553	2.742929	1.800943	2.317453	2.306865	1.640877	2.075971	2.369948	1.353845	1.770059	1.439519	1.650942	1.927741	2.267037	2.398442	2.377743	1.999617	1.664982	1.601554	1.410114	2.134482	2.755445	3.100149	1.236712	0.395147	1.491775	0	1.974863	0
Novel03014	5.349869	6.118583	5.820758	3.23114	3.001241	2.687915	2.184927	2.315413	2.872455	1.500256	1.569184	1.166855	1.673782	1.773821	0.879271	4.911754	3.074029	5.057383	2.164375	2.114914	1.62639	1.343265	2.405733	3.508506	0.729436	0.316246	0.516594	0	0.420853	0
LOC107810198	11.17821	11.9491	15.00952	8.492585	7.211754	6.718968	7.943634	7.761883	8.498509	2.481595	3.862615	3.537973	2.940597	3.268369	3.27328	10.77861	11.98171	10.35125	3.857233	4.251113	5.053291	11.00318	10.99652	13.0976	0.448009	0.896465	1.789488	0	0.465267	0
LOC107818872	13.47635	11.71556	11.00249	17.99339	15.84968	18.19443	14.52591	13.88023	14.98634	21.10296	19.83034	22.22141	23.9341	20.86641	15.62959	12.9901	13.31952	12.19564	15.48098	17.68563	14.48081	13.33074	14.62827	13.19959	14.71643	15.25267	21.17049	12.12263	17.54614	12.22818
LOC107774887	0	0	0	0	0	0	0	0	0	0	0	0	0	0	0	0	0	0	0	0	0	0	0	0	0	0	0	0	0	0
LOC107815981	33.01245	29.01703	37.8163	11.96304	9.931941	18.53493	10.82946	11.97219	13.15504	7.010552	8.179085	10.59814	9.718314	11.44476	7.981347	19.24339	32.1763	26.253	6.550868	4.840553	9.50072	12.3144	16.63028	16.86892	1.298509	0.714535	3.554979	5.24635	2.472302	0
LOC107776875	0	0	0	0	0	0	0	0	0	0	0	0	0	0	0	0	0	0	0	0	0	0.033383	0	0	0	0	0	0	0	0
LOC107781403	55.38197	55.91394	58.02875	54.60502	52.55289	49.02374	51.75404	47.92343	47.43552	33.30502	36.97639	31.51044	31.74999	32.0398	42.79504	58.02378	56.16518	59.97263	42.16627	41.68037	40.2657	51.29488	52.82279	52.97532	20.60846	30.05241	19.36994	17.68122	21.25546	38.2182
LOC107774819	43.79946	39.67533	42.81511	34.52801	32.01764	40.15324	28.59142	28.21677	34.6889	23.51158	23.22654	30.90607	25.17506	27.50156	16.55502	37.44734	37.36609	41.53079	24.4665	22.7597	26.38791	23.82991	34.58581	47.06531	11.89929	8.653365	22.01197	30.91849	16.17121	4.11266
LOC107763947	0	0	0	0	0	0	0	0	0	0	0	0	0	0	0	0	0	0	0	0	0	0	0	0	0	0	0	0	0	0
LOC107798742	0	0	0	0	0	0	0	0	0	0	0	0	0	0	0	0	0	0	0	0	0	0	0	0	0	0	0	0	0	0
LOC107787105	11.77918	9.462701	9.648059	11.61198	10.26459	9.199727	15.34508	12.60351	13.14285	8.728107	12.07554	9.316185	11.55835	11.91308	12.20007	12.64652	13.52418	11.9583	7.520391	7.935157	8.620371	14.3221	14.6534	13.69618	4.933978	5.57953	4.781735	7.681258	5.688154	8.855009
NitaMp004	0.503397	0.676103	0.276374	0.52806	0.304774	0.569698	0.088889	0.181101	0.323365	0.439204	0.315826	0.4029	0.294941	0.60699	0.598253	0.335644	0.6399	0.612338	0.322618	0.313776	0.773232	0.508905	0.303809	0.359997	0.803933	0.663575	0.375773	7.578945	1.78576	9.829194
LOC107776571	0	0	0	0	0	0	0	0	0	0	0	0	0	0	0	0	0	0	0	0	0	0	0	0	0	0	0	0	0	0
LOC107787307	1.14846	0.864648	0.824709	0.972591	0.839497	0.939472	1.005144	1.002607	0.796949	0.681674	1.120419	0.69605	1.111723	0.844355	0.614999	1.0977	0.482395	1.023748	0.734695	1.267198	0.569247	0.938259	0.902907	1.122085	0.726001	0.277913	0.531152	0	1.201977	0
LOC107830153	4.647938	5.96087	3.266155	4.614226	5.073276	2.693049	8.940702	9.155449	5.957612	4.885143	7.201327	4.185353	7.931631	7.044287	10.08379	5.185104	4.275195	4.004733	6.580376	7.204442	9.620241	7.856202	8.543273	6.167072	5.735655	8.945741	3.223724	1.990379	5.895692	8.604462
Novel02880	1.879095	3.674875	2.211973	2.766342	2.598041	2.314877	2.692466	1.605549	1.764177	1.565136	1.372523	2.348194	2.701953	1.494792	1.68757	0.962709	1.928824	1.255013	0.556143	0.496745	0.92829	0.817334	0.794212	0.941097	0.445453	0.762598	0.231348	2.799621	0.565414	0
LOC107760525	0	0	0	0	0	0	0	0	0	0	0	0	0	0	0	0	0	0	0	0	0	0	0	0	0	0	0	0	0	0
Novel01265	10.11725	10.64563	10.91344	9.499015	7.935089	6.476926	8.87149	8.801625	9.239469	5.874514	7.352194	7.226382	7.423119	8.880108	4.248028	0	0	0	0	0	0	0	0	0	0	0	0	3.288756	0	0
LOC107805627	0.051708	0.047125	0.022474	0	0	0.027796	0.021685	0.02209	0.024273	0	0	0.021842	0	0.05641	0	0.134864	0.249769	0.134926	1.233028	0.699857	0.613056	1.381835	0.547314	0.783649	5.52411	1.942577	2.750138	1.848911	1.120224	3.197157
LOC107769565	1.531451	3.847685	2.086837	7.668291	5.713515	3.849243	16.95879	11.47601	7.771857	5.287295	11.20291	4.213632	4.239043	3.29632	10.02579	6.353925	2.619133	3.008696	4.32603	5.39209	2.755669	15.10549	11.7012	5.472568	1.308256	2.303678	2.201414	1.480005	0.697441	1.279621
LOC107782372	1.255065	3.991758	2.115207	0.60332	0.521285	0.495679	0.365215	0.503353	0.240472	0.449576	0.143682	0.086556	0.285134	0.44709	0.078867	0.744403	1.435202	0.343728	0.077973	0.260008	0.14016	2.909117	0.835936	0.348027	0	0	0	0	0	1.583726
LOC107762022	0	0	0	0	0	0	0	0	0	0	0	0	0	0	0	0	0	0	0	0	0	0	0	0	0	0	0	0	0	0
LOC107819506	2.378598	2.306169	2.287629	1.833948	1.074761	2.883745	2.122375	2.248545	1.853025	1.480489	2.590528	1.111641	1.408451	1.545918	2.129669	2.526804	1.760113	2.942999	1.129787	1.584017	0.923115	3.465685	3.124801	2.062942	0.831798	0.281672	0.657966	0	0.730942	0
LOC107765618	2.148913	3.121311	1.459379	1.642618	1.723234	1.732758	2.027684	2.065601	1.576168	2.553844	2.024788	3.233791	3.513544	3.516538	1.171717	2.051759	2.270659	2.303027	2.180569	2.215484	2.266071	1.065552	2.369355	2.667177	2.462172	1.495028	2.381102	0	2.263107	8.304409
LOC107798120	5.14007	1.747408	3.156077	2.402237	2.057552	3.20169	1.334418	1.324516	2.374563	1.511621	1.430246	2.79159	1.316968	2.447746	0.20935	1.033596	2.364638	1.976898	2.028362	1.725455	1.86025	0.647298	0.683681	1.023314	1.495712	0.340573	2.169696	0	2.74957	0
LOC107769896	0	0	0	0	0	0	0	0	0	0	0	0	0	0	0	0	0	0	0	0	0	0	0	0	0	0	0	0	0	0
Novel02660	0	0	0	0	0	0	0	0	0	0	0	0	0	0	0	0	0	0	0	0	0	0	0	0	0	0	0	0	0	0
LOC107786607	0.727497	1.193442	0.632397	18.71773	21.41498	10.63721	10.5765	10.85716	6.511323	29.03815	20.37084	14.58673	8.422538	15.5558	7.616163	0.505989	0.609113	0.867807	29.52853	22.93436	57.94473	5.184439	6.844802	4.004664	89.15705	37.04866	66.55181	76.30524	33.38981	44.98222
LOC107783682	7.035205	6.142322	7.503117	8.033873	11.82267	11.37734	6.842863	7.021334	7.770542	13.02864	11.69018	13.8849	13.689	15.28563	8.009562	5.639145	7.310628	6.743628	9.53845	9.302031	10.19048	6.813354	6.101222	8.403651	14.79948	8.966372	20.75268	12.68361	15.93881	25.58808
LOC107826724	0.053972	0.147566	0.117292	0.024448	0.047758	0.058026	0	0	0	0	0	0	0	0	0	0	0.26143	0.182495	0.181073	0.054767	0	0	0	0	0	0	0	0	0	0
LOC107797222	12.23753	13.99453	12.70441	5.119602	4.896999	6.075613	8.825858	8.225006	9.696245	2.166207	3.388662	2.996221	5.466551	3.954143	3.960085	13.65009	11.25772	13.16239	4.112903	3.164977	5.473786	7.909018	13.13194	11.52789	2.463688	2.440266	2.30315	0	6.567053	14.45857
LOC107823793	17.72853	16.02384	19.67773	5.707683	5.315556	8.900077	3.625254	4.381578	6.809044	3.428938	3.493081	3.280209	4.485375	3.676381	3.383569	12.93836	14.43785	15.12898	4.162904	2.912664	7.215793	4.894401	5.75107	7.963245	0.926209	0.713536	1.385369	5.239012	1.058076	0
LOC107821288	3.393464	3.816023	3.949015	5.54119	6.150816	4.36924	6.909029	5.798936	5.511151	6.858286	7.464719	4.901462	8.89576	6.508535	9.184899	3.905385	3.701387	3.968383	6.623093	6.331607	4.392756	7.004935	4.683003	4.776803	8.63264	10.41452	6.695451	5.871307	8.03692	12.69091
LOC107769272	0.427009	0.518888	1.113569	0.128949	0.251897	0.306058	0.119384	0.486464	0.935421	0.138796	0.099806	0.240499	0.316902	0.155282	0	0.530346	0.687544	0.742824	0	0.240813	0.389439	0.237738	0.5022	1.190159	0	0	0.16823	0	0	0
LOC107764846	0.390894	0.296876	0.283163	0.649238	0.749425	0.700431	0.109286	0.723645	0.550482	0.190585	0.045682	0.165119	0.290099	0.071074	0	0	0.188818	0.048571	0.132217	0.110223	0.178251	0.272038	0.344794	0.068094	0	0	0	0	0	0
NitaMr003	1.320498	0	0	0.398768	0	0	0	0.37609	0.413247	0	0	1.487458	0	0	2.258872	0.328012	0.850475	0.984487	0.8933	0	1.204317	0.367594	0	0.460061	1.113007	0.612459	0	62.95619	8.476464	54.43226
LOC107823735	5.819974	2.169895	3.334459	4.55361	4.56468	5.261722	3.661097	3.955591	2.607841	4.127425	3.98822	4.916875	3.681179	4.329072	2.172168	1.47854	3.19465	2.563977	5.368825	3.468687	5.307914	3.313917	2.916819	3.179768	3.679108	2.024517	3.908375	18.91865	5.731246	16.35717
LOC107829102	14.47682	14.15339	15.38728	12.81719	9.432849	14.07866	9.217133	8.77102	11.1203	5.518357	7.567224	7.838586	8.717181	8.255692	5.268053	8.287255	13.73154	12.85358	5.608938	6.67985	7.501763	4.890942	10.738	10.24793	0.332784	1.007172	2.955439	0	1.900815	0
LOC107803552	5.623529	6.076412	6.138293	8.085799	8.302871	7.843744	9.826449	8.909077	7.29947	7.581562	11.94916	5.407763	7.88452	8.760956	12.87732	7.39715	6.225085	6.979692	7.068115	8.505151	3.195757	10.98485	7.741112	6.389784	3.689581	6.653984	3.159633	1.52334	5.537796	13.82942
LOC107794787	50.42558	36.45805	45.09961	35.65081	31.97305	38.10611	30.21007	31.98634	41.94566	27.20115	33.74636	34.08722	28.15254	28.00754	24.81586	41.66044	44.05111	49.59262	25.66196	23.0138	22.19073	32.54378	40.0511	55.02766	14.7272	8.957044	23.00629	13.70115	8.670239	16.58452
LOC107778391	125.2577	64.42271	62.05902	103.1996	114.4015	77.93207	26.60926	43.6517	27.76503	25.59566	25.89389	31.29688	32.7476	30.08689	31.35733	74.30641	83.29318	57.77498	112.4157	88.88408	159.7963	40.60749	39.26688	35.48967	71.91158	68.90918	47.25217	187.4346	128.2532	45.95644
Novel02885	0	0	0	0	0	0	0	0	0	0.103815	0.037326	0.224857	0.118516	0.232292	0.109271	0.039668	0	0	0	0.09006	0	0	0.093907	0	0	1.036948	0.314577	0	0.51255	0
LOC107811097	9.946908	10.34051	9.843863	2.614295	1.740991	5.194298	0.97181	0.747157	1.949817	0.852703	0.551851	0.424788	0.5354	1.025535	0.403882	5.913667	8.933713	6.845393	2.240521	2.03425	0.717765	1.20496	2.140429	2.079301	0.165836	0.121674	0.232545	0	0.420993	0
LOC107774700	3.149986	3.527012	4.694084	8.968858	4.778254	3.193099	1.132298	3.537312	2.450372	2.632829	0.946617	0.684307	4.809062	12.96044	6.558491	10.12724	4.347359	4.898192	3.926991	6.699745	4.760706	2.329991	2.222797	2.633891	0.113787	0.250456	0.638234	0	0.86658	0
LOC107772491	0.197846	0.405703	0.558946	0.089619	0.525202	0.106354	0.622283	0.211306	0.41793	0.530544	0.416189	0.208932	0.495551	0.593561	0.609192	4.533631	2.054712	3.060681	4.215972	4.476993	1.669061	4.502421	4.930003	5.324809	2.814049	2.133485	2.455301	0	4.762508	15.2914
LOC107765247	4.721063	5.975927	5.348396	5.42551	4.187178	6.485643	5.820479	5.434431	5.745629	2.099342	4.421546	2.243523	1.72752	2.157942	3.241597	5.725125	4.560944	4.970022	3.914549	5.287948	1.94354	9.482519	7.971917	6.590695	0.511222	1.474987	1.175399	0	0.105226	0
LOC107772681	17.95581	13.99969	12.99213	24.25954	23.90926	19.15591	8.87949	8.779487	12.76508	23.91909	24.57477	33.93423	38.05163	30.94972	10.90137	13.38069	20.75602	17.4364	9.268137	10.24332	13.7571	5.489633	9.612787	10.3058	3.499284	2.069982	16.68344	0	8.49466	0
LOC107760460	0.19472	0.118309	0.056422	0.088203	0.028717	0	0.272201	0.526852	0.030469	0	0.136538	0.10967	0.14451	0.21243	0.033309	0.241843	0.094058	0	0.032931	0.16472	0.059196	0.596259	0.257634	0.169601	0	0	0	0	0	0
LOC107790690	0	0	0	0	0	0	0	0	0	0	0	0	0	0	0	0	0	0	0	0	0	0	0	0	0	0	0	0	0	0
LOC107776095	16.20948	14.0554	16.62685	12.48222	10.96231	9.833902	11.867	11.87127	13.63113	6.277353	7.046978	5.184239	6.882731	6.694559	7.058825	13.54609	15.2123	15.43408	6.367825	5.465252	6.90592	13.03416	15.54378	17.71664	1.69805	1.51436	2.70953	3.312019	3.121527	0
LOC107771852	0.828606	0.827094	0.754589	0.393211	0.366603	0.784799	0.877009	0.758554	0.852021	0.28857	0.677856	0.38335	0.175698	0.215231	0.303735	0.676286	0.514609	0.911933	0.520503	0.600809	0.323873	1.037986	0.904906	0.927921	0.149659	0.054902	0.373084	0	0.189962	0
LOC107762973	0	0	0	0	0	0	0	0	0	0	0	0	0	0	0	0	0	0	0	0	0	0	0	0	0	0	0	0	0	0
LOC107794013	0	0	0	0	0	0	0	0	0	0	0	0.111888	0	0	0	0	0	0	0	0	0	0	0	0	0	0	0	0	0	0
LOC107804807	49.61747	59.92765	55.31798	61.22657	53.82886	61.78534	62.15445	55.18917	52.43276	52.71734	59.5349	47.26985	48.59927	45.52014	49.54041	62.97666	54.29516	59.62993	51.99223	62.63441	44.32382	54.67096	52.76088	54.00924	27.07252	32.42438	33.77729	25.24988	27.29727	20.54699
Novel04803	0.197535	0.390063	0.143094	0.059652	0.203924	0	0.082841	0	0.061818	0.096311	0.023085	0	0.109949	0.646504	0.101372	0	0	0	0	0	0	0	0	0	0	0	0	0	0	0
LOC107799540	0	0	0	0	0.061308	0	0	0	0	0	0	0.058534	0	0	0	0	0	0.051655	0	0	0	0	0	0	0	0	0	0	0	0
LOC107806553	0.168115	0.262656	0.187892	0.73976	0.552533	0.129103	0.443159	0.656648	0.225477	0.163933	0.404167	0.182607	0.427766	0.314409	0.665544	0.48322	0.139211	0.179052	0.389923	0.690751	0.39426	0.481361	0.550785	0.251019	0.15182	0.233919	0	0	0.115623	0
LOC107825567	0.705112	0.321311	0.569158	1.528547	1.582107	1.786907	0.73926	0.5164	0.543778	0.466568	0.953534	0.957372	1.177411	0.824189	0.594474	1.032135	0.827169	0.901185	1.686534	1.555099	1.332082	0.925348	0.666381	1.316041	0.859638	0.03504	0.803622	0	0.242476	1.557077
LOC107797717	4.203191	4.961656	3.653744	6.672853	7.792734	6.455634	6.211414	5.985534	6.013167	7.221416	6.638397	6.915941	7.932105	7.096568	6.286172	4.250874	4.486036	4.849707	9.606647	8.414944	7.70327	7.488415	6.09088	6.568841	11.71635	11.58547	6.505496	7.156847	13.105	14.85083
LOC107807501	8.95754	8.711258	6.955712	9.921177	6.69379	9.278117	6.688469	5.4835	7.512353	20.82488	22.6347	18.64208	13.25501	9.414715	9.950578	6.125689	8.337137	6.067413	3.796526	5.174852	4.10962	6.454363	8.069765	10.30437	8.010404	2.013962	26.95189	1.953021	7.625724	1.688592
LOC107812013	3.542074	4.832463	3.358963	4.900926	4.330978	3.231168	6.716012	5.319205	3.970392	4.228496	5.178148	2.77481	4.875088	3.395837	5.552381	0	0	0.016005	0	0	0.019578	0	0	0	0	0	0	1.535202	0	0
Novel00868	0	0	0	0	0	0	0	0	0	0	0	0	0	0	0	0	0	0	0	0	0	0	0	0	0	0	0	0	0	0
107781140	0	0	0	0	0	0	0	0	0	0	0	0	0	0	0	0	0	0	0	0	0	0	0	0	0	0	0	0	0	0
LOC107799141	0.271164	0.61783	0.196431	0.081887	0	0	0.037906	0.038615	0	0	0.06338	0	0	0	0.092772	0.572536	0.742239	0.438022	0.091719	0.076462	0.041218	0	0	0	0	0	0	0	0	0
LOC107795980	0.18114	0.660349	0.393654	0.574364	1.469279	0.584244	0.962225	0.980218	0.793625	1.766348	0.994957	0.382581	2.35256	1.976153	0.650711	0.832414	0.437492	0.472667	1.225392	0.58739	1.046286	0.958075	0.878778	0.631093	11.29815	1.134196	11.38263	4.318033	16.42407	3.733394
LOC107789103	7.389507	6.027675	6.600987	5.58493	4.226687	5.881655	3.184555	2.965042	4.177888	2.627494	3.664284	3.518076	3.681294	3.474055	2.597967	5.050315	6.152851	5.783128	2.485644	3.833495	3.239358	4.330047	5.293651	5.291244	1.90981	1.760999	2.460906	5.839273	2.948264	0
LOC107774559	5.054335	3.57764	3.412383	7.204249	7.304934	5.578936	5.327373	4.764813	6.200437	12.17368	9.037463	9.806865	13.95386	12.13087	9.268568	0.12555	0.260422	0.125607	0.290631	0.142521	0.568522	0.19698	0.074304	0.105656	0.660322	0.117212	0.199127	2.40971	4.298895	0
LOC107831242	0	0	0	0	0	0	0	0	0	0	0	0	0	0	0	0	0	0	0	0	0	0	0	0	0	0	0	0	0	0
LOC107789041	0	0	0	0	0	0	0	0	0	0	0	0	0	0	0	0	0	0	0	0	0	0	0	0	0	0	0	0	0	0
LOC107780864	0	0	0	0	0	0	0	0	0	0	0	0	0	0	0	0	0	0	0	0	0	0	0	0	0	0	0	0	0	0
LOC107809667	17.98447	13.31738	15.4381	6.598672	6.445105	5.848989	5.053256	4.648341	5.909614	6.006466	6.967423	8.394562	5.755911	5.346496	6.829743	18.56183	19.02509	17.5312	7.391005	8.329458	8.406095	4.91882	7.77311	6.156135	4.547567	4.504333	5.579751	0	4.978559	2.780017
107793080	0	0	0	0.659075	0	0.782148	0	0	0	0	0	0	0	0	0	0	0	0.738213	0	0	0	0	0	0.919777	0	0	0	0	0	0
LOC107801356	22.12278	24.57427	23.24341	89.88354	73.8945	73.65387	66.31287	73.63722	64.95199	72.64939	78.66775	83.15358	37.6304	43.87878	59.13438	25.52591	21.61719	23.31329	118.7837	119.929	129.4007	73.80722	88.82979	79.01735	51.84755	103.3515	68.46213	30.19259	29.13358	19.14343
LOC107764545	2.817699	3.181235	2.650433	2.324093	1.469922	3.165014	2.768971	2.048187	2.349255	1.968439	2.713001	1.350118	1.661984	2.087548	2.244544	3.964458	3.636284	2.74347	2.005712	2.454731	0.939699	4.038961	2.726517	2.835168	0.558291	0.438876	0.745588	0	0.708641	0
LOC107790388	0.040762	0.0743	0.035434	0.147715	0.144277	0.043825	0.102568	0.069657	0.420966	0.119246	0	0	0.272264	0.177879	0.041837	0.030376	0.03938	0.121561	0.744533	0.655162	0.855049	0.306376	0.215731	0.340839	2.989096	0.340308	4.384193	0	0.588735	0
LOC107792435	11.61919	13.16021	9.255917	14.9265	14.51301	12.47197	15.46435	14.46068	11.97842	16.4488	17.11999	12.32551	20.71207	19.93102	12.80758	13.25244	10.37692	13.64847	16.92731	16.57662	10.39246	12.30877	12.16026	13.76493	17.09919	12.76226	12.49654	10.68729	34.80444	16.1705
LOC107796116	0.066354	0.157231	0.149968	0.060113	0.152657	0.071339	0.022262	0.034017	0.037378	0.025881	0.009305	0	0.014773	0.028956	0.013621	0.098894	0.141028	0.148409	0.040399	0.078583	0.024206	0.033248	0.023411	0.013871	0.016778	0.018465	0.015685	0	0	0
LOC107792874	1.100801	1.776578	1.535031	7.167875	9.395628	5.794195	3.385393	3.076395	3.423403	2.146839	2.669425	2.770586	2.604031	1.901456	2.071358	1.315927	1.218555	1.333627	23.13155	17.98383	34.78264	4.634841	7.585773	8.197715	8.84339	14.23189	1.572111	19.6807	10.48888	116.2763
LOC107800732	0	0	0	0.021366	0	0	0	0	0	0	0	0	0	0.024206	0	0.022784	0.017583	0	0	0	0	0	0	0	0	0	0	0	0	0
LOC107797242	1.035173	0.896261	1.079825	2.860334	3.801356	2.726697	1.823313	2.697664	1.409206	5.249008	3.665613	4.853714	4.49424	3.896164	3.18742	0.539989	0.600039	1.427766	4.884463	3.546525	4.201224	2.67995	2.145764	2.867201	12.04072	10.37065	11.68434	3.701476	9.7182	0
LOC107820371	0	0	0	0	0	0	0	0	0	0	0	0	0	0	0	0	0	0	0	0	0	0	0	0	0	0	0	0	0	0
LOC107829032	10.55849	7.867473	9.417448	4.143994	3.621496	3.882494	6.548147	5.289458	7.394223	4.39335	6.897186	5.317199	6.470368	5.778159	3.847646	9.610996	8.439413	12.33335	5.025474	5.469612	5.42641	7.151795	7.402094	5.392053	1.347959	3.110552	3.333231	0	1.324622	0
LOC107814484	0.264188	0.321034	0.459307	0.510595	0.467543	0.113614	1.034069	1.309233	1.157481	0.772851	1.333794	0.743979	0.411738	0.307431	1.89809	1.771862	0.442396	0.656546	0.518288	0.849244	0.530077	2.044511	1.258371	1.399057	0.489888	0.637171	0.582866	1.259547	0.847931	2.178023
LOC107792475	18.04881	16.49279	18.01381	22.88319	21.33662	23.20348	22.50724	19.86831	21.92094	18.57764	16.27179	22.22679	21.73996	24.17019	17.88771	19.35661	16.837	19.47227	19.67133	19.54957	23.12354	15.27242	19.54232	21.40984	8.753393	12.15818	14.16506	13.65868	22.29805	14.76171
LOC107793666	0	0	0	0	0.051413	0	0	0	0	0	0	0	0	0	0	0	0	0	0	0	0	0.048523	0.179377	0.030365	0	0	0	0	0	0
LOC107773072	86.41927	87.98712	92.34065	83.34254	93.08761	83.28889	81.09791	84.11876	77.82824	71.53625	73.66321	52.30894	74.15318	68.50847	84.33122	86.81391	104.7502	94.40141	92.30769	92.34285	87.37986	88.2226	88.26343	77.44368	95.34764	76.96568	60.00111	31.4781	103.1303	199.585
LOC107812638	3.021909	2.086438	2.706484	1.908092	1.377507	2.559757	1.920156	2.894971	2.063347	1.518019	0.963166	2.320903	1.223284	2.097935	0.93988	2.11545	2.56555	1.433703	1.672595	1.859147	2.421968	1.605975	2.827071	2.967075	0.810433	0.89192	2.164639	0	1.322595	0
LOC107761868	7.54733	8.186544	8.200917	9.233545	9.21844	7.420773	13.93201	12.15321	12.067	9.8128	11.20633	8.801328	11.05877	10.34499	13.5065	9.085374	8.257304	9.173697	10.16225	10.91371	8.883565	14.02007	11.6644	12.15296	15.82198	14.63038	9.930546	8.073046	20.72989	60.82571
LOC107775545	0.047835	0.087192	0.332658	0.043336	0.253967	0.051429	0	0.081744	0.08982	0.046646	0.30188	0.080825	0.053251	0.156558	0.245485	0.035647	0	0	0.04854	0	0	0.119846	0	0	0.060478	0.133119	0.056538	0	0	0
Novel05386	0.843061	1.035597	0.924035	0.863393	0.518954	0.827577	0.522648	0.814291	0.791503	0.428917	0.591155	0.588373	0.734484	0.799773	0.601944	0.710196	0.708233	0.792504	0.557922	0.186045	0.468018	0.918342	0.872964	1.149349	0	0.153008	0.043323	0	0	0
LOC107781346	0.167347	0.27453	0.290943	0.212252	0.029616	0.323854	1.207109	0.82932	0.47134	0	0	0	0	0	0	0.149649	0.064669	0.224576	0.033963	0.19819	0	2.878979	0.649491	0.209894	0	0	0	0	0	0
LOC107795731	7.007623	10.79252	14.03376	5.906563	8.202331	5.67438	7.811993	7.38964	9.119084	8.995759	8.054817	4.908549	7.652863	8.806233	9.468518	17.02136	9.255125	11.11029	11.97143	12.3812	9.465255	8.667254	8.802323	5.423739	28.93442	27.03013	16.51247	6.343596	28.61255	27.42354
LOC107815412	1.612867	1.679919	1.602321	5.148904	4.553353	4.624077	4.380433	5.31544	8.003788	6.965029	5.331589	8.435109	29.41144	28.65566	18.36708	1.602547	1.854958	2.290401	3.351186	1.884136	4.062655	4.233262	3.251783	5.538951	0.291308	1.06866	1.089302	5.492513	3.32782	9.497711
LOC107773267	0.667612	1.470412	0.967236	1.587661	0.71382	0.837393	0.886596	0.546658	0.39174	0.298378	0.604668	0.564018	0.805128	0.75868	0.456813	2.176587	0.833086	1.057684	1.552483	1.670731	0.608874	0.325232	0.711561	0.290745	0.386863	0.116117	0.197266	0	0.535687	0
LOC107819612	13.46245	8.038548	6.860158	7.429928	4.107745	6.987342	5.51598	4.296979	6.755403	5.809335	7.975069	8.758865	10.42171	7.005806	9.529303	8.648477	7.624124	8.364023	2.276794	3.141602	4.657165	3.683	5.86914	7.197209	1.369476	1.614829	1.280238	0	11.17466	4.783928
107809470	0	0	0	0	0	0	0	0	0	0	0	0	0	0	0	0	0	0	0	0	0	0	0	0	0	0	0	0	0	0
LOC107820895	0.65203	0.990409	1.448479	0.32817	0.19232	0.623122	0.24306	0.185704	0.068017	0.211937	1.016009	0.367236	0.08065	0.711333	0.817943	0.701846	0.489935	0.432103	0.29406	0.122572	0.066074	0.181509	0	0.30289	0.091596	0	0.085627	0	0	0
LOC107831636	23.75361	18.01906	17.87909	31.32434	33.12424	36.26704	23.53843	22.77766	23.97241	32.02584	25.55898	34.91069	37.55198	33.37645	23.22597	18.99303	22.85746	22.0406	37.08235	33.80693	32.64509	22.22399	24.0517	29.52829	30.7426	28.94444	41.8631	16.75265	42.63063	23.1751
LOC107779562	0	0	0	0	0	0	0	0	0	0	0	0	0	0	0	0	0	0	0	0	0	0	0	0	0	0	0	0	0	0
LOC107809754	0.108898	0.049623	0.141994	0	0	0	0	0	0	0	0	0	0	0	0	0	0	0.040594	0	0	0	0	0	0	0	0	0	0	0	0
LOC107773628	6.784126	5.895318	6.788748	8.36152	8.864956	9.498856	10.65247	8.088197	10.2204	4.999996	5.47612	5.331552	6.05931	4.905412	4.938901	4.644041	5.4871	6.292911	6.884062	6.272755	8.561409	9.025434	11.06353	12.94477	1.795227	2.853837	3.170022	0	5.69672	0
LOC107773280	3.249839	4.094291	2.160307	3.290565	4.14435	2.671863	3.84816	5.635171	3.499782	5.778771	4.892695	3.149312	6.065079	4.588184	7.848343	2.493013	3.139618	1.781539	6.983391	4.204568	4.271515	5.986821	4.890036	2.5975	10.51366	8.777833	5.196713	0	12.42467	11.82014
LOC107771620	6.833386	6.253846	5.592161	5.180503	5.262339	6.02493	4.916102	4.690448	5.368606	4.96271	5.633608	5.821344	6.270222	6.113632	4.519223	6.200184	6.214919	6.608032	4.700066	5.127539	4.067855	4.966534	5.699643	5.648058	3.904032	4.853537	3.852395	10.22351	7.295451	3.53572
Novel00525	2.659353	2.337113	2.641965	4.517324	4.328153	4.186583	3.26611	1.866469	3.031729	4.584416	3.529687	4.814348	4.387779	4.869911	2.339561	2.05253	2.385648	2.372112	3.903228	3.173585	3.031719	2.657143	2.764605	3.524079	4.382908	3.105606	4.32182	10.18829	7.316042	0
LOC107812581	2.736256	1.781257	2.152039	2.714996	3.112997	3.221982	2.295016	3.117242	3.302894	2.922309	3.015044	3.412458	3.626236	2.842972	2.942153	2.330356	3.02109	2.23428	3.569868	2.755577	4.159186	3.373271	1.838902	5.311306	5.271558	7.977202	4.312046	9.318143	11.29141	16.11303
LOC107813709	391.6723	354.8177	438.7238	172.7046	153.0465	243.264	151.0464	169.4589	218.7849	76.44159	97.49352	95.36731	57.19922	91.04126	67.5123	212.0021	375.9787	276.4941	89.88727	76.74315	127.3899	124.6249	235.1609	265.2451	25.67867	16.06392	54.41224	15.28936	16.81165	4.406421
LOC107828919	0	0.128327	0.489598	0.892941	0.124594	0.756917	0.708599	0.601542	0.132195	0.41191	0.394932	0.118957	0.940482	0.614449	0.867115	0.314786	0.408091	0.62986	0.57152	0.357336	0.256835	2.939765	1.3662	0	0.534064	0.391842	0	0	0	0
LOC107759697	0	0	0	0	0	0	0	0	0	0	0	0	0	0	0	0	0	0	0	0	0	0	0	0	0	0	0	0	0	0
LOC107830671	1.587266	2.893194	1.623266	2.199267	1.982847	1.003826	2.975865	2.792182	1.840829	1.456738	3.600855	0.788804	1.974844	0.916745	2.587435	2.643978	0.631413	1.949086	2.084367	2.922385	0.340615	3.742777	2.38836	1.854187	0.94437	1.558986	0.441416	0	0	11.54624
LOC107795788	0	0	0	0	0	0	0	0	0	0	0	0	0	0	0	0	0	0	0	0	0	0	0	0	0	0	0	0	0	0
LOC107792843	0	0	0	0.170084	0.249188	0.100922	0	0.320822	0	0.183071	0.131644	0.079304	0	0	0.289038	0	0.181374	0	0.476267	0.39704	0.342446	0.156787	0	0	0.237362	0	0	0	0.903852	0
LOC107796922	11.42365	7.049772	7.525684	3.295053	2.357116	2.340707	2.169804	2.801272	3.510884	0.724318	0.880052	0.757367	0.684321	1.145669	1.41961	10.70796	5.988258	6.607219	1.637426	1.581715	1.565121	2.010713	2.327065	2.355875	0.29145	0.356394	0.363278	3.663466	0.616564	3.167452
LOC107759009	0.107336	0.130431	0.248812	1.55585	2.247803	2.231045	0.60018	0.947674	1.041304	2.930641	0.827902	2.055415	2.588895	3.317772	0.514109	0.23996	0.414781	0.293419	4.828642	3.753005	3.002017	0.74699	1.009889	0.747914	0.633289	0.448049	1.310908	0	1.550255	0
LOC107800493	1.19415	0.568895	0.943681	1.032665	1.200753	0.554393	1.206455	1.043503	1.605241	1.137982	1.027643	1.352778	1.90338	3.789844	0.919232	0.991005	1.022553	1.112862	1.26682	1.308628	0.841566	2.651821	3.542983	3.262146	0.205878	0.151052	0.930234	0	0.261321	0
LOC107787444	24.6209	24.89546	28.43166	4.628712	3.488639	7.612426	1.484684	2.303045	3.248213	2.157626	1.523311	2.99091	3.806713	4.652256	1.03228	0	0.038866	0.029993	0	0	0	0	0	0	0	0	0	2.87703	0	0
LOC107777617	0	0	0	0.095002	0.092791	0.225484	0	0.044799	0	0	0	0	0	0	0	0	0	0	0	0	0	0	0	0	0	0	0	0	0	0
LOC107782309	0	0	0	0	0.096965	0	0	0	0	0	0	0	0	0	0	0	0	0	0	0	0	0	0	0	0	0	0	0	0	0
LOC107782857	14.59889	14.52735	17.08722	13.53828	13.7318	13.75939	13.59453	12.57191	14.9292	8.33222	8.141041	7.121736	8.147174	10.74319	7.07901	14.41982	13.99271	15.59768	11.62565	11.7338	13.03487	16.79989	15.91905	18.66295	5.474254	7.570835	7.38194	5.480459	8.301292	9.476868
LOC107762542	0	0.149648	0	0	0	0	0.206582	0.140297	0	0.080058	0.115137	0	0.091395	0	0	0.061181	0.079315	0.183627	0.083309	0	0	0	0.072418	0	0	0	0	0	0	0
LOC107762599	0	0	0	0	0	0	0	0	0	0	0	0	0	0	0	0	0	0	0	0	0	0	0	0	0	0	0	0	0	0
LOC107805175	264.6351	234.8885	282.5401	134.8645	120.081	197.7317	94.80602	104.8729	142.3303	84.93755	93.87751	111.9506	70.92918	89.41148	63.59062	191.6718	263.9048	235.7652	108.0216	113.0889	127.2117	118.2277	168.6618	204.8258	55.51609	27.82956	96.71304	69.88607	24.46472	16.11303
LOC107811496	1.198673	0.453467	0.825721	0.799086	0.820514	0.48631	0.588052	0.463779	0.3822	0.749833	0.808793	0.305712	0.906371	0.962265	0.858879	2.123582	1.070622	1.63557	1.170432	1.415767	0.660051	1.378796	1.03736	1.631073	2.773628	1.856681	1.069234	0	4.464235	1.398411

LOC107830439	1.808426	1.844366	2.339325	1.950407	1.504963	2.198887	1.282061	1.103691	1.637193	1.154636	1.313357	0.800281	1.270211	1.315269	0.950156	1.877071	1.663897	2.407604	1.223377	1.329472	0.687214	1.366429	1.936969	1.800158	0.462724	0.239647	1.068707	0	0.207295	1.331163
LOC107798814	0.138205	0.041986	0.120139	0	0.081529	0	0.077279	0	0	0	0	0	0	0.050258	0.047283	0	0	0.034346	0.186988	0	0	0	0.040635	0.096301	0	0	0	0	0	0
LOC107817585	0.23871	0.362591	0.217387	0.154471	0.25146	0.281084	0.038136	0.087412	0.10672	0.166266	0.231149	0.230479	0.139195	0.12401	0.105002	0.160945	0.186687	0.279665	0.12688	0.134621	0.134771	0.085437	0.140372	0.166333	0.028743	0.031633	0.013435	0	0	0
LOC107811768	5.234487	2.957765	6.066971	2.244632	1.482182	3.038965	1.258578	1.639912	4.095305	1.293086	1.174536	1.149782	1.748131	2.246157	0.57307	5.539059	4.483833	7.128625	1.735121	1.594086	2.005055	1.748583	2.000771	3.173229	1.323596	0.825453	2.227227	0	2.016055	0
LOC107781388	1.176106	1.228736	1.446271	1.97506	1.294525	2.652278	1.227049	1.642149	1.858255	0.671328	0.603429	0.581624	0.51093	0.970129	0.853819	1.111577	1.025367	1.43288	1.862917	1.383161	1.334246	3.761096	1.9989	1.948836	1.523223	2.275087	0.847599	0	4.281172	5.321007
Novel02504	3.991991	3.41771	4.258824	5.260424	2.622522	4.812106	2.638019	3.307506	4.258928	1.828387	2.756775	3.321446	1.885308	3.299292	2.110715	3.01991	4.440932	4.238829	2.946034	4.042107	5.075003	2.374081	4.961691	6.448298	1.300008	3.282243	1.85869	0	2.620754	0
LOC107759002	0	0	0.030094	0	0	0	0	0	0	0	0	0	0.077078	0	0	0.077396	0.100337	0.077432	0.03513	0	0.031574	0	0	0	0	0	0	0	0	0
LOC107827816	26.26818	29.29196	21.40509	11.10084	10.28824	11.86901	10.17883	10.97125	12.23898	10.76508	11.8037	14.61833	12.55101	13.11146	11.41119	31.88608	30.67208	30.61648	7.34903	7.120015	9.211492	8.042565	10.91178	11.98876	4.553515	3.486164	7.032972	11.19849	10.5544	4.841135
LOC107819515	404.1985	341.2551	431.8974	210.7426	192.2102	320.5537	193.8307	201.636	277.9635	120.3005	140.4466	163.6574	112.4229	153.5793	105.0524	252.9105	469.5331	336.2721	120.14	119.765	176.0878	175.7117	267.0259	309.7038	34.88673	21.98756	87.03239	51.62794	22.78455	9.919509
LOC107829112	1.332671	1.865854	1.242409	1.207332	0.734886	0.955189	1.06917	1.039655	1.106107	0.659178	1.0699	0.55478	0.731024	0.632121	1.645345	1.381715	1.306131	1.511943	0.68595	0.80058	0.33468	1.51619	1.379939	0.868044	0.512796	0.295615	0.388071	2.762458	0.743879	0
LOC107785759	0.598436	0.583276	0.534658	0.647571	0.53689	0.652328	0.850499	0.68176	0.577445	0.761858	0.932497	0.252788	1.008533	0.81608	0.904266	0.644157	0.433605	0.694026	0.362664	0.513267	0.159187	0.645536	0.689151	0.443052	0.493895	0.705465	0.176826	0	0.520196	0.513919
LOC107792418	0	0	0	0	0	0	0	0	0	0	0	0	0	0	0	0	0	0	0	0	0	0	0	0	0	0	0	0	0	0
LOC107790299	1.611685	1.239344	2.276631	1.825131	1.916355	1.732758	1.73198	1.979534	2.553391	1.276922	1.024166	1.999844	1.682016	2.14289	0.982175	2.514656	1.11911	2.140314	0.971035	0.724293	1.378016	1.850695	2.843225	2.632082	0.254707	0.350397	0.654803	0	2.909709	0
LOC107796549	0	0	0	0	0	0	0	0	0	0	0	0	0	0	0	0	0	0	0	0	0	0	0	0	0	0	0	0	0	0
LOC107809294	6.615077	6.863004	3.86973	31.32151	56.54659	26.03267	25.22929	23.67484	21.60013	18.45908	12.86526	21.65144	13.57013	21.19629	10.37646	7.378843	5.426084	7.227134	103.1364	64.22967	112.5413	23.52223	16.55067	22.35117	51.91662	24.19772	121.9979	11.90117	26.83989	7.717362
Novel01336	0.340128	0.671634	0.591333	4.930224	4.263712	2.437864	1.901868	1.114024	0.85154	0.884448	1.152741	0.622591	1.766966	1.546097	2.327323	1.013856	0.547654	0.295843	2.013309	1.918168	1.964617	1.325567	0.750039	0.770254	0	0.157755	0.335004	0	0.818749	0
LOC107817588	6.485113	7.076644	7.032574	4.480074	3.953584	3.497804	5.421152	4.985098	5.009276	2.136135	2.874422	1.722433	1.810866	1.869299	2.693623	5.608534	5.804183	5.772806	2.200885	2.311809	2.551755	6.575141	5.528984	5.826111	0.521017	0.663941	0.512701	0	0.522101	0
LOC107759750	8.384617	8.036797	12.31517	1.484283	2.899481	4.559052	1.091256	1.276353	2.804911	1.174721	2.872071	2.971837	0.804644	1.209113	1.186999	20.39651	7.774373	21.55547	4.15627	5.869911	2.724762	1.971887	2.337749	8.461406	0.426465	0.469345	1.08212	0	2.319916	0
LOC107820272	0	0	0	0	0	0	0	0	0	0	0	0	0	0	0	0	0	0	0	0	0	0	0	0	0	0	0	0	0	0
LOC107784268	4.453162	4.58089	4.5226	4.65347	4.057493	4.9062	3.272799	3.484688	3.787578	3.095569	2.844312	2.253568	2.208708	1.851881	2.307928	4.452049	4.366021	4.930724	5.100383	4.941946	3.619032	3.277096	4.803029	5.829575	2.787203	1.134957	3.725984	3.153109	2.441086	1.363097
LOC107792336	1.788064	1.437882	0.868594	2.048691	1.628732	1.978928	2.999453	2.741	2.221827	2.205126	2.286326	2.354769	2.224674	2.008063	0.809656	2.939267	2.74355	4.234482	5.549942	3.870423	8.825206	7.422376	4.174917	6.486127	6.64899	1.756208	10.56674	11.28281	13.67213	3.251726
LOC107791651	1.605983	3.220043	1.761167	0.380523	0.371667	0.637524	0.020723	0.021111	0.046393	0.096371	0.069299	0.020874	0	0.080864	0.025359	2.540857	1.933426	2.652544	0.275785	0.313512	0.022534	0	0.152555	0.154945	0	0	0	0	0	0
LOC107766451	1.214603	1.268393	1.60574	2.246587	2.731684	1.95877	2.419507	2.832302	2.874564	3.4298	3.193802	2.522571	3.46479	4.113245	3.999622	3.149076	1.809005	1.546961	2.542022	2.461649	1.776997	2.028694	2.008801	2.433214	2.079496	1.690032	1.465471	1.80961	3.41106	0
LOC107812883	0	0	0	0	0	0	0	0	0	0	0	0	0	0	0	0	0	0	0	0	0	0	0	0	0	0	0	0	0	0
LOC107770928	0	0	0	0	0	0	0	0	0	0	0	0	0	0	0	0	0	0	0	0	0	0	0	0	0	0	0	0	0	0
LOC107776237	0.707516	0.644814	0.758535	0.897363	0.667794	0.786022	0.870355	0.725425	0.819238	0.459945	0.826851	0.219167	0.81387	0.771865	0.726175	0.544816	0.5696	0.650563	0.3829	0.478808	0.322633	0.728734	0.790496	0.961344	0.059634	0.328152	0.055748	0	0.908329	0
LOC107826605	5.525426	5.093961	6.88542	4.282381	4.097935	4.979039	2.839582	2.565208	3.748203	2.771855	1.724458	2.725268	3.057713	2.961719	0.852311	4.522169	5.02947	5.071911	3.402984	3.97166	3.029395	2.240531	4.113142	4.640169	2.140164	1.111015	2.491446	0	1.691416	0
LOC107813102	0.063722	0	0	0.115458	0	0	0.053447	0	0	0	0	0	0	0	0	0	0	0	0.064661	0	0	0	0.056207	0	0	0	0	0	0	0
LOC107796732	8.328802	6.197892	7.406107	6.749391	7.60652	8.831254	5.191227	6.13704	6.169472	5.51377	4.688203	5.809875	3.657658	6.08533	4.470293	5.124743	6.828302	5.924637	6.319236	5.461951	6.690062	5.041215	6.571459	6.389183	2.801596	2.551689	4.154338	5.464469	4.782309	2.362305
LOC107818736	0	0	0	0	0	0	0	0	0	0	0.045442	0	0	0	0	0	0	0	0	0	0	0	0	0	0	0	0	0	0	0
LOC107786687	4.330865	5.105636	5.263128	4.626268	4.594868	5.559653	4.933671	4.087018	4.895391	4.580321	5.121776	4.732821	5.420828	5.147873	3.958555	5.507395	5.016612	5.525981	3.629423	3.827656	2.652865	4.354577	4.656352	5.06711	2.506213	2.068655	2.062773	0	2.385858	0
Novel03013	0	0	0	0	0	0	0	0	0	0	0	0	0	0	0	2.291806	4.055806	2.656801	4.061894	3.881742	5.476105	3.220646	5.683801	5.408402	2.098429	1.9698	2.192478	0	1.645129	9.055134
LOC107809586	0	0	0	0	0	0	0	0	0	0	0	0	0	0	0	0	0	0	0	0	0	0	0	0	0	0	0	0	0	0
LOC107774476	7.277765	8.331146	8.03388	4.882226	4.545773	4.846309	4.794629	4.002102	4.728503	2.922187	3.77882	3.382716	3.532233	3.461592	4.08378	7.262475	7.371532	7.810267	2.913104	2.684144	1.929223	5.236626	5.042283	5.185202	0.636769	0.455516	1.309606	0	0.727427	0
Novel03564	76.04992	64.43893	70.22377	54.78586	47.43009	66.41188	43.54891	48.93005	56.48837	37.07914	34.0517	47.1547	31.61994	44.14896	24.13766	40.45596	69.78233	52.08742	41.37438	40.95092	62.39507	38.70617	60.07661	57.70016	17.47308	22.84222	36.27884	54.60486	21.6885	28.32699
LOC107783874	1.798868	1.609638	1.435777	1.407454	1.143171	1.459293	1.056152	0.894255	1.04402	0.781382	0.928825	0.690788	0.80101	0.981238	0.68817	1.535503	1.200702	1.755664	0.879495	0.954532	0.775555	0.819426	1.543446	1.299031	0.248107	0.113772	0.405895	0	0.472385	1.011152
LOC107772802	4.183763	4.308456	4.335461	8.694082	7.027655	10.24134	12.07369	10.76455	13.29273	4.540679	7.110389	5.012284	9.525351	7.632919	6.52606	5.249084	5.617509	6.097366	6.571965	7.798183	7.286405	14.96287	16.05409	14.67505	2.42064	1.578685	5.447739	6.761541	2.162149	7.30758
LOC107765788	0	0	0	0	0	0	0	0	0	0	0	0	0	0	0	0	0	0	0	0	0	0	0	0	0	0	0	0	0	0
LOC107823087	6.195456	6.416357	7.017572	5.995458	7.600249	10.04177	5.826262	6.215929	7.006319	5.904048	5.397411	6.106442	7.785101	6.451713	3.709327	4.302075	6.212048	5.668742	5.619947	5.558561	5.864389	5.369971	7.866002	7.309453	4.509874	3.395964	5.103582	3.356535	7.908708	5.804157
LOC107822435	13.40869	14.11663	14.64164	11.22007	10.08224	11.78844	12.71458	11.51319	11.90652	8.30885	8.33691	9.486531	9.228109	9.584008	9.287866	13.3393	14.03873	14.55192	10.85816	12.5721	10.51208	12.8804	12.99261	13.9112	7.181916	9.420515	11.98358	0	7.314024	6.126203
LOC107800545	0	0	0	0	0	0	0	0	0	0	0	0	0	0	0	0	0	0	0	0	0	0	0	0	0	0	0	0	0	0
LOC107773986	15.02767	18.65681	10.40704	3.327941	3.028867	2.674811	3.095068	2.896143	1.865475	4.835777	9.13242	3.625353	4.442481	4.371852	11.6194	25.99327	16.53438	15.58568	4.405277	3.983209	1.979681	3.318777	2.179776	1.780117	6.417621	7.388164	3.276008	0	7.556402	6.194554
LOC107790330	125.8025	136.2886	138.211	47.61201	40.0907	58.49797	47.28101	57.1101	65.31655	34.791	41.73718	46.61829	22.29495	30.94013	32.5945	98.23595	107.7687	103.5539	31.22998	38.94868	45.47207	64.82607	78.75411	70.95925	2.434704	6.625692	15.3944	5.007879	2.697057	4.329839
LOC107777743	185.1768	161.6025	202.4748	90.76195	86.93693	127.1336	86.83318	86.6527	118.7283	54.79468	70.00256	59.96055	49.23365	62.327	49.54695	130.6943	205.4163	175.0095	52.6752	53.24108	58.89823	92.09202	135.5547	155.3524	18.46558	11.5894	37.71368	16.77892	12.70758	36.26786
LOC107831576	204.1593	196.3907	239.5227	126.7447	109.9982	146.8765	110.7524	120.3693	171.8888	51.13627	67.2221	66.17199	44.07142	59.27002	48.00996	145.0592	235.6582	179.7724	62.36015	61.46679	100.4307	118.8201	193.3517	217.0795	14.09184	5.423823	34.19374	8.71138	6.450984	11.29786
LOC107765169	4.169332	4.869687	5.137374	3.007094	2.113288	3.786224	2.851921	2.178938	2.546229	1.6973	2.611316	1.436305	2.568519	2.031385	2.285057	4.524744	4.067031	5.130392	1.971612	1.609393	0.849102	4.394654	3.14205	3.511613	0.921199	0.281618	0.6698	0	0.9744	0
LOC107761492	7.446761	5.981591	9.106523	9.261991	6.924438	6.106408	6.986961	6.470558	7.820829	5.784611	6.0182	7.464172	6.60377	7.022484	6.995427	4.420657	8.047746	6.304673	4.866872	5.765596	5.870681	3.583822	8.349834	8.442959	2.553212	2.985553	3.878615	9.02625	4.861201	0
LOC107818587	0	0	0	0	0	0	0	0	0	0	0	0	0	0	0	0	0	0	0	0	0	0.057256	0	0	0	0	0	0	0	0
LOC107825966	0	0.029413	0	0	0	0	0	0	0	0	0.06789	0	0	0	0	0	0	0.024061	0	0	0	0	0	0	0.040803	0.044906	0	0	0	0
LOC107787547	2.689772	4.515728	3.273426	0.256505	0.100214	0.243523	0.118738	0.314492	0.053164	0.055218	0.158827	0.04784	0.787971	0.308885	0.610262	3.03828	2.790017	2.53306	0.143652	0.263463	0.129111	0.212807	0.099897	0.207152	0.143187	0.078792	0.066928	0	0	0
LOC107826288	6.950391	7.558468	7.355269	6.053352	4.664617	7.400322	6.758948	5.852536	5.83182	6.319127	8.970284	3.290524	6.316904	4.285781	8.581107	7.656563	4.83326	7.05928	6.677992	5.680727	4.807745	7.991639	6.693427	5.86077	5.263954	2.195822	8.056063	0	6.950973	8.304409
LOC107776769	6.588443	6.990376	6.382544	12.44241	11.8338	8.739695	13.30641	12.82706	11.26319	11.18705	10.71063	10.4399	15.98231	14.55413	13.75989	7.669904	7.061656	8.553171	14.33557	13.47591	14.55856	12.40044	12.66374	12.77894	12.63974	14.41233	12.47471	30.47348	14.04478	20.26733
LOC107792624	2.378355	3.723132	1.240472	2.788395	3.391998	2.617187	2.323388	2.103853	2.548138	1.964495	1.393023	1.441968	0.872156	1.678903	0.516934	6.90175	7.271515	3.525468	5.053922	4.284215	6.124516	4.159391	3.356586	3.538688	0.318384	0.233598	0.429921	4.002023	0.269418	0
LOC107815586	2.413814	2.529878	2.832674	3.28019	3.310647	3.114174	26.82555	28.97711	37.50552	3.648349	1.692568	5.098143	3.896282	5.003369	27.99544	1.349083	2.448545	2.519441	2.57184	2.960785	4.292806	24.89561	30.02092	4.28896	2.136256	2.518984	3.423521	0	4.648384	14.92497
LOC107825954	12.51452	16.97914	14.39815	86.69866	112.8662	89.34243	52.76729	46.90775	47.34228	91.22055	62.8756	95.48838	114.508	90.56511	59.31163	13.75666	22.29278	17.41478	82.45644	73.26664	76.89876	31.28266	27.99617	35.27502	58.67083	48.89045	36.74363	46.57057	87.92041	24.50923
LOC107832261	2.435672	4.720027	3.387644	2.392414	3.266894	1.488493	3.612712	3.7898	3.682816	1.525056	1.474183	1.234634	1.54123	1.985906	2.105185	9.209069	3.839209	6.728375	6.660181	4.944986	5.962634	5.053115	4.229002	1.741838	6.54784	5.850587	1.363627	5.500578	8.887218	12.68221
LOC107760178	1.618077	1.813454	1.83423	1.594656	1.470477	1.528054	2.732641	2.709009	1.662822	1.16205	1.970211	0.997538	0.900633	0.93033	2.850205	2.33826	1.57376	1.776913	1.641912	2.00693	1.146669	3.067812	2.439827	1.62265	0.829354	1.338691	0.659015	0	0.21054	0
Novel03688	0.911313	0.792797	0.828193	3.715214	4.19689	1.18018	5.489474	5.82218	4.802917	2.12063	1.960608	2.51968	2.651497	2.959996	2.59346	1.790384	1.180543	1.791204	5.401305	5.308751	5.289024	4.722037	7.855689	7.338608	6.598827	7.896337	3.892241	1.481176	7.179354	3.841897
LOC107764918	2.57222	2.679156	2.584441	0.575009	0.354712	2.154897	0.056037	0.142713	0.188175	0.390895	0.445055	0.169331	0.185937	0.255106	0.102859	1.518521	2.355891	2.465614	0.508463	0.367363	0.213264	0.111591	0.412522	0.069831	0.042235	0	0.039483	0	0	0
LOC107816374	0.474383	0.40532	0.266323	0.20593	0.297334	0.201883	0.298414	0.287106	0.185571	0.375849	0.533606	0.250482	0.275047	0.539092	0.58833	0.38297	0.181408	0.346304	0.631799	0.51834	0.414618	0.222845	0.287675	0.216923	0.662239	1.113866	0.712534	3.533862	2.664489	5.499712
LOC107812199	11.13855	10.15142	10.47697	11.85042	12.48442	17.25672	10.97131	10.5908	11.6908	10.4159	11.73557	12.93294	11.25502	12.77477	7.680165	9.024093	11.25743	9.922531	8.288599	8.987567	9.116646	9.731425	10.2784	13.97042	5.633007	2.940738	9.181656	8.169888	4.674997	3.531864
LOC107785000	4.686172	5.029011	4.290527	5.275392	4.514678	6.051812	4.581701	3.76707	3.748759	6.570504	6.727442	4.052713	5.926679	5.687149	5.17974	5.558497	4.152199	5.250947	6.330893	6.380218	5.234856	5.441912	3.962303	4.434271	9.64086	6.751963	7.76726	1.983003	10.14573	18.85967
LOC107807951	3.918965	4.081889	3.679207	6.532705	6.895884	3.972602	11.66417	10.46636	6.517611	7.402763	11.35308	7.529819	7.927551	7.597993	9.101931	6.308101	4.608163	5.209071	8.612384	9.623478	10.17104	9.668874	8.888878	5.874979	19.33773	15.26827	12.83698	3.202987	11.21256	16.61592
LOC107768445	6.215241	5.44083	6.220303	5.556605	6.078565	8.396642	4.87003	5.45022	4.64508	6.020772	6.852587	5.83807	5.780917	5.48687	4.868301	4.844902	5.016872	5.700702	4.937558	4.392919	4.512348	4.814881	5.734726	5.299506	3.360309	1.877538	3.914599	5.84839	2.756009	2.528274
LOC107832532	6.724993	6.509686	7.552459	6.735811	5.765912	8.218166	5.115025	5.389121	6.549014	5.498707	5.594247	4.658092	3.812927	5.468301	3.601225	6.287686	6.698676	7.162526	5.933964	5.476874	5.33331	4.360417	6.779301	7.29091	4.119193	4.533368	7.306605	0	3.619734	0
LOC107769602	3.654402	3.66359	4.023813	4.414271	4.419329	4.321822	5.517188	5.932598	4.460188	3.563503	3.33121	3.190252	1.762857	4.119665	4.750985	5.62808	4.001188	5.267389	4.820705	3.709647	4.666027	5.798584	6.876638	6.365966	2.464147	3.898372	3.16742	0	3.518718	7.531906
LOC107764765	3.149513	3.121204	1.488515	4.653925	4.789132	1.577994	6.770785	6.009094	6.574082	5.45655	8.147644	6.742405	4.050716	2.134968	2.855963	2.324234	2.983613	2.439284	3.599275	3.052272	6.692991	5.388162	5.151583	4.058908	0.811852	0.850935	0.72281	2.186745	2.797035	0
LOC107773916	1.63559	2.869479	1.31515	0.889057	0.832185	0.30773	7.305046	7.092273	1.497175	0.917071	5.074929	0.414537	1.274533	0.802957	3.693194	12.95021	2.844211	3.353354	3.070414	4.635048	3.430876	18.78145	11.14484	2.179635	2.429762	3.072335	2.029792	2.924195	2.362293	35.39584
800482	2.870124	0	0	0	1.269837	0	0	1.226156	0	0	2.012533	0.60619	0	0	0	0	0	0	0	0	0	0	0	0	0	0	0	51.31361	0	0
LOC107830342	0	0	0	0	0	0	0	0	0	0	0	0	0	0	0	0	0	0	0	0	0	0	0	0	0	0	0	0	0	0
LOC107829113	0	0	0	0	0	0	0	0	0.07307	0	0	0	0	0	0	0	0	0	0	0	0	0	0	0	0	0	0	0	0	0
LOC107816165	9.945885	13.01229	8.24938	5.576679	4.28691	7.475938	6.334215	5.478675	4.441397	3.390304	4.895831	3.515111	4.441449	3.171198	3.217479	11.12815	10.18673	10.26213	4.048526	5.761704	3.742686	5.116919	5.806723	4.40838	0.720609	1.625782	1.482035	0	1.783609	0
LOC107821368	0	0	0	0	0	0	0	0	0	0	0	0	0	0	0	0	0	0	0	0	0	0	0	0	0	0	0	0	0	0
LOC107827108	4.392901	4.071445	4.401163	5.362539	3.458885	6.604063	3.653291	4.166926	5.032982	2.795252	2.401594	2.799159	3.06676	3.249111	2.636474	2.85746	4.927254	4.052235	3.210992	3.243707	3.531064	2.735925	4.236042	4.66928	1.835632	1.295	3.476037	0	1.971516	2.301862
LOC107782410	46.56517	51.53551	45.7011	86.82964	81.2502	79.34062	68.35854	67.77375	70.87592	80.41892	68.59879	73.93052	67.26403	80.77303	65.10023	44.34143	52.75658	48.62451	80.43999	72.87937	90.32783	63.41017	75.65058	71.35889	52.44005	49.68963	71.75907	45.04775	55.52045	53.92872
LOC107816883	4.300894	1.972847	3.193968	1.032157	0.680492	1.102407	0.668912	0.900448	1.25682	0.166646	0.359498	0.168441	0.285366	0.559319	0.292339	0.955142	1.320803	1.189165	0.37573	0.144567	0.337698	0.475734	0.904452	1.577818	0	0.158527	0	0	0	0
LOC107816464	0	0	0	0	0	0	0	0	0	0	0	0	0	0	0	0	0	0	0	0	0	0	0	0	0	0	0	0	0	0
LOC107830109	0	0	0	0	0	0	0	0	0.027412	0	0	0	0	0	0	0	0	0	0	0	0	0	0	0	0	0	0	0	0	0
LOC107781518	0.099455	0.060427	0.028818	0	0.088005	0.035642	1.974212	2.124431	0.778108	0.096981	0.929841	0	1.03334	1.048841	0.918703	0.049409	0.096082	0.024716	0.06728	0.028044	0	2.131814	0.321662	0.0693	0.083828	0.046128	0	0	0	0
LOC107786552	104.0037	82.5249	88.79846	127.0218	143.4614	109.0538	128.5029	142.7565	158.8122	197.711	117.5266	142.2063	154.2309	150.2394	139.0963	96.20368	82.51324	82.30083	202.4814	164.1632	248.0933	144.613	139.5406	143.9388	464.6665	224.4848	510.8135	445.9397	390.9983	329.4801
LOC107819655	0	0	0	0	0	0	0	0	0	0	0	0	0	0	0	0	0	0	0	0	0	0	0	0	0	0	0	0	0	0
LOC107815947	1.870706	1.554484	2.104449	2.043687	4.040937	2.1887	2.630452	2.068494	2.996043	1.073044	0.810192	1.20856	0.979998	1.200499	1.016492	2.173081	1.275712	1.722853	17.08437	12.84608	29.70648	5.69771	6.309153	6.268337	1.252133	0.995246	1.105512	7.869524	1.059558	0
LOC107800893	0.090937	0	0.07905	0.082384	0	0	0	0	0	0	0	0	0	0	0	0.135533	0	0	0	0	0	0	0	0	0	0	0	0	0	0
LOC107792979	0	0.14666	0	0.145786	0.355984	0	0.134971	0.206243	0.07554	0.078459	0.056419	0	0.179139	0.087778	0.165165	0.179878	0	0.17996	0	0	0	0.134389	0.070971	0	0	0	0	0	0	0
LOC107820403	0.022646	0.020639	0	0	0	0.024347	0.018994	0	0.021261	0	0	0	0	0	0	0	0	0	0	0	0	0	0	0	0	0	0	0	0	1.400225
LOC107827762	7.38558	7.134288	7.396461	6.290121	6.053386	5.378982	10.04839	9.858246	10.0973	4.314486	3.794585	4.198034	3.561487	3.898696	3.667915	7.786402	7.03645	7.231729	5.905707	4.952088	6.953057	9.663977	11.31794	14.30047	6.88488	6.91414	9.493466	2.433979	10.4868	16.83545
LOC107825341	4.430442	4.553265	4.479513	16.62435	28.63799	15.64108	13.96795	15.75945	11.00348	28.46456	21.15151	30.92588	39.31632	31.39992	23.70393	5.08114	5.130144	4.732075	43.93676	32.21555	46.48166	12.3595	10.58748	12.41421	74.48704	49.79802	56.44941	65.16551	95.90812	110.7421
LOC107795479	1.390306	0.950319	1.117921	0.976148	0.615117	0.635268	0.816276	1.039425	1.14212	1.830227	2.120371	1.174569	2.747181	2.502646	2.639897	2.693749	1.712519	1.788017	1.375516	2.17579	0.824188	1.857729	1.440951	1.743776	0.83494	0.580353	1.396745	2.485663	7.195433	0
LOC107824404	0.146517	0.066766	0.063682	0	0.064824	0	0	0.062594	0	0	0	0	0	0	0	0.054592	0.212321	0.054617	0	0	0	0	0	0	0	0	0	0	0	0
LOC107804130	0	0.062978	0	0.062603	0.18344	0	0	0.059043	0	0.067384	0.145364	0	0.076926	0.075388	0	0.051495	0.066759	0	0	0	0.126045	0.057709	0.060953	0	0	0	0.163347	0	0	0
LOC107791237	8.85173	6.812353	8.085459	19.37341	19.54418	25.64975	11.57252	11.38075	10.92223	21.34597	19.05401	21.17633	23.52416	18.118	16.64173	9.172025	8.931583	9.993287	19.38998	20.56216	14.53127	10.16149	10.36084	12.21828	22.34703	13.68504	24.9096	26.12479	16.91086	12.16257
LOC107781181	58.35789	59.61446	64.42205	24.44309	22.7547	60.97353	9.524158	10.62113	12.05654	12.15235	10.7569	11.11641	14.82395	18.42679	7.369433	48.41469	66.98209	62.39724	25.86956	32.13522	15.05832	8.030246	19.05571	14.94311	2.079496	1.848472	9.981651	0	1.370515	5.867246
Novel04056	4.045525	4.162739	1.928507	0.236454	0.274255	0.227995	0.027364	0.027876	0.03063	0.763529	1.944529	1.33679	0.671898	0.391517	1.004568	9.810029	9.518644	10.25234	0.728328	0.289786	0.7885	0.095362	0.100722	0.204599	0.969332	0.590143	0.308483	1.166582	3.141387	0
LOC107804556	0	0	0	0	0	0	0	0	0	0	0	0	0	0.076242	0	0	0	0	0	0	0	0	0	0	0	0	0	0	0	0
LOC107771527	0.491829	1.008543	0.587862	5.959525	4.624026	6.345313	4.537697	3.519442	2.251034	2.397976	1.767462	1.506226	2.121604	0.871912	1.135799	0.274884	0.237574	0.137505	3.680669	4.732606	2.747406	1.540271	2.277584	1.606435	0.932732	0.342172	1.30793	0	1.479899	3.801314
107760507	0	0	0	0	0	0	0	0	0	0	0	0	0	0	0	0	0	0	0	0	0	0	0	0	0	0	0	0	0	0
Novel04417	9.070712	8.503031	9.762353	24.44412	21.04691	15.91299	7.076645	7.971698	11.24651	3.453797	4.967161	4.403293	4.584009	3.769791	3.044199	0.085835	0	0.042937	0.11688	0.048719	0.236361	0.048096	0.177799	0.12039	0.072814	0.040067	0	0	0.138634	0
LOC107831599	0.189896	0.144223	0.137561	0	0.112022	0.102081	0	0.054084	0	0	0	0	0.176163	0.138112	0.129936	0	0.091728	0.188768	0	0.08032	0.144324	0.026431	0.055834	0.13232	0	0	0	0	0.152371	0
LOC107832276	0	0	0	0	0	0	0	0	0	0	0	0	0	0	0	0	0	0	0	0	0	0	0	0	0	0	0	0	0.131684	0
LOC107779074	2.265619	3.112658	3.233433	3.492375	4.428445	3.162927	5.587368	6.47195	5.714498	5.704519	5.240186	4.513749	3.839644	4.611342	7.080229	4.435048	2.450125	3.327809	6.313654	7.065519	6.260528	5.676233	5.607523	4.4533	5.557852	10.2572	4.716089	7.254797	9.930706	27.18099
LOC107763980	19.97245	30.85485	20.95241	1.11882	1.469604	1.648234	2.143081	2.328699	2.238927	1.951718	1.672202	2.050697	0.995535	1.254375	0.917873	18.43776	29.33343	18.66844	0.993887	0.396265	1.592374	2.062711	1.765458	2.403541	0.430725	0.592542	0.956313	0	1.845182	0
LOC107772430	0	0	0	0	0	0	0	0	0	0	0	0	0	0	0	0	0	0	0	0	0	0	0	0	0	0	0	0	0	0
LOC107795035	0.717122	0.95312	1.038964	0.730885	0.766756	0.803118	0.852098	1.429699	0.645213	0.349642	0.523798	0.075731	0.066526	0.065195	0.122672	0.512133	0.923733	1.047012	0.697365	0.50553	0.436018	1.347496	1.555016	1.124303	0.717772	0.041576	0.176579	0	0	3.695059
LOC107827236	0	0	0	0	0	0	0	0	0	0	0	0	0	0	0	0	0	0	0	0	0	0	0	0	0	0	0	0	0	0
LOC107787750	1.164858	0.884687	1.046339	1.196009	0.858952	1.210619	1.40039	0.928934	0.984258	0.832981	1.388558	0.590462	1.167062	0.465961	1.354988	1.273145	0.825257	0.868451	0.827412	0.62408	0.637422	1.361924	0.958983	1.379843	0.785458	0.37819	0.275354	0	0.373869	0
LOC107795689	0.047062	0.021446	0.04091	0.063953	0.104108	0.025299	0	0.020105	0	0	0.0165	0	0.209559	0.179697	0.120757	0.087676	0.045466	0.05263	0	0	0.021461	0.039303	0	0.024594	0	0.032742	0	0	0	0
LOC107804629	2.640996	3.342976	3.82627	4.120604	4.284367	3.470371	6.153104	5.014531	4.407971	3.862957	5.041197	3.8426	4.083325	2.080865	5.873067	3.936147	5.528087	3.609787	3.275434	4.840553	3.211511	7.229352	5.564997	1.840246	3.524524	2.245683	2.948032	0	1.412744	0
LOC107815931	8.142417	7.648267	7.132268	9.128917	9.496174	9.860933	9.132027	8.556436	8.405977	9.217567	8.00638	8.433949	9.967204	10.10821	7.55666	5.719018	7.353898	7.466038	9.591937	8.128279	8.478752	6.982319	7.952649	10.95598	9.229543	9.593245	9.033724	11.15513	17.87302	15.43166
LOC107818016	0	0	0	0.219184	0	0	0	0	0	0	0	0	0	0	0	0	0	0	0	0	0	0	0.106703	0	0	0	0	0	0	0
LOC107783773	77.54314	106.2039	75.33102	85.30011	91.9148	81.76863	88.73519	83.74808	65.62162	88.36062	105.8014	73.00526	110.057	98.09574	86.72151	163.4324	108.1028	140.4219	96.8039	101.5729	71.39058	81.49448	82.65265	77.85891	106.0631	107.686	64.35741	40.40878	124.9539	67.54611
LOC107806804	11.90361	13.29271	11.37058	20.43206	22.58556	20.26498	19.45034	18.36343	16.55516	22.97049	21.94254	21.8733	30.47577	25.40424	27.06869	13.15492	12.58092	15.04519	24.23624	22.78317	19.02051	18.19029	15.87729	16.57543	35.4414	35.21989	24.67226	22.07525	43.93322	67.99522
LOC107787309	53.23128	54.17683	54.95523	63.15307	64.94163	56.11671	60.49496	57.55579	51.76921	66.00049	57.12639	58.95066	74.83443	61.41195	67.39041	61.48284	57.18435	63.17531	66.46256	61.67533	63.0513	59.59984	50.91197	55.03596	51.99569	45.75978	54.10208	39.49976	74.56663	41.26664
LOC107814314	0.019378	0.052983	0.03369	0.105335	0.034295	0.041668	0	0.016557	0.018193	0	0.054353	0.081857	0.043145	0.021141	0.039779	8.534508	8.068813	9.838683	4.011424	4.049016	3.075173	5.162502	5.777438	8.202982	2.450016	1.186398	3.023287	0	3.078716	1.198194
LOC107803504	1.258082	2.734168	1.640436	1.972659	1.712667	1.950854	2.780066	2.790708	1.203857	1.675039	2.256314	1.042423	0.781056	0.580675	2.55769	3.046954	1.54264	2.561335	2.479579	2.537829	1.081196	2.303364	2.667576	1.466672	0.734122	1.312897	0.343143	3.46041	0.815346	1.495944
LOC107773695	3.866473	4.388145	3.741538	2.908008	3.679521	4.000066	2.998224	2.493297	5.273795	2.703233	2.91579	3.266504	4.385423	4.536481	1.946783	3.968583	3.101035	3.9704	2.961079	1.974804	1.59681	2.802522	3.925275	3.659987	0.830105	0.507539	1.465804	0	0.702437	0
LOC107801068	0.06258	0.114069	0.1088	0	0.055375	0	0	0	0.058753	0	0.087763	0.105739	0	0.068272	0.064231	0.839429	0.120916	0.746501	0.063502	0.052939	0	0.104525	0	0.130818	0	0.087076	0.073965	0	0.602568	0
LOC107772360	2.483014	6.250082	2.672343	0.535592	0.313876	0.889848	0.396688	1.212317	1.776124	2.536549	2.65309	0.899022	2.237625	1.547912	1.820355	4.669917	3.1984	4.84836	0.479922	0.900197	0.970521	0.987443	1.877299	2.965994	1.046428	0.329041	0.558995	0	1.138487	0
LOC107783268	2.416228	4.450385	3.201971	2.984973	2.496869	2.270752	5.456215	4.042359	3.807207	2.504415	3.779504	1.827174	2.652159	2.470084	4.647738	5.855019	3.803406	5.064076	1.388794	2.101136	1.279036	5.630238	4.202929	2.525441	0.491339	1.645736	0.559175	1.208353	0.406734	1.044748
LOC107808035	0	0	0	0	0	0	0	0	0	0	0	0	0	0	0	0.131437	0	0	0	0	0	0	0	0	0	0	0	0	0	0
LOC107784612	0.592119	0.78871	2.058866	0.165055	0.604553	0.244846	0.382027	0.389171	3.078872	0.399732	0.31938	0.307839	0.405634	0.944114	0.560986	3.360272	1.012065	2.614742	0.184874	0.847663	0.249241	0.874874	1.0044	2.427924	0.230344	0.126752	0.215334	0	1.096412	5.632556
LOC107773271	7.81423	8.679581	8.172521	14.71166	9.507499	9.713949	9.011931	9.284773	10.2021	12.02509	11.72922	11.44979	13.04837	10.38977	10.02539	5.914161	4.128471	6.007898	7.681549	10.43188	7.460774	7.035701	6.24648	9.18836	6.637831	3.397791	7.792699	0	8.229481	7.549463
LOC107805649	0	0	0	0	0	0	0	0	0	0	0	0	0	0	0	0	0	0	0	0	0	0	0	0	0	0	0	0	0	0
LOC107812631	0	0	0	0	0	0	0	0	0	0	0	0	0	0	0	0	0	0	0	0	0	0	0	0	0	0	0	0	0	0
LOC107816505	0	0	0	0	0	0	0.106247	0	0	0	0	0	0	0	0	0	0	0	0.053579	0	0.052894	0	0.0662	0	0	0	0	0	0	0
LOC107763820	0	0	0	0	0	0	0	0	0	0	0	0	0	0	0	0	0	0	0	0	0	0	0	0	0	0	0	0	0	0
LOC107822030	0.493631	0.299923	0.286069	0.447205	0.679463	0.176905	0.736053	0.562363	0.56643	0.160451	0.538432	0.278022	1.587494	2.513128	1.125888	0.694836	0.211951	0.654263	0	0.092795	0.300133	0.824489	0.387036	0.458616	0.069344	0	0.064826	0	0.528115	0
Novel01250	0	0	0	0	0	0	0	0	0	0	0	0	0	0	0	0	0	0	0	0	0	0	0	0	0	0	0	0	0	0
LOC107797768	1.157227	2.941974	1.455985	1.324281	0.75452	0.654821	1.226042	0.702545	0.257319	2.969586	6.128572	2.392689	2.135769	0.664462	3.250674	5.605386	2.206537	2.542878	2.070431	2.85952	2.416335	1.754835	2.363844	1.177704	0.50053	1.610197	0.611888	0	0.43984	0
LOC107816675	2.47009	2.903394	2.528475	4.893829	3.697303	4.392989	3.911194	3.254528	3.749459	2.431161	3.205062	2.866913	3.315106	4.029528	3.767309	1.100988	0.981288	1.135914	1.264951	1.113112	0.821101	1.17601	1.099573	0.868622	1.371761	0.481815	0.709399	0	1.667088	2.854755
Novel01347	0	0	0	0	0	0	0	0	0	0	0	0	0	0	0	0	0	0	0	0	0	0	0	0	0	0	0	0	0	0
LOC107771784	0.374906	0.097623	0.093114	2.911252	3.080459	4.664097	0.94335	1.693173	1.407914	4.386971	3.267279	6.92284	2.146376	2.804601	2.14385	0.239469	0.310449	0.319438	3.315167	3.851045	3.712283	1.162921	1.37001	1.623387	6.094228	7.005078	18.73718	3.830157	4.125559	0
LOC107797593	8.533044	7.238422	8.330529	11.0903	9.467289	10.7266	10.35017	11.04846	10.47618	14.24136	12.74919	13.11475	15.9658	14.89461	12.86764	13.13344	9.575302	9.297937	7.593052	10.7165	6.764597	9.510693	9.871568	10.9769	10.78836	9.498486	10.27932	9.388204	15.01041	4.058546
LOC107812179	15.40424	13.38213	13.04247	8.779607	8.386298	8.295073	12.40518	12.65997	16.64279	12.31364	14.52675	14.75063	13.96822	10.74722	7.836743	13.82684	19.21479	12.81807	6.041159	7.791456	6.476629	13.97926	15.51855	13.8403	3.409082	3.454681	8.077787	1.909222	8.611474	8.253618
LOC107780237	0	0	0.05999	0.125042	0.061066	0.074196	0	0	0.064791	0	0	0.058303	0.076825	0	0	0	0	0.102902	0.140056	0	0	0	0.060873	0	0	0	0.081566	0	0	0
LOC107807889	1.386523	1.755062	1.004396	2.372671	2.794576	3.188403	0.775291	0.361986	0.650864	0.751131	0.891212	1.952289	1.371997	3.025257	1.699801	1.004538	1.153457	1.464425	4.416253	3.453548	5.444514	0.482467	0.679447	1.247917	0.194776	0.375131	1.092506	2.754334	0.927113	4.762823
LOC107771854	1.404902	1.528829	2.624778	1.120797	1.057605	1.645704	0.932129	0.842062	1.555219	0.511178	0.823382	0.478303	0.606908	0.366013	0.667172	1.609466	1.174935	1.938497	0.680882	0.567618	0.305981	0.910598	1.202225	1.227322	0.079532	0	0.198266	0	0.100951	2.59305
LOC107759312	4.169241	5.56583	6.635919	3.457928	2.909806	4.798109	0.788039	0.652254	1.653916	1.832355	1.399974	0.843365	1.568876	1.665625	0.542433	3.281961	6.580692	5.603779	4.766938	3.874613	3.159756	0.78464	1.294911	1.288894	0.593937	0.081707	0.763448	0	0.565414	0
LOC107771814	1.079995	1.287138	1.119359	2.408421	3.160998	1.295103	2.020716	1.668097	2.846851	2.268283	2.44664	2.281025	2.728218	1.767332	2.344856	1.083403	0.842719	1.26971	2.697611	2.84622	2.045715	2.185451	2.271649	1.693216	2.153197	0.92476	1.276467	0	3.999601	5.136748
LOC107800672	0	0	0	0	0	0	0	0	0	0	0	0	0	0	0	0	0	0	0	0	0	0	0	0	0	0	0	0	0	0
LOC107801185	2.430103	2.234873	2.32368	2.041437	2.599926	2.375143	2.242055	2.095222	2.322969	2.089607	1.967333	1.642415	2.975747	2.1691	2.154074	2.535282	2.027545	2.322327	2.757325	2.522904	2.256591	2.601376	2.65016	2.678485	2.09481	1.106611	0.99221	0	2.339876	1.365972
LOC107829792	0.819501	0.497916	0.178094	0.123738	0.543861	0.513954	0.229117	0.466803	0.256461	0.066593	0.143659	0.057695	0.22807	0.074503	0.280371	0.45802	0.461829	0.458229	0.138595	0.05777	0.18685	0.342193	0.84333	0.428271	0	0	0.080715	0	0	0

LOC107761532	11.43412	12.40016	12.04948	7.465232	7.461068	9.752049	7.072168	8.077676	10.31506	6.851777	6.136384	7.231404	9.030921	9.198749	7.146299	8.139904	11.35488	9.000857	3.500212	3.890606	3.786743	6.881632	8.677039	9.347102	2.099784	0.444406	3.24643	4.568163	3.382833	3.949658
LOC107815824	26.6692	31.15531	29.04954	159.886	243.1629	120.9363	143.5386	167.699	77.38267	244.9709	129.7336	123.1828	222.9947	163.6503	199.8923	32.1326	21.18965	28.31651	303.2274	250.2556	300.6335	143.0797	83.94904	75.18738	808.9647	356.5699	599.4626	585.4673	506.288	276.2158
LOC107827403	0	0	0	0	0	0	0	0	0	0	0	0	0	0	0	0	0	0	0	0	0	0	0	0	0	0	0	0	0	0
LOC107769640	0.173443	0.237108	0.603081	0.235696	0.204632	0.217551	0.218211	0.172893	0.678483	0.084564	0.182427	0.195373	0.257439	0.346899	0.207687	0.366208	0.279267	0.344824	0.058666	0.122267	0.184547	0.531105	0.484462	0.453206	0	0	0.102498	0	0.556677	0
107799897	0	0	0	0	0	0	0	0	0	0	0	0	0	0	0	0	0	0	0	0	0	0	0	0	0	0	0	0	0	0
LOC107788108	12.28825	12.89119	11.83464	16.47855	16.75998	17.9399	16.20151	16.18345	14.42094	16.22822	16.48918	13.23822	20.66679	18.13169	19.30411	15.23458	14.49773	14.58246	15.83328	16.56471	13.08166	16.16856	15.22535	16.07769	16.70967	15.59134	15.15064	15.80546	23.51504	23.23132
LOC107803279	0	0	0	0	0	0	0	0	0	0	0	0	0	0	0	0	0	0	0	0	0	0	0	0	0	0	0	0	0	0
LOC107791590	0.613346	0.670787	0.426535	0.666793	0.868366	1.18696	1.54332	0.628872	0.460669	0.837326	0.774142	0.621806	0.682785	0.936783	0.88133	0.27424	0.94807	0.914551	1.244763	0.10377	0.895011	0.409777	0.432809	1.410352	0.310182	0.170685	0.724926	0	0.590573	0
LOC107795151	0	0	0	0	0	0	0	0	0	0	0	0.532239	0	0	0	0.044712	0	0	0.030442	0	0	0	0	0	0	0	0	0	0	0
LOC107774833	301.6085	260.9379	310.6694	182.4647	170.972	262.1372	141.0008	147.7961	165.1545	146.0739	167.717	214.7978	117.3078	147.6635	107.6016	204.206	277.6462	258.9599	147.1462	147.0454	189.6757	125.2309	170.6355	222.8652	89.95907	54.39322	157.8762	348.0698	90.0093	66.87621
LOC107828460	2.703682	3.325014	2.916579	3.511788	3.458885	2.241379	4.590032	4.119213	3.302894	2.858781	3.266298	2.201586	3.698761	2.771898	3.209621	3.131416	2.486105	3.181421	2.677401	1.984015	1.841925	3.645309	2.72962	2.6897	2.388675	1.767675	1.655518	0	3.450153	10.07065
LOC107764548	0	0	0	0	0	0	0	0	0	0	0	0	0	0	0	0	0	0	0	0	0	0	0	0	0	0	0	0	0	0
107765653	0	0	0	0	0	0	0	0	0	0	0	0	0	0	0	0	0	0	0	0	0	0	0	0	0	0	0	0	0	0
LOC107799910	0	0	0	0	0	0	0	0	0	0	0	0	0	0	0	0	0	0	0	0	0	0	0	0	0	0	0	0	0	0
LOC107771179	1.925901	2.53532	2.066846	3.920345	4.186774	4.601288	3.749176	4.022437	2.567085	3.129995	4.28474	2.189494	2.752713	3.008931	4.246228	2.10848	2.434837	2.28671	4.897742	5.61162	2.840686	5.242097	3.082957	2.932448	3.877843	2.282751	2.641586	1.70036	2.632783	4.410422
LOC107811234	1.855267	3.004126	2.567864	2.725144	2.167631	1.200656	3.807138	3.293502	2.773361	1.545655	1.894535	1.445643	3.288453	2.96723	3.364694	5.221463	3.097448	3.854104	1.242879	1.371345	0.837803	4.151741	1.811236	1.581422	1.138651	1.102763	0.532227	0	3.815575	3.341182
LOC107763113	247.4047	196.4439	251.365	107.7127	99.17429	197.9498	82.69064	88.34453	113.3079	64.09099	68.82862	85.47281	41.77544	67.63315	39.47394	128.1688	232.2897	166.0484	79.4358	62.39773	107.3217	77.00091	122.5946	131.1686	23.49589	10.28344	57.49873	30.78817	18.30858	17.74641
LOC107832440	11.66051	10.82954	12.22784	11.23455	8.647457	10.74555	11.61202	8.508135	11.12117	5.378414	5.347079	5.691761	4.656549	5.936488	5.015175	10.648	11.69418	10.95645	7.625249	7.390165	8.912794	10.82	11.81998	13.65781	2.106062	3.193443	3.937654	0	4.811816	2.288848
LOC107831147	0.538565	0.506669	0.543673	0.519391	0.568794	0.672414	0.466289	0.638292	0.636113	0.355759	0.36546	0.440317	0.11604	0.435922	0.499274	0.737946	0.721705	0.88076	0.51124	0.558464	0.443647	0.870522	0.628292	0.762649	0.241613	0.21756	0.308003	0	0	0
Novel04992	0.035013	0	0	0.03172	0.15491	0.037643	0.029367	0.059832	0	0.375565	0.049103	0	0.233863	0	0.10781	0.078276	0.135303	0.052208	0.106587	0.148094	0.383192	0.204683	0.277956	0.292766	4.471001	0.58462	2.524351	2.50394	2.697057	0
LOC107801372	11.8979	15.9346	5.581214	0.953012	0.83454	0.194995	16.61185	12.30444	3.609904	0.282974	2.162009	0.091936	1.655613	1.582929	3.909225	12.1912	6.763454	7.382974	0.036808	0	0.033083	13.05647	4.127501	0.151655	0	0.100945	0	0	0	2.242881
LOC107801597	0	0	0	0	0	0	0	0	0	0	0	0	0	0	0	0	0	0	0	0	0	0	0	0	0	0	0	0	0	0
LOC107777385	0	0	0	0	0	0	0	0	0	0	0	0	0	0	0	0	0	0	0	0	0	0	0	0	0	0	0	0	0	0
LOC107779510	0	0	0	0	0	0	0	0	0.040177	0	0	0	0	0	0	0	0	0	0	0.036201	0.039029	0	0	0	0	0	0	0	0	0
LOC107823928	10.30422	10.17361	10.00225	13.04321	12.51171	14.89415	11.23537	10.61395	10.64146	17.44429	15.77529	15.08916	16.66452	15.55059	11.86858	9.150467	11.33734	11.26727	12.89574	15.76262	9.998021	9.250718	9.947405	10.41096	21.35089	13.81982	23.68091	18.42243	20.80787	24.77709
Novel04611	0.503954	0.430587	0.410697	3.680992	3.623198	1.659301	2.958818	2.718114	2.306531	2.211386	1.21472	1.303874	0.210378	0.412342	0.193967	0.164302	0.426005	0.305272	3.419841	2.451287	2.958771	1.473029	4.723037	2.04109	0.159288	0.043826	0.707315	0	0.454915	0
LOC107765412	0.962863	1.053037	0.292949	0.087231	0.0852	0.155279	0.121139	0.041135	0.180796	0	0	0	0.053594	0.262609	0	0.358763	0.744166	0.430713	0.097705	0.040726	0.043907	0.040206	0.254793	0	0	0.066988	0.056901	0	0	0
LOC107797531	0	0.208918	0.149451	0.31151	0.405681	0.18484	0.144201	0.048966	0.053804	0	0.200923	0	0	0	0	0.34165	0.055365	0.213629	0.755982	0.581746	0.313597	0.04786	0.151649	0.119797	0.217365	0	0	0	0.551805	0
Novel00806	0	0.063992	0	0	0.06213	0.226466	0.294458	0.119986	0	0.342338	0.590811	0.296594	0.312654	0.1532	0.144132	0.261618	0.135666	0.314086	0.071248	0.237585	0	0.293188	0.309668	0.220163	0.088772	0.097698	0	0	0	0
Novel00234	9.723966	10.87096	12.73561	7.869749	6.252534	8.224169	4.676054	5.317389	7.181706	5.120331	5.863868	5.914855	5.412427	6.082131	5.056742	7.584472	7.07694	6.911313	4.078446	5.154838	5.498421	4.926581	5.432214	5.962579	1.721167	1.623623	3.677742	4.636007	0.936293	0
LOC107802877	9.906478	10.00167	9.384978	11.98427	9.868203	11.47977	10.00064	12.06299	9.913255	6.941366	7.445573	8.870422	9.905416	10.16033	6.332004	9.548389	11.28974	9.375859	6.982512	5.369349	10.2251	9.313496	10.25558	15.31437	3.599942	1.733335	3.715922	12.72671	7.996483	0
107820672	0	1.989071	0	0	0	0	0	0	0	0	0	0	0	0	0	0	0.702823	0	0	0	0	0	0.6417	0	0	0	0	0	0	0
LOC107800437	0	0	0	0	0	0	0	0	0	0	0	0	0	0	0	0	0	0	0	0	0	0	0	0	0	0	0	0	0	0
LOC107832442	0	0	0	0	0.196394	0	0	0	0	0	0	0	0.082359	0	0	0	0	0	0	0.125169	0.067473	0	0	0	0	0	0	0	0	0
LOC107818873	4.070568	3.849814	2.637153	4.522686	5.063057	5.284659	3.124279	3.051456	3.425044	4.755692	3.931373	4.509539	3.248938	3.728587	2.680175	2.661372	3.487321	3.235191	5.104713	5.327556	4.097678	3.527718	4.030856	3.652497	5.680499	4.114341	5.355735	5.492513	3.32782	9.497711
LOC107759971	0	0	0	0	0	0	0.176971	0	0.049523	0.051437	0	0	0	0.057547	0.10828	0	0	0	0	0.160578	0.089244	0.176208	0	0.055133	0.333454	0	0	0	0	0
LOC107823427	0	0	0	0	0	0	0	0	0	0	0	0	0	0	0	0	0	0	0	0	0	0	0	0	0	0	0	0	0	0
Novel03982	5.477622	6.864244	7.142371	14.06027	20.29665	14.72278	20.483	17.74587	9.856713	16.80307	18.40437	17.73931	16.64182	14.69055	20.26292	7.99378	4.630363	6.976491	18.7593	21.33429	18.10935	20.68058	10.87116	10.85404	32.75127	35.56799	14.16211	8.160988	19.77842	70.56034
LOC107769873	4.259091	4.203319	3.947796	2.558135	2.186276	2.757546	4.499893	3.980875	3.998622	1.835647	2.705979	1.76928	5.291373	3.18321	3.743476	4.155854	4.091907	4.841945	1.169999	0.696693	1.030107	4.421534	3.217149	3.639983	0.357002	0.032742	0.361551	0	0.793003	0
LOC107813991	18.6587	12.10795	11.84289	8.049448	13.55279	7.278145	19.55345	18.87072	17.19983	10.97452	15.8426	12.3319	25.24564	25.20233	20.84439	12.99013	20.35575	15.64577	9.101849	9.663661	15.66649	15.19369	9.516652	8.844477	8.023902	16.48395	3.50049	27.23186	17.3141	28.77699
LOC107788479	1.674435	1.839075	2.313953	1.089095	1.48165	1.707903	0.792237	1.283946	1.410799	1.004791	0.842954	0.834257	1.099285	0.889944	1.057596	1.759705	1.368777	2.208641	0.740634	0.690069	1.096389	0.788821	1.249737	1.39112	0.434255	0.418179	0.558193	0	0.620102	0
LOC107795378	24.72149	28.0434	27.92921	24.10395	21.13827	23.04424	23.85993	21.87175	22.26314	18.59284	16.65855	19.18199	24.05588	23.76606	17.68045	24.85729	27.14367	22.28704	16.32347	15.57327	18.46887	21.81695	21.41936	18.4629	14.68564	9.331414	17.40695	34.48103	21.52455	13.55113
Novel03820	0.5894	0.443746	0.423248	0.835774	0.362811	0.303063	0.816761	0.634974	0.577414	0.774649	0.772669	0.432993	1.369312	1.118273	0.94687	0	0	0	0	0	0	0	0	0	0	0	0	3.665258	0	0
LOC107762807	0	0	0	0	0	0	0	0	0	0	0	0	0	0	0	0	0	0	0	0	0	0	0	0	0	0	0	0	0	0
LOC107788845	0	0.072938	0.069569	0.181258	0.141632	0.129063	0.201374	0	0.037568	0.156079	0.112234	0.236641	0.222727	0.087309	0.328563	0.059639	0.115974	0.059666	0.284232	0.1354	0.036494	0.100253	0.141184	0.041824	0.101182	0.055678	0.094589	0	0.192647	0
LOC107789883	0	0	0	0	0	0	0	0	0	0	0	0	0	0	0	0	0	0	0	0	0	0	0	0	0	0	0	0	0	0
LOC107820000	20.56676	11.58341	19.75312	0.767629	2.521934	0.414078	0.646076	0.658157	0.506228	40.33571	0.702167	0.715839	8.403483	5.210165	0.869666	14.29313	16.37164	11.94511	2.892059	1.694193	4.355612	1.608225	3.669015	5.555242	315.7324	10.28931	286.7828	60.59534	180.6017	33.33976
LOC107773712	1.768815	2.392085	2.132791	2.946454	3.332285	2.699177	4.54646	4.241458	4.821218	2.670687	2.800665	2.747666	6.669431	6.660545	4.099434	2.763807	1.874195	2.382217	2.605459	2.992592	3.226379	3.47853	3.825036	3.578256	3.174132	1.190892	2.360352	8.160988	4.120504	3.528017
LOC107772451	3.337926	3.159112	2.901588	3.372914	2.840016	7.729462	2.476624	3.510172	1.928487	2.37858	4.681112	3.470736	5.859571	4.201746	3.425956	2.104745	2.108469	1.435711	0.521092	1.086021	0.702518	1.715439	0.792688	1.7444	0.649254	0	0.758685	0	2.472302	0
Novel01668	1.030904	1.006652	0.810796	0.555921	1.194563	0.976402	0.494094	0.692083	0.553062	0.359022	0.877772	0.228102	0.765078	0.696222	0.856551	1.152347	0.735099	1.189474	0.797021	0.602146	0.134315	1.209409	0.822723	0.436131	0.186197	0.204918	0.087032	0	0.70902	0
LOC107772646	2.308875	4.291029	2.007056	4.798659	4.246327	4.088531	5.240083	4.40971	2.465197	4.105575	5.364869	4.474909	4.082196	5.235296	3.810211	2.631469	3.105299	3.003949	1.975375	4.902021	5.11982	4.234482	4.791953	3.69083	2.060555	1.511826	2.086809	3.237592	4.14116	0
LOC107777782	0.698397	0.636503	0.682989	0.158178	0.077248	0.281573	0.512554	0.820503	0.245882	0.085128	0.367287	0.073753	0.291549	0	0.358408	0.780669	0.253016	0.390513	0.177171	0.147699	0.398094	0.291625	0.308016	0.182491	0	0	0	12.48631	0.840583	0
LOC107810243	0	0	0	0	0	0	0	0	0	0	0	0	0	0	0	0	0	0	0	0	0	0	0	0	0	0	0	0	0	0
LOC107795830	9.946908	9.324391	8.475604	14.71531	17.06172	14.3137	12.63353	11.63697	12.97141	17.99202	13.56633	15.8834	17.69252	15.00143	13.70954	10.03531	9.54619	8.768622	15.37308	16.274	15.13287	12.12263	12.14838	12.08737	33.33309	14.87462	25.52828	18.76074	40.52058	35.1447
LOC107762176	0	0	0	0	0	0	0	0	0	0	0	0	0	0	0	0	0	0	0	0	0	0	0	0	0	0	0	0	0	0
LOC107782088	0	0	0	0	0	0	0	0	0	0	0	0	0	0	0	0	0	0	0	0	0	0	0	0	0	0	0	0	0	0
LOC107824926	18.38398	13.19103	8.446998	11.97571	9.476381	11.85902	12.33555	10.14772	10.98593	12.23569	13.64794	15.97505	18.1589	19.29198	15.66411	10.35093	21.42533	12.0091	7.876469	7.899218	9.980061	9.747901	9.343441	9.759966	1.770891	2.801616	3.310993	8.347395	4.074134	3.608601
LOC107778369	1.769003	1.395954	1.106435	2.736193	2.23346	2.272997	2.225609	2.064467	2.248181	2.966162	2.964921	3.262391	3.170059	2.847773	2.302784	1.253958	1.396383	1.109778	2.189101	2.737418	1.554336	1.459325	1.52232	2.119542	1.227379	2.461439	2.54978	1.54279	1.038611	0
LOC107830532	0	0	0.052216	0.01814	0.07087	0.043054	0.151146	0.393485	0.169185	0.078099	0.0702	0.135327	0.222897	0.240283	0.657629	0.014921	0.038688	0.059712	0.548581	0.203255	0.438268	1.003296	0.300245	0.209279	0.25315	1.142273	0.118327	0	0.192794	2.476085
LOC107762149	0.507105	0.269596	0.367347	0.459412	0.355237	0.159017	0.513942	0.559659	0.535604	0.288454	0.192607	0.446267	0.423388	0.852892	0.477106	0.771537	0.551145	0.708879	0.407372	0.125118	0.423948	0.511725	0.410025	0.441689	0.160284	0.1176	0.124866	0	0.305173	0
LOC107771256	4.216887	4.062782	3.875116	3.192668	2.425396	3.270719	3.056879	2.728003	3.054085	2.29097	2.766798	2.26476	3.654846	2.037327	2.102219	3.815807	3.346391	4.19931	1.83386	2.293198	2.060289	2.89277	3.905552	4.250079	1.142449	0.461017	1.459606	0	2.175171	1.862403
LOC107765721	12.52844	11.6167	14.45149	7.461476	6.265992	6.887066	7.584136	6.832351	8.468819	2.677075	3.697317	2.135282	2.571068	2.828663	3.55575	12.3725	11.68253	11.48473	3.670186	4.14712	3.815339	9.935135	9.667144	10.93124	0.550948	0.909517	1.75116	0	0.839184	5.388884
LOC107820209	1.895782	2.534065	1.684585	3.155079	2.398222	3.246019	3.250818	2.555697	2.214917	1.971869	3.761494	1.577882	3.235964	2.298006	3.805079	3.306856	2.374147	2.565034	2.678943	3.290558	1.434417	4.550284	4.013293	2.89148	0.568141	0.46895	0.564315	1.004261	0.811286	3.473158
LOC107804667	4.665404	4.05867	4.854354	3.458143	3.377664	3.647912	3.853786	3.261475	3.384612	2.688257	3.122685	3.344271	2.754178	3.238917	2.756982	2.949897	4.097429	4.637675	2.080148	1.494928	1.740652	3.069734	3.741086	4.876279	0.625597	0.295071	0.835475	0	1.361268	0
LOC107814639	0	0	0	0	0	0	0	0	0	0	0.079683	0.057299	0	0	0	0	0	0	0	0	0	0	0	0	0	0.227403	0	0	0	0
NitaMt014	0.717531	0	0.623734	0	0	0	0	0	1.347299	0	0.503133	0	0	0	0	0	0	0.53495	0.728101	0	0	0	0	1.499926	0.907177	0	0	0	0	0
LOC107804401	0	0	0	0	0	0	0	0	0	0	0	0	0	0	0	0	0	0	0	0	0	0	0	0	0	0.167932	0	0	0	0
NitaMp143	0	0	0	0	0	0	0	0	0	0	0	0	0	0	0	0	0	0	0	0	0	0	0	0	0	0	0	0	0	0
LOC107761558	1.236432	1.270273	1.055262	1.242343	0.815584	0.459218	0.810784	0.537825	0.590961	0.482271	1.52905	0.664728	0.825848	0.416929	0.784497	1.557988	1.085907	1.139696	0.684352	0.494443	1.373679	1.614575	1.269079	0.728398	0.085267	0.250241	0	0	0.324689	0
LOC107790906	7.291501	11.29024	6.0473	1.550324	1.942184	1.359871	2.683418	3.273958	4.191173	2.357953	2.478145	4.2429	2.898929	3.368559	4.085542	15.08111	15.59788	13.92313	4.416697	3.713453	3.901769	1.366991	3.018908	2.021918	1.175851	1.708186	0.791446	0	2.149217	0
LOC107828499	19.45128	22.23222	17.7741	5.090047	3.461233	5.925846	3.877333	4.891721	5.675487	4.2304	4.164084	3.695167	1.702189	1.706941	1.13143	15.44909	22.22696	15.66825	3.427956	4.512187	2.691793	4.603038	7.088761	4.645902	0.224793	0.346353	1.428985	2.543037	0.513594	2.198723
LOC107815536	6.787697	8.608437	7.108917	6.14931	5.933837	7.385528	7.853782	6.777838	7.524262	5.462555	6.365165	6.079883	5.416765	4.237827	4.364684	7.221647	7.703071	7.49932	3.734288	4.531278	5.220899	6.214953	6.780682	7.521879	1.654306	1.422377	3.28633	0	1.77172	0
LOC107799244	0	0	0	0	0	0	0	0	0	0	0	0	0	0	0	0	0	0	0	0	0	0	0	0	0	0	0	0	0	0
LOC107818074	0	0	0	0	0	0	0	0	0	0	0	0	0	0	0	0	0	0	0	0	0	0	0	0	0	0	0	0	0	0
LOC107828239	14.80298	15.22071	15.11155	11.41633	12.62844	9.222544	12.54324	12.32376	14.1115	7.698545	8.676495	7.824245	7.605637	8.198885	2.882844	17.87619	19.72794	19.63886	6.316539	7.128078	13.01593	10.84083	22.83337	28.56381	2.303441	2.11254	4.66558	0	3.654708	4.693796
LOC107776317	9.043988	5.426299	8.844465	4.77948	4.268115	6.320205	4.614562	4.958433	5.236051	2.572217	4.861931	4.775383	3.272065	4.193282	3.480938	5.897141	7.499475	7.304566	3.059071	4.14407	4.330341	5.098187	6.980221	6.459395	1.715152	1.258405	3.295857	0	2.177049	0
LOC107800014	3.715519	4.625105	4.411464	1.860918	1.630503	1.88365	2.12825	1.935756	2.268809	1.472805	1.800428	1.097365	1.984016	1.614789	1.147153	3.241533	2.276271	3.378143	1.164793	1.456546	1.129539	2.093845	1.971729	2.178526	0.076383	0.378283	0.24992	0	0.436289	0
LOC107820456	0	0	0	0	0	0	0.061359	0	0.068682	0	0	0	0.081438	0	0	0.054516	0.070675	0	0.148467	0	0	0.061095	0.064529	0.152925	0	0	0	0	0	0
LOC107778436	0.751863	1.256255	1.198227	0.189208	0.110883	0.583804	0.245241	0.142758	0.196078	0.081462	0.058579	0.105866	0.092998	0.501261	0.171487	0.622543	0.847423	0.809677	0.254313	0.176673	0.038095	0.034883	0.294752	0.261949	0.05281	0	0	0	0	0
LOC107805609	0.215161	0.392186	0.041563	3.725233	18.19269	1.850595	10.76774	20.20191	7.922994	21.26066	18.5739	10.30053	21.68989	13.69259	23.33494	0.694799	0.531207	0.178235	36.92212	30.69926	26.53472	18.44783	15.87878	4.447751	119.33	90.57935	105.9313	186.3544	70.5537	45.82396
LOC107799144	14.10357	12.97937	13.6388	13.02704	12.81541	14.90355	12.61018	12.55135	13.3058	9.381468	9.333302	9.293186	8.521918	11.02246	8.883508	11.56357	14.82452	11.85166	8.88772	8.488555	11.41603	9.301657	9.824476	13.47959	2.659421	2.20711	4.564694	7.398078	3.984329	8.528557
LOC107769073	8.824509	7.065868	9.42432	4.454111	5.354404	5.82799	4.969575	5.978063	7.929773	1.721002	2.784488	2.236557	2.10505	2.200481	2.00553	6.388147	7.18554	6.626039	2.878233	1.546306	3.621645	6.4747	9.34056	13.24213	0	0.175409	0.297996	0	0.910378	0
LOC107802686	0.045469	0	0	0	0	0	0	0	0	0	0	0	0	0	0	0	0	0	0	0	0	0	0	0	0	0	0	0	0	0
LOC107811928	0.600163	0.603558	0.629649	1.16243	1.776317	1.223748	0.815827	0.689622	0.738326	1.069567	1.393108	0.611938	0.967608	1.174032	1.040812	1.00244	0.579808	0.802319	1.218008	1.155448	0.585108	1.03699	0.894476	0.90849	1.098935	0.806287	0.635964	1.480005	2.69013	0
LOC107809214	4.067525	2.15614	2.814219	1.804884	0.697807	1.784933	0.800684	1.489459	1.558682	1.052297	1.455179	1.051946	1.339926	1.041447	0.724199	1.85579	2.766739	2.289856	0.800219	1.263984	0.94634	1.039866	1.171535	2.038925	0.314852	0.34651	0.24528	0	1.798394	0
LOC107762489	1.103377	0.921793	0.746	0.027767	0	0	0	0.026188	0.028775	0.029887	0	0	0.034119	0	0	0.525319	0.325708	0.479858	0.031101	0.025927	0	0.076788	0.05407	0	0	0	0	0	0	0
LOC107802962	59.68755	60.26036	63.06857	62.39905	50.02756	69.20539	68.23037	62.55188	72.63486	55.94135	58.05409	64.58482	51.34387	58.52818	58.29739	52.30549	66.75233	59.73517	41.60637	47.12299	46.93313	64.28169	79.43573	83.42448	22.12997	24.59864	44.32391	15.64701	20.85656	21.64558
LOC107775173	4.662177	6.010337	5.576836	7.219994	5.923653	8.268336	5.932397	4.630383	5.274915	6.372468	7.194862	6.042763	8.450386	8.1075	5.357711	6.072531	6.48661	6.416956	4.265857	5.191092	3.016365	4.29284	5.51828	5.435165	1.738095	1.275238	2.872901	2.849672	2.589852	3.695762
LOC107794731	98.29725	93.10576	117.134	48.55113	43.64629	50.42536	42.55694	40.13903	54.01112	31.82767	37.70225	25.67675	18.60296	22.58211	26.60853	87.66069	87.18392	93.96786	29.02192	30.66443	23.96619	53.23029	62.83528	81.76478	26.36192	9.869887	30.96925	21.663	30.86863	24.97326
LOC107830684	0.557232	0.468783	0.670693	0.504824	0.531004	1.059932	0.143807	0.146497	0.321941	0.75236	0.390731	0.362127	0.14315	0.420862	0.175978	0.223596	0.703973	0.543267	0.608935	0.32634	0.078185	0.214781	0.302471	0.31361	0.108386	0.178926	0.303971	0	0.412725	0
LOC107824338	2.373193	3.089818	3.757546	2.457135	1.349973	3.007093	2.132678	1.955302	2.466779	1.074433	2.020674	1.4321	0.849173	1.941778	0.869922	2.905403	3.439056	2.527595	1.806114	1.290573	2.62819	2.194264	2.840925	3.36634	0.214317	0	2.203868	0	0.8161	0
LOC107807924	0	0	0.073797	0	0.07512	0	0	0.072536	0	0.082783	0.059528	0	0.189011	0	0.261399	0.126527	0	0.063292	0.258435	0.143629	0	0.141795	0	0	0	0	0.354373	0.200676	0	0
Novel00441	1.401891	3.252203	2.215699	5.657463	4.961943	6.713912	10.04797	8.166914	8.734499	5.095261	7.774693	1.507363	12.48484	8.481188	15.69669	5.508371	3.693668	3.705601	2.715763	3.77333	2.09217	12.98475	7.53178	6.527052	1.933546	1.24131	3.012587	0	4.294948	0
LOC107816905	94.16869	98.78005	126.7985	197.0016	257.8901	166.3193	181.8453	163.3439	192.3577	285.8765	187.6966	216.1942	190.0367	204.6052	156.3458	155.5453	93.19462	143.3577	309.6914	222.5506	440.9097	154.707	200.7066	175.7993	481.3151	143.5673	492.7234	1250.343	461.3123	561.6148
LOC107782931	5.421935	7.559204	5.919521	2.514475	2.627303	2.498247	2.896395	3.80539	3.605665	1.353238	1.56148	1.090619	1.005962	1.37314	1.82185	5.09864	5.36276	5.413771	1.899433	1.528861	1.412827	8.84038	5.693458	4.283986	0.408034	1.212465	1.106193	0	0.621503	0
LOC107831232	1.642959	1.14205	0.992471	1.665032	1.207392	1.017915	1.19117	1.118272	1.437907	1.357706	1.776881	0.917503	2.448953	1.853146	1.200408	1.618612	1.022288	1.53631	0.819452	0.965814	0.838092	1.279056	1.129884	2.182907	0.880169	1.084909	0.789903	0	0.536257	0
LOC107813324	11.24934	11.10676	9.371366	14.11925	15.24231	10.33065	24.0797	30.83768	24.53321	24.79571	25.30749	28.31328	13.5665	12.91167	17.92038	6.025299	8.603685	5.765969	11.53396	13.38212	14.64129	18.98496	13.33358	10.65552	26.81551	23.47896	27.69963	25.14023	16.92448	0
LOC107806157	0.191516	0.087272	0	0	0	0	0.080317	0	0	0	0.134291	0	0.213199	0	0	0	0.092511	0.142784	0	0	0.174666	0	0	0.100086	0	0.133241	0.113179	0	0	0
LOC107815868	0.023034	0.041986	0.100116	0.041736	0.122293	0.049529	0	0	0.086502	0	0.032303	0.01946	0	0	0	0	0.022253	0	0.046747	0	0.042015	0	0	0	0	0	0.027225	0	0	0
107813520	0	0	0	0	0	0	0	0	0	0	0	0	0	0	0	0	0	0	0	0	0	0	0	0	0	0	0	0	0	0
LOC107786669	1.036683	1.044262	0.711447	0.346014	0.289682	0.351967	1.23562	0.419575	0.56348	0.425641	0.497368	0.553148	0.728874	0.833346	1.176025	1.341775	0.948811	1.301711	0.387562	0.461559	0.497617	1.230292	0.19251	0.456228	0.137967	0.075919	0.386929	3.901972	0.262682	0
LOC107827413	0	0.078775	0.112704	0.156612	0.038242	0.092928	0	0.036926	0	0.042143	0.030304	0	0.04811	0	0	0.225441	0.208759	0.225544	0.701668	0.329032	0.472982	0.288737	0	0.045171	0	0	0.05108	0	0	0
LOC107768152	169.7812	189.837	205.5744	225.0124	246.7351	160.9702	242.1802	272.4167	282.715	266.1504	278.5532	346.7524	266.0769	236.5742	252.4568	352.8085	257.2909	299.4355	358.8893	380.7139	444.5068	274.6739	290.9302	333.5049	206.9298	249.1378	203.5067	991.4714	515.7107	291.8235
LOC107813894	67.13738	83.6301	86.09632	39.67578	33.21362	38.48557	42.91231	42.60052	47.31956	37.34335	41.45316	32.62887	34.57461	35.2584	42.80815	89.90653	61.01787	84.01708	25.56117	28.7921	23.50899	44.99612	41.25626	45.45617	14.09663	17.29828	22.24602	10.88019	20.29955	17.47027
LOC107793509	0.060695	0	0.158283	0	0.322241	0.261018	0.101815	0.363016	0.227932	0.414297	0.170237	0.666598	1.35133	0.728368	0.186887	0.09046	0.175909	0.090501	0.184767	0.051344	0.332129	0.608255	0.374759	0.824699	0	0	0.286946	0	1.168829	0
Novel01296	1.606313	1.400306	0.971362	0.822526	0.803384	0.825948	0.644353	0.656402	1.114666	0.204308	0.391773	0.413018	0.310986	0.9143	0.358408	0.780669	1.484362	1.509985	0.070868	0.354477	1.082814	0.34995	0.985651	0.510975	0	0	0	0	0	0
LOC107775227	0.492291	0.089733	0.085588	0.178396	0.261367	0	0.082581	0.084125	0.092437	0.288028	0	0.415901	0	0	0	0.293619	0	0	0.448978	0.082225	0.26054	0	0.248962	0	0.349109	0	0	0	0	0
LOC107821194	1.873843	0.720468	1.145315	0.371351	0.440433	0.125913	0.196459	0.250166	0.30237	0.428258	0.164242	0.643124	0.293342	0.319417	0.360611	1.003655	1.244574	0.829486	0.237681	0.173375	0.213622	0.268966	0.232432	0.367226	0.40719	0.285175	0.449867	2.093848	0.98671	0
LOC107814576	6.061968	6.732239	6.721031	7.119656	5.476448	7.551549	4.779977	5.350093	5.640124	6.672205	7.635909	5.121319	6.283567	6.276704	4.843347	9.007721	6.733073	8.159375	6.059182	6.80152	3.67471	6.957159	6.787881	8.100174	5.300669	3.964855	4.52624	2.595907	3.844661	5.611087
107781001	0	0	0	0	0	0	0	0	0	0	0	0	0	0	0	0	0	0	0	0	0	0	0	0	0	0	0	0	0	0
LOC107791277	20.16469	18.26726	21.68721	4.663052	4.018705	5.664017	6.653462	6.777878	8.129745	1.948598	3.227032	3.632235	2.291949	4.294039	2.051003	11.77773	22.28883	16.11714	2.089187	2.458803	3.14793	5.96735	9.774615	10.37984	0.612476	0.505544	1.932409	0	2.040721	0
LOC107791889	61.95456	50.52423	67.07493	33.8224	26.20039	50.86484	29.1538	32.9301	37.1656	18.43789	20.98784	23.58741	21.31759	25.98241	19.57203	34.77635	61.87433	48.46925	24.49372	23.61821	33.82878	28.28894	37.11807	36.7505	9.155384	9.516163	12.45781	17.26218	17.04407	0
LOC107825235	0.399628	0.468272	0.521082	1.163708	1.035592	0.889986	1.125264	0.9268	1.55435	0.890714	1.70133	1.085196	2.701003	2.428998	0.644548	1.233755	1.103068	1.042788	0.811029	0.482939	0.520667	1.14425	2.543021	4.057566	0.252625	0.19859	0.944655	0	2.885924	0
LOC107787609	1.375125	2.364098	1.765883	1.302421	0.774328	0.840016	1.022311	0.961318	1.14432	0.975215	0.788922	0.633678	0.695822	1.022859	0.834004	1.979621	1.690801	2.050428	0.792831	0.740257	0.342037	1.592104	0.909713	1.143291	0.434644	0.347888	0.406322	0	0.300925	0
Novel01229	2.685312	2.771676	3.009259	3.517239	3.378128	3.165308	2.849279	2.847272	3.007082	4.259036	3.380222	5.165908	3.205423	3.247205	3.254227	1.687673	2.094143	2.122617	2.987507	3.557911	3.127702	2.972086	3.167676	3.00958	2.945104	2.250853	3.288539	4.627416	2.959433	2.000444
LOC107818571	49.77407	51.22715	46.87317	61.80987	60.77138	66.66282	43.62095	44.891	38.24279	64.76652	64.41509	74.55431	61.24017	55.7224	52.96974	49.53485	58.36071	54.79081	61.94697	63.36058	60.45364	42.25598	40.12809	40.21287	42.92791	56.49328	49.74691	49.43819	43.26654	27.94834
LOC107831287	8.753169	11.29532	8.819458	14.02155	12.86789	12.74726	15.41749	14.29498	10.60851	15.61599	16.78111	10.95793	12.33521	10.67783	16.29373	10.39212	8.955988	10.24857	17.199	19.28556	15.7665	13.32414	12.54642	11.04095	18.2097	29.53504	11.09795	31.29877	13.88733	24.60098
LOC107797506	0	0	0	0	0	0	0	0	0	0	0	0	0	0	0	0	0	0	0	0	0	0	0	0	0	0	0	0	0	0
LOC107800144	71.02341	64.12224	79.29621	30.36214	32.73239	44.94036	34.06682	37.35879	48.13455	22.21989	24.0708	25.75109	21.90738	23.97132	18.30005	46.69681	69.1149	61.16939	23.19742	24.03237	30.0257	34.53776	54.81642	65.49572	14.68525	8.132382	21.24831	10.58162	10.32919	18.29785
LOC107810006	1.68967	1.409423	1.568373	0.98591	1.115013	1.539493	0.552467	0.783024	0.779725	0.726079	0.602439	0.508086	0.765138	0.59362	0.587875	4.588406	3.845737	3.992673	2.150465	2.325713	1.776076	2.870008	2.70293	3.951146	0.398285	0.199242	1.015451	0	1.516634	0
LOC107807530	0	0	0	0	0	0	0	0	0	0	0	0	0	0	0	0	0	0	0	0	0	0	0	0	0	0	0	0	0	0
LOC107829098	2.625487	3.221998	3.457315	1.436555	1.104097	1.285595	1.526214	1.776861	1.561931	0.988585	1.78631	0.570992	1.562647	1.247804	1.814272	3.428746	2.787574	3.391555	1.740938	1.561284	1.137975	1.693305	2.315853	1.711703	0.492982	1.302121	0.952441	0	0	3.21461
LOC107759948	0	0	0	0	0	0	0	0	0	0	0	0	0	0	0	0	0	0	0	0	0	0	0	0	0	0	0	0	0	0
LOC107788121	0	0.059155	0	0.058802	0	0	0.544401	0.388207	0.182812	0.126585	0.273076	0	0.578041	0	0.666185	0.048369	0	0.096782	0.065863	0.109813	0.059196	0.975696	0.858781	0.339203	0.164124	0.361252	0	0	2.499874	28.09298
LOC107828690	6.895901	9.609392	8.670371	10.39759	9.104662	9.042658	10.25649	9.557746	8.43122	6.038937	7.622782	5.969191	6.88062	6.465361	9.826772	9.067467	7.423059	8.059646	7.993611	8.601531	6.808671	9.655598	8.209961	7.156925	5.546753	9.510827	3.165621	3.466815	5.484598	20.23263
LOC107802018	0.432735	0.205766	0.425233	0.272721	0.249726	0.525927	0.047342	0.080378	0.194303	0.073386	0.171506	0.015895	0.041889	0.102629	0.019311	0.22433	0.163588	0.140271	0.190917	0.222822	0.085796	0.172838	0.182553	0	0.023787	0	0.088949	0	0.09058	0
LOC107761947	0	0	0	0	0	0	0	0	0	0	0	0	0	0	0	0	0	0	0	0	0	0	0	0	0	0	0	0	0	0
LOC107769808	0	0	0	0	0	0	0	0	0	0	0	0	0	0	0	0	0	0	0	0	0	0	0	0	0	0	0	0	0	0
LOC107784800	0	0	0	0	0	0	0	0	0	0	0	0	0	0	0	0	0	0	0.052887	0	0	0.043526	0	0	0	0.21756	0	0	0	0
LOC107822960	0.141786	0.184601	0.246503	1.449659	14.10546	0.653302	7.322194	11.47423	3.365904	30.37751	15.42436	39.99097	136.4848	62.24823	122.3867	0.150941	0.195682	0.166112	6.083837	5.157468	4.858404	7.916495	1.143447	0.719802	13.41893	9.836053	1.173057	27.52203	85.03343	41.32937
LOC107778803	0	0	0	0	0	0	0	0	0	0	0	0	0	0	0	0	0	0	0	0	0	0	0	0	0	0	0	0	0	0
LOC107782408	4.535566	4.572576	5.47787	1.672687	1.74031	3.365933	1.077288	1.131728	1.921837	0.587091	1.491665	0.983375	1.563867	1.926686	1.1123	3.589284	3.683762	3.620852	1.303328	1.25629	1.793709	1.743044	2.47829	2.76883	0.304478	0.167546	0.711594	0	0	0
LOC107780568	8.344755	9.312509	10.82801	6.435855	5.274278	6.722185	5.468689	5.467015	5.870108	3.250131	4.213653	4.151825	4.170795	4.671295	4.669454	8.901732	9.495455	8.742566	3.925252	4.177828	4.326702	5.099708	6.158891	5.390818	1.784017	2.606578	2.214105	1.739848	1.991162	9.025693
LOC107767351	0	0.136082	0	0.027054	0.026425	0	0.075142	0.076547	0.028037	0.05824	0.08376	0.075687	0	0	0.061301	0.044508	0	0	0	0.025262	0.054471	0.074818	0.079024	0.062426	0	0	0	0	0.143771	0
LOC107771013	0	0	0	0	0	0	0	0	0	0	0	0	0	0	0	0	0	0	0	0	0	0	0	0	0	0	0	0	0	0
LOC107782305	8.131154	9.090842	10.12977	81.43742	66.11434	61.16848	45.69763	36.71662	44.42744	12.03165	14.25393	9.605214	8.288634	7.426624	7.132572	11.20274	7.490012	11.06689	63.48902	56.02694	41.39023	34.90021	58.17001	33.13003	5.767699	3.749376	7.487149	8.451926	4.438095	2.923032
LOC107829343	324.7608	254.209	243.9443	187.3326	182.4437	190.0619	160.657	144.0811	135.4716	348.1761	413.4851	389.624	444.1923	374.422	468.827	337.3439	376.5746	338.0381	160.1568	170.9544	183.3623	136.8042	126.4736	132.6569	199.03	155.2698	216.6431	265.6343	312.9453	116.7808
107767934	0	0.653941	0	0	0	0	0.601824	0	2.020949	0	0.503133	0	0	0	0	0	0	1.386391	0	0	0.606982	0	0	1.26582	0.749963	0	0	0	0	0
LOC107801280	9.091556	9.509876	9.384928	8.610875	9.096117	13.93982	8.318705	7.768072	7.614102	10.17508	9.091626	8.728181	12.59355	12.45441	8.536041	8.776754	9.431967	11.20704	9.749658	9.307653	8.856992	6.90236	8.338305	11.06824	10.05768	6.828253	11.17287	14.7767	17.9059	0
LOC107820100	2.84316	3.687463	2.376441	6.082754	7.295864	4.914305	8.052884	7.1757	5.07164	6.419266	6.502287	5.543033	7.328298	7.515774	8.956451	2.819536	2.831247	3.40782	10.23081	9.58347	7.699245	8.401714	7.25349	5.188994	14.59968	18.77602	8.762855	21.89666	21.5849	41.9208
LOC107825114	2.506622	2.599577	3.606544	0.234918	0.229451	0	13.12195	16.9861	70.11293	1.938562	3.272856	2.409756	73.99389	137.8616	36.46177	3.542641	1.503067	3.995351	0.175417	0.292473	0.236491	9.672701	0.991141	2.619921	0.327841	0.120268	0.204319	0	0	0

LOC107790129	1.115331	1.52473	1.052299	2.107124	2.154371	1.784053	2.886287	2.196478	0.944964	1.724226	3.395332	0.896299	1.741271	1.736125	2.498895	2.158953	1.314029	1.805021	2.746591	3.083613	1.488587	2.385401	1.763634	1.634885	2.218356	2.725291	1.109244	0	3.863494	1.682015
LOC107827847	154.43	290.8034	134.9207	156.7524	213.6198	69.51064	179.1068	185.5776	132.1906	123.6493	156.903	127.3802	148.9735	107.973	204.2587	448.7012	338.0424	371.9058	359.8547	263.4273	403.9589	210.2509	183.3251	138.4987	170.1789	183.406	94.65815	117.4261	197.4315	98.98908
LOC107822258	0.09234	0.042078	0.040135	0.020914	0.020427	0.099277	0.058087	0.078898	0.021673	0.022511	0	0	0.025698	0.050369	0.023694	0.137623	0	0.017211	0.0937	0.078113	0.042108	0	0.040725	0	0.029186	0.064242	0.027285	0	0.222278	1.427377
LOC107803284	19.45866	16.42966	17.21765	45.91135	68.73627	36.80832	46.95073	49.8119	37.99006	46.85191	39.46846	50.88639	68.25787	70.4379	66.70512	21.36174	20.29362	22.20792	80.98321	68.52619	75.7495	54.88963	36.47739	31.45747	102.8085	102.2187	55.5532	260.0546	110.8154	239.1964
LOC107808724	6.847028	6.162219	6.993572	7.288596	5.149895	9.56981	5.742897	6.215929	5.865816	5.174455	5.761368	7.086085	5.240267	7.189654	3.425956	5.803995	7.02823	7.465696	5.471464	3.982079	4.683453	3.931216	5.964037	6.172491	2.380599	2.619963	4.046318	0	4.120504	5.292025
LOC107799476	0.036784	0	0.031975	0	0	0.039547	0.061704	0.031429	0	0.071737	0.077378	0	0	0	0.075507	0	0	0	0	0	0	0.061438	0	0	0	0	0	0	0	0
LOC107814483	0.01739	0	0.015117	0	0	0	0	0	0	0	0	0	0.019359	0	0	0.025919	0.016801	0	0	0	0	0	0	0	0	0	0.020554	0	0.083723	0
LOC107827004	0.392271	0.483686	0.441285	0.815279	1.061741	0.744246	0.909629	0.867492	0.866545	5.760212	0.922263	1.24763	2.132035	2.240438	1.34995	0.584642	0.20063	0.48169	1.615614	2.108125	2.272816	0.963518	0.915907	1.109417	37.16709	4.302322	23.61802	13.20139	157.8592	4.280246
LOC107767367	6.486152	8.494428	21.35674	20.14671	22.96955	15.67551	14.74344	17.41754	17.16825	28.10292	17.48532	15.08063	17.53544	18.22535	24.4051	32.96114	6.661094	18.90597	44.41264	46.83408	28.88146	17.21754	15.09631	17.53224	35.88566	47.93113	21.03348	39.9536	37.13084	94.36436
LOC107802401	171.7119	144.0954	183.3706	75.24228	66.68313	87.97794	64.48944	68.82993	96.29586	31.74915	38.19358	42.0565	26.26214	32.96327	27.97881	118.7331	158.4551	140.8583	38.31241	34.91326	52.09294	72.76434	108.1352	127.7082	7.923682	5.459106	21.61985	3.643865	4.170206	15.75253
LOC107830420	0	0.031868	0.091187	0	0	0.150373	0.058656	0.029876	0	0	0	0	0	0	0	0.156342	0.472928	0.130345	0.070963	0.207055	0.19134	0.029201	0.061685	0.146188	0.044208	0	0.206638	0	0	0
LOC107768344	5.620197	7.173134	6.572678	5.458383	4.846685	4.647015	5.502881	4.761354	4.963477	3.796804	5.427067	3.108395	4.148896	4.325726	5.951735	7.151846	5.62514	6.382794	3.806019	4.220449	2.4977	6.54316	5.398509	4.343444	3.748525	3.661527	1.688802	1.703066	4.356737	3.6812
LOC107815001	0	0	0.168639	0	0.343326	0	0	0	0	0.189174	0	0	0	0	0.199115	0	0.749678	0.144635	0	0.16411	0	0	0	0.202768	0	0	0	0	0	0
LOC107829482	0	0	0	0	0	0	0	0	0	0	0	0	0	0	0	0	0	0	0	0	0	0	0	0	0	0	0	0	0	0
LOC107801539	0	0	0	0	0	0	0	0	0	0	0	0	0	0	0	0	0	0	0	0	0	0	0	0	0	0	0	0	0	0
LOC107827376	0.916799	0.668439	0.850084	0.830573	0.811244	0.788537	0.410111	0.20889	1.032877	0.297998	0.385716	0.464722	0.340198	0.333394	0.313659	0.501013	0.826658	0.820215	0.806263	0.672142	0.390197	0.3573	0.916501	0.76659	0	0.340176	0.361194	0	0	3.779136
LOC107811346	5.714622	6.889736	6.215068	3.180586	2.358269	3.939821	2.557753	2.627477	3.440425	2.47388	2.318006	2.381461	3.508863	2.90751	1.394002	6.817488	6.684383	6.744189	2.106292	2.753096	2.687716	2.503921	3.413192	3.642678	0.323992	0.534853	0.484608	1.832629	1.603852	0
LOC107767921	5.616296	5.793927	3.763306	11.73085	9.835802	4.86411	17.04332	18.16181	5.602378	11.18139	20.04628	3.262052	4.385181	3.701791	14.97146	9.474987	5.538852	5.466605	5.976065	8.347243	11.59599	13.25666	15.61869	7.623043	4.092768	6.729296	2.120475	11.15679	1.314388	4.823112
LOC107765443	13.91733	11.06961	13.12454	13.29612	12.42692	15.50693	10.04591	10.66617	10.9281	11.55604	9.403972	9.798121	9.014088	12.46856	9.133336	11.81689	12.06003	12.95423	12.96688	12.20121	12.23128	10.00259	10.788	10.93183	4.798836	4.166398	9.271346	0	8.121572	2.607665
LOC107774882	1.382285	0.455666	0.409052	0.079933	0.078073	0.158098	0.024668	0.201032	0.193282	0.057358	0.123735	0	0.13096	0.224596	0.030186	0.482165	0.284128	0.26312	0	0.024879	0.053645	0.442104	0.025942	0	0	0	0	0	0	0
LOC107797411	13.63056	17.79222	14.80292	14.22264	12.86203	12.58938	20.32451	15.4653	15.39798	7.81642	8.146109	5.960584	14.08402	16.26058	14.89995	15.63541	10.56316	14.12788	12.08837	10.53058	11.01635	16.30363	9.709675	8.35881	9.293769	10.94781	4.016641	5.283348	10.22571	12.56203
LOC107785507	0	0.046619	0	0.046341	0.090526	0	0.042903	0	0	0	0.071736	0	0	0	0	0	0	0	0	0	0	0	0	0	0	0	0	0	0	0
LOC107810533	9.658679	7.109869	7.185111	6.057882	5.423826	8.786683	4.829532	5.078546	5.929073	4.618654	5.144626	4.943029	4.445612	5.167254	5.242666	5.536661	5.742213	4.985277	6.69104	6.04937	3.218629	4.498466	4.669393	6.891949	3.757387	2.972163	5.159055	0	7.601014	0
LOC107774944	0.642259	1.235717	0.682369	1.422309	2.462688	1.764628	1.975197	2.317	2.009936	2.783481	2.351839	1.80866	2.383238	1.86846	2.783275	2.552597	1.861428	2.287749	2.389639	3.320207	2.147753	2.979817	1.888382	0.745876	2.977371	3.574624	1.855573	0	1.717812	4.412424
LOC107829943	3.366513	3.333908	3.076217	3.494166	2.75608	3.73341	2.934806	2.457431	3.173074	1.602615	2.871755	1.881153	2.286951	2.327971	2.244582	3.575433	3.034659	3.498019	1.909791	2.410572	1.220874	3.564138	3.27345	3.643356	0.418927	0.497933	0.563945	0	0.574285	0.819514
LOC107798172	0	0	0	0	0	0	0	0	0	0	0	0	0	0	0	0	0	0	0	0	0	0	0	0	0	0	0	0	0	0
LOC107782208	5.801199	6.647346	5.801736	7.806851	8.746516	9.991309	6.164815	5.822922	5.922322	7.057397	8.688515	7.161191	10.56475	8.817341	5.925288	6.610505	6.175773	7.474342	8.315616	8.576066	6.652015	5.315096	5.663522	6.504923	9.150297	6.62213	7.95493	12.08353	13.28663	13.92998
Novel00863	0	0	0	0	0	0	0	0	0.061317	0	0	0	0	0	0	0	0.048692	0	0	0	0	0	0.057609	0	0.082573	0.090876	0	0	0	0
LOC107802691	2.446719	2.457422	2.257098	1.176157	0.972049	1.55684	1.088906	0.767955	1.218863	0.681674	0.630235	0.506218	1.222896	0.381321	0.25625	2.716342	2.894371	2.270859	1.064041	1.520637	0.500938	1.209311	1.321327	1.46132	0.315653	0	0.590168	0	0.721186	0
LOC107763335	4.377559	4.058392	3.674102	8.957344	9.41674	9.331678	8.419468	8.83486	7.581956	11.33406	8.996949	14.41055	11.84682	10.3748	6.197251	3.993337	4.08326	5.683263	9.573372	8.810857	11.15121	5.735846	8.188612	12.14854	10.78286	5.986036	12.22116	5.397541	27.61569	9.333486
LOC107772014	0.39403	1.197033	0.639375	9.781019	21.52419	4.998843	15.8415	24.17292	21.06152	17.2647	18.64065	33.64386	19.41713	26.99569	43.78541	1.859668	0.558311	0.783377	11.08875	12.95514	11.78708	25.99613	16.9378	11.69627	45.06815	95.32491	16.08259	39.45023	38.95178	29.23618
LOC107800510	244.7554	219.2665	252.8826	152.5391	136.1533	194.691	128.0831	126.5571	168.8242	83.53359	98.57205	95.94591	83.73604	88.16135	72.60192	179.3259	243.9454	209.0603	90.45902	89.7349	106.8959	130.1298	188.0666	209.5063	37.94705	16.73438	66.77158	52.8122	23.10968	16.30775
LOC107817622	0	0	0	0	0	0	0	0	0	0	0	0	0	0	0	0	0	0	0	0	0	0	0	0	0	0	0	0	0	0
LOC107797074	0.374364	0.483348	0.366105	0.113052	0.013803	0.184473	0.013083	0.039983	0.131801	0.030421	0.021875	0.052712	0.052093	0.11912	0.04803	0.395217	0.57264	0.476803	0.09497	0.052781	0.085357	0.104214	0.220143	0.048911	0.039442	0.021704	0	0	0	0
LOC107810780	3.214015	2.968762	2.265304	2.714996	2.190627	2.428161	1.275009	1.001971	1.182518	1.185865	0.822285	0.917327	0.918647	1.231955	0.802405	2.492186	3.146969	2.881898	1.895115	2.424908	1.505229	1.196967	1.915523	2.360581	0.878593	0.060433	1.437349	3.106048	0.2091	5.371011
LOC107792593	0.671535	0.688524	0.802658	0.380236	0.371387	1.082974	0	0	0.157617	0	0.235441	0.141833	0.186891	0.27473	0	0.437875	0.729855	0.438076	0.340714	0.142018	0.306226	0.140204	0.222127	0.263208	0.318384	0	0.099213	0	0	0
LOC107832707	397.3691	295.2349	357.4175	179.4332	173.1193	289.283	144.4172	148.5829	228.3923	127.8442	145.0566	181.7488	107.4221	134.4409	96.08206	227.9275	367.2501	309.1564	118.4832	114.5985	149.1429	133.5745	205.8483	264.2047	50.16069	20.93206	117.2764	94.76219	19.51353	48.19523
LOC107769343	0	0	0	0	0	0	0	0	0	0	0	0	0	0	0	0	0	0	0	0	0	0	0	0	0	0	0	0	0	0
LOC107811225	0	0.024356	0.046462	0.072633	0	0	0.04483	0	0.02509	0.052119	0	0.112887	0.327249	2.274088	2.386337	0	0.025818	0.039848	0	0	0	0	0.023573	0.083797	0.033788	0.037185	0	0	0	0
LOC107795137	0.40657	0.802833	0.706845	1.043607	0.53964	0.36426	2.159716	1.273743	0.954264	0.792914	1.663008	1.259432	2.56473	2.439513	2.295107	0.757441	0.392781	0.909345	0.412559	1.146434	0.370799	1.924044	1.733338	1.204016	0.171343	0.188571	0.240266	0	0	0
LOC107764854	0	0	0	0	0	0	0	0	0	0	0	0	0	0	0	0	0	0	0	0	0	0	0	0	0	0	0	0	0	0
LOC107776723	0	0.133719	0.191314	0.46523	0.389488	0.473232	0.246124	0.188045	0.206624	0.143072	0.565849	0	0.163333	0.320133	0.602366	0.382681	0.354365	0.328162	0	0.310292	0.669065	0.673923	0.258837	0.230031	0.185501	0.102076	0	0	0	0
LOC107794198	1.814752	2.201265	1.611573	3.264492	3.23473	2.877493	2.387196	1.874075	1.863115	1.884192	2.334453	1.279466	2.194614	1.751914	3.09542	2.305815	1.967624	1.644984	1.934222	2.518097	1.107359	2.769417	1.946213	1.9923	0.709778	0.853809	0.509219	0	0.817117	3.229032
LOC107813179	3.442099	6.956064	6.634753	8.541614	8.342831	3.861576	10.04187	8.695197	4.355626	5.107688	8.080319	2.781547	7.663584	5.224571	11.98106	12.26766	10.69897	7.810267	8.048635	11.39392	4.913612	14.6229	10.16453	3.910522	6.622394	10.62004	3.71452	0	4.322997	0
LOC107816124	0.755477	1.587431	1.805981	1.59699	1.355561	0.586611	4.083531	5.612267	3.506973	3.028597	5.591712	2.748013	2.406217	3.640166	13.09567	6.052063	1.256972	2.784911	1.618391	2.236786	1.645964	6.046306	3.461479	2.01793	2.069498	4.350765	1.140945	3.001517	2.323726	7.785384
LOC107799064	0	0	0.03123	0.130188	0.095368	0.154498	0.060265	0.061392	0	0	0.050382	0.030351	0	0.11758	0	0	0	0.026784	0.036455	0.091172	0.032765	0.090008	0.031689	0.150198	0.045421	0	0	0	0.172959	0
LOC107828021	0	0	0	0	0	0	0	0	0	0	0	0	0	0	0	0	0	0	0	0	0	0	0	0	0	0	0	0	0	0
LOC107774727	0	0	0	0	0	0	0	0	0	0	0	0	0	0	0	0	0	0.055194	0	0.14882	0	0	0	0	0	0	0	0	0	0
LOC107798828	0.420159	0.382923	0.121745	0.380642	0.991424	0.903444	1.879494	1.675309	1.577853	0.682846	0.785641	2.12977	7.015895	3.361397	1.293718	0.104368	0.541211	0.730907	0.284232	0.4739	0.127731	0.701771	0.864751	2.195748	0	0.389747	0	0	1.348528	0
LOC107802307	3.013571	2.944088	2.751557	1.964131	1.400448	1.678251	2.745823	2.222915	3.54168	1.183901	1.702656	1.392029	1.568767	1.088002	1.268372	1.922592	2.848528	2.505363	0.989988	0.696924	0.889779	2.408077	3.461356	3.036482	0.356337	0.181	0.486864	1.550453	0.104377	0
LOC107767028	52.35663	76.41405	46.31697	14.37855	22.43753	10.42219	24.39222	22.6541	18.46288	34.83335	24.09106	24.921	11.049	9.263142	9.807136	46.8436	56.95101	38.32688	17.86581	18.4167	21.54347	23.41757	22.89713	16.17754	47.32789	28.13578	56.49439	9.92728	11.584	20.02742
LOC107799309	0	0	0	0	0	0	0	0	0	0	0	0	0	0	0	0	0	0	0.031923	0.053225	0.028691	0.052545	0	0	0	0	0.037182	0	0	0
107804790	0	0	0	0	0	0	0	0	0	0	0	0	0	0	0	0	0	0	0	0	0	0	0	0	0	0	0	0	0	0
LOC107810125	0	0.081743	0	0	0	0	0	0	0	0	0	0	0	0	0	0	0	0	0	0	0	0	0	0	0	0	0	0	0	0
LOC107801932	6.829561	5.791308	4.33644	5.326403	4.834596	3.320138	9.46406	6.545754	5.018003	6.254312	7.495657	6.773247	9.916646	10.10706	13.47076	3.496194	3.385025	2.435174	3.495214	2.662599	3.249743	4.959604	5.866973	3.413948	1.501677	1.2395	1.544207	8.49409	4.288687	3.672018
LOC107768645	5.09208	4.692946	5.570347	5.934916	3.442639	6.366539	8.325946	7.0884	8.755606	3.961178	6.158229	4.446939	5.350115	6.678734	4.903393	4.753939	5.25102	5.118689	3.599546	3.533158	3.130828	10.48798	7.973764	8.880367	1.265886	1.313559	3.178271	2.045818	1.928153	1.768826
LOC107795652	0	0	0	0	0	0	0	0	0	0	0	0	0	0	0	0	0	0	0	0	0	0	0	0	0	0	0	0	0	0
LOC107780958	2.502911	2.776985	2.648712	3.680597	3.370257	4.445893	4.502845	3.254323	2.21362	1.556359	2.848765	1.716139	5.168743	4.234336	3.723071	2.135487	3.153942	2.839605	1.288295	1.442212	1.587963	3.059636	2.687675	2.464387	0.045861	0.100945	0.557349	0	0.873182	0
LOC107807962	92.85714	106.2807	98.67413	18.96458	13.56458	24.79637	13.09061	15.43265	17.47915	4.606403	5.260878	3.52137	3.144918	4.041998	1.093286	65.60796	67.82894	79.55285	14.28648	16.06275	11.91113	23.16753	31.04671	15.68358	2.810565	2.126548	4.160087	0	1.78373	0
LOC107777519	1.38192	2.230592	1.027596	1.070945	0.618773	0.930821	0.949604	1.166524	0.609625	0.340945	1.144124	0.337586	0.537504	0.254295	1.144945	2.766835	1.335051	2.110212	0.675796	0.746476	0.258141	0.875987	0.910537	0.417653	0.063151	0.162167	0.098393	0	0	1.029472
LOC107767052	0	0	0	0.135195	2.508923	0.481322	0.750994	2.0401	0.700519	0.436555	0.20928	0.882516	0.33225	1.302422	1.837988	0	0	0	0.757142	0.378715	0.408301	1.370885	1.974462	0.31195	1.509378	2.076428	0.176378	0	5.029127	27.68136
LOC107812719	0	0	0.011747	0.048972	0	0.014529	0	0	0.025375	0	0.009476	0.011417	0.015044	0.029486	0.110962	0.030212	0	0	0	0.011432	0	0.045143	0	0.028249	0	0	0	0	0	0
LOC107800753	0.703547	0.616051	0.719504	0.412421	0.41503	0.786062	0.358685	0.271098	0.453299	0.49772	0.40627	0.128199	0.29178	0.361193	0.212383	1.459771	0.666358	1.069618	0.895888	0.758527	0.390021	0.58755	0.900442	1.11023	0.540675	0.47987	0.375006	0	0.863385	0
LOC107817431	0	0	0.130466	0.407909	0	0.16136	0.377649	0	0	0.292704	0.10524	0.633981	0.501231	0.163736	0	0.111844	0	0	0.152296	0.126962	0	0	0	0	0.56926	0.417665	0.177389	0	0.722564	0
LOC107783075	0.113869	0.41511	0.296952	0.10316	0.134345	0.081615	0.031836	0	0	0.037012	0.079845	0	0	0	0.155829	0.395992	0.183345	0.226385	0.038515	0.064217	0.069234	0.031698	0.10044	0	0	0	0.044861	0	0.182735	0
LOC107802868	15.03234	18.05693	16.24889	18.35542	17.05249	21.66257	17.57326	16.60949	16.39214	14.58559	18.08284	14.08803	18.83378	16.01247	17.51789	17.96513	16.36262	18.36318	15.00744	17.61329	10.0822	17.18558	15.59846	16.86334	7.578569	8.756307	8.475197	5.341738	6.113329	1.154624
LOC107831550	0	0	0	0	0	0.331913	0	0.175853	0.386455	0	0.216476	0.173878	0.458231	0.112267	0.211242	0	0	0	0	0	0	0	0	0	0	0	0	0	0	0
LOC107769913	0	0	0	0	0	0	0	0	0	0	0	0	0	0	0	0	0	0	0	0	0	0	0	0	0	0	0	0	0	0
LOC107776396	1.770584	1.120603	2.137681	0.401015	0.348162	1.639206	0.082504	0	0	0.191838	0.103461	0	0	0.429249	0.05048	0.879627	1.330414	1.650056	0.19963	0.457659	0.358845	0.123222	0.130148	0.205624	0.062182	0	0.11626	0	0	0
LOC107798695	26.2203	23.12035	26.17729	35.54872	31.43769	35.97335	24.95159	29.78448	28.7862	24.08503	22.43822	31.30011	30.94961	27.14494	24.97614	18.6327	22.03974	21.90685	27.40907	25.88591	33.67844	20.62711	24.09394	31.41135	22.38231	17.353	27.68274	60.90884	23.43088	41.37737
LOC107788624	3.370641	3.594151	4.189938	5.069026	3.847509	3.696328	4.127569	3.599959	3.702472	3.155329	3.497973	3.559514	2.288869	1.691514	2.940604	3.918417	4.168093	5.478244	4.377976	4.334022	3.074081	5.65797	5.55975	6.975526	1.4063	2.0636	2.390296	0	1.785021	0
LOC107774091	6.71862	6.065154	5.53588	13.26965	13.8061	16.97998	8.359319	7.100896	6.367525	18.25727	21.16646	19.90662	18.39686	15.59729	16.07932	8.020249	7.382846	7.050606	15.7677	17.29289	14.63629	5.052465	5.252189	5.225123	18.35752	11.65236	14.60217	4.554278	33.41891	7.875306
LOC107793777	0	0	0	0	0	0	0	0	0	0	0	0	0	0	0	0	0	0	0	0	0	0	0	0	0	0	0	0	0	0
LOC107772655	4.127486	4.7469	5.296477	0.267092	0.086959	1.162216	0.247279	0.419838	0.092263	0	0	0	0.328198	0.107212	0	3.734909	4.36726	4.762358	0.89749	0.083133	0.358508	0.656566	1.126888	0.410862	0	0	0	0	0	0
LOC107785646	8.370616	9.848067	10.03261	4.710871	4.444123	4.472447	5.127308	5.201514	5.286764	2.621864	3.343825	2.164378	2.371927	2.020094	3.254307	8.449374	6.469375	9.171863	4.144005	3.540482	2.243975	6.99052	7.629551	7.555924	2.437298	0.988239	1.648899	0	1.953897	3.136774
LOC107805802	0.26779	0.146435	0.093114	0.339646	0.047392	0.115163	0.044921	0.411853	0.201131	0	0.15022	0.090495	0	0	0.329823	0.199558	0.155225	0.199649	0.271735	0.135919	0.097692	0.447277	0.236209	0.391852	0.270855	0	0	0	0	0
LOC107817500	1.165614	1.770522	1.794285	1.869977	2.277701	2.114736	2.097875	2.033361	1.823882	0.852465	1.753852	1.415568	1.784169	1.748487	1.271126	1.284829	0.938401	1.611298	1.453839	1.663924	1.461706	2.230787	2.356173	1.954355	1.105267	1.081244	0.373117	3.473244	1.05219	1.501493
LOC107825480	0.453683	0.263121	0.179262	0.373649	0.401449	0.266053	0.553489	0.704798	0.890597	0.764142	1.012209	0.8711	0.459133	0.989892	0.338653	0.49176	0.478141	0.67648	0.29296	0.558232	0.677072	0.482214	0.582078	1.163919	0.417159	0.459103	0.828699	0	0.595689	7.650519
LOC107803612	2.608298	3.000655	3.531101	6.469159	5.221363	2.02273	3.29947	4.201461	2.007201	5.54549	8.035345	1.986819	3.379593	8.44328	10.00616	1.688795	1.69366	1.498296	22.17171	21.77504	25.97195	4.749317	6.939791	4.78201	37.46383	59.85299	7.42904	30.57872	27.58484	148.0558
LOC107800692	38.40312	34.76161	44.79456	27.39517	27.39333	41.84979	24.596	28.45996	36.05454	21.78091	22.44024	23.8916	23.4839	25.57931	17.2277	27.69331	49.59371	34.91248	19.48441	22.98357	27.10213	23.88995	36.46649	40.41198	11.80801	8.087971	16.59646	9.34138	11.94843	0
LOC107767131	7.868578	9.076513	8.707734	14.04663	14.38773	12.14324	12.60278	12.66478	8.886649	16.52071	16.51164	14.11694	11.47451	12.13196	15.13895	11.3487	7.321202	10.63954	17.16223	18.69161	12.71075	14.18518	11.87074	10.3941	11.28817	17.37224	8.647995	6.229313	11.3926	12.56709
LOC107812511	26.84137	6.175025	13.47856	1.652606	1.671794	2.591595	1.366081	1.781281	3.853377	1.524683	1.918665	2.036467	0.507673	1.208263	1.003007	2.815847	3.461665	4.274276	0.198326	0.992008	0.713003	4.51583	4.827118	4.766557	0.082368	0	0	0	0	0
LOC107811179	0.552416	0.615339	0.360153	0.910561	0.95726	0.800135	0.424724	0.727665	0.576258	0.478822	0.607931	0.388914	0.683286	0.996061	0.582734	0.457401	0.385436	0.42901	1.790657	1.525234	0.881672	0.826562	0.636155	0.408981	1.707253	1.601346	0.725457	1.097376	0.960385	1.897595
LOC107769064	0	0	0	0	0	0	0	0	0	0	0	0	0	0	0	0	0	0	0	0	0	0	0	0	0	0	0	0	0	0
Novel03350	1.962989	1.898555	1.427791	2.703846	3.385342	2.131989	1.965652	1.93395	2.256662	1.582114	2.275355	1.421475	1.51628	1.070762	1.069055	2.059892	2.012519	2.060835	4.329346	3.609163	4.45743	2.525927	2.243865	3.60097	0.633116	0.864	0.757582	1.432464	2.314415	2.477032
LOC107818692	0	0.025916	0	0.025762	0.025162	0.061145	0	0	0	0	0.01994	0	0	0	0	0.021191	0	0	0	0	0	0	0	0.029722	0	0	0	0	0	0
LOC107822524	0	0	0	0	0	0	0	0	0	0	0	0	0	0	0	0	0	0	0	0	0	0	0	0	0	0	0	0	0	0
LOC107824346	0	0	0	0	0	0	0	0	0	0	0	0	0	0	0	0	0	0	0	0	0	0	0	0	0	0	0	0	0	0
LOC107814230	8.494945	7.554028	7.504062	4.611362	3.651928	6.101062	4.500048	4.025865	4.617353	2.951258	3.665637	3.021796	3.752048	3.114203	4.094743	6.919917	7.176824	7.589754	2.163746	2.647525	2.728888	3.848757	5.55157	6.290729	0.652238	0.478546	0.691036	0	0.993466	0
LOC107782751	10.06665	11.7958	9.015169	13.70487	11.35479	11.80407	15.88924	13.32716	13.65723	10.89496	11.51893	11.33166	12.56095	11.40461	9.480583	7.728378	8.389346	7.010271	10.15881	11.18269	9.25662	11.50178	12.51417	12.16928	2.097907	3.809598	4.216588	3.955537	4.793186	0
LOC107814247	10.3085	7.153501	7.118736	19.03351	24.86458	14.12086	17.53376	19.35942	16.89979	26.91614	22.67567	31.05589	24.64044	30.71262	17.75031	7.779396	9.251144	8.933822	29.97397	26.69204	33.66893	14.0714	17.12397	19.82607	27.02545	29.99764	24.86242	16.83968	33.63171	24.26613
LOC107779487	2.320125	3.906463	2.90561	3.241937	2.748931	2.959479	2.935474	2.822368	1.845962	2.684221	4.329136	1.295663	1.400838	2.230837	5.650571	3.516528	2.355407	3.664727	2.553819	4.091659	1.183521	4.466324	2.983039	2.055079	2.038425	6.018835	1.394339	0	2.082525	2.431471
LOC107807922	2.970611	1.768063	1.791793	3.899528	4.452514	3.584845	4.678068	4.35115	3.870367	3.606122	5.653842	2.458438	3.644368	3.637623	4.977884	3.117255	2.167037	2.847493	3.87562	4.000179	4.146808	6.784325	5.400977	4.625641	3.60246	6.74839	4.155906	13.00657	1.751214	7.497036
LOC107773287	4.690725	30.01418	7.305606	6.905534	3.891246	1.470905	1.967157	1.836946	0.366988	4.288171	10.21004	0.495357	6.418438	4.477682	6.419242	19.44391	12.17877	16.02853	0.99163	1.818681	3.030264	1.469005	8.361258	0.102141	0.247104	0.135975	1.270514	0	0.940951	0
LOC107830186	0.209799	0	0	0.190067	0.618813	0	1.055804	1.553567	3.085837	5.114508	4.854664	2.835901	6.928677	20.52292	37.8266	3.908557	0	0	0.141926	0.177475	0	1.752084	0	0.146188	3.801909	6.908766	9.587991	0	1.010046	0
Novel05133	0	0	0	0.059765	0	0	0	0	0	0	0	0	0	0	0	0	0	0	0	0	0	0	0	0	0	0	0	0	0	0
LOC107771112	6.038013	5.273617	6.25472	3.555587	3.450578	3.813816	2.489968	2.128105	2.33836	1.422891	1.340722	1.487815	1.820433	1.262542	0.95541	4.705763	4.520752	5.739533	1.735972	2.468743	2.225653	1.428709	3.106791	3.339533	0.636157	0.700121	1.189408	0	0.605607	0
LOC107795671	0.012658	0.057682	0.011004	0	0.022402	0	0.010617	0.010816	0	0.012343	0.008876	0.021388	0	0.01381	0.012992	0.028299	0	0.018874	0	0.010708	0.011545	0.063427	0.033496	0.01323	0	0.017613	0	0	0	0
Novel04284	0.477627	0.435298	1.037978	0.072118	0.070439	0.085585	0.066768	0.136032	0	0	0	0	0	0.086845	0	0.237285	0.230714	0.178046	0	0	0	0	0.140433	0.33281	0	0	0	0	0	0
LOC107807433	1.357575	2.666005	1.980901	2.240164	2.445446	1.563813	3.348192	2.747974	1.820787	1.906912	2.91246	1.160571	1.90708	1.957098	3.649322	2.80617	1.514507	2.01221	3.903124	3.964766	2.726836	3.941118	2.737076	1.486505	3.432775	3.125789	1.833765	1.155783	1.633962	2.997889
LOC107773549	6.278962	7.830789	9.603093	6.501053	7.582113	5.887787	9.562739	12.40385	10.06133	7.803093	11.53669	7.598002	8.119763	8.13764	10.9509	13.65028	6.157223	10.31279	10.58716	14.09763	8.503665	13.10931	13.72122	11.69456	9.490407	21.34912	7.365429	18.5691	19.77396	46.70528
LOC107813453	0	0	0	0	0	0	0	0	0	0	0	0	0	0	0	0	0	0	0	0	0	0	0	0	0	0	0	0	0	0
LOC107805067	0	0	0	0	0	0	0	0	0	0	0	0	0	0	0	0	0	0	0	0	0	0	0	0	0	0	0	0	0	0
LOC107771682	1.710502	1.86115	1.972423	1.470566	1.791566	2.345662	1.537148	1.491326	1.442028	1.378616	1.860302	1.666265	1.496122	1.6947	1.845184	1.742914	1.686213	1.912878	1.399186	1.816091	0.907351	1.938658	1.616546	1.38647	0.286875	0.461436	0.907691	1.248215	1.260454	2.158427
LOC107773582	0	0	0	0	0	0	0	0	0	0	0	0	0	0	0	0	0	0.100132	0	0	0	0	0	0.070189	0	0	0	0	0	0
LOC107799121	0.080214	0.14621	0.348642	0	0	0	0	0	0.075308	0	0	0	0	0.08751	0	0.119551	0.464961	0	0	0	0	0.066989	0	0	0	0	0	0	0	0
LOC107808572	0.810222	0.698503	0.628167	0.73402	0.910705	1.035888	0.679568	0.748406	0.966259	1.110367	1.289805	1.183997	1.438245	0.931692	0.899016	0.913827	0.782743	1.028526	1.111023	0.833585	0.878735	0.749788	0.791931	0.938395	0.968996	0.944549	1.061143	6.264036	1.686788	5.415919
LOC107764710	21.77018	21.47506	19.12194	22.90302	21.81132	26.36525	26.4997	23.21892	23.45009	25.83397	25.18237	23.59816	32.55682	29.17803	28.51363	20.00606	24.13048	22.68894	20.01505	20.29614	14.92563	21.78749	21.14083	19.90331	15.29473	17.25427	17.88008	12.64284	17.144	10.93107
LOC107792319	0	0	0	0	0	0	0	0	0	0	0	0	0	0	0	0	0	0	0	0	0	0	0	0	0	0	0	0	0	0
LOC107795344	0.279359	0.954754	0.18213	1.265424	1.915761	0.750862	1.757326	1.253131	0.91796	1.225845	2.154752	1.357058	1.321691	1.752408	2.222128	1.457249	0.944594	1.301711	2.196923	1.890546	1.464984	2.741272	2.217716	1.82491	2.207465	1.554829	2.393802	0	3.362331	17.27317
LOC107789553	0	0	0	0	0	0	0	0	0	0	0	0	0	0	0	0	0	0	0	0	0	0	0	0	0	0	0	0	0	0
LOC107802011	34.45388	31.17564	29.87855	33.67181	28.37697	42.61171	29.86352	29.22755	34.43122	26.30018	23.75547	33.48415	25.72226	32.29474	25.40362	25.67507	35.90686	29.61035	26.95116	28.38045	34.72226	23.96633	29.88288	28.61987	16.21811	14.75959	31.78043	82.32733	24.54449	10.16866
LOC107764549	2.115189	2.698827	1.203505	4.912578	3.028683	1.364452	6.451272	7.163379	2.852377	1.875069	3.667479	1.072182	0.513743	0.503469	2.526222	2.350032	1.448992	1.204226	2.497568	3.383411	2.630574	3.179609	6.139968	1.205888	1.312809	0.909695	0.31818	0	0	0
LOC107790481	10.80466	8.654364	11.79985	7.4999	5.359362	7.460628	6.228757	5.799126	10.20104	2.967861	2.603663	3.214111	12.67164	10.69162	4.185935	6.736164	9.526702	7.351907	4.200224	3.06383	5.662597	5.083522	4.563864	5.376116	1.269837	0.719933	1.187092	2.17658	3.07709	3.763765
Novel02766	10.47595	13.86974	22.52019	4.873593	3.57013	2.435228	7.124297	5.866493	12.69283	2.69189	3.226171	1.734195	1.575943	2.084974	1.162403	23.31458	11.00963	17.73142	2.729394	2.934019	1.872116	6.975348	8.55369	15.01852	2.058312	2.06829	1.254905	5.062018	2.385437	13.12994
LOC107831510	3.342096	1.993686	2.429815	3.303022	2.258307	4.899766	3.108959	2.180623	2.795412	1.718364	1.491305	1.540089	1.758767	2.717974	1.995774	1.630168	2.465588	2.083946	1.603173	2.827183	2.3276	1.928377	2.733553	2.540479	0.537781	0.338202	1.436396	0	1.755277	0
LOC107804591	0	0	0	0	0	0	0	0	0	0	0	0	0	0	0	0	0	0	0	0	0	0	0	0	0	0	0	0	0	0
LOC107817055	2.265286	2.788924	3.592862	6.912788	5.169432	3.375461	5.799977	4.58413	3.693829	7.750665	7.719162	4.331178	3.716259	3.208374	4.364524	4.027732	3.378669	3.288845	10.48509	12.33813	8.191424	8.529691	6.450109	6.230726	13.56636	12.995	10.00497	8.526313	8.03592	4.914597
LOC107792733	0	0	0	0	0	0	0	0	0	0	0	0	0	0	0	0	0	0	0	0	0	0	0	0	0	0	0	0	0	0
LOC107762933	0.990634	0.564275	0.753495	0.448732	0.328717	0.931921	0	0	0.465025	0	0.173658	0	0.137848	0.135092	0	0.184555	1.315924	0.4616	0.125653	0.314253	0.112934	0.310239	0.218451	0.258853	0	0.172299	0	0	0	0
LOC107829448	7.736731	5.240665	5.634775	5.777761	3.793037	5.620224	6.48913	6.878465	11.18985	6.320892	6.891217	8.83271	9.194572	7.18574	6.009231	5.220044	5.757256	5.378328	3.288807	3.095486	4.38618	4.976829	8.392055	8.960638	6.741359	3.927806	9.267764	7.476833	7.046801	12.92902
LOC107776083	14.71091	17.69567	14.94073	22.34685	27.65603	20.31588	23.45179	23.12855	21.83294	31.39477	29.05303	29.87786	32.06354	29.42295	27.0458	26.26223	21.00693	24.00318	34.12745	31.27865	33.99324	23.24725	24.3573	22.98611	37.94961	38.18153	34.68641	37.19327	55.20423	56.65845
LOC107827719	0	0	0	0	0	0	0	0	0	0	0	0	0	0	0	0	0	0	0	0	0	0	0	0	0	0	0	0	0	0
LOC107769268	69.65144	49.75357	81.41052	88.98047	95.36329	94.7685	26.09148	43.70921	35.87714	68.74499	73.52344	87.5893	54.48542	51.49612	49.41389	66.07281	59.65092	55.85779	140.6864	138.4229	174.9465	50.73956	38.77184	46.58194	247.0456	259.5747	252.8632	185.107	186.6955	93.11682
LOC107818151	6.875467	6.568852	4.099956	3.21973	3.732613	4.356618	3.705206	3.405557	3.336638	2.655868	3.563408	2.693837	5.287453	5.254181	3.681804	6.361192	7.604928	6.512683	4.179308	4.636079	2.423398	2.718379	2.695385	2.360696	0.713892	0.92432	1.57029	4.750658	1.758987	6.161166
LOC107772797	1.577543	1.998209	1.998879	2.034756	1.395913	2.98965	0.134558	0.251302	0.326337	0.469313	1.01243	0.067767	0.238121	0.262529	0.137216	0.976335	1.291558	1.116322	1.058144	0.791648	0.341397	0.357274	0.919803	0.586878	0.169025	0.037204	0.410828	0	0.257453	0
LOC107798548	0	0	0	0	0	0	0	0	0	0	0	0	0	0	0	0	0	0	0	0	0	0	0	0	0.506009	0	0	0	0	0
LOC107802066	0	0	0.021044	0	0	0	0	0	0	0	0	0	0	0	0	0	0	0	0	0	0	0	0	0	0	0	0	0	0	0
LOC107824260	10.28432	11.32315	12.38265	15.76958	13.33949	17.03175	13.12637	14.10864	12.47402	12.86265	14.57951	12.14228	13.33305	12.64828	15.8661	11.49583	12.68167	11.81065	14.8759	15.10311	13.6614	13.73659	14.25513	12.81888	12.71984	10.88795	11.3625	22.84081	13.07004	29.62243
LOC107765771	7.290155	7.640689	7.858099	5.679879	4.967123	6.524975	5.2891	5.045414	5.287236	3.341146	4.843488	3.079463	4.138906	4.451341	3.741207	6.885861	6.215361	7.568404	4.06865	4.60981	3.257884	6.286065	6.189259	5.295669	2.46554	2.713445	2.671077	15.64047	3.334253	6.761419
LOC107814477	0.088107	0.040149	0.07659	0.199552	0.077963	0.189452	0.369497	0.301125	0.124078	0.300705	0.247123	0.223306	0.245206	0.288362	0.768662	0.131315	0.127679	0	0.536431	0.70806	0.723198	0.662226	0.233149	0.138135	0.278486	0.551677	0.052068	0	0.42418	2.723902
LOC107779981	98.04772	117.1055	125.5906	11.78499	19.2866	14.72959	29.94651	35.41355	59.05069	17.54292	21.93049	14.20503	29.19345	25.217	24.78552	119.0598	89.77565	124.7373	30.89379	37.28568	35.9073	57.38111	29.66232	42.23571	36.74248	25.03234	29.7685	282.0554	78.61725	136.9076
LOC107762052	17.23797	7.551726	8.939313	9.04832	13.58394	16.90235	5.553698	4.930601	6.945823	12.51672	6.536467	12.75054	12.80676	12.91384	1.670544	6.670973	9.684662	8.108116	11.74885	8.135957	13.15733	2.78032	5.481642	8.196631	6.173418	2.830893	11.9795	5.29081	11.39973	0
Novel01936	1.571151	1.630175	1.050591	1.620467	1.368869	0.857583	1.115055	0.95003	1.180053	0.518547	0.525422	0.449258	1.587579	1.845899	0.620226	1.404988	0.957422	1.297106	0.760356	1.022374	0.396808	1.211178	1.40718	1.010564	0.427843	0.201798	0.428533	1.728608	2.094669	7.472819
LOC107807254	0.161832	0.049163	0.093785	0.390965	0	0.1432	0.231986	0.090491	0.046091	0.10129	0	0.113477	0	0.42036	0.294252	0.387568	0	0.156344	0.040218	0.328433	0.136899	0.393584	0.04505	0.047582	0.281912	0.272807	0	0.127515	0	0
LOC107759535	3.282895	5.743483	3.490749	1.646397	1.063409	0.504216	4.621955	4.758473	3.494911	2.715335	5.179428	1.832478	3.556674	2.65413	5.324971	10.02594	6.314788	5.22287	1.695371	1.884463	3.44851	5.33639	5.89488	2.787916	2.557052	2.120815	1.039317	0	3.245675	1.812378
LOC107789662	0	0	0	0	0	0	0	0	0	0.048784	0	0	0	0	0	0	0	0	0	0	0	0	0.044128	0	0	0	0	0	0	0
LOC107832750	0	0	0	0	0	0	0	0	0	0	0	0	0	0	0	0.048332	0	0	0	0	0	0	0	0	0	0	0	0	0	0
LOC107783776	0	0	0	0.042069	0.020545	0	0	0.019838	0.065394	0.045281	0.01628	0	0	0	0	0.017302	0	0	0.04712	0	0.084701	0	0	0.029355	0	0	0	0	0	0
LOC107809890	0.424816	0.561392	0.461604	0.192431	0.35711	0.205528	0.231602	0.217784	0.299127	0.124275	0.223411	0.107669	0.141873	0.046345	0.261611	0.506517	0.246245	0.570092	0.193983	0.413269	0.232463	0.97563	0.524602	0.399615	0.214838	0.05911	0.175734	0	0.102261	1.313349
LOC107771744	0	0	0	0	0	0	0	0	0	0	0	0	0	0	0	0	0	0	0	0	0	0	0	0	0	0	0	0	0	0

LOC107824598	61.24596	35.53106	37.06016	458.8682	340.635	547.9354	172.5682	211.6848	262.5301	833.2338	475.9302	948.6795	442.0491	590.8512	342.5095	34.72244	61.11269	44.81762	590.1524	635.3358	568.3227	125.2446	130.6203	214.3229	403.0677	587.0857	565.2789	143.8899	436.2352	73.87235
LOC107771874	39.91731	18.61685	36.73562	97.36863	83.41172	185.4656	52.57833	52.68083	94.39411	246.5213	164.7208	321.3209	110.1525	119.3987	25.6784	19.76113	36.02879	25.91779	59.99733	74.11368	72.72507	16.668	50.50031	86.9689	112.4267	40.93943	458.3899	107.217	40.6006	17.38132
LOC107778214	0	0	0	0.029621	0	0	0	0	0.030697	0	0	0	0	0	0	0	0.031588	0	0	0	0	0	0	0	0	0	0	0	0	0
LOC107825286	1.119502	1.373464	1.38488	0.370577	0.190502	0.717531	0.144458	0.202343	0.060637	0.209934	0.105672	0.036376	0.071899	0.023487	0.06629	0.818211	0.873546	0.754383	0.152922	0.236755	0.098173	0.287669	0.151919	0.11251	0	0.029956	0	0	0.103648	0
LOC107779334	0	0.029377	0	0	0	0	0	0	0	0	0	0	0	0	0	0.024021	0	0.024032	0	0	0	0	0	0	0	0	0	0	0	0
LOC107816987	3.55693	5.223891	4.490241	6.793719	8.800708	6.381677	8.018942	9.136771	4.424177	9.101936	11.75573	5.320944	6.506898	5.585851	9.882565	7.985652	3.830264	5.303685	12.18435	11.67154	17.43898	10.74672	9.911936	7.435405	17.8163	21.46759	16.11982	14.58177	8.834844	15.40913
LOC107801111	16.20118	11.1758	15.28343	12.17603	12.00045	16.67612	9.501823	9.818277	13.2662	7.998085	9.936685	9.639363	9.763515	10.05553	6.959777	11.28642	14.08261	14.71236	8.322927	8.72454	10.48004	12.07191	13.93236	20.62573	3.952902	2.824908	8.542436	0	4.105171	5.021271
LOC107828493	2.126208	2.712887	2.370131	2.470115	2.036348	2.689334	2.286874	1.688453	2.066631	1.561089	2.297738	1.732882	1.531539	2.210431	0.92426	1.957265	1.884935	1.902214	1.878319	2.158351	1.368803	1.357851	2.184355	2.745209	1.075269	0.278444	0.768684	0	1.445128	1.546666
Novel04561	0.495819	0.64554	0.523363	8.374131	9.213403	11.68938	1.782278	3.02601	2.925981	13.05413	11.77107	11.24997	8.279293	7.456892	8.033276	1.715466	0.444789	0.897732	16.99837	20.61194	8.01032	2.661889	3.342568	9.25411	12.31344	7.046798	17.87356	10.13088	19.60791	4.379607
LOC107818437	0	0	0	0	0	0	0	0	0	0	0	0	0	0	0	0	0	0	0	0	0	0	0	0	0	0	0	0	0	0
LOC107808109	4.049093	4.082829	5.317131	4.917047	3.8116	6.483594	4.407768	4.269362	5.419113	5.12449	4.470272	3.930265	5.466551	4.98128	2.917957	2.82479	3.329161	3.468375	3.14712	3.716765	2.357133	1.726725	3.571568	4.862426	1.960577	1.798091	2.749233	0	0.414761	0
LOC107803553	13.92373	10.7158	11.41197	37.7625	51.74667	45.28933	28.47314	31.08279	25.64644	60.77502	43.76452	48.99448	65.39564	63.26801	52.62695	11.49593	15.71477	12.48984	67.32506	42.70181	94.29274	20.74596	17.81815	26.65755	97.12845	44.28311	133.0122	94.83275	103.4236	51.92884
LOC107775220	0	0	0	0	0	0	0	0	0	0	0	0	0	0	0	0	0.110367	0.042586	0	0	0	0	0	0	0	0	0	0	0	0
LOC107784668	4.558979	4.375955	6.366134	3.602944	3.347432	4.640744	4.067885	3.48092	4.325704	3.310539	4.387042	3.114021	2.429578	3.121742	2.787615	4.987609	5.247745	5.713066	3.740281	3.89761	2.653957	4.293364	4.748581	4.15674	2.452739	1.889549	4.012599	3.46842	1.634466	0
LOC107832187	2.950972	3.165704	1.736866	4.28863	3.862422	5.320809	4.254091	3.992202	2.799362	4.46623	5.474821	4.233014	6.125275	5.600355	4.637846	3.504765	2.791494	2.566754	3.119211	3.978509	2.214746	3.157556	2.684295	3.21287	1.63228	2.053031	2.179883	0	4.439698	3.801314
LOC107797014	0.277142	1.010322	0.481827	0.167384	0	0	0.154967	0.078932	0	0.090083	0.129555	0.234137	1.028393	0.201565	0.284451	1.445684	0.535484	0.482115	0.093741	0	0	0	0	0.193112	0	0	0	0	0	0
LOC107770934	0	0	0	0	0.069281	0	0.19701	0	0.073507	0	0	0	0	0.085417	0	0	0.07564	0.175118	0	0.066233	0	1.765441	0.828743	0.081835	0	0.108943	0	0	0	0
LOC107769524	0	0	0	0	0	0	0	0	0	0	0	0	0	0	0	0	0	0	0	0	0	0	0	0	0	0	0	0	0	0
LOC107793671	0	0	0	0	0	0	0	0	0	0.079312	0	0	0	0	0	0	0	0	0	0	0	0	0	0.085011	0	0	0	0	0	0
LOC107771201	0.026282	0.119763	0.091385	0.642871	1.25582	0.508612	0.815618	1.279989	1.554498	1.255779	0.460722	0.799331	0.643662	0.831495	1.564549	0.137097	0	0	0.53338	0.489118	0.551299	0.197538	0.231823	0.082409	1.794327	1.426203	1.491027	0	0.759182	1.625047
LOC107798897	0.260164	0.184417	0.30154	0.209507	0.204632	0.217551	0.096983	0.049398	0.162836	0.084564	0	0.146529	0.09654	0.220754	0.02967	0.409291	0.307194	0.258618	0.234664	0.293442	0.158183	0.048282	0.178486	0.090641	0.219285	0	0.034166	0	0.139169	0
LOC107784428	0.152046	0.034643	0.165213	0.103309	0.033635	0	0.159409	0.097434	0.071374	0.037066	0.026654	0.128452	0.296204	0.248812	0.078028	0.198283	0	0.056678	0.154286	0.032155	0.173335	0.031744	0.033529	0.079459	0	0	0.089853	0	0	0
Novel02734	5.429143	5.575205	5.317678	5.057079	8.660846	11.83841	5.066744	5.226825	3.51773	4.772147	4.825671	2.390247	5.107435	5.172135	5.807774	4.900551	6.205363	5.415879	7.604137	9.767534	4.463297	6.322083	5.126107	4.156	9.957761	3.830327	2.440196	0	9.939737	18.91223
LOC107775437	1.498761	2.101439	0.400874	0.313339	0.136021	0.2479	0.128931	0.36119	0.324716	0.187369	0.431152	0.486998	0.128342	0.293475	0.157773	2.46286	1.967698	2.635894	0.584938	0.260071	0.455632	0.770249	1.525391	1.486171	0	0	0.090842	0	0	0
LOC107767951	11.71913	12.6651	11.15084	19.76329	17.93705	19.36747	18.09794	17.21855	18.62237	19.26483	18.6281	26.62471	28.69216	27.55685	19.83027	13.21768	15.79678	13.10566	14.02103	17.01384	19.89565	14.31672	14.84205	16.59386	9.01006	11.07287	9.873566	2.831363	16.20171	17.13608
LOC107828528	4.711823	0.500995	0.614383	0.355723	0.764377	0.253289	0	0.201295	0.368639	1.225233	0.330393	0.530757	0	0.257018	0.080601	0.234084	0.606936	0.702573	0.398436	0.265725	0.71621	0	0.277076	0.08208	0	0	0	0	0	0
LOC107802650	0.028623	0.104345	0.024881	0.077792	0	0	0.024007	0	0	0	0	0	0.031863	0	0	0	0.027652	0.064019	0	0	0	0	0	0	0.144752	0	0.03383	0	0	0
LOC107765211	0	0	0.152794	0	0	0.188975	0	0.100122	0	0	0.041084	0	0	0.063919	0	0	0	0	0	0	0	0	0.342512	0.051681	0	0	0	0	0	0
LOC107827986	0	0	0	0	0	0	0	0	0	0	0	0.07363	0	0	0	0	0	0	0	0	0	0	0	0	0	0	0.103009	0	0.209796	2.694442
LOC107774797	1.988533	1.359227	2.042878	1.187359	0.559868	1.360492	1.895305	1.505982	0.975891	0.705117	1.10915	0.68726	0.9559	1.281913	0.974102	0	0	0	0	0	0	0	0	0.079728	0	0	0	0	0	0
LOC107761176	0	0	0	0	0	0	0	0	0	0	0.067146	0	0	0	0	0	0.092511	0	0	0	0	0	0	0	0	0	0	0	0	0
LOC107801998	1.082907	1.051302	1.145989	1.002387	1.062383	1.138948	1.401912	0.543091	0.972478	1.446222	1.436134	1.272863	1.310334	1.001621	1.280603	1.280648	1.319096	1.140826	1.051083	0.856322	0.880279	0.825724	1.162847	1.008827	0.744089	0.851662	1.001668	0	0.453348	1.455604
LOC107759811	10.66642	9.72113	11.92127	11.82021	12.13503	13.7203	9.825053	9.520543	12.51764	10.21538	9.816585	13.03419	17.38695	15.68856	8.015299	9.439	11.77676	9.94034	8.504218	9.26475	10.2491	7.953401	10.75256	13.53752	4.455065	4.637985	11.25612	13.62143	13.29649	0
LOC107770380	5.983916	4.146232	4.845411	15.48362	12.98354	13.48379	20.93529	19.78592	26.24282	8.632712	8.478069	11.01103	17.33784	19.40562	12.61999	5.467128	5.780966	5.255735	5.489812	4.403231	6.803101	15.95037	15.72617	21.03359	0.466366	0.570287	1.598581	0	5.130317	5.068419
LOC107764857	2.068423	1.552445	2.00957	1.432972	1.076633	1.111904	1.173592	0.831678	1.485002	1.008487	0.853164	0.770939	0.474064	0.331845	0.561963	1.450721	1.234226	1.768876	0.617321	0.463167	0.998702	0.863697	1.126888	1.398886	0.15383	0.169298	0.647129	0	0.292886	0
LOC107808378	0.155892	0.142076	0.271027	0.941536	0.275887	0.614545	0.305091	0.488394	0.243931	0.557387	0.327935	0.614609	0.347083	0.283451	0.320007	0.193618	0.351411	0.193707	0.580025	0.527496	0.521313	0.303776	0.275014	0.434502	0.065698	0.289217	0	0	0.250173	0
LOC107772606	4.443081	2.815543	3.952798	9.622759	9.122379	7.016007	8.122831	7.94848	7.593179	9.105157	9.200435	12.14067	11.12872	8.936994	8.906751	3.414458	3.621704	2.510258	7.502479	6.107629	13.35948	6.34849	6.552231	6.639336	5.178545	3.960487	5.087259	2.482368	5.514757	2.146269
LOC107802844	6.393686	5.33439	5.169005	7.717512	7.306987	8.837993	6.550888	5.51072	6.772697	9.033883	9.136433	8.047228	9.192625	7.625945	6.313587	6.500946	8.013683	7.101506	6.317635	6.007824	6.800174	5.277266	7.398963	10.57928	6.528125	2.982741	8.327927	11.99752	5.743483	5.762846
LOC107817999	6.691688	5.93041	5.897176	17.97794	16.86534	33.93764	7.431865	7.767995	7.148996	34.87629	22.19903	32.20445	22.29647	27.43907	15.4889	4.745918	8.114354	6.81248	21.49469	27.75699	13.51063	5.511322	6.838771	6.945914	14.58677	8.02672	36.21793	16.50173	12.66429	8.560492
LOC107779535	4.624029	5.418299	5.539576	4.840389	3.85096	5.138503	5.051662	4.266304	2.973204	3.542796	3.637451	2.199463	2.963076	3.24295	4.24745	5.371444	4.373352	4.591719	3.193813	3.533598	2.303509	3.861648	3.855914	2.39621	5.232084	1.865319	2.663726	1.389426	7.950616	2.402611
LOC107762824	1.821318	1.506212	1.935063	0.763899	0.567052	0.870284	0.622363	0.778092	0.918298	0.986675	0.543954	0.854833	1.126398	0.735914	0.415411	1.080772	1.466289	1.332724	0.2738	0.085595	0.215324	0.591512	0.654509	1.233842	0.426426	0.09386	0.558095	0	3.572341	0
Novel00561	6.168654	5.91614	6.359913	4.28884	4.157293	4.434224	3.158495	3.094984	3.821653	2.465539	3.5333	2.484529	2.495288	2.895335	2.171796	4.944327	4.920001	5.869064	2.347321	2.821499	2.502224	2.426046	3.068562	3.673561	0.929538	1.122806	1.165689	5.129604	0.949648	3.326312
LOC107787474	0.766533	1.125523	0.740368	0.46296	0.602915	0.686764	0.821514	0.327473	0.519751	0.20763	0.597215	0.575634	0.52147	0.418125	0.524499	1.428053	1.028521	0.888973	0.388912	0.252169	0.815606	0.782408	0.863948	0.71216	0.161522	0.177762	0.201329	0	0.20502	2.633105
107787943	0	0	0	0	0	0	0	0	0	0	0	0	0	0	0	0	0	0	0	0	0	0	0	0	0	0	0	0	0	0
LOC107817605	0	0.077559	0.036988	0.154195	0	0	0.107067	0.036356	0.079897	0.041492	0.029836	0	0	0.139262	0	0.063417	0.041107	0.031723	0.431774	0.287959	0.077614	0.035535	0.15013	0.088948	0	0	0.150874	0	0	0
LOC107832415	2.236713	4.11404	3.711898	6.079047	6.621294	5.290429	6.173837	5.073125	4.047128	6.701858	7.955989	3.882346	5.477869	5.235219	9.015845	5.333765	2.357294	5.093652	5.405923	5.504307	2.373725	7.539676	4.73503	2.932892	7.198255	6.167861	3.94139	0	6.461001	10.05814
LOC107818842	0.027467	0	0.023877	0.074651	0.121523	0	0.138227	0.305092	1.237792	0.026784	0.15408	0.27846	0.703265	2.786769	0.140957	0.020469	0	0	0	0	0	0	0.045877	0.024228	0	0	0	0	0	0
LOC107762507	0	0	0	0	0	0	0.099622	0	0	0	0	0	0	0.064789	0.060954	0	0	0.132828	0.060262	0	0.054162	0	0.104767	0.248287	0	0	0.210574	0	0.285912	0
LOC107778475	15.48464	14.81795	15.29834	11.76219	9.617627	13.89458	11.81375	9.744768	13.39142	5.923641	7.266399	5.232741	4.408877	5.230326	4.003815	16.44332	12.16895	15.94023	8.098104	9.219637	6.029114	14.49834	14.105	18.51885	4.367241	1.40876	4.082663	0	2.867252	1.841228
LOC107804853	0.251136	0.523153	0.405427	0.130009	0.190476	0.501432	0.060182	0.030654	0.168412	0.104952	0.125783	0.090929	0.23963	0.313116	0.036823	0.106941	0.173299	0.21398	0.29124	0.151745	0.13088	0.089884	0.094936	0.112494	0	0	0.084806	0	0	0
LOC107779123	0.353065	0.344759	0.504213	0.15993	0.223154	0.216908	0.423044	0.323216	0.35515	0.14755	0.318304	0.191751	0.336889	0.330152	0.414145	0.375864	0.462909	0.470045	0.051181	0.192001	0.046	0.336976	0.51163	0.289947	0.031884	0	0	0	0	0
LOC107817420	212.7677	202.6842	191.3346	142.7236	148.7003	154.4556	149.3257	156.6071	176.0712	211.1517	193.4586	216.8206	156.892	154.4106	128.8868	182.0466	258.2083	201.237	207.4775	206.6541	210.4348	144.3609	192.1327	172.7097	242.4831	168.4225	348.4614	180.6978	128.7907	86.7957
LOC107823775	2.664309	2.703551	5.730376	61.88602	102.4443	67.18187	59.09206	76.43735	46.85557	148.1417	98.72649	119.4592	109.8623	111.1112	125.7622	7.225386	2.839302	6.860093	136.9337	112.3658	107.0156	64.82418	32.10183	60.94837	310.3878	220.941	212.8009	196.4286	444.8433	390.6164
LOC107790211	6.530103	6.58451	5.918032	9.125656	7.806804	7.543474	6.467614	8.072464	7.630825	7.045087	6.38125	6.92536	7.037399	7.502959	6.274511	5.798074	6.711307	6.474028	5.780387	5.171421	6.969277	4.293149	6.740405	6.97048	3.074056	3.576468	6.650665	4.968029	6.020089	0
LOC107781870	0	0	0	0	0	0	0	0	0	0	0	0	0	0	0	0	0	0	0	0	0	0	0	0	0	0	0	0	0	0
LOC107763612	276.9746	253.6424	292.0305	146.807	132.0979	202.3407	131.9171	134.1442	169.1221	85.40164	98.23827	106.9272	64.55458	88.0488	72.40123	197.3097	258.0292	233.733	90.57789	83.69605	117.0299	124.6725	174.5425	219.1358	32.09246	17.65964	58.51073	35.10208	22.95567	60.69889
LOC107802024	1.083676	1.107524	1.05092	1.52087	1.856845	0.787947	1.796836	1.477194	1.711354	1.062829	1.330878	0.60329	0.85073	0.738044	1.137972	1.386383	0.992458	1.354328	1.430167	1.29294	0.765528	2.584237	1.596807	1.689166	0.760284	1.551436	0.473829	0.447966	0.87456	5.80971
LOC107793185	3.708302	4.455006	3.736385	3.015945	3.094909	1.948133	3.923235	3.924587	3.402392	2.342222	3.457168	2.136052	2.29862	2.06876	3.935853	4.019536	3.582531	3.550121	2.651154	2.887437	3.036144	3.132092	3.419648	4.140183	1.864713	1.58313	1.842816	0	2.840263	0
LOC107782211	4.404447	6.085909	13.6886	13.68683	12.44636	10.69265	20.02018	20.39454	19.67502	16.62539	14.09705	17.32465	18.79518	18.98764	20.75589	15.1934	5.05575	9.498019	21.14521	22.23496	18.74567	20.23048	16.87683	15.41934	17.24457	23.55835	15.02936	25.40163	26.67672	11.71327
LOC107766315	17.22734	14.93311	17.15055	16.91495	14.9884	14.1498	15.03455	13.03375	14.02782	7.695552	9.27919	7.72426	6.0533	7.113438	6.074985	16.69277	16.68954	18.35072	12.18306	14.77668	11.12541	15.17065	20.16182	15.94387	3.041982	2.845669	3.981265	1.720668	3.243406	8.926191
LOC107808315	0.079484	0.144879	0	0.072008	0	0	0.066666	0.271652	0	0	0	0	0	0	0	0.592314	0.38394	0.237034	0.080655	0.134475	0.362452	0.398274	0.420659	0.498458	0	0.110596	0.56366	0	0	0
LOC107769919	0.11891	0.243836	0.18089	1.131126	1.104802	2.141363	0.274271	0.482599	0.279094	7.88474	8.171202	7.63483	2.845999	1.491834	1.311992	0.022153	0	0	0.935126	1.860906	0.677798	0.024826	0.052443	0	4.510144	2.688632	5.691943	0	0.143119	0
LOC107812800	0	0	0	0	0	0	0	0	0	0	0	0	0	0	0	0	0	0	0	0	0	0	0	0	0	0	0	0	0	0
LOC107777804	2.781559	2.980394	2.973425	2.809405	2.627611	2.000413	2.774394	2.376639	2.64674	2.235874	2.965182	2.33406	2.866327	2.521952	3.375745	3.193265	2.523808	3.278799	1.94526	2.575589	1.594088	2.652561	1.98934	1.944738	0.641567	1.385999	0.977388	1.344059	1.538203	2.324162
LOC107769002	184.7322	148.2426	197.1725	74.14595	74.07573	154.3625	53.11205	58.50077	67.52977	42.32085	37.05666	59.74004	28.94669	47.85789	34.94475	76.74257	142.7735	120.0829	52.86662	45.33829	87.26779	42.72823	70.54118	69.99835	25.30701	13.1449	41.34614	60.20186	28.36967	17.35028
LOC107802638	17.87603	13.65234	15.62606	15.47099	8.615888	24.26523	4.858195	5.24769	6.516228	3.067534	4.691753	2.952938	3.112825	4.957179	1.486247	13.39566	12.49404	16.67779	6.586918	6.927347	4.645057	3.377731	8.544584	8.08945	0.378783	0.903216	1.829522	7.141837	0.961582	3.087435
LOC107768128	0	0	0.117655	0	0	0	0	0.115645	0.127071	0	0	0	0	0.147658	0	0	0.523031	0.504539	0	0	0	0	0	0.238772	0.565864	0	0	0	0	0
LOC107812484	0	0	0	0	0	0	0	0	0	0	0	0	0	0	0	0	0	0	0	0	0	0	0	0	0	0	0	0	0	0
LOC107799428	0.827561	1.286611	2.031195	31.4004	50.05354	30.14613	43.72901	71.04183	70.83965	47.56416	35.67055	79.04471	36.74173	49.70869	61.05588	2.75701	1.504929	3.302669	95.18836	84.37781	117.519	80.49493	50.19574	87.36165	109.7988	153.6902	123.8749	146.2152	94.21401	219.7273
LOC107817524	0	0	0	0	0	0	0	0	0	0	0	0	0	0	0	0	0	0	0	0	0	0	0	0	0	0	0	0	0	0
LOC107801830	0	0	0	0	0	0	0.103129	0.105058	0	0.239798	0	0	0.136878	0.13414	0	0	0	0	0	0	0	0	0	0	0	0	0	0	0	0
LOC107816877	0	0	0	0	0	0	0	0	0	0	0.234314	0.070577	0	0.091138	0	0	0	0	0	0	0	0	0	0.073688	0.087316	0	0.23248	0	0	0
LOC107767017	1.863699	1.698532	1.193739	1.510689	2.690676	1.581872	1.316349	1.508585	1.197179	0.382598	0.894145	1.408768	2.183891	3.317334	1.308792	0.80406	1.705728	1.096948	0.696741	0.580838	0.984051	0.737253	0.865214	1.742892	0	0.682421	1.159338	0	0.472237	0
Novel03039	4.752455	2.996775	4.153539	4.67785	5.955593	5.219945	3.533614	3.863083	3.834748	4.809594	4.539302	5.621009	6.520183	6.922268	3.454002	3.598965	3.598564	4.041114	5.630551	4.476603	6.18519	3.389655	5.23431	6.470869	5.748719	3.574429	9.533752	0	4.328651	0
LOC107773591	2.39125	1.71233	1.732219	1.960032	1.662521	2.32604	1.146082	1.264806	2.138106	0.66622	1.117831	1.106297	1.647888	1.80127	0.233744	0.55156	1.430092	1.31586	0.404413	0.52979	0.98658	0.903403	1.757701	2.558842	0.071983	0.07922	0.33646	0	0.548206	0
LOC107796126	0.074935	0.136589	0.325698	0.203663	0.132615	0	0.188554	0.064027	0.492468	0.365357	0.367813	0.316537	0.166838	0.327003	0.076912	0	0	0	0.152078	0	0	0	0	0	0	0	0	0	0	0
LOC107772799	6.577924	6.661074	6.565168	7.393903	6.467311	7.072072	6.232379	6.036679	3.774005	5.345255	9.139472	1.749493	4.068131	3.85388	7.751608	11.43771	10.82674	11.80622	6.427607	8.861935	2.888493	11.59717	7.413876	5.856689	2.926174	4.067867	1.727684	0	2.932265	7.531906
LOC107788453	3.248684	3.056904	3.154092	2.809364	1.829323	3.243252	2.140923	2.487373	3.62436	1.501656	2.292772	1.728728	2.489266	2.163315	1.66718	3.049735	2.282547	2.941039	1.455615	1.659605	1.13512	3.153493	2.921377	3.814452	0.640103	0.44029	0.822789	0	0.710924	0
LOC107802696	3.241246	3.330213	3.242837	4.002929	4.31563	4.93123	3.936801	3.827532	3.559765	3.995506	3.870131	2.906209	3.65926	3.669476	3.35811	3.406598	2.894991	3.487948	5.166201	5.186275	3.13734	4.162419	4.558207	4.634186	9.220321	7.466958	5.87283	2.186745	7.066195	6.617349
LOC107808720	0.43754	0.113932	0.10867	1.245794	0.829637	0.470409	1.310655	0.427252	1.114979	0.609509	0.306803	0.264033	0.347911	0.545525	0.705695	0.232897	0	0.139802	0.824544	0.687382	0.570062	0.208801	0.330805	0.195993	0	0.347888	0.073877	0	0	0
LOC107807376	0.836403	0.857563	0.817951	0.568304	0.647592	0.449618	1.315364	1.518622	0.883409	0.713649	1.172973	1.324907	2.094966	0.342178	0.858462	0.857022	0.707031	1.247148	1.379177	0.619097	0.57211	0.960441	1.567747	1.748417	0.925285	0.145474	0.494281	0	3.523401	0
LOC107821656	3.710681	4.067997	3.459357	3.946331	2.760006	3.815982	3.382944	2.711013	2.423479	1.782971	2.639641	2.589689	2.634122	1.466729	2.594224	3.406411	2.234025	3.728721	2.019097	1.728714	1.226161	3.682735	2.703837	3.822194	1.223851	0.82311	0.826296	0	0.517813	0
LOC107761987	0.317178	0.123887	0.275717	0	0	0.048715	0.114013	0	0	0	0	0	0	0.050441	0	0	0.33766	0.087549	0.23647	0.183915	0	0	0.037841	0.15987	0	0	0	0	0	0
LOC107816040	0.733394	0.809113	0.738185	0.104908	0.170778	0.497992	0.06475	0	0	0.03764	0.243595	0.09783	0.04297	0.252662	0.079235	0.604055	1.118716	1.323774	0.078337	0.032653	0.140814	0.128942	0.102142	0.363099	0	0	0.136865	0	0	0
LOC107829851	0	0	0	0	0	0	0	0	0	0	0	0.395106	0	0	0	0	0	0	0	0	0	0	0	0	0	0	0	0	0	0
LOC107789795	0	0	0	0	0	0	0	0	0	0	0	0	0	0	0	0	0	0	0.042968	0	0.038619	0	0	0	0	0	0	0	0	0
LOC107775556	0	0	0	0	0	0	0	0	0	0	0.026722	0.032195	0	0	0.097783	0.014199	0	0.014206	0.019335	0.032237	0	0.047738	0	0	0	0	0	0	0	0
LOC107821711	12.51767	11.79597	12.30754	7.101496	5.269383	12.41274	5.504387	5.451557	6.275414	3.732997	4.814786	5.082292	3.991048	4.839319	3.679708	9.011205	12.56276	10.51034	5.734428	5.140333	5.985258	4.211981	7.503872	6.986316	2.0743	1.35281	5.171025	0	2.34037	0
LOC107813458	12.54528	8.604572	11.18641	11.01388	10.64312	14.322	10.95617	11.98984	10.07813	8.071409	9.794326	11.58197	13.31769	12.91025	9.690282	10.02341	12.61958	11.18507	7.218086	7.111429	11.02867	9.774828	9.810883	11.42259	5.068993	2.699356	5.426562	0	4.669904	11.99526
107782658	0	0	0	0	0	0	0	0	0	0	0	0	0	0	0	0	0	0	0	0	0	0	0	0	0	0	0	0	0	0
LOC107793795	3.55665	5.530863	2.767414	1.284352	0.990365	0.508062	5.277821	4.675229	2.008154	0.994374	2.842726	0.714418	1.467438	0.651211	3.650449	5.245237	4.012699	2.929777	0.78238	0.904708	0.907336	5.815878	3.839232	0.727884	0.283008	0.346071	0.470341	0	0	0
LOC107773432	3.623757	4.803793	4.009161	5.670533	3.789546	1.416721	2.486783	4.222141	3.092857	2.891144	4.850963	3.061451	2.567102	2.515762	5.409927	7.364804	2.864335	3.684088	6.017135	3.065449	4.807167	6.052588	5.521043	3.098904	2.082514	4.125431	3.114901	0	1.586005	0
LOC107785324	0	0	0	0	0	0	0	0	0	0	0	0	0	0	0	0	0	0	0	0	0	0	0	0	0	0	0	0	0	0
LOC107789790	14.62858	12.25698	13.19489	9.048924	5.985014	11.92247	6.992365	5.644736	6.571625	10.27673	10.7539	14.4184	9.805866	11.58319	9.283082	8.732711	15.5001	11.19941	6.065321	5.322483	5.810014	6.89653	8.255386	9.617771	1.193224	0.875467	6.413961	0	4.922331	4.862942
LOC107795306	0	0	0	0	0	0	0	0	0	0	0	0	0	0	0	0	0	0	0	0	0	0	0	0	0	0	0	0	0	0
LOC107799675	1.776556	2.872618	2.191942	2.647838	2.332666	2.341309	3.220483	2.056561	2.690177	1.00589	2.210153	1.98504	1.084538	1.875615	0.411737	2.605078	2.657501	2.478093	1.628269	1.163493	1.411185	1.914385	2.375835	3.114727	0.579641	1.355585	0.948273	0	0.275902	0
LOC107779546	0	0	0	0	0	0	0	0	0	0	0	0	0	0	0	0	0	0	0	0	0	0	0	0	0	0	0	0	0	0
LOC107787591	4.231645	5.77534	4.483718	7.419364	7.693795	7.763635	6.586443	6.493747	5.514559	6.836255	7.98643	5.887207	7.265299	5.604129	8.016635	5.491041	4.332192	4.897113	9.250217	9.759526	6.873834	6.417385	6.610956	6.249291	12.64324	12.30334	6.220726	3.011168	11.65599	11.71563
LOC107815973	3.365218	3.328797	3.068011	3.60641	3.316699	3.029725	4.348568	3.693518	3.364906	3.028063	3.894453	2.507876	3.365496	2.984787	3.552252	2.946114	2.999984	3.171837	2.581915	2.835171	2.819613	3.770024	3.571667	3.660306	2.248397	1.884403	2.409086	0.978296	3.490537	3.383358
LOC107811556	9.544252	40.07481	18.46985	5.249727	3.518909	3.786885	1.524795	3.009534	0.32002	1.772733	2.15114	1.151893	0.252971	0.123956	1.049567	27.94152	13.28198	16.26433	4.842424	12.49514	1.450753	0.569332	2.004443	0.118758	1.436528	1.580968	0.268584	0	0.547017	0
LOC107817443	0.029344	0.160463	0.051017	0	0.051932	0	0.09845	0.025073	0	0.085843	0.041153	0.049582	0	0	0.030118	0.481085	0.028349	0.131265	0.059553	0.049647	0.026763	0.269569	0.051767	0.030671	0	0	0	0	0	0
LOC107794181	8.365552	10.05004	8.635496	0	0.042059	0	0.079734	0	0	0.046349	0	0	0	0.207418	0	8.005047	6.88792	7.087357	0	0	0	0	0.20963	0	0	0	0	0	0	0
Novel01689	0.27424	0.187452	0.119195	0.093168	0.121333	0.700248	0.115008	0.058579	0.225285	0.066855	0.192297	0.028961	0.190805	0.112193	0	0.715273	0.298056	0.460029	0.347849	0.666965	0.156319	0.944727	0.544269	0.429953	0.260042	0.524679	0	0	0	0
LOC107793615	0	0	0	0	0	0	0	0	0	0	0	0	0	0	0	0	0	0	0	0	0	0	0	0	0	0	0	0	0	0
LOC107813524	3.223831	3.975109	3.800169	5.226386	4.972279	5.204384	4.39499	3.777883	3.335901	3.824927	4.416125	3.077732	4.022137	3.092383	3.943987	4.856869	3.529114	4.487034	6.684432	7.725486	4.524066	5.668011	4.489945	4.30843	3.697335	5.846719	3.303054	2.141326	3.555819	4.319938
LOC107799666	0.656457	0.598279	1.04618	0.495597	0.338844	0.588143	0.665306	0.420671	0.847427	0.13336	0.345231	0.34662	0.517633	0.477441	0.336885	0.774554	0.79274	0.713732	0.194287	0.347073	0.174621	0.456853	0.699671	1.029192	0.069163	0.038059	0.064657	0	0.263368	0
LOC107765214	0	0.048026	0	0	0	0	0	0	0	0	0.03695	0	0.058662	0	0.054086	0.039269	0	0	0	0	0	0.044008	0	0	0.066624	0	0	0	0	0
LOC107814422	1.571393	1.884383	1.833287	1.498529	1.207515	1.585702	1.005838	1.036424	1.55294	0.83336	0.831229	0.500745	1.058785	1.03761	0.735679	1.222364	1.238448	1.63399	0.95113	1.0611	0.427427	1.174173	1.446865	1.498347	0.278838	0.383593	0.391002	0	0.066362	0.852295
LOC107785543	2.185427	2.987621	1.501412	2.586626	3.555715	3.259125	2.867777	2.59011	2.713639	3.643438	2.619949	4.943346	3.492315	4.230022	2.134528	4.701877	2.622107	3.941925	5.114835	4.114896	4.822129	2.619912	3.140272	3.094734	6.328264	6.179818	4.749386	5.041579	4.07281	2.179488
LOC107797133	0.261376	0.500245	0.681626	0.544625	0.346924	0.50582	0.657682	0.736978	0.613478	0.178412	0.568159	0.397472	0.552838	0.313663	0.34875	0.409033	0.505022	0.565114	0.238704	0.35377	0.262217	0.436564	0.414992	0.764933	0.033046	0.109106	0.030893	0	0.125836	0
LOC107759449	0.259949	0.533051	0.169476	0.412126	0.23002	0.209608	1.144661	0.777377	0.427091	0.76045	0.683537	0.164709	1.157516	0.496286	1.200621	0.290572	0.376699	0.290705	2.110228	1.594269	2.252241	1.139724	0.343939	0.203774	0.246491	0.090425	0.07681	0	0	0
LOC107812551	6.084038	4.620708	5.233634	4.27741	3.785313	4.156314	3.269074	3.276054	4.819468	1.85397	2.199724	2.221964	2.78674	1.970474	2.048973	4.368536	4.836851	5.717134	3.247603	2.62695	3.756963	2.593396	3.549732	5.895355	0.761195	0.573185	1.385735	0	1.373003	0
LOC107825671	3.69394	4.437112	4.433686	3.066479	2.625854	4.17084	3.124194	2.892085	2.727993	2.351134	4.02076	2.245891	2.391583	1.939077	3.299593	4.733772	3.583588	4.090653	2.509359	2.980999	1.198157	4.478922	3.640025	2.89164	0.762093	0.34409	0.657631	0	0.223229	0
LOC107761620	4.456504	6.62168	4.332942	6.754457	6.784157	7.062039	7.989458	7.832071	5.948761	6.693543	8.960031	4.924806	8.534874	6.03696	7.47887	5.225447	3.999289	4.157078	4.972224	5.395775	3.467266	5.2563	4.936951	4.326804	4.646357	4.555164	2.845801	7.552205	4.677436	5.223741
LOC107775073	12.93513	16.1753	15.10127	13.55812	12.41077	15.4835	11.18041	8.963789	8.349049	9.441706	10.75759	8.084783	9.669478	9.49661	9.494073	17.00884	11.82438	14.31135	12.01915	13.64598	8.076183	10.17442	9.104496	8.823239	7.939267	6.330794	7.510814	10.75633	7.512746	17.43747
LOC107784386	3.91996	4.746155	4.576308	2.075872	1.742698	3.155738	2.509559	2.443224	1.724553	1.458812	2.005075	1.471863	1.222695	1.797363	1.57435	3.146949	3.256464	3.727242	1.056878	1.34563	0.79446	2.815032	3.023368	2.889771	0.239421	0.158097	0.604315	0	0.364678	2.341807
LOC107782746	0	0	0.30154	0.314261	0.204632	0.124315	0	0.197592	0	0	0.567552	0	0.128719	0	0	0.172333	0	0	0.117332	0.097814	0	0	0	0	0	0	0	0	0	0
LOC107772923	0.498855	0.649492	0.495593	0	0	0.459711	0	0	0	0	0.099942	0	0	0	0	0.477961	0.481936	0.690704	0	0.301426	0	0	0.125721	0	0	0	0	0	0	0
LOC107789871	0.846656	0.840211	0.850466	0.477261	0.249726	0.99117	0.205148	0.353665	0.141312	0.146773	0.197892	0.158951	0.230391	0.513144	0.50208	1.023507	0.636176	1.136192	0.095459	0.127327	0.120114	0.377101	0.531062	0.15732	0	0.104716	0.044475	0	0.09058	0
LOC107786103	0	0	0	0	0	0	0	0	0	0	0	0	0	0	0	0	0	0	0	0	0	0	0	0	0	0	0	0	0	0
LOC107767384	10.44115	10.626	9.651099	6.84212	6.344124	8.26946	6.1594	5.382458	6.992529	4.921028	6.345161	6.880358	6.857707	6.872437	4.465212	6.769258	7.767011	7.395103	4.838363	4.210155	4.412093	6.336298	6.938037	9.021484	2.772165	2.808765	3.290826	2.488966	2.848486	2.151973
LOC107801568	4.493988	5.301144	4.128639	0.422111	0.226093	1.325051	0.214308	0.038526	0.056444	0.058625	0.200243	0.025396	0.050195	0.196765	0.030853	2.755303	2.802418	3.215992	0.167766	0.178002	0.150784	0.087864	0.5303	0.094256	0.038005	0	0	0	0	0
LOC107784278	0	0	0	0	0	0	0	0	0	0	0	0	0	0	0	0	0	0.059185	0	0	0	0	0	0	0	0	0	0	0	0
LOC107815266	6.265956	8.441825	6.709903	10.36604	13.64437	9.197018	12.20196	11.85596	9.054328	14.41629	14.97676	11.83017	16.98346	13.79058	17.13124	8.120692	8.492363	8.273358	15.68371	15.24105	11.13116	13.60545	11.40642	9.168846	14.19084	16.80546	11.65617	19.4828	16.7009	15.72194
Novel00800	0.341236	0.20733	0.296629	0	0.402598	0.183435	0.095403	0	0.053395	0.388206	0.199396	0.768762	0.379869	0.558408	0	0.084763	0	0.339208	0.230842	0.192442	0.207475	0.047496	0.100331	0.653877	0.431426	0	0.268876	0	1.095222	0
LOC107822400	4.508638	5.257189	5.446623	6.817674	5.544286	5.595823	5.700189	5.211939	6.691729	4.75209	5.462816	5.405451	4.871458	5.931376	3.947021	4.02687	4.387751	4.102862	4.104093	4.374879	3.144435	5.149576	6.520974	5.855883	2.305264	1.84513	2.155048	0	2.713273	6.149467
LOC107832351	8.80756	10.64374	10.08463	13.32211	10.16246	11.4715	11.68297	11.33785	11.03738	6.999425	8.978132	8.522584	9.847891	10.3282	8.920369	9.714994	10.72039	9.632663	6.968734	7.549056	6.723357	10.6281	12.1153	11.9633	3.335724	3.94106	5.732284	8.324208	6.911458	2.399052
LOC107795717	109.8092	53.11988	71.12611	43.96419	38.54848	57.69491	19.45406	23.91305	35.43596	18.52789	17.82423	22.99362	22.39023	28.75862	10.36571	55.99898	82.27163	67.25508	40.55831	29.68459	49.0982	17.29735	26.27196	31.48865	13.0933	2.619963	17.39917	18.36222	29.25558	31.75215
LOC107782279	0	0	0	0	0	0	0	0	0	0	0	0	0	0	0	0	0	0.03239	0	0	0	0	0	0	0	0	0	0	0	0
LOC107807500	24.01306	21.45096	23.53504	23.23368	20.6464	20.40492	17.51621	16.21631	18.26553	14.59339	13.2128	13.16062	12.49497	15.06517	12.91664	22.50761	24.8416	24.19154	18.43041	17.95405	18.11584	19.25859	21.0611	23.88974	6.88041	10.4118	7.316478	9.729594	11.13499	25.23678
LOC107774645	15.31996	16.58541	18.50761	7.85034	7.839241	8.471338	7.541917	7.124821	8.882804	4.637924	5.410544	4.22514	5.271977	5.166543	5.824474	15.97238	13.64671	17.18198	8.969034	8.987991	6.246004	9.853415	10.40725	7.179451	2.78729	5.524423	2.714234	1.459818	2.456886	4.417583
LOC107823142	1.110827	2.102639	1.78268	4.373765	3.780511	5.144568	3.726794	6.315303	4.69302	11.58187	9.706448	13.71592	5.707329	8.436393	8.112409	1.464551	1.320803	1.751895	14.00317	18.46839	9.468522	4.602723	6.180421	4.331557	75.7308	63.13323	75.94668	21.38765	29.61923	7.925089
LOC107773599	36.3698	36.39517	39.07297	20.96269	19.56604	22.4739	21.58654	17.96382	27.33914	13.66763	18.17373	18.32344	17.41898	22.01385	15.13001	29.93916	31.5176	33.91656	12.19968	10.47691	13.77486	19.1221	22.16863	31.25712	6.263395	6.220662	10.1396	0	7.271477	3.735547
LOC107801086	0	0	0	0	0	0	0	0	0	0	0	0	0	0	0	0	0	0.031321	0	0	0	0	0	0	0	0	0	0	0	0
LOC107815475	0	0	0	0	0	0	0	0	0	0	0	0	0	0	0	0	0	0	0	0	0	0	0	0	0	0	0	0	0	0
LOC107831796	83.35334	80.89061	87.62486	83.21495	76.01002	109.1513	55.71025	53.8997	62.26214	72.42044	60.20904	69.25622	53.13785	62.55025	47.2971	70.78341	87.80762	82.00267	60.76437	68.99732	60.35747	55.44127	72.64282	77.3009	36.02722	34.14278	54.0989	36.91748	32.47455	46.81461
LOC107781789	0	0	0	0	0	0	0	0	0	0	0	0	0	0	0	0	0	0	0	0	0	0	0	0	0	0	0	0	0	0
LOC107769760	0	0	0	0	0	0	0	0	0	0	0	0	0	0	0	0	0	0	0	0	0	0	0	0	0	0	0	0	0	0
LOC107827999	0.345361	0.52459	0.700502	0.417173	0.407464	0.453818	0.128742	0.131149	0.252187	0.336771	0.403612	0.291771	0.042718	0.418635	0.196927	0.714899	0.481936	0.457745	0.428326	0.227229	0.594953	0.448653	0.338479	0.521403	0.242579	0.106788	0.272126	0	0.369487	0
LOC107765073	0	0	0	0	0	0	0	0	0	0	0	0	0	0	0	0	0	0	0	0	0	0	0	0	0	0	0	0	0	0
LOC107773745	10.35049	9.882397	23.48688	35.6408	50.19496	25.72457	55.13255	62.02104	54.30861	79.38982	49.98751	80.85715	51.77558	61.201	64.29263	39.63449	14.80437	28.52852	79.88618	72.35857	87.57377	55.11932	40.59014	38.96472	139.9823	152.0622	133.6676	153.8091	133.0983	99.73816

LOC107811780	0.05846	0	0	0.158884	0.569017	0.125702	0.39226	0.499494	0.219538	0.057005	0.040992	0.197553	0.195234	0.127553	0.060001	0.087128	0	0	0.059321	0.148358	0.533161	0.244106	0.10313	0.488815	0.295643	0.162684	0.069094	0	0.281445	0
LOC107759522	6.672443	6.93286	6.820446	10.75086	8.058197	11.75364	6.015744	4.716884	5.611418	3.75129	6.82758	3.341843	5.177724	6.093752	7.249948	7.95246	7.663978	6.967666	5.910608	7.004973	4.360859	6.13501	5.252888	5.406581	1.126631	0.967736	1.798177	3.108625	3.348379	9.407069
LOC107788346	0.38216	0.348291	0.249152	0.043277	0.08454	0.128396	0.020033	0.040816	0.246667	0.069873	0.050244	0.080715	0.053178	0.078172	0.024515	0.160192	0.323049	0.249302	0.145421	0.04041	0	0.139629	0.273886	0.249646	0	0	0	0	0	0
Novel01983	2.549928	3.195424	2.586031	2.598868	1.692258	1.941884	3.208101	2.178727	2.294235	1.139646	1.788011	1.436166	0.946205	1.506833	1.417637	2.21691	1.642296	3.010042	0.970309	2.067186	1.162788	5.057589	3.092656	2.887282	0.940299	0.443505	0.627877	0	0.511511	0
LOC107765971	0	0.147566	0.2815	0.146688	0.573095	0.696317	0.882737	0.207518	0.456041	0.631553	0.283839	0.410373	0.360494	0.088321	0.083093	0.482639	0.547485	0.060358	0.246451	0.410909	0.29534	0.40566	0.428461	0.423086	0.307066	0.112647	0.095686	0	0.38976	0
LOC107819488	346.712	292.8718	315.7504	214.141	212.4821	305.2159	180.645	178.6411	195.6164	199.2429	220.2948	212.8571	156.9692	164.4992	188.8446	265.4934	365.3256	282.7926	168.3126	179.9964	178.1343	157.2559	192.5101	213.0987	92.55983	57.73718	117.9399	154.1032	84.59029	71.74379
LOC107800472	4.552433	6.687182	5.633381	1.868053	1.682404	2.763909	1.684554	1.670293	1.33249	0.705049	1.427087	0.791828	0.77508	0.934867	1.319292	5.188497	3.854747	5.031154	1.08694	1.517765	0.879225	1.766745	1.700702	1.343492	0.067714	0.111783	0.47476	0	0.128924	0
LOC107768428	0.214891	0.146885	0.16345	6.059435	7.106856	5.746982	1.126491	2.386917	1.437465	9.062872	1.9777	9.190775	4.096643	6.153942	3.473797	0.120103	0.155702	0.080105	13.38324	9.566345	9.725731	1.480556	1.942871	2.442572	9.916611	16.78169	4.032	1.920971	5.302137	1.660882
LOC107769926	0	0	0.039017	0.121988	0	0	0.037646	0	0	0	0	0	0	0	0	0	0	0.033463	0	0	0	0	0	0.046913	0	0	0	0	0	0
LOC107798911	4.927046	4.490395	5.07304	3.185229	2.94179	3.265735	3.289972	3.208441	4.131718	2.402246	2.784004	2.525792	2.902181	3.887862	2.454847	3.956816	3.997426	4.386588	3.422074	3.904916	2.748481	3.39563	3.797459	5.449732	3.023924	4.159967	2.34631	3.420907	2.993856	7.394337
LOC107803442	1.324136	3.643567	10.55861	17.30192	27.60214	8.076238	29.32437	45.14632	46.37932	26.99104	39.56774	49.09226	33.73299	32.53054	59.74623	16.88857	1.402229	10.28966	46.09724	49.63028	43.47483	43.67991	37.17306	46.89585	39.40598	75.15023	31.63151	76.48397	95.00996	51.95807
LOC107781172	2.372156	8.267912	2.619379	17.33763	19.23174	7.237501	19.49299	20.70658	9.991783	25.25711	26.36646	18.46988	20.94731	19.89889	32.83576	10.12863	3.778211	5.735814	30.21886	29.99174	24.29491	17.10663	12.58266	8.845343	41.13666	44.5592	23.64199	20.63222	43.67532	43.60577
LOC107813856	1.859049	1.875363	1.43099	0.03857	0.213474	0.228859	0.130931	0.169755	0.146557	0.193735	0.22887	0.14387	0.047394	0.07741	0.101958	1.755501	1.329861	2.010229	0.057601	0.168067	0.142369	0.272583	0.200281	0.22249	0	0	0	0	0	0
LOC107759451	0.443896	0.269704	0.257246	0.134049	0.13093	0.222713	0.223389	0.252851	0.138916	0.490569	0.166006	0.200008	0.230604	0.290561	0.121494	0.198475	0.171536	0.220629	0.120116	0.150202	0.026989	0.074142	0.104412	0.154653	0.187073	0	0.52465	0	0.85483	0
LOC107778999	286.0938	278.1746	339.1379	125.7237	102.7012	155.7452	99.23575	110.4881	142.0546	34.66644	52.22365	36.12751	24.99808	36.94254	33.86323	215.7132	291.4629	260.0117	63.99964	65.59909	79.26104	112.8622	166.1753	201.453	9.506757	5.978652	28.29419	10.9743	5.417818	15.81406
LOC107818972	12.00062	15.68844	16.01123	6.015221	4.721946	5.922292	7.920345	6.975848	9.096485	6.018919	6.328376	4.903025	5.39298	4.319316	7.445793	21.66082	11.94997	17.12392	4.890924	5.679127	4.350994	8.112096	8.524671	8.353463	2.207465	2.976896	3.720333	0	3.07819	6.082102
LOC107819266	2.39417	1.995723	2.53805	4.972821	10.98013	4.331896	14.93825	21.1799	11.40323	26.62034	16.358	10.53264	16.54388	13.09146	20.07802	2.872027	0.310276	1.218994	36.67859	32.92352	44.3628	34.35612	25.00699	13.5495	110.5209	77.67646	117.5022	43.84825	72.11019	84.8494
LOC107806945	2.709298	1.717698	1.655421	0.426867	0.503794	0.527686	0.362267	0.285169	0.479231	0.172298	0.206496	0.066345	0.043711	0.128509	0.040301	0.672991	1.270771	0.541566	0.21914	0.348764	0.161147	0.262331	0.363662	0.24624	0.024822	0.054635	0.046408	0	0	0
LOC107810149	0.836331	0.834015	0.611105	0.861991	1.136874	0.299719	1.33177	1.553443	1.10381	1.601508	1.495836	1.003514	2.199355	3.00165	2.730666	4.516193	0.784547	2.046773	3.283909	4.07569	1.597349	3.112621	2.229134	1.7546	3.938344	9.790202	1.969789	3.467216	12.3126	13.48998
Novel00707	0.310859	1.345721	0.743114	3.661094	3.36959	1.420399	1.042924	0.39841	0.437772	0.303127	0.708417	0.196967	0.086513	0.678264	0	1.389915	0.976027	0.927035	1.104034	4.273188	0.779649	1.103328	2.399235	0.32491	0	0.108134	0	0	0	0
LOC107775854	1.325804	1.150768	0.987851	3.241093	2.979449	3.031805	3.088913	2.912921	4.603498	5.027656	2.597129	4.498082	2.295849	5.09679	2.505542	2.854194	2.561675	2.494641	2.220869	4.29033	2.072837	2.829545	3.861029	3.519313	0.212853	0.93702	2.362926	0	0.303947	0
LOC107807232	15.13825	11.56198	14.24053	6.503113	5.376994	7.144396	5.305361	5.556383	7.039502	2.702847	3.538316	3.602595	2.452654	2.907585	1.495392	8.394577	11.77538	9.802574	3.714105	3.156388	4.602112	5.519898	8.745233	9.805489	0.494209	0.593346	0.92401	0	0.85541	0
LOC107786347	0	0	0	0	0	0	0	0	0	0	0	0	0.033454	0	0	0	0	0	0	0	0	0	0	0	0	0	0	0	0	0
LOC107774980	5.443778	3.932763	5.136125	4.210062	4.34706	4.782106	3.507971	4.367695	4.362928	3.819437	3.491323	3.645592	3.99079	3.548851	5.314791	4.254598	4.681923	4.701999	3.300908	4.099628	3.814432	2.82754	4.801772	3.191858	2.434087	1.200854	3.138589	4.747647	4.154972	0
LOC107805681	7.548933	6.800842	5.983856	10.29773	11.72163	10.04397	11.69287	9.613238	11.78496	11.22507	10.64748	11.72882	13.84497	11.80108	9.054197	7.026454	7.460557	7.52563	7.924278	8.685773	7.781618	8.792219	8.827187	10.45973	5.521709	3.984196	5.196089	2.068412	6.683816	0
LOC107823878	15.33317	15.94383	17.80155	8.204126	6.556252	12.05952	7.422892	10.02364	10.33768	4.515638	7.865286	7.824497	7.102579	9.318135	6.020404	12.72997	14.91255	16.72533	4.072501	5.832511	6.100451	7.219001	10.71097	13.2297	1.170954	0.859127	4.500237	0	0.990864	0
LOC107800995	1.053538	1.107889	1.268056	0.575119	0.59759	0.900337	0.827004	0.553952	0.87498	0.250248	0.530379	0.387974	0.34583	0.176824	0.457483	1.107189	0.665489	1.117766	0.452294	0.262796	0.197096	1.060317	0.857807	0.663518	0.068307	0.150351	0.175605	0	0.065027	0
LOC107831700	3.824186	4.963875	4.35179	5.375254	5.701344	5.581629	5.481926	5.128967	5.679225	4.429676	4.095415	3.720291	3.792722	4.02029	3.734735	5.060533	4.92598	5.011012	6.185313	5.921027	6.468139	5.496997	5.356209	4.869119	2.080487	3.128148	2.766715	0	2.677963	8.59837
LOC107798063	0	0	0	0	0	0	0	0	0	0	0	0	0	0	0	0	0	0	0	0	0	0	0	0	0	0	0	0	0	0
LOC107799857	3.305247	4.33979	4.431517	2.740625	2.875129	3.011479	2.913215	1.866773	2.892731	2.458246	2.710462	3.502288	2.057996	3.483629	2.587435	2.838797	3.680237	4.260146	2.387548	2.274721	2.810072	1.450326	3.261347	2.400684	1.345727	1.16924	2.383649	16.02521	2.427351	0
LOC107816281	7.815393	9.888522	8.709016	8.247855	5.885595	9.922105	6.624842	7.849831	6.361712	4.49963	4.430766	2.950125	3.609659	3.401413	5.034775	8.302355	8.915811	9.32893	6.580645	8.228939	4.663381	8.158549	8.250431	6.300286	2.995845	3.065697	3.68504	11.89173	4.002775	7.711237
LOC107765986	0	0	0	0	0	0	0	0	0	0	0	0	0	0	0	0	0	0	0	0	0	0	0	0	0	0	0	0	0	0
LOC107817038	0	0	0	0	0	0	0	0	0	0	0	0	0	0	0	0	0	0	0	0	0	0	0	0	0	0	0	0	0	0
LOC107830001	0	0	0	0	0	0	0	0	0	0	0	0	0	0	0	0	0	0	0	0	0	0.064424	0.034022	0.080629	0	0	0.045588	0	0	0
LOC107826729	12.68002	14.83862	12.68161	13.65263	12.76215	12.74039	13.61427	12.84783	11.18779	9.742802	10.96276	8.791485	11.67676	11.02679	14.07057	14.42111	12.71495	13.25221	11.45213	11.93385	8.885205	13.55556	11.85803	11.18885	6.190134	8.036486	5.433682	0	9.108976	15.39315
LOC107820609	3.110203	5.264194	2.961122	0.581427	0.436843	0.424616	0.62111	0.843632	0.648888	0.240702	0.796193	0.417077	0.054957	0.215434	0.405362	1.361205	1.669287	1.214603	0.300573	0.334097	0.225124	0.494745	0.78383	0.773996	0	0	0.175048	0	0.475353	0
LOC107780037	0	0.05316	0	0.634121	0.283875	0.062711	0.489233	0.647896	0.520241	0.028439	0.347655	0.147835	0.454531	0.063634	0.38914	0.043467	0.084527	0.043487	0.710263	0.740139	0.39898	0.608905	0.591679	0.304829	0.221238	0.243483	0.275762	0	0.421227	0
Novel02789	11.03678	8.158329	8.344	5.406497	5.853279	4.676783	6.482931	7.187781	9.112996	8.133038	6.150863	10.69091	14.20728	11.64838	5.682373	8.438943	10.90557	9.086069	4.85184	3.923094	7.246013	5.344118	6.754368	6.725991	11.63578	3.951768	23.66721	20.56771	21.98092	6.668606
LOC107822232	0	0	0	0	0	0	0	0	0	0	0	0	0	0	0	0	0	0	0	0	0	0	0	0	0	0	0	0	0	0
LOC107774550	0	0	0	0	0	0	0	0	0	0	0	0	0	0	0	0	0	0	0	0	0	0	0	0	0	0	0	0	0	0
LOC107766490	4.405363	3.774048	3.944373	6.944402	6.977697	7.293907	7.833331	6.812951	6.907034	7.689455	7.382813	6.289796	9.415893	6.488146	5.064129	4.169259	4.553867	4.236856	4.15734	4.919156	5.182917	6.438308	5.323575	8.057847	5.402626	2.451896	7.341566	3.150457	9.119863	16.34341
LOC107817576	14.03029	16.65234	15.16819	13.85032	11.045	11.82469	13.58032	11.86385	12.7786	8.963615	10.43738	9.346302	9.712329	8.568043	10.89127	15.65905	16.56135	15.28148	9.327671	10.66815	8.677655	13.15813	12.63169	11.90012	2.528254	5.81176	4.250502	2.306585	3.571441	0.997143
LOC107794276	1.814172	2.366623	2.133617	4.124982	3.840126	4.187745	3.878649	3.206533	3.206069	3.052472	3.479563	2.569464	4.217319	3.647886	4.527255	2.677337	1.907287	2.532701	3.032064	3.70125	2.15741	3.238074	3.059243	2.844258	1.933874	2.128321	2.144205	0	3.168241	2.19947
LOC107832356	0	0	0	0	0	0	0	0	0	0	0	0	0	0	0	0	0	0	0	0.097491	0	0	0	0	0	0	0	0	0	0
LOC107828997	6.360863	7.867551	7.61698	6.174236	3.962931	6.629358	4.627409	4.880313	5.849984	5.063384	5.734596	3.454608	4.841093	3.965365	6.262142	6.674867	7.524647	7.548957	3.556597	4.227813	2.24945	6.558846	3.721384	3.934751	1.805361	1.354695	2.68501	0	1.562421	0
LOC107770219	0.323832	0.184458	0.14075	0.073344	0.107455	0.174079	0.135806	0.069173	0.038003	0.157888	0.028384	0	0	0	0.124639	0.090495	0	0.090536	0.08215	0	0.036917	0	0.07141	0.084617	0.051178	0	0.095686	0	0	0
LOC107766518	6.466281	6.814033	5.621001	13.36384	7.688656	7.665868	1.731177	1.874542	2.208806	1.477838	1.953332	0.865771	0.64271	0.456646	0.592573	4.345417	4.239016	4.035341	4.56964	4.444398	4.475673	1.808089	4.748829	4.178839	0.291977	0.18075	0.835912	1.032211	0.347444	0
LOC107798697	10.86183	9.508463	8.820779	10.13159	11.69792	10.64063	8.840575	7.448939	8.352613	11.81109	9.545459	10.29324	10.97785	10.83626	8.654592	11.10297	14.15234	11.29452	8.846475	7.405096	9.77583	7.041961	8.540828	8.701312	5.872519	5.667543	6.292218	12.7759	9.116825	4.418444
Novel05158	2.034625	2.458038	1.480735	2.014734	1.172334	1.729628	1.389034	0.970291	1.288271	1.522617	2.189788	0.879441	1.474866	1.187268	1.068424	2.080374	2.559876	1.904943	1.200347	1.601072	1.078844	0.829826	1.168625	1.186934	0.538406	0.197514	1.118494	0	2.050202	0
LOC107767287	0.838076	1.145705	0.884634	1.084649	0.635644	0.836675	1.255233	0.613779	0.449613	0.81723	0.671611	0.404589	0.466479	0.195921	0.798737	1.249071	0.809652	0.937232	0.425211	0.911513	0.600553	1.549777	1.161661	0.875957	0.454107	0.499767	0.424517	0	0.864599	0
LOC107823739	41.60965	36.66808	32.376	21.7065	17.46486	23.36242	33.58658	30.64723	41.24762	17.48828	24.05332	21.52469	32.21832	28.15779	21.28046	24.8909	34.56056	27.02466	9.725159	10.35498	11.03411	31.02475	33.43816	37.0437	3.539142	2.970759	7.62643	0	3.197869	5.867246
LOC107797582	0	0	0	0	0	0	0	0	0	0	0	0	0	0	0	0	0	0	0	0	0	0	0	0	0	0	0	0	0	0
LOC107821006	13.35703	12.84565	11.64474	82.77085	100.7379	116.5116	94.18436	92.59477	110.893	96.20931	97.71637	134.5598	70.36952	83.06811	69.66234	15.33561	23.59485	16.35582	39.28236	50.22201	47.38169	49.09412	42.16099	76.62189	18.851	10.1571	17.25549	2.776793	20.18894	9.60332
LOC107791182	0	0.513309	0.293759	15.10345	25.1182	9.325221	33.35141	59.76917	29.71737	90.1809	46.83899	69.85135	43.38757	56.5293	74.91877	1.510973	0.217648	1.007776	46.75034	58.12667	55.57899	55.69091	32.59009	34.49669	239.4031	257.6753	455.7276	217.5035	120.3931	153.2297
LOC107802075	1.375683	1.277421	1.331231	1.740115	2.756138	1.925526	2.133523	2.06253	2.168832	2.531065	3.585509	3.333144	4.478707	3.681213	2.504236	2.727313	3.009113	2.496344	2.89725	2.568995	4.142698	2.861333	2.472676	4.449236	6.530508	2.564255	9.909068	3.712481	8.872355	9.629493
LOC107818209	0.051302	0	0	0.046477	0	0	0.04303	0	0	0	0	0	0.114221	0.055968	0	0	0	0	0	0	0	0	0	0	0	0	0	0	0	0
LOC107797369	0	0	0	0	0	0	0	0	0	0	0	0	0	0	0	0	0	0	0	0	0	0	0	0	0	0	0	0	0	0
LOC107775077	37.29889	38.58221	40.81575	78.16637	73.23281	63.4361	70.50217	67.72723	72.42221	37.3245	33.47233	31.0775	39.34488	41.43788	38.98499	38.7056	39.57585	41.82119	63.2211	60.56138	62.52836	73.25999	76.58002	76.25792	43.44884	35.40401	34.7821	24.47154	49.77599	60.45207
LOC107805922	2.569456	1.862109	1.641542	4.403183	5.396433	4.426624	3.193723	3.544402	4.098036	6.55064	5.730722	5.989173	9.201382	8.983591	3.717526	2.076248	2.392589	2.515718	4.020907	2.749714	4.376206	2.869711	2.321043	3.462153	3.52255	2.110667	3.293014	4.42777	4.769264	1.914137
LOC107776464	6.365708	7.480182	7.190839	7.172169	7.291185	6.461767	7.670009	5.494252	6.67416	6.081331	5.483251	5.978508	6.366964	7.085683	5.206972	5.634688	5.743984	5.95045	7.148055	5.330282	6.306627	6.638465	6.72657	8.510995	6.08721	3.372114	7.485563	13.86512	8.556209	7.991905
LOC107830560	0.218522	0.219072	0.113974	0.455331	1.933628	0.070481	1.447943	2.894025	0.697538	0.617951	0.858076	0.461534	2.165031	5.745366	6.504269	0.358255	0.211111	0.195501	2.926984	1.737634	1.713945	3.211887	0.98303	0.319759	4.779617	12.25352	0.180793	23.44114	12.62452	83.77164
LOC107804150	0.179199	0.146986	0.140196	0.438331	0.23785	0.288991	0.405814	0.413403	0.252359	0.366957	0.917276	0.37848	0.359076	0.234596	1.140332	0.761172	0.276994	0.467601	0.600067	0.773108	0.424924	0.987713	0.774519	0.842843	0.521092	0.473749	0.360057	0	0.086273	1.108012
LOC107773435	0	0	0	0	0	0	0	0	0	0	0	0	0	0	0	0	0	0	0	0	0	0	0	0	0	0	0	0	0	0
LOC107762736	11.68787	11.41853	11.77212	5.118482	4.522325	8.253608	4.756864	4.551012	4.393283	3.07008	5.912281	2.532322	3.048726	2.540768	2.899428	9.818627	9.728991	11.06106	3.501119	5.271921	1.750366	6.663307	7.475318	5.048743	1.33593	1.020177	2.191908	1.542155	0.830547	0
LOC107781274	0.136821	0.145478	0.079291	0.165271	0.322849	0.196133	2.907201	2.006849	0.342544	0.378018	2.014723	0.327506	0.507705	0.348286	2.270279	0.57777	0.264362	0.272016	0.277674	0.270063	0.311958	3.218409	0.80457	0.309845	0.317137	0.856696	0.080856	0	0.439138	4.229934
LOC107817215	7.127359	8.725005	8.413635	6.05585	5.0566	6.914641	5.730412	5.134898	5.246278	2.79648	4.169686	3.010695	4.953054	3.79578	3.571091	7.27556	8.00607	7.907738	4.129715	4.673565	3.096283	6.216396	7.812002	5.752435	2.026175	1.349678	2.417528	0	1.015197	0
LOC107827648	0	0.024607	0	0	0.047783	0	0	0	0	0	0	0.04562	0	0	0	0.02012	0	0	0	0.04568	0	0.022548	0	0.02822	0	0	0.031912	0	0	0
Novel00042	0	0	0	0	0	0	0	0	0	0	0	0	0	0.067149	0	2.201652	3.092091	3.120436	3.060419	2.603389	3.873342	2.107511	2.28026	1.929987	0.389095	0.25693	1.091222	0	0	0
LOC107802850	0.066811	0.020297	0.077436	0	0.059119	0	0.037358	0.019028	0.020908	0	0.031232	0.037629	0.074375	0	0	0.116171	0.107575	0.099621	0.112992	0.131874	0.101554	0.167387	0.078576	0.046554	0	0	0.078965	0	0.214434	0
LOC107806934	18.99182	8.023566	10.87777	30.8999	21.11675	42.68246	7.755643	8.799548	17.10258	12.36428	11.49228	19.41282	10.78671	16.97399	4.773717	7.509609	9.361067	8.916584	14.15871	14.14537	15.95741	4.022941	9.719112	14.29449	2.030121	2.157202	10.6017	0	5.064822	0
LOC107764798	4.468416	2.26245	2.589533	2.586323	2.30647	3.60307	1.353391	1.69686	1.514914	1.694494	1.392558	2.411832	1.243576	2.301999	0.764377	0.462482	1.319042	0.462694	0.629755	0.629995	0.792414	0.51829	0.218969	1.037864	0.470786	0.172708	1.320327	35.5061	0.597571	0
LOC107815540	0	0	0	0	0	0	0	0	0	0	0	0.023266	0	0.120176	0	0	0	0	0	0.05589	0	0.022999	0	0	0	0	0	0	0	0
LOC107780685	105.9423	82.74534	96.69561	81.33412	80.38821	106.0895	54.23147	56.20804	71.59665	55.19599	54.89562	66.47298	56.80511	53.94861	48.67407	69.46277	99.08444	89.18821	63.61018	56.12559	70.57813	56.25535	75.86138	91.36326	28.55462	27.97752	45.19998	24.16706	31.45406	24.37746
LOC107802937	0	0	0	0	0	0	0	0	0	0	0	0	0	0	0	0	0	0	0	0	0	0	0	0	0	0	0	0	0	0
LOC107783911	0	0.071038	0.067757	0	0	0	0	0	0	0	0	0	0	0	0	0.116171	0	0.174336	0	0	0.142176	0	0	0	0.098548	0	0.092126	0	0	0
LOC107792111	3.21743	3.26741	2.796844	3.08139	2.912068	3.380065	3.007008	2.607679	2.779012	2.707129	2.745952	2.298799	3.929623	3.690575	2.585215	3.055269	2.397838	3.550121	3.768542	3.281622	2.414551	3.009395	3.162327	3.7664	1.743347	1.790728	1.629747	2.62962	3.097971	2.273583
LOC107807800	1.19823	1.341648	1.031676	1.312981	1.252131	1.153285	1.943013	1.462572	0.996385	1.313088	1.856441	0.665224	1.219553	1.369458	1.370382	1.539228	1.047344	1.607996	1.042184	1.361147	0.541199	1.658258	1.389092	0.978057	0.663682	0.841564	0.552997	0	0.714221	1.411207
LOC107779914	1.638396	1.136612	1.254166	0.886151	0.757338	0.709849	0.840924	0.793967	1.147909	0.524599	0.908787	0.516479	0.843887	0.533555	0.87845	0.965814	1.157591	0.948025	0.397022	0.517153	0.535252	0.980251	0.582383	0.766769	0.309169	0.340255	0.491339	0	0.235457	0
LOC107769854	7.514665	7.109869	8.635973	5.336706	3.888249	4.998139	3.605457	4.162594	5.171746	3.943322	4.53248	3.577811	2.445825	2.952716	2.418435	6.833822	9.474652	7.715309	2.972599	3.151509	2.584583	4.946097	6.544172	8.087293	2.133659	0.753194	4.892478	2.277139	1.839573	0
LOC107796475	0.487254	0.697827	0.363051	3.058459	3.634013	1.047714	9.837525	8.831989	4.378499	3.563503	7.687407	3.028534	2.402135	2.468002	9.394806	1.893317	0.907832	1.037909	5.403428	5.417262	5.269118	14.2712	8.779994	5.674804	5.36832	8.958993	2.838337	7.466897	3.853834	12.91184
LOC107783862	14.43369	18.2612	15.29057	19.53397	15.90993	18.37756	24.06359	25.57411	20.47631	15.94207	21.60952	16.89781	15.71248	16.2881	19.619	17.20432	15.6572	15.83734	18.96464	21.83984	16.85092	19.87273	21.83432	18.05142	13.94424	14.21137	16.30499	15.2169	10.07333	4.385538
107783319	0	0	0	0	0	0	0	0	0	0	0	0	0	0	0	0	0	0	0	0	0	0	0	0	0	0	0	0	0	0
LOC107794115	0.144896	0.231095	0.157443	0.262536	0.19232	0.584177	0.303825	0.123803	0.102026	0.423875	0.203202	0.183618	0.483899	0.118555	0.148717	0.485894	0	0.216052	0.14703	0.122572	0.132147	0.121006	0.127808	0.151445	0	0.100806	0.128441	0	0	0
LOC107809062	8.344509	9.152573	8.738857	13.54329	13.1267	11.51381	11.84832	10.89494	10.85637	10.2296	11.96532	7.973787	10.18219	10.46726	12.13582	10.61232	8.433876	10.59388	13.67897	14.26757	9.444033	13.35453	12.62572	11.31314	11.28757	12.35004	8.274189	8.94008	12.13729	14.81515
LOC107766803	0	0	0	0	0	0	0	0	0	0	0	0	0	0	0	0	0	0	0	0	0	0	0	0	0	0	0	0	0	0
LOC107793895	14.78748	26.14207	18.42987	49.06747	39.25482	9.002682	209.1557	216.0099	124.8368	57.33119	107.3961	29.55142	66.11098	84.87336	185.6649	39.78581	16.35139	18.28594	47.66749	62.59621	36.93875	158.3602	196.8055	82.36925	55.78729	91.80565	52.92419	63.70567	20.5857	95.47246
LOC107826332	0	0	0	0	0	0	0	0	0	0	0	0	0	0	0	0	0	0	0	0	0	0	0	0	0	0	0	0	0	0
LOC107785685	0.030453	0.166527	0.026472	0.110357	0.026947	0.065482	0.996159	0.494383	0.142955	0.296959	1.452066	0.205823	1.118736	0.797355	1.437798	0.022694	0.02942	0.022704	0	0.051523	0	0.63581	0.107447	0	0.269516	0.211868	0.035993	0	0.293227	3.765953
LOC107831810	23.72227	29.6707	27.9757	20.06344	14.20197	19.57413	24.80154	26.68272	24.10151	18.15797	19.30948	19.7259	18.88261	17.69058	17.83525	28.8656	27.24482	28.38411	13.76128	17.89227	15.35639	21.716	25.82653	24.49108	10.2246	15.06125	16.42075	2.96587	9.783504	7.692921
Novel03441	2.320058	2.033122	1.86164	1.131768	0.710633	1.199205	0.860701	1.029282	1.298526	1.218188	1.094979	1.73389	1.589366	0.924814	0.87007	1.72891	2.112061	2.062336	0.679106	0.75485	0.610365	1.192333	1.456124	1.352361	0.338453	0.310403	0.896463	0	0.2148	0
LOC107810120	16.02102	18.93138	17.37694	11.07596	8.408458	11.24424	6.536516	7.525125	10.77094	6.610614	8.471171	8.346178	7.159731	10.05073	5.054976	15.43635	13.63041	14.94664	5.585597	6.543519	4.491763	8.855197	10.70731	14.17132	1.575016	1.169024	5.238952	0	2.650067	0
LOC107816437	0.527635	0.4153	0.813082	0.304189	0.275887	0.257851	0.44255	0.655746	0.315234	0.163708	0.269075	0.121571	0.133493	0.052329	0.196927	0.500429	0.44023	0.375494	0.243367	0.405766	0.262479	0.640933	0.655803	0.451214	0	0.166856	0.141732	0	0.115465	0
LOC107806965	17.82966	19.95623	19.2844	29.06754	27.04604	24.16009	30.87381	28.74831	23.95181	30.12535	29.32536	23.94808	27.30274	27.11527	31.58204	21.27706	17.82028	20.85801	24.47047	25.71699	23.56714	25.11403	24.71376	26.19283	24.15226	22.75081	22.38423	2.937956	19.38285	25.40172
LOC107762561	0.131965	0.148024	0.052945	0.165535	0.089824	0.076396	0.068113	0.05204	0.038121	0.108885	0.092534	0.060032	0.124304	0.099669	0.083351	0.045388	0.078455	0.068113	0.103007	0.051523	0	0.050865	0.053724	0.06366	0.038502	0.056498	0.035993	0	0	0
LOC107815464	3.306051	3.612814	3.527654	1.419486	1.233941	1.516099	2.615226	2.115238	1.64755	1.283416	2.406099	0.728045	1.203524	1.384578	2.219276	4.168395	3.284746	3.971718	1.14475	1.736335	0.943135	2.656289	1.782863	1.146369	0.356575	1.177284	0.407416	1.120519	0.528036	0
LOC107794048	0.116141	0.045364	0.15865	0.090187	0.073407	0.08919	0.041748	0.014176	0.031154	0	0.011634	0.014017	0	0.036201	0.119204	0.074185	0	0.037109	0.033672	0.014035	0.030264	0.055424	0.043905	0.069366	0	0.023086	0.03922	0	0	0
LOC107821423	0	0.02945	0.028089	0.087822	0	0.138963	0.054205	0.027609	0	0	0.022658	0	0	0	0.033165	0	0.031217	0	0	0.109339	0	0.107943	0.028502	0	0.040854	0.089923	0	0	0	0
LOC107810675	0	0	0	0	0	0	0	0	0	0	0	0	0	0	0	0	0	0	0	0	0	0	0	0	0	0	0	0	0	0
LOC107796944	0	0	0	0	0	0	0	0	0	0	0	0.122922	0	0	0	0	0	0	0	0	0	0	0	0	0	0	0	0	0	0
LOC107764208	0.998767	1.314809	1.495245	1.256711	1.37476	1.014141	1.023866	0.805964	1.666998	1.785527	1.245041	1.359433	2.532527	1.33174	1.252908	1.778007	1.554549	1.075566	1.91435	1.220394	2.226625	1.251144	1.125694	1.913836	1.12244	0.463237	0.983718	7.936215	2.404209	3.430847
LOC107770007	0	0	0	0	0	0	0	0	0	0	0	0	0	0	0	0	0	0	0	0	0	0	0	0	0	0	0	0	0	0
LOC107802839	3.282968	2.229056	2.111823	2.037329	2.251365	2.011868	4.915117	5.427786	6.488021	2.352962	4.73065	2.537792	2.905444	2.131027	4.970085	3.168181	1.93469	2.533258	2.032111	2.86048	1.272502	7.416279	7.760731	6.914186	0.664107	1.233363	0.911848	0	1.106377	0
LOC107766532	2.569573	2.311825	1.861396	2.118989	2.098825	2.833441	3.01177	3.096236	2.257786	2.441411	2.725779	2.894462	3.117195	2.479823	1.014361	3.01957	2.896161	3.34024	3.677138	3.316259	2.734076	2.696153	3.312626	4.200737	3.581924	2.429421	3.504264	7.067724	5.075216	6.110792
LOC107804480	0	0	0	0	0	0.07057	0	0	0	0	0	0	0.07307	0	0	0	0	0	0.066606	0	0	0	0	0	0	0	0	0	0	0
LOC107829897	12.52676	12.1179	9.551427	12.30514	13.19056	11.15904	9.155869	9.158886	7.577236	10.22585	11.35616	9.328121	7.51036	7.008748	8.02652	12.48217	10.71883	13.12829	17.77765	18.49895	19.86251	9.310669	10.57589	7.650034	10.67909	14.64126	8.269999	10.23818	8.701625	23.23646
Novel04330	0.005273	0	0	0	0	0.005669	0	0	0	0	0	0	0	0	0	0.003929	0	0.003931	0	0	0	0	0	0	0	0	0	0	0	0
LOC107794342	11.69903	22.61684	17.79701	10.19726	8.391454	6.765381	16.13113	16.96286	21.88393	13.65507	14.04516	14.67575	15.19411	18.85454	10.73404	19.68185	21.91951	14.14042	4.85647	8.84694	5.577351	7.919757	11.17926	7.966614	3.697784	2.589738	8.065934	6.338229	2.986842	0
LOC107819189	13.51561	12.76303	11.89038	13.17877	10.32647	13.072	9.150845	8.579915	12.28137	12.49134	11.72274	14.9952	13.89769	13.20524	7.855256	10.79993	10.43528	12.82826	12.88852	12.67303	10.98986	9.338035	11.01197	13.27551	8.715483	11.63101	11.0345	31.05404	7.578311	0
LOC107820045	3.849431	4.343587	4.34213	5.397151	4.014485	4.532763	3.958981	3.837235	2.839584	2.413081	3.37403	2.43908	1.989576	3.149655	2.963213	3.585751	3.763147	2.869914	2.929603	4.303039	1.964346	2.717238	2.667856	3.017568	1.854039	0.8927	1.299919	6.554494	0.882502	0
LOC107822764	0	0	0	0	0	0	0	0	0	0	0	0	0	0	0	0	0	0	0	0	0	0	0	0	0	0	0	0	0	0
LOC107778321	0	0.182903	0.249221	0.207787	0.202952	0.061647	0.336653	0.318452	0.242248	0.279567	0.542789	0.411758	0.351072	0.250219	0.912203	1.410076	0	0.23512	1.250963	1.188382	1.621136	0.526744	0.227598	0.299657	0.978679	1.795138	1.016563	2.050298	0.690134	3.545396
LOC107765486	3.531486	3.261716	3.4407	4.659453	3.397533	4.994421	3.518629	3.807186	4.272341	4.46056	4.304407	4.32507	4.247914	3.33554	2.870505	3.302831	4.258916	3.922804	3.126551	3.22799	2.939768	2.949236	4.494805	5.326096	1.678073	0.725528	2.82931	0	2.053913	0
LOC107808332	0	0.125626	0	0.124877	0	0	0	0	0	0	0	0	0	0	0	0	0	0	0	0	0	0.115115	0	0.144072	0	0	0	0	0	0
LOC107782990	0	0	0	0	0	0	0	0	0	0	0	0	0	0	0	0	0	0	0	0	0	0	0	0	0	0	0	0	0	0
LOC107823378	0.166815	0.114023	0.145008	0.188907	0.036902	0.269019	0.209872	0.178164	0.274072	0.975991	0.175456	0.352324	0.510676	0.13649	0.214017	0.062155	0.120868	0.155459	0.253908	0.282227	0.646585	0.069656	0.220712	0.130766	0.369082	0.058028	0.345032	0	0.803105	0
LOC107827140	3.890034	7.502815	5.268886	3.852004	2.481556	3.112381	5.387313	5.79724	4.926136	4.410784	8.563691	3.744978	2.114866	1.085632	2.321293	9.977465	4.806872	7.081848	4.222733	6.504874	2.805219	7.857246	7.50091	4.160417	7.891972	9.94426	6.308482	0	0.87107	0
Novel03853	0.699114	0.955734	0.841465	0.609002	0.808967	0.780542	0.699142	0.781139	0.757337	0.471963	0.490219	0.454331	0.538798	0.674695	0.303579	0.621169	0.545518	0.681594	0.518417	0.409432	0.220709	0.62876	0.403204	0.871235	0.237971	0.037414	0.063561	0	0.129453	0
LOC107769459	0.168967	0.030799	0	0.030615	0.089708	0.326988	0.028344	0.057748	0.158634	0.131811	0.047392	0.057099	0.037619	0.221202	0.104054	0	0.261178	0	0.068582	0.057174	0.06164	0.084665	0.14904	0.035321	0	0.094042	0	0	0.162693	0
LOC107786232	8.48675	8.329598	8.044986	13.39411	15.18917	14.8631	10.53236	11.51679	10.41935	17.1879	13.51746	14.5019	15.4752	15.04003	14.97745	7.354545	8.940898	8.188183	13.52164	14.29344	15.19993	11.48112	9.010148	10.03433	15.19655	12.07586	16.43031	8.238769	17.19374	26.11871
LOC107778247	3.396905	2.835903	2.930317	4.792325	3.969499	4.76723	5.437273	5.20661	4.406403	5.714542	6.091149	5.213835	5.513459	4.72426	5.243043	3.941993	4.083332	2.764525	4.683635	4.628387	4.280491	5.132306	5.054818	5.041089	2.62273	2.88644	3.493855	1.854403	3.620332	3.206653
LOC107802019	7.654407	9.703387	8.883549	10.27266	9.529208	8.141877	14.99157	12.6976	10.18696	7.781631	12.74728	7.515768	8.339695	8.661508	12.87092	11.37781	9.538507	8.726979	7.477961	10.05707	6.795285	13.32882	11.79751	9.340874	4.349726	7.393068	4.258777	7.279234	5.390445	7.552397
LOC107801153	0.045787	0.166915	0.039801	0	0	0	0.038403	0.078242	0	0.089295	0.064211	0	0.254851	0.149852	0.046994	0.06824	0.044234	0.068272	0	0	0	0.038238	0.12116	0	0	0	0	0	0	0
107797426	0	0	0	0	0	0	0	0	0	0	0	0	0	0	0	0	0	0	0	0	0	0	0	0	0	0	0	0	0	0
LOC107810542	0	0	0.066277	0	0	0	0	0	0	0	0	0.032207	0	0.041589	0	0	0	0.028422	0	0	0	0	0	0	0	0	0	0	0	0
Novel01603	0.410362	0.267139	0.254799	0.265548	0.907788	0.126054	0.295018	0.375669	0.770532	1.028969	0.986558	0.420975	3.785085	0.671527	1.233468	0.87372	0.622984	0.895974	0.297433	0.148773	0.347524	0.514056	0.206838	0.398274	0.815292	0.040785	0.588946	0	0.705581	0
LOC107806136	0	0	0	0.050698	0.049518	0	0	0	0	0	0	0.047278	0	0	0	0	0	0	0	0	0	0	0	0	0	0	0	0	0	0
LOC107770330	113.4836	80.76946	58.60359	68.40102	52.93897	68.43927	60.85799	52.90568	86.86615	55.12292	63.436	79.60544	93.24844	88.40451	33.99656	63.47892	110.5597	84.95054	37.28152	33.80975	56.30989	51.4144	73.10429	83.95576	7.532581	5.329264	18.65224	12.68075	22.70769	17.54215
LOC107811582	2.052889	1.644172	1.405995	0.676296	0.330278	0.802584	0.260886	0.425223	0.292022	0.303307	0.087242	0.157667	0.346258	0.067867	0.255397	0.927161	1.562571	1.205861	0.189375	0.105249	0.340413	0.207809	0.438978	0.585185	0	0	0	0	0	0
LOC107771705	75.40017	78.59996	69.53149	148.3297	89.45073	164.9983	63.59115	54.30426	105.2438	97.35514	152.471	62.64318	119.4053	82.16688	35.18445	61.78226	72.52059	77.66738	35.03927	40.78183	29.89505	32.94709	56.57589	74.97243	32.1629	2.321102	33.41885	0	62.2406	46.41477
107769387	0	0	0	0	0	0	0	0	0	0	0	0	0	0	0	0	0	0	0	0	0	0	0	0	0	0	0	0	0	0
LOC107767848	0	0	0	0	0	0	0	0	0	0	0	0	0	0	0	0	0	0	0	0	0	0	0	0	0	0	0	0	0	0
LOC107775850	0.058103	0	0	0	0	0	0	0	0	0	0	0	0	0	0	0	0	0	0	0	0	0	0	0	0	0	0	0	0	0
LOC107786884	2.534505	2.679471	2.541014	3.597275	2.721138	3.269883	2.876914	2.916273	2.712635	2.570321	2.63025	1.969922	2.520492	2.28575	2.167788	2.291642	2.220014	2.431261	3.120499	3.630535	2.373151	3.301944	2.474065	2.401818	0.74769	1.739779	1.338026	6.041764	1.220201	3.134245
LOC107799420	40.61889	48.18563	86.21289	32.72759	41.47185	36.3856	33.71546	33.29605	46.86922	49.33096	47.7818	46.56531	55.84773	41.70881	37.186	125.5245	42.25982	117.4419	32.27556	38.63732	32.4665	40.95842	30.0068	58.19654	20.24003	16.75519	55.0622	32.63848	33.29654	23.87796
LOC107809182	0	0	0	0	0.168542	0	0	0	0	0	0	0	0	0	0	0	0	0	0	0	0	0	0	0	0	0	0	0	0	0
LOC107764270	1.967755	2.732748	2.850879	3.735152	1.575371	1.108159	3.065104	2.241738	2.287276	1.736066	1.839721	0.870788	2.190532	2.044499	1.634955	2.58361	2.896787	3.702542	1.806576	2.774309	1.623709	3.13014	4.215247	1.860821	1.184686	1.303804	1.10749	0	0.902235	0
LOC107819412	0.05505	0.025085	0.143561	0.049872	0	0.088778	0	0	0.025842	0	0.0193	0	0.030641	0	0	0.020512	0.026591	0.123126	0.055861	0.023284	0.050206	0	0.024279	0.028769	0	0.076598	0	0	0	0
LOC107773392	4.550474	5.739309	5.070065	6.560262	4.250949	6.528137	4.383863	4.766772	5.198046	1.730954	2.094268	0.892655	1.458531	2.336162	4.381288	4.850312	4.151155	4.232837	5.518135	4.862385	3.096521	6.012113	7.356597	5.81635	0.587786	1.11735	0.799251	4.030009	1.085206	2.613276
LOC107821516	4.640335	5.643156	4.196094	5.297185	5.250181	3.398031	7.7118	6.02703	6.919229	5.365454	9.247659	5.57093	8.140354	6.770734	7.475838	5.588444	4.760537	3.845153	1.807671	2.017391	2.096378	7.282619	4.62531	3.693867	1.05348	0.699641	1.086713	0	2.489932	1.776588
LOC107795544	6.870474	6.150765	7.10869	3.277397	3.147324	3.039618	6.32352	5.584596	6.935503	3.112636	3.858328	2.671036	2.808892	3.250196	3.619439	6.728344	5.727008	5.98349	2.930574	2.545942	3.049817	7.413327	7.266891	6.958596	2.536727	2.284191	3.125974	6.522159	3.073518	5.639094
LOC107820037	15.09369	12.7758	12.71547	10.78339	7.486912	15.95774	5.202216	5.330127	7.808988	7.411567	9.678684	10.60107	9.977735	10.79512	5.703613	14.0264	13.12706	14.99507	7.967247	7.066498	8.632172	5.149841	6.451261	9.592958	4.260815	2.145074	11.99189	0	9.148026	0

LOC107791496	14.309	11.28948	12.6191	7.810874	6.640991	8.208481	8.189821	7.987947	12.16611	11.34479	11.69055	10.64064	10.00259	9.235925	4.771069	7.973121	11.64101	10.2033	5.243987	6.568464	5.068439	8.132816	10.42246	13.62576	9.620037	4.552905	11.57143	0	9.376852	4.817134
LOC107776819	0	0	0	0	0	0	0	0	0	0	0	0	0	0	0	0	0	0	0	0	0	0	0	0	0	0	0	0	0	0
LOC107828795	4.89672	6.891969	4.712994	10.53707	12.6236	8.412462	10.34817	10.06722	7.317695	16.65807	16.55907	8.555249	15.60025	14.45116	16.94285	7.689321	4.399011	6.330411	11.11589	10.77849	5.598478	8.305234	6.13598	5.424078	17.11764	15.31265	8.913988	3.610152	21.38722	15.60678
LOC107798307	4.389734	6.52746	6.082492	11.2728	8.119117	7.664754	10.32582	10.5189	9.853878	10.07376	8.470518	8.560382	9.736685	7.849613	8.909378	7.649279	5.293094	6.447038	11.55464	10.80519	11.70952	9.371683	10.77183	9.659261	16.14913	16.30329	18.13959	11.80181	15.57223	6.122343
Novel03909	0.578782	8.879387	2.096345	0	0	0	0	0	0	0	0	0	0	0	0	9.057488	9.78516	7.191774	0.097885	0	0	0	0	0	0	0	0	0	0	0
LOC107815267	0	0	0	0	0	0	0	0	0	0	0	0	0	0	0	0	0	0	0	0	0	0	0	0	0	0	0	0	0	0
LOC107783090	0	0	0	0	0	0	0	0	0	0	0	0	0	0	0	0	0	0	0	0	0	0	0	0	0	0	0	0	0	0
LOC107822854	0.250621	0.380683	0.07262	0	0.073922	0.089816	0	0.214137	0	0.081462	0	0.070577	0	0	0	0.124509	0.242121	0	0.084771	0	0.07619	0	0.073688	0	0	0	0	0	0	0
LOC107771803	0	0	0	0	0	0	0	0	0	0	0	0	0	0	0	1.888716	1.632363	2.069541	1.347154	1.327248	2.751799	1.814255	1.809772	2.901355	0.457769	0.503797	1.854407	0	0.581048	7.462487
LOC107791058	0	0	0	0	0	0	0	0	0	0	0	0	0	0	0	0	0	0	0	0	0	0	0	0	0	0	0	0	0	0
LOC107771294	0	0	0	0	0	0	0	0	0.049774	0	0	0	0	0	0	0	0	0.079051	0	0	0.048351	0	0	0	0	0	0	0	0	0
LOC107763850	19.70598	10.54401	13.55664	5.374982	6.824861	8.018914	10.05913	10.10239	17.02873	12.93452	10.66797	20.94446	39.39219	29.68146	8.742712	16.13762	20.18398	19.39929	10.70768	7.062302	10.12626	13.5195	13.38225	23.87443	10.34079	5.012154	10.41829	3.030658	41.00901	5.240647
LOC107817712	6.405944	7.277419	7.407468	6.127374	5.852953	6.310572	9.021542	7.459115	8.811476	5.229715	7.019824	7.450828	12.20594	8.093739	7.736958	7.79337	7.627908	6.353061	3.567611	2.621278	3.233661	9.057286	7.22734	8.003447	1.958842	2.114342	2.605939	2.13077	3.155771	1.842275
LOC107802526	0.929131	3.810548	2.62494	4.208728	1.130465	2.497324	2.435319	1.686984	0	0.566263	3.176098	0.098119	0	0.253409	4.887377	5.625665	2.917261	1.55859	2.12134	3.242171	0.105923	4.364671	2.356221	0.121391	0	0	0.13727	0	0	0
LOC107762111	0.383735	0.582878	0.833931	0.753229	0.735699	0.893883	0.482782	0.655746	0.5404	0.311825	0.493304	0.540316	0.99675	0.558181	1.050279	0.905538	0.679653	1.09668	0.778775	1.190248	0.524959	0.427289	1.015438	0.935851	0.404298	0.533939	0.529134	9.147481	1.231623	0
LOC107794914	0	0	0	0	0.089911	0	0.042612	0	0	0	0.035624	0	0	0	0	0	0	0.037877	0	0	0	0	0	0	0	0	0	0	0	0
LOC107768862	5.560216	6.545458	5.74879	8.280973	6.336421	6.589289	8.62058	7.810026	6.109978	5.380838	8.698519	4.910944	6.682073	5.560429	8.279261	4.331819	3.397967	3.674311	4.252964	5.024257	2.708381	4.34447	5.015944	3.984424	2.236756	3.164987	2.115897	4.518569	2.433533	1.30226
LOC107771546	0	0	0	0	0	0	0	0	0	0	0	0	0	0	0	0	0	0	0	0	0	0	0	0	0	0	0	0	0	0
LOC107798562	20.05892	16.72236	15.21616	13.88586	12.83543	16.40719	17.71859	14.19699	20.41719	19.58164	21.32384	22.97044	28.98184	29.03232	17.00841	16.6364	25.68791	18.64792	10.59421	11.50022	11.34516	11.89131	13.24548	11.77137	3.370175	4.945385	4.016974	6.354357	5.347218	1.373503
Novel00478	0	0	0	0	0	0	0	0	0	0	0	0	0	0	0	0	0	0	0	0	0	0	0	0	0	0	0	0	0	0
LOC107800205	16.73929	14.91803	13.85308	30.0501	30.8265	28.55578	18.90991	18.6302	16.12152	22.82799	25.07775	20.19514	20.83478	20.9572	28.08513	14.63755	17.00699	15.51922	28.70675	25.44671	26.92765	22.80038	23.75501	21.56321	24.5216	14.95469	36.42974	13.25198	15.16617	26.73471
LOC107766343	2.95522	1.868833	2.070856	1.529874	0.960602	0.51873	6.449601	4.508964	1.330623	1.058588	2.177926	0.356665	1.141356	0.921143	1.330875	4.764015	2.534533	2.158278	0.734388	0.714261	1.54012	3.651605	3.324871	1.418324	0.41938	0.419589	0	4.313061	0	0
LOC107818608	0	0	0	0	0	0	0	0	0	0	0	0	0	0	0	0	0	0	0	0	0	0	0	0	0	0	0	0	0	0
LOC107807624	0.219392	0.124968	0.071517	0.049689	0	0	0.069005	0.070295	0.051494	0.133709	0.134608	0.046337	0.030529	0.089755	0.19703	0.163491	0.264939	0.122674	0	0	0.025011	0.068707	0.048379	0.057327	0	0	0	0	0	0
LOC107759457	0	0	0	0	0	0	0	0	0	0	0	0	0	0	0	0	0	0	0	0	0	0	0	0	0	0	0	0	0	0
LOC107800000	14.64534	12.78222	16.4216	9.464751	7.809268	10.25768	10.003	9.293324	10.48022	7.489415	6.924267	7.093197	5.204343	7.286088	6.022424	11.98021	13.77994	13.94182	5.034373	6.981397	6.308586	9.242751	10.8563	12.46523	5.247252	1.327552	6.540442	3.411561	3.445011	11.79861
LOC107825407	0.016287	0	0	0.014755	0	0.105065	0.027322	0.069581	0.061165	0	0	0	0	0.017769	0.016717	0.097098	0.062939	0.085	0.066109	0.124001	0.089125	0.421659	0.287328	0.136187	0	0	0.01925	0	0	0
LOC107759403	20.19772	20.57335	19.57813	23.09493	19.72349	21.82609	20.88342	19.99396	21.84811	17.68046	18.81713	16.99813	17.85524	18.51254	17.20463	19.95942	16.8927	19.73748	19.18481	18.96489	15.85307	20.81493	22.03044	23.13533	10.12299	12.57836	12.48552	9.235438	11.06684	6.388007
LOC107799202	24.87381	25.90789	20.30663	222.9595	157.8546	272.7299	245.9374	273.9257	193.7403	711.6113	338.0334	560.6535	192.657	410.6309	484.931	13.09288	21.55088	8.045752	166.5988	345.1813	135.3318	129.1429	137.5625	84.73453	157.9058	518.5104	250.9012	65.88255	111.8313	61.03115
LOC107764069	3.632265	3.470172	3.026797	5.196954	4.699187	4.336039	4.811427	4.451925	5.43269	4.543424	4.303461	4.147952	4.378121	4.18125	3.753768	3.453462	2.589454	2.371841	3.889124	3.814319	2.832918	4.665159	4.352882	4.162995	2.755372	2.788431	4.02658	1.791436	4.944604	4.646656
LOC107811610	0	0	0	0	0	0	0	0	0	0	0	0	0	0	0	0	0	0	0	0	0	0	0	0	0	0	0	0	0	0
LOC107809749	0.057277	0.052201	0.04979	0.129725	0.152047	0.184739	0.048041	0.097878	0.376419	0.027926	0.100407	0.387113	0.159404	0.249945	0.058787	0.042683	0.083002	0.064054	0.02906	0.096905	0	0.023917	0.025261	0.179598	0	0.119545	0.067697	0	0.137876	0
LOC107759916	535.86	442.6043	530.606	267.5565	257.6752	401.8145	240.0103	240.4094	347.0662	154.3813	195.9631	198.8842	153.4739	173.7198	141.4342	377.4863	515.4059	497.2081	156.9928	172.6358	182.3557	259.8891	362.102	449.0102	56.8581	29.09832	128.8537	37.50582	32.82376	44.58813
LOC107814814	3.110676	4.298221	2.878499	2.909021	1.687035	2.049767	2.356568	1.714739	1.413116	1.565575	1.899761	1.017285	1.898981	3.065182	2.059814	2.542409	3.199056	4.937525	1.120048	2.376764	1.555768	2.178805	2.38978	2.202478	1.141792	0.977353	1.423188	0	0.966187	0
LOC107758945	0	0	0	0	0	0	0	0	0	0	0	0	0	0	0	0	0	0	0	0	0	0	0	0	0	0	0	0	0	0
LOC107783326	5.177942	6.052702	7.632258	1.291712	2.357295	0.92782	2.832367	2.885331	3.628347	4.244211	3.525536	4.057479	2.25554	3.929656	2.772781	10.14982	9.27968	8.364147	3.845478	2.570977	2.600726	1.817432	1.952681	1.451039	5.075904	5.951732	6.031217	5.36661	3.070897	4.639999
LOC107825968	5.960214	10.09713	5.485857	6.09843	3.660769	3.317061	5.234339	5.152481	2.369948	8.205122	11.60372	3.25817	5.073818	4.360366	5.685584	7.420015	4.13226	4.966368	2.774971	3.440376	1.598769	2.693722	2.597726	1.172633	2.127677	0.68297	1.906157	0	1.687917	0
LOC107805137	11.4657	17.02422	18.72199	51.74727	61.64456	20.71989	73.10502	82.15448	59.74462	82.57939	65.53987	46.90169	51.94739	38.01979	72.075	51.60312	13.06325	20.83777	102.3066	110.1109	113.0236	61.96298	78.04919	62.46949	104.0284	101.8805	75.66614	69.78378	97.64853	140.0644
LOC107824955	0	0	0	0	0	0	0	0	0	0	0	0	0	0	0	0	0	0	0	0	0	0	0	0	0	0	0	0	0	0
LOC107778820	0	0.035519	0.067757	0	0.034486	0	0	0	0	0.038004	0.027328	0	0	0.127553	0	0	0	0	0.039547	0.032969	0	0	0	0	0	0	0	0	0	0
LOC107818501	0.095817	0.232867	0.111055	0.202545	0.367401	0.274706	0.375039	0.245605	0.449784	0.249156	0.470307	0.242846	0.568877	0.731719	0.524499	0.166606	0.154278	0.309553	0.550959	0.270181	0.145644	0.480114	0.225378	0.300443	0.161522	0.133322	0.415241	9.136326	0.461295	3.949658
LOC107764814	0	0	0	0	0	0	0	0	0	0	0	0	0	0	0	0	0	0	0	0	0	0	0	0	0	0	0	0	0	0
LOC107771779	0	0	0.02949	0	0.060038	0	0	0	0	0	0	0	0.037765	0	0.034819	0	0	0	0	0	0	0.028331	0.029924	0	0	0.047204	0	0	0	0
LOC107767569	0.199669	0.212302	0.057856	0.331631	0.412253	0.17889	0.251206	0.426506	0.249944	0.194702	0.466693	0.168686	0.296365	0.036305	0.614804	0.198391	0.289345	0.124051	0.20261	0.225208	0.424903	0.500246	0.352242	0.382605	0.210368	0.277824	0.078664	0	0.320425	0
LOC107822391	1.381444	1.521311	0.900645	4.693194	4.074643	1.299569	20.08373	22.47571	7.565604	3.087064	11.70476	4.522445	4.933913	2.323427	9.038963	2.058908	1.056551	1.501974	2.45314	3.50582	3.202247	16.3438	19.24254	7.460073	4.29364	2.883268	0.680315	53.51277	6.373649	32.03129
LOC107788128	0.034035	0.093056	0.059172	0.092502	0.030116	0.036592	0.513838	0.75609	0.702977	0.232319	1.217131	0.31629	0.378882	0.222783	2.305546	1.445684	0.098642	0.405992	0.448972	0.95011	0.341445	1.47802	0.360253	0.21344	0.258183	1.420714	0.281586	0	0.491569	0
LOC107763298	0	0	0	0	0	0	0	0	0	0	0	0	0	0	0	0	0	0	0	0	0.049453	0	0	0	0.13711	0	0	0	0	0
LOC107793633	1.440543	1.40405	1.356587	1.794457	1.575656	2.194077	1.527089	1.709499	1.258526	1.463241	1.936044	1.588876	1.603633	1.135017	2.032985	1.789158	1.449673	1.596063	2.212948	2.572601	1.496272	1.821262	1.57067	1.129242	1.795989	2.477675	1.300602	2.861645	1.444852	7.422581
LOC107802405	16.29416	17.80203	14.04325	37.89432	34.53321	43.45372	37.03319	33.63393	39.71799	76.75096	58.47892	73.83147	48.09017	47.64869	40.46363	15.99246	25.34002	19.34789	42.88807	54.64748	32.09516	32.89075	40.83901	37.28051	46.55272	62.98429	40.25472	24.15789	38.55328	13.51514
LOC107794692	10.83441	12.93928	11.06367	12.95345	11.58281	11.32355	12.87085	12.85342	10.34199	15.35645	16.9452	10.82053	11.25396	10.30389	13.43512	12.24579	11.49809	12.53666	18.86148	21.65229	11.53415	14.12715	13.16263	10.58743	8.631263	17.54114	8.616277	4.320523	7.077571	3.735547
LOC107826640	0.737743	2.091793	0.831321	0.198032	0.145067	0.440647	0.022918	0.023346	0	0	0.095797	0	0	0.178854	0.084133	1.791833	0.871105	0.855585	0.027726	0.254255	0	0	0.120507	0	0.034546	0.038019	0	0	0	0
LOC107824499	0.016308	0.029725	0.113406	0	0.04329	0	0.068389	0.083602	0.01531	0.047706	0.080044	0.013777	0.018154	0.035581	0.050213	0.024305	0.015754	0.048632	0.016548	0.013795	0	0.013619	0.028769	0.051134	0.020618	0	0	0	0.07851	0
LOC107761555	9.407844	8.57409	9.438564	7.43362	6.97896	7.985197	7.979757	7.161959	9.928259	4.104084	4.88559	3.34653	6.142027	6.443623	5.735491	8.69753	9.840471	9.149773	5.419214	4.936594	6.935053	9.924298	10.26374	13.27096	1.878059	3.198764	2.717126	7.587901	5.61902	6.560539
LOC107779876	5.937787	4.566004	4.447265	0.504313	0.492576	0.541487	0.244567	0.407684	0.423077	0.56867	0.520447	0.425499	0.472145	0.549459	0.190449	2.330945	3.508424	4.683789	0.511071	0.739989	0.531866	0.110688	0.280581	0.277061	0.469198	0.479491	0.626606	0	0.255238	1.639028
LOC107799548	0	0	0	0	0	0	0	0	0	0	0	0	0	0	0	0	0	0	0	0	0	0	0	0	0	0	0	0	0	0
LOC107800868	1.236101	0.516337	0.89543	0.513262	0.31902	0.49836	0.604783	0.396059	0.77367	0.200892	0.07223	0.174049	0.172006	0.112377	0.052862	0.537334	1.094662	0.959964	0.156789	0.043569	0.187891	0.559162	0.681452	1.130475	0	0	0.060874	0	0	0
LOC107798292	0	0	0	0	0	0	0	0	0	0	0	0	0	0	0	0.265534	1.376959	0.177104	0	0	0.21665	0	0.314302	0	0	0	0	0	0	0
LOC107800738	1.942706	0.801753	1.083351	0.929808	0.648692	0.906394	1.38348	1.221437	1.686246	0.393174	0.64256	0.371604	0.163219	0.199943	0.300972	1.037979	1.239408	1.311731	0.669506	0.651157	0.367728	1.346902	1.616599	2.375321	0.185371	0.051003	0.303261	0	0.529408	2.266424
LOC107811063	8.120564	11.33383	9.793947	14.21291	15.46223	12.15884	14.15703	13.91319	12.09453	12.49974	12.47644	10.11112	13.13392	13.10317	13.13483	11.45341	8.93361	10.93564	15.74695	16.81394	12.93158	14.25576	13.18194	12.17594	17.79231	20.14329	13.36664	16.72274	16.37499	15.77298
LOC107829541	0.067068	0.061124	0.0583	0.546838	0.534112	0.360529	1.631321	1.776435	0.755592	0.196198	1.175695	0.396624	0.447963	0.585339	0.413018	0.149936	0.388757	0.050002	1.565277	0.794284	1.957336	0.392069	1.18316	0.560792	3.81572	0.279959	1.347564	0	0	0
LOC107763838	1.017423	0.831333	1.158901	0.190704	0.341487	0.905259	0.058852	0.119905	0.032938	1.915811	1.082427	0.444594	0.273389	0.421019	0.108026	0.732041	0.542299	0.837001	0.534006	1.424558	0.159984	0.087898	0.123784	0.036669	0.266138	0.195265	1.53424	0	1.520143	0
LOC107779291	0	0	0	0	0	0	0	0	0	0	0	0	0	0	0	0	0	0	0	0	0	0	0	0	0	0	0	0	0	0
LOC107789786	0.878266	1.344724	0.793996	0.859317	0.963663	0.906473	1.53221	0.87048	1.286305	0.685136	0.788276	0.712304	0.703942	0.881493	0.576914	1.125713	0.814539	0.942889	0.534722	0.564644	0.384477	0.968171	1.022589	0.881244	0.133247	0	0.581302	0	0.169131	0
LOC107777037	4.418462	4.926746	4.05545	4.159441	3.03606	6.422312	3.830177	3.058167	3.777454	4.164138	3.980964	3.19064	3.517278	3.231503	2.533513	2.943145	3.76782	3.551794	2.630016	3.403618	1.778476	2.947856	2.612766	2.373588	2.090954	1.408194	4.784647	0	2.614442	1.526258
LOC107832748	19.94617	16.9505	18.07082	35.31214	25.52711	33.83535	35.98201	30.09686	37.72376	20.20082	26.28541	21.26204	15.93678	15.80181	12.96491	12.76611	14.73701	13.43567	24.8056	26.25608	21.87777	30.54223	38.94416	33.6481	14.05396	7.432219	16.06725	6.882671	7.413499	5.950794
LOC107789142	3.328902	6.619382	3.639102	2.901581	3.548147	1.138765	6.959081	4.675863	4.545919	2.631308	6.825848	1.619231	12.26838	5.970242	16.43637	6.013821	5.043271	4.380819	2.098417	2.325351	1.932009	6.423606	6.918127	3.031267	0.382613	0.245632	0.08942	0	3.156738	0
LOC107819190	114.1838	78.7952	98.01088	85.93584	83.75462	102.6439	97.77563	96.21967	134.3856	75.31676	63.545	95.02326	113.7309	132.9132	67.8498	13.89327	12.40341	12.57585	4.365757	5.14154	7.723118	7.756381	11.74638	12.99089	3.453661	1.140276	6.215081	14.65147	9.863422	8.445162
Novel02553	1.986819	1.865611	2.093454	0.981795	1.11877	1.618237	0.504979	0.360095	1.017443	0.176127	0.422169	0.406914	0.402137	0.394095	0.370767	1.345981	1.686775	1.526144	0.488748	0.611168	0.658913	0.502801	0.902806	1.636127	0	0	0	0	0	0
LOC107770955	0	0	0	0	0	0	0	0	0	0	0	0	0	0	0	0	0	0	0	0	0	0	0	0	0	0	0	0	0	0
LOC107783977	4.067301	4.351512	3.597107	4.710095	3.004395	4.410871	6.763512	4.35157	4.681888	3.793691	4.364791	4.481959	5.866423	5.208917	4.065664	4.427833	5.398592	4.957226	1.902108	1.884881	1.612803	5.198442	4.14917	4.58384	1.565049	1.623988	2.59172	0	3.06492	0
LOC107806941	7.247794	8.408804	8.290956	6.405002	4.839503	5.999566	5.836621	4.862988	5.406067	3.121847	4.941853	2.516873	4.256918	3.61393	3.605374	8.830575	8.741734	9.033522	3.56448	4.814634	2.94008	7.222539	7.275507	7.180356	0.787042	0.680568	1.813115	0	0.21407	0
LOC107789295	1.468953	2.466154	2.049808	1.882377	1.795812	1.96374	1.969697	1.981757	1.869067	1.818789	2.636065	1.241012	1.893457	1.866133	2.410325	2.643041	1.745906	2.44251	1.431753	2.182783	0.467136	1.896637	1.721935	1.404036	0.708669	1.008523	0.662491	0	0.884008	1.792649
LOC107806894	3.006584	4.65492	4.7233	2.855081	2.371944	5.023921	2.825577	2.352252	2.550645	2.472601	2.108218	1.744369	2.379172	1.778332	1.933319	2.591433	2.134715	2.484594	1.396785	1.470861	1.65184	1.78484	1.821257	2.158089	0.641172	0.151209	0.856275	2.590521	1.395158	0
Novel03314	0.924349	0.468017	0.803517	0.744367	1.544969	0.441684	0.947578	0.965297	0	0.200301	1.080257	0.694147	0.800332	0.672279	0.52707	0.382681	0.793777	0.918855	0.937965	1.042581	0.562014	0.85772	1.902452	0.858781	2.207465	2.143606	1.335285	0	0.988921	6.35043
LOC107798691	6.491234	7.321732	7.179069	4.890904	4.748646	5.873307	2.237087	2.910428	3.439333	6.204431	7.210546	3.474994	6.58222	6.731042	3.199283	1.843912	1.769562	3.83326	1.728235	1.549479	1.201607	0.966119	0.992076	1.74654	1.218846	0.26828	2.012981	0	2.939461	0
LOC107783486	27.80481	24.57275	27.05915	23.79032	17.85205	33.36605	15.94	13.91835	16.3482	17.84724	19.4311	20.68251	35.74672	33.09128	13.98078	24.80142	29.03243	28.8261	17.05214	16.90816	15.11261	8.717468	18.0433	21.23354	4.142695	2.084221	8.243424	3.347537	8.33822	0
LOC107827386	7.109266	10.01672	7.636125	6.958846	4.121523	7.555476	9.938079	8.622783	6.904647	48.60671	28.70685	20.84878	21.33177	14.66481	9.770943	12.54429	7.499702	8.864227	8.499243	13.75605	4.806933	10.68002	7.315983	5.679713	27.17145	12.79141	85.61939	11.68766	10.22862	7.578907
LOC107818252	0	0	0	0	0	0	0	0	0	0	0	0	0	0	0	0	0	0	0	0	0	0	0	0	0	0	0	0	0	0
LOC107810425	9.59238	9.832274	11.12854	9.972734	9.989021	7.675692	8.188847	8.039577	8.35266	4.629289	6.323981	3.546749	4.456114	3.727924	5.799491	10.34951	8.81902	10.74549	6.625345	6.421393	4.630203	10.00846	9.763651	11.69694	1.342377	1.884895	2.134782	2.618285	2.115166	3.772972
LOC107832535	6.026435	7.735063	6.59197	8.234964	7.021238	7.073077	8.087409	8.324458	7.543843	7.345669	7.74719	5.727712	8.329983	5.971878	9.638864	8.719843	6.452764	8.162216	7.491132	8.624031	6.870262	9.855965	9.568285	9.395752	6.730334	7.476932	3.917519	7.182922	15.71559	27.94676
LOC107791228	19.01005	12.80069	10.81639	14.17624	9.064063	11.48589	8.169934	7.785759	10.67898	4.7492	6.78611	6.663	6.471103	8.364171	2.934772	6.813879	14.11548	8.08201	5.229048	6.565381	9.313526	5.248195	8.231624	7.356567	0.119179	0.743254	1.076995	0	0.151275	0
LOC107774518	438.0423	305.1249	394.753	181.3337	173.1815	303.3266	144.5671	158.5802	193.6877	138.7558	142.3404	194.8201	107.7786	159.1567	105.1933	181.9213	338.1467	251.86	112.9227	95.49775	176.857	118.2028	172.9672	205.0554	66.94117	34.99418	113.0895	135.2308	80.1133	58.46064
LOC107824772	0	0	0	0	0	0	0	0	0	0	0	0	0	0	0	0	0	0	0	0	0	0	0	0	0	0	0	0	0	0
LOC107814330	11.8831	11.4519	11.12747	15.62594	14.99161	15.45744	15.09832	14.27486	11.97378	14.20332	14.93239	14.2934	21.45364	19.1506	19.34532	12.52017	11.95746	12.10486	14.231	15.06847	11.67455	15.38694	11.89307	12.4694	12.61408	17.15655	11.4862	26.92466	14.6139	20.36931
LOC107775179	0.326467	0.595068	0.315323	1.906023	1.765373	1.24797	2.82949	3.564259	2.826623	0.99041	0.813933	0.612907	0.928758	1.780795	3.239072	0.621724	0.140175	0.459746	2.79744	2.117291	3.374422	4.816655	1.951764	2.38856	1.192398	2.018909	1.286191	5.188218	0.873182	2.242881
LOC107769240	0	0	0	0	0	0	0	0	0	0	0	0	0	0	0	0	0	0	0	0	0	0	0	0	0	0	0	0	0	0
LOC107804135	49.99513	9.517562	20.80061	2.905311	2.182844	1.768121	14.75934	7.868956	32.19242	14.59339	1.845085	4.793375	61.6968	60.82176	9.283715	15.93202	25.73855	18.085	0.417201	0.20868	2.324816	17.85457	6.020095	5.156733	1.975282	0	6.414398	0	49.48485	5.084332
Novel04820	0.638778	0.310489	0.444221	2.546281	6.142192	1.785586	2.107363	2.619787	1.319368	4.27717	2.986075	5.180708	8.58056	8.316041	5.944322	0.920301	1.316507	1.301711	6.438661	4.683136	6.097628	3.876475	2.17865	1.86942	8.722178	5.866162	4.580234	15.22721	25.21748	13.16553
LOC107793864	0	0	0	0	0	0	0	0	0	0	0	0	0	0	0.017791	0	0	0	0.020196	0.130641	0.019938	0	0	0	0	0	0	0	0	0
LOC107783709	6.018793	8.796387	5.891902	7.810655	5.661686	5.013519	23.78576	22.37734	8.95968	10.25768	16.72944	5.451318	11.16701	9.405648	18.58822	6.788428	3.352597	3.880878	3.301327	4.082361	3.066062	10.95857	9.80486	2.890386	3.770514	3.84784	2.499418	0	2.871556	0
LOC107761586	0	0	0	0	0	0	0	0	0	0	0	0	0	0	0	0	0	0	0	0	0	0	0	0	0	0	0	0	0	0
LOC107760119	0	0	0	0	0	0	0	0	0	0	0	0	0	0	0	0	0	0	0	0	0	0	0	0	0	0	0	0	0	0
LOC107815960	0	0	0	0	0	0	0	0	0	0	0	0	0	0	0	0	0	0	0	0	0	0	0	0	0	0	0	0	0	0
LOC107763107	7.142695	6.566788	4.629512	11.1822	19.07186	13.53977	9.459293	12.9553	10.99999	19.42877	16.82667	31.54799	23.29605	29.46047	19.35659	4.108785	4.539766	5.839015	20.85364	16.69563	25.59989	9.418502	9.063631	11.65672	17.74421	16.04115	17.03229	22.40367	33.48254	7.748133
LOC107814178	1.257114	1.336655	1.092783	1.043975	1.48317	1.464181	1.230129	1.879697	2.163763	1.736614	2.130266	1.41606	1.982536	0.800012	3.010624	2.654275	1.416891	2.733593	1.594541	0.797574	0.477712	1.487286	1.386072	1.094946	0.662239	0.583061	0	0	1.513049	0
LOC107816495	1.869446	1.092984	1.257129	1.214296	1.529363	1.858194	1.5384	1.989097	1.15904	1.58218	1.756053	2.145546	1.963296	1.731629	1.375706	1.498254	1.260822	1.604129	2.040153	1.611261	1.57629	1.207739	1.7112	1.695876	1.828405	2.355802	1.792641	2.522487	2.377406	2.180956
LOC107779729	0	0	0	0	0	0	0	0	0	0	0.090688	0	0	0	0	0	0	0.131238	0	0	0	0.11408	0	0.490548	0	0	0	0.622654	0	
LOC107814990	0	0	0.047529	0.099068	0.048381	0.058784	0.137578	0	0.153997	0.053316	0	0	0.121733	0	0.112236	0	0	0	0.055482	0	0	0	0	0.069127	0	0.45236	0	1.316152	0	
LOC107800896	6.022996	7.956282	7.05932	9.748181	8.263786	8.876469	10.10349	8.210809	8.640819	7.127007	10.01261	7.604005	7.458241	7.456742	7.571015	7.31247	9.93759	10.24215	9.339258	8.873384	7.468111	9.325209	10.2672	8.770887	4.449154	7.721414	5.598975	0	5.864531	0
LOC107817033	1.515733	0.951131	1.036796	0.450222	0.483719	0.347291	0.312617	0.148616	0.326599	0.19384	0.209082	0.209924	0.276612	0.487945	0.15302	0.555505	0.816182	0.444607	0.252141	0.210198	0.317266	0.290518	0.175341	0.20777	0	0	0.029368	0	0	0
LOC107799350	84.3628	79.96614	87.70799	33.56727	32.14532	40.09499	29.76117	30.73018	48.94981	14.71108	19.9723	14.58069	14.51029	15.27344	9.290522	61.15842	98.99116	76.21309	23.7589	22.56322	27.18777	42.13104	59.82893	85.27461	12.51236	8.732479	15.12055	0	9.296768	7.462487
LOC107796052	3.087149	2.658965	3.391354	2.243587	1.380866	1.531875	2.048697	2.202951	4.395302	1.654044	2.521531	3.209981	2.681348	3.515969	1.14904	3.311777	3.57238	4.426155	1.445827	1.377503	1.794515	1.558228	2.872689	5.318716	0.214456	0.141611	0.922215	0	0.653303	2.097616
LOC107820134	7.992633	5.886574	5.922317	13.22603	10.90873	8.371096	8.559051	8.189907	8.445276	6.097015	4.901299	5.182652	6.008282	5.75942	8.475857	3.296745	3.048732	3.078371	9.60675	9.00664	6.61696	8.596033	10.37993	6.504326	5.77968	5.129689	3.45099	2.109174	9.087381	1.823603
LOC107771950	4.761797	5.573436	5.88331	0.766437	0.727212	1.533255	0.141916	0.43371	0.40848	0.306414	0.322033	0.285891	0.538162	0.58014	0.124045	5.349764	4.646999	5.532423	0.637718	0.777004	0.529077	0.484471	1.130008	0.808451	0.03056	0.033633	0.428533	1.728608	0	0
LOC107772648	1.571695	1.212036	1.392515	0.657174	1.096544	0.649909	1.698512	0.903874	1.617459	1.208397	1.229236	1.072463	1.648693	1.714642	1.675189	3.265927	1.693588	3.965975	1.410827	2.096592	1.240453	1.893121	1.333018	1.547962	1.643179	1.261673	1.679	4.323016	2.473729	1.868851
LOC107792753	44.9037	38.2074	39.52741	15.77494	12.56072	9.76731	25.87572	22.51885	24.92139	20.16314	36.52965	28.46191	33.65855	37.99259	28.45893	51.93883	61.48273	48.61133	5.473582	6.284209	11.82413	20.66663	18.40584	18.59529	6.341227	3.160222	18.11961	6.767649	2.733602	2.925673
LOC107769347	0.434807	0.343437	0.251979	0.446435	0.359096	0.467471	0.607819	0.421046	0.054429	0.197863	0.731729	0	0.064538	0.189741	0.862797	0.907253	0.504072	0.648334	0.50004	0.416859	0.185057	0.992525	0.460234	0.090892	0.109946	0.443668	0	0	0	0
LOC107827665	1.85689	1.410272	2.08495	0.841124	0.479237	0.998192	0.843621	0.528859	0.508471	0.528121	0.705278	1.372658	0.775168	0.675258	0.158822	1.787352	1.121195	1.845853	0.471061	1.570801	0.493942	0.32307	0.81895	0.404338	0.09782	0	0.731565	0	0	0
LOC107809314	4.478328	5.321081	4.305632	4.487265	4.712116	3.338595	4.466502	5.536776	4.565866	5.105186	5.704559	2.775228	5.671	5.529588	7.019768	7.028318	4.636359	5.74003	5.872431	5.720529	3.885362	8.197927	5.953569	3.48709	10.22075	13.53381	5.899674	3.670645	10.62569	31.7366
LOC107782750	0.090544	0.06189	0.314832	0.205071	0.080119	0.097346	0.018986	0.038682	0.063755	0.044146	0.031745	0.019124	0.075596	0.098779	0	0.151816	0.109342	0.101257	0.068908	0.095743	0.020644	0.132328	0.059899	0.118296	0	0	0	0	0	0
LOC107777884	0	0	0	0	0	0	0	0	0	0	0	0	0	0	0	0	0	0	0	0	0	0	0	0	0	0	0	0	0	0
LOC107805618	0	0	0	0	0	0	0	0	0	0	0	0	0	0	0	0	0	0	0	0	0	0	0	0	0	0	0	0	0	0
LOC107817409	20.2356	9.87395	12.60903	46.64224	81.36835	64.78591	37.62481	56.68927	55.14485	60.94301	28.504	123.754	52.32938	65.05596	25.36424	16.68097	12.54269	21.76194	65.14449	64.00286	102.6469	28.41472	28.51123	43.61068	45.05492	23.92045	105.1918	32.01618	124.1476	16.60882
LOC107800658	0	0	0	0	0	0	0	0	0	0	0	0	0	0	0	0	0	0	0	0	0	0	0	0	0	0	0	0	0	0
LOC107792714	3.823212	3.966098	3.24686	1.771722	1.54341	1.761609	3.206692	2.75483	2.166873	1.872647	2.483173	1.518228	1.43176	1.403126	2.13381	4.437709	5.140324	4.702448	2.055973	1.967332	1.156922	3.251719	3.279081	2.025632	0.980105	1.348316	0.708004	0	2.714293	1.089378
LOC107773650	0	0	0	0	0	0	0	0	0	0	0	0	0	0	0	0	0	0	0	0	0	0	0	0	0	0	0	0	0	0
LOC107826790	12.70016	14.78981	13.25966	15.03655	11.41632	14.81014	15.35829	14.64073	14.13152	11.36862	11.00212	11.24037	9.23832	10.92292	11.58675	11.06657	13.92482	12.14875	10.84165	12.10771	11.70533	13.94524	15.02542	11.93976	3.992977	6.124197	8.736309	9.611016	5.337889	4.154868
LOC107778041	1.59663	1.361252	1.149135	0.482155	0.592466	0.66448	0.748779	0.630761	0.725316	0.518972	0.601913	0.261073	0.55424	0.561885	0.722454	1.164223	1.194177	1.561541	0.505208	0.406644	0.422754	1.017962	1.120609	1.32786	0.043411	0.047776	0.121748	0	0.165306	1.061527
LOC107803168	2.984035	3.535454	3.251094	4.649821	3.995912	3.336531	4.705337	4.674341	3.828776	4.946679	5.900588	3.680654	5.00495	4.622718	5.451663	4.654959	3.132674	3.811695	3.532658	3.668632	2.88478	5.133614	4.38686	3.576353	3.043333	4.705676	2.53936	5.690685	2.394368	3.690148
LOC107822419	30.15004	24.63881	28.18249	28.94084	26.35168	35.87788	21.92229	19.76064	27.76089	24.71462	18.36436	22.57203	24.62887	22.69621	15.55388	22.58589	25.76073	30.03646	25.23618	25.7282	33.42059	20.15209	26.82721	29.91234	15.02055	13.22467	22.46685	26.43272	18.0488	19.58903
LOC107796064	0.643608	1.443862	0.688584	0.852188	0.525698	0.479047	0.664396	0.423012	0	0.289661	0.243007	0	0	0.162033	0.050814	1.069915	0.143487	0.406016	0.20095	0.795731	0.090305	0.165383	1.659444	0	0	0	0	0	0	0
LOC107792377	2.43714	1.776922	1.988181	2.004117	2.090187	2.096179	1.509515	2.402721	2.18249	1.937777	1.735215	1.267054	2.379143	2.576991	1.077532	2.179391	1.956031	2.515834	1.674059	1.998217	1.641388	2.035319	1.587484	1.95946	0.853279	0.730391	1.196517	0	1.624605	0
LOC107763430	9.991091	7.521086	9.515231	13.0447	8.190717	14.26954	2.32092	3.201247	4.460134	3.008736	3.056434	2.048123	1.035893	1.228898	0.955084	5.45629	4.045421	5.750898	8.075827	8.907508	7.973037	3.742449	5.918402	8.190341	4.086751	0.885904	9.232755	0	0.707363	0
LOC107762446	0	0.036806	0.175531	0.109761	0.107207	0.173677	0.406475	0.034506	0.113747	0.315046	0.339819	0.102356	0.629405	0.660876	0.580305	0.210666	0.195078	0.210763	0.245881	0.068326	0.073664	0.573356	0.071245	0.211054	0.153178	0.056193	0.238661	0	1.361005	0
LOC107763084	1.036382	1.440028	1.491662	1.739291	1.44324	1.516093	1.781267	2.250074	1.611034	1.689861	2.156308	1.019099	1.323935	1.797906	1.865859	1.797843	0.968405	1.393333	1.448172	1.753416	1.177623	1.816151	1.903246	1.260804	1.245861	1.654811	0.682742	0	1.472315	4.202036
Novel00069	0.426347	0.388563	0.21178	1.600173	2.694713	0.785786	1.634722	1.248968	1.143638	1.36601	1.452066	2.058227	1.966263	1.328924	1.812876	0.317714	1.000297	0.726536	2.039529	1.08198	2.110822	1.220755	1.71916	1.591492	0.84705	0.593231	1.36775	0	4.105171	0
LOC107789271	3.712483	4.613824	1.760282	6.482038	5.076894	2.539965	28.76037	31.48977	15.52606	7.766845	15.6665	8.268714	10.44468	8.247551	14.82588	3.068352	2.282363	2.415547	4.383618	7.879813	7.017938	23.61935	38.16462	14.18068	8.619353	10.14346	6.701453	43.44464	14.62354	20.86804
LOC107760961	3.092431	2.668722	2.402714	2.206559	1.646675	2.236109	2.685569	2.268132	1.644353	1.57447	1.95733	1.387206	1.888826	2.239175	1.769568	2.50842	2.61741	2.121912	1.832806	1.736272	1.94679	2.536861	2.172527	1.430181	0.795794	1.332754	0.970355	3.914204	0.658764	10.15273
LOC107797829	3.377826	3.420524	3.486824	4.122688	3.009679	3.782892	2.852803	2.725767	3.611704	3.019323	4.62193	3.329295	5.405349	4.273625	3.948423	3.303772	3.739157	4.039793	2.332662	2.698664	1.711463	3.42819	3.269136	3.604055	2.075986	1.109722	3.021963	0	2.823274	0
LOC107820370	4.073981	3.66873	4.215983	10.28157	11.11519	8.603627	17.93937	19.31082	13.61459	12.39087	11.25667	15.24231	9.826295	13.17481	15.03321	4.084056	5.669439	4.375196	20.37469	17.39565	18.53347	16.12037	14.28852	10.18908	18.39554	29.01808	20.75087	17.3421	17.04515	17.99289
LOC107805961	0	0	0	0	0	0	0	0	0	0	0	0	0	0	0	0	0	0	0	0	0	0	0	0	0	0	0	0	0	0
LOC107816628	56.28975	38.49193	43.75663	25.84887	19.57445	20.8671	26.38353	24.01765	39.48665	21.39939	28.18256	22.79552	52.58477	57.60489	27.7302	44.04717	54.14242	44.35622	14.15463	14.03487	17.76563	25.59316	27.93273	27.13434	5.299697	4.068095	5.703757	2.519095	7.461797	6.534068
LOC107793859	31.72873	30.24634	30.71173	37.74797	33.07766	36.56286	47.52735	43.11875	46.39447	27.69443	33.14855	32.77489	42.62863	45.05851	40.1454	24.93521	34.13187	30.65649	22.20422	23.60098	24.23901	43.59142	41.81942	38.59471	8.587787	11.35421	14.17056	13.04053	16.02155	16.91237
LOC107810531	4.919921	4.574485	7.761569	3.961955	3.283425	3.508534	3.140068	3.227093	3.872527	1.583028	2.915531	1.48346	2.083813	1.60841	1.598213	6.40682	4.160925	5.853995	2.807172	3.195005	2.764748	3.915076	5.917766	6.544745	0.670198	1.429071	1.292211	2.369319	1.036772	0
LOC107832837	8.449336	14.89885	10.2455	1.331247	0.758488	2.106451	1.181137	1.700207	0.603568	0.567189	1.008913	0.206905	0.170397	0.233785	0.125684	11.11005	8.606398	8.878416	1.428967	1.786889	1.060962	2.761146	2.64631	0.735937	0.116114	0.042597	0.144731	0	0	1.892881
LOC107766677	0.052223	0	0.045396	6.907475	22.87416	3.312626	37.80092	42.92524	9.65878	21.03166	17.90662	30.70719	49.12448	38.17179	109.1303	0.038917	0.100904	0.116804	23.42263	16.74314	31.00605	56.95841	13.95746	13.15464	78.96693	81.45704	14.81364	358.5302	135.5157	164.6807
LOC107805215	0.691073	0.516782	0.431297	0.561864	0.564468	0.876344	0.802568	0.575331	0.865079	0.622046	0.981586	0.77845	1.26246	0.811921	0.818421	0.937543	1.010011	1.8231	0.952985	0.464682	0.581788	1.139467	1.172253	1.222369	0.358452	0.246558	0.272264	1.267217	1.109023	1.095643
LOC107785312	38.02914	27.41103	33.73364	24.6476	21.21686	28.47876	17.25229	17.5749	22.45499	30.43622	27.84004	33.18891	25.29424	24.91885	22.09362	25.53216	40.32086	29.11018	19.9621	16.64142	21.64974	17.02809	20.93876	25.37375	12.92728	10.06712	20.84824	12.8284	12.37844	7.394337
LOC107830084	2.165572	2.960477	2.706073	7.050571	7.305666	5.020298	4.086806	3.931937	3.049701	4.025439	3.511531	6.060335	5.725518	5.611014	4.514825	4.236189	4.184248	4.490399	7.828495	6.182745	12.40571	3.673567	2.029563	3.395182	4.02135	2.448253	1.599707	9.679312	3.258073	0
LOC107761121	11.76679	15.80586	15.38109	5.608492	4.467847	5.428487	23.4212	18.64466	14.30362	3.467919	12.22238	2.670692	14.85851	11.25631	8.652256	11.09165	8.186444	10.57299	2.093977	2.93417	2.282447	14.33679	9.13979	4.818498	0.610615	0.610919	0.934079	0	0.422757	8.144307
LOC107779157	0	0	0	0	0	0	0	0	0	0	0	0	0	0	0	0	0	0	0	0	0	0	0	0	0	0	0	0	0	0

LOC107821101	0	0	0	0	0	0	0	0	0	0	0	0	0	0	0	0	0	0	0	0	0	0	0	0	0	0	0	0	0	0
LOC107812944	0	0	0	0	0	0	0	0	0	0	0	0	0	0	0	0.94895	0.246045	0	3.187359	4.955215	2.709875	8.082306	7.413353	2.573212	0.643993	3.425601	1.806087	0	0.817422	0
LOC107826894	2.420697	2.910263	2.99969	3.312873	1.686249	2.048812	3.499101	3.872579	3.191391	1.45647	2.997526	2.784779	3.153435	2.640861	3.330337	2.034192	2.637141	2.495906	1.724675	1.568484	2.630471	2.0646	2.725806	3.176098	0.325585	0.573315	0.730486	0	2.231636	0
LOC107819042	3.49401	4.790383	4.278587	2.780043	3.871383	3.299176	4.561506	3.738211	3.879347	1.836872	3.323481	1.001058	2.164636	1.425279	3.087212	4.528244	3.522268	4.57562	2.158118	1.619205	0.942124	5.607517	5.011515	5.112713	1.190802	0.803231	1.077297	0	0.438819	0
LOC107797138	0	0	0	0	0	0	0	0	0	0	0	0	0	0	0	0	0	0	0	0	0	0	0	0	0	0	0	0	0	0
LOC107831364	0	0	0	0	0	0	0	0	0	0	0	0	0	0	0	0	0	0	0	0	0	0	0	0	0	0	0	0	0	0
LOC107774616	10.72838	9.969318	11.21553	1.334032	2.23369	2.789347	0.941005	0.718951	1.38247	1.435896	0.835861	1.303268	0.546411	1.529953	0.431816	7.158747	13.20968	9.462238	3.130736	2.25404	1.854572	0.70271	2.164772	1.68566	0	0.097567	0.580135	0	1.01275	0
Novel03358	2.434171	1.561129	2.899667	2.041885	2.07414	3.586303	1.436713	2.002792	0.761769	1.230768	1.896492	1.21864	0.903251	1.67202	1.110385	1.410848	2.003227	2.083634	0.640378	1.067703	1.644448	1.054065	0.874744	1.130753	0.113983	0	0.639332	0	0.434036	0
LOC107808369	1.741845	3.288345	2.271223	1.465307	2.422041	2.00646	3.130629	3.827004	2.920215	1.819841	3.489665	1.786893	3.324079	1.357334	3.95866	0	0	0	0	0	0	0	0	0	0	0	0	0	0	0
LOC107796546	0.763149	1.117041	1.105648	0.754226	0.572969	0.91993	0.834051	0.553259	1.042149	0.541212	0.810789	0.703341	0.46339	0.731642	0.59339	1.240799	0.737266	0.86206	0.774391	0.762948	0.569458	1.564344	1.305499	0.942669	0.350855	0.353955	0.409991	0	0.222671	0
LOC107802100	1.174296	1.802485	1.047653	0.50393	0.464858	0.797376	0.69982	0.686503	0.377164	0.210937	0.325033	0.182751	0.61922	0.471984	0.158587	2.256802	0.865778	1.221074	0.439008	0.496687	0.253653	1.367797	1.553709	0.581387	0	0	0.073049	0	0	0
LOC107775388	0.438029	0.463598	0.454466	0.87047	0.612653	0.546892	1.185141	0.989988	0.570431	1.171172	1.436651	0.871429	0.644916	0.940321	1.957851	1.316208	0.655235	0.905966	0.645215	0.88452	0.386603	0.814221	0.56086	0.457828	0.19651	0.373555	0.517715	0	0.40816	0
Novel00924	6.691547	5.310673	5.260186	5.482087	5.722806	6.23041	5.075408	5.307096	6.643026	7.898809	5.118673	5.896644	4.241367	4.959909	7.130911	7.01457	6.681114	6.850693	7.407403	8.396087	6.774403	4.090946	6.721378	6.190864	4.614627	6.415096	7.114199	6.868998	7.244631	3.959315
LOC107822054	0	0.097028	0.138819	0.1929	0.329719	0.400612	0.17859	0	0.049976	0.155722	0	0	0.177774	0.058073	0.054635	0	0.102852	0.277804	0.162047	0.450302	0.145644	0.08891	0.093907	0.278188	0.067301	0	0.251661	0	0.256275	0
LOC107807256	13.33572	14.98977	16.03624	28.92846	23.20731	26.88288	20.47521	22.75716	22.01462	19.03643	17.22146	21.59391	19.71154	20.20642	18.55406	16.563	12.81186	15.49349	25.97425	31.77511	29.86546	21.68624	21.07536	23.03724	17.51609	19.32884	18.30115	29.14061	25.68117	25.19513
Novel02154	1.288027	1.076055	0.653132	1.65309	1.56713	1.096289	1.620485	1.100525	1.561956	1.46532	1.655803	1.450881	1.43385	1.580821	2.038076	2.039658	1.192495	1.48043	0.871334	1.588974	0.978919	2.061691	1.420156	1.682807	0.203557	0.14935	0.317155	0	0.775128	0
LOC107761571	0.044961	0.040977	0	0.773918	0.994615	0.241694	0.754217	0.729905	0.675384	0.131528	0.315268	0.151938	0.250257	0.735756	0.507616	0	0.086873	0.03352	2.509292	2.510248	3.075401	1.351737	1.784643	0.704901	2.387473	3.50337	0.743967	3.215359	2.813968	2.780017
LOC107831910	2.104544	1.704918	1.87009	3.940328	6.621294	3.921913	7.374495	9.270614	4.478568	9.440102	8.985419	5.294421	5.154177	5.459269	9.984214	2.265335	2.213892	1.987434	11.10484	11.03786	7.165684	9.686107	6.435336	3.763878	19.6898	26.74532	12.32646	10.03364	9.456556	11.56686
Novel05374	1.857863	2.184791	2.44855	1.303068	1.007588	1.99743	0.452401	0.614481	0.900255	0.116881	0.630356	0.455683	0.266864	0.653819	0.184535	1.518464	2.200141	1.831928	1.033837	0.709766	2.022352	0.35035	0.898674	0.876959	0.303084	0.08339	0.495836	0	0.577059	0
LOC107795104	14.42341	12.45331	13.41782	10.4535	7.299417	13.82021	11.20614	9.599557	11.87836	8.043992	10.85893	11.71499	10.76057	11.53917	10.2328	9.051237	11.97855	9.583613	4.827274	5.822334	5.954095	8.83327	11.51712	8.674936	2.495395	2.253376	4.187059	0	2.680119	0
LOC107761458	38.19358	26.28415	40.92515	15.67657	28.27844	20.78279	31.11932	36.76278	43.46845	20.36995	21.97165	37.53508	26.37828	31.46307	10.08022	22.42101	29.51856	24.98821	25.31017	25.31981	37.39236	19.13787	33.96427	34.54295	18.72403	29.50011	24.13701	100.3364	29.27029	19.27809
LOC107821374	0	0	0	0	0	0	0	0	0	0	0	0	0	0	0	0	0	0	0	0	0	0	0	0	0	0	0	0	0	0
LOC107794841	0	0	0	0	0	0	0	0	0	0	0	0	0	0	0	0	0	0	0	0	0	0	0	0	0	0	0	0	0	0
LOC107777183	0	0	0	0	0	0	0	0	0	0	0	0	0	0	0	0	0	0	0	0	0	0	0	0	0	0	0	0	0	0
LOC107794759	0	0	0	0	0	0	0	0	0	0	0	0	0	0	0	0	0	0	0	0	0	0	0	0	0	0	0	0	0	0
LOC107820075	4.003967	2.572332	4.222323	2.378617	3.484892	3.881335	3.963894	3.252847	4.498596	3.1363	2.209246	2.495407	3.507361	3.795261	3.974822	2.73919	4.375469	3.22981	6.52736	4.997332	4.010867	4.111251	9.089949	10.70248	10.87135	9.68115	13.65404	18.77641	2.528068	0
LOC107796536	6.471221	3.891528	5.694459	1.489677	1.666219	3.307595	3.803833	3.195145	6.374261	1.732735	2.231619	2.599098	12.07531	6.94405	2.504317	4.703779	8.403983	4.943207	1.076483	0.942282	0.749827	2.812886	5.61447	5.682632	0.435904	0.073805	0.877692	0	6.767223	1.639858
Novel05081	0.751863	1.14205	1.525016	0.908199	1.108829	0.67362	0.420413	0.321206	0.411764	0.427677	0.219669	0.635194	0.976481	0.54683	0.514461	1.494104	1.392195	1.448076	1.335142	0.848032	1.199995	0.366275	0.828991	0.654872	0.237646	0.08718	0.296214	0	1.206578	0
LOC107809893	0.422417	0.577472	0.275399	0.382689	0.093446	0.340613	0.17715	0.72185	0.099146	0.308933	0.07405	0.267652	0.822922	0.115209	0.216779	0.629572	0.918204	0.236198	0.10716	0.178668	0	0.088193	0.09315	0	0	0.146941	0	0	1.016834	0
LOC107806734	1.898627	13.2322	5.145477	0.10118	0	0.240148	0	0	0	0.108906	0.078313	0	0	0	0	9.071742	8.631685	6.411413	0.113329	0.566862	0	0	0	0	0	0	0	0	0	0
LOC107776046	0	0	0	0	0	0	0	0	0	0	0	0	0	0	0	0	0	0	0	0	0	0	0	0	0	0	0	0	0	0
LOC107794499	58.35859	44.7763	50.53216	48.86642	42.98501	48.34955	56.44267	50.91818	66.99221	44.64474	55.25257	54.82072	90.35861	79.19301	61.59854	45.64396	57.41523	51.33361	21.62984	17.39952	24.27086	48.73679	62.10957	71.0832	10.68128	7.188794	18.16551	6.971266	23.30896	6.027394
LOC107765614	1.81211	2.09787	2.022253	0.377143	0.30336	0.526551	1.15019	0.962466	1.103538	0.310425	0.824207	0.496515	0.299864	0.293867	0.628344	2.043828	2.531346	2.00825	0.472125	0.393588	0.201001	1.431538	1.879201	1.151767	0	0.136293	0.115771	0	0.117894	0
LOC107785691	41.46969	23.27017	27.4586	12.93713	13.89966	14.73884	11.11504	13.47033	15.0157	24.17379	21.82896	35.56558	27.85139	30.59714	26.32362	28.22157	30.075	30.61931	8.114712	9.567411	10.99207	6.296804	9.069176	8.955395	3.755338	2.225423	16.74302	0	7.974994	0
LOC107788571	2.183536	2.081519	2.421716	2.819465	2.420722	2.374552	3.6418	2.766342	3.605172	1.321587	1.77748	2.502023	2.486622	2.519034	1.700161	3.291753	2.206467	3.255837	2.266637	2.547753	2.494999	3.793776	2.811551	2.99051	0.666365	1.152432	1.483198	3.58974	2.295794	1.551854
LOC107774343	14.72675	15.97812	14.77114	3.909653	2.673066	6.321624	0.76917	1.567106	0.759677	53.49659	62.45017	42.1555	13.69171	7.944805	10.74116	13.02455	10.47503	14.80009	5.473879	8.944074	6.248142	0.405451	0.570988	0.563824	18.14168	8.106408	34.93918	0	6.752364	0
LOC107760820	0	0	0	0	0	0	0	0	0	0	0	0	0	0	0	0	0	0	0	0	0	0	0	0	0	0	0	0	0	0
LOC107806417	35.85446	34.21465	35.89118	38.41681	35.26129	45.19044	23.10081	22.38795	27.93109	25.16341	29.00368	22.63952	22.20803	20.05851	17.64873	32.69122	35.93286	36.64647	26.63863	31.00208	17.10803	25.52925	30.11583	39.1608	12.6111	6.35263	16.8042	3.548928	9.317686	10.73948
LOC107798791	14.51206	15.77633	17.26185	19.97269	16.99995	13.94366	21.81612	20.72203	16.8244	14.50094	16.55308	11.53277	14.19806	15.49926	20.32613	19.50336	15.49291	18.46914	20.60525	19.55999	16.68721	23.41487	19.73096	17.66163	19.14144	28.67881	12.67856	15.39408	15.11322	42.14772
LOC107765246	0.368872	0.336181	0.160326	0	0.489603	0	3.557967	0.630348	0	0.539545	1.681244	0	1.437215	0	0.757199	0.687209	0.17818	0.137505	0	0.46806	0	1.386244	0.325369	0.192772	0.233183	1.283145	0	0	3.551758	0
107825527	0	0	0	0	0	0	0	0	0	0	0	0	0	0	0	0	0	0	0	0	0	0	0	0	0	0	0	0	0	0
LOC107804543	0.356325	0.162373	0.309746	0.161406	0	0.095773	0.224149	0.076113	0.083633	0.086865	0.124928	0.301033	0.694165	0.388733	0	0.066383	0.17212	0.398483	0.090393	0.150713	0.081244	0.223182	0	0.093108	0	0	0.105287	0	0	0
LOC107767833	15.94741	17.10366	17.9603	12.97086	13.76049	12.78802	14.85377	13.40006	11.87017	10.30988	9.452472	11.83524	11.67178	13.40884	9.088302	13.26284	14.93839	13.40029	9.02992	10.43457	11.24974	10.63241	10.68601	13.07673	7.29633	8.091258	9.528415	6.300914	12.72539	16.34341
LOC107775964	0	0	0	0	0	0	0	0	0	0	0	0	0	0	0	0	0	0	0	0	0	0	0	0	0	0	0	0	0	0
LOC107794497	0	0	0	0	0	0	0	0	0	0	0	0	0	0	0	0	0	0	0	0	0	0	0	0	0	0	0	0	0	0
LOC107819851	0	0	0	0	0	0	0	0	0	0	0	0	0	0	0	0.063059	0	0	0	0	0	0.070668	0	0	0	0	0	0	0	0
LOC107832084	2.059732	2.875192	2.515739	0.472408	0.230707	0.728811	0.284286	0.556927	0.465083	0.177968	0.603309	0.264322	0.464389	0.426658	0.294362	2.176081	1.486109	2.196516	0.502676	0.1985	0.332901	0.544347	0.643936	0.926535	0.065927	0	0.21571	0	0	0
LOC107827809	0.258028	0.448944	0.550551	0.233761	0.103782	0.277412	0.196745	0.521103	0.08809	0.091495	0.082241	0.039634	0.052226	0.153543	0.072227	0.227244	0.271939	0.279813	0.095211	0.059529	0.106966	0.352614	0.537959	0.220656	0	0	0	0	0	0
LOC107832018	0	0	0	0	0	0	0	0	0	0	0	0	0	0	0	0	0	0	0	0	0	0	0	0	0	0	0	0	0	0
LOC107796484	19.63934	22.82718	25.29827	17.50276	14.07219	18.39277	13.87472	14.42819	14.3537	10.95361	13.46088	10.48672	10.42518	10.13624	9.687603	20.0754	21.11041	21.51397	13.21925	13.55697	9.168668	14.06121	15.56703	20.93809	6.246369	3.659521	9.877497	5.273431	4.970128	3.039624
LOC107783289	0.164329	0.037441	0.107136	0.111655	0	0	0.034457	0.035102	0.077139	0.120181	0.086421	0	0.091466	0	0.084331	0.214301	0.158755	0.183771	0.166749	0.069505	0.074935	0.137235	0.036237	0.128817	0.467463	0.228651	0.291335	0	0.593353	0
Novel02278	0.779672	1.313486	0.790711	1.348473	1.547059	0.685835	2.016328	1.842068	1.242165	1.503278	2.207534	1.072864	1.518897	1.862262	3.273683	1.879487	1.004305	1.184575	1.77411	1.988639	1.115093	2.338128	1.417124	1.055682	3.196194	2.687485	1.242641	0.422406	4.549839	6.208641
LOC107787983	1.25432	1.186569	1.090354	1.179503	0.842966	0.76816	1.118638	1.044593	0.983827	0.526406	0.990863	0.617031	0.795376	0.467681	0.945179	1.774786	0.966355	1.19557	0.725011	0.980481	0.506818	1.498345	1.344478	1.228039	0.622292	1.458094	0.544211	2.270927	0.687958	1.963455
LOC107795923	1.747757	1.254985	2.117796	0.095962	0.046865	0.455528	0	0.045252	0.099447	0	0	0	0	0.057779	0	1.184028	1.841979	2.053255	0	0.044803	0	0.132691	0	0	0	0	0	0	0.25498	0
LOC107813075	0	0	0	0	0.058744	0	0	0	0	0	0	0	0	0	0	0	0	0	0	0	0	0	0	0	0	0	0	0	0	0
LOC107830428	0.130298	0.11875	0	0.118043	0	0.070043	0.054643	0	0.183494	0.127057	0	0	0	0.213223	0	0.291294	0.251757	0.242857	0	0	0	0.057466	0	0	0	0	0	0	0	0
LOC107796779	0.531933	0.256654	0.625597	0.340168	0.609127	0.807378	0.997288	0.481233	0.528779	0.396654	0.285229	0.422957	0.731486	0.512041	0.481731	0.746159	0.604579	0.863142	0.158756	0.238224	0.171223	0.313575	0.6348	0.228931	0.03956	0.043538	0	0	0	3.869438
LOC107803197	0.220392	0.167383	0.255442	0.299496	0.357531	0.236948	0.092426	0.06277	0.068971	2.364007	2.034761	1.923995	1.880964	1.883419	0.150803	0.054745	0.070972	0.109541	2.497294	1.833289	0.904504	0.184055	0.4212	0.652668	5.061998	2.044393	16.8013	2.626854	12.02517	0
LOC107800425	0.996979	0.978518	0.799987	0.833735	1.492942	1.401682	0.83621	0.982899	1.296011	1.271313	1.021736	2.073294	1.280598	1.254987	0.47228	1.2573	1.111345	1.772462	0.856025	1.038001	1.328921	0.89665	1.691157	1.843614	0.290881	0.533548	0.906423	0	0.369216	0
LOC107795685	0	0	0	0	0	0	0	0	0	0	0	0	0	0	0	0	0	0	0	0	0	0	0	0	0	0	0	0	0	0
LOC107814612	1.256201	1.0061	0.761083	1.37946	1.077885	1.145938	0.925917	0.520403	1.215115	1.187834	0.587232	0.900474	0.847527	0.74752	0.898624	0.879388	0.919391	0.709508	0.733921	0.644036	0.659632	0.508648	0.805856	0.915108	0.770046	0.317802	0.989819	0	0.733066	0
LOC107832463	6.987053	8.096853	7.26028	12.49215	13.30693	12.78522	15.5823	12.098	12.70681	10.91926	12.79989	9.655666	15.45315	11.15616	15.31623	9.085969	8.091156	8.900393	8.498584	8.435278	7.870436	12.73282	12.55057	12.71956	3.129842	3.154813	3.171999	0	5.457847	4.291589
LOC107806381	0	0.025205	0	0	0	0	0	0	0	0	0	0	0	0	0	0	0	0	0	0	0	0	0	0	0	0	0	0	0	0
LOC107810428	0	0	0	0	0	0	0	0	0	0	0	0	0	0	0	0	0	0	0	0	0	0	0	0	0	0	0	0	0	0
LOC107797966	4.677534	6.561477	5.471409	5.467883	4.673053	5.700989	4.863383	4.107118	3.824833	3.131875	5.229686	3.514655	3.16745	3.433328	4.33629	4.867135	4.102404	4.821153	2.843489	4.066279	3.71554	4.374371	4.411095	4.708719	0.735822	0.8398	0.942642	1.54152	1.037756	0
Novel01094	6.053322	5.216619	6.013742	6.901636	5.975822	6.463788	5.6298	6.544318	6.456339	4.979193	4.735464	6.60995	5.042521	5.660465	3.381205	4.173389	4.893239	4.973519	6.894634	7.141103	6.947859	3.851647	6.211174	5.982607	8.59037	7.3341	8.030605	23.55908	6.542271	10.18465
LOC107764096	0.126043	0.311797	0.422613	0.261002	0.223062	0.271023	0.377562	0.338468	0.608577	0.070233	0.227266	0.167333	0.100223	0.176794	0.166329	0.523309	0.487072	0.349032	0.109628	0.07616	0.082109	0.57142	0.285886	0.414039	0.136591	0.050108	0.063845	0	0	0
LOC107778410	0.537954	0.967656	0.726061	1.590331	1.866489	1.71988	1.864191	1.560366	0.824037	1.366652	2.809251	0.932877	1.875372	1.652535	3.61798	1.803979	1.053095	1.26653	2.197881	2.862165	1.291114	2.447283	1.211252	0.917387	2.326787	2.639533	0.803139	0	2.112816	2.625989
LOC107787442	2.385545	1.118124	1.836709	12.8436	13.72077	12.34745	11.89082	10.10402	13.88586	14.82127	13.07131	18.42629	10.47074	13.90485	7.065551	0	0	0.050815	0.069162	0.057657	0.124323	0.02846	0.03006	0	0.172346	0.142256	0.241673	0	0	0
LOC107764928	15.57649	17.25794	38.09872	21.6416	21.23447	16.67627	30.94168	29.48851	29.73935	37.07914	30.02852	24.82602	30.57565	28.48857	34.72867	52.72199	16.96611	32.46417	28.53482	27.09146	31.25722	27.52884	25.95879	30.21249	36.93205	25.2251	46.72157	29.64264	44.4273	45.86275
LOC107824991	0.032413	0	0	0	0	0	0	0	0	0	0	0	0	0.035361	0	0	0	0	0	0	0	0	0	0	0	0	0	0	0	0
LOC107778629	0.243627	0.471826	0.317669	0.965622	1.589881	0.491116	0.715191	1.274988	1.400956	1.187834	1.537481	0.926202	1.728956	2.093056	1.594081	1.588571	0.941456	1.38496	1.390588	1.030458	0.888767	1.678538	1.15506	1.686981	0.385023	0.46611	0.395928	2.177845	0.733066	0
LOC107796386	10.78202	6.339667	8.888837	1.323402	0.553973	1.121806	1.516948	1.069833	0.783688	0.203494	0.292659	0.23507	0.619494	0.227664	0.142792	4.250656	7.728253	4.563768	0.635275	0.235377	0.063441	1.394222	2.331596	0.581645	0	0	0.164432	0	0.334894	0
LOC107813421	0	0	0	0	0	0	0	0	0	0	0	0	0	0	0	0	0	0	0	0	0	0	0	0	0	0	0	0	0	0
LOC107818212	0.58537	0.335903	0.490003	0.314261	0.49879	0.256399	0.454606	0.500156	0.346026	0.591951	0.775317	0.750963	0.820586	1.040697	1.490893	0.113094	0.020945	0.048491	0.043999	0.0917	0.098864	0.126741	0.019124	0.113302	0	0.060333	0	0	0.208754	0
LOC107762434	0	0	0	0	0.097783	0	0	0	0	0	0	0	0	0	0	0	0	0	0	0	0	0	0	0	0	0	0	0	0	0
LOC107821944	1.885956	1.913399	2.443666	2.256616	3.274662	3.328379	2.626439	2.219492	2.171513	4.406769	3.742737	3.306866	3.367079	4.114971	4.236612	3.182076	3.712739	2.918238	3.610826	2.287728	2.17437	2.496244	3.107363	4.128364	2.609367	1.732943	3.448716	0	4.968118	8.800868
LOC107827542	0	0	0	0	0.14439	0.058478	0.045621	0	0	0.053039	0.190699	0	0.06055	0.178018	0.111653	0	0	0	0	0	0	0.090849	0	0	0	0	0	0	0	0
LOC107819726	0	0	0	0	0	0	0	0	0	0	0	0	0	0	0	0	0	0	0	0	0	0	0	0	0	0	0	0	0	0
LOC107773553	24.52041	20.9209	19.50102	39.41847	58.99811	36.57087	32.99363	56.34455	48.09886	87.50223	45.65482	56.94564	50.7598	54.41177	58.90166	30.01378	35.98678	35.16176	160.3011	122.3371	180.5219	54.02609	57.79904	46.34982	387.5816	294.1438	305.7203	216.3962	243.635	219.3555
LOC107823463	0	0	0	0	0	0	0	0	0	0	0	0	0	0	0	0	0	0	0	0	0	0	0	0	0	0	0	0	0	0
LOC107825757	22.68999	16.67846	15.2377	17.33151	17.79426	18.81407	22.81241	20.80612	19.35312	13.15967	18.34356	17.84107	21.65984	18.2174	17.38257	18.25687	22.80871	21.80329	18.17861	18.44146	25.7521	18.15636	19.77861	16.39939	16.72473	11.80319	12.69022	8.48447	13.13708	25.67501
LOC107793738	0	0	0	0	0	0	0	0	0	0	0	0	0	0	0	0	0	0	0	0	0	0	0	0	0	0	0	0	0	0
LOC107759891	0	0	0	0	0	0	0	0	0	0	0	0	0	0	0	0	0	0	0	0	0	0	0	0	0	0	0	0	0	0
LOC107785090	2.14581	0.852459	1.626165	1.545227	1.022406	2.307008	0.599928	0.37609	0.87815	0.375565	0.617289	0.836695	0.306249	0.600249	0.338831	0.984037	0.691011	1.435711	0.223325	0.511982	1.555576	0.459493	1.698618	1.840246	0.069563	0	0.325151	0	0	0
LOC107759973	1.103064	0.66063	1.095851	0.685248	0.55775	0.813208	0.264339	0.430851	1.153959	0.122929	0	0.026626	0.070168	0.13753	0.032347	0.681089	0.304472	0.493428	0.063961	0.053321	0.229946	0.15792	0.305792	0.296466	0	0	0	0	0	0
LOC107831832	78.51937	77.05835	88.61818	53.73803	54.35561	69.50157	66.70083	70.09564	81.64608	39.31253	42.43894	39.66531	33.46383	44.85949	34.15536	53.26831	82.65522	60.41339	40.50113	42.43863	56.11514	50.96436	77.15182	61.15727	22.87967	20.7037	37.31156	28.75926	20.81289	31.08176
LOC107812771	3.124929	6.563933	9.64981	1.536843	1.2904	0.767927	8.586936	9.052653	11.79117	4.411185	6.907504	3.042317	5.068984	6.039055	13.01264	15.59121	4.140266	8.27622	1.872378	2.391715	2.497132	10.81165	15.01578	9.860737	0.639663	0.662569	0.984911	0	2.579061	3.680363
LOC107799944	2.322929	3.715238	3.306064	3.486794	3.325041	3.378879	3.667464	3.11337	2.159487	5.884945	6.483418	2.789792	2.839438	3.627386	4.60476	4.056086	3.146202	3.141086	4.483202	5.432751	2.471642	5.91491	3.555577	2.118483	15.95133	14.6399	8.505712	8.143247	12.4991	19.71395
LOC107786667	0	0	0	0	0	0	0	0	0	0	0	0	0	0	0	0	0	0	0	0	0	0	0	0	0	0	0	0	0	0
LOC107777711	0.030912	0	0.026871	0.028004	0.027353	0.033234	0.012963	0.052823	0.043532	0.015071	0.054188	0.026115	0.051617	0	0.04759	0	0	0	0	0	0	0	0	0	0	0	0	0	0	0
LOC107777541	0.176244	0.06425	0.030641	0.031934	0.062381	0	0	0.030118	0	0	0	0	0	0	0	0.052535	0.10216	0	0	0	0.064295	0.029437	0	0	0	0	0.166645	0	0	0
LOC107767356	3.342022	3.162988	2.905148	4.483328	2.38854	5.942405	0.215623	0.43931	0.241357	2.005473	3.42501	2.823433	1.502463	1.752876	0.461752	1.293133	2.048966	2.25204	2.934738	3.425165	1.934295	0.858772	0.850351	1.209143	1.381358	1.073118	4.40577	0	0.618834	0
LOC107778460	33.82496	25.70001	36.75415	33.93599	28.32271	40.42332	8.968344	13.37565	17.38726	25.35103	14.8483	20.98938	6.22387	8.157946	9.073763	21.665	26.7701	26.70288	26.16792	33.4909	22.37185	9.834309	18.37001	27.24583	7.510387	31.94388	34.73328	4.997857	2.859888	4.321174
LOC107786337	10.69029	11.11031	11.50543	14.07825	13.32387	13.62498	14.04513	12.63662	13.40718	13.8208	13.73335	14.75817	16.07623	13.59173	12.92468	12.13931	11.9843	13.42327	13.89276	15.95602	13.21956	12.26166	13.25808	14.954	9.891249	9.95379	12.44507	17.24452	11.35109	19.87961
LOC107772787	9.980468	8.321841	7.322137	8.785294	6.764457	9.893114	9.380324	8.104228	8.439738	7.454464	8.189512	7.534779	8.431294	10.65654	4.867564	7.015473	7.590488	6.913142	5.099658	5.029747	6.907461	7.034461	8.865869	12.42912	3.93764	1.9698	3.932037	5.062018	3.407768	8.753296
LOC107783927	7.816502	9.571005	8.681254	12.36321	12.0104	11.98268	9.965176	9.412942	11.49982	15.28002	15.15318	13.12951	16.82961	16.67361	13.00612	10.00507	9.008374	9.707986	10.58175	11.07742	7.313291	11.12024	8.954962	10.61114	15.83515	12.56509	11.71656	9.206901	18.24017	7.960336
LOC107781868	6.179175	5.556966	5.371426	12.32676	11.82263	10.91096	9.404442	10.41945	9.297418	9.596867	8.938279	9.351666	7.675949	8.259057	8.442181	5.290863	4.605687	5.369559	15.23949	14.3141	13.69691	9.158839	9.078052	9.345537	15.77992	13.66549	15.01769	5.852959	15.07139	8.855874
LOC107799703	6.615815	3.388479	4.84794	25.41086	19.30404	27.86341	11.46481	10.60471	18.94165	26.55145	21.46982	24.85126	14.4253	15.74734	7.575945	4.359687	4.806781	4.157867	14.59166	14.10694	10.22244	7.579807	12.49105	15.71556	4.908037	1.217246	17.38354	0	7.896915	0
LOC107769663	0	0	0	0	0	0	0	0	0	0	0	0	0	0	0	0	0	0	0	0	0	0	0	0	0	0	0	0	0	0
LOC107781363	0	0.279168	0.177515	0.370007	0.722792	0	0.171279	0.087241	0.383442	0	0.286384	0	0	0.222783	0	0.152177	0	0.076123	0.828871	0.863736	0.74497	0.170541	1.260885	0.853759	0	0	0	0	0	0
LOC107779315	2.311701	2.749157	2.646675	2.732785	3.043372	3.728042	2.908385	2.529194	2.249729	3.683693	3.91404	3.072386	3.828744	3.044796	3.44326	2.310915	2.233298	2.984548	3.947733	3.934928	3.702398	2.848756	2.710475	2.946572	3.742473	4.236449	3.698526	8.064356	4.207438	1.743121
LOC107827683	1.964619	1.62523	2.3121	0.98576	0.962819	1.624773	0.101404	0.129125	0.198635	0.265258	0.402681	0.434092	0.437408	0.494608	0.217154	1.373942	1.547589	3.199821	1.042785	1.073864	0.964797	0.403866	1.013094	0.821368	0.267494	0.084112	0.643021	0	0.145513	0
LOC107768409	13.73895	6.782405	10.94773	5.186165	4.558924	9.385775	5.401618	5.624906	9.808413	2.511978	4.11442	4.957178	8.603097	7.338131	1.321996	9.598392	18.80339	9.175997	1.742668	2.300229	1.696792	6.095435	6.564277	7.927889	0.361879	0.199133	0.338299	0	1.378004	0
LOC107831044	1.585931	1.726426	1.761565	0.957848	0.467779	0.947261	1.071541	0.60225	1.323504	0.859157	0.957603	0.744355	0.637534	0.384483	0.904309	1.280324	1.276785	1.28091	0.625836	0.931658	1.124974	1.214081	1.74862	1.887838	0.111394	0.24519	0.468611	0	0.42418	0
LOC107818949	1.607912	1.987001	1.871528	1.678893	1.278096	1.670101	1.417199	1.583413	1.30489	1.381893	1.490552	0.805835	1.45623	1.040599	1.426545	3.066625	1.553378	2.519441	1.244439	1.890423	0.695939	1.547644	1.538478	1.224836	0.964761	1.289287	1.127369	1.948956	0.524818	3.370156
LOC107772229	0	0.052517	0	0	0	0	0	0	0	0	0	0	0	0	0	0	0	0	0	0	0	0	0.050828	0	0.145707	0.080179	0	0	0	0
LOC107820632	1.016654	1.772539	0.845331	1.321487	1.564525	0.760367	1.594199	1.359636	0.622486	1.939628	2.510571	1.008271	2.214299	1.109119	1.17957	2.470472	1.537314	1.647736	0.852216	0.785235	2.862242	0.996692	1.24766	1.062606	3.688422	0.553539	2.768911	3.161092	1.702446	0
LOC107798288	30.46894	35.89724	35.1354	39.27984	37.81828	37.41377	40.77655	38.01506	34.75263	32.0236	33.87285	32.2307	33.34453	30.70914	41.5379	39.80183	38.20147	36.36079	35.15523	38.9018	38.64955	43.05483	38.47169	36.42636	22.94373	21.16178	27.23737	33.18213	25.31676	38.25259
LOC107827786	17.42719	17.00124	21.8915	9.028156	7.297696	10.34458	5.146809	5.956107	9.263788	3.973178	4.371647	3.732586	3.224258	3.802442	1.965028	13.42575	16.97841	16.17684	5.180639	5.689246	7.073902	6.272528	8.963355	14.67454	1.737835	0.887979	4.467636	0	2.3634	0
LOC107790667	0	0	0	0	0	0	0	0	0	0	0	0	0	0	0	0.061636	0	0	0	0	0	0	0	0	0	0	0	0	0	0
LOC107789256	5.703785	6.757786	5.206088	8.267745	6.897682	5.416836	7.973351	6.741632	3.926974	3.707941	7.265754	2.48967	2.963116	4.252075	5.756639	10.98037	9.367572	7.654345	5.401953	6.835431	2.340877	12.78174	6.288895	5.961595	2.64415	2.77774	1.46064	0	0.457668	5.877894
LOC107780348	0.94591	0.78126	0.590999	3.240329	4.760456	1.875036	12.84276	12.4515	12.71039	3.084214	9.41018	7.791527	10.13513	5.546684	8.859061	0.484614	0.571143	0.484836	2.639571	2.075453	4.124716	10.9853	7.61349	11.74039	3.326146	2.138768	1.886607	0	4.411636	0
LOC107770619	0.254024	0.490805	0.547628	9.361808	7.741327	5.429366	25.46504	18.97842	13.75604	4.201086	17.26357	6.077659	4.501907	2.915384	5.715056	0.401314	0.382837	0.363621	4.608857	5.054139	3.929195	16.41132	13.94587	8.793555	0.822179	0.9614	1.333048	0	0.63594	2.51307
LOC107771514	0	0	0	0	0	0	0	0.070703	0	0	0.029012	0	0	0	0	0.061664	0	0	0	0	0	0	0	0	0	0.057569	0	0	0	0
LOC107799538	0	0.430069	0.102551	0	0	0	0	0	0	0	0	0	0	0	0	0.527479	0	0	0	0	0	0	0	0	0	0	0	0	0	0
LOC107760549	1.845153	1.578673	1.571219	1.535157	1.132903	2.348131	1.421274	0.997409	1.555545	0.80783	1.45225	0.827138	1.047985	1.437837	1.391374	1.571441	1.564299	1.76868	1.031694	1.114909	0.721205	1.415145	2.059346	1.771121	0.476089	0.052396	0.801119	0	1.087742	0
LOC107768415	0	0	0	0	0	0	0	0	0	0	0	0	0	0	0	0	0	0	0	0	0	0	0	0	0	0	0	0	0	0
LOC107809830	0	0.127983	0	0	0.12426	0	0	0	0	0	0.049234	0	0	0	0	0	0	0	0	0	0	0	0	0.073388	0	0	0.082987	0	0.338036	0
LOC107817634	15.90268	19.69717	17.1055	11.97023	11.00268	13.19082	8.489787	8.74934	8.17392	15.139	14.16207	15.74729	14.02589	11.71189	12.76222	18.19803	18.82806	19.38494	13.45544	13.51245	12.88962	7.763523	8.803433	9.519127	4.892219	10.57126	7.222677	3.374679	10.45049	4.376648
LOC107831496	3.304243	4.465784	3.541426	7.840868	10.06721	7.185668	9.668445	8.854691	6.468727	9.354941	9.912805	8.961402	7.607454	7.41435	8.844577	4.826718	3.573061	4.28305	13.39262	13.2929	14.22863	9.375891	10.61496	12.16607	10.70502	10.94545	10.25154	22.82444	9.400065	10.44748
LOC107769060	0	0.024799	0	0	0	0	0	0	0	0	0	0	0	0	0	0.020286	0	0	0	0	0	0.045448	0.024001	0	0	0	0.03216	0	0	0
LOC107772823	0.084212	0.076749	0.292814	0	0	0	0	0	0	0	0.059049	0.071144	0	0	0	0	0	0	0	0	0	0	0	0.088018	0	0	0	0	0	0
LOC107759105	0.593875	0.270622	0.516243	0.430416	0.4204	0.383093	0.298865	0.304454	0.947845	0.521193	0.083285	0.501722	0.198333	0.323944	0.304768	1.681714	0.573733	1.372553	0.301312	0.050238	0.270812	0.198384	0.366686	0.744861	0.450503	0.661067	0.842295	12.74113	2.001387	0
LOC107791343	4.073233	4.509924	5.150219	3.781634	3.66384	3.981113	4.658722	3.652857	5.40435	3.446705	3.304641	4.294366	3.372679	4.296798	3.005926	3.637437	5.658719	4.065756	3.381738	3.417199	3.223637	2.951857	4.008564	4.398081	1.702415	1.217833	2.347436	2.407387	0.972396	0
LOC107806079	22.33269	19.61486	20.37878	19.11733	14.47578	16.36191	18.37892	16.31174	17.69788	10.30319	11.51324	11.5131	11.66198	10.51183	10.90627	21.29505	17.97938	19.42496	10.59981	12.13755	13.14051	14.16344	17.39541	20.11062	5.768504	3.591923	7.024593	2.146644	4.046358	9.279999
LOC107765098	0	0	0	0	0	0	0	0	0	0	0	0	0	0	0	0	0	0	0	0	0	0	0	0	0	0	0	0	0	0
LOC107790460	0.73219	2.41255	0.832317	0.714352	0.64789	0.54498	1.417199	1.58807	0.846046	0.659057	1.658716	0.428244	0.564289	0.553004	0.867115	1.72083	0.544121	1.553655	0.628672	1.667568	1.181439	1.317015	1.29168	0.588681	0.712085	0.47021	0.332842	0	0.813467	0
LOC107830039	7.596512	7.302417	8.584533	7.603033	6.625867	5.775363	6.826631	7.047009	7.726269	4.83255	5.288631	3.514479	3.926252	4.005587	4.585292	7.588342	7.303923	8.481799	4.569989	5.263304	4.420818	8.18684	8.136475	10.79445	2.652616	2.365665	2.843181	0	3.221847	0
LOC107791247	5.623594	6.29669	5.656659	9.679913	6.113526	8.982705	8.15324	8.58027	8.447484	3.21189	4.506592	4.004388	4.292752	4.031614	3.463142	4.729514	6.364213	4.432207	4.97275	5.096972	4.909006	7.983897	9.495588	10.3281	1.015705	1.229615	2.088943	0	0.773542	9.934723
LOC107805896	7.822006	8.058059	7.495914	11.49386	8.661448	9.239611	5.290061	5.052949	7.480397	4.715663	6.894296	5.106933	3.421408	3.098729	2.84055	7.31606	7.233057	7.123937	5.321049	5.199856	4.702729	5.328071	7.362064	11.4336	0.147328	0.445889	1.256767	1.041683	0.070126	0
LOC107830188	4.186487	5.590102	4.527872	1.05844	0.689205	1.177583	0.979922	1.62215	1.462496	1.115527	1.297111	0.987031	1.138018	0.955936	0.874368	4.879182	4.538303	3.919651	1.037341	1.42071	0.732551	1.300928	1.674623	1.29745	0.215412	0.846685	0.489055	0	0.70309	0
LOC107793570	4.721556	3.496282	5.087682	2.406088	2.524174	4.758984	1.443813	2.77353	2.585803	1.246947	1.517431	1.329634	1.259274	1.180434	1.261999	3.481858	3.421065	3.923467	2.395554	2.57953	1.704513	2.094769	2.819866	4.009662	1.119278	0.684344	1.802036	0	0.473568	0
LOC107767853	0.908043	0.522675	0.623165	0.822641	0.465182	0.359674	1.964165	0.857526	1.031987	0.512633	0.368628	0.48451	0.638429	0.834215	0.490521	0.356145	1.292784	0.997662	0.193983	0.404285	0.348695	0.718419	1.053887	0.949087	0	0	0	0	0	2.955036
LOC107761085	14.05909	16.32795	17.31093	19.18465	18.39692	17.60304	23.20165	20.75971	25.8061	28.47861	28.00135	23.75502	42.81522	38.47832	32.96199	15.38869	17.95162	14.11494	10.49508	10.97361	9.56064	19.00245	20.10145	18.87207	16.75546	7.171181	21.87937	15.04375	21.77413	13.00691
LOC107824308	1.823852	2.959912	2.447691	3.043743	2.661461	2.167271	3.998803	2.774955	2.808794	2.511722	3.544983	2.797843	2.689307	3.263861	3.497595	2.742118	2.581168	2.81494	2.597501	2.787964	2.699351	3.313512	2.709488	2.524998	2.40704	2.893934	1.720736	3.432401	3.620103	7.913792
Novel03715	2.584528	2.522325	2.508787	1.951209	1.762865	2.002968	1.363879	1.82184	2.022057	0.829579	1.049608	1.228208	1.258745	1.080844	0.906335	2.527866	3.079474	2.320279	1.278518	1.375568	1.679457	2.203373	2.754667	3.691842	0.503759	0.494475	0.547301	0	0.362916	1.33171
LOC107798091	6.345803	7.694885	10.70531	0.828242	0.38069	0.982905	0.315741	0.505443	0.302935	0.419523	0.414801	0.408898	1.257195	0.938706	0.331178	5.971238	7.221614	8.459786	0.818553	0.682387	0.735696	0.31438	1.56538	0.562087	0.067992	0.074828	0.381368	0	0	0
LOC107818109	5.546619	4.44356	5.676996	5.673337	5.77879	7.261752	5.590129	5.923977	6.047315	7.066144	5.300729	7.217855	5.128879	7.319865	4.774688	4.700015	7.043835	5.836024	5.219818	7.227282	6.241671	6.537275	6.036694	6.218098	5.881546	4.792452	5.709766	9.596653	8.829349	2.765772
LOC107827833	2.149354	2.233113	1.737601	1.947206	2.282268	1.733114	2.650051	2.111937	1.654685	1.92822	2.441548	2.342426	1.674884	2.391737	2.250159	2.114246	1.931105	1.794727	1.679382	1.254562	1.97985	2.351426	2.445676	2.089249	0.597836	0.957014	0.736705	1.537092	1.759119	0
LOC107786766	6.268435	10.58326	9.251569	10.80622	10.97373	8.412046	10.42065	9.555887	7.663309	7.453751	10.65655	4.381374	7.279322	6.12518	11.22435	10.98919	7.820305	11.5826	9.586923	12.53184	5.285064	15.13988	9.20866	8.413599	5.958159	9.569177	3.011485	0	4.01656	8.365182

LOC107789143	11.70712	12.22245	9.890085	3.137004	2.48038	3.663702	2.489392	2.207209	3.147704	4.234075	2.736348	4.086217	7.281087	8.87437	1.466726	7.741158	13.43402	8.974021	1.003908	0.883404	2.055215	0.596714	2.181646	1.608525	0.27796	0.076477	0.584657	0	0.264612	0
LOC107832806	0	0	0	0	0	0	0	0	0	0	0	0	0	0	0	0	0	0	0	0	0	0	0	0	0	0	0	0	0	0
Novel00160	0	0	0	0.098656	0.337259	0.117078	1.5984	3.023966	1.482449	0.743323	0.80177	0.781998	2.000235	4.870881	7.656214	0	0	0.121782	1.491774	1.059379	0.496582	3.183014	1.536879	0.853648	3.786192	13.94013	3.796893	42.83246	7.339808	13.46661
LOC107810923	0	0	0	0	0	0	0	0	0	0	0	0	0	0	0	0	0	0	0	0	0	0	0	0	0	0	0	0	0	0
LOC107822930	0	0	0	0	0	0	0	0	0	0	0	0	0	0	0	0	0	0	0	0	0	0	0	0	0	0	0	0	0	0
LOC107775799	5.935679	8.011644	6.826875	2.067635	1.397536	3.405376	0.575003	0.570926	0.268857	0.617729	0.857977	0.117301	0.231848	0.246146	0.285016	8.529677	5.248117	7.433728	2.219043	4.316449	0.427377	0.775443	1.224716	0.426296	0.109715	0.169045	0.225645	0	0.125335	0
LOC107799480	13.26851	12.09261	12.85285	25.29917	24.84692	23.05666	20.33823	17.62065	17.40609	18.45488	19.43719	21.11577	21.84116	22.94727	23.54195	9.849386	8.818927	8.799491	21.55279	18.68533	22.0681	15.84824	16.53488	12.14815	17.88079	19.85756	14.01075	22.06712	22.03589	12.71957
LOC107795494	16.91082	18.33872	16.0778	20.19415	20.1301	15.15474	19.95531	19.98995	16.95334	15.43286	16.96072	15.81934	17.66474	16.56077	18.40549	18.27363	15.35019	17.69125	19.88944	19.05034	19.79691	18.98115	18.81571	16.89059	15.21147	19.35219	11.95291	16.4032	17.16636	33.52178
LOC107763385	41.19615	34.30127	41.66957	30.22364	27.15343	30.2745	22.04607	20.83873	26.43703	19.14099	22.35918	15.8894	17.39684	14.61336	15.23989	36.16128	35.13945	38.98853	20.21761	20.56026	12.92724	24.20239	28.49799	36.89445	6.044566	5.06984	8.488482	4.518569	2.940519	0
LOC107832765	0	0	0	0	0	0	0	0	0	0	0.044818	0	0	0	0	0	0	0	0.032429	0.027035	0.029147	0	0	0	0	0	0	0	0	0
LOC107810029	0.250421	0.228228	0.253967	0	0.036932	0.179489	0	0.285289	0	0	0	0	0	0	0	0.279921	0.483856	0.1867	0	0.10592	0.114194	0.243989	0.257703	0.087247	0	0	0	0	0	0
LOC107761217	0	0	0	0	0	0	0	0	0	0	0	0	0	0	0	0	0	0	0	0	0	0	0	0	0	0	0	0	0	0
LOC107773869	9.207775	9.775008	9.47008	5.683409	4.864711	9.42808	4.243383	3.890459	5.541452	4.209814	5.108438	3.960728	5.782178	7.027982	2.804027	7.389464	9.677506	7.920908	4.07277	6.162839	2.276285	3.999747	5.622831	5.39365	1.236635	0.234651	2.870202	0	0.324758	2.08546
LOC107769692	2.110447	1.877065	1.834567	0.092143	0.044999	0.355384	0.106634	0.130353	0.023872	0.024795	0.053488	0.042963	0.141529	0.110959	0.052195	1.440069	1.326493	1.93361	0.103207	0.086038	0.11595	0.084939	0.426139	0.079729	0	0	0	0	0	0
LOC107771956	18.41297	14.33173	16.75163	12.12238	9.715087	15.80007	8.729078	7.279966	11.64987	14.66509	12.10925	18.93748	10.18513	14.84739	7.805931	9.460074	12.98228	11.25497	9.342106	8.755514	6.623303	7.306536	10.94533	12.2524	8.964813	4.37614	13.31442	8.178807	8.809601	0
LOC107828509	0.468274	0.609677	0.465212	121.8759	155.7991	176.3519	57.17483	83.50779	102.1212	175.5401	58.30626	273.9886	77.89545	128.0078	72.84876	0.947172	0.387764	1.147102	224.9599	210.9661	355.6912	88.99582	58.94791	124.5973	100.816	102.3894	426.561	43.05625	68.59928	41.36296
LOC107777955	0	0	0	0	0	0	0	0	0	0	0	0	0.046648	0	0	0.09368	0.040483	0	0	0	0	0	0	0	0	0	0.049527	0	0	0
LOC107772016	20.26253	19.72923	18.17384	32.42358	32.89472	34.59922	29.45089	29.50617	28.88283	25.38138	24.82464	28.79366	32.59759	32.01596	30.51755	18.9886	23.12314	17.91654	25.3989	27.16897	28.14565	28.19398	27.10769	29.99991	19.42732	23.53221	21.51193	13.82249	26.9855	15.93466
LOC107793702	8.908414	6.67469	7.967277	7.100551	6.783713	8.242291	6.178662	5.891664	5.428306	5.011632	4.895161	6.549133	6.674884	5.793806	5.362928	6.798141	7.199482	6.960908	5.562857	5.470777	5.038829	6.187785	6.346693	8.460541	3.844562	2.204952	6.681884	0	5.773422	0
LOC107787794	115.666	93.99312	109.3614	117.7885	110.5182	124.6122	127.0651	114.4412	180.2687	139.1437	59.60451	82.03773	91.00602	95.23958	51.15901	88.65408	89.74568	78.56629	86.83816	74.17318	122.3729	87.12788	95.69596	122.644	333.9017	49.5868	299.7623	116.3109	335.7725	189.295
LOC107788530	0	0	0	0	0	0	0	0	0	0	0	0	0	0	0	0	0	0	0	0	0	0	0	0	0	0	0	0	0	0
LOC107807021	0	0	0.059133	0	0.030097	0	0	0.058123	0	0.033167	0.071549	0	0.075727	0.185532	0.768016	0.076039	0	0	0.034514	0.028773	0.03102	0.142025	0	0	0	0.047326	0.0402	0	0	0
LOC107828156	0	0	0	0	0	0	0	0	0	0	0	0	0	0	0	0	0	0	0	0	0	0	0	0	0	0	0	0	0	0
LOC107803429	9.728313	13.29924	8.417465	10.42506	7.99053	8.013821	19.64337	18.31748	21.64947	2.283747	3.237054	2.41616	6.918971	5.232854	4.27593	4.698783	4.764451	3.710381	2.193693	2.666962	3.265531	15.17677	11.26258	7.720171	0.512481	0.313339	0.425855	0	0.975741	1.392399
LOC107807055	5.623455	5.786387	6.898881	4.848054	4.454325	5.509572	5.173082	3.991111	3.448736	3.316681	3.91137	3.563138	2.574722	3.166407	2.466962	5.204461	6.615664	6.052112	3.911572	3.836335	3.102028	3.900954	5.480286	5.451031	1.834776	1.829953	2.412026	0	2.183332	2.804086
LOC107822323	1.794492	3.005707	2.613909	4.042328	4.205748	4.380028	4.515353	4.185392	2.959692	1.513389	3.740888	2.335516	1.295775	1.79897	2.439163	1.301115	3.37355	2.38647	2.066997	2.092401	2.300096	3.604806	2.652361	2.83875	0.613185	0.944775	0.859843	0	0.233495	2.998814
LOC107796928	0	0	0	0	0	0	0	0	0	0	0	0	0	0	0	0	0	0	0	0	0	0	0	0	0	0	0	0	0	0
107801030	0	0	0	0	0	0	0	0	0	0	0	0	0	0	0	0	0	0	0	0	0	0	0	0	0	0	0	0	0	0
LOC107807000	15.50828	17.67759	17.95515	15.54631	14.55804	13.97898	13.59185	12.99144	12.74515	10.30085	13.37238	7.614247	13.19822	11.94122	15.17908	16.9667	12.35497	16.20366	11.50606	12.9288	7.143648	15.52287	13.35221	12.76772	9.746755	10.75804	7.358378	6.429339	12.60604	12.50739
LOC107814087	0	0	0	0	0	0	0	0	0	0	0	0	0	0	0	0	0	0	0	0	0	0.262988	0	0	0	0	0	0	0	0
LOC107760838	1.66881	2.889737	1.758926	2.607953	2.971803	2.153009	3.744204	3.386456	2.604725	2.339244	3.36424	2.167655	3.343936	3.049487	3.661154	2.347293	2.398163	2.379472	3.048107	2.717519	2.340048	3.850004	3.238401	1.831451	2.373618	3.86037	1.725849	4.475381	4.318406	6.449063
LOC107815865	0.582717	0.059008	0.506543	0	0.057292	0.06961	0.108611	0.055321	0.243147	0	0.0454	0.328197	0.072076	0	0	0.48249	0.187651	0.627525	0	0.054771	0	0.324428	0.399774	0.067673	0.081859	0	0	0	0	0
LOC107810232	38.40602	29.46869	40.22932	23.02925	19.43421	16.83505	19.39589	16.63097	22.47442	6.685604	10.06322	6.749454	16.49364	13.02611	10.94306	31.99748	23.2949	32.6619	12.17499	7.937895	16.7451	17.90508	21.03837	29.45363	2.497631	1.657342	5.081798	4.155179	8.112113	10.77777
LOC107776017	5.96006	5.275172	5.280588	4.101553	4.259651	4.559392	3.941487	3.770362	5.434157	1.676484	3.455875	3.582756	2.870837	3.876272	1.999868	4.441444	6.145472	5.42617	2.384252	2.036112	3.449563	4.642385	5.560466	6.528946	0.724551	0.239221	1.016007	0	0	0
107801120	0	0	0	0	0	0	0	0	0	0	0	0	0	0	0	0	0	0	0	0	0	0	0	0	0	0	0	0	0	0
LOC107797953	1.527942	2.001763	1.784779	1.68704	2.070286	2.156076	2.523053	2.366246	1.88278	1.583057	1.874939	2.097628	2.498236	2.344092	1.813275	1.814682	1.522249	2.135898	1.211289	1.090575	1.52415	1.515279	2.611258	2.595132	1.931783	1.328762	1.580166	0	2.068891	5.904685
LOC107778014	0	0	0	0	0.410169	0.33224	0	0	0	0	0	0	0	0	0	0	0	0	0	0	0	0	0	0	0	0	0.182621	0	0	0
LOC107774662	7.957397	31.47308	17.19226	0.769893	0.615253	2.990158	0	0	0	0.075335	0.054172	0	0	0.674262	0	22.68316	22.54009	20.90802	0.940732	0.849596	0.70459	0	1.022177	0	0	0	0	0	0	0
LOC107809392	0	0.056966	0.054335	0.028313	0.221237	0.033601	0.786393	1.041428	0.38144	0.548558	0.43829	0.343242	0.452284	1.397908	2.149163	0.069869	0	0	0.665978	0.713819	0.997609	2.792707	0.827012	0.653309	6.322095	5.6097	2.327116	13.41012	7.523115	23.18892
LOC107807669	12.69419	15.47823	13.18148	7.300525	7.092288	7.965099	11.6283	9.106413	10.41287	8.956409	10.42014	8.308665	9.35659	8.365958	12.22855	13.56001	14.5657	14.43833	6.506536	5.607428	5.847925	11.90379	9.897787	8.649079	4.327288	2.773036	4.198933	3.09834	5.631696	0
LOC107779954	0	0	0	0	0	0	0	0	0	0	0	0	0	0	0	0	0	0	0	0	0	0	0	0	0	0	0	0	0	0
LOC107807587	0	0	0	0	0	0	0	0	0	0	0	0	0	0	0	0	0	0	0	0	0	0	0	0	0	0	0	0	0	0
LOC107765057	3.809437	4.390847	3.87149	6.420167	5.328903	6.896286	5.732455	5.073794	5.706569	3.223033	4.262103	4.354195	4.677263	3.575304	4.254893	3.318888	3.274328	4.15573	5.997293	5.923753	5.415775	5.497204	5.954429	5.035582	3.647629	3.42977	3.34373	2.003152	3.77588	10.39161
LOC107759071	5.768985	8.802695	6.952414	2.57361	2.417035	2.513837	5.535175	4.499741	4.267291	2.642275	4.796032	2.344593	5.400415	2.36013	3.700705	10.61736	8.993319	8.276211	1.064358	1.016701	1.514649	5.456558	6.110207	4.453786	0.359162	0.182435	0.258276	0	1.893678	1.351155
LOC107763195	6.715354	10.82808	7.813289	12.16754	12.25005	8.885941	13.25794	15.18305	13.09432	13.29812	13.402	10.29925	12.88108	13.97599	15.58755	8.083853	10.67958	10.24424	15.09625	17.21697	8.762769	13.80472	11.9379	9.070561	13.5844	21.41915	8.913871	7.38835	4.973862	25.55203
LOC107824438	1.231148	3.077109	1.670178	3.886853	3.829409	1.965397	7.854148	8.415412	4.798555	5.202275	6.592335	2.694826	3.384797	3.093251	9.898332	13.72009	1.243456	4.116523	5.848921	6.453938	3.385496	9.237898	7.897848	4.269822	3.702692	6.670524	1.940158	4.002023	7.723303	11.5339
LOC107774430	0	0	0	0	0	0	0	0	0	0	0	0	0	0	0	0	0	0	0	0	0	0	0	0	0	0	0	0	0	0
LOC107774425	16.27968	15.06491	16.91164	13.3016	12.39607	11.87532	11.94362	11.2791	13.09806	8.425246	8.986528	6.71419	8.41873	7.431626	8.907524	16.72043	15.35573	17.3163	10.15382	9.929795	7.61672	13.98127	14.59738	16.27134	4.233272	4.150186	5.18559	4.128465	5.002733	4.759323
LOC107818070	11.44663	19.98807	13.65182	20.40828	23.25556	13.68485	18.62551	18.03489	9.714924	26.20816	25.87705	24.79498	18.29829	14.48574	25.0948	12.1809	10.61607	9.455612	21.60436	18.59147	21.29661	13.84198	12.88337	14.02182	13.95102	13.50622	9.79499	52.39012	19.6185	14.15524
LOC107814601	14.2002	13.79576	14.20283	11.71098	10.84313	11.39209	11.84986	11.84562	15.15897	7.684962	8.053362	6.515988	7.007885	6.736671	7.422985	12.96341	11.95444	13.93667	7.558221	7.398496	5.14965	11.99942	13.28849	14.33978	2.126457	2.708024	3.322621	1.7183	2.776237	1.485651
LOC107787778	1.693928	1.844548	1.434249	0.876921	0.934378	0.638595	0.627353	0.733067	0.888109	0.085807	0.046277	0.074342	0.048979	0.048	0.045158	2.229547	2.125294	2.246969	0.401816	0.409413	0.240762	0.440928	1.377728	0.620822	0.027813	0	0	0	0	0
LOC107788625	0	0.025556	0.024375	0	0	0	0.047038	0	0	0.027343	0	0	0.06243	0.030591	0	0.125375	0	0	0	0.02372	0	0	0	0	0	0	0	0	0	0
LOC107773786	1.014086	0.803665	1.45643	0.439383	0.507187	0.568835	0.295846	0.527412	0.248366	0.386946	0.309164	0.707732	0.294494	0.288605	0.181014	0.887124	1.235265	1.610703	0.268441	0.223786	0.361903	0.441856	0.311127	0.875588	0.111488	0.061349	0	0	0	0
LOC107810898	23.88562	19.36143	24.1831	15.63111	13.92461	17.40195	13.1988	14.55008	21.053	9.152188	9.812716	7.976733	11.63695	10.3619	5.883731	18.84663	23.67277	24.93084	9.866079	10.98233	14.14684	14.45607	26.02604	29.78207	3.765954	4.379213	8.236775	4.019199	10.28181	3.475021
LOC107787435	0.214965	0.326523	0.062288	0.129831	0.190215	0.154076	0.2404	0.183672	0.201818	0.069873	0.4522	0.484289	0.957207	0.703548	0.588357	0.266987	0.761472	0.427374	0.290842	0.181845	0.522804	0.299205	0.632044	0.59915	0.452968	0.199405	0.338761	0	1.37989	0
LOC107784505	20.14606	17.95761	21.74118	17.18294	13.00035	16.42951	12.69363	11.20966	18.63722	10.68494	10.50869	11.0007	11.54164	10.72115	7.161429	18.85763	18.84568	19.85537	7.628667	8.479525	9.590096	11.69508	18.98373	23.93269	2.050085	1.230663	5.459113	0	1.892494	0
LOC107767487	0	0	0	0	0	0	0	0	0	0	0	0	0	0	0	0	0	0	0	0	0	0	0	0	0	0	0	0	0	0
LOC107806678	11.94327	9.226178	11.07416	5.97676	4.227276	7.031688	3.196006	3.012802	5.286066	2.218323	2.472509	2.690668	1.836033	2.047496	1.692805	9.663006	8.955842	10.21864	2.885525	2.213078	5.290624	3.704679	6.120189	8.084294	0.287618	0.237403	0.268876	0	0.273805	3.516525
LOC107787012	0	0	0	0	0	0	0	0	0	0	0	0	0	0	0	0	0	0	0	0	0	0	0	0	0	0	0	0	0	0
LOC107814377	0.072348	0.16484	0	0.098315	0.128036	0.116674	0.030341	0.061816	0	0.070548	0.177556	0.275046	0	0.118392	0.037128	0.053914	0.034947	0.242722	0.11012	0.030601	0.098974	0	0.063815	0.075618	0.09147	0.050333	0.042755	0	0	0
LOC107761872	19.59039	25.46733	23.48987	113.0048	164.5102	114.9624	80.33635	92.10017	62.93578	126.0109	87.90296	153.1236	201.3048	174.7716	162.2293	22.13078	28.83409	28.47955	168.926	138.21	147.3262	85.40202	49.66366	67.48647	266.0985	255.5028	176.6259	223.2229	318.2956	387.8376
LOC107820046	24.69033	27.36495	29.49616	17.18685	15.08967	20.47124	12.08016	11.56113	13.19447	9.045573	10.41707	8.632357	6.599654	7.418795	6.514334	22.16747	24.12246	25.97356	11.81928	14.39622	10.97275	14.44533	17.81038	21.32302	4.497232	3.493707	6.55358	4.987874	2.51839	0
LOC107765698	0	0	0	0	0	0	0	0	0	0	0	0	0	0	0	0	0	0.057009	0	0	0	0	0	0	0	0	0	0	0	0
LOC107778265	3.791435	3.488439	4.009557	2.428459	2.051411	2.557396	1.984993	1.537217	2.698015	0.953718	1.566347	1.091506	0.900591	1.211901	1.363238	3.680195	3.406213	4.01496	1.237503	0.939716	0.847945	2.843643	3.141935	2.890072	0.07633	0.084005	0.156984	0	0.232526	0
LOC107795880	3.214132	5.472291	2.763274	1.183935	1.125129	1.898673	3.140199	3.500704	5.504577	0.551065	1.48599	1.223417	1.808668	2.543147	1.087549	5.316763	5.152455	3.133587	0.86017	0.657325	0.837527	2.536722	2.679304	1.550497	0	0.344018	0.125237	0	0.340087	0
LOC107796497	0	0	0	0	0	0	0	0	0	0	0	0	0	0	0	0	0	0	0	0	0	0	0	0	0	0	0	0	0	0
LOC107783133	0.05307	1.64446	0.830382	5.432863	1.784462	2.05403	1.335354	1.405669	0.398593	3.984721	14.84778	1.255372	0.413545	0.173689	3.050278	3.91521	0.820316	0.633051	7.808458	16.0718	1.984418	3.900148	4.40023	2.218736	1.811597	6.645831	2.069895	0	0.510993	6.562755
LOC107825308	2.021426	1.519074	1.6647	2.506003	2.32216	1.792003	1.487243	1.151441	1.298497	1.037445	0.845482	0.659134	2.842459	1.741008	2.984708	2.272767	2.46678	2.273808	2.375078	2.459982	3.331372	1.836229	1.25125	1.890395	0.269021	0.148035	0.041915	0	1.365876	6.578306
NitaMt010	0	0	0	0	1.116845	0	0	0	0.592487	0	0	0	0	0	0	1.410848	0	0	0.640378	0.533851	1.151114	1.054065	0	1.978819	0.797879	1.756208	2.237663	0	9.114752	0
Novel04722	0.989631	0.901927	0.921713	0.896556	0.813141	1.67196	0.652179	0.664375	0.53092	0.689297	0.594797	0.895787	0.94429	0.462702	0.290209	0.105353	0.136581	0.105402	0.286917	0.358783	0.193406	0.118067	0.062351	0.147766	0.089371	0.098357	0.083547	0	0	0
LOC107783175	0.088679	0	0	0	0	0	0.03178	0.049586	0	0.028824	0.020727	0	0	0	0	0.057114	0	0.029995	0	0	0	0	0	0	0	0	0	0	0	0
LOC107760233	0	0	0	0	0	0	0	0	0	0	0	0	0	0	0	0	0	0	0	0	0	0	0	0	0	0	0	0	0	0
LOC107792200	109.2995	121.2579	116.6434	74.83038	64.60245	89.65805	73.61066	73.31429	85.69912	45.23873	52.44686	37.03638	44.66423	38.47761	48.46076	110.4397	118.2296	135.0508	49.55227	53.19081	57.86374	97.08449	105.8451	118.5994	8.143464	13.02885	17.60674	22.32114	15.57309	15.79007
LOC107791495	0	0	0.109453	0.114071	0	0	0.211217	0.107583	0.354638	0	0.08829	0	0.280336	0	0	0.187661	0.121642	0	0	0	0	0	0	0.131604	0	0.175199	0	0	0.606189	23.35615
LOC107817325	0.689207	0.241588	0.553028	0.912566	0.609856	1.481964	0.800402	0.905965	1.393664	5.169725	1.747216	1.433259	0.826253	0.636216	0.217656	0.395076	1.280447	1.106718	0.914548	1.031501	0.870326	0.487025	0.514399	0.942008	0.40217	0.073768	3.696975	0	0.510475	0
LOC107828357	0.682831	0.602242	0.708455	0.079821	0.155926	0.189452	0.073899	0.094102	0.227477	0.472536	0.200788	0.297742	0.171644	0.504634	0.316508	0.295459	0.468155	0.541923	0.268215	0.242231	0.180799	0.110371	0.194291	0.161157	0.027849	0.153244	0.052068	0	0.106045	0
LOC107816845	0	0	0	0	0	0	0	0	0	0	0	0	0	0	0	0	0	0	0	0	0	0	0	0	0	0	0	0	0	0
LOC107775506	0	0	0	0	0	0	0	0	0	0	0	0	0	0	0	0	0	0	0	0	0	0	0	0	0	0	0	0	0	0
LOC107804492	1.628688	1.828929	1.567475	1.60725	1.955874	1.563427	1.756351	1.987993	1.283338	2.183742	2.426828	1.302248	2.913875	2.189294	2.268655	2.037282	1.404865	1.669602	0.973901	1.107127	0.689646	1.433025	1.513572	1.367923	0.55156	0.283275	0.687492	0	1.260174	1.79829
LOC107817956	0.924102	0.497666	0.401651	0.665946	0.427437	0.406441	0.440389	0.771633	0.295768	0.430078	0.618527	0.124203	0.561122	0.756112	0.62513	0.532132	0.6087	0.407111	0.532792	0.497462	0.249008	0.473569	0.85217	0.329274	1.009026	0.584464	0.3227	0	0.707788	5.194418
LOC107780178	0	0	0	0	0	0	0	0	0	0	0	0	0	0	0	0	0	0	0	0	0	0	0	0	0	0	0	0	0	0
LOC107831021	18.961	13.87849	16.58541	11.48759	11.95428	14.52459	10.43661	10.32801	12.62788	10.11137	9.473019	15.31796	9.300558	12.0881	8.270946	10.86225	13.79568	14.88722	11.18343	9.473448	12.05089	10.3916	14.21613	15.6067	5.918203	5.111677	9.734507	0	5.134781	3.66371
LOC107821161	10.63589	7.470402	9.940708	6.230524	5.837859	6.577207	4.929904	4.407141	5.443192	4.873748	4.261653	5.202129	3.293841	4.144012	2.626499	6.912796	8.845912	7.094823	3.367605	3.991253	3.683125	3.840096	6.418961	8.316576	2.477079	1.001441	3.875199	0	3.464998	0
LOC107813569	0.618659	0.704789	0.672233	0.747298	1.09486	0.387995	0.864826	0.528599	0.822834	0.40218	0.325353	0.8711	1.320007	1.181121	0.634974	0.461025	0.647483	0.538109	0.3662	0.479731	0.235095	0.559713	0.272849	0.269426	0	0.14347	0.121867	0	0.496407	0
LOC107789507	1.328726	0.509882	0.425538	3.041073	2.908419	5.263051	3.343378	3.46565	7.550453	1.295675	1.422073	2.067845	3.036162	3.433202	0.933104	0.625369	0.135122	0.469242	1.490225	0.709901	3.12522	2.511321	3.639442	9.136734	0	0.194613	0	0	0.336682	0
LOC107803428	20.42592	18.25617	19.85146	35.58013	33.23945	29.50039	25.55109	27.22733	25.76104	23.55093	22.74415	25.47723	33.3757	31.94311	29.87231	22.40749	23.24785	23.69223	28.77735	29.70046	38.53679	20.57235	19.75685	16.72198	20.72615	23.90794	19.94547	50.15422	33.97502	54.20451
LOC107828076	0.145702	0.298776	0.253311	0.263997	0.290085	0.43078	0.39717	0.186737	0.512967	0.213116	0.33204	0.276959	0.243296	0.47686	0.18693	0.244298	0.31671	0.190097	0.110886	0.09244	0.298985	0.06084	0.160648	0.342647	0.046053	0.15205	0	0	0.526095	0
LOC107769181	104.4503	50.838	66.18191	46.40278	37.57523	95.94271	20.7183	21.0892	35.38525	20.99618	16.61602	38.56935	20.22557	35.80446	10.4745	31.60126	58.72592	40.03122	13.39571	10.61143	21.11805	11.50895	19.17997	21.23226	2.077135	3.550002	3.609435	11.05799	6.141527	1.195099
LOC107822431	6.190486	6.875552	6.270959	5.832597	4.601309	3.833585	5.289148	5.458577	5.641418	5.907726	6.111807	4.741771	6.284897	6.03314	5.930162	5.47428	5.103385	5.64909	4.807577	3.770442	3.553107	5.018188	4.746923	5.262506	5.301255	4.547985	5.502125	4.722211	5.563264	5.103561
LOC107815656	10.58666	4.540441	5.533686	0.438803	0.061227	0.297567	0.812502	0.945938	1.364207	0.134946	0.679263	0.292285	0.847303	2.717536	0.213056	6.393856	11.36401	8.460263	0.210639	0.058533	0.441742	0.92457	1.586873	2.025	0.087482	0	0.163563	0	0	0
LOC107791567	0.013101	0.035821	0.068333	0.629072	0.510095	0.211286	1.351621	1.253758	0.578112	0.421595	0.845183	0.166028	0.33545	0.457379	1.734664	0.087869	0.025314	0.117213	0.824258	1.008549	0.418207	2.407107	1.016962	0.424504	0.34785	1.276084	0.340668	0	0.504602	0
LOC107760055	22.79839	26.99412	24.83002	32.63702	29.2766	29.93995	31.89105	29.81499	32.83146	17.41758	16.11836	16.65017	15.77303	16.19758	19.33854	19.4044	21.22424	21.68894	18.4309	21.00725	22.30469	27.94562	28.61891	28.63403	10.19563	11.84998	13.4952	8.084662	9.433878	6.990042
Novel02793	0.424471	0.928448	0.811765	0.692189	0.60096	1.642891	1.281681	1.233111	0.95643	0.662261	0.714335	0.358605	0.472527	1.204001	1.132731	1.328529	0.902165	0.632923	0.947593	1.22085	0.3097	1.843333	0.599059	1.064778	0.107332	0.354373	0	0	0.408711	0
LOC107759565	16.1621	19.19719	17.99391	12.76421	8.303174	21.8031	9.655898	10.21939	10.83459	8.221466	12.29528	5.82137	4.646081	4.492048	5.864853	20.79001	18.78003	24.20347	6.565757	9.78604	4.879307	12.30438	14.52782	10.21755	0.814519	0.545645	1.820843	0	0.539411	1.731936
LOC107769879	0	0	0	0	0	0	0	0	0	0	0	0	0	0	0	0	0	0	0	0	0	0	0	0	0	0	0	0	0	0
Novel03796	5.708175	6.278027	6.290801	6.030282	5.633114	5.492082	5.047364	5.356675	5.268254	4.868059	4.938772	3.825245	4.22192	5.150748	4.19047	0	0	0	0	0	0	0	0	0	0	0	0	1.38378	0	0
LOC107765348	167.4859	157.0208	173.6985	81.76716	68.66527	81.74066	97.28057	98.38928	144.6672	75.30912	64.64773	61.81215	54.65909	69.03609	43.09426	115.8956	136.5038	122.6983	34.73118	36.3777	50.76219	94.16084	124.7547	150.193	82.45874	14.3323	104.5459	23.1228	65.37866	17.13608
LOC107796083	3.06941	3.956071	3.283906	3.488253	2.732087	2.733721	3.777886	3.491611	3.427345	2.125252	4.164456	1.488361	2.951887	2.120104	4.734859	5.386726	4.29882	4.969431	2.635452	2.903789	1.706115	6.249101	4.774036	3.056282	0.780726	0.783412	1.008914	1.298854	0.612075	0
LOC107832417	3.328297	2.933879	3.035508	3.064699	3.138218	4.751548	2.517004	1.818159	2.100243	2.074998	2.448581	1.705541	2.551057	2.26194	1.288028	2.195632	2.846433	3.98649	1.882444	1.569301	1.841183	2.232752	2.550758	2.281138	1.724582	0.303678	1.805669	3.901972	1.838775	0
LOC107797842	21.47751	17.30285	20.52119	12.5612	13.75236	20.31419	10.14719	9.407777	10.84774	11.79717	13.50321	11.98169	14.32526	15.27458	9.99883	16.47779	17.20336	17.63391	18.29952	17.01859	13.76109	11.31433	14.70803	15.81799	15.52482	8.070047	18.95819	9.721177	17.66969	30.81827
LOC107818628	1.919327	1.932288	1.707231	2.406031	2.42903	2.135494	1.778292	1.754338	1.864807	2.872667	2.316085	2.111721	2.63351	2.532147	1.878319	2.045639	1.358332	2.12977	2.264651	2.548703	1.567271	1.770623	2.047316	2.355977	1.58012	1.05582	1.028734	1.596035	2.256358	4.13982
LOC107762121	0	0.105731	0	0.105102	0.102656	0.062364	0.437872	0.346936	0.816884	0	0.040674	0	0.064574	0.063282	0.178608	0	0	0	0.117722	0	0	0.048443	0.102331	0.181885	0	0	0	0	0	0
LOC107779494	10.02098	11.91474	12.14036	11.37421	11.56815	12.84804	9.830015	9.078879	11.27354	4.913939	7.470933	5.546689	5.770082	5.654686	5.822401	11.52335	10.37659	12.40884	7.012824	7.770636	7.878707	12.26459	11.91255	14.20601	2.075187	2.363977	4.458513	0	1.386338	0
LOC107767308	0	0.330746	0	0.054796	0	0.065028	0.202925	0.10336	0.170357	0	0	0.051099	0	0.197958	0.18624	0.360586	0.233733	0.270564	0.245503	0.153498	0.165489	0.808198	0.320109	0.316093	0.076471	0	0.071488	0	0	0
LOC107791982	5.730607	7.330164	5.156285	6.603402	5.248742	4.323581	8.64328	7.34458	3.728347	4.460649	7.296397	2.887838	5.036362	4.93564	10.06087	16.18278	10.92681	13.94155	9.53868	10.63092	5.593176	16.91815	12.41524	9.142066	6.164801	14.12888	4.337171	0	5.808249	3.108176
LOC107798283	0.780493	0.908913	0.829236	0.864218	1.22779	0.792507	0.872844	1.444895	1.058433	0.718797	1.459419	0.62275	0.868856	0.756871	1.468641	1.066311	0.963473	0.775854	1.011988	1.393847	0.632731	1.158774	1.109164	0.861092	2.192846	1.025666	0.871231	0	0.417508	5.362118
Novel01263	0	0	0	0	0	0	0	0	0	0	0	0	0	0	0	0	0	0	0	0	0	0	0	0	0	0	0	0	0	0
LOC107765679	0	0	0	0	0	0	0	0	0	0	0	0	0	0	0	0	0	0	0	0	0	0	0	0	0	0	0	0	0	0
LOC107817705	1.683404	3.363472	1.913608	3.519412	4.182301	4.524663	3.258331	2.932013	3.160908	2.71484	3.17801	2.352078	4.10836	4.308737	1.329077	3.377432	2.502015	2.268743	4.336205	4.053048	2.893436	2.108782	2.398642	3.789678	4.092951	1.441436	3.443622	0	2.805406	0
LOC107797688	6.199537	3.459253	4.729233	0.916974	0.78368	2.584489	0.106119	0.108103	0.475135	0	0.088717	0	0.422535	0	0	1.414256	2.689062	1.131923	0.51354	0.856226	0.230779	0.105661	0.2232	0	0	0	0	0	0	0
LOC107777723	40.92899	64.68473	51.04644	35.64299	24.71064	48.59786	20.62255	19.41081	19.25998	87.45573	127.263	45.8454	37.34954	32.34139	58.31834	119.1528	49.79696	64.96111	37.21273	91.01981	16.31955	19.66933	21.84971	17.09763	49.02429	41.06325	68.53751	16.71208	16.79558	12.38516
LOC107789037	1.473025	0.750879	1.020035	79.50369	64.7959	22.92312	7.18259	4.138422	3.187795	2.045023	2.065772	0.949158	0.166758	0.272372	0.333124	0.632573	0.795952	0.912066	24.70094	25.78747	37.57031	8.757082	12.28834	6.05404	3.756267	1.841172	3.777078	1.785459	0	7.718588
LOC107812672	4.082487	2.325427	2.629928	3.797594	1.773972	3.644581	1.589787	1.339215	3.901261	0.319897	0.460067	0.708276	1.501368	1.033916	0.710829	1.412484	2.852375	2.146872	1.590472	0.95588	3.025178	1.187199	2.057728	4.305112	0.046085	0	0.043082	0	0	0
LOC107787573	0	0	0.128261	0	0	0.158633	0	0.12607	0	0.071939	0	0	0	0.321937	0.07572	0.109953	0	0.165006	0	0	0	0	0	0	0	0	0	0	0	0
LOC107786595	0.222184	0.050623	0	0.150965	0.049151	0.119437	0.093177	0.2373	0	0.108328	0.038949	0.093853	0.680179	0	0.057011	0	0.107324	0.165647	0.056364	0	0.151976	0.092776	0.146985	0	0	0.077288	0.065651	0	0	0
LOC107814626	9.093901	6.927634	8.890273	2.128517	1.149555	2.793444	0.857798	1.062776	1.738692	0.242581	0.348874	0.163463	0.553867	0.78403	0.05674	5.170131	4.779938	4.760347	0.981687	0.654708	0.680645	0.92335	2.876984	2.022328	0	0.230763	0.228686	0	0	0
LOC107810228	0.415413	0.791613	0.590906	0.102639	0.10025	0.081204	0.1267	0.225871	0.07091	0.110476	0.158884	0	0.126121	0	0.038761	0.450278	0.766163	0.422329	1.149633	0.479196	0.826611	0.283845	0.399732	0.394717	1.2414	1.839143	0.223175	0	4.727142	2.335054
LOC107811212	12.35826	11.04379	11.55305	11.44112	11.25468	13.51293	11.19766	9.994015	13.43742	12.28543	13.21981	15.85142	14.29295	15.68009	9.412831	9.747161	12.05531	9.79646	8.66532	7.249287	9.844931	9.893812	11.35168	13.79683	6.04455	4.225686	10.94596	8.601363	7.961892	5.577588
LOC107832125	0	0	0	0	0	0	0	0	0	0	0	0	0	0	0	0	0	0	0	0	0	0	0	0	0	0	0	0	0	0
LOC107819940	2.325942	2.833409	2.352201	3.494591	3.841195	3.330103	3.273982	2.528458	1.859386	2.885636	4.682932	1.322984	3.576271	2.663107	3.368197	3.346461	1.957831	3.013194	4.264728	5.240406	1.470207	3.259863	2.600092	1.781183	3.173625	4.662308	2.041394	0	3.104372	4.983741
LOC107765078	3.132345	3.891726	3.65378	4.098966	4.181247	4.677296	4.614498	3.271107	2.978485	1.997128	3.022399	2.284406	4.61949	3.636287	4.547647	4.229539	3.207157	3.552843	3.259986	2.71769	2.698043	4.672852	3.270654	3.665677	1.066217	1.415558	0.949277	2.871884	2.706706	4.13841
LOC107772301	0	0	0	0	0	0	0	0	0	0	0	0	0	0	0	0	0	0	0	0	0	0	0	0	0	0	0	0	0	0
Novel02941	1.336645	1.37046	0.508339	0.681149	0.887063	0.89816	1.19117	0.713791	0.705882	0.651699	1.230149	0.141154	0.836984	0.729107	1.629126	1.494104	2.098381	2.179899	0.762938	1.06004	0.15238	0.906967	1.621137	0.523898	0.316861	0.464961	0.296214	0	0.804385	0
LOC107805317	0	0	0	0	0	0	0	0	0	0	0	0	0	0	0	0	0	0	0	0	0	0	0	0	0	0	0	0	0	0
LOC107807730	4.862484	4.357696	5.870631	4.870154	3.991899	4.646902	3.353331	2.885166	3.474559	1.896614	2.954967	2.487599	2.977142	3.006014	2.71717	3.744313	3.575372	4.088407	1.973645	2.467996	2.537615	2.233433	2.525764	3.359943	1.058763	1.202807	1.117485	1.931869	1.820757	0
LOC107823009	0	0	0.015514	0	0	0	0.014969	0	0	0	0.037542	0	0	0	0	0.054952	0.132993	0	0.039916	0	0	0.014904	0.031484	0	0	0	0.021093	0	0	0
LOC107766022	22.69576	31.37716	24.54971	10.86143	9.332216	14.19929	5.296715	3.451096	2.196989	2.625739	3.956092	1.029113	5.638287	4.686207	5.132644	21.66668	18.86009	22.29798	6.115334	5.640399	2.104974	4.87231	4.524104	2.579892	0.081057	0.178415	0.568317	4.584937	0.30866	0
LOC107797747	5.782182	1.446597	2.020387	2.670538	3.711938	3.839635	0.42792	0.484358	0.532212	0.442224	0.874493	0.478916	0.126212	0.927658	0.174549	0.549172	1.040543	0.76074	0.460185	0.91113	1.395912	0.899492	0.550029	1.066506	0.071671	0	0.268003	0	0	0
LOC107778568	0	0	0	0	0	0	0	0	0	0	0	0	0	0	0	0	0	0	0	0	0	0	0	0	0	0	0	0	0	0
Novel04337	14.78825	13.68343	16.19156	14.82919	15.58287	19.47953	14.29721	15.77024	16.2155	16.23672	13.93158	18.54955	11.05877	16.74903	17.09002	12.91301	16.74052	14.43384	13.23057	13.56029	18.12012	13.29281	15.93187	11.681	16.62735	19.00603	17.3451	24.21914	13.31527	10.47
LOC107811574	4.161009	4.287965	6.92682	20.44981	20.16641	12.80676	4.151524	3.834125	6.868359	1.246424	2.212114	2.228677	1.967883	1.809849	1.926023	6.282644	3.91479	7.846764	7.699496	6.142618	10.88866	4.451592	8.012257	12.67773	0.481379	0.113524	0.642873	1.944908	1.440251	0
LOC107811686	0	0	0	0	0	0	0	0	0	0	0	0	0	0	0	0	0	0	0	0	0	0	0	0	0	0	0	0	0	0
LOC107814557	0	0	0	0	0	0	0	0	0	0	0	0	0	0	0	0	0	0	0	0	0	0	0	0	0	0	0	0	0	0
LOC107821616	0	0	0	0	0	0	0	0	0	0	0	0	0	0	0	0	0	0	0	0	0	0	0	0	0	0	0	0	0	0
LOC107771767	46.91905	42.62996	49.77983	47.32322	46.03116	60.87235	31.15699	34.44085	37.0341	33.28055	26.09805	37.99956	23.11599	34.41167	22.12393	29.07431	50.46442	36.96217	42.57942	36.77277	54.45526	29.22248	35.30143	38.30059	17.4417	18.09568	23.09898	102.768	29.4031	4.442688
LOC107792757	0.11531	0.026273	0.100237	0.026116	0.025509	0.061986	0.024179	0.024631	0.054129	0	0.060642	0.048709	0	0.03145	0.029588	0.064447	0.250649	0.107461	0	0.170703	0.052583	0.120373	0.050856	0.030131	0	0	0	0	0	0
LOC107821213	0	0	0	0	0	0	0	0	0	0	0	0	0	0	0	0	0	0	0	0	0	0	0	0	0	0	0	0	0	0
LOC107817444	0	0	0	0	0	0	0	0	0	0	0	0	0	0	0	0	0	0	0	0	0	0	0	0	0	0	0	0	0	0
LOC107807226	447.2036	805.7698	490.9508	38.13712	23.309	23.2012	59.90886	53.84413	89.07877	27.71193	65.28761	37.40439	212.7126	150.3754	91.61233	985.274	905.1816	836.8089	17.83706	14.56991	22.823	37.48252	116.6231	87.46863	0.128093	2.678471	6.22679	3.622721	4.877656	12.5289
LOC107809592	0.071459	0	0	0	0	0	0	0	0	0	0.150322	0	0	0	0	0	0	0	0	0	0	0.179033	0.126064	0	0	0	0.084459	0	0	0
LOC107816365	1.105317	0.923414	1.094277	0.584128	0.108673	0.231068	0.231769	0.367272	0.317081	0.119758	0.25835	0.025939	0.170896	0.167479	0.126052	0.823684	0.533915	0.846952	0.155778	0.233756	0.252017	0.153847	0.35207	0.641821	0.038818	0	0.072578	0	0	1.898429

LOC107813653	0	0	0	0	0	0	0	0	0	0	0	0	0	0	0	0	0	0	0	0	0	0	0	0	0	0	0	0	0	0
LOC107786051	6.043819	4.890454	5.351964	2.59271	2.96554	4.635534	2.384582	2.252213	2.492407	2.056294	3.247759	1.892873	2.179809	1.787026	1.816516	4.644168	4.492812	4.856852	2.158916	2.389091	1.030292	3.805168	3.039195	3.660316	0.476089	0.576354	0.956892	2.69295	0.634516	0
LOC107806463	0	0	0	0	0	0	0	0	0	0	0	0	0	0	0	0	0	0	0	0	0.133813	0	0	0	0	0	0	0	0	0
Novel03393	0.329817	0.178126	0.265465	2.09158	3.458885	1.904297	1.495859	1.659514	0.906002	4.049939	2.175629	1.393191	2.379718	2.638634	1.19107	0.591692	0.141614	0.47357	5.528337	5.001372	2.495512	1.193567	1.077482	0.714984	5.57529	3.382379	3.176284	2.620728	6.998322	8.308282
LOC107783914	0	0	0	0	0	0	0	0	0	0	0	0	0	0	0	0	0	0	0	0	0	0	0	0	0	0	0	0	0	0
LOC107763557	0	0	0	0	0	0	0	0	0	0	0	0	0	0	0	0	0	0	0	0	0	0	0	0	0	0	0	0	0	0
LOC107779857	0	0	0	0	0	0	0	0	0	0	0	0	0	0	0	0	0	0	0	0	0	0	0	0	0	0	0	0	0	0
LOC107824753	0	0	0	0	0	0	0	0	0	0	0	0	0	0	0	0	0	0	0	0	0	0	0	0	0	0	0	0	0	0
LOC107764259	0	0	0	0	0	0	0	0	0	0	0	0	0	0	0	0	0	0	0	0	0	0	0	0	0	0	0	0	0	0
LOC107812096	2.191938	2.84727	2.255825	2.784664	2.27398	3.873494	2.620352	2.088122	2.270773	1.940872	2.225983	0.936548	1.991343	1.539225	2.120449	3.079119	2.434019	3.399852	2.965636	2.813313	1.194855	4.25023	3.000157	2.054012	1.815664	1.297093	1.191124	0	1.698147	6.231303
LOC107782737	2.09519	2.581372	3.305331	0.913918	0.686653	1.752011	0.065086	0.099455	0.145708	0.075669	0.108826	0	0	0.33863	0.039823	1.619166	1.574323	1.243857	0.393714	0.590796	0.389247	0.162014	0.273792	0.121661	0	0	0	0	0	0
LOC107787180	0	0	0	0	0	0	0	0	0	0	0	0	0	0	0	0	0	0	0	0	0	0	0	0	0	0	0	0	0	0
LOC107763289	0.147826	0.58381	0.257004	0.491051	0.348817	0.211908	0.247976	0.926249	0.416357	4.084229	4.353546	0.832585	0.548541	6.343332	7.232215	0.954722	0.095208	0.220421	0.050001	1.167141	0.13482	0.082302	0.173857	0	0.68529	0.959884	0.05824	0	0	0
LOC107774360	1.719095	1.566743	1.829844	0.635679	0.372531	1.244731	0.411965	0.179858	0.527008	0.136844	0.270607	0.059279	0.195278	0.459293	0.108026	0.836618	1.118491	1.020095	0.391604	0.474853	0.127987	0.35159	0.495136	0.513371	0	0	0.041466	0	0	0
LOC107813999	3.99265	2.945702	2.768317	1.507141	1.135594	1.533067	0.358801	0.203061	0.535496	0.278096	0.433279	0.240936	0.052913	0.051855	0.19514	2.089813	3.030685	3.047563	0.868171	0.965002	0.866991	0.357254	1.635113	2.434318	0	0	0.056178	0	0.228834	0
LOC107792991	3.93801	5.055487	3.68089	4.680126	4.646147	4.91672	4.332939	4.739583	4.412759	4.665875	3.978697	5.366032	4.383847	4.619543	4.128787	4.354582	3.804448	3.725188	3.910084	4.226792	4.711471	5.056872	4.743498	5.355234	2.034365	2.06216	3.30313	0	1.42702	5.23641
LOC107759175	0.123829	0.564275	0.215284	7.179712	2.191445	3.860815	0.727026	1.798652	0.581282	7.607195	3.646823	3.452274	1.102787	5.403664	12.5824	0.092278	0	0.09232	15.20405	18.54093	24.05501	1.240956	1.529158	0.776558	5.322965	5.858177	3.805261	0	1.788474	22.96964
LOC107801155	11.74139	13.08094	11.55388	13.17616	11.04712	12.30385	15.8138	17.14357	17.20278	17.3914	15.2751	14.70877	16.04881	13.24938	15.38523	16.12286	10.56371	14.10407	13.03469	16.66777	12.27684	16.06477	17.56703	15.86718	11.99923	15.06694	11.81961	9.110402	15.4351	15.7538
LOC107823865	0.765786	0.418752	0.372782	0.138753	0.487885	0.197595	0.770757	0.44493	0.488888	1.822041	3.17886	4.709849	2.659749	3.107818	1.446206	1.164156	1.834738	2.101008	0.279744	0.285033	0.530791	0.537203	0.810569	0.672335	0.15491	0.468836	0.362039	0	0.147471	3.787976
LOC107760545	0.073948	0.157254	0.085708	0.066993	0.021811	0.079503	0.124047	0.168488	0.115709	0.048072	0.103705	0.104122	0.05488	0.026891	0.025299	0.27553	0.119066	0.147017	0.025012	0.104258	0.089922	0	0.043485	0.025763	0.37397	0.274382	0	0	0	1.524103
Novel02550	1.062778	1.522071	1.385775	1.513007	1.813659	1.469078	1.591781	1.426961	1.211593	2.14671	1.916281	3.976256	3.971832	2.650145	2.025783	0	0	0	0	0	0	0	0	0	0	0	0	0	0	0
LOC107832409	12.8319	15.94731	13.14282	23.76815	26.41289	26.55887	31.23283	28.61173	26.60681	32.27434	36.16737	27.24679	36.76833	37.55517	48.43199	18.86901	15.75551	16.88245	26.04185	28.59812	20.42272	29.41717	26.2688	22.23362	31.08766	30.01236	24.11464	17.99338	35.67888	32.52862
LOC107789239	0.140504	0.256104	0.122137	0.738277	1.367596	0.604234	0.518524	0.672281	1.134435	1.39749	1.280776	1.828001	1.313849	1.93136	0.605678	0.481636	0.651544	0.796111	1.824939	2.139416	2.332179	0.985641	1.561562	2.085332	3.552787	1.759505	2.358109	0	2.705738	3.475021
LOC107804635	0.087886	0.24029	0.152794	0.07962	0.077767	0.094488	0.368567	0.225275	0.082511	0.599896	0.677879	0.519737	0.489177	0	0.27061	0.130985	0.254714	0.589701	0.26754	0.817796	0.240459	0.220186	0	0.551147	0	0.122286	0.207747	0	0	0
LOC107780645	9.712804	7.903592	6.064314	10.51028	9.788212	6.713005	15.09697	14.19372	14.7276	11.65109	10.13486	12.5364	8.752918	9.292691	11.07662	8.329436	11.58029	9.798753	18.57756	15.22636	21.75893	16.48034	15.87677	11.23951	20.8564	24.40146	21.27399	19.29452	12.61802	30.98113
LOC107759124	26.8083	23.30524	22.65879	37.0047	41.80718	49.89864	30.62873	32.46961	28.85559	66.69543	53.83555	64.288	66.22681	53.09578	48.8103	27.74279	33.1281	33.28815	50.54713	53.41225	40.21356	31.73614	35.07456	34.90384	60.28177	56.23234	74.7728	95.08467	66.542	36.32566
LOC107816870	0	0	0	0	0	0	0	0	0	0	0	0	0	0	0	0.063676	0	0.04247	0.115609	0.048189	0.051953	0.14272	0.075371	0	0	0	0	0	0	0
LOC107784862	13.16052	17.13456	13.03647	15.48772	14.19875	18.2388	12.57853	10.19869	9.769607	15.00756	15.94235	14.44281	14.35844	12.87881	13.46273	16.25851	14.10809	15.54882	13.84243	18.30825	14.59455	10.55253	11.33849	10.87634	8.40237	7.117918	13.0742	6.253579	7.788371	5.406877
LOC107773922	1.045694	1.126298	0.578454	1.636324	1.345889	3.168338	0.478401	1.380816	1.338741	0.64889	0.999874	0.40156	0.317477	0.725964	0.682991	0.920935	1.102067	0.637862	0.771708	0.804168	1.127089	0.635117	0.670815	1.192319	0	0	0.224714	0	0	0
LOC107797021	304.6192	324.2714	390.4898	32.29719	31.54556	62.9525	23.88761	36.92687	36.64859	20.87743	15.08252	34.82942	50.43916	45.84536	10.22075	226.9284	310.6424	263.3367	46.68428	42.03522	34.87481	22.03818	59.37801	32.9941	10.44979	8.175046	51.78672	7.121471	13.90317	6.157261
LOC107827245	43.66152	39.85617	47.5495	20.38267	17.23314	30.76518	17.63224	19.52386	23.82911	12.95776	16.0226	14.84962	16.98422	18.79224	11.25737	28.55468	45.84859	40.09969	12.48522	14.57164	13.33747	16.26444	26.48111	29.68848	3.911213	1.663093	7.977492	5.028045	3.046407	8.694549
LOC107778347	2.035328	1.964065	2.914087	2.603159	3.07228	3.475418	2.510466	2.199373	2.304267	3.852656	2.602492	5.66424	6.397427	7.249092	3.563596	1.828996	1.850633	1.874464	2.247543	1.823027	2.238423	2.099698	1.267266	3.003281	2.421904	1.4993	3.679143	0	8.357794	0
LOC107773478	0	0	0.171821	0	0	0	0.082893	0	0	0.289114	0	0	0	0.107818	0.101436	0.36824	0	0.221045	0	0	0	0	0	0	0	0	0	0	0	0
LOC107803820	0	0	0	0	0	0	0	0	0	0	0	0	0	0	0	0	0	0	0	0	0	0	0	0	0	0	0	0	0	0
LOC107777593	0.097845	0.163485	0.127582	0.029548	0.07215	0.052598	0	0.041801	0.01531	0	0.011435	0	0	0.017791	0.016738	0.145829	0.157544	0.12158	0	0.02759	0.014873	0.12257	0.014384	0	0	0	0	0	0	0
LOC107780883	25.67501	15.64145	12.05451	0.932898	0.303729	0.221421	1.439488	2.463561	3.544828	0.200827	0.914608	0.521975	3.362562	7.189777	2.959329	23.83957	35.21668	31.06705	0.417966	0.232292	0.375658	2.006592	1.99827	1.291549	0.086794	0.191042	0.324554	0	0.661009	0
LOC107788072	1.51524	2.139125	3.021733	0.565242	0.157739	0.734678	0.099678	0.203084	0.139468	0.202801	0.124998	0.075301	0.23152	0.194477	0.060988	1.594106	1.86569	2.104298	0.572817	0.351864	0.270966	0.198497	0.576547	0.155267	0	0.04134	0.175578	0	0	0
LOC107827608	47.67147	35.28179	35.86901	38.27079	33.67684	41.05837	39.32594	38.6086	47.33461	36.47438	31.40971	44.85958	30.93845	42.59028	33.2903	30.06697	44.47527	32.71342	30.05939	27.4377	41.50908	36.0435	46.72154	47.16061	27.5313	25.02212	39.80395	56.11826	50.05717	28.30342
LOC107813486	5.22805	4.794879	5.206193	6.954638	7.319814	6.225561	6.910523	7.661732	7.517811	5.259338	5.545271	4.836118	4.641216	5.198168	6.248927	4.734317	4.667135	4.687147	8.797004	10.38464	7.574591	8.40056	7.238281	5.671863	4.392618	4.696164	5.27964	9.465303	2.389528	2.045938
LOC107832625	0	0	0	0	0	0	0	0	0	0	0	0	0	0	0	0	0	0	0	0	0	0	0	0	0	0	0	0	0	0
LOC107775188	0	0	0	0	0	0	0	0	0	0	0	0	0	0	0	0	0	0	0	0	0	0	0	0	0	0	0	0	0	0
LOC107803124	2.485029	2.300185	2.936499	1.336716	1.339965	0.208727	0.58621	0.763053	0.947804	0.037863	0.462853	0	0.043225	0.25416	0.318821	1.793977	1.275397	2.055333	0.551608	0.229924	0.212474	1.459204	1.883716	2.597352	0	0	0.045892	0	0	0
Novel04625	1.022904	1.125131	0.950513	0.894744	0.84271	1.403126	1.449646	0.964411	1.688887	0.653509	0.964593	1.013174	1.374307	0.923535	0.289622	1.366828	1.056364	1.367454	1.145349	1.193527	0.77206	1.089911	0.933382	1.732743	0.668929	0.441713	1.292369	2.522487	1.188703	0
LOC107803398	3.974455	4.21692	3.919002	3.976843	3.385633	4.400578	4.229127	2.559583	3.814932	4.135897	3.722787	3.057038	3.797076	4.109431	2.922464	3.227002	3.868312	4.090882	2.799024	2.910488	2.705052	3.145782	4.447571	4.371105	3.562443	1.980954	3.505587	6.363353	4.426625	1.83393
LOC107775221	7.954589	8.919238	8.130076	10.87519	10.51546	7.360037	12.25195	10.97757	9.436986	8.062297	9.701926	6.984995	9.713841	8.783254	13.92891	9.753874	8.756016	9.362975	8.658805	8.686572	8.727647	13.42708	11.52403	11.03512	4.236138	6.271997	3.418794	5.171505	5.57036	10.43306
LOC107823651	1.547296	1.086524	2.337267	0.344698	0.336676	0.681775	0.382953	0.563497	0.762056	0.445222	0.249008	0.364301	0.592982	0.719485	0.182241	1.323168	1.17625	1.475063	0.154435	0.493522	0.393274	0.317751	1.096328	0.689312	0	0.035294	0.26982	0	0.732711	1.568387
LOC107832305	0	0	0	0	0	0	0.070069	0	0	0	0	0	0	0	0	0	0.062283	0	0	0	0	0	0	0	0	0	0	0	0.402193	0
Novel00079	0.041018	0.074765	0.017828	1.858004	1.506254	0.639438	3.371535	2.278038	1.232299	0.099994	0.158189	0.207918	0	0	0	0	0.039627	0.030581	1.477583	2.099245	1.42154	1.627115	3.36478	0.535898	0	0	0	0	0	0
Novel00583	17.33397	16.83087	17.40832	10.31224	7.773602	11.12075	8.992631	7.829044	12.10565	3.638479	3.974253	3.35181	2.944412	3.555382	2.520811	13.55084	14.00829	15.81068	4.648947	3.276279	4.867582	8.914407	13.91488	17.13013	0.23886	0.131438	1.507244	0	0.227389	8.761189
LOC107827408	0.034631	0.078905	0.015052	0.078435	0.061288	0	0	0.014795	0	0	0.012142	0.029257	0	0.01889	0.071089	0.219362	0.033457	0.038729	0.193278	0.058591	0.063169	0.043382	0.045821	0	0.043784	0.120467	0	0	0	0
LOC107768398	3.142786	2.471128	4.606829	0.949068	1.090566	1.921323	0.930349	0.737136	1.388511	0.781176	0.993836	1.197404	0.754598	0.537823	0.252994	1.515417	4.22686	2.572794	0.812903	0.625548	1.011626	1.28658	1.902452	2.254301	0.233732	0.342977	0.364169	4.406934	0	0
LOC107800229	1.464467	1.55408	1.621805	7.396989	9.727799	6.276355	5.115159	5.022261	3.522019	8.607364	9.607705	5.643767	11.72451	9.717282	9.059712	2.287292	1.802414	2.348165	4.112055	5.074145	1.957687	4.339188	2.441951	2.998417	5.351686	2.372663	5.145224	0	6.374392	0
LOC107780584	18.73062	14.46496	19.4366	9.762983	6.908572	9.970333	9.592125	9.740174	14.6256	3.181135	7.40229	6.595976	4.243686	7.517862	5.868957	13.65761	17.35171	15.22155	4.203012	3.565859	5.616211	12.45884	16.32765	17.81491	0.463429	0.102005	2.036184	0	0	0
LOC107799709	2.664521	2.336165	2.286892	2.658369	1.731002	2.393282	1.612486	1.902002	1.836595	1.216899	1.750113	1.823645	2.402983	2.244539	2.042439	2.387752	2.052804	2.087096	0.616049	0.85595	0.861297	1.352027	2.023029	1.833136	0.042643	0.140791	0.438503	0	0.811896	0
LOC107818235	0	0.100009	0	0.033138	0	0	0	0	0	0	0.051297	0	0	0.119715	0	0	0	0.02727	0	0	0	0.061095	0	0.076463	0	0	0	0	0	0
LOC107774333	0.292624	0.222243	0.127186	5.169506	6.775421	14.57679	0.654498	1.083447	1.465219	7.704334	4.206362	3.831867	3.583289	6.384776	2.152448	0.508816	0.471166	0.436328	9.056517	9.654063	4.181094	0.855324	0.86038	1.478279	4.686238	1.967966	8.127674	0	3.287195	0
LOC107766056	0.426719	0.583353	0.556407	0.193293	0.566384	0.573469	0.089477	0.5469	0.400623	0.208052	0.149608	0.540756	0.593787	0.465529	0	0.317992	0.515308	0.477206	0.108251	0.631706	0.486469	0.267273	0.376394	0.557508	0.269751	0	0.126087	0	0.513594	0
LOC107759737	0	0	0	0	0.126914	0	0.096239	0.02451	0.053862	0	0	0	0	0	0	0	0	0	0	0.024266	0.078485	0.023956	0	0	0	0	0	0	0	1.773669
LOC107801676	0.058821	0.053608	0	0	0	0.03162	0	0.025129	0	0	0	0.024847	0	0	0	0.06575	0	0.021927	0.029844	0	0.026823	0	0	0	0	0	0	0	0	0
LOC107798973	3.613863	4.14605	3.788225	3.177684	2.633469	3.382536	4.065244	2.942476	2.714283	2.21803	3.08557	1.939612	2.981755	2.829358	2.989155	4.055424	3.368074	3.882945	2.459113	2.769355	1.725503	3.976701	2.550133	3.066205	1.666349	1.715581	1.758769	1.520249	1.94453	2.628831
LOC107832129	4.43354	4.790432	4.132105	3.6853	3.437757	5.35628	13.22595	8.70881	6.865819	2.98617	4.326682	3.243593	10.93946	5.634593	6.942976	4.155394	5.431239	4.020993	2.133475	2.126555	2.251	8.779278	5.241111	3.774029	0.635657	0.635974	0.756301	3.268668	0.220048	0
LOC107769324	0	0	0	0	0	0	0.099848	0	0	0	0	0.100572	0.132522	0	0.122184	0.088712	0	0.177506	0	0	0.099418	0	0	0	0	0.828212	0	0	0	0
LOC107774246	0.2911	0.215557	0.37957	0.362617	0.434673	0.332528	0.457796	0.310904	0.307459	0.124188	0.255149	0.046112	0.202535	0.119091	0.205409	0.284718	0.246073	0.311977	0.240003	0.277032	0.215709	0.303881	0.33701	0.285241	0.115012	0.025315	0.086014	0	0	0
Novel01921	1.533862	1.635871	1.844	2.040053	1.848187	2.175395	1.53287	1.477881	1.869011	1.400239	1.556108	1.599133	1.816507	1.352936	1.27285	1.848313	2.648395	2.408774	1.19218	1.711651	1.875132	0.953913	1.899912	2.046628	0.41261	0.454097	0.81002	2.33389	2.199656	0
LOC107809339	14.30487	19.98701	14.88775	11.22832	8.754647	11.73142	12.76204	9.857323	8.886296	9.264542	11.62095	10.16903	8.707715	9.468756	9.238193	17.91307	17.54637	17.90796	8.698484	10.39381	6.743023	11.51386	11.54667	9.818304	2.68689	3.752225	4.010449	2.554309	4.556858	1.104234
LOC107785302	1.906226	2.00166	1.657041	1.088725	1.613417	1.871215	1.981163	1.168437	1.206067	0.969814	1.307589	1.15531	1.061018	1.130216	0.935716	1.266117	2.081779	1.668332	1.513805	1.226929	0.793667	1.418902	1.791075	1.299382	0.366747	0.05766	0.538762	0	0.598516	0
LOC107767074	34.9901	27.47079	31.37811	40.38791	37.72273	47.36323	28.19855	30.21167	38.83651	33.76007	29.92984	41.66978	41.41527	42.94404	29.53077	23.01168	28.20342	29.11171	29.40971	30.31245	40.32199	22.92804	30.39877	35.80056	27.38233	19.94373	44.22047	33.91654	38.05455	42.3575
LOC107809678	0	0	0	0	0	0	0	0	0	0	0	0	0	0	0	0	0	0	0	0	0	0	0	0	0	0	0	0	0	0
LOC107799872	6.654806	6.534586	8.061512	6.495672	6.800395	7.800964	4.393316	3.77847	5.038568	3.935432	2.95034	4.171284	5.114063	3.372418	3.084656	5.151137	4.52111	6.081765	4.835903	5.302633	5.5211	3.836538	5.377658	6.013229	3.528325	2.150635	5.581929	12.28162	3.513911	0
LOC107805445	0.148975	0.678864	0.388504	0.701814	0.632752	0.448467	1.924263	1.832957	0.727296	1.074997	1.713171	0.981697	0.961881	1.170179	2.385307	0.244237	0.287846	0.222135	1.088424	1.335843	1.086945	2.015495	1.787124	0.747404	1.958842	3.026411	1.197323	0	1.147553	1.842275
LOC107761813	0	0	0	0.040215	0.078558	0	0.074463	0	0.1667	0.129856	0.031126	0.075003	0	0.193708	0.04556	0	0.042884	0	0	0	0.040484	0.185355	0.156618	0	0	0.20986	0	0	0	0
LOC107760896	2.610829	1.487156	1.702154	2.513109	3.320961	4.561311	2.463542	2.509609	3.82994	1.432062	1.487456	3.170695	1.998158	1.780179	0.837401	1.702394	0.63057	0.729932	1.490225	2.484655	1.33938	1.090186	2.302924	3.922705	0.618915	0.227049	0	0	0	0
LOC107814190	13.64277	15.10727	13.08832	16.55426	17.82656	13.03369	14.88154	13.95187	13.87027	15.99168	13.15989	16.93037	14.04631	15.61621	15.0183	9.613504	13.21421	11.67324	22.12847	17.34117	20.08197	13.69574	13.87319	16.43897	15.95273	21.68774	14.28661	17.69315	22.29076	15.2976
LOC107821300	0	0	0	0	0	0	0	0	0	0	0	0	0	0	0	0	0	0	0	0	0	0	0	0	0	0	0	0	0	0
LOC107812433	0	0	0	0	0	0	0	0	0	0	0	0	0	0	0	0	0	0	0	0	0	0	0	0	0	0	0	0	0	0
LOC107771725	1.027054	0.267438	0.637712	0.664614	0.649146	0.736139	0.369186	0.710392	0.964244	0.715362	0.480114	1.115594	0.762221	1.013755	0.70276	0.43735	0.425237	0.546937	1.240695	0.661956	0.802878	0.32675	0.474535	0.664533	1.051174	1.224918	0.809264	0	2.590031	0
Novel01980	0.079969	0.072882	0	0.036224	0	0	0.033537	0	0	0	0	0.03378	0	0	0	0	0	0	0	0.033824	0	0	0	0	0	0	0	0	0	0
Novel04303	0	0	0	0	0	0	0	0	0	0	0	0	0	0	0	0	0	0	0	0	0	0	0	0	0	0	0	0	0	0
LOC107793436	0.177659	0.431771	1.338438	0.26825	0.707419	0.191005	1.018236	1.948056	2.112724	0.692959	1.224983	1.100669	1.351444	1.80896	5.531108	4.280662	0.371872	2.273763	2.463771	1.828494	2.808483	3.140447	0.861888	1.361719	7.524597	11.45357	2.694724	2.11752	8.838236	32.95475
LOC107793902	3.810503	3.232379	3.15951	4.992301	5.057676	3.151351	6.465821	7.763824	8.310734	3.286985	3.658485	3.565904	6.754418	4.892553	6.558439	2.817748	3.539679	3.059422	5.324059	5.231864	6.068309	7.833232	6.87736	6.341742	1.889995	6.07695	2.459717	4.192382	3.386791	1.812378
LOC107800625	2.351211	5.912649	9.386607	8.007516	19.07131	9.970923	12.38017	18.7873	15.65634	18.51165	14.8991	17.87732	13.81406	14.77282	15.41779	8.5335	3.533394	5.713246	29.46963	22.54158	48.24774	21.85367	16.0153	17.93054	48.49817	57.00959	56.81394	99.64139	62.88649	34.99863
LOC107810738	1.758404	2.281304	1.798287	2.28646	1.885448	2.246358	2.37711	2.739722	2.932717	1.008627	2.161366	1.503026	1.519926	1.286411	2.739016	2.69781	1.778708	1.742812	1.406454	1.504989	0.94335	3.368887	3.065563	3.113591	0.130774	0.230277	0.709064	0	0.597571	2.55823
LOC107770361	10.50396	10.95159	11.78491	11.72378	10.01338	12.97947	10.07878	10.02792	10.62421	9.587158	9.486616	9.465642	8.450255	10.42033	7.791055	9.22609	9.498258	10.33712	8.015339	8.174779	8.787862	8.678564	10.17936	9.83695	6.091186	4.521061	7.548222	6.009455	9.304846	3.463871
LOC107810550	18.74874	19.61368	22.7346	8.624889	7.649531	12.27471	7.373184	7.355228	10.65033	5.015209	5.243306	6.255662	7.471461	6.804722	4.155632	17.39653	21.42533	21.15734	8.476088	6.850103	6.986043	7.615548	12.32279	13.72495	2.7209	2.08091	5.000978	0	2.28292	2.255376
LOC107802468	0.044427	0	0	0	0	0	0	0	0	0	0	0	0	0	0	0	0.033122	0	0	0	0	0	0	0	0	0	0	0	0	0
LOC107761536	5.181654	2.925859	3.476146	7.602751	8.273058	8.840794	6.377394	5.389812	6.186712	7.194701	6.674359	6.851904	8.025446	6.697493	6.012	4.826718	3.645611	4.618975	5.543744	5.955601	7.859137	5.079914	6.259682	7.535114	8.829859	2.429421	8.787036	0	15.18472	3.482494
Novel02189	0.479449	0.497647	0.497814	1.327199	1.803053	0.744562	1.195232	1.467927	1.462914	1.792171	1.382113	2.12652	2.031135	2.121278	1.585633	0.466456	0.360257	0.605678	1.18925	1.825112	0.9717	1.4014	1.104253	1.002239	1.481746	2.872313	1.306488	1.904853	3.077648	3.293892
LOC107822976	6.784538	7.769181	7.835213	6.589275	7.006857	7.189104	7.592779	6.999707	6.626611	9.304038	8.760602	7.713933	8.706216	7.956184	8.448468	9.07721	7.272211	7.346724	7.89632	8.253276	6.044959	7.0212	5.949919	5.251982	8.132765	5.794698	7.348621	5.592973	9.695411	10.88036
LOC107805462	14.23655	20.48233	14.09899	15.55128	15.07618	7.054802	26.77831	22.31723	21.25575	21.31633	26.1182	14.09144	15.09364	12.92188	21.59636	24.00153	17.73276	19.53997	12.43443	12.48678	12.29569	20.7342	16.64189	15.66883	25.40606	12.70774	26.5474	5.488489	32.45327	6.327169
LOC107759751	0.235945	0.322552	0.205102	3.455691	8.246792	1.987078	16.78827	22.94854	10.18971	7.439125	10.1197	5.249097	3.502095	11.75479	8.879469	0.234435	0	0.087953	16.63973	9.813329	27.0058	18.75199	19.04289	7.316082	40.37075	48.86199	26.12062	81.55474	15.14563	46.19795
LOC107825591	0.843863	1.510687	1.152724	0.873711	0.613365	0.583236	0.935286	0.952775	0.594192	0.235106	0.633983	0.203691	0.3355	0.460306	0.247462	1.72933	0.90259	1.5279	0.703384	0.484398	0.439781	0.402704	0.850677	0.976506	0.076207	0.041935	0.106862	0	0.145095	1.863475
LOC107828548	0.082229	0.149883	0.214439	0.14899	0.291046	0	0	0	0.2316	0	0.115318	0.138938	0	0.269123	0.084397	0	0.07944	0	0.16688	0.06956	0.299976	0	0.145063	0	0.103962	0	0	0	0	0
LOC107800841	5.09844	5.869389	5.55162	6.417879	5.152534	5.250649	6.504449	6.007033	5.466847	3.715634	5.362544	3.899243	5.167833	4.830286	5.838814	6.338938	5.625464	6.221806	4.492815	4.85772	3.891201	7.193509	6.745742	5.861777	3.155137	4.144452	2.53724	5.757009	2.712946	6.63672
NitaMp157	0.169514	0.154491	0	0	0	0	0.142179	0.144837	0	0	0	0	0	0	0	0	0	0	0.172011	0	0.463798	0	0	0	0.642951	0	0	0	1.632199	0
Novel04482	23.10584	17.57818	20.76628	13.83688	14.6411	16.94703	16.66997	16.0615	20.77359	12.02932	13.66171	16.04648	12.86087	14.31264	12.6615	12.91387	19.10628	15.62053	9.438092	8.447826	14.28672	10.54755	17.01285	19.64763	4.951323	3.133271	6.943028	49.01169	7.541677	6.053681
LOC107810113	0	0	0	0	0	0	0	0	0	0	0	0	0	0	0	0	0	0	0	0	0	0	0	0	0	0	0	0	0	0
LOC107832555	2.177953	2.729286	3.833827	3.748918	3.035333	2.6928	3.607817	3.396146	2.555947	2.229968	2.48167	2.529993	2.545754	2.376039	3.74428	4.057532	4.944601	2.963355	3.480807	3.776916	3.227785	4.501691	3.409949	3.983692	2.684754	4.924501	2.638519	0	2.62136	10.09996
Novel04368	2.227959	2.776411	2.411015	1.936033	1.488642	1.808717	2.059367	1.903628	2.049018	0.842414	1.562246	1.075566	1.265405	0.694455	0.373341	1.965226	1.713131	2.169519	1.153458	1.461604	1.244042	1.518879	3.449139	1.900948	0.172458	0.379597	0.537402	0	2.407919	0
LOC107810363	3.630655	3.653571	3.484807	2.603942	3.513828	4.553964	3.235511	2.746678	2.2014	3.097804	3.606575	3.035327	3.410181	3.218204	2.329003	3.015581	3.763274	4.032015	2.417715	2.751359	2.448922	3.663733	3.636147	3.873817	2.438571	1.210325	3.128958	2.704616	2.366984	2.338426
LOC107808875	3.221534	2.636991	2.696692	3.377948	3.352124	4.457709	4.203173	4.867965	4.284734	1.890659	1.966116	1.764027	2.424044	1.919978	1.77571	2.245091	2.420657	2.935522	2.482011	2.800896	2.20357	6.053372	4.525521	4.645415	1.169101	1.286652	1.551242	0	2.441328	3.688747
LOC107768082	10.42139	9.448085	7.683629	10.57816	11.87695	10.50034	9.976489	9.323894	10.29631	10.85384	11.86032	10.7403	12.75529	10.53588	10.64023	9.067147	8.960993	7.891624	9.799783	11.40051	11.09686	8.384211	10.44367	10.37918	7.79511	6.149471	10.25362	15.60789	9.98192	3.373666
LOC107773863	0	0	0	0	0	0	0	0	0	0	0	0	0	0	0	0	0	0	0	0	0	0	0	0	0	0	0	0	0	0
LOC107809956	2.942683	1.575612	1.982459	6.264917	4.133659	11.19175	2.097932	1.885731	3.557003	3.694465	4.255786	6.246227	1.433178	2.92942	1.736666	1.178679	1.06608	1.480879	2.986032	2.613773	3.086343	1.812415	3.309443	5.920706	0.604573	0.614179	0.608653	0	0.354178	0
LOC107828600	3.784506	3.898995	4.243354	3.47826	3.397313	3.832934	5.014361	4.733219	4.067998	4.706561	4.345912	4.216671	4.151908	4.667238	3.602842	3.719419	4.715905	3.925579	4.062879	3.897395	3.651622	4.946932	5.757159	6.019338	3.051155	2.518457	3.306117	3.922402	2.640574	3.391329
LOC107802168	31.26316	27.25954	26.81848	43.24362	41.32639	44.48889	29.99592	30.30553	34.2385	35.73374	31.8618	39.13347	38.63316	35.9996	27.8918	28.00941	31.05523	31.8594	35.1557	34.10924	35.96924	26.9192	33.07584	35.96539	24.61552	25.78105	33.99589	29.44554	27.18561	14.54787
LOC107824594	3.361268	3.420776	3.701047	3.146643	2.428988	3.975152	3.101164	4.260072	4.73356	1.529575	3.181846	2.366411	2.619267	3.361397	2.357441	3.673737	4.221448	4.301912	1.193774	1.232141	1.890412	3.087791	4.447291	7.553372	0.779105	0.311797	1.258037	0	1.078823	0
LOC107793082	0	0	0	0	0.018517	0	0	0	0	0	0	0	0	0	0	0.015595	0	0	0	0	0	0	0	0	0	0	0	0	0	0
LOC107804753	0	0	0	0	0	0	0	0	0	0	0	0	0	0	0	0	0	0	0	0	0	0	0	0	0	0	0	0	0	0
LOC107785992	2.253862	1.889788	1.933112	1.497382	1.329577	1.873924	1.386302	1.386548	1.749248	1.142856	1.917564	1.091699	1.137427	1.147465	1.172073	1.679581	2.177421	2.038825	1.067296	1.093124	0.931854	1.907356	2.597726	2.324326	0.455931	0	1.172109	0	0.434036	0
LOC107809763	3.491984	3.075237	3.513003	2.488194	1.492891	3.205928	0.756901	0.838103	1.436615	0.267819	0.440194	0.165737	0.087356	0.428043	0.040271	1.549643	2.312209	2.661926	0.756461	0.697006	1.216645	0.851937	2.076513	3.034682	0.049606	0	0.185494	0	0	0
LOC107798388	9.704243	9.670619	8.251214	4.963819	3.960583	5.206253	6.327023	5.604639	5.252731	4.703212	9.510236	4.139955	8.247143	6.314226	8.831482	9.603813	7.679021	9.823964	3.406386	4.06376	2.023459	6.476969	5.97313	5.202507	1.268378	1.100621	1.20853	0	1.207467	1.192899
LOC107826164	3.121973	4.324394	4.663108	1.234597	0.942767	1.265348	0.529941	0.561021	0.535032	0.302016	0.364855	0.251193	0.193079	0.283827	0.241595	4.634531	3.42302	4.599708	0.854847	1.131846	0.406756	0.682849	1.136483	0.893464	0.093979	0.137905	0.219638	0	0.238576	0
LOC107816544	9.465313	8.935781	9.047518	3.143062	2.402543	5.02737	4.301591	5.348653	5.558459	0.551586	1.401456	1.178776	1.973049	1.522188	0.890213	6.632035	8.342797	6.410154	1.301041	1.116514	1.478879	6.172618	5.887565	4.33564	0.047677	0.052471	0.757701	0	0.726205	6.995075
LOC107798458	40.32319	31.99162	36.98271	22.4463	20.08629	25.02529	18.33774	17.86737	24.10078	12.12232	12.57999	13.49975	14.16005	12.807	12.31533	30.75819	37.31154	35.03008	10.9171	9.564643	12.67937	19.41488	26.00158	35.93546	1.823346	1.846146	5.215873	8.250867	5.276786	3.566872
LOC107826268	0.627481	0.762496	0.311689	0.297768	0.819635	0.064249	0.676666	1.174396	0.420791	1.019789	2.472327	1.085471	0.532207	0.391172	2.177434	1.046533	0.433	0.445537	0.758006	0.556083	0.981041	1.222728	0.658905	0.343537	0.491107	1.912493	0.247211	0	0.86312	1.84753
LOC107764070	18.81654	22.35145	20.30819	19.32134	15.92518	16.70687	26.53279	22.76121	17.59137	18.76083	21.01431	17.68289	21.65291	17.73128	26.22958	29.69852	22.74849	24.41201	16.43884	18.35431	18.22152	22.75467	19.62787	15.137	10.29111	12.75997	11.15106	11.33974	19.21211	13.07253
LOC107827298	9.199818	9.155292	8.585357	10.96519	9.296421	8.496702	11.10546	9.626996	9.043028	8.027153	10.3386	6.819584	8.337455	8.078448	12.31665	11.3585	8.579131	10.57202	8.44853	8.410412	5.690723	12.15626	9.739475	8.712032	3.98009	6.171617	4.475994	2.016089	5.202746	5.229364
LOC107764724	9.992959	7.779195	11.0755	4.715164	3.426441	5.685183	5.552761	5.727746	8.990921	1.70527	3.328358	1.899524	2.22486	2.089517	1.02565	7.943216	7.642781	7.822684	2.239286	2.183784	2.658176	5.250636	9.144993	14.27434	0.894918	0.347612	1.328723	0	1.403199	2.574499
LOC107799932	3.462516	2.842329	2.753731	3.515067	3.58537	3.115391	2.533417	3.042396	3.089377	2.394603	3.495514	2.966722	4.209902	2.973725	2.293608	3.879556	3.890733	4.155956	3.339092	3.136784	2.911514	2.563508	2.83756	3.619021	1.924934	1.503439	2.147792	7.024648	2.246283	4.555161
Novel01722	1.922459	1.857738	2.175018	1.531602	1.410475	1.817607	1.458497	0.982259	1.433028	0.725363	1.327707	0.579469	0.946379	0.811521	1.259252	4.967371	1.745262	3.860479	1.009699	1.340241	0.519827	1.161766	1.346363	1.474199	0.219851	0.349492	0.228361	0.690869	0.651134	1.194659
LOC107775108	0.064987	0	0	0.058875	0	0	0	0	0	0	0	0	0	0	0	0	0	0	0	0	0	0	0	0	0	0	0	0	0	0
LOC107823642	2.542133	3.663841	3.80295	2.945753	2.772574	3.241588	4.66108	3.283358	2.997209	2.133007	3.772363	0.849077	1.250438	2.257371	2.973247	4.978308	3.998	4.716132	2.519591	4.200917	1.401864	5.579051	4.588953	2.965994	1.569642	3.537192	1.257738	0	0.853865	0
LOC107776360	0.147965	0.134852	0.128623	0.134049	0.13093	0	0.372315	1.011405	0	0	0.622521	0.500021	0	0.161423	0.151868	0.441056	0	0	0.06058	3.629889	0.539788	0	0.913607	0	0.374147	0.823532	0	0	1.424716	0
107767999	0	0	0	0	0	0	0	0	0	0	0	0	0	0	0	0	0	0	0	0	0	0	0	0	0	0	0	0	0	0
LOC107783067	3.692886	3.683121	3.603845	2.08309	1.603027	1.273494	3.024331	2.306943	2.273204	0.271776	0.525219	0.353191	1.163483	1.33025	1.179992	3.284159	2.507444	3.038913	0.760063	0.559949	0.57192	3.578634	3.211275	1.966315	0.484512	0.751367	0.349999	1.245724	1.174076	2.15412
Novel03791	6.586677	6.983016	7.634211	3.450419	3.290823	3.564828	2.405237	2.411929	2.271623	1.779106	1.633784	0.832798	1.147243	1.466478	0.965769	6.210627	4.761641	5.979631	2.77351	1.895196	2.411037	3.741977	3.596595	5.854075	1.019701	0.249384	1.16509	6.408714	1.078592	0
LOC107816963	23.89139	15.51918	17.67273	34.72487	45.26052	46.91727	31.06275	31.36701	33.97976	85.42277	62.37981	90.19944	100.3305	74.51996	41.93237	17.08015	20.64162	20.70831	41.58815	35.49156	49.12937	27.84674	35.35141	40.60369	68.02484	47.92775	102.3138	120.3872	125.6198	204.1714
LOC107780865	0.646664	0.147339	0.070266	0.073231	0.357632	0.086905	0	0	0.07589	0	0.11336	0.13658	0	0.17637	0	0.060237	0.156183	0.060264	0.328095	0.136758	0	0.202517	0.2139	0	0	0	0	0	0	0
LOC107769371	0	0	0	0	0	0	0	0	0	0	0	0	0	0	0	0	0	0	0	0	0	0	0	0	0	0	0	0	0	0
LOC107804538	0	0	0	0	0	0	0	0	0	0	0	0	0	0	0	0	0	0	0	0	0	0	0	0	0	0	0	0	0	0
LOC107768928	20.54769	25.5829	24.30358	18.25914	14.50581	18.77155	19.07067	19.28337	18.8167	13.46722	15.35284	12.99573	14.87433	15.18933	16.53746	19.57948	23.81011	21.38824	13.84328	16.76454	16.12855	18.94154	21.7394	19.95079	9.653222	7.18671	10.74941	24.08935	8.919367	13.88519
LOC107816925	0.056141	0.153497	0.048802	0.050861	0.099355	0.241435	0.047088	0.095937	0.105416	0.164324	0.078733	0.094859	0.062497	0.122495	0.172865	0	0.12551	0	0.251134	0.113936	0.142475	0.102403	0.140605	0.04952	0.293394	0	0	0.132709	0	3.471296
LOC107813056	30.93423	21.69441	28.65456	17.83851	15.91884	21.81824	14.35303	14.85575	19.92804	12.94304	13.03773	16.35	13.73793	14.9591	10.69594	17.00306	29.35519	23.63526	10.35199	7.42361	12.85571	14.79499	18.57772	24.93726	2.565744	2.289506	7.130841	3.922402	3.696804	0
LOC107768945	0.474383	0.198156	0.189003	1.253486	0.857102	0.637524	1.160499	1.249754	1.224771	0.828795	1.164231	0.935134	1.29822	1.207566	1.399819	0.294592	0.401007	0.427354	0.842399	0.685546	1.045559	1.337072	0.871744	1.177584	2.449036	3.135328	1.775494	2.827089	1.427405	1.222158
LOC107776269	0.710119	0.924552	0.999425	4.564595	9.245874	5.126124	3.800545	5.287352	2.825502	7.419173	5.477298	9.541722	7.754574	9.812935	8.017318	0.680377	1.012718	0.781531	11.80379	9.21092	15.11162	3.897115	2.535316	3.463678	22.31691	24.41968	16.02671	16.92786	35.16447	71.08874
LOC107765581	0	0	0	0	0	0	0	0	0	0	0	0	0	0	0	0	0	0	0	0	0	0	0	0	0	0	0	0	0	0
LOC107816642	5.190787	3.440555	4.676323	2.223043	1.336189	1.522018	1.068644	0.88703	1.329093	0.368122	1.058846	0.797331	0.472783	0.308885	0.387468	2.778057	3.37355	3.940315	0.670378	0.758454	1.463263	1.300489	1.623328	2.614061	0	0.26264	0.334641	0	0	0
LOC107816029	0.020255	0	0	0	0	0	0	0	0	0	0	0	0	0	0	0	0	0	0	0	0	0	0	0	0	0	0	0	0	0
LOC107799126	0	0	0	0	0	0	0	0	0	0	0	0	0	0	0	0	0	0	0	0	0	0	0	0	0	0	0	0	0	0
LOC107767692	0	0.103328	0	0	0	0	0.031698	0	0	0	0	0.031928	0	0.082459	0	22.30483	33.11483	27.35848	12.84683	11.34916	17.02668	23.98645	36.90193	50.20479	1.433419	2.05081	7.370079	0	4.184719	2.336738
LOC107811552	13.73895	14.79797	13.30014	16.54858	14.50567	15.35854	18.52919	13.73989	12.38572	20.4003	22.49703	15.03641	11.3839	11.24139	18.42782	12.9142	12.44342	14.02589	16.15928	18.68798	13.95404	18.12332	15.21719	11.91223	15.10024	17.92195	11.99423	11.16511	7.892207	19.30682

LOC107802238	1.237617	1.09323	1.357206	1.086719	0.842404	1.023531	0.431187	0.341639	0.554151	0.3342	0.587446	0.386058	0.254351	0.31158	0.273594	1.177673	1.122064	1.107235	0.850113	0.837551	0.347301	0.477031	0.890123	0.636828	0.168509	0.079479	0.472585	0	0.183333	0
LOC107801504	1.22129	1.442849	1.218921	4.343749	5.043162	2.139762	2.27633	2.89862	1.061667	3.043441	3.774368	3.783192	5.689997	4.737305	4.921142	3.741549	1.092471	2.697847	6.97669	4.476884	15.88249	2.417614	1.117157	1.276492	7.891972	4.342747	6.308482	19.40877	5.226421	13.98411
LOC107816315	12.80698	17.82824	12.33776	9.957611	11.83343	8.605668	8.416566	7.839994	6.818349	16.18164	17.6046	8.659052	17.40821	14.54684	9.918662	20.57149	16.80488	18.39765	10.44009	9.975044	7.798657	7.581373	8.0075	9.978172	14.39007	7.252948	10.31426	4.188476	36.1861	18.10689
LOC107792956	0.517491	0.589536	0.520132	1.128099	0.472219	0.921481	0.773137	0.552698	0.455478	0.394233	0.498939	0.464515	0.378051	0.405775	0.51454	0.927933	0.781156	0.843962	0.8369	0.998643	0.427714	0.783308	0.941451	0.693004	0.24535	0.270019	0.114681	0	0.077856	0
LOC107809811	0.363244	0.529683	0.378912	2.632644	1.414257	1.718339	0.243735	0.186219	0.272823	0.283367	0.152824	0.42963	0.080874	0.396281	0.149129	0.108276	0.14037	0.162488	1.695536	1.413485	1.987707	0.182013	0.576729	0.835257	0.918501	0.202171	0.429325	0	0	0
LOC107760990	0	0	0	0	0	0	0	0	0	0	0	0	0	0	0	0	0	0	0	0	0	0	0	0	0	0	0	0	0	0
LOC107763099	7.064376	13.50474	10.01019	13.16416	14.86524	12.13358	14.57213	12.4155	9.166659	10.66902	18.66641	4.415483	10.38964	9.116683	21.4573	15.57914	10.57009	14.5372	15.2985	17.24773	5.919022	22.15964	10.91735	7.773878	14.23234	25.41302	4.344468	6.161009	9.124747	46.16596
LOC107762311	18.30432	22.67727	17.26119	58.1878	60.95516	33.47251	118.0575	97.88779	75.4908	79.60344	79.84629	81.73826	92.96833	80.14388	81.01915	19.39494	17.68351	17.302	60.48973	63.45754	82.31407	83.66581	59.39281	50.73308	74.85062	105.1738	74.27108	61.36019	66.87944	73.26277
LOC107761617	4.164572	9.542193	6.832442	7.81148	6.903051	5.70714	2.582857	3.332796	3.083852	2.173484	5.573059	2.477709	2.220085	2.015709	6.652416	24.04076	6.431657	16.83624	6.81504	8.484831	4.547093	4.702571	3.880382	4.659346	0.370795	1.224232	1.455859	0	2.541516	3.626786
LOC107782943	0.077946	0	0	0.070615	0.206915	0.419008	0.326884	0.466195	0.512254	0.304029	0.163968	0.329255	0.433853	0.510212	0.240005	0.232342	0.150605	0.232448	0.474566	0.065937	0.426529	0	0	0.325877	0.098548	0.325369	0	0	1.12578	0
LOC107825703	0.735859	0.884031	0.523363	0	0	0.107882	0	0	0	0	0.046908	0.14129	0.037235	0.072981	0	0.22433	0.420078	0.374055	0	0.028295	0	0	0	0	0	0	0	0	0.161031	0
LOC107818635	0.187405	0.284661	0.325815	0.452746	0.221105	0.134322	1.650441	1.734678	0.322565	0.974633	1.598805	0.448588	0.9388	0.2726	2.532577	0.395688	0.301749	0.442442	0.633886	0.79266	0.769125	1.278141	1.349981	0.522336	0.710812	1.173423	0.70141	2.233687	0.60149	0
LOC107807642	0	0	0	0	0	0	0	0	0	0	0	0	0	0	0	0	0	0	0	0	0	0	0	0	0	0	0	0	0	0
LOC107762814	0	0	0	0	0	0	0	0.065145	0	0	0	0.064413	0	0	0	0	0	0	0	0.064497	0	0	0.134505	0	0	0	0	0	0	0
LOC107787048	2.382798	1.219159	1.889622	2.6889	4.512837	6.696634	5.15417	4.857652	5.02361	2.568111	2.22776	2.895973	2.978318	3.329205	1.930768	1.40184	1.978898	1.277817	3.181446	2.758304	3.431293	3.316562	2.949875	4.107141	2.06124	1.744995	1.630476	5.97908	2.012568	0
LOC107772832	0.787317	0.928585	1.650608	0.545441	0.327845	0.597503	0.660357	0.791418	0.521766	0.677412	0.71444	0.117379	0.360892	0.353675	0.57041	1.069883	1.073809	1.208485	0.234975	0.626839	0.21119	1.237665	1.429783	0.726092	0.17566	0.064441	0.054738	0	0	0
LOC107809798	15.14961	20.19273	11.29262	4.014497	4.088637	4.234796	4.860285	5.242415	7.147116	5.244336	6.878336	8.063249	17.0334	13.13934	7.23035	12.19266	11.59887	13.75127	3.651034	2.466988	4.041383	2.593625	4.109108	3.800247	2.681519	2.740344	4.297348	0	3.282102	2.341807
LOC107819591	20.51054	17.53931	18.05506	15.14158	11.57291	15.07307	16.63145	13.91998	16.87964	7.373552	8.215036	8.883277	10.76601	12.53339	9.359903	20.07297	23.29505	20.37251	8.002343	9.361585	10.32971	14.55413	18.77868	16.14605	2.17464	2.393295	4.296016	0	4.843506	2.006642
LOC107818163	3.296628	3.560854	3.644024	3.650262	4.105511	3.413009	3.993924	2.921052	3.286071	2.063712	3.110669	0.893977	1.223284	1.110018	2.589892	4.458367	2.752314	3.186006	3.0561	3.339579	2.041505	6.695812	4.631011	3.658329	1.595138	2.831492	1.154474	2.910562	1.17564	7.549463
LOC107830038	0.294268	0.268189	0.383702	0.533184	0.585873	0.632749	0.185112	0	0.828816	0.071737	0.103171	0	0	0.321032	0.075507	0	0.142144	0	0.149302	0.124465	0.268378	0.184313	0.129782	0	0	0	0.173901	0	0	0
LOC107766059	0	0	0	0	0	0	0	0	0	0	0	0	0	0	0	0	0	0	0	0	0	0	0	0	0	0	0	0	0	0
107818040	0	0	0	0	0	0	0	0	0	0	0	0	0	0	0	0	0	0	0	0	0	0	0	0	0	0	0	0	0	0
LOC107824673	4.628613	5.37944	5.333979	6.078345	4.584179	5.775276	5.7699	5.134	5.940241	3.933769	5.642556	4.466444	4.703553	4.864283	4.83785	6.170673	4.984431	6.347623	4.481347	4.741692	3.775999	5.106683	5.880648	6.036185	2.818611	3.13156	2.333809	1.5184	3.577673	14.44099
LOC107760353	0	0	0	0	0	0	0	0	0	0	0	0	0	0	0	0	0	0	0	0	0	0	0	0	0	0	0	0	0	0
LOC107779647	13.16209	13.73989	15.23591	17.03456	17.55916	17.65248	17.34797	15.23381	16.7389	14.83194	13.89099	15.82856	14.80174	14.61561	14.33631	15.81356	20.04667	17.89853	15.09362	16.24688	17.30176	15.53462	16.9705	17.16118	6.537492	7.101383	10.19906	14.40728	9.860682	16.60882
LOC107762520	8.778015	8.496434	8.159667	9.026306	8.787895	7.990825	8.03426	9.580515	9.714976	8.653392	7.255888	9.121029	10.02145	10.17054	10.91664	10.67153	8.913601	10.43757	13.16593	12.35793	13.55709	8.748741	10.08823	10.34674	7.047686	14.57652	4.884537	4.582133	16.96589	17.82781
Novel02456	0	0.036358	0.069357	0	0.0706	0	0	0	0	0.077802	0	0	0.04441	0.043522	0	0.059457	0	0.029742	0.080962	0	0.036383	0	0	0.041696	0.302623	0	0.471505	0	3.07296	0
LOC107831708	0	0	0	0	0	0	0	0	0	0	0	0	0	0	0	0	0	0.033463	0	0	0	0	0	0	0	0	0	0	0	0
LOC107814797	0	0	0	0.075323	0	0	0	0	0	0	0	0.070241	0.092555	0	0	0	0	0	0	0	0	0	0.146674	0	0	0	0	0	0	0
LOC107776706	4.603281	5.890403	5.254541	4.065028	4.58759	5.598971	3.022477	2.363874	3.383199	1.995014	3.015896	1.905207	4.115078	4.083508	3.41227	4.539177	3.638583	4.974582	2.736599	2.753375	2.438389	3.164769	2.624904	3.231865	0.235149	0.064698	0.439653	0	2.462426	2.875028
LOC107773634	0	0	0	0	0	0	0	0	0	0	0	0	0	0	0	0	0	0	0	0	0	0	0	0	0	0	0	0	0	0
LOC107787833	79.33202	98.78596	85.60755	46.88462	38.41258	31.77342	53.82565	55.17701	45.55556	31.13591	46.00524	27.84686	23.31397	25.92995	33.69278	82.07721	81.10385	74.39146	47.14453	50.53124	55.41953	53.18156	69.18495	44.95092	38.89523	50.79319	30.05326	60.93491	15.11322	27.72876
LOC107818401	0.548684	0.535777	0.493994	0.585845	0.433493	0.610971	0.657436	0.535784	0.441539	0.382169	0.769476	0.463544	0.698061	0.470319	0.824619	0.773952	0.6058	0.788916	0.278383	0.480726	0.142974	1.243742	0.760534	1.044562	0.099101	0.190864	0.138964	0	0.377366	0
LOC107776597	0.123392	0.393597	0.268154	0.950186	1.801554	0.862297	0.155241	0.263573	0.173768	1.203225	0.908484	1.824282	0.412084	1.346142	2.722885	0.091952	0.238413	0.137991	0.688651	0.678476	0.95655	0.41219	0.217679	0.193453	0.468014	0.515071	0.729195	0	0	3.814746
LOC107815856	4.420737	5.117864	4.403702	3.420456	3.002539	3.648121	2.785908	2.18465	3.253003	2.400054	3.016045	2.5033	2.1813	2.945823	2.305451	4.024435	3.970693	4.56074	2.158062	2.324641	1.678095	2.634201	2.761184	3.496635	0.483386	1.03073	0.762561	1.708893	1.610606	0
LOC107767511	4.893074	4.43768	4.284568	4.249075	3.337048	4.298338	3.413353	3.03871	3.305319	2.269308	2.719716	2.429871	2.856374	2.382617	2.560055	4.393376	4.842417	4.626725	2.73689	2.251323	2.634002	2.561434	3.800199	4.228603	1.192001	1.079373	2.355604	1.706946	2.3557	1.475835
LOC107792856	0	0	0	0	0	0	0	0.06114	0	0	0	0	0	0	0	0	0	0	0	0	0	0	0	0	0	0	0	0	0	0
LOC107791677	0	0	0	0	0	0	0	0	0	0	0	0	0	0	0	0	0	0	0	0	0	0	0	0	0	0	0	0	0	0
LOC107819684	1.746694	1.668614	1.81106	0.857936	0.651755	1.470652	0.211811	0.35962	0.296363	0.307816	0.265616	0.195569	0.538822	0.45917	0.129597	1.223226	0.935215	1.81999	0.619281	1.103736	0.249508	0.43937	0.426941	0.263948	0.106427	0.087846	0.59695	0	0	0
Novel01632	1.73279	1.917629	1.958161	1.90621	1.839944	1.729892	3.010543	1.903555	2.370508	1.544858	2.777219	1.777604	2.314759	1.782366	1.880116	1.567979	1.913167	1.974713	1.632721	1.340179	1.173962	2.522085	2.074305	1.96635	1.721322	0.998864	1.813959	0	1.310928	1.530586
LOC107780461	0	0	0	0	0	0	0	0	0	0	0	0	0	0	0	0	0	0	0	0	0	0	0	0	0	0	0	0	0	0
LOC107790008	0	0	0	0	0	0	0	0	0	0	0	0	0	0	0	0	0	0	0	0	0	0	0	0	0	0	0	0	0	0
LOC107827037	0	0	0	0	0	0	0	0	0	0	0	0	0	0	0	0	0	0	0	0	0	0	0	0	0	0	0	0	0	0
LOC107789270	2.849459	3.437114	2.185566	4.024049	4.301193	3.243724	2.671136	3.795198	2.675198	3.759259	4.231149	3.681756	3.358649	4.66293	3.354696	2.310781	3.88633	4.623678	3.91194	3.970147	3.974566	3.639476	3.696193	2.715467	3.708541	3.031917	2.971616	0	1.210439	5.181951
LOC107799225	0.141059	0	0	0	0	0	0	0	0	0	0.09891	0	0	0	0	0	0	0	0	0	0	0	0	0	0	0	0	0	0	0
LOC107779280	0	0	0.091985	0.095865	0	0	0.088754	0.090414	0	0	0.074199	0.089398	0	0.115442	0	0	0	0.078892	0	0	0.096508	0	0.186676	0.110601	0	0	0.250136	0	0	0
LOC107774517	1.122298	1.102746	0.990834	0.651352	1.070668	1.036929	0.558909	0.56936	0.543295	0.359094	0.713179	0.237037	0.409946	0.267831	0.467958	1.555066	0.931764	1.346598	0.765152	0.904885	0.543764	1.21551	0.696052	0.659827	0.731634	0.390399	0.352343	1.254065	0.337696	0
Novel04915	2.461183	5.277799	1.334012	14.19143	14.29672	3.735632	31.23165	23.10718	11.63489	7.65165	9.826811	3.547	12.44202	8.339386	10.99592	4.833331	8.168132	2.914279	8.814488	7.568651	25.45686	21.37614	13.48528	7.808074	1.757186	2.256178	3.353814	4.141397	4.182004	3.580674
LOC107819443	0.189782	0.086481	0	1.461427	2.099142	0.918174	4.21822	4.702486	0.890877	1.57302	3.127264	0.641332	1.056338	2.070426	3.700949	0.848553	0.458363	0.14149	5.488456	3.451659	6.923366	4.279276	2.427301	2.181958	5.638633	8.054057	1.345841	6.786039	5.482061	41.07072
LOC107783975	0	0	0	0	0	0	0	0	0	0	0	0	0	0	0	0	0	0	0	0	0	0	0	0	0	0	0	0	0	0
Novel03368	5.203853	5.286911	6.45169	5.719141	4.906659	6.787108	4.579352	5.90412	7.288363	3.909794	5.084595	4.468431	3.038951	4.467263	4.115267	5.467227	4.862528	5.342528	3.808892	3.10312	4.434788	5.628262	7.524823	8.114015	2.049275	2.374027	3.730652	6.100804	2.874957	0
LOC107817656	0	0	0	0	0	0	0	0	0	0	0	0	0	0	0	0	0	0	0	0	0	0	0	0	0	0	0	0	0	0
LOC107778246	0	0	0	0	0	0	0	0	0	0	0	0	0	0	0	0	0	0	0	0	0	0	0	0	0	0	0	0	0	0
LOC107819355	1.22429	1.236415	1.438175	1.049191	1.229729	1.814306	1.137877	1.583236	1.460071	0.354925	0.487241	1.090223	0.14734	1.010755	0.713193	1.134264	1.918001	1.628167	0.872985	0.979683	1.02604	0.801369	1.313398	1.245043	0.125503	0.460408	0.74306	0	0.318604	0
LOC107831060	0.781787	0.769503	0.434938	0.623269	0.470408	0.941379	0.340974	0.454227	0.557822	1.067278	1.162163	0.686895	0.55699	0.477619	0.385155	0.442768	0.543796	0.489599	0.539447	1.084595	0.427802	0.339503	0.248252	0.424905	0.751197	0.87024	0.369604	0	0.451656	0
LOC107766649	2.433473	1.957697	1.632227	5.130466	6.101009	3.181527	7.987848	9.279507	9.195019	3.705894	3.044049	3.007656	3.779189	4.522992	4.625278	2.764917	2.655691	2.475006	5.395923	5.197206	4.068842	9.007164	7.048949	5.746348	7.102883	7.712557	3.533073	5.371227	10.1246	11.14558
Novel00549	0	0.056361	0.107515	0.392177	0.273607	0.265949	0.155607	0.369873	0.116119	1.145762	0.173453	0.104491	0.275371	0.269864	0.126945	0	0	0.092211	0.376515	0.418509	0.733207	0.206582	0.381838	0.064637	6.724037	0.344192	6.651344	4.422543	5.954541	7.647508
LOC107761825	0	0	0	0	0	0	0	0	0	0	0	0	0	0	0	0	0	0	0	0	0	0	0	0	0	0	0	0	0	0
LOC107759074	0	0	0	0	0	0	0	0	0.028541	0	0	0.025683	0	0	0	0	0	0	0	0	0	0	0	0	0	0.035931	0	0	0	0
LOC107786069	17.29974	16.67619	19.08707	14.40012	11.9716	15.65841	10.29344	9.759491	12.806	9.407951	9.668181	9.02526	7.160141	7.178255	7.246563	15.20569	17.74864	16.94889	10.99037	9.381018	10.21502	9.724254	13.62922	16.69078	3.271472	5.812093	4.936965	0	4.271133	0
LOC107789620	9.017603	11.6395	11.02684	13.09464	12.0645	13.77714	11.25471	13.64023	11.05862	9.29818	8.410696	8.019286	7.925149	8.519784	8.546872	7.909581	9.545424	8.58872	12.34653	14.63606	14.2448	11.31427	11.91215	9.425196	9.655385	9.725687	9.128213	15.4279	10.38611	5.335617
LOC107760533	13.98332	12.36553	15.06387	6.940323	5.55372	7.392848	5.690022	6.092158	6.304115	5.040186	4.983456	5.692312	6.807101	7.602384	5.091915	11.8648	11.25755	13.3325	6.158065	6.558613	5.597861	6.224325	6.838771	6.728855	4.055122	4.462856	4.663604	6.600694	6.665414	5.706995
LOC107768190	0	0	0	0	0	0	0	0	0	0	0	0	0	0	0	0	0	0	0	0	0	0	0	0	0	0	0	0	0	0
LOC107776816	0.153968	0.056129	0.267681	0.055795	0.108992	0.09932	0.077484	0.052622	0.115641	0.150138	0.345479	0.182106	0.102839	0.302348	0.379267	0.275368	0.089247	0	0.031247	0.078148	0.112337	0.385747	0.190133	0.128741	0	0.042847	0.036395	0	0.148251	0
LOC107791425	0	0	0	0	0	0	0	0	0	0	0	0	0	0	0	0	0	0	0.025408	0	0	0	0	0	0	0	0	0	0	0
LOC107807001	10.27473	8.780624	9.382157	6.601493	5.719442	10.35822	5.395749	5.131941	5.20961	4.667678	5.921918	5.82126	6.482647	5.953859	3.911609	7.952102	9.24879	9.319585	3.155669	4.281377	3.00308	5.703487	7.072896	9.591933	1.002225	1.527226	2.198154	0	1.908191	9.425843
LOC107799007	34.28675	34.66449	36.5825	18.65535	15.74453	22.73005	11.98939	14.26342	22.47663	7.603047	9.813017	12.66752	7.066178	11.72319	8.925993	26.70514	28.7299	31.59879	15.82369	14.92492	20.42129	14.69254	20.27969	26.59006	1.516579	0.973623	5.375657	7.148652	3.609372	0
LOC107789658	0.744736	0.678735	0.215794	0.449795	2.855629	0.800682	2.290354	1.484753	2.097573	0.968282	1.914767	0.419449	0.5527	1.895764	3.312298	0.184993	0.479652	0	0.755707	1.469989	2.490443	1.865845	1.532781	1.556796	0.627715	1.381661	0.880218	0	4.780565	15.34938
LOC107802549	0.1276	0.145364	0.02773	0.0289	0.028227	0.137186	0.026756	0	0.059898	0.031107	0.022368	0.0539	0.071023	0.034801	0	0.023772	0.092454	0.118914	0	0.026985	0	0	0.028138	0.033342	0	0	0	0	0	0
LOC107808179	355.6991	263.5404	349.4084	162.6862	139.5817	227.6708	154.8155	171.1295	230.3272	81.62204	85.8012	111.3952	67.82006	103.7437	57.74674	174.5252	298.4508	224.7221	76.61852	67.37698	133.9483	127.3396	222.9438	264.8074	19.52897	11.64681	49.8737	24.68464	22.01856	16.00685
LOC107805665	0.691286	0.792028	0.978642	0.375762	0.436926	0.573339	1.358416	0.877543	0.908614	0.269637	0.623225	0.13349	0.46173	0.280116	0.587886	0.809517	0.64876	0.809888	0.320672	0.267328	0.090066	1.237096	0.644604	0.330301	0	0.219857	0.210097	0	0	0
LOC107773124	40.07935	26.81893	31.56587	29.43178	30.82994	35.11756	29.37105	27.2551	39.78318	30.41657	34.21136	45.14686	60.01332	46.80651	34.4313	26.13926	41.33547	35.67274	13.3177	18.27152	17.92763	23.73956	31.35535	37.89252	10.19402	4.913435	21.14633	16.83548	20.684	0
LOC107830970	0.406495	0.176414	0.353357	0.368264	0.205539	0.374599	0.032471	0.132312	0.308943	0.169879	0.257888	0.130826	0.430967	0.232292	0.059602	0.288496	0.561012	0.288628	0.117852	0.229244	0.070615	0.307144	0.136593	0.101159	0.097892	0.053867	0.13727	0	0.186382	0
LOC107759866	5.874126	7.247152	6.711713	7.227233	7.013643	7.446109	6.518976	6.027199	6.068785	4.252238	5.953578	4.680904	6.11083	6.128541	6.029042	7.512316	5.922712	6.004956	6.585355	6.987128	5.099964	6.983569	7.941747	5.871529	3.275523	3.890404	2.698272	3.668848	7.286148	15.86053
LOC107761386	8.218204	7.407573	6.358865	7.527093	5.434028	9.321045	2.575392	3.395183	4.493708	79.34529	37.9319	31.66298	15.78388	7.684849	0.741533	2.018972	4.449596	2.423876	11.54668	13.52209	7.906961	2.262606	2.549099	3.209325	25.34778	31.1636	92.863	12.91687	7.391331	5.583999
LOC107770431	16.78967	1.55766	0.611763	2.277035	1.957158	0.216179	1.096221	0.773114	0.755108	3.823413	48.99513	21.82866	6.043635	23.58139	39.72753	1.798086	1.456908	1.873864	0.204036	0	0.275074	13.51773	0.266041	0.210162	0	0	0.35648	0	0	0
LOC107815481	14.96565	13.7604	14.89528	14.56094	12.10639	15.75668	10.39838	11.72777	12.63705	9.541835	9.624601	9.688281	8.182113	9.32269	6.771269	12.63721	10.77939	13.66632	9.030789	9.139104	9.812686	8.689578	13.08352	16.28999	5.430027	2.217899	6.646154	12.66574	5.115973	0
Novel02873	0.150286	0.63005	0.174188	0.671682	0.478739	0.31238	0.907571	0.779012	0.395067	0.283326	0.723615	0.126966	0.557669	0.32791	0.812382	0.97061	0.27102	0.403361	0.742167	0.788217	0.420328	0.920392	0.592112	0.261799	0.228009	0.641278	0.094734	0.716506	0.385883	0.619495
LOC107825509	2.633031	3.199577	2.655052	3.657602	3.883132	3.849929	3.474607	2.879658	3.757448	3.697254	3.544863	3.114241	3.712761	2.910807	3.026767	1.935976	2.374147	2.303296	3.384303	2.851025	3.65008	2.638695	3.220544	4.586739	3.018249	4.15216	4.190868	2.510653	4.394467	6.512171
LOC107822330	0	0	0	0	0	0	0	0	0	0	0	0	0	0.068931	0	0	0	0.094213	0.064115	0	0	0	0.055733	0	0.159768	0.439582	0	0	0	0
LOC107810032	0	0	0	0	0	0	0	0	0	0	0	0	0	0	0	0	0	0	0	0	0	0	0	0	0	0	0	0	0	0
LOC107793798	0.123829	0.169283	0.376747	0.617007	0.547861	0.266263	0.05193	0.105803	0	0.301873	0.607804	0.052307	0.206773	0.270183	0.25419	0	0	0	0	0.104751	0	0	0	0	0	0	0	0	0	0
LOC107780016	6.384486	11.14562	5.315395	5.010103	2.625784	3.818762	3.092291	1.84397	1.477403	3.375897	4.634439	3.114724	3.353444	3.531631	5.122307	9.247412	8.03571	6.201285	2.600539	3.613235	2.829369	1.764768	2.379523	1.033855	0.73898	0.750722	0.637686	0	0.865836	0
LOC107781694	0.600251	0.230339	0.109849	0.057242	0.195684	0	1.298387	0.134966	0	0.154032	0.08861	0.02669	0.105506	0.068931	0.097276	0.117712	0.305207	0.094213	0.192345	0.133624	0.028813	0.105534	0.417995	0.06604	0.119826	0.087916	0	2.259284	0	0
LOC107813070	0.891347	1.337993	0.524149	9.286428	11.80764	4.14327	5.69504	8.511903	1.772124	5.956413	7.647221	5.2048	7.412768	3.517858	12.13527	0.547015	0.633174	0.566811	32.08235	28.8523	24.24425	10.72794	4.971731	6.329644	18.89272	35.49289	7.250564	9.374108	12.24272	12.96785
LOC107827998	1.487793	0.947006	1.334364	0.834393	0.58511	1.44722	1.010185	1.049253	0.997718	0.667822	1.589701	1.197075	1.261891	1.262418	0.702919	1.742251	1.711111	2.359278	0.838728	0.879002	0.538449	1.045273	1.270671	1.752506	0.029858	0.131438	0.474503	0	0.454779	0
LOC107781289	0.056904	0.051861	0.049465	0.103104	0.12588	0.061178	0.119319	0.14586	0.026712	0.221953	0.079802	0.264406	0.253384	0.527672	0.087606	0.212023	0.08246	0.148484	0.375322	0.120341	0.285434	0.142565	0.125482	0.118951	0.323746	0.673006	0.100883	0	0.273954	0
LOC107775959	0.259306	0	0.225409	0	0.114725	0	0.054373	0	0.060862	0.063214	0	0.219069	0.072166	0	0.066536	0.483087	0.688906	0	0.263126	0.164516	0.41386	0.162415	0.171544	0.406539	0	0.451006	0	0	0.936293	0
LOC107775771	1.867101	1.241732	1.315972	0.274297	0.133957	0.434024	4.274807	4.05293	6.727411	0.590484	1.167676	1.236324	1.29203	4.01878	0.362551	0.601672	1.560023	2.483033	0	0.042688	0.230112	0.337138	1.112775	0.738403	0	0	0	0	0	0
Novel03211	0.31416	0.332748	0.287854	0.099999	0.067619	0.118672	0.071216	0.072548	0.127545	0.041398	0.119075	0.071733	0.04726	0.064841	0.026144	0.411278	0.295302	0.341834	0.060311	0.043096	0.085181	0.099272	0.149789	0.141993	0.010735	0	0.030106	0.607212	0	0
LOC107778691	0	0	0	0	0	0	0	0	0	0	0	0	0	0.099122	0	13.20299	16.85312	15.00411	9.081369	10.14549	11.80815	9.02949	9.456869	15.85915	3.905662	3.476621	7.087549	0	9.623324	5.6179
LOC107787060	0.539843	0.929334	0.417132	1.480796	1.764794	1.418749	1.823736	2.139717	1.506406	1.681604	2.386894	0.988161	1.402242	2.208533	2.862747	2.112031	0.738839	1.050909	3.454154	2.803446	3.104514	2.567266	1.745983	0.971753	0.606689	1.710957	1.541956	1.0724	0.649749	1.854406
LOC107813154	0.882805	0.715171	0.767404	0.355456	0.086796	0.210916	0.082272	0.167621	0.184181	10.23451	24.07313	13.17612	13.32173	8.239828	3.221642	0.511675	2.653354	1.974506	0	0	0.268378	0.163834	0.173043	0.205046	4.092491	0.682421	11.36152	21.04435	24.08411	6.065018
LOC107808984	0.975642	1.208725	1.139641	1.505361	2.131301	1.409505	1.943493	1.797482	1.373963	1.724365	2.063051	0.734097	1.747939	1.546673	2.550369	1.817623	1.354918	1.852552	2.212062	2.295437	0.917608	2.749895	1.761501	1.545544	2.293553	2.439319	0.972953	0	1.688015	1.885169
LOC107829027	0	0	0	0	0	0	0	0	0	0	0	0	0	0	0	0	0	0	0	0	0	0	0	0	0	0	0	0	0	0
LOC107782586	0.467909	0.568588	0.42934	1.907556	1.955171	1.676863	5.799613	5.130687	4.002449	0.709753	1.895676	1.098061	2.025653	1.361241	2.561325	0.5424	0.401812	0.523269	1.688182	1.693223	1.94404	3.951045	3.30181	3.450575	0.262924	0.39787	0.368687	0	1.626934	0
LOC107829006	6.800544	6.670976	6.227454	8.512455	10.65707	7.367229	9.44846	8.915454	11.64833	7.947543	8.481459	8.113575	9.997631	9.061446	5.967541	6.731241	5.867771	7.58579	8.902458	8.343744	9.421678	7.716928	9.936495	12.47957	9.254287	8.234508	9.510251	11.13744	13.99583	9.629493
LOC107798813	0	0	0	0	0	0	0	0	0	0	0	0	0	0	0	0	0	0	0	0	0	0	0	0	0	0	0	0	0	0
Novel01920	0	0	0	0.617041	0.487885	0.104609	0.190422	0.277119	0.426297	0	0.022742	0.054801	0	0.070766	0.033289	0	0.062667	0	0.855688	0.493854	1.360666	0.081258	0.371908	0.237295	0	0.180514	0.076667	0	0.312291	0
LOC107823606	0.06622	0	0	0.509929	0.556657	0.035597	4.804322	2.715835	2.673308	0.678012	1.300132	0.53147	1.658625	1.986666	1.257372	0.024673	0	0	0.268781	0.168052	0.452951	2.212071	0.963767	0.311458	0.083722	0.04607	0	0	0.159403	0
LOC107803533	0.363748	0.221008	0.421598	0.988613	1.502053	1.173222	0.406789	0.310796	0.683006	0.709401	0.595141	0.921914	1.754695	1.058218	2.115601	0.271066	0.702823	1.446346	1.599462	1.743668	0.995234	0.607551	1.925101	0.760379	1.379665	2.361937	1.146457	8.67105	4.086166	0
LOC107829400	0	0	0.021307	0.088822	0.108444	0.026352	0.041117	0.146599	0.069036	0.167308	0.017187	0.062122	0.054572	0.213921	0.226416	0	0.02368	0	0.174104	0.103673	0.089417	0.061409	0.064861	0.051238	0.030989	0.13642	0.02897	0	0	0
LOC107811415	0	0	0	0	0.014161	0	0	0	0	0	0	0	0	0	0	0	0	0	0	0	0	0	0	0	0	0	0	0	0	0
LOC107797198	4.050238	4.529193	3.607185	3.444199	2.638466	3.4462	3.751409	4.267406	3.5226	5.136764	4.686919	3.148853	3.983218	3.090316	3.162421	3.851499	4.344966	4.149668	3.328264	3.195005	2.628777	3.340957	3.331483	3.739854	2.607489	2.523924	3.700423	1.776989	3.469198	1.536394
LOC107780937	0	0	0	0	0	0	0	0	0	0	0	0	0	0	0	0	0	0	0	0	0	0	0	0	0	0	0	0	0	0
LOC107775470	1.617427	1.020521	2.054916	4.395922	6.055103	6.821965	3.704578	3.508087	6.7749	4.792249	3.446044	7.462906	2.562311	7.465335	1.851631	0.741728	0.480791	1.484136	9.405645	5.73605	9.871963	2.701512	3.347206	2.990946	6.292061	4.327946	23.23413	4.448805	4.492426	7.692921
LOC107806215	0	0	0	0	0	0	0	0	0	0	0	0	0	0	0	0	0	0	0	0	0	0	0	0	0	0	0	0	0	0
LOC107818737	0.298228	0.197672	0.023568	0.122809	0.119951	0.17489	0.068219	0.162155	0.203629	0.185061	0.171097	0.022905	0.181087	0.059155	0.16696	0.161629	0.157153	0.181916	0.110044	0.022935	0.074179	0.226417	0.239143	0.085011	0	0	0.096131	0	0	0
LOC107764420	0.68124	0.206955	0.592187	1.44006	1.607482	1.627591	1.015796	2.004907	1.634477	2.583368	2.706886	2.62186	1.348205	1.321243	3.34065	0.451254	0.585009	0.902921	4.838925	4.098005	3.520712	3.097459	1.869462	1.02849	4.784967	13.05989	2.326048	0	2.915316	9.360461
LOC107775994	0.797951	0.796494	0.545004	0.877811	0.739702	1.062155	0.446184	0.243497	0.285391	0.333473	0.492914	0.43337	0.296097	0.51817	0.214499	0.821161	0.624052	1.005675	0.578361	0.94823	0.31189	0.237996	0.402197	0.556012	0.264223	0.105742	0.359281	0	0.64027	0
LOC107811597	0	0	0	0	0	0	0	0	0	0	0	0	0	0	0	0.03189	0	0	0	0	0	0	0	0	0	0	0	0	0	0
LOC107792151	0	0	0	0	0	0	0	0	0	0	0	0	0	0	0	0	0	0	0	0	0	0	0	0	0	0	0	0	0	0
LOC107797529	5.310391	6.464303	5.536224	7.334168	7.492085	7.665659	7.677791	6.980526	6.92061	4.345428	4.648052	4.517739	6.26299	5.449014	7.375245	6.987557	5.72224	6.949227	7.724941	7.759298	6.113226	8.140888	7.779563	8.073336	2.981369	3.33281	1.997054	3.983581	8.760416	8.036525
LOC107823514	50.98607	57.10529	42.27668	55.10803	42.72643	40.14448	62.07629	72.5155	69.83925	95.44355	117.0473	104.5953	44.50815	45.29687	88.95623	56.88413	46.33957	48.51111	87.12227	116.9392	69.14117	80.3342	96.9636	65.30832	41.40832	106.0515	64.07066	26.99369	26.00029	28.72479
LOC107759445	17.09465	14.46113	13.75504	37.52592	40.02697	36.4278	38.16119	34.75511	38.23004	47.52928	40.4477	56.06472	65.09237	62.3082	38.95996	16.00535	24.64527	19.14986	39.80792	33.59378	46.49199	35.06818	32.55434	42.19437	41.34147	32.01956	40.61623	31.34639	56.34366	24.39203
LOC107828412	18.28171	16.7998	19.58463	15.08476	13.49191	17.0045	9.225646	9.819458	10.46913	12.9449	11.8617	11.24722	9.457939	10.09636	8.603341	16.05431	17.66167	18.26732	12.12359	11.96778	12.69518	10.13612	11.0404	12.2894	8.248022	6.43858	12.91071	0	8.034534	7.035596
LOC107811408	0	0	0	0	0	0	0	0	0	0	0	0	0	0	0	0	0.124028	0	0	0	0	0	0	0	0	0	0	0	0	0
LOC107797604	0	0.027868	0	0	0	0	0	0	0	0	0	0	0	0	0	0.091146	0	0.091188	0	0	0	0	0	0	0	0	0	0	0	0
LOC107824516	0	0	0	0	0	0	0	0	0	0	0	0	0	0	0	0	0	0	0	0	0	0	0	0	0	0	0	0	0	0
LOC107805743	81.93111	70.69757	86.89087	45.17779	44.51863	69.1026	45.17246	42.98723	44.73737	29.89691	30.71951	37.94801	27.55121	35.14058	26.23605	49.05333	76.29743	59.92645	36.05896	37.49761	48.71452	35.7847	48.67871	51.81304	16.06561	17.0642	28.20381	10.5667	12.80436	9.136021
LOC107789969	0	0	0	0	0	0	0	0	0	0	0	0	0	0	0	0	0	0	0	0	0	0	0	0	0	0	0	0	0	0
LOC107820610	0.603851	0.48744	0.539912	0.515798	0.534327	0.575018	0.839303	0.810773	0.502131	0.302827	0.496007	0.247787	0.403329	0.225865	0.389574	0.604273	0.616706	0.437334	0.29762	0.335679	0.298964	0.619558	0.760909	0.649178	0.15269	0.144037	0.224307	1.233825	0	1.066772
LOC107804900	1.245951	1.998533	1.754587	2.234961	3.064947	1.607459	2.319972	2.427229	1.778025	2.648611	2.586037	1.66313	2.413397	2.120462	2.787805	2.729742	1.179619	1.653458	2.857328	2.951168	1.4545	3.412925	2.307923	2.031537	2.929984	3.710009	1.001377	0	1.799535	4.62234
LOC107809702	15.12571	15.31691	19.78356	10.65799	8.055318	20.7792	8.105316	8.795374	12.22836	9.423275	8.691155	7.986636	6.236351	8.403492	6.684207	14.29835	14.3421	24.11994	7.461087	6.516129	10.15458	6.900745	12.9095	12.36938	3.364318	3.800029	4.552108	5.007879	4.382717	0
LOC107813663	3.623469	4.974418	4.625029	7.479522	9.375335	8.333779	9.079007	7.485241	8.483149	11.22618	9.809334	10.30736	16.03266	16.01226	13.93459	5.468786	6.026307	5.060944	4.049184	4.190407	6.692991	4.36671	4.814437	5.992482	3.885293	2.106065	4.011595	19.6807	18.9904	0
LOC107823210	6.235686	6.192574	5.79438	4.246652	2.130991	3.837534	3.029873	3.490719	5.733212	1.719337	2.7441	1.416932	2.297926	1.970474	1.015194	4.582746	4.486988	5.5467	1.396423	1.273266	2.353262	3.914667	5.007158	7.731147	0.815566	0.179514	1.321531	0	1.24224	5.318094
LOC107825464	6.338952	5.082175	7.167555	9.585675	9.826507	10.812	8.354896	8.103897	9.620502	7.993834	6.483505	6.56329	8.276926	8.319381	6.897473	6.46414	5.939781	7.355438	7.931596	8.265226	8.563177	7.682016	8.281961	8.119938	4.820669	5.305377	5.971172	3.408456	6.654295	2.94697
LOC107772560	0	0	0	0	0	0	0	0	0	0	0	0	0	0	0	0	0	0	0	0	0	0	0	0	0	0	0	0	0	0
LOC107763221	0.133622	0	0	0	0	0	0	0	0	0	0	0	0	0	0	0	0	0	0	0	0	0	0	0	0	0	0	0	0	0
LOC107826036	18.78941	18.57255	17.60631	15.61079	14.58575	16.95135	15.00157	14.32363	16.1191	12.57932	14.4861	13.66254	12.73035	13.73354	11.38547	0.278645	0.270928	0.418158	0.474283	0.395387	0.113673	0.130112	0.10994	0.130273	0.078791	0.086713	0.073657	0	0.30003	0
LOC107797950	0.127631	0.093056	0.088758	0.115627	0	0	0	0.087241	0.04793	0	0	0	0.113664	0	0.052399	0.26631	0.221944	0.323525	0.025902	0.043187	0.046561	0.08527	0.067547	0.02668	0	0	0	0	0	0
LOC107830391	12.19572	13.93203	11.82285	10.94687	12.88026	12.87019	8.01356	9.02777	9.392065	11.67315	12.57131	12.01258	14.26465	11.15897	12.40198	13.31828	12.65081	14.5814	13.28784	13.26438	10.04631	7.932067	10.0634	8.928746	9.379357	9.618629	10.76095	0	11.63468	6.950042
LOC107798044	0	0	0	0	0	0	0	0	0	0	0	0	0	0	0	0	0	0	0	0	0	0	0	0	0	0	0	0	0	0
LOC107763255	0	0	0	0	0	0	0	0	0	0	0	0	0	0	0	0	0	0	0	0	0	0	0	0	0	0	0	0	0	0
LOC107830129	2.103605	3.131388	4.845841	3.620943	4.436356	2.223939	4.999088	4.793006	5.134888	5.059825	6.023117	5.005735	4.917701	3.710136	7.376865	10.24171	2.303227	6.796082	4.624951	6.198609	4.028898	5.70952	4.94805	6.083035	3.280168	4.341735	2.237663	5.014583	4.89496	4.335635
LOC107776922	7.355033	5.344448	7.51677	0.360178	0.263847	0.534295	0.166729	0.084924	0	0.09692	0.209082	0.083969	0	0	0.102014	3.55523	4.609025	4.89068	0.403426	0.336316	0.453237	0	0.526024	0.20777	0	0.138297	0	0	0.47851	0
LOC107817598	31.6646	21.6303	23.76957	30.45597	28.4379	32.00708	21.84248	24.62772	27.78329	30.41527	26.06287	34.10145	21.41323	27.63562	20.59325	26.46336	21.67077	29.29953	36.6354	33.39497	33.19695	20.61144	26.4685	25.42502	33.89768	38.62367	55.12319	59.25707	32.48354	7.31914
LOC107828421	0.098706	0.179916	0.057202	0.089422	0.262024	0.176868	0.055192	0.084337	0.123559	0.064167	0.023071	2.751844	0.219761	0.107683	0.03377	0.220667	0.095358	0.171708	0.066773	0.166996	0.150035	0.054954	0.087065	0.068778	0.083196	0	0.077775	0	0.158401	0
LOC107780213	0	0	0.037881	0	0.03856	0	0	0	0.040912	0	0.061113	0	0.048511	0.047541	0	0	0.084198	0.064977	0	0	0.039743	0.036392	0.115314	0.136641	0	0	0	0	0	0
Novel02707	0.652707	1.784587	1.063846	6.061027	4.476077	2.719243	1.98452	2.509609	2.374563	1.272944	2.173974	0.75821	0.908254	0.979098	0.837401	1.641594	1.26114	1.338208	4.387885	4.693237	1.4882	1.703415	3.022588	2.473009	0.82522	0.79467	1.446464	0	0.392796	5.044734
LOC107831517	5.032565	4.820571	4.99966	2.977469	2.817296	3.533468	2.670447	2.588752	2.507034	2.003015	2.052487	2.212594	2.801161	1.792745	2.160987	3.099716	3.423161	4.326275	1.92804	2.51957	2.201223	2.744703	2.400714	3.005735	1.103732	0.643082	1.27459	3.672445	0.741691	0

LOC107762547	0	0	0	0	0	0	0	0	0	0	0	0	0	0	0	0	0	0	0	0	0	0	0	0	0	0	0	0	0	0
LOC107781703	7.759965	8.840314	9.14544	6.523154	6.503394	10.18798	5.69504	6.502818	5.534099	5.275034	5.702893	5.704836	6.645001	5.942298	5.016176	7.756449	7.8572	9.150918	4.845708	5.806966	3.67471	5.390072	6.548633	5.810078	1.981058	1.297767	2.998425	0	1.975729	0
LOC107795206	2.808937	4.091802	2.982136	1.981571	1.731723	1.906034	2.375287	2.30167	2.010289	0.965409	2.050351	1.478304	1.563474	1.381497	1.654189	3.139834	3.336478	3.398754	1.752243	1.071223	1.070915	2.576552	2.518285	2.454605	0.727736	0.736835	0.571465	1.646547	0.332538	2.847226
LOC107800825	19.2366	20.37279	21.5097	20.54412	19.75159	19.53561	18.23436	17.2919	17.8782	16.19088	18.81172	14.72334	18.16003	15.04803	15.56636	21.38836	17.53872	23.43321	17.19265	19.78428	12.93344	20.65113	19.4469	23.972	12.90581	14.24841	11.93121	5.775352	15.70747	17.97624
LOC107795352	3.095304	8.052141	4.049085	8.861781	9.067716	2.827041	18.41261	17.42172	7.617691	11.45787	21.87287	7.375313	8.480525	7.786368	31.46091	14.8139	5.835487	7.651194	16.14667	19.29492	12.96373	20.80524	14.67185	8.135143	19.28207	35.64683	9.057206	12.89464	11.35727	29.73007
LOC107792348	10.54354	12.4426	12.74913	35.2686	58.13069	32.40817	39.79494	50.93372	39.59496	69.66228	44.31142	64.86469	56.2032	55.964	48.62783	13.14546	17.56423	19.24853	92.10617	68.03679	99.61071	50.51693	46.85819	44.50427	168.1661	146.7997	172.6253	140.1689	124.6232	91.93785
LOC107761550	0	0	0	0	0	0	0	0	0	0	0	0	0	0	0	0	0	0	0	0	0	0	0	0	0	0	0	0	0	0
LOC107824533	4.128454	3.586206	4.654196	2.980448	1.712404	3.328945	3.516817	2.314898	3.875975	1.132246	1.944994	1.525927	1.795255	2.463092	1.324166	3.220741	3.240602	3.510773	1.505519	1.800763	2.235599	3.232294	3.755369	4.382482	0.489339	0.807812	0.533695	0	0	0
LOC107822791	29.12376	28.25064	32.17994	40.77375	44.82674	30.2877	37.36251	36.82747	37.92786	58.56616	30.50315	37.32211	40.69722	45.61445	31.97291	37.29355	38.82056	36.69222	48.41294	38.3584	61.58425	39.76701	36.14899	34.58232	108.0169	56.29701	152.466	94.47122	144.5013	89.27849
LOC107817972	8.019259	8.26564	8.160463	9.88853	9.348656	10.64025	8.674531	7.939471	9.560423	8.347315	8.278466	8.683692	9.564804	9.685969	8.296071	7.137953	8.177683	7.639447	7.943642	8.91039	5.978659	7.680396	8.673479	10.31085	5.19009	3.85224	6.807696	2.275756	4.596139	1.96763
LOC107817750	0	0	0	0	0	0	0	0	0	0	0	0	0	0	0	0	0	0	0	0	0	0	0	0	0	0	0	0	0	0
LOC107760504	7.770027	8.368953	11.66655	1.42754	1.346238	2.687214	0.364591	1.067799	0.459116	0.476859	0.800107	0.596758	1.088774	2.37111	0.836532	7.693317	9.553726	7.332253	1.488679	1.103145	1.684877	0.9983	1.917113	1.646963	0.618273	0	1.669736	0	1.569553	0
LOC107827149	14.49914	14.07726	14.78958	13.87509	13.29212	20.22272	11.33936	8.007853	9.902734	14.13849	11.70098	13.02175	12.43266	11.86338	8.881986	12.70291	14.22822	14.68077	12.92334	14.30948	11.70455	9.845061	11.20495	14.47186	5.367277	4.1803	10.42103	2.335345	8.33246	10.09576
LOC107777516	2.033004	7.738302	3.014717	7.186628	5.290988	4.457172	0.902736	1.668934	1.272449	0.233228	0.475181	0.572513	0.133128	0.478372	0.654626	3.000289	1.617456	1.604849	2.871953	1.38257	2.21769	0.932134	2.461316	4.749767	0.050399	0.055466	0.376917	0	0	0
LOC107780384	0	0	0	0	0	0	0	0	0	0	0	0	0	0	0	0	0	0	0	0	0	0	0	0	0	0	0	0	0	0
LOC107815816	0	0	0	0.051749	0.050544	0.184235	0.04791	0.048806	0	0	0	0.048257	0.190763	0.062316	0	0	0	0	0.289811	0.144961	0.20838	0.047703	0	0	0	0	0.067512	0	0	3.531864
LOC107781997	3.533612	4.397403	3.503458	3.445547	2.511464	2.090248	4.725404	4.07412	2.23832	3.653291	7.154687	2.541696	3.443932	2.18295	6.991426	7.804577	3.975872	5.385297	3.211258	4.297714	2.795607	4.349483	4.456261	2.773706	1.794201	2.626223	1.392148	0	1.844682	1.754928
LOC107801465	8.547664	7.23634	8.134597	5.027933	4.480768	3.832706	6.557695	6.368804	8.823613	5.332853	6.59015	3.114401	2.480312	3.491381	2.910503	8.573475	12.79757	7.369316	11.09889	10.69189	19.50751	15.83299	18.90261	15.15819	8.348417	11.16068	14.17244	0	1.950308	0
LOC107769052	0	0	0	0	0.101643	0.185246	0	0.294439	1.132352	0.056005	0.120818	0.048522	0.191809	0.313288	0.058949	0	0.055486	0	4.25444	2.089162	2.88094	0.527611	0.607926	0.960479	0.36307	0.479491	0.610941	0	1.10603	10.65368
LOC107772329	0.4105	0.224472	0.142735	8.404758	11.40565	5.296048	6.541772	4.770092	3.853952	1.921387	2.187604	0.832324	2.559054	0.716536	0.674121	0.672991	0.237946	0.428463	15.49554	10.13983	17.59598	3.496754	8.255604	5.234461	6.746953	3.76979	3.881421	0	12.64827	96.4509
LOC107765625	2.967558	3.777803	3.234781	2.901827	2.834295	2.582776	2.410003	2.29408	3.626319	2.388487	2.411148	3.183588	2.621847	2.929131	1.982201	3.299591	3.185457	2.844567	2.820081	2.550197	3.694537	2.674975	2.451387	2.264727	3.215911	2.752761	3.006356	0	4.98907	0
LOC107791421	1.646737	1.64471	2.058968	1.737097	1.397258	1.091369	1.608234	2.023791	2.287276	0.813887	0.838339	1.46744	0.979365	1.107437	0.810353	2.286197	1.939573	2.135883	1.213188	1.373943	1.398985	2.298334	2.606596	3.583803	0.285202	0.87886	1.066471	0	0.977422	0
LOC107772336	0	0	0	0	0	0	0	0	0	0	0	0	0	0	0	0	0	0	0	0	0	0	0	0	0	0	0	0	0	0
LOC107796875	0	0	0	0	0	0	0	0	0	0	0	0	0	0	0	0	0	0	0	0	0	0	0	0	0	0	0	0	0	0
LOC107784603	4.095216	4.158831	4.102343	2.720709	2.17423	4.067402	1.701061	1.532923	2.453329	2.928459	2.187861	3.295002	6.512645	8.041823	2.52193	4.824687	6.254758	3.89641	2.928668	2.903389	2.881213	0.944577	1.066474	1.793657	3.155869	1.08537	3.503394	5.578397	8.82518	0
LOC107813873	0	0	0	0	0	0	0	0	0	0	0	0	0	0	0	0	0	0	0	0	0	0	0	0	0	0	0	0	0	0
LOC107780676	48.20223	34.07046	36.77992	117.1294	227.48	117.1207	85.44057	109.8275	66.0714	133.1759	88.85496	178.9079	145.3574	193.868	114.3386	32.2485	42.84202	33.70074	230.3226	173.9766	331.4678	82.299	72.94123	61.01694	107.5293	100.4558	192.9423	191.5079	176.8834	99.34723
LOC107788810	9.316301	9.774649	8.912556	14.11355	13.00685	12.20501	15.14301	12.53855	13.96849	9.31486	13.15656	8.682505	13.50773	12.46466	15.73049	11.09346	9.558917	10.41855	10.53607	10.78119	7.486535	16.36989	13.03753	11.94193	4.756757	7.15002	3.792291	7.570991	6.194593	15.10597
LOC107826789	44.92143	28.20509	38.9002	30.91073	27.83978	41.10693	22.00253	24.09868	31.71115	26.3326	18.27419	33.67778	22.61774	30.11503	15.662	21.59298	34.20482	28.76121	18.96399	18.34754	28.45945	18.61434	25.10508	35.0347	14.19859	10.25844	26.85073	18.39228	20.22351	21.20275
LOC107831747	0	0	0.109982	0	0	0.136026	0.106119	0.216206	0.118784	0	0	0.213777	0.140845	0	0.129858	0	0	0.094327	0	0.107028	0.115389	0.211322	0	0.13224	0.159961	0.70418	0	0	0.609118	0
LOC107775144	2.091605	1.198208	1.090913	4.222893	5.525929	3.340972	5.16271	4.927356	3.955434	6.206147	5.740827	6.6138	8.382253	8.214617	5.857604	2.427066	1.9918	2.606393	4.366113	3.513431	5.368472	3.468554	2.925538	3.40414	5.855511	3.242923	4.802954	4.273695	14.09762	3.695059
LOC107804421	0.238947	0.332387	0.338898	1.025404	0.734463	0.486753	1.128655	1.289451	1.322391	1.275389	1.358037	1.189966	1.385997	1.207359	1.61348	0.909063	0.498135	0.721957	1.416519	1.340461	1.341953	1.52289	1.785976	1.643077	0.747305	0.804946	1.010754	0.899374	1.210923	0.777604
LOC107820389	0.104135	0.094906	0.090522	0	0.276436	0	0.262027	0.088976	0.097766	0	0	0.087976	0	0	0	0.155203	0.100603	0.155274	0	0.088091	0	0	0.183707	0	0.263316	0	0	0	0	0
LOC107785995	18.64903	24.46436	13.32512	93.33891	103.2618	37.0621	244.7578	222.6568	77.69411	174.1367	294.7957	197.3282	136.2792	193.8957	342.0703	18.00329	7.813989	6.662163	38.0267	52.13782	39.81472	73.53544	21.18519	10.15206	51.30904	115.4917	14.86142	131.3462	25.16647	111.3788
Novel02197	2.011009	3.239344	5.000457	6.185891	19.28452	4.27388	24.35937	28.37128	31.78905	9.668124	9.936431	18.29341	27.12451	28.67392	13.68029	6.517194	3.07234	6.101771	20.16904	20.4141	34.7621	15.11501	11.96312	13.63795	13.0674	13.34012	14.53748	33.44548	29.49545	8.675142
LOC107808054	7.029214	7.168386	6.911012	6.148521	5.254752	6.580032	6.29214	5.498552	5.370494	3.79354	5.574743	3.399265	4.735485	4.693667	5.199482	7.13476	6.673729	6.866963	3.812337	4.439169	2.641676	7.408698	6.381962	6.206891	1.532252	1.500822	1.833482	2.600111	0.991895	0.749357
LOC107815245	0	0	0	0	0	0	0	0	0	0	0	0	0	0	0	0	0	0	0	0	0	0	0	0	0	0	0	0	0	0
LOC107769759	0	0	0	0	0	0	0	0	0	0	0	0	0	0	0	0	0	0	0	0	0	0	0	0	0	0	0	0	0	0
LOC107777499	5.545245	5.950803	4.757762	6.241581	5.246663	6.245713	5.214798	5.332823	5.499106	4.541208	5.285435	3.934402	4.51621	4.268758	4.04071	6.010652	5.218026	5.995507	3.70257	5.015806	4.094051	5.252452	5.80177	5.770798	2.428009	2.10431	2.525147	1.716725	2.31141	0
LOC107827962	0.125011	0.45573	0.10867	0.113254	0.221237	0	0.104852	0.213626	0.234732	0.121902	0.087658	0	0.139164	0.409144	0.769849	0	0	0	0.126853	0	0	0.1044	0.330805	0.261324	0	0	0.147753	0	0	0
LOC107772674	0.198534	0.090469	0.172581	0.119908	0.146396	0.213448	0.138766	0.197905	0.279588	0.064532	0.092808	0.195681	0.14734	0.21659	0.169808	0.271237	0.447534	0.271361	0.302187	0.27991	0.271599	0.248701	0.291866	0.899198	0	0	0.039108	0	0.318604	0
LOC107761673	0	0.132421	0.063152	0	0	0	0	0.062073	0	0	0	0	0	0.158512	0	0.162414	0	0.162488	0	0	0.066257	0	0	0.075932	0	0	0	0	0	0
LOC107807717	0	0.021928	0.020915	0.326964	0.276774	0.284548	0.625599	0.534507	1.536057	0.187696	0.033743	0.426867	0.348199	0.157493	0.172865	0	0	0.053814	0.683619	0.508839	0.526646	1.044866	0.424459	0.377221	0.06084	0.100435	0.170626	0	0	0
LOC107777363	2.043359	3.088642	3.162589	3.882962	3.880797	4.447303	4.639944	3.789883	3.041359	3.401886	4.682825	1.599973	3.051421	3.370992	5.626762	4.828116	3.514784	4.533076	4.096347	3.710039	2.636281	5.410734	4.703766	3.958892	2.520416	2.149725	2.532895	10.69237	3.59907	6.16312
NitaMt019	1.415669	0	0.615306	0	0.626339	0.761009	0	0	0.664546	0	0	0	0.787971	0	1.453004	0.527479	2.051483	1.583162	3.591308	0	0	0	0.624357	0.739829	0	0	0.836604	0	0	0
LOC107762236	3.470295	3.06391	2.922383	2.881919	2.175111	3.264617	2.728768	2.409155	2.98656	2.220734	2.610806	2.626405	2.334005	2.287327	2.226135	2.963203	3.946286	2.5064	1.944115	1.712451	1.846231	2.596245	3.061029	4.042762	0.274219	0.251493	1.538103	0	0.696135	0
LOC107780109	0	0	0	0	0	0	0	0	0	0	0	0	0	0	0	0	0	0	0	0	0	0	0	0	0	0	0	0	0	0
LOC107791620	19.56484	21.45701	21.48124	31.6301	29.29519	28.4909	34.35053	28.63053	32.2474	21.8723	23.03543	19.7977	26.87943	25.04768	26.24368	22.69925	20.57631	21.75717	21.90873	21.95413	21.33892	34.11095	30.49037	28.49912	9.022147	12.01166	12.89944	10.44152	14.76145	13.54168
LOC107812989	0	0	0	0	0	0	0	0	0	0	0.116614	0	0	0	0	0	0	0	0	0	0	0	0	0	0	0	0	0	0	0
LOC107829212	19.38051	18.87475	19.99933	17.52126	18.43266	19.53685	22.13555	22.65276	20.57844	16.38389	13.7874	14.60312	15.11572	18.41787	20.63601	13.90192	19.38494	15.98101	17.90792	17.79204	17.19764	18.40601	18.3388	16.50842	13.04102	12.67036	17.04869	48.98476	19.0101	12.45661
LOC107803744	3.203789	4.219796	4.806962	12.96172	12.34939	8.422425	10.675	9.843407	7.025204	18.23104	14.67918	10.06651	8.989542	13.38222	19.4369	10.31844	4.070308	6.805763	11.82378	12.04733	7.805102	11.72852	9.852128	8.509113	7.962409	13.70938	6.665491	0	6.127415	5.427264
LOC107817860	6.356632	4.528377	5.935889	7.77059	7.344128	7.013218	6.612092	7.257517	9.876983	4.682724	4.788164	6.66006	6.674581	6.753076	6.410312	4.385317	4.98792	4.014817	6.112266	3.733566	7.240366	7.000848	7.002592	8.065582	1.789836	2.471906	2.034095	0	5.746432	6.86533
LOC107768184	48.78008	46.00934	40.60233	70.88582	78.09265	58.18362	69.80999	57.94255	54.56721	78.3019	73.53132	73.31303	73.92825	69.4422	56.66278	69.26051	48.82205	58.49191	67.54838	72.32444	64.79463	53.8621	55.85179	51.20516	52.32646	65.74828	36.65126	60.27586	47.59451	35.82895
LOC107802486	0.631312	0.802021	0.964533	0.48528	0.355489	0.267381	0.433234	0.555756	0.5029	0.205203	0.174388	0.210108	0.660192	0.229577	0.196352	0.270868	0.351155	0.62756	0.135887	0.161832	0.20937	0.527225	0.421863	0.55987	0.072561	0.159714	0.135665	1.368113	0.828916	0
LOC107823221	3.006918	2.029952	2.226613	9.416694	14.88031	6.984453	6.071557	5.645879	5.611201	7.927595	3.696299	2.446241	5.661555	4.414364	2.590899	2.904687	1.936623	2.795312	22.82759	15.67011	28.30386	9.765604	18.50061	15.32614	19.05522	14.04966	6.58136	2.654779	61.30118	135.4248
LOC107831656	0	0	0	0	0	0	0	0	0	0	0	0	0	0	0	0	0	0	0	0	0	0	0	0	0	0	0	0	0	0
LOC107797057	11.68221	10.54785	8.265776	15.40759	13.02966	14.77967	11.07444	11.74578	14.13057	13.29906	10.47759	15.5157	14.09357	15.35294	10.03839	9.272471	11.9684	10.57303	12.29539	13.05389	17.09655	10.75442	11.8861	15.277	11.74719	8.845712	14.38542	34.97204	16.4803	20.15801
LOC107763502	6.860105	8.661819	8.323834	4.790658	3.414528	4.686485	4.405303	3.083372	4.260168	2.160141	3.031497	1.569644	6.045772	4.989358	2.457024	17.28016	13.42747	15.42341	2.284131	3.052713	1.727063	3.222592	3.057049	3.435711	1.40037	0.397722	1.984793	0	4.300389	2.209222
LOC107797276	15.67883	15.10687	17.46038	8.90414	10.35348	12.07641	11.90742	10.96373	15.15938	5.058247	5.715788	4.613003	5.470622	6.510047	5.284045	13.19523	15.48618	15.76011	5.421993	5.410861	6.295984	13.68009	13.03851	16.99898	1.13414	0.596954	1.7056	0	2.816547	2.411556
LOC107825236	1.624519	1.400519	1.793825	1.352402	1.010122	0.991289	0.25778	0.637745	0.535871	0.214069	0.215508	0.111279	0.048877	0.335294	0.045064	1.603218	1.442172	1.440284	0.49008	0.148566	0.280301	0.696673	0.852014	1.101371	0	0	0	0	0	0
LOC107769796	1.219853	1.504787	1.049682	1.719084	1.864429	1.814892	2.728383	2.221416	1.60798	1.429816	2.134085	0.8536	1.495125	1.169491	2.124647	1.450785	0.880885	1.515754	1.737953	1.594773	1.123764	1.965431	1.749844	1.262112	1.090491	1.525889	0.684476	0.881179	0.889819	0
LOC107766145	9.744047	8.840314	7.952877	9.247024	8.349073	7.655569	9.374627	8.683465	8.589317	15.73581	13.89694	11.0071	12.29514	13.05937	13.01038	9.298375	8.859829	10.2066	12.92992	14.49016	8.665571	9.499896	9.800512	9.977099	15.88706	22.36171	16.57151	14.18899	17.19374	29.98814
LOC107779173	0	0	0.197111	0	0	0	0	0	0	0	0	0	0.123688	0	0	0	0	0	0.230092	0	0.103401	0	0	0	0	0	0	0	0	0
LOC107831109	0.079969	0.145764	0.347577	1.122943	0.495333	0.25793	0.871956	0.409967	0.262775	0.3899	0.112149	0.03378	1.513386	0.654318	1.354289	0.357558	0.231771	0.149051	0.405736	0.507363	0.656398	1.202117	1.058071	0.334335	0.859395	0.389449	0.708878	2.859461	4.427497	0
LOC107799526	4.212587	3.809492	4.343198	4.112234	4.941203	4.318393	4.190632	3.571447	4.23089	4.044117	3.984288	4.27621	5.271155	4.239467	3.016523	4.550659	3.533394	4.211896	3.048582	2.071836	3.067604	3.027152	3.773389	5.529342	1.981764	2.135588	3.203503	2.335345	3.930406	4.038304
LOC107791705	15.95114	12.41974	9.110731	8.574068	8.824313	6.141524	7.369565	6.618069	9.067188	11.0226	10.14962	6.789105	7.733335	5.043649	5.117744	14.35796	10.33578	13.6427	9.947459	7.535102	10.22092	6.32707	7.451313	9.917258	15.30128	7.611586	14.64752	3.462009	11.76971	11.97309
LOC107799170	0.251675	0.315384	0.437551	0.256505	0.111349	0.405871	0.395794	0.241917	0.236283	0.276091	0.463245	0.212622	0	0	0.161445	0.46887	0.303923	0.515994	0.287305	0.532248	0.40168	0.42036	0.443987	0.427456	0.039774	0.13132	0.074365	0	0.605825	1.945177
LOC107810706	0.913703	1.377203	1.46633	1.002873	1.088371	0.850104	1.311658	1.125999	1.17126	0.616829	1.269057	0.400805	0.743299	0.440894	0.991903	1.63676	0.96759	1.61131	0.838012	0.862114	0.560883	2.054384	1.596393	1.303945	0.577599	0.733471	0.498426	0	0.592158	0
LOC107788809	1.073738	3.191022	2.069664	0.042294	0.041309	0	0	0	0.087659	0.227615	0	0	0	0.05093	0	2.817924	2.706056	2.366748	0.047372	0.157967	0.042577	0	0	0	0	0	0	0	0	0
LOC107780547	0	0	0	0	0	0	0	0	0	0	0	0	0	0	0	0	0	0	0	0	0	0	0	0	0	0	0	0	0	0
LOC107806212	11.86512	12.34775	11.5927	8.217361	7.187358	14.51354	7.169124	5.822997	6.398306	10.23131	15.53952	7.787226	8.623677	8.13028	12.47997	20.82596	13.7363	15.94952	5.141005	8.308928	3.145001	5.67781	6.112269	5.312454	1.591028	1.137014	4.133683	1.168766	0.865499	2.021042
Novel00553	1.868037	2.860176	2.533198	2.301592	1.719081	1.285356	2.882918	2.872983	1.403036	1.165806	2.252974	0.694395	0.998172	1.141245	2.147378	3.28527	3.320613	2.56257	1.895555	2.275532	0.954062	3.806514	2.8341	1.796274	1.228119	0.831756	1.059777	0	0.719472	0
LOC107796809	2.172502	2.157482	2.44855	2.904757	2.306847	3.62437	2.639006	1.651417	1.659845	1.358739	2.468893	1.189839	1.317643	0.948037	1.445523	3.327223	1.925124	2.736721	2.919068	3.903713	1.844713	3.053051	2.431706	2.677858	0.473569	0.479491	0.56667	3.21444	0.504927	0.926407
LOC107807302	191.1683	138.4501	157.5573	95.44484	94.58992	113.2678	76.55926	73.5763	89.65498	90.16633	99.40075	106.917	81.84539	65.11277	65.03758	147.504	194.4381	175.3325	75.87026	66.96689	87.6889	66.06193	88.68558	118.7433	29.43286	17.353	54.3982	55.21158	20.01387	36.72018
LOC107780406	2.102317	2.192037	2.152732	4.164279	3.294882	3.524454	4.423203	3.74478	3.880589	2.64071	2.586002	2.047026	2.657661	3.24592	3.090352	2.615507	2.495739	2.058829	2.35023	3.285547	2.469805	3.913128	3.803056	4.301575	0.765855	0.89244	1.326614	1.274113	1.715475	1.101605
LOC107780663	0	0	0	0	0	0	0	0	0	0	0	0	0	0	0	0	0	0	0	0	0	0	0	0	0	0	0	0	0	0
LOC107809770	2.933645	2.997082	2.365064	7.887468	9.839231	6.791333	8.731071	10.06677	4.93097	13.24215	10.98212	8.254754	17.25066	11.64039	18.77027	2.521131	2.422739	3.157267	8.018318	7.7652	5.89049	7.488196	5.321416	3.610256	14.98563	16.36073	7.242251	18.61103	20.95762	5.851345
LOC107822133	3.587487	3.805179	3.363325	4.392602	3.911176	3.304109	5.3299	5.304027	3.839394	5.575714	7.186054	4.427257	4.68878	4.969021	8.545485	5.711769	3.205582	4.409022	7.852188	8.710714	5.494026	6.452146	5.119201	4.363916	8.065141	18.87656	5.137348	1.751236	8.134672	20.44073
LOC107826891	0	0	0	0	0	0	0	0	0	0	0	0	0	0	0	0	0	0	0	0	0	0	0	0	0	0	0	0	0	0
LOC107794211	1.778229	2.473603	2.522057	3.928543	3.202198	4.058411	2.904456	2.638901	3.924742	1.216841	0.89689	1.844926	0.868223	2.178201	0.608375	2.417796	2.833059	2.65149	2.310929	2.243194	2.532245	2.970091	4.072637	7.043132	0.197212	0.868167	0.405596	2.231027	1.652128	0
LOC107766228	0	0	0	0	0	0	0	0	0	0	0	0	0	0	0	0	0	0	0	0	0	0	0	0	0	0	0	0	0	0
LOC107780888	0.912085	1.187505	0.906122	1.613259	2.767108	2.568247	0.910721	0.964861	1.264071	2.668195	1.553205	2.60521	1.112046	1.32672	0.757827	0.906249	1.04899	0.971426	2.512129	2.277943	2.772791	1.233239	1.953833	1.498061	0.713857	0.966934	1.84802	0	2.091002	0
LOC107828073	9.350574	3.77075	4.100093	4.572919	3.953948	3.024799	2.012736	4.524961	3.107515	4.59934	2.553023	3.285684	5.066419	12.45788	16.98615	4.439825	3.037794	4.071703	7.557066	3.289976	6.565713	6.910538	5.036282	4.151455	4.917102	14.27716	1.662634	0	3.983804	5.116461
LOC107773385	22.68946	20.71708	17.72546	43.09778	70.28861	33.77972	49.46902	56.64603	39.35698	60.515	43.01282	71.38377	96.0329	85.69261	64.60428	26.41516	27.27768	33.70614	91.34588	71.79808	112.0047	46.8431	32.49421	41.32496	84.4062	125.8135	61.53482	134.2128	101.9257	125.1679
LOC107822972	7.999142	8.331693	8.177852	10.32852	8.395035	8.714342	7.912873	8.424145	8.882196	7.566945	8.012862	7.162024	7.366393	7.653957	6.05529	7.683908	8.292669	7.568549	8.57541	7.980687	8.531441	8.078493	8.884178	11.13834	2.822329	4.54823	5.622349	3.801008	1.663253	1.643186
LOC107765837	0	0	0	0.054171	0.10582	0.192858	0	0	0	0	0.209639	0.101032	0	0	0.429598	0	0	0.089158	0.060675	0.202327	0	0	0	0.187491	0	0	0	0	0	0
LOC107794017	26.553	19.73213	25.56771	13.32314	12.49095	13.37107	12.14444	12.10005	19.21886	5.798156	7.383937	8.35954	7.730859	6.536373	5.497241	17.82551	23.98612	21.31919	6.079705	5.375524	6.871771	13.91156	19.57797	28.18016	2.467617	1.136817	4.077174	0	2.840791	5.613032
LOC107822018	0	0	0	0	0	0	0	0	0	0	0	0	0	0	0	0	0	0	0	0	0	0	0	0	0	0	0	0	0	0
LOC107802949	5.497435	7.30464	5.940008	5.445855	6.001055	6.324696	6.442381	6.25556	4.558286	7.28954	7.673583	6.228685	7.149323	6.221634	6.011546	8.30825	7.073039	8.426968	7.82021	6.215089	5.341755	6.47896	5.959409	6.63197	8.509403	5.611854	7.22622	0	16.69622	12.70709
LOC107787479	3.300727	3.429917	2.76199	3.325651	3.684996	3.780842	4.683099	5.297817	4.836551	3.91048	4.217963	3.778871	4.326882	4.173041	4.147651	2.8505	3.278185	3.035793	3.912792	4.383996	3.798066	4.997809	4.952202	4.255975	2.925086	4.034727	2.880321	4.412124	4.15836	1.907373
LOC107782089	0	0	0	0	0	0	0	0	0	0	0	0	0	0	0	0	0	0	0	0	0	0	0	0	0	0	0	0	0	0
LOC107780800	0	0	0	0	0	0	0	0	0	0	0	0	0	0	0	0	0	0	0	0	0	0	0	0	0	0	0	0	0	0
LOC107811187	0.111843	0	0	0.101324	0.032989	0.120245	0	0.063708	0.140004	0.036354	0	0.031496	0.083003	0.122015	0	0	0.072033	0	0.03783	0.031537	0.136003	0.062269	0.098653	0.077932	0	0	0.088126	0	0	0
LOC107803865	0	0	0	0	0.068564	0	0.12998	0	0	0	0.075558	0.108665	0	0	0	0	0	0	0	0	0.070667	0.06471	0	0	0.195929	0	0	0	0.37304	0
LOC107784615	0.282371	0.19301	0.030682	0	0.031233	0	0.177627	0.211107	0.066276	0	0.02475	0.029819	0	0.038507	0.072454	0.157817	0.204595	0.263149	0	0.029858	0.032191	0.117908	0.155669	0.036892	0	0	0	0	0	0
LOC107768714	0.900512	0.729516	0.913262	0.271939	0.221342	0.32272	0.545493	1.068641	1.080284	0.195136	0.24556	0.126796	0.501231	0.436628	0	0.260969	0.966633	0.372983	0.152296	0.084641	0.319387	0.501361	0.220642	0.366028	0	0.069611	0	0	0	0
LOC107805987	0.086293	0	0.075013	0	0.076358	0	0.072378	0.073731	0	0	0	0	0	0	0	0.128611	0.083366	0.064335	0.087564	0.145996	0	0.288262	0.152232	0.27058	0	0	0	0	0	0
LOC107815750	0	0	0	0	0	0	0	0	0	0	0	0	0	0	0	0	0	0	0	0	0	0	0	0	0	0	0	0	0	0
LOC107806058	0.752416	0.263744	0.352186	0.524347	0.256072	0.871166	0.970899	0.54398	0.815079	0.338631	0.974021	0.146691	0.128862	0.441996	0.712855	0.517571	0.447322	0.64726	0.411116	0.587532	0.105572	1.160054	1.939991	0.725931	0.585405	1.691199	0.342037	0	0	0
LOC107772669	0	0	0.064585	0	0	0	0	0	0	0	0	0	0	0	0	0	0	0	0	0	0	0	0	0	0	0	0	0	0	0
LOC107786763	92.77624	92.89961	97.94426	27.24304	19.70669	24.79465	39.22378	46.97633	48.07969	17.16036	24.3625	17.63708	14.3048	18.70533	20.49886	70.79378	69.09711	66.97725	15.83069	17.56448	17.18389	49.06219	64.21803	38.44128	3.287376	7.288262	8.95226	5.389775	3.265573	11.65007
LOC107774520	0	0.014693	0.014014	0.043817	0.014266	0	0.094654	0.041325	0.045408	0.031442	0.033914	0.01362	0.017947	0.087941	0.264752	0.012014	0.03115	0	0	0.040914	0.014703	0.013464	0.028441	0.016851	0	0.089729	0.057164	0	0	0
NitaMp087	0.162418	0	0.352966	0.073571	0.215577	0	0.068113	0	0	0	0.056944	0.068608	0	0	0.166701	0.121034	0.156909	0.363268	0.247216	0.068697	0.222192	0.135639	0.143263	0.08488	0	0.112996	0.191965	0	1.954844	0
LOC107824766	0	0	0	0	0	0	0	0	0	0	0	0	0	0	0	0	0	0	0	0	0	0	0	0	0	0	0	0	0	0
LOC107808707	0	0	0	0	0	0	0	0	0	0	0	0	0	0	0	0	0	0	0	0	0	0	0	0	0	0	0	0	0	0
LOC107797292	5.366779	4.956373	6.002592	2.042052	1.96287	5.000618	1.110333	1.559077	1.511571	1.70954	1.555451	1.299748	0.995729	1.600337	0.844608	3.386099	2.8689	4.401278	1.488529	1.059316	0.652612	0.776869	1.325479	0.673123	0.588054	0.647182	0.592023	2.55867	1.205754	0
LOC107817858	9.972202	9.837994	10.59002	4.0049	3.684273	9.008133	3.319778	4.084579	3.667722	0.852117	1.333624	1.042242	2.117238	2.074896	2.004832	6.856712	7.846236	7.664639	2.920978	3.73958	3.234754	3.477174	6.166367	4.566753	1.169805	0.715237	1.154332	3.676049	1.484837	3.178331
LOC107812475	0	0.169885	0.324076	0.50662	0	0.200408	0.156346	0.159269	0	0.181768	0.130707	0.15748	0.207508	0	0.573962	0.277818	0	0.277945	0	0.630743	0.170004	0.311343	0.328843	0.584491	0	0	0	0	0.897419	0
LOC107817912	18.62392	19.67372	20.30571	44.02622	47.47267	30.54643	59.57603	57.5256	43.83656	49.55489	49.75072	40.31838	36.2817	31.74653	50.28568	26.10116	19.62793	21.20211	61.8757	70.02271	65.89051	64.48883	57.89302	46.39696	27.2254	37.43516	31.1732	39.72917	28.91095	27.80719
LOC107777016	0.635762	0.933509	0.828982	0.703962	3.156612	0.303788	0.236996	0.150892	0.232121	2.342028	1.089726	1.104059	0.786377	0.385325	0.253761	0.842259	0.580078	0.658317	4.408373	3.107354	8.600753	0.206477	0.436166	0.590666	2.32208	0.589745	3.631864	0	1.020262	0
LOC107759284	4.149894	3.41313	4.355294	5.226752	5.821621	6.529235	5.136147	4.799778	6.556857	4.392118	5.074596	5.515451	5.521129	4.582542	3.584077	3.281073	5.5248	3.697614	4.056964	4.666429	4.154019	4.353238	5.044321	6.347514	3.327193	2.535048	5.144102	0	1.949177	3.129198
LOC107762164	2.842034	2.590163	2.751971	0.130367	0.127333	0.425454	0.090522	0.061476	0.168875	0.070161	0.353161	0.182357	0.040048	0.196235	0	2.439591	1.807259	2.547992	0.29204	0.395622	0.196859	0.360525	0.253859	0.112804	0.13645	0.050057	0.425197	0	0.173197	0
LOC107799886	0	0	0	0	0	0	0	0	0	0	0	0	0	0	0	0	0	0	0	0	0	0	0	0	0	0	0	0	0	0
LOC107829651	1.442341	2.444539	1.8477	4.401476	4.47817	2.475668	6.048769	6.313222	3.420969	6.218055	9.581406	6.434693	9.690144	7.011841	8.908249	2.54566	1.686775	2.037461	6.521955	5.544061	4.015552	4.818147	3.660481	4.31102	11.67717	8.133277	2.512236	16.28649	17.0553	35.98577
LOC107824844	0.080091	0.364967	0.348109	0.145117	0.07087	0.086108	0.067176	0.136864	0.075193	0.078099	0	0	0	0	0	0.082204	0.298421	0	0.059712	0.406356	0.677518	0.292179	0.066886	0.494521	0.083711	0	0	0	0	0
LOC107822613	2.629446	3.13168	2.051955	1.299352	0.766756	1.092241	1.102715	1.276517	1.374584	0.233095	0.64951	0.50487	0.432418	0.325977	0.245345	1.781333	2.742333	2.138579	0.545764	0.606636	0.190758	1.17282	1.897646	2.061222	0.037777	0.041576	0.070632	0	0	0
107759138	0	0	0	0	0	0	0	0	0	0	0	0	0	0	0	0	0	0	0	0	0	0	0	0	0	0	0	0	0	0
LOC107794877	0.026834	0.097823	0.023326	0.02431	0	0.086549	0.045013	0.022928	0.075579	0.130832	0.112896	0.04534	0.029872	0.26347	0.110166	0.079987	0.025924	0	0.190604	0.068099	0.097892	0	0.047339	0	0.339262	0.186687	0.348871	0	0.258376	0
LOC107761400	0	0	0	0	0	0	0	0	0	0	0	0	0	0	0	0	0	0	0	0	0	0	0	0	0	0	0	0	0	0
LOC107800514	45.26267	47.73769	44.24186	52.324	48.29726	52.35115	50.84737	50.35449	49.36871	45.28517	51.30532	55.97452	59.50686	58.4285	43.56598	36.85857	44.32113	39.73846	42.60422	41.32105	45.85664	38.39868	45.75078	47.52259	25.82928	25.14913	36.37058	62.24848	41.16637	31.65904
LOC107824411	8.25486	9.174081	8.130824	10.3838	9.669191	12.51437	10.78405	10.9857	9.701472	8.744455	9.18218	8.178068	7.801095	7.061982	8.472562	7.78899	6.483183	6.707797	9.792626	11.30349	8.070195	9.423248	9.612407	9.093516	15.12939	9.378885	16.0387	12.74113	12.43719	12.85206
LOC107817313	7.727257	7.771837	9.427986	13.10094	12.55185	11.80887	10.6708	10.35159	8.835175	10.33378	9.907853	9.116167	5.898576	7.617159	10.25371	9.645254	7.838439	10.57555	14.28198	13.58705	17.31644	10.34822	11.31935	13.35511	12.17711	17.54866	11.70981	5.920801	9.831895	22.18301
LOC107790591	0	0.158246	0.050312	5.400775	9.884385	6.844874	4.757403	4.599101	4.999151	8.409343	6.087633	10.90405	4.832311	5.430234	2.257374	0.172524	0.279576	0.086301	18.61766	11.75063	19.31963	2.8518	1.684729	5.142013	21.07458	6.764795	26.06321	45.5302	18.39065	3.578696
LOC107773620	4.680997	4.428157	5.305271	5.636434	5.714986	6.816366	6.311665	5.974042	6.341757	5.257914	4.362574	4.054754	6.464218	5.106737	4.561184	5.651904	7.498899	6.096249	6.854361	5.012407	7.835778	7.917413	9.459996	10.40447	2.696903	4.287213	5.742659	0	5.420047	10.99113
LOC107825138	30.21507	23.72592	28.71917	24.81596	23.22079	30.91123	22.36119	20.3674	23.55757	19.67632	20.08716	23.1417	20.48411	20.64475	15.88154	21.7372	31.00838	22.83841	12.83696	14.06235	22.1216	21.47895	25.63727	34.96834	10.83905	5.382548	27.18544	7.476833	16.10697	12.92902
LOC107778859	0	0	0	0	0	0	0	0	0	0	0	0	0	0	0	0	0	0	0	0	0	0	0	0	0	0	0	0	0	0
LOC107820278	5.253382	5.152863	5.155899	6.657434	6.120802	4.91925	7.313579	6.384125	5.770998	3.455201	5.088008	3.396982	4.030242	3.378204	4.522262	6.256834	5.134742	5.995529	4.533499	4.587354	3.554741	7.307772	5.884013	7.229866	1.090747	1.371908	1.420258	1.101733	1.483381	1.905129
LOC107772921	0	0	0	0	0	0.126835	0	0.100799	0	0	0	0	0	0	0	0	0	0	0	0	0	0	0	0	0	0	0	0	0	0
LOC107817079	7.63623	8.717476	7.260326	8.179138	8.83342	8.081601	9.623931	9.718938	7.785334	7.795333	9.816636	8.248926	10.16106	9.198538	9.919484	6.461119	5.340814	5.796914	5.468496	7.755034	6.039414	6.676147	6.349762	6.339381	4.927826	6.087386	5.358837	2.844262	7.084638	7.377493
Novel00660	0.729185	0.830702	0.845153	0.440403	0.699	0.718632	1.121264	0.882633	0.513443	0.770301	1.150436	0.205345	0.947028	0.33146	0.810783	0.769801	1.350203	1.177883	0.739926	1.130873	0.387933	1.065682	0.696788	1.143215	0.768259	0.169101	0.646378	0	2.047823	0
LOC107760527	12.0037	9.388413	10.77605	7.078467	5.484639	15.48653	5.125536	4.326287	6.433916	5.363073	5.356272	5.789621	3.012677	4.142922	3.987285	6.277881	9.445941	7.972981	4.517866	5.317161	3.543032	3.681762	6.545342	6.022204	1.821158	0.850297	3.662929	0	1.681165	5.397866
LOC107765793	1.735024	0.517999	0.546079	0.162604	0.13235	0.064323	0.175632	0.127798	0.505526	0.08751	0.104879	0.050545	0.233106	0.228444	0.122812	0.334381	0.231197	0.312232	0.030355	0.050611	0.081847	0.024982	0.079159	0.25013	0	0	0	0	0	0
Novel04826	10.5515	11.17705	10.24484	5.84042	5.058287	5.117052	5.153698	4.131202	4.255652	6.728397	8.634781	5.169811	6.587895	6.620982	2.507131	9.214148	9.950351	10.73912	3.091978	3.621464	2.801966	3.00738	4.909006	4.974636	4.966796	1.331509	7.113544	1.80091	11.63884	1.557077
LOC107785527	0	0	0	0	0	0	0	0	0	0	0	0	0	0	0	0	0	0	0	0	0	0	0	0	0	0	0	0	0	0
LOC107792858	0	0	0	0	0	0	0	0	0	0	0	0	0	0	0	0	0	0	0	0	0	0	0	0	0	0	0	0	0	0
LOC107792669	12.73789	10.20187	13.48866	14.47731	14.31117	11.24633	14.82785	13.55503	17.64843	4.102712	6.198141	3.815379	4.640727	4.84269	4.912589	11.41952	12.2313	12.11541	7.872825	5.844828	10.70188	19.14795	20.97324	32.23515	1.464052	1.18159	2.14422	2.760423	3.902484	0
LOC107829076	0	0	0	0.023573	0	0	0	0	0	0	0	0.021983	0	0.056775	0	0	0.025138	0.0194	0	0	0	0	0	0	0	0	0	0	0	0
LOC107782125	5.003758	3.654715	4.380509	3.954451	3.328591	2.89967	3.065797	2.304442	2.865293	6.367308	3.757478	5.786323	4.306082	6.736458	2.986731	12.87893	7.02823	10.76822	5.473582	5.133438	4.854799	2.282023	2.410289	4.562276	5.114857	5.727902	5.326829	2.537868	2.221052	4.388509
LOC107803889	2.32495	2.065488	1.902146	2.212486	1.694221	2.037494	1.851715	2.03658	2.292821	1.143086	2.000147	1.188413	1.304958	1.513318	1.74458	2.096538	2.076225	2.476213	1.308463	1.867584	0.784011	2.431112	2.326492	2.879288	0.61753	0.489328	0.99294	0	0.75248	0
LOC107816036	16.52624	12.12588	12.3774	20.4278	23.29846	21.78303	15.62329	14.35979	15.16492	33.23184	28.90505	40.07122	48.5396	41.86467	29.18052	9.393079	13.71069	11.65971	17.38552	16.31007	17.45651	8.889096	10.54173	13.27208	24.19948	12.34198	28.52654	23.3701	25.62204	20.20592
LOC107771020	1.594937	2.561085	2.068664	2.11006	1.88174	2.204682	2.802889	2.920195	3.256245	2.764916	2.804803	2.224357	2.508255	2.899997	2.650381	2.395964	2.127831	1.981822	1.6955	2.141599	1.824034	2.621662	2.233079	2.990066	1.664401	1.268136	1.735477	0	1.950119	3.13071

LOC107807648	11.34937	11.84765	11.56524	8.648804	7.032116	8.380319	8.34794	7.853222	8.652954	4.456538	5.358868	4.719056	5.879038	4.930483	5.003462	9.63065	10.69463	11.41442	5.358935	5.605829	5.557486	8.099901	8.555173	10.37625	1.926048	2.967591	2.52076	0	3.667108	4.709723
LOC107763893	9.080133	8.491881	8.925473	6.372362	5.933068	7.326601	5.761726	4.93285	5.128619	4.168814	5.09872	3.179591	4.738822	4.584256	3.675335	7.665099	6.424689	8.485867	3.689264	4.203778	2.482704	5.99626	5.946526	5.900565	1.339724	1.220218	2.202541	1.306555	1.143451	2.259309
LOC107824509	0.054505	0.248375	0.189522	0.148138	0.09646	0	0.777174	0.582137	0.076758	1.275593	1.700758	0.092095	0.606763	1.456838	2.825118	0.182779	0.289613	0.162545	0.138271	0.092216	0.024855	0.318633	0.120194	0	0.068911	0.151681	0	0	0.393613	0
LOC107793584	5.280408	2.621062	2.950809	3.28885	4.088396	6.133279	1.66084	1.933596	2.390213	3.448034	2.314141	4.14239	2.466755	3.291809	1.935595	2.002617	3.825989	3.022876	5.788762	4.307331	4.471834	2.04741	3.326906	3.350871	2.741945	0.918413	3.009062	3.371641	3.17772	2.915139
LOC107785092	4.903898	4.497232	5.221997	8.996434	10.84823	8.040242	8.508997	10.05606	8.574942	10.96853	9.971987	10.35737	9.178091	9.964211	13.33805	4.956267	4.26382	4.524379	14.6485	15.81562	12.57873	10.92953	9.408098	7.303913	22.55257	24.13784	15.68546	24.11049	14.60814	22.74116
LOC107829038	5.784913	10.19299	6.826845	6.304888	5.692806	7.87802	7.248678	6.111082	3.390343	6.752131	11.06287	3.391459	3.883422	2.94516	9.140115	9.444843	6.638713	6.547764	3.735538	6.332072	2.158338	8.183645	4.638797	1.484114	3.235842	8.707863	1.678247	5.014583	0.590771	0
Novel03848	0.045912	0.057534	0.014966	0.088387	0.126956	0.00617	0.154033	0.112782	0.032328	0.145502	0.245475	0.033939	0.127774	0.144002	0.294517	0.183898	0.033266	0.085573	0.128118	0.150499	0.02617	0.249225	0.080995	0.005998	0.275721	0.439196	0.020349	0	0.165777	1.064551
LOC107796920	0.422758	0.192646	0.146998	6.58756	7.743547	3.49978	9.254685	12.06462	7.303037	7.873837	5.24696	8.464645	9.835969	9.869864	13.53792	0.252032	0.20421	0.189111	18.18901	16.84411	14.18871	17.22916	6.898665	4.330296	38.4302	30.64717	37.72481	63.48972	30.9367	73.1914
LOC107791056	0	0	0	0	0	0	0	0	0	0	0	0	0	0	0	0	0	0	0	0	0	0	0	0	0	0	0	0	0	0
LOC107831762	0	0.055252	0.0527	0.164769	0.053645	0	0.050849	0.103599	0	0.118234	0.08502	0	0.877348	0.066139	0.062224	0.090355	0	0.090397	0	0	0	0	0.2139	0.063365	0.229944	0	0.214961	0	1.751214	0
LOC107802714	1.309494	1.009836	1.313441	1.870759	0.980461	1.001751	1.161694	1.183417	1.371266	0.859467	1.324353	1.233947	1.037243	1.098918	0.904635	0.938304	1.411052	0.863635	0.383303	0.426055	0.780876	1.34596	1.288337	2.026703	0.063677	0.070079	0.654803	0	0.484952	1.557077
LOC107794478	269.7067	275.446	210.7176	1558.374	1349.567	1694.999	978.1959	990.4792	868.8159	2414.631	1525.783	2489.463	1240.913	1362.537	1208.591	217.5547	293.7786	179.2969	1640.189	1890.575	1629.692	850.0443	757.8929	867.8503	1009.441	1477.798	1437.589	506.6016	820.0038	265.364
LOC107825920	0	0	0	0.166503	0	0	0	0	0	0	0	0	0	0	0	0	0	0	0	0	0	0	0	0	0	0	0	0	0	0
Novel01967	0.069561	0.063397	0.181405	0	0.061553	0.074787	0.116688	0.11887	0	0	0.097553	0	0	0.151776	0	0.933071	0.134404	0.207444	0.141172	0	0.126882	0	0.061358	0	0	0	0.082216	0	0	0
LOC107765696	11.84238	11.20798	13.06588	6.395894	5.844012	14.93562	4.106796	4.086298	6.200506	3.775248	5.269773	4.713787	2.408452	4.844796	1.986825	5.939874	10.67069	7.640479	5.084043	8.380308	8.827291	7.797791	8.838723	9.045208	0.719825	1.425964	1.884177	0	0	0
LOC107785446	0.128068	0.116718	0.111327	0.232046	0.113323	0	0.107416	0	0.240472	0.749294	0	0.108195	0.142567	0.279432	0.131445	0.190873	0.371173	0.4774	0	0	0	0	0	0	0	0	0	0	0	0
LOC107791091	0.358765	0	0.207911	8.125584	12.69837	7.457191	13.54104	22.17299	15.71849	28.33717	37.98655	45.66632	34.08066	27.78908	34.12237	0.267352	0.115533	0.356633	9.343961	18.41178	14.83307	14.18175	14.76789	24.3738	20.26029	20.63343	34.91196	68.41815	33.39301	7.394337
LOC107825080	0	0	0	0	0	0	0	0	0	0	0	0	0	0	0	0	0	0	0	0	0	0	0	0	0	0	0	0	0	0
LOC107775061	11.73346	14.57398	11.61985	6.757701	4.833503	7.061509	12.00065	9.151164	11.97649	8.802475	12.89092	9.006526	18.83038	15.50119	14.43116	15.88886	17.99971	16.00686	3.650598	3.392329	2.408127	11.46653	13.11543	12.29831	1.89867	1.377743	2.301582	0	2.701306	1.020391
LOC107799615	0	0	0	0	0	0	0	0	0	0	0	0	0	0	0	0	0	0	0	0	0	0	0	0	0	0	0	0	0	0
LOC107818638	0	0	0	0	0	0	0	0	0	0	0	0	0	0	0	0	0	0	0	0	0	0	0	0	0	0	0	0	0	0
LOC107791787	182.5869	170.1259	190.2876	132.322	117.9837	161.1351	124.3735	134.4	155.1447	87.36835	96.9891	97.95431	79.49738	88.02913	87.22584	144.6766	175.7285	153.4385	86.23647	88.08117	101.1996	133.7105	165.6366	190.6182	45.31112	28.10554	68.86399	56.87085	38.54089	55.72696
LOC107826812	3.954563	4.842314	4.407743	5.648692	4.701456	5.868826	4.59884	5.555469	12.07203	3.785225	2.806966	5.001139	5.617626	6.114037	3.187322	4.754886	4.945478	4.232521	3.766632	3.509474	3.27473	5.024752	4.429782	7.88625	0.552122	0.742667	3.010843	0	1.518422	3.000203
Novel01289	5.983628	6.900572	5.821613	9.298867	8.797361	7.002217	8.628349	8.593058	7.735132	8.348243	8.972394	6.513348	8.531279	9.23941	5.149355	10.90742	7.248094	9.076519	7.613069	6.736005	6.758495	7.32265	9.256723	10.72816	3.54979	4.066824	5.032119	3.291646	5.318274	4.268962
LOC107801803	0	0	0	0	0	0	0	0	0	0	0	0	0	0	0	0	0	0	0	0	0	0	0	0	0	0	0	0	0	0
LOC107793698	0.828656	0.914211	0.796157	1.382905	1.041986	1.078465	2.048507	1.788697	0.737032	0.93563	2.385379	1.584372	1.359431	1.570145	3.043929	0.682517	0.674148	0.487735	1.106398	0.848562	1.272839	1.347641	0.423169	0.364678	0.165422	0.12137	0.360833	0	0.209971	0
Novel01801	0	0	0	0.494306	0.482803	0	2.654295	1.212106	0.717156	0	0.459109	0.092191	0	0	0	0	0	0	0.332196	0.646183	0	0.729062	0.77004	0.342171	0	0	0	0	0	0
LOC107826737	0	0.638631	0.304566	0	0	0.188343	0	0.449044	0.328939	0.170826	0.122839	0.148	0	0.382232	0	0.522187	0.169242	0.261213	0.177764	0.148193	0	0	0	0.183101	0	0	0	0	0.843394	0
LOC107828824	0	0	0	0	0	0	0	0	0	0	0	0	0	0	0	0	0	0	0	0	0	0	0	0	0	0	0	0	0	0
LOC107794009	4.420539	3.804221	3.615899	5.790524	4.604126	4.22282	4.145326	3.938016	4.612841	2.897277	4.156626	2.559115	2.307226	2.656378	2.87103	3.11058	2.814403	3.068782	3.559112	3.727211	2.128159	5.095767	4.832461	4.847576	1.979022	2.762844	2.980661	1.036495	1.674653	0
LOC107776417	6.24906	10.11066	7.263246	27.28889	21.80767	28.9877	21.67212	20.57756	13.31587	16.97998	23.33702	9.609657	10.16124	11.71983	32.06932	10.56938	6.308449	10.78361	31.06434	30.76731	18.37847	21.93049	19.13746	15.11789	12.78317	26.86692	8.049786	0	16.22573	30.39013
LOC107822026	0	0	0	0	0	0	0	0	0	0	0	0	0	0	0	0	0	0	0	0	0	0	0	0	0	0	0	0	0	0
LOC107772134	0	0	0	0	0	0	0	0	0	0	0	0	0	0	0	0	0	0	0	0	0	0	0	0	0	0	0	0	0	0
LOC107818284	0	0	0	0	0	0	0	0	0	0	0	0	0	0	0	0	0	0	0	0	0	0	0	0	0	0	0	0	0	0
LOC107802031	13.37988	10.4779	12.32008	11.3134	11.8394	13.2129	8.146547	10.3736	10.51454	15.26991	11.08464	16.2857	17.43228	18.21896	11.08792	9.342919	10.19725	8.608289	13.22498	12.49223	12.56424	6.621564	7.999093	9.426689	9.899132	7.929518	11.47975	24.80724	10.73592	9.192203
Novel02936	0.920587	1.924178	0.65649	0.554339	0.434126	0.207431	0.19419	0.169561	0.113858	2.838202	4.754402	1.578761	0.638205	0.721664	1.957626	1.984126	1.054456	1.376784	1.05721	1.478233	0.537942	0.313047	0.32578	0.305368	1.310261	2.477487	0.8926	0	0.875791	0
LOC107772934	1.530453	1.708654	1.629728	1.83713	1.455814	2.427001	0.962743	1.50381	0.969878	1.940101	1.260957	1.745509	1.703722	4.174123	4.16266	2.109917	0.739273	1.597425	1.125924	2.395118	0.663005	0.862732	1.04622	0.719833	0.096748	0.692092	0.452218	0	0.368407	0
107786837	0	0	0	0	0	0	0	0	0	0	0	0	0	0	0	0	0	0	0	0	0	0	0	0	0	0	0	0	0	0
LOC107813806	10.32364	8.753196	8.532768	9.735999	5.952747	12.82773	4.081029	3.868136	6.554209	2.640485	3.649138	3.753188	4.662906	3.046436	1.693607	5.17083	8.747251	4.763127	4.937324	2.863307	4.206029	2.332055	6.419075	7.517805	0.267463	0.47097	0.800112	0	0.611086	0
LOC107767671	8.887434	9.795107	8.983323	10.69975	11.41744	11.49149	12.92735	11.60494	9.979433	12.29415	13.72669	9.653595	15.05407	11.66067	12.6069	11.72764	8.471909	11.55691	8.149476	8.86693	5.978135	10.13453	9.063382	8.548481	11.12443	5.01222	7.991592	6.334657	16.77375	1.825659
LOC107766787	0.577506	0.289479	0.401611	0.680148	0.587667	1.303867	0.145314	0.468765	0.786172	0.28157	0.30371	0.439104	0.064289	0.315015	0.118547	0.129107	0.446335	0.430555	0.498111	0.757222	0.447691	0.241145	0.458458	1.056315	0.036507	0	0.068257	0	0	0
LOC107770443	25.07971	20.97147	22.15219	3.520147	2.277266	7.541172	1.904617	4.009813	3.695338	2.854007	1.875359	4.050027	3.089637	11.23056	22.89251	13.04876	18.33027	15.04868	2.406661	4.86638	2.071007	4.804222	3.916968	4.79962	11.29252	42.40954	7.037786	28.87009	2.672373	0
LOC107770658	3.64381	4.289474	5.279144	0	0	0	0	0	0	0	0	0	0	0	0	0.905124	0.146676	0.226385	0	0	0	0	0	0	0	0	0	0	0	0
LOC107772789	7.817875	14.01256	8.608156	25.6154	27.32519	10.22629	46.55604	47.53794	22.8756	27.31724	41.75379	25.64848	26.6891	27.71898	51.35394	29.51784	20.39236	19.91424	45.21832	47.06525	44.20621	79.32627	42.40968	23.96897	69.02446	102.7971	39.73243	27.95443	45.79294	40.28258
LOC107782562	1.687855	1.410082	1.675072	1.057635	1.033021	0.892203	2.288677	2.35551	0.990395	1.124679	2.751695	0.796154	1.205655	1.227578	3.984446	2.714733	1.141427	1.709282	1.313084	1.404012	1.629148	2.854383	1.910626	1.249603	1.422641	3.816356	0.881085	2.011758	1.21889	9.566572
LOC107797487	4.943054	3.331417	3.899701	3.386841	3.197754	4.867801	2.403956	2.448908	3.548814	1.903738	2.35926	3.193434	3.468074	3.126819	1.534815	3.126391	4.935924	4.800124	3.161263	2.84622	2.765503	2.28952	3.773869	3.950837	1.312925	0.69357	2.111081	2.970574	1.19988	0
LOC107783902	1.017981	1.096449	1.238873	0.75456	0.753377	0.497479	0.791728	0.869791	1.129494	0.956564	1.284856	0.938202	0.515105	0.827878	1.006834	2.289595	1.305314	2.401036	1.126884	1.487427	0.641451	1.344771	1.893809	1.837807	0.35101	0.36055	0.721903	3.970912	0.534646	1.144424
107779227	0	0	0	0	0	0	0	0	0	0	0	0	0	0	0	0	0	0	0	0	0	0	0	0	0	0	0	0	0	0
LOC107806824	4.781555	4.379807	3.904595	5.644527	5.299477	4.933049	6.542375	6.437742	6.52965	5.840049	5.01231	4.998483	5.968093	6.665453	6.246109	4.66098	5.319291	5.455304	3.773771	4.269581	3.369754	6.030138	7.029413	7.774169	3.511182	3.662612	3.453641	0	4.76679	4.479557
LOC107799779	3.201129	2.305714	2.087005	0.841953	0.639612	1.24897	0.757842	1.146991	1.35726	1.233498	1.122317	1.788395	1.149529	0.591434	0.317957	2.058443	2.81822	2.194111	0.838267	1.616026	0.941769	0.711455	1.571201	1.56498	0.130555	0.143682	0.305119	0	0.124285	3.192429
LOC107803672	53.59437	51.42756	50.59179	61.25387	54.76802	61.70127	60.06321	63.30518	67.94424	50.63274	44.89066	58.44668	44.61974	58.30318	43.68418	46.34988	48.20754	47.95579	55.92448	52.7435	66.00275	52.70377	67.2725	65.27361	28.0252	38.23697	39.05928	39.81143	37.27802	34.42117
LOC107772869	4.206084	1.379998	2.827507	4.623405	5.855663	10.79264	1.034828	2.347944	3.053782	3.71866	2.792012	6.348771	3.683386	5.078084	1.036082	1.588085	1.787909	1.881488	3.755878	3.795261	6.546806	1.873392	2.077625	5.392669	2.481625	1.794754	5.700369	20.05296	5.939878	0
LOC107766902	7.376219	7.245666	8.981776	2.522181	2.742848	3.394308	3.153558	3.531329	5.0389	0.783645	1.750903	1.309371	0.639012	1.095907	0.736454	6.673117	7.569692	7.746073	2.562915	2.063738	2.224961	5.009553	6.632894	10.1095	0.435445	0.039936	0.949832	0	0.276356	1.774641
LOC107794483	6.449855	7.380759	7.064972	8.411124	7.933853	6.219177	9.873438	8.501167	6.734265	7.332959	9.714554	5.742237	7.69523	6.563169	11.13221	7.716167	4.973705	6.792474	8.834103	7.976231	5.882376	8.937146	7.1689	5.774012	6.801575	10.58428	4.341469	4.136823	5.569847	8.941799
Novel00719	3.377359	0.839467	4.37711	0.222525	0.923721	3.366995	1.236103	3.672718	11.76083	0.479033	0.129175	1.815728	0.683586	1.607796	1.008416	0.228801	2.788222	1.007186	0.373867	0.259728	2.296155	8.564126	6.012272	0.449275	1.242184	0.683542	1.016086	4.391434	1.182531	0
LOC107761566	0.080646	0.03675	0.070104	0.219184	0.499528	0.867046	0.169104	0.068906	0.189286	0.07864	0.028275	0.408793	0.044888	0.439906	0.12416	0	0.038956	0	0.28642	0.102332	0.551631	0.235724	0.248974	0.126437	0.152942	0.224427	0.095317	0	0.58239	0
LOC107815151	11.40303	11.82881	10.678	16.23075	17.32963	15.15014	17.61214	17.01074	16.03674	17.4475	20.29831	17.62243	23.40135	21.16341	20.31505	12.0382	10.65803	12.44113	20.24926	19.48841	17.24837	16.54906	15.02601	15.67323	20.043	26.18614	17.39469	26.5196	26.44488	21.49593
Novel03133	1.806199	1.034709	0.852335	1.262307	0.913282	2.108332	0.346271	0.573213	0.678296	1.006441	1.194136	1.351535	0.804274	0.619292	0.582633	1.038328	1.695084	1.538969	1.309147	1.178681	0.98837	0.215486	0.910392	0.701197	0.391472	0	0.731925	3.690536	0	0
LOC107823246	1.019826	0.996635	1.228302	0.322812	0.282683	0.620875	0.133974	0.136479	0.069214	0.203684	0.129235	0.062283	0.095747	0.17426	0.176556	0.906477	0.581647	1.071782	0.124681	0.280638	0.100854	0.123135	0.151732	0.10274	0.062138	0.017097	0.014522	0	0	0
LOC107829678	61.15672	59.23843	51.4157	32.54039	28.04025	42.74759	29.53515	32.85825	27.46732	36.46876	50.69354	36.09238	25.97654	34.34008	57.30664	56.92161	65.58672	62.28241	34.05121	36.25879	35.65161	31.93938	41.38318	34.99996	52.27502	84.06514	41.61962	113.4355	18.66705	45.76925
LOC107791614	7.060943	7.912437	7.662759	9.917139	9.745288	10.74251	9.069712	7.949222	8.484445	7.967907	9.139364	8.422676	11.12312	10.00439	8.774075	8.769705	7.915473	8.392975	8.336585	8.471243	7.593984	8.047804	8.539318	10.97731	7.607753	5.715678	6.036029	1.587916	8.017427	4.118762
LOC107812215	3.637483	2.601092	1.167503	4.055847	2.574948	1.26347	0.891806	1.147556	0.262695	9.658769	9.22142	9.455529	10.15439	10.80603	8.787881	1.751501	4.216938	2.044354	2.668925	2.887703	5.81829	0.467347	1.184677	0.643398	1.202785	1.868785	2.976378	0	4.849516	0
LOC107759588	0.217066	0.2587	0.24675	0.272286	0.251174	0.269276	0.252087	0.199734	0.094058	0.113975	0.316122	0.028213	0.074351	0.109296	0.411306	1.057649	0.403277	0.609983	0.813282	0.903991	0.213196	1.756999	0.633313	0.261782	0.295548	0.557597	0.07894	0	0.080387	0
LOC107807060	201.081	208.4788	195.9148	143.0237	144.0625	81.16841	201.2112	254.7617	238.7971	370.3307	463.2284	360.3367	211.0853	204.9185	463.4571	273.7325	155.0398	223.9576	182.2332	261.4675	186.3804	289.3733	304.6551	257.0628	46.41715	147.3159	120.2797	70.59131	77.87526	86.62836
LOC107810330	0	0.026389	0.02517	0	0	0	0	0	0.027184	0	0.040607	0	0	0	0.029719	0.021577	0	0.043175	0	0	0	0	0	0	0	0	0	0	0	0
107831159	0	0	0	0	0	0	0	0	0	0	0	0	0	0	0	0	0	0	0	0	0	0	0	0	0	0	0	0	0	0
LOC107790342	0	0	0	0	0	0	0	0	0	0	0	0	0	0	0	0.035517	0	0	0.048363	0	0	0	0	0	0	0	0	0	0	0
LOC107808819	0	0	0	0	0	0	0	0	0	0	0	0	0	0	0	0	0	0	0	0	0	0	0	0	0	0	0	0	0	0
LOC107814114	0	0	0	0	0	0	0	0	0	0	0	0	0	0	0	0	0	0	0	0	0	0	0	0	0	0	0	0	0	0
LOC107795474	0.089845	0.409414	0.624804	0	0	0.096595	0	0.076766	0.337403	0.788494	0.314998	0.303615	0.400068	0.392067	0.276644	0.803433	0.086798	0.334917	0.091169	0.228009	0	0	0	0.093906	0.113592	0	0.424759	0	0	0
LOC107770216	2.928761	3.182513	2.611189	2.653309	2.126408	2.785456	1.637652	1.924933	2.079863	2.453155	2.237951	2.283968	2.925944	2.089126	2.119615	2.742136	1.886286	2.995335	1.676459	1.68345	2.465612	1.724662	2.583361	2.433214	1.281754	0.208982	1.508885	0	1.988475	0
LOC107795143	0	0	0	0	0	0	0	0	0	0	0	0	0	0	0	0	0	0	0	0	0	0	0	0	0	0	0	0	0	0
LOC107778062	18.39808	28.18125	25.92715	14.20351	11.64994	11.65691	18.50484	17.15623	16.91825	21.82694	28.0929	10.38874	10.53489	6.656123	20.45367	29.51791	20.01867	24.06734	13.89641	19.15878	13.33391	19.97098	20.77692	21.63832	14.83053	20.343	12.12276	9.455916	8.912056	21.02306
LOC107798487	0.037683	0	0	0.034139	0	0	0	0	0	0	0	0	0	0	0.038677	0	0.036405	0	0	0	0	0	0.033239	0	0	0.104867	0	0	0	0
LOC107766041	2.090629	4.503556	4.229445	0.929784	0.73997	1.185141	0.605755	0.389736	0.642363	0.481857	0.852917	0.256905	0.253889	0.414686	0.078028	5.240341	2.680724	3.712427	0.539999	0.450171	0.277337	0.539654	1.173501	0.91378	0.048058	0	0.044926	0	0.366001	0
LOC107808736	1.800156	2.526265	2.091066	0.663886	0.3947	0.496692	0.253872	0.245007	0.508515	0.093206	0.223411	0	0.159607	0.208554	0.114455	1.495808	0.969588	1.888431	0.290975	0.390809	0.232463	0.545466	0.449659	0.566122	0.060423	0.022166	0.075315	0	0	0
LOC107809195	0	0	0	0	0	0	0	0	0	0	0	0	0	0	0	0	0	0	0	0	0	0	0	0	0	0	0	0	0	0
LOC107802507	0	0	0	0	0	0	0	0	0	0	0	0	0	0	0	0	0	0	0	0	0	0	0	0	0	0	0	0	0	0
LOC107802831	3.64127	4.702784	3.7831	5.583273	5.246576	4.982486	5.120092	4.383963	4.41491	5.041231	6.123729	3.619113	5.473325	5.374486	5.536	5.223809	2.511351	4.275323	3.922136	5.221623	2.477355	5.366328	4.491424	4.10096	3.754331	4.280838	2.508567	3.481314	3.37483	3.009962
LOC107825902	11.95403	9.032295	12.61176	4.226994	3.435496	7.725872	1.2283	1.716857	1.886482	1.527657	1.623897	1.237211	0.568692	1.226101	0.559284	6.268703	9.377066	9.420056	2.522789	1.959075	1.832576	2.019378	4.986736	5.659833	0.043058	0.094776	0.764801	0	0	0
LOC107799378	0.043469	0.118849	0.075573	0.275663	0.19232	0.327139	0.364591	0.148563	0.204052	0.127162	0.274322	0.293788	0.38712	0.474222	0.892301	0.259143	0.251966	0.324077	0.132327	0.110315	0.039644	0.39932	0.268396	0.181734	0.439661	0.604835	0.513765	0	0.418547	5.375468
LOC107795606	0.358415	0.186658	0.356071	1.530755	1.495131	1.321165	1.374253	1.574945	0.961416	2.895855	3.554392	3.590328	2.051961	1.061321	1.208706	0.152623	0.197862	0.267213	1.454778	1.949106	2.568345	1.197286	1.038764	1.819559	1.553641	1.139904	1.815503	3.661675	2.465052	0
LOC107761585	0.753666	0.80633	0.797568	2.092878	2.160153	2.342774	0.989424	0.741945	0.892159	1.709486	1.792205	1.757894	1.404398	1.465683	1.210761	0.891286	0.918045	1.270359	1.679164	2.065105	1.270114	0.930426	0.794849	1.26722	0.414288	0.455944	0.464751	0	2.050843	0
LOC107778462	0	0	0	0	0	0	0	0	0	0	0	0	0	0	0	0	0	0	0	0	0	0	0.070733	0	0	0	0	0	0	0
LOC107790249	11.16478	7.951923	10.85298	6.656478	6.653947	6.356612	5.247906	5.370563	7.356255	4.310049	5.936971	5.722435	7.827902	7.295612	4.860597	9.111372	11.25749	10.18544	4.921962	3.374819	4.947266	6.208011	7.392386	10.34949	2.794106	1.238006	3.697559	0	2.901738	15.97177
LOC107809409	0	0	0	0	0	0	0	0	0	0	0	0	0	0	0	0	0	0	0	0	0	0	0	0	0	0	0	0	0	0
LOC107785448	0	0	0	0	0	0	0	0	0	0	0	0.027779	0	0	0	0	0	0.031766	0	0	0	0	0	0	0	0	0	0	0	0
LOC107773515	25.58081	25.97819	29.01381	11.25639	8.191927	9.42943	9.195313	8.950938	14.40984	2.375669	5.808263	4.528099	2.712087	2.923633	3.500726	19.78906	25.41931	21.61446	5.933175	6.388837	7.554519	13.83522	12.8937	10.94946	2.156128	1.694945	2.303578	0	0.158302	0
Novel03072	0.357541	0.5974	0.414404	0.3779	0.369105	0.3844	0.199923	0.101831	0.335675	0.116216	0.041785	0.302061	0.066337	0.06501	0	0.222033	0.402984	0.088854	0.120936	0.100818	0.217389	0	0.105125	0.124567	0	0	0.070431	0	0	0
LOC107825414	0	0	0	0	0	0	0	0	0	0	0	0	0	0	0	0	0	0	0	0	0	0	0	0	0	0	0	0	0	0
LOC107809478	17.45992	11.43368	13.75796	2.710043	2.1743	2.707435	2.534606	2.386395	5.086723	1.75614	2.825286	2.217751	4.230525	6.776662	2.647329	11.98755	10.58658	11.84512	1.23896	1.510557	1.364097	2.561913	3.109778	2.85541	0.308736	0.233598	0.324696	0	0.661298	0.943683
LOC107815128	3.463927	3.599505	3.376955	3.607398	2.835944	2.888823	2.932498	2.600087	3.768774	1.988778	2.610509	2.461477	2.234371	2.507544	1.661346	2.557198	2.908592	3.620334	1.806752	1.916982	2.096265	3.43353	3.826405	4.026533	0.122789	0.405404	0.880037	0	0.155856	0
LOC107794539	1.913876	1.606557	1.838817	1.003822	0.935894	0.703933	0.971599	0.860667	1.276696	0.736686	0.741638	0.808448	1.065277	1.538485	0.361854	0.975836	1.070453	1.276678	0.919927	0.170422	1.148347	0.546796	1.243911	0.789625	0.318384	0.070079	0.416693	0	0.242476	0
LOC107800966	1.377097	2.008087	1.392967	2.790043	2.193383	2.8265	2.541067	2.438831	2.209648	2.539195	3.388453	2.009526	2.508551	2.567644	2.518448	2.854742	1.693226	2.109369	2.464475	2.329859	1.255936	2.509199	2.34104	2.695494	1.58279	0.975484	1.568432	0	1.446509	0
LOC107804566	6.252024	6.253846	6.031249	6.285677	6.206861	5.246197	4.156704	4.820738	6.513908	5.87347	4.54431	4.637751	5.347194	6.2384	1.956374	6.761254	6.555589	8.867552	5.570449	5.288782	5.215199	3.947758	6.590737	7.172137	2.891875	1.273059	5.136527	0	8.809601	4.714293
LOC107772507	9.978719	9.715148	9.710467	9.780709	10.63798	10.03265	9.112275	7.856805	9.240564	8.402114	10.10159	8.20012	11.71505	10.73767	8.668898	11.95368	11.5157	11.47673	7.948513	10.16991	6.491668	9.67026	10.03355	11.14168	3.659971	5.212671	4.870578	4.871123	3.935108	8.423198
LOC107792286	0	0	0	0	0	0	0	0	0	0	0	0	0	0	0	0	0	0	0	0	0	0	0	0	0	0	0	0	0	0
LOC107804499	0	0	0.139885	0	0.284787	0	0.202457	0	0.15108	0	0.282095	0	0.179139	0.087778	0.33033	0.299796	0.155463	0.659854	0.408229	0.068064	0.073381	0.403168	0.141943	0.168195	0.406906	0.22391	0	0	0	0
LOC107813754	0.096375	0	0.083777	0.130966	0	0	0	0	0.090481	0.046989	0	0.732782	0	0.05257	0	0.035909	0	0.035926	0	0.040763	0	0.080485	0.042505	0	0	0	0.056954	0	0	0
LOC107789846	1.939146	1.850459	1.586502	1.880775	2.482985	1.667855	2.353562	1.734829	1.670628	0.956579	1.279746	1.580424	1.625363	2.090625	1.826381	2.652099	2.159895	1.887935	1.064879	1.505294	1.664503	1.752797	2.0123	1.907572	0.605707	0.317433	0.781947	1.631487	1.757316	1.410592
LOC107817997	0.752583	0.823064	0.785045	0.749982	0.599341	1.132766	0.504979	0.385816	0.706558	1.614499	0.844338	1.271606	1.508014	0.985237	0.695187	0.953403	1.526822	1.402706	1.832806	1.33693	1.647284	1.131303	1.593187	1.494539	0.951493	0.209433	1.334238	5.382031	0.72464	0
LOC107797948	11.07324	9.235608	9.392378	2.279952	1.306444	2.344941	1.013193	0.831445	1.417642	0.883457	0.870572	0.907149	0.933855	0.951786	0.619924	7.926718	8.558142	9.156175	0.817189	0.539323	1.162913	0.728595	1.095124	0.946943	0.042424	0	0.03966	0	0	0
LOC107820827	3.847262	2.441427	3.121387	1.549078	0.933033	1.899624	1.003913	1.290532	2.274209	0.361262	0.979166	0.312989	0.666217	0.497443	0.263248	2.123692	2.560448	2.677077	0.404853	0.337506	0.2859	0.880586	1.483102	2.412694	0	0	0.202096	0	0	1.762089
107779212	0	0	0	0	0	0	0	0	0	0	0	0	0	0	0	0	0	0	0	0	0	0	0	0	0	0	0	0	0	0
LOC107807563	2.274258	2.018161	2.081015	2.049519	1.652297	1.299767	1.756925	1.452277	1.629475	0.828715	1.275769	0.930341	1.145944	1.240552	1.08112	1.944537	1.561115	1.918656	0.753059	0.921431	0.927915	1.999255	1.573163	1.789046	0.348069	0.433032	0.481009	0	0.461014	0
LOC107777977	0.947061	1.084445	0.781042	1.165986	0.773559	1.279285	1.670153	1.058178	1.025934	1.326057	1.702769	0.964227	1.486807	1.483565	1.096658	1.284829	0.774181	1.122477	0.911729	1.396874	0.686559	1.338472	0.87821	0.888343	0.307019	0.574411	0.459221	0	0.70146	4.504478
LOC107790335	0.028654	0.026115	0.024908	0	0	0	0	0	0.026902	0	0	0	0.063796	0	0	0.021353	0	0	0	0.029076	0.169676	0	0.071789	0	0	0	0	0	0	0
LOC107826211	6.098841	6.999394	6.959705	4.7749	4.663777	4.398317	7.578313	6.626353	8.364941	2.6187	2.780612	1.95073	2.123407	1.560706	2.576002	6.172037	6.93461	6.380693	2.521315	2.632678	2.907017	10.14468	11.73324	16.21171	1.491387	0.523833	1.364542	0	0.724988	3.103708
LOC107811038	0	0	0	0	0	0	0	0	0	0	0	0	0	0	0	0	0	0	0	0	0	0	0	0	0	0	0	0	0	0
LOC107832185	5.22018	3.081595	4.073699	8.867285	8.555942	8.673604	7.463165	7.298613	6.905863	7.635502	7.528764	7.316845	4.820636	5.112521	4.505465	3.801673	3.323883	4.334123	11.73784	11.49141	8.27746	5.053067	7.848655	7.006168	9.824843	5.034926	9.465085	25.45341	11.70914	14.67144
LOC107771719	0.036502	0.033267	0.03173	0.132274	0.032299	0.235462	0.030615	0	0.102808	0.533905	0.025595	0.030838	0.162536	0.199107	0.037464	0	0	0	0.148157	0.185267	0.16645	0.09145	0.09659	0.190757	0.138447	0	0.474561	0	0	0
LOC107794802	0.146722	0	0.765254	3.788297	5.777404	4.96894	2.215117	5.139894	3.925849	17.88406	4.526789	7.375313	4.001659	6.88286	4.743631	0.710693	0.141746	0.656325	8.635235	6.019662	13.98345	5.391381	4.270811	6.747568	25.22817	24.39628	39.53831	20.9854	14.83381	13.60807
LOC107802508	1.363639	1.407275	1.220246	1.235387	1.543786	0.991759	3.195751	2.347394	1.863885	1.192837	1.771753	0.762379	1.562669	1.334521	2.366973	1.673716	0.891175	1.255862	2.075589	2.561547	1.737468	3.433178	3.130868	1.320475	2.763557	1.813695	1.327291	8.604656	2.799797	12.39939
LOC107766682	36.63283	49.349	37.1515	72.86725	80.30778	58.06339	102.3196	133.116	114.7081	84.77567	113.0458	97.5254	58.23115	63.95882	89.64224	39.24426	34.06952	35.99331	109.8669	131.3138	61.9607	143.2597	159.5875	86.70378	55.61381	159.5086	118.4122	40.4597	20.88216	30.60896
LOC107778822	0	0	0	0	0	0	0	0	0	0	0	0	0	0	0	0	0	0	0	0	0	0	0	0	0	0	0	0	0	0
LOC107777851	2.50389	1.656281	1.825517	7.061302	7.397267	8.032544	3.861512	4.58934	4.815271	7.324827	4.700823	10.74737	4.001218	7.313694	4.766794	1.595045	2.614046	1.926974	6.761739	5.431949	7.587411	3.406411	4.595306	4.812023	6.535593	16.80178	5.059613	31.76934	6.221738	2.497085
LOC107781523	0	0	0	0	0	0	0	0	0	0	0.0149	0	0	0	0	0	0	0	0	0	0	0	0	0.02221	0	0	0	0	0	0
LOC107830245	0	0	0	0	0	0	0	0	0	0	0	0	0	0	0	0.215654	0.199697	0.154109	0.104876	0.157375	0.094261	0.82861	0.200563	0.799389	0.156805	0.028762	0.146587	0	0.398066	0
LOC107826519	1.278256	1.178062	0.961341	2.459198	1.982576	1.945612	1.590123	1.521684	1.550669	2.254834	2.135039	2.37822	2.845944	1.613876	2.270145	1.840898	1.595661	1.670416	1.617714	1.968239	1.59805	1.751188	1.596245	1.96652	1.906639	1.998427	1.629622	0	2.074375	0.88805
LOC107773473	0	0	0	0	0	0	0	0	0	0	0	0	0	0	0	0	0.036537	0.028196	0	0	0	0	0	0	0	0	0	0	0	0
LOC107775580	6.32866	4.417884	4.882675	2.858009	2.672348	4.446438	1.871556	2.021604	2.907604	3.057485	2.373946	4.111541	4.068629	2.854039	0.848964	0.057339	0.241587	0.129072	0	0	0.017544	0	0	0	0.053531	0	0	0	0	0
LOC107759371	57.6963	56.19627	63.59002	130.5755	149.0703	96.14936	231.5417	261.3162	148.9186	151.36	185.1937	106.0224	85.90894	108.5671	227.4957	126.6443	76.72562	101.7487	280.0875	282.5804	215.0122	310.6612	217.3786	134.7582	392.0541	649.7916	225.091	339.7043	171.8694	578.3831
LOC107789687	13.85076	16.37179	15.34052	11.30601	8.70989	12.32119	8.934059	8.139947	9.736273	8.535667	9.810759	9.325564	9.353061	10.04809	7.43275	12.94129	14.36589	15.25361	6.313954	8.059008	5.738959	9.981786	10.0467	10.54529	2.044498	1.760922	3.6979	0	3.723387	0
LOC107812009	0.303553	0.276651	0.117277	0.397228	0.358138	0.471404	0.707231	0.432273	0.253324	0.263113	0.331102	0.370428	0.11264	0.404753	0.311559	0.276477	0.293258	0.075437	0.239575	0.228253	0.184564	0.169003	0.089251	0.387779	0	0.09386	0.239184	0	0.487137	0
LOC107772077	0	0	0	0	0	0	0	0	0	0	0	0	0	0	0	0	0	0	0	0	0	0	0	0	0	0	0	0	0	0
LOC107777526	0	0	0	0	0	0	0	0	0	0	0	0	0	0	0	0	0	0	0	0	0	0	0	0	0	0	0	0	0	0
Novel01540	1.605676	4.627432	1.584399	10.3792	11.40486	2.706089	37.5998	40.86137	25.6679	7.997954	24.67854	6.855925	9.468713	4.97108	15.41123	4.268779	1.635068	2.71774	11.71356	15.41845	9.657149	43.38129	52.36529	28.16742	13.66177	5.796795	15.48999	24.82779	7.939224	18.78296
LOC107793837	13.28363	13.52797	15.61493	9.93741	8.05877	10.33247	7.765324	8.770372	10.86511	5.053716	5.045348	7.906676	7.72984	7.191	4.338076	11.32383	13.7567	10.99139	5.718494	5.916469	8.306044	8.572254	10.29679	10.72858	2.099318	2.520436	4.341338	0	4.602614	3.111162
Novel02920	0.365702	0.205103	0.415711	2.0643	4.007625	1.300499	3.586381	1.826722	1.531811	0.493761	0.335332	0.45155	0.125263	0.153447	0.028873	0.272521	0.298945	0.104864	3.139984	3.164977	2.873457	2.114357	3.870879	0.999682	0.17783	0.156568	0.232739	0	0.135432	0
LOC107761478	1.758946	2.013148	1.351222	0.759699	1.520235	1.29737	1.149364	1.188332	1.171325	1.057038	1.47718	0.483816	1.366104	1.071027	1.02862	2.286224	1.837604	2.729476	1.245249	0.968896	0.317107	1.91304	1.154609	1.988091	0.310304	0.284587	0.338431	0	0.295402	1.264631
LOC107772279	67.02278	75.42423	60.09799	31.54761	29.78201	19.65922	41.61124	40.08626	42.54206	31.11499	38.15905	46.95992	36.08887	35.92347	56.67726	103.474	92.54976	84.34871	25.13416	36.11331	33.28704	26.58693	36.17797	41.49831	7.736872	9.021631	14.29324	5.209866	13.67847	4.504478
LOC107774142	1.982068	1.652204	1.197674	0.744539	0.44916	0.831596	0.851496	0.681544	1.225439	0.942813	1.542369	1.368194	0.834152	1.608569	0.372135	0.486342	1.237643	0.630732	0.220749	0.265817	0.220449	1.695649	0.596985	0.454754	0	0	0.142844	0	0	0
LOC107813560	1.968679	1.705968	2.019931	4.795046	5.425953	4.198443	3.058809	4.494772	3.181469	3.147066	2.308275	3.026469	4.203485	7.112161	5.995544	1.467062	2.276055	1.58804	8.088963	6.00624	9.919225	2.560476	3.985421	2.698574	12.97549	14.10052	10.68048	32.31208	8.234914	3.991028
LOC107788729	49.05858	40.04086	48.33716	85.79439	108.8195	103.5496	69.72002	83.83397	79.91169	110.2039	53.89541	123.4563	87.67609	93.99732	58.53343	26.87086	37.49408	29.85446	38.03404	33.71388	37.47272	24.32847	35.15401	49.29242	36.11124	29.17945	38.80507	40.71623	45.68384	5.867246
LOC107764812	0.610843	0	0	0.276696	0	0.164183	0	0.13048	0	0.446737	0	0.258028	0.17	0.832999	0.470214	0.341401	0.442594	0.113852	0.30992	0.129183	0.278549	0.382598	0.269402	0.159613	0.193073	0	0.180492	10.92097	0	9.442331
LOC107760805	12.67564	12.40475	13.31773	9.993267	9.703621	10.02151	10.19877	8.653308	10.35612	7.831369	9.046483	8.801328	7.432356	7.107781	6.157373	11.24856	13.49212	11.54227	9.753144	11.26841	8.736486	10.58576	14.02573	11.12476	6.524526	7.180553	11.39821	20.75926	2.639761	7.977142
LOC107764447	0	0	0	0	0	0	0	0	0	0	0	0	0	0	0	0	0	0	0	0	0	0	0	0	0	0	0	0	0	0
LOC107813085	12.06141	10.66232	12.67059	9.150902	8.636236	5.1091	9.741079	9.504454	13.08437	2.181885	3.526435	1.800321	1.968967	2.301558	2.908964	11.30668	9.552445	10.7558	3.935536	3.028489	3.595475	13.0982	15.82686	18.95442	0.673555	0.444768	1.158584	0	1.231122	5.270496
Novel02814	0.056021	0	0	0.203009	0.297427	0	0.281924	0.287196	0.052595	0.163883	0.471385	0.047328	0.124727	0.122233	0.229994	0.250482	0.324727	0.459427	0.341078	0.4739	0.45983	0.233924	0.049414	0	0.212483	0.155899	0	0	1.078823	0
LOC107772828	1.510955	0.344262	1.313441	0.798495	0.334248	0.947602	0.105609	0.32275	0.47285	0.122781	0.353161	0.531874	0.140168	0.27473	0.516934	0	0.243285	0.093873	0.127768	0.106514	0	0	0	0	0	0	0	0	0	0
LOC107768633	0	0	0	0	0	0	0	0	0	0	0	0	0	0	0	0	0	0	0	0	0	0	0	0	0	0	0	0	0	0

LOC107799275	10.50473	10.88524	13.50968	7.104975	4.902304	6.420489	7.543469	6.762384	10.47026	4.420115	5.600121	5.409914	3.844608	3.532237	2.953909	7.131113	10.00943	8.743637	4.161576	3.347572	5.118345	6.789889	10.34477	10.90434	0.545802	0.700794	3.231496	0	1.385576	4.448791
LOC107782653	0.32534	0.711618	0.565623	1.178967	3.569741	0.139912	1.910137	2.112644	3.237703	0.380697	0.091251	19.02006	1.810866	2.981413	0.801408	2.521416	1.382946	3.153213	6.206495	1.321034	9.910304	2.28228	5.222882	0.408054	1.56305	0.36215	1.230483	0	5.325431	4.023254
LOC107817837	0	0	0	0	0	0	0	0	0	0	0	0	0	0	0	4.169367	3.481875	4.657499	0.870763	0.929167	0.563488	1.605274	1.725778	2.367839	0.30378	0.191042	0.405692	0	0.330504	0
LOC107791132	0.178162	0.324746	0.232309	0.564922	0.275887	0.861959	0.074716	0.038057	0.041817	0	0.031232	0	0.099166	0.145775	0	0.033192	0	0.033207	0.316377	0.301426	0.974923	0.037197	0.078576	0.046554	0.112626	0	0	0	0	0
LOC107798531	0.255511	0.058217	0.333165	0.98379	2.487023	0.75544	1.553734	1.528209	1.43931	0.747467	0.582285	1.565005	1.493302	2.578437	0.327812	0.095204	0.185134	0.190494	3.176118	1.675122	2.563334	2.133839	1.746677	1.402065	3.391958	2.222031	1.283472	4.568163	7.073196	0
LOC107793928	0	0	0	0	0	0	0	0	0	0	0	0	0	0	0	0	0	0	0	0	0	0	0	0	0	0	0	0	0	0
LOC107827973	0	0	0	0	0.061066	0	0	0	0	0	0	0	0	0	0	0.051427	0	0	0	0	0	0.057633	0	0	0	0.096025	0	0	0	0
LOC107815826	5.450902	27.7629	4.787711	7.278762	5.300624	0.976731	3.595614	3.032161	0.826271	8.083712	22.71408	2.734262	1.991069	0.5575	11.59726	23.31429	1.398789	3.598226	4.119591	7.685147	5.178452	1.06692	2.779657	0.682487	3.84063	2.804697	1.610632	0	0.41004	0
LOC107827667	0	0	0	0	0	0	0	0	0	0	0	0	0	0	0	0	0	0	0	0	0	0	0	0	0	0	0	0	0	0
Novel04265	4.079421	5.403332	6.146667	3.449365	4.139168	3.216309	4.151524	3.903836	4.749125	3.129321	2.631653	3.49236	3.875215	2.017536	3.517085	3.364255	3.941063	5.231176	4.415482	5.567467	7.639427	5.269232	5.997198	4.491213	3.644724	5.449168	6.943028	31.11853	7.332186	10.08947
LOC107803762	15.84944	9.397184	11.67766	10.8892	10.11442	15.77317	14.5785	13.08911	14.65889	5.802885	6.858231	7.765234	8.329983	6.877819	6.289269	11.15242	13.31964	12.21178	7.114749	7.675692	11.0696	12.64582	13.92829	18.84441	6.331873	3.689221	6.406746	4.213604	6.807869	3.643104
LOC107772459	2.049331	1.724043	1.980762	1.674826	1.90215	1.14016	2.151583	1.824473	1.735639	1.481299	2.441882	1.150186	1.276276	1.422731	2.176922	2.402881	1.495253	2.382612	1.672341	1.563871	1.097889	1.926877	1.71916	1.797449	1.105242	1.355956	0.931594	0	1.310895	4.430533
LOC107815849	0.429342	0.391293	0.275857	0.253671	0.264285	0.240832	0.328794	0.350892	0.280407	0.109216	0.287966	0.299639	0.270146	0.305473	0.229912	0.333857	0.432815	0.33401	0.416725	0.536896	0.25537	0.265019	0.362243	0.195108	0.590021	0.233765	0.772204	0	1.168308	0
LOC107827926	0.456653	0.493254	0.396959	0.459671	0.344213	0.690977	0.5958	0.520235	0.508119	0.28037	0.569254	0.271484	0.376557	0.184513	0.243027	0.390713	0.441165	0.516986	0.326082	0.200302	0.262225	0.522608	0.462472	0.318196	0.085533	0	0.05997	0	0	0
LOC107804424	0.852236	2.529281	1.291705	1.445181	0.638097	0.399395	3.958933	3.528843	1.641266	2.365262	3.662146	1.476909	2.165031	1.191985	4.485703	2.42636	0.802221	1.335924	1.108706	1.682178	1.295423	2.153424	2.602138	0.730878	0.690696	0.699333	0.335758	0	0	0
LOC107804483	149.3036	143.6788	173.7643	70.48568	62.43934	102.0989	43.14737	52.2138	70.28631	26.9088	33.17529	26.29929	19.38922	26.15604	22.11638	108.9129	130.9102	133.092	44.03353	48.88474	45.44092	57.5781	85.41812	104.554	11.68341	5.569891	23.65616	9.136326	4.100404	5.266211
LOC107811789	1.321151	1.52279	1.474968	2.217778	1.902558	2.200226	4.095648	4.548511	2.359107	1.641937	2.570284	0.952006	1.759101	1.356528	1.927638	1.737394	1.50158	1.554714	1.761197	1.873629	1.216725	4.402495	3.644552	2.193142	1.244565	1.387725	0.857291	0.926284	1.558944	2.402611
LOC107767437	145.6537	101.2496	122.2253	546.7885	387.971	551.3445	72.32206	67.65311	144.5074	52.20018	69.32368	66.40644	16.85933	18.77686	6.850548	49.20842	83.69503	62.66906	157.7695	115.102	229.0918	49.43096	147.3038	263.6795	4.926102	2.781419	24.62731	16.96071	1.631144	10.47451
LOC107808711	14.95752	16.79372	17.92136	18.26518	17.46267	14.27738	17.00562	16.30315	17.13967	14.11408	17.00847	14.12601	15.47966	15.66644	16.19839	19.72864	18.07652	18.35964	14.68732	16.26131	15.69033	20.2095	18.4609	18.30855	8.880192	9.140002	11.76332	12.20161	7.803455	14.0661
LOC107790509	2.670033	3.387682	3.208445	2.608633	2.640576	2.33589	2.876184	2.907601	3.342326	1.761272	2.936829	1.968224	3.1763	2.37028	2.847917	3.16013	3.085256	3.786086	2.231242	1.860076	1.790526	3.213556	2.770759	3.201117	2.151203	2.513194	1.918209	0	3.15061	8.092752
LOC107774484	2.831964	3.481167	3.119132	4.285348	4.834286	3.642035	4.543471	3.876806	3.904846	4.138522	5.21063	2.431637	4.458283	3.813512	5.441059	4.192014	2.191714	3.584116	4.83906	5.039344	2.681325	5.490518	3.927341	3.774564	6.575565	6.098604	3.547801	3.862884	6.687016	6.202616
LOC107774217	14.2592	5.557	9.473789	7.698674	8.539103	16.20788	4.389289	4.430345	7.572539	7.677918	4.511135	11.11367	4.596379	7.228081	2.118909	3.148437	9.230108	6.228169	8.476936	6.498211	9.457914	3.528365	5.124191	6.021702	4.491816	2.338122	7.149855	30.90105	4.853961	2.968579
LOC107779106	0	0	0	0	0	0	0	0	0	0	0	0	0	0	0	0	0	0	0	0	0	0	0	0	0	0	0	0	0	0
LOC107766831	0	0	0	0	0	0	0	0	0	0	0	0	0	0	0	0	0	0	0	0	0	0	0	0	0	0	0	0	0	0
LOC107830052	0.075475	0.206359	0	0.341883	0.333927	0.243435	0.949564	0.515904	0.637735	0.66238	0.105846	0.127527	0.16804	0.247019	0.309863	0.112488	0.364577	0.22508	0.459522	0.127694	0.550677	0.315156	0.199722	0.552206	0	0	0.178411	0	0.726729	0
LOC107815568	2.018826	2.199894	1.96475	5.407343	5.961106	5.001552	5.595174	6.03729	4.820814	4.279588	4.954608	4.430749	6.620016	6.272166	7.747732	1.831482	1.844358	1.652361	5.633555	5.253304	4.422891	4.819688	3.387274	2.706403	9.488307	11.20566	4.020043	10.98503	5.916125	17.63861
LOC107824184	2.798777	3.260218	2.287909	3.99643	5.346707	4.005394	6.38943	5.891276	4.106736	3.343674	5.406634	3.053474	6.190009	5.621359	7.438291	2.762453	2.990355	3.123001	4.307028	4.264767	3.262508	7.166786	4.872017	3.990781	5.342908	5.880126	2.059241	5.302043	8.209513	16.04461
LOC107814303	14.03854	11.82925	14.30418	8.265602	8.721985	11.26877	9.565541	9.303593	10.32476	5.586948	6.111933	5.436856	9.310235	6.694913	6.91175	10.60318	13.82474	12.40978	5.620984	5.214254	5.547306	10.47672	10.34704	13.45268	2.197166	2.115825	1.989807	3.883767	5.098402	5.036889
LOC107788259	7.022113	4.977615	4.838123	9.377586	9.389482	9.171403	7.634859	6.222102	7.081015	6.746003	6.054586	8.569132	12.33367	11.68978	6.940367	4.457644	6.984958	6.670139	6.597736	4.972188	6.973555	5.125876	5.413987	7.828811	2.367489	2.098904	2.090263	3.719855	6.260547	0
LOC107804198	6.464063	4.522918	6.779139	5.327174	4.612765	4.797506	3.218034	4.560988	5.677215	4.351295	3.421386	3.558471	4.224681	4.731648	2.953439	4.817027	5.15702	4.228489	3.089211	3.280891	2.814547	3.935541	5.738516	6.32035	1.159974	0.870413	1.922323	0	1.606209	0
LOC107784647	0	0	0	0	0	0	0	0	0	0	0	0	0	0	0	0	0	0	0	0	0	0	0	0	0	0	0	0	0.578383	0
LOC107832319	0.850975	1.285795	1.109602	0.081152	0.158526	0.096306	0.281743	0.133939	0.105123	0.021837	0.078514	0.018919	0.149576	0.024431	0.022985	1.501929	1.016825	0.951657	0.045448	0.018944	0.061271	0.486249	0.25679	0.023406	0	0	0	0	0	0
LOC107772925	0.309025	0.328578	0.447715	0.04666	0	0	0.043199	0	0	0	0	0	0	0	0	0.191905	0	0.115196	0	0.043569	0	0	0	0	0	0	0	0	0	0
LOC107763200	2.508979	3.34199	3.590265	4.895709	4.986708	4.025439	5.115283	5.177956	4.747319	4.065072	3.193802	4.500191	4.683697	5.853339	4.397559	3.538037	2.759499	3.62599	5.444392	5.779523	6.231029	4.061685	4.289981	3.671338	3.660129	4.028148	4.744658	16.56254	3.530819	4.773352
LOC107818303	0.942365	0.658451	0.655342	0.825277	0.75048	0.827412	0.605975	0.751503	0.648804	0.643247	0.704839	0.398068	0.6644	0.651113	0.660932	0.386238	0.364161	0.679154	0.478123	0.31887	0.17189	0.524662	0.429468	0.738719	0.377288	0.327808	0.27845	0	0.378073	0
LOC107800054	9.371755	9.386863	10.88911	7.649708	5.829554	7.581777	8.327455	8.364251	9.539094	4.4336	4.749684	5.448194	4.13179	4.960219	3.666615	7.917327	9.995151	10.82646	4.048731	4.434892	5.712237	6.974196	9.739747	13.43225	1.525086	1.67843	3.893282	6.635772	2.456973	2.868662
LOC107772777	0	0	0	0	0	0	0	0	0	0	0.05066	0	0	0	0	0	0	0	0	0.073312	0	0.065891	0	0	0	0	0	0	0.347827	0
LOC107808822	0	0	0	0	0	0	0	0	0	0	0	0	0	0	0	0	0	0	0	0	0	0	0	0	0	0	0	0	0	0
LOC107788863	10.87271	10.58713	12.46924	4.389354	4.219689	5.085955	5.391652	5.622858	6.232118	1.692642	2.902452	1.144167	1.550117	1.539926	1.507505	11.41437	8.79015	11.80357	3.096948	3.791979	2.000616	8.777415	9.052039	11.94233	0.48233	0.106166	1.465427	1.364127	1.010169	0
LOC107807070	5.210346	3.903195	4.477774	1.394637	1.239933	2.227972	1.589142	1.854942	2.112326	1.327922	1.965139	1.167156	1.977351	2.325367	1.073603	4.353393	4.080293	4.473099	0.761022	0.834772	0.737988	2.933828	2.73315	2.413502	0.09981	0.109846	0.349898	0	0	0
LOC107823226	0	0	0	0	0	0	0	0	0	0	0	0	0	0	0	0	0	0	0	0	0	0	0	0	0	0	0	0	0	0
LOC107761309	0	0	0	0	0	0	0	0	0	0	0	0	0	0	0	0	0	0	0	0	0	0	0	0	0	0	0	0	0	0
LOC107826240	0	0	0	0	0	0	0	0	0	0	0	0	0	0	0	0	0	0	0	0	0	0	0	0	0	0	0	0	0	0
Novel01029	7.141022	8.755824	6.943484	10.53779	9.413126	9.438541	11.50042	9.120773	7.948404	11.53986	13.61518	8.334156	14.99748	13.01094	9.898399	10.79386	6.845486	8.644533	7.376946	8.407317	6.042741	8.776408	8.652806	7.48303	7.864966	6.683894	7.113188	1.316196	18.3416	15.93186
LOC107776084	0	0	0	0	0	0	0	0	0	0	0	0	0	0	0	0	0	0	0	0	0	0.047087	0.049733	0	0	0	0	0	0	6.972486
LOC107818477	2.491401	3.001284	2.930094	1.796294	2.036734	2.150263	0.997824	1.35531	1.303062	1.134855	2.212428	1.048761	2.21685	1.570607	1.442243	2.543985	2.423559	3.245708	1.907011	2.144016	0.919887	2.462203	1.672898	1.144323	3.062694	2.818864	0.713233	1.233013	1.826151	3.731244
LOC107760835	0	0	0	0	0	0	0	0	0	0	0	0	0	0	0	0	0	0	0	0	0	0	0	0	0	0	0	0	0	0
LOC107763361	0	0	0	0	0	0	0	0	0	0	0	0	0	0	0	0	0	0	0	0	0	0	0	0	0	0	0	0	0	0
LOC107787387	5.788826	6.361619	5.568211	5.498348	4.278947	4.897581	6.430677	5.568887	4.987387	4.360055	5.268021	3.185377	4.212916	3.501717	4.934459	6.987794	4.874812	5.548905	3.31396	5.122232	2.684495	6.613646	4.562133	3.662518	0.886058	1.44322	1.242481	2.00476	1.28213	1.733326
LOC107785172	5.06191	3.125682	3.571186	7.792594	7.15684	6.822433	4.799421	5.468973	4.838437	3.165479	4.513929	4.369408	6.47207	5.022087	4.141265	3.211787	3.029817	3.514073	3.238213	3.428725	2.843525	6.417582	6.762118	5.884953	0.486941	0.331749	1.257249	0	0.618076	0
LOC107778763	12.13677	6.89644	5.638185	6.232155	3.869668	3.116852	2.349147	2.435058	4.059593	3.593589	5.547208	4.815401	29.81134	20.79904	6.606671	4.17431	8.876931	3.919787	1.944562	1.163856	4.615794	3.200759	2.34046	2.362454	1.615218	1.572514	1.045362	0	2.365617	3.038198
LOC107777714	29.75362	30.9968	33.14109	17.37792	16.42323	18.36012	18.17509	20.1907	25.10468	10.68183	12.94729	14.14444	12.19449	12.83777	10.45765	27.2343	29.25284	27.17545	11.74669	11.16847	11.86646	21.65214	22.91133	29.44855	2.661053	3.72733	7.010661	0	3.45445	14.78867
LOC107790845	19.99099	18.06622	22.86853	12.32754	9.156838	12.02872	13.21658	12.20061	13.68991	7.043957	8.999597	6.954272	8.078855	7.037588	7.517595	16.70001	16.78119	19.11236	8.114191	8.27076	6.281655	15.20788	15.49959	18.08522	3.653149	1.963483	5.400628	4.805508	1.779296	0
LOC107786226	0.61878	1.043293	0.833734	4.204378	8.623717	2.927164	7.966616	10.60049	6.419368	4.495248	3.948392	5.35832	8.26602	13.80496	13.62288	2.075021	1.763492	2.075972	10.73701	8.401303	13.65698	11.21369	6.331341	5.820742	15.13802	29.48883	4.607497	53.10186	24.87489	36.34712
LOC107824409	1.246395	0.965545	1.408504	0.649273	0.937459	1.038521	0.784054	0.878587	1.053154	0.75962	1.463905	0.868716	0.72844	0.985823	1.183321	0.859154	1.234226	0.859548	0.442665	0.711696	0.880968	0.754651	0.961978	1.302732	0.35456	0.39021	0.368285	0	0.750074	1.926662
LOC107817932	2.416041	1.673462	2.142217	2.101258	1.83857	3.065096	2.877541	2.477197	3.49316	1.177972	1.626364	1.551265	2.850944	2.635782	1.339069	2.232541	2.61419	2.48574	1.520011	1.267159	1.674637	1.896637	3.239284	3.484838	0.54983	0.067235	1.256449	0	0	2.987749
LOC107819908	2010.918	1596.325	1990.354	511.3201	745.0892	584.3213	463.0254	441.8172	702.3009	314.8025	237.5149	389.8358	713.7082	668.3284	202.0921	4638.961	3841.358	4024.256	1377.81	1115.603	2064.998	663.211	1008.163	1248.813	204.68	116.7444	370.391	845.8469	941.5882	270.9816
LOC107825099	0	0	0	0	0	0	0	0	0	0	0	0	0	0	0	0	0	0	0	0	0	0	0	0	0	0	0	0	0	0
LOC107762869	0.040922	0.783197	0.071145	0	0	0.043996	0	0	0	0	0	0	0	0	0	1.402765	0.632541	0.549159	0	0.069234	0	0	0.036096	0	0	0	0	0	0	0
LOC107762089	0.368872	0.387901	0.332985	0.141383	0.301294	0.350823	0.404585	0.303052	0.306354	0.096841	0.169119	0.107873	0.063174	0.077389	0.131054	0.581484	0.233005	0.571173	0.403098	0.252032	0.219965	0.60426	0.325369	0.459688	0.035874	0.098703	0.067073	0	0.341515	0
LOC107828694	15.02472	19.50336	15.96637	18.35698	15.33788	16.89439	18.29585	15.89969	21.25586	11.4587	13.31416	10.10224	19.37269	19.88753	15.24412	18.77201	18.47685	18.34385	11.04989	10.23204	9.585673	15.68442	21.5206	19.34165	4.57468	4.027724	5.539196	4.928807	9.954269	2.130737
LOC107794174	0.626231	1.752964	0.66102	0.445762	0.079162	0	0.150071	0.114658	0.125986	0.043618	0.125461	0.03779	0	0	0	1.100004	0.993915	0.566928	0.04539	0.189196	0.203976	0.336203	0.3551	0.046753	0	0	0	0	0	0
LOC107780085	0.52511	0.119643	0.27388	0.356793	0.162628	0.649242	0.330325	0.224334	0.295798	0.076807	0.128873	0.243995	0.116912	0.08593	0.026948	0.332616	0.25365	0.391492	0.266423	0.421997	0.2634	0.285047	0.555819	0.356749	0.099585	0.036533	0	0	0	0
LOC107762303	0	0	0	0	0	0	0	0	0	0	0	0	0	0	0	0	0	0	0	0	0	0	0	0	0	0	0	0	0	0
LOC107800092	4.889234	2.49283	2.258798	3.809902	2.269047	5.991702	2.839023	3.271883	3.85194	6.36794	3.692052	7.62565	1.78888	3.394309	2.000252	1.452289	1.354265	1.427464	1.700011	2.227053	2.245123	3.16942	3.015823	2.32283	1.167132	2.71169	6.465657	0	0	0
LOC107802440	0	0	0	0	0	0	0	0	0	0	0	0	0	0	0	0	0	0	0	0	0	0	0	0	0	0	0	0	0	0
LOC107793133	0.443687	1.617465	0.664241	0.66993	0.283547	0.583022	0.413489	0.336976	0.161993	0.048072	0.086421	0.020824	0	0.026891	0	1.047015	1.095412	0.624821	0.275136	0.604697	0.40465	1.48214	1.174085	0.206108	0	0.240084	0.0874	0	0	0
LOC107808868	24.22072	50.2502	36.03239	0.445991	0.348489	0	0	0	0	0	0	0	0	0	0	26.7071	34.33792	24.00336	0.199817	0.249867	0.089796	0	0.434233	0	0	0	0	0	0	0
LOC107767502	2.307246	2.696682	2.449636	2.090248	2.135111	2.4427	1.684055	1.941276	1.868506	1.253737	1.704292	1.160607	1.64695	1.633227	1.626935	2.00811	2.126902	2.534266	1.197425	1.81768	1.14047	1.82388	2.097285	2.319489	0.645761	0.514639	0.499599	0	0.678345	0
LOC107789302	0.593875	0.61341	0.72274	0.071736	0	0	0.099622	0.101485	0	0.038607	0.305379	0	0.044074	0.086385	0.121907	0.23603	0.68848	0.442759	0	0	0	0.033064	0.104767	0	0.050056	0	0	0	0	0
LOC107825651	20.08519	18.30517	28.85916	27.17476	31.444	26.61779	23.26245	27.59336	28.44449	34.39422	24.60032	33.36401	34.39863	34.8618	27.30602	34.82122	18.74869	29.0216	35.71466	33.85387	40.51962	22.06068	23.30064	28.35832	35.58941	31.72229	34.42834	35.03354	60.59452	100.1906
LOC107761101	0	0.022497	0	0	0	0	0	0	0	0	0	0	0	0	0	0	0	0	0	0	0	0	0	0	0	0	0	0	0	0
LOC107793486	0.11891	0.108372	0.465146	0	0	0	0	0.0508	0	0	0.08338	0.050229	0.066186	0.064862	0	0.088612	0.057438	0.132978	0	0	0	0	0	0	0	0	0	0	0	0
LOC107830032	27.16061	26.68544	26.84771	37.67198	28.88928	40.57466	34.66134	27.55697	29.68441	34.13497	37.02044	29.27252	34.40371	31.74893	31.08956	27.28209	28.24902	27.64905	26.75661	30.0984	23.57846	29.71396	29.95119	26.75247	9.893516	10.50237	13.58057	9.917267	6.294833	9.799454
LOC107813407	0	0	0	0	0	0.041377	0	0	0	0.037529	0	0	0	0	0	0	0.037181	0	0	0	0	0	0	0	0	0	0	0	0	0
LOC107760745	0.116296	0.084792	0.020219	0.12643	0.432207	0.125033	0.195085	0.397466	0.196531	0.476294	0.424044	0.2358	0.129462	0.202997	0.214854	0.12133	0	0.017341	1.486916	1.200218	1.633386	0.135971	0.492388	0.145863	0.823388	0.711997	0.632282	0	1.455716	0
LOC107830874	2.405405	2.759186	3.821423	0.676296	1.06423	2.363164	1.182682	0.850445	1.44064	0.849259	0.668852	0.350371	0.507845	0.4072	0.34053	2.719671	3.205273	2.50448	0.841668	0.947238	1.286003	0.935139	1.097444	2.297393	2.516824	0.230824	4.264492	0	9.384178	0
LOC107785963	0.800246	1.060838	0.822117	1.31815	1.577155	0.664826	1.76953	1.243186	0.853757	0.851281	1.428339	0.122922	0.971831	0.952396	2.202714	1.355328	0.737964	1.247473	1.365695	2.061631	0.530791	2.582094	1.187145	0.608303	1.701587	2.530646	0.214961	0	0.525364	4.498222
Novel02528	0.67211	1.061745	0.973751	0.162373	0.039648	0.096347	0	0.076569	0	0	0	0.189272	0	0	0	0.367295	0.432876	0.734927	0.045467	0	0.122595	0	0	0.046833	0	0	0	0	0	0
LOC107775763	2.820823	4.432469	3.678122	19.65109	15.92308	27.71287	2.406738	1.246649	1.826423	1.707305	2.966944	1.396995	1.299385	1.22034	0.449258	2.645722	1.83243	1.958006	7.057238	5.800988	7.895337	2.152662	6.520675	9.353301	0.430425	0	1.60951	0	0.702437	0
LOC107793884	0	0	0	0	0	0	0	0.04539	0	0	0	0.03725	0	0	0	0	0	0	0	0	0	0	0	0.046858	0	0	0	0	0	0
LOC107767734	0.316631	0.432855	0.1966	0.491745	0.360226	0.607887	1.119195	1.043503	0.467134	0.727779	1.030815	0.534997	0.83084	1.110306	2.344506	0.387638	0.262193	0.2192	0.573741	0.727015	0.453785	1.133257	1.077258	0.472775	0.142971	0.440568	0.213847	0	0.326651	0
LOC107779062	0	0.050839	0.048491	0	0	0.059973	0.140362	0.047662	0	0.054395	0.039115	0	0	0.060856	0	0.041569	0	0	0.056604	0	0	0.093171	0.147612	0.058304	0.070526	0.077617	0	0	0	0
LOC107761442	18.01295	16.50299	20.06732	15.58877	14.51292	16.51217	12.96128	12.92013	16.06578	7.441971	8.110233	9.130704	9.076289	9.980763	6.178883	15.57806	17.95156	17.38756	9.379652	8.621339	13.66127	12.78662	16.55761	21.35393	3.416077	2.506371	4.986309	13.5598	6.618163	2.930968
LOC107797106	2.065594	3.006435	2.090373	0.949627	0.436483	1.557851	0.517165	0.210734	0.636775	0.541132	1.124128	1.041834	0.720722	0.874478	0.632856	2.297437	1.72748	2.20655	0.656962	0.886715	0.168704	0.437695	0.543878	0.354456	0.194891	0.085795	0.692328	0	0.148425	15.25
LOC107770939	2.198127	1.759014	2.796271	2.671379	2.229689	1.55629	2.518175	2.519455	2.768376	1.097865	1.315768	1.177634	1.313017	2.573516	1.210589	1.877761	2.071781	1.918592	0.816039	0.31747	0.831229	1.074564	2.175344	1.961265	0.338915	0.149197	0.253464	3.834077	0	0
LOC107780360	0.502846	1.782207	0.89851	0.177159	0.098878	0	20.549	11.71976	3.907887	1.416532	9.970624	0.330414	8.769806	5.638183	13.13206	0.915985	0.242896	0.333238	0.170084	0.165423	0.050956	19.34055	1.798814	0.087596	0.070639	0.310966	0.066036	0	0	0
LOC107771772	0	0	0.020409	0	0	0	0	0	0	0	0	0	0	0	0.024097	0	0	0	0.071472	0	0	0.019607	0.041419	0	0	0.065336	0.027749	0	0	0
LOC107789426	49.12616	51.84795	43.06837	120.618	122.9704	83.57149	145.174	159.9168	117.2552	119.3365	129.9962	105.4588	72.25979	79.81147	140.9494	51.1564	45.03254	46.52056	165.8283	167.8099	138.231	156.7348	165.2319	101.0435	104.8342	190.8574	87.38031	142.8323	59.70929	69.71414
LOC107820123	441.3133	396.2731	520.8931	82.01947	69.96269	170.2481	35.88646	48.33507	57.14819	37.42054	24.97553	54.77917	68.13049	75.18914	21.16492	272.9877	436.3598	331.2375	104.0648	101.3059	88.50248	37.84982	80.73264	55.20834	19.10038	13.96277	72.07472	55.20264	28.1374	13.63671
LOC107789246	0.277771	0.108495	0.034494	0.03595	0	0	0	0	0.037255	0	0	0	0	0	0	0.206996	0.115007	0.177506	0	0.033568	0	0.033139	0.070004	0	0	0	0	0	0	0
LOC107776107	0.711843	0.648757	0.536284	0.677138	0.587893	0.637765	0.607004	0.547396	0.490094	0.416482	0.856865	0.350808	0.343388	0.401236	0.450546	0.857587	0.596007	0.937586	0.373203	0.642315	0.183944	0.713374	0.732541	0.570414	0.119998	0.049524	0.224358	0	0.057118	0
LOC107800735	36.66583	38.98578	35.42242	47.19828	38.44978	41.53962	50.16884	48.46019	52.11115	29.87723	34.97127	30.23878	29.37439	32.10495	30.49356	35.31899	34.49727	35.54511	33.69111	34.47102	37.29237	51.85746	54.47414	53.71712	20.08081	21.43376	28.59115	20.13921	15.59145	17.41247
LOC107832657	0	0	0	0	0	0	0	0	0	0	0	38.14011	0	0.021564	0	0.01473	0	0	0.040114	0.016721	0.036054	0	0.017435	0	0.02499	0	0.023362	0	0.380641	0
LOC107793318	5.479881	2.924552	4.677714	5.00922	5.067381	5.307695	4.47199	3.458892	3.75428	3.851226	3.91173	5.255171	5.275918	5.870564	3.85094	3.71572	3.76782	3.312554	3.406496	3.090401	3.24178	3.174614	4.790071	4.850374	3.120827	1.992079	2.68407	0	2.852119	0
LOC107766993	2.952513	4.235599	3.220078	3.566436	3.773723	3.688668	4.058544	3.678948	3.105609	4.571861	5.52081	3.591423	5.523613	4.17543	4.896305	0	0	0	0	0	0	0.022831	0	0.028574	0	0	0	0.97753	0	0
LOC107789111	6.733752	6.338198	7.628752	6.750485	9.27958	8.485769	7.407066	5.94214	5.648294	7.535051	6.385921	7.227651	8.909307	8.309628	7.08129	7.280213	5.278949	6.666297	10.24942	8.404364	9.31262	5.346965	6.572525	6.115084	7.187635	6.067151	8.61111	11.8416	7.440353	3.412771
LOC107790340	0.962655	0.258042	0.393796	0.872117	0.450964	0.669688	0.664934	0.14515	0.42531	0.33131	0.635308	0.287039	0.693415	0.432439	0.406841	0.042198	0.054706	0.042218	0	0.095805	0.051645	0.094581	0.099897	0.059186	0.071593	0	0	0	0	0
LOC107831264	0	0	0	0	0	0	0	0	0	0	0	0	0	0	0	0	0	0	0	0	0	0	0	0	0	0	0	0	0	0
LOC107805391	4.09249	7.478343	3.8793	4.657775	4.203625	1.459272	23.47587	16.21852	6.989347	7.720691	14.75205	3.891802	7.486192	5.608927	22.41631	11.2181	5.284831	5.488959	2.10769	2.818283	2.588311	16.47044	10.24906	4.341954	4.862143	3.43381	2.041746	2.941416	7.920685	5.086328
LOC107821634	2.102054	3.518749	1.603524	4.197353	3.985791	2.675061	5.900932	5.828006	3.826176	4.894345	5.895847	4.530291	4.727828	2.99525	6.076199	2.269759	1.823541	2.238815	5.310783	6.241821	4.851459	5.194789	6.849006	4.438971	1.735599	3.521764	2.991492	3.06789	3.511034	0
LOC107768424	0	0	0	0	0	0	0	0	0	0	0	0	0	0	0	0	0	0	0	0	0	0	0	0	0	0	0	0	0	0
LOC107821739	0	0	0	0	0	0	0	0	0	0	0	0	0	0	0	0	0	0	0	0	0	0	0	0	0	0	0	0	0	0
LOC107779061	3.142786	4.667686	3.187283	1.739958	1.853962	2.878304	1.610883	1.74046	1.693855	2.099827	3.468824	1.96675	3.369015	2.603215	1.911508	6.592318	5.622584	4.772941	1.653598	2.560115	1.380058	0.923478	1.54008	1.946571	0.662239	1.376672	1.85726	4.162104	1.400971	0
LOC107782291	0	0	0	0	0	0	0	0	0	0	0	0	0	0	0	0	0	0	0	0	0	0	0	0	0	0	0	0	0	0
107762991	0	0	0	0	0	0	0	0	0	0	0	0	0	0	0	0	0	0	0	0	0	0	0	0	0	0	0	0	0	0
LOC107769977	0.351328	0.407517	0.249874	0.28935	0.452186	0.206029	0.884021	0.682236	0.539741	0.342589	0.716658	0.512674	1.102199	1.149844	0.491718	0.357013	0.431979	0.714354	0.486141	0.324217	0.436932	0.853536	0.338066	0.166913	0.282663	0.355525	0.339743	0	0.461295	0
LOC107810587	0.035368	0.0967	0.030745	0	0	0	0.029665	0	0	0	0	0	0	0	0	0.105425	0.341683	0.421892	0.179444	0.149594	0.064512	0.354439	0.218377	0.369664	0	0	0.041802	0	0.170273	0
LOC107766321	3.66715	4.668681	4.551618	5.514254	4.88413	5.32459	4.708823	4.037774	4.099515	3.96302	4.546356	3.673018	5.281768	4.021303	4.73393	4.817554	3.79844	4.340603	4.085615	4.381397	2.896271	3.772914	4.285103	4.36635	3.202457	3.524457	2.613971	0	5.278289	8.181536
LOC107763222	2.596485	2.019308	2.226975	2.007282	3.277825	3.647611	2.410081	2.041027	2.5027	2.194314	3.155806	2.661547	3.892464	3.588008	1.670042	2.167092	2.07363	2.942401	2.037527	2.196448	1.957578	3.122485	2.015439	3.292799	2.713737	0.626222	1.595795	0	3.833457	6.421784
LOC107812447	0	0	0	0	0	0	0	0	0	0	0	0	0	0	0	0	0	0	0	0	0	0	0	0	0	0	0	0	0	0
LOC107795573	8.349363	3.967302	6.823689	8.792458	8.335253	10.58777	8.200059	8.963133	9.647692	8.420031	8.106339	9.947629	7.785243	9.498007	5.713038	5.052015	7.44571	6.544025	7.67581	4.829392	6.117841	6.496	7.994151	12.23237	3.969828	2.879558	8.518767	0	2.748499	4.412424
LOC107822227	2.263475	2.197414	2.224233	5.349375	5.355504	6.083781	5.220803	4.855957	5.38192	5.853809	6.24509	7.919195	9.750267	7.703267	6.110943	2.585119	3.541515	3.631829	3.295434	3.954363	3.208682	4.479158	3.038205	4.191551	2.923932	2.259395	4.216419	1.759462	5.093244	1.52124
LOC107792332	0	0	0	0	0	0	0	0	0	0	0	0	0	0	0	0	0	0	0	0	0	0	0	0	0	0	0	0	0	0
LOC107829594	127.74	113.204	131.7422	68.76084	52.98274	88.1859	51.3307	52.62549	65.23122	277.2199	165.9492	198.2021	101.8377	81.94702	33.94647	99.90229	130.4608	121.5377	48.57706	75.06531	52.82094	48.28585	64.70067	72.00629	278.029	111.1988	650.8807	119.1397	96.48223	45.44508
LOC107832629	0	0	0	0	0	0	0	0	0	0	0	0	0	0	0	0	0	0	0	0	0	0	0	0	0	0	0	0	0	0
LOC107761561	0	0	0	0	0	0	0	0	0	0	0	0	0	0	0	0	0	0	0	0	0	0	0	0	0	0	0	0	0	0
LOC107807473	0	0	0	0	0	0	0	0	0	0	0	0	0	0	0	0	0	0	0	0	0	0	0	0	0	0	0	0	0	0
LOC107769501	0.206708	0	0	1.348321	2.560721	0.80005	2.704646	1.483581	0.931519	0.241879	0.666741	1.047795	2.163034	1.307947	1.569978	0	0.239637	0.030822	3.314094	2.413076	4.072054	2.382254	0.802252	1.123465	3.292903	3.50895	1.905634	14.78253	3.98066	2.556211
LOC107770399	0.648264	1.181626	1.239749	0.411106	1.032529	0.209089	0.326236	0.055389	0.060862	1.011423	0.50002	0.273836	0.649491	0.919392	0.731897	3.236685	1.878834	2.416543	1.249846	0.712903	1.537193	0.108277	0.171544	0.067757	0	0.451006	1.149295	0	4.057268	0
LOC107785971	2.284794	5.718578	3.557236	9.051985	9.686229	7.149321	14.8446	13.92757	12.19806	12.0044	14.61019	13.48299	14.50155	13.95111	14.63031	7.852434	8.664489	7.60179	11.0732	9.433114	10.76097	11.42007	11.91156	13.36604	14.40026	13.71294	8.947737	9.754931	24.9548	10.54271
LOC107823513	4.610349	3.601512	3.064695	2.702598	2.742548	4.206938	3.411969	2.615104	3.346325	2.531177	4.129265	2.552993	3.320903	3.043158	3.459482	3.724346	4.004843	3.610515	3.341617	3.605077	3.215371	4.723803	2.904738	3.927877	2.302164	2.210198	2.610055	0	1.492159	7.186508
LOC107831431	7.230977	8.44192	7.246777	12.07314	11.87149	14.64888	8.2453	8.98668	10.23924	13.2864	11.04166	11.73823	14.63568	11.89816	10.54982	7.036265	8.602265	7.841455	12.85578	12.25909	10.11017	7.735624	8.59212	9.2755	10.35103	7.691548	12.32523	8.54739	10.64514	12.93271
LOC107797252	0.068605	0.062525	0	0	0	0	0	0.029309	0.064409	0.066898	0.024053	0	0	0	0	0.025562	0	0	0	0.029017	0	0.028647	0.030257	0	0	0	0	0	0	0
LOC107772920	0.059119	0.2694	0.128478	0.080339	0.183094	0.127121	0.198344	0.101026	0.111008	0.288244	0.601091	0.17481	0.329063	0.193489	0.242714	0.704893	0.456914	0.705216	0.569907	0.600131	0.647014	0.12343	0.208589	0.370749	0.186862	0.246781	0.104812	2.113935	0.284622	0
LOC107772866	0	0	0	0	0	0	0	0	0	0	0	0.050923	0	0	0	2.919649	2.038106	3.415307	1.406768	1.37671	1.704152	1.711491	1.382351	1.76401	0.914485	0.922565	0.712413	0	2.321517	0
LOC107787756	1.231997	2.209408	0.898215	1.332152	1.652812	0.555456	1.399995	1.222435	1.193965	1.007586	1.616287	1.275851	1.725406	1.994395	1.917128	1.806556	1.535759	2.162934	1.532437	1.580087	0.978621	1.327578	1.928022	2.243061	1.959586	0.718873	1.40915	5.684209	2.869971	0
LOC107801249	0	0	0	0	0	0	0	0	0	0	0	0	0	0	0	0.169711	0	0.169788	0	0	0	0.19019	0	0	0	0	0	0	0	0
LOC107812278	0.185964	0.451954	1.185465	3.70642	4.881735	0.73309	8.9946	11.65211	9.718892	3.868545	7.519608	6.231921	3.795318	4.733801	7.507474	4.988892	1.676794	1.894799	41.32597	45.72549	48.7323	59.947	56.1537	27.60042	26.17609	36.39818	30.99096	62.06214	12.2357	76.65608
LOC107817878	0	0	0	0	0	0	0	0	0	0	0	0	0	0	0.050197	0	0	0	0	0	0	0	0.04314	0	0	0	0	0	0	0
LOC107811394	0.065886	0.030024	0	0	0	0	0	0.028148	0	0	0	0.027831	0	0.035939	0.033812	0.073648	0	0	0	0	0	0	0	0	0	0	0	0	0.158601	0
LOC107793524	1.320498	1.604628	2.040677	5.449831	4.803684	2.050673	7.629849	4.638441	2.479484	8.298205	8.642052	4.462374	5.716655	7.202993	10.08963	3.498798	1.984441	3.391012	6.699752	5.833487	1.605755	5.146318	2.717789	2.607015	6.678045	3.878907	5.028995	0	21.19116	27.21613
LOC107773963	1.875348	3.502908	2.663198	4.592266	3.565312	4.032456	4.09586	3.395074	2.335924	6.536248	8.111306	2.305929	3.472549	3.382845	5.336094	4.04033	1.84762	3.128537	3.372596	5.356114	1.134588	5.830427	2.948045	2.406475	6.596571	6.097234	4.191618	2.65572	5.274128	3.444225
LOC107829532	5.659812	6.162818	4.975235	7.239958	6.565023	7.04218	6.738433	6.484087	7.303824	4.712881	5.395599	4.441305	5.805031	5.573307	4.394882	5.923734	4.873968	6.416367	4.258992	4.967131	3.673222	5.80653	6.37597	8.419255	2.974851	2.949519	2.806058	3.031885	2.959559	5.242767
LOC107795858	0.094548	0.086169	0.041094	0.042828	0.041831	0.101651	0.118953	0.040392	0.155341	0.069147	0.099446	0.199693	0.026313	0.154721	0.072781	0.158529	0.091342	0.123357	0.047971	0.059986	0	0.03948	0.125097	0.074116	0	0	0.055874	0	0	0
LOC107811036	0	0	0	0	0	0	0	0	0	0	0	0	0	0	0	0	0	0	0	0	0	0	0	0	0	0	0	0	0	0
LOC107776645	7.8873	10.42699	9.593745	2.250962	1.866233	4.193314	2.859412	2.5426	3.417667	1.464974	2.086629	1.098364	1.640267	2.111766	1.364045	5.834566	7.787263	7.668106	1.641741	1.906318	1.159368	3.233126	4.714532	4.620154	0.328745	0.2412	0.409765	0	0.695463	0
LOC107816502	0	0	0	0	0	0	0	0	0	0	0	0	0	0	0	0	0	0	0	0	0	0	0	0	0	0	0	0	0	0
LOC107794299	3.087166	3.255371	2.528358	10.56318	18.58026	9.16176	5.756464	6.191102	7.760917	10.69803	9.857538	14.80811	27.94784	19.62316	8.222602	1.901289	4.954337	2.187483	8.931914	5.179894	10.09874	4.581051	4.996072	8.826771	6.12886	1.420022	7.960978	9.122975	10.80925	4.732664
LOC107779908	12.7885	15.20235	12.02852	9.273163	5.490323	6.426255	5.977878	5.161096	7.248926	12.95098	22.75497	14.00687	23.98513	17.85311	13.20358	15.75464	19.30127	16.01623	2.000389	3.110764	3.641908	5.762139	7.613088	7.409709	1.581706	1.160495	4.047587	0	3.467784	2.344067
LOC107802669	0	0	0	0	0	0	0	0	0	0	0	0	0	0	0	3.773441	6.175041	5.260481	1.17927	1.404427	2.195508	4.090141	4.539698	7.374836	0	0	0.196224	0	0	0

LOC107813545	0.163177	0	0	0	0	0	0	0	0	0	0.11442	0.413569	0	0	0	0.1216	0	0	0	0.276073	0	0.272546	0	0	0	0.227049	0	0	0	0
LOC107811249	0.363538	0.41415	0.526693	0.109782	0.080421	0.227995	0.101638	0.155308	0.028442	0.029541	0.021243	0	0.236072	0.0661	0.031094	0.293485	0.292674	0.361377	0.122964	0.179391	0.082888	0.2024	0.13361	0.126657	0.038302	0.042153	0.107418	0	0.14585	0
Novel01800	8.01292	6.469585	8.040667	5.018174	4.425526	4.972341	5.232286	4.732785	6.967502	3.565942	4.449681	3.816384	4.430114	3.813494	2.20785	6.572369	7.325522	7.297067	2.073667	2.001669	3.237064	4.311495	5.787161	8.712355	0.543934	0.074828	1.970399	0	0.776719	0
Novel00368	0.201074	0.335966	0.174789	1.396581	1.304772	1.981642	0.843247	0.830381	1.32144	1.993404	1.503927	3.369145	2.574141	3.69259	1.479034	0.149841	0.259006	0.274833	0.612108	0.396888	0.580712	0.699675	0.325161	0.700541	0.08474	0.13989	0.554524	0	0.16134	0
107815858	0	0	0	0	0	0	0	0	0	0	0	0	0	0	0	0	0	0	0	0	0	0	0	0	0	0	0	0	0	0
LOC107798800	3.113258	4.826421	3.459586	1.337535	1.618809	1.725326	0.726835	0.932389	0.903978	0.688536	0.607644	0.569418	1.500622	0.630262	1.284733	5.405369	3.131696	2.464637	0.358251	1.00457	0.790333	0.589682	0.594516	0.268369	0.162314	0.357268	0.303474	0	0.154519	0
Novel04534	0.503047	0.286541	0.437288	1.993841	3.616673	1.081674	8.174838	13.32433	7.438451	4.660076	3.351	2.12494	4.199991	4.047396	7.292929	0.562307	0.485986	0.375043	4.530308	4.946938	4.473176	8.349639	8.430692	3.549045	5.406036	7.349508	2.675524	53.96245	10.29285	19.44009
LOC107762440	0.796506	1.106159	1.417743	2.474037	9.565157	2.242799	2.640443	2.689818	3.382882	6.768334	2.952127	8.331273	8.740149	7.86192	3.931844	0.621822	1.319129	0.904882	19.5517	10.52395	9.997019	5.289789	2.84374	3.131816	22.01071	12.66606	10.84859	8.13735	21.36456	9.380795
Novel03650	0.986108	1.480239	1.714406	1.734178	1.591164	1.496735	2.043403	2.924171	2.014981	1.300962	2.847188	0.784086	1.226897	1.645335	3.750778	2.507133	1.625132	1.60011	2.295574	1.962776	1.163861	2.712788	2.200115	1.333822	2.126793	4.842699	1.713972	0	4.468214	17.93311
LOC107807374	0	0	0	0	0	0	0	0	0	0	0	0	0	0	0	0	0	0	0	0	0	0	0	0	0	0	0	0	0	0
LOC107797595	8.803854	7.677283	8.946856	4.504344	4.343473	6.060464	4.781118	4.085827	4.697627	1.945492	4.063698	2.702201	3.525386	3.21304	2.242757	6.63144	8.260507	9.302434	3.50272	3.455833	2.512755	5.262997	6.033689	8.605986	0.440425	0.705031	1.796624	4.529496	0.914782	0
LOC107768965	0	0	0	0	0	0	0	0	0	0	0	0	0	0	0	0	0	0	0	0	0	0	0	0	0	0.020644	0	0	0	0
LOC107816672	4.497985	5.077915	3.632519	57.23328	70.64059	22.61946	160.2769	125.7098	83.77702	46.80397	56.01893	40.03508	39.08862	33.84303	47.14898	6.595682	6.195745	6.03619	83.01036	70.37996	107.2669	118.0717	85.74963	64.39254	109.5905	56.69097	116.5461	58.10804	62.17052	82.538
LOC107813161	10.57668	16.72859	14.44785	16.52758	16.04391	13.838	18.72792	18.83905	14.50074	13.47862	17.54032	11.11025	10.34128	12.51546	21.71123	18.22396	16.59743	16.39655	18.34176	18.17833	13.78016	22.199	15.49953	10.00191	10.40055	18.53212	7.540158	8.004046	10.50728	3.46017
LOC107786322	0	0	0	0	0	0	0	0	0	0	0	0	0	0	0	0	0	0	0	0	0	0	0	0	0	0	0	0	0	0
LOC107816723	6.07646	7.858311	7.200232	7.01191	7.028956	7.299371	8.34246	7.802341	7.425864	7.183204	6.664966	7.628645	7.822518	7.036495	6.341238	6.779629	7.57573	7.010512	6.165971	7.150427	5.448954	8.476584	8.773368	7.983244	7.639573	7.699201	9.789834	9.710677	7.190986	6.296927
LOC107803972	1.393697	4.904316	1.850919	0.946964	2.021134	0.665953	4.156278	4.56478	4.03443	0.868269	2.38886	1.406379	1.034322	1.055871	0.953635	13.3285	14.5115	16.42292	3.221295	1.702959	3.566071	19.56019	20.59132	11.1275	0.195784	1.131216	0.823619	0	0.186382	4.787464
LOC107759647	0	0	0	0.037102	0.072477	0	0	0	0.076898	0	0.028717	0.034599	0.227951	0.089357	0.084067	0	0	0	0.083114	0	0	0.034201	0	0	0	0	0	0	0	0
Novel01075	1.916333	1.600959	3.401064	2.459476	1.766351	2.317829	1.473368	1.500919	1.574245	1.4015	0.5039	1.956257	1.155531	1.132422	1.065389	0	0	0	0	0	0	0	0	0	0	0	0	0	0	0
LOC107818283	0	0	0	0	0	0	0	0	0	0	0	0	0	0	0	0	0	0	0	0	0	0	0	0	0	0	0	0	0	0
LOC107771148	0	0	0	0	0	0	0	0	0	0	0	0	0	0	0	0	0	0	0	0	0	0	0	0	0	0	0	0	0	0
LOC107759885	3.048558	2.475288	3.131873	1.883072	1.814725	2.920019	1.208743	1.823339	1.951446	2.13496	1.787853	1.802854	1.851107	1.723383	1.137802	3.056588	2.704195	3.57454	2.277916	2.063096	1.011031	2.291337	1.955658	2.346313	1.261408	0.462747	1.670551	0	0.800555	3.427216
LOC107800554	0.097451	0.022204	0.021178	0.044143	0.021558	0.078579	0.163472	0.083265	0.068618	0.095027	0.102499	0.205823	0.108484	0.026578	0.075016	0.127086	0.117682	0.036327	0.123608	0.103046	0.088877	0.040692	0.085958	0.178247	0.092406	0.033899	0.201563	0	0.234581	0
LOC107803024	0.856393	1.019424	0.805215	1.646698	1.623841	1.352904	1.509882	1.17972	1.394727	1.806523	1.92406	1.063108	2.217993	2.288038	2.421673	1.133103	0.94554	1.159683	2.092713	1.892439	1.386752	1.342817	1.187049	1.424855	1.546772	1.434793	1.115471	0	3.197412	1.080654
Novel04263	4.165029	5.033707	3.659925	2.870993	3.324954	4.137204	2.58207	2.3209	2.805224	3.222655	3.142734	4.85738	4.334183	3.160933	2.044504	3.238731	3.149036	3.47648	2.021314	1.41699	1.775421	1.814778	2.236245	2.365917	0.744089	0.503942	1.55173	0	1.307735	0
LOC107819260	8.69929	10.99293	9.65623	9.912019	9.740556	9.730509	9.99055	9.305431	9.360955	10.42419	10.1353	8.465945	10.76433	8.596201	9.306466	9.18801	8.016357	9.55392	9.166955	8.915712	5.858879	7.977533	7.747131	7.990904	6.345315	3.933939	6.030707	2.392778	9.181709	5.172021
LOC107800439	3.058302	2.941258	2.952276	3.214586	2.810846	2.452412	1.884874	2.050054	2.141554	1.631165	2.156331	2.283968	1.58001	1.087575	1.057881	2.417556	2.726046	3.275273	2.297511	2.415592	1.802978	2.652844	2.965897	4.132539	1.303116	1.152016	1.87723	1.208353	1.057507	1.044748
LOC107805808	13.43353	14.86241	14.58659	18.38356	16.82697	16.05966	21.20046	17.37844	15.3473	13.49867	19.82202	9.699224	12.78925	11.95792	21.57881	14.2708	10.41429	13.07386	12.96024	15.36717	7.052216	20.78023	14.07882	11.95849	4.280862	9.028153	4.113594	4.504983	4.890341	6.816307
LOC107821188	0.58141	0.38537	0.505407	1.580184	2.05788	2.386696	2.039279	2.212896	4.763811	11.44205	2.038425	4.465376	2.05938	3.921064	1.735981	0.984699	0.765942	0.669902	2.520801	2.14618	3.22974	2.339472	1.86488	1.933558	14.96888	3.162414	20.36551	11.33974	26.71883	6.536265
LOC107784799	0	0	0.045171	0.235384	2.161117	1.45256	2.571485	7.370316	5.464048	10.03296	3.93522	11.45808	8.503524	19.55813	17.76038	0.348513	0	0.54238	13.76241	9.538888	7.298381	13.45293	4.446066	5.376968	60.63958	108.8178	54.6	66.89096	57.28972	35.34317
LOC107823082	0	0	0	0	0	0	0	0	0	0	0	0	0	0	0	0	0	0	0	0	0	0	0	0	0	0	0	0	0	0
LOC107777662	0	0	0	0	0	0	0	0	0	0	0	0	0	0	0	0	0	0	0	0	0	0	0	0	0	0	0	0	0	0
LOC107803864	0	0	0	0	0	0	0	0	0	0	0	0	0	0	0	0	0	0	0	0	0	0	0	0	0	0	0	0	0	0
LOC107828153	20.64118	40.59053	34.3424	2.680984	3.404168	2.386214	4.591884	5.499517	4.028577	6.925679	4.461398	5.125218	5.435667	5.407671	6.606243	63.84286	44.02769	32.10152	3.303192	2.878877	2.429045	4.510297	4.894323	3.634355	1.215976	0.102942	1.661391	0	4.630329	0
LOC107760263	2.0803	1.895937	2.208103	3.055111	3.914093	2.095319	6.336514	8.026656	5.078001	3.971697	2.917415	3.58899	7.069342	5.900713	9.372241	2.725162	2.009745	1.779513	3.688598	3.389912	2.815946	6.400626	5.215155	4.371545	5.730918	4.722745	3.545772	6.264036	18.55467	36.55745
LOC107825697	9.130919	12.03408	10.00482	10.94698	9.942309	12.34455	9.974387	9.460151	8.983168	7.410946	9.891451	7.436902	8.247379	8.022791	8.810544	9.636131	9.772027	9.701689	7.129384	9.68985	4.313373	10.57063	11.62295	7.972077	2.55771	3.423505	3.941992	1.955059	2.895535	1.690355
LOC107761953	2.469773	1.125447	1.923283	0.186457	0.182118	0.331913	0.129469	0.043963	0.144921	0.050174	0.036079	0.086939	0.057279	0.224533	0.105621	0	0	0	0.038361	0	0	0	0	0.045385	0	0	0	0	0	0
LOC107817590	2.278228	6.974317	8.090926	1.093722	3.497717	0.648979	3.037758	3.660235	3.96702	6.531765	3.645565	1.562799	1.170533	0.722263	3.757285	17.81899	2.55838	9.741058	1.75854	2.355496	1.438464	7.561645	4.173675	5.128744	0.51699	2.032043	1.081676	1.392525	1.874906	0
LOC107759383	0	0	0	0	0	0	0	0	0	0	0	0	0	0	0	0	0	0	0	0	0	0	0	0	0	0	0	0	0	0
LOC107781055	2.528678	2.502114	2.512144	3.141743	1.981822	1.359319	3.696445	2.901339	4.103688	0.84541	1.089197	0.915556	1.890044	1.931064	1.038146	2.476606	2.687207	2.558536	0.953059	0.733402	0.658913	2.684959	3.345692	2.756244	0.456717	0.150792	0.555043	0	0.347827	0
LOC107776063	24.91492	25.51497	23.36664	24.73738	24.81978	26.50101	21.16456	22.105	26.53333	31.34028	29.83743	33.12159	35.36441	36.22195	20.77383	25.45488	32.12742	29.22909	23.44913	19.86296	29.50576	19.38742	24.27267	29.31702	25.32092	20.10555	27.94052	53.18713	38.87482	29.56235
LOC107759070	3.324815	3.554935	3.665214	4.526584	4.832136	6.270496	4.829532	3.396278	4.464243	4.600542	5.287894	4.174113	5.293379	5.005144	4.022554	3.501938	3.732439	4.486749	3.713056	4.98091	2.541023	4.064132	4.259797	4.931141	2.160497	1.990057	2.919806	0	2.950982	2.296964
LOC107765291	20.11179	22.27372	22.46202	10.148	7.546505	17.38022	6.138899	7.395672	10.93473	4.468451	3.391717	5.161823	8.714599	11.10936	4.507314	16.88446	20.78238	16.13299	6.781158	6.676063	5.398206	6.378183	9.038382	8.780857	1.046065	0.797015	2.783257	0	2.144865	0
LOC107819118	58.14727	55.23801	56.55661	46.81174	44.08085	52.54342	47.52589	41.55587	60.16434	37.6581	43.87107	32.8256	39.76186	36.83251	33.30214	50.64547	51.41786	52.88149	28.47517	31.09951	18.73321	46.89844	55.09779	67.3325	15.90569	12.95274	24.07781	4.824074	15.5884	22.94006
LOC107799940	2.494274	3.199352	2.007611	3.431375	3.596752	3.873494	2.247022	2.9205	3.902891	2.882646	1.619432	3.199871	2.468143	3.527392	2.370421	1.65221	3.123658	3.168186	2.531017	3.282198	3.622862	2.777378	3.422401	4.151913	1.868753	0.514163	2.4021	6.606514	2.223764	0
LOC107793583	3.424163	5.879583	4.378551	4.181115	4.63271	5.201969	7.367285	6.826627	6.056784	3.798707	6.124353	3.815178	5.579629	3.35662	6.977999	7.488655	4.410705	5.327705	4.179596	6.317959	3.598117	7.915725	6.959909	5.134992	1.694028	2.969164	2.111522	1.774464	1.791863	4.602633
LOC107769367	84.26541	87.51911	78.75662	9.934352	8.761103	17.39802	10.31358	11.14319	16.19224	5.761721	6.569365	2.383485	7.940573	17.82838	8.741651	71.64068	69.73375	63.61717	4.105187	5.403619	6.748171	12.00285	27.84353	13.96502	0.067301	0.222203	0.188746	3.806802	2.562752	0
LOC107797592	0.482763	0.251416	0.299754	0.468599	0.274616	0.185368	0.404914	0.530339	0.194245	0.134501	0.314334	0.058264	0.499031	0.489051	0.814027	0.256968	0.366449	0.308503	0.384901	0.379214	0.34594	0.287977	0.152082	0.072083	0.261582	1.247497	0.081512	2.466026	0.996082	10.6607
LOC107803772	5.898169	5.131115	6.875046	5.100559	5.723204	9.2957	4.188126	3.178356	4.845278	2.908409	4.088802	3.057711	4.02909	3.985072	2.510918	4.495216	6.831277	6.22123	3.264575	4.564208	3.178636	3.134546	4.404453	6.444981	1.101614	0.559559	2.376532	0	1.774743	0
LOC107825684	0.102105	0.186112	0.133136	0.138753	0.271047	0.054888	0.942037	0.741549	0.335512	0.099565	0.501172	0.345044	0.568322	0.38987	0.209595	0.114133	0.246605	0	0	0.043187	0	0.127906	0.180126	0.05336	0	0.284143	0.06034	0	0.245784	0
LOC107816500	0	0	0	0	0	0	0	0	0	0	0	0	0	0	0	0	0	0	0	0	0	0	0	0	0	0	0	0	0	0
LOC107789761	0	0	0	0	0	0	0	0	0	0	0	0	0	0	0	0	0	0	0	0	0	0	0	0	0	0	0	0	0	0
LOC107807216	0.155084	0.318016	0.101109	0.193186	0.171536	0.22926	0.211373	0.215326	0.1638	0.151227	0.25827	0.114642	0.237383	0.148041	0.119381	0.101123	0.187281	0.173433	0.275396	0.196786	0.03536	0.032379	0.153894	0.081047	0.049018	0.053947	0	0	0	0
LOC107814373	13.43071	16.50484	17.21125	9.027531	6.287217	7.592463	10.90153	11.29047	12.52799	8.322701	7.625235	6.441961	5.932271	7.184325	8.937958	34.67488	17.62115	24.90371	5.407459	4.031484	6.559159	10.60125	14.44542	18.11325	1.205068	1.446801	2.509119	9.295021	4.380208	5.357683
LOC107784618	0.400719	0.730413	0.845961	3.993347	4.153686	0.984737	8.354503	9.244411	3.547152	4.18663	7.626729	2.659949	2.931426	3.372418	9.518368	4.223292	0.387129	0.810902	10.57218	11.81591	9.084364	10.85226	8.786033	4.068653	9.770746	20.55051	3.788946	4.093873	8.819229	21.23751
Novel00803	0	0	0	0	0	0	0	0	0	0	0	0	0	0.019556	0	3.713656	3.169198	6.722389	3.601632	3.745547	0.801092	0.718582	0.82222	1.311537	0.657253	0.124714	0.911045	0	0.863021	0
LOC107816453	67.39079	81.07084	54.54248	299.399	277.6727	146.7853	1231.361	1204	840.2868	391.5378	576.2984	334.053	370.8579	598.9479	1038.621	137.5493	64.26115	97.40629	356.4244	339.7635	400.7495	972.941	960.8631	605.1674	589.5387	917.8016	428.5262	417.4726	200.1721	353.5827
LOC107777193	0.221948	1.517088	0.482337	0.904832	1.080169	1.073796	0.465394	0.663735	0.729311	0.216427	0.622521	1.031294	0.494152	0.242135	0.227801	0.661584	0.643262	0.248208	1.013479	0.093876	0.20242	0.185355	0.685205	0.34797	0	0.308825	0.393487	0	1.602806	0
LOC107826867	0	0.041313	0.118215	0.061601	0	0.146209	0	0	0	0	0	0	0	0.024727	0	0.050671	0.043793	0.050694	0.045999	0.019173	0	0.018928	0	0.04738	0	0	0.026789	0	0	0
LOC107830639	0	0	0	0	0	0	0	0	0	0	0	0	0	0	0	0	0	0	0	0	0	0	0	0	0	0	0	0	0	0
LOC107816756	11.7698	10.04203	10.81173	6.692632	5.355071	8.301363	6.056125	7.916766	9.678548	4.883845	3.599707	4.125474	4.506819	5.691608	2.955792	8.366534	10.04001	9.552797	4.150465	3.565958	4.149055	5.402609	8.099228	9.640762	0.949825	0.754959	1.627877	0	1.406553	0
LOC107769591	1.727218	1.539926	2.317431	2.551259	2.093183	3.39099	1.070772	1.315371	1.410077	1.46457	2.290608	1.681254	1.713767	1.556604	1.348846	1.371068	2.357858	1.987559	1.257344	1.746976	1.883453	1.00344	2.119681	2.707932	0.759558	0.626947	0.931958	2.685228	0.723082	0
LOC107785047	0	0	0	0	0	0	0	0	0	0	0	0	0	0	0	0	0	0	0	0	0	0	0	0	0	0	0	0	0	0
LOC107790417	0	0	0	0	0	0	0	0	0	0	0	0	0	0	0	0	0	0	0	0	0	0	0	0	0	0.066926	0	0	0	0
LOC107813834	0	0	0	0	0	0	0	0	0	0	0	0	0	0	0	0	0	0	0	0	0	0	0	0	0	0	0	0	0	0
LOC107772136	4.01154	4.05867	3.333528	4.851007	4.51919	3.286921	5.395301	5.058307	4.745094	4.20471	5.526656	4.508795	5.803446	5.340357	6.910593	4.938444	3.790122	4.993406	5.343828	4.260546	4.01317	5.814784	4.44254	5.615109	4.893065	4.573605	4.845752	2.527593	3.147931	18.57565
LOC107761949	3.800678	4.195648	4.420642	6.692458	5.352523	5.668874	5.298021	5.145532	4.95031	5.976804	5.949415	6.354	5.988904	7.095519	5.796425	4.487751	5.016368	4.409987	4.155421	4.777384	4.271826	5.319879	5.524458	5.287298	2.402606	2.011069	5.029881	1.9141	1.675152	1.654941
LOC107828726	0.224085	0.510564	0.24349	0.406018	0.346998	0.240918	0.610835	0.95732	0.368166	0.327766	0.903487	0.283969	0.935453	0.427814	0.574986	0.667952	1.244786	0.501194	0.682157	0.61607	0.45983	0.748555	0.840044	1.229619	0.708277	0.467696	0.860762	0	0.539411	0
LOC107813556	0.673353	0.096897	0.462104	0.288959	0.250874	0.723937	0.237798	0.423928	0.565629	0.587488	0.670958	0.359285	0.197259	0.425292	0.145497	0.290506	0.547802	0.581278	0.251732	0.449692	0.355537	0.295966	0.500161	0.851954	0.224032	0.049312	0.712075	0	0.511857	0
LOC107774210	6.850304	6.928437	5.156006	4.919413	4.287473	5.388962	5.885782	6.709635	7.058817	6.516987	5.682115	6.21079	6.509875	5.468301	6.002042	5.914161	8.232108	5.792303	4.069004	4.946854	5.485691	5.441801	7.000366	7.247252	2.429267	2.673525	3.357089	0	5.630698	15.49627
LOC107767515	6.601104	8.001403	6.771076	1.495066	0.613315	3.477527	0.193782	0.084602	0.402831	1.41612	2.129201	0.167304	0.110227	0.468096	0.16938	4.033703	5.452525	5.6104	1.272685	1.954428	0.331117	0.248074	0.524035	0.241482	0.083458	0	0.624158	2.360361	0	0
LOC107787136	8.424984	9.589231	7.887018	2.590252	4.149152	4.590423	1.822555	1.205185	2.004279	0.446086	0.267312	0.128826	1.867274	1.206091	0.586912	4.602198	4.161694	6.224339	0.889724	1.93492	0.69536	0.700409	1.513179	1.075821	0	0	0	2.726269	0.5506	0
LOC107798881	0.91133	1.067869	0.905371	0.668358	1.190407	0.466567	0.436784	0.51911	0.692626	0.507807	0.639025	0.293302	0.434788	0.236718	0.311788	1.358248	1.257745	1.294162	1.144934	0.954475	0.831148	0.688592	0.803853	1.315387	0.493799	0.120767	0.512914	0	0.417854	0
LOC107798396	0	0	0	0	0	0	0	0	0	0	0	0.026498	0	0	0	0	0	0	0	0.026533	0.171633	0	0	0	0	0	0	0	0	0
LOC107764242	849.1124	673.7907	799.7183	566.3958	512.4174	715.4068	479.1299	475.9793	597.9012	395.6137	452.1757	510.2455	374.1187	424.3305	378.398	620.3189	789.5731	777.6436	361.884	357.5104	475.924	457.0867	581.0633	748.3109	144.8427	94.24073	278.7545	148.2332	139.4378	76.20517
LOC107790135	1.943862	1.866837	1.453555	5.737583	6.047942	7.101129	5.802824	5.964911	5.08248	7.378225	5.584055	6.462964	9.377048	6.407579	5.878115	1.5576	1.615427	1.994641	4.920637	6.135458	4.231928	4.346442	3.890147	5.112079	5.866606	4.129821	6.398381	4.48431	4.025137	7.754317
LOC107813427	0.233317	0.21264	0.304227	0.105687	0	0.250845	0.195693	0.099676	0.109524	0.341271	0.899813	0	0.389598	0.254538	0	0	0.112702	0.086974	0	0.394741	0.106395	0.097425	0.102901	0	0.147492	0	0.551525	0	0	0
LOC107794951	13.58108	20.16967	28.2533	19.88178	18.43584	16.45967	26.14761	24.73771	39.35273	24.68064	27.2273	20.41793	29.92805	22.12342	26.26281	30.72994	12.91173	28.37378	14.3136	15.2747	13.62479	24.33356	25.91909	46.32713	23.41448	7.129354	49.3956	22.07362	21.84425	7.633988
LOC107797199	13.73514	13.52568	16.49293	8.436162	7.415849	11.88865	8.542559	8.105572	9.889926	6.696747	6.52955	7.031132	5.053523	6.158826	5.615054	12.62082	14.73117	15.23002	5.787593	5.563326	6.263338	8.408513	11.80728	11.37527	3.679108	1.700594	5.984504	0	4.763302	7.197155
LOC107785385	1.41683	1.291265	1.119654	0	0.075982	0.092319	0	0	0.040309	0	0	0	0	0	0.093678	0	1.119812	0.705127	1.088316	0.087133	0	0	0.035855	0.075742	0	0	0	0	0	0
LOC107794249	242.2636	226.7932	265.6816	106.5238	103.1836	154.3575	85.84972	90.43871	116.0886	48.45326	62.4589	60.55011	39.7017	52.95945	41.42253	166.7134	232.8856	207.1215	68.83537	76.99714	81.02835	74.05355	123.7114	152.6941	16.28458	11.78866	35.18307	12.28162	10.47283	24.77709
LOC107792267	0.645468	0.647091	0.617201	3.888665	12.65104	6.384409	5.549167	7.555628	5.393348	5.759131	1.697261	11.86048	19.25699	16.47768	9.341124	0.577205	0.654756	0.529347	13.68901	6.716069	17.0128	5.363523	2.846729	6.038058	14.52602	7.319696	12.93101	4.616012	57.33365	11.97309
LOC107807561	0.232386	2.58385	0.727229	0.042106	0.041126	0	0.038982	0	0	0.045321	0	0	0	0	0	3.290309	0.628612	0.693014	0	0.196582	0	0	0.040996	0	0	0	0	0	0	0
LOC107767915	11.87225	9.731276	8.551564	16.31951	10.70356	19.46729	15.35938	11.67514	10.16476	5.642849	13.77	6.718211	13.90211	10.58972	16.11302	8.819392	7.502122	8.698177	4.546089	6.742776	3.285761	14.10836	10.43918	7.394594	0.56642	0.727268	1.036959	1.334959	1.527788	2.308424
LOC107814213	1.121871	1.216359	1.008847	0.735983	0.462121	0.935805	0.892291	0.644547	1.325657	0.50926	0.474707	0.604623	2.11018	2.025775	0.933078	0.850434	1.513613	1.067134	0.176648	0.179988	0.352816	0.791522	1.313732	0.869326	0	0	0.068584	2.766539	0.093122	0
LOC107814618	0	0	0	0.174461	0	0	0.161519	0	0	0	0	0.488072	0	0.840349	0	0	0	0	0.195409	0.162903	0.702518	0	0	0.603831	0	0.535902	0.455211	0	0	0
LOC107804303	7.600449	7.504112	8.644039	10.27105	8.070453	13.55093	7.012306	7.089316	8.979009	6.423211	5.418264	7.063178	8.672449	8.7754	5.005573	4.57829	5.262249	5.147032	7.00544	6.643772	6.007506	4.654711	6.815834	6.686189	3.042938	2.46761	4.865856	4.529496	3.659127	0
LOC107803297	0.439081	0.588482	0.291876	0.514781	0.342818	0.19438	0.519919	0.662052	0.848706	0.377788	0.597657	0.305486	0.71881	1.267983	0.397642	0.769891	0.274475	0.558426	0.157253	0.087396	0.141335	0.819655	0.523992	0.242961	0.261238	0.179691	0.061054	0	0.497386	0
LOC107813029	28.49554	27.10165	22.8177	21.15587	15.85075	18.369	18.99142	15.55806	22.9786	19.08177	21.18327	24.42344	31.72163	24.83109	14.44148	22.02791	27.5862	21.55316	10.4383	9.25202	13.20988	16.68778	20.18096	19.09359	3.662498	2.056507	9.083664	25.36723	2.846217	3.65544
LOC107820181	0	0	0	0	0	0	0	0	0	0	0	0	0	0	0	0	0	0	0	0	0	0	0	0	0	0	0	0	0	0
LOC107764425	2.449413	3.949521	4.258446	5.206219	5.251782	5.671979	5.847219	3.702726	5.306809	5.787488	5.020473	2.944819	4.824199	5.961038	6.285027	4.914284	3.640522	5.970078	5.831355	6.375493	2.835342	7.55287	5.899948	3.741723	4.168773	4.719018	2.004237	0	4.081966	5.825035
Novel05078	5.77718	5.265187	6.217689	4.187066	3.554077	5.323864	3.830307	4.513078	3.254322	3.541044	3.858059	6.461146	3.307493	3.721546	2.315344	3.854144	4.99654	4.143051	3.405707	3.770046	4.265288	3.400246	4.659067	5.405722	1.18257	1.76082	2.211024	7.869524	0	3.402016
LOC107789265	36.91295	14.49826	22.1142	5.596864	3.51425	7.875511	4.052806	3.845812	7.291534	4.152417	5.507761	7.922093	3.929899	9.531981	3.170413	9.602165	19.63168	16.71279	1.880672	2.57571	3.15926	3.02189	4.456762	4.38163	0.167373	0.122801	1.747212	7.889414	0.637342	1.364246
LOC107808047	0.968999	1.64359	0.889126	0.146311	0.285811	0.231509	0.451523	0.045997	0.050541	0	0.207614	0	0.089892	0	0.082879	1.664839	2.132306	0.943172	0.136566	0.250466	0.245484	0.854194	0.73601	0.1688	0	0.187263	0.063627	0	0	0
LOC107809131	1.959787	0.893052	0.309746	0.484218	0.47295	0.383093	0.373581	0.152227	0.083633	0.260596	0.687102	0.602066	0.495832	0.29155	0.640014	0.597451	1.893319	0.86338	0.27118	0.45214	0.162487	0.074394	0.078576	0.372431	0	0	0.105287	0	0	0
LOC107775510	86.37624	90.65572	89.89773	60.76166	53.70252	84.29148	50.48089	57.73642	63.91359	38.79879	37.02302	50.91803	31.30418	48.3524	31.18836	53.85867	95.93534	62.8326	37.73406	41.25628	51.52249	39.46748	49.97857	54.65958	14.53955	11.79671	33.93071	42.02124	25.05583	25.95128
LOC107830241	8.296269	8.174836	7.21177	2.329682	2.085843	1.843145	5.238086	4.185126	5.029734	1.104526	2.146623	0.9828	1.363177	2.070668	1.728149	5.155793	5.678448	6.573223	1.522161	1.372538	1.898564	5.445593	5.508645	3.871668	0.503163	0.937123	0.578924	2.189301	0.736922	3.785762
LOC107826126	26.53524	27.6459	37.65197	13.71456	15.48364	10.30372	14.77313	22.59866	22.80489	18.29787	12.41108	11.33043	18.42208	19.30956	31.25236	28.24564	20.2135	29.11685	32.67933	27.04838	26.69414	30.66867	17.41476	17.77784	68.8074	108.2427	42.45166	67.92005	55.56159	92.53484
LOC107824897	2.522426	2.765841	1.952866	5.534446	5.545147	4.025502	5.024711	5.219697	2.812196	4.919369	5.002181	4.062249	6.186376	5.417691	4.571114	2.67272	3.921847	3.114704	5.59909	4.734367	4.780717	4.70681	3.789363	3.460327	4.335202	3.674293	3.260802	2.818581	3.225713	4.87392
LOC107793227	0.149656	0.136393	0.113832	0.101686	0.215192	0.040225	0.188285	0.239757	0.140504	0.091209	0.104939	0.742799	0.20825	0.428578	0.172804	0.195167	0.054218	0.041841	0.189826	0.221548	0.034122	0.124982	0.099005	0.136868	0.141908	0.052059	0	0	0.270187	0
LOC107830106	0.871785	0.529683	0.94728	1.316322	2.507092	2.186977	1.523341	1.303535	2.591823	3.117036	2.037654	5.70794	3.477566	4.121324	1.714988	0.757931	1.544066	1.841533	2.285287	1.843676	4.372955	2.608848	1.922431	2.809501	5.419158	2.426051	7.38439	0	4.546841	0
LOC107832639	10.51956	10.28273	10.23418	7.357501	7.427425	6.328805	8.457477	8.569056	9.210985	3.82678	4.433436	3.637771	4.125985	3.984007	4.195719	9.707593	10.84732	10.60603	5.586147	4.057494	5.219541	10.87903	13.65399	11.50776	2.75646	1.516808	2.512422	0	4.460949	6.740311
LOC107780027	0	0	0	0	0	0	0	0	0	0	0	0	0	0	0	0	0	0	0	0	0	0	0	0	0	0	0	0	0	0
LOC107817417	15.85334	12.41543	13.26159	21.5074	17.09452	21.66936	17.43963	18.17415	17.165	18.37214	18.6297	18.7776	17.47079	22.77059	18.47912	12.49664	13.96881	13.95751	14.26801	15.12931	13.36868	14.1377	15.77558	16.52828	11.88506	9.643783	12.52297	8.545765	15.34143	12.31452
LOC107774350	14.43682	13.32607	13.8904	11.01098	10.56365	14.69222	12.54864	12.17707	13.8725	10.01684	10.9667	11.90996	11.40099	11.00471	8.105333	13.05713	14.81144	14.28203	8.670746	8.350469	9.424829	10.63966	12.84307	15.18609	5.187152	4.893179	9.224318	8.824249	6.831591	5.722119
LOC107773159	1.035604	1.1922	1.137131	0.740688	0.62699	0.644601	0.685741	0.605422	0.71641	0.797246	0.840824	0.598621	0.728115	0.654091	1.286687	1.056056	0.737196	1.015904	0.995551	1.106589	0.546809	1.502125	0.865394	1.25332	2.342991	2.275212	1.095158	0	1.312044	0
LOC107784979	1.174434	0.978607	0.904235	0.881582	1.0986	1.046204	1.576078	1.462197	1.386139	1.701472	2.28231	2.267873	4.146314	3.294643	3.099618	1.450314	1.426357	1.651113	0.408595	0.482553	0.795677	1.317075	1.213516	1.473022	0.551513	0.326828	1.07081	0	1.292376	0
Novel01395	18.85422	15.98843	20.67691	13.4046	11.04864	13.89408	12.67202	16.05621	19.34234	7.366273	8.142482	10.97067	7.853417	6.470388	4.869902	40.19655	48.31133	46.40546	20.39897	18.74843	31.25912	30.55282	51.65419	67.92843	2.131164	2.77979	8.485693	8.929424	7.213582	0
LOC107784845	0	0	0	0	0	0	0	0	0	0	0	0	0	0	0	0	0	0	0	0	0	0	0	0	0	0	0	0	0	0
LOC107771356	5.953906	6.657977	6.382187	18.13422	13.02557	19.94968	8.057477	6.897341	8.196071	17.66676	17.85211	23.94662	8.417117	9.005918	6.223397	9.227575	10.23357	8.196968	41.77238	35.41066	41.14188	14.39821	18.55829	23.25043	9.651886	20.7873	14.5058	7.836597	15.65102	6.775564
LOC107783447	0.404937	0.347341	0.289885	0.561068	1.412181	0.460966	0.799148	0.671626	1.22997	2.624687	2.622287	4.86992	7.371599	8.237639	4.082816	0.213007	0.207107	0.461726	0.314219	0.382848	0.369309	0.15914	0.294149	0.298758	0.240924	0.033144	0.36599	1.703453	1.261447	0
LOC107824311	0	0	0	0	0	0	0	0	0	0	0	0	0	0	0	0	0	0	0	0	0	0	0	0	0	0	0	0	0	0
LOC107787841	2.067136	2.354925	2.278235	0.869478	0.424621	2.5796	0	0.063079	0.034656	0.35995	0.258835	0.031185	0	0.241623	0	2.035572	2.032689	3.054756	0.861509	0.93678	0.504981	0.030827	0.130239	0.154326	0	0	0	0	0	0
LOC107830232	9.02475	12.8959	9.079889	8.920383	8.552578	8.415005	9.555258	9.198451	7.871367	9.845982	11.29889	7.918509	9.968959	8.47812	10.99238	11.60314	9.714318	12.67793	9.906702	11.16872	6.388973	10.49105	8.808064	8.239026	7.553861	10.7357	6.123378	6.971884	8.247142	6.88906
LOC107796900	0.347116	0.47453	0.392262	0.503151	0.368581	0.223915	1.077221	0.533853	0.488831	0.406178	0.389436	0.322578	0.347773	0.416555	0.320643	0.362139	0.670686	0.724611	0.563566	0.234909	0.411548	0.840668	0.949155	0.72561	0.131658	0.33809	0.32821	0	0.167114	0
LOC107824854	10.52987	11.80815	8.749762	10.9915	13.10816	7.987437	12.63848	10.90392	10.18122	6.207514	11.01757	8.275873	14.69002	10.16397	15.00454	12.61304	13.61672	14.33855	20.50363	15.57237	21.85701	14.78555	14.53322	13.2289	13.08571	19.17415	6.550245	19.28112	16.72993	36.11955
LOC107763824	68.48503	60.01506	75.03723	19.63589	17.3809	33.25477	11.48827	13.24636	17.7346	6.457997	6.86025	8.265437	6.115835	6.568247	6.179443	39.14562	49.73078	46.73816	13.89878	10.37712	15.92374	14.32984	24.76078	32.80119	2.283584	1.780179	7.560685	0	1.449281	0
LOC107771176	12.46224	15.93586	24.16629	13.30502	12.55428	13.5015	14.9392	16.70929	21.78019	25.33279	22.19587	19.45333	21.30061	20.43537	22.43328	36.14738	13.72511	24.07117	17.45123	19.90043	10.75377	17.67681	17.60495	20.88093	18.42247	22.32897	15.43184	23.309	19.84538	15.41118
LOC107831196	0	0	0	0	0	0	0	0	0	0	0	0	0	0	0	0	0	0	0	0	0	0	0	0	0	0	0	0	0	0
LOC107779983	0	0	0	0	0	0	0	0	0	0	0	0	0	0	0	0	0	0	0	0	0	0	0	0	0	0	0	0	0	0
LOC107831521	4.339749	3.955146	6.141201	5.028781	5.268968	4.448749	5.417577	4.39786	3.884843	3.936561	4.953777	2.813703	4.606368	4.404142	5.282888	4.738166	3.412551	3.385954	4.301266	4.951755	3.4977	7.248476	5.252297	4.219446	5.74196	2.246863	5.248519	0	9.231833	6.240307
LOC107825278	1.382549	2.176404	1.748103	0.93939	0.556077	1.047243	0.026355	0.053695	0.059	0.09192	0.660985	0.106183	0.034979	0.034279	0	0.0645	1.685908	1.214235	1.241584	0.733342	0.717673	0.630454	0.104964	0.110864	0.197051	0.371811	0.918137	0.148551	0	0
LOC107832048	0.653457	1.003024	0.956693	0.031158	0.030433	0.036976	0.173079	0.088158	0.193735	0	0.024116	0.029506	0.038286	0	0	0.794509	0.730973	0.948719	0.279193	0.058187	0.188199	0.172332	0.182019	0.107841	0.260895	0.478546	0.284544	0	0.993466	6.379618
107767962	0	0	0	0	0	0	0	0	0	0	0	0	0	0	0	0	0	0	0	0	0	0	0	0	0	0	0	0	0	0
LOC107829704	0	0	0	0	0	0	0	0	0	0	0	0	0	0	0.047788	0	0	0	0	0.047246	0	0	0.038883	0	0	0	0	0	0	0
LOC107783944	47.78838	50.04039	46.43004	87.20303	96.78491	85.31903	120.3832	98.63209	112.5855	96.59041	92.50761	103.744	142.2664	124.75	121.8549	46.44334	51.86309	47.33557	64.64727	61.47737	66.03232	79.27412	71.79587	76.3566	46.3018	40.59955	41.19752	56.34383	84.75585	43.67568
LOC107779139	11.38149	12.83353	11.55467	6.397367	4.815009	8.976403	5.225991	5.039783	5.966688	9.194649	10.39822	10.49271	7.768485	8.881978	5.883457	10.3078	10.91521	10.15768	5.268771	6.606042	4.811218	3.885246	6.411913	7.033358	1.312925	2.485291	1.620132	2.970574	0.59994	0
LOC107822909	0	0	0	0	0	0	0	0	0	0	0	0	0	0	0	0	0	0	0	0	0	0	0	0	0	0	0	0	0	0
LOC107793170	1.054626	1.922323	1.375146	0.238859	0.311068	0.377951	0.589707	0.300367	0.082511	0.856995	0.554628	0.148496	0.391341	0	0.902033	2.030264	0.679238	0.982836	0.26754	0.371725	0.080153	0.880746	0.465125	0	0.111114	0.733718	0.103874	0	0.423112	0
LOC107785867	2.611308	4.293126	4.050311	3.432603	3.352718	2.587281	3.822142	3.849866	4.374443	2.945783	3.267169	4.022898	3.419934	3.686693	3.573566	4.845796	5.44121	4.313588	3.481077	3.551704	3.689078	5.045706	5.645456	4.388348	3.236752	1.638612	4.236174	10.98503	2.218547	12.66362
LOC107795956	0	0	0	0	0	0	0	0	0	0	0	0	0	0	0	0	0	0	0	0	0	0	0	0	0	0	0	0	0	0
LOC107816598	3.237204	3.237148	2.970368	5.702556	7.857366	5.268924	2.790604	2.958035	2.321634	4.603496	4.602913	6.685262	8.20843	7.014211	5.999098	3.115976	3.474902	3.117403	5.566065	4.373916	6.232812	4.240636	2.141571	1.879736	3.069608	2.064486	2.497603	9.646078	6.92669	2.780017
LOC107792649	7.092594	5.503156	6.290416	1.780046	0.63608	3.348996	1.406826	0.900826	3.509388	0.527116	0.302432	0.36438	0.160045	0.418252	0.098374	3.249812	3.981592	4.323158	0.486289	0.567553	0.655598	1.320716	2.029017	1.602849	0	0	0	0	0	0
LOC107759842	0.027338	0.024915	0.047529	0	0	0	0	0	0	0	0	0.026658	0	0	0	0	0	0	0	0	0	0	0	0	0	0	0	0	0	0
LOC107777856	0	0	0	0	0	0	0	0	0	0	0	0	0	0	0	0	0	0	0	0	0	0	0	0	0	0	0	0	0	0
LOC107814644	39.05879	36.31141	36.36322	42.32036	42.18884	45.7516	33.67029	35.07243	39.89572	75.15774	60.94685	84.63363	70.05238	58.26163	37.73798	29.33124	32.78439	34.82714	41.46907	50.32641	49.25549	33.27341	39.92867	41.45424	73.00637	55.43776	120.9677	99.14333	74.86893	37.2695
Novel05090	4.110919	3.221925	2.593808	3.004403	3.085154	2.283969	1.979033	1.828984	2.679582	2.569657	2.314023	2.383847	3.493177	3.839069	1.098525	2.870107	2.16983	3.40339	1.933525	1.653039	1.308902	1.063121	2.252904	2.983135	0.59458	0.676928	0.833754	0.57986	0.975909	0
LOC107768607	0	0	0	0	0	0	0	0	0	0	0	0	0	0	0	0	0	0	0	0	0	0	0	0	0	0	0	0	0	0
LOC107779898	0	0.107155	0	0.266293	0.104038	0	0.246539	0.351608	0.441539	0.057325	0.041222	0.297992	0.130886	0	0	0.131426	0	0	0.83515	0.298382	0.536153	0	0.414836	0.430114	0	0.081799	0.069482	0	0	0
LOC107767811	56.45374	54.63314	62.85058	34.06912	31.33513	42.40445	28.76308	27.12058	34.88584	21.39117	24.24468	20.6887	20.78224	23.49243	19.52862	46.30636	50.3005	52.10182	22.35083	23.55608	25.47799	27.25569	38.26508	46.93295	11.20713	5.294891	15.55654	25.61295	10.99224	11.07255
LOC107782068	347.2664	282.3828	352.6912	174.8386	165.3876	268.3408	148.7846	154.4404	228.6838	90.50098	90.81829	118.4866	77.34822	115.3023	64.77778	209.8315	331.5481	269.4649	111.3855	89.22467	172.616	141.8683	224.574	296.2488	36.6403	23.39811	86.68904	61.40809	32.72761	35.39584

LOC107775796	0.375819	0.342513	0.408364	0.127677	0.083137	0.050506	0.315216	0.120416	0.176418	0.091618	0.296465	0.277814	0.156888	0.15375	0.048216	0.770167	0.363073	0.420283	0.095339	0.119219	0.085688	0.117696	0	0.196403	0.118787	0.065366	0.055523	0	0.226166	0
Novel02222	9.793383	6.491245	7.287798	10.08217	8.074978	11.8851	9.520926	8.240949	13.72203	7.37938	6.034747	7.521566	7.515863	9.550938	4.34049	2.598545	4.085532	2.544421	3.914831	2.635986	4.736524	3.965435	4.842746	5.195566	1.969834	3.303461	2.893748	0	2.143129	4.587421
LOC107796229	4.814075	10.45198	6.541386	3.602172	1.046779	11.67628	9.191788	5.825971	9.748903	43.80307	52.67369	18.65575	27.65513	22.58504	9.612251	4.640427	5.317469	5.426519	13.47124	54.19179	4.150762	10.18125	6.883986	3.022436	125.1151	58.63987	136.4593	24.67496	37.8895	7.111367
LOC107820997	0.684015	0.983046	0.708946	5.886988	9.311715	7.778266	6.597697	7.755082	4.272989	13.03217	10.33053	10.04613	20.17856	13.08767	13.01501	0.862618	1.092888	0.961083	6.780736	8.234344	6.526255	6.745012	6.149492	8.469197	26.84215	16.3995	21.14409	15.05131	36.22401	19.52016
LOC107769542	0	0	0	0	0	0	0	0	0.063372	0	0	0	0	0	0	0	0	0	0	0	0.0571	0.184683	0	0	0	0.093921	0	0	0	0
Novel03054	0.134078	0.040732	0.03885	1.255167	1.779614	0.2883	5.772788	4.391458	2.349727	1.002362	1.03417	0.755152	0.149257	0.048757	0	0	0	0	0.770967	1.0964	0.570646	0.970424	1.971093	0.420415	0.395535	0.18656	0	0	0.215166	2.763413
107760800	0	0	0	0	0	0	0	0	0	0	0	0	0	0	0	0	0	0	0	0	0	0	0	0	0	0	0	0	0	0
LOC107815866	6.345084	5.942286	6.808971	8.404447	6.814899	6.962881	8.294816	7.552588	6.819789	6.86999	6.873211	5.175658	5.748175	4.917131	6.37768	5.445771	6.003059	6.198625	6.083322	8.1438	5.746914	6.97999	7.217921	6.906302	4.813269	3.470601	6.775301	0	3.160085	0
LOC107830173	4.183597	3.864357	5.455068	2.637723	2.726415	1.732291	3.248161	3.477968	3.768512	1.157706	1.803731	0.811961	0.534953	1.048509	1.421639	4.571107	4.014397	5.057917	1.204726	1.673868	1.649951	5.240746	5.285975	5.495413	0.679037	0.550651	0.701609	0	0.27218	1.747825
LOC107826755	0	0	0	0	0	0	0	0	0	0	0	0	0	0	0	0	0	0	0	0	0	0	0	0	0	0	0	0	0	0
LOC107794834	1.046064	0.825469	0.9315	1.502422	1.196542	1.042356	0.973677	0.948285	0.826394	0.671737	1.064471	0.560423	0.908873	0.779359	0.746319	1.036202	0.702481	0.998634	1.423928	1.327348	0.814414	1.267779	1.035222	0.973345	0.548372	0.781012	0.588027	0	1.473988	0.788777
LOC107768455	0	0	0	0	0	0	0	0	0	0	0	0	0	0	0	0.181551	0.784547	0.665992	0.247216	0.755669	0.592511	0.610377	0.644685	0.424398	0.513364	0.112996	0.095982	0	0	0
LOC107799099	0	0	0	0	0	0	0	0	0	0	0	0	0	0	0	0	0	0	0	0	0	0	0	0	0	0	0	0	0	0
LOC107787280	4.664864	2.550869	3.186124	4.648744	3.125318	6.161654	4.751042	3.587208	4.254449	4.483848	4.719594	7.375313	7.047632	7.561004	4.377498	1.936775	5.27922	2.682916	2.231546	2.931429	3.585881	2.949639	3.409338	3.970225	2.106359	1.761794	4.725852	0	5.774996	0
LOC107799190	0.028971	0.132018	0.075552	0.026246	0.025636	0.12459	0.048599	0	0.027199	0	0	0	0	0.063212	0	0.043179	0.027989	0.021599	0	0	0	0.048389	0.127772	0	0	0	0	0	0	0
LOC107812051	0	0	0	0	0	0	0	0	0	0	0	0	0	0	0	0	0	0	0	0	0	0	0	0	0	0	0	0	0	0
LOC107789169	0	0	0	0	0	0	0	0	0	0	0	0	0	0	0	0	0	0	0	0	0	0	0	0	0	0	0	0	0	0
LOC107769020	2.444389	1.750382	1.467162	0.474534	0.926981	1.126293	0.488146	0.447547	0.819607	0.28376	0.408097	0.245844	0.453521	0.444451	0.119469	2.211896	2.361485	1.605444	0.531514	0.590796	0.265396	0.340229	0.821376	1.155777	0	0.080981	0	0	0	0
LOC107828562	0.786301	0.992238	1.156718	0.347042	0.410327	0.368495	0.338208	0.413438	0.435357	0.176941	0.169648	0.085165	0.448883	0.263943	0.289706	1.277076	1.090755	0.766597	0.204586	0.051166	0.128714	0.521961	0.46238	0.568967	0.152942	0.056107	0.142976	0	0.291195	0
LOC107772503	0	0.042737	0	0	0	0	0	0	0	0	0	0	0	0	0	0	0	0.034961	0	0.039668	0.042767	0	0.041363	0	0.059287	0	0	0	0	0
LOC107798143	3.697737	2.680104	3.948354	0.34291	0.180347	1.659075	0.073263	0.248775	0.464702	0.141959	0.081665	0.073794	0	0.158821	0	1.822574	1.715841	2.257554	0.23636	0.073891	0.424869	0.65652	0.436599	0.060864	0	0	0	0	0	0
LOC107806904	53.64519	35.13395	38.53969	36.35775	47.82871	33.72076	27.78517	30.89193	40.81771	97.52644	66.99268	134.8174	102.7844	129.276	55.10634	51.49453	63.09034	56.33635	57.41631	48.74446	74.93364	25.62725	25.43325	43.83564	142.3329	111.179	253.6706	64.6401	62.44536	22.35527
LOC107782551	1.661703	2.502114	8.35288	1.439966	5.114379	2.01956	1.696729	2.407494	3.255819	11.34259	5.268672	10.19319	6.353766	6.463155	7.934404	10.12178	3.210689	8.348906	8.504218	9.595336	10.08138	5.852606	2.549099	4.379785	9.042993	18.19552	11.78399	20.667	9.739165	17.8688
LOC107761463	48.29302	62.12473	53.56642	38.30635	33.58593	39.37143	44.40363	42.03883	42.73158	28.67703	34.98687	26.85351	30.86469	32.8409	39.47785	51.59344	45.98419	48.45074	22.63202	29.03515	19.17183	44.83674	40.28869	34.86965	10.36754	11.7073	16.57422	9.550978	7.329916	8.257826
LOC107760419	0	0	0	0	0	0	0	0	0	0	0	0	0	0	0	0	0	0	0.273412	0.136758	0.049147	0.090008	0.237667	0.394271	0	0	0.063692	0	0	0
LOC107783408	4.011947	5.804208	3.999651	1.626684	1.241271	2.352728	1.600137	0.958858	1.158952	0.84809	0.511487	0.355532	0.812028	1.163076	0.719887	4.641365	3.333798	3.597659	0.626315	0.640793	0.409395	1.757246	0.965128	0.821063	0.035471	0.117112	0.066319	2.00637	0.13507	0
LOC107818478	4.291824	3.970733	5.14397	0.206191	0.287704	0.524345	0.35452	0.583394	0.488406	0.380461	0.273585	0.439497	0.904871	1.560723	0.333713	3.731318	5.936695	3.805748	0.362921	0.275045	0.237225	0.51591	1.089815	0.98552	0	0.045241	0.807003	0	0	0
LOC107767218	0.691756	0.63045	0.216478	0.701899	1.028347	1.041211	1.137203	1.087541	0.675429	2.428378	3.49243	2.174023	2.618246	3.079061	5.509595	1.072234	0.74849	0.453845	0.926569	1.029913	1.564615	3.35068	0.536954	0.665181	0.38482	0.770022	0.32704	0	3.730003	3.421785
LOC107783947	0	0	0	0	0	0	0	0	0	0	0	0	0	0	0	0	0	0	0	0	0	0	0	0	0	0	0	0	0	0
LOC107781886	0.092056	0.251693	0.080022	0.083398	0.325828	0.296914	0.231634	0.235965	0.259278	0.359064	0.129099	1.16657	0.512389	1.20514	0.566901	0.1372	0.088934	0	0.467059	0.467237	1.007477	0.538148	0.162399	0.481084	1.047479	1.537067	0.108803	0	1.772758	0
LOC107774641	6.678766	8.597481	7.494017	19.5164	12.94196	15.10673	7.795934	6.84105	7.758823	4.077647	5.03057	5.240574	5.846432	5.643026	4.515693	7.443384	5.648112	6.442067	7.300017	7.510683	9.290355	6.338948	7.464408	9.652761	4.785763	5.40484	3.34958	7.086443	4.961442	11.02856
LOC107791079	0.324333	0.443384	0	0	0	0	0.272032	0.415678	0	0	0	0.137003	0	0.176916	0	0	0.156666	0.120902	0.658221	0.274363	0.739493	0.135429	0	0.169496	0	1.128214	0.383335	0	0.780727	0
LOC107819014	1.56051	1.590633	1.463612	2.604264	2.034925	2.273778	1.928857	1.491238	2.216891	1.121249	1.526164	0.884691	1.051452	0.963223	0.758683	1.805546	2.142357	1.944147	1.229295	1.337454	1.161041	1.663324	1.829261	2.768484	0.33748	0.314272	0.485368	0	0	1.269588
LOC107796827	0	0	0.016044	0.016721	0	0	0	0.03154	0.034656	0	0.025884	0	0.020546	0	0.018943	0	0	0	0	0	0	0.015414	0	0	0	0.102724	0	0	0	0
LOC107797418	0	0	0	0	0	0	0	0	0	0	0	0	0	0	0	0	0	0	0	0	0	0	0	0	0	0	0	0	0	0
Novel05170	0.796417	0.418752	0.559172	0.388507	0.542094	0.526921	0.488146	0.287896	0.488888	1.015564	1.009503	1.035132	1.329874	0.668348	0.785982	0.365225	0.651036	0.342556	0.279744	0.155473	0.251427	0.306973	0.405284	0.256128	0.193637	0.042621	0.108612	0	0.147471	0
LOC107808457	13.97789	11.65108	14.26948	10.22975	6.558414	10.96344	6.925836	7.947889	8.219422	3.637955	4.70881	5.211047	4.54075	5.318221	4.288639	9.786167	11.96601	12.34957	6.107612	4.418344	6.305983	7.768351	8.907018	9.670465	1.069142	0.692143	2.057743	0	0.957929	12.30283
Novel03892	0	0.047666	0.045464	0	0	0	0	0.022344	0	0	0	0	0	0	0	0	0	0	0	0	0	0	0	0	0	0	0	0	0	0
LOC107780369	0	0	0	0	0	0	0	0	0	0	0	0	0	0	0	0	0	0	0	0	0	0	0	0	0	0	0	0	0	0
LOC107782328	2.975778	4.898303	9.581646	5.364299	7.38898	4.472526	5.271979	8.016927	6.642381	11.04445	10.45438	14.69932	7.842256	7.586039	12.43519	14.21044	3.285545	10.0062	6.532225	9.581164	7.311074	7.404732	5.303234	6.030139	7.332622	18.25234	6.208811	13.0292	10.37937	1.877519
LOC107822414	0	0	0	0.050998	0	0	0.023607	0.072146	0.026425	0.027446	0.039472	0	0	0.122824	0.086665	0	0	0	0.028561	0.02381	0	0	0	0	0.142341	0.078326	0.099799	0	0.27101	0
LOC107797993	3.019957	2.659804	3.728203	2.78191	1.886299	1.93718	2.256257	1.886462	2.763791	2.029217	1.939647	2.015347	1.271291	1.384296	1.093977	3.158231	3.015601	3.292118	0.95281	1.00899	1.272974	1.292813	1.813176	1.96284	0.256682	0.070623	0.629885	0	0.244355	0
LOC107809028	0	0.247346	0.1966	0.32783	0.200125	0.048631	0.227633	0.23189	0.084933	0.264647	0.380609	0.19107	0.302124	0.148041	0.092852	0.202246	0.174795	0.303508	0.321295	0.267848	0.165013	0.188876	0.079797	0.094555	0.057188	0.37763	0.427694	0	0.87107	0
LOC107819163	0.418619	0.4769	0.614076	2.441409	2.222532	1.856527	0.109723	0.290615	0.245637	0.280642	0.403612	0.132623	0.203881	0.285433	0.026854	0.311956	0.353869	0.487654	1.008867	1.261564	0.930608	0.0437	0.184625	0.464887	0.165394	0.14562	0.402004	0	0.125961	0
LOC107767243	0.080916	0	0.070339	0	0.023867	0.028998	0	0	0	0	0	0	0	0	0	0	0	0.020109	0.082108	0.022817	0	0.04505	0.047582	0	0	0	0	0	0	0
LOC107793640	0.524672	0.398478	0.456086	0.475326	0.386887	1.034159	0.293377	0	0.164195	0.08527	0.122633	0.59101	0.486727	0.095399	0.089752	0.586479	0.506877	0.717136	0.2662	0.221918	0.797516	0.146056	0.231398	1.096774	0	0	0.103353	0	0.420993	0
LOC107827726	12.94352	12.87998	12.96753	12.29521	11.01659	13.74704	10.98799	10.27345	9.456195	9.406021	10.52313	9.115697	9.589291	7.855735	9.416836	12.63781	11.81104	13.81431	9.014107	9.639963	7.569744	8.355329	9.656008	11.04325	3.743709	4.21377	5.620829	1.604237	4.535907	1.387032
LOC107820802	0.063299	0.31729	0.110049	0.372746	0.168033	0.102081	0.292003	0.081126	0.297139	0.030862	0.155348	0.106953	0.387558	0.069056	0.194904	0.117926	0.061152	0.283152	0.160578	0.107093	0.230918	0.052862	0.195418	0.19848	0.080029	0.088076	0.037407	0	0	0
LOC107832640	0	0	0	0	0	0	0	0	0	0	0	0	0	0	0	0	0	0	0	0	0	0	0	0	0	0	0	0	0	0
LOC107788156	4.594716	4.219729	4.33205	5.859632	5.441792	5.395871	5.424945	4.409032	5.840116	6.065811	5.601006	4.598373	8.498606	6.015131	4.280578	4.740906	3.619396	5.006581	4.769994	4.78377	3.287898	4.870251	6.359846	6.095348	5.049464	2.262213	3.091256	0	4.594278	4.370742
LOC107777923	3.615209	3.468229	3.80423	3.102818	2.549559	3.94524	1.641509	1.625754	2.041575	2.19999	1.829765	1.515633	2.511531	1.927526	1.590236	3.038411	3.282515	3.830152	2.427255	3.40313	1.462637	2.020337	2.925114	3.494509	1.580852	1.210298	0.931682	0	1.701231	0
LOC107784901	13.80705	18.82105	17.84851	12.00672	9.766909	14.16373	6.669611	6.219685	5.422778	4.937946	6.213923	4.07759	3.479215	2.546436	3.89809	11.73362	14.79113	13.12526	16.25854	14.3906	9.16457	6.938201	8.724021	8.394036	4.801736	6.825864	4.72264	5.658449	3.999752	4.892326
LOC107783216	1.411722	1.907733	1.396446	1.764067	2.713746	2.512178	3.470556	2.246053	2.239447	2.278523	2.799029	3.125597	4.281116	3.770637	3.097762	2.466799	1.787104	1.923532	2.815639	3.33558	1.687088	2.805126	1.80344	2.035216	2.831135	2.167513	2.934333	3.481314	3.749811	6.019925
LOC107783514	10.7376	16.50612	12.00714	16.14617	16.53713	11.58076	22.87146	19.48128	17.195	12.0444	15.05842	8.109734	12.50173	9.119274	17.0543	11.44543	8.830539	10.982	14.65412	13.39495	15.16965	33.62718	20.57603	18.66345	8.119575	20.70878	10.36162	8.50511	10.71518	16.80815
LOC107770599	1.644878	5.451286	1.875528	7.915353	5.40613	4.685232	12.30917	8.268301	3.77046	4.999369	14.67951	3.898208	6.420745	2.749984	7.345019	4.059352	2.60033	2.739327	3.554985	5.294753	1.733947	8.973526	7.499413	3.416125	1.080325	0.772817	0.631206	0	0.719912	1.320848
LOC107815970	0.67058	0.814868	0.323845	0.54001	0.988956	0.961274	0.624938	0.572962	0.419713	1.162489	1.828599	2.077257	1.078277	0.568999	0.611791	0.055524	0.431891	0.444396	0.378032	0.945441	1.155207	0.497795	0.657218	0.46726	0.47101	0.518369	1.056762	10.65688	0	0
LOC107807121	6.291042	5.982792	6.895279	4.150625	3.388443	5.513836	5.850108	6.193208	10.14344	2.13376	3.308462	3.119584	2.283677	2.163406	1.684423	4.382347	5.284935	5.200047	3.746963	3.23935	4.490246	5.596453	8.564938	9.720149	2.074902	0.761176	1.778056	0	2.63368	12.68428
LOC107831674	10.75423	10.85788	9.701194	13.23548	11.18328	11.34396	14.46609	12.3837	11.67498	9.412619	10.04095	7.469188	9.067558	9.550311	11.36511	11.51347	10.27747	10.93524	11.58917	12.6529	10.49539	14.18584	11.88939	10.87675	5.020852	8.268365	6.475228	6.218971	6.00084	10.75391
LOC107790139	7.906379	14.37044	18.97843	16.27904	30.84637	10.04584	28.37193	48.97683	33.23416	51.12069	46.08415	46.56694	27.70469	35.53106	60.21618	45.39397	8.332611	25.31973	61.67564	59.96627	71.2881	47.57034	34.03763	34.74529	103.766	141.3273	101.5175	199.1813	99.2695	124.9935
LOC107766754	17.73691	17.15737	19.15458	13.99201	13.89458	13.21607	13.38664	14.93455	15.14569	13.99864	10.448	11.45029	10.71779	12.5041	11.14632	19.6662	19.54371	18.71388	12.93893	14.13159	14.11185	13.1614	15.49348	16.47211	9.600298	12.55909	13.07262	8.196704	11.0361	10.63037
LOC107793714	0.868328	1.301937	0.462631	4.770717	9.740738	2.168265	13.57934	19.72078	15.46296	3.823938	5.420924	6.507629	4.2719	8.311818	13.28217	0.542711	0.541211	0.313246	21.11843	14.05114	28.61164	22.05899	21.91526	11.62283	16.0779	19.40938	9.402171	22.03467	14.42925	20.78323
LOC107789196	0.054449	0	0.015777	0.016443	0	0	0.030446	0	0	0	0.012727	0.015333	0	0	0	0	0.052602	0	0.147336	0.2303	0.148975	0	0.032018	0.01897	0.045893	0.151523	0	0	0	0
LOC107815817	5.203666	4.012885	3.330442	36.93699	36.73517	21.21021	70.40817	60.19408	42.57304	33.01039	25.62189	30.29031	73.90586	67.93618	36.56463	6.051039	3.148892	6.352237	47.40684	38.79515	51.47402	27.74574	20.47836	12.90985	41.643	25.14291	26.69642	44.98344	65.5214	45.96436
LOC107822085	10.65165	14.23624	11.76498	7.85916	8.453602	7.024574	9.62477	10.10968	9.794047	9.556838	9.336179	8.697303	10.08504	8.895017	8.960192	16.65264	10.58213	14.20421	9.137132	10.6594	7.611348	10.79837	11.09052	8.808923	12.70333	14.89739	7.592572	0	11.49854	18.67186
LOC107779323	0	0	0	0.046341	0	0	0.257421	0.218529	0.048024	0.04988	0.035868	0.043215	0.113886	0.167413	0.420009	0.038119	0	0	0.155717	0	0.419864	0.128155	0.090239	0	0	0.142349	0.060458	0	0	0
LOC107759715	9.953633	7.140445	9.123657	8.526436	9.679672	9.800762	6.447388	6.315337	7.309384	6.342567	5.873831	7.867926	7.515008	8.547371	5.209218	7.086978	6.283753	7.935173	9.450242	8.878608	11.05524	6.584189	8.040872	9.785503	4.547834	3.839536	6.75579	14.09555	9.489175	21.32741
Novel02875	0	0	0	1.417012	0.965605	0.312859	1.43393	0.528354	1.058659	0.28376	0.382591	0.184383	0.040493	0.079366	0	0	0	0	1.218052	1.046201	0.663489	0.273398	0.6417	0.456228	0	0.050613	0.214961	0	0	0
LOC107777199	9.489087	9.037299	10.31083	11.15413	9.047303	12.11479	10.80422	10.27655	10.53462	11.01114	10.4963	12.16526	12.01585	12.13787	9.763687	8.679995	10.63402	9.532912	9.147427	8.027115	9.476357	8.736855	8.349077	8.703037	5.398691	7.195838	8.103081	5.089529	7.880463	2.933623
LOC107788735	0.199542	0	0	0.090387	0.058856	0.035755	0.055788	0	0.062446	0	0.02332	0.028096	0	0	0	0.049566	0.032129	0.049589	0.033747	0.056266	0	0.027774	0	0.03476	0	0	0	0	0.160111	0
LOC107817408	0.031688	0.028879	0.082636	0.057415	0.224315	0	0.239201	0.081224	0.118999	0.123598	0.37773	0	0.141101	0.207418	0.390281	0.094455	0.061226	0.047249	0	0.107222	0.0289	0.026463	0.111803	0	0.480755	0.705458	0.187262	0	0.152556	0
LOC107821542	12.56585	13.01937	12.53297	10.60512	8.778231	13.22541	6.933896	6.441963	8.754894	6.642574	5.98233	5.866726	6.331629	9.09105	4.683737	9.216234	12.52303	10.35452	6.107038	6.65764	5.730134	6.738298	9.742176	8.917176	1.672322	1.196306	4.69005	4.729664	1.27361	8.178585
LOC107765863	3.43406	2.488695	2.913224	0.787142	0.475938	0.889647	0.416428	0.530269	0.310752	0.242071	0.290116	0.17477	0.230292	0.586784	0.042465	2.065752	3.157707	2.190083	0.629755	0.629995	0.716946	0.552843	1.058349	0.562176	0	0	0.48901	0	0	0
LOC107784095	5.793698	5.980682	5.704425	9.640648	8.579284	7.817948	10.01636	9.875329	9.18589	6.168427	8.249453	7.366989	9.082126	7.610567	9.647882	7.004876	7.224957	6.875856	9.298489	9.52708	10.19048	12.51583	12.12422	10.44277	6.540175	7.156645	7.16212	4.227871	8.538651	10.96632
LOC107772529	7.253497	10.18693	9.406284	1.777483	0.894363	1.917638	1.845093	2.184395	2.065298	7.710812	4.25236	3.264895	6.1553	2.724219	2.257846	6.557267	7.29468	9.929057	1.146284	2.112379	2.928089	1.886789	2.727044	3.666392	1.052367	1.075453	2.389211	0	3.721081	0
LOC107817183	33.5201	26.25237	33.23689	12.94791	12.38582	23.04861	12.48171	13.91109	16.11548	9.841818	11.67467	13.94152	9.10323	11.41267	6.427142	19.54418	38.50402	27.13271	9.56874	12.27708	13.90808	11.87417	17.15533	15.78509	4.191389	5.637896	10.36165	10.53697	8.157554	0
LOC107797263	54.12829	44.55056	55.11495	24.31212	21.93003	34.0013	17.79006	21.82522	22.98231	13.12135	11.19453	12.45989	11.00186	8.542534	7.880807	33.31157	48.98748	39.61812	19.90283	21.6082	24.89096	19.17358	30.77926	39.01441	5.478613	3.913871	14.19676	32.62026	6.954023	0
LOC107779856	6.094501	5.779564	8.591059	1.193796	0.947386	0.973995	1.726932	2.11107	2.319643	0.883405	0.404247	0.139157	0.366729	0.539092	0	3.130042	3.898678	2.210453	0.835713	0.348346	1.652464	1.444368	2.46994	2.152017	0.208251	0	0.292022	0	0.396501	0
LOC107762404	0.228733	0.083385	0.238599	0.290108	0.526234	0.196732	0.345326	0.273609	0.73013	0.133826	0.096232	0.077296	0.152777	0.499072	0.422577	0.340904	0.26517	0.51159	1.067669	0.386984	0.584102	0.382041	0.363163	0.095628	0.057838	0	0	0	0	0
LOC107816295	0.197065	0.25144	0.102782	0	0	0	0	0	0	0.038433	0	0	0	0.042998	0.040452	0.088112	0.152305	0.264456	0	0.066681	0	0	0.139059	0	0	0	0	0	0	0
LOC107761850	5.779679	5.391406	5.497014	4.517417	4.352118	3.338428	5.094802	4.899583	4.979353	4.000396	4.895044	4.614757	6.383557	5.340999	5.094631	6.367639	6.043487	5.981901	5.312836	4.448223	4.402973	5.792114	5.058075	5.401293	4.298395	3.942158	3.321798	0	5.892445	2.802873
LOC107821989	0	0	0	0	0	0	0	0	0	0	0	0	0	0	0	0	0	0	0	0	0	0	0	0	0	0	0	0	0	0
LOC107818537	0	0	0	0	0	0	0	0	0	0	0	0	0	0	0	0	0	0	0	0	0	0	0	0	0	0	0	0	0	0
LOC107818917	11.93588	10.18134	8.57488	6.925874	6.022759	4.772428	15.61654	13.52755	15.21135	6.709251	14.19693	6.229431	14.16568	19.10172	17.36354	3.822486	3.978693	3.364592	0.675653	1.31427	0.899646	7.228833	5.416385	3.763233	0.405325	0.343138	1.165888	0	1.187264	1.524821
LOC107785795	0	0	0	0	0	0	0	0	0	0	0	0	0	0	0	0	0	0	0	0	0	0	0	0	0	0	0	0	0	0
LOC107810922	7.714849	9.436521	9.283006	11.58746	10.67106	11.39389	11.20466	11.17133	12.84687	10.57173	10.10752	9.467844	10.48674	10.4985	9.41862	7.746175	8.590785	8.627621	7.540077	8.758889	7.924839	9.05393	9.920983	11.24654	6.725067	4.237361	8.206497	2.903793	8.601312	0
LOC107761504	1.963486	1.569232	1.759334	2.161949	2.138365	2.403278	3.065694	2.451958	2.779291	1.826278	2.584143	1.735368	2.522054	1.977292	1.95326	1.620766	1.750978	1.711593	0.827616	1.63542	1.349937	2.472251	2.318114	2.304818	0.687446	0.378283	1.071084	0	1.018007	0
LOC107830023	2.122952	1.823186	1.632502	2.515068	1.6979	1.49236	2.260007	2.476682	1.494841	1.114694	2.519195	1.000237	1.363442	0.935322	2.388453	2.007957	1.695978	2.252376	2.361362	2.659271	1.67553	2.932158	2.124663	1.194797	2.735673	4.885351	1.013314	0	1.179305	20.19467
LOC107828417	7.441136	5.274638	6.722085	0.528729	0.516424	0.627461	3.100205	3.116622	6.940406	0.521677	0.647953	0.780674	2.65291	3.554903	0.79868	3.298092	5.450304	2.574415	0.345459	0.082284	0.399202	1.827731	2.144959	2.795824	0.122979	0	0.114965	0	0.234146	0
LOC107813735	8.228845	8.148579	9.182181	6.84562	7.071382	6.294991	8.859633	8.416856	7.948456	3.394838	5.187516	4.31155	4.668314	4.790752	5.684714	8.372736	9.058135	8.818995	9.112809	9.203293	14.17983	14.07464	11.79488	12.65308	2.200796	3.027601	2.618493	5.658449	5.904395	14.67698
LOC107794429	3.576079	2.946633	3.321528	2.196814	1.907279	2.607038	2.927508	2.385801	3.460862	1.277818	1.940774	0.87945	1.731437	1.803695	1.621508	3.121217	3.229995	3.076993	1.342143	1.429679	1.016409	3.334218	3.575637	3.968124	0.154837	0.443055	0.723739	0.875823	0.353764	0.757241
LOC107824165	2.822536	2.968147	2.595123	2.606249	2.017265	3.734858	2.777122	2.22614	2.038401	1.905459	2.131408	1.421563	2.235716	1.717273	1.61562	3.276384	5.191422	2.589933	1.26682	1.928504	2.425689	3.898403	3.11208	3.971308	1.166639	0.906312	0.577387	0	0	3.356186
LOC107822157	56.59526	53.41881	57.73442	34.08682	25.67452	42.66992	35.80854	35.36693	48.7554	22.21495	25.1881	28.07418	27.1081	31.06702	22.87611	44.31367	58.61832	47.07558	21.29695	21.92722	25.43981	34.94253	45.92552	47.34143	7.257418	7.737538	16.80227	12.8284	9.931543	8.318629
LOC107827533	0	0	0	0	0	0	0	0	0	0	0	0	0	0	0	0	0	0	0	0	0	0	0	0	0	0	0	0	0	0
LOC107770989	0.335768	0.459016	0.408626	0.27377	0.237687	0.324892	0.309785	0.2582	0.063047	0.261933	0.047088	0.141833	0	0.036631	0.103387	0.475408	0.32438	0.150197	0.306642	0.284036	0.091868	0.364531	0.266552	0.280755	0.212256	0.093439	0.119055	0	0	0
LOC107771131	20.66251	20.99404	22.9424	27.97392	24.4805	27.09948	21.99085	21.957	24.26218	19.30198	21.30672	20.04781	24.29041	20.67909	16.54418	21.67327	22.92523	23.1719	20.58514	20.71049	20.69207	24.02279	25.34751	31.27554	14.52536	13.00612	15.90471	18.62599	15.32554	19.68283
LOC107823308	0.191167	0.248893	0.237396	0.148447	0.096661	0.205528	0.297774	0.210006	0.333313	0.239673	0.153196	0.346078	0.547225	0.268141	0.05606	0.305267	0.606817	0.325767	0.277119	0.161714	0.174348	0.342104	0.264977	0.456703	0.241693	0.075999	0.41961	0	0	3.377184
LOC107763184	5.804244	6.289764	6.553005	6.508812	5.762315	5.17486	7.272719	7.197038	6.479327	5.107688	4.36774	4.783987	4.767227	7.2588	5.957317	6.487994	5.641579	5.672998	3.806787	4.191456	5.196735	5.58619	5.80652	6.806423	2.237295	3.988846	3.220924	5.062018	4.089321	10.94162
LOC107778487	19.70017	19.40899	15.1532	7.87719	6.72749	7.767538	10.95687	11.30531	8.636438	7.208926	11.39857	7.026361	11.64327	9.85237	12.41637	14.55538	20.81754	14.84389	5.583664	6.00508	6.818988	7.962968	10.44834	8.209902	2.124264	4.792602	2.780181	3.003924	4.448954	2.597209
LOC107766511	1.100294	1.768063	1.258502	0.209855	0.076864	0.715996	0.170001	0.14844	0.135922	0	0	0	0	0.031589	0.089156	0.755208	0.895138	0.62603	0	0.048988	0.026408	0.169268	0.306484	0.121055	0	0	0	0	0	1.790337
LOC107816692	1.877194	4.690987	2.76354	4.224176	2.304057	2.14842	17.21774	14.09902	8.328724	6.52485	10.91246	2.967178	10.34748	5.813468	11.80881	6.859059	2.369285	4.356594	3.287396	5.020056	3.258384	10.61986	7.931859	2.626605	2.143665	2.317077	3.721677	6.495769	2.186487	1.872092
LOC107773063	7.448276	6.259236	6.558715	7.273561	8.259805	8.059807	7.139646	6.322685	8.809074	6.885711	7.630621	9.030162	8.668413	9.128225	8.091475	5.550464	5.373383	5.985708	4.564244	5.359711	5.734311	7.028075	6.612534	7.987142	1.895607	2.2208	4.344468	0	3.027029	5.981015
Novel02390	0.89157	2.539239	2.131314	2.423153	1.18338	2.875642	3.084669	2.856682	2.092614	2.282159	2.813264	1.788906	1.612826	1.215824	3.774719	2.325398	2.691662	2.492638	1.470144	1.791242	1.422973	3.25751	2.752484	1.863738	1.549922	0.775347	1.185485	0	2.682711	0
LOC107766442	9.78793	12.90485	10.39525	21.29653	21.58003	21.97108	18.42857	15.58016	13.86091	13.79755	20.92733	8.502658	14.57747	13.06845	22.62466	14.94655	10.11867	13.80842	18.71103	23.7398	7.824919	19.90527	13.62773	11.89973	9.656579	16.61085	6.909665	3.660959	6.457174	10.76197
LOC107795986	18.28156	22.90161	25.89109	9.552713	9.451573	5.668272	35.26139	33.40513	45.70515	3.271591	4.705117	3.007971	10.44242	16.2094	4.567941	29.84912	24.40863	26.03422	11.39454	10.02036	16.23597	24.93105	27.23829	23.25866	4.06866	5.144655	2.508717	24.48296	14.50417	12.70086
LOC107763690	3.676889	3.977175	3.749224	3.999595	3.152231	2.831463	1.557989	1.315355	2.17394	0.521066	0.57988	0.290211	0.679833	0.846677	0.509275	2.787427	2.826997	2.409288	1.923622	1.517528	1.462029	1.50877	2.188358	2.712765	0.402137	0.265543	0.300746	0	0.490017	1.57334
LOC107831680	8.850293	9.997534	8.381726	5.274946	3.833226	5.659009	5.723618	5.989845	6.428577	2.43007	4.523724	2.223416	2.722338	2.134315	4.015951	8.903586	8.257623	7.761648	3.568633	4.35413	2.52769	10.96996	11.97688	8.39832	2.296784	1.55552	1.761741	0	1.457658	4.320213
LOC107791298	4.718468	4.864988	4.101664	7.426739	7.043033	6.353973	6.476044	5.741957	5.369583	10.64295	7.352428	7.771258	9.125841	6.811493	4.74507	3.267587	2.57851	3.269084	4.594522	3.991499	4.34679	3.462878	2.732627	3.138381	11.44909	4.642205	12.95632	10.22537	12.84967	11.78788
LOC107790728	4.845337	5.71025	6.826261	1.286616	0.739219	2.963929	0.350344	0.499654	0.392157	0.244387	0.41005	0.49404	0.650988	0.637968	0.25723	3.673005	5.730193	5.294041	1.017251	0.989371	0.838092	0.767433	1.91589	0.611214	0.10562	0	0.49369	0	0	0
LOC107767888	0	0	0	0	0	0	0	0	0	0	0	0	0	0	0	0	0	0	0	0	0	0	0	0	0	0	0	0	0	0
LOC107802005	2.078377	1.93675	1.826989	0.655843	0.661244	1.255342	0.861819	0.778169	0.723505	0.569292	0.638618	0.434035	0.389946	0.535006	0.215716	1.792442	1.7146	1.497287	0.592414	0.474112	0.276873	0.663079	1.235909	1.000731	0.265722	0.032493	0.248407	0	0.224855	0
LOC107823182	0	0.117871	0	0	0	0	0.108477	0	0	0	0	0	0	0	0	0	0.124946	0	0	0	0	0	0	0	0	0	0	0	0.622654	0
LOC107831243	2.252893	2.117398	1.795193	1.764621	1.578194	2.396905	2.027382	1.644214	1.894791	1.510338	2.468328	1.486958	1.854839	1.587326	1.589711	1.766299	1.428318	2.099534	1.023973	1.052156	0.749101	1.332694	1.780201	2.354723	0.771426	0.228575	0.915316	1.678268	0.903852	1.451039
LOC107785035	0	0.032464	0.015482	0.03227	0.063039	0.019148	0.074691	0.045653	0.050163	0.034734	0.049954	2.151655	0.05948	0.13601	0.05484	0.013272	0.017206	0.026557	0.126508	0.060265	0.162432	0.089243	0.094259	0.093076	0.045035	0.049563	0.063151	0	0	0
LOC107796368	0	0.394526	0	0	0.191525	0	0	0	0	0.211061	0.151772	0.182859	0.24095	0	0	0.161295	0	0	0	0	0.394804	0	0	0	0	0	0	0	0	0
LOC107766980	0	0	0	0	0	0	0	0	0	0	0	0	0	0	0	0	0	0	0	0	0	0.044773	0	0	0	0	0.126732	0	0	0
LOC107764333	0.778468	1.266924	0.676706	0.705252	0.68884	0.657602	0.419744	0.427593	0.574247	0.32533	0.935764	0.281859	0.928501	1.031257	0.513642	0.662989	0.429752	0.78766	0.507816	0.89372	0.304275	1.160926	0.980943	0.348709	0.351507	0	0.262882	0	0.267702	0
LOC107798418	10.98489	12.14682	13.77586	4.06287	2.920503	4.577766	2.303374	2.217284	2.696543	1.793464	1.625321	1.638958	2.664473	2.418783	1.163661	10.13856	10.61232	10.6128	3.144597	2.919878	1.378679	2.503849	3.422401	2.923017	0.477806	0.210339	1.310236	0	1.091666	0
LOC107802482	1.728266	0.72697	2.08017	1.083961	3.176205	1.286375	1.003549	1.703859	1.372946	0.259274	3.169484	1.01083	1.627941	1.595384	1.364496	1.386976	3.59617	2.279646	2.967843	4.948288	3.75865	1.332296	2.3453	2.640099	3.361621	4.994494	5.028115	9.507344	1.920113	0
LOC107793469	2.575257	3.58697	3.010969	3.7354	3.421207	3.298579	7.393999	5.664025	4.125189	2.396133	5.008462	1.888299	3.461811	3.263842	5.934673	4.226125	3.352985	3.379343	2.923114	4.086872	2.08279	7.854853	3.783871	2.582831	1.246197	1.815787	0.705559	1.985631	1.804591	6.008756
LOC107763325	1.643287	1.497653	1.517754	0	0.090881	0.110421	0.086143	0.087754	0	0	0	0	0	0	0	2.21955	2.182885	1.301711	0	0.086882	0.187338	0	0	0	0	0	0	0	0	0
LOC107805706	0	0	0	0	0	0	0	0	0	0	0	0	0	0	0	0	0	0	0	0	0	0	0	0	0	0	0	0	0	0
LOC107806201	7.047136	6.287667	5.714099	4.989448	5.685554	5.411809	5.265026	5.793569	5.698795	4.128883	3.86181	3.577173	3.856561	4.134765	3.160633	4.876424	4.862947	6.159029	4.687175	4.233089	4.671804	6.45399	5.223563	6.963338	5.053834	2.224794	5.66942	8.470082	5.702087	10.98492
LOC107804828	0	0	0	0	0	0	0	0	0	0	0	0	0	0	0	0	0	0	0	0	0	0	0	0	0	0	0	0	0	0
LOC107798945	3.899096	4.46806	3.289713	16.75285	21.63971	12.85343	33.23242	25.6476	27.72398	17.89228	15.66047	17.2454	55.85238	40.84824	32.72162	3.909755	4.431593	3.997044	6.225721	5.747889	6.04004	19.20222	14.2881	15.70202	4.385932	2.991897	5.997719	6.150893	26.77718	1.772698
LOC107773483	0	0	0	0	0	0	0	0	0	0	0	0	0	0	0	0	0	0	0	0	0	0	0	0	0	0	0	0	0	0
LOC107784155	0	0.02742	0	0	0	0.032346	0.025234	0	0	0	0	0	0	0.032822	0	0	0.029066	0.02243	0	0	0	0	0	0.062892	0	0	0	0	0	0
LOC107784397	0	0	0	0	0	0	0	0	0	0	0	0	0	0	0	0	0	0	0	0	0	0	0	0	0	0	0	0	0	0
LOC107794215	10.33445	13.33339	9.642459	8.95045	9.815358	9.019037	9.409708	7.750571	9.061937	10.81657	11.56964	9.029689	13.78285	12.23918	7.002176	0	0.024411	0	0	0	0	0	0	0	0	0	0	1.806992	0.121647	1.562335
LOC107786509	7.723998	8.019824	9.090939	9.921092	8.738371	10.39235	8.39567	7.556981	12.65178	4.297684	6.411288	5.111811	5.987324	5.003745	3.956183	4.976599	5.268165	5.357585	4.699636	4.688788	3.774281	7.39875	8.091352	9.291115	0.434441	0.935459	1.35966	0	1.006973	0.923765
LOC107776965	4.407272	4.649617	3.204233	1.790695	2.458084	1.981495	1.164979	1.369343	1.429402	2.396264	3.109112	2.640219	2.289577	2.389489	1.919062	4.299453	6.347986	5.914455	1.355211	1.604277	1.437278	1.093035	1.272275	0.893378	0.168852	0.222996	0.28413	0	0.900165	0
LOC107816701	0.122526	0.167501	0	0	0	0	0.154151	0	0	0	0	0	0	0.467844	0	0.045653	0	0.091348	0.062165	0	0	0	0	0	0	0	0	0	0.294941	3.787976
LOC107768670	13.52894	10.47501	10.14727	14.697	12.55397	13.96603	10.84521	12.73591	18.09693	13.60104	12.29887	16.28469	15.32717	16.52271	10.44502	9.769517	10.06282	11.38067	9.779851	9.722808	12.77539	10.94843	10.93017	13.89018	9.536248	8.579325	14.92883	25.68613	9.510593	7.402788
LOC107761321	0	0	0	0	0	0	0	0	0	0	0	0	0	0	0	0	0	0	0	0	0	0	0	0	0	0	0	0	0	0
LOC107761306	0.067068	0.48899	0.466403	0.486078	0.296729	0.504741	0.337515	0.343826	0.503728	0.588594	0.846501	1.303192	1.567871	0.731674	0.688363	0.299873	0.647929	0.350012	0.544444	0.62408	0.672834	0.224039	0.532422	0.771089	0.67835	0.373278	1.189027	0	1.614435	0
LOC107806414	0.437409	0.166102	0.380231	0.561383	1.128891	0.666214	0.244583	0.280301	0.444881	0.568706	0.332271	1.20099	0.97386	1.19298	0.18706	0.380284	0.211287	0.163054	1.331558	1.202559	1.296505	0.152205	0.257216	0.68577	0.783443	0	0.560064	0	0.350974	0
LOC107781686	0.153456	0.093238	0	0.046341	0.045263	0.054995	0.171614	0	0	0	0	0	0	0.111609	0.052501	0	0.049417	0.152544	0.051906	0.043271	0.186606	0.042718	0	0.053464	0.064672	0.569395	0.423204	0	0.246264	0
LOC107828583	54.69211	40.40753	52.262	42.53501	49.55168	83.12085	44.01749	39.17109	43.70189	44.80256	39.83057	57.07728	46.78221	53.63396	35.49681	33.11476	58.47055	41.67475	33.66593	34.35913	44.74541	25.86	35.73814	41.82232	25.04053	14.68844	41.82697	21.56944	22.74898	6.216352
LOC107765717	0	0.040082	0	0	0	0	0	0	0	0	0	0	0	0	0	0	0	0	0	0.037204	0	0	0	0	0	0	0	0	0	0
LOC107765899	4.540929	4.929293	5.003308	4.271069	3.506251	3.544931	3.008124	3.459777	3.85591	4.568998	2.859608	3.249862	4.121295	5.616558	3.681051	4.526243	3.604539	3.946104	3.786044	3.30304	3.059891	4.106256	4.107448	3.929956	11.11655	6.117149	9.093155	12.41047	18.65899	23.24868
LOC107762729	0.593875	0.86599	0.929237	1.398853	1.156099	0.638488	0.796973	0.913361	1.115112	1.96895	2.498552	2.709299	4.892212	4.016907	3.901036	0.177022	0.573733	0.354207	0.843672	1.507132	2.166495	0.595152	0.838139	1.73801	0.150168	0.9916	0.982677	0	0	0
LOC107814611	0	0	0	0	0	0	0	0	0	0	0	0	0	0	0	0	0	0	0	0	0	0	0	0	0	0	0	0	0	0

Novel05037	4.093033	2.671698	2.404045	2.50546	3.083412	3.746381	2.969084	2.457491	4.465864	1.833806	2.249489	1.962596	2.401357	2.956751	1.646329	1.772375	3.20612	2.350503	0.673513	1.076159	1.765568	1.801483	3.463961	4.162414	0.06993	0.230885	0.784481	0	0.532576	0
LOC107819536	0.126521	0.345925	0.24746	11.72008	18.52843	4.046765	33.13558	39.29548	7.216106	28.71532	25.81656	18.67878	26.19719	19.49651	52.30025	0.683557	0.183345	0.212236	16.62585	22.12808	15.92374	22.84922	9.625503	4.628396	94.29713	98.62919	21.08483	95.00455	38.67899	101.6989
LOC107826383	9.881714	9.097241	8.299763	7.833291	7.030641	7.393765	7.700204	7.330755	7.835632	7.129278	8.076105	7.474027	7.618563	8.376712	6.133363	9.752145	9.062788	9.0846	7.960848	7.229622	6.941899	6.774838	7.980149	7.990525	4.600644	5.574196	3.748453	7.16232	8.35761	10.32097
107810630	0	0	0	0	0	0	0	0	0	0	0	0	0	0	0	0	0	0	0	0	0	0	0	0	0	0	0	0	0	0
LOC107828629	0.121063	0.331001	0.140316	0.073118	0.035708	0.130157	0.067694	0.03448	0	0.157402	0.056593	0.034092	0.044923	0.264147	0.041418	0.060144	0.077971	0.060172	0	0.034137	0.036804	0.033701	0.035595	0	0.05102	0	0.047695	0	0	0
LOC107759484	5.439617	4.786591	5.971825	16.71703	21.9297	13.68794	13.78565	18.47083	12.30511	24.6239	19.2686	24.26524	26.38822	23.68626	26.76025	7.652934	5.30043	6.799897	19.03361	19.57639	20.4856	12.96255	9.699611	10.95436	19.65375	28.97035	22.16962	25.15148	31.49363	4.349221
LOC107810819	3.461218	3.364771	3.8111	6.759149	7.758873	8.104017	7.35445	5.388964	4.043877	10.05037	13.21371	7.342823	13.44295	9.146356	16.73622	5.330561	4.829973	4.931593	6.790262	9.629709	4.910405	7.579673	4.138542	4.743159	7.00165	6.421376	7.181769	11.00116	1.851504	23.77914
LOC107813863	0	0	0	0.039611	0.096722	0	0.073344	0.112074	0	0.063953	0.061317	0.036938	0	0	0.269255	0	0	0	0.177467	0.221918	0.319007	0.018257	0.057849	0.068548	0.055279	0.121674	0.025838	0	0.315745	0
LOC107806681	2.271683	2.627763	2.468407	0.751972	0.811785	0.610584	0.512981	0.67188	0.943334	0.170398	0.490125	0.701239	0.486321	0.857871	0.224192	1.399866	1.899205	1.400507	0.487627	0.406511	0.557796	0.401318	1.11749	1.187181	0.165698	0.243145	0.206534	0	0.420642	0
LOC107791899	69.81998	62.77022	49.64391	28.86838	32.48619	29.55566	32.82591	31.21715	42.01643	30.61154	29.05963	40.00649	53.63719	53.40296	25.54565	46.61106	73.04917	56.32913	29.69517	24.2553	34.13001	25.37727	28.26378	27.92978	28.55253	20.48281	42.55347	84.55742	60.05518	29.24352
LOC107773028	2.721424	2.145827	2.445418	3.518145	2.841011	2.399865	2.487751	3.0568	2.727239	4.353313	3.323381	3.151622	3.67628	2.768786	2.824579	2.028008	2.806368	2.667254	3.009739	2.612538	3.234946	1.915224	2.373504	2.396993	4.097921	2.382619	4.770545	0	5.741283	7.562684
LOC107821758	0	0	0	0	0	0	0	0	0	0	0	0	0	0	0	0	0	0	0	0	0	0	0	0	0	0	0	0	0	0
LOC107805967	22.44417	16.64376	20.89636	16.02677	16.23948	15.36442	23.21569	17.89159	20.25244	11.47103	13.90249	16.47054	51.20954	37.58161	22.91256	22.12868	33.54173	24.31456	12.24222	10.56204	13.55484	20.47738	18.97457	17.54681	5.249227	3.307138	5.405007	25.81891	14.48448	18.60264
LOC107809231	0.21336	0.064817	0.061823	0.193293	0.031466	0.038231	0.208779	0.121533	0.033385	0.346754	0.224412	0.120168	0.316686	0.698294	0.291982	0.211994	0.137415	0.159069	0.180419	0.330894	0.064862	0.415758	0.282296	0.260171	0.179834	0.049479	0.42029	0	0.513594	0
LOC107815015	109.3898	82.06839	81.42204	85.94922	120.1405	91.21314	66.50655	72.28336	69.91566	111.6072	95.5082	131.4925	177.1847	165.9063	93.11669	94.81608	117.5617	121.2174	171.7876	126.7681	167.1553	68.19346	71.8137	85.39774	156.5958	116.7061	115.0954	124.1733	316.9601	110.3431
107821895	0	0	0	0	0	0	0	0	0	0	0	0	0	0	0	0	0	0	0	0	0	0	0	0	0	0	0	0	0	0
LOC107783174	0	0	0	0	0	0	0	0	0.132909	0	0.2978	0	0	0	0	0	0	0.105544	0	0	0	0	0	0.147966	0	0	0	0	0	0
Novel00374	0.755946	0.711176	0.445151	0.8174	0.539444	0.47191	0.449967	0.750079	0.434987	0.784701	0.991744	0.968267	0.705799	1.010923	1.101253	0.981288	0.659633	0.709033	0.420658	0.556965	0.867355	0.488757	0.623776	0.637189	0.431627	0.475026	0.259394	1.743898	2.347996	0
LOC107828574	0.468437	0.52395	0.592294	0.27006	0.452186	0.297598	0.267885	0.473017	0.659684	0.290681	0.38819	0.287817	0.213329	0.209062	0.349666	0.698159	0.349697	0.380989	0.3457	0.216145	0.25245	0.462332	0.601007	0.71216	0.161522	0.118508	0.176163	0	0.10251	0
LOC107800518	1.626056	2.380579	1.789426	0.955964	0.887796	0.985692	0.827011	0.487749	0.87699	0.961487	0.897598	0.75994	0.789533	0.490666	0.639168	2.010971	1.838295	2.050582	0.930324	0.804832	1.041249	0.92457	0.823953	0.705134	1.049785	0.361043	0.899597	0	1.165934	1.06959
LOC107776952	0.125711	0.07638	0.091065	0	0	0.045052	0	0.035804	0.059012	0	0.014691	0.017701	0.046648	0.022857	0.043009	0.062454	0.020241	0.078103	0	0.035448	0.019108	0.017497	0.073924	0.021899	0	0	0	0	0.10087	0
LOC107817374	0.099866	0	0	0	0	0	0	0	0	0	0	0	0	0	0	0	0	0	0	0	0	0	0	0	0	0	0	0	0	0
LOC107824485	0	0	0	0	0	0	0	0	0	0	0	0	0	0	0	0	0	0	0	0	0	0	0	0	0	0	0	0	0	0
LOC107817314	18.83012	16.00439	18.68072	18.94851	16.5284	22.55186	16.04714	13.68724	14.472	14.41235	15.49261	15.34644	17.02527	14.9042	12.84311	16.82516	17.98708	18.95105	12.88146	14.13921	13.03854	15.57407	17.4892	19.83919	8.292323	5.929861	12.9676	2.161508	9.458375	9.344257
LOC107791536	7.598152	9.78125	10.11377	14.06824	20.33812	14.85726	10.47545	13.26816	10.34355	21.16241	16.94916	24.27234	22.46754	19.53145	20.27619	6.546863	7.84511	7.364168	28.62367	24.22369	20.78893	11.38209	8.377591	10.47297	27.55829	33.03836	22.11425	27.16877	25.83477	29.36282
LOC107818352	7.216767	9.123204	10.32073	19.08682	30.48738	18.64641	8.005598	12.73022	5.354767	38.36441	23.50638	38.64664	33.69015	26.15914	13.38055	2.862454	3.598454	3.905134	6.023821	6.498752	5.626389	2.624622	2.156113	1.459928	8.388366	18.13967	14.58293	16.64842	8.405827	7.197155
LOC107807040	0	0	0.040046	0	0	0	0	0	0	0	0	0	0.051284	0	0.047283	0	0	0	0	0	0	0	0	0	0	0	0	0	0	0
LOC107766817	0	0	0	0	0	0	0	0	0	0	0	0	0	0	0	0	0	0	0	0	0	0	0	0	0	0	0	0	0	0
LOC107821531	0	0	0.119981	0	0	0	0.057883	0	0	0	0	0	0	0	0	0	0	0	0.058379	0	0	0	0	0	0.087252	0	0	0	0.332246	0
LOC107763938	2.620925	2.45925	1.952841	3.637666	3.176002	4.03933	3.411128	2.69168	2.181848	2.492783	4.635224	2.028802	2.960768	2.309976	4.28022	3.992824	2.332465	3.186096	3.052568	3.473127	1.259926	2.501489	2.57376	1.781278	0.652935	0.628763	0.778245	1.846632	0.870211	2.394912
LOC107825033	18.85671	19.25875	17.79675	25.59773	24.68418	22.65458	24.2009	24.60229	25.90895	29.24516	22.54094	28.11891	33.58649	29.45352	29.55327	21.14498	30.99811	23.65395	27.69945	24.61086	33.90392	20.59704	22.9164	24.33909	34.28508	34.56718	31.90949	64.21532	58.50456	103.639
LOC107792867	0.301033	0.438967	0.680372	18.6541	41.98053	14.36994	16.00783	19.95956	7.178628	49.02206	21.57285	33.31607	32.70715	33.95783	36.52051	0.44866	0.465317	0.718185	63.29289	46.44876	89.61223	18.40253	10.40882	7.991849	155.5121	80.0871	144.8093	202.3644	127.2468	316.4266
LOC107822833	0.601202	0.616412	0.391959	1.633977	1.130465	1.373528	1.638827	1.541051	1.058316	1.172497	1.528168	2.476074	2.091459	2.95147	1.774041	1.008038	1.669835	1.008499	1.601404	0.572148	1.919074	1.2552	1.193176	0.785471	0.855115	0.418265	0.799395	5.374309	1.447201	0
LOC107829180	7.194243	18.15195	25.04602	78.27565	131.9286	62.71577	45.03298	65.34548	40.326	148.5307	87.82091	117.0369	84.66374	83.35718	81.15097	36.89428	8.833315	20.39759	250.4731	199.9631	324.2819	68.42723	50.76639	86.12145	418.5819	333.1495	546.0361	261.0467	234.0892	170.9202
LOC107773101	0	0	0	0	0	0	0	0	0	0	0	0	0	0	0.099028	0	0	0.086712	0	0	0	0	0	0	0	0.121152	0	0	0	0
LOC107793440	1.902333	0.941601	1.58238	1.010279	0.957745	1.639719	0.522686	0.350303	1.216324	0.431771	0.505969	1.510161	0.346865	0.536729	0.235647	1.038774	1.552636	1.124835	0.316179	0.180346	0.299131	0.301303	0.665407	1.336973	0.041468	0	0.058148	0	0	0
LOC107765250	843.9287	470.443	668.49	34.81684	32.53928	38.9064	66.59515	60.83971	85.99007	116.5162	130.4314	204.4486	362.6723	813.4205	136.8557	235.6545	679.8929	333.4271	10.88762	5.115828	22.1509	50.50484	43.4492	13.96719	1.356543	0.950053	22.01966	48.82915	2.817596	0
LOC107806056	0	0	0	0	0	0	0	0	0	0	0	0	0	0	0	0	0	0	0	0	0	0	0	0	0	0	0	0	0	0
LOC107821007	0	0.051553	0	0	0	0.182445	0.094888	0.096662	0	0.165476	0.079328	0	0	0.123421	0.058057	0	0	0.042172	0	0.047851	0.103178	0	0.049895	0.059122	0	0.078707	0	0	0	0
LOC107785705	0	0	0	0	0	0	0	0	0	0	0	0	0	0	0	0	0	0	0	0	0	0	0	0	0	0	0	0	0	0
LOC107826823	0.156591	0.713568	0.272243	0.283727	0.069281	0	2.167107	1.538652	0.514552	3.359317	5.984201	0.198439	2.789112	4.527083	7.634244	0.116692	0.22692	0	0.079449	0	0	1.373121	0.690619	0	0.197979	0	0	0	0.753886	0
LOC107821561	2.477421	2.534337	3.076528	1.969591	1.700062	2.228669	1.9507	0.734392	1.851236	1.133946	1.205383	1.53771	1.125673	2.151164	1.245432	1.620115	2.295707	2.751687	1.180001	1.112019	0.73778	1.520052	1.917668	3.223539	0.511382	0.4221	0.836604	0	0.973648	0
LOC107820158	58.70608	50.81597	59.14113	42.79064	38.52143	46.86163	30.89261	29.36311	40.8713	27.44664	24.81162	35.82726	37.0032	40.18194	26.02766	41.23084	58.32064	51.80198	31.44472	27.25703	33.34695	26.28204	39.01432	47.85487	14.91225	10.14538	27.56425	19.17039	32.52203	16.57482
LOC107799523	0.049885	0.090929	0.043364	0	0	0	0	0.042624	0	0	0.069959	0.042145	0	0.054423	0	0.148699	0.096387	0.074383	0	0.0422	0.090993	0.041661	0	0	0	0	0	0	0	0
LOC107782151	50.88768	47.11741	47.73984	43.77761	57.6989	38.95026	44.30641	47.3939	34.35714	42.245	40.05101	39.60775	46.94514	44.09292	58.70471	45.23668	45.97537	52.10748	72.99348	68.9114	63.33686	49.75546	42.80823	46.01948	73.40565	72.40912	33.81618	151.6329	94.64432	61.50492
LOC107795348	3.408018	4.051289	4.894595	4.094283	3.671213	4.779178	4.474099	3.67153	3.75605	2.456314	4.156008	2.503642	3.876329	2.505596	3.878103	3.975119	5.654395	3.921704	2.480898	3.384332	1.959499	4.269188	3.790297	2.942571	1.124027	0.927784	0.963218	0	0.356683	0
LOC107797210	2.014606	1.787748	2.85731	2.16134	2.064128	3.704911	2.801406	2.310212	3.08597	1.861101	3.08551	2.015521	3.71814	2.429188	2.230979	1.61981	2.15115	2.055333	1.882892	1.973305	1.112083	2.435125	3.086396	3.93427	0.938396	0.295071	0.501285	0	1.276188	0
LOC107759095	17.38539	15.53895	17.59104	15.29448	15.23534	22.47778	17.11377	16.47851	17.89665	13.7913	13.32739	15.34884	15.5576	19.33252	12.96694	12.58069	19.92807	15.00371	10.72103	10.02524	12.64382	13.02507	17.50465	16.82735	7.20901	5.055891	10.24103	3.997752	12.64911	13.82591
LOC107816370	0.254683	0.464224	0.590374	0.769099	2.704321	0.821445	0.569636	3.336654	2.470777	3.973566	3.035923	3.084004	2.173626	2.130156	3.31106	3.732535	0.246045	1.582307	5.427125	6.176065	7.045675	2.48141	1.273	1.597166	10.94788	5.433712	11.0372	6.07114	6.539379	0
LOC107826692	0	0	0	0	0	0	0	0	0	0	0	0	0	0	0	0	0	0	0	0	0	0	0	0	0	0	0	0	0	0
LOC107827978	0	0	0	0	0	0	0	0	0	0	0	0	0	0	0	0	0	0	0	0	0	0	0	0	0	0	0	0	0	0
LOC107778967	0.036913	0	0.128351	0.167207	0.09799	0.079372	0.092882	0.03154	0	0	0.025884	0.031185	0.082184	0.04027	0.075773	0.082523	0	0.082561	0.262198	0.343486	0.033665	0.123309	0.03256	0.115745	0.280017	0.308172	0.087257	2.639812	0.177713	0
LOC107813582	0	0	0	0	0	0	0	0	0	0	0	0	0	0	0	0	0	0	0	0	0	0	0	0	0	0	0	0	0	0
LOC107820606	0	0	0	0	0	0	0	0	0	0	0	0	0	0	0	0	0	0	0	0	0	0	0	0	0	0	0	0	0	0
LOC107785127	4.818316	3.541372	1.891563	0.633651	0.584521	0.459541	0.879967	1.460836	0.984988	0.568363	0.953639	0.984834	0.605592	0.932613	0.55835	3.590615	2.440068	3.50535	0.512587	0.98612	0.602456	1.59009	1.850839	1.015343	0.147383	0.540672	0.321484	0	0.56122	0
LOC107813227	1.741348	3.935821	5.812674	1.640676	3.636429	0.823752	1.927918	1.368827	0.980913	2.241406	1.221035	3.707272	0.697858	1.899726	0.857891	14.84517	14.06394	13.44986	11.09676	10.95957	11.81575	10.81959	13.08659	14.48765	6.780909	7.462715	8.726487	9.962483	9.054149	8.613616
LOC107773626	5.310295	5.76242	4.31078	11.49365	12.57915	7.242042	12.14878	11.63445	7.759594	12.1295	12.85143	10.35162	13.54813	12.93906	12.87299	8.207035	5.249963	5.607371	15.28495	14.24552	12.30557	11.90657	8.803063	7.731573	18.10382	17.82533	11.79562	2.95534	19.19911	10.22081
LOC107760690	0.173731	0.277084	0.188775	0.35413	0.768641	0.046695	0.255002	0.25977	0.081553	0	0.213185	0.036693	0.04835	0.047383	0.044578	0.032366	0.209798	0.161904	0.925521	0.698079	1.584452	0.398989	0.344794	0.226979	0.109824	0.120867	0.051334	0	0.2091	0
LOC107816685	3.656451	6.303463	5.361284	4.869063	4.911675	2.604967	6.318394	5.796655	4.632262	2.706344	5.405825	1.30262	2.255891	3.075862	5.878006	6.729907	3.319642	5.649142	4.023232	4.024764	3.013324	9.013632	4.934993	3.453363	2.617767	6.252335	1.4579	0	2.757168	0
LOC107797618	18.12242	21.48937	17.55966	12.53857	10.90378	11.46107	16.8216	15.5306	13.92682	9.989856	14.29113	8.746577	12.1085	11.03846	15.70738	31.99155	23.73937	27.53395	11.00063	11.78433	9.063185	19.37458	16.25611	13.42716	4.385994	7.969572	5.637767	3.876399	4.871262	1.117185
LOC107803031	0	0	0	0	0	0	0	0	0	0	0	0	0	0	0	0	0	0	0	0	0	0	0	0	0	0	0	0	0	0
LOC107801189	10.45842	10.41017	9.726153	12.73011	12.0978	11.84084	11.85926	10.7581	11.629	10.99592	12.08586	11.25424	14.30917	15.42531	10.67427	10.62372	8.099907	10.73748	10.49391	12.33158	10.28427	10.24671	10.0496	10.99221	10.26785	11.46285	10.35839	6.26753	7.735424	9.031566
LOC107821537	26.30791	26.12784	24.46495	11.1683	8.982016	5.809123	9.327822	10.26422	14.92284	8.031117	8.920095	7.0023	11.97148	13.5348	11.89906	21.91112	23.56586	16.11332	4.817424	4.792634	5.76508	9.397121	16.65786	10.49986	4.708963	3.759093	4.619124	1.87576	8.460547	8.108962
800467	0	0.663024	0	0	0	0	0	1.243186	0	0	0.510121	0	0.809859	0	0	2.168526	7.02823	1.084759	0.738213	0	0.663489	1.822654	0.6417	0.760379	0.919777	1.012259	0.859843	0	0	0
LOC107812141	11.13329	12.03562	12.63794	24.11567	21.11777	20.02369	20.93593	20.54314	17.93035	18.79652	22.67256	25.04021	24.85339	24.55017	20.63529	11.38568	12.329	11.78825	16.31497	17.95932	19.68639	13.13053	15.56622	14.11237	12.35382	13.34877	15.11845	21.1752	9.978653	5.492458
LOC107796145	0	0	0	0.019259	0.01881	0	0	0	0	0	0	0	0	0	0	0	0	0	0.038775	0	0	0	0	0	0	0	0	0	0	0
LOC107784063	0	0	0	0.037365	0	0	0	0	0.038722	0	0	0	0	0	0	0	0	0	0	0	0	0	0	0	0	0	0	0	0	0
LOC107785124	0.884334	1.053949	0.768732	0.677906	0.84271	1.389582	0.28528	0.639353	0.638655	0.199001	0.476996	0.45976	0.151454	0	0.279279	1.064549	0.328593	0.76074	0.414166	0.978266	1.17877	0.5681	0.660034	1.279807	0.34402	0.378611	0.402004	0	0.3275	0
LOC107770876	53.03075	53.62504	51.36567	73.53918	62.52249	77.84989	76.86203	72.65149	79.87643	60.45237	58.81511	68.11236	76.09272	70.58291	58.43827	42.4293	48.81327	50.92354	47.20613	51.25689	50.60279	52.735	61.61794	64.43015	22.72887	26.33808	42.37725	28.64928	24.83175	12.38516
LOC107793037	0.116056	0.035257	0.067256	0.035047	0	0	0	0	0	0	0	0	0	0	0	0.028828	0	0	0	0	0	0	0	0	0	0	0	0	0	0
LOC107784329	0	0	0	0	0	0	0	0.230101	0.126418	0	0.094418	0	0	0	0	0	0	0.136636	0.34172	0	0.112452	0	0	0.170241	0.374718	0	0	0	0	0
Novel02853	0.366292	0.66766	0.31841	1.57625	1.377507	0.098452	2.073768	1.721334	1.633483	1.8752	2.118965	1.392542	0.917463	1.698328	1.033868	0.887124	0.884672	0.546173	2.230127	2.94365	0.417581	2.1413	1.453922	0.861409	2.315522	3.822515	1.190551	0	3.086055	5.662097
LOC107818550	2.075847	3.149548	2.194096	5.00072	4.571242	4.526983	6.509349	5.603154	4.750436	3.898911	5.350931	2.550885	5.278219	4.078939	5.459644	3.111426	2.210545	2.488522	4.404346	4.829066	2.753757	6.05773	4.171846	3.796931	2.07276	2.166293	1.826173	3.373919	2.271334	3.646385
LOC107794004	0	0	0	0	0	0	0	0	0	0	0	0	0	0	0	0	0	0	0	0	0	0	0	0	0	0	0	0	0	0
LOC107763962	10.01937	9.015836	8.213511	16.71785	15.48709	17.93069	12.2332	11.26995	11.78805	14.03687	12.40595	17.7329	14.96573	15.01234	9.046973	7.560961	9.434505	8.320866	20.65567	16.03946	25.33147	9.903236	12.13792	13.18973	18.92113	17.64712	25.93268	40.81482	26.25549	15.68387
LOC107828026	2.693188	3.473047	2.580022	3.137004	2.804613	2.619744	3.211623	2.942946	3.061701	2.322489	3.327296	2.306236	2.875723	2.678301	3.591598	3.276681	3.274432	3.469409	2.249498	2.820693	1.570652	3.258975	3.215908	3.427691	1.436126	1.30011	1.169314	0	1.764077	1.132816
LOC107782297	0.844446	1.088446	0.356543	0.163934	0.053373	0.220486	0.293427	0.175226	0.124583	0.082344	0.329899	0.071341	0.120863	0.131607	0.123817	1.204625	0.675953	0.674539	0.110171	0.102049	0.077015	0.251864	0.117049	0.151305	0.015252	0.117498	0.014258	0	0	0
LOC107761843	0	0	0	0	0	0	0	0	0	0	0	0	0	0	0	0.05444	0	0	0.07413	0	0	0.061009	0	0.076356	0	0	0	0	0	0
LOC107796164	0.919346	0.942605	0.919044	0.562195	0.305062	0.889569	0.134942	0.176741	0.409983	0.067236	0.112813	0.077669	0.025586	0.376111	0.094359	0.411059	0.5329	0.599735	0.2099	0.252754	0.167692	0.287914	0.527101	0.264248	0	0	0	0	0.331955	0
LOC107770867	0	0	0	0	0	0	0	0	0	0	0	0	0	0	0	0	0	0	0	0	0	0	0	0	0	0	0	0	0	0
LOC107782303	0.192573	0.117004	0.1116	5.640908	4.771227	9.178736	0.107679	0.054846	0	1.064102	0.180043	0.216921	0	0.140058	0	0	0	0	0.047857	6.318238	6.244623	4.683453	0	0.226482	0.335461	0.24347	0.357268	0.455211	0	0
LOC107769734	0	0	0	0	0	0	0	0	0	0	0	0.301717	0	0	0	0	0	0	0	0	0	0	0	0	0	0	0	0	0	0
LOC107791266	0	0	0	0	0	0	0	0	0	0	0	0	0	0	0	0.570526	0.739633	1.046443	0.12948	0.05397	0.52368	0.266405	0.281379	0.466786	0.241988	0.443865	0.075406	0	0	0
LOC107787131	1.54285	1.265506	1.475284	0.698872	0.409565	1.285533	0.647027	0.593214	0.506973	0.677013	0.324554	0.619135	0.429381	0.547031	0.51465	0.94853	0.447157	0.891452	0.508813	0.424172	0.527664	0.35433	0.510336	0.403147	0	0.161007	0.045588	0	0	0
LOC107814758	0	0	0	0	0	0	0	0	0	0	0	0	0	0	0	0	0	0	0	0	0	0	0	0	0	0	0	0	0	0
LOC107797942	0	0	0	0	0	0	0	0	0	0	0	0	0	0	0	0	0	0	0	0	0	0	0	0	0	0	0	0	0	0
LOC107791305	0	0	0	0	0	0	0	0	0	0	0	0	0	0	0	0.092278	0	0.09232	0	0.104751	0	0	0	0.129426	0	0	0	0	0	0
LOC107807188	1.88965	2.26603	2.377494	4.399952	5.324275	3.225618	5.783606	5.806779	4.046162	3.555985	3.951824	3.38891	4.650028	4.321948	7.077417	2.902805	2.177862	2.471603	5.617897	6.253834	4.368951	5.703293	4.269337	3.967448	3.185455	5.512324	2.292188	1.185409	5.90536	10.24911
Novel01368	4.005767	2.896801	2.838692	4.260157	4.546292	5.008867	2.702456	3.125016	3.883425	1.995522	2.534068	2.280645	3.005164	2.660058	2.279151	2.920209	3.196881	3.343547	3.313676	2.983444	2.700285	3.272597	3.955818	5.82515	0.330294	0.181752	0.617543	0	1.048108	0
LOC107819496	1.435696	1.730544	2.858369	13.76191	28.68642	11.35256	17.79079	27.0665	23.78382	19.73528	20.71877	29.61863	22.47843	24.95934	22.95901	3.796358	1.476488	2.969421	58.36781	42.62504	77.54904	29.35586	22.05951	25.46161	60.01728	99.94778	48.16943	66.24038	72.24106	31.49948
Novel04272	0	0	0	0	0	0	0	0	0	0	0	0	0	0	0	0.238656	0.309395	0.318353	0.288866	0.451525	0.324533	0.297172	0.345263	0.669464	0.854793	0.792202	0.799093	2.544765	0.685258	0
Novel03317	1.730262	1.791955	2.803059	0.570011	0.487152	0.507339	0.92352	0.87359	1.329093	0.460152	0.882372	0.46511	0.612867	0.429007	0.403612	2.578787	2.507368	1.87634	0.478841	0.731841	0.071729	0.52545	1.734325	0.411016	0.198871	0	0.278868	0	0	4.862942
LOC107799554	114.5062	99.11802	115.3859	75.34102	69.61666	106.5983	68.81098	79.44407	96.44373	53.05979	47.98826	66.28896	32.94079	50.33545	38.57561	75.19297	108.6581	97.49764	55.00134	55.10534	79.17105	72.84762	102.4246	111.4734	19.68101	16.97667	44.5875	35.10208	18.2295	13.00691
LOC107806885	7.574661	6.164633	6.244334	17.87733	22.63041	12.0803	20.02077	26.62832	17.84417	29.40873	25.00846	26.30555	23.52309	24.85174	34.82713	9.997898	6.318656	7.18928	34.23356	31.4471	35.58625	20.26123	17.33207	16.03857	67.49612	59.65952	69.11042	35.97977	47.23232	51.84716
LOC107786638	1.456048	1.89243	1.463824	1.158519	0.918691	1.157057	0.966381	0.930361	0.736993	4.555806	5.877306	4.000532	1.677272	1.160279	1.143578	1.566245	1.883708	1.708555	1.901475	2.538408	1.189373	1.184263	1.261995	1.376292	3.729799	4.175291	4.249954	0	3.230666	6.262934
LOC107775376	0	0	0	0	0	0	0	0	0	0	0	0	0.100882	0.197729	0	0.067532	0.087549	0	0.091957	0	0	0	0	0	0	0	0	0	0	0
LOC107788842	3.585664	3.289971	3.07482	2.655811	2.572566	3.281982	2.418153	2.173563	2.843225	1.937236	2.157515	1.883059	2.211568	1.691582	1.666049	2.455389	3.042749	3.106767	1.69632	1.865023	1.922339	1.962599	2.991831	3.139994	0.949557	0.539372	1.202666	0	1.399675	1.49802
LOC107786365	0	0	0	0	0	0	0	0	0	0	0	0	0	0	0	0	0	0	0	0	0	0	0	0	0	0	0	0	0	0
LOC107782833	11.37583	9.877654	8.634224	5.10169	3.931282	4.685281	11.03669	9.16371	12.40702	6.208697	13.11598	11.2363	19.78315	15.43591	11.67584	12.35743	12.71232	11.03395	2.182336	2.585329	2.400175	8.484058	8.311941	11.15059	1.109099	1.41749	2.006764	0	3.678401	6.998854
LOC107761714	428.702	495.5839	500.2234	514.8879	350.7438	467.8481	260.3976	239.2815	296.4309	175.7203	148.5093	183.4566	106.4946	161.5529	139.9457	428.3695	396.6667	467.0116	248.7813	300.4814	282.9612	180.7734	176.3929	239.6574	21.92064	51.41332	60.46893	34.84552	37.33751	15.06381
LOC107765998	1.897054	1.200646	1.694876	0.859317	0.699432	0.963128	0.618777	0.630348	0.742099	0.719393	0.665108	0.979418	0.703942	0.344932	0.594942	1.452956	1.57817	2.160788	0.534722	0.490349	0.288357	0.924163	1.162033	1.432022	0.133247	0.073323	0.249129	0	0.761091	0
LOC107798367	0	0	0	0	0	0	0	0	0	0	0	0	0	0	0	0	0	0	0	0	0	0	0	0	0	0	0	0	0	0
Novel03522	0.906573	0.688524	0.569158	0.775681	0.980461	1.028825	0.337947	0.5164	0.709275	3.781654	2.260229	4.552837	3.027628	2.857187	0.723708	0.487918	0.97314	1.013833	0.971035	1.065136	1.148347	0.084123	0.310978	0.526416	2.674428	3.78429	7.440945	0	5.091992	6.228307
LOC107762134	0	0	0	0	0	0	0	0	0	0	0	0	0	0	0	0	0	0	0	0	0	0	0	0	0	0	0	0	0	0
LOC107767646	0	0	0	0	0	0	0	0	0	0	0	0	0	0	0	0	0	0.085079	0	0	0	0	0	0	0	0	0	0	0	0
LOC107761581	0.144896	0.36315	0.535307	0.853243	0.833386	0.584177	0.091148	0.216655	0.034009	1.236301	1.11761	1.438339	0.645199	0.553259	0.557688	0.296935	0.384949	0.270065	3.749266	2.819149	3.072423	0.242012	0.159759	0	4.076024	2.217728	1.327226	0	2.790316	4.479557
LOC107778837	30.45368	37.40125	34.7672	66.63147	70.16188	65.89751	71.30902	79.08655	60.99111	71.49649	72.68621	61.70162	60.56786	66.93002	98.53523	35.99977	33.14672	35.97366	111.8251	107.2036	84.87306	94.64482	75.0679	58.39017	116.2305	169.1982	70.29934	89.89327	75.64557	143.0795
LOC107828881	6.319365	6.159702	6.110183	7.102704	5.831011	5.994785	6.745866	5.861421	7.487508	5.074735	6.232035	4.99618	5.19146	5.53004	4.890531	5.33877	5.811212	5.441993	3.669159	4.745423	5.670907	5.390354	6.915458	7.52334	2.349882	1.927863	3.71452	0	2.765788	0
107779250	0	0	0	0	0	0	0	0	0	0	0	0	0	0	0	0	0	0	0	0	0	0	0	0	0	0	0	0	0	0
LOC107790911	0	0	0	0	0	0	0	0	0	0	0	0	0	0	0	0	0	0	0	0	0	0	0	0	0	0	0	0	0	0
LOC107806768	0.722027	4.30716	9.642872	4.043649	12.95218	4.234184	8.092951	17.10549	24.03359	11.00905	11.00021	26.11859	10.88743	13.46244	17.17932	26.46253	1.331665	16.10011	30.10578	33.37107	42.38349	24.94159	13.49018	29.50043	35.43562	63.2928	31.49739	84.49384	64.78175	73.05382
LOC107823752	22.68389	30.86311	22.02194	94.17497	98.15936	97.16969	76.29355	74.26904	53.42362	178.5643	138.1689	143.2658	108.9578	115.0983	112.2318	46.37782	40.08302	39.21997	116.0739	138.396	91.15139	64.30676	36.39758	44.81782	174.4047	170.7638	174.8796	92.43414	135.6548	91.90691
LOC107764766	0	0	0	0.089959	0.131798	0	0	0.042422	0	0.048414	0.069628	0.08389	0	0.054165	0.152875	0	0	0	0.05038	0.125999	0.498089	0.041463	0.175175	0.15568	0.376629	0.69083	0.234725	0	1.43417	0
Novel04019	0.958897	0.983156	2.396453	0.434356	0.424248	0.257733	0	0.409654	0.337596	0.233761	0.084047	0	0.266864	0.130764	0.123023	0	0	0	0	0	0	0	0	0	0	0	0	0	0	0
Novel03491	2.681558	2.715455	2.486423	2.645298	1.898255	3.459603	2.599004	2.647604	2.685402	2.614857	2.381726	2.164768	2.255445	1.690259	2.813439	3.108467	2.705749	2.532339	1.20936	1.613094	1.684764	2.189674	2.680686	3.176465	0.527381	0.497492	0.9156	0	0.286888	0
LOC107786581	4.7971	4.205414	3.753015	3.642303	3.072419	4.813638	2.701516	2.517817	3.538644	2.539365	3.379747	3.184283	4.068723	4.012273	3.798215	4.800452	4.81095	5.534969	2.433883	2.859935	2.333352	2.86156	3.344789	4.035018	1.039713	0.635697	1.268952	0	0.54988	7.062188
LOC107791303	3.414216	3.915476	3.734614	3.685952	3.247707	3.701289	2.744331	3.330469	4.220465	3.162827	4.149687	3.629567	3.198967	2.452122	2.482182	4.070844	2.968578	4.306041	3.551123	3.489898	2.750543	3.017627	3.312721	3.895653	0.863321	2.019019	2.421197	0	0.410931	0
LOC107829222	0.47618	0.325484	0.750253	6.686617	11.58726	8.223219	2.521164	5.492621	7.097053	9.838104	2.441625	10.23325	5.731596	5.681906	2.74915	1.020193	0.31627	0.710024	28.62926	25.60394	36.85984	8.251654	4.01646	10.76282	7.036294	14.61793	26.10013	8.513394	8.310307	12.88127
LOC107783930	0	0	0	0	0	0	0	0	0	0	0	0	0	0	0	0	0	0	0	0	0	0	0	0	0	0	0.030437	0	0	0
LOC107810135	1.810824	2.536638	1.953064	0.698738	0.860514	0.288423	0.45002	0.830913	0.440762	0.326997	0.705417	0.509945	0.522624	0.256086	0.516272	2.373994	2.170562	1.375047	0.510416	0.992854	0.397584	0.980173	0.798633	0.490693	0.127191	0.326619	0.237805	0	0.161444	4.146888
LOC107829248	0	0	0	0	0	0	0	0	0	0	0	0	0	0	0	0	0	0	0	0	0	0	0	0	0	0	0	0	0	0
LOC107761473	0.687914	0.268692	0.170854	0.178062	0.521753	0.105656	0.082426	0	0.092263	0.95829	0.206728	0.498145	0.656396	0.107212	0.504326	0.439401	0.474702	0.073267	0.099721	0.083133	0.358508	0	0.173367	0.205431	0	0	0	0	0	0
LOC107759892	30.09882	32.38326	28.20304	13.98813	16.63724	19.33189	14.32746	8.750544	17.46864	3.659239	3.892148	1.287928	17.40593	15.82114	8.425255	17.39028	21.03434	20.92452	5.394467	10.01928	4.099769	6.65949	7.413074	4.698463	0.098842	0.10878	0.600606	2.795443	0.752761	0
LOC107823147	0	0	0	0	0	0	0	0	0	0	0	0	0	0	0	0	0	0	0	0	0	0	0	0	0	0	0	0	0	0
LOC107776660	0.048748	0.022214	0.063563	0	0	0	0.040887	0	0	0.023768	0	0.020592	0	0.026591	0	0	0	0.054516	0	0	0	0	0.021499	0.050951	0	0	0	0	0	0
107768437	0	0	0	0	0	0	0	0	0	0	0	0	0	0	0	0	0	0	0	0	0	0	0	0	0	0	0	0	0	0
LOC107825282	0.232713	0.406504	0.286581	0.474358	0.514799	0.375292	0.309045	0.149127	0.182067	0.170193	0.203973	0.081918	0.172706	0.211565	0.059712	0.447996	0.730665	0.621698	0.669066	0.672601	0.194551	0.356298	0.325008	0.445924	0.269701	0.215869	0.481334	1.386854	0.373454	0
LOC107780877	2.92292	2.852475	2.923081	0.328073	0.251773	0.500575	0.065086	0.066303	0.048569	0.050446	0.054413	0.109264	0.316745	0.507944	0.159292	2.621506	2.348991	3.104822	0.577447	0.240695	0.259498	0.194416	0.433504	0.324429	0	0	0.152861	0	0	0
LOC107802200	0.027098	0.049392	0.023555	0.024549	0	0	0.090912	0.046306	0.127202	0.052847	0.076004	0	0.180993	0.295622	0.695307	0.080773	0.026179	0	0.08249	0.091691	0	0.02263	0	0	0	0.113113	0	0	0	0
LOC107792811	4.319531	4.990488	5.651278	6.205902	6.100084	6.590761	7.117868	6.449447	6.246901	5.573571	6.073012	5.326444	4.978561	6.425994	5.664961	6.519124	5.290053	5.855261	7.128171	7.252687	5.252646	6.158006	5.888354	6.110904	7.198854	6.131732	6.394564	3.120278	7.352036	4.046713
LOC107766834	17.70346	15.16133	18.41824	14.81646	12.73107	14.32036	14.99007	14.26196	18.15096	8.384939	7.8817	8.878864	9.57233	7.48019	6.402884	16.50127	19.65491	17.76584	9.80905	8.415035	12.35286	15.58252	17.94557	24.61279	3.126452	3.753611	5.579751	16.0768	3.788034	20.85013
LOC107805822	0.263546	0	0.057274	0	0	0.070836	0.110524	0	0.123714	0	0	0	0.073346	0.071879	0	0	0	0	0	0	0	0	0	0	0	0	0	0	0	0
LOC107824046	0	0	0	0	0	0	0	0	0	0	0	0	0	0	0	0	0	0	0	0	0	0	0	0	0	0	0	0	0	0
LOC107808264	0	0	0	0	0	0	0	0	0	0	0	0	0	0	0	0	0	0	0	0	0	0	0	0	0	0	0	0	0	0
LOC107793428	0.578782	1.002228	0.771455	1.555563	2.646079	1.763074	1.423985	1.582486	0.815079	1.975349	2.840896	1.923286	2.792002	1.431225	2.910825	1.107026	0.596429	0.877396	3.75877	3.737354	3.712607	1.852864	1.582624	1.129226	2.024526	0.966399	1.29974	0	2.414934	2.385797
LOC107831937	5.166953	7.880765	6.12761	3.538898	2.796655	4.257006	6.612313	5.856037	6.5513	2.787586	3.747575	2.955125	2.767249	3.254288	3.298566	6.999559	5.231862	5.745179	2.072002	2.733682	2.526207	8.289062	5.826203	5.511848	0.448976	1.334122	0.902397	0	0.683864	0
LOC107822068	0.152989	0.15686	0.116367	0.398477	0.62611	0.287844	0.384957	0.261437	0.574533	0.37296	0.214553	0.355437	0.191599	0.125178	0	0.042753	0.184751	0	0.310486	0.17795	0.191852	0.015971	0.354235	0.159905	0.048356	0.026609	0	0	0	0
LOC107774895	0	0	0	0	0	0	0	0	0	0	0	0	0	0	0	0	0	0	0	0	0	0	0	0	0	0	0	0	0	0
LOC107801457	0	0	0	0	0	0	0	0	0	0	0	0	0	0	0	0	0	0	0	0	0	0	0	0	0	0	0	0	0	0
Novel02923	11.12173	11.24779	9.605511	5.85042	6.412678	8.331482	6.620066	7.663474	9.363731	2.51886	3.169739	3.758379	4.952346	5.557816	3.755916	13.63498	13.72527	15.08558	4.659846	3.884684	7.721924	6.471672	9.05061	10.2745	1.542201	1.497588	2.713804	20.52544	1.727225	0
LOC107796257	0	0	0	0	0	0	0	0	0	0	0	0	0	0	0	0	0	0	0	0	0	0	0	0	0	0	0	0	0	0

LOC107781206	0.081146	0.073955	0	0.367571	0.21541	0.348968	0.136121	0.208	0.038092	0.51433	0.284498	0.685544	0.496831	0.398368	0.041643	0.030235	0	0.060498	1.646828	1.098303	1.81316	0.304952	0.107364	0.127221	1.025932	2.483993	1.342713	5.803088	0.390666	2.508691
LOC107824268	0.196916	0.269198	0.34235	0.356793	0.522734	0	0.247743	0.084125	0.369748	0.288028	0.276156	0	0.328815	0.107413	0	0.440227	0.285357	0.220214	0.499543	0.416444	0.359182	0.49335	0.434233	0.411634	0	0	0.232739	7.041153	0.474013	0
LOC107816829	0	0	0	0	0	0	0	0	0	0	0	0	0	0	0	0	0	0	0	0	0	0	0	0	0	0	0	0	0	0
LOC107773581	0	0	0	0	0	0	0	0	0	0	0	0	0	0	0	0	0	0	0	0	0	0	0	0	0	0	0	0	0	0
Novel04685	0	0	0.037944	0.197723	0.115873	0	0	0	0.081961	0	0.091822	0	0	0	0	0.097584	0	0.130171	0.620099	0.664645	0.756378	0.036453	0.154008	0.091246	0.16556	0.060736	0	0	0	0
LOC107832043	12.90893	12.20566	11.05977	13.00924	10.73139	14.47862	8.050253	9.790088	9.045948	13.96633	12.57333	15.27444	15.52998	12.29727	11.37843	9.425693	11.39292	11.87072	10.60762	9.975971	9.873172	6.58641	8.105111	7.659958	7.807652	6.780982	8.837814	15.96261	7.52226	6.900681
LOC107759578	0.851703	0	0	0	0	0	0.71436	0	0.199904	0.20763	0.149304	0	0.711096	0.696875	0.218541	0	0.205704	0	0	0	0	0	0	0	0	0	0.251661	0	0	0
LOC107828008	0.376833	0.171718	0.382168	0.284493	0.222298	0.135047	0.105355	0.214651	0.412752	0.244973	0.044039	0.318359	0.209748	0	0.193385	0.093605	0.424728	0.374593	0.446115	0.637549	0.343678	0.05245	0.332391	0.45951	0.714647	0.524335	0.296924	0	0	0
LOC107822416	0	0	0	0	0	0	0	0	0	0	0	0	0	0	0	0	0	0	0	0	0	0	0	0	0	0	0	0	0	0
LOC107776815	0.058071	0.052924	0	0	0	0	0	0	0	0	0	0	0	0	0	0	0	0	0	0.049124	0	0	0	0	0	0	0	0	0	0
LOC107758934	7.644079	4.115007	4.854517	2.045251	1.103966	1.894897	1.760648	1.945856	4.480726	0.502079	0.583216	0.568833	1.058176	0.734551	1.219535	5.888223	3.577621	3.617231	0.843991	0.586328	1.228145	1.786132	3.074338	2.421715	0.150224	0.247994	0.11703	1.416217	1.144082	2.448937
LOC107760163	46.01222	46.51479	48.37443	37.77265	33.31001	37.82288	29.30242	28.41204	34.24255	19.65044	21.87985	18.38504	15.72493	17.40662	15.67512	37.21541	36.43811	42.68909	26.76135	26.18956	25.6545	34.14739	39.94023	51.02439	13.47921	9.504952	16.5801	3.489421	15.62145	13.57637
LOC107825894	78.87621	87.93156	85.27848	96.49389	102.8515	95.30171	79.85844	85.1312	93.08983	81.0995	64.58236	82.58377	74.15496	72.7675	68.90957	76.56563	78.27426	85.22072	90.52619	79.2832	91.2686	70.58976	87.65324	108.2587	92.13766	53.19943	103.578	97.09256	88.13468	56.86715
LOC107805918	0	0	0.055868	0	0	0	0	0	0	0	0	0	0	0.14023	0.065965	0	0	0	0	0.054368	0	0.053673	0.11338	0	0	0	0	0	0	0
LOC107814436	0	0	0	0	0	0	0	0	0	0	0	0	0	0	0	0	0	0	0	0	0	0	0	0	0	0	0	0	0	0
LOC107783079	4.096741	5.188353	3.884957	14.86986	27.58817	11.61186	26.64103	29.32125	10.78934	37.09494	17.90735	26.49719	39.53463	29.74725	38.33451	4.519872	3.263897	3.292291	18.30199	20.72586	18.97262	21.34977	10.72347	9.954057	88.62371	47.15716	50.52678	68.48764	84.65594	72.37362
LOC107811524	0.14172	0.206657	0.098555	0.15407	0.125403	0.152366	0.28528	0.193743	0.239496	0.082917	0.139124	0.119729	0.283977	0.494751	0.494556	0.253464	0.246444	0.295843	0.115046	0.119885	0.02585	0.094683	0.125007	0.029625	0.071671	0.157755	0.067001	0	0	0
LOC107814633	0	0	0	0	0	0	0	0	0	0	0	0	0	0	0	0	0	0	0	0	0	0	0	0	0	0	0	0	0	0
LOC107806383	0	0	0	0	0	0	0	0	0	0	0	0	0	0	0	0	0	0	0	0	0	0	0	0	0	0	0	0	0	0
LOC107797017	21.29792	18.81235	22.66259	22.48362	20.8518	24.72585	18.26379	15.62336	19.32331	17.59767	15.97992	18.69869	18.62861	18.19098	13.25659	16.42695	18.616	19.19209	14.58938	14.35775	13.52067	16.59072	20.28591	22.07352	6.298063	3.942966	10.99972	14.93238	5.313479	5.53312
LOC107830891	8.886078	9.482935	7.218317	4.816441	3.875489	2.776266	4.92629	4.84536	3.232477	3.382085	6.177668	4.470103	3.776474	3.562853	4.781079	12.4326	8.315663	9.305127	2.825834	3.169566	4.248383	5.095328	5.783676	4.366025	1.088262	2.113561	1.765399	0	3.290827	3.13071
LOC107823616	0	0	0	0	0	0	0	0	0	0	0	0	0	0	0	0	0	0	0	0	0	0	0	0	0	0	0	0	0	0
LOC107821414	0	0	0	0	0	0	0	0	0	0	0	0	0	0	0	0	0	0	0	0	0	0	0	0	0	0.031226	0	0	0	0
LOC107768999	0.084688	0.385915	0.073618	0.191808	0.224814	0.09105	0.213095	0.68742	0.51681	0.371618	0.178151	0.465056	0.329967	0.415759	0.304226	0.725764	0.286356	0.820804	0.773423	0.250742	0.386186	0.530441	0.261453	0.265549	1.552542	1.237296	1.801707	3.028208	1.83474	5.23641
LOC107810681	0.078296	0	0.03403	0	0.034641	0.042089	0	0	0.036754	0	0	0	0	0.298958	0.04018	0.087519	0.03782	0.029186	0.238347	0.033116	0.214221	0.032693	0.034531	0.081835	0.148484	0.108943	0.04627	0	0	0
LOC107781606	0.075043	0	0	0.13597	0.066403	0	0.062941	0.064118	0	0	0.10524	0	0	0	0	0	0.144995	0	0.076148	0	0.06844	0.06267	0	0	0	0.104416	0	0	0.361282	0
LOC107823929	0	0	0	0	0	0	0	0	0	0	0	0	0	0	0	0	0	0	0	0	0	0	0	0	0	0	0	0	0	0
Novel04575	4.558573	4.034734	2.286156	0.714779	0.543002	1.837884	0.698519	1.198452	0.946492	0.256453	0.522501	0.148123	0.09759	0.382552	0.044988	1.339223	3.472357	3.169857	0.355825	0.593268	0.119928	0.439267	1.237219	0.50395	0	0	0	0	0	0
LOC107832061	0	0	0.033878	0	0.034486	0	0.261507	0.199798	0.475665	0.038004	0.054656	0.065851	0.043385	0	0.120003	0.029043	0.075302	0	0.83049	0.791244	0.355441	0.390569	0.240638	0.570284	0.147821	0.054228	0.552756	2.787123	1.313411	2.409762
LOC107792696	17.79513	16.69624	15.849	14.45784	14.27613	17.95676	12.57853	12.66431	14.28497	17.39513	14.74667	18.94926	18.83633	21.70318	11.62282	14.271	16.26231	16.03778	13.75369	15.01647	11.84312	10.7351	12.53404	16.8629	11.4427	6.327038	13.17755	0	16.20823	2.703439
LOC107784010	13.91374	13.12522	15.3458	14.45698	14.12053	16.78202	12.839	12.48368	13.43356	14.24717	14.20286	12.83403	14.2995	15.10318	13.35088	13.84779	13.73357	15.2915	12.89304	14.87469	9.914383	12.06589	14.19879	15.0282	12.4225	9.825417	12.57391	3.3223	10.06463	17.23487
LOC107759194	0.484998	2.386885	0.758877	0.087877	0	0.104286	0	0	0	0	0	0	0	0	0	1.879389	1.030807	0.79549	0	0	0	0	0.34224	0	0	0	0	0	0	0
LOC107771870	0	0	0	0	0	0	0	0	0	0	0	0	0	0	0	0	0	0	0	0	0	0	0	0	0	0	0	0	0	0
LOC107832119	0	0	0	0	0	0	0	0	0	0	0	0	0	0	0	0	0	0	0	0	0	0	0	0	0	0	0	0	0	0
Novel05224	1.172167	2.513613	2.936944	0.478907	1.098223	1.210802	1.754242	2.219079	4.445083	1.501602	2.64305	3.009671	3.147045	2.281738	4.930268	5.960353	1.509882	4.386635	1.119467	2.819176	1.614034	3.781268	1.439391	5.140818	1.365742	2.174646	1.059429	6.574627	6.52844	2.842229
LOC107828728	0	0	0	0	0	0	0	0	0	0	0	0	0	0	0	0	0	0	0	0	0	0	0	0	0	0	0	0	0	0
LOC107799578	1.06821	1.144338	1.498748	1.17148	0.978388	1.22905	0.361525	0.480372	0.404672	0.420311	0.30224	0.807458	0.292071	0.470235	0.192348	1.326719	1.194925	1.690594	0.874763	1.617026	1.145142	0.500822	0.41326	0.332989	0.118469	0.495445	0.797392	0	0.090224	0
Novel04063	0.569345	1.686386	1.361029	0.644747	1.259485	1.071203	0.716302	0.121616	1.069053	0.971571	1.197676	1.202497	0	0.77641	0.876541	1.591038	0.687544	1.061178	1.155464	0.240813	0.649066	0.118869	0.7533	0.14877	0	0.396101	0.33646	0	1.370515	0
LOC107767860	18.5304	12.51474	14.34326	25.31517	22.7989	30.12179	18.60735	17.81987	21.38256	19.94122	13.3559	19.42841	18.12168	21.82658	16.67012	9.957796	14.44279	11.69614	20.41402	19.29766	18.38132	18.80455	18.94983	18.82783	13.67415	27.9409	19.02197	15.83887	19.54844	9.129583
LOC107808846	0.660423	1.128551	0.717614	0.448732	0.328717	0.532526	0.588545	0.423212	0.775042	0.603746	0.607804	0.523072	0.505444	0.315214	0.33892	0.522907	0.478518	1.015519	0.502613	0.523755	0.112934	0.620478	0.436902	0.8197	0.156558	0.057433	0.390283	0	0	0
LOC107800480	53.03675	46.37687	49.115	42.52951	38.31593	52.26924	36.16846	37.35511	46.82134	31.79929	32.70825	39.35743	44.21445	37.59628	31.38621	40.14616	54.29608	49.24832	33.9999	30.56711	39.21925	27.23382	38.30006	46.07014	15.93301	14.46018	28.7307	72.61139	27.029	18.46476
Novel00930	12.14369	15.12554	14.98987	10.63484	10.53062	6.005735	10.11753	11.34431	8.892801	4.973483	8.231321	4.035334	4.776544	3.532844	11.13446	17.31469	13.76534	14.96867	7.064942	9.450904	4.651603	21.16195	15.28179	9.561741	2.047108	3.041469	2.583515	0	3.507841	0
LOC107811525	0.069377	0.252915	0.180924	0.125704	0.122779	0.074589	0.174569	0.059278	0.065134	0.202954	0.291884	0.293059	0.154463	0.832558	0.284827	0.0517	0	0	0	0.126546	0	0	0	0	0	0	0	0	0.334006	0
LOC107797236	0	0	0	0	0	0	0	0	0	0	0	0	0	0	0	0	0	0	0	0	0	0	0	0	0	0	0	0	0	0
Novel03297	0.88575	0.807252	0.733299	0.655057	0.709125	0.524731	0.985502	0.906111	1.397283	0.593439	0.895719	0.442875	0.731137	0.670501	0.346327	0.687003	0.401659	0.575011	0.568627	0.626952	0.401162	0.885643	1.06298	0.919488	0.220924	0.301826	0.235015	0	0.116036	0
LOC107763369	9.694969	8.528971	3.921189	2.043302	1.727664	1.411485	3.246988	3.480281	4.961889	2.691712	4.532079	3.242104	6.033349	5.325092	2.729519	2.082119	7.837651	2.308948	1.844585	1.452309	2.026286	3.317325	4.127336	3.553649	1.574734	1.498871	2.625946	0	1.296529	0
LOC107769952	3.593124	2.765293	2.429332	2.02545	1.059811	1.888601	1.808225	1.637367	1.199425	1.012194	0.951811	1.753885	1.599966	1.480859	1.065389	2.082578	1.928478	2.559767	1.296375	1.823721	1.529262	1.200285	2.042485	2.837513	0.302853	0.333305	1.509967	0	1.153239	0
LOC107822327	0	0	0	0	0	0	0	0	0	0	0	0	0	0	0	0	0	0	0	0	0	0	0	0	0	0	0	0	0	0
LOC107786338	1.273827	1.749144	2.111272	1.815662	1.87861	1.661684	2.293528	2.626653	2.248339	1.540256	2.167519	1.076164	1.247877	1.130275	2.649707	1.771947	0.607108	1.101643	1.240888	1.580436	0.681561	1.914851	1.528095	1.508924	0.536835	0.85077	0.521928	0	1.717144	1.050168
LOC107827462	7.084523	7.539723	6.873597	6.169772	5.318413	7.223607	6.804656	6.463265	7.552401	5.504359	4.951647	4.788161	5.876781	5.210752	4.433184	6.607758	7.837764	7.479728	5.101789	4.523763	4.293574	5.821043	6.773129	8.575168	2.715991	3.815844	2.863141	6.537336	2.200478	5.652216
LOC107830502	0.61805	2.112287	0.268629	0.279961	0	0.49836	0	0.264039	0.290126	0	0.216689	0.130537	0.172006	0	0.63435	3.799717	0.597089	2.534305	0.156789	0	0	0.129037	0.13629	0.161496	0	0.429986	0	0	0	0
LOC107794309	37.27365	37.22888	41.72528	31.13624	28.51337	32.48183	31.3005	31.27482	38.05718	27.06377	23.81726	26.4303	28.3589	28.23057	21.00905	30.30755	33.59158	32.0874	18.86601	19.69739	23.02964	28.03031	31.14327	40.92035	13.84374	12.87261	21.44618	12.78462	11.40381	30.39754
LOC107805229	0	0	0	0	0	0	0	0	0	0	0	0	0	0	0	0	0	0	0	0	0	0	0	0	0	0	0	0	0	0
LOC107797545	0	0	0	0	0	0	0	0	0	0	0	0	0	0	0	0	0	0	0	0	0	0	0	0	0	0	0	0	0	0
Novel00502	0.946622	0.345092	1.15203	0.285864	0.335053	10.1095	0.476384	15.09797	21.68503	0.184615	1.50455	0.426524	0.140506	0.826175	0.323862	0.094057	1.707098	0.329349	0.192113	0.320311	0.460446	3.847338	8.349834	0.527685	1.595758	0.439052	0.895065	9.02625	1.2153	3.902072
LOC107812339	0	0.090756	0.086564	0	0	0.107062	0.083523	0	0	0	0	0	0	0	0	0.074208	0	0	0	0	0	0	0.087837	0	0	0	0	0	0	0
LOC107791371	2.635121	2.646648	2.571144	2.208646	2.300005	3.314894	2.059837	1.746076	2.305671	2.132573	2.22484	1.620449	3.412385	2.777006	2.097458	2.123997	2.112798	2.76647	1.837196	1.941012	1.356951	1.736574	2.134608	1.236592	1.337171	1.795876	0.932232	5.127849	5.350732	57.63635
LOC107780862	1.294326	3.686308	4.606002	1.02602	1.145305	1.087155	0.71243	1.347825	1.367067	1.617106	2.524213	1.879423	1.395835	2.294575	4.483556	9.0425	1.523959	5.156586	1.272349	1.402855	0.922224	0.439126	0.749228	0.845518	0	0.22512	0.478059	5.785164	0.19473	0
LOC107831260	0	0	0	0	0	0	0	0	0	0	0	0	0	0	0	0	0	0	0	0	0	0	0	0	0	0	0	0	0	0
107795916	0	0	0	0	0	0	0	0	0	0	0	0	0	0	0	0	0	0	0	0	0	0	0	0	0	0	0	0	0	0
LOC107816126	5.44055	5.327632	7.848716	8.389553	6.965162	9.147241	5.194308	5.884871	7.607403	8.691536	9.212618	8.459199	8.891451	7.513929	4.633558	8.065477	6.094757	7.896568	6.519117	7.637911	6.756593	3.915183	6.228392	9.981554	7.244387	1.691199	11.49244	0	9.195325	10.73609
LOC107824812	0	0	0	0	0	0	0	0	0	0	0	0	0	0	0	0	0	0	0	0	0	0	0	0	0	0	0	0	0	0
LOC107794314	21.3996	21.13515	21.44314	15.65557	14.57817	15.54669	15.77088	13.69417	18.03136	13.18395	10.79887	15.31796	13.75515	13.13818	10.61438	18.3157	17.94902	19.85944	13.35598	14.27756	15.02548	12.82401	16.38804	15.53513	6.962004	9.717682	11.42929	32.01618	12.28544	22.14509
LOC107770588	0	0	0	0	0	0	0	0	0	0	0	0	0	0	0	0	0	0	0	0	0	0	0	0	0	0	0	0	0	0
LOC107781924	16.86956	13.63934	14.20248	9.386389	6.347039	9.330238	8.095883	7.036663	13.63472	5.181087	7.482351	7.294266	6.16123	6.617661	3.453802	10.82246	10.73662	11.65268	3.729132	4.981561	5.451492	8.652601	12.22431	12.58772	0.951654	1.108949	2.98292	3.166436	0.852662	0
LOC107778532	0	0	0	0	0	0	0	0	0	0	0	0	0	0	0	0	0	0	0	0	0	0	0	0	0	0	0	0	0	0
LOC107762013	2.066934	2.980625	3.024894	9.528607	9.654125	8.269983	5.420325	5.186358	6.705876	9.23568	5.595534	9.10678	5.417402	8.562999	3.67896	2.222685	2.173766	2.360246	7.062069	7.702181	7.540313	4.785151	5.584907	9.762633	6.318068	4.550613	11.56535	3.742151	3.652882	0
LOC107776889	0	0	0	0	0	0	0	0	0	0	0	0	0	0	0	0	0	0	0	0	0	0	0	0	0	0	0	0	0	0
LOC107801044	0	0	0	0	0	0	0	0	0	0	0	0	0	0	0	0	0	0	0	0	0	0	0	0	0	0	0	0	0	0
LOC107816348	0	0	0	0.11453	0	0	0.035344	0	0	0	0.059097	0.071202	0.140732	0.137917	0	0.031403	0.081421	0.219919	0.085521	0	0.076864	0.035192	0	0.132133	0.106555	0	0	0	0	0
LOC107767939	1.065629	0.373534	0.783817	0.519834	0.870405	1.762587	0.550024	0.49027	0.923501	0.31973	0.229914	0.484762	0.091252	0.894268	0.084133	0.244341	0.791913	0.977811	0.332716	0.346711	1.12139	0.136913	0.867651	0.514059	0.103637	0	0.096884	0	0.78928	0
LOC107770680	0.247463	0.375887	0.28682	0.896757	1.16785	1.330267	1.037791	1.057197	0.464659	0.241308	0.462724	0	0.27548	0.719921	1.100622	0.06147	0.15938	0.122996	0.502218	0.209337	1.504605	1.239979	1.673473	0.862162	0.521448	0.80343	0.194988	0	1.588503	0
LOC107784856	7.026186	7.365401	6.212568	11.06427	12.59456	12.87099	8.72593	9.945487	7.842182	14.0851	13.30015	15.56586	12.45421	9.112733	12.28738	6.74153	6.875284	8.5207	15.20796	15.43313	12.98101	9.368255	9.735224	7.974134	10.027	12.62963	9.694391	2.156531	7.549275	9.322739
LOC107786867	0	0	0	0	0	0	0	0	0	0	0	0	0	0	0.030739	0	12.82918	14.3453	15.41887	11.69387	9.986957	9.636476	8.706386	9.344867	12.92849	5.877865	5.959203	8.558648	6.045014	18.17721	8.710919
LOC107815037	1.310206	3.8159	1.287491	6.58	4.032545	2.235437	18.65786	15.08858	3.235643	7.249084	20.03203	4.668224	5.263426	4.319185	26.57362	7.598683	1.953687	2.357095	7.023263	13.8543	6.701999	14.93804	11.88349	5.954031	17.78936	16.09045	4.443689	65.1814	5.622168	68.6841
LOC107760461	1.351387	2.438613	0.939786	0.440744	0.358739	0.261523	0.566733	0.900636	0.73587	0.184488	0.663315	0.342507	1.353945	1.444811	0.776735	3.021166	1.22722	1.81353	0.219407	0.342954	0.271147	1.173722	0.810569	0.310743	0.136685	0.300857	0.031945	0	0	0
LOC107762476	10.86775	11.94505	12.08256	10.01466	10.15304	10.3302	11.34516	12.27466	12.39263	5.912729	8.20918	8.038631	10.22889	10.83848	7.468156	10.60125	12.93244	10.25837	7.336119	5.405544	9.486183	8.803275	12.34259	11.89523	1.533235	2.271497	3.693571	6.671226	3.592874	5.767978
LOC107818296	3.440773	4.521444	4.368237	3.117166	2.407376	4.181014	3.704721	2.256194	3.470747	1.966296	2.928871	1.798197	2.226928	2.758543	2.217463	3.911694	3.880665	4.462328	2.370944	2.76174	1.678488	3.19424	3.063221	2.977397	0.384435	0.801642	1.059238	0	0.308188	0.989526
LOC107812121	26.68703	32.9488	37.92656	2.342044	1.68926	3.848381	1.367699	1.903009	1.754967	1.512527	1.36652	1.680026	3.409158	5.640613	2.000225	28.77866	34.84977	30.30407	1.856464	2.018664	2.10382	2.258597	6.314681	3.200868	0.351988	1.051458	3.901609	0	0	2.459164
LOC107824349	22.02413	20.66653	22.60886	17.06485	14.80862	20.95248	12.39608	13.06119	19.793	13.77592	14.27492	21.29966	21.04963	19.4431	10.93069	15.17068	25.05666	18.52643	8.601258	8.028447	10.83607	9.256967	16.35936	16.12888	4.213418	2.217728	11.2172	15.54313	6.97579	0
LOC107807634	5.227556	4.890898	4.453621	20.46979	24.51152	15.49773	20.24771	22.74832	16.81064	24.3191	18.65663	19.49958	20.86086	23.09623	21.2656	5.746305	5.402602	5.956106	39.63442	35.2303	34.60879	18.40543	15.28846	17.13576	32.62891	36.27215	31.42645	11.17806	27.34124	11.81231
LOC107823201	0	0	0	0	0	0	0	0	0	0	0	0	0	0	0	0	0	0	0	0	0	0	0	0	0	0	0	0	0	0
LOC107761626	29.62448	33.75659	33.5753	25.43305	22.85025	28.16649	29.77962	31.18119	25.20432	23.1533	31.32638	20.82272	23.69966	29.47375	43.0894	51.97687	27.58231	39.153	30.73543	35.24011	19.77384	27.75797	25.04823	22.09133	8.38305	15.36867	8.541905	25.59836	18.71001	13.70106
LOC107811270	2.788563	2.232338	2.424039	2.731132	2.967674	2.471362	2.275675	2.83339	3.290221	2.168012	2.08746	2.324019	2.475024	1.932199	1.8565	2.358857	3.058038	1.798047	2.561972	3.060236	2.130803	2.486153	2.160545	2.560126	1.810439	1.310838	2.048776	10.77955	2.177049	0
LOC107828430	10.35758	10.75821	12.49074	8.042951	6.691955	7.671236	6.894721	7.782203	8.705432	5.001879	5.994644	5.194665	5.197742	5.416639	4.55603	9.850252	10.19689	10.71276	3.970504	4.450438	3.86848	7.606407	9.426104	10.03529	2.619026	2.013079	3.147898	2.351471	1.899622	0
LOC107802121	0.835403	0.304547	0.217859	0.605466	1.256673	0.359264	1.051033	1.070686	0.62745	0.570236	0.468628	0.917503	1.766966	1.367075	1.028922	0	0	0	0	0	0	0	0	0	0	0	0.197476	0	0	0
LOC107817977	6.442431	6.739439	7.256	2.943634	3.569125	5.962728	4.134886	4.068608	5.101726	3.277661	3.692513	3.644272	3.305266	3.483629	2.127447	3.965966	4.437932	3.967783	3.069704	2.985572	2.963349	4.538117	6.127379	4.215836	1.55821	0.779493	2.979561	0	0	0
LOC107800772	15.45758	19.07416	16.66314	13.62249	9.547432	18.51102	15.59643	13.04392	14.18896	9.961671	12.87786	10.34376	12.73532	10.51879	11.23133	15.73884	16.19008	14.71913	7.493216	9.968868	6.525361	14.98597	15.28831	15.03652	2.273576	2.129514	5.155287	0	2.394648	0
LOC107783679	96.06671	94.00263	98.57327	77.59137	57.93632	107.8965	49.07428	52.80101	74.02622	49.77279	66.77594	69.67317	33.93139	43.04018	27.05215	38.70126	38.81377	40.3392	20.69511	27.5757	28.96766	28.2938	41.43472	50.67038	13.87885	6.125241	28.1882	3.984993	4.292337	3.445446
LOC107764325	0.538148	0.784729	0.654921	0.942568	1.238091	1.080007	2.136476	1.685964	1.212569	1.224446	1.43393	0.848666	0.798765	0.978489	2.025249	0.614911	0.519896	0.802425	1.310582	2.094087	0.88344	2.516761	1.677211	0.974952	1.406125	2.396141	0.508838	0	1.036335	4.436602
LOC107786101	8.105039	8.077094	13.13631	9.435783	11.52862	10.30196	7.909875	10.54955	10.31176	10.85799	10.88849	10.52702	11.1729	11.03209	10.37905	11.43062	8.342402	11.29467	11.18369	11.05339	12.15869	7.907395	8.452068	10.33192	19.91986	23.66182	21.35245	13.54264	20.96898	49.17795
LOC107827545	4.65226	3.934928	4.422337	11.81029	11.89083	9.139886	9.727055	8.936639	10.49516	10.50911	12.99	8.46865	17.94007	16.28505	7.832296	5.636781	4.34897	5.165256	14.65483	18.79976	4.380301	7.714545	11.0561	7.048935	6.220396	4.983029	5.320585	1.196771	5.559125	1.034735
LOC107781249	0	0	0	0	0	0	0	0	0	0	0	0	0	0	0	0	0	0	0	0	0.052669	0	0	0	0	0	0	0	0	0
LOC107798826	54.89929	57.67801	60.80621	37.45215	31.35895	39.2064	36.4534	35.37887	48.70956	22.24107	25.47764	20.61353	21.88466	23.97867	19.42887	52.44812	52.90923	55.73466	21.93335	22.43528	21.28541	41.41809	51.67067	57.83118	6.957705	6.181184	13.98822	7.112456	7.341799	10.24911
LOC107832393	1.229572	0.859129	0.676932	0.519834	0.834138	0.837229	0.481271	0.315174	1.000459	0.719393	1.005873	0.242381	0.593137	0.312994	0.799266	1.374418	0.791913	0.886141	0.623842	0.762764	0.224278	0.718793	0.469978	0.385544	0.414547	0.684344	0.048442	0	0.19732	0
LOC107788672	0	0	0	0	0	0	0	0	0	0	0	0	0	0	0	0	0	0	0.090857	0.075743	0	0	0	0	0	0	0	0	0	0
LOC107766068	0	0	0	0	0	0	0	0.031651	0.034778	0	0	0.062591	0.041238	0.080826	0	0	0	0	0	0	0	0	0	0	0.051544	0.043783	0	0	0	0
LOC107825263	8.293462	6.620764	3.794385	27.05974	28.44398	16.86087	25.73228	22.16423	22.89046	16.59999	16.6591	14.75063	17.14588	24.04799	26.24056	6.528811	7.409762	6.229618	18.91935	18.56786	22.71977	29.08436	23.23871	26.68931	1.773856	6.290464	3.463937	0	3.002081	1.927809
LOC107803357	15.14766	14.36432	15.87488	12.18398	8.935765	10.60339	12.5071	12.33778	13.95547	6.62619	9.397258	9.926773	7.039211	10.03877	8.427424	14.06611	14.54274	13.43929	6.17705	7.863969	8.392242	10.44332	11.61304	11.19607	1.610853	2.23244	3.513735	16.87339	2.044661	2.917765
LOC107815364	0	0	0	0	0	0	0	0.031503	0	0	0	0	0	0	0.025005	0	0	0	0	0	0	0	0	0	0	0	0	0	0	0
Novel02435	2.336525	2.654525	1.683302	2.595222	2.917173	2.271168	2.872501	2.297216	1.727861	4.01062	4.971226	0.94642	5.611858	3.491827	4.829141	0.059629	0.123687	0.059657	0.259829	0.081228	0.043787	0.173745	0.183511	0.050181	0.0607	0.044536	0.056745	0	0.077047	0
LOC107774850	37.61246	34.91178	43.06398	21.61131	17.03188	30.32849	16.56757	17.79403	24.29197	10.46153	9.115803	9.543477	6.533517	10.53373	8.226104	25.11309	33.28841	29.12383	14.02428	10.73042	17.20915	17.28667	23.4352	33.50799	3.670243	3.248984	5.668745	13.53937	5.165026	0
LOC107796190	1.342231	1.730491	1.422894	1.156677	1.332535	1.231883	1.40037	1.258726	1.69044	1.14923	1.147773	0.85738	1.494191	0.964301	1.37763	1.780902	1.075319	1.293575	1.461662	0.886193	0.746425	1.941127	2.252368	1.676636	2.648958	2.004272	1.470331	2.341183	4.25545	6.072599
LOC107826020	0.513213	0.46773	0.418242	0.087177	0.31221	0.206912	0.215227	0.164439	0.060228	0.093834	0.067475	0.081296	0.071414	0.034993	0.19753	0.501961	0.526794	0.74133	0.19529	0.162803	0.058507	0.214299	0.226344	0.368782	0.040554	0	0.227466	0	0	0
LOC107807373	1.515618	1.270795	1.159395	2.032148	1.609342	0.847327	2.033943	1.76118	1.422929	2.423787	4.208499	2.765742	4.116786	3.571484	3.795637	2.529946	2.284175	2.305114	2.7683	2.564217	2.598666	2.075801	1.818151	1.837583	2.759331	3.121131	1.576378	0	10.50728	11.24555
LOC107762065	4.062388	4.678674	4.764901	3.213976	2.388238	4.382521	1.715319	1.521541	1.893913	1.275226	2.204699	0.74047	1.115089	1.487407	1.399361	5.266666	4.744475	4.906051	2.258756	3.154049	0.685165	2.718732	2.981988	2.762813	0.228662	0.135506	0.82216	0	0.602808	0
LOC107761529	25.70187	21.9086	25.89294	15.98155	14.90591	15.74012	20.2232	18.34664	21.10955	8.671438	10.18975	8.887023	8.329983	8.676069	8.125391	21.49669	20.63942	22.36895	9.757255	10.06065	10.7477	18.92844	24.16938	27.84594	3.382037	4.627468	6.323314	0	3.828741	6.705423
LOC107774365	0	0	0	0	0	0	0	0	0	0	0	0	0	0	0	0	0	0	0	0	0	0	0	0	0	0	0	0	0	0
LOC107778366	0	0	0	0	0	0	0	0	0	0	0	0	0	0	0	0	0	0	0	0	0	0	0	0	0	0	0	0	0	0
LOC107772865	0.990166	1.534104	1.549314	0.986744	0.700931	1.383914	0.664396	0.846024	0.185922	0.482768	0.208291	0.167304	0.220453	0.108022	0.203256	1.844681	1.434875	1.771705	1.306177	0.837612	0.722438	0.496148	0.960731	1.241905	0.125187	0.137774	0	0	0	0
LOC107787533	9.148101	9.898879	9.654404	4.157717	3.113401	5.230157	2.30957	2.561892	2.901179	0.895039	2.059554	1.163163	0.987726	1.368513	1.381712	9.461966	8.424023	11.13146	2.669987	3.209346	2.45553	3.423865	3.2115	5.116571	0.580233	0.723718	0.867878	0	0.589194	1.891775
LOC107794638	0	0	0	0	0	0	0	0	0	0	0	0	0	0	0	0	0	0	0	0	0	0	0	0	0	0	0	0	0	0
LOC107807082	116.368	125.9243	125.9531	129.8236	96.89459	125.3001	112.5639	115.1828	131.846	90.765	83.35931	96.06844	69.93246	81.23678	78.53487	94.70889	109.2073	103.3805	87.62792	91.8827	94.76778	96.14749	98.36744	120.888	27.74246	52.10122	53.12433	53.15119	42.93787	70.02637
LOC107826631	7.924118	11.95482	6.927184	12.6948	11.9636	3.032327	45.05965	38.25187	13.49199	14.05706	23.88936	5.143808	10.86458	7.619166	20.99901	9.408065	5.838837	6.308293	5.81485	9.922337	12.90231	27.5548	27.60298	11.04305	15.39565	14.63882	5.873386	12.80647	10.99224	22.14509
LOC107769490	3.545039	5.736436	3.58475	4.784667	5.505551	4.66696	6.978334	7.974247	4.550857	5.855499	6.442745	4.828589	3.865848	5.209236	5.866203	4.528744	4.263534	4.692633	8.00207	7.58895	5.212606	7.492016	6.764442	3.327185	7.134624	6.945995	3.163841	0	6.617847	31.31359
LOC107783971	1.019783	0.675932	0.685004	0.419942	0.246101	0.448524	0.544305	0.396059	0.261114	0.316405	0.422543	0.704897	0.258008	0.303418	0.237881	0.345429	0.895633	0.863968	0.235183	0.392121	0.422754	0.387112	0.817742	0.436041	0.234421	0.193494	0.602651	0	0.223164	0
LOC107769548	0	0	0	0	0	0	0	0.041671	0	0	0	0	0.054292	0	0	0	0	0	0.036361	0	0	0	0.04073	0	0.050975	0	0	0	0	0
LOC107807457	7.039299	6.289385	7.201314	15.42793	15.64009	15.33451	22.79162	21.02472	14.84816	11.18056	14.06426	9.920315	15.26186	12.97807	16.15359	5.715287	4.840569	6.19917	17.0153	16.68114	12.88197	23.45063	14.95342	17.1246	11.58142	12.12572	6.212665	15.38806	19.53467	34.21183
LOC107791835	0	0	0	0	0	0	0	0	0	0	0	0	0	0	0	0	0	0	0	0	0	0	0	0	0	0	0	0	0	0
LOC107813303	12.1961	6.874742	9.988986	15.51976	19.21334	15.76507	14.36845	15.23948	12.24447	12.99264	9.392271	15.72341	17.81473	23.68812	16.71444	6.777007	8.513333	8.93503	15.2372	13.3585	19.22422	14.83636	10.94431	9.79999	15.06305	25.99447	14.4148	35.29106	16.63064	13.07693
LOC107790953	0	0	0	0	0	0	0	0	0	0	0	0	0	0	0	0	0	0	0	0	0	0	0	0	0	0	0	0	0	0
LOC107814208	2.702131	2.225865	3.116816	2.965838	2.437004	2.625782	3.138083	2.486372	2.536879	1.469474	2.295545	1.93163	2.429578	2.154228	1.386696	2.284697	1.807259	2.983088	1.581886	2.593523	1.421763	2.690585	2.612636	4.290712	0.459888	0.216913	0.675591	0	1.250867	3.213015
LOC107784826	2.348967	2.600964	2.289989	3.858324	3.593703	3.139081	4.419094	4.389186	3.421327	4.409823	5.818716	3.486736	5.425312	4.430676	5.948426	3.288234	2.481383	3.044237	2.116253	2.581326	1.942082	4.345037	3.582333	3.304113	3.164094	1.832756	1.997891	1.569947	3.487749	1.357385
LOC107811649	0.941961	0.551881	0.906558	0.24382	0.178609	0.397856	0.028217	0.086233	0.15792	0.065609	0.047179	0.028421	0.224701	0.146805	0	0.426184	0.357505	0.627029	0.170685	0.227667	0.092045	0	0.059348	0.17581	0	0	0.079523	0	0.161962	0
LOC107803741	5.520746	6.626826	5.52478	6.465375	7.268104	8.454436	5.150006	4.901156	4.652166	5.646031	4.475428	4.709583	3.657484	4.8705	3.068117	5.398458	5.931898	5.621786	6.941103	7.859557	4.691159	5.33021	4.608364	5.291771	2.791961	3.110158	3.692239	3.851819	4.148892	1.665151
LOC107800200	0.984813	0.56096	0.856077	0.223048	0	0	0.330873	0	0.12004	0.086319	0	0.052	0.205558	0.067149	0	0.688016	0.594633	0.36711	0.24983	0.104136	0.112271	0.051403	0.054292	0	0.077819	0	0.145496	0	1.777966	0
LOC107809024	2.775655	3.194683	3.096865	3.888561	4.139891	4.630076	5.172137	4.131954	4.231241	3.752986	5.297122	2.731747	5.112666	4.073892	5.389332	3.454477	2.875028	3.456059	3.164981	3.945631	2.166081	5.221524	3.521025	3.648824	2.460108	1.851429	2.063058	4.092755	3.650714	3.538617
LOC107819234	4.37179	4.542901	4.830293	0.888363	0.686921	2.723485	0.137077	0	0.20946	0.268514	0.239049	0.085949	0.241625	0	0.356591	0	4.019046	3.631435	5.513488	0.870654	1.555332	0.558946	0.102364	0.720786	0.384342	0	0	0	0	0
Novel02219	1.664317	1.432552	1.527131	0.41883	0.286358	0.298224	0.310208	0.316008	0.260422	0.315568	0.35659	0.117172	0.154395	0.050436	0.094901	0.930188	1.071914	1.378688	0.140736	0.156433	0.295144	0.077217	0.163115	0.338245	0	0.064327	0.163924	0	0.667718	0
LOC107811332	4.464889	4.088035	4.779667	4.700397	3.054575	3.355766	5.114555	4.468404	5.065133	4.301325	4.522242	3.806989	3.083474	3.540477	3.564275	3.943396	3.275033	4.145544	2.768737	3.339837	2.14914	5.783008	5.123472	5.72535	3.240476	1.006666	3.884561	1.478253	6.568089	2.556211
LOC107829023	0	0	12.18055	10.98828	17.06505	6.186666	5.504588	6.223755	14.56738	8.511112	21.2977	6.132412	13.14001	5.654025	0	0	0	0	21.60162	18.95601	26.73826	4.756446	5.353221	9.954057	4.957942	17.27877	19.31197	0	6.293133	0
LOC107818334	0	0.014639	0	0.014552	0	0	0	0.01508	0	0	0	0	0	0	0	0.01197	0	0.035926	0	0	0	0	0	0	0	0	0	0	0	0
LOC107769637	0.142802	0.026029	0.074481	0.103497	0	0	0	0	0	0	0.080106	0	0.031794	0	0	0	0.021283	0.027592	0.127758	0	0	0	0	0	0	0	0	0	0	0
LOC107822069	2.395416	2.304411	2.845788	2.170125	2.119622	2.460904	1.518016	1.228025	1.474293	0.882426	1.418386	1.034343	1.214789	0.929167	1.584424	2.14208	2.417025	2.202591	1.836531	1.733661	1.602084	0.97801	1.150365	1.390938	0.269203	0.074068	0.503322	0	0.384413	0
LOC107783117	0	0	0	0	0	0	0.050967	0	0	0	0.042609	0	0	0	0	0	0	0.090606	0	0	0	0	0	0	0	0	0	0	0	0
LOC107823023	19.12866	19.93999	19.74423	16.40572	14.42426	15.33287	15.57748	12.43626	16.66776	13.4849	16.7717	10.03826	15.01608	13.31668	14.65348	24.92845	21.86837	25.96511	15.05171	16.49522	7.116362	17.58781	18.80806	20.57465	7.579613	5.847809	8.071867	2.209967	6.174192	5.732247
LOC107760449	4.862028	5.88073	5.483396	8.760378	9.979922	7.44255	9.565847	9.219651	7.143988	7.085199	8.098569	6.474395	10.9658	9.169027	13.00434	6.034158	5.727352	5.403584	9.995684	9.770146	7.434376	9.841579	7.349659	6.441971	5.575791	6.035827	3.631633	5.170315	6.874331	6.705423
LOC107830028	0	0	0.215284	0.336549	0	0	0.103861	0.105803	0	0.362247	0.086829	0	0	0	0.135092	0.127095	0	0.119629	0.18464	0.125653	0.104751	0.338803	0	0.218451	0.129426	0	0.146356	0	0.596158	0
LOC107773902	19.86819	19.4243	17.00183	20.8047	18.21997	29.12827	14.75981	16.09405	20.73646	19.47463	20.30031	18.09313	27.63256	25.10947	16.57856	15.1519	19.54333	16.15917	12.35835	14.62438	11.81338	12.92917	13.51933	18.55475	6.524526	4.66736	11.7108	3.690536	15.40378	3.190857
LOC107794880	13.40511	14.28421	10.7091	14.4538	10.9097	10.10829	9.259784	8.753832	12.13941	6.86192	10.29002	7.8448	9.657169	10.29941	10.76417	19.48061	16.78972	18.16711	4.82926	4.239101	4.791266	7.932797	9.644027	9.269761	0.392148	0.944075	1.053959	0.693171	1.119947	0.599319
LOC107782002	0.245684	0.358257	0.298995	0	0.173918	0	0.453344	0.461821	0.046132	0	0.206728	0	0.0547	0	0	0.183084	0.142411	0.036634	0	0	0	0	0.246212	0.260051	0	0	0	0	0	0

LOC107813274	0	0	0	0	0	0	0	0	0	0	0	0	0	0	0	0	0	0	0	0	0	0	0	0	0	0	0	0	0	0
LOC107769380	41.68832	32.93297	40.12922	30.10889	33.14838	48.47337	25.78988	27.26354	32.52492	23.51799	21.85126	33.04887	24.63408	33.27357	16.50263	24.33415	36.271	32.81054	21.02506	22.15484	27.89174	19.7262	30.63103	41.49361	18.23254	7.380518	26.54628	47.41637	13.56637	5.124556
LOC107820481	11.10688	25.09673	40.39091	16.25443	31.34522	17.52325	24.64005	40.88007	39.55093	30.05653	46.08372	54.07946	29.77682	26.31337	39.12355	66.65563	18.47622	46.20503	43.94824	64.43527	63.41525	58.34324	30.14461	46.573	37.88973	71.96815	37.20734	136.2998	98.08556	121.9091
LOC107831600	0.080183	0.073077	0.034851	0.072642	0.124165	0.043103	0.067253	0.068511	0.01882	0.039094	0.042168	0.050806	0.156207	0	0.082298	0.791723	1.20069	0.657581	0.996715	1.119188	1.151775	0.853781	0.777997	0.838076	0.304128	0.669416	0.426466	0	0.675554	0
LOC107802509	4.095811	4.461183	4.399846	5.143551	4.301947	4.027589	5.069211	4.324674	5.392838	3.961462	4.821666	4.093228	5.300898	4.232197	4.015852	5.223486	4.74506	5.101748	3.142452	3.647872	2.551038	4.963924	5.213559	5.081442	2.231322	1.969174	2.400781	0	2.084089	3.088417
LOC107772538	0.077744	0.088567	0.084476	0.176079	0.034396	0.041792	0.163017	0.182672	0.054742	0.056857	0.149913	0.06568	0.129818	0.106018	0.239382	0.840052	0.394311	0.347767	0.098611	0.197297	0.141807	0.876497	0.565744	0.32503	0.049146	0.59496	0.160802	0	0	1.201751
LOC107819207	0.112282	0	0	0.050861	0.049677	0	0.047088	0	0.105416	0	0	0.04743	0.062497	0.122495	0.057622	0	0	0	0.056968	0.094983	0.051202	0.046885	0.04952	0	0.212939	0.390582	0	4.014891	0	0
LOC107813393	7.369848	8.158192	6.362572	8.795573	8.367546	5.82503	14.16926	12.10519	10.01537	9.040592	15.11387	7.661986	11.10754	12.35392	15.56017	12.47616	9.346891	8.417426	4.883164	4.497866	4.833902	14.29083	9.216736	8.037109	3.361189	5.89991	3.122281	7.21984	5.346463	6.242312
LOC107805950	14.59211	12.29957	13.52781	11.72963	9.366839	13.91997	11.77918	10.52204	10.8885	7.320197	7.422632	7.553461	6.948359	10.26011	7.012324	11.62833	14.26018	13.42586	7.103966	7.599002	7.615702	8.664213	10.8624	11.98975	3.039263	3.755626	4.635673	0	6.700297	0
LOC107792711	9.027911	10.1558	9.039182	9.447494	8.911253	8.568924	10.24607	9.368445	8.923368	9.355379	10.78044	7.966848	10.34851	9.133898	11.22318	11.52353	10.17534	11.8509	8.238764	8.884619	7.051554	10.13968	8.528261	8.875417	4.501571	3.897023	4.173024	4.26154	5.809488	2.763413
LOC107814645	14.75486	13.18864	16.08194	8.38018	6.226743	7.870628	8.139297	6.78836	8.684461	7.691968	6.486221	6.088828	7.770439	7.243573	4.892673	12.64464	14.58339	12.18503	5.125104	3.600461	5.900238	6.255871	9.310562	7.355002	7.390104	2.368882	8.048797	0	13.66061	0
Novel00291	0	0	0	0	0	0	0	0	0	0	0	0	0	0	0	0	0	0	0	0	0	0	0	0	0	0	0	0	0	0
LOC107775890	9.302114	8.427267	9.818873	6.370595	5.928368	9.22703	5.526476	5.48789	5.406288	8.314841	6.677949	9.308799	7.766432	9.060848	4.091758	6.024191	8.933133	7.843291	4.101532	5.808033	5.65579	3.560534	6.15381	5.26639	3.920233	3.312846	6.217043	11.87916	7.730518	10.27078
NitaMp031	1.705854	0.914515	0.872272	0.363628	0.088791	0.755177	0	0.171474	0.188415	0.880636	0	1.949795	0.335114	1.204179	0	0.373884	2.326586	1.196976	0.81458	0	1.189705	0.1676	0.354041	2.307358	0.126866	0.698109	3.202172	0	0	0
LOC107790964	2.334214	2.949923	3.977002	0.59211	0.468172	0.40153	0.809226	1.063688	1.402536	0.789067	0.938405	0.867684	0.658281	0.441396	0.862478	3.618075	1.232759	2.946833	0.47372	0.816162	0.596076	0.805737	1.235359	0.618062	0.472185	0.562967	0.441416	6.677172	0.599346	0
LOC107796897	0.780024	0.387761	0.719152	0.171312	0.167325	0.406604	0.019825	0.060588	0.044383	0	0.082872	0.059908	0.105252	0.103148	0	0.28183	0.388202	0.352449	0.143912	0.119972	0.194017	0.13818	0.104247	0.222349	0.029884	0	0.027937	0	0	0
LOC107771450	0	0	0	0	0	0	0	0	0	0	0	0	0	0	0	0	0	0	0	0	0	0	0	0	0	0	0	0	0	0
LOC107807844	0	0	0	0	0	0	0	0	0	0	0	0	0	0	0	0	0	0	0	0	0	0	0	0	0	0	0	0	0	0
LOC107785071	0	0	0	0	0	0	0	0	0	0	0	0	0	0	0	0	0	0	0	0	0	0	0	0	0	0	0	0	0	0
LOC107799311	1.366089	1.333952	1.204477	0.371282	0.069075	0.188835	0.622005	0.400191	0.714561	0.190301	0.50632	0.263797	0.6083	0.447101	0.300454	1.294329	1.28205	1.702312	0.079212	0.016509	0.053396	0.505237	0.51642	0.407953	0	0.054309	0.069197	0	0	0
LOC107764789	6.478258	6.459223	7.267891	6.521082	4.997467	5.952923	7.012587	5.440581	5.614222	2.699624	5.940269	2.43245	3.698301	3.503528	6.364957	7.096993	5.723624	5.696706	3.146381	4.074991	1.918929	7.398513	4.883976	4.745539	1.540092	1.771987	1.570621	0	2.399126	3.423594
LOC107810684	3.668223	3.754021	3.384662	7.129151	7.507242	4.803049	7.339382	6.846277	6.560705	5.335496	5.87717	6.18072	6.570127	8.562619	9.969791	4.886818	4.157544	4.079306	10.16863	9.447877	10.6719	8.026525	7.501566	6.297226	8.705495	11.46276	6.733415	7.327648	9.964654	6.335523
LOC107821596	2.580755	2.874715	1.703319	2.684408	2.96025	1.901133	2.32493	2.654247	2.333188	4.101064	3.250626	2.01879	3.511361	3.545415	2.256399	3.205302	1.754492	3.064247	2.279301	3.638568	2.135757	1.835959	1.896997	1.998077	5.619367	4.122922	6.721835	3.417786	6.902595	2.955036
LOC107831928	6.605559	5.899145	5.54009	6.194805	5.756917	6.138225	7.48924	6.920244	8.320726	6.376518	6.330933	6.42185	7.501208	7.351192	6.779765	5.98612	6.381526	7.721177	4.614535	5.054335	4.571264	6.431267	6.324285	8.118423	3.315394	2.401709	3.648609	4.747647	5.273618	2.05242
LOC107795128	0	0	0	0	0	0	0	0	0	0	0.033149	0.199693	0	0	0	0.176144	0.091342	0.176224	0	0.039991	0.08623	0.671158	0.166796	0	0	0.131557	0.055874	0	0	0
LOC107824342	0	0	0	0	0	0	0	0	0	0	0	0	0	0	0	0	0	0	0	0	0	0	0	0	0	0	0	0	0	0
LOC107799224	0	0	0.035379	0	0	0	0	0	0	0	0.028538	0	0	0	0.041772	0	0	0	0	0	0	0	0	0.042539	0	0	0	0	0	0
LOC107770461	0.07275	0.066302	0	0	0	0.15643	0.061018	0	0	0	0.102024	0.061461	0.080986	0	0	0.271066	0	0.054238	0	0.061541	0.066349	0	0	0	0	0	0	0	0	0
LOC107821297	9.584099	8.249462	7.868406	14.53148	17.37354	15.8139	16.5796	18.82313	17.746	11.29273	10.12723	12.25783	9.557782	15.68367	13.86723	7.092484	8.35886	8.385865	21.74681	19.93091	26.04048	16.73044	18.96236	14.53899	25.16005	27.50462	23.83523	114.2331	26.27489	94.65127
LOC107808530	0.104864	0.215035	0.068367	0.237505	0.185582	0.197299	0.043977	0	0.024613	0.306768	0.183827	0.354369	0.233473	0.114402	0.10763	0.332117	0.455885	0.390904	0.186216	0.266124	0.334733	0	0	0.027401	0.430887	0.145911	0.650692	0	2.145632	0
LOC107793529	0.059153	0.05391	0	0.107179	0	0.095395	0.049614	0.025271	0.027768	0.057681	0	0.049974	0	0.032266	0.030356	0.110202	0.057147	0	0	0.02502	0	0.0741	0	0	0	0	0	0	0	0
LOC107776692	0	0	0.032876	0	0	0	0	0	0	0	0	0	0	0	0	0	0	0.028196	0	0	0.034492	0.031584	0	0.079057	0	0	0	0	0	0
Novel02578	6.262798	3.712045	4.035499	4.404121	3.952846	4.237724	1.799937	1.646494	2.179223	1.153073	0.737031	1.775995	1.755147	3.965661	1.438425	1.305467	1.692417	1.665233	2.266488	2.0747	1.358045	0.841225	1.197554	1.739463	0.055371	0.121877	0.207052	0	0.210849	0
LOC107806981	12.32869	10.58985	11.01531	9.739346	9.492449	7.697155	9.534839	9.205004	12.73433	5.576079	8.837347	4.966259	6.136542	5.714375	5.634357	11.98276	9.722896	12.44868	6.93985	7.217253	4.151299	11.42303	13.8809	16.80671	1.735125	2.323332	3.784809	0	2.53276	5.657152
LOC107799167	0	0	0	0	0	0	0	0	0	0	0	0	0	0	0	0	0	0.023582	0	0	0	0	0	0	0	0	0	0	0	0
LOC107796778	0	0.093056	0	0.185004	0.090349	0.109775	0.256919	0	0.287581	0	0.214788	0.086261	0	0.111391	0.104798	0	0	0	0.414436	0.172747	0.372485	0.255811	0	0	0	0	0	0	0.491569	0
LOC107823416	8.962998	8.691462	8.539313	11.59539	13.67313	11.79485	12.93036	13.14152	11.30957	13.87921	15.88813	15.11409	16.00429	16.2318	14.49822	9.88527	8.451194	9.969985	16.95314	15.98302	16.97007	13.29378	11.70082	11.84131	18.9017	16.81139	16.18693	25.63924	18.29605	6.650348
LOC107763326	0	0	0	0	0.140879	0	0	0	0	0	0	0	0	0	0	0	0	0	0	0	0	0	0	0	0	0	0	0	0	0
LOC107797672	0.189165	0.362041	0.493311	1.388128	1.556686	1.057552	2.697233	4.056853	3.534167	3.228044	5.982179	3.292122	1.558299	1.42395	5.727533	2.974381	0.091375	1.170553	5.336251	7.584971	3.778222	9.920927	3.487289	5.931453	4.065753	10.97582	3.621959	4.05839	5.555314	3.508905
LOC107832530	0	0	0	0	0	0	0	0	0	0	0	0	0	0	0.274071	0	0	0	0	0	0	0	0	0	0	0	0	0	0	0
LOC107786922	2.029618	3.557205	2.103593	3.889624	3.177432	2.937426	3.862975	3.501671	3.737702	1.903013	3.202131	1.879551	2.650449	1.788403	3.004535	3.548496	3.016587	3.055432	1.980304	2.608394	1.637464	4.009297	3.064094	2.978058	0.937597	0.597398	0.691975	2.791277	0.563729	0
LOC107762460	0.816611	1.16952	1.014089	0.528434	1.651637	1.003379	0.684927	0.897087	1.204768	0.455028	0.818012	0.788452	1.038929	1.399958	1.317088	0.521605	1.239723	0.86974	1.420526	0.789482	0.744763	0.876823	0.926106	0.853522	1.327429	0.81161	0.827287	0	1.68491	0
LOC107808341	6.913408	8.209406	8.143408	4.651495	3.82589	3.510586	5.855237	6.234101	4.228412	4.852963	6.632013	3.481554	4.412098	2.874384	5.870736	8.407465	5.373604	7.504706	5.461383	6.781704	4.970179	5.999244	5.899448	5.766591	3.530425	7.332129	4.311781	8.052222	6.721771	11.13919
LOC107809534	0.064152	0.087699	0	0	0.028383	0.034485	0.215227	0.219251	0.120457	0	0.067475	0.027099	0	0.139972	0.19753	0.095612	0	0	0	0.027134	0	0.776833	0.028293	0	0	0.089262	0	0	0	3.966589
LOC107761676	3.099321	3.533625	3.831953	1.230522	0.917803	1.73909	0.403912	0.622472	0.301411	0.469589	1.307411	0.260796	0.096221	0.242477	0.544962	2.788104	1.646216	3.50744	0.701668	1.504147	0.337845	0.794028	0.707958	0.516241	0.062446	0.120268	0.175131	0	0.118894	0
LOC107771454	0.101119	0.245754	0.0293	0.183218	0.268431	0.072477	0.339252	0.230397	0.12658	0.032868	0.165445	0.028476	0.15009	0	0.103786	0.175826	0.195379	0.125648	0.376232	0.114053	0.215186	0.422237	0.178388	0.24661	0.042615	0	0	0	1.298197	0
LOC107815867	0.108335	0.049367	0.105945	0.061341	0.047931	0.043677	0.090865	0.092564	0.050855	0.039615	0.104451	0.091524	0.09045	0.073867	0	0.07064	0.039248	0.090864	0.123672	0.114554	0.148204	0.067855	0.071669	0.113231	0.034242	0.188425	0.096032	0	0.13039	0
LOC107797352	0	0	0	0	0	0	0	0	0	0	0	0	0	0	0	0	0	0	0	0	0	0	0	0	0	0	0	0	0	0
LOC107789748	55.02455	50.03051	62.91576	20.68782	20.72008	26.28479	17.7464	18.62942	27.31351	7.862821	12.52938	14.00583	9.550756	12.45621	10.3285	36.96642	53.09603	44.72628	12.37144	11.78681	20.76754	21.71024	32.66033	38.02646	3.34382	2.961978	7.014282	9.226339	4.037282	0
LOC107819375	15.11073	13.07037	13.02843	22.69511	21.67676	21.53986	18.24956	18.24897	17.97164	18.36607	20.026	19.81195	23.57062	23.06566	21.28967	16.23719	15.37126	15.44157	22.42226	21.63416	17.9914	19.40452	17.1291	17.24122	15.56379	21.75345	16.98669	6.602633	25.7805	39.9607
LOC107769961	1.790971	2.258317	2.068695	0.488981	1.780151	0.975945	0.329241	0.628872	0.506736	0.765555	0.533298	0.849802	1.33826	0.776191	0.226628	1.444329	1.730228	1.79252	2.813163	2.781029	2.125652	0.635155	0.649214	0.794926	2.698587	1.263071	0.695929	5.263551	5.197045	6.067859
LOC107796830	25.40766	25.58508	24.07587	33.20164	31.65989	33.82617	30.25265	26.33794	29.2121	32.54456	34.67695	29.98261	36.28242	30.84751	30.95429	26.4469	28.15646	29.11571	28.07497	28.55131	27.87535	28.67052	31.4064	36.48811	27.10493	20.07497	27.66715	37.29319	38.35624	14.33062
Novel00405	0.044465	0	0.231915	1.248774	2.12466	0.860495	2.163093	2.811415	0.960151	2.731616	1.870733	3.117917	1.03948	3.29862	3.057723	0.099406	0.128871	0.066301	3.11328	3.723818	4.623022	2.265167	1.372737	1.301294	4.94712	4.949583	2.837918	15.89938	4.281406	0
Novel00176	6.258955	5.514755	6.687998	7.007808	6.274327	6.773854	4.168331	4.246277	6.071405	7.035998	6.123884	10.50521	9.189767	7.917097	4.311136	5.26851	5.363664	5.286425	4.53654	3.957791	5.670344	3.455736	4.181878	5.2161	2.944455	1.244126	6.930625	0	3.103382	2.571433
LOC107781083	4.737868	5.127604	4.890752	5.603795	4.541742	5.412149	5.105298	5.847346	4.509899	2.342093	2.560859	2.751844	3.406293	3.266382	2.36387	5.124368	4.926824	5.421071	4.841048	4.787226	5.731347	4.835988	5.891388	6.465135	2.703867	2.701052	2.411016	0	3.960032	8.137486
LOC107790792	11.83637	11.3828	12.79456	7.259014	5.916901	6.073553	7.816428	7.61782	11.07642	3.316437	5.658864	3.555085	3.743298	4.423085	3.451284	12.2427	9.968433	10.94469	2.846698	2.73075	4.258403	11.00814	11.54213	14.21904	1.287553	0.588194	2.06665	0	1.295102	0
LOC107821760	0	0	0.011383	0	0	0.014079	0	0	0	0	0.009182	0.011063	0.014577	0.028572	0	0	0	0.029289	0	0	0.011943	0	0.023101	0	0	0.018221	0	0	0	0
LOC107807378	8.848087	7.14711	6.638103	9.445114	8.072141	10.27478	11.00726	9.259591	10.23883	9.898793	10.58095	9.445294	9.824362	9.503172	8.846772	7.9907	7.068111	8.590953	7.052822	7.736303	6.130397	10.0433	10.62797	13.50163	5.232009	4.294698	6.78266	1.635047	6.053957	8.482024
LOC107818708	0.146149	0.639344	0.457359	0	0	0	0.122581	0.024975	0.164653	0.028503	0	0	0	0.159441	0	0.021782	0.112954	0.021792	0	0	0	0	0.438304	0	0	0	0	0	0	0
Novel04317	8.348323	7.521513	10.15983	4.883639	3.54583	6.000741	4.121235	4.116779	5.777558	2.186351	3.278155	2.418135	2.814594	2.654218	1.175107	4.550349	6.682579	5.726107	3.001261	2.300229	2.784479	2.430206	4.965287	4.487485	1.025325	0.995664	1.071279	20.46936	0.459335	11.79861
LOC107796882	0	0	0	0	0	0	0	0	0	0	0	0	0	0	0	0	0	0	0	0	0	0	0	0	0	0	0	0	0	0
LOC107831117	0.110273	0.201001	0.239645	0.149853	0.63425	0.237114	0.231227	0.235551	0.362353	2.526962	0.618589	1.723494	0.552409	1.142875	0.565907	0.164351	0.266333	0.164427	0.895181	0.606343	0.452569	0.276276	0.340439	0.46103	3.415761	2.60843	1.629175	0	2.919919	0
LOC107799382	10.79719	8.213811	11.09227	6.628926	6.395696	8.346464	6.511389	6.404419	9.969327	5.307819	4.129635	5.578601	4.072751	4.86744	2.381244	6.450163	6.724112	6.719225	1.448759	2.717459	4.313245	7.004955	11.01931	16.22833	0.676906	1.241612	2.847588	0	3.866394	5.51741
LOC107763614	18.87838	18.40404	17.48668	21.30847	19.99103	19.88065	19.98732	19.56778	23.38967	17.20167	14.91935	20.13232	20.75728	23.54964	13.49985	11.9349	15.1735	15.97817	17.66478	17.34426	17.92303	12.01821	17.74391	20.29775	11.44491	10.98084	17.83189	44.26462	8.567239	0
LOC107822426	0	0	0	0	0	0	0	0	0	0.067457	0	0	0	0	0	0	0	0	0	0	0	0	0	0.063378	0	0	0	0	0	0
LOC107818853	0.272811	0.905737	1.50759	0.670844	0.775933	0.272355	1.340223	2.047927	1.701417	0.798076	2.664472	0.905451	0.520624	0.637765	4.740102	5.924722	0.414164	1.757891	1.779622	2.32428	1.866063	4.898384	1.753217	1.629384	1.207207	3.226574	0.875197	2.787123	1.782486	14.45857
Novel01249	0	0.04577	0	0.136491	0.044438	0	0.463341	0.429096	0.141447	0.048971	0.246501	0	0.447247	0.273939	0.97935	0.112273	0	0	0.20384	0.212415	0.091603	0.04194	0.17719	0.05249	1.333368	1.187924	1.543265	3.591461	0.241778	0
LOC107823451	2.008391	2.040792	1.886323	1.798587	2.369542	1.886255	1.703886	1.913268	2.470742	2.431161	2.687073	3.022936	3.340804	2.719931	1.966582	1.806308	1.851067	2.512779	1.358651	1.620847	1.57904	1.889327	1.873346	2.943663	2.072234	0.706663	2.264618	0	1.889366	1.427377
LOC107789055	0.962026	1.38437	0.836268	0.229354	0.179213	0.217746	1.104168	0.670563	0.380291	0.024687	0.674573	0.064164	0.310009	0.082857	0.363778	1.943183	1.491928	1.094721	0.231205	0.299824	0.207801	3.847924	1.027217	0.238147	0.032008	0.774972	0.14961	0	0	0
LOC107785859	1.625324	1.009965	1.509191	1.372066	1.144018	2.700561	0.929475	1.041541	2.184848	1.116631	1.16558	1.435533	1.480364	1.450758	0.909921	1.183666	1.320395	1.762543	1.23695	0.656208	1.280188	1.048862	1.726889	2.084876	0.373619	0.051398	0.654887	0	1.778384	0
LOC107786480	0	0	0	0	0	0	0	0	0	0	0	0	0	0	0	0	0	0	0	0	0	0	0	0	0	0	0	0	0	0
LOC107773075	2.684934	2.06062	2.252063	1.706957	1.417147	1.266067	3.200167	1.408646	2.0785	0.872717	1.486324	0.795897	0.786554	1.027765	0.773542	1.088163	1.683741	1.931496	1.625132	2.032188	1.847269	1.927555	1.288017	1.23083	0.416877	1.245296	4.119821	0	0	0
107807952	0	0	0	0	0	0	0	0	0	0	0	0	0	0	0	0	0	0	0	0	0	0	0	0	0	0	0	0	0	0
LOC107784224	1.040658	0.948431	0.904621	1.346833	1.096241	2.344224	0.457204	0.973849	1.116589	0.628192	0.625465	0.544252	0.827482	1.351555	0.559482	0.886285	1.005363	0.960582	1.659409	1.257607	0.813512	0.86908	1.48617	1.55385	0.751833	0.137905	0.702842	0	1.19288	0
LOC107822191	0	0	0	0	0	0	0	0	0	0	0	0	0	0	0	0	0	0	0	0	0	0	0	0	0	0	0	0	0	0
LOC107805951	0.186184	0	0.107897	0.168673	0	0.066724	0	0.053027	0	0	0	0.104862	0.069088	0.406235	0.063698	0.046248	0	0.046269	0	0	0.056601	0.051829	0.054742	0	0.078464	0	0	0	0	0
Novel00218	1.809483	1.323636	1.966181	1.402033	1.938233	1.663842	1.198177	1.546071	1.676469	1.555523	1.252115	2.15225	1.961332	1.766261	0.806417	1.312944	1.794115	1.810562	1.352944	1.711965	2.193133	1.391845	1.512079	1.74196	2.046922	1.755808	1.603997	3.405358	2.292498	0
LOC107800446	0	0	0	0	0	0	0	0	0	0	0	0	0	0	0	0	0	0	0.400638	0.333993	0.120028	0.439635	0.232173	0.137556	0	0	0.777747	0	0.633605	0
Novel01357	2.029474	1.660879	1.308133	2.401438	2.614312	2.508481	1.459035	0.967286	1.360971	1.628968	1.413388	1.504611	3.304329	3.057507	1.062753	1.440349	1.253744	1.358665	1.667109	1.635043	1.019892	0.79555	1.010754	0.822508	0.296734	0.26894	0.342668	0.98732	1.595202	0
LOC107810323	3.578269	5.27335	5.360671	1.517406	0.875782	2.946697	0.638563	1.170908	0.929206	0.445438	0.533848	0.06432	0.93228	0.830578	0.390706	2.836734	3.971767	2.724512	1.004314	1.159265	0.763784	0.317905	1.275939	1.03447	0.192511	0	0.359934	0	0.366533	0
LOC107784237	26.95791	26.72121	31.59576	12.99402	11.60425	17.50543	7.150412	7.694263	13.93452	5.542066	5.116171	6.504767	8.656709	8.378848	3.764069	21.20513	20.0521	26.96947	11.47586	11.36978	4.938228	6.991296	10.97477	11.33879	1.772122	1.790006	4.470677	2.746256	1.571471	2.374428
LOC107768104	0	0	0	0	0	0	0	0	0	0	0	0	0	0	0	0.663458	0.286704	0.331881	0.451711	0	0.541317	0.37176	0.130885	0.775458	0.375206	0.206466	0.175379	0	6.429386	0
Novel03103	3.574975	3.403602	4.162031	2.747151	1.86413	2.848334	1.927598	1.200004	2.247552	0.778137	1.208623	1.240455	1.030461	0.97503	0.425896	0.023786	0	0	0	0	0.026657	0	0	0.040356	0	0	0	0	0	0
LOC107774181	0	0	0	0	0	0	0	0	0	0	0	0	0	0	0	0	0	0	0	0	0	0	0	0	0	0	0	0	0	0
LOC107788081	12.61187	15.20562	12.20946	5.646975	3.824119	6.277039	6.413091	4.828749	7.100444	3.808986	5.288586	4.388326	5.296693	3.89874	4.030487	11.50411	10.55824	11.12212	4.301023	4.358905	2.728701	6.888636	7.367461	7.687643	0.499109	1.185318	1.964575	0	1.400415	1.284696
LOC107795512	0.057814	0	0	0	0	0	0	0	0.054279	0	0	0	0	0	0	0	0	0	0	0	0	0	0	0	0	0	0	0	0	0
LOC107785471	0	0	0	0	0	0	0	0	0	0	0	0	0	0	0	0	0	0	0	0	0	0	0	0	0	0	0	0	0	0
LOC107794168	0.395485	0	0.152794	0.19905	0.11665	0	0.11057	0.037546	0.165022	0.257098	0.123251	0.18562	0.195671	0.335576	0.045102	0	0.339619	0.098284	0.17836	0	0	0.036698	0	0.229645	0.055557	0.122286	0.103874	0	0.423112	0
LOC107831158	0	0	0	0	0	0	0	0	0	0	0	0	0	0	0	0	0	0	0.029995	0.125027	0	0	0	0	0	0	0	0	0	0
LOC107775744	7.168024	8.610329	7.926374	7.480567	6.477214	7.842659	8.667664	7.747669	8.750931	5.384313	8.844731	7.382446	10.6018	5.996225	7.69502	6.643993	8.931425	7.062476	3.983782	3.9853	3.603632	8.947576	8.601513	8.630379	1.761275	1.127777	2.634411	0	2.194945	1.566112
LOC107802337	11.51522	12.73618	12.43294	11.6891	11.40591	10.11575	11.37206	10.09755	9.579516	8.424672	10.56846	6.798145	9.519408	10.4986	12.34966	12.75425	10.66165	14.06713	11.56967	11.29881	8.776654	13.40856	11.72582	11.40294	9.679252	9.968053	7.244789	9.921702	12.62999	23.39552
LOC107774056	0.759627	0.849649	0.960477	0.594341	0.672168	0.705325	0.463369	0.295021	0.356586	0.134679	0.217903	0.291707	0.153751	0.414358	0.212635	0.952036	0.533719	0.978214	0.73578	0.525758	0.724284	0.288357	0.700498	0.541338	0.174618	0	0.08162	0	0.332465	0
LOC107821708	3.102148	4.325654	3.640452	1.58787	1.67444	1.284047	2.510838	1.842139	1.907643	3.554358	3.687011	1.257975	3.211619	2.267475	1.974055	3.536938	2.172778	2.555625	0.865657	0.997197	0.424382	2.00778	1.477602	1.394216	4.726079	0.669044	2.969856	6.655422	10.45439	1.918105
LOC107771166	0.013355	0	0.01161	0	0	0	0.033605	0	0	0	0.009365	0.022566	0	0.01457	0	0	0.025805	0	0	0	0	0	0.01178	0	0	0	0	0	0	0
LOC107831901	0.053889	0.049113	0	0	0	0	0	0	0	0	0	0	0	0	0	0	0.052061	0	0	0	0.049147	0	0	0	0	0	0	0	0	0
Novel02966	17.97251	27.7667	24.35368	26.12114	26.61882	23.45583	24.78798	20.81709	15.47478	18.65009	30.69712	10.19011	13.32033	10.32781	38.07671	32.08622	22.284	29.05563	29.25763	38.45592	11.26349	40.37263	21.15138	16.47442	10.38926	25.20802	5.395709	13.7464	10.17953	20.79912
LOC107786965	5.282317	7.379401	4.65884	9.116923	10.81535	9.534317	11.64221	10.64098	9.140264	10.03867	11.4904	8.094657	13.28444	13.52353	14.04874	6.249376	4.842417	6.324103	11.56145	10.82872	7.156013	10.1108	8.587493	5.621826	9.383959	12.33935	6.835701	5.514749	10.11669	8.344143
LOC107807230	0.166436	0.216694	0.248022	0.043081	0.147273	0.051125	0.039885	0.182838	0.223225	0.115926	0.200066	0.080348	0.211747	0.28533	0.048807	0.159465	0.091881	0.141811	0.120634	0.080453	0.065054	0.079426	0.125835	0.049703	0.030061	0	0.168612	0	0	0
LOC107775138	2.790136	3.040951	3.425573	2.006542	2.31618	2.690486	2.653843	2.015313	2.214425	2.243905	1.855597	1.944069	2.081352	1.851445	1.594235	1.843425	2.695506	2.273169	1.4594	1.167965	1.810112	1.72957	1.674552	2.10451	2.000174	0.840491	1.291889	0	1.80026	1.778539
LOC107831867	0	0	0	0	0	0	0	0	0	0	0	0	0	0	0	0	0	0	0	0	0	0	0	0	0	0	0	0	0	0
LOC107817689	0.030685	0.139829	0.13337	0.667183	0.597352	0.362895	0.591952	0.865205	0.489748	0.837816	0.537913	0.103695	0.512389	0.60257	1.795188	0.388734	0.177867	0.434666	0.653883	1.349797	0.419782	0.5894	0.433063	0.513156	0.426751	0.384267	0.544013	0	0	0
LOC107814342	0	0	0	0	0	0	0	0	0	0	0	0	0	0	0.101245	0	0	0	0	0	0	0	0	0	0	0	0	0	0	0
LOC107802313	0	0	0	0	0	0	0	0	0	0	0	0	0	0	0	0	0	0	0	0	0	0	0	0	0	0	0	0	0	0
LOC107794473	0.347269	0.158246	0.150937	0.419478	0.256072	0.622261	0.339815	0.148358	0.163016	0.056439	0.121753	0.146691	0.386585	0.252569	0.178214	0.172524	0.279576	0.302055	0.998423	0.832336	0.527859	0.773369	0.357367	1.028403	0.146351	0	0.136815	0	1.114585	0
LOC107817920	0	0	0	0	0	0	0	0	0	0	0	0	0	0	0	0	0	0	0	0	0	0	0	0	0	0	0	0	0	0
LOC107786640	0.71265	1.298985	1.032486	0.807031	0.788249	0.830035	0.846784	0.811876	1.003601	0.810744	0.832851	0.9031	0.594999	0.453522	0.792398	1.150646	0.631106	0.796966	0.903935	0.401902	0.595786	1.140709	0.471453	1.05522	0.825922	0.413167	0.631721	0	0.857737	7.344035
LOC107779120	14.36617	14.26282	13.17485	16.27996	15.42056	16.29463	14.86523	16.15556	14.22918	18.67845	16.23541	16.64138	17.20169	15.13421	13.83298	14.16389	14.1174	17.11486	12.52388	12.36161	12.24672	11.79142	13.58638	14.13833	12.60814	12.91417	13.42035	17.65266	16.39968	15.26258
LOC107781783	45.77575	37.26673	44.98213	28.68514	28.65797	38.90477	23.67383	23.11166	25.48001	20.99533	23.66116	21.85844	27.29184	26.25257	21.07734	33.9099	45.70898	47.75189	23.31683	19.4381	25.6595	20.24752	29.44506	38.01109	11.55202	11.45478	15.93159	19.40877	18.29247	22.37457
LOC107783348	12.41024	8.970318	11.15995	9.537205	7.270441	7.361392	5.527538	7.897886	10.12456	2.42031	3.600855	2.458438	3.525271	2.427675	1.58121	0	0	0	0	0	0	0	0	0	0	0	0	0	0	0
LOC107780385	0.63666	1.476967	1.132026	0.471912	0.409715	0.497809	0.242725	0.024726	0.407539	0.112877	0.101461	0.195588	0.225508	0.157856	0.089107	1.682105	1.230134	1.078766	0.352385	0.391688	0.237536	0.120839	0.53605	0.332718	0	0.161067	0.239426	0	0.557292	0
LOC107783916	0.031031	0.084842	0.134871	0.112449	0.054916	0.066724	0.104107	0.132567	0.116532	0.030259	0.130552	0.131078	0.103631	0	0	0.184993	0	0.231347	0.125951	0.104999	0.0283	0.077744	0.164227	0.421632	0	0.129531	0.073351	0	0	0
LOC107810028	0	0	0	0	0	0	0	0	0	0	0	0	0	0	0	0	0	0	0	0	0	0	0	0	0	0	0	0	0	0
LOC107801878	0	0	0	0	0	0.046197	0.01802	0	0	0.02095	0	0.018151	0	0.046878	0	0	0	0	0	0.018175	0	0	0	0	0.029894	0.025393	0	0	0	0
NitaMp048	0.091095	0	0.052791	0.055018	0.053738	0.032646	0	0	0.057016	0.088829	0	0.205226	0.033803	0.09938	0.093498	0.045256	0.117341	0.158469	0.092437	0	0.08308	0.050717	0	0.190425	0.345516	0.211254	0.107667	0	0.438565	0
LOC107777396	7.195022	6.793441	7.030036	4.302095	2.470252	4.950738	2.389771	2.262325	3.323462	2.553844	3.390343	3.233791	3.107724	2.982778	1.860963	6.820132	5.838837	7.552786	2.015079	2.605019	1.758611	2.78806	3.934821	3.308903	0.764122	0.200227	1.666772	0	0.831346	0
LOC107775936	1.166425	1.162713	1.394179	1.717173	2.128767	1.136482	0.275156	0.840902	0.376438	1.492853	0.562305	0.523509	0.81155	0.357894	0.636005	0.896384	1.831155	0.896795	4.771417	5.611942	3.956003	0.395733	0.8038	0.68577	0.645188	1.521558	1.550948	0	0.175487	0
LOC107776583	0	0	0	0	0	0	0	0	0	0	0	0	0	0	0	0	0	0	0	0	0	0	0	0	0	0	0	0	0	0
Novel03960	1.606373	1.737658	1.396386	0.707245	0.783776	1.01686	1.334742	1.051844	1.353092	0.49774	0.800052	0.532697	0.685212	0.622374	0.677985	1.353697	0.812205	1.275968	0.411318	0.279396	0.465526	1.028084	1.668531	1.333769	0.018981	0.020889	0.070976	0	0.289108	0
LOC107830418	1.730083	2.473878	1.244627	2.080816	2.87702	1.924191	3.127361	4.562124	2.072355	2.239704	2.447187	1.738827	1.494274	3.156574	5.32713	2.378463	1.873127	1.712388	5.024559	4.81956	3.264548	4.384335	2.631117	1.309446	8.18371	17.34905	2.43263	6.39959	4.308226	7.377493
LOC107809776	2.239515	3.288345	2.941775	3.471651	3.104616	4.815504	2.316666	2.445031	2.523066	1.310286	1.081796	1.408492	0.85872	1.330187	0.766192	2.781482	2.524153	2.949721	5.630762	4.63094	4.470751	1.994962	3.050896	2.886913	0.440444	0.519354	0.941129	17.79522	1.916768	0
LOC107782052	4.175981	4.987031	4.130794	11.93673	12.99685	13.81741	9.632082	9.873715	7.570804	14.95447	15.01965	14.23359	15.86991	11.90004	11.26952	5.204461	5.112494	5.394721	18.59384	17.63251	13.46131	7.095199	8.31961	5.907442	22.02913	20.78783	23.74229	10.29799	15.59844	24.48516
LOC107811461	111.8061	106.3216	127.75	45.60918	44.75559	71.04569	42.42275	47.22991	58.95291	20.69034	27.28576	27.11999	20.5697	30.2375	21.69733	73.92435	96.74374	96.19821	25.82092	24.3074	41.98726	45.31299	69.26909	89.36333	5.048462	3.268279	17.30481	16.79773	6.784973	4.841135
LOC107797251	0	0	0	0	0	0	0	0	0	0	0	0	0	0	0	0	0	0	0	0	0	0	0	0	0	0	0	0	0	0
LOC107784066	10.30928	9.67694	10.75933	8.528751	7.073899	9.159012	7.326508	6.408597	6.838914	5.598291	5.886984	6.336598	6.769032	6.835698	3.928334	0.023001	0.059638	0.069036	0	0.052221	0.056301	0	0	0	0	0	0	0	0	0
LOC107807718	0	0	0	0	0	0.017135	0	0	0	0	0	0	0	0	0	0	0	0	0	0.016381	0.01766	0.016171	0	0	0	0	0	0	0	0
LOC107763753	13.43071	10.00836	14.28474	3.22082	3.145864	6.540314	3.048153	2.835139	4.227838	1.540781	2.4929	3.070266	2.198713	3.275207	1.621754	7.65362	14.27271	9.424153	1.603359	1.136145	1.873381	2.837073	4.250901	6.027985	0	0	0.747013	0	0	0
LOC107823415	26.0022	25.09728	24.6499	34.12936	35.5997	32.6148	29.82303	30.84721	30.40289	30.18028	26.2724	28.28084	35.89761	33.05775	31.3811	30.05704	30.92238	26.48367	36.63462	35.59207	36.94101	34.08304	33.56071	33.16938	29.98413	29.34297	34.322	26.84354	37.45659	65.40737
Novel02429	1.829135	1.995385	1.517754	2.410332	2.476854	1.757971	2.812652	2.794208	2.471831	1.432315	1.651821	1.147271	1.480886	1.511739	1.280027	2.085915	1.713549	2.024884	0.956162	0.750217	1.314341	2.407061	2.664584	2.549081	0.175196	0.34706	0.196535	0	0.933981	0
LOC107767763	0.184544	0.112126	0.106947	0.111458	0.027216	0.264543	0.283773	0.1314	0.288764	0.3899	0.172537	0.233862	0.376635	0.771759	0.063137	0.068761	0.029714	0.114655	0.187263	0.104074	0.336615	0.102745	0	0	0.23332	0.085593	0.254469	0	0.296154	0
LOC107830823	6.310197	8.532937	8.11649	10.36446	9.169941	10.03846	8.390761	7.714814	9.488498	10.35545	7.716269	8.711682	9.651685	9.682535	7.950964	8.334274	7.70696	8.395463	10.77604	9.591026	9.310944	9.211456	8.688407	8.606213	17.22082	11.81398	14.43121	29.35078	23.46387	26.9629
LOC107779743	0	0	0	0	0	0	0	0	0	0	0	0	0	0	0	0	0	0	0	0	0	0	0	0	0	0	0	0	0	0
LOC107766950	0.986865	0.933998	1.319786	0.653344	1.242692	0.285654	1.177918	2.497182	2.10247	2.109697	2.980882	1.956061	1.859156	2.111834	4.558011	1.15969	0.330021	0.962134	1.348042	1.412772	1.800075	3.296627	1.37268	1.626551	1.967523	9.08392	3.00571	0	0.730942	0
LOC107804470	4.361873	5.878022	4.990764	5.403947	4.42048	2.845794	6.847945	5.797405	5.355155	7.088962	7.711724	3.968496	5.39522	5.978741	5.96921	8.39011	5.258416	7.6435	3.556034	3.910605	3.162081	5.074893	5.91917	3.156251	5.231927	3.683036	5.155383	7.998349	7.179354	0
107815969	0	0	0	0	0	0	0	0	0	0	0	0	0	0	0	0	0	0	0	0	0	0	0	0	0	0	0	0	0	0
LOC107824630	0.015853	0	0	0	0	0	0	0	0	0	0	0	0	0	0.016272	0	0	0	0	0	0	0	0	0	0	0	0.056212	0	0.457945	0
LOC107804645	10.99853	11.68521	11.88496	18.49692	14.1413	14.24199	19.26535	17.7565	22.07805	8.591819	12.14349	9.599885	10.75553	11.00448	17.27592	6.294259	6.633605	6.614263	5.857749	6.32261	6.927382	14.1076	10.45182	12.13078	2.919385	5.58034	3.66281	0	2.632916	15.02886
LOC107792250	8.645493	5.78721	6.893384	2.646804	3.06004	3.717985	4.600855	4.330277	6.437438	2.238429	2.759358	2.367488	4.911703	3.902817	2.570254	5.176321	6.480212	6.001059	0.907536	1.134852	1.005996	4.680602	4.996277	4.580452	0.150766	0.082963	0.916121	0	1.148206	1.843324
LOC107795178	0	0	0	0	0	0	0	0	0	0	0	0	0	0	0	0	0	0	0	0	0	0	0	0	0	0	0	0	0	0
LOC107798549	3.810492	3.649078	3.581406	5.362165	6.024693	5.98915	6.083802	5.354697	4.794157	4.696508	4.733502	4.297727	5.61997	5.423169	5.141853	3.718845	3.942868	3.634024	4.985394	5.857778	4.286709	5.298352	4.828391	4.912702	5.257809	5.409677	3.520655	8.299616	5.680452	10.76384
LOC107791789	0.96581	0.898944	1.000324	1.861648	1.927423	1.458127	1.533955	1.264158	1.50481	1.562965	1.570589	1.666607	1.692794	1.614103	1.898197	1.163807	1.429358	1.026457	1.501333	1.668783	1.29314	1.956368	1.758193	1.653803	0.805391	0.972149	0.874348	0	1.879687	1.270584
LOC107799155	0	0	0	0	0	0	0	0	0	0	0	0	0	0	0	0	0	0	0	0	0	0	0	0	0	0	0	0	0	0

LOC107790217	14.49343	14.48352	12.15677	17.56467	15.13094	15.71889	23.24619	22.81186	23.27525	16.79834	17.02715	16.21853	19.53098	18.10014	13.63602	11.89005	11.66823	11.32678	14.90044	14.46517	13.798	20.4385	20.8023	21.85906	16.31488	20.52035	30.20301	18.18395	9.793197	0
LOC107775098	16.92465	18.35172	16.41833	15.37906	10.10432	18.60713	13.46856	12.69683	13.66411	39.64361	29.11488	25.47444	13.93195	15.71105	13.2637	17.03796	18.91158	17.8819	13.26844	20.44067	9.066072	12.41002	13.26493	11.82862	58.77979	23.44302	89.14245	7.291277	35.83214	18.91223
LOC107822895	0	0	0	0	0	0	0	0	0	0	0	0	0	0	0	0	0	0	0	0	0	0	0	0	0	0	0	0	0	0
LOC107790483	14.66633	9.86579	9.612441	11.12519	9.424309	8.009195	9.372408	7.658025	9.452802	3.405125	5.794976	5.703575	4.988734	5.142937	3.046465	8.50062	8.208972	10.06657	5.728536	4.775598	6.634892	7.825263	12.834	14.47762	0.662239	0.809807	2.476347	0	1.96136	0
LOC107785079	7.726517	8.230637	6.658345	7.696786	5.120298	6.041418	5.807257	6.344535	5.464048	3.392079	5.699284	2.995338	4.840539	3.685516	3.776326	6.4308	8.498503	6.508556	3.22438	4.895002	3.843662	6.955417	8.880541	4.999276	0.59204	0.930812	1.18599	0	3.864748	0
LOC107794855	2.090493	2.590252	2.225585	4.085675	3.242356	3.964753	3.467371	2.307977	2.381639	3.41276	3.606991	3.055959	3.843746	3.510625	3.49568	2.39802	2.428049	2.591748	1.906775	2.185679	2.292162	2.60893	2.506947	2.455036	1.247267	0.980484	2.276462	0	1.809326	0
LOC107792251	2.535403	2.209138	1.501608	3.079423	3.032412	3.055369	2.687401	2.523403	1.961826	3.097215	3.731482	2.706897	3.411748	3.799451	3.174196	2.387685	2.341746	1.786391	3.10992	3.016829	2.922176	3.304046	2.752484	2.504398	2.958942	4.148106	3.819896	1.992497	4.426473	10.33634
LOC107832568	2.150237	1.82903	1.794389	2.233713	4.667886	2.25012	3.679132	3.453974	3.875975	3.71824	3.41756	3.342506	2.936239	3.221569	1.765555	3.055164	1.85573	2.757319	12.2478	10.08912	18.17241	3.759038	3.692147	5.963172	13.62901	4.627468	11.55493	0	8.143577	8.863491
LOC107802225	0.903099	0.411532	0.554149	0.33689	0.446568	0.5997	0.200506	0.13617	0.473809	0.284912	0.260752	0.157081	0.325258	0.492618	0.054524	0.316701	0.615861	0.772313	0.134765	0.247163	0.266472	0.155277	0.30458	0.222099	0.067164	0	0.094182	0	0	0
LOC107763247	0.778468	1.368278	1.111731	0.654877	0.68884	0.836948	0.326467	0.427593	0.417634	0.216887	0.389901	0.187906	0.433301	0.545959	0.285356	0.828736	0.590908	1.036394	0.225696	0.235189	0.101425	0.603682	0.68666	0.464945	0	0	0.131441	0	0	0
NitaMp005	0.066136	0.060275	0.086236	0.014979	0.058522	0.106657	0.027736	0.014127	0.124183	0.080614	0.069562	0.08381	0.073624	0.144302	0.06788	0.061606	0.271545	0.147922	0.083888	0.111893	0.165872	0.096656	0.07292	0.138251	0.397176	0.253065	0.254044	1.182416	2.388019	2.044646
LOC107776516	0.0675	0	0.14669	0	0	0	0.028307	0.028837	0.063372	0	0.023665	0.085538	0	0.110458	0	0.150903	0.130421	0.12581	0.034247	0	0	0	0.02977	0	0	0	0	0	0	0
LOC107814893	0	0	0	0	0	0	0	0	0	0	0	0	0	0	0	0	0	0	0	0	0	0	0	0	0	0	0	0	0	0
LOC107824880	31.42786	33.35671	29.82386	54.27484	58.51569	57.65212	50.46822	50.23714	51.81966	62.95226	55.13899	63.28019	70.99228	64.21528	54.43317	25.17658	29.22338	28.6051	42.18889	48.85142	49.68207	38.76786	41.00464	39.62337	45.69452	33.25269	62.37298	42.1413	60.20674	44.53239
LOC107830717	5.33531	5.815259	6.079372	4.800861	4.370145	4.767173	5.049441	5.020657	4.873645	5.167448	3.816956	4.294229	4.775551	4.129452	4.255012	5.104169	6.686734	5.294641	3.548302	3.537455	3.452153	4.064281	4.388116	4.634494	13.35417	2.708645	10.31101	0	25.51252	11.14494
LOC107826454	0	0	0	0	0	0	0	0	0	0	0	0	0	0	0	0	0	0	0	0	0	0	0	0	0	0	0	0	0	0
LOC107767316	0.116503	0.021236	0.101274	0.295528	0.432976	0.175357	0.117259	0.179178	0.218756	0.4317	0.457475	0.531495	0.830034	1.118472	0.334811	0.399364	0.292634	0.138973	0.61474	0.394214	0.361259	0.330802	0.287737	0.316599	0.61864	0.421474	0.110158	1.666323	1.682661	0
LOC107802135	10.37618	10.50229	7.155125	3.299142	1.853962	2.413485	1.673644	1.704941	0.889859	0.681025	0.734574	0.295013	1.832596	1.197298	0.307207	7.39777	7.373617	6.17383	1.569231	1.476989	1.683367	2.166355	2.68414	0.886385	0.252282	0.208236	1.120252	0	0.240166	3.084495
LOC107761226	16.0978	11.51122	15.82339	14.58379	12.60081	19.8366	10.69767	11.53253	9.649275	10.02218	12.50339	15.90138	8.960147	9.186229	5.465082	12.54976	15.91072	16.52527	9.298347	13.82713	11.40637	8.479868	13.43474	11.64836	7.358216	8.098068	7.610521	26.56662	3.576948	0
LOC107823903	1.431681	0.989849	1.029955	1.789007	2.096847	1.114616	1.946144	1.30763	1.853965	1.347929	2.042408	2.168801	2.418129	2.585202	2.077481	1.692308	0.906185	1.729889	0.801529	1.002292	1.260692	1.690379	1.916028	2.063989	1.872496	0.961693	1.050288	3.530531	6.17959	12.21006
LOC107781394	0.47742	0	0	0.308941	1.086306	0.219979	0.171614	0.349646	0.192095	0.465544	0.334767	0.05762	0.151849	0.223218	0.350007	0.203299	0	0.305089	0.968905	1.153898	0.435415	0.455664	0.541435	0.498999	0.086229	0.379597	0.08061	0	0.656705	0
LOC107784238	3.453076	4.166521	3.635845	3.260494	3.87318	3.032729	3.916048	3.573726	4.292093	2.513533	3.03515	3.245959	5.359961	4.669127	2.845298	2.826936	3.007064	2.610674	1.924701	2.468506	3.016196	2.193278	3.217438	3.609154	1.537232	1.285766	1.322098	0	3.74633	0
Novel03717	2.688004	1.853891	1.199889	5.133656	6.171303	4.530166	3.960687	3.600239	4.910822	7.934273	6.163905	11.29313	7.60212	7.450086	4.399318	1.515863	2.386284	2.220672	5.60264	5.100836	8.017085	4.671658	4.870157	4.176286	4.041406	4.953187	3.94979	0	5.596112	0
LOC107792480	0	0	0.050761	0.105805	0.516712	0.125562	0.244889	0.449044	0.219293	1.480489	0.327569	0.098666	0.520044	0.318527	0.95895	1.697107	0	0.087071	3.792293	2.371086	7.296162	0.780267	1.030154	1.037574	2.583989	1.787534	1.173297	12.52807	7.309415	10.83184
LOC107829591	49.79385	44.22999	56.3538	33.17925	31.92817	41.37931	25.37127	28.05518	40.93331	12.90116	17.79509	18.89977	10.77829	14.82719	12.03608	33.52127	47.00119	40.75507	16.9645	12.36194	27.75228	25.41253	42.06488	57.13583	6.918919	2.677662	10.59058	0	3.47428	14.87357
LOC107762418	2.391616	2.670855	2.108263	2.502366	1.788388	2.462634	1.723423	1.640526	1.454737	1.018253	1.9132	1.735926	2.024909	2.131407	1.624935	3.087534	2.147791	2.410886	0.888704	1.624213	1.47461	2.025432	1.842154	2.288473	1.362808	0.421829	1.114754	4.81787	1.783873	4.165556
LOC107763383	2.882771	2.781837	2.030951	1.143661	0.616876	0.992596	1.690953	1.271806	1.291323	0.716546	1.466506	0.939159	1.363361	1.233318	1.353698	3.383836	3.058038	3.497763	0.47798	0.573794	0.635804	1.573515	1.612099	1.358836	0.166751	0.262168	0.378578	1.347444	0.453552	1.165007
LOC107769431	12.40175	10.75132	7.471907	4.338859	3.50183	3.766957	7.441999	5.642795	6.389622	7.472268	15.0768	15.03456	26.48919	18.86459	14.48809	9.673836	14.41633	10.26084	2.302032	2.238939	2.137981	5.746887	5.047136	4.979423	2.358312	1.823819	3.783638	3.605287	11.28598	1.558575
LOC107783656	0.454757	0.414455	0.431248	0.037453	0	0.177789	0.034675	0.070647	0.038813	0.040313	0	0.10478	0.092044	0.045102	0	0.061616	0.119818	0.154109	0	0.069944	0	0.069051	0	0.08642	0	0.057524	0.048862	0	0	0
LOC107779945	11.30912	12.49722	11.78068	12.02363	10.00705	11.6419	10.8517	9.080992	10.10354	7.890031	9.896064	6.418384	7.654751	7.472532	8.852843	10.86529	9.803332	12.20416	9.213084	9.035875	5.357976	12.28793	11.17667	10.77362	5.53695	6.539557	4.118828	0	6.492393	6.604577
LOC107782298	4.66778	4.661685	4.373464	3.545079	2.868992	3.636111	3.446198	3.033002	3.490109	3.679498	3.606235	2.196064	3.516017	3.262743	2.467161	4.144962	4.482465	5.084592	2.552627	3.026488	1.784411	3.594734	3.821438	4.265265	1.060148	0.661155	2.31249	0	0.672825	0
LOC107767497	1.649756	4.510648	1.936032	11.01019	7.785669	2.837904	40.17405	34.27669	6.582671	9.974068	34.55006	5.644728	6.611514	6.20932	36.37697	9.548342	2.842283	4.550864	9.98855	14.95597	9.328554	23.76627	17.70486	7.242164	14.32245	20.39183	3.769766	25.56253	5.55976	66.30449
LOC107762749	1.324207	0.402284	0.639503	0.266592	0.781164	0.790936	0.493631	0.377146	0.828816	0.430423	0.515853	0.745818	0	0.321032	0	0.657867	0.284288	0.21939	0.597206	0.746792	0.268378	0	1.297821	0.461354	0	0.204726	0.869504	0	0	0
Novel00819	0.148338	0.165234	0.085965	0.23891	0.350024	0.194922	0.359428	0.225323	0.371376	0.353584	0.219586	0.181018	0.403656	0.557412	0.3045	0.036847	0.047769	0	0	0.069713	0.015032	0.027529	0.014538	0.051681	0.208382	0.045867	0.058441	0	0.23805	0
LOC107786143	1.343071	1.186951	1.132124	1.548596	2.268835	1.969044	1.672669	1.4953	1.222724	2.024026	2.482827	3.266456	1.85758	1.642827	2.088623	1.395135	0.825694	0.879945	3.345191	3.236293	3.414882	1.767423	1.687268	1.829164	2.830083	2.774863	3.655834	0	2.74316	0
LOC107771991	0	0	0	0	0	0	0	0	0	0	0	0	0	0	0	0	0	0	0	0	0	0	0	0	0	0	0	0	0	0
LOC107819191	1.91085	2.663476	2.149608	1.578386	2.063826	1.903338	2.569053	1.656692	1.609314	1.39749	1.793087	1.756781	1.814363	1.778078	1.874718	2.491943	2.09037	2.19978	1.76791	1.259878	1.38393	1.666203	2.206017	2.055961	2.238256	1.251204	1.328512	2.0096	1.488156	3.475021
LOC107779036	0	0	0	0	0	0	0	0	0	0	0	0	0	0	0	0	0	0	0	0	0	0	0	0	0	0	0	0	0	0
LOC107784486	7.142695	6.129954	6.571184	18.81959	26.4927	20.09409	15.42653	17.34244	14.80881	21.12726	12.72984	26.80256	22.66134	27.20822	11.91298	7.00828	7.705496	8.653421	27.72327	23.96751	38.05412	11.43301	11.81312	18.53943	35.59537	27.74509	50.01939	46.82367	36.96654	51.52508
LOC107822180	0	0	0	0	0	0	0	0	0	0	0	0	0	0	0	0	0	0	0	0	0	0	0	0	0	0	0	0	0	0
Novel02397	4.252059	3.108699	3.371282	3.174849	4.589425	2.863457	3.135284	3.513303	3.377863	2.323748	2.064148	2.368522	2.392734	2.293906	2.39791	2.576696	3.159882	2.125006	3.603482	2.924992	3.494416	3.472962	4.574904	5.17682	0.827061	0.845204	1.325431	3.341564	1.349731	0
LOC107767580	0.107777	0.049113	0.046844	0.04882	0	0.173811	0.045199	0.046044	0.050593	0	0	0	0	0	0.05531	0.040158	0	0	0.109365	0	0	0	0.047533	0.112649	0.068132	0	0.063692	0	0	0
LOC107813110	0	0	0	0	0	0	0	0	0	0	0	0	0	0	0	0	0	0	0	0	0	0	0	0	0	0	0	0	0	0
LOC107805604	0	0	0	0	0	0	0	0	0	0	0	0	0	0	0	0	0	0	0	0	0	0	0	0	0	0	0	0	0	0
LOC107822658	6.406483	5.920954	8.235874	5.027364	3.752637	4.705014	5.638278	3.970485	5.84526	4.883319	5.283115	6.74641	7.131786	7.973546	6.066073	5.211185	5.66617	6.189014	1.556543	1.488439	3.16829	3.051887	3.740764	3.536648	0.342243	0.188327	1.013148	0	1.520434	0
LOC107798859	0	0	0	0	0	0	0	0	0	0	0	0	0	0	0	0	0	0	0	0	0	0	0	0	0	0	0	0	0	0
LOC107799821	6.313438	6.706293	7.3049	6.705802	5.393905	8.472944	5.879667	6.436044	7.644216	5.264783	5.434507	6.878721	9.112434	7.885172	2.726043	5.191483	6.814403	6.232632	4.462417	5.340734	4.804919	4.3271	6.029741	6.416767	1.376225	0.727009	2.727481	3.113793	6.079027	0
LOC107809637	0	0	0	0	0	0	0	0	0	0	0	0	0	0	0	0	0	0	0	0	0	0	0	0	0	0	0	0	0	0
Novel00667	4.24218	2.848797	2.988927	2.265465	1.81761	2.544481	1.348332	1.678778	2.850807	0.870876	1.596901	1.923995	1.491301	1.412761	0.733315	2.662129	3.235502	3.129434	0.951559	0.868817	1.547576	1.790024	2.048189	3.267103	0.225828	0	0.369446	0	1.074914	0
LOC107782633	16.05357	14.94524	13.63211	14.37545	9.931941	15.14847	7.477985	6.937657	9.690796	8.124691	9.842702	9.056615	7.768743	9.726595	5.82821	11.74563	14.53498	11.47405	8.993189	8.305258	11.0595	5.717263	8.80046	11.14915	2.952232	3.765971	6.272408	1.897616	4.215688	0
Novel02089	4.566441	6.520813	4.372829	6.548349	7.714039	4.620689	12.3914	11.12086	8.024126	12.14415	12.20866	8.829745	5.763028	8.47166	11.42894	14.95828	7.407656	9.594431	13.50466	12.37366	13.20668	11.15516	12.66528	7.248595	9.848689	8.732323	8.398798	8.731687	9.287557	7.549463
LOC107776170	0	0	0	0.093689	0	0	0.043369	0	0.097091	0.100843	0.072515	0.043684	0	0.112821	0	0.115598	0.049954	0	0.157408	0.131223	0.613056	0	0.228047	0.162134	0.261496	0.143895	0.061114	0	0.248939	0
107763445	0	0	0	0	0	0	0	0	0	0	0	0	0	0	0	0	0	0	0	0	0	0	0	0	0	0	0	0	0	0
LOC107815379	0.684296	1.077216	0.432614	0.84537	0.715603	1.070112	0.782657	1.009904	1.284895	0.545952	1.221383	0.420445	0.554013	0.135733	0.766192	1.251667	0.961582	1.344998	0.631251	1.210359	0.397148	1.039043	1.536422	0.520164	0.629206	0.519354	0.735257	0	0	0
LOC107786591	0	0	0	0	0	0	0	0	0	0	0	0	0	0	0	0	0	0	0	0	0	0	0	0	0	0	0	0	0	0
LOC107824088	12.09082	13.92507	12.88591	15.30513	12.89713	14.2456	19.10137	17.22967	13.17851	11.30555	13.55914	11.26411	8.481437	10.43494	15.59711	12.92887	12.36079	11.66909	15.79835	15.65725	13.93485	13.97032	16.43564	13.15667	9.527871	14.57652	9.641112	11.84472	8.57195	12.80126
LOC107801959	2.494274	2.211785	2.13892	1.801642	1.84919	2.174311	1.300467	1.699181	1.740478	1.676268	1.654447	1.395329	1.388331	1.691514	0.968669	2.46157	2.344552	2.915029	1.539132	1.853365	0.891484	2.054885	2.55689	2.043336	0.511382	0.7035	0.637412	0	1.298197	0
LOC107807909	0	0	0	0	0	0	0	0	0	0	0	0	0	0	0	0	0	0	0	0	0	0	0	0	0	0	0	0	0	0
LOC107767319	0	0.072826	0.034731	0.036196	0	0	0.067022	0	0.075021	0.03896	0.028016	0	0	0	0	0.029774	0	0.148937	0	0	0.036439	0.066733	0.176211	0	0.050514	0	0	0	0.192353	0
LOC107798706	403.3127	315.1943	332.5644	209.9181	210.9238	177.3767	125.317	113.4069	210.284	329.0336	252.1645	365.4255	610.7843	463.0772	235.5045	245.7483	426.0062	264.3168	31.29291	17.83392	64.17186	49.39939	66.85682	68.14421	15.2378	1.810087	26.59043	35.57094	76.62873	4.731511
LOC107829352	6.683471	5.453713	6.012467	4.365151	3.610275	5.013173	3.943555	3.618888	4.122356	3.713304	3.841802	3.151469	3.720067	3.560886	2.03399	4.430356	4.279504	4.953841	3.193813	3.681516	1.949123	3.050377	3.427479	4.061373	3.881077	1.730151	2.480021	0	2.619026	2.402611
LOC107763807	0	0	0	0	0	0	0	0	0	0	0	0	0	0	0	0	0	0	0	0	0	0	0	0	0	0	0	0	0	0
LOC107826676	0.750068	1.196291	1.195367	1.585555	1.327419	1.61283	2.306753	1.441977	1.584443	1.767577	2.67357	1.478583	1.948302	1.09105	1.668007	0.885007	0.905786	1.071815	1.07825	1.321888	1.083118	0.991802	1.323219	1.829266	0.395131	1.826414	0.960397	0	1.203698	0
LOC107832844	0	0	0	0.310559	2.365986	0.147421	0.115008	0.175738	0.386202	0.467982	0.096148	1.621797	0.915862	2.767433	0.281472	0	0	0	4.174191	1.739909	4.126833	0.858842	0.181423	0.429953	3.813944	1.430941	1.701678	0	13.53294	12.71749
LOC107814980	23.00563	22.72152	22.87359	7.513829	7.033175	8.226928	8.736944	11.85303	15.99045	4.717752	7.615757	4.879802	5.495748	6.247573	7.296518	17.73245	25.51625	20.86909	3.406496	3.508023	4.502472	11.50282	14.5444	17.69871	2.995994	1.786002	3.150865	3.530531	7.367973	0
LOC107828537	5.718564	6.082559	6.148701	6.731072	6.347285	6.270812	5.801139	5.48315	5.127844	4.185718	5.699802	4.060137	5.191215	4.807356	5.869377	6.248754	6.06192	6.549312	4.326779	4.560048	3.537646	6.836071	6.276887	5.172153	1.766933	2.897049	1.938333	0	2.402973	3.527056
LOC107820025	0.316303	0.057654	0.439929	0.114622	0.055977	0.408077	0.053059	0.270258	0.237567	0	0.044358	0.160333	0.28169	0	0	0	0	0.047163	0	0	0	0	0	0	0	0	0	0	0	0
LOC107817451	18.69713	23.82362	21.32562	20.30121	17.2821	19.84563	18.24583	17.48469	18.6904	12.116	15.64346	11.58698	14.45042	16.02363	17.21416	22.49712	21.49536	23.70604	16.31398	18.7548	14.51782	17.81905	18.61052	19.06509	8.933582	13.69829	10.67391	11.35549	9.74681	26.99956
LOC107808226	1.897401	2.358064	1.774323	2.109619	3.052627	2.936274	3.158751	2.45635	1.997286	2.607108	4.031693	1.627264	3.680371	3.512678	2.773627	0.835513	0.749884	1.071661	0.320892	0.34047	0.183534	0.192069	0.431087	0.270431	0.218081	0.160006	0.339784	0	0	0
LOC107793295	32.69985	16.29534	17.23772	24.21592	14.09585	25.41578	4.202084	4.56262	8.872036	7.576697	5.826951	8.591859	2.938871	7.65844	2.801984	26.22351	34.34413	42.45099	18.08242	15.65812	24.67906	4.609875	7.171165	10.12794	0.644792	0.208713	2.907509	0	1.15544	3.709873
LOC107760208	1.190825	1.205878	1.293949	1.378501	0.93664	1.262501	1.553683	1.342499	1.45961	0.419324	1.055356	0.489048	0.570763	0.667609	0.695992	1.836434	1.374133	1.418031	1.241933	1.45507	0.55811	2.113289	1.823588	1.486665	0.271838	0.207118	0.254125	0	0.079626	0
LOC107829814	19.7901	45.98791	32.00977	25.37687	29.83938	15.98405	42.15767	36.21597	26.20586	25.73046	29.37607	16.96877	21.86621	21.53632	40.62403	39.65711	24.35044	28.26083	42.76087	42.68554	46.06511	37.45349	33.08857	19.55261	56.70114	57.40191	34.09631	70.41153	45.97924	219.1615
Novel00507	0.620613	0.565612	1.510561	0.730917	0.494244	0.733959	0.104107	0	0.407861	0.121035	0.304622	0.104862	0.27635	0.067706	0	0.647474	1.438955	0.832848	0.251902	0.367497	0.226404	0.103658	0.437938	0.648665	0.078464	0	0.220054	0	0	0
LOC107806035	8.196503	8.770964	9.117803	6.308835	5.741006	6.952139	5.296649	5.857653	6.25365	4.744962	4.4584	3.909951	4.911319	4.388413	5.060918	6.849389	7.312609	7.465222	4.915733	5.817703	3.76726	5.255747	6.199746	6.532607	3.117064	3.280019	4.345365	10.82628	3.435908	8.023252
LOC107812085	0	0	0	0	0	0	0	0	0	0	0	0	0	0	0	0	0	0	0.146827	0	0	0	0	0	0	0	0	0	0	0
LOC107792918	7.249685	7.00763	8.211694	5.885474	5.776276	6.174704	5.343578	5.38987	6.541143	4.00903	4.335265	4.268755	5.240553	5.033032	4.090873	7.834757	7.852752	7.744753	4.426626	3.10619	3.291606	5.844725	7.778837	9.05348	1.66651	1.091711	2.077223	0	3.928427	3.88103
LOC107804155	2.874061	3.929028	3.685081	2.929223	2.246456	1.750981	3.153867	3.929081	2.585866	1.51806	2.854999	0.768894	0.853185	1.097411	2.138647	4.265659	3.424454	3.160533	1.871356	2.066569	0.764514	3.480295	3.359023	2.152844	1.27179	0.766484	0.82092	0	0.345919	1.480896
LOC107830655	13.77085	15.17566	15.96826	10.52994	9.390547	10.73048	9.769989	8.894806	10.81739	6.135139	6.963043	5.080534	5.906934	5.678539	6.846572	14.23507	15.40311	16.33261	7.40991	7.60938	6.014611	10.69853	12.5034	15.21199	3.801567	2.672011	5.524893	1.806992	5.83907	10.93634
LOC107808191	3.20375	3.713562	2.352338	5.861229	6.798229	6.520996	7.278713	6.83014	6.10325	6.248122	5.125445	5.991347	8.794958	8.992335	7.629997	3.175525	3.004944	3.455255	8.742831	8.235705	7.659282	6.494018	5.157989	5.721809	7.7471	5.323374	5.110038	15.57082	7.187881	21.15553
LOC107759727	0	0	0.082636	0.043061	0.042059	0	0.039867	0.081224	0	0	0	0	0	0	0	0.070841	0	0	0	0.080417	0	0.07939	0.041926	0	0	0	0	0	0	0
LOC107780025	0	0	0	0	0	0	0.175733	0	0	0	0	0	0	0	0	0	0	0	0	0	0	0	0	0.218989	0	0	0	0	0	0
LOC107800641	1.481083	1.909508	2.041117	1.489055	1.326542	2.252586	1.181651	0.817931	1.271804	0.810185	1.380493	0.869778	1.045556	0.926123	0.574688	1.332522	1.762389	1.992965	0.953059	1.084823	0.642441	1.221807	1.513527	1.793446	0.205523	0.226187	0.384261	0	0.347827	0
LOC107822014	0	0	0	0	0	0	0.149943	0	0	0	0	0	0	0	0	0	0	0	0.151228	0	0	0	0	0	0	0	0	0	0	0
LOC107817456	2.464361	2.818462	2.56226	2.53902	2.779233	3.117047	2.654633	2.724917	2.3817	2.191029	3.371317	2.286075	3.604024	3.663736	2.97571	2.646641	2.730895	2.882017	2.941957	2.391251	2.313643	2.380884	2.57864	2.222216	1.771674	1.949812	1.856118	3.455621	3.140554	1.493874
LOC107788202	6.529333	3.636528	4.855971	2.826173	2.054252	3.041927	0.973588	1.115768	1.362228	0.707436	0.712191	0.980651	1.615232	1.741222	0.595691	2.9194	3.784731	3.353439	1.325103	0.85919	1.058642	0.605869	0.639923	1.516546	0.275169	0	0.514476	0	0.349273	0
LOC107785251	0.48332	1.266398	0.577692	1.423055	4.597485	0.519628	7.600893	5.574979	3.403213	2.297576	1.567431	2.398891	14.59428	17.26835	18.47846	1.845873	0.875489	1.441341	3.187856	2.350917	3.195768	6.559033	1.651989	0.88404	2.062337	2.774079	0.785462	17.28209	27.92247	26.14883
Novel01405	0.292476	0.145394	0.184904	1.71025	1.741031	1.800925	1.494174	1.499396	2.021975	2.126043	1.845758	3.391895	4.469438	4.351047	0.982437	0.673674	0.539426	0.59469	0.647529	0.652274	0.848727	0.599533	1.289915	2.69568	0.840405	0.073993	1.288454	1.901469	1.152068	0
LOC107783338	0.378535	0.517482	0.411315	0.557267	0.711774	0.254357	0.912794	0.929863	0.533078	0.553679	0.862644	0.679568	0.210695	0.05162	0	0.740472	1.508498	0.846641	0.720208	1.080724	0.863075	0.632249	2.045093	1.038567	0.179469	0.197514	0.279624	0	0.2278	0
Novel04866	0	0	0	0	0	0	0	0	0	0	0	0	0	0	0	0	0	0	0	0	0	0	0	0	0	0	0	0	0	0
LOC107812646	0	0	0.096878	0	0	0	0	0	0	0	0	0	0.062032	0	0	0.207625	0	0	0	0	0	0	0	0	0	0	0	3.984993	0	0
LOC107830015	6.051845	3.930594	6.470106	4.537378	4.37023	6.506473	2.80052	4.101027	3.722518	3.798546	2.682709	3.290976	4.491332	4.325622	2.070483	3.265748	6.249804	4.823073	2.682273	2.5303	3.933354	2.614166	3.804183	7.052443	1.495096	1.742214	3.535289	0	4.688509	0
LOC107805182	1.44374	1.77229	1.607184	2.65595	2.639765	2.130311	2.199438	2.032874	1.300122	2.966496	3.491579	0.896115	2.287787	1.968811	3.122396	2.409744	1.366309	2.02643	3.08698	3.638989	0.947229	2.965049	1.708794	1.124048	3.855535	4.478934	1.419085	0	3.262563	1.821808
LOC107773788	1.766602	2.133303	1.574062	1.200339	2.071248	0.902174	2.000329	1.886791	1.119531	1.765727	2.849108	0.783549	0.934138	1.638185	1.722533	1.119003	0.469339	0.724392	1.434101	1.083457	0.604189	0.811435	0.662261	0.784742	3.18277	1.843574	2.29678	0	8.292427	2.730792
LOC107804929	1.571393	3.007475	2.140033	2.609938	2.039359	2.872047	5.887044	4.206941	3.737408	0.459692	2.791383	0.663778	1.516057	2.000031	4.032086	3.356878	1.821717	1.952567	1.647692	1.728077	0.621026	15.13532	4.20442	2.025651	0.198672	0.218648	0.309543	0	0.252175	0
LOC107798904	0	0	0	0	0	0	0	0	0	0	0	0	0	0	0	0	0	0	0	0	0	0	0	0	0	0	0	0	0	0
LOC107791452	0	0	0	0	0	0	0	0	0	0	0	0	0	0	0	0	0	0	0	0	0	0	0	0	0	0	0	0	0	0
LOC107792438	21.94643	24.33729	18.18395	20.42381	18.7427	20.33085	18.04648	14.6663	14.58908	21.68028	26.63712	17.95523	29.59406	25.11612	19.28629	26.63397	20.25516	28.85628	18.31551	18.96964	11.86104	14.81416	16.27944	17.5911	18.6491	11.94197	20.40076	6.838692	31.65134	14.78192
LOC107768435	94.92694	97.07955	88.79651	108.5985	86.33385	115.2721	77.60847	81.98976	89.49198	95.9985	94.14129	93.83654	49.32354	55.68701	75.78267	87.29383	97.16059	88.05782	97.77264	112.5199	108.4955	105.583	110.2862	107.9721	30.27271	55.44315	55.52411	47.74604	21.91554	30.02289
LOC107815999	81.76748	61.12933	59.43167	768.7159	653.1134	572.8514	167.1769	180.7135	267.012	155.8358	138.2862	196.808	82.73334	74.05723	90.19742	63.25592	77.82108	72.82138	398.3477	337.3219	676.4391	152.3931	254.149	427.9937	77.3295	131.797	137.8353	83.9497	49.49954	42.54887
LOC107802913	0	0	0	0	0	0	0	0	0	0	0	0	0	0	0	0	0	0	0	0	0	0	0	0	0	0	0	0	0	0
LOC107822892	12.27062	14.5102	14.94551	11.97004	11.31634	11.57185	8.691813	8.089652	11.5202	4.271718	6.011355	4.417229	3.644368	4.41939	4.544558	13.63714	11.80894	13.22197	7.408688	6.813808	8.355668	11.03427	13.37876	16.83775	1.339963	0.655419	2.199094	0	1.247268	0
LOC107783436	0	0	0	0	0	0	0	0	0	0.028329	0.040742	0	0	0	0	0.043298	0	0.086636	0	0	0	0	0	0	0	0	0	0	0	0
LOC107789574	0	0	0	0	0	0	0.043156	0	0.096614	0	0	0	0.114558	5.894001	3.485497	0	0	0	0	0	0	0.04297	0	0	0.065053	0	0	0	0	0
LOC107829548	1.154224	1.2523	1.433346	2.888035	3.063999	1.654575	0.645398	0.657467	0.567619	1.875856	2.042626	1.95024	3.120466	2.338516	3.384752	3.194764	1.380571	1.516159	1.840498	2.138767	2.556487	0.826219	0.96962	1.148947	1.250819	2.141357	0.974428	3.930633	6.615289	3.398447
107764935	0	0	0	0	0	0	0	0	0	0	0	0	0	0	0	0	0	0	0	0	0	0	0	0	0	0	0	0	0	0
LOC107808163	0.195083	0.266691	0.508744	0	0	0	0	0	0	0.095115	0	0	0.054292	0	0	0.327096	0.376933	0.036361	0	0	0	0	0	0.050975	0	0.067861	0	0	0	0
LOC107783073	1.838407	1.770065	1.237229	1.155107	1.010155	1.65772	1.193768	0.975406	0.890827	1.272223	1.403447	0.839195	1.600922	1.47186	0.699975	1.160066	1.861993	1.713263	1.038054	0.840291	1.108758	0.755269	1.137733	1.782038	0.46861	0.350695	0.508166	1.060259	1.142031	0
LOC107819953	0	0.271237	0.086236	0.718991	0.263347	0.426626	0.832067	1.186677	0.279412	0.29021	0.904306	0.251431	0.441742	0	1.018204	0.295708	0.191679	0.147922	0.905989	0.839198	1.08571	0.662783	0.787541	0.103688	0.627121	1.242317	0.820759	0	0	0
LOC107819160	0.122957	0.224121	0.053442	0.612661	0.108801	0.198291	0.515647	0.630348	0.634907	0.299747	0.431088	0.155816	0.615949	1.140192	1.198899	0.091628	0.059393	0	0.187153	0.260033	0	0.975505	0.433825	0.128515	0.155455	0	0.217988	0	0	0
LOC107809393	8.241161	10.37079	8.107151	9.937052	9.368427	9.52241	13.25866	11.22626	9.499366	7.264013	12.35944	9.266631	7.672353	6.047126	10.5054	8.807662	8.868319	7.674703	8.83872	11.13944	9.923362	11.16861	12.57244	9.257384	1.594417	2.407656	4.29825	2.097365	2.11793	0
LOC107829162	0	0	0	0	0	0	0	0	0	0	0	0	0	0	0	0	0	0	0	0	0	0	0	0	0	0	0	0	0	0
LOC107763278	4.041903	4.961713	4.230589	2.839732	4.087476	5.055016	2.559885	2.819193	2.555625	3.297877	3.817473	3.902528	3.397584	3.779586	2.539897	5.224951	3.506367	4.181875	4.101444	4.465857	2.78352	3.03106	2.328311	3.362433	3.441559	1.606861	2.827325	0	2.77988	0
LOC107795086	0	0	0	0	0	0	0	0	0	0	0	0	0	0	0	0	0	0	0	0	0	0	0	0	0	0	0	0	0	0
LOC107808813	7.580438	6.830791	5.772786	11.68441	14.2845	12.41999	8.919166	9.213664	8.540219	19.11479	16.17082	17.64302	17.66174	17.4017	11.17741	11.87075	11.24288	14.23233	21.19121	20.35752	13.8854	11.48428	11.43288	11.82881	23.08254	17.1139	20.99797	12.21653	22.10256	3.960929
LOC107812463	2.494274	3.197925	2.719462	2.259686	2.281915	2.13623	2.304807	1.986689	1.746378	2.514681	2.845809	2.321527	3.67084	3.643548	3.210916	2.583328	2.981467	2.868177	1.887538	2.860996	0.925351	1.694671	1.901794	1.900028	1.817283	1.470594	1.948699	0	2.035309	0
LOC107814168	0.609976	0.484647	0.435068	0.297558	0.304473	0.454015	0.655915	0.534544	0.293678	0.122011	0.274177	0.118921	0.1567	0.136503	0.240793	0.617729	0.302199	0.548048	0.238062	0.330768	0.114115	0.626963	0.441468	0.425031	0.039548	0.065288	0.036971	0	0	0
LOC107767814	0.544677	0.523984	0.552389	0.932072	1.472674	1.203722	1.522813	1.370305	0.909096	1.2393	1.65502	0.894752	1.515855	1.254455	1.73924	0.63139	0.818539	0.7896	0.368467	0.639943	0.082792	0.151625	0.45375	0.158138	0.038258	0	0.071529	0	0.291363	0
LOC107790467	0	0	0	0	0	0	0	0	0	0	0	0	0	0	0	0	0	0	0	0	0	0	0	0	0	0	0	0	0	0
LOC107779020	175.2459	162.6618	196.3711	94.4772	87.96784	94.22854	94.70037	99.52854	132.5942	45.02333	61.4866	44.25188	42.6166	42.84605	43.14166	139.2997	170.0103	163.2442	47.02693	45.84137	51.97824	114.8137	150.8899	202.7228	18.42279	6.298498	29.09452	18.49824	5.603885	10.66245
LOC107789061	0.454757	0.263744	0.215624	0	0	0.044447	0	0	0	0	0	0	0	0	0	0.092423	0	0.030822	0	0	0	0	0	0	0	0	0	0	0	0
LOC107769726	3.577743	3.297308	3.4595	2.585719	4.268524	3.284661	3.472844	2.953878	2.264456	1.607177	2.70603	1.596192	2.192774	1.622654	2.186754	1.168308	3.145713	2.697329	2.651449	2.958512	2.896337	3.558582	2.907596	2.436949	2.287089	2.013641	0.760198	0	1.354738	4.971174
LOC107784768	41.69929	32.27386	30.25631	50.71304	37.93719	29.80645	73.79373	61.38803	106.5667	11.07958	17.02322	17.72635	85.84159	61.15166	26.55016	19.89775	36.26018	21.48812	25.55724	17.58931	38.34133	78.88417	101.8257	81.54697	5.793996	2.634946	5.998381	10.83411	15.86349	14.05084
LOC107782789	0	0	0	0	0	0	0	0	0	0	0	0	0	0	0	0	0	0	0	0	0	0	0	0	0	0	0	0	0	0
LOC107784024	4.141656	5.032811	5.082711	6.179979	5.964298	4.627405	5.244734	7.424421	7.166797	4.909716	5.352713	5.419997	3.977728	3.720988	5.501141	4.599291	4.864189	4.238129	6.097986	7.968872	5.925113	9.291299	9.312114	7.808919	1.334746	2.259926	2.399561	5.807587	2.345812	0
LOC107812921	41.10091	33.21893	29.24723	37.81263	35.46662	30.2126	32.44213	30.43537	31.76725	26.28409	34.2712	15.82731	34.97174	28.22428	28.97347	0	0.061561	0.095015	0.064661	0.053905	0.058116	0	0	0.066603	0	0	0.075315	0	0	0
LOC107787062	13.78415	16.67787	13.80027	9.516519	8.075335	7.563129	10.00656	10.51857	10.17443	16.96383	11.86518	8.633533	13.17528	7.36335	6.34206	13.99112	14.74015	14.91889	6.511283	6.795222	8.539865	10.55883	11.48771	13.86071	7.331507	9.325271	16.29175	10.19753	13.73003	17.63368
Novel03171	1.826236	1.437427	1.731827	0.376018	0.587627	1.07096	1.531743	2.482431	1.402814	0.971351	0.640279	0.771427	1.201313	1.177288	1.107599	1.237194	2.646446	1.423424	0.589635	0.702214	1.0599	1.386488	1.684082	0.867628	0.734655	0.808523	2.060352	0	0.399643	0
LOC107767210	0	0	0.084398	0.043979	0.042956	0	0.162866	0.082956	0.091152	0	0.102119	0.082024	0	0	0	0.036176	0	0.072384	0	0.164262	0.044274	0.040541	0.128459	0.202956	0.061375	0.067546	0	0	0	0
LOC107812786	0.029543	0	0.025681	0.214116	0.104566	0.254099	0.148674	0.126212	0.055473	0.057616	0.435027	0.823639	1.973262	2.062718	3.12318	0	0.028541	0.044051	0.089935	0.149948	0.323325	0.197377	0.104236	0.123513	0.037351	0.041107	0.069835	0	2.133459	0
LOC107818192	0	0	0	0	0	0	0	0	0	0	0	0	0	0	0	0	0	0	0	0	0	0	0	0	0	0	0	0	0	0
LOC107763948	0.147238	0.301925	0.319976	0.033347	0.032571	0.118724	0	0.031451	0.103675	0.646088	0.129054	0.559757	0.409767	0.080314	0.264461	0.274304	0.177805	0.192101	0.074703	0.186829	0.234995	0.092221	0.032468	0.076946	0.093077	0	0.348046	0	0	0
LOC107769963	0.70707	0.541302	0.540884	0.230605	0.325344	0.60815	0.094888	0.096662	0.132766	0.220634	0.257815	0.191153	0.409303	0.123421	0.290287	0.421528	0.491824	0.506065	0.315694	0.382805	0.128972	0.141718	0.149684	0.147806	0.071516	0.039353	0.200568	0	0.272327	0
LOC107808367	16.21532	15.75052	17.34134	8.118302	6.183021	7.225709	8.545045	10.11766	11.21743	3.536892	5.53549	5.542751	5.700355	5.586354	5.857885	12.40167	17.57201	15.15128	4.925432	3.519506	5.302509	8.419105	13.92659	14.60672	3.169578	2.597649	2.647825	7.629111	3.851958	32.98085
LOC107815909	0.026375	0	0	5.901809	7.001368	10.86028	4.844593	7.797141	8.963679	9.695863	4.179603	17.95922	8.426453	11.13526	4.331211	0.098272	0	0.039327	23.04296	26.48317	28.74452	6.475655	4.001417	7.856487	8.869873	11.63333	21.97664	0	12.95158	3.26155
LOC107817704	1.322721	2.832919	1.207304	2.097057	1.814168	1.066565	2.107904	2.147321	2.545749	1.999221	2.828854	1.173345	2.650449	1.731629	2.647329	6.01273	2.939078	3.599429	2.08042	1.510557	1.447613	3.424381	3.033492	2.626765	1.003393	0.920235	0.62534	0	0.636805	0
LOC107812273	3.57567	3.449456	3.885318	3.084299	2.861053	3.169488	2.855496	2.128854	3.517704	1.761911	2.627291	2.040664	2.625427	2.842663	2.205886	3.259875	2.921539	3.658404	2.026468	2.300756	1.838689	3.144972	3.573389	4.154752	0.913765	1.1115	1.326293	2.720329	2.472302	1.176006
LOC107788937	0	0	0	0	0	0	0	0	0	0	0	0	0	0	0	0	0	0	0	0	0	0	0	0	0	0	0	0	0	0
LOC107819408	68.36285	75.48157	86.40637	11.71769	8.831403	25.04581	7.492013	6.451677	8.207253	6.224922	8.651877	4.20584	5.091177	7.561996	7.407744	60.13886	58.51936	68.69057	11.69862	11.8683	6.929977	14.24306	16.80852	10.63079	0.511963	0.596583	2.646391	0	0.917416	1.472815
LOC107811805	0.885183	0.964575	0.970203	0.801931	0.612993	1.199945	1.032962	0.542581	0.722651	0.469112	0.809597	0.601513	0.514121	0.440859	0.553017	1.218899	1.134019	1.190764	0.585798	0.830196	0.789752	1.12493	1.306975	1.226887	0.389266	0.69616	0.500364	0	0.27793	0
LOC107781123	1.529674	0.612563	0.604415	1.112845	0.922879	0.921965	0.71926	0.455468	1.501404	0.474608	0.617563	0.8028	1.083635	1.036679	1.046677	0.932658	1.455403	1.123158	0.493884	0.372515	0.54958	0.696802	0.613306	0.557163	0	0.064498	0.08218	0	0.669491	0
LOC107772659	0.144829	0.307985	0.37769	0.306151	0.085436	0.207612	0.323931	0.28874	0.226619	0.235377	0.236959	0.040785	0.322451	0	0.148648	0.071951	0.373112	0.503888	0.244937	0.204192	0.088058	0.403168	0.383246	0.201833	0.183108	0	0	0	0	0

LOC107774005	0.158887	0.289612	0.184156	0.239906	0.140594	0.227764	0.266531	0.045252	0.14917	0	0.185686	0.357953	0.471667	0.635572	0.434873	0.473611	0.511661	0.631771	0.21497	0.17921	0.434723	0.309612	0.420447	0.608919	0.535684	0.073693	0.250389	0	0.25498	0
LOC107784652	12.86078	14.90572	13.71615	10.73739	8.096741	13.9431	8.76246	9.234118	9.571478	8.887518	10.07893	10.40863	8.501853	9.078546	7.173084	11.08722	12.18098	12.97235	6.653059	7.74048	7.162398	7.34076	8.960853	8.999042	7.01409	3.358415	7.153133	2.576268	6.243668	4.45491
LOC107824070	15.26025	12.2676	14.69951	12.39835	11.44626	15.23761	9.308673	10.21957	8.97637	7.824232	8.886405	8.679323	9.975706	9.735298	9.428405	0.027941	0	0	0	0	0	0	0	0	0	0	0	0	0	0
LOC107760103	49.63577	51.46112	45.10856	41.98439	38.74112	46.28422	44.80262	46.99503	44.94004	48.04438	40.06381	42.91247	33.46539	47.36478	52.38427	32.39913	48.95379	33.09589	34.21735	34.81843	35.87013	36.00326	38.08069	28.17033	31.68573	38.0958	34.66597	43.60761	17.90764	22.62202
LOC107759343	4.247008	2.131648	3.879101	2.28624	1.879016	2.415376	2.21989	1.919561	2.918225	2.460813	1.316364	4.887987	2.432433	2.350213	1.895223	3.66942	3.359676	5.277207	1.217922	1.223593	1.740197	1.028054	1.221568	2.63765	0.77819	0.385396	2.800803	2.200878	0.296328	0
LOC107760402	0.703399	0.384637	0.529923	1.104556	1.161839	0.957904	0.432645	0.681137	0.704407	3.017972	3.057987	2.892021	2.035887	2.557912	1.540158	0.803733	0.906056	0.978905	1.142017	1.190054	0.812581	0.43078	0.496355	0.147038	1.600758	1.304971	2.438658	0	2.257608	0
LOC107829901	0	0	0	0	0	0	0	0	0	0	0	0	0	0	0	0	0	0	0	0	0	0	0	0	0	0	0	0	0	0
LOC107790702	9.437795	9.948937	10.58326	17.81284	14.28052	17.0466	16.86084	16.55789	14.67909	10.9823	11.09582	10.47162	12.85269	11.90924	15.2404	9.072642	8.935349	9.311339	15.51445	14.71667	16.26804	16.07877	14.37408	12.42912	12.09134	11.64371	11.00598	4.499572	10.14758	19.45177
LOC107810671	0	0	0	0	0	0	0	0	0	0	0	0	0	0	0	0	0	0	0	0	0	0	0	0	0	0.075448	0.064088	0	0	0
LOC107760114	0	0	0	0	0	0	0	0	0	0	0	0	0	0	0	0	0	0	0	0	0	0	0	0	0	0	0	0	0	0
LOC107792297	2.499099	2.98553	1.849487	5.690738	6.574335	3.558243	5.693466	6.030774	4.058403	3.359021	3.504739	2.539276	2.142916	2.542178	2.807642	2.013331	1.076665	1.183374	5.585862	5.885102	4.46604	4.484365	4.289586	2.823846	1.195532	1.409722	0.758391	0	4.064713	0
LOC107812930	4.374093	3.4217	4.087479	1.651128	2.128767	1.449994	2.843274	2.429273	2.977278	1.919382	3.271591	2.833106	3.002736	5.686538	5.200278	4.72639	4.718745	4.674203	2.515165	3.021815	1.728673	5.327174	5.369383	5.295669	0.599103	0.253593	1.249375	2.606746	0.877435	2.253806
LOC107794085	1.412942	0.827821	1.84236	0.822892	0.803741	1.410579	0.08465	0.086233	0.663266	0	0.353841	1.023165	0.561752	0.220207	0.103586	0.75209	1.267519	0.827678	0.512056	0.597626	0.736358	0.084285	0.712176	1.898751	0.255198	0.280858	0.357854	0	0.485886	0
LOC107787264	6.008802	5.310334	6.041118	4.206473	3.786337	4.861462	4.836211	4.459911	4.615632	3.787857	4.277215	3.384269	4.560738	3.906699	3.737739	6.151275	6.801828	5.746836	2.371179	2.59286	2.823097	4.33382	4.122347	5.011631	1.649843	1.224563	2.044492	0	1.461036	0
LOC107787711	0	0	0	0	0	0	0	0	0	0	0	0	0	0	0	0	0	0	0	0	0	0	0	0	0	0	0	0	0	0
LOC107817599	0.687663	0.321003	0.306175	0.334286	0.237459	0.360645	0.084406	0.100315	0.251945	0.261681	0.199932	0.41092	0.298738	0.292763	0.154931	0.199979	0.372678	0.200071	0.187213	0.255387	0.168262	0.070035	0.10356	0.192834	0.04241	0.023337	0.218058	0	0	0
LOC107801388	4.138815	4.761402	4.128605	5.409197	6.544103	4.960358	7.853337	7.536412	5.032303	7.87325	7.469442	5.388182	7.402032	6.809876	9.053044	6.016816	5.309409	5.109048	7.022589	5.682199	5.383545	10.59595	6.583245	4.538637	8.406666	9.346346	5.132324	0	10.77949	8.390465
LOC107829372	106.0789	89.7637	102.9358	145.7668	159.77	170.4334	99.37526	103.0372	102.8741	155.0962	126.2376	187.764	209.8128	171.2873	110.0953	75.95302	94.76957	85.82441	136.761	137.7841	159.1371	87.87306	104.1009	122.8712	121.355	77.38041	185.804	245.323	208.3459	138.6857
LOC107799828	11.70523	8.320116	8.564375	6.510002	7.718178	12.05007	6.216599	6.83484	9.249751	6.874887	6.781662	5.918068	5.483846	6.11374	5.102439	0	0	0.067388	0	0	0	0.037743	0	0	0	0	0	0	0.435159	0
107806726	0	0	0	0	0	0	0	0	0	0	0	0	0	0	0	0	0	0	0	0	0	0	0	0	0	0	0	0	0	0
LOC107804035	0.068292	0	0.178094	1.113639	1.389867	0.36711	0.859188	2.042261	1.666998	4.195363	3.735124	4.904054	1.444443	2.160585	2.242969	1.221386	0.395853	0.81463	1.801741	4.506069	1.121098	1.254709	0.602378	0.927921	5.957565	8.552067	4.439344	9.767649	3.616588	0
107810454	0	0	0	0	0	0	0	0	0	0	0	0	0	0	0	0	0	0	0	0	0	0	0	0	0	0	0	0	0	0
LOC107772600	0	0	0	0	0	0	0	0	0	0	0	0	0	0	0.09919	0.144035	0	0	0	0	0	0	0	0	0	0	0	0	0	0
LOC107805344	0.744232	0.678276	0.770173	1.187941	0.90942	0.685835	0.297247	0.54505	0.465812	0.829395	0.919461	0.508986	1.223008	2.203784	2.546198	0.845109	2.19121	1.453196	0.43154	0.239836	0.711074	0.680721	0.468901	0.518581	0	0.049312	0.041887	0	0.170619	6.573856
LOC107780039	0.061915	0.479634	0.48439	0.112183	0.164358	0.23298	0.259652	0.079352	0.029064	0.030187	0.108536	0	0.034462	0	0.031774	0.299902	0.478518	0.25388	0.031413	0.104751	0.056467	0.232679	0.109226	0.09707	0	0.172299	0.073178	0	0	0
LOC107820513	21.20742	16.59176	16.76933	22.51143	19.72661	23.34998	16.12669	13.80846	16.73198	20.7422	14.37795	24.44646	20.90623	22.92719	12.91579	17.70786	15.79809	19.09706	24.95521	21.99273	28.13842	12.53631	16.17084	18.56067	15.26381	14.6654	23.40449	18.27265	33.82833	15.79863
LOC107800795	2.783466	2.825057	2.694563	3.094788	2.826845	2.890547	2.891736	2.43232	2.405369	3.577849	2.883293	3.179936	4.366199	3.554231	2.921802	2.710657	1.833451	2.358172	2.439314	2.755976	2.884736	3.328326	2.287801	3.008457	3.119244	1.672427	3.065526	15.83409	6.852577	1.955749
LOC107829786	3.575965	6.555137	4.662766	6.994684	6.616157	5.242636	9.304696	8.923162	5.91338	6.617408	7.778853	3.810674	5.066491	5.186826	10.55203	8.236696	4.789447	6.028872	6.061482	6.70317	3.335462	12.42063	6.487693	5.776287	6.524779	6.954664	2.449451	0	5.477808	0
LOC107759999	3.907115	4.228514	3.76785	3.097192	2.268835	2.100313	2.867432	2.295112	2.292607	2.143086	3.296167	1.959873	3.330051	2.597443	1.253174	3.957938	3.715624	4.187323	1.424803	1.39436	1.002194	1.2236	2.207808	3.700867	2.006786	0.339779	2.164639	0	7.053841	11.32419
LOC107790703	31.52795	38.74887	35.12827	16.54824	13.1187	23.15805	13.08201	13.75423	15.38721	12.95222	20.19202	10.81634	10.7474	8.985581	12.92665	27.90768	28.60534	29.05531	9.521542	12.71786	7.549831	16.38648	16.62698	14.36245	3.110838	3.655736	5.274066	7.455998	4.316687	2.578598
LOC107794190	0	0	0	0	0	0	0	0	0	0	0	0	0	0.191758	0	0	0.339619	0	0	0	0	0	0	0	0	0	0.207747	0	0	0
LOC107800268	0.504865	0.268405	0.548586	0.495497	1.116845	0.904653	0.670466	1.150321	2.172453	5.743585	2.566585	6.113512	2.669609	3.304699	0.863633	0.376226	0.934839	0.376398	0.554994	0.427081	0.613927	0.386491	0.816428	1.231265	2.712788	0.292701	9.398183	6.0175	3.038251	0
LOC107816049	0	0	0	0	0	0	0	0	0	0	0	0	0	0	0	0	0	0	0	0	0	0	0	0	0	0	0	0	0	0
Novel00756	0	0	0	0	0	0	0	0	0	0	0	0	0	0	0	0	0	0	0	0	0	0	0	0	0	0	0	0	0	0
Novel03856	2.937853	3.069318	3.052118	5.777501	6.657526	6.933404	5.288807	6.306064	5.852735	8.384714	8.340585	11.25971	12.52346	10.16236	6.839654	3.577622	3.184336	4.006635	7.998153	7.395048	11.04424	5.146318	7.015688	6.44086	11.95836	16.05212	11.09444	40.99473	13.45392	13.2916
LOC107770570	0.637611	0.406773	0.304844	0.288822	0.19747	0.034276	0.053479	0.136198	0.119724	0.12435	0.044709	0.080801	0.07098	0.10434	0.163607	0.190059	0.338792	0.308988	0.258802	0.134844	0.348907	0.106497	0.421811	0.166608	0.080613	0.177438	0.03768	0	0.460453	0
LOC107829760	0	0	0	0	0	0	0	0	0	0	0	0	0	0	0	0	0	0	0	0	0	0	0	0	0	0	0	0	0	0
LOC107831570	0	0	0	0	0	0	0.019143	0	0.021428	0.022256	0	0	0	0	0	0.034016	0	0	0	0	0	0	0	0	0	0	0.026975	0	0	0
LOC107797705	0.464566	0.564526	0.572102	0.210436	0.205539	0.582709	0.064942	0.066156	0.072692	0.037751	0.081438	0	0.043097	0.126705	0.158939	0.490443	0.336607	0.548393	0.353557	0.327492	0.035308	0.096993	0.136593	0.080927	0	0	0.045757	0	0	0
LOC107759542	0	0	0.079602	0	0	0	0	0	0	0	0	0.077363	0	0	0	0.06824	0	0.136543	0	0	0.083516	0	0	0.191424	0	0	0	0	0	0
Novel01000	37.55983	25.83741	16.35106	0.896886	0.822921	0.774085	2.138785	2.512004	3.661475	2.311039	2.776742	3.370848	3.974156	3.894677	2.894358	27.87785	44.54593	33.34796	1.36988	0.786712	2.298272	2.179669	3.519428	3.229652	0.606863	0.041743	1.666499	0	0.86658	0
LOC107764239	0.168695	0.025624	0	0.050943	0.149272	0.181368	0.070746	0.096092	0.105585	0	0.138004	0.190024	0.187793	0.214711	0.115429	0.062856	0.081487	0.062885	0.05706	0.047568	0.102568	0.140882	0.0496	0.146933	0	0	0.099692	2.010678	0	0
LOC107780488	0.667482	0.128069	0.305383	0	0.031086	0.03777	0.029466	0.030017	0.065964	0.034257	0.098534	0.059359	0	0.038326	0	0.523588	0.339391	0.340488	0.035648	0.118872	0.096119	0	0.092963	0.183593	0.133247	0	0.124565	0	0	0
LOC107778175	0	0	0	0.1845	0.612701	0.043791	4.236168	2.262096	0.650077	4.567528	8.396768	1.41083	6.665282	5.332231	9.447916	0.030353	0	0.030367	0.082662	0.241188	0.26003	2.789257	0.323345	0.042572	2.265827	1.813564	0.240702	2.912825	2.549201	0
LOC107827702	4.2275	3.664902	3.43586	3.892176	3.254166	4.766791	3.257511	3.582725	3.452676	2.144961	2.410021	3.368253	2.180881	3.412127	2.363515	3.790651	3.752079	3.843635	3.522498	4.012293	3.165941	3.186057	4.304949	4.023424	0.43454	0.765172	1.665522	2.457935	0.992814	0
LOC107801196	1.264934	0.995627	1.249523	0.78134	0.763157	1.607224	0.337576	0.736905	0.593788	0.1682	0.161268	0.242875	0.192019	0.815443	0.236053	0.771243	0.666563	0.985928	0.408408	0.291831	0.524382	0.480172	0.557878	0.72115	0	0	0.20387	0	0	0
LOC107774825	0.509891	0.422457	0.765593	0.209971	0.533219	0.946883	0.466547	0.316847	0.652784	0.678012	0.487549	0.665736	0.670822	0.707976	0.333034	0.4836	0.671725	0.725733	0.376293	0.784242	0.760958	0.696802	0.367984	0.775183	0.175816	0.322489	0.657437	0	0.446327	0
LOC107820359	3.431295	3.988778	4.808926	1.522569	1.580082	2.710331	0.939747	1.346231	1.18339	1.946111	2.160513	1.863548	3.19613	3.934362	2.264006	1.643789	1.589808	1.592334	0.284232	0.888563	0.510922	1.432782	1.050055	0.731916	0.044267	0	0.331062	0	0	2.164919
LOC107795988	0	0	0	0	0	0	0.01527	0.046668	0.034186	0.053261	0.076598	0.015381	0	0.079449	0.168179	0.013567	0	0.013574	0.073898	0.030803	0.066418	0.334502	0.160592	0.171264	0.046037	0.202663	0.150629	1.302014	0.087652	0
LOC107819364	0	0	0	0	0	0	0	0	0	0	0	0	0	0	0	0	0	0	0	0	0	0	0	0	0	0	0	0	0	0
LOC107776468	6.999749	5.269946	7.113553	7.444273	6.104072	9.525138	5.218658	5.547386	5.841487	4.187711	5.429876	5.913582	4.592515	4.168653	3.401287	2.116727	3.822195	3.907655	2.779381	2.51727	2.035443	1.383758	2.833589	3.251616	1.325334	0.235257	2.078277	0	0.813992	0
LOC107809001	0.589957	0.729699	0.439575	0.76353	0.298304	0.453054	0.035344	0.108016	0.079125	0.082183	0.029548	0.035601	0	0.137917	0	0.408234	0.69208	0.659757	0.256563	0.249532	0.115297	0.17596	0.14868	0.132133	0	0	0	0	0	0
LOC107827196	0.450967	0.1644	0.137205	0.306415	0.538711	0.242422	0.245859	0.308254	0.042339	0.26385	0.221353	0.114297	0.301213	0.27059	0.162001	0.016803	0.065351	0.016811	0.160163	0.019074	0	0.037661	0.039778	0.023568	0.085524	0	0.02665	0	0	0
LOC107804144	17.25408	10.75919	11.02003	8.029565	5.946308	8.904119	14.68501	14.15266	25.03155	10.6971	11.13069	16.72488	14.51682	15.9305	8.015706	10.88173	16.42325	15.08432	4.165121	3.994631	5.068653	11.37579	16.02025	20.65363	1.653302	1.11194	5.023102	10.39083	2.623178	2.245991
LOC107830941	0.798168	1.31847	0.715513	0.7231	0.640058	0.589963	0.815902	0.681976	0.327843	0.267546	0.629635	0.316085	0.194366	0.326536	0.435209	1.115242	0.771097	0.94839	0.404963	0.611896	0.204734	0.895704	0.660034	0.338912	0.031535	0.104118	0.058961	1.783759	0.120083	0
Novel04883	2.452564	5.18888	3.04566	4.285091	4.262873	3.201836	6.097747	4.939481	3.947272	4.441468	5.589153	2.959992	4.192851	4.300115	5.933505	4.960774	3.892558	4.048801	5.68844	4.59398	1.837355	7.607601	5.331048	4.577534	3.543756	2.437546	2.277576	0	4.638667	0
LOC107821425	0	0	0	0	0	0	0	0	0	0	0	0	0	0	0	0	0	0	0	0	0	0	0	0	0	0	0	0	0	0
LOC107777254	67.57602	44.74458	56.85528	17.49876	13.59441	23.33976	17.32115	17.50237	24.50982	13.24416	14.79297	16.27115	12.95087	16.33546	10.22662	41.27342	62.75664	42.45432	11.18381	10.83021	9.797924	23.38266	27.34829	20.82841	4.081816	2.865735	7.236932	7.961517	8.307566	6.88357
LOC107772305	0.03144	0.057308	0.027331	0.056967	0.027821	0	0	0.026864	0	0.061317	0	0	0.035	0	0	0.046859	0.091122	0	0	0	0	0	0	0	0	0	0	0	0.151365	1.944009
LOC107790196	15.19948	14.86475	18.19272	10.22155	9.725026	10.43329	9.806509	10.48938	12.73318	4.446425	5.615887	5.432708	5.401473	5.676109	5.040108	10.8039	13.6674	13.81615	5.872751	4.945276	7.304305	9.910684	10.93182	13.68683	1.404302	0.976106	3.454725	0	0.844335	0
LOC107785335	17.68788	18.72677	17.15918	21.88208	20.68516	18.24795	22.44893	20.63762	21.77188	23.49233	21.16533	21.87645	28.2379	24.68881	22.8126	18.90285	14.95574	19.46773	22.58539	23.95615	20.21417	17.31576	19.02936	18.87726	18.98289	20.63205	19.80342	15.56158	17.51006	23.06506
LOC107830366	0.661838	0.258507	0.43834	0.456832	0.529863	0.64379	0.370075	0.188497	0.68054	0.614644	0.574577	0.426011	0.42101	0.27506	0.549903	0.446231	0.517602	0.493428	0.543666	0.346586	0.229946	0.526398	0.500387	0.625872	0.358613	0.438524	0.70774	0	0.15173	0
LOC107829836	0	0	0	0	0	0	0	0	0	0	0	0	0	0	0	0	0	0	0	0	0	0	0	0	0	0	0	0	0	0
LOC107792261	0	0	0	0	0	0	0	0	0	0	0	0	0	0	0	0	0	0	0	0	0	0	0	0	0	0	0	0	0	0
LOC107777235	6.005807	6.789977	7.17023	4.821101	3.733487	6.906514	3.31571	3.540102	5.924011	3.29887	4.078008	3.885687	4.146857	3.483363	2.848015	4.985406	5.765393	6.319628	2.50714	3.569212	2.357361	3.809321	4.962233	5.2443	0.816986	0.264451	2.695588	0	0.366001	0
LOC107812818	0.130819	0.309985	0.272923	0.023703	0.023151	0.140646	0.065834	0.022355	0.073691	0	0.09173	0.022104	0	0.057087	0	0.311956	0.353869	0.312099	0.053098	0.110664	0.047724	0	0.069234	0	0	0	0.030923	0	0	0
LOC107795346	16.20118	15.87556	17.63065	12.35996	9.3776	11.59034	8.786632	10.09583	12.29411	7.24579	7.858238	10.03386	10.57714	6.511729	6.188784	12.07314	15.53402	13.16847	6.736626	8.037569	8.998765	8.240094	11.22826	9.867248	2.464147	2.118681	3.959275	4.35569	3.811945	0
LOC107814244	0	0	0.081019	0.084437	0	0	0	0	0	0.087503	0.181768	0.065354	0.07874	0	0.101679	0	0	0	0	0	0	0.085002	0.077836	0	0.117836	0	0	0	0	0
LOC107786565	11.31824	11.65855	12.90472	0.596148	0.302783	1.160252	0.397385	0.449796	0.642506	0.256668	0.22148	0.200134	0.234412	0.689171	0.054031	8.022455	8.315208	10.26324	0.320511	0.089065	0.648152	0.637471	1.207299	1.100448	0	0	0.12444	0	0	0
LOC107802394	56.91808	32.39959	54.40901	11.4779	9.035557	7.725475	9.992019	8.563172	11.007	32.63757	8.883842	9.744998	16.41939	17.12249	5.822507	25.92836	35.62323	25.09436	4.988937	4.798882	11.55477	5.685103	11.5089	7.708105	150.3786	7.367196	270.8783	81.13849	150.2124	46.76851
LOC107796018	0.195083	0.711176	0.63593	0.176735	0.086311	0.262172	0.409061	0.291697	0.274729	0	0.102594	0	0	0	0.100114	0.36344	0.094233	0.327246	0.049489	0.082513	0.088959	0.325838	0.215095	0.10195	0	0	0	0	0	0
LOC107823909	7.448001	7.244578	7.404347	7.37319	7.764785	4.746271	8.245986	7.543965	6.77638	4.45008	5.573878	3.866891	4.597858	4.757054	6.76879	9.485898	6.894497	7.713345	6.211069	6.861814	6.03933	9.013375	8.134396	8.138496	3.972067	5.973058	3.121042	3.873727	5.411197	8.373112
LOC107762577	0.729593	0.963008	1.180961	1.253572	1.447017	1.108982	1.730317	1.117792	1.25185	0.490652	0.564514	0.786417	0.980233	1.097863	0.877944	1.012395	0.826374	1.106642	1.250921	0.872573	1.147244	1.302646	1.442438	1.367368	0.731581	0.385067	0.297352	0	1.211214	3.111162
Novel00521	0.040354	0.294223	0.596344	0.109677	0.035708	0.520628	0.067694	0.068959	0.037886	0.03935	0.113186	0.034092	0	0.088049	0	0.330792	0.272899	0.451287	0.245692	0.068274	0.073607	0.101103	0.07119	0.168713	0	0.16845	0.047695	0	0	0
LOC107801121	7.116423	7.473418	7.944656	4.9417	4.539011	6.291706	6.21124	6.882963	6.511637	5.072463	6.61117	6.195263	7.47975	6.936068	5.524422	7.806692	5.374415	7.944927	4.508702	4.155942	4.908905	5.973279	6.691384	6.645191	5.526274	3.166624	4.354954	5.16675	13.73918	4.467199
LOC107806636	4.707332	4.446158	4.563181	0.542768	0.201957	0.521432	0.119644	0.268138	0.910675	0.584213	0.80019	0.120512	0.158796	0.995969	0.17569	2.551207	3.665704	3.169199	0.086849	0.120669	0.052038	1.119801	0.830436	0.298188	0	0	0	0	0	0
LOC107766389	0.056811	0	0	0	0	0	0	0	0	0	0	0	0	0	0	0.296349	0.439074	0.42355	0.057648	0	0	0.142333	0.050111	0.118758	0	0	0	0	0	0
LOC107801747	0	0	0	0	0	0	0	0	0	0	0	0	0	0	0	0	0	0	0	0	0	0	0	0	0	0	0	0	0	0
LOC107780817	0.119862	0.10924	0	0.054295	0	0	0	0	0	0	0	0	0	0	0	0	0.173695	0.044681	0	0	0	0.1001	0	0	0	0.250169	0	0	0	0
LOC107783986	0	0	0	0	0	0	0	0	0	0	0	0	0	0	0	0	0	0	0	0	0	0	0	0	0	0	0	0	0	0
Novel04653	0	0	0.008907	0.018565	0.0272	0.011016	0.025782	0	0.028859	0	0.007185	0.008656	0.034219	0	0.021033	0	0	0.015278	0.031192	0.043339	0.009345	0.008557	0.018076	0	0.012955	0	0.024221	0	0	0
LOC107812554	0.197287	0.224754	0.128623	1.921372	3.229595	1.74989	2.895783	5.099169	2.778329	4.472834	2.351745	3.125133	2.251135	6.941189	3.341089	0.073509	0.047649	0.073543	4.404256	2.62854	5.577808	4.530892	2.914842	2.165148	25.81611	41.58839	22.73482	28.21765	3.324338	9.148925
LOC107812502	0.839419	1.428051	1.167503	3.498168	2.22832	2.286278	2.62848	1.769149	2.154096	2.728466	3.610088	2.41116	3.239438	3.174653	4.250347	4.128539	3.568178	3.504607	5.848921	5.633388	4.185086	4.25286	3.011055	3.567934	4.174372	2.881044	5.225197	0	1.347088	0
LOC107815032	13.42069	9.796915	12.62907	15.46367	15.44969	16.39008	10.05436	11.80099	12.35527	9.91044	11.64903	12.7692	21.1047	19.33221	8.759591	13.0597	11.45497	14.37711	10.31295	6.944053	12.71523	9.358107	12.12526	16.07011	4.200772	3.988601	6.314068	9.318143	10.66411	0
800493	0	0.37006	1.76483	1.103568	0	1.746191	0.340567	0.693871	1.143638	0	0	0.686076	0	0	0	3.328435	3.53046	1.210894	3.708235	0.686972	0	2.37369	0.358158	0	0	1.694945	3.839297	0	0	0
LOC107820500	4.932009	2.372318	2.977285	2.399562	2.545763	2.847646	2.71949	2.028983	3.001177	3.028098	2.753854	3.317926	4.778665	3.387772	2.437297	3.947586	3.264726	4.834603	2.085276	1.197559	1.166168	1.105987	2.094617	2.338813	1.154733	0.953129	1.511284	3.265818	3.957408	8.470932
LOC107780214	3.110676	3.200803	4.230521	3.81809	3.662641	3.290415	6.185991	5.229954	4.875249	6.2623	6.596394	4.832102	5.417681	5.500907	7.672808	9.851836	6.446583	8.71548	7.509412	6.005574	7.389897	9.595124	11.5506	10.19957	4.154855	5.410347	3.854467	5.382031	7.367176	10.85778
LOC107766468	2.046084	1.631659	2.934719	0.880483	1.403145	2.254786	0.343228	0.917821	1.152573	0.548677	0.286943	0.777865	0.626376	0.446436	0.262506	1.867812	3.360372	2.097484	0.934301	0.778881	0.933032	0.55534	1.127989	1.496997	0	0.071174	0.78595	0	0	0
LOC107828800	0.113376	0.103328	0.197111	0.102713	0.401291	0.243786	0	0.096872	0	0.110556	0	0.191567	0	0	0	0.253464	0.109531	0	0	0	0	0.189367	0	0.474003	0	0	0	0	0	0
LOC107800961	0.246638	0.196683	0.375195	2.010974	9.275268	1.060664	1.861794	4.557129	3.849597	2.435096	2.983263	3.099455	6.314902	28.15145	29.87082	0.229744	0.268057	0.09194	31.12749	23.23715	33.71259	14.05772	5.846686	4.865712	88.44151	152.8004	25.83475	74.96197	39.77802	394.5938
LOC107783299	1.456298	1.626935	1.225093	4.128234	3.450199	3.636462	3.427969	3.211099	2.513952	4.076989	5.171667	5.318163	4.079077	3.74125	2.121516	1.995432	1.9969	1.891275	6.101681	5.007192	3.556064	2.746242	2.983474	3.093346	9.859349	8.758987	7.995379	3.359546	13.79611	5.809362
LOC107790767	7.980296	7.087924	8.223619	13.72337	14.48248	14.72605	20.59139	20.45574	22.23156	7.838391	8.017239	7.820692	15.57084	14.373	16.56022	6.638932	7.148936	7.940078	14.60558	11.41496	11.03634	21.05999	21.21983	21.59562	5.72351	9.044712	6.379508	14.52701	12.01498	5.382913
LOC107831329	1.855644	1.191521	1.466429	1.031596	0.97027	1.042864	0.919696	0.936894	1.108647	1.233741	0.975884	1.353922	1.032864	1.610331	0.086572	0.942837	1.588991	1.320577	0.77031	0.784873	0.423095	0.528306	1.0788	1.366479	0.799806	0.410772	0.847381	0	0.203039	0
LOC107786478	0	0	0	0	0	0	0	0	0	0	0	0	0	0	0	0	0	0	0	0	0	0	0	0	0	0	0	0	0	0
LOC107817807	3.059986	2.296659	3.494485	3.859327	3.132411	4.192958	4.881462	6.664501	6.590655	2.3988	2.818814	2.635851	7.146801	2.880097	2.894358	2.012034	1.680979	2.147152	2.313576	2.030226	4.924868	4.659983	6.403771	4.515242	2.806742	2.755013	2.623849	8.581658	10.68782	3.709873
LOC107780343	7.856964	3.119493	3.922116	5.403111	3.372927	5.687222	3.262364	2.414981	4.844608	6.574252	6.854816	11.93924	10.56506	12.16426	5.801946	4.75358	4.509201	5.664377	1.657691	2.061935	2.625052	1.580837	2.012778	4.878473	1.671991	0.252564	2.635715	3.708806	6.741307	0
LOC107779802	3.168991	3.276933	2.913664	1.821946	1.590805	2.260448	1.993477	1.562118	2.60329	1.753075	2.56396	2.188138	3.493844	2.692637	1.563733	2.134465	2.266696	2.612509	1.082198	1.08261	0.416852	2.137563	2.230832	2.293081	0.65492	1.059957	1.152459	0	1.760383	0
LOC107791819	1.047595	1.613989	1.344296	1.310618	0.926981	1.957604	1.50628	0.916406	1.358206	0.924248	1.154331	0.990399	1.527164	1.442199	1.356829	1.486989	0.939775	1.320307	0.759305	0.759594	0.796187	1.416463	1.298068	1.929191	0.283817	0.277648	0.560126	1.783759	0.120083	0
LOC107770557	1.035758	1.258621	1.543478	1.653273	1.571155	1.696863	1.613365	2.149237	2.130052	3.126175	2.524667	3.250138	1.756983	2.31373	1.97428	1.139395	1.191225	1.21346	2.352273	2.33648	2.563992	1.318078	1.653194	2.216699	2.431953	2.539225	2.623248	0	2.61198	3.049642
LOC107764624	0	0	0	0	0	0	0	0.022788	0.025039	0	0	0	0	0	0	0	0	0.019884	0	0	0	0	0	0	0	0	0	0	0	0
LOC107811329	1.333101	2.100864	1.279549	1.409009	1.204191	1.433247	1.351073	0.878008	0.860457	0.622889	1.538478	0.375414	1.267606	0.787772	1.282742	1.862678	1.34155	1.697884	0.732734	1.386146	0.227964	1.948288	1.200381	1.045018	0.10534	0.19322	0.131302	0	0.267418	0
LOC107763436	5.178227	4.719316	4.674451	1.804312	2.819715	3.64011	0.501139	0.51051	2.991722	0.582626	0.139653	1.009549	0.665132	1.086383	0.613245	3.265156	4.42538	5.34543	3.435639	3.201079	0.726559	1.164281	1.405397	3.12247	0	0	1.647759	0	1.917679	0
LOC107810915	0.310859	0.708274	0.31526	4.69371	4.126029	1.002635	3.432957	3.054474	1.556524	4.85003	4.03253	1.531964	1.787939	2.091314	3.562806	2.084873	0.400421	0.386264	5.940756	10.168	8.363508	1.384568	1.279592	1.028881	3.340674	0.576717	2.878049	0	0.249431	6.406962
LOC107763678	0	0	0	0	0	0	0	0	0	0	0	0	0	0	0	0	0	0	0	0	0	0	0	0	0	0	0	0	0	0
LOC107776801	0.26219	0.504458	0.405185	0.211139	0.128891	0.501131	0.244345	0.124457	0.273506	0.11363	0.142993	0	0.064861	0.095346	0.119602	0.151966	0.281442	0.238913	0.147807	0.32037	0	0.170304	0.282662	0.243592	0.110496	0	0.034432	2.083367	0	0
LOC107787208	1.035173	1.572388	1.259796	13.00436	17.34273	14.83917	9.840102	12.44169	9.329915	9.219411	7.74255	7.287859	15.90269	15.9611	18.34537	1.388542	2.0668	1.49208	25.35019	22.06727	19.826	7.377068	4.139347	3.750804	11.3427	8.834256	7.830345	19.7412	26.57969	21.33544
LOC107819358	0.62628	0.830221	1.138315	1.134755	0.906829	1.530289	1.241589	0.68105	1.069053	0.33311	0.119768	0.288599	0.06338	0.248451	0	0.678843	1.100071	0.594259	0.577732	0.192651	0.467327	1.569068	1.35594	0.416556	0	0	0.33646	0	0	0
LOC107814649	0	0	0	0.225432	0.385325	0.066882	0.208709	0.318917	0.058404	0.545952	0.436208	0.998558	0.41551	0.542933	0.255397	0	0	0.046379	0.189375	0.473619	0.340413	0.103904	0.164617	0	1.887618	1.125266	1.249937	0	0.59899	0
LOC107806018	0	0	0	0	0	0	0.043455	0	0	0	0	0	0	0	0	0	0	0	0	0	0	0	0	0	0	0	0	0	0	0
LOC107818659	7.015315	7.507759	7.700489	9.286215	9.652587	10.57543	8.960157	6.974339	6.480341	8.216317	8.479917	7.690452	9.547327	9.397427	9.28514	8.269207	7.903919	8.469302	10.68753	11.98032	8.559368	8.560259	8.560304	8.256327	10.67668	8.164947	9.469692	20.17537	9.235846	15.1179
LOC107786975	0.04702	0.042853	0.040873	0.042597	0.124818	0.404414	0.118312	0.040175	0.22072	0.04585	0.263761	0.397234	0.418742	0.307776	0.04826	0.035039	0.045425	0	0	0.119326	0	0	0	0.049145	0	0	0	0	0	0
LOC107799933	3.423514	2.392085	2.896628	3.680482	4.200699	3.729772	4.345459	3.315162	3.492705	3.38287	4.449056	3.644272	5.259323	4.307568	3.912032	3.418617	2.954613	3.539293	2.5244	2.22031	2.68518	3.983632	2.536603	3.673676	1.211941	0.8892	1.753404	3.264395	1.538321	0
LOC107788168	3.312863	3.661158	4.172311	6.049837	6.116783	5.440758	3.631925	4.92569	3.453126	6.3083	3.969189	5.553523	5.430253	4.837867	4.310531	2.488189	3.402111	2.664359	7.570363	6.399306	6.32826	4.422297	3.520403	2.971841	4.551247	7.731074	6.258695	5.596455	4.018722	4.838724
Novel01720	1.492438	2.193828	1.088096	3.925374	3.53582	2.277431	5.208987	3.948943	2.621538	6.102936	8.945874	1.789598	3.858742	4.674442	11.31554	3.73114	1.023228	1.93821	5.080645	7.941532	2.546628	8.081328	3.779425	2.515961	9.312742	13.0626	2.901969	0	3.940232	5.953529
LOC107797614	24.6493	20.9155	32.60246	0.096254	0.282043	1.370742	0.356456	0.09078	0	0	0.0745	0	0	0	0	12.03466	25.55824	11.48569	0	0.089878	0.96899	0	0.281151	0	0	0	0	0	0	0
LOC107771651	0	0.090412	0	0.089874	0.263347	0	0	0.169525	0.093137	0	0	0.167621	0	0.108227	0.10182	0.073927	0	0	0.100665	0	0	0.165696	0	0.311064	0.376272	0	0	0	0	0
LOC107782735	4.108217	4.586563	3.615825	5.396662	3.953302	5.300202	4.99632	5.045872	3.856975	4.156256	5.689351	4.121498	4.744824	4.201746	5.692357	4.286027	4.018494	3.981705	4.846154	5.473548	5.339137	4.974775	4.12198	3.757168	1.298509	4.215759	2.367096	0	1.483381	0
LOC107803211	36.65931	19.64795	20.41313	27.27241	20.37512	18.90012	74.07908	52.97551	55.11679	15.39303	21.6576	21.51975	30.64355	35.29676	24.13561	28.0477	49.31139	36.25501	14.39654	12.5259	25.46212	63.16417	36.73754	33.68016	9.319185	3.176702	12.25838	4.664874	6.123797	4.033275
LOC107772048	1.953681	1.828662	1.331095	80.41248	144.0465	93.61175	35.65141	52.55969	26.0754	147.5669	77.12291	120.3544	107.0386	125.866	90.55936	2.24285	2.346522	1.614017	193.8525	143.0238	228.0691	30.47067	22.07656	53.42278	399.5467	177.9453	417.509	275.6555	501.2991	235.0684
LOC107810091	6.285571	5.171584	4.249711	4.191718	4.21004	4.739816	4.759426	4.811129	4.5898	4.384099	5.876595	3.687656	7.43451	5.761994	3.628878	3.447956	2.234977	2.701051	1.550248	1.772387	1.273899	2.00492	2.502631	2.600497	0.607053	0.971768	1.599307	3.121578	3.572477	0
LOC107824862	82.70625	85.46131	96.97593	30.07413	35.13927	35.35648	35.88815	37.70792	51.82215	47.64823	67.03989	55.01998	64.2675	63.88479	52.35476	130.0666	77.37852	125.3266	49.47851	52.09558	45.08321	44.17553	44.28681	83.74231	37.49203	41.33372	49.53304	51.76951	70.44963	33.57014
LOC107791792	0	0.143213	2.140033	0	0.092698	0	0.131799	0.044755	0.295059	1.63446	0.330558	0.398267	0.758028	0.400006	0.053761	2.263941	0	0.937232	0	0.177239	0.286627	1.968467	0.046202	0.930704	0	0	0	0	1.765224	0
Novel00829	2.849108	2.396872	1.566441	0.728016	0.84036	0.628336	1.327594	1.227581	1.326003	0.92608	1.195263	0.946344	5.557195	2.523782	2.074466	2.486092	3.175936	2.432766	0.444781	0.47379	0.732892	2.542056	1.804278	1.578026	0.646538	0.677663	0.43172	0	2.930902	9.034085
LOC107802822	1.06016	0.286283	0.238927	0.355723	0.382189	0.464364	0.329334	0.43614	0.221183	0.229731	0.633254	0.099517	0.305974	1.028073	0.241804	0.380386	0.303468	0.234191	0.039844	0.431803	0.250674	0.098374	0.138538	0.16416	0	0.054635	0.092817	0	0	0
LOC107802522	0	0	0	0	0	0	0	0	0	0	0	0	0	0	0	0	0	0	0	0	0	0	0	0	0	0	0	0	0	0
LOC107778199	1.358988	1.146806	1.283431	2.781542	5.225775	1.334812	5.671065	6.723238	1.921693	2.961216	3.564639	2.707274	5.509742	5.289732	6.285336	2.438028	0.761804	1.501012	10.55536	8.430476	8.981969	7.944485	3.640549	2.121853	17.47871	16.27136	3.490046	49.19335	18.901	43.57022
107790979	0	0	0	0	0	0	0	0	0	0	0	0	0	0	0	0	0	0	0	0	0	0	0	0	0	0	0	0	0	0
LOC107791596	4.982762	3.699394	4.753985	3.391102	5.226367	3.553964	6.258692	5.607316	8.8084	3.697445	2.812176	3.942627	7.170357	8.697517	4.715015	4.87239	5.142512	6.215595	4.908177	3.824408	5.231559	5.01378	4.13785	4.67448	4.947589	4.396631	5.171025	5.214701	10.18061	9.017317
LOC107810369	0.558122	1.068185	1.018844	2.907375	3.234804	2.880238	3.206629	3.719623	1.729166	1.333387	2.680786	0.660124	1.615404	2.313758	4.72594	1.497288	0.862709	1.061065	7.75891	7.38888	4.377544	6.012721	3.96302	1.37087	11.32546	15.68704	2.341777	5.987044	8.732745	65.56811
LOC107824222	0.157297	0.047785	0.136735	0.237505	0	0.169113	0.131931	0.044799	0	0.051128	0.073531	0.354369	0.058368	0	0	0	0	0	0	0	0	0	0	0	0	0	0	0	0	0
LOC107829324	0	0	0	0	0	0	0	0	0	0	0	0	0	0	0	0	0	0	0	0	0	0	0	0	0	0	0	0	0	0
LOC107805712	0	0.054934	0	0.109214	0.373353	0.583236	0.707784	0.721019	0.396128	6.465428	0.803045	0.661996	18.18409	68.84868	93.10763	0.179671	0	0.044938	0.244655	0.407914	0.164918	0.453042	0.212669	0.252001	0.076207	1.174173	0.213724	4.310579	0	3.72695
LOC107809197	50.35293	50.3093	47.57087	75.28458	56.01977	83.44652	63.63309	70.25603	67.94406	98.23968	95.30518	113.1814	36.36625	50.77035	46.05185	33.16956	43.23161	35.22916	64.92693	94.43541	77.16611	57.08587	77.92714	65.67185	57.43093	67.51872	88.82343	39.78946	22.57711	49.14597
LOC107805774	1.840822	2.222385	1.870349	2.404075	2.009658	1.747785	3.208264	2.430766	1.548687	1.771722	1.760162	1.353207	1.942776	1.225813	1.423161	2.084397	2.124829	2.120999	1.479796	1.718997	1.460827	2.315961	2.593746	2.098938	1.450821	0.765085	1.045468	1.709673	3.567969	0
LOC107823253	1.20275	1.973085	1.934221	2.12478	0.266068	0.12931	118.786	78.87308	55.16116	5.395032	44.40338	2.032233	82.67819	23.74975	33.45413	0.672218	0.580979	0.538021	0.061023	0	0.164539	101.7506	22.91555	0.754268	0.836353	0.502062	1.705864	0	0.579047	0
LOC107759236	2.693816	1.303315	1.532209	3.012915	3.590213	4.648193	1.589962	1.733356	2.59152	1.459339	1.25927	2.416293	4.368613	5.478543	2.150446	0.991326	1.638582	1.165341	3.644665	3.516641	3.761036	1.721975	2.200115	3.128418	2.606911	2.08236	1.768819	0	5.603885	2.05633
LOC107765075	3.570524	3.254092	3.277088	6.14103	7.553774	6.976001	6.871207	5.714353	5.206916	8.165232	8.273494	6.73731	9.38204	7.592802	10.39878	4.457108	3.677053	4.6213	6.069187	7.758026	4.330817	6.690214	5.723344	5.228463	5.293332	5.573377	3.834481	6.480785	4.101113	3.361993
LOC107812450	0	0	0	0	0	0	0	0	0	0	0	0	0	0	0	0	0	0	0	0	0	0	0	0	0	0	0	0	0	0
LOC107771947	3.056644	2.532504	3.220698	1.84611	3.032564	2.141052	1.942226	2.453396	3.347997	1.219342	1.883524	1.565053	1.392013	1.162074	0.903149	3.968919	3.624106	4.246962	2.584726	2.350646	2.872187	3.829025	4.575305	8.277445	0.585534	0.708849	1.532664	6.624038	0.445932	0
LOC107800265	0	0	0	0	0	0	0	0	0	0	0	0	0	0	0	0	0	0	0	0	0	0	0	0	0	0	0	0	0	0
LOC107816069	0	0	0	0	0	0	0	0	0	0	0	0	0	0	0	1.552026	0.603617	1.552737	2.536049	3.523632	1.139671	1.913244	1.653367	2.721039	0.789948	0.434688	1.476946	0	1.504025	0

LOC107759668	20.26885	16.03662	16.87708	23.74352	22.30405	34.3236	16.87581	12.87768	18.2974	17.41175	17.83074	17.71957	32.39897	28.95661	16.07881	13.61053	15.20608	17.15934	11.30079	9.734939	8.971917	14.5089	12.99955	19.32258	7.462514	4.338866	12.24133	7.964338	27.88042	11.47668
LOC107816325	105.0319	76.11556	83.33179	118.3457	111.0128	134.5891	92.66319	86.71706	107.4722	126.6742	90.70785	170.2585	138.9534	144.5925	106.8701	75.9698	119.2728	82.84027	101.1442	99.52398	146.4598	73.00726	77.75138	93.45945	104.5581	75.6615	125.3194	331.0928	180.9365	61.74335
LOC107826893	1.971281	3.59316	1.822393	4.308792	4.263892	4.238737	7.532149	7.165028	3.877711	3.41733	6.911319	3.753744	4.075422	2.696748	5.491731	2.798097	1.934652	1.936237	4.159396	5.505623	3.852518	7.813243	7.369202	4.284288	1.621972	3.308888	2.181966	0	2.259631	0
LOC107814597	0.422607	0.149782	0.122454	1.680331	2.513777	1.8679	2.146443	2.367124	2.292402	1.121814	0.806682	1.051255	0.522724	1.126995	1.349451	0.017496	0.113409	0.332575	1.453265	1.191654	1.477461	1.705828	1.801708	0.981574	0.534303	0.88204	0.77698	0	0.565161	1.45169
LOC107782184	1.408689	1.241052	1.387816	1.999382	2.181377	2.322254	1.7526	1.705132	1.454793	2.976241	2.123714	2.69756	2.038624	2.484511	1.686813	0.82231	1.587731	1.347805	1.739153	1.727899	1.092036	1.41172	1.283976	1.889531	1.870062	1.306726	1.914701	1.67902	1.921547	1.45169
LOC107829674	6.531383	5.761951	4.950413	6.820699	5.992922	7.765749	6.759986	5.745535	6.872013	5.851252	8.719687	5.436349	8.95422	7.581725	6.290847	6.905182	5.501705	7.088249	4.211625	4.422802	2.905007	6.623356	6.782787	7.364657	3.21357	2.372714	3.973867	0	5.034202	0.994693
LOC107786473	3.320598	2.245331	4.935028	4.075753	3.601767	4.606515	2.874972	3.111778	3.720916	1.880131	2.478625	4.705721	2.623348	2.220309	1.868997	2.235045	2.794045	3.194383	4.456454	4.16819	4.982275	4.383316	5.574524	9.628362	1.4897	1.043309	3.92468	7.660314	3.609864	6.623148
LOC107768029	29.778	20.72097	26.99309	22.72417	20.29626	27.68864	22.33222	20.45765	22.54182	26.94453	21.91496	30.1434	22.99545	24.14524	16.79606	17.59255	26.4355	20.20069	15.58471	14.18363	19.69569	15.79478	18.63478	23.41306	19.0786	9.5186	29.01225	47.96279	32.28871	16.58755
LOC107813120	0	0	0	0	0	0	0	0	0	0	0	0	0	0	0	0	0	0	0	0	0	0	0	0	0	0	0	0	0	0
LOC107780580	10.96254	14.37348	12.16691	17.65889	16.89335	18.52537	17.74132	15.16279	15.29038	21.24017	23.90377	15.6937	21.53961	18.20481	17.97914	16.14663	12.69229	14.95901	17.39763	18.37762	14.48799	15.03541	15.8805	14.18045	25.18681	19.15658	19.5875	2.046936	19.29206	23.0073
LOC107772951	0.408685	1.117397	0.592102	1.234159	0.783534	0.805541	1.428256	0.931177	1.342919	1.527657	1.43285	0.805626	1.213209	0.297237	1.677851	3.1978	2.105727	1.980497	0.622058	1.094777	0.683334	1.478981	1.02138	1.779822	1.894573	1.232086	1.771119	4.871123	6.558513	8.423198
LOC107762529	0.416447	0.484968	0.442455	0.67072	1.003138	0.547227	0.388102	0.573271	0.760236	0.203044	0.259567	0.312734	0.54086	0.555284	0.284953	0.74136	0.312918	0.58646	0.516489	0.313143	0.253204	0.386429	0.857112	0.87054	0.058502	0.193152	0.05469	0	0.668308	0
LOC107802533	0	0	0	0	0	0	0	0	0	0	0	0	0	0	0	0	0	0	0	0	0	0	0	0	0	0	0	0	0	0
LOC107760853	0.098089	0.178793	0	0	0.086796	0.105458	0.164544	0	0	0	0.06878	0	0.109195	0.428043	0	0	0	0	0	0	0	0	0	0	0	0	0	0	0	0
LOC107772936	2.494274	4.30716	3.423505	7.135851	7.782925	3.105068	0	0.560836	0.246498	7.552722	6.535687	4.547185	2.922801	3.723653	7.545425	5.47838	5.453484	6.068127	10.79013	9.106249	13.05028	1.754134	0.926364	0.823268	10.29044	4.01859	6.671861	18.77641	14.53639	32.46837
LOC107784864	0.104655	0.023845	0.045487	0.071109	0.046303	0.168775	0.043889	0	0.024564	0.025513	0.110076	0.088415	0.058252	0	0	0.21447	0.328593	0.331605	0.212393	0.154929	0	0.15295	0.207703	0.51958	0.132316	0.109215	0.309234	0	0.377884	0
LOC107829275	12.69113	12.75809	10.63096	18.77929	17.86583	27.04095	15.70885	14.52392	13.39534	19.0882	20.17113	18.09713	19.95037	17.41164	15.86783	11.95077	11.88916	13.21782	18.49761	19.12929	14.0648	13.00749	15.19727	14.95301	14.48953	9.525041	19.09078	16.50173	12.03477	7.133743
LOC107785624	0	0	0	0.134887	0.76414	0.128061	0.249762	0.585195	0.419356	0.580749	0.48025	0.654093	0.62984	0.877135	0.855777	0.044381	0	0.066603	2.507995	1.360274	2.471394	0.746055	0.288929	0.280117	5.4967	3.397598	1.654183	6.388676	3.440702	1.841228
LOC107763688	22.17281	22.63783	21.62266	45.1014	35.94924	45.52698	41.66735	44.93455	37.51638	32.97931	35.54789	31.06227	26.87019	33.52841	40.90601	20.0004	23.75946	19.48643	47.91811	53.18348	46.90731	35.10044	40.57024	30.91225	54.20339	66.92704	58.92312	45.16148	37.32795	36.87758
LOC107822538	0	0	0	0	0	0	0	0	0	0	0	0	0	0	0	0	0	0	0	0	0	0	0	0	0	0	0	0	0	0
LOC107762386	4.771436	3.994247	3.871194	3.105925	4.28463	4.217899	4.002009	3.291674	3.185518	5.06633	4.78316	5.165705	5.429665	5.051168	4.171749	4.767244	4.507172	3.873513	3.299545	2.810465	2.739915	2.39085	2.868166	3.287794	1.563993	1.376999	2.339329	2.527593	1.871743	2.185371
LOC107772218	0	0	0.038718	0	0	0	0	0	0	0	0	0	0	0	0	0	0	0	0	0	0	0	0	0	0	0	0	0	0	0
LOC107812266	4.687869	4.839163	5.738357	5.590402	4.44443	7.251475	4.293012	4.495905	3.772438	6.810251	4.595283	5.29406	6.816132	6.784189	3.927755	5.739166	3.743254	5.813121	5.727727	6.191215	7.591044	4.114705	3.839653	6.899661	7.559811	4.925401	6.445286	3.420907	14.50869	17.74641
LOC107819821	0	0	0	0	0	0	0	0	0	0	0	0	0	0	0	0	0	0	0	0	0	0	0	0	0	0	0	0	0	0
LOC107800270	2.102008	2.692365	2.321077	0.514679	0.603242	0.732946	0.14295	0.145623	0.373357	1.163356	0.956062	0.527951	0	0.185934	0.291546	2.455467	1.811179	2.837787	1.268254	0.913106	0.829002	0.142333	0.300666	0.059379	0.646438	0.395242	1.947236	0	1.914559	7.025422
LOC107828770	13.09494	13.79214	13.47722	11.77941	11.01337	9.795296	14.09177	11.84838	14.49777	9.787728	11.60766	11.53262	11.10501	11.42201	9.763228	16.34066	15.57482	16.09486	8.712311	8.699954	11.91464	11.60653	13.23961	18.62571	4.958986	3.910565	8.286124	11.04332	4.014576	5.728867
LOC107787749	1.304531	0.956305	1.109349	1.721374	1.631126	1.951347	1.498532	1.453861	1.810502	1.438006	2.187417	1.605239	1.546932	2.196646	1.106077	2.113344	1.178094	1.924024	1.035977	1.103544	0.724198	1.160499	1.225727	1.71919	1.362485	0.27622	1.240184	0	1.228789	0
LOC107804992	0	0	0	0	0	0.302767	0	0	0	0	0	0	0	0	0	0	0	0	0	0	0	0	0	0	0	0	0	0	0	0
LOC107784945	0.328606	0.17969	0.22852	0.44655	0.261695	0.42395	0.220493	0.168462	0.370212	0.192259	0.138251	0.249854	0.292647	0.609438	0.269818	0.53873	0.476191	0.759468	0.600204	0.361371	0.209786	0.411641	0.202896	0.755609	0.29082	0.045723	0.194193	0	0.316405	0
LOC107794777	1.911875	2.511162	2.224084	1.986777	2.662023	2.297323	2.594014	2.402291	2.9036	1.973986	2.365779	2.066513	2.347419	2.57653	2.337441	2.074242	2.172979	2.704038	1.711799	2.307052	1.923157	2.535867	2.653601	2.174611	0.888673	1.877813	1.794454	2.010678	2.301112	1.738443
LOC107769981	0	0.046012	0	0.068607	0.089348	0.02714	0.359934	0.366665	0.189596	0.221538	0.920431	0.362546	0	0.027539	0.103636	0.037623	0.024387	0	0.384227	0.405727	0.322312	0.358382	0.578922	0.18469	0.031915	0.316117	0.059671	0	0.12153	0
LOC107814184	0	0.031059	0	0	0	0	0	0	0	0	0	0	0	0	0	0	0	0	0	0	0	0	0	0	0	0	0.040279	0	0	0
LOC107791178	1.288555	6.282821	1.120114	1.634312	0.456079	0.346339	0.756537	0.440391	0.362926	0	0.225884	0	0	0.140575	0	6.193501	1.18261	2.017412	0.784522	1.52604	0.117518	0.215221	0.681954	0	0	0.089647	0.076148	0	0	0
LOC107767727	419.6948	444.8125	412.0097	267.7128	206.2807	282.8607	225.9836	245.9528	292.5562	272.184	269.9669	218.0286	171.4895	176.8675	192.4459	372.8617	368.0678	367.8198	191.7134	236.7104	223.8483	247.7197	308.459	277.5721	93.52422	124.8034	235.0338	135.3605	62.85774	74.04152
LOC107800035	6.285571	4.20739	5.03174	6.659563	6.127503	8.552191	4.765631	3.974823	6.434611	6.717909	5.478182	5.850248	7.273911	6.934732	4.410237	3.704871	4.597177	4.580258	5.513328	5.88793	3.854085	3.440183	5.638261	6.235626	3.90609	3.755308	4.616917	2.539589	4.445115	6.587226
LOC107766115	0	0	0	0	0	0	0	0	0	0	0	0	0	0	0	0	0	0	0	0	0	0	0	0	0	0	0	0	0	0
LOC107765729	1.613698	1.1527	1.251104	0.592674	0.578881	0.422008	0.512127	0.857084	0.65514	0.680458	0.550472	0.700071	0.534062	1.046763	0.447637	1.365034	1.390431	1.20308	0.132768	0.18447	0.198881	0.473496	0.615519	0.638187	0.110281	0.12137	0.154643	0	0.419941	0
LOC107782949	199.6321	136.3112	124.8033	215.9543	193.4313	183.343	161.4117	159.4873	163.6252	234.9058	256.2652	282.4241	328.9005	312.5694	252.1089	197.1791	283.1614	226.8846	205.3478	179.1368	292.9904	162.2968	165.203	196.0496	334.0453	179.1332	413.3046	376.0937	233.7765	177.761
LOC107804857	11.3858	9.357607	10.97997	1.519614	1.011988	2.213239	1.811897	1.758918	3.364326	0.743477	1.140533	0.429421	0.735593	0.693156	0.15651	10.26499	9.501921	11.14128	0.541571	0.558977	0.625824	1.93143	3.362621	3.054799	0.032132	0	0.240305	1.817513	0.244711	0
LOC107785067	45.13415	51.58973	53.24823	38.48239	32.4577	35.11957	38.71689	36.88715	42.54399	20.93122	22.884	22.1642	19.66068	23.48328	22.89884	47.94254	50.42826	50.30532	24.5691	26.45811	27.03824	35.77655	42.31449	45.59646	8.015578	8.77952	12.16764	19.43115	10.75558	24.26706
LOC107790546	8.342536	9.957123	8.194973	3.650262	3.222494	5.581481	2.339636	1.213761	2.643112	0.30223	0.126776	0.218205	3.737813	2.226014	0.980849	6.236115	4.017319	6.219715	2.175327	2.425234	1.201347	1.207913	2.164314	1.241803	0.06531	0	0.091581	0	0	0
LOC107766711	4.15373	4.063967	4.194842	4.842185	6.26996	6.370287	4.636678	3.783924	4.243797	3.78237	3.769245	4.102652	5.949988	4.764755	4.420013	3.937486	4.632484	4.144223	4.09095	4.237193	3.091898	4.922761	4.283489	5.235311	2.780247	2.08236	2.960064	2.184194	3.528977	5.665399
LOC107819016	0.671789	0.756312	0.54962	0.698108	0.786763	0.807226	0.828615	0.793463	0.556504	1.021152	0.83128	0.801241	0.351927	0.431111	0.851742	0.633134	0.610828	0.500847	0.822032	1.370575	0.684763	0.957048	0.941128	1.197791	0.274788	0.494865	0.490412	0	0.85612	1.221697
LOC107794988	0	0	0	0	0.064374	0	0	0	0	0	0	0	0	0	0	0	0	0	0	0	0	0	0	0	0	0	0	0	0	0
LOC107830689	0.189782	0.691851	0.329946	0.859663	1.17552	1.224231	3.661097	2.918784	1.247228	2.035673	3.193802	1.603329	3.802818	4.140851	2.921802	1.27283	0.916726	0.424471	1.155464	0.481627	1.903926	0.95095	0.6696	1.586879	3.119244	4.753214	1.345841	0	4.568384	0
LOC107783287	2.278838	2.5961	2.709234	1.092977	0.68204	2.197821	0.618381	0.229071	0.66072	0.849647	0.986952	0.339746	0.298451	0.475284	0.240773	2.347502	2.363428	2.223652	0.748132	1.27571	0.64184	0.531753	1.478004	1.541191	0.169479	0.04663	0.514915	0	0.322681	0
LOC107765144	3.041175	1.989071	3.110151	11.89577	8.389686	13.15547	2.190656	2.751313	4.870616	1.255989	1.705979	1.178841	1.39402	1.444207	0.440665	1.653056	2.350424	2.614092	3.013363	3.208214	3.197801	2.569644	4.386705	5.721543	0.135705	0.398266	1.691494	0	0.344501	0
LOC107808320	60.00009	42.10859	53.83029	24.63508	20.15794	35.0134	16.6179	19.87574	22.36661	8.447631	9.674334	13.48564	9.197424	14.00153	8.150619	29.23026	45.87615	39.52976	11.47679	10.85689	20.7033	18.08698	25.96675	31.85908	3.042448	0.446448	7.679329	5.736437	5.792683	0
LOC107814543	0	0	0	0	0	0	0	0	0	0	0	0	0	0	0	0	0	0	0	0	0	0	0	0	0	0	0	0	0	0
LOC107800837	0.10073	0.091803	0	0	0	0	0	0	0	0	0	0	0	0	0	0	0.097314	0.225296	0	0	0	0	0	0	0	0	0	0	0	0
LOC107792663	0	0.209836	0	0	0	0	0	0	0	0	0	0	0	0	0	0	0	0.085827	0	0	0	0	0	0	0	0	0	0	0	0
LOC107765363	3.723548	2.583154	2.946938	2.517422	3.024368	4.511147	1.841231	1.40081	1.173973	1.815465	2.045897	1.244217	1.422947	2.455514	1.71123	2.2364	1.79863	2.444593	1.60723	3.478956	1.672626	1.160311	1.470634	1.829751	0.632377	0.579968	0.919598	1.987211	1.471577	0
LOC107767626	0.584161	0.141971	0.067706	0.282251	0.310142	0.334957	0.195984	0.199649	0.182812	0.189877	0.354999	0.263208	0.130059	0.21243	0.039971	0.348254	0.300986	0.406482	0.237106	0.065888	0.035518	0.260186	0.27481	0.244226	0.295423	0.054188	0.046029	0	0	0
LOC107794131	48.79741	50.91432	50.44217	21.46075	16.70622	17.45303	17.02647	17.45516	17.69503	21.17975	14.03069	12.02408	12.39489	13.62577	13.94544	44.75741	48.60781	37.87234	10.87261	10.51092	12.27394	19.21704	18.61761	19.05868	65.28546	5.8827	56.53028	6.924018	32.47353	13.9686
LOC107830393	12.00014	9.951832	11.02473	9.274282	9.712669	13.57422	6.678222	5.977011	7.955796	7.098633	8.534144	8.840766	11.0162	12.16088	8.755888	8.17965	11.31843	9.964239	9.522231	7.024118	8.817707	7.26687	8.22714	7.549304	6.902821	4.589785	6.251363	4.067203	6.571331	14.0661
Novel04745	6.146938	6.317348	5.002335	5.6873	3.008928	4.007409	5.21055	4.022893	5.771016	1.339094	1.834143	3.756714	2.183891	3.067642	1.476586	6.286288	5.054008	6.33792	1.658906	1.88081	3.101253	4.095853	5.133601	5.672942	0	0.181979	0.386446	0	0.944475	0
LOC107795639	0	0.040149	0	0	0	0	0	0	0	0	0	0	0	0	0	0.032829	0.04256	0	0	0	0	0.03679	0	0	0	0	0	0	0	0
LOC107762171	2.022883	1.3729	1.646208	7.369511	5.903126	7.49628	5.559332	6.141358	5.253028	4.322859	3.561207	4.617903	4.024675	3.145958	2.517983	1.379161	1.621633	1.315616	5.153542	5.424931	6.869321	4.421098	5.770556	8.052398	4.298843	2.155936	4.222201	3.077973	3.729784	2.661232
LOC107828719	0	0	0	0	0	0.027404	0.042757	0.043557	0.04786	0.024855	0	0	0.056749	0	0	0.018994	0	0.019003	0	0	0.023246	0.021286	0.067449	0	0	0	0	0	0	0
LOC107764219	7.001582	7.243929	8.440472	10.13297	10.16991	12.87733	8.051643	9.89532	10.60425	2.834026	5.280043	2.473939	3.308884	3.33879	4.112875	7.449008	6.147264	7.928455	14.76799	13.82	12.22895	13.64348	14.17729	17.92693	6.541667	9.619648	4.996412	11.02197	13.99205	16.33654
LOC107760345	1.659087	2.332883	1.483416	0.60122	0.419448	0.458671	0.636136	0.486024	0.934575	1.109362	0.232671	0.52061	0.949844	0.206855	0.097305	1.554274	4.350506	2.403159	0.769612	0.240596	0.518783	0.870915	1.212552	1.139537	1.55821	0	2.689245	0	2.966763	0
LOC107811022	0.154149	0.11239	0.0268	4.524692	4.501233	2.883681	2.120376	1.738558	1.881382	0.330692	0.475593	0.31255	0.13728	0.134535	0	0.045949	0.119137	0.04597	8.759495	6.285245	7.507308	2.677661	5.275614	3.351219	0.740586	0.986639	1.020272	0	1.187404	1.90625
LOC107820390	4.792919	3.66613	4.538382	5.117525	4.84696	5.429027	2.153586	4.168329	2.65167	3.505276	3.060727	4.338419	4.096936	3.268025	2.547506	3.444129	4.299623	3.06285	4.255583	3.909677	4.215108	2.144299	3.774707	4.383363	3.570899	2.262696	5.260213	0	2.472302	0
LOC107832569	0	0	0	0.147371	0	0	0	0	0	0	0	0	0	0	0	0	0	0	0	0	0	0	0	0	0	0	0	0	0	0
LOC107827331	2.093695	1.737775	1.690004	3.296787	4.102272	3.001304	4.296105	3.556523	3.311189	4.84884	4.74511	3.737668	4.633714	4.663408	4.246658	3.009003	2.371839	2.508651	3.667356	3.805804	3.262037	3.965347	3.693555	3.360649	6.018926	4.127067	3.343628	1.782486	6.599864	10.01745
LOC107806962	0	0	0	0.456283	0	0	0	0	0	0	0	0	0	0	0	0	0	0	0	0	0	0	0	0	0	0	0	0	0	0
LOC107819943	0	0	0	0	0	0	0	0	0	0	0	0	0	0	0	0	0.058165	0	0	0	0	0	0	0	0	0	0	0	0	0
LOC107776038	2.419468	2.654416	2.561708	4.300725	6.91989	5.362713	3.731626	3.840595	3.261922	5.926171	5.612226	6.684281	8.2461	7.655861	4.742939	2.674578	2.182321	3.248579	8.261267	6.566911	5.647211	3.447402	3.772658	4.793985	8.509951	7.562688	7.223581	6.560234	15.23648	15.59804
LOC107788311	12.15308	12.55284	13.76895	9.10166	8.997381	10.5835	11.89219	9.587818	11.71411	7.979528	10.7658	8.076909	7.891902	9.413471	8.149409	11.29042	13.97166	12.05064	4.439557	5.071796	4.618254	10.48766	12.22059	12.27898	3.073037	2.029214	5.458304	5.794112	1.755277	2.50481
LOC107794563	0	0	0	0	0	0	0	0	0	0	0	0	0	0	0	0	0	0	0	0	0	0	0	0	0	0	0	0	0	0
LOC107760780	52.84883	37.90515	37.37752	7.224251	6.848592	9.077585	6.426045	5.81143	7.633356	11.51136	12.77282	11.16204	18.97247	24.98973	9.14742	20.21584	35.72439	20.34167	6.10844	6.01818	8.841241	8.09585	5.8615	4.984457	2.174517	1.84926	5.728861	0	1.505521	0
LOC107807370	3.362851	4.364309	4.10425	5.642252	5.546652	6.088461	4.828812	4.631777	4.10435	3.908811	5.102783	3.000127	5.285924	4.490495	6.392248	4.651136	3.599667	4.282209	3.794576	4.346763	2.330902	5.998752	4.698551	4.23188	3.044193	3.574879	1.780629	1.923931	3.302752	2.495161
LOC107807251	11.59224	11.15184	14.15119	10.11302	9.751032	10.34743	9.602877	10.30214	10.44663	11.54812	10.1857	11.69773	8.96156	9.719121	8.592971	18.55689	12.68538	16.75153	11.94453	11.31955	12.95436	12.07135	10.50916	11.40569	8.142288	9.906858	7.654008	20.46936	7.923526	33.1836
LOC107812466	0.522057	0.581522	0.302542	0.052551	0.051328	0.31182	0	0	0.272295	0	0.081348	0	0.064574	0.126564	0.059536	0.129679	0.504351	0.086492	0.294304	0.098139	0.052903	0.145328	0.204662	0.36377	0	0	0	0	0	0
LOC107770872	0	0	0	0	0	0	0	0	0	0	0	0	0	0	0	0	0	0	0	0	0	0	0	0	0	0	0	0	0	0
LOC107773900	0	0	0	0	0	0	0	0	0	0	0	0	0	0	0	0	0	0	0	0	0	0	0	0	0	0	0	0	0	0
LOC107772300	16.65408	9.057921	12.72578	13.99267	15.33084	19.63793	11.82816	13.77067	15.00512	12.70374	9.417621	18.04115	16.14735	16.85008	10.75223	7.906778	10.63966	9.412373	11.03913	12.27037	13.59643	8.636578	14.57153	10.66871	8.490249	4.858841	10.47685	19.20971	18.75146	16.60882
LOC107782054	3.209396	5.684373	4.526942	1.097189	0.964489	2.14842	2.031591	2.276539	2.728865	0.708581	1.231368	1.381272	2.966052	1.915802	0.932274	3.294153	4.972574	3.792268	0.860253	0.922051	0.828403	1.011415	2.350181	1.455709	0	0.421287	0.071571	0	0	0
LOC107770597	0.588333	1.105383	0.448481	0.5576	0.86499	0.291937	0.948962	0.71923	0.594842	0.829657	0.8822	0.420573	1.128513	1.017074	0.836099	1.679511	0.428471	0.789529	0.936831	0.957095	0.429256	0.672748	0.630722	0.454099	0.778163	0.667492	0.363728	1.941884	0.958674	0.559654
LOC107764260	0.990634	0.789985	1.04054	0.972253	0.584385	1.286938	0.900128	0.45848	0.891299	0.402497	0.66569	0.453329	0.551394	0.675458	0.466015	1.076573	1.674812	1.230933	0.586382	0.488838	0.301158	0.68942	0.800987	1.596257	0.626231	0.229732	0.585425	0	0.596158	0
LOC107799283	0	0	0	0	0	0	0	0	0	0	0	0	0	0	0	0	0	0	0	0	0	0	0	0	0	0	0	0	0	0
107807468	0	0	0	0	0	0	0	0	0	0	0	0	0	0	0	0	0	0	0	0	0	0	0	0	0	0	0	0	0	0
107819110	0	0	0	0	0	0	0	0	0	0	0	0	0	0	0	0	0	0	0	0	0	0	0	0	0	0	0	0	0	0
LOC107805315	10.79725	12.39409	13.57534	10.79717	10.67814	11.2067	9.5575	10.11928	14.76696	7.104131	8.278371	7.543651	7.403109	7.051261	5.176716	11.86038	12.48839	12.42289	7.316842	8.312014	9.165805	12.97959	14.79664	16.43981	6.660182	3.015115	7.948331	2.671821	13.31023	9.240284
LOC107789546	10.98818	12.04737	11.65247	15.02567	11.56899	13.81219	16.02452	15.35354	15.53246	14.15893	14.93191	13.34885	15.68113	12.48329	13.88547	12.35957	9.737535	11.37971	11.08717	12.07334	10.96327	13.40255	13.93724	14.20494	8.069084	8.018237	9.667126	19.20214	11.13706	7.662586
LOC107817825	4.548645	4.551535	4.504346	6.075056	6.078905	5.671798	6.706002	7.051769	6.845957	4.321187	4.998	4.47669	5.716143	5.832038	4.93332	4.088554	4.575585	4.562399	5.567332	5.394911	5.474232	6.481274	6.493983	6.469691	8.389156	9.624159	5.099013	8.383826	7.224346	11.59792
LOC107803853	0	0	0	0	0	0	0	0	0	0	0	0	0	0	0	0	0	0	0	0	0.037944	0	0	0	0	0	0	0	0	0
LOC107783866	1.463122	1.638245	1.380877	1.666361	1.664571	1.123596	0.911622	1.178695	0.941927	0.856037	1.055255	0.706335	1.303014	1.276955	1.072649	1.152624	1.413499	1.184318	1.230159	1.520603	0.41938	1.18698	1.29057	1.267097	0.581375	0.407165	0.345858	0	0.201257	2.584772
LOC107759306	1.036368	1.180652	1.20119	1.916914	1.757672	3.342667	1.992023	1.734106	2.067599	1.726424	2.119547	3.027952	2.307398	3.062113	2.216041	1.576784	1.96072	1.641895	2.453814	2.118682	5.631727	1.442497	2.094916	1.715085	0.600547	1.081523	1.326979	6.176247	0.831574	0
LOC107827927	0.056444	0	0	0.10227	0	0	0.047342	0	0.052992	0.05504	0	0	0	0.123155	0	0.126186	0.054529	0.252487	0.057275	0.047747	0	0.094275	0.149361	0.058995	0	0	0	0	0	0
LOC107816777	0.854018	2.774055	1.52283	6.268929	6.258673	1.553832	8.154818	11.65643	4.050065	8.733464	15.10914	7.751479	9.165767	8.313555	18.3624	2.137702	0.48657	0.848942	14.73217	15.30092	12.3622	11.96003	10.21784	7.301167	18.90926	42.01719	7.453886	17.2261	16.97331	17.60174
LOC107768335	0	0	0	0	0	0	0	0	0	0	0	0	0	0	0	0	0	0	0	0	0	0	0	0	0	0	0	0	0	0
LOC107784542	73.74723	75.40803	80.12015	23.60028	19.04455	26.67361	18.25996	18.49543	21.95324	7.076371	9.220236	8.593615	8.423689	11.60468	5.856751	61.05767	64.05551	72.20682	13.94083	12.14646	16.00859	22.60998	31.95051	30.53402	1.725194	1.423994	5.278181	6.653452	0.746521	0
LOC107786259	4.41965	6.482508	5.282624	9.259202	8.554869	4.974399	10.91813	13.80698	8.233891	13.03016	17.57749	10.58895	8.802217	7.345439	17.43605	13.79165	5.937626	7.362348	14.82072	16.09402	15.14697	11.13057	11.69527	8.444872	9.560345	20.70693	10.52896	14.81566	7.812931	10.67475
LOC107807067	0	0	0	0	0	0	0.100534	0.102413	0.112532	0	0	0	0	0	0	0.267964	0.231594	0	0	0.101395	0	0	0	0	0	0	0	0	0	0
LOC107781526	0	0	0	0	0	0	0	0	0	0	0	0	0	0	0	0	0	0	0	0	0	0	0	0	0	0	0	0	0	0
LOC107809747	5.350978	6.300406	5.604903	3.041113	2.705656	3.573265	3.902692	3.37932	4.056431	2.301052	3.076263	2.386681	2.552907	2.175523	2.490206	3.789416	5.008952	4.460178	1.585098	1.54634	1.576208	3.080934	3.957694	3.056957	0.504243	0.369963	0.942772	0	0.160009	0
LOC107772540	0.213757	0.415037	0.371629	1.178758	1.51317	0.469622	2.899776	3.493979	1.814886	2.057213	3.740647	1.743065	1.365668	0.922655	4.397426	1.447479	0.233443	0.644389	2.678316	3.176208	2.3394	5.34766	3.508629	2.826733	2.267782	4.474341	1.384047	2.658548	3.132051	9.769026
LOC107776532	0	0	0.116601	0	0	0	0	0	0	0	0	0	0	0	0	0	0	0	0	0	0	0	0	0	0	0	0	0	0	0
LOC107759920	0	0	0	0	0	0	0	0	0	0	0	0	0	0	0	0	0	0	0	0	0	0	0	0	0	0	0	0	0	0
LOC107786464	0.074855	0	0.081337	0.13563	0.281506	0.100598	0.125568	0.159895	0.070277	0.10949	0.24932	0.158099	0.166659	0.285821	0.192073	0.069728	0.054237	0.069759	0.113936	0.237458	0.204807	0.265682	0.132054	0.176036	0.07098	0.052078	0.132709	0	0	0
LOC107811259	0	0.028845	0	0	0	0	0	0	0.029714	0	0	0	0.211395	0	0.064968	0.023585	0	0	0	0	0	0	0	0	0	0	0	0	0	0
LOC107768516	0	0	0	0	0	0	0	0	0	0	0	0	0	0	0	0	0	0	0	0	0	0	0	0	0	0	0	0	0	0
LOC107809891	2.650241	2.483727	1.847385	4.484857	4.955699	3.709509	6.626673	6.964214	8.215632	1.511583	1.910945	2.027771	5.343913	3.955056	3.515646	0.782532	1.376795	1.304818	3.627993	3.257131	5.267376	6.264015	6.130917	5.357136	1.548913	2.191697	2.098098	5.364048	6.379602	1.545928
LOC107819615	19.7228	18.01832	20.91312	26.97443	31.59492	19.84714	35.32234	35.04665	35.15477	48.73092	44.47867	48.96081	61.01457	51.76459	55.32582	26.98084	22.52869	26.95767	41.28355	34.71831	52.28983	30.63452	28.53246	34.4318	42.37248	39.37451	67.11699	42.58633	41.28374	60.3854
LOC107803752	4.212158	6.76371	4.583152	3.790043	4.208941	2.957443	5.984331	5.680026	4.546398	3.632382	5.625844	3.268054	5.039913	4.188874	5.617276	7.79388	5.730237	6.622491	3.547301	4.338858	2.639439	4.905613	5.434091	4.492394	1.630239	1.036624	1.320808	0	2.759024	1.771728
LOC107787167	161.8276	158.7819	146.9504	151.6849	133.039	172.7678	133.969	143.8872	145.3538	145.8213	101.1173	136.0791	125.2583	137.8034	109.4028	135.8943	149.8314	141.3803	157.1027	151.7561	190.2494	111.7445	134.1391	149.2033	148.1863	117.7969	223.8138	200.3976	166.5599	176.5968
LOC107765475	6.944112	7.208853	6.556057	5.082806	3.133343	5.339756	6.055756	6.09041	7.512466	4.2153	4.74023	5.361509	4.402678	5.769562	2.973619	6.614098	7.508297	7.028555	2.473242	3.812421	2.768131	6.797749	6.003474	5.383406	0.988417	0.895835	3.098151	0	1.107001	0
LOC107761327	0.71079	0.946781	1.425865	0.64394	0.628954	0.99932	0.321015	0.560602	0.461991	0.266581	0.613423	0.369536	0.243465	0.059649	0.336709	0.529682	0.633861	0.326107	0.499334	0.138757	0.099731	0.365292	0.626964	0.971508	0	0.152156	0.323114	0	0	0
LOC107780300	0.128172	0.194689	0.037139	0.038706	0.07561	0.091867	0.286676	0.328542	0.160445	0.083323	0.059916	0.036095	0	0.23305	0.219254	0.191028	0.1651	0.031853	0.043353	0.180708	0.116895	0	0.150742	0	0	0	0.100993	0	0	0
LOC107759514	0	0	0	0	0	0.050734	0	0	0	0	0.033089	0	0	0	0	0	0.091177	0	0.047884	0.079837	0.043037	0	0	0	0.059661	0	0	0	0.681554	0
Novel01288	0	0	0	0	0	0	0	0	0	0	0	0	0	0	0	0	0	0	0	0	0	0	0	0	0	0	0	0	0	0
LOC107817862	8.612442	8.17049	8.36224	5.977305	4.367507	7.418372	5.91408	5.594336	8.65316	5.991711	7.769537	7.829178	10.14816	8.681454	5.996436	7.656563	12.69947	8.148211	3.67971	3.408436	3.67471	6.813924	7.685594	8.843797	3.05649	1.471668	6.190866	3.601821	3.394661	3.114153
LOC107813322	55.30749	56.4702	45.57889	14.80508	15.21417	19.8017	27.994	26.42528	24.43829	12.71731	16.834	12.86183	26.60685	22.82265	10.54462	38.04176	57.08294	39.21008	8.966592	9.096092	11.89426	18.271	20.28399	16.52434	2.355526	1.851692	5.347801	0	5.638055	0
LOC107784088	3.932666	9.684804	3.12764	60.18851	66.26583	34.09465	72.70728	72.42253	46.26983	68.12971	66.88617	75.42612	49.27464	63.44234	62.86448	8.417759	3.880122	4.741057	94.75546	96.7593	129.5775	54.2953	50.18793	38.74291	150.5378	117.1245	161.3977	233.3704	130.9214	93.12612
LOC107820981	0	0	0	0	0.044524	0	0	0	0	0	0	0	0	0	0	0	0	0	0	0	0	0	0	0	0	0	0	0	0	0
LOC107773631	0.200689	0.391935	0.23676	0.207787	0.36785	0.493177	0.240466	0.073489	0.201874	0.209675	0.381963	0.157437	0.382988	0.390967	0.500241	0.277742	0.249277	0.341993	0.349106	0.448675	0.117663	0.215486	0.265531	0.299657	0.163113	0.079784	0.237198	0	0.276053	0
LOC107826033	1351.233	875.7066	1174.478	1313.778	1620.959	1115.05	1016.772	715.7223	1321.907	1897.403	1795.044	2721.219	2750.47	2257.278	1710.753	991.1833	1282.407	802.2622	554.3194	522.6369	737.1911	690.4764	827.355	1340.159	364.1737	243.2186	627.5977	164.0058	745.0419	116.2763
LOC107816477	12.29362	9.429211	12.94382	5.660593	4.667217	6.674006	4.560065	4.298669	5.71376	2.452956	3.698477	3.393444	2.348655	3.098422	1.873936	7.226179	8.584131	9.044422	1.976193	2.814402	3.515311	3.693311	6.477643	6.997139	0	0.225818	0.863173	0	0.390666	0
LOC107832112	1.67898	1.187906	1.171442	1.721212	1.583414	2.018872	1.48235	2.05747	1.908153	1.572591	1.688499	1.978363	1.91825	1.903988	1.020351	1.168864	1.301897	1.63057	1.658878	1.756689	2.317035	1.494408	1.753782	1.015977	1.424471	1.414003	1.67109	1.579879	2.12716	0
LOC107764943	1.490015	1.566883	1.793407	0.415347	0.304261	1.109041	0.384535	0.097931	0.430428	0.223531	0.080369	0.193662	0.127593	0.250082	0	1.110361	1.218021	0.854515	0.814135	1.551323	1.88158	0.095719	0	0.119797	0	0.159481	0	0	0	0
LOC107765090	4.367423	3.339234	4.484466	3.000691	2.360249	1.985351	2.925599	3.130574	4.430557	0.514485	1.089324	0.643844	0.717861	0.639549	0.541523	3.232766	3.822854	3.234246	1.130247	0.719071	1.443562	4.112447	5.455349	7.015744	0.074117	0.122355	0.242507	2.096191	0.141116	0
LOC107809954	10.69692	10.89317	11.69966	17.56186	16.57545	14.20015	12.13111	12.31505	11.97585	16.35636	14.29709	16.41944	16.49225	14.24485	14.1748	11.33954	10.96485	10.4087	18.95715	16.73827	17.86268	11.49164	12.62482	12.49268	18.54016	20.89348	16.91656	25.14023	12.81425	9.315588
LOC107762383	13.31465	13.05578	10.61918	10.27096	10.14858	12.42513	11.60985	10.58794	11.13896	13.75476	12.32507	13.58741	14.72422	11.36163	8.839921	12.70552	11.33479	14.05455	13.0203	13.45646	17.31306	8.000108	10.38779	9.277648	5.500143	7.765178	7.375025	15.71264	7.192906	5.434093
LOC107779622	0	0	0	0	0	0	0	0	0	0	0	0	0	0	0	0	0	0	0	0	0	0	0	0	0	0	0	0	0	0
LOC107793145	4.858413	4.935201	4.729233	6.854381	6.58291	6.964517	9.020094	7.502356	7.269559	5.601185	6.36986	5.451318	8.507045	6.680573	7.090239	4.902753	5.035879	4.20698	4.698889	4.045666	4.03863	7.649866	8.057522	6.955818	3.615123	3.626526	3.020664	1.80961	4.507473	9.387593
LOC107786746	0.024408	0	0	0.044225	0.151185	0.078725	0.020472	0.04171	0.045831	0.047602	0.017115	0	0	0	0	0.34559	0.282963	0.436734	0.569656	0.330361	0.779121	0.407677	0.49518	1.14801	0.246874	0.339621	0.230787	0	0.587546	0
LOC107818925	0.032155	0.087915	0.083854	2.184779	4.495489	0.760542	4.449952	10.60486	5.10179	3.825279	1.555729	1.983049	8.733954	15.2243	27.98616	0.047923	0	0.071918	12.52923	9.084979	4.868031	20.62318	5.218689	3.024713	96.10399	121.471	6.156663	96.57921	71.05478	381.7275
LOC107801481	0.323332	1.105039	0.983729	0.805536	3.50479	0.782148	1.76275	2.210108	1.366012	1.734092	1.587044	0.614609	1.259781	1.587326	2.240048	2.288999	0.312366	1.145024	11.89344	6.496017	25.9498	1.41762	2.495501	3.46395	13.08127	11.58474	12.13333	40.4649	8.950649	89.96443
107817330	0	0	0	0	0	0	0	0	0	0	0	0	0	0	0	0	0	0	0	0	0	0	0	0	0	0	0	0	0	0
LOC107827201	0	0	0	0	0	0.052729	0	0	0	0	0	0	0	0	0	0	0	0	0	0	0.04473	0	0	0	0	0	0	0	0	0
LOC107769281	29.44361	26.10503	28.14491	14.01374	10.90288	16.57325	9.797721	10.29996	12.87	5.773456	5.311078	6.173633	5.640976	6.110075	5.146179	19.11924	25.55928	23.82052	9.14723	10.60364	11.91848	10.55729	17.12595	16.05624	3.304453	1.855464	5.106519	0	1.79758	3.298085
LOC107824476	0	0	0	0	0	0	0	0	0	0	0	0	0	0	0	0	0	0	0	0	0	0	0	0	0	0	0	0	0	0
LOC107824399	3.250735	5.81349	5.11842	5.890002	6.512748	5.605088	7.819486	7.022399	4.43394	4.904338	7.483369	2.072687	5.871955	3.680218	10.32416	7.495887	4.562588	4.847121	6.65948	7.78273	3.915674	10.65421	5.572422	3.33356	4.652736	7.168782	3.407151	13.15888	9.449174	11.37723
LOC107771845	10.62474	11.64758	11.68991	12.89157	12.22022	13.65825	10.57532	11.01754	11.99862	14.54557	16.42493	12.69865	16.09574	12.5067	11.10072	12.66526	11.92291	13.68077	11.2845	15.20019	7.671926	11.11912	11.72727	11.16478	9.091925	8.705571	14.15828	6.820636	6.79568	4.717727
LOC107776765	0.560511	0.383128	0.414154	0.482405	0.247989	0.391702	0.423112	0.431024	0.526233	0.491912	0.451985	0.213091	0.623969	0.733788	0.287647	0.730964	0.460276	0.710404	0.255946	0.284492	0.383397	0.725551	0.543849	0.703015	0.141731	0.077991	0.198744	0	0.26985	0
LOC107778755	0	0	0	0	0	0	0	0	0	0	0	0	0	0	0	0	0	0	0	0	0	0	0	0	0	0	0	0	0	0
LOC107817915	0.252346	0.147846	0.078342	0.261271	0.159494	0.116273	0.468661	0.261813	0.152301	0.052729	0.379168	0	0.180588	0.196641	0.1295	0.255208	0.20896	0.268763	0.310934	0.243962	0.032878	0.346217	0.349777	0.244912	0.159521	0.05016	0.149126	0	0.086777	0
LOC107820030	4.456347	6.22105	6.333368	4.389681	3.724199	6.616306	5.102298	4.412009	5.64483	3.27637	4.166391	4.123403	4.921496	5.015994	3.666358	5.27123	5.671938	5.985586	3.804216	2.8722	2.451461	4.135124	4.648318	5.951598	3.443108	2.017682	4.347401	2.5293	4.08656	4.373693
LOC107827076	2.476407	6.360466	4.67503	4.532322	6.905877	3.684387	11.56025	14.34116	8.078649	9.878767	18.9081	10.58748	3.703541	5.048516	12.42617	11.5199	3.697372	6.042327	9.924634	14.70641	12.09109	13.26516	10.37017	10.74553	6.799497	16.04531	8.041622	28.62192	8.189058	12.37333
LOC107800244	0	0	0	0	0	0	0	0	0	0	0	0	0	0	0	0	0	0	0	0	0	0	0	0	0	0	0	0	0	0

LOC107759151	2.086738	3.105478	2.112456	2.823753	2.898277	3.464643	2.104715	2.437472	3.025479	8.860538	7.649502	8.100571	4.469542	4.927675	2.548436	2.184929	1.91389	2.205623	2.43912	5.273365	2.336767	2.823598	2.143531	2.181058	14.09304	11.17313	16.85864	3.778007	7.375764	13.06594
LOC107803453	5.144618	6.5714	5.49085	8.025871	10.72163	7.282251	10.62926	9.60605	7.962963	7.941419	9.540832	5.168592	10.96765	10.42326	16.69715	7.282693	6.083137	6.634432	10.54159	10.33388	6.249933	11.37967	7.901892	5.626288	12.07953	15.91975	6.737879	7.102566	16.83078	20.87912
LOC107767387	8.14385	9.868039	9.009987	10.75946	10.2361	10.28124	11.0221	9.198697	11.26598	6.768138	6.943415	6.671661	6.559003	6.865326	7.250473	8.942294	8.910703	8.739404	8.357723	6.967422	9.002822	8.449903	10.58465	13.63846	5.265154	4.592721	5.578343	2.206062	4.15836	5.722119
LOC107775331	0	0	0	0	0	0	0	0	0	0	0	0	0	0	0	0.048549	0	0	0	0	0	0.108815	0	0	0	0	0	0	0	0
LOC107770249	0	0	0	0	0	0	0	0	0	0	0	0	0	0	0	0	0	0	0	0	0	0	0	0	0	0	0	0	0	0
LOC107777063	0	0	0	0	0	0	0	0	0	0	0	0	0	0	0	0	0	0	0	0	0	0	0	0	0	0	0	0	0	0
LOC107784622	0.503652	0.321311	0.437814	0.27377	0.49023	0.487338	0.084487	0.172133	0.141855	0.392899	0.600373	0.297849	0.224269	0.439567	0.361854	0.52545	0.97314	0.413043	0.306642	0.213027	0.505273	0.042061	0.222127	0.31585	0.063677	0.140159	0.178583	0	0.727427	0
Novel04485	1.316423	1.894353	2.409133	1.945841	1.042241	3.128592	1.046028	1.598382	1.626205	0.472934	0.631579	0.40974	1.388331	1.511739	0.426676	2.013631	2.075001	1.394691	0.70306	0.293053	1.326978	0.405034	1.405629	2.896683	0	0	0	0	0	0
LOC107768559	96.848	86.62402	107.8554	41.22515	34.56867	60.77289	30.93627	30.48913	46.20535	14.79101	19.74169	16.68665	14.69895	17.91695	15.84834	60.70516	86.81621	82.90273	20.98372	23.21642	27.76702	34.03807	46.05803	57.02845	3.863063	1.822065	12.5752	0	1.576093	6.747332
LOC107766162	2.95864	2.890579	2.571883	2.873365	3.141599	2.54472	3.394745	3.195319	3.622123	3.300494	3.153392	2.319574	3.92597	2.401432	2.9152	2.486994	2.035106	2.558718	1.729281	1.641841	1.791691	3.498706	3.319559	3.290281	2.364072	0.922148	2.07015	1.692677	3.418547	0
LOC107819721	5.122897	5.413204	5.389048	6.120851	6.011253	7.54321	4.763837	5.074947	4.914158	4.307689	4.945753	5.300189	5.413603	5.264838	4.000653	4.149553	4.626378	4.345188	4.256629	4.396424	4.062755	4.557282	5.108134	5.082848	2.158966	1.446289	2.764171	2.654779	3.574413	0
Novel00340	0.378449	0.247904	0.503748	0.846426	1.098815	0.839189	1.160573	1.040807	0.71061	1.245496	1.451236	0.779329	1.593022	1.032174	1.55372	0.564042	0.342763	0.520214	0.972062	0.770342	0.70109	1.195075	0.792816	0.865277	0.807427	0.970891	0.489231	0	0.569372	0
LOC107791038	0	0.015107	0	0.015017	0	0	0.194641	0.212443	0.031124	0.032327	0.023246	0	0.055357	0.072334	0.153117	0.012352	0	0	0.0841	0.028044	0.030235	0.138429	0.116968	0.03465	0	0	0	0	0	0
LOC107765293	0	0	0	0	0	0	0	0	0	0	0	0	0	0	0	0	0	0	0	0	0	0	0	0	0	0	0	0	0	0
Novel01777	0	0	0	0	0	0.005224	0.004075	0	0	0	0	0	0.005409	0	0	0	0	0	0	0	0	0	0	0	0	0	0	0	0	0
LOC107774775	0	0	0	0.103686	0.033758	0.041016	0.063996	0.130385	0.214901	0.260407	0.321008	0.418991	0.084938	0.249718	0.352404	0	0	0	0.232271	0.032272	0	0.09558	0	0.199371	0.192932	0.212331	1.442882	0	0	0
LOC107774214	0	0	0	0	0	0	0	0	0	0	0	0	0	0	0	0	0	0	0	0	0	0	0	0	0	0	0	0	0	0
LOC107794169	0	0.123566	0.117858	0.081887	0.159962	0	0.075812	0.115845	0.21215	0.132209	0.44366	0.076362	0.050311	0.34513	0.092772	0.134714	0	0.101082	0	0.076462	0	0	0.039864	0.14171	0	0	0	0	0	0
LOC107800028	0.08517	0.388111	0	0.3858	0.376822	0.091569	0.857232	1.091578	0.559732	0.581363	0.358329	0.647588	0.66369	0.83625	0.786749	0.126938	0.082282	0.063498	0.3457	0.720483	0.699091	0.71128	0.676133	0.53412	0.430725	0	0.503322	0	0.41004	0
LOC107787430	10.86588	12.46155	8.20265	10.47979	12.05306	9.865544	12.68938	10.50569	8.590622	13.10509	16.8059	4.040866	13.25356	14.23631	13.71376	16.54321	7.58421	12.09332	12.08105	13.78768	3.698417	15.56538	10.61624	6.439262	14.39508	13.67227	6.667087	7.436017	13.39092	8.036525
LOC107776133	4.445317	6.101444	4.190118	6.065109	5.639609	4.145864	6.783136	6.772693	6.235048	4.857004	6.008789	5.429678	4.888968	5.375485	5.112257	4.070974	4.087584	4.751645	3.152126	4.757174	4.640354	4.159678	4.91314	5.877779	3.047114	2.086619	2.278847	3.830157	4.125559	0
LOC107759319	0	0	0	0	0	0	0	0	0	0	0	0	0	0	0	0	0	0	0	0	0	0	0	0	0	0	0	0	0	0
LOC107791965	2.032647	2.018758	2.763671	0.613825	0.714836	1.31681	0.174858	0.11133	0.611647	0.304937	0.36546	0.154111	0.145049	0.170578	0.053494	0.970982	1.560896	1.495996	0.661087	0.308625	0.475336	0.108815	0.436739	0.898836	0.032947	0	0.184802	0	0	0
LOC107808685	3.842155	1.196767	1.183763	0.308425	0.301247	0.67975	0.326337	0.623324	0.730569	0.331976	0.818467	1.479171	0.703833	1.53869	1.19802	1.30474	1.315591	1.70419	0.049351	0	0.354847	1.218488	0.257395	1.118329	0	0	0	0	0.468291	0
LOC107781033	5.234099	4.770236	4.145456	4.741829	4.94024	6.627705	4.162431	3.610852	5.496403	4.83926	5.573196	5.863128	8.977391	7.444337	5.53131	5.229501	3.895439	3.844432	3.658828	4.099711	3.748149	4.403512	3.727656	5.875914	2.843071	1.834204	3.024405	0	8.212947	0
LOC107782111	12.98527	11.35469	11.7073	9.73709	7.026114	12.87596	5.261677	4.01068	5.1071	3.593348	6.58287	3.039074	5.664947	4.642331	4.007322	11.40928	13.22296	12.16675	6.143122	5.714983	3.760884	5.348896	7.313447	8.711884	0.998351	0.305204	2.64434	0	1.056008	0
LOC107781897	4.037607	4.716939	3.360741	6.030538	4.717672	5.849349	4.288713	4.40887	3.83459	4.636443	5.378134	4.925655	5.58803	5.79941	5.680122	9.340149	5.527201	6.57829	4.176179	4.549654	3.668148	3.306816	2.805147	3.209887	1.951241	2.819863	1.87937	0	4.953434	7.711237
107767201	0	0	0	0	0	0	0	0	0	0	0	0	0	0	0	0	0	0	0	0	0	0	0	0	0	0	0	0	0	0
LOC107785362	0	0	0	0	0	0	0	0	0	0	0	0	0	0	0	0	0	0	0	0	0	0	0	0	0	0	0	0	0	0
LOC107762063	1.011843	0.814584	0.791617	0.886123	0.865501	1.015332	0.834532	0.720455	0.807469	0.60845	0.934182	0.455911	0.826025	0.699119	0.709661	1.043071	0.586516	0.968111	0.479149	0.741823	0.430649	1.253442	1.130516	0.581668	0.234534	0.117326	0.259115	0	0.243569	0
LOC107778100	0	0.058075	0	0	0.3947	0.068509	0.16034	0.054446	0.059825	0.062137	0.089364	0.053834	0	0.278072	0.130806	0.047486	0	0	0	0.107809	0.116232	0.053216	0.056207	0.133205	0.322258	0.265995	0	9.114096	0	0
LOC107830817	0.099298	0.045249	0.043159	0	0.043933	0	0	0	0.093225	0	0	15.39378	0.05527	0.108329	0	0.036999	0.287791	0.037015	0	0	0	0	0	0	0	0	0	0	0	0
Novel01327	5.121177	5.568033	6.872848	5.209294	5.00856	5.409297	5.87785	5.757464	5.735844	3.241586	7.181945	2.884342	5.300898	2.842485	5.717309	7.632615	9.894976	8.372928	3.737917	5.396204	2.540151	9.754171	7.845693	8.263745	2.158242	2.000209	0.849519	19.27561	1.730187	0
LOC107786935	0	0.107679	0.034235	0.356793	0.243942	0.677469	1.255233	1.076805	0.554621	0.307229	0.662774	0.16636	0.613788	0.171861	0.121266	0.176091	0.342428	0.029362	0.359671	0.632995	0.107755	0.49335	0.312648	0.617451	0.248962	0.109598	0.325835	0	0	0
LOC107814910	2.872091	3.926335	2.652688	1.886428	1.175405	1.814111	2.710065	2.116569	1.75269	1.225285	2.668433	0.697596	1.198969	1.527489	3.463707	4.227064	2.427846	2.1145	0.69217	1.518495	0.26194	2.788324	2.755045	0.600382	0	0	0.212161	0	0.345682	0
LOC107777887	10.74314	8.167661	8.661358	77.25677	115.6017	68.40345	48.97544	62.6851	41.44198	124.0826	49.57011	121.0931	78.01503	92.00082	89.01156	6.927298	9.894791	7.386969	105.512	85.65281	132.1451	66.70799	39.08302	42.99497	235.8304	170.4725	158.0941	358.2682	269.8619	216.8324
LOC107828622	38.66463	26.29126	27.32573	36.69773	40.98662	29.49089	29.27887	27.40392	27.88217	49.21074	32.30537	46.198	75.47145	54.01205	36.30151	20.65234	28.93082	22.22069	19.707	14.15032	30.60849	20.6288	18.47471	18.9282	36.7861	10.79925	43.1015	38.01651	104.76	63.54727
LOC107821489	0.123102	0	0	0	0.054464	0.066175	0	0	0.057787	0.12004	0.086319	0.052	0	0	0	0	0.059463	0.091778	0	0	0.056135	0	0.054292	0	0	0	0	0	0	0
LOC107795971	0.38555	0.301184	0.646362	0.32434	0.463003	0.47373	0.230984	0.447076	0.620523	0.161126	0.270348	0.093064	0.061314	0.090132	0.113062	0.574625	0.425685	0.451698	0.586845	1.048336	0.326512	0.804958	0.510121	0.63325	0.069636	0.306551	0.227845	0	0	0
LOC107771394	2.262982	4.158394	2.159081	3.650262	2.295475	1.463746	4.104749	3.568428	1.450938	6.673902	15.76484	4.196701	3.522761	3.050878	14.91792	6.498721	1.955166	2.153516	5.675453	8.108655	4.698259	5.500614	5.04705	2.653716	1.977181	4.710362	2.652918	2.631467	1.151483	2.275181
LOC107766301	0	0	0	0	0	0	0	0	0	0	0	0	0	0	0	0	0	0	0	0	0	0	0	0	0	0	0	0	0	0
LOC107811166	9.511205	10.69255	10.12757	9.591909	11.58726	10.75311	7.348861	7.067143	8.021233	10.43063	8.905091	7.252519	10.84588	8.978882	8.629196	12.34773	10.5972	12.74959	12.29229	12.69119	7.804456	8.659979	7.692389	8.616577	12.93813	14.08735	9.035057	11.69374	17.77819	17.69332
LOC107765049	23.64138	25.53258	23.19052	32.78182	28.75625	30.23657	30.28932	27.79794	29.69591	27.31855	25.80389	28.12697	31.46882	30.20849	26.67651	26.99208	26.19216	26.43848	27.95123	26.35952	27.10003	24.90584	26.84778	30.15069	19.78949	19.61644	21.57617	18.0961	24.01665	13.41085
LOC107802658	4.209205	4.646294	4.65894	5.755517	4.534273	6.085411	4.604923	3.138525	2.270437	2.60037	5.261364	2.407514	2.037281	1.882449	3.126116	5.201864	4.015931	4.716957	3.72736	4.821317	3.111627	7.336105	5.1541	4.850336	1.454382	3.255799	0.957908	1.869675	1.636273	4.849593
LOC107764825	1.667028	1.119477	1.906726	3.258944	2.251462	3.207199	3.385135	3.298503	3.130158	2.395566	2.091753	2.075465	1.855758	1.435772	1.530887	1.69995	2.288589	2.354854	0.979338	1.484411	1.440338	3.590355	1.934774	2.109193	0.554639	0.85457	0.414798	0	2.53442	0
LOC107779224	5.466999	2.962565	4.945009	1.271671	0.784469	2.859418	0.74358	1.262474	0.693603	2.521426	0.932464	1.497948	1.891576	1.611953	0.758267	1.651627	7.208644	2.258258	0.8996	1.437408	0.80854	0.55528	2.932452	2.239312	0.373619	0.205593	2.008321	0	0.355677	0
LOC107790629	0	0	0	0	0	0.074787	0	0	0	0	0	0	0	0	0	0	0.067202	0	0	0	0	0	0	0	0	0	0	0	0	0
LOC107816188	0.595902	0.760327	0.414404	0.269928	0.474564	0.640667	0.249904	0.356408	0.447567	0.058108	0.083569	0.352404	0.132673	0.06501	0	0.222033	0.345415	0.266562	0.30234	0.352864	0.326083	0.248827	0.841	0.435985	0	0.082915	0.211292	0	0.286888	0
LOC107767409	0.637581	0.446982	0.170534	0.622048	0.86796	0.474562	0.699311	0.963818	0.460453	0.382598	0.619023	0.372909	0.327584	0.642065	0.402705	0.621319	0.71072	0.255954	0.298603	0.165954	0.04473	0.942046	0.908474	0.820184	0.062007	0.136484	0.231868	0	0.236119	0
LOC107814242	0.718567	1.1719	0.526009	1.944391	1.857309	1.199482	2.529725	2.213499	1.491271	2.977948	4.388883	2.444237	2.189252	2.011377	3.765221	1.698026	0.849477	1.219473	2.782292	3.135269	1.86256	2.337209	1.642938	1.620679	1.936512	3.880934	1.631528	1.352308	3.095287	2.338426
LOC107820687	1.265212	3.367006	1.275793	2.246587	2.91081	2.230822	1.52811	2.291786	1.948052	2.71423	2.874422	1.111641	1.521127	2.926201	1.869953	2.300523	2.982414	5.169114	2.773115	3.253657	1.015427	2.747189	4.776481	4.866428	2.687348	1.971704	1.435563	0	6.091179	3.129198
LOC107794415	18.17636	15.35635	16.65377	19.80831	18.76033	21.53878	16.4026	17.2988	19.50615	13.94652	13.35928	12.80032	18.40276	16.38468	12.25558	15.58175	14.66315	16.7165	15.23996	14.725	14.37493	17.88307	19.12228	19.52488	11.00378	7.531942	9.53406	18.97616	11.11409	18.04758
LOC107804319	2.445348	3.708146	4.037023	2.38291	2.382003	2.452305	3.44709	2.4932	3.337592	1.963725	2.867405	1.649246	2.607817	2.219392	2.910568	3.812998	3.573396	3.738143	1.397074	1.6514	1.274399	4.393248	3.715769	4.166724	0.415686	0.400297	0.874348	0	0.494654	0
LOC107786319	0	0	0	0	0	0	0	0	0	0	0	0	0	0	0	0	0	0	0	0	0	0	0	0	0	0	0	0	0	0
LOC107788269	0	0	0.0372	0.116307	0	0.092017	0	0	0	0	0	0	0	0	0	0	0	0	0.043424	0.036201	0.039029	0	0	0	0	0	0	0	0	0
LOC107802174	12.37317	32.7559	16.28726	0	0.208545	0.063346	0	0.050343	0	0	0.041315	0.049777	0.06559	0	0.060474	23.62205	12.57966	18.3616	0	0.049842	0	0.098411	0.311827	0	0	0	0	0	0	0
LOC107780229	0	0	0	0	0	0	0	0	0	0	0	0	0	0	0	0	0	0	0	0	0	0	0	0	0	0	0	0	0	0
LOC107808790	18.55069	22.87125	18.00872	5.803805	5.36287	7.160112	5.489229	6.084118	6.144349	5.123418	5.768655	5.275961	6.438971	6.435899	4.990586	18.94145	17.85473	17.28361	4.209083	4.542083	4.875901	5.946681	5.833741	5.756537	2.301668	2.180386	4.194428	0	2.77359	1.424866
Novel01490	2.544766	1.111303	2.672967	0.04803	0	0.170996	0.755935	0.951264	1.74208	0.155092	0	0.134368	0.885271	1.156756	0.163242	0.987689	1.792625	1.5415	0.053797	0.089696	0	0.61985	0.327345	0.277061	0	0	0.125321	0	0	0
LOC107818317	1.259586	1.147957	1.218886	1.916222	1.787509	1.916333	3.149474	3.127143	1.740364	1.506351	2.499686	1.044055	2.460444	1.892692	1.512337	1.27514	1.653101	1.665529	2.339239	2.311984	1.820682	1.508404	1.991483	2.161078	2.914575	2.678535	1.685354	0	2.402755	11.75579
LOC107771164	2.936399	3.0075	2.844269	2.660229	2.425098	2.495524	2.087588	2.150519	2.861842	1.118074	1.80408	1.488451	1.400931	2.288191	1.980523	2.77173	2.944877	2.960646	2.412101	1.537709	1.989405	2.358844	2.442092	3.302961	1.414286	1.789963	1.289075	0	0.807821	0
LOC107760034	0.098273	0.089564	0.170854	0	0.086959	0.052828	0	0.083968	0.046132	0.095829	0	0	0	0.053606	0	0.183084	0	0.036634	0.299163	0	0.089627	0.082071	0.086684	0	0	0.06837	0.058076	0	0.473123	0
LOC107807412	8.134059	8.037746	8.262175	13.9553	10.45358	10.28271	13.44485	12.44883	19.60903	4.474312	5.599145	5.172788	7.34678	7.411134	5.32107	7.715658	7.109786	7.696978	7.30151	5.70884	8.777079	15.75072	13.28516	12.955	0.904081	1.388833	1.1445	3.196155	1.290997	0
LOC107774164	0.087397	0.106202	0.12662	0.3431	0.103112	0.219245	0.097738	0.149348	0.218805	0.284076	0.306413	0.344564	0.194582	0.444946	0.328906	0.369059	0.197009	0.173755	0.413859	0.246439	0.106276	0.243291	0.102786	0.182694	0.294656	0.243212	0.137728	0	0.140253	0
LOC107814457	0.491178	0.321389	0.383179	0.262426	0.211741	0.284349	0.232395	0.225979	0.342899	0.135092	0.203116	0.14896	0.154222	0.178617	0.168044	0.497421	0.279845	0.488259	0.383395	0.277002	0.172293	0.347089	0.399925	0.302762	0.063692	0.157717	0.089313	0	0	0
LOC107829825	0	0	0	0.211846	0.206915	0	0	0	0	0.228022	0.327935	0.197553	0	0.255106	0	0.174257	0	0	0.474566	0.197811	0	0	0.412522	0.244408	0.591285	0	0.276378	0	0	0
107780438	0	0	0	0	0	0	0	0	0	0	0	0	0	0	0	0	0	0	0	0	0	0	0	0	0	0	0	0	0	0
107806250	0	0	0	0	0	0	0	0	0	0	0	0	0	0	0	0	0	0	0	0	0	0	0	0	0	0	0	0	0	0
LOC107785677	0.215999	0.295285	0.187763	0.146763	0.38226	0.232225	0.045292	0.092278	0.456276	0.473909	0.340782	0.319343	1.202266	1.060399	0.221695	0.080481	0.365178	0.080518	0.054795	0.09136	0.098497	0.045097	0.142894	0.282203	0	0.075137	0.063823	0	0.519948	0
LOC107814399	0	0	0	0	0	0	0	0	0	0	0	0	0	0	0	0	0	0	0	0	0	0	0	0	0	0	0	0	0	0
LOC107806526	0	0	0	0.023942	0	0	0.022166	0	0	0	0	0	0	0	0.027125	0	0	0	0.026817	0	0	0	0	0	0	0	0	0	0	0
Novel03411	0.195593	0.124781	0.102015	0.106318	0.121151	0.252343	0.098431	0.050136	0.257084	0.133509	0.19201	0.181766	0.021774	0.106691	0.160601	0.072878	0.207855	0.087494	0.238169	0.19855	0.214061	0.245017	0.189778	0.12266	0.049458	0.027215	0.161822	0	0.094165	0
Novel05308	0.693942	0.632442	0.513862	0.465686	1.046151	0.525014	0.582039	0.834484	0.410206	0.751868	1.76615	0.412554	1.115842	0.897252	2.031211	1.034253	0.446937	0.632333	0.834565	0.913153	0.210962	0.837097	0.430739	0.268633	1.49475	1.931139	0.789806	0	1.856046	1.589166
800420	16.86806	4.970268	11.35559	12.40912	6.060168	6.681399	3.616774	7.152081	9.049415	5.688951	5.424823	11.67907	11.29489	16.05006	1.041378	8.695105	11.2724	8.320866	7.464356	9.226712	9.83185	9.744361	7.047826	11.53273	6.413941	1.588241	17.08859	108.8395	15.26482	0
LOC107795423	0.083142	0	0	0.075323	0	0	0	0.071039	0	0	0	0	0.092555	0	0	0	0.080323	0.061986	0	0	0	0	0	0	0	0	0	0	0	0
LOC107786817	0	0	0	0	0	0	0	0	0	0	0	0	0	0	0	0	0	0	0	0	0	0	0	0	0	0	0	0	0	0
LOC107806387	5.443697	8.85527	7.235697	2.093111	1.120219	2.552023	0.982192	1.108726	0.653705	0.617243	0.443852	0.187168	0.176163	0.863199	0.487261	8.726514	7.796876	6.890024	1.637897	2.061538	0.692755	0.898662	1.312093	0.396959	0.480174	0.396341	0.523699	0	0.152371	0
LOC107786376	0	0	0	0	0	0	0	0	0	0	0	0	0	0	0	0	0	0	0	0	0	0	0	0	0	0	0	0	0	0
LOC107783746	0.239724	0.10924	0.243118	1.375461	4.489955	0.386599	2.077693	4.71102	1.125319	1.246728	2.689517	1.721469	1.245367	4.053675	7.422402	0.922988	0	0.148937	15.77108	12.94478	22.84711	6.106101	7.295118	1.545119	16.06347	43.97418	4.816692	34.28736	8.655886	27.17461
LOC107792461	0.131773	0.140111	0.171821	0	0.019434	0	0	0.03753	0	0	0	0	0	0	0	0.229127	0.106086	0.032747	0	0	0	0	0	0	0	0	0	0	0	2.715907
LOC107760708	0	0	0	0	0	0	0	0	0	0	0	0.041727	0	0	0	0	0	0	0	0.022523	0	0	0.025812	0	0.034362	0	0	0	0	0
LOC107789875	7.083073	7.491391	8.108201	10.64203	7.995664	10.02821	7.627794	7.521378	8.56551	6.253152	7.358008	5.910101	7.398249	7.34569	7.449375	6.733652	7.54684	7.410409	6.714168	6.854808	6.353546	7.156733	7.841812	9.718638	3.316725	2.190129	3.892977	2.084526	5.192247	1.802292
LOC107831962	1.536965	1.400754	1.229167	2.116467	2.828816	1.784619	5.10491	4.727608	3.174535	2.278077	3.836686	2.233369	2.874431	1.274332	4.038396	1.924185	1.247263	1.650056	2.620138	2.236286	3.083823	5.647662	3.958657	2.377524	1.243642	2.138574	2.034557	0	0.88794	0
LOC107820420	0	0	0	0	0	0	0	0	0	0	0	0	0	0	0	0	0	0	0	0	0	0	0	0	0	0	0	0	0	0
Novel01052	4.02479	4.768529	4.298359	3.28163	3.00175	3.585345	2.218359	2.94762	2.483113	2.523007	2.822185	2.331603	1.9842	2.195424	2.183494	3.256359	2.832893	2.486254	1.16688	2.091456	2.045091	2.496897	3.397982	3.425463	0.872324	0.160006	1.427093	0	2.214488	0
Novel04958	1.523052	0.54316	0.805887	0.539925	0	0.284777	0.277706	0.282899	0.49736	0.322863	0.325033	0.503498	0.368583	0.361212	0.135932	0.345429	0.447816	0.394957	0.201585	0	0.241574	0.110604	0.11682	0	0	0	0	0	0	0
LOC107774068	3.000114	3.483616	3.496565	3.12061	2.320403	3.775017	3.821085	3.436826	2.649727	2.990501	3.132148	2.684777	3.438724	2.569893	3.398562	3.80895	3.370688	3.910104	2.006988	2.650685	2.249727	2.988009	3.234365	3.995137	0.758611	0.587514	1.392091	0	1.390867	1.374086
LOC107772142	0	0	0	0	0	0	0	0	0	0	0	0	0	0	0	0	0	0	0	0	0	0	0	0	0.096396	0	0	0	0	0
LOC107824061	35.29803	59.91626	31.99885	16.95988	13.66489	7.725475	70.41994	62.90431	32.90264	9.281118	30.45257	2.396311	19.36646	14.71572	25.61903	35.36967	20.09516	23.68456	2.62239	3.465859	2.816835	39.1114	43.92287	8.49868	0.398459	0.877047	0.819489	4.507694	0.606919	0
LOC107828715	2.606692	2.779096	1.667392	20.78593	44.08601	14.83217	7.982222	13.17427	8.219159	55.46518	21.88204	59.39724	45.85401	49.4441	34.30113	1.007906	1.781805	1.246709	59.1652	36.7375	52.61563	21.66648	9.609234	16.32138	136.3032	97.79273	97.60061	91.44904	67.00983	56.25945
LOC107809288	1.512773	1.771254	1.799022	0.923181	1.310713	1.355347	0.67847	0.843751	1.114508	0.870745	0.537745	0.550264	0.514568	0.68765	0.474427	1.479608	1.441168	1.770077	1.471097	1.404117	1.446742	0.877331	1.399231	1.712912	0.610971	0.540846	1.055402	0	1.112685	0
LOC107790813	1.358108	1.490924	1.395225	1.565935	0.873995	1.99109	1.449768	1.239523	1.043224	0.782557	1.125453	0.59976	0.927735	0.942855	1.077124	2.484157	1.043673	2.117102	0.970944	1.30553	0.450406	2.139498	1.470201	1.5808	0.429265	0.472427	0.364812	0	0.891602	0
LOC107761659	0.480548	1.386875	0.208865	0.072559	0	0	0	0	0	0	0	0	0	0	0	0.119368	0.15475	0.358269	0	0	0	0	0.070646	0	0	0	0	0	0	0
107817006	0	0	0	0	0	0	0	0	0	0	0	0	0	0	0	0	0	0	0	0	0	0	0	0	0	0	0	0	0	0
LOC107815527	7.21236	7.230494	6.818137	8.929844	9.599547	10.50044	8.670687	7.959813	6.291649	9.553106	9.50353	9.241356	12.17716	10.85174	9.870091	7.81565	8.332263	7.550373	10.76445	11.43967	10.57928	7.252973	7.475089	6.784521	15.53964	13.50607	13.39046	12.89464	12.73172	9.290647
LOC107830594	0	0	0	0	0	0	0	0	0	0.039966	0	0	0	0	0	0	0	0	0	0	0	0	0	0	0	0	0	0	0	0
LOC107785849	0	0	0	0	0	0	0.032115	0.098146	0	0	0.026848	0.032348	0.127873	0.125315	0	0	0	0	0	0.03492	0.031976	0	0	0.048409	0.106554	0.135765	0	0.184338	0	0
LOC107799516	6.209778	7.11278	6.360215	4.181067	3.50274	4.901311	6.986506	6.411847	5.477747	1.994047	2.828322	1.68005	2.401732	1.821559	3.138635	5.70403	4.821084	4.769522	1.503925	1.888557	2.036094	6.56469	5.676013	4.372726	0.640418	0.469874	0.576513	0	0.812884	1.16
LOC107763246	4.265264	5.558258	3.808365	6.346937	7.104351	5.93441	9.566875	7.931837	5.671416	5.815312	8.322831	4.663241	7.004061	7.011373	10.75619	7.24866	5.108803	6.446204	5.561896	6.563187	3.221125	8.413491	5.839141	3.913404	5.294987	7.304374	3.239142	1.380211	4.924564	16.70673
107764866	0	0	0	0	0	0	0	0	0	0	0	0	0	0	0	0	0	0	0	0	0	0	0	0	0	0	0	0	0	0
LOC107786390	0	0	0	0	0	0	0	0	0	0	0	0	0	0	0	0	0	0	0	0	0	0	0	0	0	0	0	0	0	0
LOC107807358	0	0	0	0	0	0	0	0	0	0	0	0	0	0	0	0	0	0	0	0	0	0	0	0	0	0	0	0	0	0
LOC107810202	2.101464	3.230155	5.507538	1.676498	2.19256	1.551182	1.946739	2.38513	1.64903	2.232702	2.969088	1.192416	1.326812	1.266059	2.961692	9.957038	1.757478	6.617681	5.474272	6.182128	4.605489	2.278864	1.604635	2.393146	2.418958	3.404098	1.705269	17.9444	6.342121	9.696765
LOC107782129	0.161766	0.088458	0.028124	0.02931	0.200397	0	0.054272	0.138217	0.091124	0.157742	0.204174	0.054666	0.216096	0.070591	0.265651	0.02411	0	0.024121	0.065659	0.082106	0.118026	0.297209	0.057075	0.033816	0.163617	0.495188	0	0	0	0
LOC107811192	13.65657	14.97864	13.67006	3.106431	2.824886	3.49583	3.07433	2.626686	3.996279	2.536549	2.321454	2.697067	2.435063	2.321868	1.63832	12.55591	12.56514	13.31095	2.27963	1.450317	3.073318	3.258561	4.745395	3.645701	0.448469	0.658082	0.838492	0	0.284622	3.65544
LOC107788926	0.170704	0.442791	0.194047	0.226025	0.046477	0.155292	0.066081	0.033659	0.012328	0.012804	0.027623	0.066561	0.014618	0.100277	0.013477	2.142975	1.928226	2.261434	2.305136	2.166053	0.742496	0.592169	0.637035	0.823474	0.448244	0.365418	0.512155	0	0.695393	0.811913
LOC107770295	3.045997	3.139272	2.89528	3.765325	2.896816	4.499051	2.793576	2.578259	3.714958	2.970232	3.712795	2.429043	5.165495	4.689514	3.593816	2.566874	2.942689	2.377038	2.022063	2.070996	1.350056	3.637384	3.414961	4.165556	1.367668	1.029863	1.211256	4.071623	2.466928	0
LOC107812810	0	0	0	0	0	0	0	0	0	0	0	0	0	0	0	0	0	0	0	0	0	0	0	0	0	0	0	0	0	0
LOC107799559	11.24954	28.95477	52.51394	138.9631	304.3519	137.1995	95.51872	156.1369	130.592	283.1686	209.1301	251.3676	225.2523	207.5085	288.1404	95.53736	21.13698	79.8197	412.747	377.4815	461.0165	183.6299	97.79598	188.7455	515.9047	540.8679	638.5945	694.7007	542.5881	1128.257
LOC107831132	0	0	0	0	0	0	0	0	0	0	0	0	0	0	0	0	0	0	0	0	0	0	0	0	0	0	0	0	0	0
LOC107766645	6.463313	6.528651	7.397587	4.147599	3.431498	4.57466	3.885092	3.81968	3.640825	2.521018	4.362126	2.343416	2.87802	3.084881	3.897338	4.435164	5.46359	5.802486	1.830921	2.118663	1.621029	3.553473	3.800713	3.236988	0.510724	0.974266	1.018549	1.925909	0.90757	0
LOC107762781	0.549364	0.36588	0.514285	40.38995	37.56162	42.27574	15.89675	20.65323	23.88399	33.31638	17.06797	72.17037	21.78094	35.12992	15.96136	0.314913	0.36743	0.393821	64.60067	57.07172	82.97817	32.78572	16.79241	41.98251	51.95868	65.70901	97.2458	24.1768	42.9277	14.37108
Novel04785	0.026414	0	0.022961	0.478602	0.420718	0.198791	0.288014	0.203123	0.074397	0	0.074087	0.089262	0	0	0.054222	0	0	0.019693	1.018533	0.737377	0.722711	0.35295	0.815474	0.49695	0.166979	0.220522	0.03122	0	0.127168	0
Novel03523	2.204359	1.75389	2.889511	0.63398	0.928839	0.601893	0.763034	0.538133	1.149749	0.204717	0.245349	0.118241	0.077902	0.305377	0	1.799137	1.014093	1.799961	0.355053	0.177594	0.47867	0.350651	1.357987	1.499425	0	0	0.08271	0	0	0
LOC107805205	13.29777	16.06886	15.82547	16.7799	19.47116	18.80701	10.83806	10.71948	11.42558	11.40402	8.755506	11.15074	14.80326	10.75088	9.566114	15.36565	14.7776	15.94805	17.30883	17.66026	10.19673	10.42776	9.600048	11.45826	11.78371	8.067475	10.01017	1.415147	13.90915	6.117717
LOC107805046	6.065161	5.915758	6.528697	3.741092	3.517002	4.10671	3.539331	3.142421	4.821931	2.26505	3.266585	1.798857	3.318449	3.012752	2.834414	5.346786	4.01435	5.580137	2.959401	2.816432	1.247536	4.44011	4.268516	5.368177	0.766821	0.7721	1.25068	0	0.869783	1.595821
LOC107805641	4.056864	4.095145	3.303347	3.745098	3.748821	3.064179	2.735055	2.193857	2.265973	1.677525	2.052487	1.800444	2.572495	2.997245	2.319109	3.999016	5.432408	3.503134	3.17866	2.780215	3.653094	2.573159	2.038342	2.254301	1.103732	0.571628	0.971116	1.836222	4.079299	3.175215
LOC107777428	0.493816	0.900105	1.226468	3.163561	7.053796	3.033786	1.567985	2.621992	3.311544	3.817868	4.377767	9.744353	3.259071	4.155909	7.059545	2.234238	0.681525	2.103776	8.840664	8.593391	10.93752	5.037154	2.520132	5.7881	7.893359	14.42929	7.629148	5.044974	10.52851	13.08574
LOC107800442	0.178076	0.301404	0.176912	0.092187	0.180084	0.054701	0.128023	0.065208	0.047767	0.124033	0.053514	0.128952	0.02832	0.138766	0.052221	0.056872	0.024577	0.075865	0.051628	0.06456	0	0.106226	0.022439	0.106357	0	0.035397	0	0	0.122474	0
LOC107771702	0.426936	0.413419	0.487104	0.435131	0.566672	0.788921	0.861654	0.604177	0.75155	0.507386	0.561315	0.169072	0.549533	0.305659	0.670987	0.636307	0.580017	0.487396	0.379072	0.462735	0.316366	0.924791	0.706099	0.571737	0.303625	0.167077	0.14192	0	0.064232	0
LOC107780345	19.75196	19.9675	22.97141	16.73389	15.62864	17.75687	18.4327	16.24301	18.29085	15.71091	17.96257	17.71214	18.61113	20.5188	11.07051	24.01286	23.3804	25.17982	21.82147	21.67011	19.12079	19.19769	20.39566	22.61762	14.57438	11.5374	20.87525	33.74679	19.79751	29.17765
LOC107791972	3.219354	6.264581	4.991948	2.758919	3.695606	0.935459	4.159784	2.973734	3.349225	1.187834	2.013368	0.514557	0.968603	1.139078	1.964693	4.927812	4.791338	6.681541	0.794622	1.61929	0.555479	7.920372	4.451395	4.365234	1.320079	0.96854	0.514192	0	1.67558	0
LOC107832416	25.35928	25.43014	31.11471	20.4346	18.25942	21.23659	20.89982	21.51241	25.95287	21.56467	21.24005	23.0908	12.94491	17.01853	18.94161	27.15708	22.54327	25.93102	21.33429	24.35056	21.47101	24.64648	28.64183	28.56735	7.252473	9.426344	18.77508	9.281203	10.24695	9.629493
LOC107796638	1.923958	1.578106	2.38325	1.350832	1.14915	1.809929	0.887539	0.69865	0.948306	0.656636	0.640813	1.259695	0.803166	1.469261	1.135451	1.863857	1.5799	1.004075	0.78092	0.935833	1.447613	0.803374	1.994044	1.910375	0.182435	0.133852	0.454793	0	0.694696	0
LOC107775119	0	0	0	0	0	0	0	0	0	0	0	0	0	0	0	0	0	0	0	0	0	0	0	0	0	0	0	0	0	0
LOC107809958	3.590548	4.074387	3.457786	3.157183	1.993508	2.951978	2.273423	1.95501	2.015969	1.098428	1.308214	0.951653	1.410723	1.536122	1.517452	2.675681	3.196711	2.991836	1.75028	1.667568	1.476799	2.234222	2.204551	2.207553	0.756591	0.342862	0.832106	0	0.508417	0
LOC107813007	4.073383	5.184258	5.475145	4.551886	4.57298	4.977451	5.072105	4.262351	5.424738	4.024557	5.989336	4.487344	5.433466	4.013178	5.304286	5.335509	4.333955	5.230926	4.206223	4.402126	3.46951	5.70961	4.970044	5.364074	2.382156	2.521803	2.523854	0	3.196461	2.219061
LOC107767377	0	0	0	0	0	0	0.156624	0.079777	0	0.091046	0.26188	0	0	0.101861	0.670817	0	0	0	0.094744	0	0.085154	0.233924	0.329429	0.097589	0	0	0	0	0.449509	0
LOC107819888	0	0	0	0	0	0	0	0	0	0	0	0	0	0	0	0	0	0	0	0	0	0	0	0	0	0	0	0	0	0
LOC107822717	0.189696	0.432211	0.288572	0.773347	0.860259	0.535359	0.536983	0.445723	0.445237	0.393077	0.382418	0.280456	0.52793	0.491503	0.267711	0.194372	0.114539	0.176783	0.842145	0.9227	0.713649	0.336642	0.37648	0.421324	1.139208	1.352733	0.336308	1.695742	0.228316	1.466147
LOC107823022	0.037548	0.068441	0	0.017008	0.033225	0.080738	0.015747	0.032082	0	0	0.052658	0	0.146297	0.102408	0.019269	0	0	0	0	0.015882	0	0.062715	0	0.019623	0	0	0	0	0	0
Novel03971	0	0	0	0	0	0	0	0	0	0	0	0	0	0	0	0	0	0	0	0	0	0	0	0	0	0	0	0	0	0
LOC107788290	13.47027	13.07466	13.41327	27.42928	31.3668	27.7538	21.47688	22.80494	17.03164	32.81771	24.59304	28.15068	25.67306	25.61459	22.85705	15.56988	17.12292	15.26609	44.053	40.57016	44.80454	20.51357	22.58621	24.58398	37.2246	30.46447	46.28363	29.82399	37.94669	36.10038
LOC107774396	0.851881	0.796292	0.645583	1.167536	1.237006	1.103748	1.154207	0.89584	1.209929	1.086289	1.179363	0.904229	1.750755	1.93021	0.874347	0.634823	0.801886	1.172517	0.531957	0.461944	0.378504	0.912087	0.693614	0.821895	0.276163	0.060786	0.309801	0	0.210321	0
LOC107810911	7.439364	7.359771	8.438198	13.27894	16.64063	10.70395	16.51553	14.29598	23.78332	11.83883	16.69664	16.47179	13.39218	14.15017	17.5703	21.43337	10.09928	19.58752	8.390843	11.01893	13.34265	23.21142	16.78314	27.48928	3.636373	4.348329	9.283031	3.955537	7.322922	5.129968
LOC107799371	3.473738	3.87581	3.367364	3.833656	3.11105	2.535065	4.785324	4.604984	4.42746	4.454857	4.635377	3.877375	5.366946	4.915097	4.062338	4.345767	3.844057	4.332061	2.28585	2.742641	1.958467	3.164738	3.788301	3.894805	3.060994	2.226318	2.985948	0	7.500376	3.905208
LOC107787625	0	0	0	3.650262	13.47478	3.567444	11.57967	17.01083	13.12855	5.662369	6.647732	14.71725	8.179214	11.50632	20.799	0	0	0.044176	29.82248	24.3603	29.88403	30.77894	16.62033	9.537428	32.43775	69.09008	13.58629	88.98616	38.79613	21.98226
LOC107787967	2.091008	0.698755	1.151191	2.367935	0.986806	2.885049	0.584606	0.7742	1.930412	1.563231	2.419257	4.504682	1.16387	2.965544	0.894231	0.67523	0.505022	0.857415	0.424362	0.176885	0.603894	0.174626	0.153701	0.619231	0.220306	0	0.20595	0	0.167781	0
LOC107824240	2.795362	2.304997	2.661373	1.567711	1.001187	3.577805	0.949001	1.592289	2.686895	0.519206	0.793378	0.337371	0.740913	0.943925	0.478181	1.78552	2.186163	2.332164	1.080587	1.013436	1.153308	2.779143	3.346299	2.43476	0.336589	0.185216	0.314656	0	0.640851	0
LOC107781390	0	0	0	0	0	0	0	0	0	0	0	0	0	0	0	0	0	0	0	0	0	0	0	0	0	0	0	0	0	0
LOC107773049	1.243436	1.133239	1.824007	2.182575	2.544385	2.255928	2.86804	2.191253	1.751089	1.515634	1.907278	1.313112	1.211184	1.526094	2.39293	1.911134	1.276343	1.390552	1.89263	3.287068	1.063158	2.660967	2.056487	2.111914	2.063357	3.676571	0.826674	0	1.496586	0

LOC107821406	2.424013	3.592334	2.785094	3.036254	2.55526	3.875173	3.252999	2.125172	2.058088	1.192136	1.906642	1.210917	1.220167	1.701665	2.098524	4.351013	3.930152	3.394402	2.887499	3.833634	2.268412	4.154332	3.997392	2.886076	0.959381	0.997187	0.772301	0	0.405915	0
LOC107759540	0	0	0	0	0	0	0	0	0	0	0	0	0	0	0	0	0	0	0	0	0	0	0	0	0	0	0	0	0	0
107776812	0	0	0	0	0	0	0	0	0	0	0	0	0	0	0	0	0	0	0	0	0	0	0	0	0	0	0	0	0	0
LOC107794954	10.85201	9.253768	9.807025	5.694409	4.516062	8.952585	4.325727	4.636127	5.699422	3.981377	4.407447	5.083293	3.5883	5.274807	2.591563	7.686589	10.22445	8.371004	4.470166	4.590028	4.164671	3.813554	6.586806	5.446656	1.901816	1.270774	4.381229	0	1.810486	0
LOC107826651	0	0.25258	0	0.376614	0.183925	0.07449	0.290563	0.355196	0.390289	0.067562	0.388664	0	0.231388	0.226761	0.782239	0.154895	0	0.051655	0.773366	0.468885	0.189568	0.231448	0.366686	0	0.613185	0.385622	0.16378	0	0.667129	0
LOC107790622	0	0	0	0	0	0	0	0	0	0	0	0	0	0	0	0	0	0	0	0	0	0	0	0	0	0	0	0	0	0
LOC107780882	0.087082	0.158729	0	0.039446	0	0	0.03652	0.037203	0.040878	0.084916	0.305309	0	0.145411	0	0.223446	0.227127	0.042064	0.032462	0.088365	0.294661	0.03971	0.072724	0.038406	0	0.110098	0.060584	0	0	0	5.384405
LOC107780915	543.2615	459.8434	582.321	222.4932	203.3507	360.2683	194.201	214.2703	275.3033	108.5292	134.0572	155.8657	89.0125	121.4202	86.38808	297.5391	508.7188	388.3843	122.6218	114.1753	213.0857	183.9495	302.2712	322.907	28.59886	23.21369	76.47541	75.24033	25.32602	37.17325
LOC107780101	12.10554	17.65234	23.19128	14.38366	21.27937	9.811263	17.04607	26.4351	21.83433	28.92087	23.45741	23.83701	17.23381	19.25109	40.11777	35.99752	12.797	23.7085	37.70203	34.22676	33.20631	34.62557	20.71922	20.51199	39.26344	92.09123	27.59751	23.30778	27.1228	24.47033
LOC107784362	0.058006	0	0	0	0	0	0	0	0	0	0	0	0	0	0	0	0	0	0	0	0	0	0	0	0	0	0	0	0	0
LOC107814375	1.336218	1.461358	1.471292	0.807031	0.867074	1.723918	0.896595	0.608907	1.170867	1.650443	1.748987	1.279391	0.892498	0.971832	1.097166	0.796601	0.77454	1.062621	0.813541	0.301426	1.299897	1.711063	0.942906	1.769046	0.675755	0.86765	0.421147	0	1.715475	0
LOC107779179	0.14523	0.375019	0.273532	0.131571	0.192764	0.104094	0.284226	0.351585	0.386321	0.094412	0.594041	0.265838	0.377236	0.554537	1.192484	0.487016	0.39753	0.378964	0.14737	0.122855	0.154528	0.909643	0.597813	0.556581	0.030603	0.168398	0	1.731004	0.116532	0
LOC107772382	0	0.054309	0	0	0	0	0.099962	0	0	0	0	0	0	0	0.061162	0	0	0	0	0	0	0	0	0	0	0	0	0	0	0
LOC107801138	4.636566	3.950072	4.49777	2.008932	2.140559	2.492438	1.719001	1.751146	2.365768	1.801943	1.95541	2.838478	3.628004	3.555448	2.069063	3.179762	3.667843	3.657149	0.852331	0.682125	1.685322	1.627412	1.955971	2.0719	0.679656	0.280498	0.555947	0	2.588067	0
LOC107825846	0	0	0	0	0	0	0	0	0	0	0	0	0	0	0	0	0	0	0	0	0	0	0	0	0	0	0	0	0	0
LOC107798013	56.1882	71.88321	68.03504	46.90337	36.19036	40.23323	48.23852	45.65073	46.63729	28.57937	36.3417	29.88984	27.03514	25.10953	31.55423	48.90494	61.21447	51.19058	26.65969	33.10386	30.05208	41.89993	45.18002	39.4596	10.11852	15.28308	15.98274	23.6832	11.69198	20.47663
Novel03554	1.481241	0.418956	4.639842	1.41134	0.610153	8.319522	5.783498	6.589915	9.422884	0.498068	0.286523	1.208242	0.767609	1.142318	7.86365	0.095157	2.78799	7.673178	0.95885	11.12603	2.026376	6.633005	14.86767	6.406316	0.742638	0.568563	6.338771	14.61099	1.72133	7.895465
LOC107815550	0	0	0	0	0	0	0	0	0	0	0	0	0.065813	0	0	0.044056	0	0	0	0	0	0	0	0	0	0.08226	0	0	0	0
LOC107778774	0.2869	0.261474	0.17814	0.321802	0.193423	0.190947	0.045835	0.058366	0.102611	2.344687	1.800991	1.558165	0.912518	0.70051	0.392622	0.23416	0.395957	0.285195	0.277263	0.369825	0.261658	0.182551	0.120507	0.299868	1.41637	2.186098	2.276766	0	0.855053	0
LOC107765042	4.382446	4.3761	3.610808	4.695281	4.063341	4.916521	5.417731	4.298013	3.935545	3.994736	6.119227	3.750705	5.260404	4.323717	4.576249	3.762774	4.620377	3.764498	3.557603	3.723367	2.137454	4.216836	4.000063	3.64451	1.20451	0.954447	2.274563	1.362639	1.1008	0
LOC107767141	0.270796	0.458338	0.302654	0.245328	0.445006	0.332731	0.032447	0.165268	0.108958	0.528121	0.271261	0.359506	0.17226	0.717462	0.317643	0.66305	0.971702	0.778719	0.15702	0.32725	0.141126	0.129228	0.102369	0.080868	0.09782	0.322966	0.365782	0	0.186244	2.391964
LOC107785051	1.752948	1.065064	1.342396	1.399025	1.772713	2.378228	1.155215	1.319461	1.489007	1.465154	1.551093	1.833544	2.741261	1.730249	1.24229	1.679527	2.217672	2.053696	1.270551	1.376954	1.027747	1.115377	2.024807	2.355661	0.633217	0.46459	0.937263	0	2.813106	0
LOC107788323	8.117253	7.728028	6.00648	3.184629	1.994529	3.649489	1.125337	1.089061	1.347817	0.654161	0.969021	0.442065	0.537694	0.453754	0.440665	5.319109	5.418074	8.472459	1.538449	1.668423	1.419432	1.098075	2.130234	1.584643	0.22052	0.112012	0.237866	0	0.32297	0
LOC107796261	0	0	0	0	0	0.149376	0	0	0	0.135482	0.097424	0	0	0.454725	0.142603	0	0	0	0	0	0	0	0	0	0	0	0	0	0	0
LOC107816322	0.246929	1.350271	5.688223	5.173177	8.576078	2.123829	13.66925	18.98844	16.43078	11.52766	11.42768	9.700471	6.013099	8.990796	21.57416	21.04633	1.103312	11.02274	20.07662	22.03734	21.19725	23.30248	17.28847	14.48529	36.29244	55.91819	19.4445	30.90307	24.66766	57.25491
LOC107764498	0	0	0.133136	2.220043	2.303901	2.963929	2.055353	1.832063	1.150326	1.792171	1.288727	2.070263	0.340993	0.167087	1.257571	0.456532	0	0.22837	3.885334	2.202528	5.307914	1.279056	1.621137	1.440719	4.647294	6.180105	1.629175	0	2.21206	0
LOC107786047	0	0	0	0	0	0	0	0	0	0	0	0	0	0	0	0	0	0	0	0	0	0	0	0	0	0	0	0	0	0
LOC107821744	0	0	0	0	0	0	0	0	0	0	0	0	0	0	0	0	0	0	0	0	0	0	0	0	0	0	0	0	0	0
LOC107766807	10.69116	13.19193	8.961688	7.515815	9.275955	7.575794	7.394979	6.880774	5.572677	10.89911	7.813068	9.530723	8.655675	8.103884	6.056591	10.99352	9.892618	10.68801	12.96203	11.18753	7.597809	6.55141	7.501386	6.857019	13.7802	11.10868	9.27194	7.447105	15.37448	8.585075
LOC107823943	10.02416	3.088153	5.645553	80.70917	95.38411	43.48827	103.912	145.4829	133.6114	65.60144	89.64382	92.79774	42.12142	59.83921	67.74485	5.839237	5.387678	9.157591	198.3506	162.9664	217.6102	128.2834	180.1396	111.2654	150.4765	216.094	188.0622	75.72561	27.52852	34.91881
LOC107782389	5.379947	6.775892	7.079964	9.714072	9.454944	8.836821	9.056123	5.905576	7.506245	5.537579	7.020897	4.576692	10.23126	6.236086	4.639871	3.814254	4.331234	4.066687	5.572932	4.961925	7.359903	5.335359	8.106843	6.286959	4.15671	1.09168	3.091017	2.671821	3.057755	0
LOC107807427	12.65354	14.03524	11.93739	13.06302	9.026783	11.81131	14.8912	15.42109	15.8396	10.23451	9.285351	10.02711	9.39073	9.095915	8.859516	11.91471	8.528638	11.62764	9.555301	9.293415	8.409167	14.9089	16.00645	15.07089	8.308996	6.687731	11.12965	0	6.611325	0
LOC107804431	0	0	0	0.035439	0	0	0	0	0	0	0	0	0	0	0	0	0	0	0	0	0	0	0	0	0	0	0	0	0	0
Novel02264	84.29613	82.71236	86.77148	49.32204	52.35282	71.83765	43.06816	48.0939	47.13803	45.36051	45.48272	51.54312	29.48728	39.61572	30.91963	50.19743	83.5347	62.36073	37.83936	40.45267	49.55336	37.08016	47.35149	46.01044	24.36218	16.91783	34.89981	97.04387	21.95097	13.42474
LOC107775782	0	0	0	0	0	0	0	0	0	0	0	0	0	0	0	0	0	0	0	0	0	0	0	0	0	0	0	0	0	0
LOC107792890	0	0	0	0.327265	0.532748	0	2.019915	2.880762	0.565246	0.587091	1.688677	0.101728	1.072366	0.656825	3.336899	0.089732	0.232659	0	1.344057	0.814891	0.658913	2.715127	1.593187	0.251712	1.674628	0.502639	0.426956	17.2225	0	0
LOC107806730	0	0	0	0	0	0	0	0	0	0	0	0	0	0	0	0	0	0	0	0	0	0	0	0	0	0	0	0	0	0
LOC107822090	0	0	0	0	0	0	0	0	0	0	0	0	0	0	0	0	0	0	0	0	0	0	0	0	0	0	0	0	0	0
LOC107799795	4.394946	3.859798	4.931832	3.873009	4.030353	3.994861	3.150051	2.423786	3.751062	3.428502	3.165786	3.172904	3.335806	3.792148	2.378449	4.108785	2.933522	4.617053	3.121781	2.670072	3.206612	2.836167	3.030822	4.008956	3.081358	0.945084	3.872243	2.85728	6.924709	0
LOC107772169	0	0	0	0	0	0	0	0	0	0	0	0	0	0	0	0	0	0	0	0	0	0	0	0	0	0	0	0	0	0
LOC107815705	72.61849	74.00001	78.33118	74.69437	62.67996	79.62798	68.70872	69.41712	79.66853	42.66588	45.41251	45.43247	47.68858	53.04327	45.94674	60.18406	73.69361	69.83974	47.43038	48.14142	55.90156	63.90525	76.46563	88.7944	23.94297	25.74694	34.57089	20.67651	22.50318	38.73354
LOC107777918	3.849738	5.741282	1.115498	0.105687	0	0	0	0.199353	0	0.113757	0.081801	0	0	0	0.239471	5.911526	3.155659	2.696195	0.118377	0	0.106395	0.292274	0.205801	0	0.147492	0	0	8.342747	0	0
LOC107798535	0.330621	0.043046	0.082115	0.085579	0.459729	0.05078	0	0	0.088686	0.184227	0.099356	0.119708	0	0.206109	0	0.281576	0.136889	0.316918	0.239636	0.039955	0.172304	0.078889	0.041661	0.049366	0	0.197158	0	0	0	0
LOC107799077	4.589683	5.228663	4.638043	3.707568	3.807425	4.379343	4.154322	4.084948	3.554922	3.170161	4.103318	3.651305	4.93899	3.296353	4.534073	0.057004	0	0.05703	0	0	0	0.015971	0	0.019988	0.024178	0	0	0	0	0
LOC107767300	0.05846	0.213115	0.101635	0.052961	0	0	0	0	0	0	0	0	0	0	0	0	0.112954	0.130752	0	0.098906	0	0	0.051565	0	0	0	0	0	0	0
LOC107779309	0	0	0.040259	0	0	0.049792	0	0	0	0	0	0	0	0	0	0.034512	0	0	0	0	0	0	0	0	0	0	0	0	0	0
LOC107764660	2.46367	2.391766	3.398651	1.067459	0.616092	1.20921	0.583979	0.411853	0.854805	0.104452	0.450659	0.497721	0.059622	0.409004	0.329823	1.756107	1.397022	2.156209	0.54347	0.543677	0.488458	0.626188	0.661384	1.45545	0.203141	0	0.189904	0	0	0
LOC107825789	0	0	0	0	0	0	0	0	0	0	0	0	0	0	0	0	0	0	0	0	0	0	0	0	0	0	0	0	0	0
LOC107766589	0.500643	0.741446	0.924796	0.510252	0.498377	0.740097	0.629866	0.908996	0.705038	0.366143	0.351051	0.317218	0.487657	0	0.385385	1.025969	0.483663	0.979783	0.381013	0.741142	0.456595	0.992987	0.552	0.850317	0.158241	0.261228	0.29586	0	0	0
LOC107762886	3.016296	5.823112	2.819357	0.881487	0.688779	1.046093	3.101164	3.270002	1.217992	1.612954	4.525706	0.137003	1.191471	2.052221	2.430071	6.235686	2.725996	3.143451	0.2962	1.015144	0.059159	4.577515	1.487632	0.033899	0.574078	0.180514	0.766671	0	0	0
LOC107764280	1.081588	0.563277	0.402943	0.349951	0.136723	0.49836	0.129596	0.13202	0.290126	0.150669	0.216689	0.195805	0	0.252848	0.237881	0.057571	0.149272	0.230391	0.470366	0.065354	0.281836	0.064519	0.272581	0.322993	0.195351	0	0	0	0	0
LOC107803861	27.19168	19.04291	19.73909	16.85504	12.91695	25.13131	11.52345	12.55414	18.27321	17.2117	13.84853	21.68261	17.94967	22.27461	12.04485	15.14413	21.19991	18.77878	10.45601	13.88208	12.96523	10.43793	14.47507	17.35161	5.428192	1.858573	13.08088	27.29248	9.646031	0
LOC107763034	0	0	0	0	0	0	0	0	0	0	0	0	0	0	0	0	0	0	0	0	0	0	0	0	0	0	0	0	0	0
LOC107768583	15.09597	22.957	14.14581	2.692581	2.291414	2.84737	3.529462	2.966884	4.917643	2.381675	2.269752	2.85897	2.948252	2.88929	2.084	14.47308	17.05728	11.14499	1.254133	1.020616	1.690779	4.030319	2.907118	3.598559	0.66968	0.204726	1.043404	0	2.26674	0
LOC107806416	42.48761	49.36344	45.62659	2.742406	1.961106	6.741485	1.133467	3.094494	1.826988	0.57982	1.175016	0.502343	0.120351	0.648691	0	29.52686	38.38327	43.80681	2.029515	1.005999	1.626884	3.340593	3.671399	1.977457	0	0	0.127778	0	0	0
LOC107779667	1.446384	2.295081	1.728802	2.585602	2.011202	1.888262	4.60345	2.791651	2.449122	1.460779	4.147375	1.46197	3.234646	1.704732	4.215002	2.848596	1.958755	1.954492	1.310438	1.97733	0.671341	4.745373	3.257862	2.402619	0.555132	0.9883	0.274743	1.847088	0.435213	0
LOC107767086	0.591605	0.476962	0.712065	0.329824	0.221477	0.464804	0.114509	0.155533	0.3418	0.288445	0.494609	0.422911	0.227971	0.273059	0.373666	0.373039	0.417664	0.474995	0.346338	0.096242	0.373537	0.15202	0.441552	0.38052	0.230144	0.094982	0.215147	0	0	0
Novel02749	10.45298	10.78286	10.98374	161.3	131.3223	130.5364	163.6896	167.8301	188.4018	82.6639	116.3076	141.7807	40.02411	44.23628	38.5524	14.12395	13.42762	12.84583	194.189	200.1709	229.4276	159.7221	260.5978	179.7297	48.94182	29.56861	106.8468	90.36147	35.94597	42.61473
LOC107822442	11.04743	9.780701	10.28202	11.19738	11.20139	10.78784	10.54786	9.609554	11.71313	7.403151	8.771559	6.315682	6.620659	5.41896	5.439203	10.95611	9.661643	10.86204	8.73213	10.28693	6.787665	12.65279	12.98298	17.45038	5.901975	3.744682	6.459864	2.376082	7.11816	11.29905
LOC107796674	26.78481	39.23118	35.05161	1.485165	1.216634	2.871166	1.064509	2.033277	1.737683	0.464101	1.149511	0.223382	0.559257	1.038453	0.244246	16.21632	13.15531	15.06069	1.207376	1.00653	1.302194	1.611959	2.77541	2.238532	0.869168	1.030143	0.531271	0	1.527561	1.634891
Novel01162	1.04296	1.61942	0.873044	0.944869	1.743217	0.996719	1.036771	1.056158	0.797848	0.941683	0.893841	0.750585	1.419046	1.769939	0.75329	1.266572	2.351036	1.238354	1.175915	1.176363	2.113771	0.612928	0.613306	0.524864	0.830241	0.537482	0.59352	0	0.371939	2.388436
LOC107819241	0	0	0	0	0	0	0	0	0	0	0	0	0	0	0	0	0	0	0	0	0	0	0	0	0	0	0	0	0	0
LOC107816410	8.314248	9.013132	10.04228	5.986186	4.723964	7.056974	6.496382	6.617861	9.161523	2.517574	2.976346	6.136852	5.261042	5.442261	4.536238	6.456663	8.497288	7.66672	3.72992	4.108917	5.188186	5.554756	8.491672	8.827261	1.770398	1.643969	2.637712	0	1.896051	0
LOC107779630	4.25931	4.029998	4.522172	2.179735	2.244089	3.355808	1.336266	1.639061	1.404169	1.299908	1.709903	1.840395	1.882131	2.412026	1.134624	3.246731	3.48663	4.048152	1.913581	2.145347	1.067513	1.547729	2.236988	2.820625	0.698825	0.316684	1.076004	2.325198	0.469599	0
LOC107774477	0.037228	0	0.032361	0	0.065884	0.080049	0.062449	0.031809	0.034951	0.036302	0	0.031451	0.041443	0.081228	0	0.055485	0	0.027755	0	0	0	0.03109	0	0	0	0	0	0	0.179229	0
LOC107830539	0	0	0	0.037483	0.018305	0.022241	0	0.017676	0.019422	0	0	0	0	0	0.042465	0	0.019985	0	0.020992	0.122499	0.056601	0	0.018247	0	0	0	0	0	0	0
LOC107798613	0.108	0	0	0.097842	0.19113	0	0.090584	0.092278	0	0.105313	0	0	0	0.117822	0	0	0	0.241555	0	0	0	0	0.095263	0.112881	0	0.150273	0	0	0	0
LOC107794431	11.84322	12.39513	13.2664	9.813435	8.370947	10.50724	9.792088	9.049662	9.875965	8.731893	8.236956	6.914356	8.548186	8.534797	7.115447	11.15754	9.232303	11.4139	7.034739	7.351008	5.927496	8.282356	8.599254	9.107779	3.469453	3.04794	4.808164	6.885834	3.129007	7.441911
LOC107797329	0.085925	0.093972	0.089631	0.731729	0.805938	0.221711	2.407099	3.127542	1.355256	0.955178	1.397812	0.624288	0.975657	1.574828	3.174871	0.115256	0.066408	0.051248	1.272982	2.107908	1.7397	2.884674	2.36469	0.718469	4.388857	8.106039	1.462409	4.915871	7.777045	29.75202
LOC107793028	29.5086	20.33407	22.9401	4.274429	3.821144	6.190313	7.445162	7.106088	11.1867	2.495359	3.925207	3.580686	4.184068	5.757996	3.118968	15.4942	19.23696	16.6937	0.973765	1.623561	3.427859	9.082663	7.90026	11.45096	0.202211	0.111271	0.756136	0	0	0
LOC107760177	0.739976	0.337199	0.321623	1.819606	2.385269	2.273043	0.576318	0.767739	1.339822	1.958548	1.667803	4.063492	4.942513	5.53562	1.735981	0.630207	0.970194	1.260992	2.467167	1.699059	2.747689	0.92696	0.559464	1.491602	2.606189	1.397346	3.123545	7.559826	6.870555	0
LOC107814620	2.527628	2.192603	3.176694	1.241514	1.131779	2.029947	0.791819	1.196928	1.000683	0.475134	0.512494	0.540285	0.542417	0.498347	0.406334	1.906285	2.35364	2.270426	0.988863	1.133503	0.694349	1.195322	1.369955	1.177704	0.038502	0.084747	0.395928	0	0.146613	0
LOC107763521	11.03189	8.444479	13.91904	16.23751	14.60418	14.35105	15.95583	17.31801	29.54935	15.16213	10.23288	19.10487	9.057533	10.83488	5.171056	12.4933	10.09827	13.08187	10.04852	7.397193	12.83406	10.32535	14.94108	27.72169	4.576005	2.336756	13.34681	2.070698	4.042604	3.580674
LOC107828838	0	0	0	0	0	0	0	0	0	0	0	0	0	0	0	0	0	0	0	0	0	0	0	0	0	0	0	0	0	0
LOC107818392	6.625602	6.190329	5.668937	7.682394	8.14888	6.585723	8.493046	8.010983	6.826799	7.375063	7.041863	7.51613	10.78524	9.978562	10.05081	6.521103	6.09896	5.964882	7.716884	7.349695	6.365696	7.134017	5.770707	5.966891	8.113355	9.856837	5.737756	11.92011	8.024656	10.30619
LOC107824286	30.82958	29.43268	31.60218	25.16469	23.03948	26.2211	23.32349	21.67316	25.10405	17.5614	16.41663	15.78214	17.9415	17.28299	16.04062	28.66093	28.01462	26.87624	17.6242	18.51401	16.45268	23.8857	28.27071	27.34175	10.61281	10.83046	13.85369	15.28045	12.49118	20.76102
LOC107825749	8.883387	6.711802	9.162539	8.464888	7.412591	10.19404	4.671276	5.073247	6.179463	4.847367	5.518991	7.854903	4.25283	6.276773	3.1652	5.419408	7.603828	8.544624	6.398714	5.412166	7.765972	4.266741	6.983142	6.879495	1.920378	0.896623	2.502459	0	1.772758	17.07585
LOC107832152	3.074768	4.175102	3.091304	3.390534	3.050546	3.539492	3.738161	2.958878	2.974204	3.376859	4.290281	2.833799	3.906918	2.846175	4.303442	4.779371	3.705604	4.58474	3.21461	3.349827	1.50126	3.994385	3.205267	2.726815	2.49346	3.003464	2.386044	0	2.541934	0.96019
LOC107799470	6.314984	3.468965	4.511902	6.543947	6.697841	8.788991	2.684603	2.882631	3.979595	2.572824	2.638645	3.836868	2.889011	4.246851	1.997731	1.579389	2.925044	3.32146	5.398528	4.610255	6.153849	2.311806	4.120446	7.278544	2.296784	2.949008	3.220682	0	1.457658	2.674417
Novel00869	0.058071	0.026462	0	0.078914	0.179847	0.187299	0.243532	0.17366	0.109039	0.113253	0.061079	0.07359	0.12929	0.063352	0.178806	0.043274	0.028051	0.064941	0.530335	0.442114	0.37073	0.096993	0.281722	0.424868	0.807609	0.484807	0.205905	0	0.419359	5.385897
LOC107819338	0.537622	0.349983	0.433962	2.644032	3.567926	3.591917	1.352781	2.132738	2.307398	1.797427	0.807816	2.206105	0.983231	1.005462	0.433558	0.486487	0.408091	0.65849	4.247433	2.923659	4.412884	0.705544	1.253291	2.568789	1.456538	0.961794	3.585619	0	1.109273	0
LOC107813045	0.395319	0.630498	0.085911	0.984882	1.749021	0.212508	4.973566	3.715484	1.391786	0.192743	0.277198	0.083494	0.220037	0.107818	0.304308	2.430385	0	0.221045	3.610281	2.758904	1.622419	2.641129	2.78958	0.413187	0.749705	0.550057	0.700853	7.067724	0.475802	0
LOC107827326	0	0.180142	0.085911	0.089535	0	0	0	0	0	0	0.069299	0	0	0.107818	0	0	0	0	0.100286	0	0	0.082535	0.174349	0	0	0	0	0	0	0
LOC107809000	6.479971	7.505153	7.510534	7.477931	6.228795	6.593541	6.826139	6.360571	5.667239	4.268944	5.909592	4.578342	6.612461	4.691847	5.443416	10.30449	9.203979	9.777212	4.89243	5.856838	4.098194	8.278448	6.991602	7.458213	1.8531	2.36144	2.393376	1.379195	1.671262	1.192459
LOC107779695	0	0	0	0	0	0	0	0	0	0	0	0	0	0	0	0	0	0	0	0	0	0	0	0	0	0	0	0	0	0
NitaMp045	0	0	0	0	0	0	0	0	0	0	0	0	0	0	0	0	0	0	0	0	0	0	0	0	0	0	0	0	0	0
LOC107788785	0	0	0	0	0	0	0	0	0	0	0	0	0	0	0	0	0	0.050002	0	0	0	0	0	0	0	0	0	0	0	0
LOC107790955	0	0	0	0	0	0	0	0	0	0	0	0	0	0	0	0	0	0	0	0	0	0	0	0	0	0	0	0	0	0
LOC107805079	0	0	0	0	0	0	0	0	0	0	0	0	0	0	0	0	0	0	0	0	0	0	0	0	0	0	0	0	0	0
Novel01328	1.745992	1.798812	2.573583	2.750922	2.283867	1.550693	2.228494	2.140442	2.423186	2.738905	3.406722	2.24466	1.774649	1.65634	2.181612	1.923388	1.906789	1.358307	1.771712	1.926508	1.730841	2.916247	3.013201	2.22163	1.247697	1.795659	0.538336	0	1.827354	0
LOC107794910	11.46957	20.17952	12.04961	49.12039	25.34545	7.45147	61.73278	69.59512	21.8242	39.8926	67.7804	17.25512	24.92167	12.11126	72.89861	19.74526	11.4361	10.24297	22.46796	67.91729	21.03276	36.62383	36.95111	7.436403	9.848291	18.69004	6.234362	17.54517	0.590573	7.584823
LOC107785211	0.455781	1.022494	0.822879	19.24817	20.59958	12.77822	23.46631	25.40293	17.80753	19.58973	16.9139	13.03268	15.53369	13.11935	18.78402	0.548663	0.67742	0.784163	31.6987	24.08263	21.87116	16.95288	10.51461	11.68969	52.4827	31.51417	25.15298	42.62396	49.11839	26.01381
LOC107771859	7.545493	7.836338	9.152284	7.630699	6.987295	8.866963	8.977966	7.721496	7.413532	5.903358	6.767436	4.966291	4.785903	5.360217	9.09527	8.3036	7.204818	9.48483	4.896692	6.308748	5.36126	9.452158	8.126053	8.711884	1.774846	1.831222	1.970292	0	0	0
LOC107808678	0.454379	0.943251	0.438869	0.800419	0.982823	0.583501	1.471496	1.046073	0.734684	0.738461	1.389496	0.255914	0.913287	0.619631	0.997496	0	0	0	0	0.010677	0.011511	0	0	0	0.015958	0	0	0.902625	0	0
LOC107767541	38.58045	34.37347	41.44136	10.64511	10.39737	16.10957	11.70835	14.96382	23.2055	12.61312	9.496485	9.196601	9.516344	8.627448	6.407961	28.91721	25.79652	31.58002	8.934367	10.83366	10.54121	16.95202	16.83169	25.06463	8.864941	3.162999	9.422529	4.579332	8.323619	3.959315
LOC107819731	0	0	0	0	0	0	0	0	0	0	0	0	0	0	0	0	0	0	0	0	0	0	0	0	0	0	0	0	0	0
LOC107796381	16.14247	17.62591	17.13987	20.51822	20.74803	20.69724	19.48033	19.78768	18.88675	20.61263	20.41176	17.52067	23.72933	21.87402	25.20553	21.85808	18.19057	20.59341	21.76525	23.31001	17.43579	25.33018	21.0741	21.16556	21.5319	20.66913	15.19505	28.58249	24.47988	50.79806
LOC107801690	39.82722	73.20721	31.61239	17.26855	5.850769	14.32858	21.57664	20.21464	22.64832	6.649062	11.2287	5.018704	26.56722	27.33207	23.5934	53.89235	87.33303	38.01151	4.298236	4.806769	3.721821	26.27211	27.88548	5.561117	0.06531	0	0.366323	3.694175	3.97909	12.77601
LOC107780063	0.019328	0.017615	0	0.01751	0	0	0.016211	0	0.018146	0.018848	0.094871	0.081646	0.06455	0.210863	0.059514	0.014403	0.018673	0.01441	0	0	0	0	0.051147	0	0.024437	0	0.022845	0	0	0
LOC107771195	0	0	0	0	0	0	0	0	0	0	0	0	0	0	0	0	0	0	0	0	0	0	0	0	0	0	0	0	0	0
LOC107770150	7.336416	9.454136	8.419894	12.54554	11.18084	11.48867	11.80356	11.25541	10.07935	9.664511	12.76873	8.383677	12.92529	10.9079	14.6493	10.0401	9.743934	9.746527	8.953677	10.10738	6.234994	12.91123	9.602076	10.00631	7.237071	8.538622	5.347396	1.787589	8.243367	15.45559
LOC107796926	0	0	0.075698	0.039446	0	0	0	0	0	0.042458	0	0	0	0.095002	0	0.032447	0	0.032462	0	0	0	0	0	0.055049	0	0	0	0	0.419243	0
LOC107801550	215.2729	156.7749	185.9248	117.555	100.0718	163.3978	140.2311	128.5567	193.2286	102.1538	110.1862	180.4158	155.8611	222.7307	101.5488	126.8587	185.2258	148.612	57.44642	51.75057	84.02186	100.5222	118.4812	157.675	41.0555	38.7419	76.05698	94.59327	70.68537	32.71434
LOC107792632	0	0	0	0	0	0	0	0	0	0	0	0	0	0	0	0	0	0	0	0	0	0	0	0	0	0	0	0	0	0
LOC107799973	0	0	0	0	0	0	0	0	0	0	0	0	0	0	0	0	0	0	0	0	0	0	0	0	0	0	0	0	0	0
LOC107768715	48.16619	39.03569	44.18848	33.48004	31.23003	24.77013	30.05612	25.79588	41.70016	12.59017	17.29367	18.58252	50.69302	53.89646	26.94009	41.94296	62.32976	43.02852	28.04666	22.13849	39.45092	36.28629	36.31407	32.36354	11.87632	8.982581	8.315407	35.93846	31.82428	9.560796
LOC107758967	14.42706	12.64453	12.19155	14.2087	12.14328	16.42961	10.4141	12.58457	14.48864	7.254682	6.2037	8.366238	6.547271	8.226067	4.591883	9.102813	9.712717	11.35562	10.45684	10.5884	9.352524	8.899871	11.48409	11.55893	4.131052	4.896145	7.426663	10.78472	5.324228	6.216352
LOC107785831	0.250621	0.256961	0.245092	0.141906	0.166324	0.505215	0.052552	0.080301	0.117647	0.122193	0.065901	0.026466	0.034874	0.068354	0.032154	0.256799	0.211856	0.093424	0	0.026501	0.057143	0.052325	0.055266	0.065487	0	0.04359	0.11108	0	0	0
LOC107828636	2.950972	2.330855	2.650725	2.450646	2.828816	2.062227	3.135136	2.857576	3.139903	2.0143	2.138198	1.994451	2.792304	2.1999	1.867758	3.151998	3.516095	3.043437	1.646944	2.038661	2.24278	2.094769	3.644134	3.495603	1.492371	0.889647	1.802036	3.517271	4.025326	0
Novel02451	0.077946	0.497268	0.338784	0.353076	0.689718	0.754214	1.078716	0.998989	0.256127	0.228022	0.628542	0.197553	0.433853	0.467694	0.560012	0.087128	0.188256	0.174336	0.27683	0.197811	0.142176	0.13019	0.068754	0	0.295643	0.054228	0.046063	0	0	0
LOC107810338	0	0	0	0	0.07816	0	0	0	0	0	0	0.074624	0	0	0	0	0	0	0	0	0	0	0	0	0	0	0	0	0	0
LOC107791886	2.243708	2.210943	1.8118	2.476368	2.962954	1.457152	2.856276	2.491237	2.234806	2.398914	2.946923	1.943657	2.535763	2.745981	3.530304	3.403439	2.068583	2.581345	3.21289	2.639889	2.00475	3.614396	2.270438	2.428452	5.788655	4.627468	1.978816	0.814502	5.318756	4.929558
LOC107807875	7.787003	7.692921	8.236591	4.891394	4.871765	5.657627	4.235136	3.651588	4.326498	2.120788	3.060727	1.696065	1.879325	2.455655	1.982481	6.970261	6.259288	8.40216	3.534164	4.194237	1.830953	4.686826	5.392965	6.358573	1.096041	0.740677	1.851508	0	1.317987	0.940395
LOC107786422	4.684369	5.184059	5.420549	6.035016	6.271388	5.723033	6.174115	5.457889	5.625875	4.923788	5.140315	6.424489	7.178685	5.973214	5.775733	4.669508	5.906652	5.669474	5.308963	5.866786	4.799316	4.72493	4.80269	4.451001	4.922569	4.401738	4.457999	15.22721	3.953961	13.16553
LOC107811984	114.0267	109.3683	118.9102	76.473	73.56428	108.4418	70.8563	76.71413	91.16553	57.55972	55.46979	60.47757	46.19937	68.35271	49.76353	71.46126	121.8371	91.32005	57.58064	58.68191	68.3496	64.71825	91.10168	80.50662	25.21604	20.36976	46.27276	24.01214	23.60098	20.76102
LOC107821703	0	0.079342	0.327936	0.28919	0.077034	0.187196	0.219057	0.297538	0.190712	0.367867	0.305225	0.220646	0.290742	0.189952	0.32763	1.405637	0.028035	0.519242	0.559488	0.270031	0.396991	0.751277	0.895892	0.940259	0.403581	0.44416	0.720267	0	1.955927	7.177218
LOC107789889	8.279182	9.841078	9.386503	6.696224	5.444233	8.545975	6.095575	5.34516	6.803676	4.711072	5.298665	4.535076	5.401191	5.473366	4.937465	7.646681	8.018332	8.404426	5.111917	5.658784	3.540004	6.862434	7.393843	7.401785	4.620275	2.068407	4.734048	7.382526	8.747097	1.276594
LOC107760073	12.98302	14.19308	14.67718	28.1302	46.34906	19.32163	19.74042	27.95119	13.47999	66.54339	46.39299	55.78762	48.98315	45.84059	37.54747	14.03584	13.65485	15.71113	65.25482	55.26536	76.81893	17.59896	15.85211	15.90948	166.0046	138.8685	165.9591	102.9092	114.387	81.06685
LOC107830588	3.585999	2.643934	3.292358	0.83956	0.748716	1.082974	1.149021	1.136081	1.702261	0.432189	0.649816	0.612718	0.627953	2.241793	0.454902	1.891622	4.67107	2.222922	0.2862	0.306759	0.404218	1.312311	1.848097	1.221286	0	0.112127	0.142866	0	0	0
LOC107805868	11.38824	13.83863	11.20352	18.19371	22.56503	17.90483	16.3305	16.19121	13.04857	17.28435	12.85827	16.91451	17.5509	17.83446	15.23913	11.79445	12.185	10.54455	26.68441	21.64985	29.93031	15.79989	15.41881	12.92689	24.1131	19.76478	23.62733	28.46091	24.76059	13.25017
LOC107831665	0.280683	0.314841	0.243992	2.542846	1.967829	1.810611	1.267651	1.568074	0.790553	0.968482	1.650219	1.459254	1.153699	0.918634	1.63987	0.402241	0.33374	0.370231	2.015633	2.410914	2.067592	1.586746	1.314083	2.053588	1.828938	1.922707	1.684242	1.544062	1.974988	4.005012
LOC107800278	1.011725	2.766188	1.47911	0.333299	0.325542	0.346095	0.192858	0.157172	0.129525	0.179374	0.225725	0.077703	0	0	0.0472	1.884847	2.354673	1.88571	0.559979	0.272316	0.377472	0.384054	0.649024	0.240331	0.116284	0	0.108707	0	0.885601	0
LOC107828246	13.97615	12.67015	13.60622	12.34905	10.36038	9.646368	12.5287	11.05704	13.14457	7.090673	7.950688	8.246468	8.533823	10.00352	9.133071	14.93374	14.47848	14.48115	9.179553	8.882786	7.935643	14.16267	13.50198	13.01054	6.482155	5.830609	6.788103	7.051094	5.814855	7.620517
LOC107786485	4.164081	1.997393	3.810261	0.529466	0.517144	1.021047	0.306368	0.312097	0.480105	0.854843	0.358579	0.370309	0.975898	3.347332	0.449884	0.70772	2.187867	0.762509	0	0.061799	0.066627	0.122019	0	0	0	0	0.172688	0	0	0
LOC107800882	5.033957	4.688884	4.896395	4.5806	4.179657	4.029287	5.133596	4.566418	4.143145	3.37341	3.88746	3.278611	4.048612	4.088608	4.005911	5.40387	4.263356	5.356745	3.465415	3.770637	2.973061	5.389254	4.714133	4.774744	1.514011	1.820523	2.804499	1.585899	2.028502	1.371177
LOC107823161	0.852859	1.813644	1.482745	1.996005	2.012442	1.375392	2.503654	1.943216	1.467953	2.772151	2.342266	0.900649	2.057065	1.007963	1.750702	0	0	0	0	0	0	0	0	0	0	0	0	0	0	0
LOC107816861	8.527552	5.919089	7.780018	2.176543	2.055805	2.753287	2.989404	2.842284	5.378672	1.132753	1.073723	1.226741	2.233645	2.102567	0.677434	7.751604	6.121361	6.593849	2.62542	2.54602	3.057936	1.366991	4.564351	4.966994	1.235023	0.771439	1.372974	0	3.431815	0
LOC107762579	0.056232	0.102496	0.02444	0.280187	0.621968	0.181368	0.707458	0.936894	0.739098	0.438664	0.630874	0.950121	0.469484	0.613459	1.789153	0.062856	0.108649	0.041923	1.569149	1.212986	0.948758	1.174013	0.496	0.764053	4.016804	4.538048	1.163072	6.032035	4.602224	1.738443
LOC107824171	2.914423	4.54818	3.643997	3.002007	3.9213	2.231983	2.913251	2.524274	2.923599	1.4015	2.239556	0.573386	0.799983	0.827539	2.458589	4.760178	3.856955	4.464711	2.997867	3.883851	0.946686	6.334836	3.556741	3.58862	1.312365	1.44432	0.660611	0	0.192206	4.937072
LOC107820978	6.943831	6.657671	6.140797	7.018014	6.570556	6.127724	7.372692	7.544852	7.046737	6.301439	6.52955	6.883625	6.34483	7.750531	7.415331	7.507585	8.375711	8.289057	5.539146	6.009818	6.698953	7.642579	7.788913	8.180633	3.805974	4.188656	5.645311	2.870417	4.251223	2.481777
LOC107822836	0.471359	1.100813	0.896311	0.427027	0.364953	0.348403	0.568314	0.528599	0.774432	1.00545	0.681692	0.447994	0.655904	0.48209	0.907106	1.448936	0.540755	0.571054	0.657666	1.171291	0.698567	0.492055	0.545698	0.338707	0.335217	0.450905	0.452651	0	0.992814	0
LOC107789074	0.211493	0.3855	0.306411	0.063867	0	0.227381	0	0	0	0	0	0	0	0	0	0.157605	0.068107	0.105118	0	0	0	0	0.062184	0.147368	0	0	0	0	0	0
LOC107783199	0.233491	0.212798	0.202969	0.634592	0.550955	0.334708	0.261118	0.133001	0.511493	0.227683	0.491172	0.394519	0.866417	0.594363	0.159766	0	0.075191	0	0	0	0	0	0	0	0	0	0	0	0	0
LOC107805653	0	0	0	0	0	0	0	0	0	0	0	0	0	0	0	0	0	0	0	0	0	0	0	0	0	0	0	0	0	0
NitaMp106	0	0	0	0	0.29428	0	0	0	0	0	0	0	0	0	0	0	0	0	0	0	0	0	0	0	0	0	0.196535	0	0.800555	0
LOC107830125	0.789492	0.525806	0.950246	0.880295	0.886678	1.012031	0.280153	0.467005	0.456129	0.858684	0.660052	0.590025	0.845071	1.722594	0.498654	0.520446	0.498699	0.520684	1.109246	0.462362	0.664643	0.228228	0.428544	0.444326	0.153563	0.50701	0.753671	8.68613	0.87713	0
LOC107825053	0	0.286283	0.068265	0.426867	0.138978	0.422149	0.065867	0	0	0	0.165197	0.132689	0.087421	0.171346	0.161203	0	0.227601	0.117095	0.079687	0.265725	0.358105	0	0.069269	0.08208	0.099286	0	0.371266	0	0.378073	0
LOC107805629	6.941279	5.789217	5.544069	4.780148	3.920972	5.20463	5.070037	4.158137	4.593006	3.221965	3.196925	3.159303	3.393093	3.995871	3.049532	4.924515	5.468616	5.671514	2.910979	4.203461	2.639681	4.556191	4.767095	4.417265	0.647667	1.140461	0.726556	3.663466	2.712883	0
LOC107807046	2.109252	2.162614	2.215513	0.875818	0.855436	1.133852	0.44228	0.525642	0.825108	1.028394	0.616254	0.519737	0.684848	0.671151	0.180407	1.244355	1.443381	1.638059	0.26754	0.669106	0.400765	0.146791	0.620167	1.286011	0	0	0	0	0	0
Novel01636	2.615929	2.67688	2.114394	3.215301	3.262302	2.615079	3.374539	2.653388	2.915542	2.521026	3.411137	2.003225	2.724761	2.670268	2.889034	2.530791	1.832594	2.052932	2.406093	2.821118	1.841648	2.465694	3.090056	2.6862	1.179807	0.936576	1.13909	3.282033	1.546633	0
LOC107769013	0	0.026819	0.05116	1.066369	1.458173	0.759299	1.727709	1.483442	1.436615	0.086085	0.103171	0.223745	1.539643	2.118813	2.597449	0.065787	0.056858	0.131634	1.552736	0.697006	1.905481	1.621958	1.479515	0.707409	0.111613	0	0	0	1.416712	1.819505

LOC107782733	3.191892	3.431147	2.845788	9.416536	10.42854	9.063139	7.482367	7.272637	5.839701	11.4923	8.665683	7.674935	10.9331	12.85734	6.55214	5.001162	3.637109	4.393275	11.12857	11.42359	8.285322	7.655149	7.219127	8.126554	26.69653	22.54806	16.34776	5.852959	31.52185	20.242
LOC107765418	0	0	0	0	0	0	0	0	0	0	0	0	0	0	0	0	0	0	0	0	0	0	0	0	0	0	0	0	0	0
LOC107785642	0.100057	0.250771	0.130466	0.090646	0.044268	0.053787	0.104902	0.021373	0.023484	0.12196	0.0877	0.021133	0.194923	0.354761	0.231065	0.149125	0.193327	0.130544	0.025383	0.275084	0.13688	0.06267	0.154449	0.05229	0.126502	0.069611	0.206954	0	0	0
LOC107778081	0	0	0	0	0	0	0	0	0	0	0	0	0	0	0	0	0	0	0	0	0	0	0	0	0	0	0	0	0	0
LOC107803153	3.859561	1.722864	2.464924	2.568906	3.275798	3.556715	2.114077	1.749807	2.810082	2.765064	1.380779	3.393753	1.929049	2.148261	0.485063	1.995696	2.435044	2.877467	2.55766	1.998933	2.442439	0.92092	2.292751	4.116339	0.796679	0.109598	1.582627	0	1.896051	0
Novel00908	0.018761	0.017098	0	1.206731	1.195248	0.22187	1.007064	0.593096	0.704533	0.219528	0.434115	0.459636	0	0.347938	0.019255	0.069902	0.018124	0.041961	1.751406	1.41245	1.403023	1.034056	0.959792	0.705911	0	0.052208	0.177389	0	0.270961	0
LOC107783729	30.30964	68.25417	36.32377	4.798659	3.25485	1.370956	30.19376	27.53168	10.95879	3.49121	10.69535	0.745818	6.551672	3.156817	6.392946	62.24156	30.46619	37.47904	0.597206	1.866981	1.16297	24.04266	24.22598	4.562276	0.24803	0.614179	0.463735	0	0.472237	3.032509
107803967	0	0	0	0	0	0	0	0	0	0	0	0	0	0	0	0	0	0	0	0	0	0	0	0	0	0	0	0	0	0
LOC107793434	0	0	0	0	0	0	0	0	0	0	0	0	0	0	0	0	0.12635	0	0.265425	0	0.059639	0	0.057681	0	0.24803	0.09099	0.231868	0	0.314825	0
Novel04396	3.000477	4.414369	5.924456	2.524118	2.996379	3.087628	2.696389	2.30732	4.507168	5.559104	4.057592	6.119941	5.248844	5.985597	5.587288	6.292628	2.940124	6.455294	2.000788	1.704218	2.580115	2.326793	2.571002	4.569743	1.300634	1.491052	2.330441	12.26152	3.714523	5.300687
LOC107770662	0.04144	0	0	0.037542	0.036669	0.044553	0.034757	0.035407	0	0	0.029058	0.035009	0	0	0	0.061762	0	0	0.0841	0.035055	0	0	0	0.043313	0	0	0.048978	0	0	2.562278
LOC107798499	5.361907	6.638559	6.529786	7.790516	7.497313	6.444506	8.506614	8.406361	8.738255	9.963815	7.838771	7.478589	7.601964	8.47085	9.37121	6.78515	5.571	6.411132	11.47207	12.10974	9.088297	9.61052	8.544435	7.877691	9.720946	15.8012	9.057617	5.42621	14.36824	18.76612
107807146	0	0	0	0	0	0	0	0	0	0	0	0	0	0	0	0	0	0	0	0	0	0	0	0	0	0	0	0	0	0
LOC107778422	7.370017	15.4128	11.09714	5.007646	3.843012	3.183617	22.73927	15.68663	10.87318	4.106684	13.61177	3.446754	5.787036	5.958456	17.69525	17.99658	13.98582	11.67615	2.60415	6.123196	2.160508	17.42055	11.78278	6.740247	1.663918	2.105905	1.32217	0	1.26721	0
Novel03957	0.831425	0.833515	0.216822	0.301291	0.29428	0.625718	0.13947	0.142078	0.234173	0.972893	1.515788	1.404822	0.370221	0.816339	0.341341	0.247831	0.160645	0.247945	0	0.351664	0.151655	0	0.220011	0	0	0.231373	0.786142	0	0	0
LOC107769260	106.1731	102.9903	81.4399	150.5419	123.2037	92.28173	143.392	135.5389	131.4566	148.7126	211.6169	154.5167	210.8597	202.2734	255.6677	171.4663	190.2743	172.3645	127.5792	120.2406	135.2921	154.1851	125.7925	132.3949	189.5375	139.0998	146.8358	140.4008	114.1781	116.5356
LOC107764451	82.33775	67.43446	76.66371	85.42742	78.2037	117.5845	75.4334	78.81296	89.93263	86.54525	68.47876	90.6611	82.92345	108.8517	60.02583	51.93632	81.10962	68.25858	64.9698	71.49611	80.60329	59.92004	86.41664	84.23665	59.45193	48.09733	72.06728	53.44914	65.36755	38.51034
LOC107812366	1.499161	2.31857	2.369434	1.975511	2.251125	2.63746	2.514821	1.940793	1.791336	1.949161	3.153637	2.072508	3.337773	2.081559	2.844259	2.166645	1.623869	2.269245	2.397113	1.575626	1.160099	2.010769	1.602859	3.086362	2.182576	1.769916	1.825581	6.497647	1.093559	2.80895
LOC107820645	0.634357	1.156276	1.260418	1.067291	1.20283	1.948604	0.380045	0.851733	1.020964	1.06042	0.571901	1.837448	0.807057	0.790917	0.465062	0.405192	1.138135	1.148569	0.919574	1.073245	1.405036	0.983855	1.039155	0.852467	0.916594	0.252189	1.392409	0	1.745154	0
LOC107761403	0	0	0	0	0	0	0	0	0	0	0	0	0	0	0	0	0	0	0	0	0	0	0	0	0	0	0	0	0	0
LOC107821269	8.829731	8.319998	10.234	5.875184	4.414196	6.97229	4.895409	6.009916	6.556857	2.82139	4.162588	5.183791	2.610061	2.829976	2.150446	5.762082	7.662778	7.401158	3.239702	3.333775	3.366734	4.665995	5.016262	7.247501	0.882986	0.277648	1.945701	3.567518	0.240166	0
LOC107774874	0	0	0	0	0	0	0	0	0	0	0	0	0	0	0	0	0	0	0	0	0	0	0	0	0	0	0	0	0	0
107814941	0	0	0	0	0	0	0	0	0	0	0	0	0	0	0	0	0	0	0	0	0	0	0	0	0	0	0	0	0	0
LOC107769045	6.944732	7.5093	17.90608	3.145788	2.60388	2.341171	3.949048	6.637774	4.917643	10.50233	5.488674	3.480485	5.175821	10.65827	12.02075	14.42922	4.036889	8.775581	4.538768	4.430967	4.186692	3.882868	3.789636	5.105648	3.646037	4.585872	2.712852	8.417738	2.550082	3.639011
LOC107830849	0.951623	1.383969	1.372843	2.256192	2.04244	1.719697	2.241661	2.698775	2.395141	2.645652	3.265406	2.069763	2.65967	2.054259	3.844535	1.840774	1.388802	1.856712	1.499826	2.021083	1.144884	3.567809	2.232432	2.158572	1.151943	6.169808	1.340118	2.895936	1.657121	1.251921
LOC107820922	0	0	0	0	0	0	0	0	0	0	0	0	0	0	0	0	0	0	0	0	0	0	0	0	0	0	0	0	0	0
LOC107773759	7.519867	5.90813	5.52252	4.46344	3.728575	4.042388	4.132327	4.375768	6.694811	3.160698	3.181944	3.669401	4.546439	3.606845	2.062618	6.280136	5.636501	7.249629	3.486414	3.290322	3.842982	4.168645	6.061208	10.50227	2.54077	1.713824	2.375209	0	3.745171	0
LOC107801990	24.63764	42.53049	25.69463	134.4314	122.3311	209.3513	52.99126	35.89405	22.20071	33.55745	42.03192	13.11373	10.11048	7.386171	15.11821	27.93378	34.29918	27.89733	87.20041	134.9126	69.33755	50.639	61.70032	21.40185	6.179788	10.84508	6.479721	18.8948	3.657012	26.54688
LOC107793129	13.92924	14.78414	13.8742	18.66588	19.25861	19.56193	21.46762	19.26559	17.70896	24.08112	19.65648	19.04915	17.02455	16.84237	17.09634	14.20775	13.17647	14.30079	18.99315	20.59602	16.19722	19.43626	18.63455	17.53127	28.61755	15.5456	23.01425	39.43046	32.4125	8.971522
LOC107803530	5.078257	3.695099	5.375625	2.07771	1.377064	2.465692	1.133537	1.049758	2.037803	0.758765	1.005086	1.660743	1.413297	1.74246	0.840675	2.258386	3.244306	3.236467	1.246709	0.900744	1.531368	1.60747	2.311927	1.540972	0.103556	0.28492	0.968079	0	0.197166	0
LOC107816396	12.21577	21.13375	12.04396	7.377915	6.176755	4.491527	18.98374	11.56709	6.826612	9.410935	21.50697	4.154896	11.09683	6.548021	27.32836	30.00907	15.99073	16.48002	3.890433	6.016811	4.847055	16.98077	12.54765	5.002152	2.874941	4.525271	2.31259	1.89091	1.527561	6.539565
LOC107829389	0.043111	0	0	0	0	0	0	0.036835	0	0.042039	0.090688	0.036421	0.047992	0.047032	0.132744	0.096379	0.041649	0.160705	0	0	0.039318	0.180015	0	0	0.054505	0	0	0	0	0
LOC107818514	3.89258	4.236462	4.237884	4.930224	4.681723	6.297814	4.057319	4.359223	3.796449	4.201129	3.126976	2.809643	4.417415	3.215882	2.366111	3.407683	3.687539	3.099312	3.643131	4.028152	2.791825	3.597967	3.466848	4.187024	4.013572	2.05081	4.734717	8.107995	2.911109	4.673477
LOC107785509	0.756525	0.549246	0.81368	0.836388	1.043847	0.813357	0.677549	0.514925	0.938987	0.67519	0.728281	0.649966	1.227576	0.867298	0.407979	0.630651	0.619379	0.650059	0.806704	0.629121	0.631492	0.556835	0.712546	1.045359	0.502556	0.374672	0.363723	0	1.543298	1.585664
LOC107786280	3.121178	3.245726	1.287018	0	0.141632	0.086042	0.604123	0.27352	0.375679	0.156079	0.168352	0.135223	0.311818	0.218273	0.164282	1.550603	2.590083	1.402149	0.081209	0.10155	0.291956	0.467847	0.388256	0.33459	0	0	0.047295	0	0	0
LOC107766614	0.033837	0.092515	0.029414	0.183928	0.059883	0.072758	0	0.028911	0	0	0	0	0	0	0	0.27737	0.032689	0.075681	0.103007	0	0	0.028258	0.029847	0	0	0	0	0	0	0
LOC107809828	7.966407	11.13416	9.690615	9.891892	10.99045	11.57488	11.65574	11.35177	13.50082	8.562451	5.978679	6.816251	6.063321	6.747294	7.941395	8.041879	8.925647	8.899454	7.722185	8.439938	6.453061	11.37166	9.159662	10.53447	1.995085	2.2311	2.76754	1.820162	3.308416	1.573722
LOC107772570	0	0	0	0	0	0	0	0	0	0	0	0	0	0	0	0	0	0	0	0	0	0	0	0	0	0	0	0	0	0
LOC107785953	7.531413	8.148178	7.771801	10.87291	8.771065	11.17935	8.599163	10.25455	8.302514	7.249316	7.972654	11.90449	9.628162	11.39694	10.12385	5.50379	8.167872	5.796117	8.529857	8.796169	7.046034	8.318609	6.94322	7.059254	4.730282	4.732637	8.269803	13.89942	5.146425	3.004378
LOC107822407	17.7127	15.10624	18.74041	23.50588	19.26817	26.2064	22.76165	23.41869	24.25766	14.68394	13.97742	17.16076	16.64274	18.96912	14.34372	13.11879	16.95497	16.9209	18.90803	17.36646	23.76211	21.3064	25.70517	26.26965	11.16288	9.345858	20.16673	7.747453	17.21152	0
LOC107768019	0	0	0	0	0	0	0	0.01886	0.020723	0	0	0	0	0	0	0.022655	0	0	0.032913	0	0	0	0.05841	0	0	0	0	0	0	0
LOC107805528	7.063019	7.846649	6.184548	5.324497	5.246203	4.655936	4.843026	4.757388	4.549788	5.278615	5.820201	4.660385	4.820896	5.174434	4.233162	10.18097	6.026569	8.263816	5.754577	7.762915	3.808533	4.736031	6.366474	5.065204	2.607242	2.725925	1.767078	0	7.446107	6.375432
LOC107788325	1.92772	2.278452	2.251757	1.58269	1.625815	2.072535	1.793706	2.084606	1.866385	1.938513	1.520683	1.628591	1.44182	2.267348	1.731239	2.267038	2.502519	2.784569	2.292324	1.987438	1.895465	1.936898	2.072334	1.82596	1.523265	1.802158	0.961204	0	2.755217	1.862403
107766435	0	0	0	0	0	0	0	0	0	0	0	0	0	0	0	0	0	0	0	0	0	0	0	0	0	0	0	0	0	0
LOC107817413	2.109767	1.188635	1.367146	2.206731	1.306802	1.17537	3.683887	2.425373	4.915842	1.440102	1.573512	0.972213	2.199164	1.464688	0.728364	2.529814	1.760274	2.087695	0.622792	0.697662	0.857118	3.331634	3.248211	4.891375	0.557726	0.133436	0.544053	0	2.123772	1.18591
LOC107793116	0	0.046551	0	0	0	0	0	0	0	0	0	0	0	0	0	0	0	0	0	0	0	0	0	0	0	0	0	0	0	0
LOC107760338	25.6952	27.12779	25.801	47.9912	62.63251	43.33766	29.23345	34.27248	24.46879	51.6833	23.05208	42.17421	37.0691	40.21341	45.86961	22.22358	22.96672	24.00403	55.5895	49.54649	66.0238	33.09697	24.98371	31.93839	72.87135	55.49777	82.78989	44.47364	67.77283	90.88689
LOC107783286	0.1821	0.118544	0.146989	0.070703	0.092077	0.111874	0.152735	0.055568	0.109905	0.050734	0.036482	0.032966	0.028959	0.04257	0.080101	0.155087	0.087962	0.18425	0.145186	0.055016	0.201666	0.173802	0.229463	0.244711	0.049335	0.018098	0	0	0.125242	0
LOC107832656	3.00437	2.868499	2.59609	4.098912	4.272532	4.422113	3.074871	3.269889	3.32432	5.54539	6.345078	5.046134	6.27095	4.194573	5.322886	2.691963	2.539665	3.359828	3.901517	4.175306	2.47908	3.509652	3.170599	3.102784	5.697656	4.279894	4.22729	1.278898	4.563081	5.528712
LOC107777698	1.804054	1.700868	0.811151	0.338148	0.220186	0.33441	0.130443	0.159459	0.029202	0.121323	0.065431	0.052556	0.103877	0.067867	0	1.112593	1.62267	0.881206	0.252501	0.210497	0.141839	0.025976	0.548722	0.03251	0	0.043279	0.036763	0	0.299495	0
Novel02405	3.268497	2.329599	2.841235	0.265739	0	0.495569	0.316319	0.143215	0.550776	0.081723	0.176298	0.070803	0.139944	0.27429	0.086018	1.842379	2.226543	1.187161	0.127563	0.106343	0.038217	0.20997	0.369619	0	0	0.049527	0	0	0	0
LOC107768494	14.77027	18.57158	15.63326	18.33709	14.70342	15.73281	21.79452	23.13689	19.83749	16.40328	18.02872	18.02426	14.46329	15.88984	12.91391	15.18534	14.07114	12.28639	13.18376	13.65154	13.28364	15.64723	18.99969	15.152	9.59642	7.231173	12.12311	19.5608	9.547088	25.36856
LOC107814643	0	0	0	0	0	0	0	0	0	0	0	0	0	0	0	0	0.338483	0	0	0.148193	0	0	0	0	0	0.207052	0	0	0	0
LOC107799551	0	0	0	0	0.101033	0	0.023942	0.024389	0.053598	0	0.020016	0	0	0.155705	0.234381	0	0	0	0.057931	0.024147	0.052067	0.023839	0.025178	0	0	0.079436	0	0	0	1.76497
LOC107778779	1.797707	1.997487	1.370046	2.744132	2.353408	1.270852	3.408073	2.819524	2.057688	3.121765	3.21182	1.976459	3.289697	2.552259	3.917714	3.358309	1.689154	1.854342	2.448911	2.333185	1.280188	2.879228	2.085299	1.544353	1.556745	1.233556	0.640334	1.761116	1.185589	1.52267
LOC107826263	0.679454	1.0133	0.805412	0.111918	0.163971	0.597679	0.051808	0.15833	0.173973	0.060232	0.433122	0.313103	0.137523	0.067386	0	0.64442	0.537063	0.644715	0.313392	0.104504	0	0	0.108968	0.258242	0	0	0	0	0	0
LOC107811224	0.093136	0.152788	0.178115	0.050626	0	0.140186	0	0	0	0	0	0	0	0.040643	0	0.013881	0.035991	0.027775	0.018902	0.063029	0	0	0.01643	0	0	0.022016	0	0	0.089678	0
LOC107791938	75.28439	70.79506	74.46424	100.8594	94.43689	107.0837	98.78523	89.66678	93.51016	70.59747	72.03652	96.75235	86.06347	87.49089	76.82866	54.04281	82.51949	61.48411	82.99838	79.06134	106.2392	87.17972	93.86727	84.27107	48.98864	45.96817	60.67485	122.9602	66.81302	45.56229
LOC107813470	34.78303	31.3264	34.82955	24.77245	23.78737	24.93068	27.15164	26.51967	31.88521	15.70827	21.22424	15.1696	18.50382	17.68602	20.95684	28.17596	30.67915	30.21602	20.97943	20.13682	17.17144	35.90326	38.78107	44.77376	14.65878	12.56351	17.03853	7.337696	33.59038	95.16316
LOC107792156	1.423461	1.970596	1.628962	3.199473	3.619276	2.886441	4.488527	3.895059	4.61822	3.092374	3.828277	3.881744	5.435837	4.20666	4.567941	0.013427	0	0	0	0	0	0.015048	0	0	0	0	0	0	0	0
LOC107826833	0.604847	0.551244	0.630937	0.219184	0.356806	0.260114	0.608774	0.689064	0.795	0.275241	0.254472	0	0.628436	0.175962	0.455252	0.480782	0.428511	0.751565	0.28642	0.272885	0.257428	0.707173	1.138166	1.180081	0.050981	0.112213	0.142976	0	0	0
LOC107769503	0	0	0	0	0	0	0	0	0	0	0	0	0	0	0	0	0	0	0	0	0	0	0	0	0	0	0	0	0	0
Novel03704	1.293714	1.358572	1.000075	0.583992	0.49514	0.466842	0.42803	0.378661	0.57998	0.226989	0.345283	0.173967	0.259133	0.361391	0.183783	1.070826	0.605457	1.044617	0.199868	0.223423	0.081653	0.358892	0.635722	0.650361	0.033958	0.012458	0.0582	0	0.021552	0
LOC107761364	0.042585	0	0	0.15432	0.037682	0.045784	0	0.072772	0.079962	0.124578	0	0.467703	0.094813	0.650417	0.131125	5.045789	6.212269	6.572054	2.895237	2.26952	2.990556	6.472646	4.995871	5.385711	3.015074	2.192404	5.838541	6.090884	6.150605	2.633105
LOC107783488	0.059931	0.05462	0	0	0	0	0.100534	0.102413	0	0.05844	0	0	0.066716	0	0	0	0	0.134043	0	0	0.054658	0	0	0.12528	0	0	0.070834	0	0	0
LOC107772956	0.820571	2.742113	1.545493	3.469179	3.267427	3.087748	1.605912	1.16853	1.155582	1.60032	2.397436	0.34662	0.761226	1.193603	1.544054	3.465111	1.18911	2.95689	1.52654	1.966748	1.122561	2.626907	2.292026	1.28649	0.864542	0.95147	0.969849	0	0.65842	0
LOC107770052	4.146379	4.203076	4.523838	1.226582	1.010844	1.592094	0.851693	0.867619	0.913617	0.536349	0.237342	0.214468	0.1884	0.73853	0.130277	2.900709	2.7795	4.006074	1.202131	1.037949	1.157623	1.484035	1.604769	2.122674	0.160478	0	0.50007	0	0.203695	0
LOC107803209	0.398166	0.296033	0.255034	0.113911	0.092717	0.202773	0.404266	0.214865	0.275443	0.071522	0.095514	0.026556	0.093314	0.022862	0.075281	0.648085	0.323926	0.578075	0.180751	0.265911	0.114674	0.743792	0.480601	0.613293	0.026495	0.029159	0.049537	0	0.050445	0
LOC107766861	1.77729	2.444952	2.85672	0.303799	0.178037	0.360529	0.112505	0.429783	0.125932	0.130799	0.047028	0.113321	0.186651	0.036584	0.240927	1.624312	1.943787	2.725093	0.136111	0.056735	0.061167	0.224039	0.325369	0.105148	0.211984	0.04666	0.039634	0	0.161444	0
LOC107759780	4.8716	4.476555	4.58476	6.164202	7.232021	5.497279	6.28099	6.398441	5.934434	6.634891	8.525806	5.748322	7.44	8.257513	9.462954	7.620675	5.01754	5.523017	6.986075	8.718891	5.544546	6.522889	5.433489	5.470523	7.329937	9.131335	3.473737	5.758484	8.722419	12.44704
LOC107780630	0	0.052115	0	0	0.050599	0.046109	0.035971	0.024429	0.067108	0.02788	0.050121	0.036232	0.015914	0.171556	0.029346	0.03196	0	0.053291	0	0.012093	0.013038	0.011939	0.01261	0	0	0	0	0	0	0
LOC107782132	0.265214	0.453206	0.403454	0.150169	0.117339	0.106927	0.055612	0.056652	0.031124	0	0	0	0	0.036167	0.034026	0.222342	0.384329	0.543753	0	0.028044	0.06047	0.110744	0.087726	0	0	0	0	0	0	0
LOC107801038	9.992447	8.989375	8.882362	6.979302	6.217905	10.25793	8.218881	7.35354	9.320824	6.003498	9.605973	6.481198	6.315409	6.575926	4.532479	9.392051	9.746965	9.468447	4.611903	6.380593	3.233744	8.560307	8.927728	8.624807	2.567451	1.210973	5.790841	2.305165	3.414059	0
LOC107767572	1.090717	1.803167	1.345031	0.620456	0.426456	0.845402	0.212751	0.21673	0.214328	0.296815	0.249008	0.150006	0.056474	0.276725	0.182241	1.323168	2.058437	1.985661	0.694957	0.793926	0.786548	0.296568	0.313237	0.291632	0	0	0.11992	0	0	0
LOC107796291	3.533309	1.751324	2.15539	3.874894	2.907101	3.998673	4.783256	5.243449	3.550014	4.956573	4.694322	5.551123	4.071341	0.608632	0.636227	0.092387	0.359313	0.138644	0.566109	0	0.169602	0.155303	0.492097	0.06479	0.783715	0.776265	0.219794	0	0	0
LOC107786368	140.1159	108.769	124.0131	110.1974	90.57379	129.7974	88.7816	82.98265	113.379	113.2914	111.0279	137.6418	143.6691	151.9071	111.5171	98.85766	128.3355	110.2658	57.58064	64.74136	59.78038	72.8758	77.58155	87.4056	28.92699	24.29421	73.1726	13.00657	24.34187	22.49111
LOC107773999	0.14923	0.136005	0.389168	0.135195	0.132049	0	0.062583	0.127506	0.210156	0.291036	0.10464	0.126074	0.33225	0	0.076583	0.16681	0	0.166886	0.227143	0.189358	0.204151	0.249252	0.329077	0.155975	0.094336	0	0.264567	0	0	0
LOC107802126	0.032474	0.355147	0.056457	0	0.086204	0	0	0	0	0	0	0	0	0	0	0.217794	0.094116	0.048421	0	0.082411	0	0	0.028644	0	0	0	0	0	0	0
LOC107827544	0.387202	0.646958	0.299188	0.954915	1.123037	0.601306	0.523229	0.606532	0.706848	1.237592	1.372613	0.999529	1.700206	1.290721	0.993533	0.272513	0.228598	0.224525	0.894951	1.69232	0.431609	0.718582	1.024612	0.494637	0.571131	0.209519	0.228821	0	0.41425	1.33007
LOC107763687	3.183126	2.786135	2.684835	4.853839	5.940042	1.999136	17.26134	17.12634	13.37409	8.420617	15.99976	6.869425	24.13791	11.96512	14.36218	6.247232	5.602286	6.179604	4.253388	3.039292	7.530722	15.92355	18.12513	16.70089	7.690265	4.209826	5.661924	6.761541	7.889946	5.846064
LOC107764452	3.694052	2.503423	2.511293	4.118922	3.687809	4.786236	2.860025	3.480021	3.512602	4.987616	4.649205	4.161117	4.164992	4.133363	3.451213	2.858689	3.111231	2.859998	2.595094	3.60567	2.721146	2.768589	2.882429	3.267019	3.233357	1.58154	4.422047	3.386884	4.104111	5.856636
LOC107771875	8.852447	5.510537	6.911201	4.842185	5.675395	8.296355	2.773841	3.310934	4.453478	2.052196	2.389241	2.822186	4.016242	5.10212	1.851468	6.323022	6.389952	7.272315	6.271047	4.606171	6.215134	3.738302	4.478805	5.656291	1.18257	0.976106	2.408437	0	3.05569	6.19653
LOC107803436	5.692317	4.902276	5.402175	3.21718	3.604413	3.986381	3.854555	3.25732	4.510699	3.004522	3.55203	3.000127	3.662535	3.133505	3.484023	4.164088	4.490219	5.879114	3.709467	3.0924	3.572125	2.267869	4.283973	3.875433	1.584621	1.307963	1.728258	0	4.022729	0
LOC107805881	17.72446	16.20188	17.01724	16.393	14.27926	17.69076	15.22129	15.14426	15.79606	11.14405	12.20581	14.21424	10.1895	14.25708	13.68466	15.25853	15.79435	14.87106	13.36837	12.80057	15.972	15.95099	17.59425	17.9173	12.84343	7.803593	14.69549	7.567462	11.20777	19.6286
LOC107781697	9.67115	9.461247	9.935458	5.330467	6.014307	6.653054	3.11985	4.680607	5.841487	3.000643	4.102046	2.513987	4.291366	4.869577	1.978299	6.576974	8.297758	9.151475	4.426421	2.88914	3.145684	3.417036	5.309251	6.149795	1.154323	1.411542	1.918409	0	2.11638	6.272536
LOC107820641	2.316224	2.502987	2.329843	1.588775	1.229729	2.134478	1.054618	1.300515	1.335809	0.322659	1.252906	0.782724	0.331515	0.758066	0.64527	1.282211	2.493401	1.776182	0.201458	0.727765	0.754441	1.519838	1.196651	1.348797	0	0.092082	0.195542	0	0	0
LOC107832143	861.6963	582.4198	758.9403	456.801	453.6209	783.3497	315.4199	341.6866	414.663	393.8998	387.0701	555.1347	299.0968	410.6237	231.88	414.4555	702.9521	582.4274	367.5101	340.7867	487.2864	266.5718	394.5049	469.181	256.6005	119.2901	379.7237	828.9451	377.999	108.1827
LOC107800120	5.771575	3.37439	3.407846	8.583049	7.226984	8.663792	3.288137	5.675751	5.929798	12.63648	8.781295	13.33996	11.0316	8.791345	3.241317	4.463285	5.365418	4.546517	4.088566	5.158715	5.36309	2.273581	5.66724	7.625925	7.985423	1.9698	19.17753	7.787721	16.7767	13.46661
LOC107805347	12.25352	13.38291	13.64245	11.49633	10.73242	9.381308	13.6128	11.2792	14.09053	9.858008	10.70744	8.625009	9.949592	8.159507	9.096663	13.88744	11.53687	14.10755	6.704003	7.48688	5.513813	15.36547	15.05262	16.62484	7.218126	4.214773	7.6907	0.891455	9.001957	3.853783
Novel04261	2.323564	2.783186	2.250662	2.616253	3.465899	2.105554	1.86535	2.013677	1.059568	3.43102	3.165466	2.103226	2.697479	2.245192	1.669389	1.978883	1.924078	2.845946	2.728302	3.229159	1.362297	1.330607	1.376118	1.804094	10.40782	2.124588	8.788049	0	8.629557	0
Novel02555	0.461809	0.453257	0.524961	0.289644	0.471506	0.381924	0.208567	0.440111	0.26681	0.294441	0.261547	0.345132	0.494319	0.348792	0.273454	0.211779	0.240232	0.317814	0.072094	0.22538	0.113394	0.326335	0.250674	0.259906	0.022456	0.098857	0.041986	0	0	0
LOC107780125	23.9952	16.21481	9.513518	8.536312	12.68773	2.957306	126.5471	157.9666	63.46232	181.9364	353.5396	103.5346	290.4418	198.5027	443.4244	18.27376	6.671714	9.773742	5.522991	4.010149	13.29054	94.03675	40.98851	12.29521	48.02161	67.6709	31.81898	179.9703	52.68904	47.04285
LOC107811573	4.627283	4.707569	5.004612	1.681708	1.880624	3.933638	1.534389	1.746973	1.793285	0.734542	1.565734	0.590934	0.539074	0.675042	0.607471	3.568544	3.794594	3.510007	1.010067	2.093729	0.687003	2.808404	2.301815	1.602772	0.136053	0.1123	0.222579	0	0.647598	0
LOC107807615	0	0	0	0	0	0	0	0	0	0	0	0	0	0	0	0	0	0	0	0	0	0	0	0	0	0	0	0	0	0
LOC107819263	0.834959	0.811693	3.266155	1.15986	2.438128	1.40637	1.774134	2.378039	3.240105	2.496851	1.034337	1.692952	3.37714	3.76505	2.913729	3.712534	1.532617	3.548235	3.502003	2.001234	2.893687	4.20702	2.332215	3.025356	1.900156	1.742677	1.743443	11.94227	7.369615	1.720892
LOC107825296	10.92897	13.49558	9.795665	11.33752	12.12592	10.59324	13.25119	12.33778	14.3542	10.54669	11.21715	10.50085	13.83678	12.07741	8.863325	12.6806	10.66771	12.74973	8.734062	8.047595	9.037799	10.66401	11.58806	15.68437	6.12124	5.318461	7.194791	4.049615	7.088157	5.251978
LOC107776868	0	0	0	0	0	0	0	0	0	0	0	0	0	0	0	0	0	0	0	0	0	0	0	0	0	0	0	0	0	0
LOC107799865	0.695126	0.298128	0.497624	0.852013	1.266368	0.615461	0.994584	0.978245	1.68912	1.555034	1.404924	1.865419	2.731142	2.275044	1.384948	0.335182	0.829562	0.609701	0.539397	0.518849	0.596674	0.648814	0.685282	0.940235	0.206788	0.170685	0.28997	0	1.378004	0
LOC107805823	5.901372	6.713693	6.403577	6.010028	4.501657	6.694748	6.622404	6.711465	6.457511	4.048051	5.593496	6.051539	5.482126	6.526908	4.636743	5.61087	7.038059	5.82584	3.67558	4.303581	3.971656	6.797779	6.928567	5.955419	1.286401	1.472376	2.453257	2.910562	2.54722	0
LOC107809153	0	0.014349	0.013686	0.042789	0.041794	0	0.013205	0	0.014781	0.015352	0	0.066504	0.035053	0	0.032318	0.046929	0.01521	0.011738	0.063903	0	0	0.013148	0.013887	0.016455	0.019905	0	0	0	0	0
LOC107799845	0.600664	0.812593	0.840326	1.105347	1.361986	0.877867	1.346098	1.122676	0.88114	1.079927	1.460988	0.983199	1.723908	1.095571	1.714654	0.944189	0.698164	0.79768	0.828556	1.238543	0.616292	1.238399	0.720231	0.7161	0.806886	0.718248	0.32169	0	1.129613	2.321247
LOC107786387	2.031011	1.314221	5.049371	6.329575	11.59176	7.46786	4.25874	5.726657	6.082699	10.93231	7.533731	13.24639	9.744691	11.96496	8.942821	9.444311	1.824778	6.117386	36.64332	29.39661	36.45357	10.71967	9.154491	11.65424	30.53132	37.27498	27.3896	39.21641	38.62313	47.72057
Novel01038	0.464333	0.477786	0.494778	0.759906	0.795237	0.499215	1.193495	0.627103	0.646873	0.511207	0.724702	0.265739	0.633622	0.555587	1.291375	0.747853	0.535408	0.569522	0.364779	0.481489	0.204909	1.150821	0.713449	0.391388	0.056812	0.250098	0.070813	0	0.144224	0.926142
LOC107771640	0	0	0	0	0	0	0	0	0	0	0	0	0	0	0	0	0	0	0	0	0	0	0	0	0	0	0	0	0	0
LOC107766040	8.21465	7.652397	9.82851	4.31145	4.264757	3.715203	4.169583	4.739646	6.004761	1.27101	1.891699	1.638958	1.349766	1.488118	1.213359	7.047708	6.149701	8.022699	1.66098	1.000045	1.548142	5.467963	7.112177	9.441377	0.498213	0.210887	1.182284	2.167762	2.626821	5.622777
LOC107776509	3.217689	1.105707	2.155119	0.764607	0.653461	1.361079	0.575157	0.676053	1.43617	0.617243	0.924691	1.024968	0.939535	1.783944	0.10828	0.786172	1.630719	0.825859	0.321156	0.22311	0.625404	0.748885	0.651394	1.212931	0	0	0.623451	0	0	0
LOC107759695	3.731001	5.153652	3.826057	4.938112	4.745814	3.823254	6.79656	7.471568	4.269072	4.291936	7.255813	4.013943	3.828918	3.37075	6.671529	5.843072	4.899814	5.172074	5.057809	6.509602	2.924226	8.982431	6.659112	3.777778	2.80079	5.637553	2.239323	4.169052	1.40331	7.20917
LOC107776887	0	0.059523	0.018925	0	0.019264	0	0	0	0.020439	0.106145	0.045796	0	0	0.023751	0.022345	0.032447	0	0.032462	0.022091	0.055249	0	0	0	0	0.027524	0	0	0	0	0
LOC107814924	0	0	0	0	0	0	0	0	0	0	0	0	0	0	0	0	0	0	0	0	0	0	0	0	0	0	0	0	0	0
LOC107793496	25.05735	19.67567	22.151	54.7638	71.96631	57.98887	52.24484	59.75356	54.02762	49.76545	37.87029	55.19525	76.66961	81.31401	53.61585	16.72109	17.91629	20.00062	81.16357	74.54803	82.95408	51.78309	44.32934	45.57344	92.62403	76.32977	62.99625	308.7831	120.635	109.4162
Novel02506	7.678246	7.399946	7.441725	7.315901	5.037092	9.014139	5.625814	5.957238	4.225776	3.958781	6.714518	5.96487	5.452735	5.151121	4.393287	8.418337	8.483612	7.731309	4.880791	5.748686	4.386743	5.71211	6.344561	7.42571	2.231641	1.78062	4.068135	6.311531	1.699578	0
LOC107810917	0	0	0	0	0	0	0	0	0	0	0	0	0	0	0	0	0	0	0	0	0	0	0	0	0	0	0	0	0	0
LOC107783456	37.2256	51.46205	51.36064	10.45093	11.90272	7.116561	11.13949	8.674517	14.12748	11.64329	12.93937	9.56603	46.72372	47.90229	15.94703	28.24811	55.49703	25.13503	3.131633	3.114821	3.086398	7.394304	9.424466	4.782882	0.376731	0.384996	1.257795	0	0.922216	0
LOC107785095	0	0	0	0	0	0	0	0	0	0	0	0	0	0	0	0	0	0	0	0	0	0	0	0	0	0	0	0	0	0
LOC107776753	1.352639	1.275272	0.959577	1.422616	1.733447	1.23695	1.969103	1.554259	1.795399	2.198322	2.823609	1.418582	2.751936	2.578168	2.265979	1.79584	1.517046	1.634384	0.899266	0.960109	0.708982	2.570868	2.317663	1.885037	1.847744	1.189832	0.992303	1.111871	1.871288	1.922659
LOC107786813	0.949651	1.060649	0.938817	1.24833	1.375807	1.101068	1.11668	1.113697	1.005206	0.808006	1.162053	0.865216	1.274816	1.493091	1.376041	1.366796	1.232251	1.221656	1.454908	1.409782	0.772695	1.220718	1.108661	0.943919	0.765118	0.945686	0.781286	2.663273	1.568809	6.33237
LOC107774588	26.35255	23.96768	27.99832	28.09871	24.47	33.8704	25.6959	25.75943	30.54436	18.98199	19.23885	21.80527	23.72234	26.32398	19.53433	19.83999	26.13978	24.33634	17.38699	18.94399	19.63269	23.59262	26.44921	28.90386	12.54553	6.563962	17.1114	19.38868	13.05253	13.41085
LOC107809937	222.614	215.3101	259.6814	117.0084	103.0685	148.1152	94.08517	94.414	125.2427	49.09445	61.17261	59.10354	42.73395	53.49072	47.62403	159.8768	212.2333	199.0013	72.20334	74.5576	81.85457	97.92398	142.6684	174.5539	22.60383	11.56471	40.98975	34.20907	11.8027	7.394337
LOC107792700	13.82573	19.18868	12.25878	2.540869	1.607886	2.250887	1.524077	1.620079	2.558954	2.657847	2.686792	1.935603	3.210122	2.284026	1.337947	16.24922	16.52429	12.28085	1.924031	2.105211	1.909408	2.276256	3.310128	4.293907	1.498279	0.934393	4.201946	2.824957	4.183896	0
LOC107815635	0.254176	0.338565	0.30593	0.28341	0.259513	0.378374	0.508372	0.400938	0.367125	0.30505	0.329037	0.08259	0.304718	0.405275	1.404733	0.553666	0.245555	0.174922	0.13888	0.198476	0.213981	0.50618	0.206954	0.306536	0.074159	0.217641	0.046218	0	0.282391	0
LOC107761063	0.789744	0.70176	0.755158	0.572374	0.628936	0.849071	0.496794	0.556692	0.500476	0.346545	0.567613	0.233519	0.351662	0.129236	0.324229	0.529666	0.667589	0.647666	0.440758	0.651367	0.180065	0.544117	0.766267	0.288904	0.249619	0.164831	0.233353	0	0	0
LOC107805008	2.746504	3.75465	3.410683	214.0735	288.4231	276.564	69.84879	91.60464	61.10217	195.5078	90.0679	126.9963	171.217	231.5178	88.14211	12.64567	9.191977	13.27307	372.2586	303.5711	378.4125	57.62865	45.81307	56.69525	828.7914	299.6513	832.8107	733.0447	1142.579	709.6071
Novel02588	0	0	0	0	0	0	0	0	0	0	0	0	0	0	0	0.152474	0.263559	0.101696	5.328978	4.038642	3.607723	0.968285	0.782072	0.997998	0.517375	1.043892	0.403051	0	2.298468	0
LOC107802153	5.053335	2.627492	3.449441	2.024914	2.121107	3.256289	1.018858	0.761134	1.672668	0.852861	2.31684	1.258866	1.550603	1.325226	1.496136	2.257037	2.926038	2.572027	0.871064	1.452327	0.384061	1.487881	1.500078	2.437728	0.245729	0.022536	0.650864	1.158285	0.623809	0
LOC107782489	1.353114	2.109415	1.516722	0.064519	0.22056	0.267983	0.119465	0.182548	0.133722	0.034723	0.049937	0.270746	0.03964	0.271928	0	1.326766	1.307222	1.327374	0.108398	0.120489	0.129901	0.11895	0.219862	0	0	0	0.084172	0	0	0
LOC107795404	0.374523	0.390094	0.325565	0.484713	0.189373	0.402658	0.359004	0	0.100463	0.20869	0.11255	0.045201	0.059561	0.233478	0.274572	0	0	0	0	0	0.048796	0.089364	0.094387	0.055922	0	0	0	0	0	0
LOC107831421	0	0	0	0	0	0	0	0	0	0	0	0	0	0	0	0	0	0	0	0	0	0	0	0	0	0	0	0	0	0
LOC107787937	30.51248	33.47699	37.13339	20.73207	20.52651	24.81377	20.769	20.75628	25.01057	10.52817	12.1789	9.451857	9.580414	10.4984	10.15801	28.30582	27.39907	28.66875	12.34509	13.4021	15.8405	22.90093	29.95048	34.83548	4.550114	1.959503	8.553475	2.797531	2.259969	4.83752
LOC107818622	0.657212	0.539071	0.51417	0.35724	0.639698	0.565266	0.826847	0.730001	0.555317	0.576778	0.599088	0.555231	1.755881	1.075478	1.07927	0.440779	0.761906	0.538977	1.200407	0.778338	0.539449	0.878167	1.101438	0.892993	0.166183	0.914462	0.466063	0	1.26562	0
LOC107760028	7.327226	5.594966	4.970716	9.441345	6.417899	6.49375	11.87607	9.623527	13.52584	4.10353	7.133981	7.465936	13.69566	12.50357	6.859882	5.03598	5.954731	7.197552	3.164968	2.512836	4.605543	11.39073	10.32786	11.25476	0.563342	0.275549	1.5799	0	0.715051	1.530586
LOC107822168	1.251945	1.380234	1.755305	0.457338	0.625373	0.933512	0.304856	0.276051	0.492902	0.255975	0.127432	0.136475	0.112394	0.220292	0.103626	1.414474	1.170468	0.978542	0.491763	0.580774	0.220993	0.269815	0.498715	0.506529	0.255297	0	0.214795	0	0.194429	0
LOC107775637	7.201694	6.830573	7.570571	6.486437	5.390718	5.086775	5.794542	5.580921	6.230587	5.226092	5.328747	5.093741	5.686495	5.412897	5.371818	7.582039	8.090049	8.521994	6.245604	6.021299	4.830583	5.69962	5.595256	5.010845	3.202677	4.369461	3.43937	7.485798	4.636308	2.588905
LOC107801505	0.990454	0.729085	0.761636	0.759255	1.213503	1.023903	1.022444	0.358038	0.679529	0.557202	0.721219	0.70803	1.314623	0.748064	0.586485	0.766475	0.62564	0.568019	0.6958	0.708955	0.451655	0.954408	0.873646	1.552833	0.626117	0.47705	0.585318	5.448572	0.916999	0
LOC107803353	2.130702	2.022784	2.353805	1.005369	0.824856	1.813523	0.856324	0.98612	1.541973	0.908995	0.933781	1.162549	0.790642	0.871684	0.683404	1.687039	2.015553	2.05185	0.720696	0.525708	0.526293	1.556979	2.036139	1.716653	0.056122	0.061765	0.577115	0	0	0
LOC107782003	49.0761	24.74157	22.84955	37.82392	32.32571	44.37224	28.15096	24.8319	42.16407	26.65896	17.29003	23.29898	155.6351	151.6869	15.08654	14.27183	39.1782	15.49203	11.36876	8.627021	22.48828	15.8341	29.35162	29.52552	1.45281	0.866064	4.64032	0	23.05071	2.960438
LOC107770707	1.045087	1.371554	1.562572	1.969335	1.701562	1.932586	1.367433	1.142977	1.334396	2.282766	1.582882	2.083688	2.326811	5.107821	4.419313	0.903408	0.605785	0.841489	1.399836	1.449878	0.991262	0.802957	1.06933	0.480623	1.004194	1.16333	1.086984	0	1.408798	0
107819386	0.727497	0.663024	0.632397	3.295376	1.287474	0	0	2.486372	0.683006	3.547006	2.040485	0.614609	0	0	0	1.626394	0	1.084759	0	0.615412	0	0	0.6417	0	2.759331	0	0	0	0	0
LOC107785360	4.772509	3.705175	3.48281	6.085139	3.12817	4.307532	3.409885	4.027419	5.089124	2.700353	3.305172	4.479943	5.312824	3.792442	3.447003	3.995551	3.358371	3.250617	2.929603	2.890845	3.976457	3.838031	3.949813	5.850388	3.12869	0.819827	2.85518	0	3.120273	0
LOC107781241	16.32019	16.07841	16.50963	19.21043	22.5262	20.63532	20.74956	21.38307	19.80005	28.69047	24.15674	23.91009	28.05143	28.5562	24.62455	17.08651	16.93252	16.68732	21.51712	21.75379	21.09481	20.27616	20.17396	18.94984	23.03126	28.58534	25.36795	12.32878	30.70917	17.76588
LOC107812611	0	0	0	0	0	0	0	0	0	0	0	0	0	0	0	0	0	0	0	0	0	0	0	0	0	0	0	0	0	0
LOC107793693	10.28461	12.35222	11.15792	7.511651	6.208093	5.057175	8.291799	7.288816	9.580796	6.29715	10.00676	6.600737	13.93402	11.56791	9.328419	11.34763	11.7073	11.23395	4.125905	5.327952	3.926402	7.190746	5.625865	6.666339	2.822329	2.551446	2.921109	17.10454	4.222105	0

LOC107798135	17.18599	19.70075	18.26274	18.70946	17.64173	17.55511	17.38147	16.57694	16.80586	13.65767	14.60631	15.18328	14.79623	14.57828	14.37542	16.58789	16.67215	17.60773	15.5901	16.98638	18.2808	14.35247	15.34828	17.17856	8.808778	10.44021	10.97971	7.665539	9.976903	2.209222
LOC107777806	6.870638	7.681928	8.4046	6.416959	5.45283	6.244491	7.693502	5.8402	6.583457	3.384405	5.910369	4.607701	4.021371	3.825038	4.580058	6.571556	7.116617	6.099296	3.773423	4.463923	4.231258	5.087165	7.997172	7.847485	0.985076	0.640618	1.465046	0	3.410072	0
Novel00187	3.632173	17.24829	3.157371	9.698507	4.567243	1.438695	4.97054	6.533531	1.615284	19.38685	38.47132	3.876077	3.617766	1.251323	19.81707	108.268	3.324301	9.264003	10.08712	22.80169	11.85563	4.47016	6.744878	0.999039	11.84297	21.2796	5.422657	0	0	0
LOC107773984	4.866145	5.097914	4.412729	7.645272	6.566117	8.273387	7.037442	5.497644	6.253299	13.52592	15.02023	9.260115	10.9601	10.05307	8.346253	6.469435	5.263363	6.40008	5.70885	8.137116	3.582842	7.682151	5.903642	6.74203	11.38479	6.883358	15.95486	4.62456	10.89644	6.997234
LOC107816543	0	0	0	0	0	0	0	0	0	0	0	0	0	0	0	0	0	0	0	0	0	0	0	0	0	0	0	0	0	0
LOC107819957	0	0	0.151775	0	0	0	0	0	0.163921	0	0	0	0	0	0	0	0	0	0	0	0	0	0	0	0	0	0	0	0	0
LOC107821257	0	0	0	0	0	0	0	0	0	0	0	0	0	0	0	0	0	0	0	0	0	0	0	0	0	0	0	0	0	0
LOC107766528	8.044673	10.17132	9.157584	16.04531	14.83577	11.80658	14.19099	12.11061	11.28109	8.92787	14.12023	7.497576	9.779912	9.974384	14.67881	10.63857	9.930673	10.65296	11.35071	12.54109	7.511565	15.90002	13.3809	11.43794	6.716031	10.4118	4.738986	3.652748	8.975505	12.63274
LOC107791133	0	0	0	0	0	0	0	0	0	0	0	0	0	0	0	0	0	0	0	0	0	0	0	0	0	0	0	0	0	0
LOC107781366	7.605681	9.895345	8.01318	13.02595	10.03556	12.7712	11.09036	9.713306	10.16834	10.40406	12.98414	9.967457	10.44133	9.118371	9.599221	11.17531	7.08809	10.07841	10.663	12.48128	8.970892	10.72832	10.14604	11.03935	8.800411	9.012165	7.972845	3.843913	9.057074	4.985202
LOC107825848	0	0	0	0	0	0	0	0	0	0	0	0	0	0	0	0	0	0	0	0.04674	0	0	0	0	0	0	0	0	0	0
LOC107813040	0.637667	0	0.079187	0	0	0.195877	0	0.155668	0.085524	0	0	0.07696	0.101408	0.09938	0	0	0	0.067915	0	0.07706	0.08308	1.14114	0	0.095213	0	0	0	0	0	0
LOC107816679	5.941986	6.271619	6.692541	3.249779	2.666717	7.04425	1.064302	1.073779	1.512063	0.963715	0.932548	0.74217	1.344672	1.956704	0.751379	5.537241	5.77582	6.030989	2.934282	3.736339	1.157281	2.353784	2.572182	2.308237	0.447355	0.220702	0.908513	0	0.234964	0
LOC107812969	0	0	0	0	0.076442	0.030959	0.072457	0.12302	0.135174	0.056159	0.080767	0	0.032056	0.031415	0.118221	0.021459	0.055639	0.085874	0	0.048719	0.026262	0.192386	0.0762	0.030097	0.036407	0.200337	0.034034	0	0	0
LOC107780261	0	0	0	0	0	0	0	0	0	0	0	0	0	0	0	0	0	0	0	0	0	0	0	0	0	0	0	0	0	0
LOC107810502	0.301166	0.506725	0.604148	0.482719	0.799475	0.672488	0.485771	0.435472	0.304498	0.293675	0.406111	0.528439	0.309473	0.657115	0.404219	0.466123	0.559523	0.259076	0.329111	0.215571	0.338054	0.348247	0.286083	0.435848	0.02929	0.096704	0.082143	0	0.223065	1.432428
LOC107785024	8.694616	10.34738	9.103687	13.65523	11.63418	13.69721	13.87363	11.55454	9.954742	11.83469	15.23596	8.778713	9.969519	8.791345	13.99699	12.98279	9.770793	10.53206	8.144027	10.12871	7.260155	11.74309	11.41031	9.17286	2.351146	3.041504	4.20308	0	1.806297	1.008633
LOC107769992	1.782298	1.353621	1.161985	0.852188	0.48189	0.585502	0.664396	0.613368	0.766929	3.838008	0.885239	0.961997	1.212493	1.593327	0.558953	0.977681	1.123985	1.144226	0.427019	0.314104	0.406371	0.909604	0.917061	1.241905	9.701995	0.068887	12.11256	0	27.17196	0
LOC107759441	2.118732	1.930963	1.944089	3.732291	6.561777	7.339887	17.07963	18.70645	28.40077	4.361621	3.96175	5.668218	19.78605	22.47226	4.349217	1.754313	3.752601	3.071453	3.463797	2.788024	4.723447	9.141943	7.267795	5.29019	2.381086	1.801593	4.173618	16.83548	5.66685	7.278021
LOC107825926	4.660759	5.064574	3.864507	12.50483	17.28969	11.71776	9.712807	9.434938	5.486487	10.2783	10.63398	5.512554	8.50103	8.018116	18.80352	6.759388	5.16077	4.410315	30.85035	27.2045	22.10359	11.85661	7.146985	5.171204	20.26153	21.94959	9.95793	28.20317	16.0522	6.650348
LOC107801533	0	0	0	0	0	0	0	0	0	0	0	0	0	0	0	0	0	0	0	0	0	0	0	0	0	0	0	0	0	0
LOC107809572	0	0	0	0	0	0	0	0	0	0	0	0	0	0	0	0	0	0	0	0	0	0	0	0	0	0	0	0	0	0
LOC107797012	0	0	0	0	0	0	0	0	0	0	0	0	0	0	0	0	0	0	0.04474	0	0	0	0	0	0	0	0	0	0	0
LOC107803839	2.953944	2.475822	2.384387	1.839835	1.516963	2.580379	1.659107	1.825342	1.659023	1.363079	1.960345	1.426042	2.613081	1.84149	1.407643	2.083357	2.904719	3.106803	1.391677	1.405594	0.938106	1.718031	2.326406	2.150197	0.133382	0.366982	0.685796	0	0.126976	0
LOC107808131	20.78402	20.33442	19.70161	26.70775	24.25898	23.55468	22.75512	20.44084	23.20119	20.0706	22.90836	17.88513	25.84698	22.23478	22.7451	23.43259	19.52767	20.30169	21.31165	23.34779	14.62228	25.3349	23.69355	22.47798	10.82507	11.14263	10.61575	9.604855	14.30607	19.72297
LOC107799005	0	0	0	0	0.037109	0.045088	0	0	0	0.081788	0.029407	0.07086	0	0.091503	0	11.4069	11.38472	9.41109	26.9374	26.60709	23.25453	17.58154	17.42301	16.78801	31.65388	28.12604	15.66304	11.99646	35.33274	33.70965
LOC107816427	2.224869	3.186375	13.59347	1.612495	1.349973	1.36686	4.318672	3.421778	3.640488	5.392826	1.872095	2.094446	4.245865	2.427222	1.826835	24.96436	2.947762	10.66329	1.290082	1.344347	1.681269	4.45931	3.252112	3.189164	1.205533	1.415196	1.20211	4.545987	14.38376	23.58291
LOC107769861	0.568233	0.559305	0.474195	0.277987	0.37208	0.317679	0.61005	0.388411	0.501474	0.132984	0.286881	0.086411	0.139164	0.247966	0.256616	0.609765	0.384273	0.677827	0.426687	0.365322	0.207295	0.768766	0.621512	0.878998	0.057474	0.158131	0.241778	0	0.218854	0.702695
LOC107781011	0.982121	1.316297	1.155055	0.174461	0.102241	0.269151	0.080759	0.131631	0.090398	0.131448	0.027006	0.081345	0.407312	0.273113	0.098826	1.018888	1.246477	1.464425	0.254032	0.097742	0.035126	0.128658	0.203834	0.140894	0	0.026795	0.068282	0	0.092711	0
LOC107777627	2.284476	3.123027	3.688	4.656643	3.248766	3.596418	5.406106	3.973547	4.059736	2.784565	4.862837	2.067845	3.178888	3.560358	6.029288	4.195185	3.074029	2.676416	3.891143	3.726982	1.78584	5.450929	4.030117	3.752152	2.269356	3.40573	2.314343	0	3.927957	10.08947
LOC107796639	10.98158	8.568304	9.133994	8.445705	9.85704	11.55172	9.939629	12.01559	18.32063	11.47882	7.367903	13.082	8.091266	8.101828	6.405929	8.654477	9.922207	11.78019	12.10536	6.616377	13.90625	8.90709	12.61333	21.63468	5.893232	5.496427	16.80778	5.649915	8.367792	0
LOC107765449	1.955851	1.993465	1.71024	2.505825	2.324621	1.194478	3.242085	3.025836	2.238255	2.12162	2.783463	1.71102	2.434946	2.613512	3.729784	2.932253	2.079586	2.23471	2.454405	2.721628	1.667665	4.155942	2.664346	2.15312	2.633741	3.478269	1.313131	1.655278	2.785846	2.146743
LOC107820848	0	0	0	0	0	0	0	0	0	0	0	0	0	0	0	0.020533	0	0.020543	0	0.023309	0	0	0	0	0.034836	0	0.065133	0	0	0
LOC107819537	0	0	0	0	0	0	0	0	0	0	0	0	0	0	0	0	0	0	0	0	0	0	0.078576	0	0	0	0	0	0	0
LOC107766219	2.606708	3.184548	2.930701	0.723128	0.691482	1.170221	0.402627	0.405387	0.335389	0.353793	0.606666	0.17448	0.770506	0.809902	0.441135	3.743618	2.701644	3.678749	0.79863	1.095465	0.152721	0.717874	0.792688	0.315042	0.105857	0.1165	0.244099	0	0.053746	0.345132
LOC107793154	3.037185	3.72618	2.965103	4.148469	4.630771	3.943533	4.85969	5.42965	3.72881	6.629515	8.272972	6.730549	8.244528	7.111108	10.86253	3.708353	3.814429	3.675215	4.646596	5.079209	3.579646	5.424064	4.162751	2.808181	6.468797	8.322011	4.224811	3.341564	5.736358	4.333701
LOC107767469	0.17969	0.204707	0.156201	3.459297	3.617294	2.849541	2.03464	1.573707	1.98224	1.664598	0.724495	0.910845	4.500763	4.949844	1.982615	0.234335	0.173596	0.100475	2.871815	1.59606	1.229105	1.012933	0.75287	0.939062	1.817467	1.375144	3.398074	3.212602	10.81367	19.44343
LOC107801549	1.058466	1.068949	0.770896	1.373583	1.771947	1.199493	1.271686	1.197695	1.477173	1.394781	1.464335	1.425921	1.464912	1.529243	1.379997	0.746134	0.773835	0.70382	1.85783	1.451983	1.461053	1.361765	1.185971	2.003315	1.085045	0.99512	0.879096	0	1.377252	5.306477
LOC107768361	0	0	0	0	0	0	0	0	0	0	0	0	0	0	0	0	0	0	0	0	0	0	0	0	0	0	0	0	0	0
LOC107773732	0.227244	0.069035	0.296308	0.17156	0.10054	0.366473	0.127066	0.291245	0.32002	0.295456	0.504588	0.543949	0.210809	0.702418	0.116619	0.254014	0.146358	0.197657	0.115296	0.064078	0.172709	0.126518	0.267259	0.118758	0	0	0.089528	2.708528	0	0
LOC107764681	5.445589	3.722237	4.408291	4.933428	4.848732	4.793515	5.994778	5.263547	6.230931	2.02449	2.458128	2.904119	3.106829	3.156087	2.165816	4.2356	4.603285	4.46591	1.657742	1.410769	2.265951	4.490907	5.94417	7.577113	0.215153	0.426214	1.086117	9.735916	0.163856	0
LOC107832055	0	0	0	0	0	0	0	0	0	0	0	0	0	0	0	0	0	0	0	0	0	0	0	0	0	0	0	0	0	0
LOC107804947	2.354147	2.026319	2.245366	2.39933	2.227764	3.409813	2.166492	1.368901	2.27157	1.020262	2.453167	1.90598	2.547872	2.282896	2.44979	1.3401	2.432241	1.633233	1.260769	1.714856	1.073511	1.966009	1.672747	1.845415	0.537398	0.909895	1.043404	0	1.731537	2.021673
LOC107819735	12.69812	13.07295	12.29023	13.23476	10.74195	12.86199	11.61196	11.27657	11.67319	13.09884	18.73536	13.70765	14.98649	12.57034	16.04991	14.36922	11.95509	11.98714	10.7973	10.07038	9.704368	11.61098	11.71913	12.62691	2.155437	3.72184	3.856264	0	5.094441	3.634927
LOC107828567	16.71403	16.35651	17.10947	8.150237	5.414799	9.96676	7.009389	7.452612	8.789161	3.918715	5.667815	4.166698	4.626154	4.483816	4.124657	10.44749	13.58832	12.97172	3.290102	3.785831	3.540152	7.398674	8.297905	5.775436	0.404157	1.016671	1.619233	0	0.219856	0
LOC107793667	0	0	0	0	0	0	0	0	0	0	0	0	0.059622	0	0	0.039912	0	0	0	0	0	0	0	0	0	0	0	0	0	0
LOC107795301	0	0	0	0	0	0	0	0	0	0	0	0	0	0	0	0	0	0	0	0	0	0	0	0	0	0	0	0	0	0
LOC107762442	56.6748	77.43315	62.22648	102.7337	102.8766	101.4301	115.0295	110.558	88.89763	90.60009	86.26576	81.53857	91.17445	86.76586	111.4746	76.66893	76.83491	72.3241	76.64617	78.13704	73.97561	92.64101	80.4732	75.3872	62.54137	58.52591	60.62498	35.30531	66.54943	33.57768
LOC107767335	0	0	0	0	0	0	0	0	0	0	0	0	0	0	0	0	0	0	0	0	0	0	0	0	0	0	0	0	0	0
LOC107814868	13.503	12.71202	16.85438	7.52802	6.389955	10.36955	4.81232	6.001101	7.708486	3.810268	4.09253	3.092199	4.459963	3.021766	3.553617	12.08968	14.52634	15.3034	5.922437	6.652727	2.796804	6.567752	7.37319	8.685126	0.938016	0.619399	2.338382	0	1.428753	0
LOC107824494	180.5457	189.8265	167.6953	174.2824	138.7953	173.2628	159.8414	168.8841	173.2162	178.4607	186.2829	176.1524	103.4155	119.7741	142.519	168.98	189.7011	161.865	155.3137	182.2155	170.0552	178.8315	222.7258	198.8557	44.02932	101.4019	98.80712	63.33636	34.26288	62.58395
LOC107784707	9.12177	11.20626	8.795752	12.16024	11.4267	9.178065	12.79377	11.70009	10.23195	9.338565	10.42989	6.003978	10.03388	9.265914	12.80928	13.39328	8.749601	10.27369	10.22353	11.92283	6.688959	15.11804	11.77278	9.386871	7.834559	7.325947	5.134514	3.098981	8.814384	14.06683
LOC107764079	0	0	0	0	0	0	0	0	0	0	0	0	0	0	0	0	0	0	0	0	0	0	0	0	0	0	0	0	0	0
LOC107810792	0.822104	0.874121	1.020911	1.861961	2.372878	1.557281	1.608913	2.257804	1.617162	1.889616	2.250938	2.182828	2.440473	3.33125	1.928651	1.254439	1.134602	1.19664	2.91975	2.665866	2.517094	2.239495	1.64022	2.127698	3.09342	2.369502	1.5963	4.199432	4.711787	6.051419
LOC107791872	0	0	0	0	0	0	0	0	0	0	0	0	0	0	0	0	0	0	0	0	0	0	0	0	0	0	0	0	0	0
LOC107779398	9.862919	10.54329	9.927356	17.80343	18.1984	17.46046	15.83999	16.13619	12.94972	18.65668	18.66769	15.9549	18.16164	17.25908	21.74295	10.90505	10.24483	10.39403	22.37424	24.69548	18.03538	16.80386	13.10413	11.18716	21.6892	26.75917	23.10984	12.37435	17.96998	22.92628
LOC107762916	0	0	0	0	0	0	0	0	0	0	0	0	0	0	0	0	0	0	0	0	0	0	0	0	0	0	0	0	0	0
LOC107789589	17.29123	20.83239	16.32624	17.32062	16.61899	18.65064	14.59722	14.94225	16.86728	15.46289	15.92958	16.0808	21.81534	19.66137	16.16009	17.8301	19.25803	21.96769	13.6088	13.67583	15.07755	13.00806	15.2272	19.27764	8.175795	7.980705	9.005501	4.021356	15.16027	13.90754
LOC107805394	60.05983	57.72565	61.96036	27.04923	16.49324	23.37939	30.10905	31.33566	33.86449	26.25369	33.70329	26.53655	32.37303	36.99752	24.17923	33.6961	47.68544	32.23183	7.355378	10.65768	14.1661	28.89823	37.29684	19.21117	2.182008	3.361966	8.669253	12.34232	5.816223	0
LOC107767751	0.552626	1.074455	0.424341	3.079006	2.876951	1.208086	4.542412	3.863997	2.31744	3.718275	5.483152	3.47043	2.993931	3.798195	6.645699	1.310953	0.747437	0.741611	1.934646	2.329628	1.696815	3.538264	1.949812	1.56916	2.363893	5.190339	1.785303	0	1.285928	2.847476
LOC107760773	0	0	0	0	0	0	0	0	0	0	0	0	0	0	0	0	0	0	0	0	0	0	0	0	0	0	0	0	0	0
LOC107785902	6.778745	7.588026	7.168198	5.014109	4.318761	4.527122	4.280941	4.088431	6.034744	6.50126	8.432843	6.845297	12.74863	9.744366	7.432266	8.855033	9.106737	9.370418	3.301729	3.777926	2.356559	3.769631	4.642752	4.917922	2.097226	3.018281	2.752334	2.281299	3.685868	0.986212
LOC107806252	37.14877	19.18536	26.91053	4.823869	1.643584	2.5295	3.738993	4.232122	4.068972	2.052735	0.781462	0.627686	2.61912	2.296557	0.25419	11.9961	17.82479	12.92479	0.879573	1.152261	1.581081	2.998978	4.041346	2.07082	0	0	1.170849	0	0	0
LOC107773507	0	0	0	0	0	0	0	0	0	0	0	0	0	0	0	0	0	0	0	0	0	0	0	0	0	0	0	0	0	0
LOC107815628	2.188373	1.672749	2.577318	0.255814	0.24986	0.379479	0.059209	0.120633	0.662755	26.7775	0.197998	0.119277	3.457729	3.311565	0	2.051624	2.455144	2.315713	0.644693	0.776315	1.158871	0.707445	1.93029	1.475669	274.0886	1.473368	247.2175	106.0159	196.7779	26.18911
LOC107761125	5.147571	5.464865	5.629429	6.903226	7.460909	7.617057	6.793469	6.321462	6.720836	7.664231	6.98609	6.188093	8.400402	5.615747	7.347421	6.682022	5.472067	5.790987	5.466783	6.461502	6.023987	6.640908	6.558497	5.823772	5.178484	5.622153	2.660393	3.958324	5.329514	1.140796
LOC107766737	0	0	0	0	0	0	0.042987	0	0	0	0.035938	0.086599	0	0	0	6.569232	7.179522	7.527508	3.276454	3.815314	5.235203	5.050643	6.148266	8.624577	1.036774	1.426274	2.180736	3.665258	1.973971	0
LOC107819120	10.96159	10.3379	9.800066	15.6502	13.47499	11.15105	12.80172	11.35169	12.50579	15.6275	15.68876	15.59073	20.89116	18.46765	12.99525	11.50324	8.579095	11.19816	10.87667	10.71062	12.05353	8.140385	9.332276	10.04305	8.420528	5.45413	8.404819	2.480724	13.86126	6.434542
LOC107805319	0	0	0	0	0	0	0	0	0	0.082449	0	0.035716	0	0	0	0	0	0	0.042899	0.14305	0.038556	0	0	0	0	0	0	0	0	0
LOC107792249	0.218022	0.149025	0.236902	0.962894	1.012831	1.142701	0.708599	1.001276	1.253717	0.690946	0.286645	1.036074	2.062993	1.69469	0.419572	0.385867	0.526569	0.223499	0.829626	0.553295	1.019053	0.86486	0.769239	0.996959	0.826938	0.644643	0.386527	1.948956	1.574453	0
LOC107797968	8.345987	7.527105	8.6153	12.40483	12.23154	11.44987	21.36501	19.98176	20.32354	5.00172	8.503994	6.389898	10.16189	11.80813	12.09068	7.936264	7.433009	8.523237	5.337191	5.920214	7.730613	19.7845	24.27065	16.71951	0.989237	0.483868	0.976153	0	4.813295	2.687734
LOC107832253	41.41808	38.1064	46.21732	18.85054	16.78556	29.851	21.08572	23.16251	24.83471	13.50529	13.39356	9.926173	10.08366	12.88957	7.073836	25.14185	41.53525	30.34064	12.32206	12.65991	15.92374	20.50144	28.77519	29.77485	7.63484	2.557285	13.11098	4.694102	4.42412	0
LOC107785984	1.062008	2.730831	2.340924	2.74893	1.678098	1.671905	0.890752	0.421297	0.106828	0.147942	0.239362	0.032043	0.126669	0.082757	0.077858	2.543817	1.465699	1.385601	1.039165	1.187153	0.380509	0.158377	2.308448	0.11893	0.047954	0.052775	0	0	0	0
LOC107779572	14.49797	12.84777	13.47394	12.71073	12.94699	15.01245	13.561	13.01539	16.37123	14.13735	11.10294	13.71582	18.22184	13.3384	8.845904	11.10263	15.94261	13.10013	8.203208	7.121198	10.84602	9.206265	15.91155	17.45769	5.152628	2.138138	10.4234	4.777925	10.6145	8.26204
LOC107794974	0.050904	0	0.044249	0	0.022521	0	0.021348	0	0	0.024819	0.05354	0.236526	0.056667	0.027767	0.026123	0.018967	0.073766	0.037951	0.07748	0	0.069637	0	0	0	0	0.035414	0.030082	1.820162	0	0
LOC107794661	0.465598	1.909508	1.618937	0.632712	0.755318	0.500575	1.431896	0.861942	1.602787	1.36205	2.122104	1.835633	1.03662	1.94712	3.584077	0.983065	1.199485	0.809954	0.157486	0.32822	0.707722	1.036888	0.479136	0.892178	0.196219	0.107974	0.550299	0	1.494369	0
LOC107801892	0	0	0	0	0	0	0	0	0	0	0	0	0	0	0	0	0	0	0	0	0	0	0	0	0	0	0	0	0	0
LOC107759096	26.84463	27.19344	23.74201	32.20053	29.46476	32.58205	33.81284	32.52707	36.09199	23.25822	25.38217	18.2539	27.28069	25.91877	28.41661	25.09294	30.36195	28.31222	19.93176	22.07571	20.21746	32.10476	34.32179	29.81773	14.07259	12.49416	16.47213	0	9.906868	5.783428
LOC107831022	0	0	0	0	0	0	0	0	0	0	0	0	0	0	0	0	0	0	0	0	0	0	0	0	0	0	0	0	0	0
LOC107765305	2.35076	3.186175	1.467104	2.12967	1.920099	0.453628	5.005044	3.862603	1.244973	1.410639	2.662727	0.509228	0.9394	0.723339	3.650067	4.042591	1.630485	1.617777	4.281468	6.730583	3.298358	3.271969	3.19004	1.197007	0.838278	0.838695	0.284965	0	1.741138	14.9078
LOC107810181	0	0	0	0	0.075242	0	0	0	0	0	0	0	0	0	0	0	0.082148	0	0	0	0	0	0	0	0.107506	0	0	0	0	0
LOC107832206	0.510524	1.116671	1.420121	0.925018	0.722792	1.262414	2.440731	1.090514	0.671023	0.696956	1.396121	0.258783	0.852484	0.891131	1.519565	0.951108	0.789135	1.065728	0.777067	1.122857	0.372485	1.321691	1.080758	0.640319	0.322729	0.426214	0.301699	0	0	0
LOC107808828	1.067381	0.87788	0.769438	4.339676	2.856503	3.974493	2.467419	1.89073	2.199741	1.650098	2.172325	1.71553	4.260215	2.044905	1.977299	1.435624	1.785701	1.416873	1.083104	1.982043	0.783524	2.63071	2.778575	2.74825	0.230401	0.326016	0.430776	0	0.501342	0
LOC107782740	0	0	0	0	0	0	0	0	0	0	0	0	0	0	0	0	0	0	0	0	0	0	0	0	0	0	0	0	0	0
LOC107825249	0.441651	0.362259	0.499093	0.400113	0.429882	0.474828	0.148173	0.226415	0.207321	0.172266	0.402591	0.373119	0.196661	0.24091	0.04533	0.296207	0.29867	0.493904	0.224078	0.410967	0.161117	0.184417	0.155826	0.230807	0.055838	0.307262	0	0	0	0
LOC107828204	0.205757	0.187522	0.229963	0.186405	0.208077	0.094806	0.123269	0.226034	0.22077	0.372615	0.43283	0.620818	0.425381	0.737546	0.362028	0.284756	0.227175	0.438287	0.238614	0.298382	0.428922	0.19638	0.155564	0.153612	0.111488	0.204497	0.208447	0	0.849073	0
LOC107828752	0	0	0	0	0	0	0	0	0	0	0	0	0	0	0	0	0	0	0	0	0	0	0	0	0	0	0	0	0	0
LOC107778434	0.263215	0.119944	0.457614	0.198716	0.23291	0.188659	0.036795	0.074966	0.041186	0.171112	0	0	0	0.047859	0	0.457679	0.296669	0.425182	0.267092	0.03711	0	0.036636	0.154782	0.137556	0.166392	0	0	0	0.211202	0
LOC107832070	0.181953	0.145099	0.138397	0.04121	0.060377	0.048905	0.171688	0.116599	0.234885	0.088714	0.287068	0.134504	0.227872	0.347378	0.303472	0.389826	0.153809	0.288264	0.023079	0.03848	0.20743	0.265919	0.060186	0.047544	0	0	0.053763	0	0	0
LOC107817934	58.47043	48.38043	61.69474	23.81383	24.84809	40.11643	16.50854	18.7893	24.25716	17.00479	17.17304	24.37374	18.91324	17.20607	11.71296	36.93129	71.6002	52.86509	16.13407	18.71094	22.9793	18.09715	30.02874	32.96901	7.295171	4.371173	20.45941	0	4.938552	0
LOC107827588	35.74602	27.20697	33.46719	19.80968	18.86129	24.10101	21.11194	20.04784	25.2863	13.15861	14.67604	16.47231	20.1724	18.02642	14.69811	27.37678	36.28961	29.36036	14.4755	14.39715	18.6363	20.9289	31.87043	37.70714	6.545794	4.981462	11.91302	0	7.159537	20.43356
LOC107776838	0	0	0	0	0	0	0	0	0	0	0	0	0	0	0	0	0	0	0	0	0	0	0	0	0	0	0	0	0	0
LOC107811932	1.043422	1.473973	1.632644	0.614437	0.138493	0.448722	0.350065	0.401187	0.783688	0.15262	0.329242	0.440756	0.290388	0.398413	0.160641	2.332677	2.368877	1.594726	0.370577	0.264799	0.570971	0.348555	0.736293	1.090584	0.06596	0	0.123324	0	0.50234	0
LOC107806979	4.904029	6.7221	5.817294	3.70432	3.431971	5.032132	3.396439	2.875803	4.011631	2.512818	4.056385	1.87715	2.09295	3.112498	2.84729	5.555214	5.271172	6.62618	2.6549	3.358816	1.40292	3.667269	3.606664	4.521051	0.764634	0.750047	1.36746	1.880469	0.759563	0
LOC107759944	1.091245	2.273224	1.626165	0.423691	0.206915	0.335206	0.457637	1.065588	2.853989	0.228022	0	0	1.30156	1.27553	0	0	0	0	0	0	0	0	0	0	0	0	0	0	0	0
107786467	0	0	0	0	0	0	0	0	0	0	0	0	0	0	0	0	0	0	0	0	0	0	0	0	0	0	0	0	0	0
LOC107796061	0	0	0	0	0	0	0	0	0	0	0	0	0	0	0	0	0	0	0	0	0	0.076743	0	0	0	0	0	0	0	0
LOC107801819	8.912214	6.45306	6.915444	10.77361	11.61146	12.43272	11.99219	11.3989	10.29214	14.63529	15.4506	15.28953	16.34259	13.95787	12.79493	10.4714	11.43591	11.06726	17.50444	16.09579	12.54119	12.44276	11.91231	14.40117	26.23381	24.11669	18.02977	27.37083	24.34882	33.80709
LOC107769004	0	0	0	0	0	0	0	0	0	0	0	0	0	0	0	5.740215	6.22393	3.341291	0.71058	0.157967	1.064421	0.233924	0.164714	0.146383	0.531208	0.194873	0.110354	0	5.169359	2.886559
LOC107773973	0	0	0	0.235793	1.381835	0	0.491178	2.390623	1.099597	1.459339	0.31938	1.319311	3.042255	6.317756	7.479812	0	0	0	1.980796	2.642068	1.12752	1.41284	0.918309	0.136018	15.71276	13.39954	2.691681	74.45254	9.084559	68.39532
Novel00203	0.721236	0.534071	0.528993	0.469634	0.159549	0.072695	1.890411	1.348033	1.375416	0.527472	1.216915	0.647403	1.781412	0.909776	1.087252	0.72222	0.522581	0.67214	0.160094	0.305058	0.164445	1.637566	0.755461	0.306246	0.256461	0.407691	0.266388	0	0	0
LOC107810166	61.2149	66.06042	74.99951	53.33404	41.00416	47.49428	30.92713	34.27855	34.36425	34.54942	34.83223	32.29397	35.62824	24.29347	35.06967	66.17549	85.35489	85.09293	40.25232	38.28478	43.82455	42.16261	33.6379	38.63408	7.750823	8.781038	21.73729	0	10.8509	5.574388
LOC107820136	10.11367	9.354937	9.74293	12.78644	10.91941	16.51301	20.60554	14.96122	11.44379	4.894255	10.87572	2.709949	9.36823	9.592574	13.09169	5.455685	6.999874	6.752392	3.17836	8.076619	2.718967	6.42919	10.25241	4.851527	0.954235	0.420073	1.11507	2.698771	1.635139	0
LOC107782787	0.986409	1.23774	0.683486	1.346931	1.480033	1.121989	1.918479	1.539053	1.181093	1.519481	2.506054	1.690847	1.432284	1.590792	2.362321	0.074573	0.027622	0.063949	0.029013	0.012093	0.039114	0.023878	0.037829	0	0	0	0	0	0	0
107764938	0	0	0	0	0	0	0	0	0	0	0	0	0	0	0	0	0	0	0	0	0	0	0	0	0	0	0	0	0	0
LOC107818843	5.01751	2.90999	5.385933	2.99597	6.962449	3.228489	1.91291	2.890542	3.532994	1.37144	1.572565	1.766222	2.242688	2.529585	0.70225	3.314162	3.488613	3.003949	7.637133	4.148003	6.066737	4.126765	4.056961	7.548613	3.940757	1.533814	3.010073	0	3.11101	2.350304
LOC107807404	0	0	0	0.06123	0.059805	0.145328	0	0.115496	0.063453	0	0	0	0	0	0	0	0	0	0.068582	0.114348	0.184921	0.16933	0	0.141283	0	0	0.079882	0	0	0
LOC107759318	155.8953	132.1737	158.9088	82.0944	74.91167	99.81772	73.58805	78.60042	109.9093	47.82073	57.02134	62.56109	45.70037	51.85795	39.64885	99.58411	139.7282	121.9378	43.25192	38.27248	58.16147	81.03522	116.3723	143.7801	15.23151	8.381501	34.59147	56.1884	15.13049	28.3388
LOC107829903	1.491647	2.013035	1.0473	119.3872	235.8065	81.32625	120.0102	121.6416	105.328	177.9859	104.0312	204.5619	160.911	154.6887	121.8606	3.505743	1.995309	2.673284	337.6538	239.6751	306.8242	133.6746	86.86357	92.58472	464.0391	307.975	271.6726	322.073	438.1986	271.3714
107792597	0	0	0	0	0	0	0	0	0	0	0	0	0	0	0	0	0	0	0	0	0	0	0	0	0	0	0	0	0	0
LOC107772013	4.901849	5.999122	5.052416	7.041883	7.93139	10.01317	5.109873	4.906263	5.91695	9.35499	7.218078	7.454193	5.534761	6.570003	5.246743	3.913783	4.870901	5.116351	7.390029	6.219941	8.557947	4.795433	6.300329	5.48937	10.80123	6.820566	11.83548	5.007879	7.416906	8.659678
LOC107760630	3.309575	3.553823	2.364221	4.007639	4.088344	3.34682	3.847759	4.115696	3.676311	4.137914	4.629864	3.28048	5.854699	4.772405	4.405825	4.212244	2.295112	4.311893	5.619381	4.837059	3.974709	3.270174	3.829728	2.996803	8.741477	6.611185	4.860518	3.515071	8.045615	10.1305
LOC107774580	3.563899	3.895177	3.359158	1.410242	2.029886	1.732307	2.015703	2.403406	1.987056	2.38341	2.269215	1.868823	1.96089	1.772707	2.284428	2.533715	2.189818	3.094788	2.923602	3.511507	2.154437	2.485956	2.240263	1.998077	3.418233	5.2629	2.114191	5.859062	3.418428	9.287256
LOC107770649	0	0	0	0	0	0	0	0	0	0	0	0	0	0	0	0	0	0	0	0	0	0	0	0	0	0	0	0	0	0
LOC107801524	5.069009	5.291026	5.781023	1.707381	1.475963	1.467255	2.034952	2.128532	2.135453	1.013933	1.443023	0.969954	1.302206	1.654285	1.022751	6.085862	4.750595	5.52339	0.945206	1.19112	1.126121	1.447269	2.312031	2.196232	0.301267	0.783684	0.998527	0	0.312872	0
LOC107824102	0.846884	0.308732	0.588942	0.537064	0.786847	0.682878	0.852381	0.61506	0.51681	0.330327	0.356301	0.715471	1.037039	0.785322	0.47807	0.441769	0.736345	0.50511	0.558583	0.573124	0.579279	0.671892	0.709657	0.70813	0.107072	0.117838	0.800759	0	0	2.618205
LOC107760047	12.81059	14.0758	16.28767	13.07003	12.23615	10.87677	12.4017	9.718161	10.3411	10.85749	9.570455	7.990625	6.997186	7.379707	7.188634	12.66915	14.63157	12.94273	8.564962	10.07729	8.899097	10.59848	11.19418	14.57843	9.91467	9.578858	9.976139	4.281021	6.340395	0
LOC107782063	1.125023	0.482504	1.524465	3.357411	4.040537	2.774822	8.270425	9.697321	6.46159	6.711302	5.893301	2.599763	1.989093	2.851773	10.15451	1.084948	0.447534	0.3207	8.897733	8.313312	9.294717	15.36418	6.362682	3.873467	3.430425	7.090286	4.536579	4.732651	0.796509	12.27563
LOC107806327	10.79956	2.674957	5.004662	2.079496	1.032199	2.144164	1.009958	0.691254	2.10201	0.990715	3.416932	3.751237	1.44518	1.683113	2.240048	3.995882	3.835232	6.003582	0.477293	0.620717	0.909438	1.649817	1.559995	3.932997	0.023787	0.157075	0.088949	0	0	0
LOC107791510	20.36991	20.61138	20.89661	18.74066	15.28176	17.75136	19.79115	17.67486	20.60896	14.61983	14.94877	14.91096	23.7324	19.04792	16.29716	19.30459	24.23211	20.04447	10.78433	12.22797	12.86592	16.21898	19.05571	19.50538	6.358458	6.425641	9.420884	6.786039	8.67993	1.955749
LOC107795919	2.777715	3.013743	2.874534	2.636301	2.691991	2.417548	3.32827	3.051456	3.84967	3.353533	2.59698	3.017173	2.944943	4.473374	3.394012	2.562803	2.044576	3.057049	3.623957	3.58058	3.377763	2.982525	3.150165	3.45627	2.508483	3.68094	3.75204	18.91865	5.731246	0
LOC107785450	12.49378	13.05402	16.04093	24.24765	23.82212	16.01764	27.5788	40.19882	38.77183	46.54011	49.11825	61.51983	31.13352	27.31722	30.58269	14.33564	6.666297	14.80989	37.0256	58.19514	32.89635	32.00014	40.66919	44.74855	23.79303	31.85887	47.94523	44.861	15.10029	12.92902
LOC107829720	9.657519	9.099998	9.96026	7.266303	7.0972	7.303306	7.27643	4.964973	6.30073	5.267303	5.509308	7.329217	4.099914	5.803662	4.620099	0.06099	0.079068	0.183053	0.083049	0.069234	0	0	0	0	0	0	0.096732	0	0	0
LOC107804547	44.22998	34.97281	39.83659	57.95327	70.77382	83.04099	46.74983	46.92419	61.48015	84.70216	68.48944	115.8714	85.7096	85.38053	53.29878	30.61029	42.9277	35.26528	59.0109	47.73943	66.7826	36.83501	48.83946	65.83375	67.00061	72.42674	97.8244	134.7233	88.92169	81.03129
Novel01596	2.640996	2.647637	2.448813	4.811802	4.024708	3.659663	9.746517	11.08211	9.20164	9.41417	10.9466	10.51137	8.721982	8.035339	6.294723	0	0	0	0	0	0.026763	0	0	0	0.0371	0	0	0	0	0
Novel01841	1.523239	0.537385	0.427135	3.116078	2.913121	5.494112	1.277606	0.923643	2.12206	1.820752	2.136192	3.736087	1.039294	1.661779	0.403461	0.256317	0.759524	0.659403	1.047072	0.997591	3.495455	0.779672	1.516965	2.516527	0.186371	0.06837	0.058076	0	0	0
LOC107808519	0	0	0	0	0	0	0	0	0	0	0	0	0	0	0	0	0	0	0	0	0	0	0	0	0	0	0	0	0	0
LOC107811995	0	0.05319	0	0.026436	0	0.156865	0.073426	0.024933	0.027396	0.028455	0	0.024653	0	0.031835	0	0	0	0.021756	0.177665	0.024685	0.053227	0	0	0	0	0.040603	0	0	0	0
LOC107774079	0.335768	0	0	0.060838	0.059422	0.072198	0	0.057378	0.126093	0.065483	0.094176	0.056733	0.074756	0.146522	0	0.100086	0.064876	0	0	0	0	0	0.059234	0.070189	0	0	0	0	0.323301	0
LOC107771946	1.322395	0.823064	1.233642	0.876602	0.799122	0.970942	0.541049	0.799191	1.029556	1.289503	1.062962	0.980953	1.113058	1.161172	0.529667	1.586335	0.90363	1.178273	0.687302	0.57297	0.735394	0.592587	0.910392	1.247322	0.815566	0.538541	0.953027	0	0.15528	0
LOC107792961	2.316646	1.968196	2.389268	1.209457	0.833866	1.773025	1.152669	0.805182	2.064378	1.225233	1.046245	0.99517	1.442448	1.370765	0.725413	0.351126	0.493135	0.556203	0.318749	0.066431	0.071621	0.295122	0.519517	0.902879	0.049643	0	0.046408	0	0.378073	0
LOC107824415	57.06137	62.98036	57.65302	37.31117	33.11076	37.53094	44.07543	38.52266	44.27152	26.86168	35.44417	29.43438	51.43657	46.26275	35.48097	61.53332	67.54748	56.32599	19.38097	20.54775	23.08117	34.9893	41.20513	33.86249	5.361394	5.098007	6.25502	4.852194	18.12915	10.48808
LOC107811992	0.203586	0.132531	0.2781	9.248277	11.29774	10.60004	5.000721	4.075385	5.269878	5.700418	3.895161	4.05417	7.122806	7.868767	3.910445	0.390118	0.252876	0.238515	5.548282	5.855471	3.633902	2.331702	2.360145	2.523058	3.3829	0.809357	3.471835	0	9.941373	1.79829
LOC107812513	6.703449	5.97713	6.40736	9.701002	7.754801	6.832636	10.49041	10.36424	9.726307	10.04559	10.70322	11.54717	11.53276	11.14382	10.15654	6.920059	7.373217	6.274176	8.30398	7.094458	7.463427	8.942619	8.318994	7.946701	7.264455	4.764625	11.21559	6.225862	7.26487	3.588609
LOC107810565	0	0	0	0	0.054337	0	0	0	0	0	0	0.103756	0	0	0	0	0	0.045781	0	0.051946	0	0	0	0.128364	0.155273	0	0	0	0	0
LOC107815483	0	0	0	0.230133	0.314688	0.436971	0.170449	0.173636	0.190791	0	0	0.085843	0	0	0.104289	0	0	0	0.15466	0.300842	0.231674	0.127285	0.224066	0.212405	0	0	0	0	0	0
Novel04808	4.803384	4.516667	5.037087	6.009384	6.071929	6.311831	4.668298	5.407044	3.865396	3.717386	4.97201	4.637751	5.43207	5.157077	3.834493	3.409036	4.124865	5.115895	6.653591	5.804761	6.605919	3.629391	5.178436	7.411208	6.554917	3.819176	4.956298	0	7.708401	0
LOC107764945	14.074	16.7305	19.89392	10.64376	11.26239	16.71019	11.49653	14.11655	12.5239	11.09848	10.29777	11.06297	10.89904	12.9508	10.30003	11.94716	21.8729	16.33222	11.67343	11.80211	10.04535	11.85577	13.92549	11.6402	11.91412	8.854897	12.87353	0	6.481129	15.1342
LOC107831410	0	0	0	0	0	0	0	0	0.057516	0	0	0	0	0	0	0	0	0	0	0	0	0	0	0	0	0	0	0	0	0
LOC107831899	11.33028	12.62877	13.26808	6.300378	4.194694	10.9773	1.267093	1.313042	1.271593	2.336685	3.561462	0.836187	1.304796	2.216416	1.176276	8.967408	7.95158	11.9426	6.528288	10.62022	3.943323	1.283381	3.561101	2.368481	0.922064	0.579872	1.908671	3.725404	0.626989	0
LOC107799267	0	0	0.014807	0	0	0	0	0	0	0	0	0	0	0	0	0.038081	0.065825	0.0127	0	0	0	0	0	0	0	0	0	0	0	0

LOC107787679	34.34373	24.41507	24.23237	33.4371	41.26768	39.14153	51.57879	45.68361	55.50995	55.42616	47.47954	53.88414	118.3576	86.64318	58.5679	21.52138	33.20586	23.85893	46.16446	47.39885	37.50003	33.75645	34.47354	41.02406	40.77759	28.19886	42.70017	17.94201	70.99547	58.60376
LOC107761474	3.67287	3.287055	2.444897	14.98844	13.46846	16.6845	9.824598	9.612504	15.34628	14.64871	10.34808	21.80446	13.11328	16.02768	4.754619	3.698685	3.452401	2.688942	7.588255	6.913765	10.20005	4.006846	8.259812	11.13622	1.589709	2.117878	3.637085	2.366326	3.823244	2.045938
LOC107821229	0	0	0	0	0	0	0	0	0	0	0	0	0	0	0	0	0	0	0	0	0	0	0	0	0	0	0	0	0	0
LOC107804869	3.59267	3.56625	3.600438	6.696134	6.256819	5.191084	7.830813	6.569496	5.113145	5.846284	6.193659	4.137135	5.757114	4.942971	7.586218	4.80884	4.089822	4.930466	6.385594	7.472055	4.7166	7.051738	6.479238	5.524958	4.918336	5.158141	3.840555	0	4.406725	2.829812
LOC107773274	0	0	0	0	0	0	0	0	0	0	0	0	0	0	0	0	0	0	0	0.091929	0	0	0	0	0	0	0	0	0	0
LOC107822454	0	0	0	0	0	0	0	0	0	0	0	0	0	0	0	0	0	0	0	0	0	0	0	0	0	0	0	0	0	0
LOC107821301	0	0	0	0	0	0	0	0	0	0	0	0	0	0	0	0	0	0	0	0	0	0	0	0	0	0	0	0	0	0
LOC107800433	2.434442	0.823064	1.945547	1.209457	1.007588	1.477521	1.119736	1.241322	1.953786	0.76577	1.459237	0.895653	1.704712	1.499274	1.209021	0.555949	1.85874	1.141681	0.597654	0.996469	0.644589	1.18049	1.039035	1.641599	0.248216	0.109269	0.27845	0	0	0
LOC107792614	0.837629	1.221434	1.100291	0.168633	0.098825	0.120074	0.296635	0.365799	0.489318	0.145208	0.313251	0.031451	0.103607	0.142149	0.210154	1.165178	0.755272	0.957549	0.056665	0.2047	0.169763	0.808341	0.492563	0.603115	0.070601	0.1036	0.066001	0	0	0
LOC107797706	2.113981	2.925628	1.565396	1.844228	1.108498	2.188611	0.13134	0.200694	0.441044	0.992525	0.329406	0.066146	0.435799	0.427083	0.160721	2.742261	1.210242	2.743517	0.635592	1.192189	0.071407	0.19616	0.483433	0.818345	0	0.108943	0.092539	0	0	0
LOC107786173	0.273014	0.213273	0.339037	0.247337	0.379627	0.251592	0.523403	0.399893	0.512636	0.114096	0.218786	0.09885	0.217088	0.042549	0.160123	0.319708	0.226076	0.407088	0.395766	0.296938	0.177853	0.19543	0.240817	0.774534	0.098621	0.054269	0.046097	0	0	0
LOC107762932	0	0	0	0	0	0	0	0	0	0	0	0	0	0	0	0	0	0	0	0	0	0	0	0	0	0	0	0	0	0
LOC107786094	1.944903	6.083031	3.573446	0.24027	0.195566	0.095046	0.370744	0.264374	0.082998	0	0.061989	0	0.344447	0.530448	0.181472	1.218766	2.6903	0.922732	0	0.186961	0.12094	0.40606	0.038989	0	0	0	0.104487	0	0	0
Novel04037	2.298053	1.516629	3.0998	1.507597	2.454186	2.470688	0.864041	1.218736	2.23191	0.927266	1.611396	1.405884	2.117152	2.074811	0.731998	1.121991	2.067001	1.358821	2.010263	2.011029	1.662236	0.463247	1.188262	1.15955	1.001875	0.110261	1.03025	0	2.28903	9.799454
LOC107772477	2.031974	1.594686	2.723125	2.505622	2.372393	1.790175	2.509114	1.929081	2.967543	1.018235	2.750696	1.239816	1.696516	1.631799	1.332442	2.691963	2.56288	3.008804	1.088228	1.360804	1.132507	3.016807	2.339993	4.011656	0.820663	0.706836	1.200815	0	0.95109	0
LOC107815613	0	0	0	0	0	0	0	0	0	0	0	0	0	0	0	0	0	0	0	0	0	0	0	0	0	0	0	0	0	0
LOC107789351	9.843783	6.63938	5.966343	3.436281	3.0633	6.537678	0.757468	1.054565	1.413116	2.025463	2.153063	3.382471	3.418165	3.875265	1.544861	7.470197	12.88347	10.66057	5.37623	4.278175	3.871116	1.156443	1.646293	3.398109	0.685075	2.136215	1.992463	2.152812	0.289856	0
LOC107806821	0	0	0	0	0	0	0	0	0	0	0.024502	0	0	0	0.358647	0	0	0	0.070916	0.118238	0.191212	0.029182	0	0	0	0.048621	0.0413	0	0	0
LOC107809373	6.509624	5.346346	6.974649	4.731626	4.487553	5.45243	4.634568	4.462534	5.329815	2.952421	2.494581	2.973573	3.159856	3.468262	2.680289	4.794572	5.265078	5.727905	3.648396	3.61777	4.280081	4.108874	4.373205	5.419351	3.205928	3.422955	3.220682	8.119711	4.008559	4.680231
LOC107778799	0	0	0	0	0	0	0	0	0	0	0	0	0	0	0	0	0	0	0	0	0	0	0	0	0	0	0	0	0	0
LOC107789323	2.982609	3.959963	3.873073	2.00155	1.417353	2.078397	2.702391	1.856258	1.901373	1.597836	2.194686	1.399884	2.869379	1.908139	2.021948	5.666369	4.251029	4.900291	0.78466	1.46401	0.772399	2.413976	2.35478	2.424661	0.139664	0.333031	0.500492	1.316659	0.975017	0
LOC107791679	8.89233	7.858679	7.135305	12.99476	15.11342	14.99793	11.96122	12.34429	9.185308	14.00723	13.88046	12.64339	10.59131	11.28402	10.80754	5.560762	4.966208	6.166	18.13078	23.32088	13.47878	11.63268	10.45816	8.535988	13.6274	8.450576	17.02672	5.929392	14.46986	1.281646
Novel02816	5.373734	7.887546	5.654698	23.8292	25.87739	23.71783	27.09054	26.14718	14.28559	59.1852	55.05342	32.35261	57.36534	46.89983	58.11546	10.49604	9.672544	9.952608	23.30395	30.24159	9.492338	21.35222	13.57134	10.99676	85.5333	43.83976	60.43783	56.63338	66.17547	24.48276
LOC107784255	0	0	0	0.126206	0.061634	0	0.058422	0	0	0.067921	0.048841	0	0	0.075989	0	0	0	0.10386	0	0.058922	0	0	0.061439	0.145605	0	0	0	0	0	0
LOC107808045	0.628284	0.485468	0.736121	0.730052	0.592204	0.73422	0.698806	0.618513	0.62833	0.586019	0.670407	0.323091	0.410526	0.298012	0.546724	0.74301	0.488219	0.722984	0.595961	0.693241	0.286503	1.003767	0.746949	0.728061	0.155415	0.342083	0.177574	0.976765	0	0
LOC107785253	3.588632	3.573829	4.090498	4.629076	3.575018	3.398298	3.607941	3.08653	3.12373	2.062542	2.466357	2.28889	1.931316	2.463092	1.756263	4.020234	3.604678	4.695374	2.001617	1.568128	2.319202	4.465669	4.045856	5.514475	0.811273	0.39682	1.123569	0	0.457668	0
LOC107762849	10.80511	9.260591	7.464363	10.82612	9.371121	11.15522	11.3434	9.599025	11.9582	7.745265	7.225322	8.342567	7.089589	7.103936	5.508315	11.1448	9.885608	9.49609	9.003783	7.74814	11.09441	6.633267	11.48749	14.21037	6.96618	6.173118	9.979812	10.23468	14.81355	4.42448
LOC107791170	3.070937	3.696503	3.777595	8.608805	8.04956	7.101626	6.123427	4.356651	3.807909	5.19809	6.663175	6.020997	7.482241	6.194787	7.790609	3.238395	3.638508	3.326275	7.408265	6.175906	8.77215	3.774346	2.964314	2.725253	1.684901	3.06365	1.643593	8.287375	5.02118	3.582654
LOC107811210	12.49086	15.69195	13.16509	19.73737	17.31518	14.99074	25.48278	20.78098	19.98296	20.49771	24.25744	15.94049	20.29895	18.95668	24.08207	19.2855	16.68491	16.7841	15.47513	17.77078	13.23433	25.64857	19.07728	16.00879	12.97101	12.32776	10.58081	6.609845	15.19279	8.980568
LOC107831153	11.17812	10.66559	11.7164	21.16758	21.92475	21.04207	19.76654	20.37752	17.08672	20.42288	19.35473	23.31917	21.29344	21.21979	17.35433	10.43498	14.34669	11.82371	25.83861	27.44605	26.7195	20.18681	19.96884	15.64811	26.78549	23.47067	24.32467	43.28845	28.36481	22.45645
LOC107824752	0.058525	0	0.101749	0.106041	0.15536	0.188764	0.196349	0.300031	0.494512	0	0.041038	0.098887	0.586356	0.191543	0.180205	0.087226	0.22616	0.174531	0	0.148524	0	0.34213	0.103246	0.12234	0	0	0	0	0	0
LOC107761001	0	0.060581	0.115565	0.06022	0.117637	0.071465	0.501775	0.056795	0.062407	0	0.13983	0.056157	0	0	0.068225	0.396279	0.256869	0.297345	0.134902	0.449844	0	0.333074	0.46906	0.208429	0.084041	0.184981	0.078564	0	0	0
LOC107769372	9.091556	9.03909	9.340024	6.270963	4.75375	5.22049	6.672295	6.797063	6.983635	4.331964	4.129265	4.538654	4.485375	4.621092	4.983775	7.544929	10.47996	9.165895	3.459556	3.75802	4.899613	8.886789	10.47984	7.018886	1.69805	0.287505	1.465294	0	3.481704	0
LOC107801692	119.5227	56.34878	59.64927	153.488	171.1832	209.1959	55.37461	62.91894	80.00179	202.58	114.7824	305.1733	213.1893	275.9263	86.61182	50.079	111.7607	60.44812	132.1893	90.76739	144.5524	42.31353	48.80683	71.53566	98.40266	46.096	187.3313	118.2469	83.01584	25.55923
LOC107777336	0	0	0	0	0	0	0	0	0	0	0	0	0	0	0	0	0	0	0	0	0	0	0	0	0	0	0	0	0	0
LOC107797063	2.002503	1.303596	1.392587	2.436166	1.822574	2.306717	0.983757	0.757725	2.068042	0.669495	0.200594	0.942558	0.382151	0.96748	0.322978	2.792672	2.017497	2.324738	0.667658	0.459795	0.913158	1.075077	0.908404	2.302317	0.036168	0.07961	0.135246	0	0.27545	0
LOC107802351	0	0	0	0	0	0	0	0	0	0.141312	0	0	0	0	0	0	0	0	0	0	0	0	0	0	0	0	0	0	0	0
Novel02022	3.627112	3.824201	2.843856	4.316875	3.775891	4.396595	3.340471	3.61562	3.438678	3.190138	3.76513	2.553571	3.008521	4.073383	3.321293	3.497907	3.091849	3.419974	3.608375	3.098368	2.789087	3.177581	3.763944	4.757404	3.641642	1.880203	3.824636	0	4.964745	8.794894
LOC107761433	5.107493	5.164505	4.537058	3.208594	3.826684	3.206528	6.410177	5.032912	7.210209	2.581109	4.496327	2.740152	9.254878	6.792175	4.13253	6.86211	7.095261	6.170389	2.269809	2.901416	1.632042	7.876981	4.899758	4.987655	1.461169	0.933727	1.454082	2.666116	3.948645	13.83083
LOC107824390	0	0	0	0	0	0	0	0	0	0	0	0	0	0	0	0	0	0	0.104629	0.174448	0	0.08611	0	0	0	0	0	0	0	0
LOC107783088	22.96335	34.01899	20.6757	18.45077	21.89064	12.19462	12.20304	11.35567	10.93501	23.41293	31.93438	16.0865	24.54532	23.53403	30.6954	41.00834	22.21013	31.79034	18.19221	14.81846	9.148195	11.39793	10.70615	8.475931	15.47962	15.9667	7.595675	8.52847	41.21046	13.92821
LOC107773285	0	0.031099	0	0.247314	0.120779	0	0.114484	0.174937	0.09611	0.232924	0.119638	0.028829	0.113961	0	0	0.25429	0	0.025441	0.450142	0.46186	0.031121	0.11399	0.300993	0.035666	0.086285	0.142442	0.322651	0	0.164283	0
LOC107828588	0.503954	0.373175	0.438077	0.313883	0.222966	0.372496	0.211344	0.107648	0.473135	0.184282	0.26503	0.266097	0.315568	0.103086	0.129311	0.399019	0.334718	0.375719	0.031961	0.106578	0.057452	0.052608	0.416739	0.230446	0	0	0	0	0	0
LOC107796637	0.290999	0.378871	0.216822	0.037661	0	0	0.13947	0.142078	0.117087	0	0.087449	0.035121	0.138833	0.544226	0.17067	0.092937	0.120484	0.061986	0.042184	0	0	0.069434	0.25668	0.0869	0.052559	0	0.147402	0	0.200139	0
LOC107791149	2.3243	2.231794	3.138936	1.165654	1.799607	1.47257	1.392493	1.950482	1.792485	0.68804	0.582072	1.016882	1.016496	1.313129	0.937199	1.91765	1.88459	2.970625	2.063722	1.860866	1.816972	2.010408	2.269849	2.385976	1.941589	1.328289	1.815072	0	1.798394	2.566339
LOC107826664	0	0	0	0	0	0	0	0	0	0	0	0	0	0	0	0	0	0	0	0	0	0	0	0	0	0	0	0	0	0
LOC107790700	6.15843	5.934522	6.715725	6.499098	6.601762	6.739722	4.832092	4.469811	5.180829	2.62595	3.86944	3.356653	2.997811	3.034181	2.831919	6.546699	5.373797	6.220567	6.316344	5.825798	4.610034	5.327405	6.561405	7.105846	2.986077	3.409174	2.817588	3.157095	2.975514	5.459283
LOC107824911	25.77054	23.69513	30.22252	10.91359	9.580155	19.59127	12.98171	14.72279	15.53317	10.18564	10.47865	10.04846	10.18513	13.05905	10.01663	16.76109	24.01261	19.95199	8.278305	8.642644	10.49993	14.13552	15.60256	13.54737	2.506292	2.440029	6.127786	0	2.2024	0
LOC107811506	2.026915	2.352856	5.54552	1.9136	1.850186	1.651591	3.972775	3.463703	4.867565	3.952997	2.019706	1.550168	3.135195	3.444918	4.248335	6.948115	2.246743	3.722205	2.749582	2.508776	2.17938	3.813097	2.822958	3.36735	4.369971	3.384366	3.253042	6.103289	11.29908	19.78851
LOC107819201	5.879361	6.94145	6.736969	7.989605	6.384819	5.890053	5.379571	6.621867	5.896153	5.081629	4.684786	6.773247	6.247487	7.580293	6.171561	4.779607	3.743608	3.586347	4.610067	4.40836	6.702595	5.579554	6.718208	6.843414	3.378773	2.974801	4.106187	0	3.859819	0
LOC107804582	0	0	0	0	0	0	0	0	0	0	0	0	0	0	0	0	0	0	0	0	0	0	0	0	0	0	0	0	0	0
107796259	0	0	0	0	0	0	0	0	0	0	0	0	0	0	0	0	0	0	0	0	0	0	0	0	0	0	0	0	0	0
LOC107830123	0.804193	0.586338	0.745672	106.6609	63.33264	128.1346	12.59088	8.382915	12.18086	10.08991	10.2254	7.156394	12.05588	9.88464	2.806365	0.319619	0.880507	0.719472	23.88275	26.34997	17.35802	5.50714	11.25504	9.694253	3.728062	1.044377	9.188082	11.50223	3.097337	0
LOC107803483	0.2752	0	0.159484	0	0	0.098625	0	0	0.086123	0.089452	0	0	0	0	0	0.13672	0	0	0	0	0	0	0	0	0	0	0	0	0	0
LOC107760999	0	0	0	0	0	0	0	0	0	0	0	0	0	0	0	0	0	0	0	0	0	0	0	0	0	0	0	0	0	0
LOC107823786	0.922708	1.114727	1.063236	1.516332	1.158252	1.061235	1.763806	1.191747	1.188615	1.46472	1.564845	1.269001	1.719915	1.662108	1.894084	0.991427	1.223102	1.375836	1.480661	1.12544	0.861095	2.311732	1.552068	1.278409	1.600661	1.642173	0.811584	1.534573	1.343004	2.653601
LOC107772592	1.461081	3.129249	2.032139	4.368096	5.171443	3.220224	8.394481	7.552744	6.035601	3.918031	3.841916	3.085905	3.496966	4.064618	6.373358	2.667559	3.175936	2.450921	4.225422	3.584325	3.331327	7.931216	4.897327	3.51238	3.971589	6.098964	0.690752	0	5.627332	18.06817
107769536	0	0	0	0	0	0	0	0	0	0	0	0	0	0	0	0	0	0	0	0	0	0	0	0	0	0	0	0	0	0
LOC107815008	0	0	0	0	0	0	0	0	0	0	0	0	0	0	0	0	0	0	0	0	0	0	0	0	0	0	0	0	0	0
LOC107820560	4.64388	3.342076	4.927712	5.628836	3.726629	9.073011	0.402933	0.383103	0.932112	1.358511	1.695517	0.703484	0.784358	0.663853	0.460199	3.281627	3.016703	4.357608	3.34735	4.212873	1.475052	0.468062	1.214738	1.071179	0.182212	0.089126	0.340677	1.145183	0.231282	0
LOC107772117	2.523968	2.219099	2.994208	0.914635	0.604324	1.819691	0.448298	0.22834	0.669067	0.289551	0.291498	0.401378	0.396666	0.647888	0.121907	2.345548	1.491706	2.722967	0.632754	0.326545	0.704111	0.24798	0.838139	1.148328	0.075084	0	0.140382	0	0.571825	0
LOC107800284	9.228196	11.63236	10.84648	0.1413	0.253022	0.61485	0.065409	0.155475	0.097621	0.025348	0.091138	0.021961	0.028938	0.170155	0.02668	6.160119	7.031718	5.639672	0.211023	0.219899	0.308201	0.130254	0.779594	0.02717	0.032865	0	0.030724	0	0.125149	0
LOC107806750	1.213873	2.483946	1.373743	4.523325	5.471905	2.043776	10.10444	9.862861	4.924094	4.332713	6.680869	2.689554	4.410848	5.99672	10.22567	2.952684	1.150577	1.502631	8.692003	7.94358	9.462337	10.57729	9.2122	4.332866	5.06742	8.349473	2.463353	9.827443	8.049306	11.32915
LOC107760384	1.671304	1.623617	1.08563	3.261174	3.396547	3.060579	1.910137	1.490777	1.189752	1.88046	2.858968	1.955027	2.412541	2.04371	2.525945	1.519184	1.011355	1.15018	4.398219	4.707514	2.311511	1.702508	1.4742	1.28614	2.69354	3.168809	1.584619	1.313427	2.917871	3.406788
LOC107831402	0	0	0	0	0	0	0	0	0	0	0	0	0	0	0	0	0	0	0	0	0	0	0	0	0	0	0	0	0	0
LOC107775360	3.795992	3.863945	4.842528	4.868162	4.44951	5.406206	5.912887	5.349502	5.484618	4.7832	6.187709	4.852088	5.872148	6.911032	7.007924	5.492237	3.619621	4.612652	5.527742	5.713342	3.934101	4.405249	4.131039	4.070624	5.890035	8.745914	3.962154	3.525547	7.832253	13.71693
LOC107798437	0.593096	0.34139	0.434161	0.622154	0.359081	0.469848	0.471273	0.480086	0.615438	0.426148	0.306437	0.448321	0.486495	0.578929	0.384466	0.395452	0.844393	0.837812	0.380105	0.21125	0.370099	0.677793	0.743424	0.84829	0.078932	0.217171	0.479626	0	0	0
LOC107811567	0.144496	0.065845	0.251214	0	0	0	0	0	0.06783	0	0.05066	0.122074	0	0.157638	0.074153	0.323036	0.069798	0.430911	0.073312	0	0.131783	0	0	0.075514	0	0	0.085391	0	0	0
LOC107771456	0.233491	0.124132	0.067656	0.193903	0.137739	0.251031	0.06528	0.199501	0.07307	0.094868	0.327448	0.164383	0.108302	0.148591	0.199707	0.289996	0.169179	0.145064	0.197442	0.131678	0.141965	0.064998	0.085814	0.061011	0.123001	0.027074	0.114986	0	0	0
LOC107781740	7.927592	5.149471	6.690814	3.812987	2.576365	6.632545	2.006853	1.995118	3.220691	2.417508	3.072518	1.753514	2.599395	2.044218	3.668895	3.54459	6.294076	5.45902	1.989154	1.755804	1.551184	2.431543	2.568213	3.284236	0.656043	0.160446	1.60138	0	0.277573	0
LOC107768981	23.25854	14.89066	19.54978	16.45632	12.84167	21.59589	12.0917	10.92179	17.59524	11.62116	12.53494	15.91444	11.341	17.3004	6.313236	13.9661	26.7408	19.05992	10.43522	6.829381	14.81346	12.92245	14.92041	22.30074	4.860473	1.604755	6.815633	13.7464	2.776237	5.942605
LOC107767451	3.418412	3.581554	3.228932	4.267393	4.07281	1.794197	7.111483	5.565588	3.638955	3.385878	5.964171	2.68331	3.056325	2.084895	4.807009	2.667754	2.210317	2.889719	9.122587	7.195199	12.78973	4.877895	6.790283	3.207191	6.295698	8.277008	6.585351	9.624598	6.868071	26.62873
LOC107764952	9.019474	22.55971	8.334095	19.09637	51.61062	8.11678	15.55032	32.42432	7.370192	34.56159	36.33051	23.50881	33.09681	36.58949	91.16767	28.92658	15.49079	13.82238	105.9976	76.60939	107.3939	62.29573	35.15273	16.48006	171.346	237.9376	93.37627	160.0605	129.6256	431.6916
LOC107829323	0.056201	0.819531	0.341983	4.022338	4.376306	1.389739	4.760997	7.587168	7.598075	5.480352	6.068909	6.077511	3.503598	4.65979	5.941415	1.21456	0.271477	0.586608	2.965527	5.039512	2.921631	6.570943	6.84113	9.339938	2.700118	3.988212	3.454131	0	4.870329	3.475021
LOC107789292	0.635227	0.747786	0.851292	1.342795	1.56917	0.825227	1.842573	1.469962	1.192758	1.316281	1.076435	1.632333	1.237501	1.559253	1.629949	0.078895	0	0.078931	0.134288	0.13434	0	0.044208	0.070039	0	0	0	0.031283	0	0.254851	0
LOC107762399	0	0	0	0	0	0	0	0	0	0	0	0	0	0	0	0	0	0	0	0	0	0	0	0	0	0	0	0	0	0
LOC107820471	0	0.041565	0.019823	0	0.020178	0.049033	0.019126	0	0	0	0	0	0.025385	0	0	0.016993	0	0.034002	0	0	0.020797	0.019044	0	0	0.028831	0	0	0	0	0
LOC107823574	29.12071	25.1296	27.47447	20.94407	17.88401	30.50167	18.44642	18.91156	19.3712	16.50739	20.48522	19.33296	18.16637	21.38414	16.98987	21.10672	27.58942	24.05311	11.37258	13.92363	12.23149	21.6173	26.14138	28.37842	5.217285	3.58867	10.75227	0	3.386412	2.89948
LOC107829824	0	0	0	0	0	0	0	0	0	0	0	0.258481	0	0	0	0	0	0	0	0	0	0	0	0	0	0	0	0	0	0
LOC107788880	181.9735	148.9978	166.6494	133.707	109.2747	175.3042	110.4531	104.2521	164.3071	71.26727	70.38951	112.9204	140.2181	206.7931	84.75287	117.4678	154.3102	141.5037	63.28139	48.06296	82.72852	85.48035	105.5589	115.9999	17.14031	15.6912	42.02506	57.29014	40.05129	19.05129
LOC107789898	3.176281	5.052722	4.969933	3.139145	2.963887	4.532491	3.148463	2.269808	2.873595	2.083621	2.915621	2.000361	2.314394	2.70913	2.489491	3.356791	3.626474	3.186107	1.582235	2.198385	0.737373	2.556137	3.565787	2.957683	0.438086	0.883913	1.36513	0	1.668191	0
LOC107801312	2.822364	1.891351	2.742059	2.632123	3.3054	3.926853	2.715362	2.482431	3.662903	2.509324	2.386494	3.787007	4.158391	4.528031	1.277999	2.226949	2.726641	3.403841	3.285108	3.370625	2.649751	2.842301	3.953931	3.557274	3.673277	2.194564	4.905599	0	7.992863	0
Novel05359	0.328057	0.199322	0.047529	1.931819	3.048007	0.99932	2.109526	2.709574	1.745301	2.719124	2.261998	3.602971	2.738982	2.624556	3.928268	0.203724	0.158465	0.12229	6.158456	5.08775	7.679299	2.46572	2.797223	2.914523	10.78386	12.47677	14.66938	23.46071	7.896915	13.52284
LOC107787257	0.071411	0.162705	0.093114	0.064694	0.094783	0.115163	0.269529	0.183046	0.134087	0.069634	0.050073	0	0	0.038953	0.036647	0.106431	0	0.133099	0.072463	0.120817	0.097692	0.149092	0.062989	0.149277	0	0	0	0	0	0
LOC107826835	0	0	0	0	0	0	0	0	0	0	0	0	0	0	0	0	0	0	0	0	0	0	0	0	0	0	0	0	0	0
Novel03146	7.258166	9.095519	9.30632	5.917977	5.67321	6.372789	4.92092	5.529737	6.7575	3.892695	3.944308	3.525842	4.376608	3.233307	1.365757	7.301871	7.421031	9.244254	3.068785	3.37695	4.247557	4.394576	7.469212	8.724167	1.835306	1.094081	1.85869	0	2.32956	0
LOC107779139	0.040573	0.036977	0	0.441085	0.107705	0.218105	0.03403	0.138667	0.152367	0	0.085349	0.102832	0.090333	0.177053	0.166572	0.06047	0	0.302489	0.247024	0.68644	0.481043	0.169418	0.107364	0	0	0.056454	0.287724	0	0	0
LOC107794470	0.799332	0.428525	0.613096	0.042597	0.499272	0	0.078875	0.281223	0.264864	0	0.06594	0.079447	0	0.153888	0	0.35039	0.817647	0.350551	0.143137	0.159101	0.128648	0.117802	0	0	0	0	0.222293	0	0	0
LOC107789931	0	0.29614	0.112984	0.058875	0	0.069869	0.054508	0	0.061013	0.063371	0.091138	0	0.217034	0.283592	0	0.145286	0.062783	0.048451	0.065945	0	0.05927	0	0	0.067925	0	0	0	0	0	0
LOC107770503	1.153739	1.22674	0.96949	2.316925	2.228975	2.29476	1.790228	1.44582	1.516454	2.306335	2.453975	1.803215	2.590123	2.538323	1.736777	2.493327	2.619331	2.867205	4.097572	4.196731	2.998854	4.22342	4.138542	3.235799	4.765012	3.772558	4.181789	5.500578	4.258459	4.755829
LOC107794587	9.435933	7.595346	15.20747	9.702834	11.27492	6.923629	12.65136	14.59456	15.38986	4.701357	6.906255	9.921032	5.942164	6.837713	6.397612	10.5475	8.350701	9.756415	9.505043	8.040414	12.75156	15.47279	18.2258	14.00178	1.65451	2.875054	4.314476	0	3.647499	0
LOC107816729	26.09812	29.13772	26.06878	38.87389	35.50072	33.70197	34.69704	31.42551	32.46265	28.13345	33.30551	26.01775	36.08103	32.79659	31.08125	34.16453	22.44699	30.70469	33.14976	31.20618	24.90665	33.78105	31.47074	34.26023	25.01381	25.53701	20.6073	10.50005	28.4514	29.5048
LOC107816487	0.91493	0.982747	0.596497	0.917689	1.040902	0.737746	1.233308	1.368047	0.920332	1.178942	1.397662	1.02141	1.9279	0.962496	1.576278	2.82463	1.673096	2.021997	2.022603	1.188594	1.370852	1.528164	1.441123	2.185794	1.81775	1.363992	1.699302	9.347208	2.359714	4.040823
LOC107825675	2.456766	2.683429	3.064852	1.240279	1.443741	1.492046	0.865136	0.753122	1.30292	0.713211	0.828467	0.649598	1.043858	0.879764	0.731444	2.515583	2.337207	2.78239	1.084722	1.348486	0.547325	0.751772	1.124871	1.117292	0.474214	0.730653	0.35465	0	0.632017	0
LOC107765670	0	0	0	0	0	0	0	0	0	0	0	0	0	0	0	0	0	0	0	0	0	0	0	0	0	0	0	0	0	0
LOC107832204	2.763265	3.432122	3.805013	4.253527	4.00307	4.837486	4.532787	4.366821	3.627393	3.004522	4.286732	2.148551	2.967216	2.13422	3.890279	4.245937	4.134253	4.120262	3.69727	3.599385	2.430935	4.656193	4.464939	4.779527	1.607677	1.871402	1.213895	0	1.412744	1.512007
LOC107794088	0.724832	0.446873	0.407699	0.309017	0.339554	0.412562	0.697352	0.49181	0.400297	0.24946	0.224229	0.414242	0.261054	0.162803	0.503259	0.746672	0.638462	0.635756	0.605714	0.504954	0.233315	0.836774	0.451306	0.490208	0.51211	0.207643	0.226772	0	0.205271	0
LOC107761531	5.209037	5.099054	6.456743	9.176074	7.724843	8.607948	7.48401	6.264008	7.199864	3.715537	4.701006	5.134196	5.691388	5.209236	4.801871	4.16932	6.150672	4.78253	5.628367	5.100101	7.961871	4.430762	6.806985	6.351898	0.914695	1.677777	2.50827	0	3.483078	11.92899
LOC107815732	7.578869	5.848842	7.278843	8.471846	7.625691	7.228251	6.869363	6.423369	8.090871	6.079162	4.414304	8.210092	6.463756	6.934597	6.022253	5.465595	7.20373	6.288313	5.705864	5.222027	7.079283	7.145997	6.415504	8.368609	3.940981	4.677414	6.429254	13.11281	7.356325	7.558272
LOC107789461	37.45153	34.60983	44.96346	11.38882	9.501557	16.75361	14.05861	18.12565	21.88351	7.278456	7.62121	9.956673	4.227466	7.714406	4.704101	20.00465	29.6029	26.84779	3.189082	2.437032	3.70227	8.748741	13.16769	16.8348	0.662239	0.364413	3.095433	9.364734	0	0
LOC107788668	12.99374	15.69736	11.75262	15.67723	18.55395	14.1657	13.0034	11.82852	9.063816	22.08463	19.23007	11.67853	24.6713	20.86586	15.60071	18.08389	13.88265	18.16762	24.8916	20.85363	13.77022	14.07979	12.74672	10.91562	32.39548	19.31896	23.443	7.237043	59.82818	26.28018
Novel01255	1.567383	1.229154	1.204064	1.023699	1.451432	2.351342	1.559215	1.245781	1.50575	1.421764	1.942507	1.170196	1.744833	1.113448	1.346835	1.602626	0.915577	1.222902	1.29457	1.325898	1.030555	2.435279	1.639752	1.523934	1.382546	1.166528	1.637112	7.820237	1.052922	0
LOC107765683	14.68364	15.00891	15.79655	11.71024	11.43771	13.28642	9.548715	11.55258	11.93233	9.782924	9.897983	7.904569	9.211576	8.90935	7.355036	14.83151	15.15224	15.72537	7.710652	7.846267	7.324757	12.33044	14.66933	16.64748	6.222384	3.762654	6.296338	7.735454	7.03017	1.672029
LOC107822910	18.00048	19.59758	19.7355	21.50829	19.54409	27.12867	25.43499	27.15764	31.51554	10.50002	10.46143	11.20682	11.95082	13.19791	11.45129	16.24179	17.60931	15.23365	16.19224	17.01053	18.16194	26.00245	27.57841	27.4584	7.422002	7.987754	9.123382	9.277755	6.089671	24.06479
LOC107767350	0.132161	0.080299	0.229769	0.518835	0.350834	1.941884	4.027514	4.140468	4.83906	1.890145	0.432466	0.33496	0.588493	0.144181	0.361723	0.032829	0.170238	0.164219	0.670539	0.70806	0.68302	4.304469	5.828732	5.525381	4.73426	0.122595	3.176138	6.300914	0.63627	2.723902
LOC107820688	1.671082	1.750557	1.87006	2.227369	2.090552	1.755315	1.997694	2.281225	2.236112	1.610785	2.12803	1.75255	2.095478	2.179299	1.30115	2.046859	1.837082	2.563326	1.305882	1.169892	1.31384	2.133448	1.965339	2.389047	1.141373	0.587979	0.953489	0	2.034414	1.187649
LOC107761677	8.065639	7.414942	16.06074	4.67312	3.630745	3.503911	10.8358	10.57765	10.47806	7.064797	9.79871	6.358484	8.613367	8.38995	11.64745	15.77762	6.274441	13.02294	4.020851	3.649499	5.303162	7.969422	7.383286	8.209646	5.573009	7.7972	4.683332	10.06059	9.030432	21.7461
LOC107826074	12.16402	13.12221	13.71836	17.54202	16.4434	18.91135	13.71238	13.57488	14.94937	13.34846	13.67691	14.95036	12.55419	14.23758	9.645704	12.13024	12.67651	12.29443	15.94181	12.32991	15.36312	11.04699	13.63863	14.27063	8.832848	7.15503	9.933889	17.75305	9.219662	10.96384
LOC107787026	0	0	0	0	0	0	0	0	0	0	0	0	0	0	0	0.028492	0	0	0	0	0	0.063859	0	0	0	0	0	0	0	0
LOC107810314	5.889674	6.32056	6.058898	12.75527	10.51565	11.35285	14.05978	12.92318	15.4433	9.141504	12.65834	12.57189	13.81126	15.36	13.77115	12.38786	13.60194	13.6147	22.20829	22.31698	23.07512	28.72591	27.85056	29.39526	21.94246	36.46555	13.05725	27.41506	24.49602	23.7032
LOC107775393	24.20326	22.92531	25.17428	17.41479	15.27637	28.78906	10.02108	10.73443	16.36587	10.17718	8.92712	11.58302	9.282235	7.417698	3.675976	14.22052	21.78751	18.79554	10.6757	13.042	7.247344	10.95929	15.86974	14.35947	1.167409	0.661861	2.612599	4.002023	3.906554	0
LOC107801984	0	0	0	0	0	0	0	0	0	0	0	0	0	0	0	0	0	0	0	0	0	0	0	0	0	0	0	0	0	0
LOC107822295	272.1807	331.4626	319.6631	407.049	311.0748	341.3	238.5634	205.0603	188.998	195.6881	164.8838	170.3598	119.6856	133.5452	187.0199	253.0822	221.8485	252.0248	249.1221	281.5672	263.8541	171.492	127.5714	153.4953	40.22516	76.78769	59.4906	41.26673	58.845	38.92302
LOC107799752	1.488245	1.385838	1.2937	1.875861	1.603179	1.878314	1.166847	1.713892	1.245357	0.946452	1.701454	1.065981	1.980879	1.905968	1.560701	1.639454	1.281491	1.49548	1.181871	1.88843	1.032732	1.648157	1.883483	1.487882	1.227127	1.53058	1.032448	0	3.426712	2.000444
LOC107788210	31.19535	30.43204	65.01451	3.15966	3.200428	7.360484	5.525502	5.297719	16.4932	3.715827	2.49085	1.036727	3.523038	3.734425	5.502066	45.86792	29.32618	43.14426	9.10979	7.485111	6.243834	17.15228	10.02666	16.20142	0.979886	0.718941	1.755733	4.618858	8.706406	0
LOC107827616	7.645631	7.008386	6.155656	6.986698	7.421332	6.982826	7.145867	6.721712	8.975165	6.408886	7.851598	6.272288	10.71601	10.61034	7.1091	7.742378	6.488419	8.603832	5.905707	4.942017	4.762995	6.653035	6.509719	6.672835	3.609163	4.557088	4.302482	1.582549	4.794198	0.68414
107775047	0	0	0	0	0	0	0	0	0	0	0	0	0	0	0	0	0	0	0	0	0	0	0	0	0	0	0	0	0	0
LOC107829425	1.840504	0.634689	0.864817	1.171689	1.27647	1.711366	1.919207	1.360067	1.821349	1.115639	1.116162	1.386811	2.32575	1.085351	1.123215	0.704307	0.816957	1.038402	1.110475	1.178225	1.224903	1.080092	1.57957	2.131662	0.691798	0.346071	0.823097	0	0.718447	0
Novel02549	1.808911	1.738197	1.264795	1.638781	1.73983	1.395183	2.259326	1.814379	1.993639	1.744743	2.57818	1.993328	1.751047	1.973433	2.159326	2.06596	1.538612	1.920317	1.137248	1.430417	0.878675	2.298843	1.994474	1.459106	1.044071	1.285842	1.254905	0	1.419903	0
LOC107825853	759.9626	1250.194	782.3179	358.706	362.7632	371.5425	437.8819	342.0801	422.0338	408.4278	509.6236	429.8638	1261.546	736.2168	689.1874	1324.669	1234.098	1130.019	135.7284	131.4263	219.9841	326.8549	324.6874	308.7628	58.75781	21.10991	117.6321	61.80209	293.3179	50.46584
LOC107759947	0	0	0	0	0	0	0	0	0	0	0	0	0	0	0	0	0	0	0	0	0	0	0	0	0	0	0	0	0	0
LOC107765588	390.5258	256.2726	355.5644	293.2012	283.722	388.671	233.6309	248.4404	320.4183	368.3389	294.1805	444.8732	290.8335	358.3763	225.6795	243.4133	328.7486	277.8345	271.4128	260.5989	381.3877	174.8555	239.0802	278.9648	204.6347	142.6829	303.5467	286.1801	259.7435	216.5038
LOC107776157	2.372272	2.652094	2.942023	2.521679	2.183108	2.788527	2.467261	2.999861	2.821111	1.758081	2.528426	1.843828	2.394367	1.552819	0.941469	2.804941	3.69746	3.466513	1.989966	2.194078	1.846231	2.086807	3.124801	3.140697	1.519632	0.924236	0.934611	4.524026	1.370515	0
LOC107762296	0	0	0	0	0	0	0	0	0	0	0	0	0	0	0	0	0	0	0	0	0	0	0	0	0	0	0	0	0	0
LOC107805930	4.864403	5.635801	5.112919	5.688649	6.132986	5.178555	3.066655	3.17833	2.88044	3.84433	3.500097	2.726292	2.866829	3.260402	2.545293	5.034665	4.085119	5.345114	4.823143	5.284887	3.175083	3.90308	3.098857	2.974133	1.346587	1.260792	2.367372	0	1.377586	0.982919
LOC107779670	0.024673	0.067458	0.085789	0.11176	0.065496	0.079578	0.020694	0.105404	0.092655	0.120294	0.121103	0.041688	0.109863	0.107666	0.101293	0.055158	0.095343	0.036789	0.075108	0.271326	0.067505	0.020605	0.065288	0.025788	0.124774	0.27464	0.058322	0	0.118782	0
LOC107804029	0	0.251251	0	0.083252	0	0.296393	0.077076	0.078517	0	0.089609	0.128873	0	0	0	0	0	0	0	0	0	0	0	0.243171	0	0	0	0	0	0	0
LOC107790455	0	0	0	0	0	0	0	0	0	0	0	0	0	0	0	0	0	0	0	0	0	0	0	0	0	0	0	0	0	0
LOC107779454	0	0	0	0	0	0	0	0	0	0	0	0	0	0	0	0	0	0	0	0	0	0	0	0	0	0	0	0	0	0
Novel00237	2.576054	1.149422	1.469546	2.503945	2.255718	1.904081	1.778033	1.857136	2.821598	1.726985	1.674619	3.423173	2.330005	3.600759	0.743622	1.159806	1.529504	1.400406	1.143626	0.930685	1.835472	0.537832	1.30181	2.019368	0.203557	0.298699	1.110043	0	1.033503	0
Novel02093	1.013233	1.043886	0.612718	0.838117	0.701668	0.994624	1.551886	1.882031	0.992628	1.20282	1.390069	0.707137	1.471233	0.913146	1.266032	0.590919	1.063988	0.624033	0.581133	0.521729	0.602665	0.294323	1.126888	0.690673	0	0.551677	0.260339	3.150457	0.63627	0
LOC107809291	38.95447	25.53075	46.49748	14.22166	12.95529	14.49065	16.00391	16.12253	42.8743	6.18489	10.74806	11.11879	10.2969	16.14556	5.750436	44.03573	46.51823	59.8577	5.685211	8.405872	8.484092	17.56811	13.38052	25.63345	0.334127	0.073545	1.624243	3.779913	2.54465	0
LOC107763703	31.86075	31.20704	31.53416	25.64837	29.8013	33.60263	20.91364	22.19241	20.88982	30.22401	29.899	32.25633	32.33548	33.20004	25.36993	29.61381	37.17875	30.49877	36.23957	34.67873	33.71622	19.52199	22.10843	22.21564	36.3958	29.87585	37.55451	24.76624	36.88838	24.08965
LOC107808589	0.211921	0.547229	0.368437	2.463866	4.406755	1.215151	6.635892	9.898542	6.66518	4.132991	5.176199	4.296878	3.853249	5.317489	9.570428	0.684336	0.272978	0.342325	8.530023	7.49948	4.928521	10.76632	10.00066	3.765493	12.45885	24.52355	9.30926	20.20711	7.311879	17.47118
LOC107826515	0.637482	0.847271	0.738866	0.866289	0.446568	0.542585	0.891139	0.839718	0.548621	0.595724	0.428378	0.673203	0.975774	0.637506	0.436196	0.435465	0.179626	0.237635	0.26953	0.269633	0.193798	0.177459	0.140575	0.305386	0.100746	0.036959	0.062788	0	0.127878	0
Novel03184	0	0	0	0.057242	0	0.033965	0.026498	0	0.02966	0	0	0	0	0.034465	0	0	0	0	0	0.05345	0	0	0	0	0	0	0	0	0	0
LOC107817502	0.200228	0.583947	0.243676	1.015822	1.133922	0.86108	1.007641	1.265997	0.639143	2.772522	1.993684	2.841864	2.808503	2.097018	1.274156	0.477474	0.580313	0.477692	2.03178	2.303561	2.264385	1.304285	0.635813	0.837115	3.240315	2.173106	4.25977	0	7.518974	2.476085
LOC107810378	17.58397	17.12791	17.24659	15.5509	14.0776	17.90466	14.96303	12.83816	17.57861	14.01666	14.46642	13.97117	19.60061	16.9249	11.45886	14.94087	15.52702	17.04594	9.369934	11.49061	9.439696	13.63592	14.38183	20.00757	5.616684	3.462895	9.181836	6.653452	7.054624	1.438153
LOC107789289	0	0	0	0.081887	0.079981	0	0.303249	0.347534	0.33944	0.308489	0.3169	0.381811	0.301863	0.098609	0.88133	0	0	0	0.642035	0.42054	0.824352	0.415169	0.358776	0.188947	2.856943	2.578246	1.655883	0	1.958217	8.383226
LOC107762334	0	0	0	0	0	0	0	0	0	0	0	0	0	0	0	0	0	0	0	0	0	0	0	0	0	0	0	0	0	0
Novel02537	0	0	0	0.088368	0.143852	0.034956	0.163624	0.41671	0.183152	0.158525	0.227987	0.604309	0.253364	0.177355	0.500569	0	0	0	0.263942	0.165027	0.207572	0.244378	0.114717	0.067967	0.739932	0.995293	0.115286	2.325198	1.408798	0

LOC107779405	2.939296	4.395989	3.341242	5.803655	4.935008	5.915064	3.603151	4.507667	4.882264	4.99745	5.548943	6.621864	7.383122	7.317688	4.641251	4.717713	4.732678	5.506519	3.594409	5.291659	6.804822	4.217019	4.121654	7.08958	4.002022	3.880082	6.146328	0	4.354098	0
LOC107764038	1.087362	1.189195	1.377321	1.238405	1.484489	1.50306	1.667688	1.380334	1.750051	1.54503	1.39421	1.154853	1.660068	1.999692	2.774152	2.106788	2.100965	1.598186	1.134905	0.972395	0.595015	2.4648	1.753828	1.721001	1.492592	1.167159	1.028139	0	2.841828	0
LOC107773678	9.782408	9.286123	7.796553	6.826155	4.678471	6.305768	6.086368	5.24912	6.89314	3.026624	5.508558	4.358276	6.362378	4.927393	4.130403	8.629792	8.809557	7.819842	2.51964	3.06021	3.728771	5.666839	5.796543	5.996028	0.433013	0.714827	1.340891	0	1.236656	2.647094
LOC107806552	7.031464	6.789424	7.889448	7.520706	9.319148	7.37652	8.49565	6.801866	9.596819	4.908334	6.331415	3.388893	5.034443	4.342384	5.007322	8.886979	5.895234	7.69018	6.113507	6.930756	3.503035	10.39915	9.303325	13.09597	1.977711	2.198116	2.892244	1.107597	2.833425	6.703441
LOC107807338	0	0	0	0	0	0	0	0	0	0	0	0	0	0	0	0	0	0	0	0	0	0	0	0	0	0	0	0	0	0
LOC107794286	10.66495	10.25294	11.0311	11.78182	9.476869	18.57803	10.60586	10.49659	9.801487	10.18027	11.61183	12.84977	10.31945	12.81316	6.604678	7.410992	12.3465	9.93058	9.680489	9.402482	8.043952	6.839652	10.39951	11.38217	5.916916	2.441943	10.47767	16.09061	6.499351	2.782405
Novel01418	0.511854	0.310995	0.444944	0.541	0.226461	0.275153	0.357762	0.437342	0.40046	0.083187	0.9571	0.216214	0.284902	0.279204	0.612912	0.572152	0.412079	0.572414	0.865657	0.793822	0.46682	1.068657	0.601986	0.35666	0.32357	0.356104	0.100828	0	0.821416	0
LOC107830104	0.052188	0.079272	0.060488	0.09456	0.015393	0.037406	0.058364	0.014864	0.032665	0.033927	0	0.029393	0.077462	0.018978	0	0.077782	0.134449	0.064848	0.105914	0	0.111059	0.029056	0.122756	0.054547	0.021994	0.048411	0.020561	0	0.083751	0
LOC107786132	4.079981	3.937129	4.485458	2.826549	2.229852	3.676901	3.12011	2.56327	5.182396	1.930741	1.935305	1.368615	2.604909	3.141924	1.539552	3.845221	3.303993	3.310193	2.252692	2.030226	2.681317	4.158909	3.440042	4.828801	0.379289	0.333941	0.992808	0	0.57772	0
Novel01927	1.86577	3.904666	3.003471	1.565086	1.40637	2.340253	1.188166	1.210384	1.362408	0.336919	1.235597	0.233519	0.461556	0.716182	1.453963	3.501682	2.870633	3.554805	1.156989	1.549085	0.535693	1.788991	1.676209	0.758373	0.087367	0.480756	0	0	0	0
LOC107789551	4.22829	4.551193	4.768726	0.379759	0.451557	0.842564	0.244583	0.389307	0.307994	0.444301	0.38339	0.323344	0.953571	0.715788	0.430239	4.454758	3.503844	4.864432	0.665779	0.53961	0.116353	0.365292	1.12532	0.533377	0.161297	0.101437	0.883179	0	0	0
LOC107777924	3.34695	5.744792	5.38245	6.317014	5.306202	5.907341	5.637855	4.81387	4.844323	3.753253	3.8528	3.016102	2.546012	2.677662	3.377964	4.863594	4.715426	4.470733	5.122682	5.709766	3.968217	6.941227	5.141802	4.343637	0.952101	2.871839	1.417504	0	1.477062	0
LOC107790181	0	0	0	0	0	0	0	0	0	0	0	0	0	0	0	0	0	0	0	0	0	0	0	0	0	0	0	0	0	0
LOC107802757	0.034897	0.127216	0.091005	0.094844	0.030879	0.112554	0.556116	0.327982	0.065525	0.102086	0.244695	0.147408	0.116542	0.152282	0.071634	0.026005	0.033713	0.026017	0.141643	0	0.063653	0.116572	0.030781	0	0	0	0.041245	0	0.168005	0
LOC107780368	0.277877	0.25325	0.362329	0.503484	0.184413	0.149376	0.116534	0.178069	0.130441	0.270965	0.194847	0.410826	0.618672	0.227362	0	0	0	0	0	0	0	0	0	0	0	0	0	0	0	0
LOC107773662	27.33569	22.4765	21.20112	24.91304	22.54689	24.34435	19.08347	16.59653	21.20733	17.34486	20.0095	17.97733	21.13733	16.96455	10.69623	22.6882	18.97622	28.23086	16.05614	15.6007	13.08732	14.99133	22.0424	29.08451	9.105792	4.251486	12.51071	15.60789	10.2446	10.121
LOC107820012	0.975642	0.611309	0.795098	1.546793	1.780581	1.507843	1.074035	1.302523	1.48846	1.248678	1.667544	2.524263	1.83279	3.791843	1.376886	1.317777	1.708375	1.772994	1.175641	0.876909	2.057666	1.01848	1.398443	3.059221	1.927356	1.527226	2.450401	4.360761	3.522815	0
LOC107776142	1.668145	1.216247	1.305075	2.266883	2.730757	2.600517	2.728333	4.275926	2.740724	1.301322	3.099717	2.959521	1.671302	3.002776	2.054566	1.740345	2.256196	0.994938	2.28517	1.622807	3.651304	3.204157	3.163541	2.52814	3.479921	3.133488	2.267356	0	2.810866	10.31439
LOC107785348	3.645816	2.3974	2.647712	5.435192	5.349539	4.118164	7.896357	6.82164	7.538941	4.950182	5.95426	7.797688	13.10046	10.06938	9.141764	2.476131	3.655918	3.30302	5.151233	5.777825	7.660232	7.977927	6.228167	8.200038	7.059997	2.632764	4.309061	0	10.2203	8.560492
LOC107827187	1.246148	1.854994	1.336009	1.392368	1.029162	1.473738	1.811677	1.455149	1.754908	0.972121	1.252446	1.193151	1.38723	1.495435	0.980576	1.423901	2.367638	1.610364	1.390956	0.597355	0.795556	1.630416	2.344928	2.040542	0.105034	0.057797	0.540044	0	0	0
Novel02889	21.2138	17.00655	18.66837	9.134781	9.269812	15.06417	6.80964	7.943957	7.130582	10.15153	12.16639	14.76906	9.62113	15.21452	9.744209	12.78346	17.9009	16.25512	7.906265	8.861935	9.673673	5.850721	6.757103	7.93836	6.042935	3.279718	8.744599	4.682367	6.619589	4.048399
LOC107782517	28.06954	24.55408	26.14313	34.3602	32.04516	34.71389	28.76326	30.15767	32.70585	27.20842	24.89586	27.41923	27.52747	29.57439	28.33821	11.26803	14.24478	13.17282	13.64812	14.23987	14.93327	13.77892	16.13769	16.41546	13.41378	13.60011	16.34112	17.92294	15.48442	23.2444
Novel03844	9.6359	8.749766	7.363765	6.523245	4.622413	5.312703	3.078874	2.985657	3.678291	3.751604	3.563973	3.429222	4.715085	5.15988	2.390995	6.654626	5.080785	6.21032	3.259282	3.224693	2.156787	2.741351	3.518108	3.357146	1.071007	1.71893	0.959501	0	2.209079	0
LOC107821147	0.108055	0.246197	0.140895	0.220258	0.191229	0.46469	0.022658	0.023081	0.101447	0.342444	0.45461	0.045644	0.210505	0.294707	0.083179	0.221438	0.130488	0.08056	0.246706	0.274222	0.123185	0.13536	0.190624	0.112939	0.409844	0	1.245197	0	0.260108	1.670304
LOC107797315	50.66474	39.07588	50.9369	32.693	38.19213	46.1734	28.34978	33.64234	36.69783	28.43024	24.50252	28.37433	21.23975	27.51943	17.89594	37.64892	52.39819	45.76412	33.42807	32.76377	40.86297	29.48075	48.5346	58.18302	23.03834	22.17302	35.8952	30.66216	34.74714	17.67378
LOC107760261	3.84389	3.182513	3.294349	18.56446	20.69539	22.26393	22.06875	19.63655	18.4761	25.57804	21.67659	24.03552	27.27155	20.08152	13.86399	3.146884	4.707279	4.823395	24.99625	25.39505	20.86134	17.29402	14.01593	17.31543	46.06585	17.06244	52.91831	17.42276	39.22719	26.78011
LOC107792022	0	0	0	0	0	0	0	0	0	0	0	0	0	0	0	0	0	0	0	0	0	0	0	0	0	0	0	0	0	0
LOC107817082	0	0.053458	0	0.690811	0.311416	0	0.836354	0.250586	0.275344	0.457576	0.575814	0.545096	0.522373	0.767889	0.301014	0.087421	0	0	2.73792	4.019131	1.016409	1.028687	0.517384	0.367843	6.748464	3.264619	1.109226	0	5.083031	18.13393
LOC107794963	0	0	0	0.368427	3.6705	1.661457	0.613973	1.459392	0.83997	1.506927	0.057032	6.321697	5.794771	7.542265	1.085241	0	0	0.060639	2.558528	0.481627	2.670441	0.475475	0.358714	0.510068	2.056644	0.679031	0.384526	0	2.741031	10.05814
LOC107820275	1.987009	1.106669	1.10353	3.800273	4.49327	2.84837	5.416417	5.140423	2.850056	7.265942	7.353485	4.663001	6.451567	6.744046	5.38178	0.575836	0.639873	0.4938	2.74438	4.529018	6.392988	2.304726	2.044785	1.961442	11.44439	11.44311	10.242	3.947201	17.27225	13.65108
LOC107800776	15.34033	18.38314	16.1234	10.47872	10.11799	13.59179	9.432336	9.028324	10.1329	7.948564	10.87572	7.300922	6.658585	7.657591	5.868022	21.92117	20.34338	23.64744	9.994601	10.40703	6.091864	12.00746	12.16641	14.16015	3.196689	2.651709	4.504881	0	4.087848	8.1668
LOC107809840	1.987308	1.35839	1.912617	0.9645	0.69084	2.289213	0.29765	0.363859	0.399808	0.692099	0.199072	1.019352	0.237032	1.39375	0.364235	0	0	0	0	0	0.194192	0.118547	0	0	0	0	0	0	0	0
LOC107776662	14.3533	17.26338	15.67744	12.77778	10.11968	12.69709	12.59069	11.41283	13.14154	8.654453	11.11215	7.032217	8.395039	9.430173	10.22283	16.38212	14.53662	15.92552	9.276673	10.9232	7.834805	13.21966	12.67625	12.23217	3.687853	5.197063	5.339491	2.543901	4.195778	3.299205
LOC107760450	0	0	0	0	0	0	0.26443	0.153928	0	0.702691	0.536877	0	0.150412	0.343943	0.600942	0	0.043511	0.033578	0	0.038099	0.041076	0.188064	0.238362	0	0	0	0	0	0	0
Novel01810	0.386852	0.282054	0.201769	0.210281	0.479237	0.166365	0.194682	0.396644	0.072639	0.603567	0.434017	0.065365	0.258389	0.084407	0.476465	0.461252	0.224239	0	1.413182	1.439901	0.211689	0.452298	0	0.080868	2.249853	3.552624	0.82301	0	1.117466	4.783928
LOC107761816	0	0	0	0	0	0	0	0	0	0	0	0	0	0	0	0	0	0	0	0	0	0	0	0	0	0	0	0	0	0
LOC107808057	6.163747	13.87852	6.429617	0.503659	0.235274	0.051975	0.851496	1.218517	0.567333	0.212133	0.728812	0.081683	0.403622	0.15822	0.396944	17.13005	7.495913	6.469511	0.711301	0.265817	0.374763	3.411484	3.04889	0.101056	0.152801	0.168165	0.028569	3.457216	0.349111	0
LOC107832186	6.143792	5.994131	4.690202	14.78905	18.43508	11.85572	16.05377	20.66119	12.64537	35.36978	25.97245	29.94488	19.68507	23.30863	32.095	5.796322	4.223279	5.197057	22.43947	24.43695	17.42033	17.59615	10.97742	8.788384	37.96673	61.48443	33.81703	16.89877	30.43162	18.26346
LOC107776615	0	0	0	0	0	0	0	0	0	0	0	0	0	0	0	0	0	0	0	0	0	0	0	0	0	0	0	0	0	0
LOC107763775	0.056322	0.051331	0.09792	31.43151	40.7174	16.89439	43.83868	57.26675	39.65841	48.27589	32.85838	44.77529	25.26762	36.37537	40.34977	0.083943	0.380885	0.167963	65.26759	65.08281	69.13986	39.36934	43.37065	26.43177	101.3298	121.0008	74.4901	80.55685	68.33124	66.16739
LOC107805652	0	0	0	0	0	0	0	0	0	0	0	0	0	0	0	0	0	0	0	0	0	0	0	0	0	0	0	0	0	0
LOC107810387	0	0	0	0	0	0	0	0	0	0	0	0	0	0	0	0	0	0	0	0	0	0	0	0	0	0	0	0	0	0
LOC107779377	0	0	0.037018	0.01929	0	0	0.017859	0.018193	0	0.041526	0.029861	0.017989	0.047406	0	0.021854	0.047602	0.02057	0	0.021606	0.108072	0	0	0	0	0	0	0.025166	0	0	0
Novel04554	0.07331	0.033406	0.063727	0.190943	0.389215	0.098521	0.107604	0.234892	0.129049	0.330624	0.179917	0.069676	0.234627	0.219937	0.225729	0.040973	0.026559	0.061487	0.28826	0.410849	0.058502	0.091834	0.105079	0.028734	0.046343	0.255013	0.054154	0	0.044117	0
LOC107807692	3.081162	2.865021	3.800417	7.789451	9.045067	7.610088	7.15922	7.399822	6.821373	12.60681	10.49601	11.09815	9.59471	8.925871	10.9189	3.707868	2.775536	3.725088	9.443028	11.05981	7.917568	5.615746	5.325397	5.396396	9.317677	5.416951	10.80203	4.466487	7.918049	1.287249
LOC107779919	0.133964	0.061046	0.077634	0.101137	0.197566	0.096018	0.074907	0.038154	0.125771	0.087088	0	0.150902	0.09942	0.048716	0.022916	0.066553	0.06471	0.116521	0.090625	0.037775	0.040726	0.111876	0.059082	0.093346	0.084685	0.0932	0.052778	0	0.214983	0
LOC107780669	13.20909	7.877017	5.755368	4.816255	4.357851	5.189644	2.735564	2.145773	3.521351	6.519776	4.368111	7.494714	4.211673	4.732328	2.678015	5.225525	16.10104	7.513613	2.647639	2.952139	3.093529	2.015588	2.416565	1.874908	2.020531	0.544577	4.818545	0	0.942123	0
LOC107806476	8.232014	12.25306	9.25572	24.44657	26.91508	19.61445	26.23194	24.71284	15.75555	32.26398	36.34984	22.74353	31.45478	27.18489	37.09128	17.00608	13.1114	14.66795	29.96215	35.94784	19.47953	25.87992	18.86785	14.94921	51.71736	45.15361	35.01145	20.45694	38.71373	41.27009
LOC107826580	4.399082	4.537567	5.691577	6.889395	9.022375	5.75612	8.237468	8.245819	7.555753	8.413054	7.43661	8.153178	11.04699	8.705492	7.875169	6.454752	5.402952	6.864493	4.775318	5.07715	7.122142	6.066022	6.196417	6.807771	5.21686	3.274024	4.514173	7.316198	10.67146	6.325624
LOC107810266	0.332687	0.303203	0.353464	0	0	0	0.031004	0.094752	0.104114	0	0	0.031229	0.205751	0.161309	0.15176	0.055093	0.107135	0	0	0	0	0.092612	0	0	0	0	0	0	0	0
Novel03248	0.4128	0.543424	0.558193	0.249317	0.446445	0.493123	0.461644	0.391897	0.215308	0.357807	0.418103	0.232497	0.408476	0.350268	0.376611	0.786138	0.797599	0.786498	0.651593	0.620801	0.836624	1.264048	0.849607	1.006737	0.115979	0.19146	0.433686	0	0.220819	0
LOC107760032	0	0	0	0	0	0	0	0	0	0	0	0	0	0	0	0	0	0	0	0	0	0	0	0	0	0	0	0	0	0
LOC107787761	0	0	0	0	0	0	0	0	0	0	0	0	0	0	0	0	0	0	0	0	0	0	0	0	0	0	0	0	0	0
LOC107768199	0	0	0	0	0	0	0	0	0	0	0	0	0	0	0	0	0	0	0	0	0	0	0	0	0	0	0	0	0	0
LOC107825764	0.120691	0.329984	0.272776	0.153076	0.149513	0.337369	0.202457	0.123746	0.090648	0.117689	0.135405	0.040785	0.376193	0.052667	0.123874	0.251829	0.41975	0.413908	0.244937	0.163354	0.066043	0.201584	0.170331	0.100917	0.15259	0	0.057059	0	0.11621	0
LOC107814080	0	0	0	0	0	0	0	0	0	0	0	0	0	0	0	0	0	0	0	0	0	0	0	0	0	0	0	0	0	0
LOC107825558	0	0	0	0	0	0	0	0	0	0	0	0	0	0	0	0	0	0	0	0	0	0	0	0	0	0	0	0	0	0
LOC107812308	0	0	0	0	0	0	0	0	0	0	0	0	0	0	0	0	0	0	0	0	0	0	0	0	0	0	0	0	0	0
LOC107772806	9.517786	7.189762	6.357908	15.27768	19.64183	15.93293	8.46517	9.496727	6.476895	16.13282	14.28837	17.86265	22.50619	20.20937	15.5055	8.28271	8.42359	8.929421	22.88101	19.69619	19.77845	8.455339	6.197885	5.441349	21.84582	23.55353	20.49781	79.94285	36.13481	25.67278
LOC107818803	0	0	0	0	0	0	0	0	0	0	0	0	0	0	0	0	0	0	0	0	0	0	0	0	0	0	0	0	0	0
LOC107766799	0	0	0	0	0	0	0	0	0	0	0	0	0	0	0	0	0	0	0	0.024603	0	0	0	0	0	0	0	0	0	0
LOC107828902	7.942464	8.284376	7.784356	13.86096	12.26418	15.36074	10.54924	9.593037	9.885983	6.691774	6.436992	5.759587	7.814726	7.65844	7.158916	7.528361	7.629614	8.890553	10.02296	11.03935	9.172567	10.61649	9.923199	11.21755	7.623718	6.261393	7.844955	9.654365	7.149286	16.69443
LOC107796737	0	0	0	0.041444	0.060719	0	0.03837	0	0.064423	0	0.032077	0	0	0	0.023476	0.03409	0.022097	0.068212	0.04642	0.019349	0	0.019102	0	0.023907	0	0	0	0	0	0
LOC107764207	197.4882	116.5069	164.3945	7.138727	3.268402	9.682035	24.78432	26.29977	50.29933	7.306498	8.90468	10.49088	10.61248	10.93752	5.199198	93.27136	134.3416	120.2225	4.462002	4.999345	6.737379	24.88309	41.57907	35.18682	0.177902	0.146842	0.166309	0	1.18551	4.350194
Novel02213	0.993582	0.935914	0.82892	0.664529	0.678567	0.702589	0.464221	0.518482	0.613531	0.682759	0.556425	0.664764	0.675519	0.967552	0.34221	0.750357	0.579795	0.835216	0.676656	0.71076	0.462204	0.167067	0.39997	0.515761	0.261355	0.296912	0.559582	0.47688	0.417349	0
LOC107825572	0.975863	0.646821	0.583893	0.838156	0.908365	1.076423	0.861018	0.693032	0.820995	0.543765	0.746483	0.449692	0.634877	0.580701	0.70242	0.963323	0.636673	0.982661	0.655872	0.846955	0.277404	0.899641	0.648376	0.675566	0.320464	0.158709	0.284603	0	0.305075	0
LOC107813483	1.189027	1.825097	1.251195	2.154396	2.54726	2.153009	0.997288	1.015937	1.527583	3.478354	2.85229	2.80209	5.433896	6.758938	7.258077	1.259144	1.632363	1.213064	1.587556	0.847019	1.826379	0.992987	0.8832	0.850317	0.553844	1.131988	1.553264	0	1.807705	11.60831
LOC107774706	2.968437	3.356022	3.690951	2.00843	1.861942	3.151035	1.76489	1.733682	2.963285	1.868666	1.923384	2.126884	2.467922	2.254595	1.735475	3.22012	3.049264	3.809886	1.753919	1.303226	1.302228	2.071079	3.181802	4.43791	0.66509	0.888812	1.687611	2.687155	1.628101	0
LOC107780000	2.404894	2.338773	2.434695	2.045864	1.790641	2.427899	1.709605	1.390753	1.060074	0.886553	1.100216	0.780478	1.403877	1.775743	1.279311	2.010682	1.515831	2.142794	2.05344	2.778658	1.203642	1.567524	2.030733	1.226144	0.315176	0.26525	0.363965	0	0.564781	0
LOC107769999	15.12938	12.67434	12.53724	10.08947	10.5308	11.68601	13.41067	11.03361	14.22207	11.8099	15.08262	10.26411	10.5902	8.169347	10.82281	10.36337	12.58622	11.69261	6.377388	7.126392	4.390353	14.05478	15.4682	14.77499	5.941316	6.671603	6.615331	1.366117	6.437723	2.362305
LOC107797803	0	0	0	0	0	0	0	0	0	0	0	0	0	0	0	0	0	0	0	0	0	0	0	0	0	0	0	0	0	0
LOC107832841	0.798111	0.43104	0.462521	0.508812	0.209251	0.317803	0.297516	0.176796	0.166512	0.115298	0.020727	0	0.032906	0.064496	0.030339	0.088112	0.257014	0.11019	0.089985	0.025005	0.107836	0.222175	0.026074	0.154479	0	0	0	0	0	0
LOC107784731	9.523593	9.876764	11.60915	9.322091	8.572397	11.47477	10.65046	9.16606	8.992555	8.219268	9.940166	8.461958	10.47994	9.255257	10.89829	9.748168	11.95019	9.181348	7.66446	7.593298	4.891933	8.04482	9.076336	6.807659	2.283584	2.513194	4.140182	0	2.108044	3.384242
LOC107821287	12.09654	8.466188	10.28886	29.96644	20.84427	28.344	8.155548	8.036063	11.19375	7.421668	10.45044	5.720961	4.34908	7.482358	5.940459	5.456058	5.59152	5.372256	9.338195	10.60005	8.788849	6.017781	11.691	11.34268	3.731957	4.066931	6.532903	0	3.204431	1.789348
LOC107819587	1.420722	1.209628	1.381253	0.999197	0.744364	1.507351	1.567922	1.118069	1.368937	0.619777	1.310804	0.789648	0.874024	0.509848	1.016895	1.379127	1.444775	1.240389	0.663918	1.138578	0.528518	1.967062	1.43455	1.504476	0.25998	0.130055	0.419795	0	0.269994	0
LOC107772009	0.498855	0	0.108411	0.112984	0.110355	0	0	0	0.234173	0	0.087449	0.421446	0.138833	0	0.384008	0	0.120484	0.092979	0	0.105499	0	0	0	0	0	0	0.147402	0	0	0
LOC107762148	388.7917	294.8251	370.0124	181.3933	179.1234	273.1896	163.3124	174.9807	234.0502	103.8435	122.5027	133.4211	98.46917	135.2545	93.85969	227.0397	409.0874	293.3842	102.1465	94.14973	142.8827	160.3059	232.888	264.5983	20.91282	14.02853	66.09797	78.86092	28.06156	19.48102
LOC107794796	0	0	0	0	0	0	0	0	0	0	0	0	0	0	0	0	0	0	0	0	0	0	0	0	0	0	0	0	0	0
LOC107817864	0.708733	0.464257	0.519823	1.805838	2.684914	1.285831	1.969097	1.665291	1.538713	1.684565	1.568542	1.758848	3.303816	2.5612	2.364127	0.742709	0.813076	0.891658	3.056484	1.985973	2.989489	2.700457	2.129414	2.199152	2.492148	3.513158	1.806215	1.583887	3.412091	5.47775
LOC107829295	0	0	0	0	0	0	0	0	0	0	0	0	0	0	0	0	0	0	0	0	0	0	0	0	0	0	0	0	0	0
LOC107797544	0	0.180142	0.085911	0.179069	0	0.318762	0.082893	0	0	0.096371	0	0.083494	0.110019	0	0	0.073648	0	0.073682	0.100286	0.083603	0.180269	0.247606	0	0.30989	0	0	0	0	0	0
LOC107812609	0	0	0.074562	0	0	0.030739	0.023981	0	0	0.02788	0	0	0	0	0	0	0	0	0	0	0	0	0	0	0.036148	0	0	0	0	0
Novel02492	2.272989	2.854134	2.502757	0.869445	0.983297	1.140412	0.550753	0.215789	0.806171	0.246272	0.389601	0.128019	0.224918	0.165315	0.103686	1.99496	1.610325	2.221821	0.153765	0.299101	0.967402	0.590561	1.113848	1.267054	0.063861	0	0	0	0	0
LOC107829837	0.867046	2.138202	1.950764	2.44891	2.978615	2.412702	3.037249	3.529825	2.729364	1.889894	4.434627	1.034124	2.100745	2.782071	4.397212	2.622501	1.330368	2.471603	2.535946	3.753595	0.69773	3.961212	2.609289	1.439316	2.708282	4.257991	0.723373	0	2.946541	6.307146
LOC107796710	20.49427	16.0939	25.90127	19.60326	22.40425	15.56274	19.44241	18.78592	29.93551	89.25115	18.24228	28.57671	50.61276	45.69329	22.66852	30.39643	23.01895	28.25937	26.24759	18.72536	32.93629	15.86904	15.70794	19.34093	281.6247	24.01735	250.2803	120.95	230.8609	129.1797
LOC107827764	6.454598	6.51738	6.539259	4.58548	3.98569	5.391828	6.30955	4.205671	4.316016	3.622474	3.972433	5.09995	3.876987	3.343517	3.669866	7.336076	8.254432	6.612416	4.52352	5.420864	4.616191	4.692366	5.693383	6.163926	2.759331	2.067592	3.84185	3.320828	3.353389	0
Novel03875	1.412042	1.127369	1.024573	1.099511	1.06876	0.953529	0.660686	0.722895	0.920312	1.035534	0.781462	0.670408	1.695319	1.559565	0.586899	1.056604	0.63698	1.144091	1.047988	0.710771	0.473615	0.350846	0.669078	0.524481	0.206558	0.211089	0.386196	0.834554	0.505642	0.36078
LOC107802417	7.364566	9.131297	6.426674	42.77272	62.5144	46.86238	50.27773	45.51076	21.9485	51.3274	48.19769	50.35309	73.65793	53.59367	70.87097	7.359988	9.348503	7.916674	57.90168	61.47816	41.65338	33.49314	24.0454	17.78169	45.14777	48.8532	29.04815	44.9099	50.70981	26.47455
LOC107831781	23.43095	19.31372	24.19137	27.89246	26.11115	28.9741	22.46963	23.50475	23.0491	17.77943	16.48587	27.49697	22.87884	24.5151	16.99017	15.61338	19.7005	17.64763	26.53511	22.6622	34.49739	17.36391	19.39796	22.48401	22.64758	17.3583	29.11125	45.75137	36.95997	19.77844
LOC107812742	8.473776	9.303283	8.822158	7.962461	6.364713	7.860322	8.644486	6.970821	7.678035	4.804071	6.75709	5.127757	6.449626	7.309584	8.070241	8.928647	8.148304	8.403824	5.879045	6.134673	4.349374	8.508464	7.283225	7.765138	2.840305	3.619453	2.748391	5.637161	2.276974	1.21848
LOC107792390	5.209037	3.985467	5.031228	4.631733	4.210964	4.80524	4.692677	3.846321	6.007433	6.333659	4.080969	5.921982	5.11867	5.998515	3.069237	4.62459	4.286832	5.657529	3.882758	4.107281	3.343106	4.215937	5.672488	6.183858	5.366213	1.923851	8.094871	4.59901	7.275762	0
LOC107772893	7.51349	9.195375	8.210799	14.78057	9.117848	9.23191	11.3434	11.92236	10.68177	12.35056	16.85909	10.33753	12.90465	11.94398	16.96561	6.238955	4.769979	4.641347	7.406337	8.171866	6.460862	9.143152	7.006104	8.526221	14.1133	10.75317	9.134065	0	8.268027	0
LOC107817597	1.112544	1.836065	1.346113	0.953427	0.58535	1.115302	0.970968	0.590906	0.649287	0.586417	0.621985	0.330238	0.585777	0.672472	0.663527	1.523694	1.031237	1.345052	0.671257	0.52144	0.315367	0.841225	0.861985	0.738554	0.057024	0.146435	0.195464	0	0.434285	0
LOC107765270	0.239724	0.300409	0.338629	0.054295	0.106062	0.128866	0.025133	0.179224	0.028133	0.116881	0.084047	3.696095	0.033358	0.032691	0.092267	0.156312	0.202645	0.178725	0.060814	0.101395	0.027329	0	0.052863	0.1566	0	0	0.070834	0	0	0
LOC107793619	2.971688	4.103527	3.313835	3.290466	2.443618	4.453537	2.51766	2.000496	3.297216	1.814766	1.578598	3.093828	1.470278	1.997575	0.801026	2.952674	2.377918	2.461689	2.893627	2.843945	3.914777	2.180918	2.250547	3.168756	0.417458	0.751798	1.09981	0	0.289026	0
Novel03498	9.592438	5.751529	7.625342	4.745341	5.528382	6.106408	5.769536	6.740164	7.880078	3.384613	3.628621	3.785402	3.723402	3.098155	1.554539	8.041833	10.54743	9.59816	3.45804	2.615872	4.777122	4.795997	7.960175	9.168526	0.478727	0.351242	1.566364	0	1.82295	0
107785457	0	0	0	0	0	0	0	0	0	0	0	0	0	0	0	0	0	0	0	0	0	0	0	0	0	0	0	0	0	0
LOC107795643	0.461089	0.392211	0.574502	0.751903	0.598403	0.446155	0.580103	0.472761	0.663766	0.569519	0.883731	0.324618	0.889705	0.922214	0.662549	0.641395	0.445451	0.641689	0.358709	0.559072	0.210261	0.705958	0.569396	0.546188	0.388638	0.406329	0.199823	0	0.665955	0
LOC107794136	3.098521	12.41628	6.156523	0.712915	0.348162	0.687409	0.618777	0.840464	0.646451	0.095919	0.58628	0.041551	0.219004	0.214624	0.20192	12.9012	8.505148	7.076909	0.499074	1.206555	0.897112	1.026848	2.125745	1.696396	0.062182	0.068434	0.406911	0	0	0
LOC107811427	0	0	0	0	0	0	0	0	0	0	0	0	0.049901	0	0	0	0	0	0.022743	0.01896	0	0	0	0	0.062373	0	0	0	0	0
LOC107823076	0.59584	0.049367	0	0.34351	0.910685	0.232946	2.271622	4.581918	4.576917	5.017894	3.304445	1.144051	2.050192	17.2554	43.14235	1.53389	0	0	0.714548	0.77897	0.592818	2.578481	1.051141	2.604319	7.327778	10.92862	17.41381	7.747453	0.521561	0
LOC107808133	2.334214	1.40405	2.272573	2.410735	2.272013	2.660139	2.388523	2.473076	3.593999	2.094061	2.487863	2.760812	2.702419	2.240931	1.629126	2.087351	2.300148	2.505968	2.605459	2.172043	1.830804	2.534172	3.994326	3.952346	1.711671	2.403437	1.986374	3.338586	1.123774	0
LOC107812884	29.47884	26.64429	24.98999	28.14098	30.61194	40.46797	29.01632	25.91609	25.61749	38.96097	36.72872	42.91403	53.56373	36.01385	40.63343	20.06138	26.24308	21.43283	25.09239	24.42184	31.55123	21.26148	22.24163	23.29944	22.02331	21.86478	20.7322	43.5569	18.18005	15.06381
LOC107770260	0	0	0	0	0	0	0	0	0	0	0	0	0	0	0	0	0	0	0	0	0	0	0	0	0	0	0	0	0	0
LOC107803015	0.101708	0	0	2.119279	2.879942	1.421535	3.071056	2.954679	2.291717	3.074531	2.496127	0.85926	0.792561	3.106844	1.461468	0.075793	0	0	6.089185	5.506445	7.420773	3.397569	4.665098	3.826997	4.886427	8.208139	4.447807	7.27358	4.896598	6.288776
LOC107826849	0	0	0	0	0	0	0	0	0	0	0.024767	0	0	0	0	0	0	0	0	0	0	0	0.036917	0	0	0	0	0	0	0
LOC107790789	13.84201	12.92361	13.87054	14.09811	12.59756	17.79155	14.87296	12.14013	16.99207	14.40489	14.07473	12.74206	17.38626	16.42344	11.08209	11.51256	12.28314	13.22029	8.553422	9.467675	7.147686	11.96007	12.65717	15.4695	5.702815	4.275676	7.696933	6.048267	9.907837	3.486243
LOC107803673	116.6151	113.643	89.94801	179.3107	163.3474	99.79847	187.9204	240.38	184.9103	221.9044	293.1493	206.9224	106.5462	93.88514	242.827	140.1647	83.4941	109.0387	285.6919	335.5066	249.3577	260.2937	294.6821	187.8186	83.86597	284.0123	114.8891	176.3215	68.69459	48.65377
Novel05299	2.308531	2.24904	2.145154	1.370235	1.549665	2.567537	1.268587	1.428341	1.270516	0.69862	1.004737	0.605269	0.708935	0.607912	0.081704	1.542356	2.999281	2.84873	0.969326	0.942759	1.597214	1.063677	1.263896	2.412875	1.006443	0	0.376344	0	0	0
LOC107823033	1.066255	1.631168	1.439963	1.793949	2.139706	1.00306	2.379513	1.887148	1.80546	1.262533	1.66888	0.932973	1.780454	1.454039	1.641562	1.986436	0.974908	1.703439	3.149281	2.093878	2.622121	3.593631	4.618562	3.661761	3.899774	5.033696	0.675122	1.361648	6.691662	14.12746
LOC107797022	0	0	0	0	0	0	0	0	0	0	0	0	0	0	0	0	0	0	0	0	0	0	0	0	0	0	0	0	0	0
LOC107810118	0	0	0	0	0	0	0	0	0	0	0	0	0	0	0	0	0	0	0	0	0	0	0	0	0	0	0	0	0	0
LOC107814062	6.255882	4.412999	5.130868	7.396584	5.910912	7.371823	4.713477	5.073406	5.541473	5.686697	4.584894	5.583739	5.90181	5.051168	3.954093	4.135124	5.224223	5.53359	6.59909	4.424988	6.253453	3.719101	3.834613	6.649471	4.423867	3.639213	6.015417	0	6.806338	0
Novel01835	2.467418	1.670498	2.635131	0.830275	1.497143	1.819047	0.413906	0.421646	1.25754	0.206232	0.296598	0.833817	0.627832	1.53819	0.361784	1.681118	2.0432	1.787006	0.429217	0.894543	1.543081	0.765368	0.994938	1.768419	0	0	0	0	0	0
Novel02413	17.13808	19.02908	16.20919	20.66519	16.87362	20.11237	18.8285	19.85087	22.37867	19.06556	20.77627	21.51416	29.89389	23.23933	21.3682	17.71795	19.00537	19.52567	16.47202	17.35632	16.29065	17.68899	18.47032	22.39089	16.78487	17.38099	16.61834	12.94664	24.40401	26.11871
LOC107782644	14.00822	13.11723	12.84559	16.5975	15.74944	13.37731	14.4908	13.30672	16.29759	18.37375	17.12209	15.77938	19.78316	18.24884	11.36117	14.40984	13.32084	15.46081	12.65376	12.26823	12.34988	13.25845	14.48822	16.96679	10.9042	9.248869	14.34905	5.892858	13.62035	6.793329
LOC107820681	8.458093	9.158443	9.348103	11.3662	11.35115	9.288345	11.89117	12.57812	11.11716	12.03773	12.34175	8.098383	8.631028	8.656146	12.64968	11.10525	8.073952	9.924234	14.30448	15.24689	13.02184	11.87353	13.1271	13.1764	8.325732	10.73204	8.568673	12.96157	11.73132	3.735547
LOC107783884	117.1948	105.5924	117.404	84.08074	90.12168	93.35775	71.71256	70.92854	76.25621	72.51912	79.32723	89.29809	78.90573	73.19164	83.02316	88.46379	107.9165	109.6015	72.57146	77.99734	76.7327	70.1875	67.89909	83.40485	43.44628	47.98352	48.32245	88.92973	58.55362	67.51239
LOC107779135	0	0	0	0	0	0	0	0.043033	0	0	0	0	0	0.109892	0	23.5702	50.65191	16.22132	3.526389	4.21794	6.430742	114.9955	81.43175	36.69123	0.191031	0.140159	1.309606	0	3.394661	6.228307
LOC107807496	0	0	0	0	0	0	0	0	0	0	0	0	0	0	0.015701	0	0	0.011405	0	0	0	0	0	0	0	0	0	0	0	0
LOC107773066	0	0	0	0	0	0	0	0	0	0	0	0	0	0	0	0	0	0	0	0	0	0	0	0	0	0	0	0	0	0
LOC107796649	53.65905	40.41835	53.13168	3.734461	3.333107	9.589537	2.742097	2.611196	3.76997	1.767246	2.392101	1.441038	2.096624	3.295263	1.860121	25.71336	36.52772	39.66068	3.46169	2.615293	3.208515	3.205102	5.359981	3.751342	0.539137	1.730591	0.882009	0	0.342164	0
LOC107791483	0	0.049935	0	0.099275	0	0	0.09191	0.093629	0.154319	0.053428	0.076838	0	0.060994	0.119548	0.168707	0	0	0	0	0.249849	0.091514	0.096658	0.057267	0	0	0	0.259032	0	0	0
LOC107804504	10.24997	20.4485	23.64328	6.873067	17.06845	11.15013	12.35409	25.37708	53.83644	21.56405	48.16046	63.54815	39.30751	27.73541	47.67264	39.78526	14.11277	42.42094	13.7997	20.20903	17.15053	20.99563	24.5962	66.68374	7.346878	4.828894	8.823654	20.20128	47.98704	4.990323
LOC107801229	23.51224	20.82423	23.58652	15.20173	11.95959	18.86294	11.9351	16.03673	15.89735	11.5383	9.906383	10.4705	9.424966	11.07264	8.637381	17.14128	20.25116	18.25184	11.38588	13.80632	13.53596	12.86329	15.25085	14.23323	7.99588	10.00628	7.83654	7.294827	13.50498	15.76787
LOC107767898	16.65771	17.67585	17.12601	13.97952	14.02999	19.30426	13.93196	11.47091	14.04408	16.77254	18.82761	14.35196	15.18158	12.89595	15.57136	18.79584	14.10965	20.10616	16.44819	15.42855	13.72885	14.83739	13.34043	21.43037	4.892219	6.139211	7.362111	1.687339	11.8136	13.12994
LOC107829573	10.42522	10.70342	9.966461	8.916186	8.820911	10.17209	8.48878	8.755881	7.811074	7.544043	8.306205	7.928908	7.962899	7.167182	6.690855	8.884129	9.164948	10.6091	8.442444	9.999181	5.89911	7.859039	8.278399	7.980117	6.157383	6.07061	5.636237	5.441976	4.518387	4.705162
LOC107820666	8.046406	9.789609	9.28648	9.112046	6.826204	7.244054	7.404097	6.724884	6.509184	6.417938	7.038987	7.11132	6.718029	5.944484	5.27188	8.193825	7.264076	8.241259	6.797508	7.038002	5.753209	6.08367	7.063009	7.124091	5.086552	5.108849	3.231623	4.190038	7.051869	10.86819
LOC107766366	3.605966	3.683027	3.566947	0.281623	0.44011	0.434475	0.234658	0.39841	0.321033	0.394065	0.588531	0.525245	0.761316	0.94957	0.319057	1.876386	2.222339	2.456642	0.346982	0.289262	0.737123	0.363449	0.164519	0.292419	0.550229	0	0.110223	0	1.646245	0
LOC107794900	3.441186	4.099483	4.25199	5.834253	6.176975	4.571766	7.421839	5.94349	5.376869	6.399591	8.324717	6.333563	7.497376	6.355264	9.485778	6.172818	5.105443	5.772487	5.861366	6.175492	4.730046	6.630323	5.788852	4.084853	6.463904	6.532417	4.415822	7.031241	7.455192	6.079248
LOC107796829	1.210626	0.882669	1.578558	1.316119	0.964118	1.128028	1.320026	1.206791	1.74277	1.73142	1.499709	0.784124	1.033226	1.18866	1.408227	1.834392	1.910292	1.414031	1.269409	2.08235	0.846485	1.98835	1.708564	1.096633	1.37754	1.684498	1.430863	2.885896	1.165677	0
LOC107790706	0.263321	0.489568	0.366239	68.24589	65.36215	38.44525	18.56978	25.97266	25.8688	39.33731	19.83779	38.83274	22.41876	29.66924	19.8699	1.091022	0.457902	0.973731	80.91876	91.22999	80.67218	20.69756	17.41997	22.19396	17.13859	36.25811	33.76157	11.29869	13.94492	6.512607
Novel05225	0.52643	0.119944	0.62922	0.357689	0.291137	0.778218	0.331154	0.618469	0.185338	0.449169	0.322991	0.611521	0.366268	0.861463	0.74293	0.539407	1.207867	1.177427	0.734504	0.333993	1.080254	0.604498	0.754562	1.581895	0.083196	0.091561	0.233324	0	0	0
LOC107787260	0	0	0	0	0	0	0	0	0	0	0	0	0	0	0	0	0	0	0	0	0	0	0	0	0	0	0	0	0	0
LOC107797101	1.801094	1.461587	1.329733	3.039879	3.864241	2.095552	16.41027	20.34304	10.37731	4.306529	3.511979	5.27354	7.333367	27.75092	28.71651	2.371793	1.525487	1.784258	17.1246	9.955589	17.68638	24.705	11.16431	9.283576	29.82105	71.57808	16.24264	33.52425	32.78391	181.5392
LOC107768816	0.153325	0.401744	0.383187	0.069452	0.050877	0.164843	0.996654	0.998916	1.169582	0.018689	0.430047	0.016192	2.858955	1.129075	1.199938	2.984996	0.481407	1.557481	1.089088	0.210767	1.310956	2.801006	1.487674	0.761214	1.163099	0.880032	0.29448	2.741232	1.199514	1.185042
LOC107821149	4.104327	6.25749	4.695178	8.708316	8.262634	6.758414	9.943509	8.578091	6.374457	9.045949	11.28715	7.424725	10.66665	9.188275	10.30435	6.173949	5.011683	6.449784	8.190233	8.98326	5.148661	9.403687	8.142284	6.873315	10.47469	11.8888	7.10516	6.546857	13.07519	10.37749
LOC107763620	0	0	0	0	0	0	0	0	0	0	0	0	0	0	0	0	0	0	0	0	0	0	0	0	0	0	0	0	0	0
LOC107777424	0	0	0	0	0	0	0	0	0	0	0	0	0	0	0	0	0	0	0	0	0	0	0	0	0	0	0	0	0	0
LOC107830522	4.663381	3.187573	4.850056	6.33718	4.200153	6.356661	4.959069	5.265258	6.09819	6.658671	6.656716	6.824216	7.045391	7.722128	6.239371	5.212735	5.551072	5.028868	4.394071	6.199128	4.101186	4.31178	4.774494	5.570474	6.422354	4.403083	8.858155	0	10.02285	0
LOC107786767	0	0	0	0	0	0	0	0	0	0	0	0	0	0	0	0	0	0	0	0	0	0	0	0	0	0	0	0	0	0

Novel04064	0.848255	1.449525	0.921713	1.440893	1.970304	0.113997	1.600803	0.724772	0.796379	1.137339	1.635692	1.074944	1.180362	1.388107	1.85008	0.553106	0.409743	0.237154	1.291126	1.435131	1.063732	0.7084	1.30938	0.110825	0.268113	0	0.626606	0	0	0
LOC107828394	1.137739	0.319049	0.874896	0.118931	0.116163	0.141139	0.183514	0.037389	0.164332	0.085341	0.30684	0.295752	0.097427	0.907044	0.359306	0.032609	0.422751	0.032624	0.177615	0.370173	0.039909	0.219267	0	0	0	0.121775	0.10344	0	0	0
LOC107813555	210.5687	159.6678	214.3409	110.2174	109.0457	158.5613	101.8651	99.91328	152.1611	64.07641	68.74674	86.99618	59.71212	67.9083	49.3439	114.2061	186.5932	155.4227	65.93325	57.36282	91.23442	80.73738	148.0514	205.6041	18.04901	11.83065	44.5395	30.0272	19.20369	0
LOC107775431	2.160279	2.045536	2.316871	0.813342	1.042668	1.90028	1.082445	1.294458	1.39601	0.711301	1.14101	1.042894	1.030651	1.346719	0.979046	1.672564	2.791717	2.175329	0.626315	0.68826	0.690853	1.804106	1.781775	1.818068	0.070942	0.195186	0.530551	0	0.13507	0
LOC107763495	0.553795	0.757074	0.58838	0.668947	0.939232	0.396931	0.632225	0.407458	0.346618	0.300011	0.970803	0.181946	0.650742	0.419558	1.184166	1.215139	0.505289	0.906037	0.686831	0.702708	0.210446	1.682944	0.461346	0.273335	0.466777	0.684947	0.21818	0	0.296241	0.951166
LOC107809688	3.982087	6.500622	4.463236	0.766443	0.425931	0.674333	3.829318	3.227921	3.149702	0.312919	1.431919	0.24646	0.941791	1.082087	1.781559	5.804474	5.580309	4.382536	0.222019	0.666311	0.332576	4.409697	3.718323	1.128182	0.129092	0.10148	0.0862	0	0.070224	0
LOC107796813	0.708366	0.997076	0.738921	0.777223	0.807887	0.803891	0.64695	0.632147	0.376859	0.544922	1.026528	0.325821	0.543233	0.558128	0.694735	1.108539	0.570285	1.085574	0.694841	0.66581	0.279952	0.762475	0.742849	0.756837	0.507501	0.339498	0.213959	0	0.568388	0
LOC107820490	19.51672	21.0779	23.24309	20.04141	16.62257	24.02955	8.24226	7.732486	8.668096	11.7664	12.8198	9.92385	8.446283	14.11141	13.51066	41.31552	21.06261	22.21769	18.923	30.93171	15.92374	5.343908	8.345462	9.172325	3.640583	0.763169	9.291702	9.806004	1.320287	11.30443
LOC107764665	3.48724	5.18888	4.701737	17.92398	23.86725	17.4453	13.66942	13.80342	9.755107	19.91721	12.82511	17.07545	23.29226	21.0407	16.216	3.92456	6.256652	5.358947	25.7813	23.41911	26.67659	11.70857	9.981228	10.04197	39.86033	30.40077	32.21606	40.71623	55.50587	52.80521
Novel00541	0.082358	0	0.143184	0.447674	0.583007	0.88545	0.345386	0.422214	0.309286	0.722786	0.404247	0.556627	2.108691	0.179697	0.507181	0	0.15913	0.184204	0.668571	0.418016	0.450672	0.412676	0	0.602565	0	0.229191	0.486703	5.88977	0.793003	0
LOC107793197	2.320058	2.602396	1.706503	1.980594	2.526695	3.069964	2.918898	3.316574	2.261948	1.087668	0.969838	1.356957	1.142357	2.287697	1.831726	2.626613	2.88792	2.029073	2.716424	2.226806	2.238005	3.502477	5.391593	4.476782	0.733315	0.807048	0.949196	3.19071	2.792396	0
Novel04717	0.955254	0.411115	0.530522	0.865412	0.704393	0.656148	0.511886	0.453442	0.348769	0.413997	0.483762	0.42593	0.797552	0.897394	0.136173	0.632761	1.05103	0.633051	0.134629	0.179573	0.242002	0.332399	0.491515	0.443747	0.16774	0	0.188172	0	0.638741	0
LOC107814535	0	0	0	0	0	0	0	0	0	0	0	0	0	0	0	0	0	0	0	0	0	0	0	0	0	0	0	0	0	0
LOC107801438	1.665961	2.456102	1.192622	1.598057	2.037798	2.05451	2.465846	2.846884	3.128154	1.290062	1.924049	1.573032	0.763647	0.855285	0.854948	1.898728	1.278099	1.899597	1.392178	2.072482	1.474696	3.519138	2.593213	2.919174	0.929241	0.81814	1.331992	0	0.707694	0
LOC107798926	0	0	0.032995	0	0	0	0	0	0	0	0	0	0	0	0	0	0	0	0	0	0	0	0	0	0	0	0	0	0	0
LOC107797718	6.459613	5.856637	6.167996	8.702319	9.417892	8.168323	9.179645	8.950938	9.206833	7.637055	9.270188	9.896586	14.19557	12.77975	9.652961	7.307862	6.143526	7.261303	12.29444	10.1077	10.01212	7.938155	6.878698	8.080913	13.41405	11.40974	8.505024	9.574169	15.1466	18.62522
LOC107775264	1.135101	1.335452	1.363467	0	0.109572	0.288452	0.05193	0.035268	0.019376	0.04025	0.043415	0	0	0.067546	0	0.845879	1.176356	1.307866	0.104711	0.069834	0.188224	0.017236	0.109226	0.086284	0	0	0	0	0	0
LOC107790719	0.030471	0.027771	0	0	0	0	0.025557	0.026035	0.028608	0	0	0	0	0	0	0.068121	0	0	0	0	0	0	0.026877	0	0	0	0	0	0	0
LOC107825713	4.789789	3.897591	4.69899	5.176172	5.691459	5.92205	4.90697	5.349516	4.464744	4.50384	5.925535	4.682433	5.370146	5.374722	4.284037	5.456016	4.263762	4.693302	6.075433	5.961981	4.368368	4.714377	5.40185	5.649951	4.195769	3.808367	5.337651	2.446697	3.29425	6.346283
LOC107805864	12.47756	12.00352	17.06061	15.7393	17.11733	12.52989	19.96797	19.45293	20.19528	18.06068	19.38207	18.50234	17.86916	18.3862	20.21046	23.7623	10.29644	16.89317	16.70597	16.25421	16.75351	18.90495	16.8912	19.3359	10.65389	13.98575	11.29103	4.647511	11.05481	8.036525
Novel03813	3.53122	1.762387	2.265668	3.351445	1.636724	2.169425	1.692449	3.01717	1.578698	1.639707	1.827593	3.338424	3.275836	3.026876	1.984762	2.130237	3.16778	2.131213	3.412608	2.489308	2.453739	2.036224	2.076513	1.845415	1.488178	1.520825	3.378643	6.01267	2.023875	0
LOC107770285	70.23508	59.17426	64.12013	64.02534	61.56857	66.58419	61.08228	61.98445	65.61741	55.82539	54.56776	61.47266	68.49152	57.89295	49.21164	62.29535	74.54782	65.32604	50.33225	48.90436	56.11268	58.15984	64.67787	79.44559	25.10037	24.19286	39.73518	49.11183	36.2182	27.0215
LOC107791219	0.218933	0.349179	0.523363	0.099171	0.145295	0.294225	0.183629	0.280594	0.25693	0.266859	0.115137	0.184961	0.12186	0.417979	0.224707	0.326299	0.687401	0.326448	0.111079	0.185202	0.049918	0.274255	0.048278	0.400451	0	0	0	0	0.263506	0
LOC107787169	27.66588	27.34856	25.80959	19.27311	16.25563	26.6229	17.78198	15.13363	17.93439	15.48721	19.14273	14.79872	17.81615	15.41045	15.02408	24.05276	28.99022	29.66592	12.20476	13.68296	10.4576	20.21134	20.80938	21.77653	3.331246	2.987271	8.275634	1.744711	6.342543	1.508486
LOC107787395	8.037314	8.468438	9.201951	6.926209	6.244634	9.905646	7.661996	6.16382	6.183862	5.772911	5.883193	6.790146	6.284897	6.159206	5.191009	6.398449	9.582802	7.19059	5.251482	6.666351	5.899889	4.616664	6.121128	5.777972	4.808175	3.109513	4.216833	8.411438	3.208811	4.848383
LOC107796007	38.02464	27.21448	31.76805	33.85275	30.15598	44.29772	23.20737	26.87154	40.38023	28.42354	23.8583	31.75396	31.35071	38.61387	16.58948	23.80388	31.8123	26.67419	21.2939	23.54531	23.78567	19.95006	33.78189	38.36893	12.53821	9.682105	24.93184	7.836597	20.57493	20.32669
LOC107765881	165.5238	126.3847	136.6797	83.6409	81.27758	115.0601	83.67845	83.23084	105.6939	109.503	93.67145	128.5374	102.1472	124.0513	75.61371	85.78937	124.5969	108.234	56.01924	55.62617	73.11747	54.83699	87.00347	74.34212	50.02528	29.88712	85.51413	87.58384	62.1366	58.25035
LOC107806911	4.083197	3.023581	4.547716	3.410172	2.709811	3.361045	3.692312	3.870381	5.570533	1.24426	2.505248	3.233998	3.338081	3.20172	1.30965	3.280522	3.205078	3.139328	1.618495	1.996904	2.734772	3.303422	3.826753	5.934849	0.241988	0.532638	0.904877	9.125197	2.15009	3.944847
LOC107765574	0	0.219989	0.349713	0.291572	0.21359	0.086505	0.337428	0.068748	0.226619	0.078459	0.056419	0	0	0.263335	0.412912	0.299796	0	0.059987	0	0.136128	0.513669	0.134389	0.425829	0.420486	0	0	0.095098	0	0.387365	0
LOC107806609	0	0	0	0	0	0	0	0	0	0	0	0	0	0	0	0	0	0	0	0	0	0	0	0	0	0	0	0	0	0
LOC107817394	12.15301	13.78687	11.69713	13.73044	12.70902	12.88323	13.16332	11.25524	11.59596	12.56895	15.53373	8.950894	11.88903	10.26053	12.99688	14.84074	9.770342	13.89712	10.77975	12.53324	6.278208	15.49601	14.04313	13.67943	6.661792	4.966582	6.395108	2.025902	7.228375	8.75803
LOC107800352	7.040773	7.867728	7.504305	7.23177	7.122993	5.062533	7.880135	7.222825	6.883858	6.29715	6.5879	4.811634	6.539892	5.601848	6.443974	7.886898	7.018602	7.422428	5.506263	4.742046	4.642152	6.348081	5.537961	6.468432	3.543661	3.182375	3.604272	3.207101	4.749868	6.932191
LOC107801557	2.645988	2.239242	2.605217	2.617276	1.720171	2.902817	1.29082	1.522582	2.408124	1.211101	1.438857	1.186133	1.803398	1.178222	1.330173	2.796727	4.277801	3.744097	1.36988	1.233362	1.575958	3.179311	3.596167	4.769225	0.477905	0.525957	1.084997	0	0.649935	3.338886
LOC107824191	0	0.253699	0.24198	0.168125	0.12316	0.04988	0.155653	0	0.17423	0.180963	0.065064	0	0	0.101229	0	0.10372	0.403392	0.069179	0.047078	0	0.042313	0.271219	0.12277	0.242459	0.058657	0.064555	0.10967	0	0	0
LOC107811042	0	0	0	0	0	0	0	0	0	0	0	0	0	0	0	0	0	0	0	0	0	0	0	0	0	0	0	0	0	0
LOC107807503	12.86675	11.80696	12.00382	8.909184	9.900305	8.135393	12.37241	12.95597	12.3563	7.350018	8.795073	9.253344	6.522578	8.126682	10.15388	12.61621	10.52457	12.05126	13.20568	10.01264	16.11171	14.45829	14.46585	16.80272	12.43328	13.60148	8.491126	16.84494	14.17509	30.94898
LOC107799206	0	0	0	0	0	0	0	0	0	0	0	0	0	0	0	0	0	0	0	0	0	0	0	0	0	0	0	0	0	0
LOC107805781	2.642303	2.719774	2.783299	1.661573	1.265316	2.105533	1.09507	0.690577	1.050654	0.485003	1.416835	0.47272	0.622895	0.678264	0.574303	1.135098	0.870917	1.691838	0.946315	1.630386	0.65207	0.752859	0.850015	1.364621	0.117906	0.129761	0.440893	2.223082	0	0
LOC107781900	0.463911	1.49901	0.989839	0.229244	0.335863	0.136026	0.176865	0.360344	0.158378	0.164499	0.147861	0.071259	0.328639	0.138028	0.21643	0.785698	0.774124	0.440192	0.213975	0.142704	0.038463	0.176102	0.2604	0.2204	0.159961	0.117363	0	0	0	0
LOC107782162	0.0194	0	0.016864	0	0	0	0	0	0	0	0	0	0	0	0	0	0.037484	0.028927	0	0	0	0.016201	0	0	0	0	0	0	0	0
LOC107824984	11.31958	10.1542	9.747043	15.55982	14.17402	13.26064	15.95805	15.03989	13.45125	17.1124	17.59684	15.75806	13.6314	13.68887	16.82434	9.244418	9.577306	11.34519	16.2723	15.89909	10.84308	14.98255	13.20293	12.70554	13.52382	18.15255	12.03254	8.909703	11.99608	5.502412
LOC107788158	18.04702	18.7973	17.86245	16.65032	14.61396	19.56742	12.84595	12.14832	12.48583	11.22596	13.22735	9.186793	9.88881	8.131566	9.510379	15.10359	15.36346	18.06029	12.43307	13.73341	10.08038	13.19559	16.05376	16.83507	6.874123	9.128086	7.240779	12.77839	4.301227	1.578323
LOC107802366	11.30927	13.37198	12.18515	22.3786	19.72468	20.79802	19.9197	20.52104	20.00586	19.64719	18.99042	19.61163	25.94127	23.63673	22.42084	14.12745	14.05965	14.84395	21.44174	20.11559	18.48421	20.33088	19.39976	21.2768	19.52854	20.49824	19.34646	17.02679	22.63842	22.08218
LOC107813689	0.288027	0.318751	0.500751	0.167746	0.254865	0.331783	0.362371	0.298833	0.328358	0.200616	0.043278	0.104286	0.251928	0.24689	0.232275	0.275963	0.377636	0.536841	0.271393	0.278458	0.412791	0.566984	0.3085	0.344052	0.104044	0.400769	0.097264	0	0.79238	0
LOC107813387	4.195466	2.607034	2.652385	5.816498	5.793632	7.312703	4.371989	4.725313	4.834091	4.958921	4.323648	7.303708	7.784086	9.015397	5.480506	1.894828	3.991762	2.464405	5.611855	4.194362	4.811907	3.503744	3.700679	4.650864	6.590247	4.06869	7.738583	9.091975	13.46565	3.930485
LOC107759991	3.908937	3.67213	3.34568	3.21441	2.234972	2.844827	4.690912	3.442668	4.065101	1.993816	2.108423	2.235457	2.61089	2.361854	2.283769	4.391824	4.589733	4.887021	2.685029	1.984015	2.687474	3.616013	4.508846	3.519919	0.684289	0.585739	0.852932	0	0.86857	0
LOC107815162	0.351542	0.106796	0.203725	0	0.103689	0.125984	0.098284	0	0.220029	0.114266	0	0.098997	0	0.383515	0.120271	0.349293	0.226413	0.087363	0	0.099127	0.106871	0	0	0.122477	0	0	0	0	0	0
LOC107787135	0	0	0	0	0	0	0	0	0	0	0	0	0	0	0	0	0	0	0	0	0	0	0	0	0	0	0	0	0	0
Novel04951	0.223726	0.407797	0.21879	0.304026	0.222713	0.0902	0.375297	0.573472	0.446342	0.681752	0.333363	0.945048	0.653768	0.518657	0.717581	0.250081	0.189121	0.083398	0.454043	0.212914	0.102021	0.09342	0.123338	0.02923	0.141429	0.155649	0.396639	0	0.269274	0
LOC107802188	6.727912	6.061184	6.229364	6.352031	3.854818	6.124772	4.410613	4.118665	4.59819	3.594485	4.139211	3.941727	4.620025	5.005702	5.106251	4.725507	5.827343	6.072944	2.798817	3.445339	2.774116	3.896462	4.661169	4.984376	1.173259	1.39883	1.249141	1.843452	1.737425	4.781574
LOC107799130	1.303288	0.339367	1.186869	4.779076	3.459705	6.538905	0.936963	1.060538	1.28185	0.363106	0.565727	0.262156	0.207263	0.406235	0	0.231241	0.779434	0.185077	2.078193	1.732487	2.264039	0.725607	1.861235	1.945994	0	0	0.293406	0	0	0
LOC107808165	0	0	0	0	0	0	0	0	0	0	0	0	0	0	0	0	0	0	0	0	0	0	0	0	0	0	0	0	0	0
Novel04805	0.026535	0.024183	0	0.697137	0.727873	0.713205	0.823469	0.906883	0.871922	0.077624	0.204669	0.403513	0.088617	0	0.027235	0	0.025635	0.019783	0.619291	0.965206	0.871208	0.553998	1.146868	0.998431	0.033548	0.110764	0.15681	0	0.127748	1.640689
LOC107783993	3.362467	7.286638	4.936489	13.47108	10.31448	9.720503	7.332639	7.022847	7.155486	13.11532	12.15558	16.60235	11.06307	17.44474	21.16702	7.016	3.970298	5.292264	16.60506	15.92873	17.4457	8.174644	5.997746	8.434679	7.935537	11.22871	10.1562	21.37457	8.993396	0
LOC107765286	0.19501	0.391001	0.440748	0.494674	0.345116	0.335456	0.261702	0.599839	0.292935	0.114096	0.246134	0.5931	0.260506	0.382944	0.480368	0.552223	0.376793	0.523398	0.712379	0.461903	0.426846	0.228001	0.275219	0.815299	0.345173	0.054269	0.50707	0	0.37554	0
LOC107782072	1.256486	1.109347	1.382362	2.34777	1.250799	1.583058	1.218536	1.761335	1.714171	0.248875	0.963647	0.381482	0.305974	0.492618	0.604511	1.111898	1.365605	1.127044	0.697263	0.332156	0.519252	2.557728	3.117104	2.872797	0	0.136587	0.092817	0	0	0
LOC107805491	6.365136	5.438471	6.532105	4.605183	4.302444	6.795776	3.447919	3.24802	5.104389	2.974097	3.16146	4.107769	4.477805	4.388254	1.996195	4.809186	5.978444	6.228441	3.005183	3.627037	3.346001	3.654538	4.171863	6.468037	1.39713	0.676547	2.141988	0	2.340863	2.733097
LOC107829638	0	0.041403	0.118472	0.329252	0.080397	0	0	0.038816	0.042651	0	0.031855	0	0	0	0.099122	0	0.033854	0.043888	0.067739	0.092197	0.07686	0.124296	0	0.040071	0	0.057436	0	0	0	0
Novel00644	3.111669	2.310735	3.105635	2.035955	2.243519	1.796617	2.464897	2.314049	3.624665	2.528559	2.303121	2.044641	1.924419	1.697339	0.946291	4.208228	3.562825	5.62786	1.929587	2.729749	3.048109	4.282937	4.981118	8.492157	1.019951	0.160358	1.770765	0	1.10968	0
LOC107798568	8.89277	10.86762	10.57059	15.68556	15.14169	10.10404	24.18443	35.8901	34.94531	15.01102	20.54919	18.46911	12.37444	15.61807	18.32374	8.384036	8.753875	10.59785	15.68905	16.04267	12.96428	34.09477	44.98421	29.08121	12.94667	19.31038	25.67916	7.226804	5.675961	4.165556
LOC107806023	0	0	0	0	0	0	0	0	0	0	0	0	0	0	0	0.086072	0.167376	0.172222	0.586013	0.195412	0.632034	0.626977	0.560338	0.543248	1.606314	2.008892	0.819078	4.129982	3.05835	24.99563
107817467	0.717531	0	0	0	0	0	0	0	0	0	0	0	0	0	0	0	1.386391	0	0	0	0	0	0	0	0	0	0	0	0	0
LOC107776393	0	0	0	0	0	0	0	0	0	0	0	0	0	0	0	0	0	0	0	0	0	0	0	0	0	0	0	0	0	0
LOC107803570	0.637441	0.599104	0.458876	0.60456	0.502357	0.61037	0.467818	0.510607	0.504949	0.485614	0.47491	0.210363	0.631995	0.478099	0.562248	0.7348	0.442622	0.608901	0.394163	0.454977	0.17259	0.623841	0.606193	0.582972	0.113332	0.152445	0.200123	0	0.287707	0
LOC107802517	0	0	0	0	0	0	0	0	0	0	0	0	0	0	0	0	0	0	0	0	0	0	0	0	0	0	0	0	0	0
LOC107789272	0	0.114616	0.054661	0	0	0	0	0	0	0	0.044092	0	0	0.2058	0	0	0	0	0	0	0	0	0	0	0	0	0	0	0	0
Novel03580	0.602561	0.588386	0.523793	0.506898	0.64744	0.601553	1.010788	0.992914	0.888974	1.259085	0.965751	1.236289	1.19782	0.892137	0.927678	0.481103	0.914767	0.802205	0.393067	0.473316	0.510292	0.287551	0.531499	0.854724	0.652989	0.538984	0.661309	0	1.243261	0
107792033	0	0	0	0	0	0	0	0	0	0	0	0	0	0	0	0	0	0	0	0	0	0	0	0	0	0	0	0	0	0
LOC107782549	0	0	0	0	0	0	0	0	0	0	0	0	0	0	0	0	0	0	0	0	0	0	0	0	0	0	0	0	0	0
LOC107784692	27.74141	27.82813	27.02825	19.73477	18.83617	20.68429	13.74213	14.74147	17.24671	13.49543	13.49041	13.6321	13.05754	16.24941	10.19169	23.67907	29.73841	25.91084	15.99579	14.96239	19.52734	13.47555	18.99554	19.45994	7.218724	6.649243	11.73624	44.38263	11.05506	19.18673
LOC107762686	2.943824	3.330537	4.323834	0	0.269471	0	0.085142	0.086734	0.190606	19.4013	0.498258	0.343038	5.085164	2.99008	0.208377	1.664217	2.35364	0.983851	0.721046	0.257614	0.37032	0.084775	1.074475	1.591492	118.9721	2.259926	101.6214	29.03793	66.95339	12.55318
LOC107819295	4.925302	3.68183	4.81063	5.489856	5.166218	5.62254	5.175433	4.988505	6.208751	4.074278	4.695378	3.90389	4.990069	5.01102	4.771196	4.247698	4.277084	4.724118	3.369342	2.738633	3.381587	4.020816	5.271908	7.49052	3.498359	4.004116	3.957183	3.957627	4.928932	10.26536
LOC107780748	0.522305	0.680024	0.583751	0.54078	0.858316	0.521432	0.21904	0.605655	0.245182	0	0.392401	0.031518	0.041531	0.325605	0.038291	0.444826	1.369604	0.305958	0.227143	0.347156	0.204151	0.934695	1.678293	0.896858	0	0.259553	0.220472	0	0.179612	0
LOC107805866	0	0	0	0	0	0	0	0	0	0	0	0	0	0	0	0	0	0	0	0	0	0	0	0	0	0	0	0	0	0
LOC107821555	0	0.02918	0	0	0	0.068844	0.026854	0	0	0	0	0	0	0.104787	0	0.095436	0.061862	0.02387	0	0.027084	0.0584	0.026738	0	0.033464	0	0	0	0	0	0
LOC107775071	0.06258	0	0	0	0	0	0	0	0	0	0	0	0	0	0	0.046635	0	0	0	0	0.057074	0	0	0	0	0	0.073965	0	0	0
LOC107813958	0	0	0	0	0	0	0	0	0	0	0	0	0	0	0.115865	0	0	0	0	0	0	0	0	0	0	0	0	0	0	0
LOC107809008	0	0	0	0	0	0	0	0	0	0	0	0	0	0	0	0	0.059815	0	0	0	0	0	0	0	0	0	0	0	0	0
LOC107772556	18.24171	17.33757	16.87652	15.46367	10.83784	14.42888	18.68799	20.26207	13.33391	15.50094	20.28303	12.32888	12.76435	8.102471	16.18184	20.8761	20.1406	18.16567	13.7506	15.54145	13.19056	23.50408	21.14737	16.34248	9.225226	13.7788	12.62814	0	7.527607	8.056516
LOC107806890	7.306476	5.381893	5.56832	8.735055	9.06316	11.11945	7.61135	7.924716	7.266835	9.987645	9.498001	9.244978	9.433573	8.043801	9.279791	6.290105	6.510737	6.641214	10.63027	10.04729	9.158687	7.35563	7.945638	8.12492	9.828139	10.86276	13.01265	9.543678	9.637254	8.251515
LOC107797843	1.067613	1.307466	0.739542	4.669778	5.550078	3.38964	3.246017	3.549018	3.304531	4.391961	4.924456	4.129236	3.064054	4.986429	5.102173	1.230673	0.789669	1.156616	5.806025	4.783751	6.602774	3.594228	2.972257	3.10351	5.335878	8.936244	2.740543	5.964799	3.854902	3.094318
LOC107761510	18.45778	20.07551	20.34495	17.40345	14.85946	16.74638	15.2128	12.67958	15.63065	9.803265	13.30181	9.223649	10.60342	10.14686	10.72923	19.54059	16.81104	21.26818	10.88368	13.84	9.212605	18.28893	17.26942	21.04894	3.744324	5.145441	5.695143	2.289666	8.246548	2.969486
LOC107825551	1.323785	1.343565	1.464576	1.689897	0.958395	1.326192	1.186019	1.46526	1.016859	0.674766	1.139202	0.762525	1.105242	1.24725	0.957263	0.919226	1.714872	1.345824	0.79376	1.196183	0.301829	1.105527	1.326893	1.352174	0.646644	0.209313	0.640067	0	0.434534	3.720528
LOC107780795	9.997008	16.9403	15.36478	26.03395	33.83683	15.28424	27.25896	31.04897	23.37486	56.63677	43.92521	51.57367	33.25948	28.11572	44.0857	36.00256	13.22001	21.28269	71.27991	64.43874	61.81139	27.04618	28.80107	25.18853	24.6057	33.90258	22.4183	46.21204	40.62613	21.15274
LOC107782399	0	0	0	0	0	0	0	0	0	0	0	0	0	0	0	0	0	0	0	0	0	0	0	0	0	0	0	0	0	0
LOC107771393	0.170729	0.03112	0.059365	0.030934	0	0	0.114558	0.145876	0.064115	0.033297	0.143659	0.028847	0.342105	0.26076	0.140186	0.101782	0.29689	0.050914	0	0	0.031142	0.199613	0	0	0	0	0	0	0	0
LOC107768489	1.743465	3.661502	3.360584	5.905924	5.500178	2.933904	7.947388	7.44832	3.202517	4.656792	7.760338	1.985251	3.544161	2.811704	13.45974	3.78472	3.515132	4.069025	3.999813	6.668895	3.59494	13.10412	4.881007	3.406852	6.516973	13.81711	1.88145	0	8.758604	14.06101
LOC107767794	1.663494	1.590024	2.116156	1.249741	1.112952	1.308629	2.00779	2.114668	2.361687	1.186914	1.507841	1.850969	2.529321	2.301685	1.290934	0.967522	1.842256	1.39145	1.482145	1.269913	1.628144	1.694179	1.181007	2.162752	1.231119	0.677453	1.486575	0	1.171998	0
LOC107777453	2.976123	2.441132	2.393049	2.763622	3.489347	2.879726	2.558607	2.670024	3.772056	2.104019	1.512973	2.954316	3.313061	3.733824	1.985497	2.384146	4.097138	3.106356	1.962976	2.265836	2.307133	2.112622	3.872077	3.810537	1.316953	0.724685	0.703508	0	2.50742	0
LOC107806312	11.52134	9.057749	15.67883	3.234702	2.540567	2.611923	1.45106	2.421723	3.21392	0.28715	0.490405	0.466464	0.204884	1.405503	0.151121	6.308992	7.503364	8.013345	1.419362	1.089837	1.611398	1.321841	3.019553	3.270219	0.046538	0.051218	0.478563	0	0.708854	0
LOC107770209	0	0	0	0	0	0	0	0.029099	0	0	0	0	0	0	0	0	0	0.025391	0	0	0	0	0	0	0	0	0	0	0	0
LOC107795228	1.881041	4.069196	1.976554	0	0.036582	0.066671	0.052012	0	0	0.18141	0.028989	0	0	0.022551	0	3.04997	2.496214	3.035956	0.041951	0.017486	0.075408	0	0.200563	0	0	0	0	0	0	0
LOC107821239	0	0	0	0	0	0	0	0	0	0.073492	0	0	0	0.164442	0	0	0	0	0	0	0	0.062941	0	0.078773	0	0	0	0	0	0
LOC107794362	8.815758	9.383331	7.775225	0.991042	0.740218	2.559757	0.053972	0.054981	0.120827	0.188244	0.631698	0.054363	0	0.351006	0	6.569513	5.719287	6.76442	1.110041	0.870952	0.117374	0.053739	0.340558	0.134514	0	0	0	0	0.619594	0
LOC107796626	3.468858	1.225057	2.563094	0.471391	0.306947	1.491779	0.036369	0.037049	0	1.268466	0.881733	0.146529	0	0.094609	0.044504	1.809498	3.85389	4.170217	0.307996	1.797329	0.355911	0.072423	0.076494	0.181282	0.383748	1.025666	1.024978	0	0.208754	0
LOC107807518	0	0	0	0	0	0	0	0	0	0	0.021124	0	0	0	0	0	0	0.01615	0	0	0	0	0	0.022642	0	0	0	0	0	0
LOC107817728	0.106952	0.097474	0.023243	0	0	0.028747	2.265058	1.644889	0.577365	0.234656	0.618707	0	1.458491	1.020945	1.481931	0.059776	0.077493	0	0	0	0	2.344609	0.141508	0	0	0	0	0	0	0
LOC107799644	0.302075	0.110122	0.157552	0.164199	0.080189	0.324767	0.329372	0.335531	0.198521	0.147281	0.360086	0.127601	0.302646	0.164774	0.124016	0.292638	0.437744	0.382856	0.214567	0.178874	0.110199	0.201816	0.42632	0.505166	0.26734	0	0.285622	0	0.14543	0
LOC107799446	0	0	0	0.032794	0	0	0	0	0	0	0	0.091745	0	0	0	0	0	0	0.036732	0	0	0	0	0	0.045766	0.050368	0	0	0	0
LOC107829649	0	0	0	0	0	0	0	0	0	0	0	0	0	0	0	0	0	0	0	0	0	0	0	0	0	0	0	0	0	0
Novel04171	4.693771	4.184798	4.050629	3.481971	3.461131	3.912771	3.737172	3.196764	4.757979	1.65834	2.122634	1.436749	1.817451	2.077954	2.09459	3.852653	3.5488	4.260146	2.346943	1.409853	2.698764	3.635841	4.710246	6.541237	0.086005	0.094653	0.522606	0	0.655	0
LOC107797677	6.060248	5.425844	6.196334	1.910713	1.228415	3.76005	1.500266	1.687995	1.955026	1.249587	1.198083	1.014951	2.050653	2.300895	0.712431	4.07842	4.204042	4.916249	1.083616	1.151781	0.41392	1.293137	2.825836	2.483441	0.30378	0.037147	0.4102	0	0.257059	1.650724
LOC107821808	0.437044	0.458059	0.417905	0.098985	0.212699	0.281926	0.256598	0.224054	0.225674	0.127852	0.321779	0.110768	0.170283	0.119198	0.269142	0.374539	0.40111	0.325835	0.332612	0.184855	0.099648	0.456234	0.250576	0.13704	0.027628	0.182435	0	0	0	0
LOC107825501	4.648114	7.354185	14.37764	3.526943	2.171307	4.895823	2.552873	2.681249	4.120187	0.943312	1.637902	0.777398	2.153788	1.82757	1.549871	12.72983	5.356651	11.80335	1.436523	1.536867	1.162003	2.66009	3.600458	5.055495	0.119322	0.06566	0.501962	1.687339	1.363107	0
LOC107819059	0.987552	1.512054	1.064488	0	0	0	0	0	0	0.03852	0	0	0	0	0	0.735925	0.457948	0.677361	0.040084	0	0	0	0	0	0	0	0	0	0	0
LOC107802490	0.718187	1.439985	0.728355	0.498825	0.762599	0.411807	2.871317	1.677278	0.809119	0.583602	1.846508	0.303372	1.572341	0.705156	1.793676	0.999028	0.647574	0.481895	0.21863	0.344271	0.371166	2.079226	0.781302	0	0.211868	0.09993	0.169768	0	0.461014	0
LOC107807957	0.731164	0.684876	0.847447	0.846397	0.970473	1.179136	0.630294	0.381777	0.705517	0.356489	0.598141	0.789293	0.836551	0.819821	0.250149	0.756756	0.863336	0.893381	0.948027	1.048038	0.926158	0.356191	0.680765	0.742984	0.41085	0.536941	0.720147	0	0.29334	1.255804
LOC107818733	24.92772	24.01264	21.39484	18.08089	15.61256	20.58086	12.81384	10.8966	11.65225	24.97434	26.44025	26.70219	28.0426	22.54768	22.29253	16.34232	22.40566	19.27081	12.434	15.21477	11.72697	8.827796	8.44261	9.152036	5.751376	4.756396	7.614268	11.28281	9.747721	0
LOC107825239	4.155198	3.289395	4.271154	2.940078	3.408414	4.565171	3.714095	3.109764	4.470584	3.519484	4.33854	3.638545	4.017878	3.871348	2.863941	3.5937	4.570995	3.03004	3.139224	2.847929	2.295896	3.470115	4.012948	3.962602	1.840619	0.928441	3.082887	4.338035	4.526589	3.750689
LOC107820808	0.529981	0.563514	0.65266	0.280079	0.664363	0.474828	0.555647	0.150943	0.248785	0.344532	0.495497	0.298495	0.639147	0.674547	0.317309	0.460766	0.341337	0.724392	0.31371	0.336245	0.040279	0.479484	0.428522	0.830904	0.27919	0.368715	0.417596	0	0.212626	0
LOC107770343	3.38987	3.249642	3.579745	2.479589	3.332866	3.617528	3.348693	3.025125	3.583326	2.301643	3.169305	2.333512	3.074826	3.013333	2.371058	3.349468	3.42045	4.343197	2.879244	2.591457	3.206122	4.927982	5.20497	5.117798	3.809623	3.633649	2.671035	0	6.165348	4.657794
LOC107807228	0	0	0	0	0	0	0.13127	0	0	0	0	0	0	0	0	0.181974	0	0	0	0	0	0	0	0	0	0	0.144309	0	0	0
LOC107818486	5.763761	6.224508	5.24591	8.233551	7.706191	6.62411	7.516678	8.38282	7.17545	7.117993	8.22247	6.1974	7.562786	6.958173	8.456394	5.587749	5.376268	6.088721	8.323808	8.116053	6.097052	8.691402	8.09618	6.968526	8.246583	11.11215	6.128936	7.752798	7.306894	18.99215
LOC107810662	9.997356	11.48613	9.584192	10.24544	10.72851	7.985055	13.08331	9.844517	9.985061	10.75121	14.92765	8.20644	14.10882	12.22161	11.68009	15.17748	10.58302	14.16685	9.011449	9.116847	5.706681	13.21933	10.96037	11.76465	7.19387	6.289363	4.525303	2.535292	11.77669	4.384054
LOC107796931	12.15374	11.97172	11.02743	13.79115	16.62048	11.87887	15.44525	15.94386	12.14043	17.91681	14.49063	16.35245	19.08738	19.46459	19.44642	15.30843	14.07403	13.60695	16.90047	14.53911	18.32474	13.12311	13.49977	13.34466	17.69421	22.3203	16.34776	87.79438	25.80852	27.83275
LOC107798265	0	0.198494	0	0	0	0	0.091337	0	0.102238	0	0.076359	0	0	0	0	0.11177	0.081151	0	0	0	0	0	0	0	0	0	0.128708	0	0	0
LOC107801179	0	0	0	0	0	0	0	0	0	0	0	0	0	0	0	0	0	0	0	0	0	0	0	0	0	0	0	0	0	0
LOC107787072	3.634679	2.576437	3.931884	4.170924	2.43002	5.774748	1.829137	1.518278	2.237016	3.347367	2.945094	2.934203	2.382748	1.453927	1.575115	2.558091	4.408765	2.92057	2.294893	2.869709	2.430918	1.618888	2.529199	3.376858	1.021186	0.16858	1.909288	2.888121	1.749864	0
LOC107806113	2.390778	2.178899	2.078252	0.590706	0	0.701012	0.182295	0.742816	0.612155	0.635812	1.219211	0.734471	0.483899	0.237111	0.446151	0.647858	1.259832	1.944465	0.88218	1.287003	0.198221	0.726037	0.383423	0.681502	0	0	0	0	0	0
LOC107796233	1.647946	1.851178	1.965556	1.952994	1.364953	1.85415	1.51078	1.547217	2.293761	1.121131	1.538481	0.688021	1.386553	3.031221	2.17326	2.056272	1.740152	1.514337	0.943055	1.053641	0.707787	2.336412	2.864938	2.022857	0.532989	0.559918	0.486935	0.685183	0.461267	1.777238
LOC107776402	16.74757	15.81982	17.40172	17.3851	17.79094	21.47553	17.00993	14.30958	17.85256	13.99192	15.59671	16.0648	20.34291	16.27241	14.05579	15.01537	16.22169	17.10325	15.53383	15.05275	15.07518	12.38373	15.92656	18.18832	5.78977	7.221508	10.41261	6.237958	8.188858	10.78674
LOC107765485	0	0	0.025296	0.026363	0	0	0	0.024864	0.02732	0	0.020405	0.024584	0	0.031747	0	0.021685	0	0.021695	0.029529	0	0	0.12151	0.025668	0	0	0.080981	0	0	0	0
LOC107820087	149.1319	133.5912	151.6276	94.81441	87.46298	132.3147	83.63129	92.647	110.5632	64.30614	68.52213	69.02776	58.2531	71.49923	55.17454	112.6535	146.7347	136.4705	68.57406	75.15818	77.30845	93.91906	116.3383	141.9059	32.75732	18.0255	59.61798	18.23707	34.37631	56.76432
LOC107832503	0	0	0	0	0	0	0	0	0	0	0	0	0	0	0	0	0	0	0	0	0	0	0	0	0	0	0	0	0	0
LOC107797822	0	0	0	0	0	0	0	0	0	0	0	0	0	0	0	0	0	0	0	0	0	0	0	0	0	0	0	0	0	0
LOC107779746	255.6892	223.242	285.5155	132.4989	117.3271	188.2653	117.0326	121.7802	167.968	53.9976	65.97851	71.65471	44.64731	71.33006	46.26308	160.3257	263.6733	211.0515	79.68998	74.09665	111.0664	125.6183	189.4492	223.0361	13.76202	13.41772	41.70416	0	13.71662	54.20451
LOC107817787	13.14057	13.86112	16.02367	13.94392	12.86577	12.68878	14.74644	12.52715	17.07753	8.898411	9.256832	10.84454	9.142664	11.88006	8.242128	11.15242	15.10457	13.37996	8.333837	9.572125	11.98442	12.14256	16.04474	19.01213	4.307248	2.624113	6.183676	0	8.200807	0
LOC107812728	0	0	0	0	0	0	0	0	0	0	0	12.81949	0	0	0	0	0	0	0	0	0	0	0	0	0	0	0	0	0	0
LOC107774165	5.170534	6.453808	6.937373	7.637351	8.752612	7.734201	6.316571	6.050527	7.703603	4.165068	5.359556	7.501928	8.38361	7.234938	3.576386	7.119837	7.166984	7.793507	7.527875	7.22647	11.89155	4.130307	6.246249	8.811263	8.100353	5.786817	9.963841	24.11519	12.4464	6.950042
LOC107784863	9.070442	7.000535	7.813709	7.84721	8.098432	9.312563	5.620155	4.468487	4.986689	3.585741	3.036852	4.280213	3.183847	5.259721	3.187089	6.028569	7.104981	5.909484	12.10624	7.603844	14.30901	4.845247	3.892247	4.185052	2.892777	3.297341	4.539325	5.843828	5.507718	10.10521
LOC107769316	13.90357	5.103753	7.511832	6.604115	12.26007	12.82002	3.036855	3.712371	6.435993	4.801698	1.929527	12.39868	10.90959	11.06008	2.279275	6.979255	9.607622	8.602091	7.837989	6.084923	9.906475	2.136789	4.130538	6.509127	5.859446	1.948015	6.162337	6.904873	11.85338	0
LOC107789085	5.782011	5.844454	5.745837	4.559623	4.348858	5.693629	3.317582	3.188744	3.516127	2.524372	3.685772	2.18706	3.554767	3.655709	2.468211	5.043209	5.230442	6.299551	2.746909	2.912477	2.217179	3.237429	3.998954	5.026973	0.448582	0.2377	1.211459	0.939763	1.202038	2.437571
LOC107775161	1.77743	1.52073	0.788307	0.2629	2.022154	0.545987	1.429957	1.022787	1.021671	0.955039	1.55156	1.287104	0.605712	5.104947	6.77599	14.19153	14.92866	14.14394	9.128489	10.77056	7.60899	14.02586	14.27027	12.13237	3.439609	2.321745	5.273383	0	6.461543	2.242881
LOC107787632	0.331517	0.201425	0.288181	0.901014	0.391131	0.237615	0.185372	0.755353	0.518739	1.293086	1.627222	0.56015	0.492067	0.843895	0.680521	0.411745	0.747305	0.659094	0.224267	0.280441	0.100783	0.830577	0.682314	0	0.698565	0.461282	0.522436	0	0	0
LOC107759324	0	0	0	0	0	0	0	0	0	0	0	0	0	0	0	0	0	0	0	0	0	0	0	0	0	0	0	0	0	0
LOC107766845	0.197496	0.15187	0.241424	0.234835	0.196603	0.15925	0.093177	0.0791	0.098504	0.078237	0.164451	0.067783	0.089316	0.060598	0.076014	0.243758	0.214648	0.262275	0.25677	0.224498	0.197006	0.113392	0.087102	0.187071	0.117045	0.0687	0.124007	0	0.178278	0
LOC107778038	0	0	0	0	0	0	0	0	0	0	0	0	0	0	0	1.452702	1.391999	1.011038	2.064131	1.720764	2.62819	1.344871	1.420463	1.328818	1.714536	2.476594	1.001758	6.061316	2.040249	26.20323
LOC107764306	5.55426	4.429271	5.096434	8.386464	6.27999	10.11839	8.864276	9.425509	4.490337	6.168342	9.033426	6.191353	4.809062	5.554473	9.501234	4.771395	5.962092	5.463736	8.297562	9.788588	7.035526	11.27415	7.689061	6.369717	4.779047	6.118276	3.920578	0	3.342523	0
LOC107763083	32.8683	36.57901	35.06011	30.48882	24.68087	32.02896	25.31648	23.77593	28.03056	21.3246	20.79764	25.61077	24.41726	28.78616	23.1173	26.15242	34.66323	29.67901	24.11743	24.70264	28.48359	23.73096	27.7792	23.19917	15.47985	13.75659	20.1977	37.45894	12.92396	16.1936
LOC107806507	0	0	0	0	0	0	0	0	0	0	0	0	0	0	0	0	0	0	0	0	0	0	0	0	0	0.057615	0	0	0	0
LOC107780051	18.1514	18.68359	18.16531	17.49447	14.84682	18.10465	15.60817	15.90004	14.23453	12.40481	13.94707	13.47917	13.78789	14.14449	12.90017	15.98167	17.06423	16.73954	15.81849	14.45161	14.94068	14.03773	14.90748	14.34845	8.253887	10.99394	8.941962	10.90825	14.54007	13.20387

LOC107825262	0	0	0	0	0	0	0	0	0	0	0	0	0	0	0	0	0	0	0	0	0	0	0	0	0	0	0	0	0	0
107801969	0	0	0	0	0	0	0	0	1.366012	0	0	0	0	0	0	0	0	0	0	0	0	0	0	0	0	0	0	0	0	0
LOC107807639	0.146312	0.133346	0.127186	0	0.129467	0	0.368155	0.250026	0	0.285346	0.102594	0	0	0	0.300342	0.327096	0.2827	0	0.148467	0	0	0	0	0.152925	0	0	0	0	0	0
LOC107820548	1.180193	1.613401	1.931138	0.566045	0.522156	0.89566	0.262027	0.444878	0.097766	0.236937	0.292077	0.175952	0.115924	0.302949	0.213762	1.267488	1.542578	1.811526	0.24656	0.264272	0.158288	0.289885	0.489886	0.217683	0	0	0.082053	0	0.167114	0
LOC107777788	0	0.07233	0	0	0.070226	0.17065	0	0.06781	0	0.232168	0.05565	0	0	0	0	0	0	0	0	0.134272	0.072381	0.066278	0	0	0	0	0.093801	0	0	0
LOC107768657	3.034065	3.37966	3.150286	4.810241	6.599987	5.164819	5.761147	5.328797	5.459652	4.766628	4.875494	6.052147	7.083501	7.079756	4.152108	3.140262	4.315322	2.419109	4.831942	4.883689	5.956991	6.053031	6.541935	6.430497	5.327751	3.69397	3.984468	15.06795	9.53517	18.23897
LOC107778660	11.14463	7.973211	11.86754	2.927976	2.563991	4.792736	3.271618	4.142189	5.17922	2.173484	2.149021	2.165517	1.302668	3.465099	1.086662	7.765167	8.936319	10.38599	1.69632	2.026932	2.134459	4.560514	5.799877	7.513195	0.281804	0.077535	0.856184	0	0.536542	0
LOC107772100	4.1393	3.67213	3.617345	3.371007	3.877034	3.811122	2.862396	3.21692	4.650986	2.189929	2.439318	3.366789	2.696127	2.594168	1.920848	3.724504	2.914101	3.72621	3.41831	3.222183	3.67471	2.94199	3.942451	4.510497	1.976419	2.175143	2.576275	0	2.226007	0
LOC107831612	0.194927	0.152273	0.121033	0.302733	0.123203	0.269448	0.326988	0.190344	0.183006	0.190079	0.605311	0.32936	0.433992	0.303794	0.25723	0.58104	0.403535	0.519024	0.254313	0.32979	0.07619	0.186044	0.221064	0.203738	0.457688	0.154987	0.131651	0	0.402193	0
LOC107761392	1.293327	1.650192	1.292901	2.167625	3.891032	3.962883	3.796693	2.099603	3.096294	2.396199	2.856678	1.69359	2.951488	2.821913	3.119474	2.023957	2.124087	1.735615	1.574855	0.929956	0.825675	3.18627	2.053441	1.8925	0.81758	0.899785	0.535013	0	0.933981	0
LOC107801382	10.98876	10.74933	8.533385	7.300525	8.621573	11.89302	6.083053	5.445676	4.333028	10.35825	10.32514	12.81138	9.215408	9.910244	10.07552	9.389867	11.46535	10.48651	6.987732	6.940816	5.07778	4.037881	5.492595	5.130168	3.519594	1.936741	4.762205	5.239012	1.76346	4.529678
LOC107799203	21.06803	21.8845	22.35172	32.3119	24.29381	36.87428	27.02776	24.73379	22.19364	20.9484	28.90606	18.77989	19.80597	25.83458	20.55949	19.40856	28.12627	23.37515	21.8413	26.90857	19.74393	27.3961	27.25321	22.06364	13.73767	11.71431	18.8716	0	6.788554	15.38584
LOC107760226	207.2467	218.4702	174.1288	472.8244	505.9204	440.4551	469.6326	433.1333	450.7598	344.0929	332.53	406.011	645.9963	570.6811	548.4823	218.9031	263.1369	207.7474	447.214	405.6942	462.011	446.4431	419.6266	360.7284	137.8042	148.7663	140.5691	201.0663	267.9357	133.359
LOC107796691	0	0	0	0	0	0	0	0	0	0	0	0	0	0	0	0.080316	0	0	0	0	0	0	0	0	0	0	0	0	0	0
LOC107776630	14.98875	20.4906	15.46656	11.40543	11.1877	10.28909	20.30467	17.22929	18.27348	9.91044	15.34932	8.813936	10.53359	7.881896	7.858038	27.92409	20.80906	22.77157	7.358578	8.186914	7.105447	19.18149	19.33225	14.90547	2.522168	3.075849	6.499932	0	2.076581	0
LOC107760547	0.606715	0.368631	0.351603	0.091609	0.402646	0.326147	0.381659	0.518394	0.047468	0.147906	0.070905	0.085428	0.112567	0.110316	0.259465	0.037677	0	0.113083	0.051304	0.04277	0.138334	0.042224	0.044597	0.052845	0	0	0	0	0	0
LOC107830962	0	0	0	0	0	0	0	0	0	0	0	0	0	0	0	0	0	0	0	0	0	0	0	0	0	0	0	0	0	0
LOC107814277	3.60687	3.250692	3.657938	0.399378	0.212773	0.258522	0.067227	0.102727	0.150502	0.039079	0.056203	0.067715	0	0.306049	0.082266	1.075137	2.555329	0.776844	0.162667	0.237313	0.146201	0.167344	0.38885	0.083776	0	0	0.094734	0	0	0
LOC107793344	0.033815	0.030818	0.05879	0.030635	0	0.036355	0	0	0	0.098922	0.142267	0	0.037644	0	0.034707	0.025199	0	0	0	0.028605	0.06168	0.05648	0	0	0	0	0.039967	0	0	0
LOC107827401	8.68236	9.318481	9.500444	11.21218	8.541975	9.109422	9.725662	9.370666	9.581449	7.278131	9.412486	6.016068	7.503343	6.896301	9.478066	10.69837	9.436376	10.3058	7.80558	9.358023	5.713098	11.46464	10.54712	9.31361	3.562757	4.47734	4.118243	4.084944	3.483331	9.418305
LOC107790477	11.27412	8.54732	8.2826	4.790535	4.855616	6.596859	6.150643	5.029575	7.353047	5.059044	5.981535	6.026684	6.886119	7.728011	4.403294	7.323421	7.470004	7.996226	2.328536	2.616381	3.048259	4.582674	6.248326	6.882519	0.946056	0.763532	1.474016	0	2.161498	6.16899
LOC107782506	9.976161	11.4592	8.757588	12.51401	10.15083	12.56608	14.12727	12.35714	11.61982	12.03051	15.43931	11.95233	12.11321	9.252049	11.83472	12.96228	13.2306	15.18337	12.65889	16.77842	8.721568	18.79895	17.52984	17.6047	8.20958	8.733867	9.88399	5.628691	6.441731	6.083245
LOC107805431	6.239259	7.640376	6.448745	7.322937	7.304293	7.583921	10.10661	10.53375	10.58813	5.331398	5.337166	4.655232	6.348927	5.800921	8.648437	6.103472	5.385528	6.282102	6.331169	5.531908	4.614821	13.14281	11.29056	11.69795	3.713745	3.758989	3.674481	4.599951	7.019193	9.279999
LOC107812120	0	0	0	0	0	0	0	0	0	0	0	0	0	0	0	0	0	0	0	0	0	0	0	0	0	0	0	0	0	0
LOC107817711	0	0	0	0	0	0	0	0	0	0	0	0	0	0	0	0	0	0	0	0	0	0	0	0	0	0	0	0	0	0
LOC107811882	0.867257	0.895009	0.742801	0.519942	0.58684	0.644458	0.684617	0.468578	0.455005	0.32335	0.715436	0.301693	0.55371	0.473067	0.549785	0.883884	0.517491	0.694119	0.220008	0.377609	0.27916	0.702967	0.584983	0.5732	0.064499	0.053238	0.120592	0	0.429809	0
LOC107775126	14.57672	13.87934	16.02393	11.92116	10.71524	10.85437	10.87031	10.00741	11.87476	5.807334	6.20431	5.390727	5.114348	4.022029	5.850564	13.22953	11.096	12.91844	7.4245	8.348547	8.819701	10.84711	12.25727	14.90953	1.541759	1.973	2.983146	4.056192	3.686367	1.753503
LOC107762798	0	0	0	0	0	0	0	0	0	0	0	0	0	0	0	0	0	0	0	0	0	0	0	0	0	0	0	0	0	0
LOC107777143	4.547059	5.18459	4.602884	3.673582	3.431328	4.253756	4.243075	3.330112	3.603684	2.073019	3.643887	2.827065	2.892486	2.297776	2.46481	3.740523	4.602025	4.285227	2.556698	2.86406	1.741379	3.123376	3.90663	3.332982	0.572398	0.766897	1.233055	0	0.568602	3.651319
LOC107807078	1.017756	1.834038	1.568357	2.388911	2.251445	2.213294	3.06533	2.391397	1.44413	2.176604	3.187103	1.426534	1.570723	2.00615	4.071556	2.180496	1.541896	1.776236	2.816588	3.218786	1.603274	3.766846	2.397343	1.486845	3.026795	4.441511	1.284923	1.654181	2.449921	8.581284
LOC107805330	1.745992	1.856466	1.517754	0.210904	0.205996	0.312859	0	0	0	0	0.04081	0.049169	0	0.063493	0	1.171004	1.686775	1.21493	0	0.147699	0	0.048604	0.462024	0	0	0	0.068787	0	0	0
LOC107795729	53.91358	40.74828	49.46589	24.03936	23.00488	36.52619	24.18575	25.03117	30.74427	15.33054	19.89696	19.24276	18.61135	22.7571	13.22383	29.43226	49.86236	36.93582	12.52909	12.45601	20.49337	20.11058	30.77906	37.1929	3.975376	1.173805	8.067161	0	4.799814	4.741903
LOC107809278	0	0	0	0	0.036611	0	0	0	0	0	0	0	0.138175	0.135412	0	0	0	0	0	0	0	0	0	0	0	0	0	0	0	0
LOC107782543	8.412951	7.950726	10.19078	8.914083	6.958832	9.851131	8.728341	8.454011	9.667014	9.29157	10.77239	9.038879	10.95346	9.537259	7.527312	8.354578	8.587005	9.462858	4.75096	4.904388	4.436853	7.163338	8.517763	10.13131	2.7285	3.231876	4.042221	0	4.754693	2.261676
LOC107804368	2.8357	3.487877	3.547215	2.840521	2.733615	2.949579	3.132558	2.265312	2.748858	1.955849	2.39254	2.415155	3.182405	2.238465	2.602873	2.989358	2.939978	3.248549	1.894921	2.18428	1.661059	2.560701	2.907986	2.240977	0.874436	0.577415	1.144438	0	1.109924	1.425493
Novel00753	0.267244	0.162373	0.619491	0.080703	0	0.191546	0.224149	0.076113	0.669067	0.086865	0.249855	0.150517	0	0	0.182861	0.265534	0.60242	0.929794	0.090393	0	0	0.297576	0.785755	0.279323	0	0	0	0	0	0
LOC107795927	0	0.092515	0.352966	0	0.089824	0	0	0	0	0	0	0	0	0	0	0.075646	0.098068	0	0.103007	0.257614	0	0	0	0.106099	0	0	0	0	0.488711	0
LOC107795191	2.725532	2.393659	2.00309	6.958636	9.361896	5.221226	11.07681	11.68618	7.653285	10.51014	8.808638	6.907814	6.978429	6.89293	7.705596	4.560674	2.920339	4.193308	16.46837	15.69912	14.98218	12.91205	12.0205	9.038227	18.57654	20.06513	14.23255	12.40362	13.59883	18.38441
LOC107820284	2.201563	2.84082	3.050666	2.310466	2.60388	2.109163	2.815525	2.830925	1.78042	1.636671	3.332027	0.791856	2.450811	1.498151	3.780955	3.118778	1.832078	2.795185	2.366707	2.581504	0.815073	4.314298	2.845592	1.685935	1.984238	2.729686	0.772892	0	1.574125	5.391127
LOC107827450	0.073983	0.134852	0.321558	0.067025	0.392789	0.397702	0.24821	0.44249	0.138916	0.072142	0.31126	0.125005	0.329434	0	0.151868	0.165396	0	0.165472	0.075073	0.125169	0	0	0.065258	0.077327	0	0	0.087442	0	0	0
LOC107776116	5.916312	9.714876	6.029636	1.663411	0.947741	1.809526	0.727229	0.610093	1.197089	0.696277	1.072894	1.120301	1.135538	0.834621	0.209391	6.233191	5.075107	4.791108	0.155262	0.388303	0.232577	0.170375	0.899755	0.426464	0	0	0.120562	0	0.245545	3.153573
LOC107785721	0	0	0	0	0	0	0	0	0	0	0	0	0	0	0	0	0	0	0	0	0	0	0	0	0	0	0	0	0	0
LOC107792069	0.984403	1.258921	2.153103	2.157627	2.023117	1.638741	2.010884	1.899259	1.624805	2.090142	2.705389	1.529165	1.520051	1.472331	3.34072	5.620156	0.812965	2.663398	2.513372	2.350468	1.013636	2.373484	2.184776	3.650927	0.421553	0.861601	0.750636	0	0.840838	1.963455
LOC107762506	0.927075	0.812418	0.681904	1.292128	1.703778	0.383354	2.213107	1.675635	1.138188	1.634182	2.250228	1.777305	3.969359	2.100587	2.269021	1.860001	1.171212	1.089928	1.8091	1.176363	1.691017	1.667561	1.352419	1.080784	0.721295	1.58764	0.168574	0	0.858321	2.20471
LOC107826561	0	0	0	0	0	0	0	0	0	0	0	0	0	0	0	0	0	0	0	0	0	0	0	0	0	0	0	0	0	0
LOC107768299	0	0	0.056633	0.059022	0	0	0	0	0	0	0	0	0	0	0	0.097098	0	0.048571	0	0.055112	0.237668	0.108815	0.114931	0.068094	0	0	0.077001	0	0	0
LOC107763670	0	0	0	0	0	0	0	0	0	0	0	0	0	0	0	0	0	0	0	0	0	0	0	0	0	0	0	0	0	0
Novel01998	0	0	0	1.624831	1.46494	1.186612	4.242887	4.047176	1.122552	0.313905	0.580436	0.194258	0.102388	0.10034	0.094401	0.03427	0	0	1.819933	2.217429	0.92271	1.152161	3.123429	0.480661	0.290711	0.127976	0.434828	3.288756	0	0
LOC107818093	51.4588	50.72786	44.70702	120.8498	114.1409	86.88546	148.2141	118.5262	165.2274	91.54549	111.1952	99.39653	220.264	166.1878	147.9318	59.45096	62.28093	58.77011	67.1693	63.59169	66.09228	112.7963	112.1551	120.5343	23.65141	17.41975	37.72347	34.98912	56.5315	40.92887
LOC107819164	0	0	0	0	0	0	0	0	0	0	0	0	0	0	0	0	0	0	0	0	0	0	0	0	0	0	0	0	0	0
LOC107784944	1.357994	1.149241	0.674557	1.406027	1.802463	0.938577	3.823812	5.428578	5.646182	4.634754	2.686638	2.37649	4.589204	6.613859	2.439163	1.445684	1.077662	2.350312	0.885856	0.697467	1.548142	3.483295	1.36896	1.926294	4.476248	0.742323	5.961575	6.93684	17.74564	5.997629
LOC107766733	0	0	0	0	0	0	0	0	0	0	0	0	0	0	0	0	0	0	0	0	0	0	0	0	0	0	0	0	0	0
LOC107832297	150.8707	152.0731	165.2751	118.8742	103.6508	111.3728	114.957	95.54627	109.5248	64.28642	69.69517	63.81965	76.40605	70.82115	68.70086	148.4142	191.6868	172.8002	75.36065	76.39599	79.8448	114.0176	103.5103	105.4413	11.41792	14.4139	33.46584	15.19632	14.3223	19.70823
LOC107822561	6.363896	5.481231	5.775165	7.307023	6.456277	8.070012	7.253762	6.094764	7.878733	6.341989	7.469971	6.636797	6.357775	5.84916	7.321278	6.496891	6.350742	5.995866	4.730873	6.034161	3.80554	7.183546	7.587312	7.333752	2.858799	2.075873	4.54603	1.667064	4.040184	2.882705
LOC107793130	0.329778	0.910005	0.334447	0.365154	0.291809	0.265914	0.968097	0.743563	0.369812	0.348373	1.104816	0.23991	0.377311	0.239848	1.560747	1.433548	0.654886	0.669295	0.390409	0.759417	0.375954	1.101625	0.767616	0.258513	0.092653	0.203939	0.054135	0	0.044102	0
LOC107824538	0	0	0	0	0	0	0	0	0	0	0	0	0	0	0	0	0	0	0	0	0	0	0	0	0	0	0	0	0	0
LOC107792814	20.25574	24.05995	21.28987	38.696	34.2701	28.85619	40.02877	38.00028	32.65961	27.13332	30.93564	21.89564	28.05784	22.89867	31.46469	22.5794	23.13407	19.5882	33.08662	36.36302	30.21559	45.74002	38.24924	27.37366	23.40902	34.63237	21.43755	16.49513	20.19014	16.85483
LOC107817811	0	0	0	0	0	0	0	0	0	0	0	0	0	0	0	0	0	0	0	0	0	0	0	0	0	0	0	0	0	0
LOC107778106	24.51169	18.90969	22.14742	15.84852	13.67972	22.03379	13.94771	11.81871	18.28599	11.20716	13.30079	13.18964	12.7376	11.98354	8.8732	16.67449	22.79603	23.54454	8.772555	9.894393	11.45582	13.25052	17.09041	24.92863	2.957574	1.627476	9.61688	14.54716	3.182789	0
LOC107805476	25.66689	23.3922	18.67467	13.19365	8.116425	8.563979	12.07993	11.82459	15.25905	6.355188	12.63784	8.972731	27.76661	30.81022	15.03	22.48471	20.28794	23.8747	3.42912	2.654497	7.191367	7.727383	6.458402	7.400558	1.627624	1.007594	2.187249	5.754061	3.873653	0
LOC107783632	0	0	0	0	0	0	0	0	0	0	0	0	0	0	0	0	0	0	0	0	0	0	0	0	0	0	0	0	0	0
Novel03042	2.376662	1.911207	2.673623	2.02648	1.360782	2.054182	1.680717	1.672328	1.575046	0.727073	0.980304	1.33858	0.881911	1.169312	0.478302	2.083637	2.521159	1.945618	0.851178	1.300907	1.615041	1.206455	2.055268	2.045718	0.176754	0.064842	0.771104	0	0.673064	0
LOC107797925	2.284476	2.490986	2.482308	2.106577	2.526818	2.587667	2.531974	2.126196	2.183066	1.63096	1.7449	2.033381	2.860999	2.536755	1.256102	1.915193	1.537015	2.189795	1.821386	2.139564	1.227765	1.941894	2.662756	2.430371	2.21778	1.759627	1.591111	0	4.124355	0
LOC107801497	0.082618	0.075296	0.071818	0	0	0	0	0	0	0	0	0	0	0	0	0	0	0	0	0	0	0	0	0	0	0	0	0	0	0
LOC107812322	16.14714	15.67328	17.04145	10.2677	7.356994	12.65551	8.588438	9.085539	8.873942	5.760551	7.517575	8.318022	6.332736	7.16087	7.006466	14.83728	15.64177	14.84407	6.882591	8.1438	6.944188	6.395279	8.260081	6.677617	2.821571	1.643969	3.982429	6.258803	2.317396	0
LOC107766029	0	0.029468	0	0.732306	1.173032	0.104286	8.271367	9.531091	5.706894	1.828678	2.76599	0.846795	2.087638	1.869518	8.130545	0	0.031237	0.072317	0.951475	1.559044	0.530791	9.20778	4.905441	2.635982	4.537566	6.703401	3.43937	9.24912	3.891587	3.998419
LOC107763613	21.77063	21.63883	22.9038	19.16679	14.29165	19.10497	18.85302	16.4233	16.77065	9.725474	16.10062	11.9315	14.56193	11.36785	11.51615	22.64357	20.60083	24.05507	13.16039	16.63777	10.4367	17.80827	18.54662	15.18922	2.823045	2.951552	4.376492	0	4.031214	1.150522
LOC107799743	4.573258	8.093599	5.732025	5.781126	5.693641	5.431362	7.448567	6.361073	4.942605	4.511359	5.779646	3.81869	4.499037	4.69913	4.475548	5.904554	5.599751	6.660532	4.85647	6.027915	3.7344	10.12545	8.349269	6.669723	3.496086	4.291565	1.634137	0	1.280075	3.28804
LOC107788348	1.680818	0.937872	1.968011	1.926727	0.940944	2.470911	0.517876	0.732722	1.223775	0.80278	1.082379	0.811429	0.610975	1.3472	0.246449	1.457045	1.358699	1.278695	0.800577	0.899542	1.157521	0.54429	0.847196	1.075586	0.086737	0.095459	0.162171	0	0.165144	0
LOC107782538	0	0	0.110785	0.115458	0	0.137019	0.106893	0	0	0	0	0	0	0.417108	0.130806	0.094972	0.738734	0.285046	0	0	0.116232	0.106432	0	0	0	0.17733	0	0	0.613564	0
Novel00968	4.370038	3.706171	2.004912	3.024261	2.900173	2.74069	3.308987	3.578301	3.532953	1.065335	2.00029	1.999795	1.824295	0.993228	0.996731	2.080578	2.755913	2.896043	2.032439	1.437626	2.103484	2.280958	4.175884	4.250371	0.230211	0.337811	0.430419	0	0.584414	0
Novel04312	0.058821	0.160823	0.051132	2.637781	2.238079	0.916971	5.525563	3.442668	2.540276	1.347902	1.814782	0.670859	0.22918	0.449193	0.120744	0.087666	0.284128	0.175413	1.462334	2.064965	0.777858	4.396476	3.216788	1.014408	0.483386	0.859368	0.695212	0	0.566367	1.818484
LOC107823288	13.24546	10.6541	13.36757	3.431736	3.041102	9.385775	0.52602	0.407251	0.494591	2.568521	3.430125	0.996091	1.75935	3.284123	1.004159	9.047986	10.29999	16.90728	3.054676	3.183166	0.777884	0.502801	1.261273	1.284779	0.063433	0	0.711594	1.79401	0.72464	0
LOC107803802	8.745633	9.513257	8.74684	1.448309	1.497815	3.841933	0.157749	0.883845	0.971168	0.3668	0.06594	0.635575	0.837485	1.128512	0	5.956632	7.086273	6.520241	1.049668	0.397753	0.943417	0.785345	1.90782	1.474344	0	0	0.778026	6.725123	0	0
LOC107794866	0	0	0	0	0	0	0	0	0	0	0	0	0	0	0	0	0	0	0	0	0	0	0	0	0	0	0	0	0	0
LOC107786743	68.23331	52.78648	67.14397	16.03542	12.11532	27.30994	7.366265	7.350082	14.29203	5.138431	6.410946	7.001157	8.623645	12.52937	3.744328	43.93362	56.65128	68.76796	8.363456	6.667406	11.33694	8.989463	17.36067	13.55737	1.081904	0.626678	4.471477	0	6.071277	0
LOC107804526	0	0	0	0	0	0	0	0	0	0	0	0	0	0	0	0	0	0	0	0	0	0	0	0	0	0	0	0	0	0
LOC107824436	0	0	0	0	0	0	0	0	0	0	0	0	0	0	0	0	0	0	0	0	0	0	0	0	0	0	0	0	0	0
LOC107778273	1.223826	1.829201	0.808523	2.660939	2.902231	0.526305	8.04757	5.855754	1.976249	1.813945	5.663775	0.330855	1.035409	1.441945	2.210739	2.152312	0.898562	1.16789	1.092832	2.360422	0.625044	3.761141	6.001996	1.227977	0.309458	0	0	0	0	0
LOC107792064	0	0	0	0	0	0	0	0	0	0	0	0	0	0	0	0	0	0	0	0	0	0	0	0	0	0	0	0	0	0
LOC107821515	0	0.308982	0.147355	0.153571	0.449991	0	1.563964	2.172558	0.954882	0	0.35659	0	0	0	0.695937	0	0	0	0	1.43397	0.463798	0.990958	0.448567	0.177176	0.428634	0.235866	0.200352	0	1.632199	0
LOC107799862	5.237976	21.74717	10.4725	25.62484	33.01083	9.135487	11.52025	17.10624	6.283655	52.09842	49.46134	19.6675	20.27888	14.47642	35.78103	41.41884	7.02823	10.89098	65.31712	76.31111	59.07708	11.22755	12.78267	10.64531	39.88153	39.11367	35.28794	87.40418	40.06778	89.96443
LOC107798753	1.120423	0.663733	0.97396	4.846846	3.321269	7.829844	0.798785	0.95732	0.815224	1.393006	2.298	1.088549	0.779544	1.680698	2.127447	0.918434	0.97418	1.858593	3.21182	4.265102	1.072937	1.543896	2.001281	1.639492	0.283311	0.038975	0.728337	0	0.94397	0
LOC107791894	0.363748	0.110504	0.316199	0	0	0	0	0.103599	0.113834	0	0.08502	0	0	0.264554	0	0	0.117137	0.451983	0	0	0	0	0.10695	0.25346	0	0	0	0	0	0
LOC107827546	8.753491	9.907823	10.35018	13.13176	12.40972	15.07796	10.38133	11.70136	10.78082	14.77696	13.2329	18.7663	13.14985	18.27778	10.24022	3.822684	3.728182	4.771771	5.39632	5.772599	9.786019	5.109328	3.320928	8.50969	5.057534	5.500575	8.232239	6.731165	7.703454	11.6396
Novel01108	2.793587	2.015592	2.327223	3.901725	3.398931	3.629166	5.271979	4.773834	4.152676	5.107688	3.183156	3.343475	3.239438	3.809583	4.6593	3.643123	3.710905	3.47123	1.889826	2.067785	1.804691	5.346453	3.490849	4.014803	0.441493	0.809807	0.825449	0	0.560388	0
LOC107808851	0	0	0	0	0	0	0	0	0	0	0	0	0	0	0	0	0	0	0	0	0	0	0	0	0	0	0	0	0	0
LOC107769698	0.107043	0.162594	0.031017	1.48696	1.957522	0.537061	2.424105	4.359619	2.81391	5.880104	3.777954	4.220206	3.932342	4.904708	9.155509	0.132948	0.137884	0.079805	5.213758	5.795271	3.221629	4.857101	1.888382	1.678221	26.93167	41.80324	14.08549	5.103397	13.7425	13.23727
LOC107827353	4.491136	4.369057	5.263886	1.005756	1.696786	1.953109	1.185095	1.465953	2.179302	0.639691	0.919987	0.639478	0.730278	0.93588	0.51793	2.857941	3.900058	4.364118	0.819289	0.640313	1.334649	1.264269	2.092017	2.215209	0.255198	0.070214	0.656065	0	0.242943	0
LOC107764320	9.895188	7.720291	9.051858	6.266799	7.872139	6.06496	2.776849	2.812971	2.049018	8.152101	6.056608	7.609698	7.535811	9.059866	8.447639	16.4569	11.31068	13.72037	8.445661	6.680872	8.130554	1.482838	1.468297	1.604529	5.986345	4.658105	4.699987	5.29081	16.11711	11.43616
LOC107771138	0	0	0	0	0	0	0	0	0	0	0	0	0	0	0	0	0	0	0	0	0	0	0	0	0	0	0	0	0	0
LOC107826611	0.117773	0	0.025595	0.080023	0	0.06331	0	0	0	0.028711	0.020646	0	0	0	0.09066	0.043882	0	0.021951	0.059754	0.099628	0.053706	0.319656	0.025971	0.153871	0	0	0	0	0	0
LOC107825332	23.0193	11.16255	18.9908	3.738038	3.766341	5.369971	0.983578	1.781281	2.691247	0.338818	1.2182	0.513703	0.676897	1.32672	0.22289	7.476558	12.42004	11.0095	1.498463	1.138972	0.871449	1.595956	2.988216	2.859934	0.164736	0.302167	0.821342	0	0	2.685505
LOC107812318	0	0	0	0	0	0	0	0	0	0	0	0	0	0	0	0	0	0	0	0	0	0	0	0	0	0	0	0	0	0
LOC107797957	0	0	0	0	0	0	0	0.072069	0	0	0	0	0	0	0	0.081487	0	0	0	0.076926	0	0	0	0	0	0	0	0	0	0
LOC107761793	0.865975	1.130517	1.118987	1.802297	1.698223	1.45945	3.023104	2.039758	1.933658	2.008385	2.642236	1.74002	2.032248	2.834207	4.323953	0.976709	0.474829	0.66307	0.92623	0.732555	0.981893	1.622309	1.403827	1.149742	0.384679	0.586187	0.2213	5.021305	1.014108	1.447149
LOC107815653	0	0	0	0	0	0	0	0	0	0	0	0	0	0	0	0	0	0	0	0	0	0	0	0	0	0	0	0	0	0
LOC107831755	0.21336	0.097225	0	0.096646	0	0	0	0	0.100156	0	0	0.090126	0	0.116382	0	0.079498	0.206123	0.079534	0.216502	0.180487	0.097294	0.089091	0	0	0	0.148437	0	0	0.513594	0
LOC107812937	0.874616	2.214179	1.436094	7.747496	3.396638	6.947911	5.501834	4.525289	3.740693	5.875263	3.032334	6.650097	3.840447	2.385407	4.538279	2.643268	2.534857	2.934284	12.86874	12.82432	11.21161	8.27803	11.65775	11.17292	11.36496	12.10203	9.935249	6.94971	9.357136	6.008756
LOC107832311	5.975492	5.98561	5.05398	3.804076	2.619937	3.299008	3.521877	2.253833	2.527052	2.309746	2.076135	2.228512	2.636829	2.819013	4.033468	3.249445	4.836693	6.140652	1.474909	2.185883	2.062067	2.157963	2.516678	4.782653	1.837663	0.374525	1.336158	0	1.555035	3.328592
Novel02400	0	0	0	0.983628	1.350225	1.009562	0.319961	0.075218	0.192849	2.518076	1.934174	2.627843	1.894663	2.433011	1.084259	0.043735	0	0.021877	0.71464	0.322704	1.284604	0.098025	0.181186	0.368049	3.116421	1.143257	6.589718	0	4.80333	0
LOC107808244	4.835055	2.325683	0.11675	4.015289	1.9015	3.176724	4.280667	2.86889	5.295923	1.964495	3.296167	4.198255	3.887325	6.740031	3.446228	1.501287	1.816527	1.802369	0.272571	1.022531	1.714864	1.794614	3.080161	6.597753	0	0	1.111181	0	0	0
LOC107786447	0.053421	0.097374	0.069657	0	0.118177	0.258456	0.179228	0.022822	0.025077	0.078139	0.262214	0.045132	0.05947	0.02914	0.301567	0.059715	0	0.039828	0.271042	0.158168	0.146164	0.066921	0.070682	0.027918	0.405246	0.706155	0.12628	0	0	0
LOC107795194	0.646664	0.392903	0.65582	0.292922	0.190737	0.347621	0.813577	0.736703	0.101186	0	0.151147	0.819479	0	0	0.221239	0.803158	0.416488	1.124936	1.640474	1.185238	0.393179	0.360031	2.186534	0.675893	0	0.149964	0.382152	0	0	0
LOC107827241	8.452281	9.851223	9.502152	5.153978	3.883397	6.247475	5.146554	5.381673	7.515715	2.813389	3.476264	3.5017	3.347503	3.502216	2.669289	7.297955	7.223428	9.543189	2.639013	2.475017	2.14952	6.312125	5.985883	5.606397	1.027524	0.621962	1.392825	0	0.195636	2.512583
LOC107774219	0	0	0	0	0	0	0	0	0	0	0	0	0	0	0	0	0	0	0	0	0	0	0	0	0	0	0	0	0	0
LOC107759917	2.122283	2.633804	3.611207	2.290854	2.437321	3.009921	3.560101	2.565678	2.776772	2.355701	2.865474	2.746668	3.342765	2.93108	2.827095	7.638444	3.206327	5.706208	2.497198	3.495117	3.088657	3.129938	2.947395	3.940863	1.941047	1.91631	2.028042	6.458437	2.934793	1.396
LOC107832724	0	0	0	0	0	0	0	0	0	0	0	0	0	0	0	0	0	0	0	0	0	0	0	0	0	0	0	0	0	0
LOC107823268	0	0	0	0	0	0	0	0	0	0	0	0	0	0	0	0	0	0	0	0	0	0.04671	0	0	0	0	0	0	0	0
LOC107810176	0.810222	0.798289	0.685273	3.531232	4.476017	2.448463	4.444743	4.958191	2.446486	2.882684	5.143864	3.440991	3.534671	3.057859	4.989538	0.88119	0.655811	0.946897	7.288314	6.131481	6.670397	5.833713	4.075547	3.066948	4.20821	9.811121	2.122287	9.396054	5.482061	10.83184
LOC107798925	0	0	0	0	0	0	0	0	0	0	0	0	0	0	0	0	0	0	0	0	0	0	0	0	0	0	0	0	0	0
LOC107780096	26.42583	26.87933	24.81733	22.10217	21.62956	24.55522	22.63943	23.46597	23.12621	19.00428	22.33503	27.54779	29.62772	26.04931	16.37051	26.19813	27.26193	28.88392	20.39863	17.96338	28.57666	17.85216	18.39772	24.21705	17.95802	17.46556	21.36128	26.99743	23.17282	17.50659
LOC107782165	0.130949	0	0.341495	0.118634	0.347618	0	0.109833	0.223773	0.245882	0.127692	0.091822	0	0.583099	0.714297	0	0.292751	0.632541	0.390513	0	0	0	0.109359	0	0.547473	0	0	0	0	0	0
Novel02603	0.628776	0.610425	0.546581	0.371504	0.628954	0.764186	0.401268	0.455489	0.564656	0.719768	0.642178	0.843003	0.654312	0.715788	0.491033	0.85564	0.382958	0.672596	0.970928	0.740036	0.62332	0.194061	0.349653	0.271451	0.328355	0.304312	0.258491	0	1.184537	0
LOC107803882	12.33424	15.79702	12.80014	10.82962	12.4527	11.15778	11.25673	12.25647	11.37587	11.12671	12.30641	13.45	13.79113	13.27821	10.59608	12.71422	13.07076	12.76055	13.01216	13.88125	13.57813	11.3897	12.02988	13.28929	14.90726	13.60879	12.13769	15.54313	23.2817	47.03535
LOC107779867	0.840154	0.751775	0.624098	0.387488	0.432537	0.394153	0.653424	0.443762	0.64536	0.163851	0.342759	0.141957	0.136039	0.133319	0.266532	0.694384	0.57554	0.728867	0.418515	0.452271	0.250768	0.357196	0.471591	0.718469	0.077252	0.063764	0.16249	0	0.294167	0
LOC107789970	7.255474	6.82946	6.666498	9.52474	8.449278	9.147762	7.556924	8.661853	7.435288	8.064771	8.725268	6.584849	8.662784	9.883954	7.768358	7.647943	6.45252	7.483282	8.837368	9.25415	5.428548	8.685951	7.670927	7.216691	9.25234	9.380586	6.842537	20.61138	13.09137	13.94664
LOC107780173	0.060276	0.329604	0.052397	0.819104	0.586697	1.231275	1.870572	1.390537	2.546535	0.705319	1.352496	2.851675	2.012999	3.353661	0.618655	0	0.058232	0	0.183491	0.203957	0.164918	0.704732	1.169681	1.70101	0	0	0.356206	0	0.870569	0
LOC107812481	1.422355	1.85991	1.72024	0.95243	1.860529	1.595693	0.933645	0.739747	1.045072	2.351828	1.561079	1.462872	2.753713	2.496244	1.777228	2.53464	1.792323	1.844219	2.070832	1.412469	1.748416	2.117464	1.745546	1.551282	1.641916	1.720959	2.119659	0	3.572725	0
LOC107781749	0.185964	0.056494	0.10777	0	0.054851	0.133289	0	0	0	0	0.043466	0.052369	0.069006	0	0	0	0	0.277288	0	0	0	0	0.21871	0.06479	0	0	0	0	0	0
LOC107785630	4.539865	5.230459	5.956843	3.686078	3.638193	4.328354	3.66409	5.379345	4.744741	4.468705	4.985256	5.355092	4.958485	5.420012	4.74751	9.351434	5.544428	9.227993	4.215603	4.094025	4.140433	3.719825	4.38224	5.013654	3.357224	3.873565	3.897765	3.062873	7.010584	7.944529
LOC107768074	0	0	0	0	0	0	0	0	0	0	0	0	0	0	0	0	0	0	0	0	0	0	0	0	0	0	0	0	0	0
LOC107830695	52.5642	85.16584	55.75786	27.58833	27.70789	14.60744	40.20331	40.21969	29.41735	30.89392	44.57453	25.90016	47.95281	47.12794	70.083	179.0866	69.3716	137.4436	34.54007	39.81977	30.18409	26.47555	35.10371	26.34554	24.92466	22.83997	29.18621	21.98294	28.01942	15.20526
LOC107760246	2.269487	2.647498	2.880313	1.809315	1.927864	2.293578	2.702994	2.016676	2.556833	0.840954	1.432229	0.613544	1.465326	1.386503	1.30443	2.908906	2.587168	2.774878	0.875109	1.267088	1.20049	2.577619	2.642426	2.656715	0.573864	0	0.643764	0	0.437045	0
LOC107806002	17.76095	21.28991	21.51024	17.94503	17.42085	20.1956	18.02775	19.80524	19.67057	14.85339	14.39597	17.14124	14.40991	18.25973	12.60606	15.56852	17.79839	17.7302	14.96791	12.68173	17.84557	14.63152	18.42786	20.51451	11.3418	6.869396	14.08956	30.13937	16.5218	11.168
Novel04258	0	0.052401	0	0.312536	0.203508	0.185449	0.09645	0.098254	0.053981	1.317571	1.915054	1.287239	0.608061	1.191801	0.295067	0.06427	0.055547	0.214332	0.670956	0.535024	0.419506	0	0.076074	0.15024	1.308486	1.000036	1.393115	0	2.352893	1.777563
Novel03722	2.23224	1.230109	1.308668	1.222784	1.607747	0.94881	0.566037	0.931465	0.43864	0.860562	0.582418	1.140286	1.213585	1.189315	0.372971	0.696335	0.902734	0.890169	1.000868	0.526973	1.230973	0.216768	0.549484	0.434072	0.328166	0.28893	0.552208	14.84992	1.499553	0
LOC107803416	10.03361	9.88185	11.11352	10.21397	8.353332	9.395441	8.732338	9.679181	8.710963	4.944621	7.640784	8.020938	5.224469	6.591246	4.207876	9.085028	10.94406	9.813106	5.692834	4.243872	7.084507	8.46943	11.89558	11.8403	0.682018	1.200949	2.486548	3.857769	3.376182	6.670895
LOC107797617	17.51876	17.01183	16.37947	17.35028	14.29129	20.73252	12.43601	14.0259	17.06882	15.663	14.01694	18.08101	16.25996	17.52344	13.4969	14.76497	16.58354	18.32485	14.41848	15.00631	16.58108	11.49792	17.63243	17.15754	7.141251	8.718898	12.93458	18.93459	10.40991	5.456983
LOC107787215	0	0	0	0	0	0	0	0	0	0	0	0	0	0	0	0	0	0	0	0	0	0	0	0	0	0	0	0	0	0
LOC107821772	20.32548	14.45493	18.71124	26.86612	33.72985	31.3098	25.48794	24.48412	27.40366	20.27479	17.56997	29.21975	41.39556	33.2248	17.78359	13.40844	17.12527	15.84908	24.27651	26.43923	29.23404	20.09094	21.98435	28.69706	27.63544	22.25423	22.84162	47.65768	51.65413	32.96407
LOC107828802	23.69011	24.95539	25.47419	34.07448	34.43851	27.04095	28.83719	32.3995	29.96801	40.05148	29.9331	42.04253	57.02552	48.58477	35.13027	26.93939	34.99873	27.86923	33.71716	29.73498	46.50855	27.81355	28.56273	26.28836	38.02285	41.63192	41.36335	99.0104	84.42857	118.8957
LOC107778044	28.01708	21.66763	35.89299	29.94854	31.81619	25.10902	37.83818	39.58655	43.89203	40.09965	35.28549	40.91613	31.37605	36.61416	42.37948	38.90825	24.9567	37.64211	39.08565	37.20307	53.90065	37.5347	34.86485	45.83496	46.99935	52.13416	48.90139	54.07064	53.58968	72.72185
LOC107760645	3.226179	4.243967	3.545247	2.757316	1.992929	2.323266	3.267545	3.354651	2.857433	2.34461	2.667687	2.159882	2.236172	3.121158	2.436589	3.402103	3.998863	3.630572	1.38978	2.18845	1.693228	2.668849	3.060474	2.035926	1.385277	1.439866	1.00723	0	1.025697	3.763765
LOC107760266	0	0	0	0	0	0	0	0	0	0	0	0	0	0	0	1.244689	1.742714	1.593932	4.338887	4.182291	7.250989	5.300577	3.889489	2.723399	4.89922	5.949601	2.684815	0	22.83726	0
LOC107807813	1.782673	2.126133	2.047055	1.734641	2.005863	2.721086	2.196658	1.617186	2.066236	1.523722	1.774707	1.227153	1.298497	1.368569	1.287557	2.755303	2.04114	2.658116	1.719603	1.992074	1.043741	2.040148	1.785976	3.450461	0.69563	0.52059	1.27459	0	0.211912	0
LOC107825384	0	0	0	0	0	0	0	0	0	0.070744	0	0	0	0	0	0	0	0	0	0	0.066165	0.121174	0	0	0.091723	0	0	0	0	0
LOC107822577	14.45345	14.80634	15.31552	8.602517	6.890395	10.96178	7.917367	7.084165	7.494803	5.954417	6.520821	5.916026	8.231983	8.189585	6.698582	12.08576	13.93619	14.6808	4.803519	4.075542	3.831916	8.023578	9.067532	11.21295	1.168658	1.052316	2.582287	0	2.427351	3.463871
LOC107767866	0	0	0.031038	0	0	0	0	0	0.067044	0.034817	0.025037	0.030165	0.119243	0.077906	0	0	0	0.02662	0	0	0	0	0	0.037319	0	0	0.042201	0	0.171898	0
LOC107814763	26.17477	24.84674	28.98008	23.76778	18.82847	25.47645	22.48628	20.47921	28.88725	16.74072	18.5657	17.5935	19.51514	17.01453	15.44896	27.72659	29.1414	28.82092	12.23739	12.2216	11.79809	20.95053	26.82033	36.3929	4.547403	2.481289	7.251852	0	7.857727	3.737703
LOC107759779	5.741537	5.189812	6.913757	4.476737	4.122693	7.538978	4.776202	4.744882	5.434592	2.753471	3.365974	4.731333	3.457729	2.618447	2.704964	5.506068	7.547296	6.280493	3.772648	3.901481	4.120429	4.04816	5.147439	6.197809	1.190008	0.851279	3.78238	0	1.132861	0

LOC107764380	25.8183	26.74114	28.37713	18.05374	16.51322	23.8812	13.36695	12.74674	20.18223	7.220011	9.727417	6.278511	4.657437	6.185818	5.565395	22.31656	24.30445	26.80034	13.37861	15.87977	10.14166	16.80328	23.01623	28.97033	2.505583	0.804275	4.879821	3.93683	1.192629	5.105706
LOC107774413	27.58837	24.87304	29.83934	16.39369	13.98722	19.50054	16.35053	17.127	24.15056	8.967381	10.87598	11.06297	7.73691	9.755122	7.829294	23.33797	27.14398	26.57134	9.503601	9.392504	10.93791	19.11133	25.38142	28.61389	3.214754	2.594527	7.513187	0	1.22415	2.620323
LOC107809764	4.691334	10.26137	4.326308	17.73959	19.05943	5.350769	31.03378	28.19824	16.20065	29.43683	43.93716	16.12919	26.97513	20.96161	52.25383	22.83032	7.606252	11.2227	19.16595	20.25684	12.6497	31.41098	19.79075	12.5356	26.56178	47.45317	11.76457	2.917363	13.55145	7.567102
LOC107766020	0.037044	0.135043	0.080503	0.100679	0.032779	0.05974	0.09321	0.015826	0.017389	0	0.025975	0.046943	0	0.080826	0.01901	0.096617	0.125256	0.096662	0.018795	0.015668	0.118246	0.108277	0.163375	0.174231	0	0	0.021891	0	0	0
LOC107823588	0	0	0	0	0	0	0	0	0	0	0	0	0	0	0	0	0	0	0	0	0	0	0	0	0	0	0	0	0	0
LOC107775396	0	0	0	0	0	0	0	0	0	0	0	0	0	0	0	0	0	0	0	0	0	0	0	0	0	0	0	0	0	0
LOC107819734	6.597148	7.22585	5.510206	20.07229	24.68668	18.45822	18.88326	19.23637	15.33499	29.68505	26.59906	22.042	24.79718	25.51525	26.61544	8.114695	7.19887	7.259163	29.96317	30.79489	24.04872	18.93459	14.47769	14.01913	50.01968	52.22885	32.26954	28.42104	38.36195	28.25893
LOC107779362	20.9519	15.64112	15.07933	10.07687	7.443114	12.62111	5.297822	6.160351	8.70712	6.579904	9.225391	7.991369	6.757086	7.025319	4.933376	11.54931	14.07961	14.17334	5.440198	8.60129	6.744173	4.837539	6.44116	5.893387	1.051792	1.028931	2.913349	0	2.670086	0
LOC107818208	11.12925	12.31468	10.02354	16.30023	18.55068	14.97822	22.59163	20.1316	19.08739	17.23159	20.98963	15.18543	23.94391	19.69702	26.44547	16.24122	12.70901	13.70689	26.22701	22.70975	19.6571	26.80224	19.46815	18.13406	20.50245	31.97695	10.81213	15.31802	27.0264	39.18028
LOC107798237	0.066388	0	0	0	0	0	0	0	0	0	0.046551	0.28043	0	0.144851	0	0.148416	0.128272	0.098989	0	0	0	0.110884	0.175675	0.069388	0	0	0	0	0	0
LOC107778502	0	0	0	0	0	0	0	0	0	0	0	0	0	0	0	0	0	0	0	0	0	0	0	0	0	0	0	0	0	0
LOC107758951	2.075781	2.948288	2.038774	3.297072	2.480855	3.275119	3.165539	3.201699	3.492716	1.971573	2.230566	1.434827	2.010686	2.558675	1.549472	2.390624	2.187686	2.431915	1.696029	1.984015	1.86856	3.782266	4.018635	2.930376	1.056584	0.562655	0.669111	0	1.297863	0
LOC107828444	3.684092	4.388145	4.153741	3.304555	2.872609	4.392229	3.028818	3.303619	4.109451	2.845509	3.069252	3.482216	4.304211	2.666178	2.171411	4.675317	4.581075	3.752844	2.627957	2.746212	3.925491	2.741597	2.799171	3.850612	2.167497	1.877895	2.328042	5.217122	1.053655	0
LOC107779238	0	0	0	0	0	0	0	0	0	0	0	0	0	0	0	0	0	0	0	0	0	0	0	0	0	0	0	0	0	0
LOC107816154	0.56439	0.395671	0.245306	0.865292	0.49941	0.630127	0.382344	0.315304	0.244557	0.338678	0.38053	0.165051	0.362473	0.402588	0.245078	0.194116	0.356509	0.064735	0.264325	0.275443	0.514733	0.199412	0.670155	0.431081	0.027445	0.030204	0.102625	1.55238	0.104507	0
LOC107776155	4.222334	6.76024	3.967984	10.77782	9.214274	8.312238	4.187529	4.509596	3.589129	26.26175	28.28672	22.63209	12.35433	7.718763	7.408263	4.294531	6.339188	4.04126	11.23242	12.38064	10.38165	3.835913	5.687225	5.009558	11.14553	20.36426	14.16211	6.120741	7.004856	0
LOC107776576	0.836165	0.489897	0.571105	0.595197	0.581345	0.44949	0.250474	0.051032	0.560735	0.407683	0.08376	0.201833	0.332439	0.130316	0.122602	0.712127	0.750105	0.846038	0.121212	0.252621	0.108942	0.249394	0.316094	0.43698	0	0	0	0	0	0
LOC107787729	3.069011	2.985287	2.873044	3.181384	2.167308	3.172656	1.237554	1.563262	1.773122	1.697767	2.007147	2.044312	2.529499	1.545296	1.272095	1.847217	2.736852	2.64009	2.155999	2.221725	1.910849	2.316568	1.535742	2.652546	1.790845	1.683486	1.813662	6.331088	1.420703	5.473892
LOC107823674	0	0	0	0	0	0	0	0	0	0	0	0	0	0	0	0	0	0	0	0	0	0	0	0	0	0	0	0	0	0
LOC107784438	5.923902	5.605563	6.406104	6.188459	5.367261	5.942292	9.485569	8.112998	10.61764	4.44988	5.90283	4.382085	6.973205	5.937013	5.87649	4.667962	5.44916	4.310862	2.962441	2.997137	3.541481	8.947576	7.725404	9.56893	1.469254	0.907089	2.244524	6.080996	1.773957	1.752554
LOC107781690	0	0	0	0	0	0	0	0	0	0	0.059144	0	0	0	0	0	0	0	0.08559	0	0	0	0	0	0	0	0	0	0	0
LOC107779489	0.734786	1.243667	0.912477	2.662716	2.600749	2.70852	1.936933	0.807199	1.379699	2.149528	2.870581	0.975492	2.570776	1.603231	1.616067	2.112031	1.318321	2.191257	2.236831	4.084659	1.436009	2.191568	1.94439	1.645711	1.725273	1.606631	2.605375	0	3.537523	12.98084
LOC107801530	3.846311	4.652672	3.708264	4.656643	3.805697	4.849526	5.103051	4.511321	3.742398	2.38677	2.868665	3.101767	5.410596	3.471349	3.158198	3.126846	4.627934	3.806071	2.696598	2.514234	2.61498	5.110246	4.842309	4.166351	0.574707	0.632493	0.826551	0	1.178387	2.162029
LOC107816703	1.202876	0.996612	1.045634	0.495338	0.387049	0.117567	0.825467	0.373734	0.513324	0.319897	0.153356	0.277152	0.365198	0.357894	0.336709	0.977874	1.373366	1.79359	0.221926	1.757586	0.199462	1.461168	0.675192	0.114295	0.138255	0.760779	0.129246	0	0	0
LOC107767104	0	0	0	0	0	0	0	0	0	0	0	0	0	0	0	0	0	0	0	0	0	0	0	0	0	0	0	0	0	0
LOC107790055	1.213005	1.608007	1.126333	2.347695	2.268664	1.155932	4.162089	3.698151	1.682351	1.639837	3.730865	0.954909	1.626539	1.142875	4.951685	3.471924	0.958798	1.130433	5.902599	7.392719	4.500552	7.022016	6.079265	2.333964	2.858086	7.058106	1.042672	5.914569	1.327236	5.113768
LOC107812685	0.060555	0	0	0	0.026791	0	0.025395	0	0	0	0	0	0	0	0	0	0.022563	0.02925	0.045146	0.030723	0	0	0	0	0	0	0	0	0	0
LOC107784140	6.985602	5.093212	4.964483	11.30266	10.38896	9.565848	9.271809	7.685995	12.49546	11.97451	15.96677	16.8973	13.39736	14.94776	10.91827	5.223945	9.424471	5.33598	5.521573	6.075215	6.505113	8.842902	10.83173	15.62745	0.805719	0.409261	1.390555	0	3.068107	1.515545
LOC107810649	9.608122	12.42875	12.43198	2.266372	1.782656	2.094543	2.599595	2.704954	4.44791	1.770205	1.785208	1.608479	0.837927	1.135146	0.999785	9.65113	5.881612	9.457487	2.021819	1.554397	1.756592	2.311058	3.378283	2.684145	0.895675	1.139754	1.517626	1.583218	2.238238	0
LOC107776098	0.044826	0.020427	0.077933	0.020305	0.059498	0.072291	0.037598	0.01915	0.08417	0	0.047149	0.056806	0	0.122259	0.023004	0.050107	0	0.05013	0.022743	0.01896	0	0.037436	0.07908	0	0	0	0	0	0	0
LOC107787134	0	0	0	0	0	0	0	0	0	0	0	0	0	0	0	0	0	0	0	0	0	0	0	0	0	0	0	0	0	0
LOC107818132	0.049137	0.089564	0.085427	0.178062	0	0.052828	0.041213	0	0	0	0	0	0.0547	0	0	0	0	0.073267	0.049861	0.083133	0.134441	0	0.130026	0	0	0	0	0	0	0
LOC107769701	4.874335	3.032084	3.22831	3.014028	2.875422	3.410488	2.206392	2.247651	2.978188	3.093282	2.766861	4.771621	4.823269	2.701034	2.461737	3.747673	2.690867	5.249146	2.904875	2.356201	3.034214	2.778404	2.320358	5.013786	1.565115	0.645932	3.017704	0	3.724887	0
LOC107786127	0	0	0	0	0	0	0	0	0	0	0	0	0	0	0	0	0	0	0	0	0	0	0	0	0	0	0	0	0	0
LOC107774227	3.854788	3.745652	3.843662	3.954451	3.43605	5.393773	2.377336	2.639752	2.528009	2.459871	3.179976	2.610095	1.766966	2.288224	1.658217	3.632984	2.573975	3.78257	3.50891	4.651557	1.964617	2.674805	3.300172	3.703146	1.325912	0.749334	1.87602	0	1.228124	0
107792532	0	0	0	0	0	0	0	0	0	0	0	0	0	0	0	0	0	0	0	0	0	0	0	0	0	0	0	0	0	0
LOC107802265	2.427774	3.015424	2.390556	3.075324	2.452432	2.794944	2.288561	2.680141	2.400326	3.184449	3.223932	2.522974	2.941803	2.976725	2.557955	2.561671	2.241654	2.931253	2.572543	2.653492	1.665527	2.296634	2.918446	2.357862	1.711283	1.345249	1.777525	0	1.551527	1.328433
LOC107811913	0	0	0	0	0	0	0.078733	0	0	0	0	0	0	0.102408	0	0	0	0	0	0	0	0	0	0	0	0.118681	0	0	0.903852	0
LOC107783478	3.22085	4.900639	3.891271	2.645917	2.536035	3.433462	2.586997	3.381673	3.357015	2.235778	2.392439	1.983148	2.035832	2.471564	1.624881	2.929035	2.689699	3.052475	2.04961	2.955518	1.692779	2.894972	3.033613	3.908484	1.311365	1.367261	1.709828	3.904006	1.314095	5.063136
LOC107760841	21.59628	18.92645	21.57392	12.687	11.77074	14.81603	13.11038	14.20048	22.16234	6.874538	8.432843	7.788546	6.889231	8.317512	6.613735	18.58963	22.49718	20.26293	8.675131	8.527319	10.82271	19.02132	22.4259	27.14026	4.678427	2.663189	8.332408	4.562599	4.146602	11.83454
LOC107783130	1.114463	5.755608	1.589791	9.268042	5.234182	4.485509	11.28888	9.815268	4.560825	11.31326	22.52214	6.542422	2.70395	1.028774	9.766756	10.71131	2.429398	3.877438	23.37152	32.94822	17.17471	21.38263	24.67658	18.2491	10.58571	17.21668	21.58191	20.43586	4.539972	8.834478
LOC107828897	0.059053	0.02691	0.102667	0.641985	0.992821	1.047567	0.866776	0.756844	1.469194	0.662214	0.393374	1.371958	1.15042	1.288472	0.545491	0.044006	0.05705	0.022013	2.576673	1.548591	1.454141	0.961671	0.885503	1.357881	2.239818	1.602267	1.500605	4.223104	2.274406	1.825659
LOC107800999	0	0	0	0	0	0	0	0	0	0	0	0	0	0	0	0	0	0	0	0	0	0	0	0	0	0	0	0	0	0
LOC107767359	9.221789	10.56729	8.59348	8.165106	9.052211	6.213119	4.630514	4.02372	3.382322	8.249036	9.587403	6.772823	7.021824	6.183865	10.60079	17.41845	16.04812	13.22795	7.498584	7.894611	6.347069	4.980211	3.307919	2.557445	5.378752	6.997415	2.150817	5.275906	4.854069	5.32184
LOC107762779	0	0	0	0	0.054916	0.066724	0.104107	0	0	0	0	0	0.069088	0	0.063698	0	0	0	0.0525	0	0	0	0	0	0	0.086354	0	0	0	0
LOC107773613	2.135386	1.659944	1.528673	0.625884	0.555744	1.080377	0.158033	0.107325	0.294823	0.551189	0.044039	0	0.279664	0.411106	0.515695	0.842449	2.48769	1.826142	0.191192	0.159387	0.343678	0.472054	0.055399	0.196933	0.873457	0.436946	0.668079	0	0.907104	0
LOC107785458	0.981284	0.704615	0.878858	0.457966	0.552558	0.59144	0.723282	0.7114	0.656058	0.405947	0.677652	0.2889	0.595843	0.308184	0.381501	0.886369	0.675093	0.886775	0.482783	0.50309	0.325436	1.092661	0.747527	0.916858	0.281964	0.186189	0.1933	0	0.07158	0
LOC107809308	0	0	0	0	0	0	0	0	0	0	0	0	0	0	0	0	0	0	0	0	0	0	0	0	0	0	0	0	0	0
LOC107832360	7.075132	10.13275	9.561332	8.618099	9.680167	10.03564	12.06791	10.41397	8.93102	11.47925	14.79988	7.735289	11.38399	8.302383	15.80492	8.01936	6.20335	7.93438	6.395056	6.186254	3.253432	8.540202	7.49937	5.344232	5.337003	5.956355	5.200047	0	7.155926	0
LOC107818836	0	0	0	0	0	0	0	0	0	0	0	0	0	0	0	0	0	0	0	0	0	0	0	0	0	0	0	0	0	0
LOC107764476	0.23755	0.541244	0.464619	0.538021	0.735699	0.574639	0.199243	0.558165	0.390289	0.463282	0.49971	0.301033	0.859443	0.647888	0.365722	0.398301	0.458986	0.487035	0.903935	0.502377	0.812436	0.595152	0.314302	0.744861	0.901006	0.826334	0.421147	0	0.857737	0
LOC107781619	2.465522	10.0428	2.930533	9.435981	5.253784	4.435928	2.827591	3.955266	0.661354	7.801368	22.82755	2.848104	1.456347	0.768504	11.82642	14.54849	2.527732	2.926037	13.22402	31.24236	3.854739	3.151564	5.104013	1.262186	1.844855	4.200727	2.021993	7.196722	1.453457	6.222324
LOC107782386	1.339678	1.643009	1.452098	1.648208	0.892735	1.031339	1.969848	1.822973	1.18011	1.741807	2.77176	1.439199	2.080523	1.660001	2.020075	2.16921	1.613808	1.664645	0.923058	1.511034	1.206725	1.17405	1.590168	1.694107	2.509275	1.679959	3.264524	7.096736	11.78461	1.022646
LOC107776057	0.042021	0.382974	0.182642	0.609109	0.743667	0.180713	1.180715	1.202794	0.37479	0.942453	1.959454	0.40826	0.42101	0.458434	1.509539	0.673261	0.182683	0.407274	0.938091	0.995324	1.015594	1.298449	1.890351	0.680773	0.531279	1.578685	0.273163	0	0.606919	2.598251
LOC107799324	291.3322	255.0099	294.6422	162.3982	155.0159	211.4211	122.625	120.4367	145.7151	116.8914	144.6163	134.7962	95.41617	104.9141	105.6453	236.2836	275.6844	278.8903	133.3336	135.702	130.5999	126.9951	165.4217	192.4812	52.48182	44.17128	84.29856	83.90012	47.67732	36.27025
LOC107827873	0.64058	0.785898	0.706762	0.357128	0.741236	0.768168	1.074565	0.421022	0.48575	1.129169	1.813977	1.477838	3.949493	3.46733	2.731046	0.789482	0.428438	0.47758	0.175004	0.395994	0.29211	1.687198	0.63023	0.38627	0.155748	0.205689	1.455989	0	0.711688	0
LOC107761667	12.08186	6.15529	8.023656	6.798483	3.98416	1.29088	8.748868	9.361296	11.20208	0.219528	0.42096	0.443787	0.668308	1.88296	0.770217	4.529671	8.917192	4.979325	0.989925	0.571328	4.243289	6.705697	7.016412	6.431633	0.379507	0.208833	0.266083	0	1.80641	0
LOC107768269	0	0	0	0	0	0	0	0	0	0	0	0	0	0	0	0	0	0	0	0	0	0	0	0	0	0	0	0	0	0
LOC107798033	17.78251	14.74577	15.53682	16.41143	14.02246	17.33009	16.91883	15.19386	17.85911	15.42333	16.87994	16.58168	17.57376	15.30872	14.49565	12.21486	14.98815	13.48308	11.32382	12.12632	10.28791	13.36052	17.28589	18.23857	8.373582	7.911046	11.65255	10.81378	14.41415	11.21958
LOC107812242	10.59683	7.674752	9.071498	10.33024	8.735015	13.60215	9.293554	10.32801	14.14768	7.386503	9.408203	10.28005	9.015605	8.000124	7.609271	9.398056	10.78239	11.62528	7.563848	8.759682	8.157855	10.2966	13.54086	16.67687	1.528245	2.074351	5.476536	2.881457	2.521748	0
NitaMr002	1.487123	0.401579	1.747571	0.124746	0.194949	1.89492	7.853457	28.16581	15.22866	0.402815	2.471754	0.581649	0.8584	6.249158	17.46813	0.246268	1.250448	0.390103	0.307395	0.186371	0.401861	16.46714	17.19837	0.662034	5.431617	1.302844	8.039664	187.0977	28.50557	22.13636
LOC107806115	1.582305	0.895082	0.616587	2.916408	4.538345	3.812971	4.667898	2.843788	5.327446	4.575637	4.17024	5.347102	15.30634	13.75021	2.688058	0.853857	1.317793	0.406785	0.996588	1.24621	1.691898	3.918707	2.550758	3.250622	1.931532	0.683275	1.999134	0	2.889503	0
LOC107773171	1.380552	2.442394	1.835423	6.179979	6.53917	4.889334	1.634722	1.179581	1.372365	8.077274	3.815232	7.409617	2.079267	2.746444	2.167116	2.42068	1.019911	1.755796	11.1247	14.6325	8.665478	1.831132	2.220581	2.121989	1.232073	4.067867	3.935279	0	0.390969	5.021271
LOC107789698	0.09718	0.265701	0.236533	0.493022	0.498747	0.31344	0.521655	0.614443	0.346698	0.473812	0.831337	0.5747	0.324545	0.360461	0.598454	0.420026	0.225321	0.289806	0.414166	0.34527	0.691309	0.211009	0.41145	0.406288	0.393166	0.405655	0.528349	1.389942	1.497142	2.403502
LOC107763411	43.84821	33.03063	39.49609	30.79678	33.53284	38.82298	31.06385	31.92727	37.37905	26.95724	22.30621	31.56857	30.33292	34.92118	20.36407	31.1972	37.12183	36.14221	20.66997	20.47644	32.20939	27.56074	37.6853	43.68725	16.89045	8.650209	24.70093	42.56697	27.70102	16.35717
LOC107776535	0	0	0	0	0	0	0	0	0	0	0	0	0	0	0	0	0	0	0	0	0	0	0	0	0	0	0	0	0	0
LOC107832802	0.896914	0.490456	1.039557	1.083411	0.899468	1.285723	0.802432	0.817437	0.561375	1.282753	1.635183	2.172181	3.061934	1.500349	1.718393	1.292204	0.750962	1.515691	1.213501	0.859891	1.908668	0.848908	0.896622	1.124945	1.20957	0.998392	0.70672	0	1.151483	0
LOC107776502	7.920987	10.36714	12.11442	0.728392	0.843189	1.536727	0.374643	0.381649	0.419356	1.335723	0.354968	0.553463	1.889519	3.118704	0.641833	8.277135	8.486617	10.3012	1.208672	0.831279	0.434531	0.571976	1.91744	1.089344	0.075297	0.207171	1.337424	2.129559	0	0
LOC107827118	0.693709	0.63223	0.536941	0.792038	0.967007	0.643654	0.605755	0.600843	0.570989	0.982248	0.852917	0.529866	1.142501	0.798271	0.624222	0.743562	0.807889	0.871429	0.896785	1.053078	0.364004	0.809481	0.746011	0.715132	0.973175	0.99169	0.83114	0.679589	0.960753	0.587576
LOC107788738	0.484998	0.357822	0.321218	0.355691	0.408722	0.422112	0.348676	0.374929	0.368606	0.33781	0.194332	0.156091	0.257098	0.226761	0.142225	0.430263	0.446237	0.464897	0.328095	0.410275	0.021063	0.327885	0.509286	0.386224	0.467188	0.192811	0.191076	0	0.111188	0
LOC107810751	0	0	0.114547	0	0	0	0	0	0	0	0	0	0	0	0.098197	0	0.098242	0	0	0	0	0	0	0	0	0	0	0	0	0
LOC107773230	0	0	0	0.095288	0	0.226163	0	0	0	0.205128	0	0.355437	0	0.344239	0	0	0	0	0.213459	0	0.095926	0.087839	0	0	0	0	0	0	0	0
LOC107788743	0.082229	0	0.10722	1.37816	1.709895	0.707248	3.069114	3.969608	2.547601	3.528073	3.401875	3.265052	2.425764	2.691228	5.317037	0.857878	0.11916	0.275873	2.544924	2.712837	2.212325	5.116022	3.263907	3.265931	3.690659	6.693301	2.96423	5.880524	5.146425	17.79516
LOC107790705	127.0651	110.3331	139.9744	37.003	36.66263	46.25394	39.29303	45.15698	65.03444	14.17527	18.94212	17.99908	12.44818	16.75788	11.59791	77.54063	103.4808	92.23136	17.55787	13.24312	28.17965	48.16723	69.6151	92.14772	1.190543	1.637812	7.651633	0	1.700055	10.91703
LOC107828243	0.468933	1.453072	0.61145	0.021241	0	0	0	0.080134	0.066038	0	0	0	0	0.025579	0.04813	1.397796	0.611588	0.716699	0.071376	0.099171	0	0	0.124089	0.024506	0	0	0.027712	0	0	0
LOC107804007	21.85927	20.65169	15.85944	11.27541	10.88419	9.390122	9.197679	9.784258	14.23588	4.54227	6.192337	4.52972	7.832267	4.393637	3.511034	20.89981	25.45398	21.34348	11.71841	10.85679	12.36874	12.27822	15.36514	12.75493	2.913976	3.848364	3.096867	1.735013	2.686439	6.000407
LOC107780845	0.613027	0.496621	0.503286	0.277686	0.180816	0.32954	0.228521	0.261894	0.44764	0.03321	0.047762	0.028772	0.379128	0.445855	0.06991	0.203035	0.197412	0.330083	0.034559	0	0.031061	0.853258	0.570771	0.320368	0	0	0	0	0	0
LOC107779245	23.56037	23.03351	20.50666	48.65132	61.13396	44.52185	37.83343	36.33269	36.79063	81.9047	45.6633	74.00701	83.76818	73.50331	38.5242	19.08004	24.96776	20.6571	49.797	41.25909	62.98237	24.82073	22.82288	34.92772	86.77882	58.24755	107.9761	36.53482	70.89252	35.30446
LOC107832587	1.134068	0.936667	1.29389	1.38058	1.630684	1.485975	1.991557	1.514029	1.829975	1.589673	1.76437	2.544932	2.209305	2.165122	1.855087	1.056386	1.643407	1.400352	1.474429	1.259138	2.650366	1.479828	1.281664	1.629825	1.254581	1.430038	1.675471	0	2.729903	4.38257
LOC107770287	359.8237	299.0411	379.6881	149.4366	129.2895	206.4253	121.6833	130.3873	188.8871	71.51884	90.97608	100.3916	56.07211	80.70301	64.07716	212.7152	301.622	278.8402	76.40508	73.84946	101.671	127.394	199.2986	247.8637	19.31532	11.82744	49.4862	12.32202	10.50728	35.51228
LOC107772277	0	0	0	0	0	0	0	0	0	0	0	0	0	0	0	0	0	0	0	0	0	0	0	0	0	0	0	0	0	0
LOC107772604	2.84478	3.299757	5.187917	2.775475	4.013515	3.336531	6.173669	6.646063	5.248225	3.685761	5.258917	3.731074	4.717055	2.799675	5.921281	5.677864	2.248606	3.96001	4.622735	3.988376	6.749296	5.914455	4.59743	5.780385	3.395455	4.650315	2.092621	4.268014	4.118313	7.380295
LOC107783239	91.29044	93.46538	108.662	56.37322	55.34008	75.87658	53.18225	59.35885	66.70246	29.49402	32.80731	32.73974	27.97121	33.34101	27.72944	65.44708	105.3917	83.62271	38.36489	31.78303	56.89445	45.32243	71.83954	82.65609	14.24064	10.85019	21.03964	16.89877	12.13473	9.74051
Novel00096	0.375115	0.528347	0.533585	0.679671	0.754379	1.099895	0.600649	0.844978	0.736366	0.764823	0.50215	1.210012	1.518486	1.302104	0.45501	0.279536	0.197669	0.406785	0.657471	0.692339	0.404314	0.28479	0.391036	0.605927	0.301802	0.237248	0.403051	0	0.164176	0
LOC107759387	0	0	0	0	0	0	0	0	0	0	0	0	0	0	0	0	0	0	0	0	0	0	0	0	0	0	0	0	0	0
LOC107824420	0	0	0	0	0	0	0	0	0	0	0	0	0	0	0	0	0	0	0	0	0	0	0	0	0	0	0	0	0	0
LOC107758946	0	0	0	0	0	0	0	0	0	0	0	0	0	0	0	0	0	0	0	0	0	0	0	0	0	0	0	0	0	0
LOC107815770	3.974049	2.614272	3.610401	1.028654	0.951835	2.280856	1.077653	1.608412	1.402636	0.874105	0.859029	0.479627	1.496831	1.206114	1.042715	1.714533	2.597999	2.561839	0.636725	0.682465	0.517771	0.873377	1.607728	1.342918	0.113332	0.166304	0.176579	0	0.143853	0
Novel05381	0.038728	0.08824	0.016833	0.280686	0.479768	0.249825	0.406037	0.281268	0.218158	0.321001	0.35303	0.310827	0.582021	0.802759	0.655866	0.476194	0.243195	0.317608	1.788087	1.163027	1.04196	0.857086	0.631974	0.708376	2.497169	2.397986	0.823923	0	3.635792	2.394617
800473	0.188416	0.171718	0.491359	0	0.166723	0.202571	0	0.321976	0	0.36746	0	0	0	0.205553	0.193385	0.982857	1.456209	1.123779	0.573576	0.159387	0	0	0.332391	0.196933	0	0.262168	1.558851	0	0	0
LOC107771809	1.934022	1.273005	0.934002	1.703456	2.139187	3.292241	0.901193	0.642631	0.806998	4.714789	3.541025	5.310225	2.810835	2.696013	2.371005	2.32199	2.387436	2.323054	2.834739	4.499136	3.135752	1.031903	1.326839	1.403777	3.328178	3.438564	6.095622	7.683884	4.138253	0
Novel01470	1.923798	2.072087	2.470467	2.693516	2.553454	2.115325	4.290634	3.511637	2.914463	1.875946	2.605961	2.105473	2.774343	2.575761	2.9618	2.020096	2.027509	2.444783	3.460606	3.513705	3.110313	4.089562	3.393833	3.061828	3.482561	3.528541	2.635511	3.126789	3.788937	13.51719
LOC107816263	2.103501	1.443728	2.121996	1.552764	1.654503	1.6752	1.655389	1.996987	1.609144	1.316806	1.147203	2.150066	1.561097	1.983174	1.252739	0.774089	1.154065	1.045499	1.264881	1.142342	1.397373	0.759063	1.236951	1.058575	0.820822	1.73444	1.227738	1.857161	2.12542	1.605711
LOC107768566	0.214232	0.317275	0.30262	0.072781	0.094783	0.547024	0	0.045761	0.100565	0.104452	0.056332	0.135742	0.059622	0.029215	0	0.319292	0.310449	0.339403	0.326082	0.249185	0.17096	0.111819	0.141725	0.083968	0	0	0	0	0.773542	0
LOC107811595	0	0	0	0	0	0	0	0	0	0	0	0	0	0	0	0	0	0	0	0	0	0	0	0	0	0	0	0	0	0
LOC107764523	1.879778	0.740837	1.98736	0.184106	0.269733	0.655457	0.127837	0.217045	0.143093	0	0.320619	0.429213	0.282783	0.221702	0.208579	1.476532	1.227043	1.893857	0.721745	0.558706	0.648688	0.466713	0.448132	0.477911	0.963491	0.494838	1.200944	0	0.244592	0
Novel04605	2.936518	2.435502	1.536891	1.31636	1.375636	1.125201	1.3295	1.172043	1.688502	1.4466	1.902341	0.867011	2.556359	2.383299	1.147183	1.908134	1.590248	1.909008	0.64957	0.515729	0.324344	0.992825	1.236844	1.486833	0.167005	0.084829	0.540425	1.453305	0.440266	0
LOC107823685	62.47833	75.82618	74.90556	57.90013	47.52402	59.95876	50.99647	52.60691	56.59062	33.59694	33.37193	34.00811	25.01218	33.16548	35.18325	62.05252	71.97207	64.81636	43.04587	48.53668	45.79536	62.56605	65.12722	66.32496	23.37056	30.48526	30.31418	27.48821	20.18744	10.80293
LOC107809927	2.662926	3.688932	3.3102	2.822597	2.497712	2.920231	3.707628	3.253645	2.675078	1.84365	3.585112	1.552303	1.867576	1.830227	3.443775	3.988676	2.109541	3.633144	2.675132	3.198767	1.335749	4.42552	2.983074	2.170966	1.649707	2.371371	1.479261	0	1.41023	0
LOC107817841	0	0	0	0	0	0	0	0	0	0	0	0	0	0	0	0	0	0	0	0	0	0	0	0	0	0	0	0	0	0
107792349	0	0	0	0	0	0	0	0	0.67365	0	0	0	0	0	0	0	0	0	0	0	0	0.599229	0	1.499926	0	0	0	0	0	0
LOC107813215	0	0	0	0	0	0	0	0	0	0	0	0	0	0	0	0	0	0	0	0	0	0	0	0	0	0	0	0	0	0
LOC107796464	0.769752	0.801753	0.987761	2.258105	2.335292	2.049238	3.197375	3.194527	2.340096	3.645796	2.673049	3.096702	4.080468	4.638681	5.19177	1.20187	1.239408	1.585009	2.78961	2.82168	2.540667	3.061141	2.748219	2.337009	2.363486	4.23321	3.379199	5.242678	3.352919	4.532848
LOC107813022	0	0	0	0	0	0	0	0	0	0	0.17658	0	0	0	0	0	0	0	0	0	0	0	0	0	0	0	0	0	0	0
LOC107810689	0	0	0	0.251076	0.245233	0	0.232451	0.236797	0	0.540496	0	0.468274	0	0	0	0	0	0	0.140612	0	0.126379	0.462896	0.611143	0.289668	0	0	0.16378	0	0	0
LOC107780738	4.42861	5.74602	6.409839	3.280827	2.702569	4.339113	3.824253	3.317924	3.748141	1.127478	2.355778	1.52974	1.699997	1.499399	2.14955	4.87715	3.688284	4.879384	1.970209	2.01156	1.770778	4.281454	4.329672	4.400763	0.193073	0.12142	0.541475	0	0.210058	2.697809
107761592	0.727497	0	0	0.659075	0	0.782148	0	0.621593	0	0.709401	0.510121	0.614609	0	0	0	0.702823	0	0	1.230824	0	0	0	0	0	0	0	0	0	0	0
LOC107806066	0	0	0	0	0	0	0	0.08152	0	0	0	0	0	0	0	0	0	0	0	0	0	0	0	0	0	0	0	0	0	0
LOC107762903	0.613825	0.279713	0.444654	0.092682	0.181051	0.439958	0.085807	0	0.480239	0	0.215207	0.345718	0	0	0.210004	0.991084	0.494172	1.220354	0.934301	0.951966	0.746425	0.768932	0.270717	1.390068	0.258687	0.569395	0.725492	0	0	0
LOC107830159	0	0	0	0	0	0	0	0	0	0.055278	0	0	0	0	0	0	0	0	0	0.047954	0	0	0	0	0	0	0	0	0	0
LOC107780494	17.35181	16.8486	17.78544	18.46227	15.11486	20.31163	14.21367	13.48645	17.66088	11.75454	16.52603	11.46256	15.01374	13.2097	12.06713	18.6708	18.77386	19.28386	11.5463	14.10685	8.701363	16.92868	17.54643	21.66728	2.460332	2.858142	5.909754	0	4.293998	1.671166
LOC107829228	0.735859	1.097418	1.104878	0.727255	1.183884	1.654198	0.280544	0.228632	0.188415	0.587091	0.422169	0.14129	0.558524	0.364903	0.205981	0.573288	0.775529	0.822921	0.746699	0.933729	0.244042	0.223467	0.324538	0.45448	0.042289	0.046541	0.039533	0	0.966187	0
LOC107822885	0	0	0	0	0	0	0	0	0	0	0	0	0	0	0	0	0	0	0	0	0	0	0	0	0	0	0	0	0	0
LOC107797019	0.131773	0.120095	0.114547	0.11938	0.116601	0.283344	0	0.056295	0	0	0.184799	0	0.220037	0.071879	0.135248	0	0	0.098242	0	0	0.06009	0	0.116232	0	0.083301	0	0	0	0	0
Novel03114	0	0	0	0	0	0	0	0	0	0	0	0	0	0	0	1.564182	2.027817	2.062821	0.774519	1.089582	0.652612	1.235023	1.683148	1.994438	0.663446	0.132755	0.676597	6.823121	0.229667	0
LOC107766076	1.333744	0.994535	1.053996	0.549229	0.321868	0.782148	0.508486	0.414395	0.113834	0.118234	0.08502	0.20487	0	0.396832	0.248894	0.451776	0.234274	0.361586	0.123036	0.512843	0	0.405034	0	0.25346	0	0	0.429921	0	0	0
LOC107826597	0.769307	0.838306	1.104878	0.499988	0.606741	0.755177	0.406789	0.428685	0.423935	0.34247	0.492531	0.226063	0.316497	0.328412	0.137321	1.134114	0.484705	0.685767	0.40729	0.424422	0.259295	0.391068	0.486807	0.27968	0.253732	0.139622	0.158132	0	0.161031	0
LOC107764370	3.774652	4.052756	4.44988	2.623288	3.111289	3.780253	2.949117	3.954141	3.082629	2.974863	3.607609	3.014195	3.482476	3.666677	2.494348	3.911052	3.421839	4.12487	3.672848	2.668203	2.805911	2.655714	2.782179	2.891393	2.288093	2.482182	1.925097	0	2.738325	1.598578
LOC107779558	0	0	0	0	0	0	0	0	0	0	0	0	0	0	0	0	0	0	0	0	0	0	0	0	0	0	0	0	0	0
LOC107800181	1.624519	3.521305	2.289989	9.228157	13.32584	5.192466	19.92275	21.3457	10.01666	12.7157	17.94874	5.489755	8.553419	6.370594	33.21204	4.384311	3.944763	2.160426	18.40026	20.68775	7.888459	28.45358	13.12877	5.04795	22.8703	34.94456	11.83166	3.139894	13.10548	24.43292
LOC107821146	0	0	0	0	0	0	0	0	0	0	0	0	0	0	0	0	0	0	0	0	0	0	0	0	0	0	0	0	0	0
LOC107783215	1.107025	1.327522	0.861017	2.216956	1.6498	1.75396	1.514937	0.846307	1.31283	1.931717	1.593348	1.821268	3.17818	3.051056	1.13622	1.215725	0.731749	0.999089	2.660524	0.985755	1.487869	1.167796	0.513931	1.339756	1.620608	0.324283	1.239551	12.5002	1.40253	0
LOC107829849	0	0	0	0	0	0	0	0	0	0	0	0	0	0	0	0	0	0	0	0	0	0	0	0	0	0	0	0	0	0
LOC107767687	12.17413	13.10931	10.00976	14.36598	15.88424	15.42296	12.45478	12.45578	13.06761	11.64447	11.55419	13.26574	12.55972	12.77381	12.21663	10.28565	9.963335	10.14583	13.33702	13.93902	14.07324	13.30767	13.64527	11.46816	10.24486	10.57364	8.523325	19.40877	11.01282	7.191827
Novel02131	2.746504	4.589017	4.035975	4.14699	4.802711	4.358937	5.704181	3.631779	3.622234	3.762217	5.227309	2.430815	4.294985	2.140215	4.832463	4.385782	5.369883	4.485297	4.844007	4.702025	1.908463	8.027871	4.729835	3.212389	3.555093	3.548592	2.395966	0	0.62965	0
LOC107788695	1.582624	2.97779	1.730772	0	0	0.054888	0.04282	0	0.143791	0.049783	0	0	0	0	0	2.967456	2.515366	1.788901	0.051804	0.086374	0	0.042635	0.180126	0	0	0	0	0	0	0
LOC107788573	5.761067	4.799539	4.116984	4.258639	3.659136	5.091878	2.816228	3.261475	3.251883	4.962936	6.17102	5.344862	6.373955	6.632068	5.18748	6.031486	5.053496	5.797094	5.20037	4.126002	4.545035	3.335384	2.83699	3.804975	3.485471	4.622784	3.049482	0	7.997447	4.370742
LOC107764582	21.2137	15.48966	20.78859	18.69149	14.68977	28.16849	6.970752	7.86447	10.47511	6.767028	9.499058	5.143118	4.209599	5.757442	4.435668	15.29752	15.39575	16.1565	14.02049	17.96291	6.613311	9.057602	12.55401	14.94096	0.577916	0.925126	2.995977	1.485876	1.300386	0
LOC107770044	5.266443	4.637561	4.701737	5.64154	5.415787	5.279498	7.670396	6.749688	6.948843	5.72533	6.362652	5.59161	5.426939	4.736098	6.026216	4.057146	4.262774	4.032475	3.719151	4.244337	4.640819	6.716085	5.900852	5.57887	2.834313	3.168809	2.060818	0	4.28286	8.800868
LOC107800913	0	0	0	0	0	0	0	0	0	0	0	0	0	0	0	0	0	0	0	0	0	0	0	0	0	0	0	0	0	0
LOC107812750	1.147078	1.4921	1.187492	2.305123	1.974656	1.053868	1.985433	1.969099	1.067137	5.582562	6.215292	2.37866	2.623538	2.263907	6.14352	4.118601	1.057802	1.834783	3.682396	4.428321	2.025736	2.717109	1.756851	1.362416	5.418682	11.02743	2.465008	3.728741	2.359589	1.289556
LOC107774682	0	0	0	0	0	0	0	0	0	0	0	0	0	0	0	0	0	0	0	0	0	0	0	0	0	0	0	0	0	0
LOC107764654	0.263688	0.411976	0.491182	0.085317	0.233329	0.364496	0.300155	0.370139	0.212196	0.165297	0.184898	0.095473	0.104836	0.041096	0.11599	0.421073	0.181961	0.365097	0.172011	0.270861	0.051533	0.487614	0.282431	0.314979	0.095252	0.052415	0.066784	0	0	0
LOC107812846	115.3146	96.79809	131.322	50.56119	49.26777	92.48653	33.30953	36.52524	53.0164	28.43323	25.44181	33.99703	21.44394	28.06809	24.51075	52.11016	117.3945	73.87293	31.19463	38.17105	41.27337	30.30106	41.8964	39.57803	13.01125	5.140336	23.70307	37.742	9.528016	8.157984
LOC107766281	0	0	0	0	0	0	0	0	0	0	0	0	0	0	0	0	0	0	0	0	0	0	0	0	0	0	0	0	0	0
Novel03048	0.170341	0.388111	0.148074	0.46296	0.829008	0.366274	0	0.072772	0.319847	0.083052	0.179164	0.359771	0.189626	0	0.087417	0.063469	0.411409	0.126996	0.51855	0.504338	1.009798	0.213384	0.450755	0	0	0.237017	0.201329	0	0.41004	0
LOC107768701	0.885329	0.806868	0.723652	0.682352	0.4677	0.667706	0.609567	0.609675	0.645099	0.386556	0.426216	0.446538	0.426586	0.490133	0.284809	0.787759	0.727645	0.768417	0.335213	0.402409	0.168718	0.551762	0.594431	0.649123	0.116944	0.183861	0.249884	0	0.063616	0.817033
LOC107800973	0	0	0	0	0	0	0	0	0	0	0	0.02267	0	0.029274	0	0.019997	0	0	0	0	0	0	0	0	0	0	0	0	0	0
LOC107764978	0.077514	0.070644	0.016845	0.140447	0.102884	0.145839	0.081267	0.06623	0.072773	0.094482	0.027176	0.098228	0.172578	0.084563	0.059668	0.129967	0.112327	0.158921	0.294958	0.131142	0.229754	0.016183	0.102558	0.121526	0.098001	0.026964	0.183229	1.385828	0.373178	0
LOC107762108	0	0	0	0	0	0	0	0	0	0	0	0	0	0	0	0	0	0	0	0	0	0	0	0	0	0	0	0	0	0
LOC107780455	0.160674	0.073217	0.034918	0.181953	0.177719	0.129558	0.269529	0.411853	0.678816	0.039169	0.140831	0.203613	0.089432	0.043822	0.082456	0.029934	0	0.029947	0.08152	0.06796	0.109903	0.201275	0.212588	0.419841	0.050785	0.055892	0	0	0	0

LOC107787129	0	0	0	0	0	0	0	0	0	0	0	0	0	0	0	0	0	0	0	0	0	0	0	0	0	0	0	0	0	0
LOC107808613	3.73854	3.590402	3.669167	6.191159	5.442368	5.532061	5.05754	4.052996	3.962797	3.880745	3.35435	3.701807	4.161795	4.429408	3.507059	2.785965	4.03894	4.165875	3.752821	4.080707	4.106199	5.572874	3.581308	3.445341	1.829671	1.566165	1.948009	2.874822	1.93534	4.971174
Novel03653	0.203135	0.026447	0.227032	0.10516	0.025678	0.249594	1.241325	1.041383	1.471206	0.028297	0.142438	0.784521	0.129219	0.158293	2.025351	0.021625	10.59725	0.151446	0.117787	0	0.396991	1.647962	1.663799	0.121324	0.44027	0.161513	0.342984	6.225862	0.977963	3.588609
LOC107782975	0	0	0	0	0	0	0	0	0	0	0	0	0	0	0	0	0	0	0	0	0	0	0	0	0	0	0	0	0	0
LOC107822123	0.280857	0.383949	0.061036	0.190831	0.12426	0.150978	0.235567	0.119986	0.13184	0.068468	0.443108	0.118638	0.70347	0.536201	0.432395	0.366266	0.203498	0.261738	0.213745	0.059396	0	0.527739	0	0	0	0	0.082987	0	0.676072	0
LOC107786374	0.974422	1.256696	1.022846	1.332493	1.610585	1.00809	1.649999	1.492347	1.346354	1.362528	1.856418	0.714492	1.637343	1.303736	2.113461	0.72614	0.40852	0.904664	0.97012	1.182006	0.452728	0.783057	1.135194	0.57649	0.720583	0.639546	0.499789	0	0.796621	1.136792
LOC107832679	7.528985	6.861741	7.527909	7.435636	6.604955	7.64295	7.453189	7.730607	6.67416	5.325104	7.318555	6.770182	7.410139	6.415892	5.240137	7.0554	6.774156	9.299084	7.410369	8.473781	8.015899	7.71789	7.724154	8.173257	2.982324	5.395382	3.666398	9.243414	6.844967	0
LOC107772973	27.94759	27.17951	25.97498	20.38267	19.54541	28.47228	20.14832	19.57392	21.17777	19.71088	19.35037	24.89787	23.87183	25.12024	18.76228	19.69138	29.77328	27.16994	14.09046	13.77862	18.2749	18.20208	24.34154	19.84131	7.259448	8.39699	14.47305	25.14023	13.82166	3.622729
LOC107812541	1.937063	1.729366	2.302404	1.2893	1.364236	0.935036	1.558384	1.283531	1.410343	0.848069	1.774067	0.667953	1.100186	0.603783	1.866425	2.828085	1.527645	2.239926	1.04297	2.207123	0.937398	2.277974	1.150702	0.950331	0.649744	0.77008	0.513959	0	1.141924	0
LOC107804647	0	0	0	0	0	0	0	0	0	0	0	0	0	0	0	0	0	0	0	0	0	0	0	0	0	0	0	0	0	0
LOC107800701	4.523409	5.287592	4.245532	4.187066	5.510839	6.519531	5.30607	5.881405	5.84701	5.465674	5.125472	5.538408	6.020732	6.543996	4.97913	5.300539	5.224992	5.57178	4.323953	4.464241	4.603735	4.708334	5.522316	6.064001	7.210867	4.925734	4.726444	9.376454	7.574713	12.1604
LOC107801522	9.076454	6.978114	8.55114	6.477184	5.249603	6.269298	5.741507	4.852396	5.760259	5.587307	5.688863	8.139261	5.926948	7.080735	3.695103	6.196987	7.152057	6.540059	2.366856	2.273391	4.578268	4.827476	4.830456	8.320743	3.846502	1.128869	6.35268	0	8.055924	3.135256
LOC107762345	2.362384	2.190143	2.230602	1.8081	2.198517	2.277109	2.083921	1.496463	2.25615	2.144752	2.827483	2.477555	2.357787	1.910716	2.591906	2.579972	2.754454	2.277488	1.6119	1.826137	1.56018	2.040919	1.652652	2.000874	2.368819	1.75689	2.455165	0	0.980462	0
Novel00156	3.125284	3.417973	1.956055	4.530159	4.314113	5.712106	3.355277	4.699777	3.462302	4.997977	4.645876	5.49188	6.888638	3.818675	2.758627	2.468703	3.804302	3.68145	4.756984	5.076049	4.047443	3.445208	3.142646	4.638495	4.34644	5.914102	5.171368	31.29028	4.513869	11.59446
LOC107805877	9.504944	8.631424	8.827145	11.89433	9.25771	12.71858	8.517069	9.056106	10.14344	7.73488	8.990385	9.387637	11.9893	12.01063	8.808126	8.611821	8.422865	9.915776	7.910255	8.27191	7.078373	6.281733	9.017312	9.755885	7.04602	4.61463	7.233454	7.335301	8.394854	8.456187
LOC107815764	0	0	0	0	0	0	0	0	0	0	0	0	0	0	0	0	0	0	0	0	0	0	0	0	0	0	0	0	0	0
LOC107761943	0.34385	0.793887	0.338755	0.913764	0.689658	0.394326	1.499688	1.468972	0.322821	0.737653	2.025304	0.30986	0.637964	0.500164	1.576366	0.939536	0.354334	0.888696	1.16305	1.221667	1.045322	0.976337	0.909894	0.86254	0.28982	1.243948	0.406403	0	0.882888	1.417383
LOC107766514	0	0	0	0	0	0	0	0	0	0	0	0	0	0	0	0	0	0	0	0	0	0	0	0	0	0	0	0	0	0
LOC107817770	4.636897	4.471915	4.883826	5.178756	5.970019	6.805236	5.391329	5.01001	5.712297	4.2584	4.524428	4.228282	6.145069	6.744836	4.885088	4.20501	4.811457	4.993449	4.779888	5.229994	4.36318	4.896837	5.734726	5.769623	3.784225	3.755076	3.682623	5.263551	8.149912	6.067859
Novel04555	3.240762	2.987899	3.24297	0.955896	0.800272	1.134396	0.821771	1.030324	1.37977	0.220476	0.396353	0.318359	0.461445	0.575549	0.425448	2.499265	1.893071	2.107086	0.344146	0.350652	0.61862	0.566465	1.030414	1.693622	0	0	0.044539	0	0	0
LOC107774340	1.99442	2.583001	3.284918	2.329877	2.322097	3.103513	2.333124	2.15253	2.808674	1.535377	1.545698	2.128347	2.278643	0.744357	1.346722	1.642691	2.585938	3.091238	1.278189	1.775939	2.058279	2.629882	2.453635	3.785135	0.066357	0.876344	1.36472	0	0.50536	3.24521
Novel01222	0	0	0	0.03991	0	0	0	0	0	0	0	0	0	0	0	1.969729	3.106844	2.758883	0.804646	0.484462	0.843731	0.515065	1.282321	1.012986	0	0	0.052068	0	0	0
LOC107793307	0	0	0	0	0	0	0	0	0	0	0	0	0	0	0	0	0	0	0	0	0	0	0	0	0	0.046452	0	0	0	0
LOC107778275	0.033068	0	0.028745	0	0	0	0	0.028254	0	0	0.023187	0	0	0.036076	0	13.77507	11.50074	10.62571	5.570155	6.9094	4.282521	6.517371	6.533675	5.149842	9.992123	5.291352	6.448819	14.18899	17.98974	14.31252
LOC107801492	7.142695	6.288268	5.19528	28.39281	27.77504	23.40349	31.17706	34.50014	31.88713	6.775505	12.91297	10.26249	8.708619	9.753663	13.51509	3.548496	7.32292	4.49199	14.05208	11.75563	17.77019	28.9731	42.90224	45.04719	2.150128	3.380455	8.671807	0	3.508926	15.02189
LOC107761997	0	0	0	0	0	0	0.060514	0	0.033868	0.035177	0.101181	0	0	0	0.074051	0	0	0	0	0	0	0.09038	0.06364	0	0	0	0	0	0	0
LOC107788691	8.813902	11.12439	10.14697	19.72752	19.42571	22.66282	19.58107	14.42883	15.06167	19.54384	19.98475	15.66837	22.2125	17.84126	17.36291	11.30381	11.54888	11.57404	14.20476	19.49826	8.092467	17.975	11.96872	13.93455	13.65307	7.152225	12.04558	7.415513	23.891	10.99113
LOC107800571	0.903099	1.02883	0.654204	0.272721	0.066593	0.323647	0.631224	0.70733	0.211967	0.366932	0.422169	0.190741	0.502671	0.246309	0.077243	0.280413	2.035763	0.841624	0.152734	0.19099	0	0.691352	0.531062	0.07866	0	0	0	0	0	0
LOC107761223	3.066049	3.559253	3.63308	3.243219	3.395091	2.964898	3.864624	3.527103	2.942867	2.171352	3.675274	1.85227	2.116546	2.205024	3.21723	4.097364	2.945513	3.422419	2.833117	3.187746	1.718389	4.405841	3.656306	3.06141	1.754133	2.240342	1.13371	1.224949	2.144063	9.531876
LOC107823660	0.775555	0.434968	0.311157	0.37833	1.161366	0.705537	0.200151	0.968496	0.280048	0.232697	0.501987	0.504008	0.199236	0.911176	0.244926	0.711316	1.786675	0.667164	0.242147	0.756999	0.598501	0.548042	0.789335	0.810609	0.150852	0.830098	0.352555	0	0	3.688747
LOC107813386	0	0	0	0	0	0	0	0	0	0	0	0	0	0	0	0	0	0	0	0	0	0	0	0	0	0	0	0	0	0
LOC107786921	0.030312	0.055252	0.0527	0.054923	0.026822	0.065179	0	0	0	0	0	0	0	0	0	0	0.029284	0.022599	0	0	0	0	0	0.031682	0	0	0	0	0	0
LOC107805190	1.826835	1.463994	1.560649	1.198463	1.086959	1.794754	1.083139	0.995745	1.005411	0.58356	0.750918	0.77168	0.420757	0.790322	0.711212	1.384831	1.004154	2.066457	0.830989	0.985844	0.545793	1.867591	1.528041	1.514357	0.238932	0.131478	0.632861	0	0.303277	0
LOC107805599	0	0	0	0	0	0	0	0	0	0	0	0	0	0	0	0	0	0	0	0	0	0	0	0	0	0	0	0	0	0
LOC107830394	0	0	0	0	0	0	0	0	0	0.040313	0	0.10478	0.046022	0	0	0	0	0	0	0	0	0	0	0	0	0.057524	0.146587	0	0	0
LOC107791654	1.032679	0.575153	0.797943	6.652833	18.19949	11.19508	1.08269	2.37744	1.238837	7.776218	2.75566	12.40797	8.047147	10.26459	6.330037	0.3634	0.748241	0.406339	20.78317	11.59914	20.77237	5.581755	2.454345	6.955847	25.49585	20.5157	15.46021	49.23409	26.10134	30.15237
LOC107801741	62.24708	52.10805	62.68751	45.61543	42.10584	50.06848	46.02153	48.45798	50.90799	33.90138	34.72416	45.18678	35.10912	38.33058	27.63778	37.59032	61.1159	46.13283	27.79217	28.55166	38.94028	37.42859	54.17396	50.50632	15.27348	11.03504	26.48557	32.97442	17.75879	5.701971
LOC107761867	0.342007	0.407605	0.663207	0.023834	0	0.028285	0.022066	0.022478	0.024699	0	0	0	0.08786	0.028701	0	0.333284	0.228744	0.372665	0.026696	0	0.047987	0.021971	0.139234	0.054995	0	0	0.062189	0	0	0
LOC107805377	0.145905	0.232705	0.190248	0.198273	0.419595	0.117649	0.183565	0.155831	0.068491	0.355689	0.383657	0.523873	0.365452	0.278556	0.449258	0.13591	0.176195	0.271945	1.036378	0.524557	0.798405	0.304622	0.160872	0.114375	0.553403	0.152262	0.258671	0	1.756092	2.255376
LOC107778491	0.728219	0.663682	0.452161	0.471235	0.391228	0.391462	0.588975	0.533323	0.512763	0.481857	0.528864	0.417471	0.289523	0.340479	0.347018	0.445764	0.402012	0.678648	0.527819	0.52802	0.403233	0.912232	1.055269	0.788318	0.164409	0.18094	0.399609	0	0	0
LOC107788077	18.13704	14.81059	13.43275	15.24819	16.04884	21.99547	14.66467	13.32723	13.7585	12.59236	12.00551	15.50654	13.81024	14.32551	11.02029	9.67727	14.78849	14.1169	13.83997	12.581	13.82851	9.390962	12.4145	14.78632	12.93293	11.81062	18.94988	20.75287	18.51145	13.45728
107815274	0	0	0	0	0	0	0	0	0	0	0	0	0	0	0	0	0	0	0	0	0	0	0	0	0	0	0	0	0	0
LOC107775781	22.59621	20.6879	20.85601	21.82951	22.0993	24.8496	18.12839	16.08165	18.25305	19.26098	18.56378	17.12412	21.98852	18.33338	12.47174	19.99839	18.33306	20.2003	15.5309	14.82821	11.36512	16.88429	17.60526	23.29328	13.00944	8.201992	17.29531	3.697822	16.18101	25.57725
LOC107797104	1.972635	3.748633	1.860708	4.068522	3.193926	2.887931	1.830548	1.470306	0.709275	6.834807	6.710055	3.687656	2.943528	1.83153	2.843138	2.283207	3.243798	1.783595	2.427586	2.591832	1.837355	0.595868	2.110206	0.921229	0.159192	0.233598	0.446457	3.001517	0.404126	0
Novel04701	0.643486	0.293229	0.503432	1.515711	2.505355	0.138365	3.022429	2.034304	0.845786	1.505952	1.443881	1.685268	1.71921	1.263621	2.972054	0.239763	0.310831	0.527721	0.457076	0.381041	0.352122	0.483653	0.340558	0.672571	0	0.089536	0.15211	0	0.929391	0
LOC107801447	2.909987	2.959179	3.035508	6.993134	10.57084	4.215366	7.039583	9.291178	5.924178	10.45433	9.407708	11.51584	6.27428	7.452079	8.928753	4.405531	2.071478	3.562578	18.71177	17.54249	20.33769	8.313862	6.781758	8.164073	17.04008	24.33683	20.85344	15.33407	16.81165	5.681964
LOC107811786	0	0	0	0	0	0.069353	0	0	0	0	0	0	0.07181	0	0	0	0	0	0.196372	0	0.058832	0.107743	0	0	0.081557	0	0	0	0	0
LOC107818499	12.65844	11.08196	11.8168	12.59775	13.18741	16.82735	13.70296	13.74608	11.41596	13.68131	10.79999	15.22475	15.5493	12.9934	9.920213	9.107807	11.32549	9.995282	11.51613	13.0819	10.97601	8.332135	12.65066	11.40569	11.90454	6.507376	20.34142	13.37819	13.50937	3.855619
LOC107793262	4.737868	2.158991	3.2033	3.57689	5.356926	4.669305	1.655772	1.686735	3.088972	4.106684	2.676213	6.226395	3.516174	6.460976	1.080626	1.667258	3.051453	3.434163	2.670923	1.558632	4.200987	1.099088	1.044778	2.200897	3.827012	0.732489	4.199834	0	11.40489	0
LOC107781118	3.960122	5.246309	4.294191	2.626027	2.312034	4.91601	1.095761	0.906954	0.613268	0.915642	1.23097	0.758801	0.636273	0.489931	0.670443	5.871752	4.811845	5.448316	3.52133	4.489679	1.228722	0.613708	1.296404	0.512056	0.051616	0.056806	0.337771	0	0	0
LOC107761193	23.82032	17.8448	18.86351	19.48979	17.54643	21.98953	16.31803	16.99613	21.07561	24.93038	21.29391	31.08168	13.25856	12.44917	9.856211	13.84758	18.67501	17.29416	15.81886	15.61389	24.22684	11.71706	14.90578	14.72963	13.95433	10.23827	27.9326	31.21578	6.30437	11.56686
LOC107804812	5.757775	4.591564	6.325905	1.666303	1.344477	2.278379	1.341471	1.195736	1.01356	0.97475	1.037376	1.655223	1.557898	2.311923	1.27221	2.264537	3.901472	3.994564	0.649177	0.947077	0.984598	0.701235	1.304954	0.794045	0.808842	0.333814	0.850653	2.859461	0.962499	0
LOC107826573	0.056505	0.411976	0.098236	0.255952	0.149997	0.121499	0.805678	0.869023	0.212196	0.110198	1.228253	0.28642	0	0.369862	0.347969	0.505288	0.545882	0.758278	0.286685	0.621387	0.206133	1.274089	0.747612	0.177176	0.571512	0.39311	0.267136	0	0.272033	0
Novel00009	2.053448	1.656707	1.989857	1.524853	1.519153	1.628637	1.609377	1.380607	1.580219	1.116076	1.628716	1.73481	1.42402	1.505716	1.762094	1.756004	1.821197	1.932489	0.478226	0.68344	0.675429	1.658662	1.633119	1.688863	0.212802	0.187359	0.477445	2.407387	0.162066	0
LOC107777831	107.5688	98.11831	92.01054	82.01723	74.17453	72.2932	86.11812	128.844	119.7931	87.57299	74.79188	97.15507	45.09432	69.10636	57.57466	68.61245	89.56251	85.60215	101.7049	98.35521	98.18722	74.6973	107.2727	82.59456	56.02683	108.9456	94.57673	168.5004	62.38927	84.04982
LOC107797749	5.868727	7.972945	5.459146	22.45962	14.99671	17.30717	34.7555	30.5316	24.79419	16.31251	26.44083	16.40331	16.11917	13.76237	21.61705	6.049164	5.457733	5.622574	20.09177	25.51866	19.23355	30.04803	34.32525	25.1379	14.25028	6.334301	16.0768	9.806004	12.01461	25.43497
LOC107801064	0	0	0	0	0	0	0	0	0	0	0	0	0	0	0	0	0	0	0	0	0	0	0	0	0	0	0	0	0	0
LOC107773406	17.71481	15.44796	19.71972	16.51055	14.32197	17.53838	18.38565	16.87829	16.87075	16.90135	16.53239	17.22701	20.42974	17.24045	22.62939	37.13402	24.99382	33.54044	24.95916	26.19769	23.71126	24.05372	22.82017	19.71436	18.69094	17.91033	19.12993	27.34229	17.17979	0
LOC107767268	0	0	0	0	0	0	0	0	0	0	0	0	0	0	0	0	0	0	0	0	0	0	0	0	0	0	0	0	0	0
LOC107828813	0.517823	1.125375	0.761762	3.716883	5.815661	1.027796	22.81852	20.04602	6.245219	8.545182	15.27803	4.475665	6.828686	5.258094	14.55435	0.771764	0.654187	0.504846	2.991027	3.740208	3.996072	10.27894	7.343197	3.663698	11.93542	4.489347	6.591036	14.24294	1.917679	14.77743
LOC107818244	11.50933	12.78154	12.54761	11.61568	10.11164	13.9611	15.31699	12.77704	11.67812	10.60992	15.98735	9.723403	13.29928	10.79647	13.94535	10.08432	14.23547	11.94368	5.853308	6.244056	6.25813	12.46559	13.43149	12.6296	1.347982	1.863908	2.29411	1.955059	3.816842	1.690355
LOC107800815	1.277555	1.099649	1.172249	2.1862	1.004858	1.449835	0.05953	0.060643	0.133269	0	0.049768	0.059962	0	0.232292	0.072847	0.952036	0.34284	0.264575	0.792229	0.360241	0.258923	0.237093	0.25042	0.8902	0	0	0	0	0	0
LOC107768111	9.100502	10.42827	26.38676	9.291952	10.8856	8.063161	9.19936	11.06836	20.09359	12.19833	10.20589	15.55202	15.21271	16.36374	13.44789	32.03085	9.536437	29.65868	11.03936	9.403581	13.78796	10.00139	10.01445	22.27692	5.54677	7.548115	7.392602	8.479669	19.83718	9.164458
LOC107831802	8.122801	22.97152	8.259206	34.20749	37.1141	9.156423	116.1504	112.6439	45.94114	32.16307	65.24181	20.33756	20.60575	15.73601	65.12982	19.73684	7.894869	7.377179	75.43098	90.24348	83.77923	106.6458	95.87869	34.31998	47.17833	63.4298	60.68678	35.20577	18.24949	109.5807
LOC107826595	0	0	0	0	0	0	0	0	0	0	0	0	0	0	0	0	0	0	0	0	0	0	0	0	0	0	0	0	0	0
LOC107770645	0	0	0	0	0	0	0	0	0	0	0	0	0	0	0	0	0	0	0	0	0	0	0	0	0	0	0	0	0	0
LOC107791887	99.58698	78.15237	83.10176	177.3645	188.0432	150.6526	80.80984	87.8587	120.6739	148.3387	105.6197	221.6806	202.8185	191.4536	89.35673	104.2131	119.5694	125.0526	150.3162	140.9686	182.872	59.92966	78.0851	134.4419	36.79546	39.28117	109.1228	20.05535	73.2072	13.48664
LOC107799698	0.345868	0.35024	0.300656	0.38297	0.306047	0.330534	0.35456	0.328354	0.288636	0.112422	0.26947	0.227266	0.299464	0.125775	0.354989	0.400931	0.334137	0.687624	0.506946	0.422616	0.140194	0.417218	0.711849	0.843502	0.583043	0.053472	0.227104	0	0.925073	0
LOC107818034	139.7761	167.029	130.4393	21.78017	19.01236	19.66642	39.06252	31.30844	43.12478	25.14206	14.98467	25.462	8.985669	14.57102	8.701916	87.93347	126.5081	86.19868	8.838918	9.333523	9.90379	29.2433	29.45276	21.66828	8.296347	1.131216	12.76609	12.45863	3.354876	0
LOC107828188	0	0	0	0	0	0	0	0	0	0	0	0	0	0	0	0	0	0	0	0	0	0	0	0	0	0	0	0	0	0
LOC107772908	0.796896	0.785159	0.805059	0.37073	0.38116	0.231557	0.379357	0.644085	1.05147	0.336032	0.271841	0.072783	0.239761	0.093986	0.15474	0.786447	1.019556	1.043724	0.34968	0.109317	0.196428	0.251814	0.588929	0.787893	0.108921	0	0.101823	0	0.10369	0
LOC107815943	17.70025	10.97343	9.244406	4.931497	3.413179	6.278715	4.172592	7.946807	7.648914	0.667901	0.70778	0.2741	1.003267	0.904547	0.333001	5.104169	15.01033	5.536528	1.755861	0.945354	1.972659	4.455656	15.35841	8.741484	0	0	0.085215	0	0.173555	0
LOC107767733	12.67344	13.79473	14.82074	19.15299	16.36046	20.18366	17.68449	15.87861	18.94137	17.36429	18.86249	17.01257	21.23618	17.17412	16.50749	15.17576	14.69599	15.39456	17.72353	17.65977	14.35728	17.92464	17.71232	22.57213	11.30477	10.35908	13.50124	8.128521	12.12993	10.54194
LOC107778719	1.958122	1.147234	1.155033	0.950335	0.433169	0.977424	0.41059	0.298763	0.525249	0.068193	0.441333	0.827138	0.233551	0.839227	0.215332	0.677483	1.013416	0.729932	0.212889	0.177475	0.57402	0.584028	0.370113	0.804033	0	0.194613	0.16531	0	0	0
LOC107797663	3.172783	3.207048	12.0852	4.076394	3.981527	4.093356	6.096452	6.752635	10.99429	6.862753	4.206814	7.943894	3.339332	3.524284	3.789332	17.71122	4.458436	16.25705	2.107323	4.587125	4.577199	5.829283	4.376	16.70155	2.115082	0.963206	4.363606	0	2.221805	0
LOC107769793	0	0	0.056845	0.029621	0	0.070305	0	0	0	0.063766	0	0.027623	0	0	0	0.024365	0	0	0.033178	0	0.02982	0	0	0	0.124015	0	0.154578	0	0	0
LOC107766862	12.18282	12.78017	10.78331	10.69211	11.17318	11.63131	10.96333	11.57496	16.38221	8.260162	9.944118	13.56236	42.41682	22.46293	11.69004	11.27738	16.58168	13.97901	9.722418	6.467978	11.05306	11.49895	9.486746	14.292	12.03342	6.842402	9.261926	6.80659	27.34058	1.961671
LOC107806887	0.425223	0.422769	0.907292	0.385231	0.17103	0.457167	0.129692	0.165146	0.435511	0.07539	0.325273	0.457215	0.344265	0.33738	0.119028	0.547333	1.195058	0.806965	0.627618	0.228906	0.352555	0.581097	0.511466	0.888886	0.195495	0.053788	0.274134	0	0.744428	0
LOC107773240	0	0	0	0	0	0	0	0	0	0	0	0	0	0	0	0	0	0	0	0	0	0	0	0	0	0	0	0	0	0
LOC107771319	1.56191	2.033555	1.163773	0.960186	0.83912	1.079514	1.263254	1.000904	0.680824	0.65274	1.056097	0.754026	0.558881	1.034551	0.801551	2.452582	1.293374	2.287352	0.226417	0.471882	0.406997	0.931708	1.2793	0.874558	0	0	0.263722	0	0.268557	0
LOC107769136	0	0	0.147833	0	0	0	0.14264	0.145307	0	0.165834	0.119249	0.143675	0.189318	0.371063	0	0	0.172569	0	0	0.142025	0	0.177751	0	0	0	0	0	0	0	0
LOC107778721	9.523593	10.18347	11.06553	7.47466	7.050397	9.478703	6.841077	6.20362	7.701799	6.298409	7.0416	6.771214	7.242812	6.429314	6.097124	6.956458	9.382782	8.928028	4.640578	5.663343	5.546794	5.945364	6.113191	6.208966	1.43058	1.836826	4.067806	0	3.858661	0
LOC107818348	1.191352	1.121962	0.897534	1.69091	2.108373	1.366239	1.998476	1.3233	1.379475	2.865572	2.561821	1.54328	3.00612	3.55253	2.649337	1.65722	1.381135	1.7468	2.901363	2.956218	1.122751	1.359736	1.436163	1.36972	2.912046	2.762798	2.675355	0	4.2061	7.366307
LOC107789346	1.595323	0.72697	1.791258	0.903301	1.058735	1.071979	0.501775	0.397567	0.811286	1.101912	0.466101	1.066987	0.591978	1.160279	0.477574	0.693488	1.284347	1.288496	1.146666	0.899689	1.151844	0.333074	0.410428	1.389526	0.840405	0.184981	0.707079	0	1.600094	0
LOC107823871	1.774583	2.048597	1.619737	0.964609	1.282385	1.271929	1.413998	1.617335	1.693824	0.490292	0.953993	0.824569	1.185294	0.838926	0.75891	1.344461	1.685823	1.345077	0.540217	0.675529	0.485535	1.3091	1.721829	1.391095	0.037394	0.205767	0.279655	0	0.284782	0
LOC107808468	0.083407	0	0	0	0	0	0	0	0	0	0	0	0	0	0	12.36888	17.5661	11.31743	10.24095	9.454612	13.61632	10.23937	13.24273	18.74311	11.17793	5.918811	14.09704	23.85919	14.85743	15.47159
LOC107831398	2.087599	1.867997	1.517754	0.137546	0.100759	0.367269	0.286521	0.129724	0.106905	0	0.21292	0.064133	0.211268	0.165634	0	1.385971	1.356754	1.386605	0.077031	0.064217	0.10385	0.19019	0.3348	0.079344	0	0.211254	0.089723	0	0	0
LOC107787577	5.737421	5.04086	5.166822	9.124218	9.277117	8.83106	6.88944	6.665592	7.944183	9.74043	8.248767	12.76295	13.55509	12.92376	7.117316	4.244773	5.144066	5.38533	6.952818	7.542072	9.335905	5.308535	5.315644	9.016697	6.575427	4.479783	9.220439	23.61477	17.08986	0
LOC107808966	0	0	0	0	0	0	0.076539	0	0	0	0.063987	0	0.20317	0	0.374642	0	0	0	0	0	0	0	0	0	0	0.253946	0	0	0	0
LOC107810150	0.083299	0.08972	0.065827	0.137208	0.341738	0.048849	0.107975	0.181167	0.127971	0.228912	0.260186	0.127951	0.261328	0.380022	0.411933	0.389375	0.095105	0.180663	0.653154	0.486849	0.442006	0.474306	0.280541	0.158298	0.42126	0.96938	0.295357	0.541549	0.984346	1.404678
LOC107823600	6.395203	4.329698	4.394427	1.820887	1.616828	2.75025	3.014019	2.497936	3.716823	2.256885	4.057242	5.248479	6.915823	4.651235	2.125441	7.670529	7.296283	7.810267	0.865255	1.133503	0.999863	1.831132	3.330872	3.373962	0.231014	0	1.007815	0	0.293227	0
LOC107813311	0	0	0	0	0	0	0	0	0	0	0	0	0	0	0	0	0	0	0	0	0	0	0	0	0	0	0	0	0	0
LOC107784796	11.39472	12.39636	11.73452	20.99482	24.70739	16.83093	38.20743	40.12792	30.23832	32.43294	25.66152	34.45185	40.19696	37.82518	45.938	15.56748	11.55371	14.59881	20.02616	18.8658	19.21616	32.02014	23.88219	23.17574	9.377128	14.17658	11.1927	9.176613	28.66465	3.17366
LOC107823090	0.262555	0.119643	0.114117	0.237862	0.116163	0.705697	0.330325	0.224334	0.616246	0	0.46026	0.665442	0.58456	0.143217	0.13474	0.097828	0	0.195746	0.266423	0.777363	0.239455	0.219267	0.347387	0.274423	0	0	0	0	0	0
LOC107793781	0.500216	0.781518	1.490836	0.064739	0.189696	0.076828	0.119872	0.183171	0.670893	0.69682	0.551181	1.509273	0.715947	0.701629	0.806784	1.491046	0.276143	1.864661	0.145024	0.544048	0.065172	0.358066	0.252128	0.970962	0	0.397722	0.422296	0	1.032093	0
LOC107819114	0	0	0	0	0	0	0	0	0	0	0	0	0	0	0	0	0	0	0	0	0	0	0	0	0	0	0	0	0	0
LOC107789596	9.117959	10.78518	6.464507	1.113105	0.686653	2.572398	0.7051	0.663032	0.910675	0.504463	0.362753	0.27316	0.359938	0.776026	0.066372	10.79444	10.05818	10.31727	1.640474	1.203473	0.530791	0.864073	1.1408	0.94625	0	0.089979	0.458583	0	0	0
LOC107775101	2.63898	3.789863	3.406257	5.795836	3.750382	3.868945	6.573206	4.372977	4.354554	3.275159	3.869132	3.715807	3.560909	2.96624	5.417154	4.231108	4.712669	3.696462	3.65162	2.908879	3.792525	5.676664	5.290353	4.931437	3.235368	2.781779	2.362926	0	7.314995	0
LOC107770506	0.228566	0.20831	0.132459	0.172558	0.303376	0.081912	0.031951	0.065098	0.214588	0.074294	0.026712	0.128733	0.212036	0.290914	0.078198	0.198716	0.331221	0.198807	0.077311	0.193351	0.069485	0.190882	0.235212	0.079632	0.048163	0	0.045024	0	0.5502	0
LOC107811026	10.49977	12.355	11.33571	15.39224	13.36553	14.98025	11.17058	11.14204	12.11242	14.70952	13.37488	12.15711	14.58299	13.07875	12.63051	13.1492	11.21747	13.08124	13.39358	14.7587	11.53099	12.41532	11.15231	13.42232	12.02018	11.37842	11.58881	14.19437	15.19356	12.27253
LOC107816577	64.22122	57.02272	52.905	95.19676	102.5154	111.3103	99.14041	99.62704	94.09624	150.734	126.0446	147.356	146.9029	165.5539	127.0121	47.97697	74.61661	55.3161	85.35611	80.18156	103.18	68.1992	72.26773	63.89045	111.0027	78.07791	121.926	194.5423	138.1569	105.5387
LOC107817851	4.323567	5.208572	4.017584	5.132532	7.299757	5.93067	5.835524	4.84064	4.852323	5.572905	5.54067	5.290073	6.638696	5.80112	6.885916	4.77734	5.425207	4.112617	6.101817	6.263891	5.801439	5.021811	6.706802	4.259279	4.461006	6.223373	3.348002	7.107957	3.349571	0
LOC107817810	11.27061	9.774748	9.074919	9.316584	10.01893	15.32808	6.098803	5.658129	6.558482	10.12928	12.40072	13.07591	12.98024	11.02078	9.115674	6.734578	10.03535	9.138438	6.060889	6.085166	5.684231	4.120627	5.176869	6.758592	6.041252	4.299966	7.335732	0	9.876951	3.211422
LOC107772208	1.967315	2.801508	1.122283	1.893681	2.012811	1.255843	2.836061	1.943572	2.077877	1.558684	2.672747	1.454287	1.984726	2.280384	2.397798	1.511859	1.781805	2.200075	0.873379	1.196153	1.401738	2.464434	1.952215	2.377524	0.466366	0.427715	0.508639	8.793177	2.367839	3.801314
LOC107769487	0.037819	0	0.065751	29.26008	35.70718	45.33634	8.628029	16.96477	14.48663	20.02509	6.576695	49.65157	14.94585	21.12462	9.044295	0.028183	0	0.084588	90.45326	86.82777	132.8626	18.76084	8.006194	22.1361	13.10134	18.99684	44.1183	10.81847	18.02549	2.338426
LOC107817426	459.0399	520.9187	492.2968	274.1563	193.0098	344.1361	258.2181	255.0824	316.1038	181.529	176.5519	168.6545	129.0547	168.5493	141.0262	389.0129	455.3928	399.2352	141.0732	178.5476	173.36	241.2207	318.4571	293.3399	140.1772	89.84305	288.0894	105.2521	71.94613	65.3344
LOC107767936	1.024185	1.422352	0.890303	52.04853	87.99416	50.70416	25.32088	45.62978	45.65074	96.44685	70.14023	135.5986	58.58134	80.44818	80.99213	1.235696	1.507732	1.054459	179.1013	166.8833	173.293	54.86292	49.08469	55.00219	153.4126	232.4236	90.15377	244.1458	169.9949	138.7161
LOC107788459	0	0	0	0	0	0	0	0	0	0	0	0	0	0	0	0	0	0	0	0	0	0	0	0	0	0	0	0	0	0
LOC107783493	5.810155	6.402609	5.588356	3.562508	3.420955	4.013992	4.558228	3.586415	4.231122	3.726824	3.407979	2.855562	3.861093	3.422358	3.786635	6.799164	4.930136	6.48934	2.891829	3.233055	2.538665	5.202752	4.774184	4.456445	2.038949	1.291046	2.689411	7.899396	2.12716	1.365972
Novel00985	0	0	0	0	0	0	0	0	0	0	0	0	0	0	0	0.181551	0.171174	0.297219	0.854018	1.049193	0.848368	0.277444	0.42979	0.90281	0.420025	0.400623	1.151789	0	1.066278	0
LOC107794871	27.139	25.27449	24.90629	23.72671	18.37165	29.56041	15.97117	16.32051	18.26712	15.32885	20.77689	16.66336	14.29682	15.79057	15.4647	24.77718	25.67413	26.86714	13.96502	15.68151	12.19979	17.1408	19.56931	20.8015	2.51246	1.898415	8.027794	2.121118	3.28427	3.667859
LOC107793188	0.44082	0.401753	0.24795	0.328885	0.504792	0.306664	0.500229	0.332337	0.146069	0.303427	0.490928	0.219069	0.404128	0.424335	0.47906	0.734293	0.626278	0.521973	0.868314	0.92129	0.733123	0.887867	0.663302	1.0299	0.262273	0.288644	0.490366	0	0.249678	0
LOC107815908	1.106225	2.142399	0.769294	2.806115	2.789753	0.981195	9.255191	7.6324	4.180256	3.074321	9.153092	2.967259	3.817542	3.771367	9.565738	2.555517	2.40459	1.58762	1.178647	0.889001	3.480691	15.31261	5.7814	2.803849	0.979023	1.308347	0.817168	1.977769	0.532576	5.129968
LOC107770629	44.61537	28.68616	42.93669	22.23871	20.39993	25.68513	22.54267	23.2704	33.92445	11.82281	13.31783	12.5641	10.37211	9.513098	6.988337	28.75213	39.92868	34.64303	14.60602	13.3889	24.78439	22.84449	40.30199	43.19856	8.985917	5.152477	17.08312	4.271258	11.21416	25.85067
LOC107802331	0	0	0	0	0	0	0	0	0	0	0	0	0	0	0	0	0	0	0	0	0	0	0	0	0	0	0	0	0	0
LOC107825174	0	0	0	0	0.194472	0	0.12289	1.189285	1.788234	7.429365	0.410951	3.403991	6.605735	20.93939	43.00892	0	0	0	0	0.061972	0	0.48944	0.064619	0	1.296693	9.785638	2.33781	0	0.352692	0
LOC107817111	7.250406	6.672318	8.608462	7.273413	7.417101	6.464207	7.742441	6.859767	7.637122	5.725025	5.927187	6.394262	5.787679	7.022391	5.626589	8.170407	7.722036	8.015939	5.311547	5.594807	6.773772	5.936857	6.644912	7.541156	4.292707	4.035364	5.392449	7.587901	6.810934	10.93423
LOC107826600	0	0	0	0	0	0	0	0	0	0	0	0	0	0	0	0	0	0	0	0	0	0	0	0	0	0	0	0	0	0
LOC107795489	0	0	0	0	0	0	0	0	0	0	0	0	0	0	0	0	0	0	0	0	0	0	0	0	0	0	0	0	0	0
LOC107809910	19.26149	20.38415	17.84319	24.53409	21.82629	24.23199	23.96793	26.43032	23.04976	20.63259	18.50547	22.1988	24.8345	21.3897	16.34669	14.9107	16.77517	14.10306	24.27109	25.1461	27.53007	16.37388	20.84434	21.87489	19.77268	23.36084	14.67867	45.23033	18.82315	21.33075
Novel02471	4.701357	4.847345	4.582158	1.849952	1.680836	2.246459	3.146618	2.556252	3.120897	1.250295	1.431854	0.92275	1.744539	1.502419	0.779854	3.963506	3.349082	3.859107	0.96376	0.964127	1.905652	2.696802	3.89558	4.864222	0.300199	0.19823	0.505148	0	0.457253	2.936282
LOC107817649	0.126445	0.086429	0	0.143191	0.055943	0.135944	0.106055	0.027009	0.029678	0.06165	0.044332	0.026706	0.07038	0.172431	0.06489	0.023557	0.122156	0.047135	0.128307	0.080223	0	0.105597	0.167299	0.13216	0	0	0	0	0.152188	0
LOC107826010	0.444996	0.388661	0.515768	0.419942	0.344542	0.239213	0.357686	0.411902	0.539635	0.253124	0.351036	0.454267	0.495376	0.182051	0.228366	0.38688	0.376166	0.469998	0.451551	0.329382	0.405844	0.495504	0.425226	0.387592	0.046884	0.232192	0.460206	0	0.357062	2.292899
LOC107821848	0	0.207195	0.158099	0.247153	0.201168	0.048884	0.266955	0.660442	0.341503	0.266025	0.223178	0.153652	0.151849	0.297624	0.233338	0.508248	0.043926	0.372886	0.092277	0.269243	0.082936	0.455664	0.401063	0.190095	0.229944	0.316331	0.05374	0	0	0
LOC107798090	6.002592	6.317266	5.901226	9.063407	11.76115	7.605935	8.570862	11.29552	7.916533	6.410741	6.914821	8.331186	7.398116	8.497501	8.874624	5.112158	6.144188	5.487432	13.1972	11.36455	13.3603	9.250034	7.815961	8.888036	21.05072	23.16733	10.13512	40.88288	15.4814	53.02133
LOC107830713	0	0	0	0.024767	0.072572	0	0.481522	2.125614	1.103646	1.892724	1.246016	0.785263	4.169339	24.63504	38.86179	0.040745	0	0	0.027741	0.069378	0.17453	3.926888	0.819876	0.428606	10.36909	22.59513	12.69838	0	11.05568	40.56851
LOC107778681	0.257016	0.140543	0.044684	0.093137	0	0	0	0	0.048259	0	0	0	0	0	0	0.038306	0.04966	0.076646	0	0	0	0	0	0	0	0	0	0	0	0
LOC107801527	7.16975	6.016528	5.644539	9.804424	10.46206	10.87897	6.785132	7.281884	7.493309	13.719	11.02241	15.97731	8.915147	7.29055	7.025605	5.988088	6.01175	7.745719	17.29616	18.46999	14.70643	6.642898	8.018601	9.840942	11.25397	11.85848	14.61377	9.674312	12.50454	8.364462
LOC107801617	0.404087	0.398965	0.526896	0.62539	0.536344	0.615461	0.268316	0.244561	0.34776	0.213436	0.177091	0.199141	0.224918	0.11021	0.086405	0.401501	0.536775	0.426791	0.820079	0.370316	0.307112	0.323403	0.400985	0.492743	0.106435	0.070282	0.0796	0	0.081059	0
LOC107824119	0.142627	0.812418	3.192552	0.323032	0.44172	0.460024	0.448603	1.127245	1.606854	0.834476	2.000203	1.235076	0.55571	0.894694	2.049438	6.058291	0.206684	2.525444	0.470366	3.076642	0.74795	3.811569	0.503226	2.049763	0.676215	0.595365	0.674295	0	2.059971	0
LOC107827747	0.59956	0.769963	0.758087	0.839446	1.085176	1.318501	1.302909	1.303987	1.535164	1.195869	1.471442	1.703766	2.062993	2.319049	1.398573	1.523158	1.290093	1.198766	0.636046	0.899104	1.068763	0.728303	0.649045	1.538166	0.172279	0.303362	0.386527	0	0.524818	0
LOC107827997	0	0	0	0	0	0	0	0	0	0	0	0	0	0	0	0	0	0	0	0	0	0	0	0	0	0	0	0	0	0
Novel00243	0.581766	0.574392	0.505717	0.652539	0.527042	0.871191	0.482144	0.402396	0.474663	1.452007	1.558895	0.924474	1.040835	1.171133	0.526025	0.449016	0.441599	0.542165	0.822254	1.001845	0.473733	0.549471	0.48872	0.492241	2.215345	1.397326	2.783148	0.495292	1.13367	0
LOC107804326	0	0.042528	0	0	0	0	0	0	0	0	0	0.03272	0	0	0.071841	0	0	0.017395	0	0	0	0.03897	0.02058	0	0	0	0	0	0	0
LOC107770143	0	0	0	0	0	0	0	0	0	0	0	0	0	0	0	0	0	0	0	0	0	0	0	0	0	0	0	0	0	0
LOC107796308	8.723352	9.070523	9.237503	8.980585	8.175118	9.46393	9.112556	9.215197	8.15264	9.202345	10.31518	10.08313	12.53596	11.54987	7.976674	9.573687	9.615001	10.13975	6.638891	6.742047	7.160259	6.556589	8.359104	9.324864	9.224228	4.634474	7.545265	0	11.26292	19.61374
LOC107802537	0	0	0	0	0	0	0	0	0	0	0	0	0	0	0	0	0	0	0	0	0	0	0	0	0	0	0	0	0	0
Novel02684	0.436498	5.171584	1.138315	0.098861	0.193121	0	0.183055	0	0.102451	0.21282	0.153036	0.092191	0	0	0.112002	3.415428	0.737964	2.277995	0.332196	0	0.099523	0.091133	0	0.114057	0	0	0	0	0.525364	0
LOC107761878	18.93623	22.15258	18.94666	30.45612	21.78591	28.11983	35.83097	28.66446	34.73781	23.09002	24.47766	25.956	36.95806	41.69743	29.02816	18.63315	24.35829	17.28971	12.59779	15.90074	15.0438	28.8324	24.94684	22.96325	5.379042	4.027468	7.666452	12.46969	13.26352	6.468814
LOC107760910	24.06802	23.9241	23.18791	44.92696	60.56492	47.51548	43.62807	45.99788	38.58984	62.4273	41.95746	57.41476	67.55578	65.54335	55.25452	24.53145	26.12159	26.21502	66.37769	59.02828	53.74263	38.73141	33.52883	26.80337	98.26284	117.3376	56.03307	99.71707	54.87137	56.22777
LOC107781149	31.48254	18.97404	19.11623	10.93445	6.394166	14.32013	1.670836	1.401713	3.703817	0.723684	2.492404	2.111946	0.652236	1.917575	1.523433	11.87595	13.69797	17.18142	3.408663	4.692001	8.015306	3.522983	5.788222	13.67662	0	0.217398	0.046166	0	0	0

LOC107812236	57.91705	33.41639	40.91431	31.59041	31.58357	37.48947	31.25531	32.35125	39.6924	39.767	34.94475	45.453	34.73603	35.75566	25.26774	33.06692	38.45847	40.1113	32.11861	30.95347	40.81027	25.43384	35.97188	40.12196	41.53187	23.63479	53.06457	69.56659	68.0872	18.50697
LOC107803044	2.834919	2.92173	2.510397	4.56052	4.501274	5.81092	4.488871	4.278521	4.402745	3.565306	5.331888	3.357502	6.193766	3.98879	3.779869	2.843105	3.327478	3.930812	3.71011	3.339475	2.537167	4.381009	4.977802	4.984581	4.354635	3.022951	4.885054	7.578945	6.377714	16.38199
LOC107813035	0	0	0	0	0	0	0	0	0	0	0	0	0	0	0	0	0	0	0	0	0	0	0	0	0	0	0	0	0	0
LOC107812815	0	0	0	0	0	0	0	0	0	0	0	0	0	0	0	0	0	0	0	0	0	0	0	0	0	0	0	0	0	0
LOC107775123	18.67719	21.0869	20.76714	22.98169	20.54751	24.69573	28.01061	25.91437	29.7571	15.73896	18.00274	16.41488	16.88163	18.09501	15.99387	18.61308	19.60573	18.28908	17.70769	20.63383	17.41527	29.54272	28.74434	29.60259	9.057772	10.977	13.64033	7.974228	9.528692	15.51276
LOC107769359	0	0	0	0	0	0	0	0	0	0	0	0	0	0	0	0	0	0	0	0	0	0	0	0	0	0	0	0	0	0
Novel03507	5.324316	4.72131	5.128674	3.780629	4.074643	4.331896	2.775997	3.811526	5.404003	1.683853	1.816255	1.945137	0.80096	0.784942	0.738477	2.680869	4.587658	6.007898	0.730101	0.730379	2.887272	2.8842	5.457977	5.715379	0.181934	0.200227	0.680315	0	0	0
LOC107790323	0	0	0	0	0	0	0	0	0	0	0	0	0	0	0	0	0	0	0	0	0	0	0	0	0	0	0	0	0	0
LOC107770041	6.996272	8.012348	5.950451	24.82108	30.76081	18.30857	20.98153	21.70359	18.17732	24.57318	22.11723	17.5335	16.60393	17.69955	22.97161	8.114259	5.478507	6.829606	33.38559	33.50901	28.65967	21.60564	17.50719	16.97482	58.63004	58.15202	42.35647	28.79611	36.50962	47.71976
LOC107793819	12.95828	15.64882	14.31105	11.20064	10.81194	12.58351	10.38322	10.30265	9.509254	8.230621	11.12684	7.103662	8.465492	8.682061	9.554238	16.06626	11.63347	14.43149	11.17516	11.17942	7.492663	11.46628	10.25302	9.611867	5.610131	7.919106	4.92153	5.748762	6.656548	6.958575
LOC107822878	3.030427	3.3461	3.059824	3.452885	3.537545	3.771853	3.441138	2.868124	2.965468	2.59135	3.636912	2.195854	3.529214	3.051735	3.445307	4.082271	3.141591	3.971175	2.885833	2.859325	2.00907	3.971391	3.104835	3.191766	1.193622	1.7353	1.474016	0.833532	1.68341	2.882705
LOC107765551	0	0	0	0.013624	0	0	0	0.012849	0	0	0.010545	0	0.016741	0	0.015435	0.201723	0.130758	0.145756	0.793532	1.539325	0.397751	2.536959	1.830587	1.037417	0.608431	1.590318	0.728755	0	0.941221	0
LOC107816974	1.267645	1.140491	1.455122	1.222039	1.351788	1.747274	2.330925	2.027361	3.082109	1.014248	0.934457	0.974832	1.863456	1.808459	1.184313	1.429087	1.083346	1.223762	1.286319	1.003601	1.05236	2.212294	1.533869	1.715631	0.821892	0.587945	0.441793	1.162238	2.112541	0
LOC107827181	5.49488	2.666547	2.729476	0.937431	1.673365	0.728868	0.957671	1.128013	1.205961	1.739676	2.952309	3.13501	3.535136	3.114101	2.783275	3.695947	3.240263	3.750844	1.774126	1.780838	1.822336	1.370716	0.660934	0.895051	1.172904	0.744713	0.927787	0	2.404937	0
LOC107795612	2.632724	2.441132	2.467677	2.903618	2.208063	2.436692	1.862551	2.679804	1.826921	1.651962	2.215281	1.392542	1.783956	1.748279	1.64479	1.927789	2.521316	2.235894	2.671506	1.781683	2.129661	2.982525	2.665524	2.464586	0.984097	0.477814	0.811739	1.637191	1.102163	2.831049
LOC107782942	4.857439	4.630964	4.611637	3.204119	4.001073	3.802442	2.121559	2.44812	1.786323	1.615252	1.89922	2.250416	1.719394	2.124575	1.263617	3.419598	3.741181	4.622744	4.338423	4.73394	3.735955	1.719838	1.382123	2.45661	1.471643	1.681907	4.418268	0	1.185437	5.536273
LOC107771599	0.309253	0.281846	0.168017	0.070042	0.034206	0.083121	0.064846	0	0	0	0	0	0	0	0	0.201649	0.560184	0.374662	0.039226	0	0	0.161416	0.170489	0	0	0	0	0	0	0
LOC107799447	5.801481	5.894078	11.47099	4.157289	3.36625	3.655467	6.00267	5.789871	8.616478	5.239844	3.984641	3.274197	4.208475	4.487457	5.515216	14.5113	6.431644	11.23856	2.509188	2.4136	2.341939	5.083245	6.270777	7.082607	1.322675	1.554917	2.444872	6.801441	2.289376	2.940281
LOC107831368	0.191633	0.058217	0.111055	0.63657	0.16957	0.206029	0.53577	0.272894	0.47977	0.062289	0.089582	0.053966	0.213329	0.696875	0.131125	0	0.308556	0.047624	0.194456	0.054036	0	0	0.281722	0.26706	0	0	0	0	0	0
LOC107763797	0.791634	0.240492	0.229383	0	0.466993	0	0	0	0.12387	0.128657	0.185031	0.222931	0.146876	0.143939	0.406256	0.196642	0	0.098366	0.133883	0	0	0.110186	0	0	0	0	0.155941	0	0	0
LOC107832275	273.6926	261.9518	260.9702	234.0065	212.8823	292.4076	224.0313	217.434	232.9902	235.9471	215.0144	272.6683	179.1827	249.4753	222.8871	181.4685	243.6552	201.5313	165.7469	179.3465	194.2663	182.0959	195.5056	206.1994	116.5569	101.6967	148.6855	118.7916	133.0182	54.7775
LOC107807635	2.169198	2.790998	2.856187	1.011492	0.733907	1.268966	0.615385	0.57238	0.419287	0.279959	0.536839	0.24255	0.071023	0.348013	0.196448	1.854208	2.095628	2.330713	1.068206	1.592126	0.640053	0.799215	1.547584	0.833546	0.120994	0.044386	0.339329	0	0	0
LOC107829749	0	0.048465	0	0	0	0.057172	0	0	0	0	0	0	0	0.174042	0	0	0.154122	0.039646	0.053961	0	0	0	0.046906	0.055581	0	0	0	0	0	0
LOC107798462	1.385042	1.032787	1.696528	0.285177	0.055708	0.406115	0.211217	0.053792	0.118213	0.06139	0	0.053187	0.140168	0.480777	0	0.703728	1.033961	1.079544	0.191652	0.266284	0.401921	0.105153	0.166595	0.065802	0	0	0.074409	0	0	0
LOC107825974	1.034297	1.615945	0.899093	1.003951	0.653724	0.714854	0.49572	0.441866	0.485522	0.648367	0.362625	0.499316	0.657939	0.402988	0.758267	1.266248	0.7851	1.487145	0.8996	0.499968	0.067378	0.123396	0.390994	0.772176	0.560428	0.205593	0.087318	0	2.489737	0
Novel03857	1.326443	2.068544	2.382967	0.427268	0.208662	0.285218	0.07417	0.176299	0.193717	0.08623	0.186021	0.024903	0.164068	0.12863	0.060508	1.40582	0.854304	1.780055	0.059821	0.174546	0.161298	0.1477	0.390003	0.27728	0	0	0.034839	0	0	0
LOC107775897	0.28848	0.112677	0.25077	0.186677	0.1094	0.221537	0	0	0	0.120559	0.057795	0.034817	0.045877	0.089919	0	0.122843	0.079627	0.430148	0.083637	0.17431	0.075171	0	0	0	0	0	0.048709	0	0	0
LOC107798688	17.26854	11.18978	14.16656	17.36491	13.71713	20.56007	12.89101	12.56603	18.99056	12.79075	11.61321	16.37991	9.685537	12.72002	7.343429	8.063404	9.728113	8.363439	10.48686	12.55316	17.27981	12.2084	21.50399	18.9723	3.517798	5.530721	7.621134	9.475279	5.740911	0
LOC107826845	45.54682	55.8954	55.20269	41.79704	44.23043	44.0775	56.81652	51.75745	54.06986	46.85639	50.63759	48.51524	49.04263	44.08347	55.44355	84.32545	49.58905	71.70396	49.5444	50.52612	44.47897	59.84767	51.59594	51.47479	26.26604	36.82571	23.07426	25.02531	37.24101	33.02492
LOC107825514	3.759418	4.195403	4.08736	2.829927	2.608892	3.063781	3.337045	3.371351	2.623978	2.009302	3.673641	1.926008	2.074216	2.104478	3.059852	4.296213	2.922473	4.461609	1.757254	2.651719	1.189532	3.789474	3.606089	2.886864	0.873009	1.052291	0.984528	2.351471	0.949811	2.033095
LOC107819861	20.98111	21.11816	21.41218	18.3022	16.2394	17.53291	16.98531	16.55424	17.00152	18.08577	18.15955	23.07184	21.02624	21.721	18.78642	17.44897	22.19771	18.76351	10.91678	11.03624	11.72082	16.13964	16.10214	19.94512	9.231962	5.215578	7.997495	0	11.4838	6.019925
LOC107778705	0	0	0.09486	0	0	0	0	0	0	0	0	0	0	0	0	0	0	0	0	0	0	0	0	0	0	0	0	0	0	0
LOC107767512	8.5162	9.210897	16.92422	8.342691	8.897567	10.67276	8.842571	8.306576	13.67885	19.83544	12.59065	8.473303	11.30789	11.47352	12.21605	23.8559	9.292958	16.7718	7.652547	8.289077	6.901328	5.762516	7.873869	15.06758	5.578119	3.854712	6.700203	0	10.00302	11.10237
Novel04719	0.658865	0.900711	0.76365	0.696381	1.068846	0.47224	1.289443	1.032079	1.237143	0.963715	0.692995	0.556627	0.611215	1.078184	1.352482	0.654649	0.742605	0.900555	0.334285	0.557354	0.200299	1.375588	0.871744	0.459097	0.138834	0.305587	0.259575	0	0.528668	0
LOC107777078	0	0	0	0	0	0	0.026402	0	0	0	0	0	0	0	0	1.712405	1.52053	1.830531	1.405445	1.065136	1.320599	2.628829	3.109778	3.487509	0	0.175199	0.483661	0	0.454642	0
LOC107783223	0	0	0	0	0	0	0	0	0	0	0	0	0	0	0	0	0	0	0	0	0	0	0	0	0	0	0	0	0	0
LOC107813890	15.37426	17.35176	15.57899	14.73809	13.48552	15.23186	15.36911	16.42024	15.77674	12.42057	13.78894	13.21515	14.2292	13.60333	12.88983	13.9881	13.25529	15.06075	10.47608	11.15303	11.29064	12.61551	14.34956	16.20932	7.458669	7.151451	9.455333	12.78462	9.252148	8.290238
LOC107783250	3.724482	3.054966	2.884419	3.527564	2.636533	3.385431	2.925091	2.025099	2.988096	2.410221	3.442576	2.431422	4.636144	3.213643	2.536887	2.80072	2.84582	3.155408	2.027104	1.947678	1.420476	2.516606	3.434569	3.680492	1.883551	0.895132	2.241038	0	2.282125	0
LOC107829258	0.915562	2.415431	1.089097	1.4188	0.916748	0.569881	3.920439	3.149709	1.289354	2.607881	5.034572	1.485919	1.367895	0.893692	4.154496	3.698663	1.769019	2.532768	1.320227	1.9974	1.494224	3.199287	2.550271	1.561331	1.980017	1.810332	0.825829	0	0.927966	0
LOC107787291	8.882639	10.62991	9.854498	12.15234	11.92743	12.34631	12.92475	11.37736	11.59532	9.682594	11.84908	6.965317	9.706173	7.923742	12.96178	11.77587	9.197718	10.72021	10.29164	11.95614	6.504764	14.63133	12.06284	11.1136	7.34213	7.556848	6.786365	8.189024	5.512879	11.12614
LOC107761579	0	0	0	0	0	0	0	0	0	0	0	0	0.12495	0	0	0	0	0	0	0	0	0	0	0	0	0	0	0	0	0
LOC107812427	4.590787	4.694173	4.749875	2.170806	2.377891	4.068908	1.709242	1.473327	2.270652	1.244712	1.585977	0.927038	2.268576	2.296499	1.08028	3.921703	3.158647	4.758286	1.249818	1.856498	0.306357	2.28163	2.429627	1.919316	0.509633	0.592035	1.138124	0	0.646879	0
Novel01908	0	0	0	0	0	0	0	0	0	0	0	0	0	0	0	0	0	0	0	0	0	0	0	0	0	0	0	0	0	0
LOC107776756	0.187071	0.596721	0.406541	0.127107	0.082766	0.050281	0.039226	0.079919	0	0.045604	0.032794	0	0	0.051021	0	0.069703	0.180726	0.104602	0	0.039562	0	0.117171	0.123756	0	0	0	0	0	0	0
LOC107793462	1.200912	1.295138	1.061326	0.52585	0.566744	0.624045	0.268602	0.22232	0.338241	0.214691	0.659629	0.202913	0.423343	0.502219	0.349232	1.118651	0.8508	1.16393	0.24372	0.372492	0.091271	0.551602	0.158893	0.251038	0	0	0.070969	0	0	0
LOC107814332	5.840466	7.00377	5.183878	9.078528	11.58726	9.253581	10.20982	9.192571	6.753101	12.70927	8.578657	11.32266	12.31899	11.26778	11.35799	5.864182	6.058136	6.187712	18.5905	14.92591	16.20409	10.4225	8.296912	8.41772	21.68601	13.94351	14.02391	39.5693	20.42261	11.40394
LOC107785091	0.972664	1.510271	1.346563	0.489547	0.573785	0.619694	0.513661	0.461706	0.67643	0.386414	0.378907	0.39565	0.120309	0.432312	0.295797	1.046977	0.904872	0.510299	0.292442	0.121897	0.19713	0.240681	0.762626	1.242546	0.091092	0	0	0	0	0
LOC107797944	0.040292	0.257049	0.105075	0.328524	0.071306	0.043319	10.07083	7.505017	4.312394	0.03929	1.864689	0	2.108126	0.967048	1.240642	0.42036	0.077851	0.240316	0.776828	0.306759	0.845183	4.542616	0.746347	0.210567	0.35659	0.448508	0.095244	0	1.745826	2.491323
LOC107771030	5.787819	5.538627	5.893724	29.13872	31.02131	14.26756	18.2737	22.78356	19.8724	37.04787	19.48043	22.24818	35.02275	30.93971	29.65986	8.19487	6.829642	7.952048	51.38944	37.76989	51.54164	13.05061	17.94127	17.67297	48.60952	16.16418	64.44951	85.73868	79.6132	61.34907
LOC107829766	27.06218	31.12554	28.37751	35.49207	33.2088	26.30418	35.59839	33.46167	33.53117	35.30334	32.01163	26.75393	32.84495	35.62919	40.91176	30.61937	27.45223	29.83218	28.42387	28.85501	24.75754	34.82712	28.53642	24.39049	52.7427	43.86939	39.32594	31.93941	46.0943	50.91512
LOC107824057	0	0	0	0	0	0	0	0	0	0	0	0	0	0	0	0	0	0	0	0	0	0	0	0	0	0	0	0	0	0
LOC107761421	7.129468	4.972676	4.110583	5.536231	7.338601	6.335398	6.650993	6.588885	4.507839	8.796574	6.52955	9.034758	10.44719	7.698532	5.376115	0	0	0	0	0	0	0	0	0	0	0	0	0	0	0
LOC107789031	0	0	0.015477	0.064519	0.031509	0.019142	0.089599	0.136911	0.050146	0.416671	0.287138	0.180497	0.059459	0.291352	0.785768	0.026535	0.0172	0.079642	0.144531	0.316283	0.194852	0.059475	0.125635	0.037218	0.135059	0.47069	0.168344	0	0.085715	0
LOC107796445	16.49275	14.01048	16.99176	10.23776	8.287878	13.35352	7.215456	6.350034	9.558101	4.517003	4.104765	5.074559	4.079992	5.719928	4.806634	6.865944	8.901058	4.781796	2.324404	2.540594	4.410366	4.251089	5.163535	6.118504	0.643576	0.779114	1.38377	0	0.490136	6.294887
LOC107831465	2.482152	3.074238	2.268332	1.729772	1.858468	1.368521	1.601452	2.175198	2.390106	1.179175	1.383463	1.935684	1.629559	1.319236	0.587911	2.1817	2.951344	3.368949	1.033319	1.453659	0.870678	1.807152	2.975368	3.25956	0.724199	0.265672	0.902678	0	0.612819	0
LOC107784111	2.013294	0.932984	1.156855	1.360228	1.721105	2.568095	1.774499	1.836837	1.409617	1.896666	1.43565	2.85403	2.773043	2.456995	1.646107	0.839156	2.373573	1.373793	2.319962	1.818573	3.361102	1.852339	1.053475	1.997296	4.271121	5.080417	2.984522	14.64193	5.421348	4.21983
LOC107776032	2.696017	3.042108	2.879268	1.930702	2.226572	3.257416	2.368945	2.45712	2.53114	1.377073	1.72841	1.800444	1.800746	1.932803	1.159554	1.588126	1.810803	2.182281	1.511166	1.390107	1.28795	1.372352	2.423362	2.415323	0.71418	1.250437	1.001464	0	0.49446	1.587608
LOC107823140	2.336187	1.568844	2.244566	2.896218	7.07204	7.667253	3.300144	1.891043	2.770503	4.795951	5.173059	6.440414	6.843883	5.63389	3.533597	0.824651	1.544231	0.550019	4.86597	2.18428	3.812727	0.821478	1.843758	1.670693	0.777276	1.026516	2.325208	8.793177	8.879395	7.602628
LOC107766680	2.782345	2.189979	1.777329	4.583026	3.842216	4.283086	3.182281	3.097709	3.601654	4.316356	4.759215	4.790244	4.129794	5.311954	3.591288	2.041992	2.769434	2.954387	3.101387	3.904957	2.55677	2.886909	2.788878	3.591071	2.664947	1.965044	3.21377	3.014804	4.160631	3.909923
LOC107818314	13.31689	17.53079	17.05176	8.694047	8.603942	11.68109	7.588133	7.982879	7.938084	5.565278	5.365536	5.35737	4.376771	4.612086	6.118097	13.20018	17.97049	15.41251	16.47306	12.83872	17.11899	12.67473	13.57359	12.2839	6.57429	6.705907	7.045296	12.09328	3.460026	7.841936
LOC107772111	0.018951	0.069085	0	0	0	0.020374	0.03179	0	0	0	0	0	0	0	0.01945	0	0.018308	0	0.01923	0.016031	0	0	0	0	0	0.052737	0	0	0	0
LOC107831869	40.09916	51.59356	40.74827	41.44966	36.67506	46.49279	59.41497	58.92648	49.8443	38.1525	55.73629	36.24922	44.38887	47.46321	63.52193	40.54011	45.46697	31.48671	28.9882	36.39158	23.97098	53.99956	53.73218	25.0061	17.94058	21.88041	29.47189	26.77551	13.51902	27.7803
LOC107816101	18.39062	14.55542	15.37171	10.92744	9.124868	13.24815	7.43148	8.620129	11.98828	11.14471	10.23288	12.17163	6.423619	11.16882	6.419242	9.987239	13.27122	10.82446	6.950853	6.42443	8.14861	6.684362	9.260185	9.494203	2.776981	2.538201	6.028065	7.98698	5.018404	4.603724
LOC107775308	2.640143	2.514134	5.914091	1.456567	2.74051	1.091722	2.626053	2.226889	2.20857	3.185085	2.800639	3.402891	3.711485	3.027972	5.315319	12.96491	2.0928	7.065831	2.541648	1.918416	2.39242	3.50515	2.060075	1.998852	2.246693	4.356473	0.940132	4.841219	7.170076	10.46436
LOC107797413	3.453153	3.753798	4.629209	4.372197	3.423719	6.038505	3.140576	2.666086	3.359152	2.515303	1.867068	3.128211	3.010439	2.76868	2.177775	3.224368	3.416423	3.939253	3.757324	3.941766	5.957174	3.300756	3.486282	4.304991	1.946216	1.794568	3.048719	17.85176	1.001489	0
LOC107810153	11.06615	28.54952	13.27007	116.14	78.63773	22.75771	310.7688	280.7472	63.61501	128.1072	267.8451	41.18349	58.37306	39.56105	457.5814	39.20262	9.265385	13.28507	86.89643	240.6548	85.9675	96.01815	154.8757	39.2069	101.7395	147.8972	25.35371	81.16779	2.732122	42.10686
LOC107781642	14.58899	27.15282	73.9523	7.006544	8.009988	5.480285	8.034756	13.1786	27.97116	96.79755	51.39556	50.52584	42.99865	20.0866	32.24767	136.0604	18.29698	117.4816	23.27602	33.60398	24.52684	15.37635	11.89945	48.91433	89.55788	102.8428	166.9768	25.14023	62.40904	54.34093
LOC107792277	5.087183	4.530626	5.660967	2.762236	2.346045	2.797026	1.598328	1.472473	2.800303	0.969507	1.068979	0.713966	1.604859	1.066587	0.561252	4.013247	3.890095	5.102247	2.034582	2.368977	0.39293	1.300825	2.177842	2.70186	0.125702	0.046114	0.509214	0	0.398885	0
LOC107806559	2.107232	2.332014	2.355135	9.72704	9.456274	13.37743	15.94892	15.26118	26.04843	6.947239	6.684346	8.816466	14.29821	15.54485	8.702715	2.467633	3.635291	2.319141	7.789424	3.989568	15.69495	15.29354	11.37358	22.39186	3.298511	4.537711	5.396253	7.176041	6.521763	3.102222
LOC107828742	0	0	0	0	0.04884	0	0	0	0	0.053822	0	0.04663	0	0.120429	0	0	0	0.04115	0	0	0	0.092189	0	0	0.069783	0.153599	0.065236	0	0.531454	0
LOC107774822	1.004751	1.330366	1.928091	1.064825	0.88907	1.059849	1.160739	1.765567	1.815416	0.665497	1.95408	0.624619	1.751618	1.344315	3.268865	2.641787	0.970674	2.148318	1.673604	2.309299	0.553268	3.008073	2.140394	1.24831	0.407458	0.712208	0.560158	0	0.547611	2.34435
LOC107813534	0	0	0	0	0	0	0	0	0	0	0	0	0	0	0	0	0	0	0	0	0	0	0	0	0	0	0	0	0	0
LOC107776969	0.155892	0	0	0	0	0	0	0	0	0	0	0	0	0	0	0	0	0	0	0.065937	0	0.065095	0	0	0	0	0	0	0	0
Novel04451	3.80216	2.918063	2.652808	1.948898	2.523301	2.25904	1.888245	1.538843	2.1136	2.195281	1.403199	2.704986	3.007386	2.237721	1.437739	2.721531	3.093227	3.31955	1.97985	1.777465	2.418219	1.211621	2.029905	2.509905	1.13852	0.487276	2.246924	0	2.649401	0
LOC107787319	15.45417	13.51519	12.97665	13.37513	13.58758	16.08485	12.7966	11.68735	14.07687	11.12597	13.34961	11.19492	19.61965	16.46515	12.95937	15.33895	14.39	18.50832	10.14314	9.93004	6.927277	11.75286	13.10954	16.6682	6.900926	4.254917	7.345096	7.054413	13.29735	10.16547
LOC107817836	5.786999	6.040464	4.901528	3.80882	3.632646	5.530428	3.899639	3.63447	4.922286	3.424418	2.462454	2.883267	3.634044	3.237606	1.675277	5.639395	7.693224	5.531351	4.165782	2.928874	3.56367	3.263223	4.842746	5.273112	1.375757	1.238798	2.689139	0	1.905003	3.058281
LOC107801849	2.89077	2.071636	2.46993	12.893	10.84393	12.6708	9.491221	9.520927	5.659928	10.72133	10.27365	16.26048	14.54997	12.23739	10.01682	2.890685	3.866852	3.297259	10.47984	9.050042	7.007948	8.913812	7.976454	7.953855	5.872689	3.231588	7.913796	8.834655	14.03615	0
LOC107785263	7.684284	6.399999	6.879928	7.144085	6.290229	8.385313	6.734809	6.786975	6.592884	5.472523	7.426467	7.002495	5.895065	5.682977	6.262288	6.369746	6.561741	7.166564	4.847872	6.159532	4.593387	5.311736	6.778046	7.821045	2.983716	4.164721	5.578583	2.058183	2.216921	1.779516
LOC107812819	4.436718	5.06949	4.086998	8.038884	10.07843	8.685698	7.609157	6.167208	4.973596	8.329858	9.518822	4.699314	6.781933	6.718544	5.777115	8.339639	5.693666	8.689046	10.75122	8.45861	7.186821	7.631646	6.658755	6.782853	11.135	10.68822	11.03555	4.735643	22.95397	8.188924
LOC107831248	0	0	0	0	0	0	0	0	0	0	0	0	0	0	0	0	0	0	0	0	0	0	0	0	0	0	0	0	0	0
LOC107819441	0.291403	0.265578	0.379966	0.131998	0.064463	0.156647	0	0	0	0	0.204332	0	0	0.079477	0	0.162866	0.14076	0.271567	0.295696	0.246507	0.132882	0	0	0.076144	0	0	0	0	0	0
Novel00382	0	0.156989	0.012478	0.416144	0.215931	0.200628	1.276217	0.760425	0.606451	0.265953	0.060393	0.181907	0.303614	1.973174	0.780855	0.074879	0.027735	0.05351	0.349584	0.510004	0.052366	0.263733	0.291218	0.195044	0.199634	2.256984	0.135727	0	0.483755	0
LOC107774115	2.893815	3.979442	3.00673	2.916391	2.969734	3.092795	3.073454	2.560337	3.2795	2.571376	2.713531	2.372445	3.640793	3.119649	2.425315	3.981183	3.192687	4.39152	3.197074	2.723184	1.733444	3.446301	3.232205	3.454146	1.537071	1.381887	1.558342	0	2.143362	0
LOC107823968	0	0	0.162038	0	0.49483	0	0	0	0	0.545305	0	0	0	0	0	0	0	0	0.189151	0.473057	0.170004	0	0	0	0	0	0	0	0	0
LOC107809385	5.60746	4.199332	4.232077	0.90575	1.076991	0.56081	1.203149	1.151365	2.979153	5.934244	2.712744	2.570649	3.387296	2.133999	3.569205	5.215259	6.719104	4.083376	0.617526	0.294172	0.832528	4.791842	1.687059	2.680574	18.08106	0.483868	10.94319	24.869	31.39106	5.375468
LOC107771399	3.652885	3.388603	3.118672	3.309329	3.088013	4.242885	3.364744	2.786718	2.878321	3.27579	3.247496	3.196275	4.53844	2.988838	2.811916	3.11101	3.434467	3.550121	2.71383	3.614301	2.022692	3.186808	3.193317	3.170299	3.09265	2.722887	3.122417	0	3.61147	2.016637
LOC107773633	0.419458	0.430069	0.364626	0.095002	0	0	0	0	0	0.051128	0	0	0	0.057201	0	0.195363	0.25327	0.469085	0	0	0	0.043787	0.184995	0	0	0	0	0	0	0
LOC107828227	0	0.024037	0.045854	0.812395	0.723475	1.786416	0.022121	0.04507	0	1.51739	1.01716	0.623894	0.528488	0.805652	0.351911	0.019654	0	0	1.445203	1.53946	0.144324	0.066078	0	0.055133	0.900325	0.183491	0.623451	0	0.634881	0
LOC107817499	5.248595	4.894047	5.854759	3.792975	2.859064	3.86521	3.99508	3.823525	3.887971	1.789836	2.765557	2.012032	2.414802	2.449254	2.475535	5.346894	3.986124	5.179702	1.939494	1.950498	1.452644	4.573263	3.97397	4.518675	0.28768	0.548783	0.735087	0	1.16849	0
LOC107784328	1.274284	1.222476	0.932806	5.529143	8.367756	4.975302	8.550372	8.882173	10.26345	10.72549	11.05154	19.60455	14.78279	17.77968	8.122684	0.199915	1.814201	1.500051	3.674998	1.758771	4.037005	2.856503	2.484637	5.467721	4.154895	2.146351	4.042691	14.38884	11.30105	0
LOC107758997	0	0	0	0	0	0	0	0	0	0	0	0	0	0	0	0	0	0	0	0	0	0	0	0	0	0	0	0	0	0
LOC107823313	18.51564	18.65101	20.43673	27.36848	26.73155	27.10961	28.60764	28.04974	23.44458	21.4998	21.48203	21.09683	20.06945	22.45882	24.63011	17.88277	23.35987	17.48228	25.95764	23.649	26.9963	21.15975	23.90706	22.34454	19.25115	18.89863	23.61168	21.77845	16.71391	18.82976
LOC107828785	7.084025	7.536811	6.040573	6.689707	7.437098	7.955049	7.968393	6.847852	7.411691	8.327434	9.397922	6.428568	9.373021	8.154036	9.458266	7.21392	7.017758	7.843836	6.472587	7.389178	4.311729	8.347653	7.069366	7.388534	4.231501	4.510787	4.807234	4.293288	5.20246	10.208
LOC107774851	0	0	0	0	0	0	0	0	0	0	0	0	0	0	0	0	0	0	0	0	0	0	0	0	0	0	0	0	0	0
LOC107759604	11.79658	6.964564	10.77046	0.873788	0.328251	0.957189	0.062228	0.126784	0.487585	0.217041	0.156071	0.125359	0	0.16188	0.152298	2.266816	5.73408	2.157227	0.301141	0.690377	0.270658	0.619599	1.112523	0.54282	0	0	0	0	0	0
LOC107777239	4.747463	5.843477	4.933969	10.6175	8.649758	4.708583	19.38055	16.09073	9.506346	5.415174	8.303885	2.752792	4.153848	4.223668	12.90987	5.130485	3.350985	3.500253	14.41664	17.41266	10.12942	16.22467	18.14101	6.6832	10.45408	22.13291	5.072783	7.516843	4.723007	10.83184
LOC107771552	0.661918	0.563039	0.882266	1.958902	0.976181	1.328399	0.96231	1.658978	0.952871	0.860605	1.082987	0.932011	0.933351	1.396098	0.951124	0.854987	1.108412	1.085673	1.253781	1.455836	0.764662	1.326684	1.712642	1.429795	1.227402	0.736808	1.877601	0	2.549366	0
LOC107807058	0	0	0	0	0	0	0	0	0	0	0	0	0	0	0	0	0	0	0	0	0	0	0	0	0	0	0	0	0	0
107798956	0	0	0	0	0	0	0	0	0	0	0	0	0	0	0	0	0	0	0	0	0	0	0	0	0	0	0	0	0	0
LOC107782660	0.878138	0.670534	0.474513	0.623538	0.462021	0.408262	1.333721	0.811141	1.180947	0.115716	0.366122	0.340862	0.924715	0.414273	0.219234	0.65439	1.192283	0.442358	0.337163	0.200769	0.411261	1.288328	1.737562	0.71938	0.360076	0.066047	0.252459	1.697278	0.114261	0
LOC107777786	0	0	0	0	0	0	0	0	0	0	0	0	0	0	0	0	0	0	0	0	0	0	0	0	0	0	0	0	0	0
LOC107829913	0.041374	0	0	0	0	0	0	0	0	0	0	0	0	0	0	0	0	0	0	0	0	0	0	0	0	0	0	0	0	0
LOC107804008	1.373252	1.385645	1.065838	1.643985	0.65097	1.634601	0.781582	1.46668	1.38136	2.726013	1.960241	0.621515	0.818959	0.856086	0.201353	0.292385	0.995008	0.548474	1.094878	2.364842	0.670944	0.573419	1.297821	0.871446	1.302156	1.296601	5.796691	0	1.416712	0
LOC107765126	0	0	0.044552	42.67092	127.7547	59.23508	85.45903	126.732	110.5267	94.50722	47.29452	184.5849	146.2882	247.5856	118.2535	0.343739	0.346598	0.496739	184.3135	97.59401	246.8024	98.61595	81.60015	109.9767	377.6449	369.9756	483.7599	516.8014	363.4574	354.9282
LOC107805578	6.56292	8.234133	7.595932	2.435374	2.318547	4.232236	1.010732	1.293641	1.502678	1.699349	1.260979	0.55341	2.524744	2.76415	1.33832	8.275499	6.489587	8.915094	2.476969	3.314339	0.462157	1.537945	2.19129	0.884896	0.625049	0.584712	1.139423	0	0.267765	0.382105
LOC107772710	0	0	0	0	0	0	0	0	0	0	0	0	0	0	0	0	0	0	0	0	0	0	0	0	0	0	0	0	0	0
Novel00267	0.246493	0.112324	0.589246	0.167483	0.163585	0.066253	0	0.052653	0.115709	0.360543	0.086421	0	0.1372	0.134456	0.063248	0.367374	0.119066	0.137828	0	0	0.056201	0.102926	0	0	0.155821	0	0.291335	0	0	0
LOC107764071	15.88071	17.79975	12.83822	4.248252	4.234939	3.846132	8.81907	9.76879	6.785303	2.309891	4.338972	2.736387	3.309698	2.557888	5.58203	13.2213	13.14704	10.70443	7.162066	5.33679	7.847972	12.65681	12.55802	6.063385	7.487248	15.16846	6.770814	19.01469	4.771189	13.45107
LOC107761711	0	0.034668	0	0	0.033659	0	0	0	0	0.370929	0.080019	0	0.169382	0.041499	0	0	0	0	0	0.064357	0	0	0	0	0.81758	0.105857	0.404632	0	0.183133	0
LOC107773314	0	0.117581	0.224299	0	0	0	0	0.110233	0	0	0	0	0	0	0	0	0	0	0.130915	0	0	0	0	0	0	0	0	0	0	0
LOC107785040	0.48693	0.57057	0.241873	0.693211	0.24621	0.299148	0.408409	0.237741	0.457151	0.067831	0.097553	0	0.077437	0.227664	0.214188	0.051837	0.336011	0.155583	0.423517	0.235377	0.444088	0.464741	0.55222	0.290822	0.087947	0	0.164432	9.949252	0	0
LOC107806454	0.197846	0.811406	0.257975	2.643769	3.763946	3.0311	2.198732	1.901757	2.461143	1.832787	0.867061	2.089324	13.26977	6.367292	1.472213	0.737176	1.290168	0.442508	0.752852	0.376569	0.992414	0.454373	1.003452	1.085641	0.062534	0.068822	0.409217	0	2.381254	0
LOC107829294	0	0	0	0	0	0	0	0	0	0	0	0	0	0	0	0	0	0	0	0	0	0	0	0	0	0	0	0	0	0
LOC107795692	1.246148	2.460706	2.347042	2.257894	2.793441	2.009643	4.564032	3.442668	1.949898	2.389799	3.32044	1.193151	2.774459	1.31417	3.879671	2.785893	1.685437	1.610364	1.264505	1.862342	1.36381	4.509661	2.528126	2.257621	1.733061	2.138507	0.687329	5.941148	1.59984	2.568374
LOC107762083	0	0	0	0	0	0	0	0	0	0	0	0	0	0	0	0	0	0	0	0	0	0	0	0	0	0	0	0	0	0
LOC107793595	0.018424	0.218287	0.064063	0	0	0	0	0	0	0	0	0	0	0	0	0.01373	0.035598	0.013736	0	0	0	0	0.032503	0	0	0	0	0	0	0
107781628	0	0	0	0	0	0	0	0	0	0	0	0	0	0	0	0	0	0	0	0	0	0	0	0	0	0	0	0	0	0
LOC107764015	0	0.195991	0.240348	0.055664	0	0.033029	0	0	0	0.359485	0.409294	0	0.034199	0.067031	0	0.068681	0	0.022904	0.65465	0.54575	0.084055	0	0	0.03211	0.116523	0	0.07262	0	0	0
LOC107812018	32.32935	32.7695	29.49937	45.81061	35.75892	37.20889	56.57861	51.12925	47.3767	44.96382	46.42859	43.7704	55.13773	57.93505	57.32396	31.19588	37.83982	28.69945	29.33577	32.87068	32.79251	48.07048	47.34491	41.15525	21.48512	23.71749	26.75973	18.52569	27.68685	16.01741
Novel01472	0.313443	0.222183	0.121097	0.22086	0.246538	0.486762	0.262898	0.297571	0.130788	0.543371	0.757041	1.029798	1.550795	0.911868	0.857891	0.259531	0.538332	0.77895	0.42408	0.117845	0.921121	0.349019	0.122879	0.436814	1.453052	0.387673	0.493952	4.981241	4.024066	0
LOC107788702	1.609289	2.50753	3.339359	1.693085	2.158975	2.567368	2.612477	2.395196	1.852036	5.062129	4.804947	2.192858	1.560324	1.529119	6.447076	2.475859	0.852582	2.051259	1.73835	3.55707	1.230973	1.864202	1.098967	0.705367	4.528694	5.056277	2.392902	0	1.749478	0
LOC107761199	3.392213	4.758615	3.787157	4.519372	3.501929	3.682799	4.100428	3.637207	3.653106	3.599704	4.663965	2.135329	3.331993	3.011385	6.246534	4.684015	3.694841	4.091092	2.969727	3.094644	1.698532	3.777234	3.960207	2.954617	2.606911	2.498833	1.336441	0	2.561776	4.11266
LOC107762047	20.84561	23.76121	22.79197	29.85231	31.92703	25.77649	28.95032	30.39989	26.4455	27.78421	27.32915	28.28728	31.52152	26.89686	30.18383	24.18138	25.92918	24.47863	35.05473	33.26972	36.08424	26.01444	28.59653	29.50306	32.25337	28.26564	34.30452	25.33863	36.24835	52.94412
LOC107811941	0.25386	0.192802	0.073558	1.303244	0.786212	1.501117	0.745231	0.939921	1.668344	0.495091	0.712027	0.643404	0.847801	0.784688	0.130277	0.094588	0.286125	0.157719	0.558132	0.823201	1.19621	0.318008	0.709084	0.840225	0	0	0.350049	0	0.814781	0
LOC107815639	0.19454	0.310273	0.169109	11.94046	14.63201	6.170036	19.53945	20.61126	21.00386	9.86443	17.29012	17.50353	6.172077	10.61165	10.43276	0.507399	0.046985	0.217556	17.0262	14.52304	11.84301	18.52101	19.13303	20.94326	10.8836	21.24897	25.52223	20.86849	7.024368	0
LOC107785452	11.67414	12.75249	10.75447	10.1115	7.570528	16.34514	9.017226	8.309331	8.340538	7.902612	9.121038	6.049317	7.537117	7.744511	7.559833	11.60454	12.11069	11.97697	6.807871	9.754029	4.135308	8.892669	8.813386	7.741394	2.645845	2.198183	3.758654	1.467252	2.469397	0
LOC107802760	11.72042	13.95225	12.1344	12.64629	9.817494	12.74247	19.85414	17.13112	17.03093	13.90087	16.39057	10.95867	20.08411	18.35352	18.99043	14.96569	12.78599	13.22984	8.8864	9.970373	9.758421	16.85411	15.8848	14.8998	8.699437	11.3149	6.809564	23.54427	8.400544	10.17825
LOC107766234	1.334016	1.358826	1.978196	1.386279	1.492891	1.434231	1.349258	1.240392	1.473451	1.147795	1.183022	1.624226	2.140213	2.311432	0.885952	0.614009	1.023437	0.555787	0.557393	0.896151	1.109294	1.048538	0.588345	0.697157	0.297636	0.109187	0.139121	0	0.37779	0
LOC107804061	0.048862	0.044531	0	0	0.043236	0	0.081965	0.041749	0.045874	0.333524	0	0.04128	0	0.106611	0.05015	0	0	0.072857	0.099163	0.289336	0	0.122417	0	0	0	0.067988	0	0	0	0
LOC107791686	1.489913	1.018404	1.133256	2.305884	1.757831	2.03567	1.83543	1.684102	2.928729	1.543657	2.187399	1.796298	1.330329	1.422244	1.401772	1.133416	0.644723	1.110794	0.645691	0.800856	0.707722	1.671982	1.36896	1.8168	0.215841	0.496682	0.11006	0	0	0
LOC107780107	0.232489	0.063566	0.343566	0.126374	0.102861	0.049991	0.194999	0.258238	0.240098	0.090682	0.146719	0.157131	0.155286	0.253634	0.071586	0.138601	0.336906	0.207996	0.117957	0.059001	0.12722	0.232989	0.225578	0.194398	0	0	0.027478	0	0.223857	0
LOC107761820	4.656526	5.557422	4.572537	8.526436	9.625169	6.317518	10.42534	9.357225	7.263122	7.808084	9.864581	6.317237	9.037209	8.829596	12.13798	6.453556	5.652998	5.767695	9.262738	10.20206	7.089286	11.02852	7.649694	6.412079	9.282566	12.34136	5.270681	6.166805	9.904326	22.8508
LOC107826846	2.3243	1.210465	1.154551	3.835379	3.011587	8.924667	3.690107	3.900963	7.092005	3.399729	2.328287	5.75064	2.495035	2.626258	1.107599	0.247439	1.20293	0.433216	1.853138	1.404427	2.725458	3.327572	2.855617	3.296986	1.049508	0.808523	2.649024	5.93644	0.399643	5.132678
LOC107811222	3.115876	2.366447	3.139473	3.635457	2.485596	3.299191	2.732211	2.137899	2.371283	1.104863	2.201406	1.356074	1.681763	1.287601	1.623252	2.744128	2.075212	2.569398	2.203662	1.857053	2.131298	3.33154	3.206477	3.207368	1.163918	0.853965	1.31127	0	1.250078	2.91908
LOC107783135	0	0	0	0	0	0	0	0	0	0	0	0	0	0	0	0	0	0	0	0	0	0	0	0	0	0	0	0	0	0
LOC107772697	2.051015	2.875765	1.782904	0.285864	0.279211	2.205092	0.396986	0.40441	0.592487	0.76923	0.553143	0	0.175632	0.516359	0.485794	0.940565	1.829033	2.35249	1.44085	0.400389	0	0.395274	0.834983	0.659606	0.19947	0.439052	0.186472	0	0	0
LOC107781483	0.423944	0.296679	0.342202	0.267478	0.381832	0.358122	0.36193	0.355761	0.255868	0.479838	0.557381	0.422116	0.53936	0.429466	0.466205	0.394904	0.380311	0.400729	0.253504	0.300991	0.248557	0.569003	0.340558	0.284854	0.306282	0.28441	0.268429	0.541392	0.291574	0
LOC107826659	4.404726	4.525748	3.828934	4.549631	4.518226	4.072029	5.976981	5.657261	4.635807	4.568741	6.098502	5.925533	10.5876	7.651814	6.162232	4.829528	4.96004	4.015992	3.615545	3.417565	3.863661	4.10024	4.330703	4.22261	1.738068	1.756678	1.226899	4.012741	6.753477	3.469437
LOC107822044	0	0	0	0	0	0	0	0	0	0	0	0	0	0	0	0	0	0	0	0	0	0	0	0	0	0	0	0	0	0
LOC107823115	1.270752	1.956116	1.374061	3.229078	3.620163	2.299237	3.02203	2.290699	2.626138	2.092943	2.037466	1.767457	2.441079	2.662763	2.624435	2.257705	1.901365	2.195193	2.468865	2.91028	2.04229	3.423139	2.091411	1.959889	1.312723	1.121271	0.924967	1.108253	1.566767	2.395503
LOC107802234	1.097481	1.682185	1.214203	0.768293	0.926981	1.179926	45.23024	30.04958	17.89085	2.967324	22.77181	1.601497	38.59559	14.31315	15.30913	1.226766	1.060259	1.041369	0.455583	0.337598	0.318475	26.62117	4.092214	0.625684	0.189211	0	0.058961	0	0.240166	3.084495

LOC107759068	0	0	0	0	0	0	0	0	0	0	0	0	0	0	0	0	0	0	0	0	0	0	0	0	0	0	0	0	0	0
LOC107804669	0	0	0	0	0	0	0	0	0	0	0	0	0	0	0	0	0	0	0	0	0	0	0	0	0	0	0	0	0	0
LOC107813805	8.891626	12.81845	8.03379	25.16691	90.86228	25.5212	14.75738	23.57449	8.271961	43.66758	27.98109	33.55312	49.67138	39.09526	67.5333	16.38442	11.29723	12.33412	191.334	118.7745	167.3222	43.45118	16.51784	20.13597	183.4103	165.3356	191.0124	36.6111	111.0399	63.3083
LOC107809080	3.960269	4.844056	5.707431	4.890741	5.108909	5.154146	4.073389	4.772884	5.224873	6.727596	7.103127	5.212318	5.963247	6.662601	7.31648	7.12947	3.705133	4.957173	4.610823	5.465977	3.003523	5.605043	3.897697	3.659987	3.452183	1.450112	4.237282	1.490606	6.92402	2.577572
LOC107785192	7.883024	8.460972	8.26836	6.07923	6.312465	7.949891	7.103621	5.009853	5.810648	5.939911	5.801676	6.879956	6.889849	6.716522	5.282501	7.500833	8.433876	7.747124	3.867357	5.428486	4.337437	4.679053	4.740919	5.787962	1.976834	1.72235	2.618028	2.329536	1.881902	0
LOC107824768	4.578027	6.773226	6.356994	7.917879	7.996659	7.862318	6.781964	6.857983	5.302793	6.145459	7.212337	6.630247	4.897765	5.253852	5.675127	5.006562	5.571527	6.604596	9.954569	9.00276	6.832207	6.107237	7.237154	9.259193	9.396133	13.07089	6.464923	4.251866	5.72474	14.70474
LOC107787434	0.441558	0.469496	0.447809	0.416697	0.667479	0.55385	0.462942	0.550198	0.639104	0.412634	0.567637	0.575103	0.286736	0.341217	0.509853	0.589546	0.58655	0.61725	0.541408	0.5136	0.520164	0.583864	0.470625	0.557665	0.628046	0.383997	0.413159	0	0.177151	0
LOC107810691	0	0	0	0	0	0	0	0	0	0	0	0	0	0	0	0	0	0	0	0	0	0	0	0	0	0	0	0	0	0
LOC107829693	10.9606	4.960643	4.018537	15.50257	12.5027	21.88455	53.50152	43.28941	55.0216	3.417243	4.7316	4.346448	14.85761	13.58435	7.231927	4.778473	7.959658	5.030813	5.977164	8.199655	7.854201	48.19591	38.14576	31.21181	0.801286	0.881854	1.189704	0	2.153806	4.610277
LOC107816392	0	0	0	0	0	0	0	0	0	0	0	0	0	0	0	0	0	0	0	0	0	0	0	0	0	0	0	0	0	0
107769750	0	0	0	0	0	0	0	0	0	0.709401	0.510121	0	0	0	0	0	0	0	0	0.615412	0	0	0	0	0	0	0	0	0	0
107806043	0	0	0	0	0	0	0	0	0	0	0	0	0	0	0	0	0	0	0	0	0	0	0	0	0	0	0	0	0	0
LOC107798369	0.09718	0.531403	0.42238	0.880397	0.687927	0.417919	0.407543	0.58123	0.456182	0.663336	0.817708	0.4926	1.406361	1.166199	0.598454	0.362091	0.281651	0.579612	0.690277	0.57545	0.443147	0.649257	0.342875	0.406288	0.614322	0.135218	0.229717	0	0	0
LOC107787473	0	0	0.215794	0.374829	0.146443	0.088965	0	0	0.077688	0	0.116047	0.069908	0.092117	0	0.084931	0.246657	0.079942	0.123385	0	0	0.075468	0	0.145979	0.259466	0	0.230277	0.097802	0	0.39838	0
LOC107775612	0	0	0	0	0	0	0	0	0	0	0	0	0	0	0	0	0	0	0	0	0	0	0	0	0	0	0	0	0	0
LOC107765160	0	0	0	0	0	0	0	0	0	0	0	0	0	0	0	0	0	0	0	0	0	0	0	0	0	0	0	0	0	0
LOC107772063	0	0	0	0	0	0	0	0	0	0	0	0	0	0	0	0	0	0	0	0	0	0	0	0	0	0	0	0	0	0
Novel00257	1.145482	2.420102	1.538876	0.613215	0.829307	0.559788	0.480382	0.756292	0.733247	0.203089	0.328587	0.21994	0.347773	0.113606	0.053441	0.349206	0.251507	0.155274	0.105669	0.044045	0	0	0.045927	0	0	0.072448	0	0	0	0
LOC107764116	5.209746	5.192439	5.086446	5.78927	5.495577	6.208131	5.768784	4.626771	5.421213	5.68077	6.784286	4.097798	7.113748	6.075548	4.214495	6.655387	5.925614	6.811502	4.843779	4.906412	2.878913	5.465284	5.432915	5.311106	3.342022	2.856741	2.608596	12.84726	4.447963	3.17366
LOC107820170	6.347821	7.390198	6.30123	11.16769	9.494491	9.158285	9.92704	9.832735	9.073993	11.02206	11.22825	12.10958	11.07842	10.76751	12.18978	7.599165	7.833811	6.86986	10.63858	11.15537	11.50394	8.310962	10.98165	8.560956	12.78914	12.65046	15.39246	20.50137	8.280956	10.12891
LOC107831560	2.372602	2.009862	2.379753	2.989951	2.637751	3.074087	3.099814	2.63798	4.440729	2.120788	2.367531	1.927347	3.081416	3.15253	2.638105	2.538762	1.895418	2.619297	1.743933	2.45736	1.068056	3.670712	3.42091	3.211079	0.538406	1.015786	0.862839	0	0.658993	0.940395
LOC107782825	0.257124	0.133907	0.239477	0.549075	0.487543	0.45415	1.155325	1.192621	0.293127	1.540186	2.1893	1.039578	0.470241	0.581055	1.300673	0.273727	0.035486	0.109541	1.602741	1.631317	2.14401	0.904936	1.0692	0.403118	3.877769	3.935457	3.950693	1.313427	0.972624	1.135596
LOC107818254	0	0	0	0	0	0	0	0	0	0	0	0	0	0	0	0	0	0	0	0	0	0	0	0	0	0	0	0	0	0
Novel04908	2.535948	3.018161	2.24767	2.7299	3.097564	2.202547	2.660847	2.200772	1.970045	3.306857	3.939952	1.881986	3.653363	3.200571	3.378572	3.979679	2.805421	3.544055	3.612718	3.979206	1.750493	3.014803	2.035117	1.985331	2.187766	3.237997	2.315551	2.844802	4.021774	6.763986
LOC107787490	0	0	0	0	0	0	0	0	0	0	0	0	0	0	0	0	0	0	0	0	0	0	0	0	0	0	0	0	0	0
LOC107809906	0.351542	0.106796	0.305588	0	0	0	0	0.100122	0	0	0.082167	0.197995	0.130447	0.127838	0.120271	0.26197	0.226413	0.349453	0.118907	0	0	0.097861	0.103361	0.122477	0.148152	0	0	0	0	0
Novel05082	0.956985	1.656025	1.0109	2.337552	2.85126	2.083798	3.088733	3.105089	2.797734	2.929479	3.898817	2.947396	4.032066	4.070369	2.983968	1.462401	1.2171	1.625634	2.8272	2.408134	2.032818	1.942366	2.104967	1.607981	2.909933	1.972078	2.262157	5.197817	3.907427	6.741091
LOC107830224	25.25015	16.38423	21.66528	7.106907	7.01382	12.91459	8.841463	8.168953	11.04743	6.215284	6.875892	8.00814	10.00638	12.03495	7.296755	13.15324	24.78849	14.80417	3.482617	4.285803	4.695144	6.005376	10.16309	7.430602	1.859643	1.59182	3.573511	0	0.786817	0
Novel02028	0	0	0	0.115458	0.056386	0	0.427573	0.163338	0.239301	0.124275	0.178729	0.592178	0.354683	0.34759	0.261611	0	0	0	0.193983	0	0.116232	0.053216	0.056207	0	0.241693	1.329975	1.054405	0	0	0
LOC107778765	0.521748	0.560421	0.388752	0.675253	0.824423	0.841414	0.593903	0.700536	0.962186	1.072051	1.319673	0.755635	0.912712	1.138403	0.631134	0.569325	0.28803	0.791863	0.605067	0.504415	0.356882	0.684711	0.558834	0.759568	0.471177	0.544481	1.409518	0	0.17942	1.152159
LOC107759221	0	0.02586	0	0.025706	0.050216	0.061013	0.023799	0.024244	0.053279	0.055338	0	0.047944	0.094761	0.030955	0.029123	0.021145	0	0.021155	0.028793	0.024003	0	0.023696	0.025028	0.029657	0	0	0.033537	0	0	0
LOC107809011	0	0	0	0.019298	0.018849	0	0	0	0	0	0	0.053988	0	0	0.021863	0	0	0	0	0	0	0.035578	0	0	0	0	0	0	0	0
LOC107816765	0.410178	0.510897	0.689348	0.545014	0.411346	0.499791	0.447244	0.537383	0.706005	0.199988	0.402664	0.161714	0.136985	0.134245	0.224531	0.509442	0.488729	0.560643	0.263606	0.416379	0.224454	0.776448	0.747729	0.528752	0.069145	0.152195	0.193919	0	0.065825	0
LOC107768606	3.908937	3.829703	4.204971	4.11676	4.193898	5.463361	3.196629	3.966134	3.945124	4.478756	5.070756	2.187826	4.188303	4.051236	3.911726	4.733535	4.862066	4.444275	2.627819	3.265358	1.292319	3.468484	3.146246	3.370637	2.656371	2.1756	4.215795	0	1.176189	0
LOC107810995	0	0	0	0	0	0	0	0	0	0	0	0	0	0	0	0	0	0	0	0	0	0	0	0	0	0	0	0	0	0
LOC107830876	2.916932	2.86097	2.801264	0.4656	0.331855	0.16427	0.186404	0.237362	0.169529	0.135447	0.409071	0.093878	0.077314	0.378837	0.057026	2.070191	1.98602	2.640703	0.140948	0.047	0.012668	0.371201	0.355309	0.29036	0	0.019327	0.032834	0	0	0
LOC107767850	0	0	0	0	0.116163	0.141139	0	0.224334	0	0	0	0	0	0	0	0	0	0	0	0	0	0.109633	0	0	0	0	0	0	0	0
LOC107790032	0.112645	0.410647	0.783357	0.510252	1.594806	0.726641	0.56688	0.86622	0.846046	0.549214	0.236959	0.761323	2.257157	1.843347	1.271769	0.167886	0.326473	0.335925	0.914432	0.381158	1.027338	1.222942	0.59616	0.470945	0.142417	0	0.133137	0	0.542311	0
LOC107826200	0	0	0	0	0	0	0	0	0	0	0	0	0.099335	0	0	0	0	0	0	0	0	0	0	0	0	0	0	0	0	0
LOC107815530	38.5557	32.74657	33.16072	32.07597	24.82617	48.85202	15.36193	13.7055	15.82627	11.03443	9.570736	12.4674	13.18141	16.7995	6.226236	26.34107	31.33119	23.61345	19.53224	28.37201	17.07635	15.44182	27.73174	13.16862	1.548667	1.623221	3.929615	4.171374	2.246546	3.606592
LOC107770583	12.75507	9.401775	5.283174	27.92869	28.02172	26.3948	27.06417	22.17236	20.83429	23.86187	17.80362	25.60529	20.69778	20.54558	12.64003	9.654077	13.28818	11.05958	25.11503	24.624	35.00792	16.09501	15.05987	18.59737	11.82931	12.57364	24.9525	2.859461	8.662494	7.416915
LOC107803424	5.175652	6.272827	7.891064	11.07164	13.93109	9.060453	12.22765	11.78486	9.540176	11.0715	10.10847	10.11864	11.61371	10.67196	10.73554	8.319599	7.408547	7.858753	14.27085	12.90551	13.74071	10.34189	8.939318	9.856215	14.59462	16.06208	12.29846	13.56506	11.21937	23.45684
LOC107771577	2.397095	2.952238	2.196378	1.02713	0.573272	1.253758	0.733578	0.941039	1.155661	1.010798	1.15842	1.094666	0.68515	0.56543	0.864434	3.282963	4.068289	3.936529	0.690277	0.739865	0.620406	1.027991	1.657229	1.52358	0.08191	0.090145	0.459433	0	0	0
LOC107831508	0	0	0	0	0	0	0	0	0	0	0	0	0	0	0	0	0	0	0	0	0	0	0	0	0	0	0	0	0	0
LOC107804859	3.273735	2.433994	2.658566	2.965838	2.09638	2.222947	1.806462	2.502729	2.426468	1.932185	2.053909	1.928741	1.822184	1.832735	2.077939	2.439591	3.204318	2.697625	1.22388	1.238922	2.317847	1.762699	1.671798	1.980988	0.05446	0.359618	0.30547	6.161009	1.244284	0
LOC107808417	4.387411	5.15155	3.470712	5.039588	5.485988	4.06106	5.892372	5.013629	4.56554	5.826859	7.184686	4.313004	7.011568	6.411297	8.591839	6.091038	3.709865	5.284439	4.597711	5.252192	2.553036	6.01682	4.526855	3.722967	5.172682	6.316993	3.563089	3.849839	6.868071	5.547653
LOC107822281	0	0	0	0	0	0	0	0	0	0	0	0	0	0	0	0	0	0	0	0	0	0	0	0	0	0	0	0	0	0
LOC107817277	0	0	0	0	0	0	0	0	0	0	0	0	0	0	0	0	0	0	0	0	0	0	0	0	0	0	0	0	0	0
LOC107799602	6.687389	7.219509	6.387041	8.112583	6.171409	7.066331	3.394288	3.604898	4.338304	5.093695	3.803687	6.207387	3.929926	3.913955	3.446605	4.833733	6.044663	5.349498	7.688745	6.652521	9.292485	3.954911	4.6329	4.949757	7.874299	3.873761	13.73864	4.105089	4.974407	1.774641
LOC107793749	8.714454	9.4684	8.734498	9.243441	9.165533	6.901795	10.01437	8.505776	9.957571	13.69913	12.74306	10.29662	15.50097	13.12716	11.26788	9.590815	9.497473	10.28884	6.954678	6.925846	7.070933	10.72226	10.12131	10.69663	12.50766	7.07682	8.760314	11.08909	20.90259	17.25783
LOC107776804	15.34357	15.97284	17.59215	15.69797	10.47536	14.07866	11.87083	12.7144	13.22548	7.867904	9.089431	9.889624	9.791937	9.379655	11.06448	14.83469	16.16493	16.41931	10.46921	8.727663	8.082505	12.37196	15.16746	15.06934	3.010179	2.944752	5.706228	4.729664	2.54722	0
LOC107819826	0	0	0	0	0	0	0	0	0	0	0	0	0	0	0	0	0	0	0	0	0	0	0	0	0	0	0	0	0	0
LOC107789377	4.122373	6.34663	5.52708	0.907291	0.556436	1.903029	0.566501	0.676594	0.699709	0.635906	0.555259	0.452554	0.362978	0.406536	0.095618	5.172064	3.667555	4.21942	0.5672	0.669866	0.531027	1.264269	1.294243	1.31452	0.058892	0	0.055054	0	0	0
LOC107783098	0	0	0	0	0	0	0	0	0	0	0	0	0	0	0	0	0	0	0	0	0	0	0	0	0	0	0	0	0	0
Novel04789	3.007686	4.972989	3.214571	5.285836	5.119193	4.329178	3.156791	2.569849	1.681905	2.067436	2.489301	2.263275	2.872498	3.855006	0.691624	11.2679	8.653522	7.425513	4.419552	8.522695	3.717372	2.97847	7.915443	4.50072	1.766249	1.692285	1.825985	5.876833	2.927665	2.032457
LOC107791100	3.325177	1.830295	2.117796	2.32644	1.71879	2.796265	0.717952	0.618858	0.896365	0.70628	0.623303	0.50065	1.136145	1.652176	0.40549	0.613348	1.01779	0.343632	0.367483	0.640555	0.570492	0.384923	0.958315	0.447338	0.041624	0	0	0	0	0
LOC107823039	79.20455	68.42403	89.27152	53.34914	47.61312	76.4514	47.66082	52.34943	59.90583	30.02702	35.39313	46.93381	37.45968	37.40318	21.01572	55.11998	97.21959	79.28604	34.30679	44.84676	52.40359	47.72041	60.76317	72.16691	16.05429	12.1471	32.26755	45.40477	22.16082	19.6286
LOC107790179	0.305421	0.092785	0.088499	0	0.045043	0.164183	0	0.043493	0.047791	0	0.035694	0.043005	0	0.055533	0.104492	0	0.049177	0.037951	0.051653	0	0.046425	0	0.0449	0	0	0.060164	0	0	0	0
LOC107765631	2.036701	2.570125	1.929348	2.223639	1.432523	1.572094	2.606205	2.364904	1.8386	2.138813	3.094294	1.654482	1.947538	1.481293	2.010013	2.665795	2.144206	2.375006	1.245321	1.590377	1.571735	2.573159	1.404959	1.637507	0.462181	0.326991	0.432064	0	1.257103	0
LOC107810647	17.83268	22.16718	23.39991	15.63086	13.28803	12.55969	18.87635	18.37621	23.51019	13.51405	12.37827	10.07182	14.16065	15.29352	19.2458	25.64035	16.08964	23.46355	11.02339	11.183	10.30824	18.66154	15.30686	14.6939	7.548974	15.28229	6.585024	8.568571	8.171887	8.643171
LOC107788679	0	0	0	0	0	0	0	0	0	0	0	0	0	0	0	0	0	0	0	0	0	0	0	0	0	0	0	0	0	0
LOC107771553	0	0	0	0	0	0	0	0	0	0	0	0	0	0	0	0	0	0	0	0	0	0	0	0	0	0	0	0	0	0
Novel01962	1.05886	1.088214	0.90086	0.306151	0.199351	0.169549	0.359024	0.211743	0.274965	0.175748	0.552905	0.209364	0.200636	0.24578	0.184985	0.537235	0.435297	0.520684	0.205747	0.057174	0.184921	0.056443	0.317952	0.188378	0.142417	0.031347	0.159764	0	0	0
LOC107767115	0	0.053398	0	0	0	0	0	0	0	0	0	0	0	0	0	0.043662	0	0	0	0	0	0	0	0	0	0	0	0	0	0
LOC107801988	4.655979	5.62244	4.097935	5.325327	4.583407	5.381178	5.760126	5.768382	4.863002	5.334697	5.999024	5.064382	7.191552	7.873138	4.599565	4.423792	5.735035	4.122085	4.488337	4.578666	3.71554	4.811808	3.798865	3.893142	5.886573	1.943536	6.259654	8.324208	10.08699	10.79573
LOC107782815	0	0	0	0	0	0	0	0	0	0	0	0	0	0	0	0	0	0	0	0	0	0	0	0	0	0	0	0	0	0
Novel00212	2.261375	3.498847	2.925791	1.858115	2.838647	1.470061	2.029042	1.617639	1.629339	1.179486	0.516267	1.199599	1.287969	1.032718	0.755679	1.80275	2.489518	1.803576	2.187958	3.381059	0.815372	1.185823	2.087458	1.703983	0.19947	0	0.559416	0	0.759563	0
LOC107779038	7.302079	7.346795	6.221848	11.00369	9.37217	11.11524	7.943746	6.269981	6.821575	8.706687	8.99841	9.009182	11.46882	8.873252	8.2738	5.468456	7.124268	5.039751	8.216636	8.011825	8.769597	6.611368	6.600345	6.460863	10.37463	7.192694	10.33947	0	9.223786	6.705423
LOC107806509	0.490412	0.292237	0.31153	1.110721	0.650923	0.648926	1.771881	1.514923	0.903132	0.625356	1.547447	0.382443	0.713913	0.699635	1.645552	0.843359	0.492001	0.393748	0.478496	0.574414	0.481669	2.016275	1.74694	1.182873	0.286168	0.341186	0.06688	0	0.181617	0
LOC107777619	0.355744	0.272342	0.284502	0.18048	0.138506	0.168286	0.238702	0.243166	0.227112	0.152634	0.119735	0.060109	0.031682	0.046572	0.102235	0.212081	0.082483	0.159133	0.129954	0.132411	0.051911	0.237673	0.125516	0.104111	0	0.039599	0.033637	0	0	0
LOC107768673	0	0	0	0	0	0	0	0	0	0	0	0	0	0	0	0	0	0	0	0	0	0	0	0	0	0	0	0	0	0
LOC107794008	2.717376	2.505019	5.29449	14.96891	23.49237	9.973435	8.566574	12.54306	9.237075	21.86834	9.614734	9.948097	13.35182	8.96172	13.36816	22.50767	1.267344	8.755696	34.29325	26.42199	44.40986	11.50326	12.34268	20.37109	14.69011	19.47013	19.82406	22.33687	32.63085	23.1751
LOC107816739	0.174405	0.052983	0	0	0	0	0	0	0	0	0	0	0	0	0	0.779803	0.056163	0.043342	0.058992	0	0	0	0	0	0	0	0	0	0	0
Novel05026	3.371132	3.99988	2.681642	4.955669	4.502641	2.974726	5.388281	4.537968	4.060713	5.799257	6.177204	3.251049	5.841609	4.822696	4.830996	4.716247	3.07245	5.145197	4.647114	4.681168	2.755474	4.96665	4.404237	4.720169	3.377542	2.920615	3.420576	2.274374	6.736911	3.932871
LOC107798607	0	0	0	0	0	0	0	0	0	0	0	0	0	0	0	0	0	0	0	0	0	0	0	0	0	0	0	0	0	0
LOC107811707	0.988297	0.504398	0.309278	0.573022	0.524706	1.020039	0.198943	0.033777	0.111343	0.077097	0.221758	0.100193	0.088015	0	0.081149	0.530266	0.305529	0.117891	0.240685	0.066883	0.144215	0.627268	0.104609	0.702418	0	0	0	0	0	0
LOC107809988	0.145803	0.398645	0.253487	0.990677	0.870859	0.705403	0.42802	0.591746	0.581767	0.959691	0.869016	1.786088	0.97386	1.272512	0.748241	0.380284	0.281716	0.407634	0.48084	0.555027	0.731362	0.152205	0.16076	0.380983	1.198206	0.304312	0.430819	0	2.632305	0
LOC107826354	0	0	0	0	0	0	0	0	0	0	0	0	0	0	0	0	0	0	0	0	0	0	0	0	0	0	0	0	0	0
LOC107785181	0	0	0	0	0	0	0	0	0	0	0	0	0	0	0	0	0	0	0	0	0	0	0	0	0	0	0	0	0	0
LOC107793696	6.888484	6.464479	6.877322	4.69591	3.319269	3.739645	5.034008	4.137478	4.930449	3.059292	3.730261	3.16908	3.720292	5.134008	2.730058	5.743204	5.534731	5.491594	3.183545	2.538575	3.794329	3.702267	3.790042	4.704847	1.379665	1.613287	1.854036	0	1.313411	0
LOC107810977	0	0	0	0	0	0	0	0	0	0	0	0	0	0	0	0	0	0	0	0	0	0	0	0	0	0	0	0	0	0
LOC107764413	1.298988	1.114228	2.158731	0.796082	1.217043	1.191192	1.474052	0.816096	1.398892	0.819614	0.723323	0.322771	0.637964	0.583525	0.352918	0.939536	0.959653	0.996934	0.69783	0.614066	0.452973	1.467696	1.213192	1.317769	0.386427	0.05316	0.270935	0	0	0
Novel03394	0	0.090327	0	0.269367	0.570045	0.266389	0.581896	0.33873	0.232623	1.691287	1.459419	2.302605	1.323971	1.513742	0.864654	0.110786	0.287246	0.147782	0.754277	0.293442	0.903902	0.08277	0.349687	0.258975	0.250611	0.413714	0.819982	0	0.477152	0
LOC107830960	6.493865	27.13511	10.33138	19.81388	11.27556	2.700469	357.3744	251.7255	169.7306	19.35545	94.33482	5.330928	192.7295	68.773	75.39137	9.267593	2.781699	4.293363	3.792086	6.581671	3.184748	263.5367	91.16195	3.777885	1.549098	1.363885	1.086117	4.381162	0.884824	3.787976
LOC107832159	0	0	0	0	0.075283	0	0.118931	0.121155	0.1065	0.082962	0	0.047918	0.12628	0.247509	0.145536	0	0.027398	0	0	0	0.129321	0.023684	0.025015	0.059282	0.035855	0.07892	0	0	0.136532	0
LOC107764383	0	0	0	0	0	0	0	0	0	0	0	0	0	0	0	0	0	0	0	0	0	0	0	0	0	0	0	0	0	0
LOC107766598	0	0	0	0	0	0	0	0	0	0	0	0	0	0	0	0	0	0	0	0	0	0	0	0	0	0	0	0	0	0
LOC107791285	43.683	38.42007	39.76169	37.28912	32.72044	39.11334	34.52289	31.99195	41.38549	22.20453	23.18239	32.58598	29.56141	30.3463	21.3273	32.99787	38.80224	40.38769	22.56744	21.7898	32.87677	30.38219	34.84212	49.95952	7.134392	7.389872	12.78975	28.48588	11.50608	12.31452
Novel04183	2.177672	2.529149	1.675225	1.693518	2.097474	1.740409	2.0528	1.82773	1.555987	1.973169	2.243182	0.879176	1.373007	1.408621	1.701052	2.168526	1.638369	1.810327	1.8382	2.575765	0.89637	1.866913	1.529881	1.36969	0.828408	1.126221	0.819982	2.75636	1.020575	1.191582
LOC107822352	0	0	0	0	0	0	0	0	0	0	0	0	0	0	0	0	0	0	0	0	0	0	0	0	0	0	0	0	0	0
LOC107796176	0.123464	0.618874	0.134156	0.055926	0	0.099554	0.025889	0.026373	0	0.060197	0	0	0	0.033673	0.03168	0.805051	0.059638	0.092048	0.187925	0.287217	0.112602	0.077331	0.108904	0	0.039024	0.042948	0.072962	0	0.743002	0
LOC107777981	0	0	0	0	0	0	0	0	0	0	0	0	0	0	0	0	0	0	0	0	0	0	0	0	0	0	0	0	0	0
LOC107797033	5.680391	5.923655	4.415572	14.84465	12.51763	16.32479	22.08111	23.19404	22.51142	7.616261	8.847064	7.013853	8.26918	9.772236	8.072582	5.4134	6.701369	4.397856	10.36424	10.48832	12.60284	17.4245	23.75161	22.32138	2.002601	2.659949	4.131548	3.906041	3.418428	3.377184
LOC107793194	3.55558	4.473433	3.678794	4.965324	3.898232	4.158333	5.848057	5.112705	4.08718	4.312783	6.044429	2.945241	5.34829	4.257398	5.660942	4.032596	3.485257	3.775824	4.065551	4.357607	2.151287	5.982169	4.283667	3.951958	2.609486	2.413332	1.680964	1.240362	3.006057	7.506966
LOC107817291	3.224642	1.695498	1.976554	0.636707	0.84138	1.333417	0.797524	0.777114	0.621013	2.378481	3.768535	2.514708	3.865848	2.074674	1.569978	2.064121	2.356426	2.712326	0.377555	1.293968	0.71638	0.483356	1.09398	0.907414	0.62722	0.46019	3.224919	0	0.199033	0
LOC107802148	0	0	0.139457	0.07267	0	0	0	0	0	0	0	0.056246	0	0	0	0	0	0	0	0	0	0.066989	0	0	0	0	0	0	0	0
LOC107830486	5.219112	5.186388	5.630083	4.699768	3.783597	5.070347	3.876455	4.056397	4.988485	3.096498	3.527368	3.745207	3.114994	2.949797	3.162421	4.967043	4.768734	5.719403	2.041829	2.579855	2.465982	3.308348	4.132149	6.375137	0.834756	0.7437	1.783683	4.496871	0.756827	1.944009
LOC107810085	0.177759	0.324011	0.103015	0.161041	0.262155	0.191113	0.397585	0.20251	0.055629	0.231117	0.415483	0.100117	0.131923	0	0.243263	0.441555	0.343461	0.265054	0.60126	0.400993	0	0.098968	0.679447	0.247725	0.299656	0.494678	0.210097	0	0.570531	0
LOC107763705	4.779175	5.644895	5.051794	3.22129	2.097549	3.90503	4.200908	3.658778	4.558691	2.274226	3.7533	3.326966	3.064461	3.461993	2.864645	4.045804	5.171135	4.104666	1.668254	2.005255	1.499389	3.38455	3.709683	4.036116	0.290032	0.425592	1.084531	0	0.736277	0
LOC107777025	1.237874	1.015353	1.199036	1.361762	1.392662	1.540002	1.601884	1.888702	1.710051	1.396768	1.438397	1.07567	1.417391	0.906737	1.90578	0.962	1.31548	1.4107	1.435553	1.959678	1.741827	2.362928	1.856208	2.421302	1.475618	1.870047	1.274959	0	1.106777	3.28027
Novel04124	12.45534	11.65831	10.30044	12.86976	10.42556	10.20613	10.22095	8.226119	7.901097	7.287319	10.19718	4.265927	6.745359	5.875964	7.393886	13.29546	9.821454	12.39805	8.676379	9.454249	4.666598	11.13272	10.33319	12.52573	3.40483	2.154627	2.864668	4.814773	3.241322	0
LOC107761201	14.47021	15.4766	15.10823	6.247671	6.948832	8.700056	2.57447	1.702994	2.020949	0.427584	0.531085	0.202063	1.996914	0.913256	0.572798	8.614691	9.319625	14.94887	2.912403	3.102352	1.854134	1.065296	2.672286	1.916573	0	0	0.471147	0	0	0
LOC107779005	2.909987	3.140638	3.095419	4.440085	3.964064	5.351538	3.211488	3.075249	3.630716	2.576246	2.953333	2.393742	3.239438	2.965794	1.768459	1.94026	3.033236	2.85463	3.924187	4.340275	3.492049	2.686017	3.546238	2.801398	1.210233	1.225366	1.719685	2.738226	1.843383	0
LOC107759315	2.51695	1.23994	2.838397	0.55465	0.060194	0.804495	2.111074	3.952362	7.79159	0.199001	0.476996	0.17241	2.120359	2.894294	0.279279	1.216627	2.037274	1.775061	0.069028	0.057545	1.488973	2.38602	11.5806	5.616932	0	0	0.080401	0	0	0
Novel03025	2.446076	3.843615	3.116161	4.852321	6.456028	3.355301	7.286821	7.098771	4.038644	3.989096	7.540921	2.173401	4.366199	4.324889	13.15893	7.228418	3.381699	3.898845	7.103966	7.599002	2.769346	13.45418	5.431202	2.909277	9.917595	21.65353	2.492297	6.032035	3.857747	2.607665
LOC107806607	33.23997	26.97155	32.25033	29.85469	28.14784	32.39389	23.78776	21.97271	24.76438	21.80047	24.94947	23.55269	23.53483	19.63281	19.60787	30.35048	31.85363	34.52133	28.83339	25.73015	19.00418	18.67055	23.47165	25.96341	7.27537	11.58761	10.43428	11.50223	12.13123	17.67981
LOC107820199	0.123829	0.338565	0.107642	0.149577	0.219144	0.088754	0.484684	0.740621	0.426273	0.160999	0.173658	0.278972	0.459495	0.675458	0.59311	0.246074	0	0.09232	0	0.139668	0.150579	0.413652	0.436902	0.431421	0	0.287166	0.048785	0	0	0
LOC107821580	0.266502	0.485767	0.315906	0.153642	0.085752	0.390712	0.447053	0.269108	0.386679	0.023625	0.220848	0.122808	0.02697	0.185017	0.074599	0.343032	0.421304	0.469628	0.245843	0.184453	0.088383	0.566523	0.406034	0.481128	0.030631	0	0.11454	0	0	0
LOC107794452	47.70078	33.62394	43.37297	2.775931	2.56477	9.393194	3.472977	5.802189	7.502807	0.767163	3.368009	2.02894	1.428938	3.794525	1.784955	60.69472	34.92225	59.14811	1.974794	1.996562	3.776381	6.812261	6.20902	16.44583	0	0	1.37031	0	0	0
LOC107794986	8.075585	9.691213	8.881575	10.73832	9.856742	6.219875	11.2889	9.809943	9.606298	10.28209	10.62651	8.908172	10.41366	10.04315	13.05105	12.0802	8.290198	9.60206	9.733853	11.285	7.134487	11.84881	10.25846	9.54425	8.70576	11.58838	6.80257	11.69927	9.880785	13.79352
800481	0	0	0.632397	0	0	0	0	0	0	0	0	0	0	0	0	0	0	0	0	0	0	0	0	0	0	0	0	0	0	0
LOC107800543	0.572144	0.495367	0.571955	0.69975	0.480957	2.152936	0.095976	0.097771	0.188004	0.223165	0.280831	0.26585	0.477689	0.343299	0	0.149227	0.221096	0.533197	0.638629	1.137387	0.443534	0.023891	0.252334	0.239202	0	0.039805	0.135246	0	0.137725	0
LOC107809787	0.965291	1.435372	0.75078	1.380798	1.258752	1.802506	1.7471	1.649542	0.85856	0.792658	1.282477	0.686741	1.131133	1.053086	0.78217	1.514392	0.834389	1.098437	1.44349	1.289321	1.52905	1.400138	1.658089	2.655059	1.541585	1.060368	1.140897	0	2.935109	6.282676
LOC107782770	0	0	0	0	0	0	0	0	0	0	0	0	0	0	0	0	0	0	0	0	0	0	0	0	0	0	0	0	0	0
LOC107826739	5.428613	6.8504	6.234102	10.93813	14.23406	8.1788	14.86266	14.25635	11.86371	11.1183	12.06892	9.67866	13.35973	10.39878	15.2432	8.104347	7.20899	7.417723	14.53602	13.147	14.12439	14.98051	12.82778	11.00639	11.45433	12.12605	8.991241	9.088823	14.94693	20.20692
LOC107780566	1.869422	1.605454	1.781303	8.630854	11.73837	7.073151	20.50415	26.69309	21.22991	11.63866	10.58755	12.81695	10.64546	19.96305	22.98778	1.660098	2.709028	2.251415	25.13472	17.36501	21.3118	22.4573	19.08981	13.79016	70.40555	89.23994	61.86617	56.56119	34.26947	66.68606
LOC107828808	6.320748	7.062975	7.692289	3.385973	1.945396	4.845542	3.918488	3.146447	3.973332	1.715068	2.428027	2.135977	2.019125	2.338516	1.579551	4.505436	3.87622	3.44209	1.171226	1.813303	0.952417	2.019647	3.005823	3.217051	0.06949	0.229431	0.714581	0	0	0
LOC107795053	37.28111	41.02343	38.56549	14.22869	10.53062	17.32089	12.59598	11.34431	15.65741	8.407555	10.18981	7.386712	7.074692	8.611306	8.433936	30.07447	31.5586	35.67131	8.502575	8.38939	9.967721	11.89937	15.67454	20.09658	1.791219	2.027646	5.358401	0	1.364161	5.005749
LOC107792220	0	0	0	0.105687	0.103227	0.062711	0.097847	0	0	0.170635	0.449906	0.049278	0.259732	0	0.059868	0	0	0	0.118377	0	0.106395	0	0.05145	0.060966	0.221238	0	0.068941	0	0	0
LOC107826409	6.328874	12.29492	6.515001	15.69207	21.22183	13.96675	16.97259	16.7919	9.850922	24.5234	26.91882	14.21125	21.32158	18.80565	36.75246	13.90063	8.447284	8.816041	26.11092	25.14873	13.82252	21.48936	11.53224	8.703865	50.01013	49.70849	24.40914	11.91063	40.89321	92.68196
LOC107783124	33.0682	39.57791	31.64275	34.28013	33.51127	34.14712	30.58603	30.93462	30.52436	46.13807	40.48637	41.933	37.86687	36.25402	30.78773	48.65181	41.89053	46.84699	49.23879	45.27713	49.55929	30.83618	31.61825	29.40576	39.66	40.10135	43.75703	37.38883	34.45183	38.38782
LOC107771120	14.0609	16.12438	11.5915	2.552957	2.056531	2.686112	5.750541	5.485738	4.063942	1.756388	2.7297	2.208912	5.465541	2.408721	2.534497	11.56066	14.005	12.69223	1.356053	1.86774	0.98033	5.022156	5.099434	3.12755	0.220379	0.444653	0.824075	2.077589	1.538504	0
LOC107823796	0.209799	0.12747	0.060791	0.190067	0.309406	0.300746	0.293279	0.179258	0.196968	0.136387	0.049037	1.240707	0.389252	0.22888	0.071777	0.573255	1.013416	0.729932	0.283852	0.354951	0.82914	0.350417	0.123371	0.219282	0.530499	0.486533	0	5.001193	0.673364	8.648116
LOC107818779	28.15791	28.74379	29.47776	16.68641	16.29808	20.34489	18.45362	18.10883	20.89288	11.16999	13.69366	14.19641	12.07767	11.50582	8.494055	21.96102	26.5204	24.79271	9.677849	10.79995	11.87377	16.56192	19.13973	23.2597	2.679581	2.457506	7.514925	14.43504	1.457658	0
LOC107808695	6.106975	6.927767	6.800222	8.440991	7.820077	7.974107	6.422072	6.510633	6.149219	5.793151	7.413018	3.678564	6.592981	5.313825	7.252737	8.400649	7.914	8.610826	7.001976	8.193934	3.886986	8.875088	7.111819	5.727352	2.845833	3.465711	3.18375	5.277765	2.842407	3.422388
LOC107813305	25.58327	26.1043	28.1541	22.07802	17.45675	21.9474	20.92687	22.03932	22.43396	13.21929	15.57179	14.48324	15.91337	16.74605	16.51274	22.3666	25.07231	23.32069	13.43504	15.03762	18.85833	23.3476	22.0078	20.01337	6.135552	7.339639	10.7857	3.7723	9.142289	3.26155
LOC107783569	0	0	0	0	0	0	0	0	0	0	0	0	0	0	0	0	0	0	0	0	0	0	0	0	0	0	0	0	0	0
LOC107820056	32.1119	28.51562	27.47373	24.67348	18.66292	33.2985	15.61832	13.63746	19.14729	23.71647	26.2919	28.25271	33.98472	29.43559	21.12742	23.45783	36.71336	28.71005	12.21132	13.71617	13.69018	11.47809	17.98933	18.66837	1.361314	2.908254	6.887058	0	6.708399	0
Novel02034	4.371154	4.52394	3.477739	3.154611	2.753409	4.858831	2.796312	2.785299	3.895163	2.022847	1.662403	3.505099	3.381478	1.616513	1.82499	2.208399	1.288343	1.546588	0.826966	1.316129	1.351378	1.608679	0.980249	2.400519	0.187338	0.309261	1.225914	0	1.070049	4.580933
LOC107788228	4.975338	4.281559	5.43433	5.462504	4.38614	4.991164	4.591884	4.567128	5.365648	3.228376	4.876412	3.031379	3.314933	3.51095	3.094304	4.575956	4.574305	5.22615	3.734859	2.957107	3.20499	4.340389	5.449013	6.766087	3.227014	1.029415	2.317203	0	2.760388	0
LOC107761720	0	0	0	0	0	0	0	0	0	0	0	0	0	0	0.497788	0	0	0	0	0	0.110582	0	0	0	0	0	0	0	0	0
LOC107759553	1.63873	1.927973	2.046119	2.024463	2.240995	2.626736	2.399081	2.393027	2.433646	1.743238	2.736896	2.517172	1.990098	2.112823	1.620786	2.375757	2.446687	2.243564	1.874508	2.091981	1.820633	1.716903	1.892249	1.557091	2.034183	0.704781	2.570724	2.13077	2.008218	0
Novel01711	2.432497	2.216921	7.330328	1.028402	1.147964	5.753509	0.272032	0.138559	0.913494	0.316266	0.341134	2.877057	0.722104	0.707662	0.332886	1.08762	16.29331	3.747961	0.329111	0.685908	5.324347	0.677147	0.858249	4.067912	0.615083	0.451286	1.341674	0	3.122908	0
LOC107790871	11.76863	15.76502	12.24379	11.44027	11.90133	8.595817	10.12155	9.033935	7.89208	9.107861	9.352463	8.143356	14.76463	12.55369	10.46847	18.87638	12.09136	17.10237	13.72382	13.21073	11.41466	9.04827	8.502299	8.786124	9.258126	7.017941	5.475517	0	11.33168	11.55035
Novel04205	0	0	0	0	0	0	0	0	0	0	0	0	0	0	0	0	0	0	0	0	0	0	0	0	0	0	0	0	0	0
LOC107800411	23.10173	16.8868	18.32662	13.88093	8.670742	23.75176	16.38777	9.590291	22.41375	1.795219	2.665123	3.010332	12.89164	12.37467	8.289701	9.20517	16.00715	10.62621	6.809642	8.691127	11.8074	12.79578	11.78633	12.22814	0.225252	0	0.350956	0	0	3.672018
LOC107832562	5.668351	4.751447	4.896961	9.699896	9.164543	9.066019	8.305334	7.085412	10.67625	8.359276	11.08977	9.902725	12.81493	9.428527	8.331722	4.615179	3.954964	4.930329	6.390945	6.837365	7.020487	8.415623	7.530654	9.691408	4.55649	2.921147	5.789721	2.502267	9.938754	2.163473
LOC107780880	5.824803	5.625521	7.443921	2.402206	1.884734	3.645264	1.786494	2.117027	3.428066	1.653111	1.036329	1.689283	2.322717	2.133999	0.579996	4.502615	6.467138	6.546365	1.80847	1.985662	1.942564	1.597281	2.492246	4.089011	0.769407	0.544351	1.438542	0	0.209274	2.687734
Novel00815	1.441395	1.016223	1.394821	1.798598	1.227312	1.549676	1.18615	1.20833	2.553293	1.087306	1.563736	2.021893	1.634856	0.949429	0.33496	1.01333	0.84076	1.419311	0.607129	0.506133	0.942527	0.726791	1.127473	1.620248	0.171921	0.26489	0.225006	0	0.130932	0
LOC107795022	1.192991	1.189195	1.944453	0	0	0	0	0	0.070002	0.036354	0.052283	0	0.041502	0	0	0.9168	1.98091	1.167371	0	0	0	0.031134	0	0	0	0	0	0	0	0
LOC107817678	3.565115	4.332217	3.522884	0.745342	0.458368	0.982804	0.383361	0.338459	0.486329	1.010247	1.730673	0.334656	0.40705	0.232697	0.500395	2.656728	3.061512	3.543926	0.772998	0.541305	0.361272	0.432602	1.0751	0.286635	0.308198	0.339186	0.2521	0	0.146699	0

LOC107831860	0	0	0	0	0	0	0	0	0.019285	0	0	0	0	0	0.021083	0.015307	0	0	0	0	0	0.017154	0	0	0	0	0	0	0	0
LOC107786345	0	0	0	0	0	0	0	0	0	0	0	0	0	0	0	0.089732	0.232659	0.089773	0.366561	0	0.109819	0	0	0.125856	0	0	0	0	0.579712	0
LOC107790087	1.275611	2.107143	2.564242	3.972508	5.996454	5.31432	6.486327	4.972743	4.865248	4.897783	5.087235	7.40899	7.632648	6.784189	6.218946	3.029995	2.541716	2.496432	5.096706	3.776776	4.798936	5.592803	4.922632	5.666388	8.265393	7.210609	5.088383	0	5.757416	9.859116
LOC107807104	6.621245	7.58088	6.701631	5.062811	5.059228	4.659839	5.692748	5.862234	4.934958	5.161643	5.793826	4.503096	6.262153	5.472922	3.956366	4.755485	5.666139	5.541439	2.919582	3.479246	2.724978	3.157556	4.099651	4.37593	2.285192	2.591952	2.812048	1.318977	2.752612	1.140394
LOC107774607	10.70535	10.95683	10.48764	12.71967	9.923886	13.67902	12.2571	9.727702	8.794325	8.699226	12.86846	6.424239	9.671022	8.295807	7.412324	12.83704	10.28071	13.03295	8.707685	11.66139	6.140908	13.25083	12.79653	13.23171	3.867092	3.044164	6.200908	9.114096	2.96556	6.566747
LOC107763785	4.86588	5.869389	5.888553	7.908902	6.753961	7.64197	7.562266	5.869468	6.942028	7.86156	8.496444	6.528966	9.558997	6.661566	6.414126	7.607614	6.636492	8.215718	6.147744	7.344591	5.438436	6.055595	6.900907	10.42094	5.910698	3.318881	5.24363	0	10.10537	2.949653
LOC107789736	0.652707	3.06354	2.978769	0.73915	0.490925	0.421044	2.299306	1.812495	1.103023	2.418594	4.691207	1.157993	0.944584	0.640864	4.187006	8.828128	1.48184	4.063285	1.589573	2.595084	1.458436	2.098608	1.75598	0.75043	0.660176	2.043438	0.617158	2.33389	1.256946	0
LOC107800310	8.994505	7.715183	8.880098	4.68266	5.113882	7.219827	5.598321	5.981202	7.107083	3.373376	4.22366	4.022898	5.255591	4.129268	3.884838	5.853503	6.723509	7.342986	3.799476	2.995293	3.266408	5.472212	5.348997	4.721796	0.926209	1.585636	1.587402	11.64225	2.9391	0
LOC107789223	0	0	0.050761	0.317414	0.051671	0.125562	0	0.049894	0	0.170826	0.122839	0	0	0.191116	0.059934	0	0	0	0.592546	0.395181	0.532567	0.097533	0.051508	0	0.516798	0.568761	0.345087	0	0	0
Novel03737	1.934748	1.505243	2.051019	0.812266	0.542827	0.710275	0.71243	1.007989	0.930365	0.322106	0.2978	0.318932	0.262657	0.566289	0.387468	1.793429	1.185301	1.477618	0.574609	0.758454	0.90378	2.049255	1.789823	2.120842	0.119322	0	0.055774	0	0	0
LOC107829077	4.930356	5.607163	5.348159	6.757243	6.599987	6.433371	6.574069	6.480969	6.567407	5.629552	7.889436	4.343306	5.300898	6.436142	6.703925	7.159798	5.658771	6.094899	5.644393	5.881815	4.688728	6.968024	6.802125	7.267342	5.327751	4.22168	4.382914	6.027182	5.274775	7.8167
LOC107820334	0	0	0	0	0	0	0	0	0	0	0	0	0	0	0	0	0	0	0	0	0	0.081459	0	0	0.123322	0	0	0	0	0
LOC107763189	0.625286	0.482199	0.878039	0.130726	0.680978	0.413698	0.201713	0.041097	0.406417	0.797343	0.202362	0.284447	0.107089	0.314842	0.345572	0.143374	0	0.143439	1.220187	0.935833	0.438671	0.080337	0.042426	0	0.425682	0.200779	0.113698	0	0	0
LOC107783787	35.94631	26.419	27.5924	30.20494	27.92996	24.41681	53.94168	53.55889	76.50284	35.02332	47.23451	52.18169	56.16735	51.97529	40.05495	35.17204	35.81417	37.90342	19.89045	19.68762	29.00299	44.01571	60.22768	57.34696	8.722543	6.138785	20.33292	14.82264	10.69141	14.64656
Novel00516	0.92272	1.163308	0.534734	0.320443	0.326593	0.479485	0.503051	0.315359	0.447583	0.19495	0.301939	0.259847	0.359515	0.402657	0.220979	0.687612	0.609141	0.676462	0.39013	0.208149	0.210384	0.295392	0.18991	0.273254	0.038887	0.064195	0.036353	0	0.148077	0
LOC107806176	3.175277	4.105262	3.59468	5.307289	6.46883	4.604675	5.326483	5.762587	4.529408	5.016479	5.592155	4.491732	5.096634	4.806734	6.290661	4.328899	4.30413	4.404286	5.569903	6.225842	4.983652	5.591301	6.100977	5.09397	5.912852	5.993636	5.469374	0	3.792103	4.565864
LOC107817227	0.390408	0.237206	0.339374	0.825277	0.748494	0.769517	0.436603	0.833938	1.099597	1.078642	0.684386	0.824569	1.158954	0.993804	0.534272	0.339421	0.440028	0.48511	1.056425	0.770603	0.890147	0.59774	0.688732	1.428191	0.740392	0.814837	0.384526	4.653284	1.253043	0
LOC107763598	2.927206	2.165615	2.095518	4.77342	4.936586	4.554045	6.90337	6.355696	4.106119	6.682643	6.833812	6.807981	5.865491	5.334919	7.281261	2.822933	3.193894	2.413429	8.107243	8.098678	8.228837	5.14801	5.376612	4.03136	9.273964	18.16076	7.204361	17.23948	13.09784	8.517343
LOC107805105	0	0	0	0	0	0	0	0	0	0	0	0	0	0	0	0	0	0	0.086145	0	0	0	0	0	0	0	0	0	0	0
LOC107802374	8.466111	9.848644	9.63305	5.300315	5.055154	7.918091	5.54216	5.175312	7.883737	4.161323	7.384355	6.105712	10.34407	7.283763	4.30937	5.74474	8.57795	9.134215	3.562049	3.726438	3.452585	4.138695	6.131989	5.827243	0.870223	0.766177	1.87109	0	3.31373	17.02349
LOC107823158	0	0	0	0.044515	0	0	0	0	0	0	0	0	0	0	0	0	0.04747	0.036634	0	0	0	0	0	0	0	0	0	0	0	0
LOC107818503	13.87577	11.89943	13.9758	10.48335	5.88991	22.95524	103.7132	55.99805	64.60715	12.38227	2.333692	11.54961	7.409858	13.29444	45.93084	3.586652	24.23812	10.15411	9.663884	3.551704	16.20392	69.74192	85.26897	12.14823	6.214563	7.053156	7.201497	296.5957	48.56152	91.81121
LOC107779486	0	0	0	0	0	0	0	0	0	0	0	0	0	0	0	0	0	0	0	0	0	0	0	0	0	0	0	0	0	0
Novel03812	0.610487	0.111277	0	0.663684	0.756278	0.525078	0.204817	0.834586	0.458521	0.595302	1.027377	0.206302	2.310648	1.065618	2.005078	0.181974	0.235913	0.091029	0.247792	0	0.334065	0.203933	0.53849	0.510464	0.154368	0	0	0	0.58782	0
LOC107824890	0	0	0	0	0	0	0	0	0	0	0	0	0	0	0	0	0	0	0	0	0	0	0	0	0	0	0	0	0	0
LOC107766320	21.31486	48.71061	55.67847	3.030313	13.06716	6.388958	14.50511	25.87381	46.77106	12.07524	36.17979	36.64609	14.49824	16.34342	21.84047	99.06862	35.71792	88.68792	10.76026	36.00161	17.26514	43.62484	21.09241	49.31721	11.74215	31.58907	15.30894	58.52959	31.00791	30.80304
LOC107774054	85.43972	70.54247	68.22414	113.5675	112.1425	104.1729	90.87629	77.30737	78.5069	149.5395	175.2155	132.7556	163.8204	125.6304	169.7853	105.1028	112.296	110.6852	89.44195	89.99168	67.92436	72.81038	69.48791	81.62	51.18293	59.06536	51.68255	77.34365	69.81846	45.59436
LOC107781298	87.40067	55.75155	59.5021	51.12744	52.85537	57.41486	44.06032	40.31808	39.07071	71.47807	65.72368	75.79175	73.33037	65.04133	67.99891	73.03946	84.10686	74.37273	50.30534	45.91277	63.39539	40.73672	41.28797	43.14025	42.74367	37.02142	47.21544	51.49926	57.1134	42.18303
LOC107821709	3.904539	3.088515	3.842415	3.871024	2.933485	4.356267	2.965952	2.895521	4.219075	2.514333	3.151128	3.049707	4.920665	3.938177	1.436655	3.293963	4.127973	3.570094	1.495116	1.807286	2.21723	2.460968	3.833956	3.773021	1.39713	0.820058	2.089744	0	2.128058	0
Novel01664	1.671695	2.285315	2.543045	3.786176	2.711913	1.048411	1.869496	2.023483	2.223402	2.44517	2.930483	2.471514	2.481272	2.583627	2.859636	4.77537	3.229995	2.492638	2.26176	1.885518	1.14346	3.024831	1.105909	1.164836	1.585148	2.132204	2.469761	0	2.682711	0
LOC107789893	6.043129	7.779234	6.62491	8.853854	8.203514	7.904487	9.069393	8.672083	7.811661	7.379132	8.474892	5.650788	7.485863	5.947175	9.092095	8.913152	6.5831	7.660532	7.660638	8.767885	5.3806	9.838965	7.956116	7.853175	4.806394	6.262765	4.599172	0	4.575579	6.652625
Novel04180	4.457852	4.893806	5.064072	3.120727	3.406701	3.812404	2.846733	3.895476	3.614515	2.222882	2.273344	2.439417	2.030131	1.768472	0.935881	3.925996	3.621509	4.154397	1.747724	2.099781	2.818225	2.580625	4.110853	4.182822	0.448325	0.493403	1.077714	0	0.243883	0
LOC107767055	0.488497	0.672007	0.480724	0.701405	0.652459	0.455829	0.86583	0.842645	0.571115	0.53926	0.853104	0.513923	0.943957	0.874803	1.068979	0.824215	0.480833	0.570343	0.617278	0.592563	0.369863	0.90828	0.601615	0.558744	0.302978	0.320617	0.217873	0	0.665603	1.709688
LOC107763566	0	0	0	0	0	0	0	0	0	0	0	0	0	0	0	0.032406	0	0	0	0	0	0	0	0	0	0	0	0	0	0
LOC107787286	0.137841	0.502502	0.239645	0	0.487885	0.296393	0.231227	0.235551	1.164705	0	0.096655	0.116452	0.460341	0.90227	0.141477	0	0.133166	0.411067	0	0	0.125714	0.115115	0.364756	0.144072	0	0	0.162918	0	0	0
LOC107781994	1.865446	0.626361	0.426735	10.85158	19.24333	11.13626	15.06986	16.35832	11.29168	28.81751	16.07527	28.49207	28.0893	30.52665	10.58092	0.585319	1.375346	0.658785	19.57684	17.52454	29.23581	7.461438	6.495185	11.08287	26.87438	17.69128	30.63522	52.66017	25.05204	9.106053
LOC107787636	0	0	0	0	0	0	0	0	0	0	0	0	0	0	0	0	0	0	0	0	0	0	0	0	0	0	0	0	0	0
LOC107825943	0.609901	0.88282	0.654921	1.300093	1.111108	0.964292	1.143466	1.164848	1.380982	1.084509	1.182363	0.969904	1.557593	0.743651	1.288795	1.12288	0.693195	0.508202	0.618886	1.092567	0.52352	1.048651	1.202529	0.487476	1.043254	1.19807	1.187289	0	2.76356	2.218301
LOC107807625	7.718768	9.430806	9.317485	10.932	11.78113	10.58165	10.79953	11.14547	9.841615	10.84644	11.88838	10.73549	9.493179	9.523958	12.17756	9.017382	8.954887	9.14716	10.84233	12.23221	9.806319	9.514397	10.19783	9.793921	13.509	12.0533	11.0352	9.64194	14.28017	0
Novel04495	0.235415	0.536379	0.434862	0.186615	0.338504	0.379649	0.246816	0.301717	0.580171	0.315644	0.474585	0.472352	0.622409	0.609961	0.332232	0.438578	0.454861	0.087756	0.179162	0.398289	0.295215	0.122876	0.129782	0.276812	0.223227	0.081891	0.521702	0	0	0
LOC107798356	0	0	0	0	0	0	0	0	0	0	0	0	0	0	0	0	0	0	0	0	0	0	0	0	0	0	0	0	0	0
LOC107824141	0	0	0	0	0	0	0	0	0	0	0	0	0	0	0	0	0	0	0	0	0	0	0	0	0	0	0	0	0	0
LOC107804800	3.285142	2.661335	4.283556	2.480143	1.453455	1.275419	2.372697	1.949246	3.169909	1.601714	0.95981	1.079314	0.507926	0.696875	0.374642	4.080153	5.554016	5.17056	1.759366	0.308778	2.91288	3.581802	6.036901	10.11013	0	0.126973	0.323564	6.525947	0.439329	0
LOC107798096	5.537222	4.917648	5.489312	6.809554	7.401672	6.003856	10.83013	11.01251	9.268971	10.13266	11.66463	9.296278	7.685468	9.382576	13.39797	6.725063	5.099023	5.428188	9.181342	10.38477	9.713267	9.897924	9.518986	8.471919	11.20117	14.32735	9.496561	25.27593	11.79765	21.85371
LOC107795136	19.39561	19.26444	20.89856	27.61978	25.79515	27.28326	25.08379	24.36203	20.98994	31.31432	25.00159	32.47754	46.8677	41.30568	32.72159	15.57876	14.08139	16.06213	15.14401	16.40735	12.86963	13.82146	15.05932	14.02065	16.29902	11.23136	18.53142	37.37588	22.36584	0
LOC107801480	1.585715	2.587346	2.156574	2.826814	2.579977	2.309781	2.316788	2.185288	1.584787	1.646032	2.833563	0.73465	1.622882	1.2556	3.517575	3.392557	1.556643	2.669525	3.114338	3.440058	0.88638	4.763109	1.466385	1.015819	1.519788	1.601425	0.574348	0	1.10819	0
LOC107763498	0	0	0.084949	0	0	0	0	0	0	0	0	0	0	0	0	0.036412	0	0	0	0.082667	0.044563	0	0.043099	0	0	0	0	0	0	0
LOC107778927	1.289653	3.224705	1.695975	5.242643	4.828027	2.986383	5.96315	5.453065	3.259801	3.482515	6.979385	1.676207	3.239438	2.308838	9.978396	4.43562	1.789004	2.416055	6.140593	7.021293	2.684115	8.864729	4.34606	2.834141	6.020358	9.386398	1.641518	4.729664	3.661629	8.178585
LOC107806898	28.10131	35.32956	34.54299	13.85324	10.74159	13.23142	17.04449	14.12552	28.99583	8.730811	13.05022	13.45688	11.64704	14.19448	8.74221	56.10123	40.26879	60.42707	6.975691	9.332831	10.36776	13.94646	13.22182	20.36376	3.179767	5.412537	3.923789	2.398139	10.17094	0
LOC107765303	0.172302	0.134599	0.149778	0.111498	0.196025	0.317564	0.454196	0.31547	0.277311	0.048005	0.500532	0.04159	1.260458	1.208397	0.833702	0.256799	0.190238	0.110107	0.074931	0	0.112244	0.370013	0.108558	0.051454	0	0.068499	0.029092	0	0	0
LOC107772574	8.684492	8.536428	8.912851	6.034657	5.009078	6.403836	2.612345	2.777743	4.631634	1.307958	2.056426	1.152393	1.138865	1.88495	0.466677	4.337051	4.744055	6.559404	1.476427	1.634688	1.637989	3.246603	5.213814	5.940464	0.086229	0.063266	0.456791	0	0	1.405694
LOC107821844	0	0	0	0	0	0	0	0	0	0	0	0.022071	0	0	0	0	0	0	0	0	0	0.021817	0	0	0	0	0	0	0	0
Novel01999	0.517587	0.56606	0.809869	1.219159	1.465583	0.667763	2.517908	0.796032	0.87468	0.40377	1.451728	0.437272	0.806658	0.903458	4.356141	1.002836	0.800051	1.003295	1.680676	1.401096	0.283229	3.976701	0.730473	0.757374	2.48667	5.473398	0.611746	0	4.983692	25.60253
LOC107811600	2.399645	2.810612	2.640044	3.303396	3.491939	2.610146	3.506476	3.379828	2.65771	3.436812	3.365266	2.415323	2.796537	2.750835	3.386529	3.024604	2.263925	2.71151	4.051983	4.741801	2.624511	4.712547	3.489159	2.68446	6.68402	6.443095	5.195985	2.011038	6.859444	9.273356
LOC107779644	1.520703	1.385933	1.615673	0.841915	1.495131	0.908301	1.629779	2.093365	1.586336	1.070967	1.954916	1.141984	1.786916	0.921673	1.474096	0.944358	1.632363	1.826595	1.028736	1.143475	0.693453	2.328294	1.937521	1.589438	0.961315	1.293079	1.397938	0	0.406734	0
LOC107801244	3.432292	3.910139	4.832165	4.562828	4.555677	5.174209	4.005303	4.143953	4.413269	3.565195	3.243847	2.899696	3.156375	2.604843	3.254771	4.364853	4.433191	4.172151	2.763568	2.777245	2.858107	5.016194	4.771617	6.745929	1.509378	0.72675	1.146457	0	2.334952	2.30678
LOC107827911	12.6889	10.82424	10.50074	14.39236	12.39568	14.13323	11.23872	12.79325	14.53373	14.05604	11.99378	15.73686	13.78644	16.05784	10.93977	8.056325	14.51411	10.55748	12.51529	11.67852	14.30359	8.689399	11.19245	14.42952	4.042741	5.155456	7.858561	10.88923	11.72906	3.138294
LOC107812420	0.745378	0.494051	0.883557	0.306943	0.17988	0.218556	0.397842	0.405282	0.699794	0.528609	0.332602	0.858704	3.017329	2.291664	0.834585	1.41389	1.047415	0.656749	0.20628	0.515895	0.247199	0.622485	0.239081	0.283298	0.171343	0	0.400444	0	0.326229	0
LOC107830643	0	0	0	0	0	0	0	0	0	0	0	0	0	0	0	0	0	0	0	0	0	0	0	0	0	0	0	0	0	0
LOC107815566	2.813993	5.444167	5.149777	4.472517	4.281063	4.670772	5.54858	4.386888	5.005706	3.273542	4.673306	2.00197	1.978469	2.315911	6.080432	4.598664	5.723271	4.085484	3.406496	3.340974	1.800989	7.668548	5.312624	4.437577	2.496661	2.335541	1.400384	0	1.901412	9.157548
LOC107799991	0.072851	0.265578	0.189983	0.395995	0.257853	0.391618	0.244413	0.124491	0.136791	0.142078	0	0.061546	0.081099	0.317907	0	0	0	0	0	0	0	0	0.064259	0.152287	0	0	0	0	0	0
LOC107770221	69.27965	53.23203	90.63568	56.13828	68.343	59.39602	12.55825	27.06392	24.95936	43.02986	36.34757	44.29363	33.99576	32.45333	29.97438	117.5423	64.44754	101.5335	115.2281	98.81893	125.8276	28.35087	27.1984	37.80663	176.3681	174.3682	267.0249	116.6174	157.4903	30.55396
LOC107816146	0.03428	0	0	0	0	0	0.028752	0	0.032184	0.033427	0	0	0.076322	0	0	0.051091	0.033117	0	0	0	0.031264	0	0	0.035829	0	0	0.081032	2.451501	0	0
LOC107806303	0.094651	0.051758	0.082278	0.137198	0.134005	0	0.047633	0.097047	0	0.166134	0.146012	0.047978	0.105367	0	0.194294	0.070534	0	0.056453	0	0.032027	0.034529	0.126473	0.033395	0.059357	0.095734	0.21072	0	0	0	0
LOC107830649	0	0	0	0	0	0	0	0	0	0	0	0	0	0	0	0	0	0	0	0	0	0	0	0	0	0	0	0	0	0
LOC107830834	0.680257	0.958136	0.913878	0.392177	0.328329	0.598385	0.959579	1.030362	1.103131	0.422123	0.433633	0.444086	0.4819	0.134932	0.444307	0.94473	1.075394	0.899057	0.533396	0.340039	0.423004	1.446073	1.036418	1.228098	0.234559	0.21512	0.328913	0	0.148864	0
LOC107795896	0.026495	0	0.023031	0.048005	0.117221	0	0.044444	0.022638	0	0.025836	0.018578	0.111917	0.117976	0.028904	0.08158	0.019744	0.025596	0	0.107539	0.067238	0.217471	0.044253	0.04674	0.027692	0.133989	0.110596	0.187887	0	0	3.276398
LOC107802403	4.382748	5.592068	5.663261	4.163709	4.213551	3.413009	4.411199	4.594896	4.337134	3.719303	3.588152	3.542552	3.665795	3.7734	3.185306	5.296263	4.714731	4.980767	3.053018	3.24657	3.327349	3.482086	4.555462	4.382756	1.287937	2.340419	2.548027	3.388416	1.71082	4.394463
LOC107767887	98.20648	100.6351	102.8456	42.28402	38.42041	71.6514	41.72716	39.1175	50.34829	37.03617	52.20167	48.31928	59.55052	64.68186	49.46026	71.72191	108.8724	82.95501	27.32206	32.69488	24.88169	45.90297	62.79596	54.28138	8.735924	4.403971	19.44195	0	13.52086	8.269071
LOC107796517	0.557891	0.112989	0.592732	1.123158	1.371274	1.266247	0.467927	0.264821	0.698363	1.027582	0.565057	3.508729	1.31111	1.758269	0.763472	0.461934	0.838397	0.323502	0.566109	0.4195	0.678408	0.103535	0.164032	0.129579	0	0	0.073265	0	2.089022	0
LOC107807128	3.270329	3.129526	4.287931	13.60397	13.43206	15.61691	8.343186	9.942899	10.43912	9.832698	6.459057	13.12361	5.915935	9.15727	10.37741	2.985389	2.474873	3.393118	16.23301	16.43746	17.22448	11.69837	8.173164	10.39686	22.77525	38.52692	13.04527	17.5406	19.54945	15.1657
LOC107776109	8.969477	11.04245	9.28314	14.20909	13.39606	13.42601	14.06942	14.50187	9.677102	12.75714	12.89154	12.01541	11.75024	12.59649	15.43662	11.42938	9.742443	9.513452	17.17237	16.47465	15.81829	12.00158	11.34298	8.468482	18.73317	17.85865	17.04392	17.71946	17.77391	27.57661
LOC107781411	3.186447	4.807135	3.807154	6.056053	5.507175	5.554202	6.835058	5.398831	4.761062	4.892168	5.980421	3.963539	5.55476	4.792797	6.304375	5.587756	4.676511	5.074753	5.407322	5.643376	4.427155	5.50335	5.166824	5.073657	4.131496	4.792178	3.157134	4.848426	4.765406	4.191975
LOC107774454	0	0	0	0	0	0	0	0	0	0	0	0	0	0	0	0	0	0	0	0	0	0	0	0	0	0	0	0	0	0
LOC107774095	6.406158	8.211258	7.936195	4.034626	3.061645	4.2724	3.965191	3.336844	3.666522	2.455298	3.387017	2.098274	2.516973	2.821683	2.443689	7.837326	6.685322	7.815375	2.172636	2.94142	1.468442	3.590474	4.200219	4.672678	0.519743	0.476669	0.809793	1.224949	1.814207	2.118195
LOC107766441	0	0	0	0	0.039379	0	0	0	0	0	0	0	0	0	0	0	0	0	0	0.045158	0	0	0	0	0	0	0	0	0	0
LOC107799307	9.617834	10.68128	11.78992	7.095837	6.344649	8.916228	7.173254	7.67645	7.840112	3.706514	6.84499	4.111362	5.738026	4.586579	4.019525	9.07705	9.736745	8.050715	4.266135	5.091106	2.941384	7.647333	8.686765	8.908853	1.747526	0.961618	2.008033	2.059315	0.970436	1.780495
LOC107760721	1.991906	3.429046	2.372826	3.943312	3.264018	2.934707	3.898295	3.466913	2.077877	2.805632	5.483443	1.932124	3.120811	2.414524	4.164596	3.628462	3.634882	3.905134	1.796666	3.370032	1.009251	5.483366	3.123543	2.236158	1.958736	1.642425	1.220734	0	2.486231	4.561577
LOC107804194	0.171737	0.052172	0.149287	0.051862	0.101309	0.123092	0.048014	0.097824	0.107489	0.111643	0.080281	0	0.063727	0.187357	0.058755	0.127979	0.110608	0.170716	0.058089	0.242129	0.052209	0.095615	0.050494	0	0.361879	0.079653	0	0	0	0
LOC107828036	0	0	0	0	0	0	0.045955	0	0	0	0	0	0	0	0	0	0	0	0	0	0	0	0	0	0	0	0	0	0	0
LOC107761218	1.918447	2.44678	2.022591	3.253079	3.221915	3.048748	3.354214	3.230557	2.200205	3.037888	4.541576	2.178336	3.536574	2.526162	4.024193	3.025979	1.853381	2.18921	4.455293	5.038954	2.479105	3.587311	2.609832	2.27992	3.415501	3.190803	2.082352	1.199966	2.746592	0.691665
LOC107784791	26.26981	22.63048	28.57947	14.96496	14.42809	22.97271	10.05591	11.65534	14.75793	11.17144	10.64871	14.58556	13.9398	14.06794	9.188071	19.65571	27.84981	26.02098	12.54437	11.17884	10.10825	10.14605	14.28844	16.0399	5.659014	3.188151	11.33628	15.2427	8.20915	3.29473
LOC107805709	113.2661	99.14029	124.0495	74.58966	66.6952	91.8993	59.1541	66.42724	81.02494	37.85521	37.41558	44.64387	35.18106	42.29537	30.32002	79.68051	106.735	98.51237	49.38473	49.41226	63.66362	62.34339	85.7655	107.6625	23.26963	10.97543	38.02264	40.55593	22.33832	38.25259
LOC107772070	0	0	0	0	0	0	0	0	0	0	0	0	0.021343	0	0	0	0	0	0	0.016219	0	0	0	0	0	0	0	0	0	0
LOC107781777	0	0	0	0	0	0	0	0	0	0	0	0	0	0	0	0	0	0	0	0	0	0	0	0	0	0	0	0	0	0
LOC107791286	4.334877	5.289556	5.233634	2.901749	2.727669	2.977556	3.35306	3.786144	4.024554	1.62037	2.397921	1.586964	2.251968	2.495934	2.076293	4.917319	4.606641	5.009344	1.832806	2.302066	1.954777	3.057031	4.163528	4.404956	0.761195	0.737203	0.91084	0	1.507252	1.489066
LOC107828647	11.48526	11.15465	10.99237	10.40507	8.315114	9.541685	8.124959	9.020325	9.911528	6.617935	7.443362	8.085891	10.59006	9.175906	6.132224	7.00268	7.901505	5.794993	5.238617	4.465316	5.925113	5.522461	4.656057	6.972249	2.86017	3.470601	4.593445	0	4.468214	0
Novel03217	0	0.076996	0.07344	0.153076	0.149513	0	0.03543	0.180462	0.039658	0.082382	0	0.463931	0.658337	0.276502	0.086712	0	0	0.062986	0.128592	0.107201	0.154101	0.035277	0.11178	0.044151	0	0	0	0	0.203367	0
LOC107803531	0.538148	1.177094	0.686108	0.097507	0.095238	0.270002	0.300912	0.122616	0	0.139937	0.050313	0.181857	0.159753	0.234837	0.073645	0.133676	0.103979	0.267475	0.036405	0	0.03272	0.119846	0.063291	0.037498	0	0.04992	0.042403	0	0	0
LOC107800137	0.132607	0.090641	0.057636	0.120135	0.029335	0.035642	0.083417	0.056652	0	0.096981	0.302198	0.056015	0.036905	0.072334	0.204156	0.321161	0.096082	0.148296	0.10092	0	0.030235	0.138429	0.058484	0.103951	0	0	0.156731	0	0	0
LOC107774843	0	0.080366	0	0	0	0	0	0	0	0	0	0	0	0	0	0.065713	0	0	0	0	0	0.073643	0	0	0	0	0	0	0	0
LOC107822111	0.060069	0	0.052216	0.054419	0.106305	0	0.403057	0.667214	0.56395	0.292872	0	0.20299	0.200607	0.065532	0.123305	0	0	0.044784	0	0	0.054784	0.200659	0.158953	0.125567	0.075945	0	0	0	0	0
LOC107820588	1.222874	0.866832	1.151603	0.615479	0.360693	0.913013	0.113964	0.116095	0.350805	0.231867	0.119094	0.028698	0.529402	0.555873	0.104594	0.683465	0.721966	0.557153	0.206815	0.172411	0.06196	0.113473	0.089888	0.142016	0	0.047265	0	0	0	0
LOC107761863	0.885541	1.10971	0.731293	0.72203	0.881533	0.88066	1.132681	0.964704	1.039231	0.777163	0.574371	0.916459	0.887217	0.579649	0.56806	0.643409	0.470529	0.75924	0.96598	1.030022	0.969154	1.072331	1.757488	1.41149	0.643766	0.831712	0.470987	3.166436	0.852662	2.737717
LOC107762410	0	0	0	0	0	0	0	0	0	0	0	0	0	0	0	0	0	0	0	0	0	0	0	0	0	0	0	0	0	0
LOC107785843	0	0	0	0	0	0	0	0	0	0	0	0	0	0	0	0	0	0	0	0	0	0	0	0	0	0	0	0	0	0
Novel02079	0.4751	0.649492	0.206497	0	0	0	0.398487	0.202969	0.446045	0.231641	0.416425	0	0.264444	2.20282	1.462888	0.177022	0	0	0	0	0	0.595152	0.104767	0.124144	0	0	0	0	0	0
LOC107820613	11.62439	14.74252	11.5049	18.8418	15.61492	15.28325	18.26633	17.23175	13.80622	10.99382	13.69962	11.24045	12.23884	10.69535	17.17769	12.47192	12.98907	12.32101	21.17528	20.97009	20.37302	21.46249	14.76254	11.41789	19.12349	23.96942	10.42846	30.04727	17.19374	47.62823
LOC107817233	1.678619	0.999088	1.310291	1.148317	0.515326	0.478803	1.40793	1.05374	2.187048	0.400865	0.768685	0.868251	1.563574	3.625208	1.160313	0.663748	0.99287	0.715132	0.243335	0.173877	0.468652	1.516296	1.178479	1.754492	0	0	0	0	0.164928	0
LOC107798630	0.425852	0.599808	0.302878	0.946964	0.959182	0.499465	0.974128	1.091578	0.76327	0.830518	0.84153	0.621423	0.474064	1.098106	1.390717	0.576991	0.336607	0.606118	0.785682	0.851479	0.776768	0.775942	0.682963	0.4451	0.48946	0.754144	0.503322	0	0.372764	2.393732
Novel03510	0.44769	0.816029	0.353789	1.290497	1.65661	0.700104	2.594344	2.399436	2.025136	3.055882	2.796748	4.50427	2.446568	2.974849	1.127856	1.000858	0.6291	0.455144	2.601815	2.375577	3.971656	1.971356	2.297556	3.147864	7.255304	4.813537	6.349607	52.39012	12.54016	17.61541
LOC107784729	0.772183	0.603214	1.006864	0.816152	0.78089	0.711593	0.585981	0.644065	0.811264	0.519912	0.605914	0.279584	0.593537	0.581667	0.603846	0.876849	0.870326	0.808715	0.391779	0.622108	0.452728	1.044076	1.151411	1.268277	0.023245	0	0.10865	1.31481	0	0
LOC107807381	176.6614	146.2828	178.4855	105.9917	89.71767	147.2902	84.46468	87.57118	117.0038	58.71831	67.38419	78.1667	52.80029	64.64292	46.98809	116.4345	166.6853	134.3735	62.91671	58.49807	80.74716	90.0707	123.449	166.9003	11.73259	8.608183	39.81003	9.831742	15.88503	17.00115
LOC107780953	0	0	0	0	0	0	0	0	0	0	0	0	0	0	0	0	0	0	0	0	0.074408	0	0	0	0	0	0	0	0	0
LOC107814267	0	0	0	0	0	0	0	0.034413	0	0	0.014121	0	0	0	0.020669	0	0.038911	0.015014	0	0	0	0	0	0	0	0	0	0	0	0
LOC107763602	184.1677	221.9821	185.2835	117.0788	95.09667	90.17472	150.135	179.0854	220.8911	94.3382	145.5201	105.9508	63.47658	84.14129	112.2059	226.1935	157.65	187.8709	102.068	109.273	108.2945	237.8218	284.4562	178.6116	13.58503	58.04018	51.39848	29.28658	19.2464	34.96756
107780539	0	0	0	0	0	0	0	0	0	0	0	0	0	0	0	0	0	0	0	0	0	0	0	0	0	0	0	0	0	0
LOC107775516	11.68407	13.54409	12.22803	14.67534	11.76297	12.65169	24.01773	21.34152	13.8738	10.61894	16.64957	9.835713	16.49709	13.38523	19.95127	16.49499	14.19557	13.08983	10.18063	12.766	10.57982	20.23103	15.9662	11.75341	6.52726	8.143309	7.435957	5.97908	8.050274	5.169544
LOC107822315	20.41089	15.19538	13.46739	53.64686	64.01828	38.70641	65.63161	67.65732	50.23845	90.59552	66.04272	99.63944	101.399	89.60569	85.21016	17.00613	23.7574	21.15739	62.63377	63.03207	80.02241	50.72627	47.28698	39.42835	111.1194	111.8218	93.88027	119.5872	119.3391	51.69787
LOC107788207	0	0	0	0	0	0	0	0	0	0	0	0	0	0	0	0	0	0	0	0	0	0	0	0	0	0	0	0	0	0
Novel02432	5.441439	7.287868	5.676153	1.929	1.172334	2.136599	2.143081	2.183156	2.843082	0.553679	0.995358	0.719543	1.158823	0.722685	0.582777	4.196009	5.074039	5.961767	0.768222	0.840563	1.553536	2.252386	4.340606	3.808079	0	0.131676	0.223699	0	0	0
LOC107793520	2.254169	2.135491	3.248646	2.310868	2.020882	2.55106	2.039932	2.534241	3.090931	1.822108	1.996578	1.779096	1.948065	1.779675	1.583001	1.547193	1.977137	2.2334	1.444658	1.405063	1.02792	2.402684	2.773191	3.627087	1.499976	1.114287	2.03324	2.121118	3.28427	0
LOC107812582	0	0	0	0	0	0	0	0	0	0	0	0	0	0	0	0	0	0	0	0	0	0	0	0	0	0	0	0	0	0
LOC107787941	5.583337	3.199999	4.303082	3.024503	1.833589	3.403633	2.558745	2.50823	3.350482	2.52578	2.906009	3.063592	2.883456	2.700199	1.890502	3.603089	4.226206	3.990961	0.759305	0.486919	0.892429	1.15368	2.335507	2.466637	0.145547	0	0.340157	0	0	0
Novel01819	0	0	0	0	0	0	0.013794	0	0.01544	0	0.011532	0	0	0	0	1.250051	0.603744	0.821488	0.634145	0.612127	0.029998	0.315889	0.478706	0.326593	0.436641	0.114415	0.116625	0	0.395879	0
LOC107770179	0.297612	0.610283	0.840801	0.404432	0.263347	0.799924	0.062405	0.190716	0.069853	0	0.260857	0.125716	0	0.16234	0	0.166336	0.646917	0.665648	0.301996	0.12588	0.203571	0.31068	0.196885	0.311064	0	0.103526	0	0	0.358203	0
LOC107763858	12.34376	11.47185	13.27152	14.01531	13.29392	14.49338	12.90749	13.42641	13.68553	9.581865	9.338776	10.59987	10.66754	10.76429	9.210244	14.03994	13.37652	14.3491	15.03895	14.97598	14.07214	12.47883	14.2547	13.45341	6.365712	3.446387	8.398462	5.807587	12.31551	2.510635
LOC107780053	14.56201	15.38435	12.95234	40.1024	40.66494	32.55812	28.84316	32.25061	23.94205	50.48817	34.95071	39.49822	28.92643	34.67089	35.54333	14.6128	14.83802	16.11385	61.18901	56.97542	61.51454	33.65987	31.93058	27.05815	56.17891	70.22806	70.18599	39.72132	49.8769	91.08432
LOC107777658	2.767894	2.410833	2.588811	2.729761	2.588727	2.975824	2.468485	2.484709	3.14137	3.672752	3.807995	3.226391	5.694477	5.102803	2.733008	3.733635	2.335535	3.787588	3.448617	3.349159	2.23678	1.63856	2.549632	2.893002	3.565905	2.998181	3.561301	5.011229	4.132631	0
LOC107781137	0	0	0	0	0	0	0	0	0	0	0	0	0	0	0	0	0	0	0	0	0	0	0	0	0	0	0	0	0	0
LOC107773505	50.98483	26.69751	25.58557	214.1406	238.1021	259.5273	137.4739	122.7029	94.55494	184.7201	163.2985	241.2935	229.0468	237.1732	146.9662	42.77568	83.01359	66.66287	113.2386	90.92038	156.0357	75.31373	64.90486	88.13515	131.6543	63.85721	239.3087	69.83024	88.31155	38.81288
LOC107828624	0.367148	0.390378	0.301423	0.129351	0.072195	0.263153	0.256619	0.522835	0.287245	0.139228	0.300352	0.189552	0.204357	0.311531	0.753661	0.471198	0.29558	0.441	0.165581	0.103527	0.204628	1.073152	1.115479	0.341105	0.077364	0.113524	0	0	0	0
LOC107814935	0.169076	0.030818	0.029395	0.030635	0.029922	0.145422	0	0.028893	0	0	0	0	0	0	0	0	0.032668	0.025211	0	0.028605	0.03084	0.02824	0.059655	0	0	0	0	0	0	0
LOC107774438	26.28375	17.53804	18.80872	24.66217	22.88381	31.38684	23.02948	22.13673	26.21862	42.15216	28.76425	36.79726	33.23036	35.53563	17.96856	13.88556	22.26362	14.94168	22.47979	25.09293	20.97482	19.55924	22.97701	27.12837	48.65917	9.991972	82.60036	30.20882	46.77436	14.51039
LOC107808477	0	0	0	0	0	0	0	0	0	0	0	0	0	0	0	0	0	0	0	0	0	0	0	0	0	0	0	0	0	0
LOC107815003	0.433189	0.246749	0.188281	0.425151	0.559	0.388109	0.393612	0.323862	0.271131	0.228808	0.455629	0.381219	0.20093	0.33475	0.296409	0.282461	0.104624	0.20185	0.549463	0.68709	0.395076	0.256252	0.302497	0.433904	0.09128	0.125573	0.106666	0	0.434485	0
LOC107791599	0	0	0	0	0	0	0	0.167046	0	0	0	0	0	0	0	0	0	0	0	0	0	0	0	0	0	0	0	0	0	0
LOC107778708	0	0	0	0	0	0	0	0	0	0	0	0	0	0	0	0	0	0	0	0	0	0	0	0	0	0	0	0	0	0
LOC107772539	0	0	0	0.129654	0	0	0	0	0	0.139554	0	0	0.47795	0	0	0	0.13826	0.106698	0	0	0	0	0.126236	0	0	0	0.169149	0	0	0
LOC107805572	0	0	0	0	0	0	0	0	0	0	0	0	0	0	0	0	0	0	0	0	0	0	0	0	0	0	0	0	0	0
LOC107813522	0.845572	0.895602	0.33772	0.786749	0.545997	0.171991	5.117868	4.569196	1.137151	0.77997	2.371663	0.386142	1.526437	0.523568	4.761568	1.515697	0.485721	0.63041	0.602939	0.985948	1.104657	5.801958	3.205141	1.743697	0.751231	1.812526	0.189075	3.268668	0.55012	4.239162
LOC107826578	12.1829	8.449419	8.728297	6.852697	6.604371	6.225198	11.62194	10.32361	14.5801	13.25062	8.659999	8.39796	13.33862	11.64783	10.85529	11.32344	8.600936	10.2307	7.234019	7.474603	7.78381	8.469229	10.00806	12.83851	12.22921	9.081221	9.335747	16.7548	30.45434	57.94514
Novel01352	2.537916	5.332744	3.799491	9.771698	8.920478	5.45714	14.48671	10.96279	6.486258	7.630597	12.01222	3.871295	6.042881	6.306578	18.16157	7.354891	6.53824	4.572633	8.655875	11.74833	5.207899	17.13246	9.078805	3.610523	12.21087	18.63755	4.332773	5.041579	4.412211	13.07693
107762711	0	0	0	0	0	0	0	0	0	0	0	0	0	0	0	0	0	0	0	0	0	0	0	0	0	0	0	0	0	0
LOC107777139	1.849568	1.820505	1.189765	0.87132	0.589183	0.874945	0.651551	0.600522	1.250248	0.180356	0.466891	0.750032	0.700048	0.686048	0.759338	1.323168	1.286523	1.296195	0.675653	0.531966	0.10121	1.050343	1.239895	1.237227	0.140305	0.154412	0.262325	0	1.068537	0
LOC107776693	0	0	0	0	0	0	0	0	0	0	0	0	0	0	0	0	0	0	0	0	0	0	0	0	0	0	0	0	0	0
Novel00696	1.369406	2.964105	3.025588	2.274455	1.918589	2.147073	3.158593	3.412667	2.839162	1.168425	1.440342	1.108707	1.016294	1.742946	0.819887	4.507132	4.40987	3.871102	4.110835	5.212903	3.226379	1.620137	5.385249	3.101155	1.226369	1.667249	1.348773	0	1.373501	0
LOC107810527	0.267517	0.243808	0.325565	4.750188	2.083104	5.119514	0.089751	0.091429	0.150694	0.313035	0.11255	0.045201	0.536046	0.525326	0.658972	0.239224	0.41351	0.079778	3.040323	3.39451	3.415716	0.089364	0.188774	0.391452	0	0.074446	0.063237	0	0	0
LOC107771237	1.445726	0.608123	0.87005	0.503752	1.377679	0.717384	1.02604	1.235267	1.670537	1.518209	1.091724	1.503249	2.352203	2.183837	1.027283	0.497242	0.322314	0.331646	1.579871	0.282227	0.4057	0.371496	0.68666	1.162363	1.124823	0	0.131441	0	1.070806	0
LOC107818056	0	0	0	0	0	0	0	0	0	0	0	0	0	0	0	0	0	0	0	0	0	0	0	0	0	0	0	0	0	0
LOC107830201	7.31678	8.109677	6.873785	11.16855	10.97608	8.460513	10.53181	10.54967	9.874641	9.828909	10.26106	9.046764	9.73599	8.397989	10.20856	8.050917	7.234296	6.918152	12.06491	11.58918	9.731497	12.22087	11.21026	10.59497	12.50281	19.32755	11.59802	8.175832	13.57653	24.74104

LOC107775655	1.869929	2.100538	2.287026	0.49246	0.557959	1.168839	0.291794	0.334406	0.67365	0.572468	0.381161	0.330649	0.09682	0.474419	0.089267	3.143417	3.970118	4.60381	1.765093	1.857732	0.674231	0.653704	1.304178	1.136308	0.219922	0.181526	0.925161	0	0.209361	0
NitaMp152	0.692854	0.12629	0.120457	0.251076	0.36785	0.297961	0	0	0	0.135124	0	0.117068	0.308518	0.151174	0	0.103263	0.803226	4.648968	1.406121	0.117221	0.252758	0	0.244457	0	0	0.192811	0.16378	0	1.334258	0
LOC107779108	0	0	0	0	0	0	0	0	0	0	0	0	0	0	0	0	0	0	0	0	0	0	0	0	0	0	0	0	0	0
LOC107810512	0	0	0	0	0	0	0	0	0	0	0.039156	0	0	0	0	0.083227	0	0	0	0	0	0	0	0	0	0	0	0	0	0
LOC107820352	0	0	0	0	0	0	0	0	0	0	0	0	0	0	0	0	0	0	0	0	0	0	0	0	0	0	0	0	0	0
LOC107784200	0.241381	0.439979	0.209828	0.218679	0.071197	0.346019	0.337428	0.137495	0	0.078459	0.338514	0	0.537418	0	0.33033	0.119918	0.155463	0.119973	0.244937	0.272256	0.073381	0.134389	0.283886	0.168195	0	0	0	0	0	0
LOC107768328	9.716724	8.316008	10.17218	3.849279	2.804365	5.204612	2.862666	2.767411	2.256099	1.799917	2.857221	2.442092	1.240636	1.063846	0.893636	6.981383	9.050715	9.191604	1.767	2.062285	1.524614	2.065029	2.242537	2.984893	0.660478	0.436133	1.769995	2.490621	1.341355	4.306808
LOC107761350	1.926401	1.254055	1.71445	1.371246	1.704606	1.479369	1.731167	0.979744	1.80859	0.491984	1.222147	0.813739	0.765892	0.750575	0.376611	1.640636	1.152088	0.991671	0.465423	0.465601	0.627468	1.187439	1.416011	1.869654	0.057989	0.12764	0.108421	0	0	0
LOC107818197	10.21649	12.41476	9.750276	13.09988	9.111967	13.19239	13.48825	12.79365	13.98153	8.644591	10.89732	8.56267	12.69699	13.44559	11.15169	11.05747	12.63776	10.03486	6.335379	6.950537	6.729383	10.65378	12.30157	10.45227	2.904559	3.685499	5.302806	5.798597	6.115695	0
LOC107815975	4.825396	5.345968	5.52005	8.028077	7.269818	7.887908	5.175688	5.395086	4.74923	4.425497	5.320633	3.531057	4.892439	4.461909	6.186215	6.376356	5.718861	5.871074	8.500578	8.634317	6.920283	7.924802	6.471502	5.530978	4.014259	4.667483	4.367529	3.848521	3.281727	2.218301
LOC107830940	0	0	0	0	0	0	0	0	0	0	0	0	0	0	0	0	0	0	0	0	0	0	0	0	0	0	0	0	0	0
LOC107766510	0	0	0	0	0	0	0	0	0	0	0	0	0	0	0	0	0	0	0	0	0	0	0	0	0	0	0	0	0	0
LOC107801909	0	0	0	0.101396	0.198073	0.360991	0	0.09563	0	0	0	0	0.124594	0	0	0	0	0	2.271426	0.946788	1.837355	0	0.098723	0.233963	0.849025	0.77866	0	0	1.07767	0
LOC107763262	8.846582	8.062569	7.878417	8.769221	8.255558	8.597656	7.978955	7.345235	8.258637	8.659026	8.761622	6.685637	8.308787	8.160796	5.471873	9.4604	8.192448	10.61988	5.815544	5.66555	5.804264	7.05409	8.15596	11.78878	5.960997	3.013594	6.005771	1.191442	10.90833	2.060254
LOC107792781	4.622593	4.502562	4.319697	8.585063	6.928072	10.77837	6.10654	5.381424	5.804609	9.437813	9.541769	6.907491	6.59323	5.862513	5.456179	4.52125	3.153981	4.329464	6.889449	8.529551	4.005089	5.404628	6.320021	7.488877	8.255163	4.060201	10.79048	2.06613	4.450962	0
LOC107798507	3.168948	3.257514	2.834778	5.32453	4.858255	5.130318	4.83682	4.738361	4.73948	5.767035	5.761875	4.467214	6.152995	5.004851	6.788693	4.063983	4.093826	3.090591	4.561707	4.862687	3.175787	4.831349	4.014068	4.198	2.795242	2.922483	3.048615	1.317585	4.346312	1.139191
LOC107793676	0.186249	0.239637	0.276186	0.357315	0.329611	0.388702	0.294052	0.355716	0.401146	0.138883	0.284242	0.185116	0.060981	0.131475	0.224895	0.138793	0.232853	0.253209	0.233461	0.240965	0.099919	0.320232	0.367223	0.332079	0.083109	0.091465	0.142438	0	0.10549	0.677415
Novel04700	1.083719	1.316902	1.256072	1.227243	0.71921	1.553508	0.795342	1.388939	1.483772	1.71724	1.551472	1.754816	1.306946	0.886713	0.973262	0.908537	1.265081	1.144608	0.549842	0.95495	0.864824	1.395273	1.593187	1.840642	0.456717	0.18849	0.800543	3.229219	0.869568	0
LOC107798985	0	0	0	0	0	0	0	0	0	0	0	0	0	0	0	0	0	0	0	0	0	0	0	0	0	0	0	0	0	0
LOC107767729	1.190449	2.025236	1.17281	0.575193	0.491581	0.81059	0.36611	0.576386	0.335294	0.270862	0.473021	0.335242	0.353393	0.086581	0.162913	1.567253	0.843388	0.917115	0.201331	0.335679	0.289523	0.099418	0.105005	0.207376	0	0	0	0	0	0
LOC107799416	1.702342	2.148196	1.821305	0.711801	0.811109	1.407866	0.439332	0.783207	0.491764	0.638461	0.367287	0.663778	0.437324	0.428578	0.268806	0.683086	1.518098	0.878655	0.531514	0.332323	0.59714	0.328078	1.270566	1.505551	0.33112	0	0.773858	0	0	0
LOC107764137	0	0	0	0	0	0	0	0	0	0	0	0	0	0	0	0	0	0	0	0	0	0	0	0	0	0	0	0	0	0
LOC107818311	0	0	0	0	0.036727	0	0.069625	0.070927	0.077934	0.080946	0.029104	0.035065	0.046204	0	0	0	0	0	0.084234	0.035111	0	0.069324	0	0.043381	0.052475	0.057752	0.049056	0	0.199822	0
LOC107799262	6.264569	8.646579	7.208413	7.676528	6.344358	7.494345	6.211083	5.692957	5.609478	5.402532	5.357594	5.093631	6.409451	5.451667	7.433274	7.299378	7.503905	6.816773	4.464494	6.003938	4.177712	6.244781	6.052788	5.298737	2.174654	2.84678	2.375341	2.589626	1.830512	3.358506
LOC107824704	3.225355	2.90032	3.214947	2.025926	2.054884	1.895649	2.597034	2.094431	2.260985	2.138687	2.231466	1.925574	3.638383	3.143375	2.295222	4.134086	2.949779	4.103917	2.007359	1.164129	1.725726	1.687976	2.579445	2.831758	0.434968	1.256597	1.321531	0	3.519681	2.659047
LOC107775933	0	0	0	0	0	0	0	0	0	0	0	0	0	0	0	0.097645	0.031647	0.024422	0	0	0	0	0	0	0	0	0	0	0	0
LOC107816656	0	0	0	0	0	0	0	0	0	0	0	0	0	0	0	0	0	0	0	0	0	0	0	0	0	0	0	0	0	0
LOC107806977	27.07587	21.51103	25.44651	15.7964	12.60644	19.43198	12.00879	15.01917	19.76373	9.12334	9.492809	12.7546	10.73091	10.28433	6.83836	15.63451	27.7325	21.19024	8.77465	8.274337	12.34683	13.43684	22.0071	25.55862	2.061097	2.071089	4.858867	0	3.071141	4.38257
LOC107778844	0	0	0	0	0	0	0	0	0	0	0	0	0	0	0	0	0	0	0	0	0	0	0	0	0	0	0.100664	0	0	0
LOC107788241	0	0	0	0	0	0	0	0	0	0	0	0	0	0	0	0	0	0	0	0	0	0	0	0	0	0	0	0	0	0
LOC107794571	7.052157	9.763413	11.09068	3.852846	3.263012	5.44047	0.745013	1.103918	1.339337	1.154873	1.207933	1.728234	2.516973	3.318213	1.02218	7.80268	7.775115	9.612328	2.922506	2.709584	2.037519	1.416163	2.967781	3.46039	0.170154	0.14981	1.717918	0	0.518345	0
LOC107759377	2.471538	2.201309	2.587913	1.679316	1.21775	2.113687	1.389843	1.535818	1.107458	1.150257	1.752738	1.16265	1.219349	1.194963	1.470144	2.825478	2.523379	2.889589	1.253973	1.425512	1.024584	1.735675	1.312991	1.174205	1.349335	0.742504	2.091285	0	2.028216	0
Novel01688	2.615547	2.164192	2.393304	2.244839	0.822224	1.443017	1.270078	1.52907	1.64783	0.671181	0.917011	0.959469	0.996095	1.126355	0.635809	2.282508	1.329915	2.232238	0.977817	0.553143	1.475195	1.264601	2.428511	2.877651	0	0.191544	0.203379	0	0.497059	2.127937
LOC107823815	31.18977	29.72756	30.16536	30.3594	24.5966	26.81345	26.60952	27.31053	33.69703	19.21187	17.40441	23.13166	28.62485	24.09128	16.67818	23.19829	29.09687	28.71161	18.72378	20.04006	23.39704	25.99768	32.07918	39.1941	7.073921	8.447758	12.87419	21.28349	16.33405	25.76254
107830333	0	0	0.632397	0	0.643737	0	0	1.243186	0	0.709401	1.530363	0	0	0	0.746683	0	0.702823	0.54238	1.476427	1.230824	0.663489	3.037757	0.6417	0.760379	0.919777	3.036776	0	0	0	0
LOC107819844	0	0	0	0	0	0	0	0	0	0	0	0.027937	0	0	0	0.073927	0.127786	0.024654	0	0	0	0	0	0	0	0	0	0	0	0
LOC107805122	0.485747	1.328097	0.563	1.100156	0.573095	0.522238	0.882737	0.276691	0.380034	0.236833	0.454142	0.478768	0.540741	1.324816	1.080209	0.542969	0.156424	0.905363	0.821505	0.273939	0.221505	0.27044	0.428461	0.507703	0.307066	0.225294	0.095686	0	0.38976	0
LOC107778960	3.139931	1.951132	2.357274	1.48696	1.704938	2.07152	1.376652	1.463369	2.076934	2.157198	1.200939	1.868949	1.90659	1.55705	1.318393	2.871671	3.92968	2.606969	1.0862	1.448818	1.887419	0.893945	1.825436	2.013866	0.631563	0.496476	0.59041	0	1.030687	0
LOC107793387	0	0	0	0	0	0	0	0.037992	0	0	0	0	0	0	0	0	0	0	0	0	0	0	0	0	0	0	0	0	0	0
LOC107796573	5.032426	5.684597	5.08314	4.013313	2.446026	3.35297	4.666784	3.906194	4.458485	2.522742	4.597303	2.185648	4.102996	2.783728	4.364911	6.73446	5.683451	6.024157	2.301548	2.818071	1.842327	5.948907	3.844991	4.00048	0.582484	1.627284	0.921509	0	1.535571	6.573856
NitaMp008	0	0	0.070266	0	0	0	0	0	0.151779	0	0.17004	0	0	0.088185	0	0.060237	0.156183	0.120529	0.082024	0	0.221163	0.135011	0.0713	0.084487	0	0.112473	0	0	0	0
LOC107777880	2.528018	1.727982	2.031071	1.856495	1.626877	1.709	1.750901	1.81637	2.247552	2.05428	2.779834	1.828323	3.773619	4.34585	2.083613	3.73922	3.86694	3.58387	1.02999	0.842451	0.960664	2.239166	2.381916	3.002595	0.774832	0.186537	1.177057	0	1.475246	2.368351
LOC107827684	52.65628	54.21792	52.84668	68.10244	66.6224	65.3182	67.12563	61.66427	56.37117	63.28757	60.85445	60.47089	80.51689	62.78917	69.81652	62.55362	63.46396	59.07766	68.5837	67.36674	67.36967	61.29397	58.08403	52.31135	51.74058	44.08134	60.50802	67.79898	74.35924	95.25646
LOC107794114	1.28627	1.37851	1.211304	1.251613	1.254101	1.28046	1.188733	1.1092	1.039884	0.975548	1.361251	1.066553	1.604251	1.078429	1.528	1.819431	1.36921	1.47397	1.317303	1.370195	0.74948	1.541672	1.292155	1.232375	0.587252	0.629727	0.337837	0.851727	0.458708	2.945629
LOC107810248	0.23304	0.19263	0.244976	0.176754	0.287733	0.06992	0.250008	0.175963	0.193348	0.29066	0.307814	0.2564	0.434383	0.301535	0.300373	0.3554	0.230372	0.319199	0.225474	0.187967	0.123568	0.253456	0.210337	0.243573	0.315189	0.301635	0.21138	0	0.208732	0
LOC107801166	0.028498	0.233754	0.074319	0.206544	0.050434	0.030639	0.047805	0.170448	0.026755	0.083368	0.099915	0.024076	0.126899	0.124361	0.263248	0.127422	0.082595	0.12748	0.115672	0.09643	0.025991	0.095198	0.075412	0.089359	0.216183	0.356879	0.033683	0	0	0
LOC107778964	1.399459	0.838143	1.181763	1.376511	1.592143	1.160684	0.804882	1.161572	0.900942	0.54586	1.177562	1.418763	1.246318	1.134151	0.41039	0.804506	0.656683	0.626014	0.770898	0.642659	1.239864	0.400706	0.881725	1.170172	0.505526	0.278178	0.661619	2.859461	1.924999	0
LOC107805139	0.346503	0.315795	0.326309	0.078479	0.127754	0	0.169533	0.172703	0.135547	0.056314	0.121484	0	0.064289	0.094505	0	0.150625	0.195272	0.301388	0.058601	0.024427	0.026335	0.241145	0.02547	0.120722	0.036507	0.040178	0.170641	0	0	0
LOC107780377	0	0	0	0	0	0	0	0	0.116948	0	0	0	0	0	0	0	0	0	0	0	0	0	0	0	0	0	0	0	0	0
LOC107818951	12.4419	16.00934	14.23339	12.47559	11.65371	10.11804	13.45859	11.47058	11.2623	9.283478	10.97713	6.612791	8.814873	8.39035	12.36833	9.325979	6.357281	8.780613	7.074534	8.199792	4.200215	10.37535	7.650895	7.600887	2.911322	3.824596	2.581769	5.20715	3.856018	3.376597
LOC107796770	0.064907	0.059155	0.056422	0	0.057434	0	0	0	0	0	0	0	0	0	0.133237	0.145106	0.062705	0.048391	0.197589	0.219627	0.29598	0.054205	0.114504	0	0.492371	0.270939	0.153429	0	0.937453	0
LOC107768486	27.30074	26.63152	25.92511	35.85445	43.18359	33.56207	25.80402	30.59792	24.07559	42.19075	31.9759	47.14686	42.12899	40.547	36.83277	22.84201	20.59054	23.18936	59.99111	55.84102	43.78743	30.03252	23.01817	23.02457	48.41822	59.67886	38.36468	45.78015	58.73806	18.62671
LOC107774968	0	0	0	0	0	0	0	0	0	0	0	0	0	0	0	0	0	0	0	0	0	0	0	0	0	0	0	0	0	0
LOC107827781	0	0	0	0	0	0	0	0	0	0	0	0	0	0	0	0	0	0	0	0	0	0	0	0	0	0	0	0	0	0
LOC107823328	0.208352	0.417752	0.217339	0.226508	0.737455	0.224004	0.803869	0.320439	0.273854	0.40634	1.168774	0.105613	0.371105	0.409144	1.796315	0.527899	0.322057	0.093201	0.422843	0.599256	0.076008	1.914005	0.330805	0.21777	1.580524	2.725126	0.394009	0	0.802466	2.576547
LOC107806358	18.66665	19.8993	17.97211	19.21709	16.51748	16.51126	11.5289	9.569577	13.43589	21.31881	28.97444	25.30385	31.95709	28.78786	28.2159	20.02256	25.16501	23.21572	10.3892	10.74246	10.24038	9.920279	10.20345	12.35657	4.863097	2.636682	8.824993	2.022621	11.16541	13.99015
LOC107814054	0	0	0	0	0	0	0	0	0	0	0	0	0	0	0	0	0	0	0	0	0	0	0	0	0	0	0	0	0	0
LOC107819616	3.07887	2.521857	2.744153	3.919143	3.552048	3.100658	2.124744	2.897067	2.74422	3.002287	2.541496	3.160848	3.123744	2.933719	2.560055	2.178206	2.522632	3.225222	3.361507	2.86826	3.092334	2.538697	3.368926	4.643745	2.710057	2.494494	3.961417	2.787123	3.002081	4.819523
LOC107806595	1.128874	1.152289	1.21682	0.13636	0.079912	0.064729	0.012624	0.115745	0.028262	0.044032	0.042217	0.025432	0.067023	0	0.231729	0.841238	1.00334	0.807959	0.168007	0.127327	0.219638	0.238831	0.265531	0.125856	0.01903	0.104716	0.05337	0	0	0
LOC107818049	4.135244	3.94823	3.822914	3.330064	3.020239	3.881335	3.248191	3.477181	3.142854	5.312508	6.029402	6.37715	7.964633	6.731218	5.995918	3.326159	3.994994	3.425556	3.063863	2.554192	3.232639	2.13785	3.010684	3.841917	1.742735	1.187311	3.49109	14.08231	7.268197	4.058546
LOC107778913	0	0	0	0	0	0	0	0	0	0	0	0	0	0	0	0	0	0	0	0	0	0	0	0	0	0	0	0	0	0
LOC107794010	5.392034	8.313453	5.850166	22.03719	25.47046	31.68789	4.998587	7.10117	5.138404	87.76368	56.42919	66.78883	41.79176	36.84268	33.45508	4.441113	4.38666	5.349912	64.6291	51.95755	74.94758	5.281748	6.651431	8.644312	147.6712	123.0317	247.3472	168.1593	55.6268	32.58774
LOC107791215	0	0	0	0.090675	0	0.035869	0	0	0	0	0	0	0	0	0	0.049724	0	0.198988	0.067709	0.028223	0.030428	0	0	0	0	0	0	0	0	0
Novel02998	5.626732	7.179302	5.780508	7.878008	6.789413	6.929342	7.379398	8.828562	9.892914	6.48437	9.899538	4.926479	6.035984	6.91975	7.035151	6.708876	8.499765	7.169581	5.398185	8.481149	4.945068	12.21748	9.475104	8.554267	4.397684	6.26335	5.803937	7.316198	2.462645	6.325624
LOC107798698	2.50317	2.553728	2.240906	2.369286	2.942273	2.89205	3.196279	2.681451	2.420238	2.185887	3.300869	2.241001	3.202469	1.752626	4.371449	3.118222	2.815302	2.924672	3.411999	3.192066	1.942197	4.586537	3.427283	1.640076	1.889413	2.183359	0.662361	0	1.259075	2.310071
LOC107768910	19.59644	37.73049	23.38285	9.376451	7.810072	8.830348	12.38237	11.02782	7.267128	13.45656	22.27546	8.477541	12.12596	11.15745	36.25418	56.54697	24.19346	25.13334	8.103317	11.18482	8.017771	8.643441	12.03333	5.363077	5.247434	5.872521	4.988294	6.261942	3.119515	2.165643
LOC107770845	0.020679	0	0	0	0	0	0	0.017669	0	0	0.0145	0.01747	0	0.02256	0	8.644994	5.933346	6.829744	3.756058	4.950508	4.413133	10.91434	8.481691	7.327019	2.039269	1.007063	1.539773	1.478837	2.488895	2.55722
LOC107766092	3.31933	4.022466	3.075671	3.139327	2.162526	2.862792	2.661689	3.552949	2.979352	1.636168	2.276362	2.557734	3.410884	3.66102	2.321164	2.908482	5.250616	3.344926	1.443526	1.049115	1.69661	3.228992	3.732228	5.032483	0.322819	0.456785	0.603566	0	1.756092	0
LOC107831588	0	0	0	0	0	0	0	0	0	0	0	0	0	0	0	0	0	0	0	0	0	0	0	0	0	0	0	0	0	0
LOC107830013	0.876808	0.799103	0.79533	0.897954	0.708392	0.368873	1.406884	1.726345	0.787395	2.156084	3.715642	1.932396	1.867274	1.039733	3.795365	2.727229	0.8839	1.108444	2.011551	4.063333	0.486752	1.209797	1.412301	0.796904	1.494136	0.583485	0.450573	0	0.367067	2.357147
LOC107786420	0	0	0	0	0	0	0	0	0	0	0	0	0	0	0	0	0	0	0	0	0	0	0	0	0	0	0	0	0	0
LOC107766641	0	0	0	0	0.076864	0	0.145715	0	0.081553	0.084705	0.18273	0	0.0967	0.66336	0.178312	0	0	0	0	0	0	0	0	0	0	0	0	0	0	0
LOC107776998	1.931575	2.07105	1.810769	1.852845	2.111345	2.565309	2.541325	1.68275	1.920121	2.400577	2.7354	3.135708	4.553483	1.652748	2.682227	1.947439	1.317221	1.750674	1.921597	2.69126	3.350549	1.423331	2.338517	1.54385	1.292875	1.844462	3.133483	0	2.005729	0
LOC107760889	0.097784	0.029706	0	0.501996	0.173052	0.280347	0.710804	0.696246	1.468867	0.190704	0.182844	0.220296	1.015978	1.280134	0.367998	0.02429	0.40936	0.048602	0.099225	0.137865	0.089181	0.626077	1.121278	0.68136	0	0.090706	0	0	0	0
Novel03718	234.2175	132.1214	124.2553	276.8836	267.9588	428.4531	360.0718	362.4722	436.8189	725.6059	827.5257	1476.39	874.8475	1386.094	538.2238	278.7914	331.1689	285.1904	239.5444	191.4198	450.8341	296.2416	325.627	430.683	420.2731	189.8434	660.7586	3041.939	1131.053	313.5435
LOC107785817	0.886005	1.192	0.550132	0.477783	0.335998	0.272161	0.477727	0.468635	0.178247	0.102853	0.236673	0.106932	0.117418	0.207126	0.281472	1.383385	0.631776	1.022286	0.406716	0.838725	0.307829	0.440432	0.651262	0.176391	0.053342	0.205469	0.074799	0	0.101561	0
LOC107759583	4.36498	12.89466	4.710271	0.681802	0.399561	0.323647	0	0	0	0.293545	0.211085	0	0	0	0	5.27176	5.816466	3.927577	0.152734	0.254653	0	0	0.132766	0	0	0	0	0	0	0
LOC107831435	3.984514	4.43837	4.906843	4.913296	4.578592	6.068774	4.850518	4.421092	3.507035	4.77581	6.441593	3.950643	4.68204	3.833733	4.827996	5.010634	4.384011	4.827265	4.604767	5.336823	2.473101	4.945142	4.368849	3.962167	4.198031	4.312125	4.382335	0	3.330359	1.710893
LOC107790454	0	0	0	0	0	0	0	0	0	0	0	0	0	0	0	0	0	0	0	0	0	0	0	0	0	0	0	0	0	0
LOC107777508	0	0	0	0	0	0	0	0	0	0	0	0	0	0	0	0	0	0	0	0	0	0	0	0	0	0	0	0	0	0
LOC107782217	6.909495	4.37407	5.610654	3.748295	1.75731	2.402047	2.776187	3.040208	3.729018	1.492768	2.349942	3.180822	2.901677	4.468587	2.505457	4.224	6.635182	5.305553	1.805299	1.29499	2.792315	2.418688	4.342881	6.054205	0.784644	0.345415	0.684614	0	0.796761	0
LOC107785946	1.785473	1.740017	1.690377	3.747586	3.816797	3.554114	2.046161	1.631288	1.659684	2.258208	2.045305	1.896722	3.28645	2.73858	1.759984	1.92335	1.69076	1.594739	4.466652	4.247029	3.434111	2.081628	2.276595	2.032469	2.078578	1.229879	1.901346	1.264223	3.574533	0
LOC107764505	0.124025	0.188389	0.179687	0.037453	0	0.088894	0.034675	0	0.077627	0	0.028989	0.069853	0	0.045102	0	0.123231	0.039939	0.184931	0.041951	0	0	0	0.145864	0	0	0.048862	0	0	0	0
LOC107765079	0.054562	0	0	0.247153	0.869045	0	0.045764	0	0	0.159615	0	0.138287	0.182218	0.119049	0.168004	0.04066	0.105423	0.081357	0.221464	0.738495	0.248808	0.091133	0	0	0.068983	0	0	0	0.525364	0
LOC107832330	18.52153	16.0065	17.60559	15.63105	13.5162	15.15908	13.48598	13.02483	13.8472	11.15611	12.14173	11.18052	15.00774	13.96547	10.66337	18.15724	16.2504	18.46524	10.22866	10.98587	9.588085	9.890106	13.99164	13.73531	4.964841	4.646589	7.674628	0	6.996586	7.647508
LOC107762093	0.682675	0.876699	0.836203	0.449795	0.466786	0.467065	0.026027	0.291648	0.233064	0.242071	0.239346	0.262156	0.103631	0.236971	0.063698	0.832467	0.959303	1.457485	0.062976	0.419997	0	0.28506	0.301082	0.194599	0.117697	0	0.293406	0	0	0
LOC107824293	0.057814	0.368834	0.15077	1.466551	2.609053	1.118834	3.491377	5.730181	3.636667	3.269822	3.324233	6.496137	12.67886	13.43446	9.612921	0.344666	0.055853	0	3.519958	2.200812	2.056377	2.655523	0.917929	1.148122	7.601865	9.653327	3.894916	33.07632	9.741853	0
LOC107817021	0	0	0	0.038424	0.03753	0	0.035573	0.036239	0	0	0	0	0.141643	0.09254	0.130594	0	0	0	0	0	0	0	0.074822	0.08866	0	0	0	0	0	2.622445
LOC107823765	3.567486	2.322374	2.362774	1.282525	0.701499	1.461137	0.379962	0.290301	0.637964	0.441746	0.158827	0.239199	0.441264	0.926655	0.232481	0.843967	2.844723	1.477618	0.287305	0.622731	0.258223	0.189162	0.749228	0.177559	0.357967	0.709128	1.204709	0	0.545243	0
LOC107823159	1.219627	1.053037	0.948596	0	0.0568	0	0.107679	0	0	0	0.225053	0	0	0.070029	0.131768	0.621857	0.930207	1.004998	0	0.054301	0	0	0.113241	0	0	0	0	0	0	0
LOC107800505	0.071014	0.181217	0.172846	1.376767	1.696617	0.244315	2.51353	3.859	2.346814	1.717336	2.868186	1.139894	1.644313	2.463627	6.370288	1.143062	0.082326	0.211775	5.289195	5.154244	4.378165	3.6888	2.142249	1.246956	6.913291	12.4106	2.16546	10.15698	3.145348	22.83262
LOC107782673	12.94355	8.623138	12.04114	15.01777	18.15115	20.10075	13.58627	16.49197	13.5022	12.54779	14.01211	16.88222	12.38664	15.11171	9.400433	7.163655	12.35831	9.875699	19.66293	15.49558	23.33334	11.82092	14.28803	15.26912	12.63231	6.529945	13.41951	32.47885	12.75451	9.360461
Novel01342	4.649167	5.366271	4.752002	5.403741	5.085851	4.435414	4.852895	4.507117	4.532721	3.910788	5.078075	2.622094	3.924293	3.845812	4.758668	5.806001	5.268859	5.389675	4.186269	3.857243	3.086899	5.098633	5.002163	5.112953	2.567571	1.883823	2.611607	0	2.33666	0.789739
LOC107803368	3.503825	3.899657	3.551095	3.539989	3.229004	3.471927	3.304467	3.545609	2.895406	3.731572	4.3137	2.97377	3.541013	2.624666	5.187189	5.161946	2.854623	4.068851	3.752054	4.589411	2.429794	3.762175	2.905454	2.750867	1.510657	4.133909	2.709935	0	2.254337	4.991861
LOC107805333	1.991427	1.838821	2.049993	2.302642	1.715773	2.141027	2.637308	2.574682	2.238647	3.091697	2.829526	2.8114	3.792038	3.630443	3.657587	5.506471	1.772	2.969386	2.07394	1.662444	1.481649	2.407107	2.519291	2.327925	1.689555	1.421922	2.291766	3.747767	4.667568	3.24034
LOC107780484	0	0	0	0	0	0	0	0	0.070656	0	0	0.06358	0.125668	0	0.077243	0	0	0.028054	0.038183	0	0	0	0.033191	0	0	0.052358	0	0	0	0
LOC107810012	17.75168	22.60997	20.23408	14.84313	13.36015	14.05661	18.94976	18.29918	16.51198	13.57605	17.37662	13.74928	14.18925	13.87564	15.21595	24.15386	19.71281	20.61666	11.87926	13.42016	11.94904	18.47972	16.6956	14.03704	5.671398	8.487114	9.213561	5.868241	12.64166	8.456187
LOC107787514	0	0	0	0	0	0	0	0	0	0	0	0	0	0	0	0	0	0	0	0	0	0	0	0	0	0	0	0	0	0
LOC107769086	11.13652	9.069004	7.840297	0.767234	0.412158	0.682878	1.136509	0.904501	1.590184	0.4542	1.039212	1.001659	2.78115	1.801622	1.173445	4.543911	7.199816	4.830117	0.128904	0.322382	0.772372	0.954794	1.643416	1.283486	0.053536	0	0.20019	0	0	0
Novel02940	3.698283	3.095384	3.083621	4.1026	3.272484	3.894961	3.608343	3.675817	3.401251	4.931053	3.59878	4.272155	4.6211	5.105061	3.873282	4.387046	3.718683	3.319926	3.523001	3.830808	4.818423	2.45822	2.396667	2.839918	3.148977	4.305745	3.032989	0	3.997007	0
LOC107813862	6.660702	4.662074	5.651047	5.117051	5.186568	5.786144	5.76539	4.962626	6.153307	6.598946	7.285962	6.167352	9.431608	7.964083	6.562908	4.963563	6.795147	8.342605	3.784939	3.831457	5.442906	5.384526	7.332224	8.465505	3.907415	1.70529	4.53451	11.43203	6.413398	6.58946
LOC107760537	6.682976	8.967781	7.466231	11.41817	10.97325	7.863815	13.15897	12.25487	10.05449	10.10618	12.27075	8.50417	11.84233	10.47484	15.36906	10.67938	7.446055	9.352071	10.0573	11.57876	8.354804	16.76328	11.66356	10.38418	11.58204	15.68045	8.644967	7.667283	10.55269	19.15095
LOC107791612	10.27636	10.32432	11.60589	12.04662	12.2436	11.57031	9.772979	9.517861	11.16725	8.311171	9.569893	11.73518	11.43979	10.94618	12.12533	11.35628	10.42292	12.82943	11.41304	9.719836	9.593603	10.40659	11.27701	11.64297	6.47917	6.004747	7.01334	1.928884	9.219574	5.003171
LOC107828941	0	0	0	0	0	0	0	0	0	0	0	0	0	0	0	0	0	0	0	0	0	0	0	0	0	0	0	0	0	0
LOC107778126	0.144496	0.065845	0.062804	0.130906	0.255719	0	0.181792	0.061731	0	0.211353	0.253302	0	0.080427	0.078819	0.370767	0.053839	0.069798	0.107728	0	0	0	0.241345	0	0	0.365373	0.201055	0	0	0	0
LOC107813304	0.057687	0	0.050146	0	0.10209	0	0	0	0	0	0	0	0	0	0	0	0	0	0.17561	0.146398	0.052611	0	0	0	0	0.080267	0	0	0	0
LOC107824478	2.326955	2.841782	2.366644	4.005397	3.418011	4.177942	2.634819	2.445503	2.687117	2.178312	3.132792	3.361649	2.046415	2.716295	2.268907	2.913204	2.248034	2.602266	4.746079	5.551012	3.947333	2.662322	2.709337	2.626704	1.588669	2.266452	2.557755	3.328204	1.456363	2.877583
LOC107782682	7.480432	8.632439	7.186298	9.82422	8.011131	7.214752	8.632235	7.864243	9.788152	8.175565	8.777343	8.836238	12.14634	9.913436	9.893426	11.02415	8.099754	11.27872	9.39064	8.196582	8.592715	8.315497	9.447139	8.063422	8.59007	7.590969	7.535847	1.196771	7.815002	13.45155
LOC107829879	1.86057	2.327967	1.425464	1.485597	0.837129	2.135956	0.899294	0.862222	1.243474	0.768767	1.171958	0.639401	1.158474	1.0665	0.582601	2.443997	2.132587	1.786816	0.479994	1.013707	0.316366	0.579387	0.834481	1.186576	0	0.131636	0.260903	0	0	0
LOC107797318	11.00227	9.976183	10.61049	26.50391	21.92353	32.98816	15.47405	17.2943	21.44734	35.78931	22.86957	39.47428	23.1245	25.68567	16.14846	8.574426	11.60278	10.08113	19.14699	19.86949	17.23441	14.98649	17.82904	25.10593	20.74144	31.27993	23.3936	8.008324	27.76484	13.84808
LOC107795563	0.171549	0.156346	0.24854	0.10361	0.050599	0.061479	0	0.048859	0	0	0	0	0	0	0	0.298291	0.386706	0.213162	0	0	0	0	0	0	0	0	0	0	0	0
LOC107825714	0.244385	0.890906	1.982758	0.8856	0.144165	1.751622	0	0	0	84.99574	39.01356	55.33205	15.32561	12.53074	0.501659	0.060705	0.629589	0	7.02623	24.94573	1.857357	0	0.071854	0	117.205	63.02136	529.3528	34.95391	37.25755	10.07378
LOC107776691	0	0	0	0	0	0	0	0	0	0	0	0	0	0	0	0	0	0	0	0	0	0	0	0	0	0	0	0	0	0
Novel01195	1.953011	5.304188	3.05588	4.034071	4.147567	1.76377	3.177865	3.57103	2.346973	2.666206	3.451021	3.068922	4.348239	1.53406	2.36533	5.967084	3.24525	3.057711	5.311173	5.286762	5.592902	3.196781	4.478981	3.837619	0.888912	0.923941	1.98514	13.96679	3.008797	2.415153
Novel00392	30.64844	22.27358	25.03433	19.2686	17.24208	25.84935	13.35169	13.93999	18.10784	16.16683	14.45064	20.42394	22.86806	23.77987	10.64376	2.608794	4.530682	3.545645	3.016156	4.805337	4.036156	2.371979	2.854878	4.004217	3.590958	0.919075	6.870066	18.8948	3.498011	0
LOC107829592	0	0.082878	0.158099	0.123577	0.201168	0.048884	0.038136	0.077699	0	0.044338	0.063765	0.038413	0.050616	0.24802	0.140003	0.033883	0.043926	0.067797	0.230692	0.038463	0.041468	0.037972	0.080213	0.047524	0.114972	0	0.10748	0	0	0
LOC107822670	0.655772	1.19531	1.496377	1.85655	2.103478	1.762587	1.787578	2.031121	2.154836	1.998313	2.58653	0.277007	1.186273	1.251976	3.028797	1.588216	0.554339	1.71117	1.247685	1.733555	0.747593	3.559738	2.530649	1.542178	0.932732	1.482745	0.581302	17.58635	0	0
LOC107830646	0	0.018921	0	0	0.018371	0	0.034826	0.017739	0.058474	0.080978	0.029115	0.017539	0.138668	0.045298	0	0	0	0.015478	0	0	0	0.052014	0.018312	0	0	0.086662	0	0	0	0
LOC107792989	0	0.06621	0	0	0	0	0	0	0	0	0	0	0	0	0	0	0	0.070185	0	0	0	0	0.064081	0	0	0	0	0	0	0
LOC107773222	34.17687	36.22648	41.98043	20.86604	17.42199	22.63236	13.86543	14.54792	16.97342	14.55027	13.19803	17.41829	15.85257	21.14183	13.98044	30.86688	36.78605	35.58934	16.58624	16.96966	14.28619	10.96178	15.51003	13.13677	6.418869	5.513579	9.732686	13.28331	12.8174	3.828274
LOC107811726	0	0	0	0	0	0	0	0	0	0	0	0	0	0	0	0	0	0	0	0	0	0	0	0	0	0.053394	0	0	0	0
LOC107808119	31.99344	27.61939	40.72619	6.959324	4.070949	12.75134	5.770431	7.176618	13.43337	4.156942	4.735371	3.280558	3.289034	5.986049	2.555929	30.35237	31.6831	41.1282	7.923451	7.962174	8.892146	8.21296	11.02008	16.05804	0.747087	0.646018	2.045331	0	2.235232	0
LOC107766826	0	0	0	0	0	0	0	0	0	0	0	0	0	0	0	0	0	0	0	0	0	0	0	0	0	0	0	0	0	0
LOC107774954	0	0	0	0.171312	0	0.406604	0.095162	0.258511	0.071013	0.147514	0.053038	0.223656	0.126303	0.123777	0	0	0	0	0.115129	0.25594	0.137967	0.063168	0.066718	0.079057	0	0	0.044699	0	0	0
LOC107795114	0	0	0	0	0	0	0	0	0	0	0	0	0	0	0	0	0	0	0	0	0	0	0	0	0	0	0	0	0	0
LOC107766878	0	0	0	0	0	0	0	0	0	0	0.054656	0	0	0	0	0.058086	0	0	0	0	0	0	0	0	0	0	0	0	0.37526	0
LOC107828660	0.639487	0.211932	0.505356	0	0.051442	0.125005	0	0	0	0	0.040764	0	0	0	0.119337	0.086645	0.056163	0.476764	0.058992	0	0.10604	0	0.051279	0	0	0	0	0	0	0
LOC107762395	1.034297	0.942634	1.070348	0.803161	0.958796	1.429709	0.66096	0.631237	0.878563	0.288163	0.535303	0.353682	0.356384	0.59105	0.278031	1.192842	1.189545	1.248468	0.549756	0.687456	0.78608	1.151691	1.672584	1.595831	0.342484	0.102796	0.436591	0	0.355677	0
LOC107825723	38.37579	33.94481	40.83413	34.05428	30.43559	41.20584	21.88415	23.93074	31.50797	15.23682	19.19127	19.09556	16.51931	15.86731	13.16291	30.02574	33.70386	33.55631	18.59799	19.57773	20.61422	23.88259	31.42284	40.6753	4.845654	2.939918	10.10517	10.54191	7.806538	15.19099
LOC107831371	17.69409	15.68581	17.48025	7.398436	6.293837	10.99859	7.033725	8.478256	10.84107	5.908307	6.403666	7.270216	6.891612	7.75967	6.038553	10.76447	16.28805	14.82838	4.187953	4.382684	5.005367	8.506739	9.759437	12.66577	1.165719	0.916378	3.891995	6.279788	2.959302	0
LOC107812849	14.68361	4.723164	6.328444	4.639144	3.493922	5.173784	0.724457	1.26515	1.911451	2.526772	2.941759	5.577092	1.236252	1.615371	1.013167	5.103315	6.37756	5.381633	1.815535	2.087617	4.220073	0.978952	2.067953	4.965304	0.468014	1.545214	1.53131	0	0	0
LOC107800934	361.5684	332.6457	390.6473	192.4965	182.1207	359.0805	160.9071	182.8142	219.5562	112.0804	119.7252	130.3319	76.75064	123.6768	74.37276	208.7531	356.2791	274.5323	128.1831	132.6942	156.3115	157.2764	234.4405	260.9686	68.096	16.22475	136.8335	59.56368	38.76185	28.6106
LOC107770322	3.922353	2.048029	6.286484	4.108681	11.42457	3.689883	3.872424	10.82212	8.55409	16.45456	12.69175	21.36655	13.82699	19.56794	20.29672	4.749781	2.170966	4.782418	29.93392	26.23326	44.04492	10.68002	5.87441	11.48754	66.06897	70.66544	53.89241	75.96976	62.55194	73.26277
LOC107826821	1.808911	4.157337	3.555099	1.211273	0.278373	0	34.82989	30.44125	16.02295	18.63616	69.59707	1.860439	52.88163	41.87109	37.61666	0.99635	0.987751	0.527721	0	0.266124	0	27.19203	4.16238	0	0.696047	0.3283	0.185912	0	0	0
LOC107814954	0.526959	0.528284	0.229037	0.907057	0.466288	1.019782	0.132595	0.045025	0	0.154156	0.147802	0.133557	0.175986	0.517398	0.594942	0.196345	0	0.235722	0	0.044577	0.144179	0.220039	0	0	0	0	0	0	0.253697	0
LOC107819062	16.39024	11.2583	15.05756	16.75562	15.93821	25.52337	14.67333	14.09272	19.69649	9.690476	9.774969	11.54397	16.32523	14.94471	9.456013	9.436943	13.73422	11.96237	9.103659	8.377389	11.04592	11.2385	16.58782	15.68846	3.751818	1.248319	5.057098	4.935301	4.485323	0
LOC107781417	2.316112	1.558782	1.115084	38.41467	63.87972	45.62636	29.43821	41.77104	19.26913	56.94899	38.97742	53.34308	57.71488	74.8311	58.44239	2.230483	2.271983	1.779891	99.90287	85.69551	81.08109	33.50708	20.99538	29.90618	128.1231	138.5761	70.45796	198.7617	114.9368	288.6206
LOC107785982	74.32092	67.04083	66.36957	43.66173	42.9823	67.0867	33.18922	41.07028	38.69816	33.51534	40.64171	41.89464	23.86046	31.54667	36.18789	48.57917	61.50807	51.53263	31.78786	34.22466	38.17071	33.78751	35.68661	34.99586	14.91241	7.764734	24.13389	72.55968	12.21186	7.841936
LOC107796785	0.405259	0.46168	0.228984	0.403859	0.14344	0.413918	0.424886	0.363578	0.380475	0.079036	0.184709	0.154068	0.22557	0.198953	0.332758	0.603999	0.254484	0.468314	0.390668	0.342821	0.221762	0.693807	0.571945	0.593008	0	0.056389	0.191594	0	0.097553	0
LOC107810869	1.190449	0.834575	0.557217	3.401381	4.53767	2.559757	3.917118	2.816729	2.149319	5.714896	5.457939	4.177625	9.276572	3.696361	5.639282	1.433047	1.503943	2.048147	5.854084	3.176043	3.841742	2.676625	2.423204	3.062787	6.599239	5.224104	2.922262	0	16.31201	22.64839
LOC107803718	11.2137	10.81542	13.77888	3.800092	3.152109	3.580569	1.962407	1.728954	3.067343	2.075962	2.276146	1.175301	1.431349	1.609683	0.995176	12.53469	8.837751	13.10616	2.160277	2.906429	1.614802	3.080543	3.253691	2.996231	1.172577	0.938529	1.320386	3.014804	2.334012	1.303308

LOC107815095	7.776908	7.018205	6.925995	24.34837	21.18429	20.57493	11.44692	11.75869	13.67205	15.09259	13.36562	21.06312	13.70749	14.93057	7.551603	6.84648	8.176072	7.44647	14.19691	17.83352	20.33928	8.850619	12.47532	14.70288	12.00128	7.850529	27.12447	5.452538	8.626067	0
LOC107829726	24.01464	24.22569	21.2536	16.98706	12.54966	17.96082	21.49222	18.17695	17.80808	12.1753	13.48347	12.38612	14.6259	14.57071	11.5649	22.04548	20.51029	22.54209	14.96538	14.90483	15.51374	17.40302	20.1464	13.32306	6.38038	7.385116	6.324556	6.222415	6.911768	5.379933
LOC107832619	1.606741	2.123302	2.3744	1.892314	2.772413	0.950094	3.032197	3.226182	1.734751	1.880131	1.351977	2.375484	1.967511	1.4023	4.122788	2.514425	1.785084	1.677051	3.34234	2.650425	2.124794	3.220396	3.259679	1.847302	3.148684	7.936604	1.899039	0	2.320627	4.967361
LOC107810552	4.171459	7.289993	13.96259	13.188	13.81339	7.883126	12.57037	12.52984	12.89973	19.41592	18.45483	13.37003	11.60932	11.09565	12.00577	22.46507	4.694719	13.70644	18.24078	15.67937	20.66949	13.55768	15.6284	14.18126	41.10452	35.06504	43.02601	12.30179	26.50113	35.89714
LOC107788556	0	0	0	0	0	0	0	0	0	0	0	0	0	0	0	0	0	0	0	0	0	0	0	0	0	0	0	0	0	0
LOC107760821	0.235098	0.128558	0	0.63896	0.249636	0.30331	0.276061	0.321398	0.044144	0.04585	0.09891	0	0.157028	0.410368	0.820413	0.140156	0.09085	0.035055	0.811107	0.357978	0.686122	0.589009	0.207372	0.147434	0	0.196273	0.111147	0	0.226369	0
LOC107776792	0.210784	0.16809	0.251941	0.02387	0	0	0	0.045025	0.049473	0	0	0	0	0.028744	0	0.098173	0.127272	0.216079	0.026736	0.022289	0	0.022004	0.046481	0.027539	0	0	0	0	0	0
LOC107807878	5.547404	6.148229	6.421577	2.90428	2.417354	5.094992	1.613299	1.88165	1.439438	1.277602	1.544209	0.565218	1.148199	1.398942	1.545025	5.442664	3.635678	5.549072	1.697223	2.287407	0.584746	1.699463	1.868751	1.602502	0.105733	0	0.296529	0	0.671035	0
LOC107761196	0	0	0	0	0	0	0.053059	0	0	0	0	0.106889	0	0	0	0	0	0	0	0.107028	0	0	0	0	0	0	0	0	0	0
LOC107786484	11.55967	9.876764	10.12708	4.745341	3.596048	5.534371	4.696304	3.935326	3.561052	6.780896	5.509308	5.569633	5.529385	4.236519	3.429591	7.201	9.335428	6.396339	2.107726	2.139088	2.635654	2.790547	2.788077	3.020541	9.362695	2.890173	13.6626	0	15.65223	5.583999
LOC107810384	0.858685	1.135516	1.346513	2.18124	1.81761	1.918789	2.513694	2.388068	1.691378	2.364214	3.376539	1.778041	1.780597	1.212307	3.974627	2.722681	1.07355	2.058637	2.767766	3.204653	1.84267	2.418488	1.24751	1.407838	1.745536	2.413021	2.009892	1.20408	1.621182	3.123163
LOC107802892	5.556607	5.312058	4.762685	3.027443	2.750686	6.015808	2.86804	2.656065	2.042938	3.031269	3.542087	3.249952	2.595395	1.950009	2.113754	5.270096	6.344177	5.851906	2.917805	3.911611	2.728772	3.18018	4.729921	4.02076	1.817719	0.97321	2.6178	0	2.619026	0
LOC107813406	11.57354	14.45997	15.58689	10.14046	9.712147	9.814171	10.16296	9.610188	11.73297	7.682726	9.486983	5.416724	3.750221	5.038608	7.417254	15.26516	14.17311	13.93533	6.06499	7.170445	4.7573	14.47536	10.11277	13.914	3.434852	2.494944	3.789016	0	2.354329	3.359668
LOC107810832	4.005511	3.439922	4.151503	2.721593	3.271698	2.650101	4.716354	4.6071	4.122142	5.032575	4.915167	4.815646	6.774236	8.235422	6.324841	3.386727	3.051079	2.813993	2.501241	0.977419	2.107554	1.929869	3.329292	2.09328	2.337316	0.321541	1.365632	5.508667	1.483381	0
LOC107773209	0.03284	0.119718	0.057094	0.357016	0.174354	0.035307	0.413165	0.561187	0.493306	0.384277	0.138164	0.194209	0.329021	1.289765	2.460542	0.244724	0	0.073451	0.299914	0.250023	0.029951	0.713064	0.057934	0.205946	0.871914	1.416527	1.125612	2.348523	0.790517	4.06109
LOC107820673	0.887434	0.924328	0.789795	1.761079	1.177891	1.999068	2.463376	2.184477	1.309255	1.998571	3.733617	0.321313	1.458335	0.437971	3.751787	1.22816	0.469494	0.99243	1.093473	1.876771	0.809355	3.98793	3.093828	1.833008	0.454138	0.7644	0.174813	0	0.508622	2.612924
LOC107803400	2.131879	3.411449	3.361613	0.920771	0.723861	0.666288	0.956425	0.614239	0.930931	0.290072	0.677908	0.418854	0.469128	0.513834	0.432532	2.697059	2.418802	2.328665	0.477935	0.754921	0.271299	1.32494	1.596203	1.165939	0.094024	0.13797	0.205093	0	0.238689	0
LOC107794256	0	0	0	0.156097	0	0	0	0.147219	0	0	0.120818	0.291131	0	0	0	0	0	0	0	0	0	0	0	0	0	0	0	0	0	0
LOC107821832	23.76294	14.46364	18.01492	21.45154	16.99341	31.85567	8.744151	6.094716	10.69914	10.78335	11.21691	20.89573	9.867103	9.577679	5.1985	15.85243	30.6637	24.85943	6.595737	7.212372	13.1265	5.287314	10.61055	12.74937	1.173994	1.11585	2.294761	0	2.844841	0
Novel04449	0	0	0	0	0	0	0	0	0	0	0	0	0	0	0	0	0	0	0	0	0	0	0	0	0	0	0	0	0	0
LOC107780112	0	0	0	0	0	0	0	0	0	0	0	0	0	0	0	0	0	0	0	0	0	0	0	0	0	0	0	0	0	0
LOC107803872	0.411262	0.425925	0.276251	0.7621	0.479701	0.341666	0.689885	0.543062	0.421211	0.729149	0.353917	0.394824	0.790784	0.754575	0.690721	0	0	0	0	0	0	0	0	0	0	0	0	0	0	0
LOC107808806	2.486202	1.956885	1.866493	2.917847	1.899961	1.943979	1.848321	2.027721	2.387205	3.361046	2.416885	3.532512	3.270889	4.006843	2.029817	2.02115	0.927999	1.516557	2.924185	2.150955	2.885856	1.415654	1.844109	2.244226	2.428926	3.066259	4.407737	0	3.264399	3.493764
LOC107798664	5.817991	7.612007	5.398756	6.871448	5.700855	5.928868	7.439449	6.816132	5.881065	5.011973	6.457247	5.231823	5.741586	4.906379	7.217	7.341216	4.896533	6.426506	4.99822	6.144476	4.671326	6.55783	5.95043	5.185606	4.783467	5.040944	3.311639	2.552568	4.467833	4.413928
LOC107799782	0	0	0	0	0	0	0	0	0	0	0	0	0	0	0	0	0	0	0	0	0	0	0	0	0	0	0	0	0	0
LOC107765378	0	0	0	0	0	0	0	0	0	0	0	0	0	0	0	0	0	0	0	0	0	0	0	0	0	0	0	0	0	0
LOC107817212	4.871102	4.776579	3.88595	5.306738	4.746755	4.77299	5.637098	5.558116	5.788867	2.916102	3.480473	2.604584	2.539689	3.22884	3.860424	3.698874	3.842154	3.562659	2.87812	3.129583	2.755491	4.994867	5.302808	5.381284	0.584673	0.471871	0.910957	0	0.148425	1.90625
LOC107800872	6.925341	3.255981	4.22837	2.21582	2.042666	2.215949	1.867044	2.301133	3.354111	4.609246	4.200872	6.361498	4.58892	5.366595	5.274573	3.542911	4.301011	3.954306	2.704975	2.487479	2.531424	3.190123	2.327089	2.58513	3.405009	6.19464	5.586721	0	3.043033	1.699223
LOC107762375	1.836966	1.530668	1.41434	0.713228	0.928839	1.072123	0.704339	0.493288	1.231874	0.307075	0.368023	0.532087	0.467414	0.515324	0.269344	1.016904	1.419731	0.93911	0.479321	0.399586	0.718004	0.525976	0.879605	1.097141	0.066357	0.073029	0.186098	0	0	0
LOC107777342	9.957687	9.552846	10.73142	6.910947	5.771089	7.825827	7.033213	7.612526	7.899938	4.343948	5.124463	4.624432	4.278435	3.716408	4.452702	8.179886	9.535577	9.963626	3.988568	4.507321	3.372399	7.975506	9.271301	9.464377	1.840576	1.336924	1.995944	0	0.700875	1.800289
LOC107790965	0.508541	0.463473	0	0	0	0	0	0.289674	0.318294	0.165297	0	0	0	0.554794	0.521953	0	0	0	0.172011	0	0.154599	0	0.149522	0	0	0	0	0	0	10.48129
Novel00143	0	0	0	0	0	0	0	0	0	0	0	0.004929	0	0	0	1.708828	1.166857	1.814014	0.811155	0.745323	0.35122	0.482414	0.674225	0.585468	0.280329	0.121782	0.441367	0	0.337095	0
LOC107771945	1.414819	0.859622	1.257203	1.053887	0.639873	1.487302	0.738372	0.859634	0.796977	0.551851	0.925934	1.195279	0.839998	0.617399	1.387593	1.124613	1.306086	1.218889	0.414747	0.877683	0.688181	1.260323	0.748779	0.952984	0.198751	0.131241	0.483081	0	0	0
LOC107779355	0	0.03692	0	0	0	0.043553	0.033978	0.103839	0.038033	0.039503	0	0	0.13529	0	0	0	0	0	0	0	0	0.033831	0.071465	0	0	0	0	0	0.585092	0
LOC107819380	0.38973	0.994535	0.64369	0	0.068972	0	1.765172	1.931378	0.804971	0.722069	2.842103	0.230479	3.90468	2.721131	2.040044	1.074582	1.242491	1.278466	0.079094	0	0.17772	1.562275	0.893797	0.366611	0.492738	0.271141	0.276378	0	0.18763	0
LOC107802196	6.371852	6.228948	6.673998	8.116046	8.08805	6.041963	7.708059	6.12217	6.967566	6.455125	8.617168	5.781091	8.050039	7.510372	8.779235	9.059359	5.988078	8.416555	5.568398	5.690766	4.838928	9.055182	7.246008	7.635946	4.722818	4.565225	3.565267	3.546128	6.684344	5.110002
LOC107822380	0.281612	0.07333	0.069943	0.546698	0.284787	0.129757	0.2362	0.27499	0.11331	0.196148	0.282095	0.13595	0.537418	0.351114	0.495494	0.149898	0.155463	0.059987	0.28576	0.136128	0.293525	0.201584	0.177429	0.168195	0.15259	0.111955	0.237744	0	0	0
LOC107811218	0.054308	0.049495	0.070813	0.0492	0.144165	0.029194	0	0.023201	0	0.079435	0.01904	0.045881	0	0.148118	0.02787	0.08094	0.026233	0.020244	0.165323	0.02297	0.148589	0	0.047903	0.028381	0.034331	0.075565	0.224656	0	0.130728	0
LOC107777188	8.472208	9.498514	8.358362	10.72118	9.698199	14.74735	7.444387	9.479491	7.890954	9.244981	10.41983	13.17899	7.110961	6.822039	9.661824	11.224	9.224213	10.22654	18.08102	18.31542	21.46332	6.12075	12.75163	12.0177	14.62197	12.06914	24.15948	24.04296	8.740334	12.4726
LOC107769070	0	0	0	0	0	0	0	0	0	0	0	0	0	0	0	0	0	0	0	0	0	0	0	0	0	0	0	0	0	0
LOC107827818	3.771912	3.632216	3.773763	1.902005	1.889228	3.328379	1.731062	1.793836	1.302908	0.902173	1.147773	0.901873	1.46567	1.125794	1.351333	2.996454	3.472098	3.687592	1.697088	2.197423	1.720024	1.396708	1.694925	2.268741	0.404902	0.495126	1.135553	0	1.199201	0
LOC107790041	0	0	0	0	0	0	0	0	0	0	0	0	0	0	0	0	0	0	0	0	0	0	0	0	0	0	0	0	0	0
LOC107774201	0.271946	0.357998	0.223263	0.410615	0.374322	0.259889	0.91237	0.968157	0.510629	0.721883	1.324226	0.765824	0.824109	0.692252	1.069951	0.574187	0.437871	0.382967	0.536573	0.664581	0.716499	0.706561	0.533054	0.505311	0.573037	0.567589	0.178566	0	1.091036	1.868312
LOC107777680	5.115211	6.260244	5.61535	2.462705	2.250205	2.41977	4.069701	3.196764	4.034004	1.824174	2.828439	1.629812	2.342808	2.869943	2.580055	5.794029	4.969962	6.188939	1.186414	1.607215	0.853058	5.004163	3.403303	2.505178	0.147821	0.36604	0.414567	0	0.703613	0
LOC107806171	0	0	0	0	0	0	0	0	0	0.035153	0	0	0	0	0	0	0	0.026876	0	0.091486	0	0	0.031798	0	0.045577	0	0	0	0	0
LOC107830462	0	0	0	0	0	0	0	0	0	0	0	0	0	0	0	0	0	0	0	0	0	0	0	0	0	0	0	0	0	0
LOC107789428	1.105058	1.812824	2.377494	1.751972	3.715747	1.158371	2.409836	4.201653	3.683045	3.663743	4.70732	3.664317	3.659745	4.430449	7.797635	4.982119	1.414542	2.883537	9.811697	9.207812	9.272052	8.997914	5.287934	5.25528	15.99713	30.29088	8.456932	31.61092	19.15252	39.28826
LOC107802408	5.488097	4.80254	5.78393	4.773941	5.457886	5.84816	4.440162	4.460945	5.289259	4.617521	6.027801	5.641755	8.02876	6.490597	5.607909	4.632622	5.677324	5.377027	3.572994	4.067369	3.609972	5.435009	4.969383	6.066114	2.671063	2.162488	3.444154	0	4.325669	3.002985
LOC107827741	13.206	8.686273	10.11056	20.78145	20.11914	25.26995	13.7524	12.21524	12.58795	21.26563	22.08821	25.58898	20.99515	19.20946	15.37809	11.13522	13.57743	14.45231	22.08835	19.43886	21.50994	12.51266	13.35845	14.81596	18.58559	13.82353	24.58208	43.32182	29.1644	22.47377
LOC107767008	0	0	0	0	0	0	0	0	0	0	0	0	0	0	0	0	0	0	0	0	0	0	0	0	0	0	0	0	0	0
LOC107779882	13.82797	13.42726	13.24757	31.12183	44.87563	31.17349	24.01599	29.44469	22.36219	57.92478	37.99785	49.94009	45.21333	45.49385	32.73226	10.22369	13.21909	11.44282	48.89044	43.14605	46.68176	23.33804	20.53086	20.09041	123.4319	80.58893	99.30201	82.83939	126.5231	82.81453
LOC107806710	0.993766	0.792483	0.719883	0.637714	0.84271	1.335525	0.625136	0.955238	1.049615	0.605655	0.754899	1.01447	1.198464	1.355188	0.424989	0.647986	0.840054	1.080472	0.840338	0.80563	0.302111	0.48412	0.986138	0.995406	0.785264	0.230459	0.342578	0	0.797391	0
LOC107821994	2.436268	8.344248	2.869272	19.15224	31.08482	11.19534	23.82871	31.19063	9.296669	20.19319	17.30355	20.11751	18.94087	12.34614	29.52225	12.18148	3.112878	2.695216	61.32543	51.19047	49.59893	36.58982	19.47919	15.0319	46.20275	75.23367	13.42206	84.30368	41.05171	77.74871
LOC107796773	276.6413	252.3024	299.8448	136.611	129.5009	190.5146	110.5494	116.7459	145.4669	73.4974	92.76313	81.90515	66.24394	84.51736	75.3057	193.2756	288.481	242.606	83.51649	89.65173	95.49793	124.8714	155.5725	173.9873	11.23277	13.92445	39.34922	20.94628	14.10111	15.09189
LOC107795626	4.870894	4.396329	4.21371	6.800376	7.724843	6.855916	7.006412	6.634792	5.765969	6.998405	6.682449	6.699408	7.701305	5.750314	6.955621	5.067686	6.024197	5.315613	6.446931	7.882584	6.073341	6.484916	6.807903	8.140773	6.902046	5.107677	5.812628	6.731165	8.723028	4.364851
Novel04903	1.079777	0.540281	0.625751	0.537064	0.580768	0.500777	0.479465	0.5427	0.795092	0.784528	0.979828	0.965886	1.178454	0.785322	0.630183	0.993981	1.063609	0.947082	0.687487	0.716405	1.11994	0.831024	0.728332	1.084324	0.401519	0.206216	1.176114	0	0.91737	0
LOC107813955	0.065189	0.14853	0.198337	0.029529	0.057684	0.070087	0.027339	0.08355	0.091804	0	0.114277	0.082611	0	0.071119	0.267635	0.072869	0	0.024301	0	0.055146	0.029727	0.081662	0.057501	0.034068	0	0	0.038524	4.661971	0	0
LOC107816409	1.474687	1.628303	1.060045	0.436767	0.150565	0.213429	0.285435	0.048462	0.026625	0	0.099428	0	0	0.495019	0	0.993272	1.616455	1.28973	0.028777	0.04798	0.155185	0.615775	0.575341	0.088924	0.07171	0	0.033518	0	0	0
LOC107827963	0	0	0	0	0	0	0	0	0	0	0	0	0	0	0	0	0	0	0	0	0	0	0	0	0	0	0	0	0	0
LOC107771489	1.293327	1.581951	0.917161	0.246671	0.180698	0.182959	0	0.116321	0.09586	0	0.071596	0.086261	0.340993	0.445565	0.244528	3.195722	1.545389	1.623967	0.069073	0.115165	0.124162	0.056847	0.060042	0.142293	0.129092	0	0.080453	2.433979	0	0
LOC107783696	87.64171	96.81164	97.24671	50.44208	40.25135	59.3491	50.02047	61.98008	47.76549	35.95755	45.87877	38.49201	26.76787	34.67014	60.45778	73.99695	86.97102	80.04294	55.27147	47.90654	46.16659	58.55963	67.08888	45.93564	19.41766	64.16118	32.40565	76.01883	7.235247	24.93066
LOC107775010	0	0.050731	0.096775	0	0.049255	0.059846	0	0.047561	0.05226	0	0.039032	0	0.123932	0	0	0	0	0.083	0	0	0	0	0	0	0	0	0	0	0	0
Novel02008	0	0	0	1.066077	0.812696	0.216001	0.505532	0.416893	0.511974	0.251886	0	0.072743	0	0	0.029458	0.064165	0	0.085592	0.902845	0.971171	0.889984	0.455414	0.506328	0.539973	0	0	0.101768	0	0	0
LOC107790332	1.369795	2.415381	1.811986	1.348875	1.870826	1.568742	0.674358	0.966844	0.531184	0.464599	0.709935	0.301889	0.861886	0.487297	0.550143	1.88621	1.639787	2.79731	1.51084	1.637367	0.760429	0.845529	0.682924	0.995972	0.489432	0.745814	0.211172	0	0.860176	0
LOC107788011	0.136051	0.049598	0.21288	1.947439	3.635692	0.789868	1.620392	3.929113	1.558318	3.582015	8.490536	3.034414	2.605013	2.137325	9.774755	1.257182	0	0.365155	7.123663	9.046079	8.13972	1.59068	2.784145	1.592649	5.366719	10.10891	2.926591	9.729594	4.715997	25.23678
LOC107825399	0	0	0	0	0	0	0	0	0	0	0	0	0	0	0	0	0	0	0	0	0	0	0	0	0	0	0	0	0	0
LOC107788833	4.599657	3.89259	4.426782	2.806384	2.221931	2.220291	2.716298	2.74704	3.613322	1.739177	2.05694	1.546437	1.80259	1.38251	1.035721	3.14786	3.468771	3.429239	1.619307	1.68742	0.920324	3.547317	4.429801	6.033978	1.038458	0.457149	1.303632	0	1.129815	0
LOC107809542	12.6362	10.07205	21.82375	13.29904	12.76813	21.70087	8.534786	6.378256	14.44753	2.887308	3.743054	3.558471	2.599804	4.504165	1.498121	5.6872	5.841936	7.15112	6.474649	6.279556	9.774844	3.099674	5.002809	10.89716	1.107247	0.116055	2.563098	0	2.810866	2.578598
LOC107829892	0.320226	0.166769	0.218716	0.165776	0.161918	0.245915	0.057554	0.214979	0.064423	0.289956	0.096232	0.212564	0.458331	0.299443	0.117382	0.374994	0.309365	0.409272	0.301733	0.25154	0.354634	0.611266	0.363163	0.239071	1.243506	0.127306	0.486618	0	18.05968	1.414288
LOC107826214	18.40629	19.15904	19.68828	15.28797	15.72255	17.31967	16.05353	20.851	19.67656	11.91379	13.37623	12.42394	11.39919	12.38927	10.21527	16.52147	17.96693	18.76462	12.00923	13.35767	13.38293	19.31437	21.86314	18.97151	10.52646	9.276776	13.53545	13.6878	7.064581	3.944847
Novel00027	4.888778	5.81339	4.148527	4.323533	3.563728	2.853276	3.553705	3.659939	4.043395	4.60827	6.121454	4.189178	6.323383	5.409608	3.153988	3.799257	4.543048	4.113407	2.409528	2.343489	1.549911	1.827515	3.039092	3.382167	1.501076	0.971768	1.595868	1.664842	3.250253	7.197155
Novel02757	16.57587	11.22224	12.67997	10.89798	11.56631	9.979811	15.25347	10.52099	13.25007	12.00722	12.15435	11.20301	18.55794	16.84345	15.55476	36.56299	29.55669	34.03751	21.62578	17.78797	20.30061	24.9964	31.49785	28.90816	20.71744	23.72309	19.14355	74.51145	58.82559	87.84954
LOC107824374	4.881741	6.13255	6.193353	13.80572	14.19951	10.40828	11.32911	11.1464	8.005105	12.6084	12.60526	10.85397	14.76107	11.98291	18.53541	6.857894	6.245742	6.934809	17.1537	17.00677	12.39405	11.88628	8.859342	7.860445	14.03297	19.66637	8.108943	8.256063	15.00662	20.39496
LOC107800219	0.844625	0.817142	0.982718	0.541517	0.517417	0.516904	0.294268	0.399695	0.573379	0.430815	0.656033	0.296403	0.506288	0.453634	0.34676	0.600366	0.67789	0.804084	0.21097	0.252821	0.059255	0.618554	0.492856	0.285213	0.131429	0.072322	0.046074	0	0.312794	0
Novel00496	23.78874	21.04285	16.80864	12.9366	8.21732	8.616449	10.21639	8.423773	12.92859	19.81671	23.7499	26.2502	39.08568	31.53835	12.73033	16.11582	28.48161	19.65608	3.420792	3.255295	5.626969	6.347751	7.966901	8.343397	0.844385	0.26551	3.90923	0	2.296674	0
Novel00467	0.303007	0.717998	0.509235	1.098035	1.429975	0.10859	1.711242	1.501604	0.872393	2.324363	2.974559	0.409582	1.978893	2.093581	7.194416	5.118155	0.351276	0.783135	2.27528	2.35817	0.829042	1.568903	0.873088	0.274476	2.528411	4.272332	0.668509	0	2.528556	6.245121
LOC107792551	0	0	0.033529	0	0	0	0.161757	0.032956	0	0	0	0	0	0	0	0.028743	0	0	0	0	0.035178	0.032212	0	0	0	0	0	0	0	0
LOC107775578	0	0	0	0	0	0	0	0	0	0	0	0	0	0	0	0	0	0	0	0	0	0	0	0	0	0	0	0	0	0
LOC107812876	0	0.042097	0	0	0	0	0	0	0	0	0	0	0	0	0	0	0	0	0	0	0	0	0	0	0	0	0	0	0	0
LOC107771301	0.955399	1.001338	0.934322	2.769738	3.00117	1.643474	5.509174	9.428485	5.157582	5.356901	4.789975	5.024495	4.546735	7.999603	13.5077	0.622969	0.461498	0.712291	8.458653	6.990947	6.055814	7.320538	5.351306	3.794615	20.53456	43.83592	11.91311	18.78925	8.509319	32.49057
LOC107829776	0.219468	0.066673	0.190779	0.132551	1.100466	0.078652	0.122718	0	0.343411	1.640738	0.256485	5.995017	3.01322	1.356765	0.375427	0.163548	0.2827	0.272705	1.707376	1.052045	2.735503	0.244378	0.387171	0	3.144712	0.101791	6.052523	0	20.77977	0
LOC107786871	2.693286	3.978141	2.664142	2.187569	4.602034	4.193644	3.193723	2.380569	1.918229	2.173484	4.819017	1.098451	3.308362	2.938242	5.909914	5.398244	4.037494	4.015917	2.07328	2.985404	0.677606	3.800428	2.785252	1.261906	0.587092	0.387673	0.439069	0	0	0
LOC107790711	5.078576	3.885233	4.156905	5.776353	7.085206	6.576021	5.969687	5.986296	6.055696	6.868087	4.938752	7.860737	7.758144	8.573584	6.125651	3.480691	4.61983	4.228489	7.259882	5.456393	8.384475	5.231908	5.820262	6.392998	8.904847	8.768609	10.2962	26.51022	11.77887	2.292087
LOC107823152	0.490727	0.272231	0.204016	0.850489	0.717419	0.940489	0.984246	1.075571	0.88137	0.644963	0.763	1.063487	1.591349	0.884506	0.722655	0.238494	0.329797	0.55674	0.671158	0.487316	0.544845	0.69491	0.884527	1.048116	0.107901	0.178125	0.327826	1.525822	0.616313	0
LOC107806194	0	0	0	0	0	0	0	0	0	0	0	0	0	0	0	0	0	0	0	0	0	0	0	0	0	0	0	0	0	0
LOC107762685	0.150863	0.137493	0	0.136675	0.186891	0.097318	0.075921	0.051561	0.028327	0.029422	0.190414	0.025491	0.033589	0.098751	0.092905	0.044969	0.029149	0.022495	0.153086	0.102096	0.110072	0.100792	0.026614	0	0.038147	0.041983	0	0	0	0
LOC107820065	17.3894	23.57964	17.13909	29.26549	32.66523	23.29006	30.62546	28.90256	24.3896	36.22415	43.1518	18.44424	38.29235	30.81054	43.70808	33.48428	19.68131	27.8848	41.68819	36.73981	21.91765	35.29884	28.94182	21.89008	56.38129	58.22367	32.05449	19.66745	71.90445	70.63443
LOC107770567	0	0	0	0	0	0	0	0	0	0	0	0	0	0	0	0	0	0	0	0	0	0	0	0	0	0	0	0	0	0
LOC107791562	0	0	0	0	0	0	0	0	0	0	0	0	0	0	0	0	0	0	0	0	0	0	0	0	0	0	0	0	0	0
Novel02455	0.175519	0.11426	0.021796	0.113579	0.088749	0.404366	0.084123	0.128544	0.258947	0.073351	0.070328	0.06355	0.055826	0.109418	0.180147	0.280279	0.145342	0.411263	0.254434	0.042422	0.137208	0.23034	0.176936	0.393111	0.158506	0.034889	0	0	0.120716	0
LOC107784333	5.453331	6.563405	4.347942	9.985775	11.27991	8.078737	11.42029	10.14004	8.620006	20.77397	23.2599	17.70466	17.25838	16.06694	21.41415	8.722995	6.263886	6.845205	13.29959	14.16264	8.584882	10.37511	8.241677	7.914399	18.32723	22.61874	14.87505	9.108052	15.21745	12.88615
LOC107831965	28.66256	41.99857	31.81779	30.63311	25.37766	25.83004	31.07328	28.54007	30.05851	25.74702	26.19366	23.20296	27.43098	23.5781	29.45359	53.16253	40.18834	44.25648	25.03896	25.69411	21.45882	30.02467	30.39911	29.33211	18.34626	21.90526	18.18226	19.57997	19.52481	20.10313
LOC107806342	28.3354	31.37882	30.68805	27.92385	23.30968	31.90277	21.60667	18.18276	21.44043	19.94282	27.44709	16.51611	21.32577	19.91541	23.12581	34.28571	33.96503	38.56333	21.47604	25.06493	14.49985	26.23894	27.7779	26.6819	9.140044	15.42254	12.98002	11.44261	12.04501	8.09455
LOC107810597	0.723027	0.811633	0.60935	0.962568	1.240552	0.739423	1.127819	0.945495	0.662253	0.98448	1.43131	0.588486	1.153339	1.159132	1.307716	0.565083	0.357771	0.394425	0.662099	0.604172	0.172895	0.2025	0.229437	0.281086	0.34001	0.226972	0.302222	0	0.679202	0.81779
LOC107774599	1.900112	2.174268	1.633375	2.582108	2.391245	2.451424	2.248896	2.146638	2.140691	1.852849	2.561092	2.033339	2.326755	2.34932	1.755201	1.667693	1.570516	2.01474	1.970949	2.393187	2.118031	2.239198	2.253322	2.250796	1.334622	1.380686	1.646905	4.529496	1.219709	1.305409
LOC107763037	0	0	0	0	0	0	0	0	0	0	0	0	0	0	0	0	0	0	0	0	0	0	0	0	0	0	0	0	0	0
Novel02250	0.124912	0.037947	0.144778	0	0.184217	0.223826	0.209538	0.213456	0.117273	0.081203	0.583922	0.422116	0.278108	0.363394	0.170942	0.279254	0.281576	0.310424	0.042251	0.070445	0.113922	0.278179	0.183634	0.348155	0	0	0.098424	0	0	0
LOC107796452	40.09315	46.34467	42.44089	47.95937	38.5722	45.00116	58.72547	53.19328	54.50249	40.61501	42.82956	38.09337	47.47903	43.83586	46.24907	42.95433	42.14098	45.91052	34.53944	33.6674	30.74837	45.8548	49.1322	41.93607	27.09161	25.80748	32.83035	29.42902	30.28362	38.16673
LOC107761431	10.52514	12.37146	13.51015	19.33782	19.32299	19.3532	16.25321	14.07777	15.28394	15.68276	18.58853	13.17169	19.60088	16.41877	15.54783	14.58715	10.73834	14.00715	17.91675	18.88882	12.60443	16.63493	16.87581	16.14146	13.92879	13.68688	9.707744	3.517271	11.36563	18.24631
LOC107808373	0	0	0.273469	0	0.556745	0.507339	0	0.134398	0.147677	0	0	0	0	0	0	0	0	0	0	0	0	0	0	0	0	0	0	0	0	0
LOC107784020	2.735459	2.298265	2.340722	2.110331	2.250322	2.550357	3.083029	2.793744	2.407659	1.938046	2.052972	1.534643	1.617736	1.632012	1.75475	2.038467	1.734261	1.832274	1.973388	2.133228	1.656693	2.909108	2.846416	2.59106	0.783555	1.070491	0.985083	0	1.131752	2.642774
LOC107763210	5.418596	6.0358	5.233634	7.384439	5.860226	3.933561	6.992015	7.201904	4.913295	5.645102	5.423251	3.169233	5.000936	3.890423	6.797387	6.660882	5.234819	6.007898	5.404427	6.85605	3.716948	8.122174	5.964237	4.985829	4.098741	7.346258	3.448493	3.312019	6.466021	8.590768
LOC107784004	4.649804	6.196192	5.161168	6.931303	7.368312	5.407401	7.543607	6.442143	6.130748	5.816713	7.891317	4.491703	7.516871	6.972446	9.202616	6.881761	4.832141	5.545221	5.648801	6.44077	3.792976	8.191792	6.111301	5.208498	6.757067	8.686628	3.536074	5.961634	4.949851	17.18154
LOC107782587	0.327374	0.165756	0.158099	0.560214	0.643737	0.234644	5.766228	4.599788	2.049018	0.24829	1.93846	0.368766	2.389085	2.14289	3.136067	0.189746	0.31627	0.244071	0.922767	0.461559	0.829362	4.25286	1.796761	0.304152	0.965766	1.467775	0.300945	0	0.700486	4.498222
LOC107824601	3.775118	2.867129	4.102037	3.847574	2.644541	1.522018	9.499062	10.07989	8.26991	1.073688	2.867708	0.93022	3.502095	3.432057	2.260229	3.985398	2.279426	2.579968	1.755751	1.729807	1.0042	10.64036	11.51592	6.576254	0.397741	0.6566	0.743648	0	0.757282	0
LOC107767454	0	0	0	0	0	0	0	0	0	0	0	0	0	0	0	0	0	0	0	0	0	0	0	0	0	0	0	0	0	0
LOC107786579	6.099994	6.0292	7.368865	6.797591	6.563371	7.574305	6.413425	6.68017	8.361875	10.10926	6.225207	5.250223	9.309177	8.654356	5.290873	11.11888	7.165797	9.28777	7.933473	6.71065	5.484941	6.553185	6.66891	7.932224	7.277754	4.662256	5.788071	6.144167	7.445293	10.62456
107823289	0	0	0	1.694765	0.551775	0	0	0	0.585434	0	0	0	0	0	0	0	0	0	0	1.054992	0.568705	0	0	0.651754	0	0	0	0	0	0
LOC107808955	0	0	0	0	0	0	0	0	0	0	0	0	0	0	0	0	0	0	0	0	0	0	0	0	0	0	0	0	0	0
LOC107829675	3.168131	3.512955	3.993284	4.400921	4.158333	4.200891	3.277272	3.473902	3.61883	3.089328	4.035716	3.033395	3.468027	2.76502	2.87232	4.446352	4.591021	4.054725	3.37554	3.305359	3.996987	3.263139	3.819151	4.083973	2.00274	1.322467	2.371501	3.776102	1.779459	0
LOC107824472	0	0	0	0	0	0	0	0	0	0	0	0	0	0	0	0	0	0	0	0	0	0	0	0	0	0	0	0	0	0
LOC107800383	0.053123	0	0.046179	0	0	0	0.044557	0	0	0.051802	0.149001	0	0	0	0	0	0	0.079212	0.053906	0	0.193798	0.08873	0.046858	0	0	0	0	0	0	0
LOC107796213	16.03876	15.44999	13.05974	10.57586	12.30585	15.17003	5.874783	8.152986	9.435012	9.997607	7.758586	10.71993	12.88242	11.84958	6.563862	12.33034	13.72957	12.33598	11.0217	11.07742	9.813468	7.544942	11.5506	11.03434	10.90898	8.192232	8.758396	21.77845	17.59359	25.10635
LOC107809783	9.091955	5.984475	6.893559	7.550446	6.972472	10.75246	6.397211	5.61052	7.208117	5.368737	5.985684	6.827676	6.916215	5.345172	4.562181	5.796676	8.685997	8.171784	5.176748	5.469275	6.311145	5.483782	8.064259	10.92834	1.915832	1.546208	4.178984	0	3.404482	3.123163
LOC107760503	1.074067	0.692137	0.811061	0.363665	0.307202	0.594873	0.209292	0.241015	0.295385	0.285641	0.136934	0.137485	0.060387	0.177539	0.066812	0.662954	0.565985	0.81694	0.121099	0.385461	0.069262	0.163088	0.325369	0.181433	0.274333	0.347204	0.205165	0	0.31339	0
LOC107805472	6.910838	6.448338	7.15172	3.826086	4.610639	3.30222	5.428391	5.623626	8.033008	4.011273	3.461345	5.653119	6.655264	8.43693	4.953907	5.23171	6.782426	7.074221	6.456082	4.918142	6.502889	4.488883	6.67637	9.45896	7.003789	6.181667	5.963976	3.922402	11.61853	33.91329
LOC107777953	0	0	0	0	0	0	0	0	0	0	0	0	0	0	0	0	0	0	0	0	0	0	0	0	0	0	0	0	0	0
LOC107821830	0	0	0	0.058584	0	0.069524	0.054238	0	0.060712	0	0	0	0	0.141096	0	0	0.062473	0	0	0	0.117954	0	0.05704	0.067589	0	0	0	0	0	0
LOC107796416	0	0	0	0	0	0	0	0	0	0	0	0	0	0	0	0	0	0	0	0	0	0	0	0	0	0	0	0	0	0
Novel04187	0.146271	0.119977	0.07629	0.331286	0.608323	0.550408	0.711568	0.39993	0.494373	1.169591	0.984629	0.432509	1.856277	1.372344	1.186018	0.163502	0.042393	0.141767	0.385908	0.371206	0.186763	0.073293	0.141923	0.107018	0.203425	0.183173	0.224745	0	0.915463	0
LOC107774785	15.8991	19.33949	16.31847	55.59048	48.94807	48.6489	61.07084	55.37506	47.19818	33.0412	40.47472	37.07736	38.88115	44.00138	39.62728	26.6303	22.80334	22.50093	50.84043	49.63908	54.00225	48.73842	50.5204	42.33407	36.51601	65.53809	31.3694	36.53045	19.05913	32.90044
LOC107791594	0.296649	0.167365	0.085957	0.089583	0.087498	0.015187	0.509473	0.470721	0.755948	0.358144	0.316968	0.441564	1.65117	2.789379	1.406373	0.252644	0.068235	0.115848	0.057337	0.0239	0.012883	0.460088	0.299045	0.250999	0.053579	0.019656	0.133568	0	0.340042	0.873441
LOC107800069	1.862125	1.642352	2.297517	1.850248	2.498172	1.420782	1.259552	1.539726	1.35348	1.171488	1.010882	0.964204	1.872336	1.376168	1.048096	1.745763	1.973063	2.552668	2.925763	2.540692	1.917423	1.856098	2.172361	2.636912	1.442953	1.170134	0.993946	0	0.289191	0
LOC107772954	0	0	0	0	0	0	0	0.103121	0	0	0	0	0	0.131668	0	0	0	0	0	0	0	0.100792	0	0	0	0	0	0	0	0
Novel04240	0.224412	0.502013	0.363552	0.24027	0.37007	0.252237	0.290891	0.174312	0.450106	0.427713	0.536447	0.413649	0.987918	0.600927	0.47113	0.41808	0.305492	0.448691	0.144911	0.207095	0.055818	0.2726	0.269926	0.20257	0.03869	0.056773	0.108506	0	0.392872	0
LOC107787594	3.205055	2.941877	3.084596	0.829605	1.255962	0.615326	2.59221	2.758047	6.19004	0.870629	2.793181	2.93981	6.600638	4.545525	1.691784	3.292595	3.339638	3.294103	0.650454	0.329224	0.626371	1.472144	1.635662	2.321018	0.810433	0.318543	2.462276	0	0.330649	0
LOC107774255	0	0	0	0	0.05027	0	0.0953	0.097082	0.053337	0	0	0.047996	0	0	0	0.084671	0	0.042355	0.172944	0.192233	0.051813	0.094889	0.100222	0	0	0.158097	0	0	0	0
LOC107821909	3.286414	2.958184	2.574656	1.690826	0.897542	1.875701	0.442394	0.658667	0.457101	0.435202	0.967294	0.891207	0.812996	0.619684	0.208215	2.418805	2.195029	2.359415	0.329366	0.652118	0.444039	0.576021	0.572609	0.975359	0.102593	0.112909	0.815219	0	0.195333	0
LOC107806844	0	0	0	0	0	0	0	0	0	0	0	0	0	0	0	0	0	0	0	0	0	0	0	0	0	0	0	0	0	0
LOC107805601	0.41303	0.444867	0.195839	0.238117	0.132901	0.161476	0.125973	0.160411	0.17626	0.366143	0.210631	0.063444	0.083598	0.040963	0.231231	0.083943	0.072549	0.083981	0	0.09529	0	0.031357	0.03312	0.039245	0	0	0.044379	0	0	0
LOC107829567	5.385229	5.157205	4.388686	10.61508	11.8944	10.58444	6.898742	7.063692	6.41861	11.61025	10.91536	10.07663	10.79552	10.07474	9.521553	4.953644	4.816454	5.316628	16.13752	15.28595	13.19944	5.762223	6.902529	6.793944	29.6013	16.74251	30.87974	19.55687	43.64954	13.00691
LOC107831161	4.485478	5.701629	5.643479	4.758708	3.6557	5.488695	4.009675	4.210723	4.155754	2.820019	4.448831	1.894728	3.120811	2.768655	3.937436	5.453689	4.361858	5.25818	3.862831	4.792934	1.964676	5.323178	4.97164	4.009662	1.716226	1.806668	1.883419	0	2.131055	9.123154
LOC107785661	42.35493	32.73877	43.06354	23.9916	19.66324	36.91439	19.339	17.55926	19.37253	16.211	14.00026	16.02101	13.11275	20.59727	14.66213	20.54393	35.51106	33.25903	15.08922	14.55788	17.29516	14.8603	20.55897	20.08275	5.175396	4.300888	12.34224	17.92294	10.8592	10.33084
LOC107772357	0	0	0	0	0	0	0	0	0	0	0	0	0	0	0	0	0	0	0	0	0	0	0	0	0	0	0	0	0	0
LOC107831915	24.42603	24.90662	21.11658	3.640927	5.966503	7.585434	5.056246	2.747091	7.546255	1.393402	1.001977	0.377254	3.877383	4.092135	1.604126	11.68009	15.61669	15.1145	3.081238	3.588593	2.850798	1.342518	3.387389	1.680224	0.056457	0	0	0	0	0
LOC107787240	0	0	0	0	0	0	0	0	0	0	0	0	0	0	0	0	0	0	0	0	0	0	0	0	0	0	0	0	0	0
Novel00319	2.557606	3.170081	2.04541	1.575602	2.3084	1.319875	1.458718	1.485996	1.200596	0.798076	1.076037	0.345718	1.195808	1.618329	0.630013	2.020286	1.136597	1.296626	1.038113	0.908694	0.419864	0.982525	1.443825	0.962355	0.323359	0.498221	0	0	0	0
LOC107828213	0	0	0	0	0	0	0	0	0	0	0	0	0	0	0	0	0	0	0	0	0	0	0	0	0	0	0	0	0	0
LOC107831001	0	0	0	0	0	0	0	0	0	0	0	0	0	0	0	0	0	0	0	0	0	0	0	0	0	0	0	0	0	0
LOC107779767	6.294106	6.728001	6.046286	6.26269	5.324006	5.092404	5.171765	4.338744	4.42688	4.348297	5.430764	4.127772	4.774047	5.446695	4.620612	7.536399	5.668389	6.37865	4.698104	4.728772	3.677703	6.004737	5.40126	5.463581	2.697513	2.404681	2.319999	3.051644	5.033224	7.915404
Novel01178	9.314121	9.81965	9.50712	6.468247	6.260282	7.955229	6.287832	6.682702	5.880453	7.373552	7.828178	6.74471	7.370022	7.116414	5.496029	9.093297	7.08571	10.35562	7.837685	7.192772	6.541165	5.827074	7.385516	8.106944	6.031549	3.702834	8.246817	6.962627	8.593318	0

LOC107761438	0.371927	0.112989	0.323309	0.056158	0.164553	0.466512	0.155976	0.158892	0	0	0.043466	0.157107	0.138012	0.743883	0	0.092387	0	0.092429	0.251604	0.4195	0.734942	0.103535	0.164032	0.194369	0.156743	0	0.219794	0	0	0
LOC107805392	0	0	0	0	0	0	0	0	0	0	0	0	0	0	0	0	0	0	0	0	0	0	0	0	0	0	0	0	0	0
LOC107762488	4.925498	5.985312	4.742022	9.356335	9.982153	5.6941	14.48151	15.74786	8.502699	8.004971	12.55238	5.100823	7.605637	5.431256	13.91593	5.643867	4.400283	4.461882	9.996111	10.84221	13.47641	10.08449	15.36966	6.421323	7.767419	9.874894	8.701015	18.93778	4.589633	6.549483
LOC107776068	4.918469	4.341395	4.780504	4.876912	3.855282	4.954856	3.670571	3.35867	4.16318	4.248539	4.453612	3.664481	4.785506	4.478549	3.73645	5.03611	5.462532	4.547568	2.908097	3.439938	3.143541	4.608856	4.406737	4.735997	1.811672	1.643564	2.81507	4.154411	1.771283	0
LOC107771037	0.959389	1.278921	0.771739	1.906958	2.255367	1.339359	2.906459	2.495888	1.922425	2.206164	2.951953	1.185534	1.450574	1.405942	2.763012	2.187496	1.245023	1.441206	2.52825	2.931367	1.527948	2.917842	2.071404	1.167384	1.792283	2.829232	1.387783	3.072083	1.30982	2.65614
LOC107775573	0.784252	1.128551	0.574091	0.523521	0.401765	0.576903	0.519305	0.387945	0.54253	0.523246	0.839348	0.418457	1.010888	0.450305	0.25419	0.615185	0.6779	0.615466	0.418844	0.34917	0.150579	0.310239	0.473311	0.992268	0.156558	0.114866	0.097571	0	0	0
LOC107807666	5.799517	6.082571	6.481796	4.79538	4.724509	5.542391	6.138289	4.24737	5.531268	3.455993	3.195205	4.860707	4.201591	4.971205	3.543134	6.139709	6.581091	5.833679	2.615533	3.854708	3.652106	3.805472	4.993758	3.752452	2.211344	1.601112	2.937669	0	4.65349	2.845975
Novel00513	0.850321	1.588673	1.330498	1.425143	1.12863	0.822779	1.747342	1.453074	0.838235	1.077921	1.788736	0.682456	1.940507	1.345104	1.701855	1.425735	1.232222	1.331296	0.862847	0.82721	0.504079	1.7043	1.012553	1.155382	0.806298	0.650738	0.150752	0	1.023437	0
LOC107760597	29.6172	29.37931	26.19531	42.12222	41.54249	38.8293	42.49584	44.67181	32.72357	58.99065	45.43479	56.38017	38.0094	44.02185	49.71247	29.78108	22.42786	27.96268	52.9217	48.24831	47.3289	34.53593	33.11173	30.48276	44.55808	53.44725	40.39349	57.807	49.34532	23.99052
LOC107815680	0.094719	0.129487	0	0.343243	0.335255	0.305504	0.15889	0.242792	0.13339	0.092363	0.298877	0.200054	0.527214	0.206668	0.534695	0	0.045753	0.035309	0.192229	0.240378	0.215964	0.593269	0.29242	0.099001	0.538893	0.395385	0	0	0.228006	5.856636
LOC107789328	48.62365	56.92884	37.2133	93.41516	98.5259	49.51042	157.0891	169.0391	158.7365	159.101	205.2551	170.574	109.0017	75.55548	126.3229	62.95967	38.92176	48.60318	154.5984	221.0261	112.436	172.8263	236.1406	147.9468	51.65207	112.9752	116.5196	106.7101	57.96553	31.81453
Novel01653	1.634546	1.625113	1.356291	1.951984	0.98615	2.875642	0.249266	0.444373	0.627784	1.738787	1.771314	0.941529	1.075218	1.215824	0.610056	1.660998	1.22022	1.827935	0.678528	1.257012	0.948648	0.806622	1.048565	0.54359	0.281804	0.206759	0.439069	0	0.357695	0
LOC107790169	0	0	0	0	0	0	0	0	0	0	0	0	0	0	0	0	0	0	0	0	0	0	0	0	0	0	0	0	0	0
LOC107771831	13.48992	18.94001	12.12794	34.68173	52.86589	23.93699	49.19291	55.32552	38.04042	56.31493	54.50508	56.14136	98.81563	91.03224	105.3519	26.84343	26.06479	19.426	58.92279	57.20163	57.72449	49.20406	30.95883	21.46704	89.73267	136.0926	43.55152	78.22052	97.47639	132.254
LOC107813048	1.415275	2.260559	1.826378	2.194225	1.897858	1.89807	1.982499	1.533378	1.64378	1.835353	2.179169	1.491498	1.949077	2.053355	1.752104	2.739953	1.536422	2.588919	2.146782	1.814352	1.077847	1.888656	1.969072	2.028243	0.719424	1.015078	0.948461	2.086849	1.404874	1.804301
LOC107770255	1.118301	3.024702	1.379776	0.326814	1.372596	0.892036	1.301051	3.359684	1.761139	0.140708	0.075886	0.060953	0.24095	0.511618	0.148102	1.129067	1.603133	0.94132	0.073211	0.061033	0.361903	1.656959	3.372903	0.339343	0	0.050195	0	0	0	0
LOC107807472	0	0	0	0	0	0	0.086143	0	0	0	0	0	0	0	0	0	0	0	0	0	0	0	0	0	0	0	0	0	0	0
LOC107759505	0.024138	0.021999	0.020983	0.087472	0	0.129757	0	0.020624	0.067986	0	0.050777	0	0.026871	0.052667	0.049549	0.071951	0.069958	0.017996	0	0.020419	0	0.040317	0.021291	0	0	0	0.028529	0	0	0
LOC107786896	0.061951	0.02823	0.026926	0	0	0	0	0	0	0	0	0	0	0	0	0.046166	0	0.115468	0	0	0	0	0	0	0	0	0.036611	0	0	0
LOC107769187	49.8489	59.00967	51.85768	72.82468	65.98442	69.54705	57.85733	54.67872	48.41269	53.75045	54.07372	46.4282	42.62977	42.43461	57.47202	60.32954	46.96483	56.59467	77.62514	82.73178	52.0029	63.31955	46.70736	50.3543	51.76781	90.78361	42.80369	32.32005	40.17971	81.96932
LOC107813313	1.375829	1.029988	0.683416	1.290946	0.304356	0.79242	0.576983	1.637367	1.845269	0.383316	0.861368	0.954778	1.148694	0.428846	0.806922	0	0	0	0	0	0.268881	0	0	0	0.186371	0	0	0	0	0
LOC107816832	0.070498	0.1285	0	0.031934	0.187143	0.075794	0.029565	0.030118	0	0.103116	0.024717	0	0.196197	0.038455	0	0.131337	0.20432	0.157677	0.143072	0.208727	0.064295	0.058874	0.093275	0.221052	1.069566	0.098092	0.583258	0	0.848502	0
LOC107826440	0	0	0	0	0	0	0	0	0	0	0	0	0	0	0	0	0	0	0	0	0	0	0	0	0	0	0	0	0	0
LOC107805632	0	0	0	0	0	0	0	0	0	0	0	0	0	0	0	0	0	0	0	0	0	0	0	0	0	0	0	0	0	0
LOC107786996	0.202395	0.221349	0.211125	0	0	0	0.033951	0	0	0	0	0	0	0.088321	0	0.211155	0.273742	0.120715	0	0	0.06761	0	0.042309	0	0	0	0	0	0	0
LOC107766230	62.90925	53.51176	61.7444	55.49705	53.02111	71.71244	44.26996	46.08894	53.99354	39.89566	37.32314	40.89718	47.73066	49.40452	31.09355	45.84935	66.46526	59.14248	42.46676	38.53507	52.04378	37.96835	52.34175	61.92882	29.72744	21.97653	35.27845	25.52568	58.42551	82.76115
LOC107802801	3.53041	3.808509	3.538642	2.823054	2.900801	2.168927	4.532307	4.401596	3.111576	2.14284	3.928003	1.491294	2.065309	1.748897	3.623516	3.27516	2.018562	2.26949	1.352545	1.782748	1.018506	4.587975	3.495368	1.901471	1.161424	1.604019	0.532227	2.576268	0.520306	3.341182
LOC107766410	0.182508	0.041583	0.099156	0.082671	0.181682	0.073582	0.095673	0	0.042837	0.200214	0.095981	0.115641	0.050793	0.124442	0	0.102004	0.044079	0.119059	0.115748	0.057896	0.062419	0.019052	0.020123	0.047689	0.057686	0	0	0	0.109832	0
LOC107817954	0	0	0	0.108589	0	0	0	0	0	0	0	0	0	0	0	0	0	0.060814	0	0	0.05005	0	0	0	0	0	0	0	0	0
LOC107797011	18.39939	19.62819	23.34413	16.23533	12.94009	13.98814	17.98955	17.62926	22.20012	20.69003	24.36152	20.47117	24.31058	26.18363	24.03333	42.90378	21.2859	36.14396	14.29961	13.84021	14.42028	18.29686	17.52721	21.65807	9.748702	13.98458	10.20288	2.535292	15.70226	13.15216
LOC107818858	0.371488	0.22571	0.322926	0.224366	0.109572	0.266263	0.103861	0.105803	0	0.241498	0.520975	0.418457	0.827091	0.540366	0	0.184555	0.478518	0.09232	0	0.104751	0.564672	0	0	0	0.626231	0.172299	1.170849	8.85554	1.788474	0
LOC107783612	0.954401	0.409326	0.341617	1.525833	0.993549	1.38825	1.836434	1.966712	1.950191	0.821172	1.417185	0.711445	1.437435	2.572387	1.382924	0.794894	0.433897	0.502268	0.911492	0.759866	0.614421	2.34425	1.683689	1.114897	0.07098	0	0.331772	16.05957	0.270284	0
LOC107776353	0	0	0	0	0	0	0	0	0.11388	0	0	0	0	0	0	0	0	0	0	0	0	0	0	0	0	0	0	0	0	0
LOC107831004	2.479697	3.180653	3.645803	2.607025	3.033954	2.007711	3.312319	2.511076	1.609515	1.701568	2.769144	0.672442	1.499494	1.369312	2.513672	4.813594	3.253278	4.222383	1.739612	3.366603	0.837602	5.087667	2.403282	1.919836	2.206175	2.300211	0.832203	0	0.736922	1.892881
LOC107829551	84.17501	77.97075	85.64077	45.71582	43.12207	52.51	38.69925	39.28442	45.04246	38.01132	42.90384	38.91153	33.07935	30.9148	30.49885	76.37594	82.62501	87.78998	34.01797	34.73476	31.04267	43.29251	48.79966	64.68597	23.19033	13.15571	34.29082	13.52308	23.40973	21.71395
LOC107759376	3.941261	4.241716	5.004612	1.072393	0.868896	0.809867	1.116945	0.942446	2.083747	0.485322	0.565928	0.409108	0.688817	0.557643	0.234705	4.079768	3.209811	4.512866	0.559632	0.398264	0.368037	0.864989	1.423803	1.687128	0.05102	0.074867	0.222579	0	0	0
LOC107788223	0.355024	0.472897	0.284876	0.173188	0.434976	0.205528	0.48102	0.606685	0.179476	0.239673	0.727681	0.161503	0.456021	0.476694	0.364387	0.834396	0.3166	0.590453	0.554237	0.438938	0.174348	0.456139	0.650399	0.228352	0.172638	0.075999	0.096833	0	0.394434	0
LOC107766306	0	0	0	0	0	0	0	0	0	0.043956	0	0	0.050181	0	0	0	0	0	0	0	0.041111	0	0	0.047115	0	0	0	0	0	0
LOC107802192	0	0	0	0	0	0	0	0	0	0	0	0	0	0	0	0	0	0	0	0	0	0	0	0	0	0	0	0	0	0
LOC107772137	128.3064	118.0929	138.2091	60.17192	58.01226	70.70699	52.28219	56.95518	74.0869	27.54671	34.89951	34.50835	25.21921	31.38038	26.35212	85.35692	111.9837	103.4374	36.53721	32.69508	38.94325	59.42379	81.29324	107.7008	8.071857	7.880493	19.63551	12.27357	6.279583	14.85651
LOC107778089	0	0	0	0	0	0	0	0	0	0	0	0	0	0	0	0	0	0	0	0	0	0	0	0	0	0	0	0	0	0
LOC107759022	28.36409	34.58116	32.15873	19.97723	16.6216	17.98841	20.47622	17.18143	21.28284	15.56198	15.53967	14.0793	16.68454	15.72476	14.54474	29.16406	24.39533	29.67112	10.52724	11.67027	10.62925	16.71957	16.93894	18.45674	3.348872	4.514852	10.14853	1.578548	4.994613	8.188924
LOC107775387	0.213504	0.843193	0.37119	1.031596	0.503794	0.535601	2.566747	1.581008	1.403132	1.457357	2.145836	0.721498	0.633803	0.388205	0.876541	1.166761	1.168825	0.95506	2.816445	3.190778	2.790982	1.48586	2.385451	1.264544	1.259695	3.564911	3.196371	15.26859	0.342629	0
LOC107788209	0.247593	1.855352	0.502198	0.124615	0.048686	0.147885	0.161519	0.164539	0.025828	0.026826	0.077161	0	0.06125	0	0.028236	2.788104	0.823898	0.471734	0.362903	0.37235	0.12545	0.091899	0.436788	0.028754	0	0.038279	0	0	0	0
LOC107772206	11.68128	12.13785	10.80106	15.84027	19.09265	16.07847	12.23139	14.36727	15.8566	22.92655	18.4687	26.58884	28.98929	30.43878	16.18944	12.36429	12.0039	14.58878	18.27078	16.86789	17.57492	8.512625	14.17574	15.47545	22.85855	10.45617	31.13021	37.2461	42.62614	32.20318
LOC107766459	0.147341	0.268566	0.192121	0.400451	0	0	0.061791	0	0	0.071838	0	0	0.410055	0.080371	0.378067	0.439195	0.071172	0	0.149512	0.311601	0.268755	0	0.129965	0	0.093142	0	0.174145	0	0	0
LOC107817221	0.025982	0	0	0	0.022991	0	0	0	0	0.025336	0.018219	0.02195	0	0	0.026667	0.019362	0.025101	0.019371	0	0.021979	0	0	0	0	0	0	0	0	0	0
LOC107771718	0	0	0	0	0	0	0	0	0	0	0	0	0	0	0	0	0	0	0	0	0	0	0	0	0	0	0	0	0	0
LOC107792709	2.668088	4.103382	3.108523	4.783948	5.820274	6.075687	5.190547	5.255945	5.235976	4.2278	3.429919	4.367271	5.692793	5.194886	3.556185	3.42423	2.810297	2.707486	7.106903	7.601766	6.539605	5.183637	4.837607	4.396052	7.238485	6.213198	6.854408	1.325042	8.741823	10.31074
LOC107811040	0.947307	0.897888	0.95523	1.315925	1.173535	1.127108	1.112366	1.338216	0.972382	1.133126	1.629632	0.554882	1.110798	1.088584	1.387134	1.298919	0.805357	1.035844	1.422667	1.549289	0.541415	1.213052	1.080693	0.976923	0.798456	1.652029	0.447856	0	0.912135	3.123916
LOC107790020	0	0	0	0	0	0	0	0	0	0	0	0	0	0	0	0.826834	0.357305	0.379139	19.4685	14.27452	19.56386	14.94158	7.911093	4.783746	54.35858	94.4322	27.59389	56.20493	39.17279	102.9072
LOC107811236	0	0	0	0	0	0	0	0	0	0	0	0	0	0	0	0	0	0	0	0	0	0	0	0	0	0	0	0	0	0
LOC107795587	1.497255	2.024835	1.238554	0.568828	0.683804	0.493305	0.607651	0.309507	0.453448	0.682909	1.473213	0.428441	0.967799	1.027481	1.041018	1.673634	1.516465	1.692405	0.906685	0.715002	0.440491	0.806707	0.85205	1.135836	0.427448	0.537631	0.542307	0	1.278895	0
LOC107786224	0.702674	0.334122	0.50459	0.415165	0.33792	0.509115	0.204996	0.182725	0.458923	0.521345	0.428448	0.438776	0.357103	0.683259	0.219497	0.455333	0.501753	0.899695	0.511518	0.310129	0.208973	0.267897	0.538961	0.622673	0.173816	0.106274	0.523579	0	0.147084	0
LOC107826925	0	0	0	0	0	0	0	0	0	0	0	0	0	0	0	0	0	0	0	0	0	0	0	0	0	0	0	0	0	0
LOC107817466	0	0	0	0.147257	0.053936	0	0.136333	0.052081	0.171679	0.277376	0.199458	0.360469	0.113091	0.221659	0.083415	0.015141	0	0	0.061852	0.017188	0.037061	0.033936	0.053765	0.021236	0.077064	0.084813	0.408242	0	0	0
LOC107806631	0	0	0.028264	0	0	0	0	0	0	0	0.045597	0	0	0	0	0	0	0	0.032993	0	0	0	0.041107	0	0	0	0	0	0	0
LOC107772903	1.087759	1.067616	0.581886	3.259579	1.480801	2.518866	1.193073	1.286876	1.571132	4.161216	1.936179	2.686216	1.583495	2.008247	1.374087	0.872953	1.778389	0.561441	1.103782	0.778604	1.22099	1.397563	1.40231	2.623673	1.269469	1.047833	1.58233	0	0.80567	0
LOC107787612	0.468124	1.29515	0.595863	0.151463	0.118351	0.161772	0.140227	0.042855	0.109874	0.065211	0.140678	0.042373	0.24195	0.054718	0.051479	2.51668	0.484551	0.847587	0.050895	0.254572	0.030496	0.111698	0.058988	0.087372	0	0.139577	0	0	0	0
LOC107801985	0	0	0	0	0.086311	0	0	0	0	0	0	0	0	0	0	0	0	0	0	0	0.088959	0	0	0	0	0	0	0	0	6.031135
LOC107764120	0	0	0	0	0	0	0	0	0	0	0	0	0	0	0	0	0	0	0	0	0	0	0	0	0	0	0	0	0	0
LOC107797067	14.72739	19.63094	15.13658	19.38599	16.08335	17.20134	17.28401	16.76188	15.12713	16.29058	15.9759	13.03914	15.63951	14.7408	16.71151	17.35757	15.86643	17.02832	17.43984	18.79515	14.48863	15.32092	16.88021	16.51578	12.29414	13.01897	11.89395	8.086117	12.79246	8.739125
LOC107778556	0	0	0	0	0	0	0	0	0	0	0	0	0	0	0	0	0	0	0	0	0	0	0	0	0	0	0	0	0	0
LOC107772188	0.297465	0.165019	0.281066	0.210904	0.171663	0.208573	0.390517	0.287314	0.327843	0.189174	0.272065	0.131117	0.215963	0.169315	0.159292	0.125293	0.16243	0.192846	0.196857	0.317279	0.15334	0.35643	0.239568	0.364982	0.016352	0.017996	0.030572	0	0	0
LOC107761420	13.86647	17.56932	15.25104	17.0817	15.18783	17.77161	18.65121	18.92773	15.39988	15.99502	19.476	13.82202	15.10692	14.25135	19.52584	15.56298	13.84544	15.03429	16.25857	17.63089	15.76952	18.14711	19.80103	16.2607	15.28656	15.70594	15.63956	9.06996	19.13191	20.91183
LOC107770336	129.6718	115.5601	120.4948	131.7025	121.2895	155.6093	100.8141	99.24262	119.4927	90.94177	75.09978	98.30753	103.2867	110.8031	78.8934	95.20356	117.7143	106.7165	91.50245	86.77311	105.155	80.28571	99.21311	102.9294	44.01469	48.66248	63.54446	137.0449	58.17448	62.53625
LOC107761100	4.838777	3.858705	4.416559	3.287765	3.10421	3.771651	2.638019	3.204146	4.315714	2.713091	4.750135	5.927503	3.231956	1.979575	0.869118	3.425569	3.973466	4.59958	2.086774	2.455963	2.537502	2.828692	2.560873	3.793116	2.447074	1.346561	5.290117	0	4.076729	0
107828194	0	0	0	0	0	0	0	0	0	0	0	0	0	0	0	0	0	0	0	0	0	0	0	0	0	0	0	0	0	0
LOC107808980	37.62229	37.64444	37.88627	32.85975	34.205	36.54613	35.88875	36.76359	40.47043	34.80049	33.86771	36.19387	38.19489	37.31541	31.19071	32.71545	36.75583	35.71311	27.47632	25.68482	31.05009	31.10959	38.37348	36.91359	27.26671	20.86574	31.09525	35.99999	33.54678	26.21118
LOC107781183	0	0	0	0	0	0.022976	0	0.01826	0.040128	0	0	0.018055	0	0.046629	0	0	0	0	0.015933	0.021686	0	0	0	0.022337	0	0	0	0	0	0
LOC107829178	0.090717	0.347243	0.063086	0.525982	0.513741	0.487657	0.745662	0.837116	0.579147	0.353841	0.547051	0.275904	0.363553	0.633391	0.744872	0.243368	0.157752	0.229952	0.331391	0.214872	0.198564	0.348495	0.304069	0.246524	0.252325	0.403922	0.10722	0	0.262045	0
Novel04138	0	0.013162	0.012554	0.026167	0.025558	0.031053	0.060564	0.012339	0.027117	0.042247	0.040506	0.073204	0.09646	0.047265	0.029645	0	0.013952	0.032301	0	0	0	0.024121	0	0.030189	0	0	0.017069	0	0	0
LOC107829731	0.197287	0	0.171498	0	0.174573	0.106054	0	0.084284	0.185222	0	0.484183	0.083337	0.219623	0.538077	0.40498	0.294037	0	0.220629	0.400387	0.417229	0	0.16476	0.08701	0.206205	0.498862	0.137255	0	0	0.474905	6.099284
LOC107796793	8.154105	5.856999	8.409718	3.944017	5.808919	4.160449	5.564094	4.90058	6.422779	5.862386	2.761922	9.107247	7.923374	7.91569	3.4044	6.848879	5.474231	5.048853	4.207232	6.079428	6.554363	4.674459	4.083854	5.344724	4.805167	3.9422	6.288875	4.941812	11.97664	0
107799653	0	0	0	0	0	0	0.610183	0	0	0	0	0	0	0	0	0	0	0	0	0.6417	0	0	0	0	0	0	0	0	0	0
LOC107823449	80.7205	35.20751	48.46547	63.04197	41.64698	67.92218	84.32903	82.23044	134.3839	83.15415	153.3025	175.9386	470.7983	401.6626	98.51883	28.47368	57.44814	44.14499	9.671665	6.992509	18.77001	62.62183	68.52242	74.75961	2.66602	0.821543	20.53653	12.06407	7.715494	5.215329
LOC107825409	9.712484	12.0225	9.955009	12.30111	15.64922	14.90987	13.73925	13.41815	13.88191	24.17199	23.61616	23.22815	28.7784	21.2444	14.58098	10.08244	8.869574	14.01381	13.8272	12.99859	11.98688	10.73416	11.20963	15.80803	21.80991	12.64016	22.15919	6.911243	18.14542	14.93874
LOC107815809	66.3151	62.05343	70.66322	48.72696	39.69686	73.3339	46.13751	44.5458	52.73412	43.68653	41.5716	51.37762	36.40115	49.07561	34.94161	37.98589	74.10395	46.70667	33.42892	36.39908	43.75777	29.70693	45.87894	43.37622	20.40037	16.49852	39.29792	48.08031	24.12875	22.67482
LOC107825611	0	0	0	0	0	0	0	0	0	0	0	0	0	0	0	0	0	0	0	0	0	0	0	0	0	0	0	0	0	0
LOC107801543	0	0	0	0	0	0	0	0	0	0	0	0	0	0	0	0	0	0	0	0	0	0	0	0	0	0	0	0	0	0
LOC107831723	12.28748	16.52582	20.72161	4.07107	2.547334	3.264891	5.373862	4.919416	7.102896	2.002679	3.237149	1.705418	2.833423	2.661857	3.080817	22.72155	12.44732	18.45254	2.208703	2.390703	1.28073	6.757992	7.23074	7.265394	0.643597	0.073273	1.016597	3.765979	0.84509	0
LOC107819004	12.86855	10.3381	12.55358	5.267804	3.205212	6.353973	5.436679	6.067742	9.486263	1.440749	2.70703	2.617263	2.705918	2.599806	1.418629	9.412072	10.08381	9.167647	2.85344	2.056227	2.69501	7.443197	11.09867	11.15869	0.120517	0.596855	0.844977	0	0.688375	0
LOC107823501	0.991638	0.774648	1.354588	1.860918	1.410215	1.865732	2.168439	3.08653	2.194486	1.899411	1.614176	2.15425	1.182756	2.279568	1.199539	1.662683	1.129079	1.584233	1.724994	2.396737	2.680873	1.715439	2.217966	1.813805	3.805974	2.020411	3.976554	2.53272	2.728058	0
LOC107822628	0	0.21407	0.136121	0.070932	0	0	0	0	0	0	0	0	0	0	0	0	0.15128	0.058373	0	0	0	0	0	0	0	0	0	0	0	0
Novel02834	0.754118	0.887744	0.273141	2.932034	2.168702	0.912115	3.452456	3.033761	3.480995	4.25896	10.72999	2.999677	3.183083	3.496498	3.354025	1.732739	0.576762	1.007323	0.57392	0.53161	0.659111	3.857423	2.577579	2.003351	0.238359	0.306046	0.222827	0	0	0
LOC107772326	0	0	0	0	0	0	0	0	0	0	0	0	0	0	0	0	0	0	0	0	0	0	0	0	0	0	0	0	0	0
LOC107821144	0	0	0	0	0	0	0	0	0	0	0	0	0	0	0	0	0	0	0	0	0	0	0	0	0	0	0	0	0	0
LOC107803656	0	0	0	0	0	0	0	0	0	0	0	0	0	0	0	0	0	0	0	0	0	0	0	0	0	0	0	0	0	0
LOC107827856	8.895973	10.111	10.89797	7.685897	7.233493	7.902515	9.535657	8.59877	8.738893	12.89482	10.25996	7.051283	6.38541	8.056331	10.43495	13.33537	9.888374	12.70129	7.388911	7.641603	6.640983	7.515312	7.695353	8.005672	10.98666	9.319416	11.16386	9.825295	9.09483	12.7425
LOC107829762	3.860396	4.217111	3.148899	4.048272	3.322345	5.628622	3.548362	3.004732	3.525014	4.047991	5.22842	3.730471	4.238578	3.865352	3.36516	3.625521	3.474025	3.922875	2.065954	2.684079	1.808603	2.362734	3.731644	3.979613	1.337182	0.662235	1.812571	0	0.76378	0
LOC107764998	0.405596	0.36965	0.654783	0.052493	0.153813	0.24918	0.194395	0	0.326392	0.226004	0.243775	0.293707	0.32251	0.126424	0.297352	0.431786	0	0.302389	0.235183	0.147045	0.158533	0.53228	0.357762	0.423928	0.659309	0.161245	0.342415	0	0.278954	0
LOC107766876	0.448009	0.544407	0.162269	0.811748	0.726785	0.96333	0.438392	0.414691	0.315458	0.982948	0.654468	1.135472	0.664974	0.936783	0.574781	0	0	0	0	0	0	0	0	0	0	0	0	0	0	0
LOC107786734	0	0	0	0	0	0	0	0	0	0	0	0	0	0	0	0	0	0	0	0	0	0	0	0	0	0	0	0	0	0
LOC107774040	137.6159	270.7306	170.1954	11.07246	8.708005	9.727076	23.23132	16.95253	15.42352	12.69183	6.789248	3.888801	26.32779	62.25205	31.52358	119.9392	241.6689	116.2073	4.993007	5.840821	10.71234	15.57541	41.86219	5.640632	0.100339	0.331285	1.031811	17.02679	0.764166	0
LOC107779410	0	0	0	0	0	0	0	0	0	0	0	0	0	0	0	0	0	0	0	0	0	0	0	0	0	0	0	0	0	0
LOC107791437	0.336487	0.715554	1.267503	1.016133	1.091734	0.844117	0.470376	0.383338	0.526514	0.874979	0.393241	0.379031	0.874024	1.468362	0.460481	0.501501	0.650149	0.668974	0.455258	1.043697	0.818351	0.187339	0.692542	0.468928	0.141807	0	0.927967	8.021185	0	0
LOC107810669	4.383876	2.686537	3.646551	4.485135	3.176884	4.753834	4.469391	4.326933	4.257699	1.732044	2.358482	3.448198	3.702215	4.040467	2.055802	3.717472	3.87009	4.564442	2.262576	2.269832	1.378679	3.377039	3.800199	5.372031	0.812271	0.157755	1.206013	0	1.091666	0
Novel00285	2.643263	2.507335	2.016377	1.51499	1.097866	1.565907	0.76917	0.599187	1.215483	1.683275	1.39955	2.369823	1.861592	1.530111	0.941235	1.406973	1.980354	3.700024	0.656865	0.547596	1.771127	0.765853	0.428241	1.240413	2.046054	0.450356	2.932851	3.857769	5.194126	3.335448
LOC107784551	0	0	0	0	0	0	0	0	0	0	0	0	0	0	0	0	0	0	0	0	0	0	0	0	0	0	0	0	0	0
LOC107801260	0	0	0	0	0	0	0	0	0	0	0	0	0	0	0	0	0	0	0	0	0	0	0	0	0	0	0	0	0	0
LOC107778184	2.996852	3.918766	2.208672	3.246191	2.190627	4.132543	0.546432	1.057636	0.672812	0.762342	0.593872	0.110079	0.435148	0.568594	0.534937	1.699218	2.706393	2.088566	0.661087	0.881785	1.247756	0.924929	1.551574	0.680937	0.082368	0	0.077001	0	0	0
LOC107821814	0	0	0	0	0	0	0	0	0	0	0	0	0	0	0	0	0	0	0	0	0	0	0	0	0	0	0	0	0	0
LOC107772680	0.120691	0.219989	0.419655	0.10934	0	0.129757	0.101228	0	0	0	0.084628	0.101963	0	0.131668	0	0.089939	0	0	0	0	0.110072	0	0	0	0	0	0.142647	0	0	0
LOC107765858	0	0	0	0	0	0	0	0	0	0.176737	0	0	0	0	0	0	0	0.045042	0	0	0	0	0	0	0	0	0	0	0	0
LOC107817357	0.204608	0.596721	0.498013	0.926824	1.194937	0.659937	1.990721	2.272699	1.344668	1.995191	2.238156	1.486587	1.594411	1.473237	2.184047	1.768704	0.553473	0.762721	1.993176	2.561653	1.7541	0.888544	1.227252	0.769884	1.552124	2.049824	1.983012	5.852959	3.152185	0
LOC107823191	0	0	0	0	0	0	0	0	0	0	0	0	0	0	0	0	0	0	0	0	0	0	0	0	0	0	0	0	0	0
LOC107814413	0.132944	0	0	0	0	0	0	0	0	0	0	0	0	0.29007	0	0	0	0	0	0	0	0	0	0	0	0	0	0	0	0
LOC107784509	0.844835	0.909956	1.00145	1.983024	1.834933	1.527597	1.642662	1.673379	1.838708	1.797427	1.992614	2.270991	3.761928	3.267751	1.891888	1.659781	1.038776	1.775061	1.441789	1.331889	1.646076	1.218666	1.253291	2.207553	1.650743	1.549557	0.680814	0	1.294152	0
LOC107776795	0.795354	0.207105	0.197538	0.514679	0.201081	0.610788	0.0953	0.097082	0.213347	0.775571	0.079672	0.479955	0.505943	0.867692	0	0.084671	0.109768	0	0.230592	0.288349	0	0.189777	0.100222	0	0.143653	0	0.402876	0	0	0
LOC107810373	0.040107	0	0.139457	0.21801	0.141957	0.12936	0.100918	0.068537	0.037654	0.078219	0.196861	0.033884	0	0.08751	0.041165	0	0	0	0.162791	0	0	0.033494	0	0	0	0.055806	0	0	0	0
LOC107768075	0	0	0	0	0	0	0	0	0	0	0	0	0	0	0	0	0	0	0	0	0	0	0	0	0	0	0	0	0	0
LOC107782666	10.67472	16.10267	9.27931	25.21067	25.47081	8.468963	106.0818	105.4865	84.66778	26.31016	58.46139	26.96162	32.90431	29.35494	67.43757	14.78499	11.45063	11.44372	17.62997	20.4578	18.4975	106.7924	102.6973	58.94089	14.65955	29.91107	20.14316	39.48588	12.75937	29.58774
LOC107803123	0.216669	0.345568	0.094173	1.128675	0.814823	0.99002	1.726432	1.38846	0.711965	2.429717	1.975071	2.013529	4.040085	2.541035	4.225282	0.242193	0.05233	0.282688	0.769513	0.595683	0.395212	0.678548	0.812245	0.283078	0.616355	1.959615	1.088363	3.873727	1.303903	10.04773
LOC107806797	8.37757	8.507708	7.629227	10.73394	11.15484	11.02275	11.77797	11.45284	10.7117	10.54214	10.85357	12.09933	14.12227	11.66377	12.24264	9.096907	10.13607	9.606688	9.553482	9.88784	14.15309	9.861491	11.68256	10.59087	11.24748	11.54576	11.69333	19.97049	14.02038	12.33328
LOC107786306	0	0	0.142512	0	0.145067	0	0	0	0	0	0.057478	0	0	0	0	0.061085	0	0.061113	0	0	0	0	0	0	0	0	0	0	0	0
LOC107831346	12.11282	10.44262	11.21241	6.821428	6.77855	7.250511	7.139139	7.32858	7.683817	6.193072	6.702992	7.854708	7.215848	6.64296	5.510518	8.636153	9.48811	8.932993	6.511042	7.034161	7.225398	7.272391	7.565645	10.67573	5.380695	3.461924	4.875307	60.87077	5.989152	8.096799
LOC107829685	19.4117	14.2111	11.89491	13.20393	10.30605	17.24337	13.18529	12.28987	12.90657	9.061026	11.06771	14.67893	17.0041	21.59381	15.28567	13.89648	18.38441	14.80439	7.943642	6.945259	9.867689	9.72673	12.7997	11.17564	2.413995	2.56816	4.814282	0	4.596139	0
LOC107789688	0	0	0	0.067597	0.46217	0.240661	0.250331	0.191259	2.661972	0.072759	0.15696	0.063037	2.159625	0.488408	0.765828	0	0.072084	0.055629	0.605714	0.252477	0.884652	0.997008	1.118862	0.623901	0.47168	1.245857	0.176378	0	8.980585	23.0678
LOC107784962	3.915497	7.464704	5.140219	0.217178	0.070708	0.300688	0.268089	0.034138	0.037511	0.03896	0.028016	0.033754	0.088955	0.130764	0.082015	4.168333	4.245887	3.336193	0.081085	0.304185	0.218633	0	0.070484	0	0	0	0	0	0	0
LOC107824790	0	0	0	0	0	0	0	0	0	0	0	0	0	0	0	0	0	0	0	0	0	0	0	0	0	0	0	0	0	0
LOC107782883	31.26385	35.42959	34.92913	4.225304	2.539674	5.014318	1.09618	1.445112	1.924713	1.499321	1.850811	1.645373	2.767152	5.311796	0.999473	26.31512	28.91615	33.81647	4.394609	3.403434	3.061659	1.540874	4.452972	3.803385	1.231169	0.499196	3.059088	0	1.973971	0
LOC107822872	3.40399	2.975692	3.019404	2.989439	2.766191	4.331896	3.26294	3.353634	4.46762	4.132215	3.750811	3.785472	4.485375	4.395673	2.994653	2.7955	3.724775	3.806728	2.009037	1.880517	2.217497	3.567955	3.860414	2.904367	2.239669	1.111605	3.366386	0	3.846168	0
LOC107812175	2.594446	2.215404	3.210242	8.00419	10.63071	7.337741	8.292605	9.845632	6.824573	7.612529	8.342222	9.280849	6.765091	7.573267	7.268911	2.142399	2.370969	2.5616	15.13189	14.5524	13.70678	7.749331	7.360224	6.767071	20.7744	24.74952	17.56979	21.72986	22.16798	14.45212
LOC107777171	1.339255	0.813711	0.991714	48.44202	37.83418	11.19894	20.84329	17.46111	5.821073	8.077499	12.03422	2.17907	2.595231	1.893969	3.156431	0.221781	0	0.184902	7.19758	11.4131	8.052346	5.426539	8.137925	4.3549	67.79174	18.15164	70.52663	21.28349	5.731246	9.200908
LOC107789975	0	0	0	0	0.04677	0	0	0	0	0.051541	0	0	0	0	0	0	0	0	0	0	0	0	0	0	0	0	0	0	0	0
LOC107769965	15.75467	13.3682	14.99389	19.60478	18.94823	22.77896	13.7837	10.25064	12.07096	17.83497	18.63419	17.28768	25.04755	23.26249	18.91166	13.79834	18.36937	17.41615	17.87025	19.68468	21.09861	12.81687	12.01982	17.36583	9.043546	8.693	10.22001	35.61351	22.6674	8.397717
LOC107763802	0	0	0	0	0	0	0	0	0	0	0	0	0	0	0	0	0	0	0	0	0	0	0	0	0	0	0	0	0	0
LOC107817307	0.844118	1.29568	0.656535	9.176741	8.687992	5.970595	14.38355	12.64064	11.55375	9.011019	9.283426	9.49595	8.160416	6.688581	5.973461	0.72836	1.502217	1.059917	9.557327	9.884177	12.92538	10.53708	11.09015	9.24064	4.381228	6.181732	7.718892	0	3.849997	5.494011
LOC107814160	69.73606	62.37536	68.77181	63.64671	56.75582	80.71209	55.05768	58.83183	70.72456	55.97743	50.0615	62.98561	58.48291	63.30464	46.31648	46.21469	67.87143	53.11136	44.42424	42.16212	51.26783	46.12343	56.48485	68.77259	29.80405	20.32928	46.7836	40.75651	40.65727	35.23829
LOC107787690	0	0	0	0	0	0	0	0	0	0	0	0	0	0	0	0	0	0	0	0	0	0	0	0	0	0	0	0	0	0
LOC107800794	0.34827	0.190443	0.181646	0.252412	0.123269	0.074887	0.292109	0.059514	0.392365	0.339607	0.146524	0.117691	0.07754	0.227967	0.142982	0	0.134583	0.20772	0.14136	0.058922	0.381153	0	0.368636	0.436814	0	0.096918	0.082325	0	0	0
LOC107778892	6.782991	2.633014	4.913592	1.820754	0.555744	0.94533	0.421421	0.643952	1.29722	0.489946	0.792706	2.122392	0.978823	1.370354	0.128924	5.335509	7.645096	10.48861	0.382384	0.318775	1.374711	0.314703	2.659132	2.888347	0	0	0	0	0	0
LOC107828408	0	0	0	0	0	0	0	0	0	0	0	0	0	0	0	0	0	0	0	0	0	0	0	0	0	0	0	0	0	0
LOC107763133	3.405788	2.040564	2.001132	0.314261	0.306947	0.135616	0.105799	0.107778	0.148033	0.061501	0.154787	0.026642	0.070211	0	0.032367	1.433499	1.492811	1.833838	0.255997	0.186736	0.431408	0.237022	0.389424	0.164802	0	0	0	0	0.151821	0
LOC107796296	0	0	0	0	0	0	0	0	0	0	0	0	0	0	0	0	0	0	0	0	0	0	0	0	0	0	0	0	0	0
LOC107825123	0	0.07041	0	0	0	0.08306	0.194395	0	0.072532	0.150669	0	0.065268	0	0.252848	0	0	0.149272	0.057598	0.078394	0.130707	0.070459	0	0	0.161496	0.097675	0	0.091311	0	0	0
LOC107798256	0	0	0	0	0	0	0	0	0	0	0	0	0	0	0	0	0	0	0	0	0	0	0	0	0	0	0	0	0	0
LOC107778160	2.588885	2.963029	2.72149	2.89084	3.169849	2.767185	3.673721	3.999629	2.840363	3.082226	3.472342	2.110865	3.016028	2.479513	4.402853	4.094027	2.937315	3.287943	3.421237	3.552414	1.866921	4.575491	3.252756	2.53285	2.245524	4.02111	1.583296	3.229219	2.681169	5.583999
LOC107767298	1.13343	1.20708	0.913309	0.686477	0.535274	0.595596	0.710322	0.467895	0.567926	0.533991	0.937641	0.231319	0.744291	0.611312	0.450951	0.085412	0.123032	0.137672	0.012923	0.037706	0.029037	0.111672	0.0337	0.019966	0	0.00886	0	0	0	0
LOC107762102	310.1885	257.6874	330.8856	149.8906	132.3925	188.6612	134.1234	135.0532	168.3395	58.79631	84.92939	87.61203	57.86925	74.84823	63.24045	220.9274	352.8207	272.3757	83.27493	86.83357	113.3514	160.3201	188.9993	236.0892	27.02337	18.72551	50.6809	56.613	16.51523	48.9479
LOC107830341	0	0	0.017326	0	0	0	0	0	0	0	0	0	0	0	0	0	0	0	0	0	0	0	0	0	0	0	0	0	0	0

Novel02586	0.357948	0.761193	0.259297	0.75666	0.84463	1.731772	0.850642	0.356814	0.672115	1.512527	1.045806	1.108817	1.992365	1.496932	1.040933	0.844688	0.230539	0.489254	0.484295	0.958865	0.326455	0.896796	0.526223	0.498837	0.905111	0.664079	1.057665	4.266394	2.010505	0
LOC107778756	0	0	0	0	0	0	0	0	0	0	0	0	0	0	0	0	0	0	0	0	0	0	0	0	0	0	0	0	0	0
LOC107816860	1.602311	1.028386	0.823942	0.04089	0.039939	0.024263	0.075714	0.15426	0.233064	0.022006	0	0.038132	0.025123	0.049241	0	0.521343	0.65407	0.790785	0.0229	0.019091	0	0.131928	0.179156	0.259466	0	0	0	0	0.108649	0
LOC107801268	5.731792	5.625654	6.937208	4.913106	4.447637	5.024708	3.254308	4.143953	4.884527	2.751617	2.720646	3.687656	3.141273	3.896164	2.172168	3.482783	5.792965	4.306166	3.847658	2.946519	4.302627	2.982525	4.355783	5.299614	1.282113	0.981584	0.93801	0	1.27361	0
LOC107803112	2.628652	2.959385	3.393944	2.416447	2.839094	4.031381	2.723532	2.642343	3.484086	2.337097	1.843213	1.926835	3.270517	2.741213	2.499596	2.246945	3.846594	3.083759	2.471244	2.25636	2.961463	2.84092	2.966502	3.313121	2.541435	1.990153	2.010318	2.764497	5.397099	4.780398
LOC107789313	8.53767	8.949357	7.421616	5.690693	3.92481	6.009101	5.010488	5.060369	5.752896	7.900291	5.98662	4.093786	5.051811	3.412402	3.157777	5.636256	7.827033	6.957751	1.899192	2.602624	2.034311	5.930987	6.015586	5.681072	8.492742	3.139339	22.18167	9.16763	12.59023	3.170553
107808738	0	0	0	0	0	0	0	0	0	0	0	0	0	0	0	0	0	0	0	0	0	0	0	0	0	0	0	0	0	0
LOC107772667	1.267252	1.79658	2.44799	1.530755	1.121348	1.816602	1.535299	1.082775	1.057558	1.235731	1.086064	0.35687	1.410723	1.075286	0.867115	0.524643	1.224272	1.994557	1.4288	1.310232	0.513669	1.528678	0.7452	2.060383	0.356043	1.175526	0.998527	0	0.677889	0
LOC107832835	13.18443	10.71108	12.96487	9.620395	8.023983	12.37896	9.056836	8.767434	10.47379	6.806373	6.525832	8.719334	10.8916	11.45478	7.347766	8.935906	8.818107	11.56418	5.387781	5.34946	5.821778	7.77223	8.156463	12.03443	2.790758	1.743206	5.852414	8.532787	4.021011	0
LOC107771077	16.60632	23.19698	16.89766	16.76687	14.41971	16.47725	17.57326	15.15029	13.44156	13.16649	11.67157	12.45608	10.75493	11.47108	11.70798	19.19868	15.93065	18.42644	15.23672	17.46129	16.80839	14.67844	14.57943	16.22143	8.143092	10.36553	10.31811	11.09894	8.966216	4.798103
LOC107796012	0.554156	0.439169	0.565492	0.371071	0.490353	0.388556	0.404169	0.349968	0.520266	0.305428	0.439258	0.386746	0.563251	0.578272	0.321479	0.359093	0.279319	0.592776	0.440076	0.428014	0.131843	0.46279	1.168874	0.654752	0.121847	0.704018	0.313245	0	0.81197	1.489751
LOC107818569	0	0.02498	0	0	0.024254	0	0	0.02342	0.025733	0	0.01922	0	0	0	0	0.020426	0.02648	0	0	0	0	0	0	0	0	0	0	0	0	0
LOC107772587	0	0	0	0	0	0	0	0	0	0	0	0	0	0	0	0	0	0	0	0	0	0	0	0	0	0	0	0	0	0
LOC107778750	30.68734	31.36122	45.91448	30.05635	31.76317	26.44809	39.38896	36.83683	44.95401	60.78639	47.6564	41.86609	44.05842	43.15448	45.28046	59.59462	36.03259	48.26832	40.09701	40.6113	36.59154	33.5415	35.74045	42.24114	60.96094	40.98192	71.79823	37.3991	49.95156	29.74862
LOC107824722	0	0	0	0	0	0	0	0	0	0	0	0	0	0	0	0	0	0	0	0	0	0	0	0	0	0	0	0	0	0
LOC107765637	16.17244	14.62563	15.98624	21.08539	17.90458	21.48637	15.67549	13.77559	13.87327	12.39258	16.93156	12.27348	12.23342	10.33046	12.58349	16.657	16.05726	16.81325	14.00849	14.5872	12.22689	14.73385	17.10061	20.41955	4.59976	6.553194	7.598685	7.128247	5.158667	0
Novel04767	1.89722	2.781566	1.649213	3.063921	1.970747	2.837904	1.868024	1.691516	2.323296	3.86093	3.41259	2.09064	3.397584	2.429734	2.285907	2.458801	2.231325	2.398428	2.594791	4.186741	1.805527	2.13552	1.891752	2.155406	4.484456	2.410291	4.19224	0	2.77988	0
LOC107771238	3.910706	4.212149	8.035167	0.107361	0.104862	0	0.248491	0.151882	0.445036	0.115559	0.124645	0.050059	0	0	0.060816	4.724638	6.239542	5.963722	0.120252	0.050124	0.216159	0.346387	0.470387	0.371588	0	0	0	0	0	0
LOC107778401	11.15917	9.184317	8.562111	3.146367	2.922006	4.590868	1.623617	2.091795	2.512274	1.221404	2.794577	1.683495	2.02817	1.925496	1.986825	6.02473	8.140523	7.343349	1.271011	2.50446	1.713533	2.329828	3.063421	2.32081	0.359913	1.029863	0.605628	0	0	0
LOC107801920	0.046935	0	0	0	0.083063	0.050461	0	0	0	0.045768	0.098733	0.039652	0.104498	0.051204	0.240865	0.139905	0.090687	0.244946	0.095253	0.19852	0.385252	0.078394	0.0414	0.14717	0	0.065307	0.110947	0	0	0
LOC107772159	0	0	0	0	0	0	0	0	0	0	0	0	0	0	0	0	0	0	0	0	0	0	0	0	0	0	0	0	0	0
LOC107782314	1.614535	0.723664	0.966331	0.62344	0.374727	1.052876	0.421794	0.293992	0.621228	0.567808	0.927962	1.140397	2.062502	1.299378	1.032301	1.420116	1.329646	1.183972	0.376008	0.492578	0.313808	0.331559	0.466927	0.553283	0.50195	0.036828	0.625656	0	0.637127	0
LOC107761459	18.34255	21.21432	19.46017	25.66758	23.66443	22.46375	25.49981	25.21565	25.0854	18.37923	19.17455	20.21432	21.893	21.19256	17.41802	18.31487	18.30182	17.8746	20.61569	19.38548	20.06567	22.77648	22.29436	23.92679	11.80831	15.90958	9.957735	13.77167	17.73104	20.83735
LOC107817023	5.77541	6.750047	5.369083	18.86994	23.63589	16.84553	13.00727	16.35751	12.77734	24.71714	14.75523	21.21108	18.48414	26.42789	16.8501	6.276437	5.966999	6.578326	28.81406	28.00172	28.09211	14.89385	14.66968	15.28677	70.0439	67.59965	48.03531	28.68219	91.39567	69.43656
LOC107771257	2.215811	2.292745	2.375111	0.27168	0.176905	0.376147	0.237552	0.185054	0.156414	0.081229	0.093457	0.08445	0.166918	0.436212	0.102598	2.470629	2.591324	2.297868	0.287396	0.211401	0.212722	0.208701	0.220431	0.278612	0	0.023181	0.157528	0	0.080208	0
LOC107811104	3.50653	4.047975	3.860992	3.233214	2.496037	3.568884	2.954149	2.117226	3.072612	2.705054	3.322086	1.84328	2.602345	1.836217	2.687258	4.389958	3.191874	3.834258	2.32468	2.438944	1.535047	3.943571	3.189217	3.974515	1.477773	0.715598	1.473577	0	1.425564	2.890854
LOC107784327	1.377264	1.414598	1.501284	1.029874	1.02525	1.269195	1.356867	0.971304	1.251988	1.279054	1.670881	1.292834	1.72788	1.693325	1.36871	1.677983	1.161594	1.727647	1.042619	1.165071	0.638013	2.300379	3.335984	2.102151	0.856821	1.946781	1.70533	0	3.157448	8.110316
LOC107763788	0	0	0	0	0	0.095287	0.074337	0	0	0	0	0	0.197326	0	0.181933	0.066046	0.085623	0	0	0.149948	0.080831	0.074016	0	0	0	0	0	0	0	0
LOC107780014	0.683214	0.27674	0.395936	0.343865	0.201518	0.489693	0.063671	0.129724	0.07127	0	0.05323	0	0	0.082817	0	0.395992	0.220014	0.169788	0.077031	0.064217	0	0.443777	0.26784	0.158688	0.28793	0.211254	0.089723	0	0	0
LOC107778226	2.75683	3.311945	3.158961	3.405747	2.772073	3.906997	3.520959	3.533265	3.294115	2.138386	2.987504	1.958516	1.604219	2.324028	1.993536	2.101083	2.300148	2.615879	1.144407	1.59006	1.942849	3.662751	3.426495	3.143386	0.396076	0.348721	0.740534	0	0.301645	3.874066
LOC107785413	4.117187	4.195289	4.548291	1.243321	1.037288	2.366937	0.695449	0.879459	1.073721	0.390326	0.661598	0.748804	0.636571	0.93576	0.117382	3.004213	3.867061	4.732203	1.131497	1.112579	0.6519	1.098368	1.134885	1.793034	0.506078	0.039783	0.709652	0	0.5506	0
LOC107821311	0.511214	1.146852	0.957142	0.071251	0.034797	0	0	0.0336	0	0	0.055148	0	0	0	0	0.556784	0.759809	0.967488	0.11971	0	0.035864	0	0	0	0	0	0.046478	0	0	0
LOC107798782	0	0	0	0	0	0	0	0	0	0	0	0	0	0	0	0	0	0	0	0	0	0	0	0	0	0	0	0	0	0
LOC107774402	0	0	0	0	0	0	0	0	0	0	0	0	0	0	0	0	0	0	0	0	0	0	0	0	0	0	0	0	0	0
LOC107811430	5.582464	6.118153	5.835546	6.353796	6.330984	6.837529	6.223247	5.8566	6.883041	5.254114	5.698216	4.656521	4.877184	5.30001	4.968147	6.582371	6.58781	6.677581	4.517418	5.125875	3.560688	7.583226	6.981005	6.961125	1.920829	2.974299	2.108862	0	2.296364	4.369268
LOC107787735	15.208	14.13632	13.77302	23.54831	22.03524	25.47025	22.76698	22.77856	24.31801	21.44697	24.02555	20.06563	27.14833	22.67242	21.73455	12.9359	16.44825	15.7425	18.10651	16.78591	15.58298	19.7593	20.33548	19.6951	14.43981	11.17056	17.00787	10.83254	17.79371	13.11222
LOC107829439	0.106104	0.14505	0.184467	0.080104	0.109535	0	0.029665	0.01511	0.016602	0	0.0124	0	0	0.019292	0	0.06589	0.170842	0.171393	0.017944	0.089756	0	0.029537	0.046795	0	0	0	0	0	0	0
Novel05263	0.772563	0.140819	0.537258	0.559922	0.410169	0.66448	0.388789	0	0.580253	0.301339	0.216689	0.39161	0.860028	0	1.110112	0.115143	0.298544	1.267152	0.313577	0.261414	0	0.258075	2.180645	0.968979	0.195351	0	0.365243	0	1.487757	0
LOC107797424	5.840641	6.679209	7.922934	5.965379	5.135265	5.15951	2.184177	2.193234	1.746322	4.824734	5.164976	4.52576	3.975674	3.93675	3.207341	9.009854	7.475481	8.986245	3.963702	4.122562	4.74998	1.646603	2.395438	2.799578	1.599158	1.190554	2.770061	0	4.656637	0
Novel01243	0.729258	0.780217	0.716615	1.091543	0.785577	0.852219	0.74463	0.406368	0.833499	0.772955	0.867082	1.044687	1.0236	0.899357	0.585775	0.70884	0.490104	0.850998	0.643479	0.59008	0.549427	0.873815	0.92293	1.060481	0.28061	0.264707	0.4497	0	0.305296	0
LOC107760115	2.447502	2.626821	2.477489	4.084523	3.932475	4.10285	3.565433	3.12306	3.431617	3.108891	3.838846	3.237611	4.23029	3.214665	3.470594	3.22779	2.753389	3.265282	3.300515	3.882034	2.305589	3.630741	3.735393	4.459895	1.180753	1.053023	2.112469	0	1.08529	0.99561
Novel02468	0.024272	0.066364	0	0.087958	0.322167	0.156575	0.061075	0.124434	0.091152	0.260355	0.255297	0.676697	0.378285	0.450159	0.672637	0	0	0	0	0	0	0	0	0	0	0	0	0	0	0
LOC107784167	2.617392	3.287239	2.608206	20.647	28.7528	18.01657	19.94528	18.65461	11.71724	45.16304	21.41949	20.14391	23.23867	26.9178	30.56621	3.924754	3.947115	4.02174	39.02949	37.93729	29.22749	17.96664	11.51541	9.975287	114.2878	72.8382	69.83116	38.80572	99.42542	120.3912
LOC107777278	40.51212	47.67612	48.58639	74.44432	62.58916	73.916	55.03922	53.58633	55.4979	43.32532	44.71872	40.9796	35.57254	38.30056	41.95034	43.96718	44.46821	43.60117	63.00013	66.19781	57.32136	56.02429	57.56431	57.38347	41.11237	44.96412	50.06457	22.54622	24.06828	40.37956
LOC107813940	1.254377	2.875347	1.916467	2.376114	1.950831	0.490403	8.384921	8.249413	6.245192	2.409287	5.837148	1.991013	2.792781	2.819866	3.70632	5.551929	1.909557	3.514053	2.622854	2.572405	2.357361	5.650493	7.946279	5.522407	2.354843	2.009825	2.515882	0	1.647005	2.350304
LOC107781449	0	0	0	0	0	0	0	0	0	0	0	0	0	0	0	0	0	0	0	0	0	0	0	0	0	0	0	0	0	0
LOC107790046	42.54047	30.25522	35.18266	30.22486	26.41201	40.00251	35.74987	36.70097	40.71513	46.84399	40.3813	58.95594	23.88542	32.42806	29.49013	23.90684	34.10827	25.92121	41.53106	45.67359	40.04186	29.45018	39.20094	37.67929	16.77813	27.15122	29.70834	5.913013	15.92264	12.78106
LOC107827543	0.472173	0.602459	0.300997	0.171106	0.111416	0.101529	5.465243	3.335085	2.777995	0.460429	2.361763	0.132968	4.345207	2.30086	3.19853	0.09383	0	0.234684	0.063884	0.133142	0.057417	3.916955	0.305425	0	0	0.0438	0.148819	0	0	0
LOC107819362	4.580395	4.95976	4.218173	4.806969	3.41097	5.021999	5.401306	3.874865	4.896354	4.466463	5.946555	5.670371	6.764956	7.520216	4.142634	5.238256	5.43273	5.511141	4.27972	3.721245	4.136037	3.711587	4.680244	5.119229	2.694827	2.271666	3.912842	6.486396	8.514994	2.804086
Novel01978	0	0	0	0	0	0	0	0	0	0	0	0	0	0	0	0.608841	0.263102	0.27072	4.790071	4.684385	2.40098	3.714804	3.042793	1.613006	8.148873	11.87342	4.184468	0	3.933403	5.613032
LOC107827900	6.395203	6.799846	5.823939	8.345731	8.825184	8.021563	7.662761	5.464235	6.861827	5.716453	7.847561	2.830062	6.18695	4.235946	4.375908	7.545712	7.281573	8.797903	7.416469	7.921642	3.055136	7.947621	8.125715	8.275756	4.042741	2.542417	3.32939	0	1.466133	0
LOC107777208	0	0	0	0	0	0.113601	0	0.107679	0	0	0	0	0	0	0.140058	0.131768	0	0	0	0.390819	0.108602	0	0.107215	0.226482	0	0.162314	0	0	0	0
LOC107790988	8.043401	9.385716	7.532175	6.337845	5.59331	6.375964	6.403817	5.977406	5.784481	5.886827	6.92243	5.955253	6.878589	5.927453	6.815821	8.097789	6.724229	9.37232	4.558405	4.881574	3.335889	5.29373	5.763555	5.753107	2.873446	2.668245	2.644234	1.269794	2.820939	5.489355
LOC107830115	0.134135	0.229214	0.218627	3.09875	3.100817	1.514222	4.725206	5.959651	4.140013	4.218257	5.220087	4.292036	3.807687	4.097375	10.0501	0.724693	0.080991	0.600021	7.077774	7.162735	6.774216	10.58587	5.575643	5.029603	6.444327	14.55786	3.428361	8.393488	9.686613	24.88133
LOC107818656	0	0	0	0	0	0	0	0	0	0	0	0	0	0	0	0	0	0	0	0	0	0	0	0	0	0	0	0	0	0
LOC107800056	77.80421	67.32519	74.88745	75.65352	70.28058	87.45713	49.49719	48.88392	55.60177	56.4041	62.93345	62.73877	60.04748	59.3378	55.91776	67.75493	70.46757	78.90831	73.61997	73.76482	61.21287	53.57037	61.47966	74.19908	42.20116	41.05651	43.63763	51.51945	68.35455	57.53599
LOC107802105	0	0	0	0	0	0	0	0	0	0	0	0	0	0	0	0	0	0	0	0	0	0	0	0	0	0	0	0	0	0
LOC107806688	0.654747	1.153661	0.607102	0.632712	0.502115	1.173222	0.842052	0.522138	0.532745	0.170256	0.489716	0.258136	0.485916	0.238099	0.806417	0.715613	1.054234	1.496968	0.79727	0.738495	0.437903	1.020687	0.731538	0.456228	0.110373	0.182207	0	0	0.420291	0
LOC107776455	40.46053	36.87478	41.44455	20.93533	19.87413	23.62426	21.89857	23.07014	25.34946	16.68301	16.43127	18.35836	10.38024	15.39166	11.81747	27.19049	37.67827	29.25828	9.379652	10.08285	13.45877	16.65782	21.09863	19.32258	2.767874	2.482071	7.091705	5.798597	4.684362	20.05399
LOC107773825	0	0	0	0	0	0	0	0	0	0	0	0	0	0	0	0	0	0	0	0	0	0	0	0	0	0	0	0	0	0
LOC107820734	12.44758	11.40653	12.57774	7.078913	5.889104	7.375119	10.02118	8.888833	8.636797	7.708046	12.20043	9.575753	9.507596	9.020088	10.93408	2.403622	2.655244	2.116833	1.083311	1.537196	1.325828	1.745196	2.103753	2.564055	0.545644	0.663719	1.127565	0	1.202915	0
LOC107809240	342.7415	300.2521	367.3967	148.6724	139.8342	188.2822	144.8551	155.4592	200.5099	63.31606	82.08523	77.23205	61.61044	76.2276	69.48375	229.1927	350.6901	288.8694	79.47633	75.66086	112.3074	163.0485	232.0582	272.2388	16.61846	11.20742	45.84745	28.27089	9.753932	27.49856
LOC107777636	0.366658	0.047738	0.227663	0.189814	0.092698	0	2.548123	2.8643	6.933876	0.102154	0	0	3.498593	1.885744	0.322567	0.546468	0.202413	0.156205	0.053151	0	0.143314	2.449648	0.970251	0.656968	0	0.072883	0	0	0.252175	3.23872
LOC107811464	47.03223	39.4882	28.45788	18.17314	20.19693	19.01461	17.13131	18.56606	21.1106	20.75634	20.5593	21.02405	21.86621	24.24625	21.84716	33.51279	37.52403	29.54132	6.855679	7.304035	12.56136	21.43616	13.69046	11.72378	0.890461	1.415549	2.558974	0	0.50234	3.225816
Novel00263	1.211093	0.896806	0.789583	0.137149	0.133957	0.488277	0.190462	0.258698	0.355321	0.147621	0.530762	0.447635	0.505577	0.165155	0.310758	1.635795	0.438757	1.185084	0.153617	0.256125	0.207101	0.252854	0.400599	0.712032	0	0.105322	0.626244	0	0	0
LOC107773074	0	0	0	0	0	0	0	0	0	0	0	0	0	0	0	0	0	0	0	0	0	0	0	0	0.03426	0	0	0	0	0
LOC107795200	21.30702	18.88969	21.63838	16.8902	13.02245	18.42891	14.63484	14.26665	17.4073	10.68819	13.81517	12.26014	12.84794	9.387369	7.009277	24.2496	21.31011	24.97351	10.67185	11.58291	12.95491	14.40063	21.1736	26.19591	3.540002	3.230781	7.385453	0	5.424882	6.333871
LOC107776905	15.85291	16.96153	16.27244	16.78182	13.79711	17.62751	8.196485	9.370281	10.5815	12.72687	11.72517	13.06274	14.21485	15.16252	14.53245	17.23492	10.1752	16.10949	18.09174	17.02946	16.65655	10.68202	10.95679	8.352824	20.67439	29.85407	16.09317	43.48467	28.33307	30.88331
LOC107788138	1.113924	1.245933	1.430459	0.412838	0.358427	0.462711	0.403446	0.346097	0.546669	0.197494	0.408294	0.299433	0.366374	0.193333	0.233857	1.528134	0.929398	1.207968	0.205515	0.128496	0.253979	0.359421	0.625262	0.714441	0.064015	0.105678	0.089766	0	0.48753	0
LOC107816805	1351.7	1405.225	1450.355	1745.51	2041.865	1120.236	1471.936	1381.619	2060.846	2354.246	2227.381	3189.97	2549.041	2637.393	2096.388	1404.677	1879.349	1198.782	711.2221	606.664	983.6116	1048.209	1045.333	1501.16	489.7804	333.0113	732.44	244.0472	584.6364	139.3426
LOC107826353	0	0	0	0	0	0	0	0	0	0	0	0	0	0	0	0	0	0	0	0	0	0	0	0	0	0	0	0	0	0
LOC107827457	1.827719	1.584485	1.898807	0.323087	0.236676	1.150257	0.261729	0.266624	0.209261	0.130409	0.218809	0.188306	0.049625	0.048633	0	1.063039	1.248931	1.495583	0.09047	0.188552	0.528533	0.335058	0.825745	0.652308	0	0	0.158065	0	0	0
LOC107807890	0.934371	0.450828	0.191113	0.298762	0.340444	0.472736	0.0461	0	0.051602	0	0.07708	0	0.183557	0.239848	0.507713	0.245751	0.106198	0.040977	0.167318	0.27897	0.451145	0.091802	0	0.114895	0	0.076477	0	0	0	0
LOC107781100	2.644037	3.500426	2.516149	4.210797	3.201582	3.321427	6.09275	5.398148	5.539534	4.858003	7.357454	5.619659	7.064109	5.890482	6.7987	3.899198	3.818099	3.755734	3.869148	4.802961	2.842921	5.555125	5.278172	3.723515	4.081816	3.950067	4.342159	1.990379	4.153783	1.720892
LOC107763789	3.971249	3.248093	3.469826	3.413251	2.396744	2.868281	2.767174	2.209893	3.365105	2.363199	2.170593	2.89048	2.357787	3.177121	1.400465	2.564796	2.911851	2.732986	3.699103	4.272472	2.823183	1.989896	3.556795	3.171598	2.240079	0.963456	3.417974	2.912825	1.862878	1.259222
LOC107830453	15.5544	11.51036	11.44094	5.871456	3.882028	6.146015	5.38014	3.634899	5.522987	2.203825	3.309314	3.144804	3.218883	4.82241	4.366388	11.39301	18.75146	14.02478	2.428235	3.683101	3.303974	5.384695	5.62872	6.287604	0.084041	0.138736	0.353539	0	0.160009	0
LOC107822571	0	0	0	0	0	0	0	0	0	0	0	0	0	0	0	0	0	0	0	0	0	0	0	0	0	0	0	0	0	0
LOC107822439	13.30012	13.11876	15.76903	10.27617	9.888048	10.09093	9.037276	9.116363	10.43196	6.197356	6.787952	6.329316	6.910974	6.152882	6.436651	14.11415	13.09301	13.99509	7.708976	7.654946	7.408474	10.45701	12.10284	14.3192	3.591897	3.923773	5.098946	3.009959	3.039471	1.301213
LOC107832549	7.942552	4.992177	6.071015	5.14854	5.149895	6.551639	4.422031	4.855738	4.564087	4.874003	5.185231	4.916875	5.945321	6.797491	3.373249	4.847292	6.019472	4.390085	5.419355	4.459928	5.183022	3.545242	4.650439	6.870251	2.337316	2.000699	4.45095	0	1.318561	0
Novel03176	3.600226	2.895143	4.29553	3.837203	4.435018	4.705537	4.203847	3.377712	3.84398	4.199043	4.801464	6.321697	4.47933	2.772473	3.187993	3.103739	3.273526	3.789348	3.868158	3.224693	4.184811	2.947689	3.860579	5.238624	2.677518	1.669817	4.672352	0	2.718866	0
LOC107801618	0	0	0	0	0	0	0	0	0	0	0	0	0	0	0	0	0	0	0	0	0	0	0	0	0	0	0	0	0	0
LOC107816830	0.315041	0.589354	0.129723	0.255368	0.49885	0.285228	0.250331	0.26918	0.140104	0.226362	0.313921	0.084049	0.2215	0.397962	0.289313	0.92672	0.288338	0.568649	0.319682	0.490927	0.090734	0.276947	0.292513	0.121314	0.272527	0.276857	0.07839	0	0.079827	0
LOC107776320	0.401377	0.182903	0.209345	0.618167	0.426198	0.776754	0.302987	0.137179	0.263782	0.587091	0.36588	0.440823	0.312773	0.569248	0.32957	0.209375	0.426541	0.359093	0.773851	0.814891	0.878551	0.636882	0.42485	0.839039	0.507463	0.390941	0.901352	0	1.159424	0
LOC107758987	0.558853	0.463023	0.309145	1.288744	0.809198	0.600835	1.704487	0.694544	0.572374	1.387151	1.923716	1.158876	1.131133	0.665107	1.355761	0.530037	0.392654	0.568157	0.927958	0.730615	1.946063	0.890997	0.98589	1.221327	1.220422	1.908662	1.441133	3.633262	1.712147	0
LOC107771792	5.356164	7.081588	5.071323	8.70245	9.901743	8.786491	12.61259	10.82879	8.499047	15.61977	19.29007	13.44764	19.90318	14.97863	23.25434	6.352541	6.534938	4.818144	8.190873	11.29546	7.72871	10.79417	9.271536	7.332933	8.107108	7.942561	7.48967	8.991583	10.41144	9.329005
LOC107758978	0	0	0	0	0	0	0	0	0	0	0	0	0	0	0	0	0	0	0	0	0	0	0	0	0	0	0	0	0	0
LOC107831593	85.29638	39.02416	31.76004	11.07452	16.01369	9.544856	44.56323	46.972	55.3315	2.59713	20.59107	5.423307	7.298238	10.87743	6.378442	56.64177	51.85679	36.04739	4.989437	3.119586	10.83728	67.01285	77.04418	53.31974	0.604391	0.475115	1.614307	9.767649	1.315123	8.445162
LOC107828925	12.94208	13.0644	14.73461	12.86351	10.79073	10.07966	9.516003	9.0264	11.3852	6.094777	6.955115	7.088336	7.751962	8.486328	5.055361	13.18835	12.50314	13.21971	7.066167	7.270005	8.178731	9.41823	12.46446	15.90583	2.576807	1.843335	4.857943	2.429244	3.27075	8.401348
LOC107812650	0	0	0	0	0	0	0	0	0	0	0	0	0	0	0	0	0	0	0	0	0	0	0	0	0	0	0	0	0	0
LOC107828957	0.121109	0	0.105278	2.578393	2.464805	2.14842	3.453705	1.759144	2.728865	1.240017	2.505196	0.562741	3.168283	3.170982	4.847826	0.090251	0	0.045146	0.798807	0.512251	0.331361	1.31484	1.33533	0.569625	0.229678	0.252772	0.286283	0	2.623784	11.23255
LOC107824728	0.613526	0.419365	0.466659	1.042169	1.560803	1.566586	0.643238	0.524213	1.584014	0.673048	0.645307	0.712695	0.597612	0.167332	0.157427	0.40005	0.370448	0.114352	0.07782	0.324375	0.139886	0.128093	0.202939	0.641257	0	0	0	0	0	0
LOC107807509	0	0	0	0	0	0	0	0	0	0	0	0	0	0	0	0	0	0	0	0	0	0	0	0	0	0	0	0	0	0
LOC107801026	2.688149	2.426131	1.860326	4.870654	3.440962	3.563508	3.940194	3.567888	4.557457	2.519487	4.831282	3.527803	4.212722	3.615972	2.732256	3.033991	3.454233	3.093753	3.09851	3.775264	2.713463	4.533478	4.811313	5.755696	0.956898	0.581027	1.388087	0	1.130829	0
LOC107762253	0	0	0	0	0	0	0	0	0	0	0	0	0	0	0	0	0	0	0	0	0	0	0	0	0	0	0	0	0	0
LOC107822927	0.064093	0.102222	0.139286	0.014516	0	0	0	0.027381	0	0.015625	0.022471	0.013537	0	0	0	0.035821	0	0.035838	0.065037	0	0	0.040144	0.014134	0.016747	0	0	0	0	0.077141	0
LOC107788611	0.792541	0.393984	0.4071	0.065273	0	0	0.030215	0.092341	0.135286	0	0	0	0.360928	0.157204	0.073949	0.348992	0.730859	0.698304	0	0	0.032855	0.330936	0.349537	0.112959	0	0	0	0	0	0
LOC107762049	0.476613	0.99906	0.41431	1.122647	0.716955	0.871109	2.038755	2.972787	1.297649	0.185903	1.036024	1.046905	0.636687	1.507888	0.391346	0.639311	0.552538	0.426402	1.547628	1.370818	1.521377	2.109569	1.219172	0.996311	0.180775	0.066317	0.394323	0	1.606209	0
LOC107788605	5.474012	4.439651	4.627476	5.641633	3.688372	6.695126	4.001582	3.861862	3.819071	4.260488	3.274948	4.75188	3.577979	4.437817	3.556586	4.116664	5.336872	4.492963	2.649924	4.163325	4.534373	3.229401	3.278024	3.464355	1.841318	2.585481	4.333013	0	2.176005	0
LOC107762099	3.547459	4.270847	3.083731	2.380606	2.131437	1.412575	2.240738	2.507161	2.631515	0.555184	0.153548	0.111	0.780065	0.238895	0.53941	1.011737	5.11956	1.20811	1.111023	1.593074	1.677585	2.231075	3.167724	2.014115	0.221485	0	0.103526	0	0.421697	0
LOC107803071	3.831604	3.849174	2.725144	5.759101	7.204717	4.213066	10.73678	9.598263	10.21954	5.137409	4.304863	3.568107	9.984901	8.122677	6.211804	5.191483	7.192981	6.622172	7.024993	4.861909	7.544915	9.417806	7.181921	8.009582	10.40426	5.08906	4.168414	12.45517	17.3986	8.076607
LOC107805027	19.64585	13.88687	14.99391	18.07273	15.23838	19.72938	13.49656	12.68952	19.3498	10.6399	11.39923	13.40825	14.75384	13.55699	5.938896	14.22945	14.72046	15.28364	11.04408	11.79416	11.30829	12.0347	18.76289	23.52896	6.688583	3.987266	9.086016	23.64583	11.40822	5.111078
LOC107768500	0	0	0	0	0	0	0	0	0	0	0	0.856582	0	0	0.017638	0	0	0	0	0	0	0	0	0	0	0	0	0	0	0
LOC107811817	0.532569	0.428269	0.435719	0.028381	0.194045	0.067362	0.052552	0.026767	0.088235	0	0.175736	0	0.104623	0.068354	0	0.560289	0.242121	0.443765	0.063578	0.053002	0	0.052325	0.193431	0.098231	0.039608	0	0.037027	0	0	0
LOC107783788	9.995264	10.63928	10.64528	14.34316	16.50742	17.30865	16.47894	18.09093	16.90552	13.28073	14.04412	11.86066	13.84488	14.44201	13.11725	6.794608	6.22866	6.911489	6.774573	7.067603	5.114618	8.219866	7.806963	8.373587	4.003341	3.291131	5.050088	2.728255	6.061012	0
LOC107784898	40.80026	39.59254	32.89968	42.10258	49.64988	33.92247	40.80439	32.40399	49.6142	52.97143	39.12644	52.39317	51.73488	48.24153	30.07115	29.18823	34.53635	25.78315	30.28208	25.4419	28.35087	22.45597	25.50044	33.87185	8.253429	7.353142	21.30981	22.23082	32.17661	0
LOC107818931	3.450413	5.607285	3.794385	0.075323	0.22071	0.134082	0.034868	0	0	0.081074	0.02915	0.070241	0	0.045352	0.042668	4.36803	3.614518	3.905134	0.168734	0	0	0.069434	0.51336	0	0	0	0	0	0	0
LOC107778079	0	0	0	0	0	0	0	0	0	0	0	0	0	0	0	0	0	0	0	0	0	0	0	0	0	0	0	0	0	0
LOC107796374	0	0	0	0	0	0	0	0	0	0	0	0	0	0	0	0	0	0	0	0	0	0	0	0	0	0	0	0	0	0
LOC107787149	14.89343	15.5936	15.31275	13.73929	12.66255	13.50381	13.04622	14.12082	14.93182	6.332472	7.580241	7.161774	12.68359	12.13297	8.660854	13.32991	15.55289	14.17677	8.759913	7.861924	10.35575	13.41214	14.7491	18.73683	3.146498	2.543046	4.366238	2.780916	5.429153	14.42637
LOC107773614	0.667258	0.868748	0.290017	0.302251	0.506086	1.024834	0.159902	0.081446	0.044747	0.092952	0	0	0.159172	0.155988	0.048918	0.816897	0.736717	1.243673	0.338544	0.766045	0.130404	0.159213	0.210202	0.249078	0	0	0.112664	0	0	0
LOC107832368	0	0	0	0	0	0	0	0	0	0	0	0	0	0	0	0	0	0	0	0	0	0	0	0	0	0	0	0	0	0
Novel01793	0.63343	1.198993	0.93183	0.529712	0.431154	0.576243	0.040868	0.083265	0.091491	0.095027	0.068333	0.123494	0	0	0.05001	1.052996	0.84731	1.089805	0.296659	0.49462	0.26663	0.040692	0.214895	0.152783	0.308018	0.474584	0.863842	0	0	0
LOC107818894	2.121396	2.189281	2.549176	1.158779	1.214627	1.844732	1.282147	1.412745	1.347299	0.730104	1.093768	1.317805	0.833494	1.259273	0.448276	2.13882	2.170003	1.930471	0.601475	0.870887	0.91047	0.599229	1.293337	1.141248	0.078885	0.086817	0.036872	2.231027	0.600774	0
LOC107814530	0.223527	0.543245	0.064769	0.270005	0	0.480637	0.124988	0.063662	0	0.145311	0	0	0	0	0.076474	0.055524	0.287927	0.166649	0.453639	0.126059	0.271814	0.186673	0.065722	0.077877	0	0	0.176127	0	0	0
LOC107787376	39.38793	38.90434	38.27249	45.91211	47.71763	55.03981	41.64137	40.7902	44.48143	44.89336	41.03061	50.5238	46.25764	56.97501	39.7388	28.58356	45.07475	35.16926	36.77698	36.76482	43.16337	29.92311	37.60767	37.0191	39.76044	33.28497	36.4993	62.67735	54.10838	31.87716
107807622	0	0	0	0	0	0	0	0	0	0	0	0	0	0	0	0	0	0	0	0	0	0	0	0	0	0	0	0	0	0
LOC107824819	33.94011	31.89092	30.52541	31.95327	27.45901	35.65818	24.15935	26.25009	25.41605	34.51385	36.0128	33.69207	30.99757	33.75295	36.23241	31.95533	38.70716	32.84649	28.09833	27.45472	24.18189	26.58729	27.94523	24.3501	18.11086	26.08793	22.52554	13.27546	14.44829	15.30405
LOC107799443	0	0.061046	0.058226	0	0	0	0	0	0	0	0	0	0	0	0	0	0	0	0	0	0	0	0	0	0	0	0	0	0	0
LOC107761704	29.31481	28.07302	30.01013	14.79549	13.69404	24.63766	15.69487	12.7144	16.97425	14.43793	21.10336	15.74587	15.03302	15.13822	14.58577	23.95235	31.69891	30.06509	10.26788	12.36769	9.466031	16.6835	18.44159	18.78051	2.869077	2.174055	7.386829	0	2.865623	2.300227
LOC107828469	24.38604	25.6793	23.81828	20.81	20.56815	27.83819	19.07473	19.50946	20.06501	17.81234	17.48377	18.05569	20.58893	20.67537	16.82672	20.07824	25.05898	22.53878	17.51632	19.66314	19.61661	17.3907	21.75179	19.95151	10.73902	9.46775	16.73207	13.06327	10.9928	2.823644
LOC107764712	0.136169	0.206836	0.118369	0.041121	0.040164	0.097599	0.03807	0.038782	0.085228	0	0	0	0.202114	0.148554	0	0.270596	0.131551	0.20304	0	0	0	0.037906	0	0.047441	0	0	0	0	0	0
LOC107798491	0	0	0	0	0	0	0	0	0	0	0	0	0	0	0	0	0	0	0	0	0	0	0	0	0	0	0	0	0	0
LOC107781524	0	0	0	0	0	0	0	0	0	0	0	0	0	0	0	0	0	0	0	0	0	0	0	0	0	0	0	0	0	0
LOC107809720	0	0	0	0	0	0	0	0	0	0	0	0	0	0	0	0	0	0	0	0	0	0	0	0	0	0	0	0	0	0
LOC107792594	5.687081	7.623016	6.520397	8.54635	8.249334	6.701895	8.237883	7.688088	6.320878	9.337963	12.27438	6.142934	8.79101	9.099102	13.95042	8.293061	6.068105	7.269347	8.744697	9.454249	5.136147	11.07318	6.986604	5.58815	10.00419	13.72128	5.57955	3.965107	9.647698	18.61052
LOC107824758	9.716639	10.4976	9.900827	12.70865	12.12819	12.93715	11.28014	13.30545	13.95547	10.85541	10.10717	9.459247	14.25512	12.63621	10.17103	9.158957	10.00875	8.395557	12.53693	10.94133	9.389921	9.726795	12.77094	9.214228	11.79665	8.147811	11.10401	0	12.70168	15.91508
LOC107778698	0.057942	0	0.050368	1.574781	1.743217	0.685245	13.31602	10.34705	21.32429	5.424094	3.981653	5.776241	31.99303	30.65787	14.27287	0.215893	0.055977	0.086397	0.940732	0.441136	0.845508	3.677568	1.942137	2.180203	1.098849	0.161245	2.465389	0	12.8319	0
LOC107803606	1.337509	3.360417	2.608148	1.670203	0.959608	0.932748	2.001096	1.72965	0.678764	0.493496	2.230592	0.366475	1.006037	0.552113	2.189032	3.259523	1.466761	2.344697	1.467256	1.987667	1.2528	1.600011	1.689943	0.717874	0.182813	0.905374	0.427251	0	0.696135	0
LOC107815563	5.493794	6.191759	6.525491	6.914748	7.941312	6.267203	10.9745	12.03969	12.04803	9.119411	6.410617	7.475698	7.889808	8.464046	8.522584	4.906528	5.793647	6.22195	5.957719	4.611896	8.184982	11.80275	12.79907	11.52806	9.543883	6.768922	8.773285	11.99646	12.11408	23.33745
Novel00225	0.856929	0.341681	0.977694	0.776334	0.900442	1.324373	0.898429	1.006752	0.95537	1.25342	0.563324	1.900387	1.073188	1.285442	0.989469	1.077611	0.517416	1.237824	0.54347	1.223273	0.928071	0.939282	0.708626	1.175556	0.338568	0.447133	0.50641	0	0.257847	0
LOC107761320	0.452851	0.240752	0.131218	0.20513	0	0.040573	0.189913	0.419172	0.248008	0.257592	0.555694	0.095645	0.12603	0.576378	0.96832	0.196855	0.145831	0.140675	0.076587	0.255387	0.137669	0.945469	0.166435	0.11833	0.047712	0.315055	0.044603	0	0.181682	0
LOC107808079	15.47839	13.08442	16.57504	11.78714	7.741385	18.93233	7.902325	6.325074	11.37271	25.26501	16.83072	21.3205	11.11259	12.23635	4.144329	10.44793	12.13611	12.37601	9.218973	13.3783	11.35462	7.399899	9.200909	13.01274	24.67445	12.95344	65.3554	8.021185	15.65968	6.93516
Novel03212	0.048455	0	0	0.482875	0.085752	0.052095	0.568977	1.324838	0.409424	1.039493	0.917369	0.982465	1.132754	1.37441	1.293053	0	0	0	0.983374	1.106717	0.441917	0.242796	0.341924	0.506451	1.592805	2.224909	1.603555	0	2.79935	0
LOC107798796	0.628557	1.909508	0.546391	26.66882	26.32627	4.730431	68.36	72.05505	27.14539	10.72615	17.33596	11.8595	17.49296	18.17171	34.7297	2.966543	1.012065	1.17154	42.20218	70.18652	36.0195	43.91868	58.67707	12.70138	62.5154	36.8786	20.42986	119.8686	74.64375	155.4585
Novel01116	0.060485	0	0	0	0	0	0.050731	0	0	0.05898	0	0	0.067332	0	0	0.585953	0.8765	0.586221	0.184127	0	0.330978	0.101025	0.160055	0.316093	1.070595	0.08416	0.142976	0	0.291195	0
LOC107825530	0.219468	0	0	0.066276	0	0	0	0.062507	0	0	0	0	0	0	0	0	0	0	0	0	0	0	0.064529	0	0	0	0	0	0	0
LOC107779332	0	0	0	0	0	0	0	0	0	0	0	0	0	0	0	0	0	0	0	0	0	0	0	0	0	0	0	0	0	0
LOC107783737	28.6586	35.5098	23.69935	21.97637	15.48135	10.73209	127.6582	87.49175	87.36878	6.593942	13.0959	12.06045	50.30422	42.15522	25.22808	24.07597	24.78315	19.92578	10.56492	12.2578	28.09496	94.12068	84.92534	63.49791	0.135705	0.298699	0.126862	7.676011	2.067007	6.63672
LOC107799860	5.907246	5.294246	6.088085	3.602368	3.59093	3.184302	3.993924	4.138516	4.900744	2.377524	2.868535	1.589805	2.058424	2.213628	2.317725	5.438589	4.363167	5.611876	2.009158	1.771833	1.55208	5.67124	5.585859	7.405681	1.448196	0.614755	1.179765	0	1.10292	0
LOC107814765	0.784644	1.18603	1.497236	1.716437	1.219266	1.31682	1.091507	1.488008	1.38348	1.380961	0.764902	1.406618	1.278258	1.482355	3.437414	1.169435	0.684078	1.426793	3.786816	2.622641	2.391179	2.23753	2.076323	1.620216	1.354966	4.207327	2.261917	5.474452	1.566303	4.73324
LOC107760906	1.579607	2.418556	2.581463	0.085863	0.307503	0.37362	0.132488	0.215945	0.207621	0.215644	0.110762	0.160139	0.175844	0.516982	0.162127	2.330707	2.472173	2.119795	0.192345	0.293973	0.2305	0.131917	0.390129	0.231141	0.159768	0	0.448072	0	0	0

LOC107777742	0	0	0.066085	0	0	0	0	0	0	0	0	0.064226	0	0	0	0	0	0.056678	0	0	0	0	0	0.079459	0	0	0	0	0.732002	0
LOC107804128	7.29749	5.763994	5.544735	5.337897	4.065707	6.567137	1.496176	1.616526	3.349478	1.73946	1.440342	1.598365	1.263682	1.061494	0.610292	2.37665	6.162213	4.231569	1.974664	2.194906	1.626884	1.534867	2.002581	2.259951	0.136685	0	0.83056	0	0.260242	0
LOC107784589	1.173307	1.222085	1.396334	1.708323	1.285414	1.877155	1.546447	1.479888	1.678555	0.898953	1.733596	1.144649	1.212846	1.37145	1.304604	1.811153	1.389903	1.332952	0.892943	1.146144	0.50956	1.621433	1.355271	1.664318	0.264896	0.349837	0.495269	0	0.672466	0
LOC107786751	27.54963	24.06641	25.66135	35.42045	33.51501	37.28886	28.4954	26.63729	28.34398	35.43483	33.32945	31.76546	40.365	34.52703	29.73247	24.34819	29.50905	27.94418	29.91514	33.04055	29.65482	28.04337	29.75867	32.50235	30.64539	22.3748	34.75084	22.54864	36.62132	19.49568
LOC107801129	0	0	0	0	0	0	0	0	0	0	0	0	0	0	0	0	0	0	0	0	0	0	0	0	0	0	0	0	0	0
LOC107772390	0.179383	0.163485	0.051978	0.9209	1.216927	0.192858	0.300912	0.357629	0.168412	2.565505	4.486271	0.505158	0.332819	0.26093	3.498157	1.247645	0.46213	0.133737	2.184303	3.287818	2.563068	0.049936	0.263712	0.062497	0.680383	0.998392	0.070672	0	0	0
LOC107788606	0.020525	0	0	0	0	0	0	0	0	0.020014	0	0.01734	0	0	0	0	0	0	0	0	0	0	0	0	0	0	0	0	0	0
LOC107763482	0.506901	0.61597	0	0.459227	0	0	0.28344	0.28874	0	0.494292	0.11848	0.285496	0.564289	0.921673	0.173423	0	0	0.125972	0	0	0.154101	0	0	0	0.213626	0	0	0	0	0
LOC107827744	5.945288	6.255385	6.400617	5.794834	5.774029	5.335254	6.243347	5.960868	5.490939	3.487871	5.411583	4.410215	4.304636	4.253702	5.17602	6.71169	5.245554	6.030074	4.348897	4.974787	4.18788	6.512444	6.025784	6.432935	1.751848	1.883156	2.837401	1.152228	3.257872	4.981113
LOC107784342	0.126828	0	0.110248	0	0.224451	0	0.106376	0	0	0	0.355726	0	0	0	0	0	0	0.094555	0	0.429149	0	0.105917	0	0	0	0	0	0	0	0
LOC107815850	23.58298	19.54193	20.80033	29.56063	26.91728	29.32668	23.68973	23.51318	28.26753	27.27243	21.95976	27.91631	30.32729	30.51182	18.21571	16.41618	21.21534	19.30686	23.01941	22.84124	26.89296	18.1088	21.95909	26.30902	17.46182	19.89018	21.3844	7.407832	23.93749	10.67475
LOC107826225	0.184994	0.289027	0.275677	0.167595	0.070155	0	0	0.022581	0.024812	0	0	0.022327	0	0	0	0.059082	0.102126	0.098515	0.107268	0.067068	0.024103	0	0.116555	0	0	0	0	0	0	0
LOC107785570	3.515844	4.566548	4.337311	17.48986	24.51582	14.05603	20.43033	21.08219	17.63078	25.41527	21.24302	24.84722	27.56126	26.43587	26.98862	5.694994	6.325407	5.132551	26.63977	22.70693	19.21985	25.52888	13.1662	12.54259	58.7977	56.68322	20.18003	30.11168	51.08364	53.37118
LOC107782150	16.34367	16.00854	17.72239	13.26101	10.58555	10.95951	13.3195	12.90274	16.3526	4.826324	8.122556	5.515916	5.603563	5.859129	6.485061	14.81608	13.55117	16.503	6.390132	5.184606	7.856225	16.65673	17.723	23.51031	1.171634	1.377355	2.812899	1.50619	2.332135	11.72034
LOC107775483	0.186239	0.509202	0.161894	0.590531	0.288394	0.250287	0.312414	0.278474	0.393411	0.090803	0.359125	0.15734	0.103662	0.101589	0.143363	0.451053	0.044981	0.173561	0.188983	0.078773	0.084927	0.155533	0.32855	0.145993	0	0	0	0	0.224155	0
Novel03477	1.25256	2.386885	1.385775	1.237915	1.309865	1.346655	2.005644	1.264806	0.320716	0.66622	0.718605	0.769598	0.507042	0.993804	0.818104	0	0	0.084894	0	0.096325	0	0	0.10044	0.119016	0	0	0	0	0	0
LOC107831114	0	0	0	0	0	0	0	0	0	0	0	0	0	0	0	0	0	0	0	0	0	0	0	0	0	0	0	0	0	0
LOC107801725	0	0	0	0	0	0	0	0	0	0	0	0	0	0	0	0	0	0	0	0	0	0	0	0	0	0	0	0	0	0
LOC107812673	10.00618	5.180503	5.7171	5.915717	2.410982	4.343551	1.812489	2.408324	5.336635	1.053604	1.679968	1.825638	2.14413	3.63875	1.591137	4.20091	4.810713	3.327244	1.668428	1.311407	1.670922	2.432386	3.895091	2.70054	0.415756	0.261462	0.16657	0	0	0
LOC107813627	0	0	0	0	0	0	0	0	0	0	0	0	0	0	0	0	0	0	0	0	0	0	0	0	0	0	0	0	0	0
LOC107787472	1.527602	4.02197	1.268893	0.184524	0.090115	0.036497	0.455561	0.81214	0.063741	0.331023	0	0.086037	0.340109	0.111103	0.139368	1.517827	2.459653	0.404939	0	0.258449	0.18576	2.069534	1.197729	0.106443	0	0	0	0	0	0
LOC107804732	5.801344	5.348333	5.80089	9.053211	8.931541	8.508487	7.453449	7.907999	7.115155	10.39849	9.382048	9.915594	10.34048	9.328844	6.229685	6.297332	5.798964	7.30025	9.493745	9.900177	8.71626	7.589337	6.951067	8.517027	11.15038	6.485713	12.32624	4.796279	10.0095	4.146888
Novel04857	0	0	0	0	0	0	0	0	0	0	0	0	0	0	0	0.601175	0.556691	0.902176	0.526251	0.731183	1.524054	0.529352	0.45745	0.722737	0.80139	0.400894	1.225914	0	1.10968	0
LOC107768315	14.51232	24.90998	29.86469	26.67817	37.21403	23.79044	32.1869	39.11517	39.16636	51.81952	46.28175	51.61528	39.32855	33.64927	45.4234	52.02359	19.62	38.9567	55.89909	64.78232	69.45735	49.53851	31.34897	42.71882	63.70979	79.12297	80.31124	90.77287	65.18251	62.78616
Novel04663	0.600915	0.182553	0.08706	0.816598	2.658646	3.660991	0.252007	0.342292	0	0.976613	0.210681	3.046018	0.891929	2.622275	1.027938	0.223901	0.774046	0.224004	3.455347	0.423611	5.754469	0.16728	0.441706	0.104679	1.772725	0.418065	2.604189	0	1.928679	0
LOC107825002	22.56851	19.91198	20.27923	43.46573	43.05612	41.04215	33.93157	33.60875	35.91495	59.89535	43.68726	59.09265	59.06715	55.61584	43.53462	16.93736	24.31585	19.83891	35.81322	38.55517	41.3482	25.79843	29.57802	28.52381	44.62148	33.90796	58.9764	74.40278	33.71321	24.74194
LOC107787212	11.63318	10.21365	13.13034	11.42193	11.15611	16.23957	12.6691	11.70908	14.92447	11.52199	11.10403	13.37847	11.59417	13.95371	10.68972	8.759835	10.29717	11.7154	7.663685	7.986046	10.49856	9.969496	12.46391	12.98657	5.929353	3.98312	9.430273	4.35569	11.43583	7.531906
LOC107790678	1.408058	3.978141	4.283983	0	0.124594	0	78.77263	59.31199	31.19795	8.512814	50.45263	1.189567	123.6734	54.99317	45.52356	1.364073	1.088242	0.734837	0	0.476448	0.128417	82.78379	10.1844	0	0.712085	0.195921	0	10.06961	0	0
Novel03501	2.78363	2.536935	3.511589	1.937502	2.403062	2.773761	2.534058	2.581444	2.135334	3.773665	3.308679	3.470173	3.249935	3.259008	1.707256	2.02377	2.328471	2.176549	2.376827	2.699358	3.188876	1.530888	1.736708	2.732043	3.476435	2.408952	3.651127	9.710677	4.576082	2.098976
107767200	0	0	0	0	0	0	0	0	0	0	0	0	0	0	0	0	0	0	0	0	0	0	0	0	0	0	0	0	0	0
LOC107806560	4.461368	3.062039	4.213323	0.449086	0.292423	1.42119	0	0	0.05171	0.053709	0.077242	0	0	0.120176	0.056531	1.641786	2.341265	3.079758	0.78246	0.698891	0.251163	0.137993	0.340081	0.057568	0	0	0	0	0	0
LOC107800057	0.021129	0.057771	0.036735	0.038284	0	0.022717	0.070889	0.036107	0	0	0.029632	0.089253	0.047043	0.023051	0.021687	0.031491	0.020413	0.126023	0.021441	0	0.038541	0	0.07455	0.044169	0.026714	0.0588	0	0	0	0
LOC107759450	0.79665	2.333728	1.063499	0.953708	0.730105	0.428248	0.978414	0.60775	0.74793	0.887811	1.675835	0.432664	0.728478	0.434553	1.051277	2.438266	1.017013	1.46363	0.63516	1.034935	0.778456	0.451456	0.777987	0.594756	0.359717	0.475063	0.100883	0	0.547908	0
LOC107829328	32.51548	21.37355	27.08321	21.28048	16.40763	27.97462	20.08192	17.74699	36.19981	15.50351	16.02082	23.70522	19.08629	20.92936	7.558345	15.16873	19.30002	17.65334	6.97444	6.165658	8.43832	12.89919	21.65218	29.09068	1.098162	1.261127	3.97251	0	0.545439	0
LOC107805464	0.076467	0.20907	0.066471	0.207825	0.270651	0.082211	0.128272	0.130671	0	0.14913	0.214474	0.193804	0	0	0.23545	0.113966	0	0.285046	0.465559	0.388114	0.069739	0.191578	0.134898	0.159846	0.290032	0.53199	0.180755	0	0.368138	0
LOC107828451	0.252037	1.168475	0.523911	1.231009	1.163575	0.377002	2.702165	2.733969	0.967068	3.216348	5.33258	2.120017	1.488244	1.267204	5.263644	1.984337	0.899849	0.898671	3.09123	3.84697	1.938832	3.157234	2.271458	0.847553	2.479934	5.794016	0.569871	0	1.529931	4.742895
LOC107763610	0.556608	0.74757	0.713039	0.1327	0.051845	0.062992	0.073713	0.150184	0.137518	0	0.102709	0.049499	0.065224	0	0.030068	0.2838	0.283016	0.240249	0.118907	0.024782	0.026718	0.244652	0.568486	0	0	0	0.069249	0	0	0
LOC107778759	0	0	0	0	0	0	0	0	0	0	0	0	0	0	0	0	0	0	0	0	0	0	0	0	0	0	0	0	0	0
LOC107824943	34.17808	37.07732	34.86866	10.64859	16.40898	16.25641	18.2337	13.45566	21.74905	4.618062	5.281254	2.747666	18.42033	18.30094	12.18118	16.88048	18.79707	20.6317	5.210918	7.915889	4.215108	5.718132	8.556002	5.784847	0	0.158786	0.741825	0	0.5494	0
LOC107832677	3.389279	5.741004	4.672301	6.451183	7.69455	4.527256	8.154914	8.658424	4.885501	5.30799	6.961653	3.702118	5.068767	3.884281	11.45499	6.17392	3.968883	5.462082	7.920595	9.441146	5.401583	13.58056	6.975658	6.01147	9.868666	14.57652	3.115665	7.344889	5.603885	31.75215
Novel03348	3.333258	12.1848	6.240834	0.995526	1.231653	0.984522	0.73734	1.157988	1.066062	0.428617	0.513688	0.247563	0.285433	0.559449	0.451143	10.26334	5.980385	7.509872	1.040726	2.014076	0.668129	0.85652	1.55085	0.535988	0.463104	0.101934	0.259757	2.619506	0.705384	0
LOC107799982	5.657404	5.689409	6.613676	6.141549	4.35871	7.078657	4.008799	3.917077	4.578811	4.042398	5.437492	5.685995	6.460778	5.42706	4.655295	5.233536	7.585776	5.854064	4.355046	5.404625	3.736297	2.973269	5.549452	5.352391	3.083051	1.017914	3.862086	0	5.869991	0
LOC107782816	0	0	0	0	0	0	0	0	0	0	0	0	0.058544	0	0	0	0	0	0	0	0	0	0.043919	0	0	0	0	0	0	3.251726
LOC107765447	0.043451	0.099	0.094427	0.610143	0.499824	0.350361	1.056874	1.132325	0.509917	0.614363	0.761691	0.367083	0.266034	0.142208	0.178386	0.291415	0.062965	0.145774	1.763629	1.194579	2.694685	1.451471	2.41456	0.908292	1.867784	0.39298	0.462197	0	0.627561	1.34331
LOC107803135	0	0	0	0	0	0	0	0	0	0	0	0	0	0	0	0	0	0	0	0	0	0	0	0	0	0	0	0	0	0
LOC107811487	1.318283	5.566728	1.986322	0.676768	0.544368	0.472438	1.068844	0.863555	0.783852	0.856995	2.773142	1.447838	0.63593	1.246424	0.902033	7.760847	3.396192	3.079552	1.293112	1.821455	1.883597	2.27526	1.744219	0.780792	1.833381	1.834294	5.401427	0	1.904005	0
LOC107821187	13.31943	11.41158	8.022413	26.30275	15.97939	14.26638	65.60686	59.11881	52.64219	14.93391	15.39108	13.90773	13.77224	18.55811	17.56198	12.04461	13.39781	10.07896	11.23772	10.67652	13.83091	39.53598	34.3658	20.54328	6.433183	3.956485	6.603591	3.567518	6.724662	6.16899
LOC107786674	0.197766	0.411976	0.589419	0.307142	0.274994	0.334121	0.33175	0.337953	0.477441	0.055099	0.396211	0.358025	0.157254	0.123287	0.086992	0.652663	0.545882	0.400202	0.315353	0.525789	0.283432	0.707827	0.722691	0.915408	0.178597	0.039311	0.100176	0	0	0
LOC107778944	2.713977	1.051219	1.415522	3.196344	2.761732	3.06375	0.512174	0.463779	0.3822	0.396971	0.285456	0.343927	0	0.07402	0	0.202246	1.179869	0.607016	1.170432	0.746147	1.732635	0.623291	0.77802	0.780078	0.257347	0.094408	0.561348	0	0.653303	0
LOC107820439	7.973947	14.01234	9.671011	10.33853	10.56268	7.751606	11.61405	10.6889	10.04149	7.868727	10.71394	4.316273	6.697397	5.938366	15.04528	14.94444	11.02477	13.63415	10.07792	11.55202	5.182111	16.62819	11.45584	7.935116	5.734981	9.89928	3.49894	0	3.907905	2.952342
LOC107830203	9.451782	12.73502	10.93919	18.76668	20.33044	18.39085	17.91139	15.7328	14.92171	18.59273	19.14745	12.94419	18.5876	15.30371	22.74295	12.85892	12.35477	12.35712	23.06543	23.97515	13.15385	21.73537	15.24463	15.03202	20.23222	28.61486	11.47709	13.6356	19.67042	53.89445
LOC107791865	4.685055	6.116272	4.494557	7.532894	7.450957	7.237862	8.23083	7.122523	6.855376	9.137041	10.71378	6.721579	9.984569	9.600702	7.2779	8.22502	7.747476	7.772506	9.95785	10.55077	7.410121	8.21296	8.860737	8.205479	14.2747	10.01328	9.603076	0	15.84983	9.134181
LOC107787089	0.209101	0.381139	0.393828	0.473587	0.215864	0.149873	7.892185	3.722113	1.276035	1.189415	5.962613	0.235539	3.375223	3.459801	8.513077	0.571348	0.303013	0.363752	0.388999	0.383251	0.413191	10.88499	1.598487	0.072851	0.528734	0.290949	0.04119	0	0.671124	0
LOC107831047	0.068114	0.186233	0.296051	0.28797	0.241087	0.146462	0.152347	0.213395	0.042632	0.287819	0.159206	0.191816	0.278027	0.074309	0.209731	0.203035	0.131608	0.118491	0	0.076826	0.14495	0.132729	0.100135	0.142386	0.086117	0.031592	0.026835	0	0.327926	0
LOC107784080	0	0	0	0	0	0	0	0	0	0	0	0	0	0	0	0	0	0	0	0	0	0	0	0	0	0	0	0	0	0
LOC107762405	10.15362	6.2059	9.631899	491.9094	364.5175	392.0366	208.0742	223.9112	319.155	391.9561	142.1119	537.5242	134.7407	257.7622	64.55474	16.66428	21.40907	14.80947	316.455	281.8777	403.3361	85.56942	194.2634	400.7082	158.7846	100.7462	557.0827	80.68078	115.8064	42.35249
LOC107810925	0.068203	0.80806	0.266793	4.386969	4.677151	0.80659	13.5575	12.1502	3.137559	5.353762	12.76897	1.152393	3.036973	5.431632	19.63542	3.532325	0.494172	1.32205	10.24271	12.83711	5.660392	14.46732	8.001199	1.746496	17.33205	26.71414	2.337697	9.754931	13.46246	71.69041
LOC107783084	0.117707	0	0.204641	0	0	0	0	0	0	0	0	0	0	0.128413	0	0	0.341146	0.087756	0	0.099572	0	0.0983	0	0	0	0	0	0	0	0
LOC107803895	0.115501	0	0.100403	0	0.051101	0.124178	0.048438	0.098687	0.162656	0.056314	0.080989	0.048789	0.064289	0	0	0.043036	0.055792	0.129166	0	0	0.158008	0.048229	0.305639	0.120722	0	0	0.068257	0	0	0
LOC107762905	11.01624	10.29574	11.711	4.990077	4.439327	5.280677	4.943584	4.676311	6.884041	1.950022	2.706068	2.134049	2.187109	1.684076	1.080264	8.18317	8.575099	8.893137	2.456426	2.196193	3.583642	5.50825	8.633941	10.45076	2.572665	0.536978	2.405025	2.508971	4.222619	0
LOC107780536	141.8981	127.3383	136.8142	111.1943	106.5148	134.0984	111.3982	111.7273	133.045	96.14829	88.15766	112.7319	100.6226	118.1559	93.85854	95.12667	144.2974	114.3713	87.80706	83.25167	101.1593	91.79944	116.4389	118.7926	58.83077	63.53425	88.3044	151.2594	84.75709	84.62213
LOC107769079	26.79195	23.91457	27.08406	18.13593	14.42859	22.7632	19.44169	17.10452	25.71871	7.485405	9.041457	8.858853	7.372514	9.086074	5.973461	16.97432	22.8781	19.48826	8.196714	8.658212	10.02098	18.10084	25.88928	36.49821	4.059705	0.698109	5.277654	3.588021	5.555576	9.306665
LOC107781162	0.519216	0.306189	0.371695	0.553393	0.37836	0.886586	0.563574	0.574113	1.003601	0.416954	0.342659	0.593465	0.645999	0.19992	0.031348	0.29588	0.324569	0.273245	0.371905	0.206692	0.278549	0.357091	0.646564	0.60653	0.154458	0.084994	0.288787	0	0.147041	0
LOC107817225	34.43852	35.16995	33.60815	39.0158	39.48135	35.97773	35.09203	31.46091	32.77299	29.21696	31.05868	33.18128	34.84126	36.54679	34.71688	33.51874	33.20076	36.22543	32.34504	32.82764	34.07527	31.86568	32.54229	31.80702	21.2227	25.41599	17.83447	25.81594	24.15734	26.78472
LOC107760923	0	0	0	0	0	0	0	0	0	0	0	0	0	0	0	0	0	0	0	0	0	0	0	0	0	0	0	0	0	0
107780468	0	0	0	0	0	0	0	0	0	0	0	0	0	0	0	0	0	0	0	0	0	0	1.2834	0	0	0	0	0	0	0
LOC107808771	6.311525	5.053314	4.255864	5.611045	4.80193	5.580731	5.54112	6.199129	6.866979	3.50866	4.59109	4.68432	5.384471	3.989766	3.329801	4.835226	6.211435	4.749487	3.471598	3.343185	4.249917	6.009834	7.180105	7.151676	2.162719	2.708476	2.30066	0	3.975729	3.647207
LOC107788575	0	0	0	0	0	0	0	0	0	0	0	0	0	0	0	0	0	0	0	0	0	0	0	0	0	0	0	0	0	0
LOC107819729	8.854117	11.23126	9.158328	13.15044	12.58073	11.23547	18.51227	17.22174	14.18743	8.965954	12.17824	6.113628	12.48651	12.44577	14.74554	11.46737	9.150121	10.33012	14.79426	15.43007	9.278604	17.6859	15.24436	10.81154	11.59794	12.64562	5.811012	19.78733	16.08754	40.79655
LOC107789569	7.029319	5.441959	6.110438	8.52518	9.363446	10.64534	7.163705	6.619561	8.337995	7.333549	5.617957	9.578329	8.077559	8.163392	3.91766	4.421539	8.470386	5.804167	7.516354	7.480854	7.824003	4.450117	6.467004	8.374048	4.156914	2.418904	4.556049	8.107995	4.184719	0
LOC107775558	1.3578	0.856708	0.726343	2.696754	4.574832	3.537211	2.058683	1.874075	1.47088	3.462839	2.233751	4.014876	1.918471	1.994049	1.018407	0.622667	0.605423	0.506149	3.55049	1.678731	4.762834	0.785032	1.427991	1.965008	2.70706	3.415237	2.901004	0	3.519888	0
Novel01559	0	0	0	1.284695	1.96062	0.524079	8.325743	11.24547	4.867717	2.808796	4.567785	3.931004	1.28262	3.819252	7.140864	0	0	0	4.361829	2.39917	3.394909	10.47332	6.33231	2.732734	6.555162	16.95662	3.142572	0	2.773496	0
LOC107761477	34.38753	18.43529	32.11345	9.645002	10.26839	13.62082	11.96554	11.09771	18.59109	14.43026	12.69082	24.10468	15.88115	26.59739	10.48998	14.75655	22.52462	18.01759	10.69509	5.583739	17.08889	6.490428	9.296827	12.79663	9.82591	3.55525	10.31811	15.22721	41.51659	0
LOC107760415	10.13802	8.38768	8.844015	7.100098	5.853279	5.681711	10.07116	10.50522	10.86809	3.084946	4.285438	4.039464	4.482297	3.569033	3.874345	7.903137	8.891169	9.30049	3.4656	3.162804	4.623022	11.2887	9.671747	11.49806	0.318166	0.250112	1.104753	0	0.519234	0
LOC107785098	16.35865	14.6458	16.42297	22.3464	20.46398	20.38087	17.43337	17.89639	21.0498	17.48437	18.9379	17.83082	20.56736	20.40098	14.5514	16.13447	18.00397	17.38538	16.69727	15.62913	18.48831	15.2152	20.05971	22.1269	14.47762	10.93462	18.12146	11.46936	18.22206	13.88306
LOC107775846	4.927185	3.914813	5.117752	4.07463	3.868011	4.020535	5.213487	4.382635	5.85942	2.463911	3.082874	2.775082	2.869082	2.398218	2.826804	5.309906	5.101823	5.933995	2.487063	2.479461	1.866604	5.233208	6.106831	8.00214	1.34173	0.949267	1.6724	1.806992	1.581415	1.562335
LOC107767465	0	0	0	0	0	0	0	0	0	0	0	0	0	0	0	0	0	0	0	0	0	0	0	0	0	0	0	0	0	0
LOC107826385	1.598664	3.513906	1.675796	0.63896	0.499272	0.758276	0.197187	0.482097	0.485584	0.917	0.527522	0.556128	0.3664	0.410368	0.096519	3.153511	1.726143	2.76935	0.715683	0.755731	0.686122	0.667543	0.580641	0.442303	0.59447	0.196273	2.334079	0	1.358213	0
LOC107805559	32.0604	31.6277	28.84862	31.32161	35.19308	33.77016	22.74786	23.02516	23.30694	82.46656	63.88792	67.787	42.3458	48.4942	37.93074	26.21602	27.62461	24.16079	74.42949	71.06407	74.60055	23.62667	20.63631	23.95478	112.7286	117.2512	122.9468	130.1303	64.45163	69.64988
LOC107808596	10.27577	10.30769	10.20859	13.75242	13.52094	15.35202	12.62029	12.68525	11.83993	17.53531	17.0309	17.13703	20.05563	18.08945	15.71743	8.129899	10.8942	8.506724	11.51049	11.90999	12.29272	9.751782	9.181626	10.28692	9.659416	5.988444	13.91959	21.47328	12.68905	8.251515
LOC107818308	2.011338	1.78145	2.339426	8.263925	6.016103	5.604054	4.918423	5.397672	6.117133	4.69609	5.661269	7.060197	2.869767	3.523195	2.762201	1.921063	1.861017	1.985303	5.404249	5.871212	6.019846	5.65427	6.671738	6.040266	3.008551	1.576693	6.12725	0	2.182151	1.751606
LOC107779361	1.408619	1.14114	0.498863	41.82895	30.88398	37.74876	0.787646	0.668646	0.342864	2.69629	1.280384	0.440756	0.406543	0.341496	0.321282	0.583169	0.856828	0.972394	33.77547	27.58321	28.02515	1.219944	3.31332	6.052741	18.07307	5.371826	33.05084	18.65485	1.004681	0
LOC107795690	1.186597	0.463473	1.105161	0.537498	0.299994	0.364496	0.355446	0.362093	0.477441	0.991784	0.178295	0.644445	0.849173	0.277397	0.173984	0.063161	0.081882	0.379139	0.172011	0	0.0773	0.070783	0.074761	0.354352	0.321475	0	0.901582	0	0.40805	0
LOC107783858	4.264866	5.748231	3.655141	3.319562	2.923392	2.970727	4.181712	3.644017	4.229624	1.932955	2.990527	3.856815	5.683876	4.3251	3.390898	5.461103	5.10675	6.135359	2.742903	3.201272	3.287011	2.658734	4.238754	5.022689	1.442953	1.588039	1.49092	8.591499	5.205444	3.714128
LOC107770289	0	0	0	0	0	0	0	0	0	0	0	0	0	0	0	0	0	0	0	0	0	0	0	0	0	0	0	0	0	0
LOC107826977	0	0	0	0	0	0.067282	0	0	0	0	0.043881	0	0	0	0	0	0.060458	0	0	0.105877	0	0.104525	0.0552	0	0	0	0	0	0	0
LOC107774317	0.520904	0.290119	0.452811	0.157304	0.076822	0.124452	0.218452	0.222537	0.326032	0.028219	0.081168	0.146691	0.161077	0.126285	0.029702	0.194089	0.391406	0.151027	0	0.122402	0.158358	0.193342	0.076579	0.21173	0	0	0.034204	0	0	1.789348
LOC107762338	3.264461	2.434217	3.040421	9.544454	7.315311	7.566355	6.169489	6.411639	9.871108	4.609528	4.161895	4.727841	5.828628	6.243954	4.112043	2.179934	2.15028	2.29156	8.195334	7.065161	6.863126	6.709375	8.096174	6.181505	2.144037	3.568918	3.30714	1.515942	4.592419	1.310692
107826757	0	0	0	0	0	0	0	0	0	0	0	0	0	0	0	0	0	0	0	0	0	0	0	0	0	0	0	0	0	0
LOC107816518	0	0	0	0	0	0	0	0.116246	0	0	0.095399	0	0.151454	0	0	0	0	0	0	0	0	0	0	0	0	0	0	0	0	0
LOC107812644	0.705235	0.642735	0.613046	0.609866	0.453846	0.4825	0.457077	0.219117	0.571819	0.281329	0.382123	0.324983	0.392539	0.41966	0.42772	0.69276	0.557441	0.454085	0.422869	0.379642	0.087707	0.803128	0.282757	0.368556	0.243172	0.089208	0.037888	0	0	0
LOC107776988	3.703058	3.509876	3.31556	4.545731	4.358024	3.384055	3.773686	3.622773	3.476594	4.694234	4.258402	3.300511	4.534526	4.484239	2.888545	4.235869	3.362818	4.500083	3.701244	4.620486	2.75246	3.448161	3.805289	4.025253	4.002932	3.117284	3.195004	6.620526	6.863719	6.86897
LOC107831958	0	0	0	0	0	0	0	0	0	0	0.087311	0	0	0	0	0	0	0	0	0	0	0	0	0	0	0	0	0	0	0
LOC107777091	5.008877	6.393557	5.36842	5.285493	5.98093	5.997105	6.755276	5.07001	5.343812	5.369941	6.296067	4.580268	6.447194	5.74387	7.988957	7.231953	6.309941	6.184985	5.37145	6.054813	4.165869	7.938277	6.112626	4.952691	2.986464	5.128117	2.051849	3.052888	2.8773	11.43802
LOC107767944	0	0.065754	0	0	0.063842	0	0	0	0.135472	0	0	0	0	0	0.074051	0	0	0	0	0	0	0	0	0	0	0	0	0	0	0
LOC107800965	0	0	0	0	0	0	0	0	0	0	0	0.021529	0	0	0	0	0.059324	0.045781	0	0	0.028002	0	0	0.064182	0	0	0	0	0	0
LOC107784212	0.212926	0.097028	0.185092	0.1929	0.157009	0.228921	0.208355	0.030322	0.099952	0	0.074652	0.089943	0.316043	0.309722	0.036424	0.185118	0.17142	0.052915	0	0	0.161827	1.126193	0.406932	0.778925	0	0	0	0	0	0
LOC107772614	2.46856	2.249788	1.944692	4.542668	7.713466	6.469135	6.987896	6.920828	4.780036	8.951618	8.222041	6.386869	8.759364	7.069329	11.40148	3.334228	3.949886	2.818138	9.080351	8.744472	4.150961	9.470287	4.627046	4.353984	18.43347	16.3154	3.647049	0	24.51184	0
Novel02417	6.3647	4.859995	6.355127	6.155697	6.088546	5.640712	9.08962	7.128415	5.975461	6.625737	6.634088	3.923812	4.97884	6.662079	7.06222	7.499039	5.484096	6.797113	4.800205	4.94755	2.588588	8.547621	7.814201	4.045368	4.023458	2.63287	2.236438	0	3.726721	21.27238
LOC107780904	0	0	0	0	0	0.262539	0	0	0	0	0	0	0	0	0	0	0	0	0	0	0	0	0	0	0	0	0	0	0	0
LOC107818253	0.945673	3.303812	1.18741	1.856252	1.069236	0.169452	0.440654	0.538672	0.345271	0.051231	0.294714	0.08877	0	0.229263	0.215692	3.327828	1.370399	1.410078	1.759273	2.22215	0.814554	0.482629	1.065853	0.604033	0.531386	0.36551	0	7.51433	0.505867	12.99386
LOC107783188	2.497773	4.48587	2.809867	8.785204	8.418237	10.42571	26.43384	19.23887	23.86402	12.60804	15.89174	10.02339	41.01319	31.29682	26.46587	6.678937	6.990772	7.120159	4.547312	5.468796	4.18752	19.6632	12.7656	15.35689	7.058934	3.168809	7.857972	5.253708	11.49464	11.35596
LOC107804009	0.249725	0.227593	0.10854	0.113119	0.497189	0.268485	0.314182	0.586772	0.175839	0	0.175107	0.052744	0	0.272437	0.320388	0.325667	0.301569	0.232725	0.063351	0.316875	0.170815	0.729931	0.550684	0	0.157864	0	0.51652	0	0	0
LOC107826447	5.002562	3.821697	4.860223	3.665643	2.083104	2.451902	2.406451	2.388593	4.420353	1.506481	2.218167	2.423909	1.637918	2.006452	2.114203	2.741114	5.188255	4.662027	1.86627	2.115911	2.817965	2.826138	3.17966	3.229476	1.767212	0.614179	1.304256	5.261086	0.354178	0
LOC107761572	2.692683	3.112766	2.82115	3.710507	3.43605	3.108276	3.82751	3.099892	3.126748	1.837994	2.842103	2.119205	2.28759	2.210919	3.60735	4.203278	2.92995	3.307107	3.019964	2.697423	2.145569	4.426447	3.200167	3.347644	1.290077	1.774739	0.88776	0	1.978644	2.628831
LOC107763218	0	0	0	0	0	0	0	0	0	0	0	0	0	0	0	0	0	0	0	0	0	0	0	0	0	0	0	0	0	0
LOC107762336	0.030739	0	0	0	0.0272	0.033049	0	0	0.057719	0	0	0.051939	0	0.033535	0	0	0	0	0	0.026003	0.05607	0	0	0.064257	0	0	0	0	0.14799	0
LOC107787029	0	0	0	0	0	0	0	0	0	0	0	0	0	0	0	0	0	0	0	0	0	0	0	0	0	0	0	0	0	0
LOC107760857	0	0	0	0	0	0	0	0	0	0	0	0	0	0	0	0	0	0	0	0	0	0	0	0	0	0	0	0	0	0
LOC107806541	10.10954	12.47255	11.49348	17.73754	19.45371	14.00154	18.32863	20.067	17.88422	16.81039	16.80885	15.14224	16.61082	18.71078	20.66168	14.32707	10.91819	12.26011	26.20611	25.24512	26.41207	22.17601	21.10553	16.588	36.80968	44.01213	28.8281	31.57095	31.66797	35.48534
LOC107796243	8.146422	8.782977	9.281301	12.37369	12.48449	10.02557	16.28231	15.37239	13.57152	14.16361	19.25159	13.88179	17.8287	15.08958	18.50152	15.34472	9.678584	11.16491	16.28662	15.92333	14.51158	15.71994	13.57635	11.34077	9.203857	9.35753	10.5298	9.91633	8.678419	6.430284
LOC107821756	0	0	0	0	0	0	0	0	0	0	0	0	0	0	0	0	0	0	0	0	0	0	0	0	0	0	0	0	0	0
LOC107777927	0.144297	0.131509	0.313586	0.326814	0.319208	0.271489	0.030257	0.061646	0.16934	0	0.151772	0.060953	0	0.118066	0	0.134413	0.104552	0.026895	0.109817	0.24413	0	0.060253	0.127279	0.414752	0	0	0.085274	0	0	0
LOC107826415	2.257748	2.527981	3.00934	0.545441	0.703988	1.595119	0.162315	0.110233	0.100937	0.440318	0.226162	0.054497	0.14362	0.070374	0.066208	1.890783	1.433343	2.484794	0.589116	1.200508	0.431431	0.053872	0.322431	0.15732	0.108742	0.029919	0.050828	0	0.41408	0
LOC107783667	0	0	0	0	0	0	0	0	0	0	0	0	0	0	0	0	0	0	0	0	0	0	0	0	0	0	0	0	0	0
Novel00894	7.181096	6.764673	7.711169	6.505709	4.431995	5.644441	3.897297	3.970174	4.362425	1.118042	2.200338	2.192201	3.22451	2.238349	1.36261	5.261423	5.421777	7.018443	1.775794	3.062881	3.577338	3.074155	6.866488	7.379534	0.076295	0.167932	0.64191	0	0	0
LOC107769942	2.615422	2.643667	2.769574	5.105065	5.806782	6.032799	4.187909	3.067616	3.638565	4.590659	4.05133	2.912629	3.917323	3.216443	2.879626	2.888086	3.123941	2.800777	5.404405	5.209353	3.296063	3.514587	3.334627	4.945399	3.426931	1.786501	4.074787	5.101081	2.060439	4.410422
LOC107820599	0.121531	0.05538	0.316932	0.05505	0.107538	0.195991	0.101933	0.05192	0	0.237016	0.170435	0.051336	0.541159	0.132584	0.124736	0.543389	0.058704	0.815457	0	0.102807	0	0	0.267995	0.127024	0.38413	0.422753	0.359099	0	2.632916	0
LOC107770877	11.78842	10.82487	8.982625	15.8178	23.6212	15.29179	15.71532	16.00919	15.50005	17.54682	15.28281	19.26612	25.05606	19.98735	16.70131	9.824748	10.29851	8.877316	23.95427	20.29604	25.21259	17.30902	13.54118	11.94882	21.69923	18.22065	20.21507	23.35875	27.30464	31.21215
Novel01427	2.857078	2.291409	2.814743	2.726414	2.662964	2.129718	3.22709	1.855285	3.111527	2.005928	2.37735	2.896487	3.095725	1.537686	1.329367	2.441365	2.208142	1.902865	1.430255	1.385684	1.215995	1.749748	1.814495	2.468606	1.589375	0.742078	0.720392	0	2.384198	2.355432
LOC107799104	0	0	0	0.816754	2.552788	0.969271	6.578632	6.778679	3.554922	0.703296	1.137895	1.21864	5.720591	6.786434	5.181798	0	0.087097	0.067214	0.640378	0.68638	1.808893	5.044455	2.1471	1.69613	0.113983	0	0	0	2.170179	5.574388
LOC107784090	0.114976	0.134725	0.228448	0.282726	0.261613	0.317863	0.2342	0.210511	0.277572	0.256265	0.161242	0.208146	0.182847	0.17919	0.10115	0.09792	0.158681	0.208176	0.316675	0.277891	0.11984	0.23319	0.420154	0.171675	0.145365	0.114272	0.155306	0	0.237229	0
LOC107789557	4.659814	4.482782	5.475916	5.003325	4.963244	7.42203	3.836009	5.529822	4.779916	5.048786	3.388482	6.852844	4.80312	4.707063	2.391353	1.864861	4.33504	2.702069	3.414997	3.065908	4.171128	3.387075	3.349104	5.411596	1.854707	2.281334	3.773675	18.51348	1.661778	0
LOC107814446	0.221012	0.805699	0.336211	0.650732	0.293348	0.534633	0.695145	0.283258	0.363117	0.37715	0.34869	0	0.553575	0.241113	0.396971	0.617618	0.266895	0.082387	0.336401	0.09348	0.201566	1.061292	0.633577	0.288752	0.069856	0.153761	0	0	0	0
LOC107792326	0	0	0	0	0	0	0	0	0	0	0	0	0	0	0	0.111524	0.048194	0.185959	0	0	0	0.124982	0.044002	0	0	0	0	0	0	0
LOC107761337	0.615428	0.716689	0.445815	0	0	0	0	0	0.032099	0.06668	0.023974	0.144425	0.038061	0	0.035092	0.662448	0.39637	0.407847	0.069388	0.028923	0.093547	0	0.030158	0	0	0	0.04041	0	0.164605	0
LOC107784357	1.890966	3.351021	2.502193	2.760026	3.458053	1.874896	4.12369	3.339098	2.781338	1.536609	2.003653	1.508788	2.292165	2.406776	3.773847	2.755671	2.374882	2.913577	3.645761	3.270349	2.203647	4.667399	3.428579	3.008577	3.240803	4.677585	1.936974	0	3.641514	10.393
LOC107790133	5.308012	4.266493	4.165545	8.348594	9.654695	6.459738	7.574683	8.787163	7.371273	8.339083	9.201566	9.840671	11.32549	7.519972	9.760997	5.274049	6.267539	5.139057	11.22126	10.7578	9.581139	6.864776	7.738335	7.705463	9.367356	9.437299	11.37098	15.81658	11.89002	18.23347
LOC107793058	2.821805	1.339441	1.839702	2.077085	1.924708	3.223397	1.429923	0.954365	2.152504	1.834411	2.061095	1.986616	2.421398	2.116435	1.327436	1.139024	1.363051	1.665489	1.073765	1.442178	1.501228	0.785521	2.177892	3.379464	0.81758	0.408993	1.111716	0	2.830245	0
LOC107769082	0	0	0	0	0	0	0	0	0	0	0	0	0	0	0	0	0	0	0	0	0	0	0	0	0	0	0	0	0	0
LOC107788364	0	0	0	0.247475	0.120858	0.146844	0	0	0.256461	0.066593	0.095772	0.230779	0.076023	0	0	0	0	0	0	0	0	0	0.120476	0.214136	0	0	0	0	0	0
LOC107772967	6.972444	9.978176	12.82327	27.5637	34.46861	19.14326	33.44527	25.84842	36.78765	12.86755	22.10188	16.79527	15.58779	16.5962	12.95236	17.30526	11.07816	16.02437	18.94504	20.22939	32.63579	25.89011	25.05807	28.48788	9.179556	12.10701	7.083059	24.72537	9.709701	28.50358
LOC107798805	0	0	0	0	0	0	0	0	0	0	0	0	0	0	0	0	0	0	0	0	0	0	0	0	0	0	0	0	0	0
LOC107821446	3.065482	1.822049	2.664759	3.260158	5.189208	4.872005	5.030515	4.099666	2.752879	4.158932	3.925207	2.927605	2.077197	2.617271	2.462343	0.695252	1.673899	1.09304	3.11064	2.48044	3.768214	2.114836	2.586395	1.81098	1.685088	1.298164	1.890341	9.531536	5.13333	16.48203
LOC107803092	12.94442	9.739587	4.448589	8.317983	6.259787	2.589179	35.47477	40.63932	19.92493	7.338632	19.41978	6.866671	5.8645	9.195545	18.07487	16.26394	9.160934	10.77278	13.13511	11.71405	14.41373	32.68208	30.93437	15.10271	3.805974	11.72824	7.115938	0	7.971043	0
LOC107796206	0	0	0	0	0	0	0	0	0	0	0	0	0	0	0	0	0	0	0	0	0	0	0	0	0	0	0	0	0	0
LOC107773571	0	0	0	0	0.101978	0.061952	0.048331	0	0	0	0.121217	0	0.064147	0.062864	0.059143	0.085882	0	0.214804	0.877085	0.389964	0.57809	0.433106	0.152483	0.240912	0.218561	0.160358	0.068106	0	0.83226	0
LOC107780017	0.567104	0.939718	0.941127	1.120947	0.958002	1.385695	1.167515	1.321497	1.258452	1.055723	0.939908	1.39376	1.090441	1.349852	0.846632	0.845213	0.747095	0.960909	1.360173	0.348895	1.081435	0.645822	1.364244	1.239358	0.325905	0.071735	0.426536	0	1.241018	3.187716
LOC107790017	2.149562	2.708113	2.6105	4.811209	4.209737	4.31621	6.973097	5.415399	5.074936	4.39255	5.995847	3.231429	7.460287	5.103968	5.158735	3.674846	3.023369	3.700096	2.66238	3.690244	1.946021	5.873853	4.419483	3.601362	1.578664	2.155249	1.307666	1.130324	2.81547	0.977284
LOC107826113	0	0	0	0	0	0	0.039156	0	0	0	0	0	0.103939	0	0	0	0	0	0.047372	0.039492	0.085154	0.116962	0	0	0	0	0	0	0	0
LOC107763087	0	0	0	0	0	0	0	0	0	0	0	0	0	0	0	0	0	0	0	0	0	0	0	0	0	0	0	0	0	0

LOC107822368	7.624902	7.467108	6.586997	8.881425	9.093803	12.5766	8.897856	8.659587	7.91447	9.143963	9.099159	10.16273	11.86232	10.54008	9.96477	7.870218	6.359722	7.097033	8.746427	9.775371	7.299581	6.407306	6.642119	6.930039	5.089543	5.733081	4.701924	6.773768	9.348253	5.856636
LOC107816022	18.78264	16.87696	17.54287	11.77776	8.560865	13.89582	10.61877	9.784034	13.05694	6.707065	7.817441	6.928324	7.4465	7.338807	7.292277	15.88375	15.29781	17.10257	6.595984	6.425862	6.341923	12.3404	13.83406	17.1036	1.767883	1.577546	5.583393	5.40533	3.274998	0
LOC107831223	16.81711	14.34457	14.66806	25.02416	30.26365	24.91016	20.40858	21.49291	18.66414	29.36852	24.7377	30.47558	35.4216	34.74414	31.40676	13.3352	12.49328	13.59502	28.54692	25.98125	27.70059	21.2916	17.83558	16.53979	47.83039	36.57942	40.08812	52.7305	56.93389	63.1261
LOC107781803	0.070956	0.245737	0.111025	0.244274	0.288818	0.122058	0.880806	0.67902	0.373053	0.083029	0.378134	0.107902	0.252766	0.356084	0.524357	0.105753	0.05484	0.148122	0.129602	0.072029	0.116484	0.118515	0.137693	0.059331	0.035884	0	0	0	0	0
LOC107819064	2.420392	2.177608	2.643481	4.694739	5.6838	4.93754	5.127271	4.666366	4.311677	4.236234	5.548474	5.479054	4.76704	5.788851	5.127693	3.329869	2.817954	2.8687	4.975068	5.197462	4.697881	4.509126	4.297262	4.119022	4.629399	3.151914	6.821689	4.438263	5.975707	0
LOC107806483	3.657649	3.715194	3.90765	1.5177	0.914134	2.431496	0.538626	0.393631	0.563589	0.122519	0.352408	0.094354	0.481771	0.548288	0.358217	3.245853	2.872733	4.142439	0.694141	0.755815	0.432895	1.165877	1.120581	0.569069	0.141202	0.03885	0.115501	0	0.067211	0
LOC107812198	32.13281	25.0268	31.35266	18.19419	12.6934	22.73737	17.05074	15.26842	20.93269	15.34704	15.92153	19.04424	14.41778	18.42193	13.96612	20.70789	37.69507	24.75085	10.31419	9.70791	15.02663	13.96513	18.7268	21.07643	4.456384	3.649834	9.494599	0	7.892796	5.068419
LOC107770668	0	0	0	0	0	0	0	0	0	0	0	0	0	0	0	0	0	0	0	0	0	0	0	0	0	0	0	0	0	0
LOC107820451	0	0	0	0	0	0	0	0	0	0	0	0	0	0	0	0	0	0	0	0	0	0	0	0	0	0	0	0	0	0
LOC107818224	0.027028	0	0	0.048972	0.023916	0	0	0	0	0.026355	0.018952	0	0.030088	0	0	0.020141	0.026111	0	0	0.022864	0.073949	0.022572	0	0	0	0	0	0	0	0
LOC107817914	0.574273	0.826388	1.077229	0.219058	0.58839	0.682405	0.507019	0.723099	0.567529	0.206312	0.381487	0.153209	0.302821	0.164869	0.434308	1.013564	0.729995	0.74362	0.674743	0.357955	0.303222	1.363047	0.773151	0.473866	0.076427	0.210279	0.178617	0	0.145513	0
LOC107815725	0	0	0	0	0	0	0	0	0	0	0	0	0	0	0	0	0	0	0	0	0	0	0	0	0	0	0	0	0	0
LOC107769267	5.183883	5.094212	5.799335	6.697383	5.065689	6.203335	5.141919	5.353616	5.586307	5.362633	4.804445	6.283614	6.021674	4.425936	4.117679	3.795853	4.354841	3.898412	4.29968	4.156415	5.591124	4.705648	4.65205	4.852817	3.077533	2.94792	4.58188	6.447321	5.20843	2.787194
LOC107760914	2.30374	2.510018	1.882135	4.095681	3.264666	4.152833	4.125998	3.670358	3.382506	4.307078	5.757081	3.321818	4.184274	3.665968	5.475673	3.936906	2.392945	3.254278	3.796526	3.795042	1.97467	4.527705	3.407122	2.896683	1.751956	2.145024	1.944882	2.477443	1.917996	0
LOC107794000	1.636868	0.942191	0.89867	1.014629	1.295944	1.111473	1.300653	1.398584	1.213234	1.26012	1.510227	1.674002	1.246757	1.221824	2.033728	2.246992	0.998748	1.991104	2.01066	1.821944	2.435704	1.654778	1.89977	1.890943	1.307051	1.678218	1.221882	6.161009	1.244284	0
LOC107766239	0	0	0	0	0	0	0	0	0	0	0	0	0	0	0	0	0	0	0	0	0	0	0	0	0	0	0	0	0	0
LOC107805465	8.906	10.41391	9.077309	8.655572	6.648605	7.226444	9.503415	9.229886	9.037464	6.601137	7.238016	5.921883	7.348862	6.86144	8.820574	8.962311	7.815443	9.753885	6.16283	6.7622	6.217703	8.399914	9.062618	9.258368	4.006215	6.279529	4.482846	0	8.552217	17.81147
LOC107770233	0	0	0	0	0	0	0	0	0	0	0	0	0	0	0	0	0	0	0	0	0	0	0	0	0	0	0	0	0	0
LOC107774263	0	0	0	0	0	0	0	0	0	0	0	0	0	0	0.060136	0	0	0	0	0	0	0	0	0	0	0	0	0	0	0
LOC107761758	11.26218	12.78169	12.00657	7.796605	8.60231	10.90882	9.356961	7.353205	8.977441	6.578868	6.369788	6.59739	5.026714	7.418255	5.997694	8.828054	14.26745	11.84214	5.714041	6.47119	7.897271	8.47368	10.73058	10.32759	2.149256	0.813092	3.013809	7.598161	1.534532	0
LOC107773229	0.887166	1.751842	0.739062	1.640944	1.013988	0.278195	0.868122	1.452869	3.08871	1.658106	6.505934	3.778742	5.802183	8.146109	2.314347	3.002574	2.428385	3.362218	0.487627	1.407153	0.910249	1.018731	1.825925	2.665889	1.308589	1.543034	1.966048	0	0.177964	0
LOC107816770	0	0.313548	0.037383	0	0.11416	0	0.03607	0.036744	0	0	0.211085	0	0.047873	0	0.044139	0.032047	0	0	0.043638	0	0	0	0	0	0	0	0	0	0	0
Novel02846	1.244734	1.900923	1.315972	9.874698	10.15095	3.689206	3.132037	4.599069	3.663754	7.774708	4.694294	5.428458	2.958562	3.963728	2.140778	2.406687	1.040015	1.655355	13.85964	13.66002	9.910151	5.422309	3.679576	4.28977	7.826079	6.834208	9.383715	2.40584	2.753354	0
LOC107764755	0.920963	0.918033	0.700502	0.260733	0.101866	0.030942	1.255233	0.959029	0.2702	0.53322	1.513546	0.024314	0.80096	1.381497	1.506494	0.600515	0.55608	0.364765	0.029204	0.097384	0.104992	1.129645	0.660034	0.060162	0	0	0	0	0	0
Novel02255	2.165141	1.669681	1.158222	1.659738	1.547425	1.342957	1.257229	0.498065	3.518186	0.081203	0.817491	1.055291	1.019728	1.726123	0.683767	0.620564	1.206755	1.241696	1.183019	0.986225	2.050593	1.738621	1.322168	2.175966	0	0	0.295272	0	1.202742	0
LOC107801005	0	0	0	0	0	0	0	0	0	0	0	0	0	0	0	0	0	0	0	0	0	0	0	0	0	0	0	0	0	0
LOC107826511	5.309042	6.495831	5.469001	12.06218	12.13287	10.29214	15.74301	15.60878	11.08726	16.24432	20.53349	12.14896	14.5891	11.90305	23.0834	7.990489	4.422232	6.404668	12.36522	15.31212	9.493244	15.01181	11.76263	10.04939	9.566268	12.88388	7.33714	10.46339	9.861585	10.33909
LOC107781599	0.505922	0.399607	0.234553	0.580563	0.447673	0.435142	0.169735	0.244955	0.253324	0.39467	0.496654	0.170967	0.356693	0.588731	1.073146	0.439849	0.211797	0.35204	0.1369	0.128392	0.169183	0.549261	0.34213	0.176263	0	0.117326	0.159456	0	0	0
LOC107787063	73.7976	78.35283	88.25619	33.63269	29.19259	68.99958	30.06617	33.19681	32.8078	28.94601	30.51066	31.04615	27.77458	34.51581	20.97996	45.92172	86.68621	58.49296	22.64843	25.30165	25.42772	29.05787	37.7839	34.01088	8.551155	6.27397	23.89301	16.12293	7.959607	0
LOC107767434	7.281672	8.304599	7.151628	13.88614	16.37524	7.590799	19.95888	22.32578	14.03152	18.90865	26.16428	16.38203	17.77959	16.89734	32.55812	7.704761	4.275237	5.503779	22.94245	22.06976	21.92266	23.14855	16.69603	12.65664	28.38169	49.14819	15.57226	11.50812	34.37867	62.1874
LOC107822818	11.69837	10.00275	9.455016	9.198936	6.33883	7.454449	5.980863	7.271935	5.553173	7.626285	8.410278	6.35739	8.669662	8.567977	7.622346	14.25187	11.01589	13.86641	14.00475	10.25738	11.6581	6.202056	9.768013	7.487399	5.982589	8.092988	4.505273	2.349996	7.277316	8.127276
LOC107809788	56.72463	63.16838	51.01811	22.77389	16.78208	24.72803	27.82337	25.75089	42.02173	19.67082	31.4334	28.95745	58.65536	48.6794	24.56237	57.86922	71.62409	62.85748	8.537809	8.459385	12.98849	18.17135	31.63299	42.81752	2.441436	1.103555	6.64326	0	8.96474	0
LOC107819923	0	0	0	0	0	0	0	0	0	0	0.016187	0	0.025698	0.100738	0	0.017203	0.022302	0	0	0	0.042108	0	0	0	0	0	0	0	0	0
LOC107770697	2.037909	2.359276	3.207871	4.590656	7.505526	2.960812	21.48151	25.27158	14.11689	7.68033	11.97256	6.607536	4.782514	5.407925	7.801303	31.89169	1.809159	5.256121	13.80482	8.899209	19.13863	20.28494	17.82995	8.750359	9.261603	12.41534	10.41576	27.5723	11.93256	0
LOC107822899	0.05056	0	0.04395	1.763471	3.355386	0.652293	5.322019	6.911921	4.43822	2.736262	2.871647	3.054063	2.476481	4.605698	8.121255	0.037677	0	0.056542	7.823922	6.565188	8.415298	11.54817	5.909092	3.593453	14.09496	31.30576	10.6667	12.65505	9.858185	14.0678
LOC107764357	0	0	0	0	0	0	0	0.082879	0	0	0	0	0	0	0	0	0	0	0	0	0	0	0	0	0	0	0	0	0	0
Novel04376	0.212926	0.097028	0.185092	0.09645	0.376822	0	0.089295	0	0.099952	0.20763	0.298607	0.359771	1.422192	0.696875	0.655624	0.079336	0.102852	0.079373	0.324094	0.540362	0.48548	0	0.093907	0.111275	0.807609	0	0.251661	0	2.050202	0
LOC107766608	0.150049	0.239315	0.108696	0.090625	0.088516	0.053774	0.314632	0.192309	0.035218	0.121931	0.271805	0.105638	0.153117	0.122772	0.308013	0.605676	0.314081	0.456795	0.203013	0.074043	0.079828	0.240178	0.154412	0.065347	0.079045	0.12179	0.088673	0	0.060199	0
LOC107818861	2.848575	3.080476	2.531643	3.042873	2.783953	2.216932	5.634285	4.940453	3.712182	3.0472	4.605996	4.112695	4.70928	6.099353	5.018289	4.261364	3.696666	3.787853	2.631683	2.373733	2.404071	4.562554	3.468931	2.133012	2.418894	2.425477	1.030136	6.080996	1.842186	13.14415
LOC107784556	10.3526	10.51916	11.39273	9.319068	9.33608	11.84076	7.999604	7.509302	8.520054	8.548612	6.363429	6.7178	6.988358	5.935452	6.759707	9.700915	9.49077	9.869576	11.37681	9.670614	9.260948	9.068817	9.500833	11.21192	9.941946	8.121906	7.810176	1.575229	9.756133	4.085853
LOC107823239	7.224795	4.986798	6.788331	16.36324	28.2513	24.0451	14.16911	19.01847	15.5609	22.37851	13.18658	26.88324	20.99392	26.13776	17.12069	4.552584	4.926889	5.029939	29.21708	25.88476	29.748	16.0157	11.48032	14.43641	43.72392	29.1974	44.45369	94.97702	58.56799	39.41646
LOC107795547	8.675905	12.19306	8.035167	13.0754	11.76663	12.20441	9.249087	10.11484	8.678193	10.43676	12.79251	7.329645	12.81734	13.35713	13.64834	11.72212	9.478318	11.96929	10.86065	16.59898	4.880651	11.10521	7.581201	8.051075	8.303621	5.302605	9.487551	5.798597	10.14945	5.013498
LOC107818770	0.090466	0.109931	0.122329	0.163915	0.124523	0.140489	0.10117	0.146005	0.103807	0.107819	0.148014	0.067936	0.145467	0.098694	0.123802	0.179774	0.11653	0.07494	0.214197	0.221081	0.064172	0.083945	0.16846	0.105061	0.16521	0.083918	0.059402	0	0	0
LOC107788404	0.898405	0.570669	0.780965	0.468615	0.457709	0.614661	0.205509	0.255874	0.460071	0.265472	0.190898	0.482999	0.424292	0.59401	0.167654	0.60863	0.604925	0.913363	0.497258	0.4606	0.27312	0.090943	0.576329	0.455279	0.13768	0	0.128708	0	0.262136	0
LOC107783254	78.30861	43.03265	71.50083	65.89876	49.22342	79.07749	48.6038	51.59428	106.1949	21.60727	18.83211	40.52747	31.12443	42.4623	12.44306	42.23222	56.71151	53.01986	19.48295	15.75128	43.43319	24.26976	47.9932	78.3317	0.733377	0.403558	6.170299	8.296553	4.468214	7.173244
LOC107767118	4.116804	3.033499	3.426367	3.531232	2.712738	3.296008	2.387672	2.619422	3.618332	2.306147	2.5489	1.960995	0.731311	1.242255	1.798032	2.15402	3.04635	2.318265	1.288787	1.889459	1.837355	2.669975	3.863078	4.211332	0.387598	0.121877	0.983499	0	0.632546	0
LOC107818875	22.63072	25.10736	20.99033	18.05675	15.59148	15.7037	22.43956	18.53062	18.10484	17.43429	23.38814	18.79104	18.44181	17.38719	23.56913	23.27127	21.47021	20.53882	15.92852	14.65519	14.82206	19.88771	17.09147	14.02984	7.947808	11.937	9.637035	11.90012	8.901335	4.572848
LOC107800975	0	0	0	0	0	0	0	0	0	0	0	0	0	0	0	0	0	0	0.049907	0.124816	0	0	0	0.051406	0.062182	0.068434	0	0	0	0
Novel04432	0.939683	1.160291	0.816847	0.768921	0.616915	0.554021	0.813577	0.647493	1.166802	0.502492	0.680162	0.589001	1.113557	0.231485	0.591124	0.722842	0.644254	1.039561	0.615178	0.487201	0.165872	0.354405	0.802125	0.760379	0.38324	0.16871	0.358268	0	0.291869	1.874259
LOC107785755	2.436268	2.127843	1.76483	1.839279	2.15577	1.855328	1.191985	1.387742	2.191973	0.79189	1.067695	1.62943	1.582051	2.547105	0.833506	1.739864	2.549776	1.286575	0.412026	0.6011	1.018379	1.610718	2.148949	1.90979	0	0	0.239956	14.51897	0.977422	0
LOC107819200	0	0	0	0	0	0	0	0	0	0	0	0	0	0	0	0	0	0	0	0	0.071407	0	0	0	0	0	0	0	0	0
LOC107793257	0.297612	0	0	0	0	0.127988	0	0	0	0	0	0	0	0	0	0	0	0	0	0	0	0	0	0	0	0	0	0	0	0
LOC107820477	302.3308	287.6097	356.5843	70.59185	67.3402	189.1428	25.85158	40.68608	42.51119	27.77569	18.63755	52.80968	48.76826	67.81687	14.0401	191.4898	316.6676	239.9237	97.25382	102.3151	71.69632	23.93251	63.30876	41.1736	15.10563	5.180087	66.92275	37.14936	10.42045	32.11953
Novel00877	0	0.038426	0.012217	1.375092	1.367963	0.317308	2.051079	1.885293	1.84725	1.493797	1.744294	1.519785	0.391131	1.594567	0.216372	0.010473	0.067887	0.020956	1.22646	1.081884	0.935685	2.112656	1.586775	1.836172	1.22604	0.391106	0.946819	0	1.285571	0.868988
LOC107763409	0	0	0	0	0	0.134082	0	0	0	0	0	0	0	0	0	0	0	0	0	0	0	0	0	0	0	0	0	0	0	0
LOC107806208	1.479874	1.277738	1.794222	1.658223	0.999348	0.711784	2.384476	2.894913	5.155298	1.443064	1.802302	1.809556	2.080947	3.058996	2.15844	2.147566	1.768292	2.35179	1.936369	2.075472	2.202094	4.195493	4.912227	2.930711	0.738557	1.300508	0.828517	2.785051	2.812359	0
LOC107830100	2.790725	3.179252	2.425918	4.375827	2.849327	6.520036	3.826146	3.897693	2.972755	4.291295	4.177139	4.171284	7.04976	4.039875	5.177816	2.319611	3.266399	2.520732	4.193294	3.813538	2.936756	2.778801	2.792974	4.150923	2.849801	2.090895	3.869291	0	2.325383	6.63672
LOC107800811	0.458968	0.209147	0.3491	0.467777	0.203063	0.431766	0.144359	0.098039	0.107725	0.223776	0.201143	0.339281	0.191599	0.312945	0.117768	0.171012	0.277126	0.17109	0.116432	0.339724	0.104647	0.095824	0.10121	0.239857	0.145069	0.079828	0	0	0.552409	0
LOC107828772	0.280706	0.537241	0.292814	0.45775	0.546452	0.422511	0.23544	0.33578	0.368955	0.328469	0.334613	0.308293	0.718718	0.459355	0.230487	0.376529	0.433897	0.376701	0.256357	0.356187	0.102403	0.586063	0.346642	0.821503	0.141959	0.039058	0.033177	0	0.135142	0
LOC107831380	0.96593	0.480178	0.64883	0.994413	1.204376	1.132902	0.920645	0.712774	1.236624	1.755366	1.416195	1.038602	1.61293	1.19748	0.991404	0.425344	0.805919	0.392805	0.623738	0.742828	0.920988	0.623339	0.426007	0.9637	0.999188	0.610919	1.608691	0	2.113787	0
107772092	0	0	0	0	0	0	0	0	0	0	0	0	0	0	0	0	0	0	0	0	0	0	0	0	0	0	0	0	0	0
LOC107832425	17.38045	17.60015	17.51254	16.09139	12.80559	22.78731	14.39776	11.77433	14.0568	14.55354	19.45937	14.4015	19.02967	15.65796	13.85016	15.33267	17.13647	19.62343	9.483539	11.03204	7.066749	12.80255	14.42642	15.92332	4.069291	3.151501	6.988324	0	6.312979	2.948311
LOC107785631	11.64791	14.0453	15.96679	13.42592	10.46541	9.864215	10.23554	9.186731	9.505536	6.43048	9.813593	3.693964	5.844952	5.08292	9.453723	16.7725	12.94945	16.01866	8.091809	8.443547	3.693567	14.74087	11.60197	10.88203	1.155464	2.618089	2.435681	2.563047	1.466634	3.324036
LOC107812775	0.288859	0.058502	0.0558	4.245219	3.379619	2.553483	14.07907	9.680396	18.80275	2.159501	0.472612	0.569418	16.75695	15.86159	3.162421	0.287011	0.527117	0.263214	1.009615	1.303226	1.580666	11.84725	6.284887	6.977599	1.541979	0.133975	2.920936	4.590556	1.854227	0
LOC107814227	5.342736	6.0786	5.828175	3.574824	3.321683	3.791853	3.543942	4.102513	4.212781	2.485742	2.86484	2.020836	2.643381	2.666708	2.490933	5.282528	3.879583	5.701495	3.047345	3.293686	2.006391	3.922352	3.927205	4.124297	2.317838	2.210773	1.650898	4.994525	1.344932	1.079573
LOC107782374	3.49359	3.513354	3.120681	1.680733	0.703551	1.709644	1.899594	1.976288	2.69181	0.869294	1.604981	1.017752	0.590072	0.788552	0.840791	3.160022	2.630252	2.407028	0.513422	0.591067	0.28566	1.790796	2.890307	3.450038	0.030462	0	0.256292	0	0.347987	0
107821900	0	0	0	0	0	0	0	0	0	0	0	0	0	0	0	0	0	0	0	0	0	0	0	0	0	0	0	0	0	0
LOC107800878	26.47517	23.19282	25.7423	19.38456	15.34871	21.34804	18.28156	16.2833	21.58834	15.02261	18.08429	17.01625	19.88126	15.93551	14.52371	17.39072	21.11225	22.4609	9.958643	9.991397	9.731175	15.86782	21.99396	25.7038	3.751247	2.699356	9.104215	4.080494	5.494005	3.528017
107792608	0	0	0	0	0	0	0	0	0	0	0	0	0	0	0	0	0	0	0	0	0	0	0	0	0	0	0	0	0	0
LOC107780737	0	0	0	0	0	0	0	0	0	0	0	0	0	0	0	0.163149	0.264385	0.081612	0	0.138902	0	0.685638	0.820733	0.572072	0.0692	0	0	0	0	0
LOC107769213	12.60151	8.924873	5.873047	2.544603	2.754075	2.285232	2.419507	1.880994	3.349699	1.998661	4.524553	4.553454	2.281691	2.981413	2.181612	7.636981	15.621	10.35709	2.696084	1.990724	3.738618	2.599264	2.209681	3.253101	0.28793	0.105627	0.358891	0	1.096412	0
LOC107761362	0	0	0	0.136951	0.200645	0	0.063396	0	0.070962	0.073704	0	0	0.168282	0.082459	0.232732	0	0.146041	0	0.076697	0	0	0	0	0	0.095561	0.210339	0	0	0	0
LOC107810279	5.696355	3.123027	2.553231	1.975546	1.260127	1.578915	1.082465	0.798512	2.548651	1.215083	1.092188	2.556608	3.913747	3.010121	1.27894	2.4541	3.998388	2.322509	0.632217	0.376463	0.243524	1.040632	1.413158	1.023314	0.11253	0.061922	0.315592	0	1.071261	0
LOC107772767	0.571119	1.264082	2.517769	0.369575	0.324877	0.307011	0.068432	0.209134	0.382994	0.198898	0.17163	0.137856	0.136238	0.133513	0.08374	3.404788	1.103498	4.014623	0.331161	0.241564	0.44646	0.136273	0.143933	0.810124	0.051576	0.113524	0.096431	0	0.196398	0
LOC107803100	0	0	0	0	0	0	0	0	0	0	0	0	0	0	0	0	0	0	0	0	0	0	0	0	0	0	0	0	0	0
LOC107764942	10.41088	8.104536	7.070282	7.761531	8.924353	7.345389	4.27507	4.72565	7.941535	7.085199	5.475089	10.16969	9.054329	8.163392	4.340963	5.010506	4.505052	5.821317	10.12407	10.36645	9.593807	3.894367	5.261144	6.574211	5.484384	6.186723	6.79329	7.755473	7.309415	20.11627
LOC107823743	41.93394	33.96104	34.53145	22.24805	15.99776	26.67252	21.60854	21.75516	34.23019	15.57282	16.72689	22.82599	23.92774	35.55733	15.87578	34.65483	34.30153	32.27445	11.97562	10.91811	14.15274	18.49566	21.88302	25.30029	3.42866	3.004748	9.259589	0	7.011572	9.315588
LOC107825096	16.38405	18.09774	18.49094	13.28361	10.90726	11.17039	10.90594	10.0593	12.26525	6.084863	7.242283	6.596231	8.110001	9.322747	7.855943	16.03487	15.17504	17.00475	7.704452	6.136786	10.68124	13.68274	11.52349	15.61455	0.738412	1.924717	3.088167	8.793177	3.255778	7.602628
LOC107791177	0.121156	0.110419	0.140424	0.109761	0.32162	0.303934	0.101619	0.172532	0.34124	0.669473	0.283182	0.989441	0.854193	1.542044	0.331603	0.030095	0	0.060218	0.327842	0.239142	0.18416	0.101181	0	0.042211	0.459534	0.16858	0.334125	0	0	0
LOC107783829	78.89139	56.26089	69.10068	48.61934	50.22097	51.70247	47.09952	51.02255	57.47319	51.53703	43.79772	56.75548	38.96877	49.60882	38.39453	50.92572	60.92284	53.15587	39.65675	38.10414	51.72282	37.90405	44.5753	52.55025	42.08827	38.73941	63.78468	55.22202	44.40425	45.09274
LOC107810195	0	0	0	0	0	0	0	0	0	0	0	0	0	0	0	0	0	0	0	0	0	0	0	0	0	0	0	0	0	0
LOC107782850	0	0	0	0	0	0	0	0	0	0	0	0	0	0	0	0	0	0	0	0	0.063502	0	0	0	0	0	0	0	0	0
LOC107832009	0	0	0	0	0	0	0	0	0	0	0	0	0	0	0.030529	0	0	0	13.94431	14.66924	11.9089	9.414461	8.60556	4.943113	3.046076	1.572708	4.745979	0	5.155192	5.51741
LOC107772396	22.45427	16.09759	18.56601	27.25812	26.04889	26.32382	22.03469	24.14672	22.60591	19.71805	18.59214	24.18417	29.01934	32.82443	24.75513	17.91303	18.9468	18.01205	22.66143	25.521	30.21808	20.68501	19.80615	14.68416	22.33133	25.08518	17.80474	58.07587	37.533	52.72334
LOC107784471	6.066172	5.363042	6.725691	17.40836	14.04614	13.86387	14.34987	12.04218	15.27811	8.890636	9.271328	10.52593	12.93978	12.28472	7.6056	6.442416	7.229036	5.416274	12.49536	10.84696	15.07344	12.58921	13.07253	14.08547	5.970258	6.267298	7.169727	20.78166	9.618318	13.47595
LOC107774568	2.654259	3.061925	2.296898	4.419309	3.935597	3.817724	5.194562	4.423369	3.502179	2.588237	3.252852	1.494928	3.061235	2.778274	2.957416	2.51254	2.044459	2.344328	3.736731	3.954595	2.311687	4.772767	4.165705	3.449043	2.297658	1.680243	1.243543	3.420127	2.302441	6.653384
LOC107794674	0	0	0	0	0	0.116957	0	0.046474	0	0	0.11442	0	0.06055	0.237357	0.111653	0.283732	0.262738	0.486621	0.220774	0.046012	0.099213	0.181698	0.383821	0.454806	0.137537	0	0.514298	0	0	0
LOC107820795	11.05207	8.679581	10.98711	8.468117	7.178642	9.164561	6.755155	7.333541	9.382708	6.477764	7.667275	7.499477	6.675205	6.349305	4.706363	8.148399	8.689448	10.60654	5.488132	5.669251	4.932607	6.922405	9.696803	16.95876	3.493294	2.535759	4.863756	4.204145	3.679318	7.269853
LOC107815447	0.543147	1.515973	2.154168	3.936511	4.595856	1.751849	8.143153	8.49846	6.533485	6.256339	7.093428	5.276958	4.383633	5.814367	11.56753	8.449239	1.213429	2.834575	5.821504	7.696042	5.232234	10.48942	8.833255	5.215719	5.665302	14.78435	3.410393	7.283008	6.373829	4.197951
LOC107805406	0.048232	0.263744	0.167708	0.174782	0.192054	0	0.040454	0.103026	0.090564	0.070548	0.135281	0.16299	0.053692	0.026309	0.148511	0.161741	0.023298	0.161815	0.391539	0.367207	0.285924	0.100699	0.127631	0.100824	0.396368	0.268444	0.142515	1.724629	0.348308	0
LOC107795011	42.91784	54.37606	45.90507	46.06248	46.19109	27.21683	83.55387	71.38785	66.44014	40.35317	43.56372	36.68049	56.75972	42.96493	57.93891	53.0291	49.49943	46.5049	30.61547	28.69406	38.39282	82.59718	61.63471	44.11133	12.86559	8.743927	15.99413	15.32063	19.59641	11.03858
LOC107830335	0	0	0	0	0	0	0	0	0	0	0	0	0	0	0	0	0	0	0	0	0.15485	0	0	0	0	0	0	6.07114	0	0
LOC107829745	2.78882	6.908117	2.983707	17.3294	21.13391	8.456807	34.63673	31.31301	13.09134	20.11697	25.34658	16.9456	29.45346	25.70493	40.58692	14.52098	8.186328	12.07526	94.83799	87.04658	94.62941	69.75106	50.08152	38.30443	184.5682	134.4224	167.3858	150.8585	161.2886	190.1228
LOC107817657	0.150193	0.171103	0.228479	0	0	0	0	0.032082	0	0.036614	0	0	0	0	0	0.139905	0.181374	0.083981	0	0	0	0	0	0.039245	0	0	0	0	0	0
LOC107800760	0	0	0	0	0	0	0	0	0	0	0	0	0	0	0	0	0	0	0	0	0	0	0	0	0	0	0	0	0	0
LOC107764790	4.833945	8.022037	5.393671	4.117858	5.873434	4.34383	6.0514	5.054937	5.147946	6.824322	8.246256	6.0953	8.83483	6.611673	8.589936	10.48419	7.597458	7.422981	5.27121	5.920163	2.368821	6.206063	5.409373	4.373752	4.83453	4.919075	3.496219	25.79817	5.210224	0
107766018	0	0	0	0	0	0	0	0	0	0	0	0	0	0	0	0	0	0	0	0	0	0	0	0	0	0	0	0	0	0
LOC107813081	0.754723	0.678904	0.25561	1.385242	1.066792	0.885185	1.52089	1.850821	1.361922	1.12782	2.192452	0.803225	1.047483	0.588119	1.197151	0.3506	0.359829	0.591908	1.064221	0.737942	1.108464	1.686228	1.478408	1.178133	0.656787	0.76374	0.996285	0	1.887536	0
LOC107783549	2.717683	3.676141	3.65554	12.64733	11.61891	8.734768	9.189733	7.601698	10.12535	5.830185	8.204286	7.371285	4.076278	3.932339	5.314456	2.451583	1.077841	1.194361	8.447322	9.195891	7.540079	6.092106	8.907401	5.800644	1.627568	2.905751	2.197741	0	1.377252	3.537651
LOC107806375	0.201979	0.153399	0.204838	0.152485	0.208511	0.036192	0.169408	0.230101	0.189626	0	0.094418	0.170637	0.26232	0.403972	0.207305	0.025086	0.22765	0.050195	0.170795	0.028477	0	0.224903	0.029693	0.105554	0.04256	0.421558	0.238722	0	0	0
LOC107811651	26.16377	24.05923	24.51407	33.21266	30.03678	34.19304	28.95296	27.30795	32.31033	24.23988	24.26	24.1775	23.57393	24.12799	23.93256	22.45494	23.40968	23.02978	22.89271	24.25325	21.93285	30.1583	29.20472	31.27638	13.66735	15.15057	15.15312	18.67345	13.95384	11.30161
LOC107796387	19.71094	22.915	23.54612	16.70577	16.00829	18.27145	15.75909	15.26599	16.21278	13.07296	15.51622	11.89453	14.20298	14.57138	13.78557	20.11096	21.88314	20.91903	10.92359	14.10237	9.70424	16.79409	17.45229	18.2057	4.379224	4.022066	5.802096	3.564123	4.918729	0
LOC107772003	0.02941	0	0	0	0.052048	0	0	0.025129	0.027612	0	0.020623	0.024847	0.03274	0.032085	0.090558	0	0.028413	0.06578	0	0.024879	0	0.049123	0.077826	0.03074	0	0.040922	0	0	0	0
LOC107803681	5.381482	6.006569	6.133389	3.057627	2.292762	4.100026	2.975686	2.486372	2.170649	1.347538	2.347955	0.886834	1.434819	1.681551	2.563951	5.871852	4.133498	5.775477	2.170819	2.78762	0.957363	3.650857	3.328637	2.958188	0.151196	0.388264	0.502556	0.950252	0.063971	1.643186
LOC107812188	0.263172	0.363642	0.23615	0.415313	0.360576	0.465486	0.291938	0.232115	0.111583	0.115896	0.392884	0.078893	0.047253	0.268585	0.304966	0.30999	0.188634	0.32912	0.344579	0.452433	0.147107	0.219782	0.142276	0.079858	0.064399	0.106312	0.070237	0	0	0
LOC107793951	13.16074	6.417002	8.465863	5.66341	3.66833	7.782175	2.207697	2.13653	2.471177	5.775024	2.168656	6.893509	2.344116	4.881626	2.093713	6.080589	4.386463	4.807828	1.7361	2.67194	1.560367	0.989179	2.844118	4.952018	1.41433	1.73966	1.788818	4.705896	1.900815	0
LOC107819221	0.942957	1.35839	0.62138	0.812936	0.834391	0.523249	1.620067	1.117568	0.88529	0.830518	1.535696	1.027918	1.96398	1.012128	2.263463	2.50476	1.748486	2.029672	1.620468	1.543891	1.830953	3.137252	1.743994	1.224025	0.423033	0.804164	0.287613	1.087658	1.537651	0.940395
LOC107768544	7.199967	6.807953	8.527586	1.168669	0.76983	1.64493	0.327108	0.435756	0.619634	0.526566	0.483826	0.506894	0.968492	1.178222	0.400283	6.393797	7.39051	7.000612	1.217672	0.685201	0.71137	0.526127	1.746483	0.75254	0.227574	0.083485	1.347382	0	0.28886	0
LOC107826562	0.4926	0.37412	0.499574	0.396685	0.314806	0.176535	0.459072	0.549496	0.488167	0.386946	0.690822	0.439282	0.472206	0.447835	0.6039	0.958502	0.594866	0.377455	0.152734	0.115752	0.162232	0.617074	0.736245	0.47196	0	0.038079	0.032345	0	0	0
LOC107777874	0	0	0	0	0	0	0	0.050915	0	0	0	0	0	0	0	0	0	0	0	0	0	0	0	0	0	0	0	0	0	0
LOC107814187	13.4905	13.50924	12.77664	12.22171	10.97935	13.20574	9.778446	8.751712	10.94547	5.846638	6.715108	7.000098	5.608502	5.950581	5.213721	12.06996	14.32016	13.2551	9.928911	9.016913	9.455513	8.658333	11.2017	12.57709	2.421543	2.954701	4.773562	2.977658	2.806397	0
Novel00856	1.331219	1.044736	1.414356	1.40702	1.505158	1.888419	1.271627	1.090037	1.614334	0.793287	0.868628	1.233992	1.975908	1.754856	0.70214	1.308923	1.303931	1.433582	1.257021	1.016636	0.927433	0.555868	0.782815	1.661937	0.374014	0.231537	0.699286	0	1.424214	0
LOC107785751	0	0	0.550243	0	0	0.34027	0	0	0	0	0	0	0	0.17264	0	0	0	0.11798	0	0	0.144324	0	0	0	0	0	0	0	0	0
LOC107789396	42.65209	44.49835	50.60625	37.04756	34.86111	39.62138	33.92268	35.48408	44.0129	21.92658	23.19156	24.55979	18.07606	22.65341	19.66122	40.20446	43.15735	41.05969	26.68958	28.16829	31.32427	36.93219	44.81634	50.95411	10.87965	12.23387	16.58268	16.05383	12.06837	20.82034
LOC107766184	4.5045	3.850986	3.846362	3.178006	2.857134	2.528588	2.57447	2.554491	2.282924	1.088396	2.320003	1.784893	1.863786	1.870001	0.818282	3.119112	3.542998	3.863526	1.739352	1.854667	1.89049	3.462211	3.973267	3.583157	0.856779	0.665595	0.989408	5.701512	0.191914	2.464779
LOC107830478	0.819145	1.430887	0.94942	0.226754	0.060403	0.097853	0.133593	0.058325	0.021362	0.066564	0.03191	0.05767	0.05066	0.024824	0.070062	1.237812	0.85731	1.509804	0.023089	0.038497	0.062256	0.057007	0.080282	0.047565	0.057536	0.031661	0.08068	0	0	0
LOC107793613	0.578782	0.644708	0.503123	0.72826	0.995836	0.725972	1.051807	0.604422	0.603762	0.877933	1.037152	1.222428	1.431796	1.08745	0.924071	0.646963	0.7766	0.74315	0.880962	0.788816	0.90909	0.456503	0.368712	0.97463	1.097634	0.984296	0.570061	0	2.322052	1.988164
LOC107787217	0.449826	0.36441	0.521366	0.40752	0.707619	0.913501	0.167684	0.128115	0.093848	0.048737	0.105139	0	0	0.054526	0.051299	0.409702	0.43457	0.81978	0.456453	0.338242	0.319083	0	0.440863	0.522398	0	0.069544	0.059073	0	0	0
LOC107789671	0	0.044079	0	7.448827	6.547928	2.07995	19.35006	30.16706	17.84519	6.178275	10.71678	8.580697	8.183843	9.814161	27.9975	0.036042	0.233625	0.108175	11.28792	14.1971	7.322274	22.05361	23.7198	8.138797	5.381077	15.27641	12.63325	20.75287	9.313936	11.96203
LOC107762152	0	0	0	0	0	0	0	0	0	0	0	0	0	0	0	0	0	0	0	0	0	0	0	0	0	0	0	0	0	0
LOC107807035	3.240378	3.717564	3.844093	7.218168	6.88152	8.811972	5.883335	5.472189	7.086559	5.724774	5.39971	8.051652	7.511535	7.194955	4.147512	0.738624	0.957558	0.881071	3.249428	2.644391	3.720175	1.241634	2.051199	3.346997	1.494136	1.591323	1.622061	5.452538	2.2024	0
LOC107767681	0	0	0	0	0	0	0	0	0	0	0	0	0	0	0	0	0	0	0	0	0	0	0	0	0	0	0	0	0	0
LOC107801113	0	0	0	0	0	0.07449	0	0	0	0	0	0	0	0.075587	0	0	0	0	0	0	0	0	0.030557	0	0	0	0	0	0	0
Novel04748	5.274237	8.682312	7.278341	8.600744	8.925621	6.485576	7.18856	5.238749	4.301777	3.407268	7.928226	1.726631	2.752197	3.416398	11.33416	10.1207	7.069806	10.32194	7.091308	9.70407	1.443058	13.32407	6.076969	2.928585	1.625383	3.302422	1.441548	0	2.063104	2.038212
LOC107801663	12.76586	16.81797	15.00482	6.570126	5.098616	4.592755	5.311975	4.307825	5.153149	4.577302	5.781857	4.469251	5.059606	4.145563	4.690399	12.64099	10.91725	11.18398	3.099866	4.601147	2.582228	4.998688	5.717795	4.438971	2.260846	0.898505	2.172234	0	2.630558	3.071332
LOC107814366	0	0	0.0496	0	0	0	0.047857	0	0	0	0	0	0	0	0.17569	0	0	0.04254	0	0	0	0.095302	0	0.119275	0	0	0	0	0	0
LOC107820482	9.703445	9.444508	9.376771	4.950176	3.709592	6.786118	7.309024	6.318783	7.517979	3.628663	4.822296	4.457024	6.292433	5.395767	4.25448	6.528978	9.647383	7.690865	2.246505	2.191576	2.190946	5.507301	6.772476	6.301849	0.595539	0.983129	1.725871	0	2.040983	0
LOC107815730	3.156634	2.524233	2.797105	3.837601	3.964538	3.021548	4.253249	3.532349	3.116547	3.078117	3.141648	3.303406	4.42085	3.532603	4.222299	3.156672	2.518358	3.036651	2.872488	2.911483	2.470285	2.959333	2.712506	3.490886	3.089764	2.465314	2.407024	1.456413	5.000356	11.333
LOC107824330	23.80615	25.04738	25.27182	16.45767	13.62072	16.68334	13.56414	13.49829	16.38239	9.26737	12.08268	11.87246	11.65504	11.38796	12.50482	29.88463	24.68451	27.90045	6.924538	6.405265	7.587696	12.30861	13.46769	17.03322	1.812196	1.690911	3.093592	4.456744	4.200413	5.779987
LOC107764336	0	0	0	0	0	0	0	0	0	0	0	0	0	0	0	0	0	0	0	0	0	0	0	0	0	0	0	0	0	0
LOC107763497	0	0	0	0	0	0	0	0	0	0	0	0	0	0	0	0	0	0	0	0	0	0	0	0	0	0	0	0	0	0
LOC107771519	14.99905	12.02576	12.83509	15.94895	12.91744	16.01807	11.87989	12.78705	15.49308	10.00038	9.908322	11.65563	13.35029	12.86463	11.21167	11.57561	12.68309	12.5024	13.01666	12.17951	12.06467	11.77286	14.37932	16.44514	5.912852	6.414414	7.185827	19.1117	11.2578	8.26204
LOC107795674	6.688968	14.1775	8.677139	2.610404	2.61036	1.493611	4.934209	4.088592	2.350936	3.595786	7.059515	1.651838	2.863943	3.18089	10.24525	24.59082	9.726297	10.39579	1.67077	3.3225	1.767567	3.552207	4.81086	1.452041	1.040848	0.882992	0.973024	2.453106	0.660576	0
LOC107811733	1.219853	1.078057	0.546263	2.679092	1.439209	4.252412	0.558078	0.442178	0.381751	0.108137	0.10368	0.031229	0.04115	0.040327	0.07588	0.468291	0.428538	0.385828	0.675176	0.18762	0.707972	0.123483	0.554299	0.115908	0	0	0	0	0	0
LOC107788903	2.626386	1.753079	3.119113	0.770783	0.916507	0.874945	0.558472	0.695341	1.423894	0.504997	0.259384	0.312513	0.741227	0.766759	0.759338	1.295602	1.393734	1.571982	0.150145	0.281629	0.472314	0.401602	0.55469	0.425297	0.093537	0.051471	0.262325	0	0.890448	0
LOC107809508	21.25357	9.705831	13.628	28.15735	27.78517	46.48661	8.012243	6.092261	9.569235	24.95903	30.38292	24.36556	16.38375	21.5411	10.60211	13.3177	18.28076	21.94508	33.20801	31.55034	23.6355	6.030982	10.24033	21.06768	22.53694	4.070233	24.36371	6.537336	23.32507	0
LOC107802938	5.894181	7.371759	6.035997	8.547568	7.504401	7.41972	8.233578	7.880304	7.424735	7.846054	7.976102	5.140777	6.80931	6.198957	7.485868	7.820912	5.427995	7.042044	7.013699	6.833429	4.399091	8.746971	7.480658	8.210435	6.406607	6.549258	3.90923	2.274374	6.073427	6.554786
LOC107790352	0	0	0	0	0	0	0	0	0	0	0	0	0	0	0	0	0	0	0	0	0	0	0	0	0	0	0	0	0	0
LOC107779986	4.038983	5.023802	3.798065	3.498021	3.236792	3.304594	3.579423	2.951813	3.863521	3.319254	3.028071	2.553818	2.969223	2.688139	3.154752	4.297088	3.484802	4.261179	3.660278	3.481167	2.803258	2.906347	3.338584	3.106419	2.151796	3.039721	2.101652	1.816631	2.690517	0
LOC107786017	0	0	0	0	0	0	0	0	0	0	0	0	0	0	0	0	0	0	0	0	0	0	0	0	0	0	0	0	0	0
LOC107761888	12.36117	10.2883	11.57942	15.34054	11.28759	14.80687	17.65848	13.60002	13.72489	8.366041	10.68616	9.632413	14.2633	14.71698	8.863638	0	0	0	0	0	0.051478	0	0	0	0.071362	0	0	4.036523	0	0

LOC107803506	0	0	0	0	0	0	0	0	0.063535	0	0	0	0	0	0	0	0	0	0	0	0	0	0	0	0	0	0	0	0	0
LOC107826152	5.793805	8.757635	7.493227	38.7906	56.22741	26.08097	54.71891	55.25916	43.95807	60.5849	39.00775	46.75895	45.20065	47.12303	44.14023	10.63592	6.871485	8.814645	71.2668	59.57101	83.25596	40.71469	32.28352	27.61982	55.02781	62.0682	47.99035	60.63497	52.83879	34.95021
LOC107801578	47.29716	56.16199	54.02563	110.9632	91.6961	128.4846	79.31824	64.82567	78.91964	39.98326	54.43754	45.18631	45.23487	41.51469	43.13866	42.5659	52.30784	44.11682	77.92327	100.2481	75.22347	74.79756	79.16432	88.60312	22.74055	15.71978	31.31228	22.59966	25.86408	34.19463
LOC107823418	1.288614	0.913432	1.078677	1.361989	1.499218	1.103203	0.94071	0.856354	0.806538	0.860977	0.937043	0.725771	0.850076	0.885142	0.783762	1.10254	0.806886	1.03188	0.96859	0.706532	0.761728	1.096084	0.926153	1.521451	0.482726	0.332039	0.338453	3.413115	0.689316	1.475499
LOC107762770	1.665493	1.568488	0.868667	1.986656	1.4983	1.909983	1.629741	2.087129	1.511517	1.921812	2.588723	1.641564	1.946753	2.240931	1.937339	1.551409	1.66264	1.696984	1.436523	1.5263	0.987323	1.414079	1.664952	1.50867	1.228319	1.004212	1.476359	0	1.470017	3.432665
LOC107802150	5.030144	3.541209	4.346414	1.609978	0.959497	1.748701	2.248448	2.239021	5.429485	0.440571	0.190085	0.330808	0.838267	1.445845	0.43281	2.558835	3.928372	3.660361	0.733544	0.35672	0.961468	2.087825	3.77271	3.431545	0	0	0	0	0	1.862403
107817567	1.415669	0	0	2.565049	0	0	0	0	3.987278	1.380456	0	0	0	0	0	2.109917	2.735311	1.055441	0	0	1.291114	1.182262	1.248714	0	0	0	1.673207	0	0	0
LOC107778469	5.494381	8.067559	6.951949	8.793814	8.211022	7.219827	8.807114	8.345863	6.361985	5.119594	7.106023	3.300839	3.058211	3.796263	4.824719	5.231757	5.072121	6.599585	6.876225	7.694804	7.015355	10.55355	9.854361	8.805512	7.100936	3.567681	3.896349	0	2.35128	15.09893
LOC107797912	0.70256	0.782585	0.407147	0.565763	0.37991	0	1.505906	1.133874	0.916103	0.685085	0.903165	0.659491	1.1297	0.894202	1.442177	1.221613	1.018098	0.494689	0.039606	0.132071	0.177985	1.401624	0.791845	0.244772	0.493472	0.325854	0.230658	0	1.315368	0
LOC107831719	0	0	0	0	0	0	0	0	0	0	0	0	0	0	0	0	0	0	0	0	0	0	0	0	0	0	0	0	0	0
LOC107784430	0	0	0	0	0	0	0	0	0	0	0	0	0	0	0	0	0	0	0	0	0	0	0	0	0	0	0	0	0	0
LOC107832414	0	0	0	0	0	0	0	0	0	0	0	0	0	0	0	0	0	0	0	0	0	0	0	0	0	0	0	0	0	0
Novel01098	3.698638	3.62768	3.307009	3.031657	3.14812	3.938617	4.904442	3.912649	4.79528	2.713567	3.581483	3.5711	4.54872	2.651593	1.337702	4.593716	3.981561	4.385725	3.10973	2.950005	3.469598	2.176889	4.784917	4.454892	2.182228	1.568422	3.83026	12.59547	2.882967	8.71209
107814169	0	0	0	0	0	0	0	0	0	0	0	0	0	0	0	0	0	0	0	0	0	0	0	0	0	0	0	0	0	0
LOC107824796	0.290653	0.586551	0.523363	0.6771	0.698083	0.691936	0.609457	0.425728	0.896598	1.093203	0.917126	0.456024	0.462227	0.498281	0.916262	0.789024	0.240681	0.91321	0.990136	1.088862	0.359752	0.953589	0.531062	0.759475	0.288729	0.866619	0.687056	1.484698	0.399802	0
Novel03833	3.91926	2.899561	4.20856	2.339253	2.978417	3.321375	2.011025	2.285011	3.030238	0.989165	1.616581	1.480256	2.053165	1.257565	1.230449	3.058079	2.271801	3.712627	0.748611	1.443185	2.144659	2.348914	3.497718	4.72292	0.174887	0.320786	0.762959	0	0	0
LOC107818277	0.169789	0.154741	0.147594	0.025637	0	0	14.88174	10.27593	3.453774	0.413913	9.008557	0.143442	8.190475	5.742159	9.381335	0.084351	0	0	0	0	0	8.838545	0.32449	0	0	0	0	0	0	0
LOC107773120	0.850321	0.774963	0.295666	2.002904	1.655324	2.376917	0.998481	1.162459	1.117646	0.663336	2.265733	1.436749	1.70386	1.484253	1.396394	1.774248	0.492889	1.14111	1.380555	1.438626	0.620406	0.994175	1.500078	2.133012	1.075064	1.183159	0	0	0.818749	0
LOC107802186	0	0	0	0	0	0	0	0	0	0	0	0	0	0	0	0	0	0	0	0	0	0	0	0	0	0	0	0	0	0
LOC107811860	0	0	0	0	0	0	0	0	0	0	0	0	0	0	0	0	0	0	0	0	0	0	0	0	0	0	0	0	0	0
LOC107819346	0	0	0	0	0	0	0	0	0	0	0	0	0	0	0	0.051427	0	0.051451	0	0	0	0	0	0	0	0	0	0	0	0
LOC107767520	0	0	0	0	0	0	0	0	0	0	0	0	0	0	0	0	0	0	0	0	0	0	0	0	0	0	0	0	0	0
LOC107831816	4.415235	5.869067	4.044015	1.756088	1.52464	1.042006	2.61937	2.447522	2.001836	1.680161	2.159625	1.728589	1.654351	1.409796	2.365314	7.447173	6.22137	6.567433	0.896055	1.056727	1.178566	2.410221	2.659678	1.688342	0.653526	1.378536	0.458206	0	0.622142	5.326841
LOC107828485	1.530352	1.52152	1.874517	0.630191	1.292603	0.822658	1.633637	1.485879	1.567376	1.017468	0.634095	0.587674	1.161551	0.986545	0.71396	1.14042	1.276842	1.296525	1.129378	0.764974	0.761294	1.568499	1.288514	1.890345	0.703574	0.387159	0.575512	0	0.334894	0
LOC107792941	15.44215	14.35508	11.8664	35.59006	40.88337	42.03676	29.63416	36.20486	32.64897	16.26269	14.55289	19.82985	9.007776	19.27254	11.98214	15.74227	17.48438	12.5259	82.73561	66.56902	86.58534	25.94703	39.55537	45.1278	13.74459	17.61903	21.09859	22.08664	18.13993	7.638489
LOC107809295	0.173875	0.237698	0.151146	0.177212	0.269248	0.210304	0.291672	0.092852	0.163241	0.148356	0.137161	0.018362	0	0.118555	0.111538	0.22675	0.104986	0.372689	0.2426	0.183858	0	0.435622	0.325909	0.318034	0.302267	0.030242	0.128441	0	0	0
LOC107771441	0	0	0	0.088368	0	0.104869	0	0	0	0	0.205188	0.082406	0	0	0	0	0	0	0	0	0	0	0	0	0.123322	0.135722	0	0	0.469599	0
LOC107802584	0	0	0	0	0	0	0	0	0	0	0	0	0	0	0	0	0	0	0	0	0	0	0	0	0	0	0	0	0	0
LOC107773105	19.82705	18.17346	18.07477	21.51359	16.13671	21.74405	13.67551	13.88269	17.92113	16.95176	15.09781	21.88596	18.15069	18.53143	10.78722	12.02332	19.15459	14.44306	12.16371	13.16795	14.71478	10.43776	13.68032	21.55453	9.409259	11.62011	22.35964	24.37675	6.837712	0
LOC107771125	6.96728	6.825102	5.421845	22.39438	40.35008	12.85077	31.16088	35.09438	18.25266	25.04008	19.58573	21.07736	29.44521	35.09186	33.22872	9.233722	6.670521	6.687405	48.21936	38.0982	55.24796	26.79324	17.60881	11.73001	92.65022	66.49704	41.71623	99.95017	93.19722	122.5322
LOC107774974	11.53014	11.53768	12.27294	16.07362	12.70122	15.63453	15.86804	17.7732	16.96653	13.30844	12.5399	16.46027	14.14525	13.60565	14.00785	9.293681	10.27523	9.508456	9.885294	11.06747	13.34847	16.11403	16.85371	16.42911	7.735052	5.697024	12.07024	10.09675	7.476881	14.5495
LOC107829641	0	0	0	0	0	0	0	0	0	0	0	0	0	0	0	0.336154	0.205079	0.395657	0.242331	0.538719	0.338803	0.421039	0.304271	0.33281	0.100644	0.073843	0.376344	0	0.127748	0
Novel02522	1.336438	1.689482	4.871802	0.429619	0.381474	0.092699	0.976292	0.515692	1.416605	0.840772	2.297434	0.910532	6.814817	4.185838	3.141598	8.224334	1.332761	5.271127	0.043746	0.218813	0.432497	0.936079	1.02672	1.486964	0.163516	0	0.305722	0	0.415103	0
LOC107763968	0	0	0	0	0	0	0	0	0	0	0	0	0	0	0	0	0	0	0	0	0	0	0	0	0	0	0	0	0	0
LOC107774866	0	0	0.019508	0	0	0	0	0	0	0	0	0	0	0	0	0	0	0	0	0	0	0	0	0.023456	0	0	0	0	0	0
LOC107800983	0.534487	0.584543	0.74339	0.290531	0.614834	0.804495	0.986255	1.050365	0.70252	0.364835	0.299826	0.316085	1.011498	0.932959	0.32915	0.477961	0.567996	0.996208	0.27118	0.22607	0.536208	0.446364	0.660034	0.614511	1.419084	1.41303	0.631721	7.644681	1.029285	13.21926
LOC107796260	0	0	0	0	0	0	0	0	0	0	0	0	0	0	0	0	0	0	0	0	0	0	0	0	0	0	0	0	0	0
LOC107794508	7.937768	7.754126	7.18936	9.817947	8.075335	9.964933	5.621212	6.619795	5.39959	4.495878	7.132438	6.746203	6.084969	6.533677	4.585797	8.18215	5.234819	7.299977	7.282991	8.36335	6.892581	5.239718	6.330821	6.110635	3.06481	2.18251	2.865102	10.19753	6.636179	5.877894
LOC107768960	15.13967	17.63951	16.74067	19.81167	17.67389	16.04832	20.38538	19.23014	17.3724	14.2163	16.58148	12.98643	14.85191	14.38613	19.21033	18.06864	15.33602	16.02993	17.28547	18.43374	14.37193	20.89493	18.01024	15.29853	10.82953	15.38902	8.798389	6.913794	9.68113	14.34649
LOC107821901	0	0	0	0	0	0	0	0	0	0	0	0	0	0	0.02524	0	0	0	0	0	0	0	0	0	0	0	0	0	0	0
LOC107803999	0	0	0	0	0	0	0	0	0	0	0	0	0	0	0	0	0	0	0	0	0	0	0	0	0	0	0	0	0	0
LOC107804390	9.117452	10.96845	10.14478	12.10083	10.4477	8.724114	22.82689	20.48822	14.08098	12.53584	16.43043	11.47699	7.764501	8.007087	15.90843	12.12789	7.883362	8.938643	17.6246	20.20737	16.58896	27.79191	22.43773	13.86551	5.360162	12.62284	7.758788	3.260134	4.389466	2.818729
LOC107761486	3.4856	2.792699	3.379569	3.470085	2.525049	3.520952	2.232801	2.274553	2.229571	1.269919	1.786077	1.148769	1.85483	1.483439	1.533225	3.025628	2.368269	3.112684	2.779029	3.434607	1.309997	2.766969	2.838028	2.602249	1.259102	0.85274	1.856128	0	1.383043	0
LOC107769414	0.077256	0.211229	0	3.499514	9.023711	2.408739	13.41322	22.50936	9.50164	10.99886	10.56357	10.83453	8.600277	18.96363	28.62504	1.439287	0.149272	0.057598	33.00402	29.40908	30.86106	29.42055	18.87621	9.528293	92.98701	159.9548	36.34166	104.9734	65.83325	248.3974
LOC107832783	0	0	0	0	0	0	0	0	0	0	0	0	0	0	0	0	0	0	0	0	0	0	0	0	0	0	0	0	0	0
LOC107784190	8.168814	15.29216	14.05802	13.851	13.13793	19.93859	0.462942	1.461955	0.362734	9.257348	7.469592	11.09794	9.339412	5.058034	3.965522	8.678672	6.718662	9.587946	20.10679	30.16233	9.765666	1.521119	1.26582	2.365268	8.164596	2.687978	2.805134	0	18.06943	0
LOC107774179	24.95519	24.94116	26.44074	25.95067	21.95346	21.52013	23.4895	22.11671	24.19273	14.10776	14.89112	14.01432	12.48342	13.5966	16.66804	24.63676	21.33027	26.51072	17.56972	15.92494	17.77842	23.63082	25.44592	26.4779	4.885072	6.791059	7.176323	4.155179	5.874289	14.37036
LOC107815623	4.913205	3.718835	3.185111	8.147803	19.56387	7.431027	9.045063	11.06416	7.3491	14.90081	12.43755	19.76912	17.75253	23.80232	23.58995	3.816467	4.062741	3.445706	27.96995	22.61273	31.86014	12.30944	7.859552	6.658457	39.74489	52.95287	15.64941	95.28505	54.9252	144.1719
LOC107794709	331.4422	283.1232	352.848	179.0964	157.3811	271.5748	139.9438	140.4573	191.313	106.0212	114.5974	133.8533	75.81807	106.2307	73.83385	200.2121	275.7415	246.6454	109.4252	108.7496	137.1396	130.2032	183.9049	216.5592	38.47213	19.31484	88.5795	63.57261	35.22546	16.91237
LOC107799521	0.799691	0.835999	1.042732	1.662041	1.331989	1.112638	1.459836	1.185688	1.457406	1.192635	1.253427	1.212108	0.91641	0.949402	1.255312	1.086697	0.999795	1.017053	1.312674	1.273381	0.793684	1.414255	1.431507	1.007921	0.535263	0.81817	0.63938	0	0.679411	2.908594
LOC107820509	3.225528	4.196486	3.515012	3.557418	3.57804	3.417578	4.352147	3.295191	3.379371	3.555553	4.589041	3.455635	3.850898	3.467894	3.62246	3.762261	3.793551	4.164779	3.178172	3.756733	2.21696	3.298834	3.174999	3.39575	1.773064	1.463506	2.292913	1.671528	2.138028	2.890424
LOC107784698	3.797143	5.214644	4.612042	2.309054	2.20929	2.740236	2.355899	2.266623	2.588233	1.217324	2.443718	1.713826	1.215996	1.815889	1.227911	4.38012	3.718611	4.769925	1.68902	1.892071	2.277079	2.519499	3.119925	2.718337	1.315272	0.434256	1.229566	0	1.001687	0
LOC107780976	0.236584	0	0.123395	0.042867	0.041869	0	0.039687	0.202144	0.222116	0.04614	0	0	0	0.361343	0	0.070521	0.091424	0	0.096028	0	0	0.158062	0.041737	0.049456	0	0	0	0	0	0
LOC107784216	6.59193	6.137395	7.873906	1.439284	1.237933	3.594552	0.437542	0.425463	1.291187	0.716787	0.814716	1.021659	1.108653	1.836671	0.535421	4.258517	5.795665	5.745443	1.154941	1.223581	0.583895	0.47526	1.003946	0.867431	0.089937	0.197961	0.392359	0	0.228316	0
LOC107759302	4.775131	7.070051	4.901769	6.954125	5.999131	5.238979	7.477113	6.795361	5.370543	5.975391	7.462307	4.750124	4.698898	5.191653	6.657417	7.129011	4.329774	6.148094	6.433068	8.230515	3.968549	7.798738	6.583916	5.43385	3.070121	5.034207	3.255309	1.165493	4.943066	1.007691
LOC107764535	132.7944	122.074	130.3491	80.01522	62.71753	82.23072	66.92472	63.95867	77.56648	82.62024	72.31492	63.60448	63.96482	65.50879	55.08919	102.3155	105.4196	108.9364	46.39195	51.89056	43.88134	56.91084	70.67743	78.84014	102.2745	21.338	122.5146	34.27167	162.4255	29.63147
LOC107767151	0.161916	0.319727	0.351875	0.024448	0	0.145066	0	0	0	0.078944	0	0	0	0	0	0.462529	0.078212	0.301788	0.054767	0.251111	0	0	0	0	0.034118	0.037549	0	0	0	0
LOC107822099	11.72105	9.821444	12.61477	13.73037	11.70124	14.07866	11.99159	10.78515	14.55139	8.791922	10.68746	11.82468	10.84946	12.45921	9.7387	9.630386	9.664392	10.97919	9.410405	8.53506	11.47293	10.90007	14.05008	15.30232	7.002368	4.361009	7.611721	3.070405	8.681428	2.654688
LOC107786725	18.52511	18.14961	19.00212	19.34219	14.42517	22.60553	11.84758	11.19857	13.60939	12.64503	10.61918	13.42033	12.06411	13.84384	10.64766	14.70226	19.32856	15.01974	12.2657	13.16362	11.44651	10.67486	13.56251	10.84297	4.215848	4.44642	9.086506	3.312019	4.905257	5.727179
LOC107773934	14.87123	17.39339	13.07088	12.15027	15.88832	11.42874	14.34411	14.49468	11.6348	19.81847	14.425	21.31163	23.26877	19.94924	18.4857	17.36189	19.1026	15.29613	19.81299	15.63186	24.41305	12.71833	10.88259	16.29179	17.23494	18.96787	15.75383	21.66395	30.36174	15.32517
LOC107815457	29.56344	30.92404	31.73399	14.08542	13.38268	15.5242	12.82181	11.58553	12.97499	11.85543	11.26774	15.47577	10.95805	13.83256	8.430498	28.49175	29.15906	33.00044	13.42841	14.08976	18.07399	13.63758	12.76532	13.4228	9.931531	10.52195	14.36959	32.6338	10.98459	16.12306
LOC107794897	0.894289	1.047901	1.184588	0.81018	0.489868	1.373528	0.678642	0.473017	0.239885	0.415259	0.477772	0.287817	0.474064	0.232292	0.305958	0.920301	0.699395	0.761977	0.432125	0.648434	0.388384	0.746844	0.63857	0.84569	0.215362	0	0.150997	0	0.615061	2.633105
LOC107807175	2.412364	2.963293	2.078782	5.83428	6.16255	5.638231	8.128603	8.119293	6.282451	9.22534	8.060608	8.293904	7.309168	6.957028	8.267634	3.048268	2.715593	2.736878	8.578294	9.156505	5.892486	7.673105	4.14471	5.525159	6.656872	12.2298	8.008209	3.000315	7.978298	9.07931
LOC107780714	1.21971	1.173372	0.824653	0.859441	0.77948	1.602746	0.397842	0.752666	1.654058	0.330381	0.237573	0.171741	0.528033	0.665322	0.625938	0.807937	0.589171	0.505192	0.412559	0.229287	0.370799	1.471328	1.016094	1.133191	0	0	0	0	0	0
LOC107801865	10.01241	8.364658	9.106523	6.929038	5.742361	5.133105	7.192597	6.89143	8.964906	3.887267	3.997905	3.485325	3.612117	4.096145	2.664269	7.910321	7.120281	7.810267	4.98588	3.92121	5.284427	8.864875	8.749838	11.82154	2.871658	2.321924	3.999409	0	3.347453	0
LOC107823819	0.025614	0	0	0	0	0	0.021483	0.021885	0	0	0.01796	0	0	0	0	3.397533	2.573466	2.788017	0.441845	0.845026	0.49056	4.085598	3.366337	2.382646	0.259067	0.534591	0.333005	0	0.123313	0
LOC107773490	0	0.065215	0	0	0	0	0	0	0	0.069777	0.150528	0	0.079658	0	0	0	0	0	0	0.181597	0	0	0	0	0	0	0.084575	0	0	0
LOC107809465	1.248967	1.103256	0.935373	1.566694	1.343205	0.640409	1.369889	1.641771	1.100426	1.854956	2.142284	1.785659	1.946515	1.781812	1.774946	1.675317	1.503618	1.676084	1.403851	1.479156	1.174128	1.043045	1.576238	1.626754	1.603368	1.336805	0.953839	0	2.960233	1.188085
LOC107803215	0	0	0	0	0	0	0	0	0	0	0	0	0	0	0	0	0	0	0	0	0	0	0	0	0	0	0	0	0	0
LOC107812132	15.08882	7.612492	12.32004	4.491475	5.531369	5.561941	0.316391	0.552527	0.860082	0.472934	0.529015	0.728426	0.179969	0.529109	0.05531	5.782735	10.30807	10.04407	3.116901	3.14544	4.81644	1.440122	2.091467	3.041517	0.476921	0.899785	0.764304	19.269	2.334952	0
LOC107806359	3.296127	3.167275	4.266736	1.622894	1.14129	1.925946	1.051751	0.826523	1.715457	0.628854	0.753667	1.180454	1.794764	1.446182	0.404496	2.990251	3.703521	3.792948	1.308789	1.272918	1.274335	1.406261	1.58011	1.90979	0.407671	0.448662	1.016284	0	0	0
LOC107830760	2.263871	2.42734	1.890761	0.84451	0.392789	2.33849	0.223389	0.455132	0.625124	2.510559	0.809277	2.850121	10.92075	3.050895	0.911206	1.223931	2.615931	1.389963	0.450435	0.262854	0.242905	0.444851	1.213792	0.649544	0.448976	0.185295	0.367255	0	0.641122	0
LOC107794271	0.256484	0.155836	0.198183	0.542177	0.428691	1.041729	0.334638	0.462644	0.722396	0.333473	0.479592	0.385218	0.158623	0.341992	0.087749	0.148658	0.16519	0.212466	0.318098	0.482151	0.649772	0.309395	0.351923	0.446795	0.03603	0.079306	0.067365	0	0	0
LOC107831520	5.709117	5.253672	6.552842	3.866574	2.746611	5.124931	4.463051	5.209541	4.787546	1.459339	2.409715	2.107232	2.468143	2.418783	1.706703	3.882693	5.783229	4.793603	1.51861	2.719536	1.718753	3.88833	5.818082	4.924361	0.140156	0.308498	0.655118	0	0	0
LOC107788859	6.580614	7.183721	6.369956	5.918028	5.37504	6.893556	6.489896	5.787422	5.544512	3.102691	5.763688	3.176849	5.125259	4.707193	4.873883	6.879804	6.869747	6.271936	3.375466	4.098594	2.110464	5.817732	5.10289	6.298586	1.493315	0.939123	1.908827	1.723835	1.856787	0
LOC107786646	0.939223	0.823064	1.318876	0.818162	0.479473	0.543728	0.302987	0.555575	0.169574	0.317029	0.126651	0.244148	0.201069	0.236457	0.11123	1.130624	1.116761	1.265802	0.256593	0.275026	0.23062	0.754202	0.701002	0.679622	0.045672	0	0	0	0.173914	0
LOC107782366	0	0	0	0	0	0	0	0	0	0	0	0	0	0	0	0	0	0	0	0	0	0	0	0	0	0	0	0	0	0
LOC107810013	0	0	0	0	0	0	0	0	0	0	0	0	0	0	0	0	0	0	0	0	0	0	0	0	0	0	0	0	0	0
LOC107773792	5.133757	8.23185	5.603498	9.092031	9.461083	6.473902	9.421187	7.02487	5.145949	6.511644	10.85351	2.217485	7.004061	6.274442	10.26097	10.93052	5.742742	7.108091	7.285301	7.444809	2.112213	12.12059	6.775446	4.51857	4.636164	7.358083	1.916112	2.760423	2.415824	19.09341
LOC107762018	54.46989	61.07192	57.14976	68.74146	61.42511	63.46463	84.99765	76.68367	67.90747	51.07688	63.59798	49.01417	61.76476	60.87502	70.7931	39.31665	43.19939	42.07345	42.67534	43.07736	41.64819	56.54416	52.79478	47.79632	41.80036	38.51234	32.11465	45.29496	39.33561	62.65963
LOC107817424	2.147947	3.606085	2.800747	1.655748	1.350458	1.215424	4.36171	3.38075	1.450528	0.955395	2.840531	0.923241	1.92968	1.068876	3.867709	4.380734	2.220718	2.654929	1.376582	1.801077	1.976148	3.115559	3.058001	0.945277	1.143435	1.468139	0.445386	1.347444	1.088524	4.660028
LOC107798582	0	0	0	0	0	0	0	0	0	0	0	0	0	0	0	0	0	0	0	0	0	0	0	0	0	0	0	0	0	0
LOC107788512	49.21891	50.64781	47.13074	76.67349	68.83863	81.14303	46.23262	49.16402	45.72439	84.82411	72.73372	76.07835	51.52382	60.55689	71.30636	40.49962	48.017	38.49827	75.04684	78.33317	67.92103	38.86833	39.06152	31.21676	58.10906	69.56161	67.09313	50.74486	41.3045	65.81142
LOC107767761	9.248874	8.804767	8.477664	8.544932	7.495259	7.630044	8.75591	7.980731	8.425332	5.670248	6.035839	6.227756	6.422242	6.393706	5.216336	9.690125	8.404387	8.465674	7.991289	6.468283	8.059306	7.953401	8.036958	8.805512	2.199746	3.312846	2.976378	0	4.188218	0
LOC107779011	0	0	0	0	0	0	0	0	0	0	0	0	0	0	0	0	0	0	0	0	0	0	0	0	0	0	0	0	0	0
LOC107802751	5.364192	7.253318	5.181087	10.70402	9.369087	7.953408	8.674888	12.1323	11.38892	8.58119	9.784492	6.042425	12.80163	8.950225	9.427993	7.550649	7.316133	5.959642	8.894137	11.86337	5.243963	10.86273	8.226133	7.072444	10.10647	11.22022	9.323594	2.507292	12.99696	4.335635
LOC107794026	0	0	0	0	0	0	0	0	0	0	0	0	0	0	0	0	0	0	0	0	0	0	0	0	0	0	0	0	0	0
LOC107785578	0.969996	0.701128	0.814121	1.030278	0.680733	0.86306	0.476924	0.371527	0.910675	0.554474	0.6098	0.932511	0.335114	0.693315	0.823926	0.697916	0.840156	0.698236	1.289752	0.70737	0.762631	0.726268	0.649076	0.41952	0.422886	0.232703	0.158132	0	0.483094	0
LOC107786853	19.82305	23.33456	22.49373	13.63575	9.844048	14.07133	10.90896	10.29754	11.00774	11.56608	11.70119	10.94203	9.531131	9.281025	12.42579	19.48625	21.46885	20.89785	9.850018	11.46377	8.604291	11.29458	9.644543	10.34528	3.998947	5.425411	5.575325	3.899941	3.41309	1.685955
LOC107812080	0	0	0	0	0	0	0	0	0	0	0	0	0	0	0	0.055235	0	0	0	0.067569	0	0.06535	0	0	0	0	0	0	0	0
Novel01369	1.337539	0.668485	0.937657	2.110778	2.176191	2.458547	1.411362	1.511485	1.45828	1.851221	1.724495	1.567406	2.113373	1.647472	0.797118	0.868125	0.666929	0.707685	1.225896	1.204464	1.574011	1.765603	1.636494	1.713672	0.436402	0.780456	0.509956	0	0.415445	0
LOC107806922	5.318848	4.365561	4.272062	9.355423	10.76157	7.357021	11.37462	10.01931	8.497827	16.37857	16.1397	14.74187	15.78937	14.18414	14.58958	4.844414	4.988207	4.266892	12.46721	11.05111	10.66626	10.9619	9.355714	8.582714	19.62337	15.71045	18.7123	13.34641	19.91642	13.46261
LOC107788792	5.179123	5.542579	5.286558	2.203829	2.81219	2.868462	2.435246	2.648405	2.283849	1.759952	1.953363	1.756816	1.222979	1.455346	1.57055	4.61969	3.866316	4.621806	1.950874	2.456117	2.397507	2.588579	3.149378	2.419544	1.934632	1.47403	1.715821	0	1.700055	4.852014
LOC107782223	9.448898	7.633871	7.162211	8.456839	7.876311	7.582234	9.322636	8.404911	8.571055	9.970563	10.22643	10.21939	11.30627	10.10909	10.354	8.861191	8.246456	9.018391	6.276261	7.761433	6.161343	7.528873	6.784407	6.39315	3.029854	5.017627	3.938416	0	6.263165	4.23362
LOC107771954	0	0	0	0	0	0	0	0	0	0	0	0	0	0	0	0	0	0	0	0	0	0	0	0	0	0	0	0	0	0
LOC107827073	14.55861	13.05494	11.60369	3.748183	4.662536	5.07755	5.008773	3.334925	6.266147	0.951507	0.711585	0.560568	6.213348	5.280048	2.643991	6.660702	6.6742	7.827727	1.703062	2.93857	1.174702	1.98835	1.996826	2.488514	0	0.054309	0.046132	0	0.939549	0
LOC107792436	6.951333	8.209099	6.553292	10.11157	10.48268	10.94715	12.07135	11.87881	10.11104	12.02932	10.84699	12.24164	10.68106	11.21513	9.546373	8.24445	10.59358	8.613192	7.053732	11.0153	11.51867	10.8746	11.1404	7.470194	10.39778	6.402772	9.835956	7.00167	8.484386	6.053681
LOC107760171	0.306651	0.279474	0.299886	0.295173	0.559648	0.22666	0.578702	0.49127	0.467833	0.467224	0.7929	0.874351	0.384039	0.439085	1.121253	0.614138	0.42586	0.400087	0.19448	0.324257	0.29715	0.912328	0.862175	0.761214	0.072694	0.346679	0.113261	0	0.184541	2.370084
LOC107808732	0.052093	0.023738	0.022642	0.401148	0.368764	0.112013	0.546161	0.378334	1.027056	0.20319	0.310486	0.242054	0.260959	0.397818	0.106934	0.03882	0.025163	0	0.02643	0.154236	0.071265	0.130513	0.275698	0.680598	0	0	0	0	0	0
LOC107775301	4.42318	4.667686	4.603853	1.898136	1.905461	3.691738	2.294287	2.237734	2.34954	1.589059	1.428339	2.458438	1.03662	2.158764	0.657081	3.469641	4.04826	3.51462	2.539454	2.117018	1.433137	2.284394	3.542185	2.737366	0.588657	0.161961	1.100598	0	2.521748	0
LOC107777825	2.97125	2.484593	2.742608	2.192292	2.439424	3.029794	2.132428	2.250821	2.616991	2.538909	3.17886	2.484316	3.068941	3.241487	1.949235	2.853323	2.544959	2.831793	1.523051	2.150703	1.257137	1.918584	2.026422	3.169581	3.175651	1.1934	2.896312	0	3.539296	9.46994
LOC107820464	10.10268	10.35364	10.65132	7.682233	6.462301	8.985916	5.853218	5.373336	6.285136	4.589402	5.377017	4.558869	7.633129	6.683738	5.663452	7.861115	9.407266	9.861143	4.322923	5.319907	3.700327	4.5404	5.296636	5.258456	2.257051	2.032358	2.397702	2.901544	2.929994	5.017381
LOC107815979	2.801562	4.217212	4.514953	4.306169	3.983122	4.636483	3.432278	2.582001	4.166993	3.591343	3.686116	3.829489	3.609326	4.086602	2.746213	2.861828	5.017751	3.98962	2.683122	2.902497	3.67471	2.339657	3.831691	4.14553	0.915355	0.919793	1.227756	2.251138	3.182495	1.946346
LOC107829719	0	0	0	0	0	0	0	0	0	0	0	0	0	0	0	0	0	0	0	0	0	0	0	0	0	0	0	0	0	0
LOC107783256	0.232799	0.238688	0.278255	0.052726	0	0	3.319394	2.61069	0.846927	0.397265	3.407609	0.122922	2.980283	2.539722	2.688058	0.108426	0.140565	0.130171	0.029529	0	0.02654	3.30508	0.51336	0	0	0	0	0	0	0
LOC107769411	21.48426	16.40351	14.4889	4.851559	3.981589	7.120243	3.348807	3.14068	4.045973	3.893338	4.177253	5.059652	4.973801	5.081749	3.122245	13.2709	26.57207	19.49023	4.983945	4.771399	6.560235	2.619859	2.878916	4.371836	1.722704	2.469103	3.333255	0	2.135782	5.877894
LOC107822821	0	0	0.05546	0.0578	0.056454	0	0.187291	0.109025	0.089847	0.031107	0.13421	0.08085	0.106534	0.139205	0.229189	0.023772	0.030818	0.047566	0.194219	0.05397	0.145467	0.106562	0.028138	0	0.32265	0.75457	0.150813	2.281299	0	0
LOC107821984	0.451196	0.485975	0.17828	0	0	0	0.034403	0	0	0	0	0	0.045662	0	0	0.0917	0.11888	0.030581	0	0	0	0.13702	0.03618	0.042872	0	0	0	0	0	0
LOC107779000	82.4896	85.68968	79.78085	33.42841	27.07046	48.95465	31.02005	29.70702	35.4689	35.89785	49.89449	36.5486	37.78759	34.05693	38.60074	55.96772	71.92577	70.66936	18.36086	24.19731	17.17588	27.61262	33.64959	36.42893	5.027848	5.968153	14.26854	10.15698	4.51289	7.025422
LOC107817595	0	0	0	0.051524	0.050325	0	0.047702	0	0	0	0.199396	0	0	0.062045	0	0	0	0	0.173131	0.192442	0	0	0	0.059443	0.215713	0.791342	0.067219	0	0	3.516525
LOC107828507	0	0	0	0	0	0	0	0	0	0	0	0	0	0	0	0	0	0	0	0	0	0	0	0	0	0	0	0	0	0
Novel02276	0.623569	1.633879	1.490651	0.776767	0.275887	0.251405	0.130753	0.332996	0.365896	0	0.054656	0	0.086771	0	0.080002	0.929368	0.753025	0.987906	0.158189	0.065937	0.213264	0.13019	0.550029	0.570284	0	0	0	0	0	0
LOC107829468	1.491451	1.14179	0.829752	0.75666	1.002998	0.897956	0.800604	0.764602	1.624278	1.745224	0.92031	1.108817	0.996182	1.431848	0.796008	1.378175	1.210328	1.11194	0.181611	0.3028	1.088183	1.295372	1.947027	1.49651	0.226278	0.332039	0.634599	0	1.14886	0
LOC107786942	0	0	0	0	0	0	0	0	0	0	0	0	0	0	0	0	0	0	0	0	0	0	0	0	0	0	0	0	0	0
LOC107778212	10.71797	11.28059	9.61745	15.254	13.36933	14.38533	11.68651	11.10743	12.075	13.82279	13.81873	14.69221	14.70256	13.01293	12.49104	9.45563	9.41922	9.923937	13.92811	13.77548	14.28407	10.85652	12.7171	11.88902	6.731675	8.996072	11.68705	0	8.489053	2.137769
LOC107819590	0	0	0	0	0	0	0	0	0	0	0	0	0	0	0	0	0	0	0	0	0	0	0	0	0	0	0	0	0	0
LOC107776088	2.301362	2.305415	2.727989	1.119997	0.538551	2.228866	0.478575	0.40627	0.482122	0.241104	0.400095	0.17675	0.232901	0.394238	0.195211	1.502377	1.635327	2.311317	0.540391	0.884906	0.31223	0.714766	0.872377	1.073477	0.096186	0	0.314714	0	0	0
LOC107810239	3.902806	2.134156	2.071288	1.712045	1.345032	1.634229	1.378295	1.22856	1.619929	5.608442	1.267501	1.249465	1.966529	2.868392	0.758981	1.408266	2.500396	1.62331	1.333995	1.598624	1.011626	1.372352	1.775622	2.018136	44.04541	2.286513	57.00453	17.62773	34.21666	12.70086
LOC107806706	17.54094	15.69104	17.32564	13.83599	13.19138	17.02941	10.9748	12.39148	14.07214	9.243612	8.408122	9.103635	9.380089	11.7116	8.232566	13.31304	19.33334	15.97692	10.81114	10.72616	11.97052	11.36727	14.04296	15.53926	5.377814	4.791201	7.229859	5.794112	9.556509	10.01924
LOC107810364	6.593846	6.132434	6.640784	5.07229	4.984091	5.221706	7.015727	5.850098	8.043022	3.535586	5.002012	3.875245	4.449273	4.341894	4.154113	7.804179	7.575825	8.247803	3.679183	3.181281	3.06068	7.407981	8.880503	10.52291	2.025523	1.947604	2.631019	2.412037	1.136654	3.12819
LOC107767714	0	0	0	0	0	0	0	0	0	0	0	0	0	0	0	0	0	0	0	0	0	0	0	0	0	0	0	0	0	0
LOC107827658	4.541976	4.675495	4.374312	7.489528	5.956273	6.288411	6.85171	7.789494	6.503682	8.443777	8.042284	8.585361	8.948366	9.44672	7.344786	4.602198	6.976494	4.458137	7.394107	7.463264	6.973466	4.85738	7.061504	7.240443	5.866375	6.729025	5.329629	11.68401	6.135258	16.16329
Novel01290	0	0	0	0	0	0	0	0	0	0	0.005467	0.003931	0	0	0	0	0	0	0	0	0	0	0	0	0	0	0	0	0	0
LOC107827574	1.001727	2.666776	1.833221	4.704742	4.711882	5.753334	3.957743	3.175849	3.019388	5.321044	5.563837	3.563312	3.492137	2.760845	6.439433	2.848441	1.782701	2.378063	6.072149	6.846034	3.486073	5.085454	3.534357	2.920591	5.03262	7.556024	2.86643	1.885201	2.284422	0
LOC107815472	0.430047	1.254192	0.822428	1.051923	1.67435	0.832236	0.973888	0.698144	0.645996	0.629026	0.995113	0.21799	0.382988	0.46916	1.500722	0.865274	1.41257	1.025979	0.872765	1.418783	0.39221	2.011205	0.986258	0.854022	0.108742	0.718055	0.254141	0	0.41408	0
LOC107814275	3.137815	3.482643	3.942919	0.478474	0.329887	0.701428	0.208461	0.132724	0.46668	0.030295	0.261414	0.052493	0.069169	0.101679	0.159434	1.319637	1.11051	1.320241	0.06305	0.052562	0.085002	0.051891	0.246632	0.259774	0	0	0.036719	0	0	0
LOC107770685	0	0	0	0	0	0	0	0	0	0	0	0	0	0	0	0	0	0	0	0	0	0	0	0	0	0	0	0	0	0
LOC107772494	0.203812	0.990666	6.259996	0.86167	3.787277	1.314739	2.849103	4.759902	5.676656	3.577369	3.620471	2.066236	4.235218	1.704677	5.159955	21.56713	0.3938	4.963724	18.61332	13.85037	21.31427	11.06358	6.172307	6.177712	6.098443	6.049919	6.10254	68.01882	19.29742	33.60539
LOC107817821	16.89514	14.97869	14.58138	17.84985	15.37829	16.73305	9.237333	6.142395	8.135472	8.662299	12.33909	8.793118	9.026714	8.265246	9.192064	11.1653	14.03048	14.96005	8.891309	8.927457	8.476964	8.853856	10.31228	9.638968	2.172782	1.987095	4.377091	0	2.796763	1.496636
Novel04882	1.687913	1.240585	1.088618	0.789246	1.011778	1.170783	0.411017	0.465225	0.920141	0.849512	0.305436	0.827998	0.666745	0.118802	0.167654	0.811506	0.578623	0.487127	0.386756	0.27636	0.993165	0.818489	0.912522	0.910558	0.826078	0.303046	0.450479	0	0.262136	0
LOC107802887	12.44668	16.62931	14.65476	23.01533	21.73642	18.98097	22.0983	18.21767	16.13612	14.75111	20.21078	9.799468	17.28724	15.30793	24.62772	21.4518	14.91071	18.64074	21.01298	24.35765	12.19762	29.0364	17.74389	15.76533	13.15059	18.3045	7.683546	7.835505	12.41357	13.0976
LOC107798272	0	0.052924	0.05048	0.368264	1.952621	0.499465	3.068502	3.770905	1.253944	1.245778	0.895822	0.588717	2.90903	2.090625	3.754936	0.086549	0.224405	0.216471	1.82671	2.161448	2.701034	2.376321	0.921999	0.606955	1.394961	1.45442	0.549079	8.305751	5.591459	14.36239
LOC107789540	12.50356	11.90876	8.567965	8.87838	8.771435	10.6574	9.636952	9.961528	9.993919	9.55632	11.0976	11.99083	10.72149	10.87575	7.861846	10.57681	10.28389	13.01711	9.544385	8.480776	9.759712	7.149509	9.339843	9.242288	8.473816	6.426209	11.64948	8.055685	7.321204	0
LOC107824097	2.683389	2.934694	1.726134	2.236534	3.846592	1.846382	3.240971	2.705458	2.972755	4.029631	3.462134	3.581863	5.735398	4.566816	2.423658	2.959504	2.333142	3.881127	4.193294	3.404944	2.39835	2.599524	2.745635	2.636397	2.103424	1.045447	1.649206	11.51402	5.167517	0
LOC107828481	35.73707	45.0301	48.64167	87.72087	86.57269	80.96781	80.89895	77.26099	75.57897	82.99993	65.52922	68.1323	74.30015	65.89152	69.68819	52.18791	38.98345	50.16892	113.5027	107.2606	107.1696	83.24794	68.99832	79.52831	73.18913	74.40769	79.77218	94.88497	112.2011	96.30554
LOC107773475	7.140198	11.71334	8.800956	10.06789	6.804126	8.91665	14.81842	12.82728	7.717657	7.676707	14.49379	5.50635	7.092569	6.271631	15.69051	14.17538	9.887179	9.895917	7.003867	9.679674	4.641874	13.97475	10.67452	5.070967	3.14801	6.674326	2.791408	9.165066	2.467981	3.396071
LOC107775425	0.088742	0.020219	0.115712	0	0	0.023852	0	0	0	0	0	0	0.024697	0	0	0.247989	0.064299	0.049621	0.022512	0.018767	0.020233	0	0	0	0	0	0	0	0	0
LOC107762273	15.05452	13.49355	17.08825	5.748513	5.284453	9.497245	4.069818	5.315284	7.008517	3.397037	3.053457	2.417561	1.662039	2.171734	2.043179	9.64247	17.66907	10.48171	3.030006	4.420443	5.106188	5.922545	8.779556	8.972837	0.943809	0.346236	3.382184	0	0	0
LOC107811046	3.718316	2.283748	2.880922	1.464611	1.072895	1.651201	1.627154	1.657581	2.124907	0.630579	0.623481	0.682899	1.979656	1.234587	0.580753	1.32521	3.592206	3.133749	0.492142	0.341896	0.442326	0.607551	0.9982	1.351785	0.204395	0	0.286614	0	0.389159	4.998024
LOC107779172	22.82575	24.41327	24.70677	17.14703	13.85464	21.29546	15.24212	16.01068	16.58893	10.48517	11.7285	11.63405	7.769984	10.77019	9.100027	22.53913	21.14038	22.50257	12.25097	13.61738	15.54142	15.5965	15.2529	16.69366	2.94152	5.599625	6.763131	0	2.421847	7.776037
LOC107763882	2.282343	3.900138	4.067183	1.033843	1.666143	1.104209	1.052864	2.193857	2.35704	2.225572	1.920456	1.590754	1.206849	0.809225	1.112704	3.359089	4.134253	4.551735	0.752688	1.351493	0.572422	2.19195	2.466142	2.683692	1.731345	0.476357	2.630107	0	0.5494	0

LOC107770936	9.318562	13.14539	11.01841	13.68483	11.52538	11.61631	19.3564	18.98639	18.4822	13.0079	16.05962	11.22544	12.5339	14.72462	16.31138	14.04475	12.90417	13.20394	13.03947	13.56207	10.36425	21.56524	23.61011	20.13736	10.23421	13.35533	9.340025	2.500597	8.164539	3.243044
LOC107829725	2.435447	1.833591	1.779577	3.613366	5.215831	4.136319	3.522942	4.88562	4.473597	5.610197	3.860971	5.695491	3.693483	5.429426	4.455945	1.604475	1.977755	1.578895	10.49417	9.196347	8.788103	5.128979	4.763456	3.025121	18.74263	23.81945	9.302987	32.81443	7.81674	15.27698
LOC107809013	9.85972	9.547539	9.84308	9.002191	8.383727	8.861276	10.85408	9.938174	9.401376	5.708593	7.345744	5.531485	5.316489	6.638759	8.380415	9.700963	12.42756	9.590549	6.409429	6.776774	9.413741	10.35697	10.59938	11.43253	3.019033	3.965672	3.732728	5.508667	7.416906	4.762823
LOC107824748	0	0	0	0	0	0	0	0	0	0	0	0	0	0	0	0	0	0	0	0	0	0	0	0	0	0	0	0	0	0
LOC107795867	7.255695	7.165923	8.19185	2.173639	1.585895	2.206589	5.479522	5.705482	6.594852	1.296654	1.621577	1.123392	2.05951	2.270612	1.958188	4.394495	3.60255	4.180993	0.762658	0.733604	0.738187	4.876506	5.074106	2.840092	0.328927	0.241333	0.341659	2.06727	1.809201	0
LOC107814817	0.704209	0.764048	0.408103	0.972157	0.801168	1.009482	0.22501	0.114609	0.503728	0.261597	0.141083	0.198312	0.111991	0.182919	0.034418	0.42482	0.712722	0.500017	0.544444	0.31204	0.489334	0.196035	0.147895	0.315445	0.127191	0	0.039634	0	0.161444	0
Novel01783	0	0	0	0	0	0	0	0	0	0	0	0	0	0	0	0.087814	0.022769	0.079069	0.071745	0.029905	0.021494	0.039364	0.062365	0.098533	0.059594	0.131172	0.027855	0	0.113464	0
LOC107823680	0	0	0.096672	0.10075	0.492028	0.119564	0.093276	0	0.417634	0.32533	0	0	0.371401	0	0.114143	0	0	0	0.225696	0.094076	0	0.092874	0.294283	0	0	0	0	0	0	0
LOC107771044	18.83419	19.46466	19.44683	25.1452	23.68287	24.48779	23.0531	21.21276	22.67403	20.76028	21.95825	18.17658	19.36139	19.1708	19.37714	21.06967	19.65365	20.35708	23.66093	24.73719	18.34826	23.80034	24.60154	26.46709	16.4919	17.64856	15.04447	12.8891	20.49937	25.07396
LOC107809349	0	0	0	0.763838	0.746061	0.226618	0	0	0.197893	0	0.073901	0	0	0	0	0.157076	0	0.078574	2.352777	2.49632	6.632222	0.440078	1.022589	0.660933	0	0	0.373694	0	0	0
LOC107798368	3.106366	4.685909	2.141616	0.58225	0.28435	0.921303	1.976543	1.189797	0.703957	0.313355	1.051538	0.271484	1.192431	0.350575	0.659646	1.436814	0.827865	2.156209	0.652164	0.543677	0.683842	0.268366	0.472417	1.119577	0.135427	0.298088	0	0	1.03139	0
LOC107808683	3.845693	3.245255	3.807282	3.903374	4.348178	4.593989	2.449027	3.25544	2.908463	4.027817	3.795218	4.512428	5.945943	4.46739	4.020208	3.608804	3.508859	4.141406	4.733398	4.759299	3.052682	2.676365	3.015249	3.275162	4.321889	5.598733	3.78775	7.639484	3.257187	13.21028
LOC107793211	0.615603	0.878159	0.977195	0.872929	1.184186	0.805728	1.818388	1.074844	1.130781	0.965685	1.388822	1.062769	2.651803	1.985577	1.37356	0.877605	0.801585	0.538777	0.841948	0.679249	0.878776	1.519965	1.204049	0.699378	0.778309	0.297936	0.316345	0	2.706015	0
LOC107826591	5.213726	8.17729	2.740389	0.549229	0.214579	0	1.322063	0.517994	0.683006	0.472934	0.595141	0	0	0.132277	0.746683	5.963445	6.676818	5.3334	0.123036	0	0.221163	1.113844	2.459851	0.50692	0	0	0	0	0	0
Novel05251	1.436919	1.210739	1.107677	1.670203	2.207098	2.565056	1.205206	1.297237	1.323589	3.119603	3.440941	3.80218	3.470826	3.815499	1.140894	0.929368	0.73338	1.374478	0.412666	0.389888	0.4698	0.747175	0.693514	0.793439	0.377051	0.150896	0.41657	0	0.391576	0
LOC107760966	123.4165	122.4854	123.6449	95.7295	85.68549	94.70091	87.72784	79.42282	75.3341	82.8339	88.76831	84.35405	68.46471	66.23991	77.78209	119.192	122.8196	126.6014	81.3396	88.7241	91.85326	79.73144	79.95311	78.30289	55.89113	59.673	59.71332	67.89248	54.64781	76.56547
LOC107767431	0.141896	0.092372	0.158589	0	0	0	0	0	0	0	0	0	0	0.044229	0	0.045317	0.215416	0.347593	0.020569	0	0	0.067715	0	0	0	0	0	0	0	0
LOC107800860	1.757208	1.737775	1.267503	1.964524	2.381964	2.53235	1.975581	1.916689	2.492167	1.85933	1.677829	1.831984	2.788552	2.24333	2.225658	1.588085	1.372537	1.505191	2.8833	1.359969	1.602604	1.342598	1.714865	2.03202	1.276264	1.612677	0.972156	0	3.959919	9.246879
LOC107819718	147.8253	152.0939	155.2224	135.0083	110.1334	147.9581	113.3909	110.127	133.3882	71.09998	85.09683	69.93563	79.79967	87.59358	84.04912	135.4259	153.1164	152.4698	89.36541	92.9619	80.59992	125.0957	140.0985	145.9928	23.31829	35.15816	38.83824	37.371	24.71432	38.01314
LOC107803579	0	0	0	0	0	0	0	0	0	0	0	0	0	0	0	0	0	0	0	0	0	0	0	0	0	0	0	0	0	0
LOC107777562	8.0255	7.875013	6.860123	15.60776	15.12617	23.92941	4.981186	5.120046	4.018503	15.83436	13.93741	15.91079	8.130029	9.45586	4.85992	9.828139	11.83672	12.02653	16.12457	18.35299	14.71198	3.395834	7.59815	9.087271	12.00689	10.90634	21.43722	5.739367	12.49283	0
LOC107783330	4.078139	1.72765	2.591023	0.497131	0.485562	0.911761	0.449792	0.500826	0.655686	0.304029	0.944453	0.295013	1.582697	0.680283	2.035244	0.306691	0.180726	0.195257	0.126551	0.105499	0.034122	0.166643	0.209011	0.143386	0.047303	0.017353	0.04422	0	0	0
LOC107794837	17.31361	22.93543	16.76736	1.74526	1.510444	1.651687	1.861228	1.416815	1.259173	8.98839	7.831357	2.801795	2.361713	2.979562	2.502847	14.04696	15.10088	15.78052	1.732121	3.032366	1.60127	1.282986	1.548684	1.478279	32.00207	4.207376	40.11958	0	18.66657	1.507784
LOC107781416	8.11358	7.5321	7.905879	11.79498	14.65939	11.76603	11.55303	12.76863	11.44379	14.05718	12.46342	13.13528	9.87235	12.5568	13.40156	8.858458	6.926958	8.131006	21.06142	20.23943	21.82057	11.03047	13.58111	12.93741	33.87536	28.93251	32.51543	8.096312	24.34541	25.66709
LOC107761853	0.453668	0.723559	0.460091	1.113126	1.087221	0.56904	3.202634	2.454967	1.934403	2.138188	3.393198	1.676812	2.209505	1.752876	3.492244	1.591765	0.620899	0.901944	0.74807	0.687591	0.60339	3.157251	1.667355	1.442279	0.740867	1.525511	0.647907	0	1.27407	0
LOC107798641	6.255615	5.500474	5.322989	6.625119	5.964177	9.141066	4.50786	5.608451	5.211295	7.045087	6.4561	6.996933	6.767673	7.737715	4.250251	5.022809	4.766665	5.97758	5.990145	7.751398	5.624871	4.010146	5.362433	6.216053	6.739358	4.168224	9.89289	9.451371	4.453887	19.06731
LOC107823395	0	0	0	0	0	0	0	0	0	0	0	0	0	0	0	0	0	0	0	0	0	0	0	0	0	0	0	0	0	0
LOC107832115	0	0	0	0	0	0	0	0	0	0	0	0	0	0	0	0	0	0	0	0	0	0	0	0	0	0	0	0	0	0
LOC107772895	5.850445	6.081766	5.641912	5.548654	6.875515	6.683065	4.753675	6.248473	6.694173	4.367831	4.615127	6.101044	8.344521	6.482275	3.28384	4.087273	3.797452	4.566212	3.617632	3.866464	5.252334	3.893419	4.273522	6.305973	3.236074	1.780727	4.105636	0	3.520765	5.652216
LOC107828328	0	0	0	0	0	0	0	0	0	0	0	0	0.080874	0	0	0	0	0	0	0.122912	0	0.060671	0	0	0	0	0	0	1.399028	0
LOC107785180	0	0.016928	0	0	0.065743	0	0	0.01587	0.034877	0	0.013024	0.031384	0	0.101319	0.019064	0.041525	0	0.013848	0.037696	0.015713	0	0	0	0	0.046967	0.025845	0	0	0	0
LOC107814071	0.599441	0.962558	1.01735	0.801665	0.6062	0.521716	0.694312	0.658516	0.723577	0.306183	0.360282	0.289386	0.540201	0.373692	0.673845	0.872137	0.910031	0.936381	0.608272	0.482939	0.2343	0.858187	1.007137	0.596701	0.216536	0.238308	0.202426	2.041359	0.27485	0
LOC107814309	0	0	0	0	0	0	0	0	0	0	0	0	0	0	0	0	0	0	0	0	0	0	0	0	0	0	0	0	0	0
107787645	0	0	0	0	0	0	0	0	0	0	0	0	0	0	0	0	0	0	0	0	0	0	0	0	0	0	0	0	0	0
Novel04165	4.849482	3.540647	4.262132	2.548649	2.465628	3.312626	2.179804	2.815768	2.892731	1.175683	1.634475	1.629737	1.431649	1.490706	0.714982	3.573908	3.390806	3.79527	1.413745	1.246564	1.979268	2.394157	3.68674	3.472464	0.338741	0.372801	0.380002	1.916058	0.515959	1.656634
LOC107827381	0	0	0	0.723206	1.141067	0.990293	0.669556	2.465968	1.49893	16.94579	2.325148	11.5169	4.306591	13.33131	24.07592	0	0	0	1.869333	3.168687	1.232083	2.358981	0.595811	0.577639	22.82513	119.5343	10.23344	8.782869	7.982087	3.796858
LOC107776770	0.416373	0.303578	0.337815	0.125738	0.024562	0.119374	0.11641	0.09487	0.130303	0.162407	0	0	0.030901	0	0	0.45508	0.643603	0.227644	0.028167	0.023482	0	0.069545	0.024485	0.029013	0	0	0	0	0	0
Novel00538	0.019856	0	0	0	0	0	0.016654	0	0	0.019362	0.027846	0	0	0.129971	0	0	0.038365	0	0.020148	0	0	0.016582	0	0	0	0	0	0	0	0
LOC107808731	23.04947	20.30472	22.50877	18.03659	14.36611	16.62684	14.21367	17.66899	19.30342	14.15592	12.42295	17.42042	12.46671	13.70416	11.555	14.70378	16.65786	15.1081	15.03149	15.8392	17.72507	11.82664	15.94088	17.52657	11.76149	16.32439	18.27846	50.84923	12.26642	3.66371
LOC107768728	5.03371	4.609768	5.665154	4.251861	3.744074	5.176555	3.161393	2.38941	2.830955	2.750658	2.540659	2.239301	3.383814	2.891675	3.19472	4.222282	4.322655	5.457034	3.18316	3.558762	2.572638	2.558824	2.724098	3.253322	1.137551	1.387274	1.580769	1.739041	2.10731	1.503584
LOC107802070	8.926557	5.02655	6.872848	5.083262	5.585584	4.969948	5.481618	6.483029	7.482719	7.896243	4.761545	6.679529	7.807775	8.764591	6.936923	8.196314	6.344656	7.344408	7.472894	5.178002	6.105878	5.750846	5.708515	5.79795	11.60834	9.049333	13.15041	22.79911	11.05087	17.74101
LOC107798586	0.024742	0	0	0.044831	0.065681	0.053202	0.020753	0.042281	0.069688	0.072381	0	0.292644	0.027544	0.026993	0.076185	0.036876	0.07171	0	0.125535	0.062791	0.045131	0.144641	0.087298	0.051722	0.031282	0.034427	0	0	0.119119	0
LOC107767933	0.70135	0.908328	0.641756	1.003243	1.273864	1.269957	0.216725	0.410015	0.034656	0.323955	0.284719	0.218297	0	0	0	0.467631	0.606241	0.660488	0.187285	0.281034	0.235658	0.154136	0.488398	0.23149	0	0	0.043628	0	0	0
LOC107808483	1.24843	1.038851	0.896497	1.131014	0.768482	1.283857	0.773952	1.298582	0.81536	0.952729	1.217947	1.008851	1.14807	1.065894	2.284153	1.294374	0.73414	1.618708	1.597295	0.964252	1.435612	1.903871	1.388466	0.737529	2.539156	2.265781	1.796314	3.881755	1.567926	0
LOC107800404	3.047672	3.143851	3.362543	2.457625	2.104081	3.078582	2.345536	2.546782	2.452533	1.338972	1.726062	1.032732	1.826844	0.913423	1.323404	2.558139	2.313384	3.033719	2.225968	2.450631	1.114866	3.076603	2.73256	2.222797	1.058567	1.048503	1.029172	0	2.337938	1.035396
LOC107790693	35.45102	33.70496	35.42225	40.1601	45.26862	38.55918	30.11617	26.34709	27.37637	39.29146	33.55069	34.47835	42.5517	39.44296	38.93488	35.03912	33.36896	39.02048	33.03323	30.39178	24.8199	28.63822	23.54844	27.55752	32.23544	33.25935	19.20123	19.24007	31.78339	29.43127
LOC107767891	3.233319	2.799433	1.967459	3.222145	3.647843	3.476213	3.322106	2.969833	3.035582	5.517564	5.271252	3.414497	3.599375	3.262837	6.886073	1.867341	2.030377	1.807932	4.593328	4.71816	2.801399	4.590389	4.420601	3.126004	5.825254	5.061293	3.821522	17.3421	7.783173	4.998024
LOC107809391	6.229563	7.787	6.831134	4.659909	4.298603	4.854181	6.015943	5.786613	7.592433	2.675058	3.646823	2.921155	3.212929	4.707423	4.076814	8.432761	7.315801	7.179651	2.928689	3.65017	2.658301	7.326415	5.217621	5.943653	0.180644	0.477137	1.013235	0	1.100599	0
LOC107775688	3.772504	3.768765	3.563148	3.647734	1.990057	4.211899	4.350722	3.471278	5.585134	2.829744	4.400325	2.604854	2.705514	3.363725	3.387995	3.027526	4.415519	3.028912	1.214678	2.209347	1.190972	4.665188	4.863412	4.435897	0.596199	0.454255	0.98608	0	0.698545	0
LOC107790521	71.14684	82.90276	79.6212	57.85763	48.39387	58.04625	71.59696	67.0722	76.59163	44.38499	51.80321	49.5763	45.52536	49.35142	39.17089	77.23198	90.04403	76.79748	32.26032	39.27448	41.3847	66.14189	81.34837	83.65799	14.69676	15.39499	34.67878	10.01576	15.50808	30.30887
LOC107785242	9.863721	6.509686	7.923858	2.341857	2.467937	2.998572	0.513504	0.58123	0.319327	3.184013	2.289583	2.528679	3.256266	4.526972	2.443689	4.460967	12.28936	7.911699	0.828333	0.920721	1.737135	0.05681	0.360019	0.284402	0.086005	0	0.241203	0	3.274998	0
LOC107807865	2.131082	0.868887	1.998755	0.60968	0.793988	0.422058	1.081866	0.814593	1.421588	0.492176	0.825806	0.521168	0.187291	0.795363	0.575601	0.877626	2.383879	1.46338	0.056907	0.521848	0.869498	1.0772	1.137747	1.406783	0.212711	0.078033	0.530267	0	0	3.46758
LOC107801114	8.734538	8.786539	8.261293	34.11569	45.74144	40.92926	21.70165	22.83498	19.521	53.96954	44.16306	64.417	53.29529	44.6782	39.83456	10.19332	9.79161	10.15703	50.19696	42.2185	45.86739	22.38797	17.97703	22.82334	67.8561	75.36682	53.92254	31.42858	96.40034	18.68165
LOC107819875	0	0	0	0	0	0	0	0	0	0	0	0	0	0	0	0	0	0	0	0	0	0	0	0	0	0	0	0	0	0
Novel00643	1.218134	0.965373	1.519288	0.719718	0.890427	0.626351	0.177687	0.452525	1.093914	0.72303	0.371372	0.492185	0.707501	0.577793	0.217436	1.184028	1.483816	1.855827	0.483683	0.582433	0.869446	0.840375	1.868652	1.937468	0.267842	0.442159	0.375583	0	0.509959	0
LOC107776450	0	0.023965	0	0.095288	0.255944	0.056541	0.176438	0.022467	0.074061	0.076923	0.110629	0.066644	0.087816	0.143433	0.053977	0.07838	0.07621	0.09802	0.080047	0.133463	0.263797	0.087839	0.162358	0.109934	0.232715	0.146351	0.155393	0	0	0
LOC107768469	2.715007	2.957202	2.993295	2.1597	1.669972	1.708662	3.054771	2.319775	3.263922	1.356023	1.555515	0.951052	1.695483	1.336485	1.087457	3.552982	2.590938	2.986859	0.940732	1.316406	0.845508	2.737438	3.913479	3.841309	0.627913	0.23035	0.782663	2.367821	0.956415	0
LOC107809992	4.255222	4.155118	4.623718	6.103775	6.678926	5.99092	5.948397	7.444687	5.374213	8.743335	7.637016	9.543684	9.530338	8.897624	9.462795	4.794245	4.453478	5.702855	6.888088	7.542072	9.240082	6.049662	5.183249	5.612394	14.15425	16.35277	13.77079	18.11361	16.09627	9.396672
LOC107813574	2.231564	2.203278	1.832082	1.067	1.097019	1.599469	1.143822	0.63557	0.931151	0.060446	0.173864	0.523691	0.414035	0.47338	0.699849	2.217285	2.814619	1.571296	1.132218	0.786562	1.13068	1.553031	3.936774	2.656378	0	0	0.146529	0	0.298432	0
LOC107786729	0.142724	0.130075	0.248134	0.732705	0.420972	0.35804	0.199515	0.365842	0.044665	0.185565	0.300235	0.281347	0.476648	0.415213	0.439464	0.248169	0.137884	0.248283	0.627582	0.24147	0.173556	0.357578	0.251784	0.447526	0.661638	0.330984	0	0	0.458083	2.941616
LOC107769083	1.200719	1.48261	1.234555	1.672625	1.61085	1.554656	1.786781	1.378934	1.139409	1.070134	1.8559	0.654452	1.624111	1.225414	1.855204	2.039708	1.409457	1.645989	2.017577	2.151591	0.671176	2.22115	1.639918	0.998595	0.979403	1.526996	0.457791	0	1.491791	1.596608
LOC107767825	5.279976	6.015059	6.206622	3.016798	3.212048	4.547747	3.573029	2.383841	3.351658	1.345668	2.124628	1.672751	2.204154	2.061888	1.416388	5.790187	5.042936	5.010022	1.522089	2.309382	0.793451	3.20687	3.387119	2.57118	0.303432	0.208713	0.886436	0	0.57772	0
LOC107778255	0.149585	0.136328	0.065016	4.58498	4.323853	3.618504	1.484652	0.78816	1.755465	14.708	5.89128	8.403855	8.020731	8.485887	0.409414	0.185785	0.120427	0.074348	1.467291	3.732895	1.182343	0.249845	0.813655	1.120483	10.21255	2.150748	33.5621	5.34873	35.28767	0
LOC107762009	3.316622	4.210923	3.817582	9.200574	11.33427	7.45124	8.53723	8.755503	6.979187	10.59455	10.28083	8.174037	11.48374	13.37513	12.1843	3.238584	2.320237	2.933113	10.0036	10.25508	8.511205	8.328496	3.893915	4.422818	26.66309	30.64889	9.624229	34.35099	33.69672	48.08579
LOC107799035	0	0	0	0	0	0	0	0	0	0	0	0	0	0	0	0	0	0	0	0	0	0	0	0	0	0	0	0	0	0
LOC107805440	16.9558	16.56638	15.70003	21.50027	22.3124	17.94814	18.00259	21.64427	18.23979	19.39844	18.90479	18.89489	22.53397	20.11612	21.25371	17.79371	16.49312	17.02126	26.11824	24.75043	25.67604	19.84376	19.31728	17.35742	25.5556	27.76095	22.92786	33.27834	25.20348	26.97434
LOC107791174	504.8091	165.6779	302.7825	49.01139	41.91071	89.74906	14.96492	12.18356	31.48368	10.13153	8.039118	16.52635	12.28416	53.15697	15.59147	152.396	314.8339	198.3017	23.70362	26.73128	33.91692	27.16777	33.94076	13.70555	2.98959	5.284239	23.62861	15.37302	5.174586	8.861066
LOC107812670	0	0	0	0	0	0	0	0	0	0	0	0	0	0	0	0	0	0	0	0	0	0	0	0	0	0	0	0	0	0
LOC107783815	0	0	0	0	0	0	0	0	0	0	0	0	0	0	0	0	0	0	0	0	0	0	0	0	0	0	0	0	0	0
LOC107761059	8.105276	6.31948	7.49374	4.626495	3.399484	8.008971	4.322583	4.003103	5.234342	4.477222	4.435042	4.987242	5.267708	4.804573	4.375908	5.271961	6.381985	6.846209	3.422986	3.131006	2.221917	4.890844	4.917788	5.680401	1.480857	0.521521	2.159605	0	1.804471	0
LOC107821554	1.149624	0.851289	1.186721	1.106595	0.762947	1.544984	0.662915	0.552527	0.539659	1.541415	1.058029	1.578256	1.43975	1.254184	0.811211	1.713403	1.665951	1.392777	1.239469	0.972503	0.589768	0.24002	0.507022	0.525694	1.180948	0.199952	1.613532	0	0	0
LOC107778256	28.25694	45.5625	27.78638	18.88097	12.36981	15.85425	8.579299	7.5016	10.44349	13.13287	11.71494	19.0834	8.018202	11.11258	9.667384	52.91273	49.98564	44.26242	10.46593	12.47449	13.95433	3.559293	10.67655	13.76478	2.694221	2.846514	5.641799	0	4.514114	2.635248
LOC107831712	0	0	0	0	0	0	0	0	0	0	0	0	0	0	0	0	0	0	0	0	0	0	0	0	0	0	0	0	0	0
LOC107784448	0	0	0	0	0	0	0	0	0	0	0	0	0	0	0	0	0	0	0	0	0	0	0	0	0	0	0	0	0	0
LOC107805976	0.174599	0.716065	0.303551	0.593168	0.27037	0.187715	0.475943	0.484842	0.286862	0.340513	0.428502	0.147506	0.291549	0.09524	0.537612	0.390335	0.168678	0.58577	0.487221	0.664645	0.039809	0.94778	0.539028	0.273737	0.33112	0.182207	0.051591	0	0.210146	0
LOC107808355	5.410757	6.091529	4.624406	3.212991	3.138218	6.11053	2.783959	2.408673	3.671157	1.906516	2.168015	2.957808	2.024649	2.728217	1.960042	0.372715	0.61497	0.27119	0.27683	0.423096	0.995234	0.18986	0.240638	0.095047	0	0	0	0	0	0
LOC107777427	0.106607	0.161932	0.432467	3.798848	8.238435	2.101293	3.189178	7.195971	7.206315	8.004586	5.03338	11.22809	6.408549	9.963326	12.91143	1.350547	0.308975	0.741816	20.95044	22.06465	25.92739	14.69005	6.770503	8.802655	28.43945	66.0097	20.70622	38.11968	28.74177	17.57785
LOC107788882	0.152377	0.138873	0.463605	0.483162	0.438209	0.409561	0.351465	0.325489	0.250353	1.188698	1.602708	1.158595	0.932958	0.748064	0.625584	0.113552	0.073605	0.227208	0.850422	1.192333	0.833825	0.34995	0.168009	0.238897	1.589375	3.44536	2.386298	0	0.1834	0
LOC107802489	0	0	0	0	0	0	0	0	0	0	0	0	0	0	0	0	0	0	0	0	0	0	0	0	0	0	0	0	0	0
LOC107783603	10.73638	9.404236	11.91707	7.500844	6.043639	5.784666	7.64503	6.549467	8.142251	1.892624	4.16902	2.760553	2.105938	2.59988	3.530282	10.85687	9.873806	9.451449	2.717401	3.616268	3.719523	8.309663	9.405182	10.52833	0.155309	0.307666	0.842097	1.756986	0.236562	0
LOC107773369	0.617686	0.675533	0.322165	0	0.054657	0.132818	0.051808	0	0	0	0	0.052184	0	0	0.063398	0.41427	0.358042	0.690767	0	0.104504	0.056334	0.103169	0.108968	0	0	0	0	0	0.297376	0
LOC107827259	0	0.190951	0	0	0	0	0	0.059673	0.032784	0.204308	0.244858	0.118005	0.272113	0.45715	0.143363	0.026022	0	0	0	0.118159	0.12739	0	0.092405	0.036498	0.176597	0.145765	0	2.497262	0.168117	0
LOC107787424	9.890815	12.34726	9.898395	33.19179	48.61429	27.66584	27.96887	28.6055	20.56951	31.10649	24.90932	26.30396	33.32786	27.81927	41.25499	14.5307	11.93215	10.99287	55.04059	46.74242	61.4613	24.04446	18.54841	22.72595	31.79842	28.12607	20.49214	15.45434	43.05619	30.83516
LOC107765345	8.363017	9.418761	8.598066	6.698254	4.550582	5.95296	5.636344	4.436161	7.651038	5.223044	9.412599	4.747222	6.968653	5.879281	5.750487	6.819836	6.698423	6.504473	2.234091	2.64079	1.948011	6.54509	7.057897	7.126767	0.664732	1.097354	1.378769	0	0.553709	0
LOC107775569	0	0	0	0	0	0	0	0	0	0	0	0	0	0	0	0	0	0	0	0	0	0	0	0	0	0	0	0	0	0
LOC107773363	58.22974	39.28927	48.74024	50.30714	54.39245	59.50775	36.00708	43.44742	49.42991	59.85298	45.24827	74.66554	76.44405	80.51181	47.38741	40.50895	44.43	44.15082	46.84991	43.39607	57.12847	35.476	45.7261	39.32023	47.33533	40.4486	57.33466	148.0336	77.12563	22.25924
LOC107762563	0	0	0	0	0	0	0	0	0	0	0	0	0	0	0	0	0	0	0	0	0	0	0	0	0	0	0	0	0	0
LOC107769805	8.425488	10.49196	9.309134	10.54362	8.91864	9.905012	14.71484	13.49638	11.5918	10.05874	14.97419	7.836547	10.95721	8.727908	17.10141	12.02925	9.189527	10.06224	9.076962	12.82929	5.745186	14.33573	11.9465	11.71981	8.501991	8.56798	7.799077	12.3887	4.700793	12.65886
LOC107772483	7.508671	6.705813	8.440704	9.94418	9.205772	7.748533	8.093616	9.043694	9.28605	13.32057	10.02269	10.85279	7.888786	9.80359	9.842283	9.348255	7.661863	9.464947	13.03539	10.79044	12.45847	8.083898	8.378676	11.78785	11.9714	19.21718	16.07416	8.626122	11.17873	11.18729
LOC107803280	2.954173	2.795917	2.864308	3.525552	2.789992	5.497092	1.524795	1.67466	1.866784	1.911228	2.848269	2.03981	3.257005	2.107253	2.44899	2.709481	3.347938	2.753077	2.277092	4.084948	1.39894	1.826608	1.778943	1.989192	0.754177	0.671911	1.678651	2.031396	0.820525	0
LOC107819460	0	0	0	0	0	0	0	0	0	0	0	0	0	0	0	0	0	0	0	0	0	0	0	0	0	0	0	0	0	0
LOC107824877	13.08201	11.92264	12.38325	13.65515	11.96474	13.39766	16.39362	15.08759	18.66568	7.588421	9.11873	8.147063	12.23184	11.67695	12.41867	12.8506	12.41353	14.07082	7.765451	7.742164	7.47457	16.47406	17.81051	17.56454	2.64765	3.05776	3.57518	3.697822	7.841566	20.78152
LOC107820336	0	0	0	0	0	0	0	0	0	0	0	0	0	0	0	0	0	0	0	0	0	0	0	0	0	0	0	0	0	0
LOC107774447	0.192927	0	0	0	0	0	0	0	0	0	0.06764	0	0	0	0	0	0	0	0	0	0	0	0	0	0	0	0	0	0	0
LOC107818686	10.68232	8.076134	8.705541	8.247986	9.291295	11.4848	5.701639	6.638007	8.034619	6.392008	8.724667	7.640243	10.60794	8.541765	7.911548	9.543523	10.26138	10.67916	8.437702	8.728441	8.247871	7.147002	7.816399	9.642632	4.45074	3.715916	6.241082	4.340549	8.181802	0
LOC107772984	0.057308	0.052229	0.099634	0.051918	0.10142	0.061613	0.048067	0.34276	0.107607	0	0.120554	0.048416	0.063796	0	0	0.085412	0	0.042726	0.058152	0.096958	0	0.095719	0.101099	0.059899	0	0	0	0	0	0
Novel03414	0.845484	0.550396	0.909952	1.422508	1.923789	1.125427	0.438994	0.172001	0.302392	0.863714	0.112925	0.442179	0.268916	0.263538	0.578521	0.030003	0.27227	0.060033	0.28598	0.408698	0.036719	0.067246	0	0.126243	0	0	0	0	0	0
LOC107788194	5.74435	5.483384	6.247719	22.00023	19.94648	21.01555	14.88795	15.05004	15.23345	13.32671	12.14109	16.26096	9.455656	12.2663	6.287037	5.518244	6.022944	5.825225	14.58624	14.46283	15.94029	9.34442	12.34306	15.59331	4.61227	4.280528	9.717472	3.89386	6.684468	5.049978
LOC107810979	1.24427	0.62187	1.081618	1.199971	1.243078	1.467202	1.34661	1.57756	1.243542	0.508812	0.337735	0.440823	0.893638	0.875766	0.370767	0.807589	1.046964	0.867807	0.81458	0.611168	0.475882	0.871522	1.168337	1.216607	0	0.111697	0.04744	2.870417	0.386475	7.445332
LOC107797366	250.0571	200.0878	222.6109	70.68649	65.5701	91.07196	76.47173	78.58452	90.85021	35.13749	45.43969	47.25929	68.33821	64.27681	43.54579	129.7687	198.9038	167.9587	62.07136	47.81266	65.7931	67.83005	97.14682	86.18336	26.72839	23.75508	39.4979	72.7358	41.62109	44.91983
LOC107803361	3.483303	2.670225	2.744974	9.024701	12.09857	8.504947	10.83994	9.763142	9.963652	13.36443	10.18086	15.23651	19.67823	19.07159	11.15987	3.444842	5.409422	3.834749	9.051258	9.693602	11.46034	9.406664	6.288582	6.907212	10.20729	11.05243	9.773027	2.328088	15.51604	24.15453
LOC107805689	0	0.015807	0.015077	0	0.061389	0	0	0	0	0	0.012162	0	0	0.018922	0	0	0.016756	0	0.0352	0.044016	0.09491	0	0.091793	0.036256	0.043857	0	0	0	0	0
LOC107806304	0	0	0	0	0	0	0	0	0	0	0	0	0	0	0	0	0	0	0	0	0	0	0	0	0	0	0	0	0	0
LOC107831803	182.8454	117.3499	139.4812	2676.506	2974.452	3398.641	1226.265	1334.65	1760.187	4038.712	2473.81	6111.001	3444.283	3837.728	1498.74	154.1427	196.8498	166.7152	2304.909	1862.526	2757.118	483.6046	724.1629	1570.867	2383.771	1634.635	3622.719	807.1626	2011.854	338.9689
LOC107782772	5.473462	5.626435	5.421867	6.688452	6.504637	5.542511	6.592643	7.205342	7.409328	5.151131	4.88675	5.32313	3.755071	4.756193	2.906891	5.169681	3.135803	4.365398	8.008225	9.58338	9.200169	6.484486	8.055949	6.48586	2.413995	4.870649	2.820869	6.827267	4.596139	3.935261
LOC107768615	3.534569	2.677968	3.516747	3.819421	2.78848	5.768818	3.678956	3.74775	4.357911	2.159348	3.135379	3.633691	4.313983	4.413541	2.316537	2.157947	3.908381	3.079658	3.197724	2.593737	3.573132	2.916247	3.643605	5.786301	1.022971	0.592542	1.207974	3.045442	0.820081	2.633105
LOC107813375	0.180932	0.054966	0.131067	0	0	0.064841	0.025293	0.077297	0	0	0	0	0.067139	0	0	0.921342	1.427495	0.989211	0.305995	0.714261	0.275021	0.982156	1.516141	1.071623	0.038125	0.125877	0.285129	0	0.435535	0
LOC107810462	18.42469	13.83235	17.30485	9.529054	8.05799	13.50944	9.414249	8.685546	10.53781	6.401819	5.692457	7.574108	4.164992	5.621959	3.840082	9.995091	17.868	12.15749	4.297954	4.478741	7.017606	5.541656	8.094762	8.927796	0.714005	0.785796	2.419611	0	1.359433	8.729702
LOC107813965	0	0	0	0	0	0	0.074463	0	0	0	0.062252	0	0	0	0	0	0.085768	0	0	0.075101	0	0	0	0	0	0.12353	0.20986	0	0.427415	0
LOC107800367	0.0776	0	0	2.952657	2.815276	0.750862	3.90517	5.039047	1.602787	1.816067	2.122104	1.638958	1.03662	0.253972	2.150446	0.115655	0	0	6.614392	6.433108	9.129612	4.925217	9.856515	3.974249	1.471643	6.910352	2.292913	27.74736	0.747185	0
LOC107775318	6.666756	8.177738	7.408267	9.750043	10.28546	8.464297	10.48304	8.357531	8.063341	7.754587	10.72494	6.147338	8.188761	7.35986	11.90309	10.69667	7.016848	9.140305	8.432668	9.911374	5.475801	12.51876	9.107206	8.062484	4.172511	5.772333	3.493328	3.79139	4.658087	14.75125
LOC107819751	0	0	0	0	0	0	0	0	0	0	0	0	0	0	0	0	0	0	0	0	0	0	0	0	0	0	0	0	0	0
LOC107774248	55.73744	42.17386	55.6156	33.9585	30.68329	46.4038	25.19244	29.94078	38.34386	14.46583	16.18118	17.43256	12.09692	16.65028	13.15832	39.16994	52.3726	39.96162	16.35427	12.54924	30.73393	28.80559	44.15615	59.72434	4.090756	2.718233	11.83336	8.731687	4.996471	7.549463
LOC107783822	0	0	0	0.150526	0.588093	0.178635	1.567797	1.597114	0.779959	0.202525	0.291267	0.070185	0.231205	0.181265	0.255802	0	0.040129	0.030969	0.295051	0.175693	0.41672	1.422278	1.099185	1.736632	0	0.173392	0.09819	0	0	0
LOC107819562	2.932837	2.868499	2.704908	3.207844	4.161763	3.172385	3.164868	3.025411	2.837424	3.696927	4.28857	3.338909	4.200541	3.472717	3.322215	3.718107	2.833368	3.253167	4.04669	4.160178	2.5117	3.569391	3.296792	3.738294	2.871438	1.169506	2.155918	2.557797	4.304794	1.105742
LOC107795335	17.12818	21.98321	17.25686	21.56758	17.17233	18.35858	24.07537	19.80395	19.37551	23.75075	27.32617	23.94027	31.48734	27.37185	32.14917	25.39127	25.14982	20.58005	13.39414	15.04313	14.85685	24.56209	15.80123	15.30187	4.966796	5.174666	5.633688	1.872947	12.60874	11.33552
LOC107773948	0	0	0	0	0	0	0	0	0	0	0	0	0	0	0	0	0	0	0	0	0	0	0	0	0	0	0	0	0	0
LOC107769915	0	0	0	0	0	0	0	0	0	0.028906	0	0	0	0.032339	0	0.02209	0	0	0	0	0	0	0	0	0.112435	0	0	0	0.285427	0
LOC107795618	19.80618	17.95724	16.23453	21.34123	24.45885	21.98453	21.93426	20.25923	20.81376	26.12712	26.33516	25.67434	39.80743	29.90308	20.22305	2.086634	2.109503	2.183351	6.256168	5.758736	6.93491	4.39797	4.917079	5.531116	8.639318	6.148019	6.011729	3.674246	5.689084	6.353545
LOC107831055	4.050387	6.307683	4.78571	1.068771	1.252677	1.564296	1.319314	0.806391	1.144496	0.460152	0.330889	0.365443	0.9193	0.815113	0.64578	3.428615	3.647081	3.957906	0.279324	0.598779	0.645557	1.806234	1.942444	1.80847	0.099435	0.16415	0.23239	0	0.757282	0
LOC107780140	5.8637	5.236441	6.055051	6.524398	6.651142	6.769605	4.555054	5.010104	5.98541	5.449224	5.298208	6.217206	5.344707	5.881813	3.514065	5.308081	6.235112	5.926649	4.951742	4.294476	5.347793	4.009566	6.074697	7.897433	4.627218	3.121194	5.721086	8.443036	7.76797	0
LOC107829697	1.12382	0.712503	0.722066	1.372253	0.518833	2.521551	0.409824	0.250493	0.458735	0.190585	0.308357	0.247678	0.435148	0.053306	0.05015	0.619001	0.51925	0.546427	0.793304	0.744006	0.311939	0.326446	0.60339	1.225686	0.123552	0	0.115501	3.494304	0	0
LOC107810260	4.807003	8.346543	5.655554	4.918035	4.656907	6.326487	1.320775	1.026809	0.93373	2.666989	2.847638	3.360902	0.7381	1.265842	0.34026	4.045398	3.883319	3.522035	0.672802	0.771213	0.340143	0.034607	0.219315	0.909568	0.209569	0	0.979567	0	0	0
LOC107779336	0.378193	0.215423	0.7397	0	0.209156	0.050825	0	0.080785	0.044383	5.163006	0	0.119816	0.894646	1.392492	0	0.105686	0.365366	0.035245	0.143912	0.039991	0.258689	0.03948	0.166796	0.296466	12.13309	0.263114	22.1262	13.52308	25.71819	2.923032
LOC107798504	0.051127	0.419365	0.466659	1.296921	1.492942	1.126843	8.769479	14.63428	10.44009	14.40822	16.02512	14.75063	9.13493	18.18337	27.1036	0.2286	0.172876	0.114352	18.80661	19.15977	6.831122	33.77381	16.43805	11.11512	162.9904	304.4779	174.5166	31.07867	51.56723	33.19332
LOC107793148	0	0	0	0	0	0	0	0	0	0	0	0	0	0	0	0	0	0	0	0	0	0	0	0	0	0	0	0	0	0
LOC107823719	0.100009	0.045573	0.065202	3.510873	2.477849	2.822453	0.146793	0.213626	0.164313	0.073141	0.245443	0.168981	0.083499	0	0.025662	0.055895	0.193234	0.037281	1.040194	0.951759	1.254137	0.208801	0.330805	0.418118	0.03161	0.139155	0.118203	0	0	0
LOC107829586	6.764577	8.08125	9.058845	22.93996	37.77489	23.29245	26.60525	36.70978	23.51543	16.93649	9.48665	16.06351	6.309272	11.76782	11.25888	3.746667	5.033832	5.588498	37.75324	33.87022	46.93752	23.97125	16.85219	14.23621	35.71239	20.85995	28.52336	143.8214	35.20765	90.43545
LOC107789153	0	0	0	0	0	0	0	0	0	0	0	0	0	0	0	0	0	0	0	0	0	0	0	0	0	0	0	0	0	0
LOC107777731	4.648704	5.17954	4.746775	2.811615	2.247929	5.420285	0.549165	0.492302	0.799117	0.98323	1.414056	0.398267	0.553944	1.014301	0.376328	3.708179	3.592831	4.344461	1.54139	2.31518	0.537426	0.94049	1.617084	1.970903	0.281452	0.091103	0.541701	0	0.378262	0
LOC107830538	8.141217	8.325172	9.595915	7.025505	5.445648	5.10333	7.476508	7.639894	7.8247	4.144278	4.779765	3.730401	3.440836	2.739769	3.455669	8.082271	8.85157	7.777347	4.788663	4.482327	4.782156	6.430187	8.446911	8.076527	1.570115	2.764778	2.511574	3.947201	2.125815	1.706385
LOC107774788	0	0	0	0	0	0	0	0	0	0	0	0	0	0	0	0	0	0	0	0	0	0	0	0	0	0	0	0	0	0
LOC107812996	0	0	0.030662	0	0	0	0.059169	0	0	0	0.024733	0	0.039266	0	0	0	0	0	0	0	0	0	0	0	0.08919	0	0	0	0	0
LOC107820756	1.405443	1.625742	1.879571	1.175317	0.717478	1.917836	0.952112	1.062289	1.268742	0.685242	1.099208	1.278692	1.444208	1.769156	0.943178	1.168184	1.044443	1.329922	0.548518	0.640181	0.739493	0.85772	1.668818	1.129975	0.205028	0	0.83056	0	0	0
LOC107759652	0	0	0	0	0	0	0	0	0	0	0	0	0	0	0	0	0	0	0	0	0	0	0	0	0	0	0	0	0	0
LOC107804804	0	0	0	0	0	0	0	0	0	0	0	0	0	0	0	0	0	0	0	0	0	0	0	0	0	0	0	0	0	0
LOC107787847	13.80552	18.98406	16.55411	6.621406	7.22183	8.905572	4.76794	5.689744	5.375098	5.226472	6.121454	5.214175	4.610549	4.518343	4.084181	15.49235	15.73016	16.95252	8.734953	8.999329	6.332465	4.950838	7.879481	8.700154	5.082303	4.124365	4.847112	2.903793	3.323234	2.510635
LOC107759006	1.09212	0.765641	0.876329	0.875243	0.817706	1.716084	0.563697	0.538348	0.670408	0.737277	0.530166	0.248407	0.327321	0.5499	0.344899	0.719944	1.13624	1.252649	0.468857	0.497462	0.306471	0.456029	0.44461	0.526839	0.531066	0.058446	0.744691	0	5.05563	0
LOC107824624	0	0	0	0	0	0	0	0	0	0	0	0	0	0	0	0	0	0	0.127461	0	0.229119	0	0	0.131289	0	0	0	0	0	0
LOC107816041	0	0.266989	0.152794	0.15924	0.155534	0.062992	0	0.150184	0.055007	0.057133	0	0	0	0	0.120271	0.087323	0	0.043682	0.237814	0.049563	0.053435	0.04893	0.103361	0	0.296304	0.570669	0.138498	0	0	0
LOC107819621	9.867539	9.622093	11.48871	11.67229	10.33749	10.93862	9.884425	9.78531	14.40013	6.456277	5.915314	7.12695	4.439405	5.772941	4.565369	8.782052	11.16285	9.948656	6.329396	6.422632	8.113414	11.10138	14.04788	16.93987	2.327049	1.458364	6.193888	1.828157	1.476866	1.580634

LOC107815841	0	0	0	0	0	0	0	0	0	0	0	0	0	0	0	0	0	0	0	0	0	0	0	0	0	0	0	0	0	0
LOC107813482	0	0	0	0	0	0	0	0	0	0	0	0	0	0	0	0	0	0	0	0	0	0	0	0	0	0	0	0	0	0
LOC107825373	65.69565	66.26357	72.06343	53.59193	48.53878	71.39101	46.157	47.32217	60.79291	45.04418	38.54657	44.40121	40.14134	49.04463	31.68824	50.75667	72.17652	59.08217	33.42139	41.6182	44.4396	49.35314	58.36368	64.2311	27.11531	19.18395	43.80264	33.70883	29.78443	7.286208
LOC107785372	5.803985	6.251365	5.670729	7.34398	5.178192	7.683958	8.32799	8.237814	7.700704	5.612844	7.063216	7.172695	6.514474	6.907486	7.778628	5.00429	5.699817	6.758885	4.477954	5.640151	6.168262	6.008751	6.134936	5.414569	2.850298	2.00227	4.195276	3.430305	3.694869	5.931721
LOC107769338	2.068145	1.558634	2.281817	0.324283	0.387122	0.470358	0.500378	0.203894	0.560096	0.155131	0.055776	0.100802	0.132824	0.043389	0.040821	1.304079	1.344809	2.342487	0.484295	0.403733	0.580364	0.398576	0.45606	0.748255	0.050284	0.05534	0.047007	0	0	0
LOC107782175	0	0	0	0	0	0	0	0	0	0	0	0	0	0	0	0	0.120484	0.092979	0	0	0	0	0	0	0	0	0	0	0.600416	0
LOC107785734	0	0	0	0	0	0	0	0	0	0	0	0	0	0	0	0	0	0	0	0	0	0	0	0	0	0	0	0	0	0
Novel00068	0.188078	0	0.081746	0.425973	0.41606	0.30331	0.394373	0.482097	0	0.2751	0.791283	0.317787	0.418742	0	0.289557	0.070078	0	0.14022	0.572546	0.079551	0.257296	0.078534	0.082949	0.196579	0.237788	0.261697	0.222293	0	0.452738	0
LOC107786353	43.97643	42.43351	44.80988	48.09903	42.56546	48.0143	34.4442	32.75414	35.93448	32.63245	33.43896	30.88099	32.06382	30.7423	26.69771	39.29899	38.38274	41.17658	35.04254	38.33138	32.49743	37.569	40.93785	46.27451	23.0132	21.85652	26.60248	6.370567	23.73074	18.36009
LOC107775635	4.575059	4.056144	3.219478	5.188014	4.213551	4.851827	5.795105	5.079112	3.740097	3.702543	7.289003	2.340117	5.127666	3.599071	8.209516	3.641267	3.728344	2.854019	5.463568	5.84477	3.434532	5.562183	5.984626	4.586673	1.219811	1.992038	2.611714	0	2.54722	0
LOC107766926	0	0	0	0	0	0	0	0	0	0	0	0	0	0	0	0	0	0	0	0	0	0	0	0	0	0	0	0	0	0
LOC107820418	0.14849	0.101498	0.258158	0.100893	0.164242	0.159645	0.093409	0.253747	0.034852	0.108597	0	0.125448	0.330602	0.080998	0.076203	0.082991	0.071727	0	0	0.094209	0	0.093006	0	0	0	0	0	0	0	0
Novel04025	3.11853	2.263198	2.811275	3.296102	3.526003	3.849513	3.681279	2.911275	3.09047	2.703076	3.199084	4.195878	4.178768	3.52817	2.489491	2.75429	4.742312	2.841663	2.168247	1.95412	2.844152	2.700824	3.514847	4.647787	1.606314	0.160711	2.525491	0	3.892445	0
LOC107807714	1.427303	1.21822	1.083172	1.498313	1.042453	1.315308	0.8551	1.045309	1.148584	0.927867	1.016712	0.823024	0.907939	1.087511	1.092895	0.827266	1.028699	1.114787	0.850606	0.862429	0.619869	1.362261	1.159057	1.231341	0.171861	0.283713	0.401657	0	0.327216	0
LOC107772884	2.327989	1.742805	2.276631	2.033718	2.906012	2.949815	2.75454	3.587479	3.161342	1.459339	1.778137	2.001871	2.498995	2.49437	1.749371	1.796778	2.008066	1.704622	1.940447	1.476989	2.009425	2.534358	3.410178	4.301575	0.735822	1.21471	1.031811	5.945863	3.20222	0
Novel01820	0	0	0	0	0.031275	0.037999	0	0	0	0.034465	0	0	0	0	0	0.97452	0.273162	0.500658	0.215188	0.269087	0.032234	0.295167	0.187054	0.110825	0.446855	0.393428	0.125321	0	0.680634	0
LOC107826826	0	0	0	0	0	0	0	0	0	0	0	0	0	0	0	0	0	0	0	0	0	0	0	0	0	0	0	0	0	0
LOC107811902	1.040658	1.896862	1.809243	1.414174	0.844105	1.678251	0.72737	0.815069	0.569925	0.845644	0.912137	1.392029	0.965395	0.946088	0.534051	1.292499	1.843165	0.840509	1.319984	0.953685	1.423646	0.579387	0.917929	0.906412	2.302488	3.378664	2.562445	12.40362	1.252524	0
LOC107799605	0.392148	0.306338	0.340886	0.203009	0.198285	0.06023	0.093975	0.095732	0	0.054628	0.039282	0.094656	0	0	0	0.083494	0.054121	0.250597	0	0.18956	0.051092	0	0.247072	0.17566	0	0.077949	0	0	0	0
LOC107792347	15.22667	19.62998	17.80874	16.55352	15.23738	19.22794	20.43403	19.06569	18.55812	21.27304	27.4883	20.25482	21.38851	20.05468	19.03804	19.68178	21.39672	16.71859	9.10203	10.67929	7.019239	20.20719	19.87778	19.32939	7.280433	3.235803	9.685499	7.919437	7.197379	13.69437
LOC107799883	40.32213	13.60702	11.62207	8.417599	7.123383	10.59951	15.3186	14.57482	25.53712	2.282379	8.405083	4.524058	15.75196	14.74053	4.295068	6.289752	16.89059	7.667493	2.015218	0.509996	4.010583	13.86057	13.5135	14.38183	0.044837	0	0.544897	2.53615	0.341469	4.385538
LOC107828045	0	0	0	0	0	0	0	0	0	0	0	0	0	0	0	0	0	0	0	0	0	0	0	0	0	0	0	0	0	0
Novel04687	1.422516	1.633879	0.982475	0.600229	0.344859	1.026569	0.277851	0.316346	0.530549	1.67216	1.557691	0.707898	1.388331	0.7228	0.120003	0.987454	0.545943	0.755457	0.573434	1.153898	0.284353	0.276653	0.32658	0.590652	1.724582	0.379597	1.750394	1.393562	1.876301	1.204881
LOC107794643	0.042794	0	0	0.038769	0	0	0.035893	0	0	0.041729	0	0.072307	0	0.046686	0.043923	0.03189	0	0	0.043424	0	0.078058	0	0.150988	0	0.054105	0	0	0	0	0
LOC107804138	0.241753	0.29377	0.210151	0	0	0	0	0	0	0	0	0	0	0	0	0.120103	0.311405	0.180237	0	0	0	0	0.071081	0	0	0	0	0	0	0
LOC107794978	0.343313	1.050408	0.447652	6.353781	10.7193	4.666523	4.463247	5.86672	2.256222	7.723705	4.350359	5.324313	12.61197	7.731527	15.32797	1.242638	0.900245	0.950688	19.1106	14.35501	10.73511	5.590839	2.50912	1.666	12.06045	13.17073	2.115792	7.014782	14.04907	9.097527
LOC107823525	0	0	0	0	0	0	0	0	0	0	0	0	0	0	0	0	0	0	0	0	0	0	0	0	0	0	0	0	0	0
LOC107803888	109.6034	85.97274	106.8375	62.84974	57.10088	101.3311	57.84303	58.85452	69.29406	52.27775	51.23599	58.33202	43.50392	62.1595	34.04311	51.39202	103.2687	64.6979	35.15654	35.48941	49.79289	46.07488	61.12043	52.43042	22.52444	16.56423	36.67943	29.35653	35.96851	20.30545
LOC107789847	0.673262	0.613595	0.663286	0.772592	0.635463	0.965118	0.527047	0.153401	0.294974	0.700283	0.440619	0.56879	0.149897	0.587596	0.230339	0.43482	0.693789	1.238131	0.364362	0.303751	0.24561	0.374839	0.395908	0.32839	0.113494	0.062453	0.106099	0	0.864352	0
LOC107811314	11.1369	7.228421	6.319984	19.46039	22.0487	25.26165	9.701329	14.37233	14.92357	19.65735	16.49896	26.99783	9.638605	12.25796	7.733465	6.67386	8.045438	7.046487	15.6939	13.75417	17.99333	12.52974	14.86639	28.91073	19.05244	15.26626	54.76085	14.18004	4.932126	0
LOC107794465	0.159451	0.07266	0	7.006059	8.818314	3.685738	13.90882	24.72748	19.16159	11.81687	14.36725	16.77126	10.2952	10.17628	17.83856	0	0.308087	0.178317	11.81141	16.38851	9.161605	12.11774	13.78337	18.74908	20.0587	27.28938	23.55733	11.40302	9.211866	4.929558
LOC107762069	0.179506	0.229036	0.093624	6.309775	5.559346	2.701868	8.852878	7.607377	15.90903	2.940684	6.293475	5.914405	5.355397	10.80992	2.026637	0.107014	0.381519	0.240892	5.428069	3.522908	7.465277	9.564251	7.536788	9.155822	3.313467	2.997229	4.115929	0	1.209886	15.53875
LOC107802128	12.41481	10.61614	13.38999	10.62234	11.15497	13.05907	12.34114	12.60465	15.03712	11.58291	11.31148	12.8515	12.28474	10.70139	10.0286	12.13549	15.88061	12.76953	9.176095	8.168279	10.23919	14.8479	15.78385	18.5428	7.944935	6.077994	11.63901	0	7.00998	2.369217
Novel04816	3.004053	2.688045	2.801277	1.929805	2.271539	2.994835	1.465966	1.493379	1.640924	2.84944	3.063918	3.022417	3.344154	2.770786	1.765877	2.197922	2.691101	3.074427	0.969915	1.178203	1.419687	0.752629	1.180354	1.569918	0.414331	0.379993	0.806943	3.906041	0.788868	0
LOC107782420	0	0	0	0	0	0	0	0	0	0	0	0	0	0	0.057622	0	0	0	0	0	0	0	0	0	0	0	0	0	0	0
LOC107801806	0	0	0	0	0	0	0	0	0	0	0	0	0	0	0	0	0	0	0	0	0	0	0	0	0	0	0	0	0	0
LOC107829170	0.220546	0.1005	0.191716	0.249755	0.243942	0.177836	0.092491	0.235551	0.258823	0.053765	0.077324	0.093162	0	0	0	0.205439	0	0.041107	0.167846	0.093284	0.100571	0.092092	0.145902	0.172886	0	0	0	0	0	0
LOC107796385	4.946672	8.565735	5.781161	1.195049	0.875428	1.595483	0.691498	0.610505	0.412814	0.535959	0.192701	0.742949	1.040155	0.59962	0.169238	7.454449	5.203692	4.876295	2.119362	2.650211	1.854706	0.734417	1.212026	0.574473	1.389799	0.305908	1.689009	19.65317	3.704562	6.796893
LOC107781998	6.838273	5.268494	5.239621	5.875792	5.021668	7.124599	6.356413	5.089867	5.692023	6.805668	5.981393	9.023095	9.260519	8.383134	5.282051	5.358564	6.265813	6.517316	5.40098	4.591985	5.690112	4.651081	5.472157	8.252623	3.83261	3.97274	6.83245	5.041579	6.278915	2.179488
LOC107768850	20.46312	16.28932	17.16985	14.52352	11.86162	16.35321	19.58024	18.77765	25.05053	15.17012	14.48943	14.28325	17.35897	12.37584	10.10829	15.43942	17.70762	14.71224	18.21063	18.22066	14.72059	16.78467	19.81942	16.39369	5.349566	9.33872	4.46208	6.521402	6.760954	2.255376
LOC107769203	0	0.057239	0.054595	0.512087	0.611319	0	0.684809	0.912266	0.353787	0.428703	1.100981	0.265299	0.769075	0.274071	0.966927	0	0	0.187297	0.573576	0.318775	0.229119	0.944109	0.443189	0.065644	0.952862	1.747785	0.965003	0	0.302368	0
LOC107824670	2.068711	0.678735	0.719315	2.623806	2.196638	1.067576	2.151545	2.191778	1.08763	1.613804	1.740698	2.796327	2.118684	2.798509	1.783545	1.726599	2.87791	1.912467	2.602989	2.939978	3.622462	2.902426	2.700615	2.767637	3.138575	1.496799	1.46703	0	1.593522	0
LOC107764937	2.453578	3.115513	3.618645	1.698333	1.292895	1.985832	2.242904	1.719522	1.967057	0.457004	1.043869	0.838457	0.767235	0.751891	0.933746	2.855606	3.089462	3.309087	0.615437	1.189365	0.930282	2.233231	2.06695	2.045821	0.034855	0.153437	0.260668	0	0.132724	0
LOC107800188	14.71592	12.5717	13.37244	10.5388	7.481098	13.05352	6.691276	7.115127	9.3101	6.694543	8.446714	6.739819	9.093228	6.484151	3.001229	10.16102	13.04999	13.43574	5.289335	7.582117	5.681529	5.786486	8.662953	8.836641	0.884057	0.796048	2.892577	0	1.071131	1.965242
LOC107769468	0.084511	0.046213	0.044078	0.06125	0.119649	0.090859	0.014177	0.101092	0.031737	0.494452	0.142222	0.099956	0.188157	0.147515	0	0.025191	0.032658	0.075608	0.120058	0.057192	0.246641	0.070577	0.119271	0	1.346276	0.658507	1.418366	0	1.546086	2.090171
LOC107795568	0	0	0	0	0	0	0	0	0	0	0	0	0	0	0	0	0	0	0	0	0	0	0	0	0	0	0	0	0	0
LOC107767213	0	0	0	0	0	0	0	0	0	0	0	0	0	0	0	0	0.038025	0	0	0	0	0	0	0.064483	0	0	0	0	0	0
LOC107824908	2.537779	2.497903	1.706002	2.820229	3.203714	2.582907	4.427373	3.931937	3.303843	3.695485	4.958852	1.743776	5.424175	2.842421	3.64659	3.580589	2.288261	3.102916	2.678169	3.406235	1.604718	3.504018	3.492043	3.678114	2.566819	1.883272	2.079619	2.419828	2.443554	0
LOC107782136	10.37308	8.203695	9.315183	4.659909	4.475605	8.571624	4.242318	5.933109	6.519297	3.343822	4.207873	3.548841	3.912774	2.992798	1.935753	5.813494	8.033577	7.158346	3.39264	4.351195	2.892856	3.221713	4.310209	5.376168	1.517406	1.312126	4.964852	0	2.476348	0
LOC107794247	3.700949	5.059438	4.64518	4.173984	4.160388	4.202283	3.230845	3.081523	4.307813	3.480004	4.541439	3.15857	6.032781	4.058154	4.418725	5.192266	5.271932	5.968914	3.678825	4.216926	2.893138	3.46922	3.381071	4.085326	2.458928	3.730833	2.321017	0	2.545384	2.335054
LOC107795785	0	0	0	0	0	0	0.138026	0	0	0	0	0	0	0	0	0	0	0	0	0	0	0	0	0	0	0	0	0	0	0
LOC107782769	4.324701	7.001838	5.329461	8.688653	8.981678	6.509415	9.985779	8.564035	6.591886	7.39238	9.18664	5.845804	8.127701	6.077941	11.95853	8.89106	5.111936	7.700263	8.854258	9.619439	5.753892	10.13392	7.943494	5.663515	10.77466	14.93757	3.84862	3.638556	6.981042	7.864788
LOC107768966	0	0	0	0	0.08005	0	0	0	0	0	0	0	0	0	0	0	0	0	0	0	0	0	0	0	0	0	0	0	0	0
Novel00504	6.21589	4.402115	5.747503	5.093261	5.149895	5.576129	3.420344	3.92407	7.285397	3.551832	2.609599	6.823419	4.936286	4.57841	1.25971	3.687969	4.628113	5.047451	2.048919	2.344427	4.441316	2.347546	5.657438	8.193475	0.901006	0.440711	2.386502	0	1.14365	2.448012
LOC107825949	3.197602	1.965942	7.257864	4.483244	6.063104	4.147203	5.427789	5.008882	7.600427	5.246269	3.594574	5.231327	8.531775	7.835116	6.537814	8.585848	4.53566	8.741142	7.313463	5.281095	7.336956	7.057488	4.521748	7.055613	5.903685	4.696409	4.079253	16.33384	16.49399	17.26062
LOC107792005	6.034535	3.871813	5.119796	4.373586	3.631032	4.723164	2.631941	2.804902	3.988503	1.835943	2.335744	2.202398	1.558513	2.159349	1.189187	2.842068	3.264726	3.347257	1.763548	1.633536	2.025324	2.902808	4.00279	6.357752	0.610359	0.201519	0.798821	0	0.464838	0
LOC107789409	1.786886	1.725752	1.92424	2.174545	1.769949	1.404999	2.30403	1.731852	2.478853	0.702177	1.271667	0.833666	0.712544	1.396588	1.040185	1.609832	1.442863	1.630453	0.893073	0.72195	0.899967	3.073641	2.117219	3.122046	0.202313	0.482421	0.535869	0	0.128399	3.298085
Novel04846	1.265094	1.042114	1.438095	4.05547	2.604847	3.609578	2.489478	1.642183	1.644544	2.016505	4.316009	1.870377	4.197878	2.73377	3.096323	0.03626	0	0.018138	0	0.02058	0	0	0	0	0	0	0	0	0.117127	0
LOC107787614	0	0	0	0	0	0	0.078733	0	0	0	0	0	0	0	0	0	0	0	0	0	0	0	0	0	0	0	0	0	0	0
LOC107776873	0	0	0	0.056291	0	0	0	0	0	0	0	0	0	0	0	0	0	0	0	0	0	0	0	0	0	0	0	0	0	0
Novel04837	5.027705	3.329541	3.155321	2.692468	2.609019	2.399593	2.433615	2.268347	3.815888	1.981678	2.298119	1.478701	2.929225	2.960351	1.097166	3.440267	2.122182	3.722094	1.203922	1.271953	0.728514	1.814888	3.077398	3.499211	0.415849	0.163451	0.652547	0	0.735204	0.726333
LOC107783191	1.454993	1.812264	2.065832	0.615137	0.343326	1.355723	0.040679	0.497274	0.182135	0.14188	0.136032	0.081948	0.107981	0.158733	0.049779	1.192689	1.780485	1.048601	0.246071	0.65644	0.575024	0.202517	0.4278	0.405536	0	0	0	3.46842	0.233495	0
Novel04688	12.59928	10.4918	11.61943	16.86074	14.14806	29.84195	7.349015	10.76517	13.6301	8.356901	6.63718	13.99418	3.987	4.116582	0.984636	5.00429	8.526556	6.150943	9.604886	9.576083	16.33204	5.127468	11.28264	18.3828	2.183207	0.800908	5.744882	13.72122	1.847435	0
LOC107803538	4.495001	4.435204	5.87726	5.788643	6.015516	6.310436	5.826597	5.808587	4.010843	7.498521	11.07071	4.048576	5.872343	5.14699	8.731421	6.173377	4.773215	6.563948	5.729792	7.196394	2.507142	6.980379	4.161494	3.494509	3.475583	3.049698	2.722225	5.313324	4.11349	0
LOC107792310	0	0	0	0	0	0	0	0	0	0	0	0	0	0	0	0	0	0	0	0	0	0	0	0	0	0	0	0	0	0
LOC107830709	0	0	0	0	0	0	0	0	0	0	0	0	0	0	0	0	0	0	0	0	0	0	0	0	0	0	0	0	0	0
LOC107823524	1.574255	1.532563	1.617279	1.977225	0.601525	2.38491	0	0.183421	0.335905	0.069777	0	0.030227	0.079658	0.15613	0.073444	0.799866	0.864127	0.986953	0.835028	0.514525	0.358937	0.089639	0.252472	0.299166	0	0.099566	0	0	0	0
LOC107791825	17.3609	22.68067	17.90318	16.80455	15.27445	13.48429	22.97702	20.07898	21.72191	10.42779	14.51097	12.1853	16.7177	14.97908	16.39597	18.20086	17.02718	16.31445	11.01878	11.39152	9.873322	21.44462	17.32227	16.24826	2.96276	3.995455	3.861977	4.720723	4.131408	16.32625
LOC107771583	15.1153	16.00349	15.02576	24.31422	21.25435	22.39178	19.56072	16.70842	20.56043	14.66636	18.66169	17.06858	14.4664	16.38121	14.0803	15.65056	13.85565	15.47177	20.77965	20.88885	23.04393	22.10099	21.0771	26.48727	8.325296	8.572384	14.47484	5.351277	8.405827	7.711237
LOC107818033	0	0	0	0	0	0	0	0	0	0.031046	0	0	0	0	0	0	0.024654	0	0	0	0	0	0	0	0	0	0	0	0	0
Novel02744	0.436498	0.132605	0	3.427191	3.991169	1.877155	2.806841	3.853876	3.278429	3.405125	4.59109	2.458438	3.40141	2.380989	4.330759	0.650558	0.140565	0.433904	3.83871	5.04638	3.317446	4.00984	2.566801	1.216607	14.34852	11.13484	13.06961	10.40526	12.60874	0
LOC107822741	4.52986	4.93511	3.847189	1.603793	2.027195	2.351109	1.353805	1.690535	1.857559	0.710811	0.912742	1.231663	0.579621	0.795241	0.855048	2.677245	2.716278	3.998298	0.686847	0.836863	1.662021	1.652347	3.031172	4.571346	0.197487	0	0.307697	0	0	0
LOC107762178	0.141951	0.258741	0.123395	0	0	0	18.69243	14.91823	2.931928	0.276839	10.25219	0.599619	39.03128	30.35278	21.41704	0.105782	0	0.10583	0.144042	0.12008	0	18.73037	1.377308	0	0	0.592542	1.845516	0	0.683401	0
LOC107791928	2.210834	2.54446	2.303734	2.580664	2.545687	2.483574	2.626956	2.228047	2.102239	2.031467	2.762604	2.035395	3.044861	2.272763	2.603692	3.37952	2.163234	3.254278	2.387209	2.421687	1.809516	2.852336	2.787646	2.918079	3.368534	2.208564	2.361775	1.013499	2.660936	1.752554
Novel02319	0.4568	0.24979	0.21178	0.68973	0.592837	0.392893	0.332053	0.182141	0.60041	0.742397	0.49114	0.231551	0.372912	0.398677	0.437591	0.113469	0.147102	0.15893	0.185412	0.18033	0.083322	0	0.107447	0.095489	0.192511	0.127121	0.287947	0	0.146613	0
LOC107821461	0.058788	0.133944	0.025551	0	0	0	0	0	0	0	0	0	0	0	0	0.262852	0.113588	0.131486	0	0	0	0	0	0	0	0	0	0	0	0
LOC107768613	2.46023	2.797954	4.332087	6.64843	6.828625	4.747522	7.671989	7.743585	6.001459	8.79646	7.00367	5.880117	8.403552	6.606744	10.05728	10.26372	3.311253	4.232782	7.510751	9.552106	5.062868	6.848674	6.102206	5.186819	7.071685	6.875718	6.784852	3.007542	7.693812	18.20236
LOC107804327	7.700547	6.652573	5.09017	7.339655	8.517438	8.02031	6.391501	5.962723	7.229613	6.257505	6.918274	9.351852	9.197424	10.67617	6.4217	4.722272	6.044493	5.681282	8.465148	7.531966	10.24192	5.292118	6.438621	7.96477	10.04008	7.031554	10.99756	17.20931	26.64634	29.75853
LOC107784045	3.120144	2.919121	3.408345	2.926752	2.931938	3.888887	2.941229	3.302929	3.784796	3.957987	3.155921	3.545749	4.241836	4.187127	3.03239	2.674934	2.90762	3.499593	2.577715	2.545996	3.147814	2.329001	3.409772	3.607493	3.176794	1.882577	3.589854	0	6.247874	3.41457
LOC107773446	0	0	0	0	0	0.110638	0	0	0	0	0	0	0	0	0	0	0	0	0	0	0	0.093853	0	0	0	0	0	0	0	0
LOC107801999	5.853367	6.534586	4.553262	11.34474	10.4602	12.57078	8.97869	7.324606	7.027123	11.69465	12.5841	10.09572	15.77234	13.75509	15.10013	6.484247	7.867009	6.79664	10.35435	12.27596	6.982952	8.569464	7.725649	6.043146	8.124194	11.66918	5.903312	4.093873	7.165623	8.84896
LOC107789128	3.28074	3.658559	3.303572	1.91992	1.965395	1.369253	2.068046	1.802027	2.257467	1.808207	2.057551	1.38583	2.722111	2.423135	2.175125	4.904807	3.199	3.357458	1.271663	1.447972	0.771253	2.6122	2.04006	1.352443	1.223745	0.765544	0.927245	0	2.501637	3.149893
LOC107774679	1.394138	1.497475	1.77456	0.947264	0.969277	1.338276	1.169324	1.233732	2.430774	0.728283	0.837918	0.799226	1.385691	0.869106	0.459934	1.113121	1.250651	1.373479	0.606289	0.379075	0.726559	0.665304	1.844583	2.289812	0.188852	0.27712	0.41194	0	0.23971	0
LOC107814203	19.75364	13.51577	15.31505	15.63867	13.28008	24.17129	16.02093	14.19175	23.83637	20.59272	19.50823	25.20803	35.39535	33.65204	20.21371	12.33334	17.76558	17.11536	8.005811	9.183092	8.547965	13.97024	16.37753	20.8325	4.349931	1.980954	10.37654	12.72671	8.567661	3.667859
LOC107770624	150.0321	137.2225	151.9539	317.7704	258.1733	564.0299	106.3355	147.1113	119.0262	493.5028	515.2103	394.3103	116.3911	160.9885	303.8032	157.2053	148.2378	186.1906	613.596	810.1894	377.0742	154.4239	152.631	191.0359	490.6285	966.9094	618.8924	246.7883	197.982	129.5488
Novel03196	0.606047	0.552337	1.053647	1.333402	1.60881	0.744657	1.307102	0.295899	0.4877	1.266369	0.364252	1.097154	0.67466	0.75562	0.62203	0.258072	0.167283	0.903667	1.229949	1.098587	2.131939	0.433822	0.763676	0.63344	1.094611	0.722803	0.818627	6.19156	1.250454	5.353255
LOC107782554	7.150549	9.593569	9.658784	0.650212	0.693883	1.085997	0.133773	0.340685	0.199651	0.401772	0.372786	0.280715	0.769377	1.014987	0.381962	9.221048	7.370279	9.453178	0.809206	0.640866	0.254554	0.410687	1.477164	0.666803	0.134431	0.05548	0.596937	0.950493	0.127975	0
LOC107819681	7.574524	9.418834	10.71356	14.01504	11.64407	10.69702	17.06717	14.69884	12.05305	10.45323	10.84758	6.453399	9.289564	8.333463	14.75796	10.71507	8.123807	8.85355	13.93921	13.62957	8.781475	17.31522	10.58805	9.92966	11.68658	14.02276	7.662715	4.590556	5.871718	7.938038
Novel00964	0	0	0	0	0	0	0	0	0	0	0	0	0	0	0	0	0	0	0	0	0	0	0	0	0	0	0	0	0	0
LOC107823355	19.89155	23.97784	21.79605	32.57456	28.7477	26.78589	35.87541	33.14027	29.97741	29.9309	31.64149	27.81739	34.7463	29.92001	31.5857	25.84407	25.72525	25.20208	25.28128	27.28046	27.99379	38.61697	36.9549	33.49836	25.75376	28.12137	22.28523	22.80605	26.48411	41.90124
LOC107782958	0	0	0	0	0	0	0.244073	0	0	0	0	0	0	0	0	0	0	0	0	0.246165	0	0	0	0	0	0	0	0	0	0
LOC107779664	0.219445	0.24615	0.278801	0.44349	0.418232	0.127039	1.118505	1.153843	0.443745	0.395052	0.970595	0.499135	0.507369	0.29465	1.559299	0.628963	0.163078	0.22653	0.548129	0.871057	0.400275	1.480209	1.250726	0.617517	0.298787	0.281853	0.119707	0	0.325072	0
LOC107826537	1.107324	1.712563	1.429275	2.583946	2.880115	2.597473	2.392261	2.350983	2.39424	3.501106	2.564658	2.437964	2.577439	2.928571	1.963091	2.025439	1.62086	1.776198	2.792064	2.753388	2.203413	2.690196	2.279043	2.840828	1.866658	1.027173	2.300258	0	1.130829	0
LOC107811110	17.07817	11.73758	12.24333	33.36483	41.49548	26.93685	34.52975	42.16245	37.83963	73.31404	73.8801	70.73171	57.45995	56.20121	73.31066	35.17054	19.68228	27.84673	46.93304	56.80204	33.86315	39.06227	27.68246	31.03818	51.36356	80.79431	45.83188	90.52217	36.95082	47.20803
LOC107818188	0.260496	0.109574	0.104513	0.471993	0.248235	0.215435	0.436979	0.428029	0.526756	0.097699	0.196711	0.186217	0.089227	0.021861	0	0.164257	0.212944	0.313725	0.264333	0.220362	0.164476	0.435094	0.724674	0.481709	0	0	0.094734	0	0	0
LOC107767918	0.689207	0.448663	0	0.089198	0	0	0.247743	0	0.277311	0.096009	0.069039	0.16636	0.21921	0.537065	0.20211	0.366856	0.570713	0.513833	0	0.083289	0	0	0.26054	0	0	0	0	0	0	0
LOC107830277	5.119049	6.220509	6.877088	0.608978	0.457543	1.890126	0.867387	0.574344	0.582544	0.504214	0.652633	0.262104	0.287808	0.902567	0.106142	3.968851	5.295109	4.471821	0.209877	0.393669	0.424424	1.079558	0.820971	0.540447	0	0	0	0	0	0
LOC107817270	4.704391	5.035622	4.363885	13.88706	15.64525	7.7055	21.99306	23.80712	14.71736	28.54025	30.43016	18.40494	22.4066	24.38684	35.4357	8.046681	4.834609	5.825923	24.86164	25.7338	21.45242	27.31675	17.60092	13.26608	65.18607	84.19502	42.13073	46.28741	47.19728	70.27963
LOC107758988	0.088629	0	0	0.24088	0.392124	0	0.074337	0.075727	0	0	0.062147	0	0.098663	0.19338	0.090966	0.132093	0	0	0.179869	0.299896	0	0.296066	0	0.092635	0.336162	0.863246	0	0	0	0
LOC107822265	5.320197	2.644748	4.414519	4.513113	4.365285	5.40787	3.407558	3.553927	4.54076	3.301368	3.696612	5.148418	6.353247	5.276431	3.67343	3.171694	2.94368	3.677965	3.190063	3.886814	3.749357	2.62543	3.11429	3.63971	3.302025	2.961067	4.573124	3.458812	4.889816	5.981015
LOC107776436	2.381865	1.724992	1.848665	1.522054	0.80918	0.75452	10.34561	6.995759	7.00809	0.518442	1.386834	0.6468	11.90819	6.12503	3.710676	2.83678	2.773625	2.93321	0.237379	0.089951	0.135769	7.512622	3.939304	2.889627	0.053775	0	0.025135	4.562599	0.20477	0
LOC107827307	0.078296	0.071357	0	0.141864	0	0.589241	0.19701	0.066898	0	0.229044	0	0	0.261479	0.085417	0.08036	0.116692	0	0	0.635592	0.264931	0.214221	0.065387	0	0	0	0.108943	0.092539	0	0	0
LOC107830784	0	0	0	0	0	0	0	0	0	0	0	0	0	0	0	0	0	0	0	0	0	0	0.01496	0	0	0	0	0	0	0
LOC107784994	3.356545	2.871238	2.917773	3.574343	2.709557	2.78566	4.716826	4.503142	4.505766	4.450206	6.193714	4.676421	3.408785	4.753948	5.983535	3.620304	2.929812	2.700008	3.585252	3.237919	3.678841	3.90964	3.37623	4.062195	6.104961	6.432024	3.44517	6.316853	3.827274	1.820528
LOC107764147	3.693631	5.274737	4.651861	2.819275	2.033079	2.220067	3.317551	3.404438	2.867032	1.588176	2.345254	2.0148	2.233971	1.745089	2.507461	5.591689	6.602868	5.095538	1.888777	2.189651	2.121987	3.643285	3.001489	2.492659	0.808954	1.982925	1.443733	2.079896	1.120155	1.79829
LOC107794552	0	0	0	0	0	0	0	0	0	0	0	0	0	0	0	0	0	0	0	0	0	0	0	0	0	0	0	0	0	0
LOC107774405	2.027285	2.148046	2.177771	2.314436	1.91055	1.896056	2.419227	1.915242	2.073518	1.783994	2.438565	1.406369	1.761406	1.744161	2.486749	2.370503	2.069988	2.494469	1.639029	1.547632	1.412994	1.968329	1.70097	2.170598	0.83353	1.215475	0.915578	0	1.428303	0
LOC107819840	20.57507	19.64458	16.87	5.803655	8.936365	10.29059	6.95345	5.183817	12.27642	2.131266	2.219577	1.018748	10.99078	9.743214	4.409188	10.27788	10.99438	12.58633	3.059071	4.685987	2.130803	3.744965	4.188132	2.678286	0.047643	0.209734	0.400847	0	0.544262	0
LOC107791109	1.027054	1.002893	1.020339	1.063382	0.84389	0.946465	0.553779	1.065588	1.101993	0.715362	0.823053	0.867684	0.816665	0.400166	0.376479	1.25738	1.842696	1.367344	0.744417	0.868817	1.672662	1.041517	2.329533	2.22363	0.185501	0.306229	0.867068	0	0	0
LOC107766621	26.5867	24.2305	33.11463	23.24738	21.06775	28.58395	17.86171	15.03125	25.33331	19.47629	19.84835	26.70757	31.06915	22.79978	12.48996	21.48812	25.17384	23.76609	17.18024	19.35751	13.63169	10.38361	13.06735	22.25838	5.685894	7.36188	15.32083	0	8.278466	16.35717
LOC107826766	10.36266	12.81508	11.41212	6.942929	7.253097	6.62736	16.43301	16.05703	12.88848	8.707764	10.30367	7.487913	8.531344	9.923818	9.986168	10.50328	12.20015	10.55777	6.187468	6.90577	5.044544	17.78132	15.48898	12.01515	7.49864	7.557167	5.946697	7.148652	3.047914	14.42178
LOC107821540	0	0	0	0	0	0	0	0	0	0	0	0.103151	0	0	0	0	0	0	0	0	0	0	0	0.127616	0	0	0	0	0	0
LOC107764161	3.909565	2.164671	4.056276	0.209465	0.42778	0.723142	0.070519	0.125715	0	0.163971	0.162125	0.035515	0	0.114654	0.151014	1.863957	2.61951	3.44755	0.17063	0.124465	0.115019	0.105322	0.129782	0.065908	0	0	0	0	0	0
LOC107814043	0	0	0	0	0	0	0	0	0	0	0	0	0.082709	0	0	0	0	0	0	0	0	0	0	0	0	0	0	0	0	0
LOC107805527	0.815436	3.803278	1.584468	1.26021	0.80644	0.876693	0.603478	0.409841	0.1351	0.327416	0.672687	0	0	0.366306	0.246159	2.895339	2.038959	2.109916	1.752243	2.678057	0.481212	0.320467	0.380789	0.20054	0	0.200227	0.170079	3.430305	0	0
LOC107815433	0	0	0.205102	0	0	0	0	0	0	0	0	0	0	0	0	0	0	0.087953	0	0	0	0	0	0	0	0	0	0	0	0
LOC107791085	0.544488	0.496234	0.094662	0.197311	0.770878	0.351235	0.456686	0.093045	0.408952	0.106189	0.229077	0	0	0	0	0.243452	0.420817	0.487127	0.663011	0.36848	0.595899	0.363773	0.864494	0.682919	0.13768	0	0	0	0	0
LOC107776817	1.572133	1.854218	2.025816	1.491297	1.391126	1.431728	1.272076	1.32747	1.232884	1.009995	1.296918	0.93754	0.967713	0.827293	0.797305	1.433432	1.268655	1.558192	0.656885	0.985702	0.759077	1.421053	1.272524	1.101906	0.374147	0.463237	0.393487	0	0.267134	1.143616
LOC107761844	0	0	0	0	0	0	0	0	0	0	0	0	0	0	0	0	0	0	0	0	0	0	0	0	0	0	0	0	0	0
LOC107826160	6.657204	4.733766	3.942768	0.861584	0.7768	0.865169	0.674951	1.500157	1.57969	0.99871	1.128536	1.29789	2.198836	0.877907	0.675769	6.650953	6.855469	6.435835	0.445402	0.24754	0.467037	0.733135	0.516228	0.688165	0.092492	0	0.864646	0	1.056598	0
LOC107790360	0.097723	0.089063	0.169898	0.088532	0.057648	0.035022	0	0.11133	0	0.031764	0.022841	0	0.108787	0.142149	0	0.024275	0	0	0	0	0	0	0	0	0	0.045325	0	0	0	0
LOC107787728	1.676791	0.946021	2.082284	1.012725	0.847849	1.201837	1.004569	1.023354	1.124461	0.389306	0.279945	0.337286	0.266661	0.958203	0.245859	1.07104	2.237034	1.012001	0.405117	0.405271	0.873864	0.333412	1.690332	0.834563	0	0.111102	0.094373	5.710204	0	0
LOC107832130	0	0	0	0	0	0	0	0	0	0	0	0	0	0	0	0	0	0	0	0	0	0	0	0	0	0	0	0	0	0
LOC107792132	110.9091	91.91484	110.3097	38.86723	31.75295	24.81267	59.2855	61.56861	111.8696	59.61321	73.20379	87.89254	106.2303	193.7678	108.8589	60.32934	76.8135	69.52648	10.13106	10.40421	16.95746	24.53031	33.75839	40.00183	11.52516	5.838663	20.00915	10.34777	19.85354	0
LOC107785288	6.203637	8.187415	5.952308	5.805752	4.764373	4.121351	8.590283	9.200964	7.664929	3.966305	5.601643	4.375744	6.124166	4.820506	6.607516	7.108887	9.385631	5.999507	3.355365	4.455721	4.109927	9.506314	9.36954	5.841752	1.368875	3.42019	1.729292	2.092678	2.535836	9.046703
LOC107781978	15.90246	20.46968	18.8591	16.98124	11.48451	17.36148	17.07366	14.38127	16.52041	13.53524	14.52289	13.04242	13.14025	13.44383	14.86354	19.34366	19.48106	18.63953	9.926024	12.96699	9.419678	15.83627	14.09933	11.13795	6.252756	5.360693	8.36429	3.908079	6.840423	8.447364
LOC107820504	0	0	0	0	0	0	0	0	0	0	0	0	0	0	0	0	0	0	0	0	0	0	0	0	0	0	0	0	0	0
Novel02015	0	0	0	0	0	0	0	0	0	0	0	0.028476	0	0	0	1.281021	0.586138	1.005182	1.368117	1.368639	0.952965	1.604499	0.951401	0.880748	1.278454	1.3132	0.836604	2.410485	2.109571	0
LOC107809109	0.542496	1.062474	0.712388	1.171173	1.399255	0.806622	1.500582	1.104567	0.726051	0.585279	1.796777	0.351051	0.758106	0.617021	2.262755	1.574069	0.747117	1.239179	1.077551	1.640376	0.221066	3.489472	1.038485	0.615274	1.006931	1.638173	0.122781	0	0.333418	0.713689
LOC107765981	1.003218	1.366085	1.118309	2.865596	3.728394	2.817001	2.60352	2.319418	1.16348	1.657294	2.987622	0.279192	2.181037	1.995782	2.640814	2.339545	0.934986	1.962246	4.071984	5.111887	0.947244	3.21326	1.936379	1.011555	4.267699	5.205901	0.739333	0	2.102404	8.757241
LOC107803929	0.618506	0.951231	0.655266	0.910545	1.145899	0.706531	1.199651	1.222084	0.762144	0.77275	1.301091	0.865443	1.118861	1.201916	1.269636	0.993841	0.989658	0.691684	0.804135	0.817522	0.564088	0.920071	0.733101	0.424241	0.464301	0.699243	0.365512	0	0.093053	0
LOC107814870	0	0.044325	0.042277	0	0.043035	0.104577	0	0	0	0	0	0.041088	0	0.053058	0	0.072486	0.093971	0.181297	0	0.041142	0	0.040616	0	0.050833	0	0	0	0	0	0
LOC107781838	0.737094	0.629785	1.40162	2.337195	3.872613	1.485875	2.743408	3.542588	2.811317	6.491301	5.749967	5.818519	4.154002	4.221702	9.50395	2.179969	0.467312	1.219281	8.063861	8.534581	7.730787	5.21308	2.763205	2.792739	10.83347	18.42898	5.281566	4.941812	8.760691	12.81815
LOC107776678	0.106607	0	0	0.096581	0.031444	0.114616	0	0	0	0	0	0	0	0	0	0.052963	0.171653	0.158961	0	0	0	0.029677	0	0	0	0	0	0	0	0
LOC107828827	4.994079	5.292427	3.903757	3.437128	3.083086	3.454631	3.247093	3.770905	3.45289	5.39837	3.040367	4.709734	5.085415	5.025947	1.788065	3.490797	5.086506	3.983063	3.692704	3.373168	5.296145	2.748126	2.561109	4.32961	8.957119	3.716852	6.680462	30.45442	14.35141	7.181196
LOC107775550	0.126725	0.192491	0.07344	0.420958	0.074757	0.317905	0.14172	0.14437	0.158634	0.247146	0.414679	0.463931	0.094048	0.046084	0.086712	0.094436	0.163236	0.031493	0.085728	0	0.07705	0.070554	0.1863	0.39736	0	0	0.149779	0	0	0
LOC107813018	2.040054	2.537635	1.965092	1.698333	1.488049	0.889179	2.751603	3.156383	2.562351	0.967773	2.261716	1.048071	2.240327	1.744388	1.527949	3.204852	2.18393	3.103553	1.063027	1.04944	0.829711	3.315313	3.696193	3.198396	0.383402	0.421952	0.228085	0	0.265447	0
LOC107818613	0.408502	0.45565	0.408103	0.44189	0.312914	0.39986	0.363084	0.411549	0.360623	0.214034	0.273616	0.319359	0.312217	0.286018	0.306634	0.517962	0.359306	0.368194	0.63106	0.598291	0.355879	0.483722	0.45713	0.732853	0.31605	0.381762	0.245012	1.308076	0.704481	0.75398
LOC107760586	1.50873	2.049878	2.011513	1.987358	1.867395	1.651923	2.134939	2.23813	1.8162	1.254583	2.083393	1.063484	1.792882	1.484384	1.881023	2.31759	1.368139	2.056423	2.047534	1.667779	1.012996	2.666828	2.228885	1.905853	0.713847	1.120478	0.459474	1.323871	0.980358	0
LOC107808645	0.077143	0.070306	0.067058	0	0	0	0	0	0	0	0	0	0	0	0	0	0.074526	0	0	0	0	0	0	0	0	0	0	0	0	0
LOC107770892	0	0	0	0	0	0	0	0	0	0	0	0	0	0	0	0	0	0	0	0	0	0	0	0	0	0	0	0	0	0

LOC107772128	7.884699	9.244213	8.266133	10.23012	9.396027	10.26613	9.604148	8.70042	8.927642	8.036302	7.334631	8.636146	7.542352	9.336648	7.889307	6.613839	8.689061	7.17812	6.553459	6.133639	6.395996	7.5443	8.457628	8.696623	4.157782	4.079662	4.401978	2.833505	5.913328	4.899727
LOC107789764	0.031403	0.02862	0.027298	0	0	0	0	0	0	0	0	0	0	0	0	0.023401	0.121351	0.023412	0	0	0	0	0	0	0	0	0	0	0	0
LOC107820091	0.570674	0.631549	0.425207	1.366363	1.55098	1.183265	1.059866	0.696571	0.95674	1.232205	0.571653	0.964243	1.315943	1.423035	0.962262	0	0	0	0	0	0	0	0	0	0.773042	0	0	0	1.766205	0
LOC107806173	0	0	0	0	0	0	0	0	0	0	0	0	0	0	0	0	0	0	0	0	0	0	0	0	0	0	0	0	0	0
LOC107821854	0.096375	0.307418	0.25133	0.043655	0.213197	0.259037	0.080834	0.041173	0	0	0.067578	0.04071	0	0	0	0.107728	0.279319	0.359258	0	0.081527	0	0	0	0	0	0	0	0	0	0
107814000	0	0	0	0	0	0	0	0	0	0	0	0	0	0	0	0	0	0	0	0	0	0	0	0	0	0	0	0	0	0
LOC107811894	0	0	0	0	0.11374	0	0.269529	0.384396	0.181018	0.062671	0.22533	0.16289	0.214638	0.210345	0.197894	0.143681	0	0.143747	0.391298	0.163103	0.175845	0.429386	0.11338	0.067175	0.731307	0.178853	0.151923	4.596188	1.856502	0
LOC107801021	0.601202	0.410941	1.012562	0.323391	0.349114	0.444377	0.346675	0.481578	0.546797	0.146562	0.302999	0.063489	0.27189	0.163971	0.25068	0.630024	0.671564	0.644319	0.247836	0.286074	0.119942	0.59622	0.464013	0.648013	0.166272	0	0.199849	0	0.27135	0
LOC107788242	24.75482	24.14132	26.69064	21.93908	19.94703	23.01443	20.36172	20.74247	21.55679	13.38144	15.21978	13.74031	12.74697	16.24291	13.93126	22.83636	25.47493	23.53779	15.62383	14.1882	14.23321	20.59849	24.78897	25.405	4.762681	6.822345	8.409967	2.138067	5.901351	5.545753
107804612	0	0	0	0	0	0	0	0	0	0	0	0	0	0	0	0	0	0	0	0	0	0	0	0	0	0	0	0	0	0
LOC107790466	6.934895	7.203025	6.028357	7.265426	7.61057	7.539165	5.7841	5.660541	7.129127	8.122433	6.411226	8.280862	8.582593	8.537749	6.163446	7.67966	5.913694	7.336567	6.604607	6.88242	8.550766	5.953286	7.347277	9.799445	10.82507	5.282912	10.62338	11.08253	11.19119	19.16402
LOC107831815	30.18495	43.93486	21.91739	24.04842	27.70469	11.93107	20.3284	23.23704	12.50248	30.61727	42.47666	30.35129	32.31751	24.98828	38.93887	71.31876	54.69154	54.20855	26.33545	22.80571	28.58177	27.50664	18.14163	13.76415	12.27201	36.35895	3.777478	8.465296	19.80356	85.9999
LOC107799691	17.14038	15.76113	17.19973	12.15863	12.75787	16.49038	12.60747	12.28624	14.90413	10.76877	11.61553	12.27779	10.88508	13.3447	9.32752	12.25868	14.3734	14.98016	7.704224	8.012071	9.512279	11.5924	12.61603	15.91119	8.338593	4.001608	11.55689	2.742235	9.968843	9.483805
LOC107772909	0	0	0	0	0	0	0.158033	0	0	0	0	0	0	0	0	0	0	0	0	0	0	0	0	0	0	0	0	0	0	0
LOC107773826	0	0	0	0	0	0	0	0	0	0	0	0	0	0	0	0	0	0	0	0	0	0	0	0	0	0	0	0	0	0
LOC107781193	2.68148	2.174602	1.343262	1.708735	1.367348	0.928398	11.6075	8.640276	7.829826	1.59546	2.055534	1.535857	4.098135	3.198066	2.355695	2.167585	3.92971	2.541302	0.737893	0.422912	0.953352	7.875765	5.712663	2.090136	0.114922	0.442671	0.134292	1.625117	0.437614	1.405084
LOC107814808	0.769482	0.951748	0.191113	1.692986	1.459047	0.768196	1.106397	1.549743	0.464415	0.267979	0.770802	0.278606	0.244742	0.779505	0.620538	0.860129	0.637187	0.491727	1.505862	1.394848	1.553943	0.504912	1.308988	0.459579	1.11184	0.917724	0.389771	0	3.175339	10.19534
LOC107806533	45.12232	51.47619	47.02163	125.7393	96.88629	137.9757	63.48002	62.20209	60.81457	98.19772	53.6391	92.61667	65.48571	71.35592	77.6807	39.90684	35.97099	34.0775	102.6892	93.19522	98.09134	53.68204	31.88841	36.93795	72.20803	97.4068	42.03609	116.0518	126.0874	172.806
LOC107763530	2.127507	2.699818	2.013267	4.879528	5.885973	5.298499	5.80505	4.901156	4.702733	5.435946	5.835051	4.277302	7.314967	5.523406	5.224091	3.512008	2.991966	3.57385	6.312578	5.51308	3.217496	5.262739	4.560855	5.038442	6.537273	6.220316	4.869936	7.703637	10.24258	23.31212
LOC107771008	0	0	0	0	0	0	0	0	0	0	0	0.052369	0	0	0	0	0	0	0.125802	0	0	0	0	0	0	0	0	0	0	0
LOC107787785	8.324101	8.079579	7.664864	5.06112	4.337742	5.030844	4.525476	4.256523	5.155232	7.154799	8.85016	5.970171	7.955374	7.15382	8.510957	11.49299	11.05553	11.2372	4.441393	4.766218	2.598313	3.495775	4.113433	3.643167	1.972029	3.100446	3.404882	1.138224	2.835147	0
LOC107789719	1.953715	1.903368	1.991137	0.762917	0.357678	0.760519	0.39554	0.172687	0.347872	0.131387	0.094479	0.113831	0.187492	0.587974	0	1.029178	1.789826	1.607258	0.205086	0.34194	0.30721	0.337572	0.68338	0.176036	0	0	0.079625	0	0	0
LOC107803137	0.747428	0.899169	0.753679	0.988613	1.216927	0.883934	0.476444	0.561988	0.659615	0.568493	0.471687	0.568303	0.915252	1.027413	0.460284	0.646102	0.476572	0.780135	1.365189	0.998991	0.9816	0.686616	0.804323	0.828084	0.81268	0.291198	0.318024	0	0.863612	0
LOC107769800	2.4719	2.532109	2.326354	6.422127	6.67036	5.578752	7.607771	5.882344	6.655312	5.796947	7.004815	5.315748	8.619128	7.109538	6.940305	3.958151	3.552491	3.899041	4.622759	4.821529	4.341145	6.602502	5.423918	7.366545	2.918606	2.558282	3.718383	0	6.491239	0
LOC107792009	23.90637	18.55038	23.28094	10.72682	9.878486	19.88289	7.140912	7.274444	9.26359	9.181743	8.183902	8.478831	6.653226	9.903276	5.786992	12.85709	28.86946	20.13519	9.955153	9.157651	10.13017	6.168381	11.83657	11.4327	3.920685	2.667394	7.663613	0	3.528819	0
LOC107825272	6.801897	5.612056	5.218445	1.143737	1.276708	1.412714	0.47542	0.37424	0.290269	4.698169	4.661097	3.526219	2.896851	1.967566	3.781527	8.140771	7.865533	6.761471	1.69938	0.871809	1.174895	0.172135	0.409072	0.430869	1.009809	0.358498	0.669941	0	3.101018	3.186148
LOC107820114	0	0	0	0	0	0.060359	0	0	0	0.10949	0	0	0	0	0	0	0	0	0.056968	0	0	0	0	0	0	0	0	0	0	0
LOC107799528	7.707781	5.58954	5.763622	6.006761	4.473564	6.504698	5.56116	4.178049	5.446756	3.636803	4.416745	4.481203	4.336336	3.255023	3.062344	4.632135	5.444654	5.313948	2.859409	2.453859	4.384068	4.706601	5.482881	5.890534	0.838278	1.153206	1.077524	5.927047	2.394065	0
LOC107802855	0	0	0	0	0.039113	0	0	0	0	0	0	0	0	0	0	0	0	0	0.065909	0.044853	0.037392	0	0	0	0	0	0	0	0	0
LOC107823826	6.680489	6.411722	6.475293	5.67727	4.080391	4.57636	5.454456	4.495674	6.327441	2.133007	2.404363	1.698153	0.987188	1.22543	2.42714	6.872708	4.454914	4.804284	2.4596	2.000437	4.367347	7.899541	7.039871	8.589025	0.074745	0.164521	0.838492	8.455742	0.569243	0
LOC107788388	0	0	0	0	0	0	0	0	0	0	0	0	0	0.087109	0	0	0	0	0.081023	0	0	0	0	0	0	0	0	0	0	0
LOC107814703	0.112645	0.102662	0.391678	0.510252	0.199351	0.36332	0.28344	0.673727	0.317267	0.109843	0.789865	0.190331	0.376193	0.368669	0	0.167886	1.088242	0.671851	0.114304	0.571738	0.205468	0.470362	0.89424	0.235472	0.284834	0.313474	0.532548	0	0	0
LOC107771729	0	0	0	0	0	0	0	0	0	0	0	0	0	0	0	0	0	0	0	0	0	0	0	0	0	0	0	0	0	0
LOC107804572	0	0	0	0	0	0	0	0	0	0	0	0	0	0	0	0	0	0	0	0	0	0	0	0	0	0	0	0	0	0
LOC107823980	0	0	0	0	0	0	0	0	0	0	0	0	0	0	0	0	0	0	0	0	0	0	0	0	0	0	0	0	0	0
LOC107778587	0	0	0	0	0	0	0	0	0	0	0	0	0	0	0	0	0	0	0	0	0	0	0	0	0	0	0	0	0	0
LOC107787386	9.96538	9.642846	10.74816	10.97865	9.253482	11.30922	11.63468	11.22152	10.33773	10.85721	12.3148	12.05688	13.52461	11.03951	10.48074	10.49755	11.5291	10.63994	8.301974	7.649468	6.087114	8.964506	10.74349	13.30909	7.544007	4.921609	7.852185	4.39917	7.551918	3.803546
LOC107801908	9.045413	7.220828	5.796799	15.81116	14.74212	10.43477	2.381251	2.106103	3.40929	3.133288	2.654345	1.877916	5.193989	5.258185	4.065969	13.56331	9.163867	10.82936	16.45906	16.68129	24.32719	2.187185	2.213057	3.266436	2.031239	2.08236	2.653228	4.721715	8.476464	8.164839
LOC107826376	0.295811	0.770273	0.238775	0.593407	0.430024	0.249883	0.974717	1.299854	0.476093	0.988984	1.437146	0.678326	0.517474	0.898994	2.450589	0.582589	0.22454	0.346563	1.565167	1.841023	1.040599	1.058742	0.857326	0.507942	2.377544	4.586401	1.573314	3.0221	2.339661	0
LOC107767645	0	0	0	0	0	0	0	0	0	0	0	0	0	0	0	0	0	0	0	0	0	0	0	0	0	0	0	0	0	0
107812176	0	0	0	0	0	0	0	0	0	0	0	0	0	0	0	0	0	0	0	0	0	0	0	0	0	0	0	0	0	0
LOC107776336	1.911081	1.569996	1.403883	0.682783	0.59544	1.012853	0.29349	0.229983	0.151623	0.104988	0.264235	0.09096	0.14982	0.205553	0.110506	1.103207	0.858123	1.404724	0.43701	0.478162	0.245484	0.17983	0.379876	0.112533	0.034031	0.074905	0.19088	0	0.129586	0
LOC107790365	0	0	0	0	0	0	0	0	0	0	0	0	0	0	0	0	0	0.211088	0	0	0.516446	0	0	0	0.357967	0	0.334641	0	0	0
LOC107770739	9.644171	9.342656	9.165149	7.718817	7.26069	8.410943	6.241149	6.281023	6.37394	6.291444	6.541811	6.058519	9.509766	8.04427	6.045245	9.012876	8.665536	9.838255	6.706653	5.895279	5.556224	6.082816	6.543689	8.223845	4.121232	3.50337	4.251239	1.60768	8.117215	2.780017
Novel02044	1.163995	0.858773	1.108201	1.305596	1.961865	1.787767	1.208743	0.947189	1.717272	1.675538	1.982185	1.826268	3.023475	2.963009	1.536033	1.445684	1.392259	1.446346	2.249793	2.016207	2.224269	1.573848	1.02672	1.506275	2.172426	1.696738	1.048189	0	3.20222	0
LOC107773754	16.8967	11.15406	14.44976	24.57483	33.53942	34.61362	17.31281	20.40689	18.09281	27.87631	21.58253	34.99255	29.38371	32.23366	21.18574	20.38452	25.14721	23.15162	39.20318	31.90915	44.18942	17.31442	16.15272	17.08765	19.45725	25.35324	21.05003	22.86073	34.73725	25.41279
LOC107823854	2.197752	1.00149	0.742956	1.069269	0.972358	1.050157	0.750994	0.904135	0.573152	0.714362	0.741994	0.515756	0.770216	1.110018	0.584814	0.363948	0.550463	0.424801	0.867272	0.413144	0.593892	0.645789	0.323094	0.510464	0	0	0.048103	0	0.19594	0
LOC107818754	0	0	0	0	0	0	0	0	0	0	0	0	0	0	0	0	0	0	0	0	0	0	0	0	0	0	0	0	0	0
LOC107785412	0.271599	0.388974	0.539646	0.175753	0.034333	0	0.488146	0.298365	0.582832	0.151339	0.081619	0.065558	0.431925	0.126986	0.358408	1.590252	1.087033	1.677761	0.2756	0.229754	0.460019	1.166499	1.060944	0.932732	0.049055	0.053987	0.458583	0	0.186796	0
LOC107793949	0.314279	0.143213	0.273196	10.4872	9.269812	2.42153	13.92681	17.81237	8.064934	6.078149	7.235558	2.920624	1.574367	1.8286	3.978325	1.327138	0.30362	0.078103	8.610521	8.15298	11.03515	7.742636	14.92338	4.927258	12.91367	9.18321	7.119496	3.745894	2.269573	9.716159
LOC107782597	10.10231	7.441279	10.22529	6.519359	6.367638	9.670944	7.196449	6.917171	8.380131	5.195403	4.560766	6.488717	4.77571	5.284098	3.906028	5.156335	10.22761	6.861067	5.125561	5.502126	7.320293	6.818702	8.239532	6.65357	2.449499	1.925564	4.743332	4.94834	3.997487	4.278361
LOC107785076	1.068333	1.088202	0.792109	1.707981	2.029683	1.452627	1.739405	1.396067	1.209498	1.19496	1.498232	1.831661	2.168694	2.536675	1.999515	0.796123	0.880321	0.866766	2.263795	1.807473	2.808386	1.548218	0.748329	1.313673	3.575378	2.754412	2.488231	6.74126	2.117845	1.942843
LOC107786666	1.442046	2.243512	1.38016	17.70925	31.86176	11.2598	19.02223	28.96111	10.13341	37.81053	29.27267	28.40193	33.98707	38.26533	67.5752	3.093586	1.787156	2.019897	58.62022	45.96081	49.49821	36.49364	15.18667	10.33744	143.9763	208.6356	65.05919	61.45932	90.88392	265.6903
LOC107813650	14.5563	10.44588	11.35825	11.83739	10.19274	10.59751	10.23825	11.80074	9.89985	9.555959	11.57316	14.91201	15.24733	12.25402	9.469962	10.80467	16.38792	12.04866	13.37507	10.71383	18.44994	11.1513	11.27258	9.943166	7.535328	8.930911	9.482728	12.29506	12.69151	14.17383
LOC107825374	141.0874	135.588	160.8093	51.15685	48.47118	58.83445	56.89367	54.39488	74.91601	53.68122	33.78584	23.18504	20.81196	27.44208	20.53105	122.5837	116.714	129.2026	33.43392	38.08511	26.22198	71.85166	85.07302	111.2037	81.79794	10.23845	83.45752	31.1833	129.6299	58.97772
107809654	0	0	0	0	0	0	0	0	0	0	0	0	0	0	0	0	0	0	0	0	0	0	0	0	0	0	0	0	0	0
LOC107798141	1.33028	0.454645	0.795014	0	0	0.178777	0	0.284157	0.078058	0	0	0	0	0	0.17067	0.743494	0.321291	0.619862	0	0	0	0	0.220011	0	0	0	0	0	0	0
LOC107787867	0.794636	1.14667	1.151267	0.779892	1.69927	1.637468	0.388789	0.792118	0.683869	1.162306	0.882232	1.174829	1.99035	1.80606	0.815593	0.641511	0.575764	0.938022	1.142318	1.624501	1.207869	0.94013	0.934562	0.484489	1.255827	0.552839	1.878392	0	1.27522	0
LOC107811857	5.20813	6.256222	5.046207	4.556068	4.661316	4.155411	4.117952	3.939943	4.315196	3.376022	5.284332	2.836659	4.917301	4.184029	3.905725	7.617641	4.584568	6.530807	2.680282	3.130712	1.810135	4.623623	4.580752	4.148942	0.792423	0.789043	1.428661	1.067206	1.724272	1.845424
LOC107769975	0.676353	1.301314	0.82747	0.408494	0.33249	0.269319	0.378191	0.321052	0.188145	0.024427	0.105391	0	0.055772	0	0.051421	1.325383	0.774416	0.597629	0.457544	0.656911	0.274153	1.861879	0.90593	0	0	0.348554	0	0	0	0
LOC107762796	0	0	0	0	0	0	0	0	0	0	0	0	0	0	0.025169	0	0	0	0	0	0	0	0	0	0	0	0	0	0	0
LOC107780029	8.370856	8.757553	8.137744	11.44267	10.97914	10.25113	10.82231	11.10932	13.76475	10.31198	10.00272	9.436216	14.2811	10.80733	10.32011	8.729469	7.3931	10.11827	10.02714	9.301889	10.05116	9.348537	11.22839	11.98487	6.63805	7.78793	7.522708	12.39776	7.153896	21.43833
LOC107824442	29.63785	47.12259	30.55914	2.897715	1.0646	3.249529	0.443023	1.178415	0.743845	1.516568	2.3457	2.206396	0.391999	1.696705	1.325205	35.49093	47.85339	30.30034	0.923077	0.719877	0.562014	1.494883	3.028393	1.748233	0	0.040831	0.346827	0	0	0
LOC107800927	0.125913	0	0	0	0	0	0	0	0	0	0.08829	0	0.140168	0.137365	0	0	0.243285	0	0.127768	0	0.114835	0	0.222127	0.131604	0.159192	0	0	0	0	0
LOC107794091	3.016884	3.593858	3.5724	4.97131	4.393176	3.856468	5.578814	5.277197	5.530954	3.914736	3.96437	3.271227	4.680657	3.913245	5.095728	4.903523	3.121108	3.542071	4.772775	4.963487	4.094676	5.098473	5.217415	4.270539	4.835399	5.652121	4.155321	0	3.545315	7.344035
LOC107814135	0.418619	0.14307	0.341153	0.071109	0.023151	0.028129	0.197502	0.31297	0.368455	0.102052	0.476996	0.419973	0.495139	0.34252	0.187976	0.116983	0.303316	0.195062	0.026549	0	0.071585	0.10925	0.161547	0.246117	0	0	0	0	0	1.617742
Novel02631	0	0	0	0	0	0	0	0	0	0	0	0	0	0	0	0	0	0	0	0	0	0	0	0	0	0	0	0	0	0
LOC107764329	0	0	0	0	0	0	0	0	0	0	0	0	0	0	0	0	0	0	0	0	0	0	0	0	0	0	0	0	0	0
LOC107823624	0	0	0	0	0.103458	0	0	0	0	0	0	0	0	0	0	0	0	0	0.098906	0	0	0	0	0	0	0	0	0	0	0
LOC107784339	7.088671	7.414688	6.789304	8.165268	7.950203	6.875803	8.330924	8.003135	10.36132	6.636407	7.917212	5.737683	7.922709	7.517289	5.286078	6.168172	6.342493	6.329228	6.030138	6.247879	5.200379	7.361515	9.896951	10.64778	2.862191	4.488719	3.796128	0	3.26969	3.499427
LOC107766655	799.7054	668.3607	757.4085	502.2453	509.3912	357.9697	577.9017	610.7917	726.8731	620.3	605.6051	603.3485	823.6321	687.1071	803.7051	826.7702	713.7612	746.0235	769.6479	674.3216	898.0132	557.1806	580.6114	506.4221	643.8413	502.5727	639.9653	994.0727	1058.019	709.5191
LOC107812110	0	0	0	0	0	0	0	0	0	0	0	0	0	0	0	0.057234	0	0	0	0	0	0	0	0.080275	0	0	0	0	0	0
LOC107761908	0	0	0	0	0	0	0	0	0	0	0	0	0	0	0	0	0	0	0	0	0	0	0	0	0	0	0	0	0	0
LOC107766911	5.481002	5.584321	6.000596	6.417687	4.827074	5.11446	8.934088	8.195454	7.451709	5.470722	7.142703	5.897338	6.821047	6.17694	6.103191	4.662437	6.319163	4.934423	3.410502	3.892953	3.654758	8.269418	8.118489	7.458173	3.105269	2.805945	3.14738	7.395644	3.734079	3.197157
LOC107806142	22.45556	25.50835	14.34353	3.124597	2.612761	4.188252	1.311127	1.18724	1.024994	8.395868	6.820303	4.695604	6.380664	5.197347	6.672393	13.86788	22.38912	15.68713	2.668898	2.413838	2.828708	0.683819	0.963006	0.881766	19.79503	2.14056	19.12101	12.42125	24.13042	3.068422
LOC107782797	18.62703	15.76365	17.53127	18.52459	17.22599	24.61884	14.74228	16.45393	19.13147	18.02713	14.19064	20.46945	20.11223	19.25164	12.00282	14.61146	16.7099	15.03581	15.41958	15.34252	16.28564	12.69035	17.66563	20.9328	8.587864	5.846199	13.98739	10.01576	25.95917	4.329839
LOC107825145	0	0	0	0.16307	0	0	0.150973	0	0	0	0	0	0	0	0	0	0	0	0	0	0	0	0	0	0	0	0	0	0	0
LOC107793088	12.36041	10.5263	12.09506	7.924199	6.813401	7.915276	9.88567	11.22474	10.6216	8.726869	9.709075	9.900323	11.65446	10.97926	10.43334	12.25615	14.35553	11.25464	5.414517	5.056617	5.143646	9.278968	9.502624	9.318691	3.202318	3.430323	4.35077	4.830295	4.227302	4.176299
LOC107773360	0	0	0	0	0	0	0	0	0	0	0	0	0	0	0	0	0	0	0	0	0	0	0	0	0	0	0	0	0	0
LOC107777386	0	0	0	0	0	0	0	0	0	0	0	0	0	0	0	0	0	0	0	0	0	0	0	0	0	0	0	0	0	0
LOC107787776	0	0	0	0	0	0	0	0	0	0	0	0	0	0	0	0	0	0	0	0	0	0	0	0	0	0	0	0	0	0
LOC107790390	1.546967	2.052968	1.792994	3.933961	4.346725	2.8595	5.94122	4.68417	4.102282	1.508488	5.747188	2.178201	3.595272	2.635126	4.373316	2.305603	2.228641	2.387595	1.26682	2.112171	1.658379	6.300907	3.11208	2.326052	0.411755	0.528682	0.064154	0	1.698587	1.678093
LOC107810224	9.445531	10.64316	11.04726	6.262294	5.774637	6.046901	5.581672	5.97952	6.570293	4.52156	3.853505	5.331989	3.728009	5.152305	4.274452	8.222622	9.374045	9.122648	5.92507	4.685203	5.951825	5.378325	5.49127	6.776102	2.876942	2.329854	4.110329	6.140809	3.307211	2.654688
LOC107811014	23.34239	13.96888	18.09081	14.96853	14.99345	12.01883	17.1015	20.66525	25.61135	31.5374	20.65682	26.78872	26.61122	28.0733	20.34986	14.04157	14.73947	17.35032	22.18802	21.88719	20.19857	16.73417	20.65155	22.5603	30.22301	32.67489	29.417	35.19632	48.74252	26.08365
LOC107828270	18.96038	2.237704	23.59633	0.617883	2.896816	9.825733	8.351877	11.07212	48.9203	1.064102	0.286943	5.992442	1.366638	2.083366	15.12032	0.406599	20.42579	4.779721	0.55366	0.11539	4.354148	11.04984	24.42471	4.134563	6.380953	1.897985	3.708071	29.26479	3.940232	134.9466
LOC107810814	0	0	0	0	0	0	0	0	0	0	0	0	0	0	0	0	0	0	0	0	0	0	0	0	0	0	0	0	0	0
LOC107815804	0	0	0	0	0	0	0	0	0	0	0	0	0	0	0	0	0	0	0	0	0	0	0	0	0	0	0	0	0	0
LOC107788490	3.085024	3.290192	2.967038	1.040645	2.787913	0.987976	0.660649	1.26188	1.140055	9.184878	2.117194	4.741273	4.932227	4.368131	2.357945	2.225592	2.219441	2.006397	11.75591	7.579286	14.21763	1.918584	1.157955	1.475022	33.69288	21.91958	76.02818	37.55282	21.17257	4.058546
LOC107827078	0	0	0	0	0	0	0.089295	0	0	0	0	0	0	0	0	0	0	0	0	0	0	0	0	0	0	0	0	0	0	0
LOC107759743	0.256136	1.167181	1.113267	0.348069	1.359875	1.239198	0.53708	1.203671	0.84165	0.374647	1.43682	0.865562	1.85337	1.397158	0.262891	0.095436	0.86607	0.4774	0.519818	0	0.7008	0	0.677786	0.803139	0	0.356394	0.151366	9.158664	0	0
LOC107820416	0	0	0	0	0	0	0	0	0	0	0	0	0	0	0	0	0	0	0	0	0	0	0	0	0	0	0	0	0	0
LOC107809312	0.885065	0.219989	0.699426	2.1139	0.854361	1.038058	0.944799	0.618728	0.604319	0.941509	1.015541	0.543802	1.164406	1.843347	0.412912	0.239837	0.310926	0.299933	0.57152	0.612576	0.440288	0.134389	0.141943	0.672778	2.543162	0.671729	0	0	0	0
LOC107823412	0.689856	0.58381	1.028018	0.357128	0.261613	0.37084	0.041329	0.252613	0.23131	0.432448	0.17276	0.208146	0.329124	0.322542	0.050575	0.330481	0.999688	0.624527	0.100003	0.125051	0.04494	0.246907	0.304249	0.206011	0	0.137126	0.174719	0	0	0
LOC107825618	0	0	0	0	0	0	0	0	0	0	0	0	0	0	0	0	0	0	0	0	0	0	0	0	0	0	0	0	0	0
LOC107807977	0	0	0	0	0	0	0	0	0	0	0	0	0	0	0	0	0	0	0	0	0	0	0	0	0	0	0	0	0	0
LOC107768372	0	0	0.042714	0	0	0	0	0	0.092263	0	0	0	0	0	0	0	0.142411	0	0	0	0	0	0	0	0.062124	0	0	0	0	0
LOC107810992	0.574859	0.847506	0.529107	0.291031	0.239375	0.527155	0.297804	0.202248	0.158736	0.082435	0.106701	0.114272	0.112931	0.387353	0.364424	1.083563	0.457357	0.718504	0.291663	0.157329	0.13878	0.25416	0.268445	0.19439	0.320645	0.423463	0.05995	1.209133	0.569795	1.045423
LOC107779649	2.757694	3.336103	3.495923	2.587557	2.469238	2.788378	1.955033	1.879388	2.20377	1.76072	2.186919	1.567052	1.589771	1.325176	1.516297	2.727816	2.093272	3.377677	1.965484	2.43002	1.46712	2.330439	2.331115	2.539205	1.992322	1.393243	1.125259	0	1.02735	1.014954
LOC107811627	75.87224	62.71647	68.7079	65.9233	56.8678	64.57638	59.9806	53.54483	68.58198	38.75114	41.72119	42.71499	45.32109	45.98874	40.98081	57.59188	67.48111	61.28955	38.1926	33.37819	38.56025	56.26436	68.39064	79.23608	17.92408	24.52696	19.7218	26.9166	38.95874	29.0903
LOC107775257	0.065149	0.277084	0.037755	6.748143	9.166045	5.533405	7.486123	8.999181	7.829083	3.070542	2.603902	4.274746	1.668069	1.611018	1.203608	0.194196	0.104899	0.129524	26.37736	25.62686	14.43832	6.782814	9.864943	7.467606	3.816388	5.439001	5.338724	3.106048	5.332055	1.342753
LOC107819456	0.109965	0.066813	0.09559	0.099622	0.032435	0.118225	0.030744	0.031319	0	0.035743	0.077107	0.216769	0.204023	0.039989	0.075243	0.027315	0.070823	0	0.148779	0.062015	0	0	0.032332	0	0	0.051003	0.043323	0	0	0
LOC107765924	23.9979	26.72273	28.85382	11.2444	10.68044	10.52839	13.56198	12.52645	16.46341	6.329092	9.481599	7.046631	5.704227	8.229942	8.034955	25.41418	27.77679	25.40459	6.528374	5.418303	7.42531	17.94918	21.21796	22.67253	1.295686	0.911033	3.364601	0	1.644618	3.520347
LOC107825103	3.927715	7.494846	5.094745	5.837854	6.136431	3.16708	7.43801	6.423491	4.897477	3.919784	6.282828	2.488682	4.064954	3.381089	8.314554	8.026212	5.217442	6.336977	4.172398	5.191526	2.378766	10.40419	5.440354	3.72038	2.521708	3.671883	1.269363	2.194431	1.034109	11.3839
LOC107780562	22.6766	28.19352	23.75787	83.16748	65.32472	148.4882	48.98471	52.7466	61.81323	134.5885	129.6063	133.4495	118.8038	122.4011	57.78856	18.08868	25.96282	19.1855	120.0073	150.6838	108.2759	57.30883	60.28852	48.64308	272.2219	142.0195	407.6912	107.6781	100.3867	50.78132
LOC107775296	13.04632	20.25012	11.81988	15.22561	18.92969	11.34308	14.19128	15.2598	9.920492	17.2258	19.15985	16.84091	21.39713	24.61142	26.58116	24.49253	18.28731	19.40484	24.60224	19.19355	21.41724	15.34819	11.8794	8.283241	45.67239	46.02519	26.41462	32.45205	34.09666	71.14761
LOC107812733	3.059298	2.736058	3.504421	4.22211	3.314261	5.410141	2.637908	2.442942	2.442716	1.840106	2.265472	2.246411	1.559598	1.902712	1.966156	2.684616	2.458346	2.685845	4.177836	3.918214	2.268612	2.674288	3.076798	3.257346	2.241203	1.392408	1.993783	6.134105	1.78945	0
LOC107822397	0.135348	0	0	0	0	0	0	0	0	0	0	0	0	0	0	0	0	0	0	0	0	0	0	0	0	0	0	0	0	0
Novel05282	1.005073	0.634154	0.772878	0.595356	0.376265	0.207803	0.194538	0.429381	0.798436	0.188476	0.189742	0.130633	0.473364	0.548243	0.198381	0.57614	0.597529	0.317022	0.117678	0.130803	0.105767	0.419681	0.920638	0.444443	0	0	0	0	0	0
LOC107797343	0.047553	0.325041	0.186016	0.02154	0.126234	0.204502	0.019942	0.060946	0.022322	0.023185	0.083361	0.100435	0.079405	0	0.024404	0.177183	0.229702	0.177264	0.144761	0.020113	0.043369	0.099282	0.104862	0.19881	0	0	0.056204	0	0	0
LOC107787232	2.861362	2.362341	3.423725	4.544061	4.051075	3.510618	3.529335	3.595332	3.824131	4.365826	4.650102	4.237487	7.944534	6.316661	4.042452	4.038809	3.707436	4.040659	3.142626	2.904619	2.732416	2.97997	3.236544	3.553649	2.2557	1.967269	1.869992	0	6.158511	4.162879
LOC107805025	0	0	0	0	0	0	0.087169	0.088799	0.292717	0	0	0	0	0	0	0	0.077483	0	0	0	0	0	0	0	0	0	0	0	0	0
LOC107759951	1.999459	1.6566	1.264063	1.454616	1.393957	1.237685	6.682719	5.953487	6.086614	1.15211	1.529478	0.895787	2.563072	2.445713	1.679064	0.790151	1.199961	0.383963	1.506314	1.486386	1.547246	7.488801	7.241674	4.337988	0.497924	0.505837	0.930957	0	0.43755	0
Novel00613	1.622311	1.639565	1.25665	2.8376	2.586792	2.417679	3.246824	2.552705	2.367586	2.051847	2.736915	2.076215	3.558315	3.539723	2.439942	2.154561	2.048338	2.47888	2.933838	2.90778	1.157291	1.810917	1.671844	1.561331	2.518175	1.698583	2.41104	0	2.861229	0.993168
LOC107774810	84.46142	58.61551	77.51779	31.33716	28.5225	29.99634	40.68122	42.54619	61.45269	16.60555	16.15211	15.6712	15.82576	15.92395	10.06559	56.82229	61.25271	61.836	13.80855	11.38289	20.80024	44.56906	65.58195	72.14885	9.131022	3.173409	17.25176	0	12.07804	9.401218
LOC107796279	0	0	0	0	0	0.071194	0.055541	0	0	0.064573	0.603633	0	0	0.072242	0.135932	0.049347	0	0	0	0.056017	0.060393	0.110604	0	0.138426	0.083722	0.368559	0.156533	0	0.318805	0
LOC107767528	24.55301	27.31864	25.52317	8.434102	6.789413	11.43891	11.66975	9.833794	12.39015	3.341944	7.8192	4.75362	3.872141	3.850506	5.355115	21.19395	21.54592	22.69096	4.51579	4.500201	4.245294	11.49127	16.06256	17.05507	0.258687	0.64057	1.934646	3.658099	0.246264	0
LOC107808933	2.864345	4.027046	2.914551	4.083528	4.086733	3.270499	4.991135	4.477366	3.689796	3.182832	3.861264	2.663742	3.608835	3.996913	5.059337	3.640255	2.831551	3.095634	3.852854	3.681516	1.923809	4.543115	3.231623	2.69211	2.72307	3.738362	1.705834	3.175832	2.672476	2.745841
LOC107804534	5.377149	4.208758	4.536764	4.584871	3.10673	3.91074	4.24475	3.4593	4.127732	2.652543	3.127264	2.832548	2.359156	2.450004	2.272512	3.512069	3.850248	4.904999	2.760276	2.943275	2.884736	3.064173	5.440502	4.231676	1.759573	1.452371	1.570147	2.262013	2.741031	3.911497
Novel05214	4.739121	4.849544	3.97507	3.690821	3.310647	4.380028	4.253846	2.202215	4.605411	2.837605	2.62348	2.59892	2.869216	1.995496	2.389385	4.522925	5.220971	4.46301	2.952853	2.180317	3.488058	3.610592	4.620241	6.343736	0.525587	0.231373	1.375748	5.945863	0.400277	0
LOC107812887	37.71343	43.70651	44.45332	39.50936	38.65855	37.12595	31.30645	31.62665	36.17199	28.1112	34.03528	33.00862	54.55213	44.36048	37.55316	63.95705	38.60841	50.27498	44.80463	42.1106	40.55246	32.14352	42.12976	42.25681	25.01793	27.74938	19.99421	22.19789	59.96157	57.57724
LOC107816182	2.337264	1.483482	1.342396	0.604984	0.110795	0.942317	0.350065	0.356611	0.548582	0.732577	1.346232	0.634688	1.440324	2.185578	0.214188	0.839764	3.145063	2.489328	0.084703	0.141226	0.494841	0.243989	0.662664	0.436234	0.052768	0	0.246648	0	0	0
LOC107822503	0.098644	0.089901	0.042874	0.223415	0.087286	0	3.350834	4.129905	9.214792	0	0.069169	0	3.843401	1.506615	1.113696	0.330792	0.238245	0.367715	0	0	0	3.789474	2.001234	1.391881	0.124716	0.137255	0.174883	0	0.474905	0
LOC107759703	421.3793	416.2221	403.8605	412.593	292.596	436.4812	499.7397	501.083	553.8309	364.8901	399.3692	370.8441	282.8471	344.5941	347.4518	356.7126	371.5506	387.3478	342.8666	385.897	335.3757	450.3945	594.4711	528.2701	228.7234	251.6107	411.5519	317.8334	162.7674	193.8325
LOC107788488	0	0	0	0	0	0	0	0	0	0	0	0	0	0.08234	0	0	0	0	0	0	0	0	0	0	0	0	0	0	0	0
LOC107828937	0.325745	0.059375	0.22653	0.059022	0.230592	0.280172	0.218573	0.389655	0.183494	0	0.411142	0.275198	0.435148	0.426446	0.200601	0.436942	0.314697	0.145714	0.198326	0.551115	0.534753	0.108815	0.40226	0.408562	0	0.09065	0.077001	0	0	0
LOC107792333	0	0	0	0	0	0	0	0	0	0	0	0	0	0	0	0	0	0	0	0	0	0	0	0	0	0	0	0	0	0
LOC107811399	3.577853	2.173848	2.405184	5.575127	6.838386	5.282704	5.921774	5.339585	5.464048	5.023956	3.54576	7.335011	4.673287	5.776827	2.741917	2.66622	3.456506	3.094231	8.955375	9.4834	8.657991	4.422178	8.289504	7.080253	1.869711	2.124084	4.115968	10.23468	3.215344	0
LOC107811831	38.26206	37.99619	33.69208	25.81551	22.66187	30.17855	25.07066	20.48815	22.75659	22.16022	32.1853	21.21693	30.80025	22.8833	36.45564	42.82226	43.49824	44.55202	16.65329	19.6843	13.3285	27.27316	23.77912	27.11413	3.737243	4.47496	6.540238	5.073445	5.920131	7.310879
LOC107761299	0.033416	0.030455	0.07262	0.151367	0.029569	0.017963	0.070069	0.099931	0.094118	0.081462	0.08201	0.084693	0.074399	0.109366	0.205784	0.037353	0.064566	0.024913	0.152588	0.141339	0.030476	0.08372	0.088426	0.10478	0.084496	0.395217	0.07899	0	1.12614	0
LOC107760714	0.805842	0.734426	0.200143	1.772985	7.130624	2.104064	0.482782	0.885258	0.64848	1.122569	0.32289	3.793018	1.665997	2.637404	0.590782	0.686303	0.889728	0.514963	5.373544	2.337214	3.569718	0.9614	0.507719	1.082914	3.638678	0.480545	2.72126	8.232733	6.650764	0
LOC107812706	1.281818	0.796513	1.31685	1.636324	0.850678	1.879243	1.270592	1.418808	1.859843	0.653375	0.714964	0.886022	1.653951	1.112364	0.897016	0.911794	0.759893	0.977369	0.886841	0.640741	0.743935	1.581391	1.464704	1.096164	0.405152	0.162142	0.137728	0	0.701265	0
LOC107784940	0	0.388111	0.123395	6.944402	10.04858	3.662741	2.500261	2.061869	2.39885	0	0.796287	0.479695	0	0.464583	0	0	0	0.21166	10.371	7.685147	18.51297	2.489479	1.502518	2.077134	0.358937	0	1.006645	0	0.683401	0
LOC107826425	10.7526	13.01072	13.11303	6.76217	5.727238	7.407607	7.333139	6.377599	6.811683	4.555437	6.51491	3.483705	5.607278	4.982638	5.81274	12.25266	10.89218	12.80308	4.60804	5.98302	3.189509	10.13494	8.218367	8.074342	1.583831	1.670454	2.097553	1.866414	2.764248	0
LOC107766802	0	0	0	0.171436	0.468849	0.162759	0.412667	0.58207	0.532982	0.442863	0.504224	0.7354	1.263943	1.07351	1.514946	0	0	0.056433	0.691275	0.928454	0.759369	0.442494	0.600898	0.079115	3.875823	3.475616	1.789268	5.413141	2.186487	4.680231
LOC107811161	1.015112	0.462575	0.264725	0.367856	0.538943	1.091369	0	0.260202	0.285909	1.484793	0.284719	0.600316	0.113004	0	0.312565	0	0.490342	0.227043	1.545098	0.858715	0.740639	0.254324	0.179079	0.106099	0	0	0	0	0	0
Novel05064	0	0	0	0	0	0	0	0	0	0	0	0	0	0	0	0	0	0	0	0	0	0	0	0	0	0	0	0	0	0
LOC107815242	0.89252	2.23691	1.454716	1.010722	0.66636	1.019541	2.011849	1.072397	0.576082	0.598345	2.033965	0.164943	1.024615	0.730272	2.089757	1.725121	1.131702	0.998117	0.028302	0.353911	0.127186	2.865003	0.984077	0.233215	0.070526	0.155235	0.164826	0	0	0
LOC107775415	5.861597	6.768028	4.980429	7.765829	7.526604	7.391742	7.430009	6.533394	7.406462	9.046431	10.10542	6.906793	11.922	10.64984	5.699542	7.208956	5.407331	6.719393	7.893324	8.33253	4.843443	5.944139	5.986682	8.153365	11.03268	6.040335	10.78258	7.879456	14.216	4.087572
LOC107824421	5.698351	5.868186	6.660579	4.345764	3.902779	5.226498	4.698445	4.098616	6.468196	1.726631	2.3026	1.713502	1.326039	2.072207	1.354768	4.462338	4.758634	4.512385	1.535411	1.525102	2.642539	4.758842	7.638875	9.859227	0.488437	0.358366	0.875169	0	0.309987	0
LOC107806770	23.96398	20.20004	27.83006	15.36015	13.66166	25.76421	17.47793	18.20941	18.49674	12.93086	13.94762	15.36412	10.86061	15.19011	9.332858	17.57564	28.2756	18.99604	9.899802	10.73688	15.29025	14.87128	19.55039	15.24609	2.155571	4.085644	7.612639	0	4.560123	17.56991
LOC107799723	3.893939	2.404057	2.802567	0.910377	0.92624	1.620565	1.053553	1.073254	1.022052	1.224865	1.70285	0.742837	0.978823	1.370354	0.859491	1.840907	2.184313	2.434855	0.934716	2.231422	0.992847	0.594439	1.292633	1.444174	0.952862	0.582595	1.534108	0	1.612629	0

LOC107826931	0.236097	0.061478	0.146596	48.79787	46.05061	18.74738	51.59954	79.423	42.87501	99.02929	48.48286	71.29311	46.03214	98.31815	202.8592	0.201074	0.065168	0.226312	182.0768	164.3139	98.68003	118.6685	29.03642	14.38307	528.2564	878.6274	146.5796	400.3982	240.9707	402.4938
LOC107797353	0.108447	0	0	0.458487	1.727294	0.388645	4.426668	5.436042	1.934476	0.422997	1.039253	0.671871	1.287727	2.878877	3.264997	0.107753	0.069846	0.053901	0.806991	0.978544	0.923115	3.773612	3.379887	0.226697	6.764074	3.269407	0.640876	28.43674	9.91992	42.46768
LOC107765294	0	0	0	0	0	0	0	0	0	0	0	0	0	0	0	0	0	0	0	0	0	0	0	0	0	0	0	0	0	0
LOC107802353	11.69815	8.645827	12.39499	3.216287	4.119916	6.82033	3.758726	4.525196	5.682609	2.553844	2.530201	4.7202	3.563382	4.127048	1.612835	7.936803	12.65081	9.676053	3.248139	3.003211	3.662461	4.471579	8.573114	6.265526	1.986718	0.890788	5.434205	0	1.400971	3.598577
LOC107825003	4.919472	4.828376	6.03081	4.571069	3.739178	3.966775	6.956304	7.274995	3.641602	2.552307	8.071031	2.637529	2.738212	1.92658	6.376247	3.900996	5.910314	4.913744	2.047975	2.400885	2.818531	6.136233	6.481133	2.274271	2.073236	2.281695	1.975412	0	0.303642	0
LOC107783248	16.15451	14.96307	14.95931	2.763283	2.599013	3.441226	4.769164	4.118332	5.656527	12.74168	4.567986	4.612884	4.904569	5.628104	3.053293	15.34961	16.37057	12.6615	1.68128	1.943127	2.404016	4.685703	3.653678	4.368765	48.70388	2.357813	69.91985	10.7718	46.22903	11.64169
LOC107761511	4.17343	4.540874	4.442751	3.327207	2.261207	2.526497	5.320662	4.279071	4.412496	2.053594	2.890395	2.299926	5.174841	4.917248	4.481194	6.851669	4.540522	5.63894	2.501854	1.987906	1.592765	4.214581	3.080916	2.550132	1.21765	1.143537	0.546387	0	3.64754	3.175994
LOC107771863	0	0.068294	0	0	0.066308	0	0.062851	0	0	0.219214	0	0	0	0.081751	0	0	0	0.055867	0.076039	0	0.068342	0	0	0	0.094741	0.312801	0	0	0	0
LOC107765537	0	0	0	0	0	0	0	0	0	0	0	0	0	0	0	0	0	0	0	0	0	0	0	0	0	0	0	0	0	0
LOC107787351	0	0	0	0	0	0	0	0	0	0	0	0	0	0	0	0	0	0	0	0	0	0	0	0	0	0	0	0	0	0
LOC107764835	0.511771	0.233208	0.31141	0.649094	0.633988	0.770303	0.300471	0.30609	0.384379	0.523993	0.538281	0.367505	0.370312	0.33499	0.210107	0.648333	0.568576	0.57232	1.531964	1.298769	0.630104	0.470133	0.654553	0.401177	0.582331	0.676487	0.483898	0	1.601501	0
Novel01557	2.204715	1.944509	1.391017	0.773172	0.755178	0.688163	0.387733	0.577284	0.367237	0.416105	0.423889	0.210294	0.791716	0.349147	0.291982	1.907949	1.339801	1.219526	0.252586	0.330894	0.421606	0.326667	0.470493	0.483174	0	0	0.168116	0	0.342396	0
LOC107789910	135.7935	109.4378	134.9888	65.29304	61.2644	48.65078	66.49433	79.54894	110.2218	12.40439	19.27567	16.53623	12.36345	12.76067	10.42885	93.56291	87.31915	106.9948	26.01619	20.42243	45.61613	84.92199	137.1482	198.531	6.174262	4.164721	10.70601	5.632923	7.3946	29.22153
LOC107785305	0	0	0	0	0	0	0	0	0	0	0	0	0	0	0	0	0	0	0	0	0	0	0	0	0	0	0	0	0	0
LOC107794374	0.026177	0.095428	0.022755	0.023715	0	0.056287	0.021956	0.022366	0.098304	0	0	0.022115	0.087421	0.199903	0	0.058521	0.050578	0.039032	0	0	0	0.065583	0	0.02736	0	0	0	0	0	0
Novel03597	1.762542	2.692315	1.812673	1.551794	1.405848	1.654743	1.311748	1.187802	1.701365	1.791322	2.576233	1.342237	3.122757	2.816564	0.891773	2.404905	1.798694	2.646607	1.10837	0.860994	0.475448	1.223165	1.116741	1.790315	0.596329	0.207249	0.49879	0	2.748825	0
LOC107796550	0	0	0	0	0	0	0	0	0	0	0	0	0	0	0	0	0	0	0	0	0	0	0	0	0	0	0	0	0	0
LOC107787538	0	0	0	0	0	0	0	0	0	0	0	0	0	0	0	0	0	0	0	0	0	0	0	0	0	0	0	0	0	0
LOC107764612	0	0	0	0	0	0	0	0	0	0	0	0	0	0	0	0	0	0	0	0	0	0	0	0	0	0	0	0	0	0
LOC107779845	0	0	0	0	0	0	0	0	0	0	0	0	0	0	0	0	0	0	0	0	0	0	0	0	0	0	0	0	0	0
LOC107788385	53.27744	44.17884	57.15962	27.69132	27.64263	23.54888	34.74748	39.34731	56.55078	5.864869	9.599194	6.825482	5.59615	7.687719	5.850614	36.52488	46.39716	39.21865	8.972424	8.049401	13.99979	41.26977	57.28783	79.70496	1.418679	0.874342	4.137854	6.419698	3.45741	0
LOC107827869	1.297787	2.133869	1.628242	1.612083	1.74031	0.891828	2.771773	2.263456	1.180737	1.382925	3.002092	0.46343	1.7277	1.401226	4.215753	3.170534	1.551058	1.97501	1.92784	2.840799	0.915158	5.206786	1.569584	0.643263	3.281595	4.896074	0.34789	0	2.125611	4.963555
LOC107766735	0	0	0	0	0	0	0	0	0	0	0	0	0	0	0	0	0	0	0	0	0	0	0	0	0	0	0	0	0	0
LOC107821564	0	0	0	0	0	0	0	0	0	0	0	0	0	0	0	2.106283	2.904894	3.183289	3.295262	4.934602	4.716791	2.561342	3.500987	3.708486	4.181764	2.593985	4.62005	4.300681	4.053327	3.718392
LOC107794757	0.259949	0.059228	0	0.883128	0.402535	0.698693	1.035645	0.666323	0.854181	1.901125	3.326547	1.756898	4.051307	2.552326	0.466908	0.387429	0.062783	0.096902	0.263778	0.549748	0.592695	0.542726	0.343939	0.135849	1.643274	1.085101	1.689815	0	0.938616	0
LOC107815470	0	0	0	0	0	0	0	0	0	0	0	0	0	0	0	0	0	0	0	0.048006	0	0	0	0	0	0	0	0	0	0
LOC107783143	161.3145	136.7558	199.2877	48.48501	50.71527	77.53466	52.21042	66.051	82.43585	19.12299	28.92165	31.42525	14.78874	28.43384	16.49195	79.76403	171.3666	116.9654	26.4473	23.22512	43.96337	48.9211	80.01722	87.5428	3.679108	4.401124	19.73899	18.0961	3.654708	15.64599
LOC107761670	8.071635	9.860573	8.807949	4.045209	2.178153	4.308225	2.30469	2.690173	2.364768	0.781504	1.364783	1.160705	0.318633	1.4364	1.292618	9.257116	8.074399	11.69406	2.323557	1.985461	1.983942	3.920201	3.484116	4.547318	0.434255	0.398266	1.014896	0	0.826803	3.539584
LOC107832551	0	0	0	0	0	0	0	0	0	0	0	0	0	0	0	0	0	0	0	0	0	0	0	0	0	0	0	0	0	0
Novel03694	1.163995	0.28289	0.202367	0.070301	0.274661	0.166858	0.260345	0	0.218562	0.302678	0.163239	0.196675	0	0.33863	0.079646	0	0	0	0	0	0	0	0	0	0	0	0	0	0	0
LOC107824551	0.03506	0.079882	0.015238	0.158813	0.201653	0.358092	0.161735	0.104847	0.148122	0.17094	0.073752	0.103669	0.214662	0.019124	0	0.03919	0.016935	0.039208	0.053365	0.148292	0.127902	0.102479	0.108239	0.219869	0.088653	0.268309	0.020719	0	0	0
LOC107808619	18.298	18.44039	20.32923	2.313919	2.224755	4.312093	1.606698	1.99478	2.060726	0.758857	1.637052	0.623741	0.710825	1.697986	0.532491	13.62082	12.80021	19.11656	1.336377	1.097192	0.909928	2.566297	4.312225	3.754101	0.025228	0.083294	0.306595	0	0.192133	0
LOC107764977	0	0	0	0	0	0	0	0	0	0	0	0	0	0	0	0	0	0	0	0	0	0	0	0	0	0	0	0	0	0
LOC107804387	0	0.035414	0	0	0.034384	0	0	0	0	0.075782	0.054494	0	0.043257	0.127175	0.079764	0.057913	0	0	0	0.032871	0.035439	0.032451	0	0.040614	0	0	0	0	0	0
LOC107769894	6.029325	4.636395	4.422232	4.665683	5.946462	3.98389	2.844593	3.863714	5.306809	3.980812	5.020473	5.624339	5.243694	4.590685	3.287552	4.867482	5.339432	6.227611	3.632647	4.303458	3.093101	3.25193	4.044096	6.039272	3.732045	0.961282	5.641557	0	4.837886	0
LOC107813694	0	0	0	0	0	0	0	0	0	0	0	0	0	0	0	0	0	0	0	0	0	0	0	0	0	0	0	0	0	0
LOC107774280	9.285125	7.551511	9.012417	10.41108	7.737124	10.07217	12.57229	12.73623	13.01729	8.039128	10.71498	9.532797	9.687413	9.629948	10.55565	8.315554	7.40143	6.922455	7.055865	8.277244	7.556815	13.24829	15.02129	16.01511	2.684754	3.765795	5.905437	0	5.412337	0
LOC107801321	1.931438	1.071467	1.660709	0.190194	0.167191	0.157997	0.193693	0.215253	0.31536	0.061415	0.132488	0.017736	0.280448	0.091613	0.043095	0.672721	0.588174	0.657377	0	0.017759	0.05744	0.298053	0.111108	0.109714	0.053085	0	0	0	0.101072	0
LOC107775776	3.401723	4.904875	3.810322	6.669542	5.525946	5.003725	5.933461	6.044414	5.35459	4.934406	5.613145	4.103486	5.275207	5.852849	6.253317	4.376288	5.4282	4.163793	5.134817	4.552658	4.368096	6.035077	5.642815	5.784288	9.885448	10.80876	5.400756	7.261829	10.4292	35.57883
LOC107810852	3.682952	5.651638	5.034856	5.104664	3.788144	3.959624	3.6963	3.066127	3.428164	3.253696	4.767671	1.675402	2.067478	2.129154	7.301695	8.163248	6.173354	5.608936	3.226134	4.127404	2.928284	4.469009	3.387437	2.928192	0.994951	2.321381	0.744095	2.251138	1.667021	5.839038
LOC107776319	0	0	0	0	0	0	0	0	0	0	0	0	0	0	0	0.084671	0.137211	0.211775	0.230592	0.072087	0.103625	0.260944	0.125278	0.207826	0	0	0	0	0	0
LOC107826587	0	0	0	0	0	0	0	0	0	0	0	0	0	0	0	0	0	0	0	0	0	0	0	0	0	0	0	0	0	0
LOC107807119	1.482446	0.750593	0.787514	1.15649	1.348204	1.460993	1.070698	0.738875	0.966518	1.124334	0.895118	0.90452	1.146028	1.302806	0.845301	0.705794	0.795649	0.614015	1.169999	1.079874	1.014012	0.790963	1.053357	1.032968	0.468566	0.343786	1.022077	0	0.991253	5.092326
LOC107764363	5.27313	7.252401	5.695049	31.79002	32.54897	29.75502	22.35525	25.14891	30.75402	22.74931	15.97778	25.08236	25.72181	28.76363	18.04065	5.76333	6.205768	6.552237	28.11607	25.22326	25.27007	18.86931	26.61079	35.94149	6.384004	9.382692	12.20043	4.57095	14.46274	1.976034
LOC107802988	9.214502	7.454805	8.609648	2.098128	1.308064	3.390534	2.603753	1.936703	3.932264	0.768796	1.58939	1.956574	0.658249	0.91387	0.404599	0	0	0.036737	0	0	0	0	0.043464	0	0	0	0	0	0	0
LOC107829457	9.333859	10.77863	7.987736	3.965393	2.667572	2.649001	3.938668	2.773949	3.565087	4.890039	5.792853	3.110121	5.550249	3.92132	2.915657	10.73582	8.065156	8.385123	2.176647	2.108817	1.586206	2.517623	3.196072	3.120627	7.219766	0.887337	7.879907	4.145981	14.23455	7.169274
LOC107821047	9.971762	11.18134	10.61616	7.968113	8.7741	5.962728	8.222784	9.429598	8.415218	3.05915	6.952924	2.271754	2.743994	2.81135	5.634859	13.02507	8.497017	10.56683	7.21949	7.771964	3.780824	11.64939	10.08053	11.47644	1.699866	1.870784	1.920162	0	0.269706	3.463871
LOC107782632	11.10416	9.434646	10.88322	8.216173	6.302533	8.466223	9.387745	8.618302	11.33882	4.615901	4.963341	6.391107	5.909785	6.467282	5.176327	7.945155	9.915502	8.575462	2.828155	3.218439	3.631259	8.164999	10.45798	10.81999	0.727121	1.292682	1.673207	3.163761	1.277913	2.735405
LOC107816639	6.2932	5.619607	6.16127	8.148856	7.481098	9.192136	7.304421	7.006499	8.086658	9.669895	8.26842	9.237042	10.04855	8.876698	6.915876	5.637114	5.926231	5.592303	7.546978	9.598637	9.044067	6.9544	6.616365	8.205453	10.16666	7.650906	12.1338	13.63796	9.946216	9.826212
LOC107789024	12.80394	12.02283	13.67852	21.12946	17.31891	21.41347	16.19922	17.68087	15.74455	10.08926	11.60809	12.20114	10.75013	11.05249	10.53099	11.27633	12.16144	12.18145	16.53598	15.13458	20.17007	16.34539	19.12742	20.18666	7.848763	6.478455	7.337323	3.08304	10.79267	10.66245
LOC107772180	0.925782	1.332215	0.974186	1.412567	0.5605	1.885886	0.20434	0.333058	0.320219	0.237567	0.478328	0.329316	0.216967	0.265785	0.300062	2.360163	2.447785	2.361244	2.719372	2.060915	1.555342	1.261446	1.031496	0.814844	0.431226	0.271191	0.287947	6.969104	0.234581	0
LOC107780364	2.565604	2.977252	3.296855	2.641915	2.397122	2.844001	2.486026	2.627823	2.558311	1.740374	1.944265	1.736688	2.412578	2.520792	1.750059	2.93315	1.939766	2.720644	2.134463	2.493851	1.802139	2.169219	2.642548	2.465045	1.4506	1.352552	1.563255	1.139609	1.917971	2.955935
LOC107783139	153.7156	111.5726	115.9037	147.4331	108.5819	149.0257	103.1638	98.34002	158.0735	182.1233	173.592	239.7495	201.8227	209.5503	98.35959	92.06365	160.4343	118.8	75.19765	76.89907	90.61066	66.88485	106.7425	117.287	53.84488	50.39897	141.1381	43.47708	42.671	15.82758
LOC107815273	6.69375	6.953748	6.835996	9.41435	14.41418	8.807027	16.52892	15.87812	21.00655	31.22126	21.49894	60.4657	42.72931	55.91814	28.49005	11.3368	3.753414	8.131333	9.927288	9.701403	15.32607	9.264753	10.40483	12.57378	9.173111	4.75463	17.86997	6.695073	22.98644	5.788596
LOC107778624	0.109352	0	0.475288	0.198135	0.290286	0.705403	0	0	0.20533	0.319897	0.38339	0.554303	0.365198	0.715788	0.224472	0.896384	0.845148	0.489161	0.554816	0.370018	0.797849	0.091323	0.48228	0.342885	0	0	0	0	0	0
LOC107799990	12.77982	13.14701	16.67906	2.220414	1.611064	7.001688	0.411139	0.418827	0.591695	1.024269	1.055705	0.709923	0.233863	0.916745	0.431239	10.54112	9.6065	13.46958	2.131739	1.747507	1.69243	0.409366	1.358895	1.024682	0.177069	0.048718	0.124148	0	0.337132	2.164919
LOC107768043	7.683887	7.349594	8.696498	13.19873	12.35302	14.64099	12.57056	10.88803	11.14237	10.9053	11.38937	11.82621	20.45292	13.0306	11.24416	7.483539	8.12151	8.791513	9.148056	9.460453	8.985292	11.05505	12.4817	13.31906	9.666676	4.498927	11.82424	2.720329	17.76395	9.408045
LOC107819308	0	0.255282	0.081163	0.253762	0.247856	0.250957	0.117468	0.319107	0.175317	0.227615	0.13094	1.380406	0.311818	0.356512	0.431239	0.173946	0.225505	0.174026	1.658019	1.816618	1.745651	0.155949	0.205893	0.048794	0.590231	1.104282	0.275885	3.338586	0.674264	0
LOC107820273	33.67861	36.46315	37.40436	24.55133	21.89316	30.20351	23.667	22.16524	27.42382	12.99112	18.42238	18.28099	17.9167	18.82223	15.75465	27.31726	30.73768	34.79455	12.93098	13.1249	16.37655	20.86983	23.97708	30.66022	3.87091	3.281445	6.601635	5.917683	3.983804	10.23292
LOC107820898	0	0	0	0	0	0	0	0	0	0	0	0	0	0	0	0	0	0	0	0	0	0	0	0	0	0	0	0	0	0
LOC107786263	0	0	0	0	0	0	0	0	0	0	0	0	0	0	0	0	0	0	0	0	0	0	0	0	0	0	0	0	0	0
LOC107770760	2.714357	1.203471	2.231991	6.978443	4.154538	6.940741	0.61531	1.002906	0.895369	2.718377	2.314835	2.16921	0.979998	0.720299	0.677662	1.312049	3.331027	2.242444	4.689826	4.778494	3.880576	0.367594	2.200115	2.146953	0.278252	0.714535	1.994257	10.4927	0	0
LOC107777210	2.262054	3.078988	1.634373	25.09734	31.89585	8.148727	91.95657	117.8231	60.62243	36.78223	58.72887	30.12999	19.39302	25.41503	63.62088	2.430013	2.185334	2.190204	39.23006	43.68839	27.9179	103.14	78.80064	26.71349	37.69899	91.11349	41.45762	23.10983	11.03176	27.24666
LOC107758973	364.6176	317.0527	407.4282	122.7626	117.0163	202.5865	95.28361	106.83	137.4385	49.48488	61.91413	70.91794	43.013	64.26816	26.2522	206.4211	376.9614	283.5026	57.56914	62.4939	112.0452	94.53186	168.1288	163.6731	9.288553	5.679165	27.17549	48.64797	11.78999	25.23678
LOC107794765	2.778118	4.685492	3.710339	6.18119	5.641757	2.620617	3.535365	3.328636	4.187141	4.473509	4.567766	3.246277	4.727828	5.074541	5.407797	4.52125	3.743057	4.682034	3.175473	3.349563	2.242461	2.586754	2.985647	2.770185	1.547603	1.510674	1.081923	0	2.254764	1.316285
LOC107783495	379.0454	334.4327	419.1257	169.4536	147.9725	203.9503	151.6387	166.9229	209.9413	67.35476	89.83647	85.1151	56.86527	86.35913	68.17011	251.7834	379.9803	311.795	86.07169	87.92077	127.0672	174.9256	233.3534	286.067	12.67801	12.39333	45.73433	21.09174	10.79126	21.88324
LOC107803193	0	0	0	0.038269	0.22427	0	0.10629	0.57748	0.079317	1.153349	0.444299	0.856488	1.786916	1.428594	2.774769	0	0	0	0.042864	0	0	0	0	0.053406	0	0	0	0	0	0
LOC107826844	8.043751	8.808655	6.964625	5.674247	6.329917	5.417828	6.346642	6.451738	6.357863	10.85093	11.82676	7.883415	11.00721	9.312956	7.489034	9.46517	6.143035	8.959881	5.920046	6.25311	4.291436	5.695311	5.763032	5.23381	7.617261	6.392436	8.060339	1.136842	16.76063	4.914597
LOC107773737	0.075367	0.068687	0	0.068278	0.266757	0	0	0.064395	0.070757	0	0.317082	0	0.167798	0	0.112327	0	0.112378	0	0	0	0.137471	0.125881	0.066478	0	0	0	0	0	0	0
LOC107799674	4.49596	4.613866	4.496068	4.073112	3.202063	4.676513	4.307473	4.231864	4.924506	3.760371	4.216242	3.103499	4.150459	3.230037	3.770409	4.943875	5.40286	4.373859	2.726186	3.540445	2.500239	5.036784	4.771778	4.164311	1.77922	1.169784	1.728086	0	1.759768	1.130049
LOC107799823	3.096539	3.390201	3.495786	4.226177	3.985485	5.253152	4.283694	3.487602	4.322995	1.647009	3.031353	1.74969	2.372686	2.062001	1.878029	3.656109	2.758412	3.387947	1.754709	3.027686	1.668784	5.255962	4.292124	4.707638	0.813499	0.13989	0.950613	0	0.871237	1.24327
LOC107808351	0	0	0	0	0	0	0	0	0.061901	0.068017	0	0	0	0	0	0	0	0	0	0	0	0	0	0	0	0	0	0	0	0
LOC107798029	9.318785	8.35293	8.234148	9.39327	10.42061	11.34	7.322194	7.196624	9.373806	13.4807	12.47628	13.47455	15.93119	11.73039	11.03601	8.737695	8.508074	10.57402	7.793455	8.402812	4.389536	7.226478	7.248766	10.32867	5.923256	4.09653	6.172711	3.661675	6.285883	3.165904
LOC107821559	15.81276	15.66454	14.56745	16.07732	14.18228	16.44628	8.685719	9.141852	11.01326	10.09805	8.255676	10.1645	8.466652	11.0631	6.880016	13.92909	15.94065	15.50521	14.60681	13.99444	14.73501	6.243975	9.551278	10.7788	6.899459	5.440786	11.78245	6.145847	9.516031	10.62746
LOC107787469	0	0	0	0	0	0	0	0	0	0	0	0	0	0	0	0	0	0	0	0	0	0	0	0	0	0	0	0	0	0
LOC107812960	0	0	0	0	0	0	0	0	0	0	0	0	0	0	0	0	0	0	0	0	0	0	0	0	0	0	0	0	0	0
107810341	0	0	0	0	0	0	0	0	0	0	0	0	0	0	0	0	0	0	0	0	0	0	0	0	0	0	0	0	0	0
LOC107827237	0.020802	0	0	0	0	0	0	0	0.01953	0.020285	0.014586	0.017574	0	0	0.021351	0.015502	0	0.015509	0	0	0	0	0	0	0	0	0	0	0	0
LOC107814617	9.88645	10.82913	9.768427	7.259871	6.384542	8.813606	10.40387	9.417898	9.887172	9.520779	10.65693	8.585798	9.399307	9.378809	11.37619	12.92726	11.41984	11.14771	4.984887	5.791945	5.880397	8.410279	10.37252	10.26913	5.085191	2.093346	6.205382	6.587152	4.582309	1.898429
LOC107789613	3.516763	2.866346	3.554129	1.994494	1.998677	1.752148	2.278193	1.807777	3.274849	1.338259	1.764262	1.183593	1.113999	0.810992	1.144479	2.684616	2.182127	2.856375	0.783344	0.846528	0.860508	3.295105	2.900261	4.21363	0.46993	0	0.743445	0	0.2753	1.76786
LOC107827069	0.074544	0.181168	0.410398	0.382689	0.94545	0.374006	1.042058	1.995702	1.306395	0.36345	1.271915	1.133587	0.66387	1.11143	1.683224	1.111009	0.072016	0.555759	2.950052	3.110926	6.164029	3.361708	1.884918	1.869927	1.979179	5.116996	1.027895	3.553979	4.306591	10.75476
LOC107790701	5.909042	7.034369	6.848806	2.863389	2.371159	2.954857	3.649891	3.150636	4.06399	1.764352	2.746223	1.857534	1.631759	1.97392	1.974612	6.161381	4.735066	5.908073	1.301476	1.414345	1.169737	4.953922	3.616192	3.854096	0.405394	0.095605	0.514326	0	0.551322	0
LOC107773829	6.196497	5.206144	4.797337	4.473427	4.112301	3.435089	4.060366	4.301745	6.726535	8.59149	6.3817	5.807548	6.143555	5.598186	5.664299	6.710003	4.676826	5.99124	3.733367	5.159907	7.417343	3.395999	4.355496	5.464612	4.284359	2.42493	5.49282	6.924018	5.127399	5.986543
LOC107809012	0	0	0	0	0	0	0	0	0	0	0	0	0	0	0	5.866567	4.850726	6.3314	8.491638	8.075373	8.593167	6.574498	7.326773	8.422663	9.169469	7.676394	13.7005	22.16505	11.04197	11.49841
LOC107787928	4.471443	4.380114	3.278771	2.921058	2.530088	4.022475	3.418725	2.624985	3.512602	2.105957	2.708797	1.670367	2.539629	2.024256	2.341514	4.533503	3.702677	4.558257	1.512437	2.470226	1.165152	3.632608	3.836786	5.43658	0.653779	0.296271	1.222355	0	1.46443	3.761579
LOC107764778	0.07012	0	0	0.063525	0.062047	0.075388	0.147032	0.209694	0.197496	0.034188	0.098337	0.02962	0.195147	0.191244	0.107954	0	0	0.026139	0	0	0	0.234237	0.061851	0.07329	0	0.048784	0.041438	0	0	0
Novel03094	3.390976	2.564422	2.445967	10.7195	8.235577	9.463343	2.11799	2.280886	3.657751	1.547784	1.97303	2.986697	2.971716	2.754864	1.110768	2.580725	5.088206	2.259168	5.124787	4.150217	10.33069	1.747338	5.918491	8.370457	0.36487	0.100389	1.193831	0	1.042045	0
LOC107820749	4.048925	4.530877	4.366141	0.696479	0.861675	0.881638	0.859749	0.613078	1.058592	0.449796	0.718762	0.129898	0.057055	0.335482	0.157812	2.97907	2.822295	3.056856	0.36405	0.086712	0.701143	0.642031	0.723325	0.642826	0	0	0.181728	0	0	0
LOC107788928	0	0	0	0	0	0	0	0	0	0	0	0	0	0	0	0	0	0	0	0	0	0	0	0	0	0	0	0	0	0
LOC107803461	11.56268	11.18645	10.03714	14.46018	13.37955	14.43074	14.33694	12.73968	11.84242	14.1451	18.66486	9.700165	17.51637	15.87816	18.17489	13.49721	9.686328	12.39419	12.25813	13.55693	6.725433	14.90987	12.15327	10.80072	10.46825	11.4533	9.442075	3.469491	11.75622	4.999567
LOC107797134	0	0	0	0	0	0	0	0	0	0	0	0	0	0	0	0	0	0	0	0	0	0	0	0	0	0	0	0	0	0
LOC107828294	20.36991	17.87877	22.19933	23.99943	20.422	33.44357	16.70638	15.7756	20.25466	13.16061	11.25785	17.20906	18.04032	18.00794	11.63795	16.89954	27.24045	24.16395	17.76803	18.71702	19.63013	15.54494	16.28591	23.17846	4.250004	6.352795	8.894923	7.176041	6.521763	12.40889
LOC107793735	14.60426	11.23696	13.9148	7.010163	7.110367	6.056567	7.934358	7.737621	10.89705	3.669078	4.337686	3.017173	2.768773	2.272763	3.22916	10.85143	9.590795	12.36203	4.508375	4.621585	4.420389	9.693208	11.53185	11.97597	2.956426	1.44937	2.939657	0	3.479685	2.628831
107769489	0	0	0	0	0	0	0	0	0	2.09905	0	0	0	0	0	0	0	0	0	0	0	0	0	0	0	0	0	0	0	0
LOC107767220	4.161141	5.25294	3.275024	9.908415	12.83742	3.778492	17.30915	25.17601	13.75251	26.10048	20.62171	13.82426	9.608767	11.50236	22.10469	4.986561	3.015009	3.856922	21.65426	20.21644	18.43667	20.35738	19.14561	13.54724	106.7474	65.44961	54.16593	12.06407	42.09682	24.3382
LOC107788954	4.781804	4.795651	4.469779	7.123449	7.57731	8.389119	6.32651	6.154197	5.184375	9.228176	8.698169	8.434563	12.18316	9.6913	7.084644	4.726359	4.32969	5.310266	7.57275	7.717804	4.944997	5.246571	5.117914	4.914293	7.816348	4.704798	6.857721	2.861645	13.19631	2.474194
LOC107821622	5.01611	6.241945	6.540597	12.58433	6.743233	9.022789	7.848097	7.08822	7.516839	4.421019	6.561116	5.215691	7.839082	6.945649	5.544428	6.182095	5.684712	5.897255	5.775194	6.936137	8.709671	8.458728	7.487684	7.864255	1.463513	1.476443	2.394258	0	4.644103	0
107815768	0	0	0	0	0	0	0	0	0	0	0	0	0	0	0	0	0	0	0	0	0	0	0	0	0	0	0	0	0	0
LOC107799747	1.381481	1.425911	1.273235	0.271421	0.058912	0.339999	1.67524	0.782176	0.281276	0.162303	0.618564	0.08437	0.518804	0.435796	0.956665	1.314759	0.787912	1.091998	0.067558	0.0704	0.106259	1.820917	0.631301	0.086983	0	0.023159	0.019672	0	0	0
LOC107824928	12.19126	12.58409	11.10504	11.1667	9.93336	10.50016	9.6604	8.920245	9.443223	7.81588	9.225474	8.497575	8.047913	9.111647	8.595332	11.47748	11.04032	11.91794	9.523047	12.11726	7.576234	9.76874	9.882128	9.222329	8.345409	7.747488	6.315586	4.816837	7.458235	5.552884
LOC107798508	17.56586	19.40578	18.10757	37.82139	49.64692	29.53956	40.10446	49.49429	26.36486	35.15938	36.34381	31.14473	54.67556	54.24401	62.77571	25.83572	17.22129	20.50572	83.87537	74.41972	89.37672	47.36288	41.23304	28.50605	93.14454	108.942	39.43988	169.4227	128.2433	257.2397
LOC107805403	0.322905	3.090022	0.400992	0.167163	0.020409	0.074392	0	0	0.021654	0	0	0	0	0	0	0.023673	4.81258	1.136401	0.894174	0.023404	0.1756	0.042071	0	0.122067	0	0	0	1.649447	0	0
LOC107794617	12.11837	6.311085	5.633696	2.694389	4.359954	1.33628	4.579474	5.802939	7.043065	3.376268	3.330486	5.550236	5.336837	5.133252	3.918186	7.740534	9.563157	8.803098	6.12592	4.58117	9.473277	3.855378	5.285869	5.242751	3.535685	1.667653	7.659886	3.174486	1.282245	0
LOC107784787	0	0	0	0.029166	0.042731	0.017306	0.067506	0.068769	0.045338	0.09418	0.045149	0.040798	0.053758	0.105366	0.066086	0.035987	0	0.012001	0.049002	0.068085	0.146808	0.053772	0.028397	0.050474	0.244219	0.515151	0.038051	0	0.232491	0.995304
107761643	0	0	0	0	0	0	0	0	0	0	0	0	0	0	0	0	0	0	0	0	0	0	0	0	0	0	0	0	0	0
LOC107805019	0.218067	0.556476	0.303298	0.237069	0.154368	0.328229	0.475546	0.260852	0.245677	0	0.3364	0.147383	0.097102	0.428221	0.358109	0.42251	0.210671	0.390188	0.177024	0.18447	0.079552	0.291382	0.34623	0.364678	0.055141	0.060685	0	0	0	0
LOC107783335	0	0	0	0	0	0	0	0	0	0	0	0	0	0	0	0	0	0	0	0	0	0	0	0	0	0	0	0	0	0
LOC107784201	16.8711	20.0657	18.2264	14.51898	12.00635	14.89063	14.51555	12.9933	13.50789	10.55988	15.74345	11.09541	12.25476	11.28352	11.90313	17.68525	16.2989	18.07508	10.41725	11.08825	8.218705	15.07298	13.88782	14.71702	3.172017	3.74031	6.38452	7.32335	1.725536	1.582952
LOC107823032	46.40178	62.23421	68.60797	29.6554	23.25609	25.02873	25.42336	27.78886	40.17682	21.46821	18.33666	20.15696	15.24441	17.52523	18.24473	61.69995	49.00044	64.31985	19.6946	20.53973	22.48058	30.9219	29.29173	43.18679	7.757757	10.84628	17.55479	13.18313	11.41063	6.513262
LOC107819491	2.112797	1.363934	2.448813	2.233102	3.310647	2.744748	1.735175	2.406975	2.851406	2.360696	2.62348	2.788984	4.066992	4.081696	2.12334	0.984037	1.615902	2.198689	2.411911	1.898986	1.886763	1.801211	2.678963	3.266436	1.669511	1.286164	2.080963	6.295619	5.085879	0
107801241	0	0	0	0	0	0	0	0	0	0	0	0	0	0	0	0	0	0	0	0	0	0	0	0	0	0	0	0	0	0
LOC107822386	0	0	0	0	0	0	0	0.062946	0	0	0.051658	0	0	0	0.075613	0	0	0	0	0	0	0	0	0	0	0	0	0	0	0
Novel04914	0.208684	0.418418	0.544215	1.550271	3.323837	2.871823	1.50528	1.390783	1.293085	3.337294	1.843753	3.314483	3.670502	1.957913	1.070939	0.839764	0.483856	0.622332	3.599893	4.907605	2.588401	0.766822	1.030811	2.093921	1.266434	2.322952	2.56514	2.984776	6.630892	2.580653
LOC107775178	1.700312	1.503367	1.698649	2.437045	1.774019	3.192255	1.660265	1.452793	1.572502	1.484793	1.654928	1.286392	1.610302	1.661155	0.807459	1.475102	1.838781	1.892022	1.030065	1.717429	0.694349	1.525943	1.96987	2.228088	0.385023	0.317802	0.749863	0	0.488711	0
LOC107791365	1.845596	1.161404	1.413345	2.547834	3.343976	3.732263	1.621694	2.55312	2.351557	2.442435	2.218513	3.638158	2.73939	2.972242	2.164879	1.506325	1.570739	1.146642	2.541634	1.74711	3.486658	2.128469	2.79075	2.801666	1.444482	0.794861	3.89526	3.142528	1.904005	0
LOC107828634	3.197258	6.043657	3.22537	0.929758	0.419132	0.848751	0	0	0	0.192452	0.221424	0	0	0.086125	0.12154	4.176904	2.173614	2.795687	0.32043	0.901553	0.035999	0	0.24372	0.041256	0	0	0	0	0	0
LOC107790065	40.4335	36.96979	32.75153	58.39505	76.66762	40.22475	46.90608	49.46571	34.14003	55.94135	60.38607	53.56806	90.02227	70.17653	64.27085	46.27275	24.73091	48.44715	82.19146	73.40525	64.2936	45.60747	33.11752	42.12387	97.09525	112.7032	33.20415	84.49384	110.603	284.0982
LOC107773350	0.311784	0.615665	0.316199	0	0.045981	0.055868	0	0	0	0	0	0	0	0	0	0.697026	0.401613	0.27119	0.105459	0.175832	0	0	0	0	0	0	0	0	0	0
LOC107804243	23.54806	68.72326	28.40827	63.4682	37.51792	11.15072	95.0335	84.71623	32.02898	69.90472	73.77762	20.27981	63.10247	44.9749	100.0239	59.6439	26.46746	31.6593	42.89176	48.50337	29.68598	74.79466	67.43366	26.99867	22.26708	41.47865	4.857093	74.63798	31.71813	32.2662
LOC107772294	0	0	0	0	0	0	0	0	0	0	0	0	0	0	0	0	0	0	0.0841	0	0	0	0.073105	0	0	0	0	0	0	0
LOC107779759	2.422186	2.373088	2.000277	2.029799	1.446734	1.627591	0.863426	0.931311	0.966473	0.295242	0.679375	0.306949	0.269641	0.066062	0.186455	1.75989	1.696525	1.309236	0.860253	1.690427	0.331361	0.707991	0.694372	1.265834	0.153119	0.084257	0.071571	0	0.583063	0
LOC107821551	1.608935	1.512896	1.776016	3.377961	2.463212	2.800631	2.806067	2.531226	2.133936	2.440534	3.545727	1.812364	4.037057	1.671684	2.804702	2.207646	1.455676	2.913142	2.539656	3.197383	1.3975	2.431391	2.79332	2.348983	1.517565	1.243731	0.996093	1.826374	2.33609	3.158186
Novel02284	6.77467	5.760148	7.792254	1.27241	1.827644	2.84238	0.346476	0.70591	0.969567	0.523659	0.695181	0.453686	0.459857	0.495726	0.127195	4.155771	3.47199	5.851541	1.257524	0.629002	1.167908	0.551971	1.71255	1.813397	0	0	0.244119	0	0.397752	0
LOC107818637	0	0	0	0	0	0	0	0	0	0	0	0	0	0	0	0	0	0	0	0	0	0	0	0	0	0	0	0	0	0
LOC107786451	0.053503	0	0	0	0	0	0	0	0	0	0	0	0	0	0	0	0.103377	0.039889	0	0	0	0	0	0	0	0	0	0	0	0
LOC107797317	0	0	0	0	0	0	0	0	0	0	0	0	0	0	0	0	0	0	0	0	0	0	0	0	0	0	0	0	0	0
107823546	0	0	0	0	0	0	0	0	0	0	0	0	0	0	0	0	0	0	0	0	0	0	0	0	0	0	0	0	0	0
LOC107798365	10.72021	11.27189	9.756989	5.725319	5.249005	5.418878	7.820818	6.261019	5.769403	3.402605	5.342112	4.192628	5.956154	5.921632	4.357399	12.07697	10.8061	11.40322	3.088366	3.591347	3.42991	6.783351	6.97653	6.645862	0.784295	1.672362	0.985223	0	1.959908	2.397276
LOC107786691	0.939872	1.18603	0.785587	2.226937	1.919216	2.176412	2.941005	2.316495	2.681116	0.740245	0.86182	1.038346	1.6499	2.326764	2.15193	1.34691	1.012764	0.889368	2.127522	2.293461	2.307789	2.596245	2.327658	1.322399	0.685548	1.961644	0.427251	0	3.132606	2.235141
LOC107766015	0	0	0	0	0	0	0	0	0	0	0	0	0	0	0	0	0	0	0	0	0	0	0	0	0	0	0	0	0	0
LOC107786627	4.467464	5.809954	4.559807	8.435657	9.305345	7.933161	9.872857	10.18614	8.57701	9.862953	9.749742	8.52384	9.72297	9.939233	12.00417	5.573537	5.213081	5.239278	10.44181	11.86828	11.46784	8.153475	10.07288	7.423809	21.83137	17.49602	15.18794	7.17948	20.42048	27.93337
LOC107782817	0	0.121316	0.115712	0	0	0	0	0	0	0	0	0	0	0	0	0	0	0	0	0	0	0	0	0	0	0	0	0	0	0
LOC107777706	19.64645	16.28559	16.12321	11.68039	9.692986	14.3479	14.82507	14.35684	16.16967	13.80116	19.53125	18.83639	22.87791	17.80234	16.91437	17.60238	19.04256	18.04408	7.312001	7.899751	8.07484	13.87061	13.28213	12.36121	6.168917	5.03569	7.600138	13.86512	10.42302	9.989881
LOC107794909	43.83498	54.00628	47.44131	23.00771	19.68665	20.42117	29.31096	29.63303	30.72285	34.87674	42.36788	37.70349	24.08964	23.66559	35.21627	53.34573	55.43356	52.62069	26.36093	24.706	29.29003	28.61015	28.21147	31.77003	6.020358	13.6931	11.22485	3.783731	17.83054	3.271434
LOC107759244	0.182349	0.124641	0.079256	0.165199	0	0.098024	0	0	0.128398	0.088907	0.031966	0.038513	0	0.1492	0.04679	0.033972	0	0	0.046259	0	0	0.038071	0	0	0.230545	0.063431	0.05388	0	0.65842	0
LOC107803830	0.058394	0.053219	0	0.105805	0	0	0.489779	0.449044	0.16447	0.170826	1.228385	0.049333	0.71506	0.445938	1.078819	0	0	0	0	0	0	0.487667	0	0	0	0.081252	0	0	0	0
LOC107770891	2.931999	2.816596	3.134242	3.840783	3.120322	3.152257	3.090608	2.945278	3.013079	3.786335	3.389489	3.280396	3.308049	4.279297	3.700654	2.893555	3.13878	2.658563	3.497858	3.753921	4.73376	3.606705	2.900757	3.561474	5.961157	6.064363	5.010762	8.500515	6.10408	9.799454
LOC107807930	0	0	0	0	0	0	0	0	0	0	0	0	0	0	0	0	0	0	0	0	0	0	0	0	0	0	0	0	0	0
LOC107775197	0	0	0	0	0	0	0	0	0	0	0	0	0	0	0	0	0	0	0	0	0	0	0	0	0	0	0	0	0	0
Novel00094	0	0	0	0	0	0	0	0	0	0	0.043988	0	0	0	0	0	0	0	0.044045	0	0	0	0	0	0	0	0	0	0	0
107786756	0	0	0	0	0	0	0	0	0	0	0	0	0	0	0	0	0	0	0	0	0	0	0	0	0	0	0	0	0	0

LOC107824371	4.321763	3.702428	3.982225	4.541746	5.162643	5.900957	1.159951	1.033937	1.379537	5.26783	3.030423	5.29416	4.762936	2.970343	2.217869	3.961316	4.843216	4.51088	3.596049	2.010753	5.045146	1.082765	1.067382	1.30996	1.420646	2.16483	0.766196	6.181343	8.946796	0
Novel00060	0	0	0	0.059022	0.057648	0	0	0	0	0	0.228412	0.110079	0.072525	0	0	0	0	0	0.462761	0.496004	1.069505	0.598484	0.229863	0.136187	0.164736	2.991451	0.308003	9.318143	1.568251	0
LOC107792662	4.644339	4.137267	3.521189	3.66973	3.182635	4.692887	2.548123	2.595772	2.917801	2.690049	3.379042	2.035586	2.682255	1.904792	2.007083	4.840149	4.486822	4.972537	4.074938	5.080843	2.515951	3.936934	3.542185	4.452781	2.737256	1.506241	2.228712	0	2.017399	0
LOC107783562	4.775031	6.368564	5.340401	6.910947	5.822617	5.728505	8.254257	7.562771	8.145956	5.791931	6.063608	6.174109	6.093528	6.956354	6.873299	6.162036	6.722722	5.166325	5.73172	5.76346	5.09843	8.024138	6.857168	6.968946	2.944922	4.740003	4.370428	0	3.64455	0
Novel02042	3.063833	3.544081	3.329156	5.978382	5.526434	5.067685	6.424422	4.832902	4.370009	3.73453	6.073253	3.036406	3.541883	3.985278	8.043007	3.776015	3.870665	3.953454	11.53905	8.57285	6.233366	6.298307	5.97669	4.433978	6.331873	6.558616	3.273011	0	6.240547	3.643104
LOC107831418	38.83609	53.35171	51.06462	25.79632	21.69454	23.85917	33.29203	26.28118	33.58857	9.149286	11.6136	8.167987	9.718314	13.17332	13.44029	39.64146	34.24784	38.83844	12.45994	17.32356	13.98908	33.89797	27.99492	32.95925	0.464187	1.986676	2.700066	0	2.356774	2.522367
LOC107801453	21.34151	15.38653	16.18101	15.41255	12.41082	20.47805	10.74729	9.949596	11.29839	12.95918	14.96468	18.79791	20.33617	15.72634	9.508171	14.61335	20.36676	16.26601	13.44158	12.52389	14.64721	10.12251	9.660505	11.26618	8.921076	6.866627	15.29809	6.19156	7.294313	2.676628
LOC107810081	0.331136	0.617298	0.235514	4.390804	6.43293	1.877155	8.950751	9.259591	5.058954	7.837659	8.105649	7.248152	5.211027	7.71769	18.02852	0.953403	0.174494	0.291763	14.14315	14.38791	10.99562	16.55473	8.948398	5.364607	30.7713	59.62552	16.66909	18.29891	22.10153	61.42399
LOC107802022	3.98394	3.73402	3.168	1.168904	1.021522	1.533199	0.911319	0.947701	1.275102	1.412671	1.952305	1.204783	1.385931	1.703941	0.604058	2.226628	3.127167	3.69587	0.528298	0.459564	0.289022	1.134236	0.978357	1.395891	0.085856	0.062993	0.26754	0	0.326934	0
LOC107796212	4.070966	3.627735	4.954326	1.024469	1.641029	0.972619	0.872593	1.507282	1.401401	0.088216	0.539195	0.19107	0.50354	0.838898	0.185703	2.460659	3.277413	2.900186	0.64259	0.229584	0.990077	1.473234	1.875227	3.120313	0	0	0	3.234796	0	0
LOC107779244	0	0.024722	0.02358	0	0.144016	0.08749	0.159261	0.023177	0.025467	0.158706	0.019021	0	0.030197	0.029593	0.055682	0.040428	0.026206	0.06067	0.082576	0.091786	0.148435	0.045307	0	0.028352	0.034295	0.150974	0	0	0	1.677224
Novel03575	2.34655	2.609806	2.350963	1.693478	1.196559	2.266269	1.667926	1.155398	1.530929	2.443313	1.952172	2.150433	2.656487	2.169466	1.551195	1.837566	2.651196	2.550049	1.089663	1.68222	2.357729	1.461445	1.508507	1.829067	1.357666	0.719418	1.880294	2.844262	3.446581	0
LOC107810967	5.979835	5.900625	5.471731	18.81843	15.67513	16.77355	19.4211	15.82741	16.29365	19.59774	17.74959	21.08137	27.57832	28.7926	18.32032	5.76288	7.557911	6.502969	12.86582	16.08841	13.36774	14.04304	12.05625	14.37998	10.4594	5.943218	13.44454	9.646078	25.97509	8.34005
Novel01014	11.01318	8.568304	7.238518	9.369007	9.745187	14.00646	10.58902	8.606671	11.4745	4.452856	6.780687	10.55237	11.21344	10.98918	2.619133	7.506435	12.58594	7.109346	6.677992	8.74832	10.77915	6.393311	13.38685	15.02042	2.886685	1.30815	5.079685	9.604855	7.759225	0
LOC107800940	2.081721	2.42734	2.66117	2.939837	2.844332	3.620463	1.463583	2.171034	2.040635	1.88068	2.575948	1.629379	1.465415	1.970474	2.1052	2.258511	3.312428	2.716019	3.10645	3.107633	2.010244	2.352087	2.187256	2.143816	1.664307	1.618667	1.085482	0	1.621229	5.678643
LOC107820897	0	0.038221	0.018228	0	0	0	0	0	0	0	0.029407	0	0	0	0	0.015626	0	0	0	0.017738	0.019124	0	0.018496	0	0	0	0	0	0	0
LOC107803511	0.043541	0	0	0	0	0	0	0.111608	0	0	0.030531	0	0	0	0	0.227127	0.336514	0.292155	0.044182	0	0	0.399984	0.038406	0.182036	0	0	0	0	0	0
LOC107803890	2.476007	3.400754	2.849578	2.938194	1.974927	2.362066	2.339982	2.711503	2.390066	2.278396	2.323055	1.738256	2.290468	1.9403	2.362341	3.014568	2.493103	2.235962	1.80474	3.068047	1.781084	2.708499	2.799214	2.442124	2.160435	3.105518	2.514267	2.493937	2.014712	0
LOC107783798	0	0	0	0	0	0	0	0	0	0	0	0	0	0	0	0	0	0	0	0	0	0	0	0	0	0	0	0	0	0
LOC107781155	1.955189	2.47488	1.689113	6.833728	8.073707	2.906563	11.18575	9.456233	4.294439	2.377311	9.622248	2.997708	5.669763	3.423358	2.502247	2.842068	1.655683	1.781605	17.48851	17.29507	17.93072	14.83658	14.58463	4.642168	4.669246	2.502191	2.182494	9.4942	3.370078	16.41747
LOC107790635	0.286228	0.195646	0.062203	0.064827	0	0.153865	0.060018	0	0	0	0	0	0	0.15613	0	0.373271	0.345651	0.106698	0.145222	0	0	0	0	0	0	0	0	0	0	0
LOC107768817	4.683693	4.470673	3.661882	9.239606	7.504133	8.938833	7.531399	6.227769	7.389474	6.323804	5.946555	6.251456	6.077802	6.349305	5.034775	4.605535	4.57839	5.124196	5.680728	7.619388	7.405804	5.624191	6.184768	8.516249	1.471643	1.850987	5.502992	0	1.734536	1.713608
LOC107806717	0.192927	0.281327	0.352186	0.139826	0.085357	0.124452	0.161816	0.131874	0.108677	0.075251	0	0.032598	0.042954	0	0.019802	0.158147	0.130469	0.230137	0.195769	0.261125	0.123167	0.273902	0.20421	0.221812	0.024392	0	0.022802	0	0	0
LOC107806543	3.800605	4.323217	4.769374	4.582244	3.287167	5.99092	2.97202	2.856682	3.299891	0.417978	0.661237	1.08638	1.781426	1.278174	0.967877	4.833931	5.410932	4.836145	3.044677	3.65017	2.918918	4.367211	5.419268	6.242329	0.216772	0.119284	0.709265	0	0.963024	0
LOC107808574	1.368128	0.950004	3.058161	0.236087	0.057648	0.140086	0.16393	0.278325	0.550482	0.127057	0.274095	0.05504	0.072525	0.213223	0	1.165178	1.069969	0.971426	0.264435	0.110223	0.237668	0.272038	0.344794	0.204281	0	0.09065	0	0	0	0
LOC107810752	0	0.070202	0.03348	0	0	0.082816	0	0	0	0	0	0	0	0	0	0.028701	0	0.114857	0	0	0	0	0.067945	0	0	0	0	0	0	0
LOC107770072	0.072952	0.132974	0.253664	0.132182	0	0.078433	0.061188	0	0.136982	0	0.051154	0.123264	0.162423	0.238762	0.074876	0.434913	0.070478	0.271945	0	0.061713	0.133067	0.060924	0.064349	0.152499	0	0.101508	0.086224	0	0	0
LOC107797825	4.590586	3.271909	4.809063	2.505967	2.395569	3.669943	3.800959	4.173752	2.37594	3.156437	2.971312	2.535782	3.013769	3.017703	1.57055	3.771773	3.184025	3.422477	2.986032	3.435244	3.596261	4.079469	3.685811	3.629316	1.562587	1.555921	1.669447	0	0.850027	0
107823818	0	0	0	0	0	0	0	0	0	0	0	0	0	0	0	0	0	0	0	0	0	0	0	0	0	0	0	0	0	0
LOC107787348	4.067843	2.975024	2.881258	0.909941	2.044158	1.295831	1.30578	1.201468	1.744514	1.860903	1.549438	3.521482	2.124425	1.972363	1.133984	4.977421	5.094287	5.691086	1.274002	0.977107	1.83207	0.671044	1.506119	1.207275	1.079393	0.489145	1.246483	0	1.692448	0
LOC107785482	2.087185	3.622019	2.907924	2.175811	3.213063	1.998063	4.14871	3.664412	2.3085	2.258312	2.606296	1.280213	2.132512	2.214632	3.991003	4.04823	2.430724	2.877691	3.568569	2.805634	2.529372	4.488983	3.177677	2.002222	1.988164	2.784816	0.878616	2.044702	2.2024	3.53572
LOC107827906	1.094094	0.87249	0.475536	0.619496	0.363047	1.323321	0.745602	0.99325	0.449393	0.26672	0.38359	0.462161	0.228368	0.746002	0.070184	0.254788	0.924864	0.509809	0.693882	0.231382	0.124729	0.114213	1.085696	0.285887	0.086454	0	0	0	0	0
LOC107780814	0	0	0	0	0	0	0	0	0	0	0	0.081948	0	0	0	0	0	0	0	0.088465	0	0	0	0.122637	0	0	0	0	0	0
LOC107782777	0	0	0	0	0	0	0	0	0	0	0	0	0	0	0	0	0	0	0	0	0	0	0	0	0	0	0	0	0	0
107763372	0	0	0	0	0	0	0	0	0	0	0	0	0	0	0	0	0	0	0	0	0	0	0	0	0	0	0	0	0	0
LOC107783258	45.21234	38.72673	41.49949	26.52405	20.58385	24.87202	19.53778	19.38367	24.24275	8.393208	9.872106	10.29931	7.195233	8.831623	8.30884	30.09177	34.68348	30.36796	15.52007	14.23756	21.04035	19.40008	22.24038	27.05594	2.427267	3.606287	4.122202	2.288267	2.310704	1.978448
LOC107762451	2.891452	2.702771	3.447976	3.257594	3.378594	4.304304	3.979791	3.452414	3.306271	2.096574	2.703317	2.818591	1.609402	2.750017	2.244804	2.458583	2.57851	2.266249	3.009277	1.975591	1.893269	2.507601	3.040923	3.719563	1.499764	1.341081	1.051527	0	2.141612	2.292087
Novel02199	0.627896	0.391539	0.373454	0.778416	0.848027	0.497416	0.58208	0.677674	0.372314	0.676728	0.880562	0.027919	0.588617	0.685004	0.440943	0.443282	0.35119	0.517399	0.804816	0.782758	0.421954	0.441577	0.408097	0.414491	1.00276	0.597775	0.390591	0	0.795504	0
LOC107787400	6.059479	4.998597	5.621311	7.138625	6.061194	6.411682	5.725172	5.770838	6.52088	6.866302	6.868791	7.4866	7.438709	8.073808	6.120954	5.800583	6.039071	5.946088	6.100134	6.422573	6.378234	4.860412	6.485661	6.483564	5.935022	5.432038	7.756275	6.8512	6.111232	2.961792
LOC107805705	41.24217	34.62239	40.11663	31.21935	26.58972	39.42025	34.12913	30.66874	34.73409	24.08678	24.43427	30.74341	27.49771	32.2411	23.93786	27.11798	38.51174	31.03553	18.40716	19.5429	25.79646	25.50949	32.82803	33.13653	9.689608	9.896692	19.81077	11.82914	12.74146	17.04589
LOC107811974	1.709234	1.859258	3.450893	1.698333	0.829404	1.185572	1.294872	1.177755	1.294117	0.806477	1.198516	1.304266	1.04344	1.263178	1.188404	1.438075	2.716596	2.21976	0.279744	0.746268	0.703997	0.92092	1.750828	1.786491	0.348547	0.153437	0.521336	0	0.265447	0
LOC107786642	0.778618	0.322552	0.922958	1.603156	2.567988	2.206925	1.36549	0.786231	1.063274	5.314757	3.921039	3.58799	3.073088	2.007753	1.888905	0.369235	0.273531	0.580493	2.873047	2.694507	3.61512	1.123149	1.248714	1.775588	3.221705	1.18188	2.593471	15.18605	3.066991	4.376648
LOC107762114	2.502024	2.743759	2.121908	2.708991	2.663946	2.361935	6.892815	6.117923	6.302222	2.400118	3.080934	3.437039	3.328758	5.08191	4.071233	3.228787	3.498011	2.896623	1.032065	1.084081	0.649318	6.285504	4.557442	2.806464	0.077154	0.254735	0.456802	0	0.48966	0
LOC107792412	0	0.074358	0.141846	0	0	0	0	0.069711	0	0	0.05721	0	0	0	0	0	0	0	0	0	0	0	0	0	0	0	0	0	0	0
Novel04818	0	0	0	0	0	0	0	0	0	0	0	0	0	0	0	0	0	0	0	0	0	0	0	0	0	0	0	0	0	0
LOC107772907	0	0	0	0	0	0	0	0	0	0	0	0	0	0	0.085743	0	0	0	0	0	0	0	0	0	0	0	0	0	0	0
LOC107799855	3.575476	4.417414	4.238298	6.03669	6.412226	5.241918	5.628961	5.146095	5.376288	4.586572	5.382946	3.061044	3.841918	4.265704	5.573341	5.150792	4.340852	4.96071	5.403415	5.919818	3.949692	6.866141	5.396887	5.046065	4.544662	5.254359	2.655327	1.367364	4.28039	7.093381
LOC107760635	3.148783	3.826304	3.432325	1.765919	1.326786	1.934473	1.886443	1.622785	1.26695	0.58485	1.261674	2.280154	1.001506	1.635794	1.179873	2.718934	3.04199	1.751348	0.811471	0.761044	0.638165	1.794828	1.807537	1.932873	0.189572	0.417267	0.590732	0	1.203124	0
Novel04718	1.816183	1.415339	1.533008	0.309997	0.46582	0.339586	0.110385	0.089959	0.172982	0.102667	0.129197	0	0.087904	0.172293	0	1.039585	0.991722	1.29517	0.133546	0.089065	0.144034	0.109909	0.325042	0.632758	0	0	0	0	0	0
LOC107808097	0	0	0	0	0	0	0	0	0	0	0	0	0	0	0	0	0	0	0	0	0	0	0	0	0	0	0	0	0	0
Novel00312	0.862725	1.319807	1.339195	1.981878	1.663113	2.186333	1.809013	1.737535	1.706711	2.824251	2.074092	1.900228	2.435295	2.252136	2.18207	1.377652	1.875297	1.722853	1.844665	2.13729	1.376935	1.415238	2.554722	1.900054	2.064629	0.471593	2.075761	0	2.818424	0
LOC107806745	7.961285	8.781934	8.447875	8.406317	7.141836	9.267715	7.161013	7.083814	7.809464	6.558614	8.065689	6.725914	7.48738	6.888397	6.142522	5.35994	4.429111	5.628468	4.81928	4.714289	3.530263	4.516515	5.763194	6.025648	6.907004	3.246867	4.510118	1.963257	6.344021	6.789768
LOC107761411	5.624291	5.695386	5.382923	2.21312	2.010805	2.626388	2.430142	2.524126	1.706774	1.828131	1.633273	1.199888	1.328099	0.681758	1.282804	3.937214	3.89678	4.193148	0.980014	1.489805	1.19169	1.565664	2.004443	2.315776	1.077396	0.316194	0.738607	4.062791	0.547017	0
LOC107778007	11.62711	11.80723	10.04027	14.50983	14.76833	12.76962	18.16421	16.36564	13.30167	12.98978	17.6827	12.01843	19.86522	14.6377	18.88852	15.56461	10.56327	13.11757	17.15064	18.36873	14.06275	17.08914	15.14611	12.03193	12.68014	15.64258	8.509313	22.02657	15.4771	16.66375
LOC107788123	6.229885	7.152724	6.928219	9.080786	8.422902	12.36677	7.181102	6.229972	6.976191	9.146325	10.59154	6.968568	9.066121	6.891422	7.019313	6.911905	6.422074	7.978927	9.376536	11.30559	6.126144	8.138364	7.598071	7.384521	4.53227	3.244608	5.944054	3.733449	6.115868	0
LOC107761895	27.03837	32.27192	36.58344	12.09396	13.11388	23.23131	5.598394	5.799744	8.072157	6.288083	3.490418	5.161126	8.56387	9.503387	4.180133	23.09971	27.65145	34.15938	13.77573	15.1208	6.190655	6.141125	10.47787	8.9866	1.85942	1.888966	7.220449	8.090483	0	0
LOC107823828	2.797446	3.252845	2.515614	1.48565	1.365718	1.659364	3.276783	2.431424	1.811286	0.940642	1.082246	0.570466	1.073847	1.525938	2.673206	2.372199	1.910436	2.912669	1.517212	1.264825	1.055718	3.46405	2.297358	1.361121	0.731756	1.207999	0.342037	0	0	0
LOC107768431	15.60902	16.41785	16.32679	12.65733	9.849458	10.75673	11.99738	9.576236	10.47431	9.930923	13.6544	9.230851	10.45419	9.630968	11.03062	23.49254	17.94722	21.46202	8.386854	10.04381	7.677935	8.462388	10.40514	9.334056	3.882205	4.806621	4.082887	3.659886	5.66685	4.746536
LOC107818236	44.24055	45.22518	47.09221	33.69708	24.54913	33.40301	28.92175	25.20022	31.88046	18.64712	23.50393	19.85235	21.19031	23.53539	19.40252	70.86024	79.09665	82.80058	35.61186	37.38744	37.75403	55.62065	62.52958	72.76773	6.085742	10.4118	15.82627	6.258803	10.74429	10.82279
LOC107792935	0	0	0	0	0	0	0	0	0	0	0	0	0	0	0	0	0	0	0	0	0	0	0	0	0	0	0	0	0	0
107763373	0	0	0	0	0	0	0	0	0	0	0	0	0	0	0	0	0	0	0	0	0	0	0	0	0	0	0	0	0	0
LOC107768288	0	0	0	0	0	0	0	0	0	0	0	0	0	0	0	0	0	0	0	0	0	0	0	0	0	0	0	0	0	0
LOC107760052	0	0.035572	0	0	0.017269	0	0	0	0.018322	0	0.013684	0	0.021725	0	0.02003	0.058172	0	0.01455	0	0.016509	0	0.016298	0.017214	0	0	0	0	0	0	0
107798921	0	0	0	0	0	0	0	0	0	0	0	0	0	0	0	0	0	0	0	0	0	0	0	0	0	0	0	0	0	0
LOC107798590	0	0	0	0	0	0	0	0	0	0	0	0	0	0	0	0	0	0	0	0	0	0	0	0	0	0	0	0	0	0
LOC107819466	3.124929	3.187034	4.33336	3.168054	2.765143	2.079802	3.993924	3.687176	1.746322	2.321676	2.139031	1.508587	2.15349	1.379892	3.741898	3.714832	3.234583	3.605592	3.170962	2.391715	3.189272	5.157284	3.675192	3.032877	1.50509	2.89874	1.758769	0	2.50742	0
LOC107786947	7.672903	6.507848	5.860252	5.946744	5.18042	8.01089	6.026395	4.926426	6.703984	3.805898	5.069248	6.032644	5.579184	5.709536	3.96039	5.25514	7.626909	5.026082	2.610312	2.926465	3.478684	5.963367	5.594365	6.860798	0.953266	0.493701	2.149242	0	0.854106	2.742354
LOC107786192	4.931861	2.867361	2.439247	3.158425	3.160163	2.651177	2.781483	2.54288	2.634451	2.404593	2.563856	1.724099	1.987837	0.927658	1.396394	3.548496	3.039481	3.867097	0.949131	1.366694	1.473463	0.710125	3.525184	4.088273	1.075064	0.236632	1.105512	0	1.228124	5.257662
LOC107826325	1.51672	1.446597	1.379776	1.789489	1.123614	4.019766	0.7692	0.904135	1.09281	1.100647	1.558188	0.893977	1.570637	1.346822	0.832664	3.233074	3.407627	3.786796	3.14971	3.58058	1.704966	1.620137	2.769034	3.318019	0.356762	0.441713	1.125612	2.522487	1.358518	0
Novel01144	0.62394	0.568644	0.74125	0.339155	0.248446	0.089442	0.296551	0.417604	0.595545	0.192666	0.444799	0.11421	0.474629	0.453792	0.320197	0.619948	0.210972	0.341127	0.073865	0.105562	0.037936	0.303957	0.24766	0.33694	0	0.043408	0.049163	0	0	0
LOC107798346	1.449959	1.817006	1.890628	1.058169	0.60587	0.931	1.283706	1.032403	1.134404	0.628397	1.327374	0.612483	0.650129	0.659097	0.661421	2.236057	1.478603	2.011872	0.510874	0.511069	0.275497	0.958628	1.136853	1.073477	0.127305	0.140105	0.166613	0	0.290859	0
LOC107814838	0.498538	0.272614	0.202239	0	0.117637	0	0.641157	0.255579	0.312033	0.421319	0.815676	0.056157	1.10996	1.160279	0.511686	0.346744	0.256869	0.446018	0.202353	0.028115	0.060623	0.777172	0.058633	0.034738	0	0.092491	0.078564	0	0.480028	0
Novel01993	0	0.016827	0.01605	0.468345	0.424771	0.198501	0.185829	0.347058	0.156006	0.126027	0.116517	0.093589	0.164427	0.020142	0.0758	0.082552	0	0	1.348924	1.155769	0.841932	0.246704	0.37457	0.328059	0.116715	0.12845	0.130931	0	0.088888	0
LOC107805650	1.324872	1.98368	2.426761	3.129267	2.888966	2.492699	2.460575	2.304442	2.53212	2.030156	4.080969	2.238577	2.001604	1.34213	5.779202	2.080374	2.559876	2.751585	1.776513	2.121421	1.510382	4.109617	3.213718	2.621145	0.897343	0.724218	0.167774	0	0.911201	0
LOC107762831	0	0	0	0	0	0	0	0	0	0	0.173249	0	0	0	0	0	0	0	0	0	0	0	0	0	0	0	0	0	0	0
LOC107812710	1.873279	1.017791	2.787072	10.67212	15.30093	6.816629	25.62264	35.36667	23.303	11.10062	14.22164	12.29557	9.544533	16.89946	18.59825	1.583889	1.948956	1.63833	27.16057	20.05211	23.65563	27.88887	28.31248	16.60493	58.34448	71.4791	46.58052	69.55923	32.43239	80.18838
LOC107773908	3.994099	3.588127	3.769585	5.427678	4.998428	6.870633	5.072892	5.606524	5.731893	5.61957	3.680874	5.832764	8.066835	7.594267	4.802194	4.03941	3.142032	3.828562	5.268817	5.309438	6.192566	5.718132	5.989202	5.427021	5.410453	3.334499	4.383511	8.160988	5.768705	0
LOC107782354	9.164647	11.32367	12.34356	1.082962	0.609013	1.947256	0.24306	0.216655	0.272069	0.529843	0.482604	0.642662	0.846824	1.738814	0.48333	8.63811	8.85382	11.01863	1.212998	0.73543	0.165184	0.272264	1.118316	0.643641	0	0.050403	0.770647	0	0.174395	0
LOC107778294	9.310378	9.456857	7.722628	10.1088	6.854881	13.32092	9.994745	8.157458	8.273917	5.174682	6.943738	5.944282	8.623849	8.916595	5.543891	7.979703	9.475236	6.23479	4.375191	5.771681	4.472476	9.575737	8.107886	5.323687	0.658945	0.263709	1.512016	1.694208	0.798383	0
LOC107779829	11.0847	14.07703	11.74186	18.25975	19.20156	17.07837	19.3819	17.15661	14.18852	21.66227	20.46148	15.69733	19.89178	17.95757	23.46902	14.96339	11.49874	12.89292	18.79275	20.16289	15.19177	18.96952	14.50587	12.0289	23.60628	23.24068	18.81064	12.99529	23.8396	15.91738
LOC107821729	0	0	0	0.057905	0	0.034359	0	0	0	0	0	0	0	0	0	0	0	0	0.032429	0.027035	0.029147	0	0.028189	0	0.040405	0.222339	0	0	0	0
LOC107780991	10.49105	11.3956	9.622647	14.67819	13.37934	12.52338	13.52601	12.83312	13.10899	9.494118	12.27819	9.522018	11.45473	10.34735	11.18087	9.580258	11.35049	10.84134	10.54347	11.36473	9.866295	14.18204	13.44796	11.7804	7.347613	5.075879	8.385323	1.79918	6.298314	4.666743
LOC107829935	0	0	0	0	0	0	0	0	0	0	0	0	0	0	0	0	0	0	0	0	0	0	0	0	0	0	0	0	0	0
LOC107765558	0.688718	0.424109	0.404519	1.146706	1.087078	1.981219	1.342662	1.224631	1.188343	1.143512	2.545167	1.195147	1.906364	2.538368	2.636474	0.984853	0.611411	0.735508	0.717751	0.755815	1.714603	2.704835	1.264245	1.673159	0.329472	0.3885	0.638007	1.331163	1.164987	0
LOC107783720	0	0	0	0	0	0	0	0	0	0	0	0	0	0	0	0	0	0	0	0	0	0	0	0	0	0	0	0	0	0
LOC107808717	17.6022	21.08091	17.38614	8.028594	9.100747	7.473655	8.097886	8.89189	8.854429	8.383982	11.44477	10.18291	9.48073	8.648064	7.781494	22.09927	20.77541	20.91224	4.991319	5.639726	4.745609	9.149343	10.29111	8.32788	2.055846	3.195862	2.882825	0	3.620671	0
LOC107826497	0.341236	0	0.148315	0	0	0	0	0	0	0	0	0	0	0	0	0	0	0	0	0	0	0	0	0.35666	0	0	0	0	0	0
LOC107810052	3.044958	3.011283	3.125616	3.785708	4.098896	4.214021	2.907142	3.293635	3.892754	3.948429	3.543402	3.831331	4.435446	3.958008	3.324747	3.234684	3.504987	3.839927	5.55509	3.480104	4.224666	3.435653	3.800199	4.096738	4.750759	3.245237	3.445751	18.53256	5.926186	2.002919
LOC107825720	0	0	0	0	0	0	0	0	0	0	0	0	0	0	0	0	0	0	0	0	0	0	0	0	0	0	0	0	0	0
LOC107761015	27.01755	28.52947	23.10035	20.80773	23.61919	16.81177	26.75163	26.64571	23.64465	52.11456	36.83236	33.98804	29.9776	30.89374	41.14002	30.90516	28.30013	30.58874	28.85531	29.25639	35.61275	26.14254	21.40642	23.01735	113.3879	56.3483	114.9782	76.10168	72.43622	133.4235
LOC107796640	19.23454	26.09921	19.92985	15.36401	14.51257	12.50924	15.77273	12.32588	10.60115	13.52281	17.94288	7.653399	14.19511	10.44513	19.82993	14.23761	18.78957	15.4925	9.889639	15.94422	11.15967	6.561556	12.61099	6.372228	5.591038	3.405171	1.319365	6.140809	6.20102	2.654688
LOC107806818	0	0	0	0	0	0	0	0	0	0	0	0	0	0	0	0	0	0	0	0	0	0	0	0	0	0	0	0	0	0
LOC107775562	6.243945	8.708321	8.169006	9.370692	6.697034	7.933551	12.61657	12.0711	7.460843	4.551101	8.690179	3.037155	4.142424	2.924274	8.900853	8.694991	5.361934	6.277367	6.751919	8.323069	5.061852	17.40794	11.48802	8.141232	3.827513	5.221572	2.497219	2.255204	3.340064	19.49861
LOC107798220	1.71618	1.586116	1.533863	1.335791	1.646921	1.169432	1.804362	0.908725	0.74888	1.414219	1.796606	1.368194	1.910476	1.819529	1.116406	2.053445	1.657975	1.928239	1.349019	1.329086	0.837705	1.352482	1.449822	1.061092	0.733445	0.739925	1.428442	0	1.512816	2.989127
LOC107821925	0	0	0	0	0	0	0	0	0	0	0	0	0	0	0	0	0	0	0	0	0	0	0	0	0	0	0	0	0	0
LOC107788492	10.99755	8.877436	8.959027	9.593161	9.008455	10.81025	10.19924	9.342911	11.29848	7.93576	7.711489	10.29978	11.33317	8.878362	7.320805	8.94468	9.835306	9.909247	5.579777	6.698283	8.568444	9.525474	11.22494	10.96917	5.32334	5.683707	6.684799	11.23543	10.28668	5.828529
LOC107800276	7.374376	5.930148	7.447364	5.403597	4.702078	7.345389	2.910686	2.409155	4.072582	3.066728	3.041716	4.306083	2.776661	5.205641	2.003521	4.606433	5.971813	5.093652	3.081238	4.403446	5.340882	2.083034	4.782859	4.647287	3.153521	0.603583	3.717083	0	4.176809	0
LOC107831660	7.420144	6.715502	7.089582	13.67837	11.40742	10.79399	11.40812	9.515471	11.38848	10.87224	12.03479	9.463077	10.94657	8.813004	9.695285	7.914397	7.243284	7.80257	9.087718	10.79632	7.626448	10.27045	9.299673	9.684299	5.123015	7.631217	4.072336	2.768584	4.100404	0
LOC107790578	0.953598	0.807009	1.302624	0.431956	0.482175	1.025234	0.11426	0.058199	0.255794	0.06642	0.238808	0.115089	0	0.074309	0	0.203035	1.052864	0.406256	0.345588	0.345719	0.062121	0.056884	0.120162	0	0	0	0	0	0	0
LOC107761972	3.284055	2.859245	2.982346	3.806246	3.7339	8.067493	1.677308	1.630293	1.481321	4.472582	3.602116	3.378953	2.736786	3.182436	2.109019	3.240256	4.307037	5.348186	6.311143	7.309932	3.865202	1.424926	1.375553	1.802538	2.412361	1.965661	2.081692	1.312047	3.533097	1.134403
LOC107814196	0	0	0	0.190672	0.209514	0.028285	0.507515	0.517005	0.123497	0.615693	0.940816	0.22026	0.17572	0.143505	1.2961	0.019605	0	0.453829	0.53412	0.455878	0.834887	0.510524	0.357456	1.696344	1.061575	0.528602	0	0	1.139916	0
LOC107768301	462.0798	376.7727	475.2702	219.9552	204.3846	309.4979	208.6696	230.2198	301.6193	130.6169	139.0661	160.9682	105.9491	136.5513	116.5989	279.2252	444.6753	375.068	136.6122	131.098	188.3777	215.6715	291.9425	358.54	52.6195	30.11678	105.1059	203.437	35.72725	34.41378
LOC107799039	21.38813	13.8555	13.89466	0.737312	1.415	0.979994	0.901053	1.251685	1.222534	2.388748	4.702372	3.767873	3.551503	2.343995	2.906911	24.74464	20.91433	27.18303	3.66675	2.808941	4.809781	1.359345	1.550609	1.565181	0.452743	0.362375	2.885736	0	1.880732	0
LOC107801743	42.7035	21.28154	24.46944	5.071357	5.307144	6.706172	6.439059	7.857694	9.835286	4.834758	6.785	5.81017	8.902272	16.13984	7.879497	13.94478	24.56769	14.54737	2.515561	2.097099	3.865458	4.875253	5.995733	4.429935	0.808842	0.556356	3.21358	0	2.309998	0
NitaMp021	0	0.172028	0	0.085502	0	0.405871	0	0	0.088606	0	0	0	0.239199	0.210126	0.205923	0	0	0.070331	0.182354	0.070363	0	0.086074	0.157635	0	0.394575	0.357967	0.26264	0.223094	0	0
LOC107764520	0.967011	0.860328	0.480344	0.646618	0.529704	0.742611	1.042809	0.845913	0.799792	0.381673	0.339034	0.311222	0.948337	0.728426	0.73257	0.77209	0.533837	0.858271	0.841077	1.14913	0.545957	0.57684	0.731115	0.697878	0.029109	0.192218	0.10885	0	0.332538	0
LOC107774730	20.76027	7.609754	11.77978	1.653429	2.099435	2.305565	3.520767	3.664583	5.740106	7.830602	2.847436	5.859134	6.196695	8.362499	3.886913	5.576209	6.567844	6.599267	0.555589	0.463167	0.749026	5.182181	2.776974	3.481319	14.13316	0.19046	9.706933	9.78892	30.97269	2.821184
LOC107762492	2.049381	1.648022	2.462639	2.784953	2.400124	3.110591	3.28614	2.26606	2.037228	3.056384	3.634833	1.935065	2.549799	3.156386	2.289025	1.706872	2.154569	2.471603	2.324225	2.294517	1.539234	1.761829	2.233028	2.142012	1.219313	1.425782	1.638549	0	2.321517	0
LOC107806005	6.971407	7.359357	6.270679	7.900773	5.954401	6.250752	7.043755	7.198468	6.747228	5.223175	6.285028	5.230181	5.393514	5.168191	5.884443	9.848627	5.980857	7.685849	7.729618	7.286277	6.873557	7.148252	7.217643	4.329982	3.641296	4.456851	3.499462	5.774759	4.535518	1.664296
LOC107765784	0.777917	0.807444	0.525954	0.430683	0.21033	0.394946	0.453106	0.461579	0.344884	0.147499	0.136369	0.109534	0.336773	0.259316	0.088715	0.644116	0.709782	0.789404	0.350834	0.237634	0.315322	0.649659	0.45745	0.316197	0.300521	0.21047	0.204319	0	0.104033	0
LOC107823343	10.53901	13.50447	9.905203	10.64948	12.91238	10.55597	14.35477	13.81508	10.6556	17.21594	15.12731	16.39945	16.84619	14.88784	14.88476	12.14971	14.83724	12.65895	16.58981	15.15249	22.59172	11.99849	12.3948	12.80419	17.99378	17.23364	21.13305	32.20889	15.39502	16.70879
LOC107823171	0.591384	0.333651	0.391678	0.357176	0.323945	0.605534	0.18896	0.312802	0.608096	0.549214	0.671385	0.951653	1.06588	1.198175	0.578077	0.314786	0.326473	0.482893	0.14288	0.119112	0.436619	0.258699	0.19872	0.29434	0.106813	0.195921	0.299558	0	0.135578	0
LOC107768824	1.060856	0.48342	0.518726	0.24027	0.117339	0.285137	0.500504	0.113303	0.311243	0	0.046492	0.056015	0.14762	0.144668	0.068052	0.296457	0.256219	0.197728	0.06728	0.112176	0.18141	0.221487	0.233936	0.415802	0	0	0.156731	0	0	0
LOC107817801	4.943926	4.387981	5.561664	1.727175	1.429644	2.431848	0.65046	0.635014	1.061798	0.346604	0.566452	0.272991	0.251801	0.458278	0.165827	3.516566	3.652425	4.215906	0.950888	1.066056	0.795696	0.485741	1.567633	1.553594	0.040854	0	0.343725	0	0.155567	0
LOC107810489	0.158606	0.14455	0.20681	0.035922	0.105259	0.213152	0.099773	0.067759	0.186133	0.115996	0.083411	0.100496	0.132422	0.173032	0	0.236387	0.191534	0.236496	0.040236	0	0.072326	0.132456	0.139901	0.124332	0	0.055172	0.046865	0	0	0
LOC107820345	12.77293	8.771475	8.584018	7.033736	7.344933	7.231663	5.131537	6.786572	6.583729	8.129039	7.225322	7.345086	7.647198	8.392012	9.657912	9.758365	7.120403	8.695857	8.495505	9.382513	7.92924	6.005796	6.753631	7.067788	7.282824	10.75317	7.78087	7.676011	10.16278	6.63672
LOC107795284	12.95353	16.00708	14.57929	9.147337	8.734245	7.449832	9.512526	10.29455	13.99351	3.668048	4.601006	4.587668	3.841148	3.671763	4.702649	13.48888	14.80938	14.8024	5.309396	4.928614	4.92673	12.49483	16.01617	17.08636	1.072742	0.826423	2.5071	2.022621	0.953145	0
LOC107823534	2.068011	1.50779	1.489505	1.579104	1.202514	1.492831	1.709751	1.590268	1.248133	0.921861	1.5951	1.622319	2.400802	1.224739	1.27353	1.36496	1.027477	1.828122	1.199128	1.524473	1.751342	1.801066	2.188947	1.852701	0.971135	1.027674	1.291946	0	2.41792	10.96013
LOC107828082	4.908661	5.776091	5.266228	1.745025	1.621942	2.672107	2.240956	3.158842	3.354264	1.060315	1.764547	1.1811	1.556314	1.220151	0.637736	2.801334	4.291972	3.868074	0.851178	0.946114	0.878356	3.476665	3.946114	4.35121	0.235672	0	0.917981	0	0.29914	1.920949
LOC107830648	2.352528	1.268921	1.919799	1.130879	1.699324	1.342054	1.489942	1.312695	1.487463	1.264047	0.774299	1.014021	1.443049	0.995171	0.886985	1.538441	0.510207	1.038028	0.535898	0.365524	0.6568	0.32076	1.016368	0.752713	0	0.0607	0.133607	0.170235	0	0
LOC107813432	0	0.131148	0.250179	0	0.127333	0	0	0.122952	0	0	0	0	0	0	0	0	0	0	0.107235	0.27804	0.321852	0	0.12173	0.13124	0.360525	0.634649	0	0	0	8.897581
LOC107792152	0	0	0	0	0	0	0	0	0	0	0	0	0	0	0	0	0	0	0	0	0	0	0	0	0	0	0	0	0	0
LOC107825139	0	0	0	0	0	0	0	0	0	0	0	0	0	0	0	0	0	0	0	0	0	0	0	0	0	0	0	0	0	0
LOC107798521	5.962795	5.499434	5.152293	16.04423	14.69142	18.19574	13.08711	11.86747	10.3247	27.4708	22.38274	16.37953	29.77103	26.09837	16.93091	7.742834	7.105842	7.826239	15.57947	18.45481	10.12737	10.91356	11.21204	11.79288	32.59283	17.93499	28.14797	17.87407	53.11658	35.32346
LOC107789467	7.71371	8.302208	8.301879	3.327729	2.730239	4.502013	2.464694	2.573552	3.517528	1.934188	1.545388	1.986059	1.635621	2.885238	2.639047	5.036575	7.38112	6.517684	1.640014	1.926508	2.613012	3.251636	3.434401	4.760636	0.185761	0.10222	0.781456	0	1.768407	0
LOC107822470	0.778468	0.608123	0.386689	18.75956	42.80646	28.69536	36.47105	61.47831	70.47577	58.77637	54.74217	102.5028	38.87325	61.3901	63.91984	4.889542	0.646627	1.906965	40.2867	39.32366	50.30685	49.78052	34.33	48.9355	42.74327	88.35663	45.21567	95.43678	102.262	130.649
LOC107798676	16.18076	13.52087	13.82486	16.05674	13.47763	16.80082	10.81738	11.22248	13.37123	10.14594	10.68018	9.659209	11.27442	10.4447	9.014332	13.94473	12.34505	14.48203	12.68567	13.05194	11.61808	10.63858	13.3303	16.78807	5.802098	3.853311	7.995756	2.829225	6.856717	0
LOC107817106	0.213795	1.558782	0.433644	0.129125	0.25224	0.153237	0.179139	0.182672	0	0	0.199884	0.301033	0	0.23324	0.073144	0.106213	1.445807	0.371917	0.14463	0.120571	0.324974	0.178546	0.06286	0.96832	0	0	0	0.084229	0	0
LOC107791577	3.36437	5.075104	4.386863	4.887228	4.619508	5.67512	6.130209	5.204034	5.064682	2.488796	4.880893	2.058227	2.841235	3.164105	8.21599	3.760699	3.194225	3.070482	2.589878	3.238581	0.634834	7.217954	5.679366	3.45581	0.95339	1.614233	0.895123	0	3.35116	0
LOC107828321	1.804395	3.145969	1.773112	3.458911	3.216435	3.064552	3.728726	2.860011	2.798858	2.142016	3.410655	1.148826	2.299791	1.569099	3.757644	4.28725	2.501109	3.606838	3.104698	4.313723	1.049393	5.110348	3.344658	2.623935	1.620056	2.692618	1.112687	0	0.88129	0
Novel00606	14.14794	11.36846	13.3312	13.20868	12.0412	13.81741	10.19068	9.550743	11.00132	11.47913	10.33705	15.146	9.678238	11.13419	5.819506	7.1226	10.06848	8.736227	8.602849	10.46074	12.41067	6.268428	9.14522	11.62675	5.939673	4.733613	11.99879	0	5.199481	0
LOC107815261	0	0	0	0	0	0	0	0	0	0	0	0	0	0	0	0	0	0	0	0	0	0	0	0	0	0	0	0	0	0
LOC107813300	1.058177	1.008234	1.254342	2.570938	2.979278	4.136981	1.896105	2.013756	1.851454	5.253086	2.900523	4.429251	2.837855	2.466259	1.727861	0.896085	0.929353	1.326813	2.53799	2.604058	1.666948	1.164892	1.357647	2.513651	2.189221	1.606228	3.467795	0	1.852524	0
LOC107782505	3.932852	3.770163	2.152543	2.771195	2.887149	3.163392	2.614487	2.688268	3.555582	3.295283	4.412349	3.740982	4.994283	2.923929	2.780749	2.257775	3.433591	1.933019	1.005087	1.355413	2.019251	2.94382	2.415477	4.750045	0.589329	0.770172	1.239551	0	0.701265	1.80129
LOC107792079	156.2905	139.6401	176.474	83.40962	80.78899	94.6399	69.15405	75.45447	111.8233	46.50519	42.39673	46.74446	37.70346	39.85953	24.68201	120.2026	142.7121	141.2598	53.11035	48.30985	63.51066	74.25629	117.6094	156.8916	33.26527	12.09087	59.13806	8.67105	26.07363	19.9921
LOC107776471	0	0	0	0	0	0	0	0	0	0	0	0	0	0	0	0	0	0	0	0	0	0	0	0	0	0	0	0	0	0
LOC107827606	10.55298	15.37435	14.59722	25.82024	25.90094	14.57556	39.86288	38.83128	26.9024	24.26152	32.13763	20.43396	16.72122	21.51294	35.26099	21.23879	17.56231	16.30968	38.22208	44.57032	38.07648	43.42206	35.33126	24.79731	29.70339	48.65986	24.21721	22.03467	13.35043	28.57694
LOC107777978	15.70622	17.14201	18.54474	18.90804	15.67137	17.23833	16.61637	14.9347	14.92662	12.23365	13.48612	10.55012	11.45603	10.84848	13.21282	18.65506	16.75604	17.29868	13.43451	15.99256	12.18008	18.36334	15.82577	16.01329	7.991705	13.30014	8.56426	9.647261	5.845112	17.87373
LOC107817231	8.632237	7.644562	8.990426	5.946432	5.534216	4.500269	5.792183	5.441257	7.217436	2.414081	3.597496	2.407985	1.885643	2.096692	2.657967	8.059147	6.907596	7.795696	2.925308	3.51336	3.802684	7.535452	8.820983	11.72913	1.338481	0.747863	1.559267	1.164768	1.568251	5.035323
LOC107769976	0.778881	0.709854	1.015597	0	0.430753	0.52337	0	0.166374	0	0.664569	0.204807	0.65802	0.325148	0.106215	0.299783	0.725529	1.410871	0.362931	0.395177	0.4118	1.331911	0.081308	0.171756	0.101761	0	0.677348	0.345216	0	0.468726	0
107789595	0	0	0	0	0	0.771434	0	0	0	0	0	0	0	0	0	0	0	0	0	0	0	0	0	0	0.907177	0	0	0	0	0

LOC107764746	1.225839	0.603892	0.806397	0.600296	0.410428	0.498675	0.361247	0.169847	0.311046	0.613827	0.743402	0.391857	1.180213	0.759025	0.510068	0.81474	0.672149	0.815113	0.302569	0.252237	0.453237	0.193679	0.4968	0.380911	0.041887	0.046099	0.274105	0	0.47851	0
LOC107805727	4.756768	6.22556	6.274558	4.760374	3.744143	4.876242	4.337646	4.584166	4.154292	3.317031	3.587547	3.08408	4.125409	3.650683	4.059052	5.275906	5.26338	5.979349	3.676256	4.187662	3.329357	4.688475	4.512907	3.72883	3.111896	3.38631	3.366733	0	3.062313	3.419978
LOC107782930	0.939872	0.724796	1.068398	0.753229	0.671725	0.388645	1.849498	1.606103	1.798724	0.563996	1.039253	0.519173	0.965795	0.788734	1.224374	1.34691	1.187378	0.99717	0.440177	0.519851	0.527495	2.052845	1.052229	1.020136	0.182813	0.251493	0.1709	0	0.522101	0
LOC107806428	0.071753	0	0	0	0	0	0	0	0.067365	0.209905	0.251567	0	0	0	0	0	0	0	0	0	0.06544	0	0	0	0	0	0	0	0	0
LOC107803053	6.764274	7.045487	6.741584	6.150537	5.196181	5.247865	7.689342	6.647575	8.281366	4.010767	5.941922	3.977226	5.792372	6.595588	5.772839	7.958099	5.48162	6.631707	3.042249	3.647059	2.982877	8.214878	7.452707	6.293092	2.380804	2.413332	1.698535	1.771946	3.57864	0
LOC107791806	0	0	0	0	0	0	0	0	0	0	0	0	0	0	0	0	0	0	0	0	0	0	0	0	0	0	0	0	0	0
LOC107789692	5.33164	6.697723	7.119038	6.766626	11.73071	3.821443	9.689065	11.20406	8.500465	24.21527	15.94778	12.70136	17.45821	16.42784	19.12822	14.63978	8.468678	9.865789	22.44494	17.04531	17.50078	13.5784	12.37149	12.02383	60.72805	42.13892	33.40967	30.91522	65.78976	43.06413
LOC107817028	0	0.038732	0.092358	0	0.075211	0.091383	0.071291	0.054468	0.05985	0.352254	0.119201	0.053856	0.378482	0.440459	0	0.09501	0	0.174266	0.08625	0.179755	0.155038	0.070984	0.074973	0.06663	0.214926	0.059134	0.226036	0	1.943741	1.313882
LOC107779635	6.850906	5.899612	5.416083	4.838195	3.603866	5.770657	4.479282	3.963855	4.127579	3.313948	4.047346	3.349653	4.323699	4.266655	2.685289	5.105303	5.211458	6.012538	2.299029	3.194312	2.361502	3.311187	3.354552	4.031342	1.398137	0.825653	2.263396	0	1.558238	0
LOC107783908	0.145298	0	0	0	0	0	0	0	0	0	0	0	0	0	0	0	0.070185	0.054163	0	0	0	0	0	0	0	0	0	0	0	0
LOC107759400	0.800564	1.302885	0.646205	10.95676	18.89888	4.088345	10.3358	9.454184	7.999222	3.178365	2.686489	3.091834	4.869766	5.333832	6.602762	1.491453	0.856278	1.193709	36.26594	20.77621	58.61884	8.834701	9.356493	4.602121	17.13436	22.47744	5.440663	8.178807	16.2427	54.80366
LOC107797752	20.43847	23.35143	22.18696	18.8293	14.54688	19.21386	14.82382	14.55266	15.15617	14.24954	13.74298	16.14088	10.88158	12.60286	14.9984	20.85954	17.02673	17.9246	14.67799	17.83245	16.25392	15.66693	15.5895	15.47992	5.305405	5.632775	8.11056	0	5.704237	12.21006
LOC107783819	1.265619	1.30725	1.246866	1.05486	1.209495	1.088556	1.245528	1.312074	1.204062	0.773394	1.301598	0.613025	1.18348	1.25186	1.108477	0	0	0	0	0	0	0	0	0	0	0	0	0	0	0
LOC107778816	0	0	0	0	0	0.111294	0	0	0	0	0	0	0	0	0	0	0	0	0	0	0	0	0	0	0	0	0	0	0	0
LOC107779318	1.980677	2.032682	2.14135	4.553839	4.639324	3.3821	4.495227	5.105476	3.312807	3.603136	4.668411	2.657644	3.557514	3.250312	6.782071	2.493081	2.138619	2.605904	6.637587	7.180786	3.552102	7.367069	4.169522	2.887847	8.43845	11.4639	4.603313	4.761225	4.888041	4.11658
LOC107768757	0	0	0	0	0	0	0	0	0	0	0	0	0	0	0	0	0	0	0	0	0	0	0	0	0	0	0	0	0	0
Novel01231	2.793587	1.803424	2.124855	3.110835	1.544969	3.504023	0.488146	0.795639	0.655686	0.397265	0.816194	0.688363	0.583099	1.142875	0.597346	1.214374	1.686775	1.17154	2.244169	2.65858	1.22082	0.583249	1.488744	0.851625	0	0.080981	0.619087	0	0.280194	0
LOC107828698	0	0	0	0	0	0	0	0	0	0	0	0	0	0	0	0	0	0	0	0	0	0	0	0	0	0	0	0	0	0
LOC107800979	1.212845	4.214181	1.91092	0.274694	0.134151	0.081497	0.063579	0.129536	0.071167	0.073917	0.372071	0	0.084385	0	0.622416	19.37551	2.489885	2.486626	0.999953	2.629083	0.069133	2.848722	0.534905	0	0	0	0	0	0	0
Novel03674	0.209379	0.286235	0.212344	0.189687	0.123515	0.375181	0.292693	0.08945	0.032762	0.204171	0.269165	0.029482	0.038847	0	0.143268	0.286055	0.202278	0.130084	0.354106	0.501842	0.190958	0.116572	0.123124	0.072948	0	0.048556	0	0	0	0
LOC107763458	0.528732	0.787062	0.505577	0.734474	0.935715	0.871626	0.783465	0.602351	0.67841	1.048348	1.099884	0.521136	0.863269	0.73064	0.886372	0.696088	0.40864	0.906643	0.894202	0.864724	0.353623	0.750649	1.010483	0.736841	0.445652	0.441416	0.499935	0	0.339401	0
LOC107775348	4.54395	5.253825	4.34299	11.2643	12.88252	10.84002	12.98844	12.76774	9.699451	9.280694	10.28785	11.38287	16.10631	12.67669	17.00774	5.8626	5.831277	6.033827	10.55228	8.969028	12.04074	13.44218	11.60544	9.711327	9.88929	9.814146	9.699113	9.700199	12.29856	15.37588
LOC107803691	4.523707	3.471832	3.725396	4.961038	4.424228	8.191222	4.992405	3.661747	3.576467	3.482515	5.175411	1.810304	2.915494	4.155909	3.909902	3.903346	3.680237	3.550121	6.523122	5.236598	5.211406	5.965051	4.620241	4.230474	4.214251	4.969269	5.065254	0	8.023744	14.72145
LOC107796106	1.056044	1.828662	1.560594	0.430525	0.467228	0.794763	0.088575	0	0	0.257444	0.1481	0.089217	0	0.288023	0	1.849368	1.785397	1.850214	0.5358	0.178668	0.288939	0.044096	0.1863	0.055189	0	0	0.249632	0	0	0
LOC107815565	2.159516	2.093759	2.596158	2.664051	2.520738	3.853108	3.391332	3.533265	6.384308	2.285018	1.933091	2.950125	4.091921	5.313366	1.980674	2.67071	2.61894	2.603422	4.056288	2.759638	3.687603	2.877875	3.404388	3.505749	0.929459	0.511457	1.411952	0	3.31809	0
LOC107765765	25.81169	21.54228	25.80729	13.70496	14.34813	16.72159	11.5384	12.52162	14.82394	7.145232	9.812005	9.70506	7.104543	9.386427	7.132572	15.57111	23.06376	18.04538	9.69005	8.79795	11.12362	11.37021	16.63787	17.68911	4.90105	1.710242	6.928406	0	2.503541	0
LOC107823567	0	0	0	0	0	0	0	0	0	0	0	0	0	0	0	0	0	0	0	0	0	0	0	0	0	0	0	0	0	0
LOC107796431	0.903676	1.921709	1.251056	1.182545	0.858864	1.151482	2.723014	4.089406	2.890883	0.522192	1.267317	0.367587	0.633398	1.168434	0.652691	4.888536	6.822547	3.144069	0.543401	0.39638	0.366297	3.018735	2.538919	2.483744	0.338525	0.139711	0.356024	0	0.80567	0
LOC107827166	0.069377	0.094843	0.090462	0.15713	0.061389	0.037294	0.029095	0.088917	0.130269	0.202954	0.072971	0.058612	0	0.075687	0.071207	0.07755	0.067024	0	0.0352	0.029344	0.126546	0.086908	0	0	0.175428	0.193067	0.286994	0	0	0
LOC107768931	3.868428	2.274577	2.576294	15.40328	15.59691	7.680795	25.09345	31.42691	29.37698	38.23921	44.42885	48.81148	29.20703	32.19654	43.93109	3.463958	1.95903	3.512062	36.05682	42.304	42.59293	30.89937	26.38959	21.09679	22.91609	50.26687	54.42358	17.84821	20.5765	11.57374
LOC107829880	0	0	0	0	0	0	0	0	0	0	0	0	0	0	0	0	0	0	0	0	0	0	0	0	0	0	0	0	0	0
LOC107769500	2.660007	3.557104	3.608897	5.134968	4.443526	4.516932	5.004252	4.991624	4.574551	3.902885	5.124938	3.612398	3.846736	4.013894	4.771398	3.33461	3.60251	3.465876	3.582104	4.584485	3.242186	5.605506	4.034762	4.677037	3.425918	2.628894	3.966621	1.777833	2.274002	9.222742
LOC107813466	0.056081	0.255555	0.341251	0.203227	0.099249	0.180882	0.047038	0.047917	0.210606	0.328117	0.353917	0.331652	0.06243	0.061182	0.05756	0.25075	0.325074	0.209054	0.056907	0.047441	0	0	0.098935	0	0.070904	0	0	0	0	0
LOC107780576	0	0	0	0	0	0	0	0	0	0	0	0	0	0	0	0	0	0	0	0	0	0	0	0	0	0	0	0	0	0
LOC107767524	14.54065	12.21622	13.65688	9.811682	9.642497	8.4454	10.34546	9.168	14.12214	9.746004	7.055102	7.540046	6.623586	8.205069	3.568066	10.83571	13.17558	11.81259	5.325312	5.429137	7.255617	10.80333	15.09613	17.39895	11.32611	3.906918	13.59067	21.51439	19.15048	22.73511
Novel03752	1.333101	1.316204	1.207121	0.704505	0.442356	0.41803	0.093177	0.616979	0.573638	0.054164	0.31159	0	0.061834	0.181793	0	0.993428	0.965916	1.076707	0.338185	0.23494	0.303953	1.159695	0.881912	0.812791	0.070227	0	0.065651	0	0	0
LOC107773189	0	0	0	0	0	0	0	0	0	0	0	0	0	0	0	0	0	0	0	0	0	0	0	0	0	0	0	0	0	0
LOC107824777	0.081971	0	0	0	0.072534	0.088129	0.137506	0	0.076958	0	0.229914	0.277007	0.273755	0.536561	0.336533	0.061085	0.316765	0.18334	0	0	0	0.136913	0.144608	0	0	0	0	0	0	0
Novel00611	0.10476	0.127301	0.151775	0.031636	0.061799	0.112629	0.380754	0.208855	0.196706	0.374564	0.220372	0.295013	0.466479	0.152383	0.179204	0	0	0	0	0	0	0	0	0	0	0	0	0	0	0
LOC107769756	0.060345	0.219989	0	0	0	0.064879	0	0	0	0	0.042314	0	0	0	0	0.044969	0.116597	0.08998	0	0.102096	0	0	0	0	0	0	0	0	0	0
LOC107810436	4.295894	3.640428	2.620582	1.911792	1.333786	1.296452	0.695345	1.030324	1.132119	2.278249	2.16673	2.228512	2.600872	3.124406	2.475334	3.257469	5.02392	2.809449	1.070675	0.446284	1.512183	0.755287	0.265913	0.551412	0.476431	0.524335	1.425235	0	0.362841	0
Novel02087	3.270935	3.47109	3.038104	1.136609	1.427345	1.589721	0.789218	1.454815	1.00961	0.349542	0.377027	0.378545	0.448921	0.928769	0	1.202057	1.601643	2.20478	0.86388	0.87179	0.694706	1.64647	2.134243	1.966969	0.05665	0	0.158876	0	0.215718	0
LOC107771939	0	0	0	0	0	0	0	0	0	0	0	0	0	0	0	0	0	0	0	0	0	0	0	0	0	0	0	0	0	0
LOC107821804	0	0.039815	0	0.197887	0.193282	0.093936	1.648868	1.791681	0.492174	0.894591	1.347843	0.036907	0.389057	0.238298	1.61418	0.78132	0	0.06514	0.886595	0.480422	0.717166	1.349889	0.693614	0.091322	1.546514	2.917736	0.516336	0	0.630963	2.701184
LOC107827585	5.416437	4.765209	5.57931	12.84901	13.13177	15.21472	7.037709	7.196062	5.908226	11.99834	4.390762	5.898487	6.691869	6.45557	3.502669	4.152992	5.444462	4.901841	13.47052	10.27624	13.04928	4.967905	5.689	8.410077	34.99221	9.19201	31.4909	26.86833	49.44013	13.55113
LOC107791307	0	0	0	0	0	0	0	0	0	0	0	0	0	0	0	0	0	0	0	0	0	0	0	0	0	0	0	0	0	0
LOC107821365	120.8545	133.2094	122.8046	197.5937	190.2777	188.8161	200.8237	198.6992	186.6675	155.6708	136.5463	151.0137	152.0742	152.8797	121.1231	124.39	142.799	114.5675	175.2668	172.0909	197.8278	154.1716	176.4822	156.871	92.97237	95.35673	134.8412	146.4193	113.9031	84.3966
LOC107828750	9.131998	11.14926	10.14748	11.12189	10.15156	9.416649	11.58544	9.532455	10.90563	9.380897	10.92398	7.872661	11.58845	9.774588	11.9371	11.44896	8.572591	11.03671	9.10625	9.498401	7.005922	11.30765	11.17065	10.00999	7.715234	6.992576	5.549379	3.080505	12.78847	11.54149
LOC107795094	6.49007	5.914898	6.652924	7.821076	6.609685	9.643596	7.317914	7.245499	7.616454	6.000265	6.504268	5.922665	7.633789	7.314132	4.55603	6.205202	7.186786	6.915578	4.411159	5.464256	4.271769	6.084747	7.479876	8.927236	1.819126	1.789053	3.147898	0	1.768614	0
LOC107830180	0.106463	0.145542	0.277638	0.09645	0.047103	0	0	0	0	0	0	0	0	0.058073	0	0	0.051426	0.039686	0	0	0	0	0	0	0	0	0	0	0	0
LOC107772595	0	0	0	0	0	0	0	0	0	0	0	0	0	0	0	0	0	0	0	0	0	0	0	0	0	0	0	0	0	0
LOC107813239	0.118372	0	0	0	0	0	0	0	0	0	0	0	0	0.129138	0	0	0	0	0	0	0	0	0	0	0	0	0	0	0	0
LOC107822328	2.653158	2.60121	3.424556	1.638836	1.778552	3.241442	0.539471	1.167812	1.132228	0.783989	1.268452	0.950923	1.342515	0.789399	0.783931	2.186832	3.145713	2.78724	1.34612	0.850147	0.8799	0.906431	1.276506	1.722671	0.96566	1.342427	1.472885	0	0.580602	0
LOC107811465	2.666606	1.874789	2.152451	1.000836	0.606751	1.064859	1.054396	0.716075	1.108705	1.188698	0.534236	1.062045	1.314623	0.872741	0.664683	1.447787	2.64977	1.732459	0.3479	0.257802	0.208456	0.159068	0.235212	0.199081	0	0.053006	0.045024	0	0.1834	0
LOC107773559	0	0	0	0	0	0	0	0	0	0	0	0	0	0	0	0	0	0	0	0	0	0	0	0	0	0	0	0	0	0
Novel01440	1.775585	0.970936	1.234783	1.166228	1.256924	1.193107	1.042482	0.758554	1.291923	1.255279	1.213915	1.237553	1.72953	1.162246	0.728965	1.124693	0.814798	1.489246	0.945914	1.126517	1.174039	0.444851	1.057174	1.299089	0.785708	0.494119	1.154229	0	0.85483	0
LOC107766706	104.3653	107.4355	101.6405	102.8157	89.15964	120.0774	77.56801	84.55268	101.5255	84.08463	61.45149	86.26737	67.27581	79.75546	57.74988	84.06884	102.9477	89.60812	88.29984	85.99888	116.5515	67.59108	85.0025	94.4244	49.98839	47.49125	105.6441	80.55685	69.68702	73.13238
LOC107818683	2.644107	6.650986	3.631577	25.58316	13.28938	5.401203	117.85	99.56281	35.39908	29.34149	92.31313	14.27408	28.46323	16.70178	74.54916	7.99979	3.729467	2.503543	19.85462	43.01237	12.44319	85.3455	66.32997	15.39336	32.66067	38.4832	8.844095	35.9273	3.691605	13.07913
LOC107816787	1.050747	0.191525	0.593705	0.142789	0.371908	0.564841	0.044065	0.359115	0.049324	0.204922	0.184196	0.221925	0	0.343894	0	0.195755	0.304533	0.430857	0.373179	0.355544	0.239575	0.087751	0.32439	0	0.132846	0.073102	0.24838	0	1.517602	0
LOC107803260	25.26604	19.61984	18.63886	18.06266	19.84759	15.10655	20.18258	21.14742	22.3896	22.83585	26.36393	30.92912	32.67075	27.89215	16.62671	20.33326	25.11482	22.87338	18.13861	17.19317	20.22145	15.82525	28.35062	23.71336	16.18928	24.87216	18.84177	27.65631	23.16947	21.25493
LOC107796596	0.07275	0.265209	0	0.197723	0.257495	0.312859	0	0.062159	0.068301	0.21282	0.408097	0.061461	0	0	0	0.271066	0.070282	0.325428	0.369107	1.046201	0.464442	0	0.06417	0.38019	0	0	0	0	0.350243	0
LOC107801333	2.045197	2.62333	2.14	2.642019	3.552422	3.257536	3.04959	2.168159	3.05797	2.917624	4.620967	2.335782	3.415112	2.933627	1.982515	3.358627	2.634443	2.202461	2.113756	2.69126	0.690835	2.467107	2.30511	2.889771	0.909801	0.47429	1.298157	2.708528	1.094034	0
LOC107798114	0	0	0	0	0	0	0	0	0	0	0	0	0	0	0	0	0	0	0	0	0	0	0	0	0	0	0	0	0	0
LOC107784667	36.72572	62.49944	43.71664	31.47999	30.30373	21.96586	38.66492	36.889	32.50534	29.99969	35.52754	29.08552	42.75955	37.36146	47.38586	99.59188	59.80629	69.48459	27.34589	28.77007	25.6067	38.77733	31.75571	30.74351	28.52507	39.6097	21.74457	30.87549	27.15523	36.16757
LOC107821095	0.405521	0.246388	0.176255	0.673532	0.358831	0.581313	0.170064	0.115496	0.126907	0.329528	0.473919	0.74229	0.601908	1.179742	0.138738	0	0	0.251944	0.342912	0.343043	0.308201	0	0	0.211925	0.08545	0	0.718939	0	0	0
LOC107824903	1.839969	2.326029	1.341471	1.693804	1.680646	0.60622	7.766089	7.83524	1.783168	2.054651	5.098321	1.053019	2.378647	1.197914	3.198255	5.61728	3.096403	3.451563	1.264792	1.531383	4.465865	5.898585	7.643685	3.536087	0.525289	0.619399	0.947045	6.366958	0.571501	0
LOC107813995	2.898061	1.56517	1.959395	0.583444	0.474888	0.923191	0.180054	0.275131	0.503857	1.046657	0.677374	0.54408	0.119487	0.351294	0.220333	0.399933	1.659123	0.880256	0.32675	0.453993	0.195784	0.268916	0.473385	0.78531	0.27141	0.14935	1.395482	0	0.516752	0
Novel00834	0	0	0	0	0	0	0	0	0	0	0	0	0	0	0	4.488564	4.419498	4.973787	3.094694	4.418068	3.754943	3.279186	4.405034	5.89709	1.783323	1.326103	1.261603	0	1.6518	0
LOC107771340	0.20413	0.502306	0.390381	0.092466	0.072251	0.241411	0.119849	0.209297	0.134152	0.059716	0.085882	0.103473	0	0.066809	0.062854	0.42593	0.157765	0.36525	0.144996	0.08634	0.074468	0.187522	0.360112	0.106678	0	0.056806	0	0	0	0
LOC107774643	0	0	0	0	0	0	0	0	0	0	0	0	0	0	0	0	0	0	0	0	0	0	0	0	0	0	0	0	0	0
LOC107823249	0	0	0	0	0	0	0	0	0	0	0	0	0	0	0	0	0	0	0	0	0	0	0	0	0	0	0	0	0	0
LOC107804443	358.162	283.233	387.3318	77.6871	67.04337	116.867	69.57308	79.25622	113.1551	53.2122	49.31967	51.35586	23.01193	44.53008	29.30792	187.204	293.2958	229.7368	36.08604	39.71569	58.31604	76.44176	118.751	137.9669	45.40437	5.118846	86.23732	18.79211	47.65176	37.91143
LOC107815922	11.26411	15.35388	8.507034	33.97605	35.85877	22.80832	109.5712	154.9158	121.1066	20.68694	39.19265	26.57887	41.34701	57.93193	62.15396	42.80221	78.52229	33.05285	41.92126	40.1704	31.50804	130.6278	133.9146	60.30785	25.78374	94.58747	45.37381	112.7291	35.73135	36.54981
LOC107782755	4.812124	4.618525	5.645304	4.224511	5.803053	3.090438	5.625588	5.275959	4.897652	5.39837	5.56903	4.515131	6.210239	6.086041	7.408549	8.012966	4.936903	6.69905	7.583792	6.754523	7.612325	7.93077	5.540533	4.940611	11.65649	24.64973	7.046515	4.568163	16.50412	47.3959
LOC107785668	0	0	0	0	0	0	0	0	0	0	0	0	0	0	0	0	0	0	0	0	0	0	0	0	0	0	0	0	0	0
LOC107779287	14.55882	16.18474	11.29276	22.46573	20.98024	10.45794	34.62051	35.2679	24.15263	16.3808	20.95579	20.43642	14.71105	13.99795	21.60955	15.47509	11.77754	10.37711	29.57904	37.3533	36.76954	35.86075	32.31629	18.79535	20.18677	32.76824	34.52817	6.864802	18.17754	27.69827
LOC107814358	2.30875	3.156211	2.445967	3.268141	2.681351	3.413009	3.388784	3.082279	4.402848	0.844246	1.112992	0.914295	2.971716	2.754864	1.777228	2.580725	3.972983	2.474327	1.17138	1.464783	2.368821	3.856195	3.627462	2.337695	0.273653	0	0.426368	0	1.389393	0
LOC107774328	0.610357	1.441233	1.30231	0.402148	0.417338	0.357932	0.674821	0.474096	0.885592	0.08116	0.486344	0.46877	0.463267	0.454002	0.284752	0.496188	0.455644	0.579151	0.394131	0.211222	0.227723	0.486556	0.685205	0.985916	0	0.077206	0.065581	1.984054	0.267134	0
LOC107827424	1.639144	0.929524	1.963159	0.494994	0.773559	0.626589	0.672135	0.87144	1.162725	0.248636	0.33204	0.461598	0.56769	0.556337	0.373861	1.6558	1.618741	1.249208	0.776202	0.770335	1.262383	1.186373	1.542222	1.142155	0.875003	0.354783	1.033246	5.209866	1.929015	0
LOC107820019	2.123816	2.461573	1.444843	0.125483	0.183844	0.198553	0.232348	0.276142	0.195059	0.29264	0.226621	0.078011	0.33408	0.352584	0.18955	3.079325	2.988469	2.512779	0.32795	0.371037	0.252646	0.17351	0.427612	0.168899	0.087559	0.32121	0.163707	1.650901	0.111139	0
107795584	0	0	0	0	0	0	0	0	0	0	0	0	0	0	0	0	0	0	0	0	0	0	0	0	0	0	0	0	0	0
LOC107785626	0	0	0	0	0	0	0	0	0	0	0	0	0	0	0	0	0	0	0	0	0	0	0	0	0	0.08899	0	0	0	0
LOC107768318	0.062881	0	0	0	0	0	0	0	0	0	0.088184	0	0	0	0.064539	0.046859	0	0	0	0	0	0	0	0	0.079501	0	0	0	0	0
LOC107805554	0.421182	0.732815	0.199704	0.520322	0.37269	0.123497	0.738642	0.883316	1.114378	0.410706	0.859151	0.679305	0.809859	0.501261	1.218272	0.855997	0.51787	0.513833	0.854773	0.615412	0.663489	1.119174	0.472832	1.080539	0.338865	0.372937	0.316784	0	1.290368	0
LOC107790889	3.469098	4.05087	3.706691	2.50444	2.49412	2.70015	3.485556	2.717084	2.798934	2.149493	3.104014	2.59497	2.735476	2.109134	2.540627	3.204541	3.857647	3.502362	1.998447	2.628929	2.290514	3.787399	3.251222	2.549461	2.512809	2.287795	2.498556	5.168532	2.348644	1.117185
LOC107791071	0	0	0	0	0	0	0	0	0	0	0	0	0	0	0	0	0	0	0	0	0	0	0	0	0	0	0	0	0	0
Novel01991	0.404838	0.766301	0.622622	0.959225	1.010384	0.714256	0.931599	0.780502	0.662703	0.981858	1.317459	0.29817	1.016898	0.713447	1.065421	0.781288	0.300852	0.541735	0.863736	0.825428	0.312416	1.118299	0.595156	0.347189	0.564334	0.808844	0.122689	0	0.449777	1.283678
LOC107806011	5.364192	5.859371	5.86301	6.003142	5.270111	7.63301	5.359316	5.055123	6.072993	5.230765	4.840005	6.231251	4.610345	5.206621	5.019867	5.114324	5.753834	4.910823	4.802834	4.404275	4.568483	4.578596	5.462183	5.3593	3.839792	2.689193	5.780628	0	2.658469	2.438795
LOC107803926	8.875946	12.66619	7.867955	6.454087	5.373804	2.887931	18.12182	14.86834	14.03474	1.594373	2.78434	1.529329	4.550381	2.866743	3.296392	3.82937	6.036286	2.742736	1.362855	1.679519	4.473559	19.40914	12.10431	5.493041	0.147657	0	0	0	0.843394	0
LOC107815125	5.073293	5.495161	4.22539	4.716465	4.747723	4.626254	4.678481	4.720576	3.865287	3.522544	3.24077	3.949458	2.779477	3.071621	3.789452	6.551762	4.208384	5.307179	4.608967	4.830923	3.294567	4.015016	4.568697	3.720152	2.350748	2.365359	2.197569	0	3.069065	1.642353
LOC107786923	0	0	0	0	0	0	0	0	0	0	0	0	0	0	0	0	0	0	0	0	0	0	0	0	0	0	0	0	0	0
Novel03278	0	0.008913	0	0.310095	0.224995	0.0736	0.295294	0.309172	0.128542	0.038146	0.05486	0.008262	0.032661	0.010669	0.030113	0.014576	0	0.007291	0.754202	0.951384	0.437041	0.326689	0.53483	0.204434	0.012364	0.040823	0	0	0.094165	0.60469
LOC107763700	0	0	0	0.060335	0	0	0	0	0	0	0	0	0	0	0	0	0	0	0	0	0	0	0	0	0	0	0	0	0	0
LOC107768882	1.431352	0.951933	1.176988	0.350468	0.890011	0.873418	1.005855	0.859396	0.871665	0.264061	0.434017	0.457553	0.344519	0.46424	0.476465	1.15313	1.831285	1.38439	0.549571	0.58905	0.388097	1.066132	0.921318	1.61735	0.14673	0.161483	0.32006	0	0	0
LOC107815491	0	0	0	0	0	0	0	0	0	0	0	0	0	0	0	0	0	0	0	0	0	0	0	0	0	0	0	0	0	0
LOC107806822	0	0	0	0	0	0	0	0	0	0	0	0	0	0	0	0	0	0	0	0	0	0	0	0	0	0	0	0	0	0
LOC107781752	0	0	0	0	0	0	0	0	0	0	0	0.041708	0	0	0	0	0	0	0	0.083524	0.045025	0	0	0	0	0	0	0	0	0
LOC107825529	22.19303	19.07591	20.20624	17.29477	15.58929	25.78261	14.55617	14.33408	20.09519	12.41025	14.30798	19.68231	16.2167	18.58893	11.22723	15.83285	21.69437	21.72133	14.19504	12.14512	17.60245	13.35149	18.13769	20.72263	6.383031	5.195448	11.37478	3.760937	6.582877	16.25863
LOC107829799	9.090702	8.285054	9.972019	11.80453	13.48336	15.73087	9.403875	8.322153	10.16042	9.37113	9.07594	8.557801	10.64999	9.91751	6.442452	9.709976	8.573279	9.811245	12.60698	13.98867	13.068	7.664187	10.50055	14.2524	8.866346	4.698218	10.74448	6.19156	5.627041	5.353255
LOC107812978	0	0	0	0	0	0	0	0	0	0	0	0	0	0	0	0	0	0	0	0	0	0	0	0	0	0	0	0	0	0
LOC107772812	8.200499	9.067244	7.449624	11.55659	9.805866	8.419397	14.0867	13.782	14.14949	11.1423	11.39678	11.48427	14.83105	15.31356	12.91582	8.790328	7.63688	9.455307	13.19413	13.97828	11.54415	14.41671	13.42411	12.86961	15.13156	15.89917	15.71729	33.4612	19.9179	24.36272
LOC107827443	0	0	0	0	0	0	0	0	0	0.469672	0	0	0.134046	0	0	0	0	0	0	0.101861	0	0	0	0	0.608956	0	0.284638	0	0.579712	0
LOC107802742	1.825512	1.588105	1.640977	1.465888	1.174788	1.918043	1.478938	1.240724	1.129597	0.78891	1.236412	0.455663	0.738977	0.814723	1.192327	1.391292	1.663394	1.484724	0.842002	1.333677	0.472982	1.974963	2.08597	2.558488	0.104909	0.144322	0.245183	0	0.499356	0
LOC107795501	57.95079	46.19243	53.53372	49.75751	49.08178	58.72348	44.84738	45.47904	50.88197	46.23491	37.57794	59.59935	52.5126	50.66966	46.73802	40.07134	48.73123	45.7781	44.36449	42.20945	51.30574	40.65889	42.24984	48.7346	46.4716	45.58323	62.12338	60.62718	53.64181	29.95348
LOC107795773	0	0	0	0	0	0	0	0	0	0	0	0	0	0	0	0	0	0	0	0	0	0	0	0	0	0	0	0	0	0
LOC107801140	0.054905	0	0.143184	0.298449	0.388671	0.94448	0.046052	0.093825	0	0	0.0385	0	0.061121	0	0	0.081831	0.106086	0.081869	0.222857	0.139339	0.100149	0.045853	0.04843	0.172161	0	0.152794	0.324469	0	0	0
LOC107802455	0	0	0	0	0	0	0	0	0	0	0	0	0	0	0	0	0	0	0	0	0	0	0	0	0	0	0	0	0	0
LOC107772776	0	0	0	0	0	0	0	0	0	0	0	0	0	0	0	0	0	0	0	0	0	0	0	0	0	0	0	0	0	0
LOC107771653	30.63144	152.6117	49.65984	34.73442	52.53797	10.1542	117.1551	165.9762	51.71673	56.30405	263.6521	20.6595	32.7922	94.9611	212.0055	77.26799	19.62972	16.51879	26.57568	60.5911	24.81682	659.5238	228.7155	55.38763	28.46468	16.19614	29.26482	14.60387	3.195198	135.7358
LOC107811165	0.419878	0.382667	0.638734	2.662716	6.037453	1.918535	0.968466	1.255643	0.88695	4.196698	0.736047	3.369882	1.986509	1.603231	0.646427	0.46934	0.101409	1.095629	6.497461	5.150223	12.44541	0.350651	0.18518	0.767998	3.052406	3.213262	4.838553	15.0136	4.548243	0
LOC107796379	4.390997	4.708602	4.04462	8.932299	8.679866	7.741775	8.480849	8.407101	6.760459	6.923515	10.08433	7.010872	10.35923	8.9215	10.92589	5.283514	4.90368	4.835428	7.122957	7.829377	5.79574	8.048977	7.488682	6.41052	5.029505	8.730219	2.368741	6.482031	7.127417	15.56777
LOC107827929	1.432609	0.693625	0.661585	0.689494	0.554604	0.577586	0.788544	0.803289	0.504374	0.698487	0.847586	0.340399	0.847238	0.244204	0.367598	0.800686	1.124517	0.700921	0.499714	0.227229	0.408301	0.710368	0.473871	0.795474	0.226407	0.373757	0.370394	0	3.017476	0
LOC107813317	0	0.076749	0.073204	0	0	0	0	0	0	0	0.118099	0	0	0	0	0	0	0.125567	0	0	0	0	0	0	0	0	0	0	0	0
LOC107775145	0	0	0	0	0	0	0	0	0	0	0	0	0	0	0	0	0	0	0	0	0	0	0	0	0	0	0	0	0	0
LOC107798519	1.394588	1.120199	1.438305	0.556764	0.794794	0.914859	0.535287	0.686669	0.776704	0.25354	0.348061	0.559139	0.499949	0.541525	0.582251	1.144935	1.324453	1.691755	0.743543	0.519879	0.474263	0.829077	0.938226	1.03763	0.26896	2.137802	0.307308	0	0.227595	0
LOC107808200	5.332248	6.309538	4.968126	15.69325	23.48735	11.94073	23.15263	31.18732	22.98403	27.2908	18.4883	24.3908	18.10296	25.00441	30.08568	5.729884	4.610645	6.105889	25.9778	24.92108	23.69754	29.30189	25.90765	17.67433	57.76903	81.73675	38.78867	42.13604	43.25834	38.25259
LOC107795012	1.889658	2.081963	1.788902	2.808276	3.29265	2.219468	4.114324	3.809733	3.736534	2.536806	3.625679	1.711248	3.760533	3.325266	4.622901	2.416081	1.387932	2.229024	2.449402	2.923322	1.410591	6.225939	3.561936	2.83409	3.9682	4.709366	1.866285	0.925598	3.956783	9.60332
LOC107792987	0	0	0	0	0	0	0	0	0	0	0	0	0	0	0	0	0	0	0	0	0	0	0	0	0	0	0	0	0	0
LOC107825101	3.506764	2.038816	3.819204	5.313175	3.156515	6.218663	2.197503	1.911416	4.238369	1.159498	1.823011	1.089696	1.413437	2.154708	0.682616	1.817256	1.985968	2.058497	1.554253	0.937681	1.672636	2.911759	4.568688	6.235169	0.025481	0.084128	0.381123	1.441283	0	0
Novel02183	0.308535	0.335267	0.216625	0.612788	1.102549	0.586877	0.716626	0.780723	0.401076	0.798438	1.139972	0.551394	0.885084	0.996844	0.889117	0.25645	0.378321	0.300803	1.155988	1.22469	0.627714	0.634254	0.408223	0.508527	1.065224	1.552099	0.490893	3.394557	0.685568	0
[truncated: 20,180,109 more chars]
